# Longitudinal associations of dispositional forgivingness with multidimensional well-being: a two-wave outcome-wide analysis in the Global Flourishing Study

**DOI:** 10.1038/s44184-026-00187-5

**Published:** 2026-01-21

**Authors:** Richard G. Cowden, Everett L. Worthington, R. Noah Padgett, Chris Felton, Dorota Weziak-Bialowolska, Renae Wilkinson, Katherine Jackson-Meyer, Zhuo J. Chen, Matt Bradshaw, Byron R. Johnson, Tyler J. VanderWeele

**Affiliations:** 1 Human Flourishing Program, Institute for Quantitative Social Science, Harvard University, Cambridge, MA USA; 2 Department of Epidemiology, Harvard T.H. Chan School of Public Health, Boston, MA USA; 3 Department of Psychology, Virginia Commonwealth University, Richmond, VA USA; 4 Department of Quantitative Methods & Information Technology, Kozminski University, Warsaw, Poland; 5 School of Nursing, University of North Carolina at Charlotte, Charlotte, NC USA; 6 Institute for Studies of Religion, Baylor University, Waco, TX USA; 7 Department of Sociology, Baylor University, Waco, TX USA

**Keywords:** Health care, Psychology, Psychology

## Abstract

In this preregistered longitudinal study with nationally representative samples from 23 countries in the Global Flourishing Study (*N* = 207,919), we examine associations between dispositional forgivingness and multidimensional well-being outcomes approximately one year later. Following the analytic template for outcome-wide designs, we conducted a series of country-specific weighted multivariate regression analyses where each Wave 2 outcome was regressed on Wave 1 forgivingness (controlling for Wave 1 sociodemographic and retrospectively recalled childhood variables). Random effects meta-analyses were used to pool country-specific estimates of association for the 56 main outcomes covering psychological, social, physical, volitional, and material dimensions of well-being. We found some evidence of association between forgivingness and higher well-being for both composite well-being indicators and numerous specific indicators across different domains of functioning (observed associations were mostly very small in magnitude). Associations were generally stronger and more consistent for some domains (e.g., psychological well-being) compared to others (e.g., physical health & health behavior). Meta-analyzed estimates of association generally attenuated after using a more conservative analytic approach that additionally adjusted for principal components extracted from the Wave 1 outcomes treated as covariates. Country-specific results showed some cross-national variation. Our findings contribute novel population-level evidence on the potential benefits of forgivingness for different aspects of well-being.

## Introduction

All people experience interpersonal hurts or offenses at some point in life^1^. From a stress-and-coping perspective, interpersonal transgressions can precipitate a perceived sense of injustice to which they might respond with unforgiveness—a complex cognitive, emotional, and/or behavioral response that may involve (among other factors) bitterness, resentment, hostility, hatred, anger, fear, and revenge-seeking or avoidance motives^2^. It is not uncommon for individuals who have been wronged to struggle with unforgiveness. To illustrate, recent nationally representative data from Wave 1 of the Global Flourishing Study (GFS) indicated that approximately 25% of people across the 22 countries analyzed reported ‘rarely/never’ forgiving those who have hurt them, with national estimates ranging from 8% in Nigeria to 59% in Turkey^3^. When unforgiveness persists or remains unresolved, it can have detrimental effects on well-being^4^.

Individuals may attempt to cope with their unforgiveness through various strategies (e.g., seeking revenge, deferring to divine judgment, excusing the offender’s behavior). From a stress-and-coping perspective, forgiveness is considered an adaptive strategy for reducing the negative effects of unforgiveness on well-being^5,6^. Given how common interpersonal hurts or offenses are, the frequent struggles that people have with unforgiveness, and the potential benefits of forgiveness (especially when practiced consistently across time and situations), the implications of forgiveness for population well-being may be considerable^7^. In the present study, we use longitudinal data from 23 national representative samples to explore the population-level associations of dispositional forgivingness—the general tendency to forgive others—with a wide range of well-being outcomes assessed approximately one year later.

While scholars do not completely agree on the definition of forgiveness^8^, there is broad consensus that forgiveness involves a prosocial shift in a person’s thoughts, emotions, motivations, and/or behavioral intentions toward someone who has hurt or offended them^4,9^. Aggregation of specific instances of forgiveness can develop into a more enduring tendency to forgive others across situations and over time (i.e., dispositional forgivingness). Whereas a single incidence of forgiveness can temporarily boost well-being, the practice of forgiveness over time (i.e., forgivingness) can affect well-being more generally. However, forgivingness—despite being a disposition—is not perfectly stable. Recent events or highly salient transgressions can shift people away from a forgiving disposition. For example, offenses that re-trigger interpersonal traumas (e.g., a survivor of child sexual abuse learning that a child in their family has been abused), serious and ongoing abuse, or repetitions of earlier wrongs (e.g., a subsequent spousal infidelity after a couple reconciles) can influence the stability of forgivingness. In these types of cases, the cognitive and emotional demands associated with processing the transgression may temporarily suppress forgivingness so that a person’s score on a measure of forgivingness may be lower than usual^10^. However, people will typically return to their usual level of forgivingness after the immediate hurt or offense they are dealing with has been resolved^11^. Events like reconciliation of warring groups or individual experiences of reconciliation in broken important relationships also have the potential to move low levels of forgivingness higher.

Well-being has been conceptualized in different ways over the years. One increasingly popular framing positions well-being as a multidimensional concept that is embedded in a broader notion of human flourishing defined as “the relative attainment of a state in which all aspects of a person’s life are good, including the contexts in which that person lives” (p. 19)^12^. Compared to other frameworks that adopt a narrower view of well-being, human flourishing offers a holistic approach by considering “the quality of [an individual’s] personal subjective state” across the various dimensions of human existence (p. 4)^13^. Although there are many ways to carve up the broad conceptual terrain covered by this framework, in alignment with prior work^14,15^ the scope of the well-being outcomes that are central to the present study might be organized thematically into psychological (i.e., psychological well-being, psychological distress), social (i.e., social well-being, social distress, social participation), physical (i.e., physical health and health behavior), volitional (i.e., character and prosocial behavior), and material (i.e., socioeconomic) dimensions of well-being. If forgiveness—or more broadly forgivingness—is a ‘whole person experience’^16^, its influence may extend across these dimensions of well-being.

An abundance of observational (mostly cross-sectional) studies (and to a lesser extent experimental intervention studies) have reported evidence on the association between forgiving others and different aspects of well-being^16^. The psychological dimension has received the lion’s share of attention in this area of research, in part because theorizing (e.g., stress-and-coping theory) suggests that the emotion-regulation function of forgiveness has more direct and proximal benefits for reducing psychological distress and improving psychological well-being^17^. Several recent reviews^4,6^ and meta-analyses^18,19^ have shown that forgiving others is negatively related to indicators of psychological distress (e.g., anxiety symptoms, depression symptoms) and positively related to indicators of psychological well-being (e.g., life satisfaction, sense of purpose). While most existing studies are based on correlational data, findings for the psychological dimension of well-being tend to replicate in more rigorous longitudinal and experimental research^10,20–22^.

A similar pattern of findings has been observed for other dimensions of well-being, though fewer and less consistent evidence for non-psychological dimensions may reflect more indirect and downstream effects of forgiveness compared to the psychological dimension. For example, the psychological relief that forgiveness provides to the forgiver is thought to lay the foundation for the possibility of reconciling (when safe and appropriate) with the transgressor, experiencing deeper social connectedness more generally, and building trust in others^23^. Providing some support for this theorizing, prior (mostly cross-sectional) studies have positively linked forgiving others to indicators of social well-being (e.g., social connectedness, trust) and social participation (e.g., marriage, religious service attendance), and reported negative associations with social distress outcomes (e.g., perceived bullying victimization, loneliness)^3,24–27^.

Viewed as a moral virtue, forgiveness represents an intentional response to wrongdoing that is guided by an aspiration to act in accordance with one’s ethical commitments^1^. This suggests that forgiveness of others should be related to volitional types of well-being outcomes that reflect choices, actions, or experiences that are guided by a person’s agency, will, and commitment to upholding their moral convictions or personal values^6^. Evidence in support of this idea comes from (largely cross-sectional) research that has reported positive associations between forgiveness of others and character strengths or virtues, such as hope, gratitude, and temperance^21,28,29^, as well as prosocial behaviors like conciliatory behavior, making charitable donations, and volunteering^6,20,30^. While these findings suggest a pattern, further work is required to expand and strengthen the robustness of existing evidence and better understand how forgiveness of others is related to various indicators of volitional well-being (e.g., helping strangers, showing love to others).

A growing number of (mostly cross-sectional) studies have identified links between forgiveness of others and indicators of better physical health, physiological markers associated with health, and health behavior (for meta-analyses, see refs. ^20,31^, for reviews, see refs. ^5,32^). For example, prior work has shown that forgiveness of others is related to more favorable physiology (e.g., lower blood pressure, lower cortisol), subjective experiences or perceptions of physical health (e.g., lower bodily pain, higher self-rated health), and health-related behavioral outcomes (e.g., lower risk of substance abuse, higher quality dietary intake). Forgiveness of others generally tends to exhibit weaker associations with outcomes corresponding to the physical dimension of well-being compared to other dimensions (e.g., psychological, social), although disproportionately fewer studies have attended to the physical dimension of well-being^16^. While many hundreds of studies focusing on psychological outcomes have been published in the last two decades, a somewhat recent review of studies addressing physical health over an 18-year period prior to the database search date yielded just 55 studies^33^. With more than 80% of those studies based on cross-sectional data, there is a need for more rigorous research to draw firmer conclusions about the potential implications that forgiving others may have for the physical dimension of well-being.

Forgiveness of others may also influence material well-being through indirect pathways over time, such as by lowering chronic stress that can be disruptive to socioeconomic aspects of life (e.g., maintaining steady employment), supporting stability in the network of relationships that can contribute to socioeconomic standing (e.g., connections to potential employment opportunities), or freeing up cognitive-emotional resources to focus on important goals (e.g., educational or vocational pursuits) tied to material well-being^34,35^. Although research has examined forgiveness of others in relation to selected indicators of material well-being, evidence is generally quite limited and mixed. For example, some cross-sectional studies have reported that forgiveness of others is positively associated with educational attainment^36^, whereas in other studies this association has been more negligible^37^. In one of the largest studies to date, a recent analysis of Wave 1 GFS data from 22 countries showed that the socioeconomic subgroups with the lowest forgivingness in the greatest number of countries included those who were unemployed (in eight countries out of 22) and those with eight or fewer years of education (in 11 of 22), indicating that these subgroups were not consistently lowest in forgivingness across all countries^3^. While these findings represent a step forward in identifying vulnerable subgroups on selected socioeconomic indicators across a wide swath of countries, the evidence is insufficient for addressing questions of causality because the data were cross-sectional and the analysis did not include adjustment for potential confounders. Hence, additional work is needed to strengthen insights into the potential causal effects of forgiving others on outcomes representing the material dimension of well-being.

Prior research has contributed considerably to strengthening our understanding of the relationship between forgiveness of others and well-being, but some important gaps remain. First, most observational studies have relied on cross-sectional data that are typically not informative for drawing potential causal inferences. For example, when forgiveness of others and indicators of well-being are assessed contemporaneously, temporal sequence often cannot be discerned. Longitudinal studies can allow for clearer examination of temporal dynamics.

Second, prior work in this area has typically examined one or a few well-being outcomes, often within a single domain^6^. Longitudinal cohort studies that include multidimensional assessments of well-being are needed to address temporal dynamics between forgiveness of others and outcomes of interest, develop more precise estimates of associations, and build a more integrative understanding of how forgiveness of others might be implicated in human flourishing. A few recent studies have attempted to address some of these gaps^10,20,25,28^. For example, Chen et al.^20^ examined associations of forgivingness with 25 well-being outcomes assessed between 3 and 6 years later in a large United States sample of young adult children of nurses. Forgivingness was associated with better well-being on 11 of the outcomes, with stronger and more consistent associations observed for psychosocial outcomes (e.g., depression symptoms, life satisfaction, self-esteem) compared to physical health and health behavior outcomes. Findings have been similar in other studies that have examined a wide range of well-being outcomes^10,25^.

Third, existing research on forgiveness of others and well-being has tended to focus on population segments (e.g., university students, young adults, nurses). Reliance on selective samples can make it difficult to determine if findings apply to the general population due to potential sampling bias (e.g., convenience sampling), the influence of shared characteristics or context-specific features within a sample on forgiveness-related processes and outcomes, or the inability to account for broader socioecological variation that may be relevant. For example, one large longitudinal study of young adults found little evidence of association between forgivingness and physical health^20^, but this may mask population-level effects because young adults tend to have comparatively fewer physical health problems. To more accurately estimate the potential impact of forgiving others on well-being at the population level, studies with representative samples of the general population are needed.

Fourth, most previous research reporting associations between forgiveness of others and indicators of well-being has been concentrated in more Western, educated, industrialized, rich, and democratic (WEIRD) societies. For example, one recent review on forgiveness of others and well-being reported that about two-thirds of the samples were from North America or Europe^6^. Comparatively fewer studies have been conducted in less WEIRD contexts. Although the findings of studies involving samples from less WEIRD contexts generally align with the broader empirical research linking forgiveness of others with well-being, methodological weaknesses of this literature (e.g., cross-sectional designs) constrain its utility. Principles of socioecological systems theory^38^ would suggest that contextualized historical events, cultural dynamics, and systemic factors have the potential to impinge upon experiences of forgiving others and its relationship to well-being outcomes^39,40^, which implies that we should not assume forgiveness of others will have the same effects on different aspects of well-being in different countries or regions around the world. While some prior research points to the possibility that the benefits of forgiving others for well-being may vary across different socioecological contexts^22^, no prior study has used nationally representative data from multiple countries to explore this possibility across a diverse range of cultures and geographic contexts.

To help address some of these gaps in knowledge, this preregistered study uses two waves of nationally representative data from the GFS to examine longitudinal associations of self-reported forgivingness assessed in Wave 1 with two subsequent composite well-being indicators and an array of specific indicators of well-being across eight domains: psychological well-being, psychological distress, social well-being, social distress, social participation, character & prosocial behavior, physical health & health behavior, and socioeconomic outcomes. Based on previous research, we expected that forgivingness would generally be associated with improved subsequent well-being across the 23 countries included in the GFS, although associations were anticipated to be somewhat stronger and more consistent for outcomes in some domains (e.g., psychological well-being) compared to others (e.g., physical health & health behavior). Due to the diversity of socioecological contexts represented in the GFS, we expected that the pattern of associations between forgivingness and the outcomes would vary to some extent across the different contexts.

## Methods

The study design, sampling, and survey development for the GFS are described elsewhere^15,41–43^. The methodology for the analyses outlined below is described in detail in Padgett et al.^44^ and VanderWeele et al.^45^. The GFS was ruled exempt by the Baylor University Institutional Review Board (#1841317-2) because it met the criteria for exemption according to Baylor’s Institutional Review Board guidelines (e.g., minimal risk to participants, adherence to specific federal regulations). Ethical approval for all data collection activities was also obtained from the Institutional Review Board at Gallup Inc. Data collection activities were performed in accordance with relevant ethical regulations, and informed consent was obtained from all participants. All direct common identifiers were removed from the data used in the present study by Gallup Inc.

### Study sample

Wave 1 of the GFS included nationally representative samples from 23 countries: Argentina, Australia, Brazil, China, Egypt, Germany, Hong Kong (Special Administrative Region of China), India, Indonesia, Israel, Japan, Kenya, Mexico, Nigeria, the Philippines, Poland, South Africa, Spain, Sweden, Tanzania, Turkey, the United Kingdom, and the United States (*N* = 207,919). The countries were selected to (1) maximize coverage of the world’s population, (2) ensure geographic, cultural, and religious diversity, and (3) prioritize feasibility based on Gallup Inc.’s existing data collection infrastructure. Data for Wave 1 were collected from March 2022 to January 2024, except in China where data were collected in March and April 2024^46^. Data for Wave 2 were collected from January 2024 to December 2024, with data in China collected at least six months after Wave 1. The rate of nonresponse to the Wave 2 survey was 38% in the total sample. Descriptive statistics for the total sample by retention status are reported in Tables S3–S4. Attrition was 38% or below in 11 samples (lowest in China at 9%), whereas attrition in the other 12 samples ranged from 41% to 80% (highest in Hong Kong). Descriptive statistics for each country by retention status are reported in Tables S9c, d to S31c, d.

The GFS survey assesses various aspects of well-being, including happiness, health, meaning, character, relationships, and financial security, along with a range of other demographic, social, economic, political, religious/spiritual, personality, childhood, community, health, and well-being variables. Gallup Inc. translated the GFS survey into multiple languages following the TRAPD (translation, review, adjudication, pretesting, and documentation) model for cross-cultural survey research^47^. Additional details about the translation, cognitive interviewing, and pilot testing phases of the GFS can be found elsewhere^42,48,49^.

### Sampling design

The precise sampling design varied by country to ensure samples were approximately nationally representative^43,47^. In most countries, local field partners implemented a probability-based face-to-face or telephone methodology to recruit panel members. Recruitment involved an intake survey gathering basic sociodemographic information and details for recontacting participants. Following recruitment, participants received invitations to participate in the annual survey via phone or online. Follow-up for Wave 2 data collection relied on the respondent-provided contact information. A minimum of three contact attempts were made on different days of the week and times of the day to maximize the possibility of retention. Post-stratification and nonresponse adjustments to the Wave 1 sampling weights were performed separately within each country, using either census data or a reliable secondary source. Additional information about the sampling design and weighting scheme for Wave 2 is available elsewhere^44,46^.

### Outcome-wide analytic design

An outcome-wide analytic approach for longitudinal designs^50^ was employed to examine the associations of a single exposure (i.e., dispositional forgivingness) with a range of subsequent outcomes. Compared to traditional analytic strategies focused on a single outcome, this approach provides a more holistic assessment of an exposure’s possibly differential relations with multiple life outcomes. The outcome-wide analytic design has several strengths, including (1) reducing researcher subjectivity or degrees of freedom in the analysis by ensuring a consistent analytic strategy and the same set of covariates across models for all outcomes; (2) mitigating publication bias by reporting results for all examined outcomes simultaneously; and (3) providing insights into beneficial, detrimental, and null associations with the exposure^51,52^. Further details about the outcome-wide approach can be found elsewhere^50,51^.

### Measures

The exposure was taken from Wave 1. The measure of dispositional forgivingness used in the GFS survey comes from the Forgiveness-Short Form measure that is part of the Brief Multidimensional Measure of Religiousness/Spirituality^53^. The Forgiveness-Short Form contains three items, one for each of three types of forgiveness: forgiveness of others, self-forgiveness, and divine forgiveness. The original forgiveness of others item (i.e., “I have forgiven those who hurt me”; response options: ‘Never,’ ‘Seldom,’ ‘Often,’ ‘Always or almost always’) has been used in previous research^54^, and other studies have employed variations of this item as well^25^. To strengthen the cross-cultural equivalence of the original item for use in the GFS survey, slight modifications were made to the phrasing and response categories based on the results of cognitive interviews conducted during the GFS survey development process^42,43,48,49^. The version of the item used in the GFS survey is: “How often have you forgiven those who have hurt you?” (response options: ‘Never,’ ‘Rarely,’ ‘Often,’ ‘Always’). Following prior studies using Wave 1 data from the GFS^3,55,56^, we dichotomized responses to this variable into (0) never/rarely and (1) often/always. In addition to maintaining consistency and comparability with earlier work in the GFS that supports the cumulative development of evidence in this area, our approach to grouping response options reflects a meaningful threshold for habitual forgiveness and facilitates clearer interpretation of results for non-technical audiences.

Country-specific analyses adjusted for 17 covariates (9 sociodemographic and 8 childhood variables) taken from Wave 1, unless data were not available (as described below and in relevant tables). Additional details for all variables can be found in the GFS codebook (https://osf.io/cg76b). Weighted descriptive statistics for the sociodemographic and childhood variables are reported in Table S1 for the total sample and Tables S9a–S31a for each country by wave (see also Table S32).

Year of birth (age) was classified into 1998–2005 (18–24 years), 1993–1998 (25–29 years), 1983–1993 (30–39 years), 1973–1983 (40–49 years), 1963–1973 (50–59 years), 1953–1963 (60–69 years), 1943–1953 (70–79 years), and 1943 or earlier (80 years or older). Gender was assessed as male, female, or other. Marital status was assessed as single/never married, married, separated, divorced, widowed, and domestic partner. Employment was assessed as employed, self-employed, retired, student, homemaker, unemployed and looking for a job, and none of these/other. Education was assessed as up to 8 years, 9–15 years, and 16 or more years. Religious service attendance was assessed as more than once a week, once a week, one to three times a month, a few times a year, and never. Immigration status was assessed with yes/no responses to: “Were you born in this country, or not?” Religious affiliation was assessed in all countries, but there was considerable cross-country variation in endorsement of the response categories because some religious affiliations are only applicable in certain countries. Religious affiliation response categories included Christianity, Islam, Hinduism, Buddhism, Judaism, Sikhism, Baha’i, Jainism, Shinto, Taoism, Confucianism, primal/animist/folk religion, Spiritism, Umbanda, Candomblé, and other African-derived religions, Chinese folk/traditional religion, some other religion, or no religion/atheist/agnostic. When more than 5% of a within-country sample endorsed the no religion/atheist/agnostic category, this was used as the reference category in the country-specific analyses; otherwise, the most prominent religious group was used. Additionally, all religious affiliation categories endorsed by less than 3% of a within-country sample were collapsed into a single religious affiliation category. Racial/ethnic identity was assessed in most countries, but not in China, Germany, Japan, Spain, and Sweden. In countries where racial/ethnic identity was assessed, response categories varied across countries to be locally meaningful. Country-specific analyses that adjusted for racial/ethnic identity used a binary variable based on whether an individual was in the most prominent racial/ethnic group in the sample versus a minority racial/ethnic group.

Quality of relationship with mother when growing up was assessed with the question: “Please think about your relationship with your mother when you were growing up. In general, would you say that relationship was very good, somewhat good, somewhat bad, or very bad?” Responses were dichotomized to very/somewhat good versus very/somewhat bad. “Does not apply” was treated as a dichotomous control variable for respondents who did not have a mother due to death or absence. An analogous variable was used for the quality of a person’s relationship with their father when growing up. Parental marital status around age 12 was assessed with responses of married, divorced, never married, and one or both had died. Subjective financial status of one’s family around age 12 was measured with: “Which one of these phrases comes closest to your own feelings about your family’s household income when you were growing up, such as when you were around 12 years old?” Responses were lived comfortably, got by, found it difficult, and found it very difficult. Childhood abuse when growing up was assessed with yes/no responses to: “Were you ever physically or sexually abused when you were growing up?” Similarly, participants provided a yes/no response to whether they felt like an outsider in their family when growing up: “When you were growing up, did you feel like an outsider in your family?” Self-rated health when growing up was assessed by: “In general, how was your health when you were growing up? Was it excellent, very good, good, fair, or poor?” Religious service attendance around age 12 was assessed with: “How often did you attend religious services or worship at a temple, mosque, shrine, church, or other religious building when you were around 12 years old?” with responses of at least once a week, one to three times a month, less than once a month, or never.

Consistent with prior outcome-wide studies that have applied a multidimensional approach to well-being^57,58^, we examined a wide range of well-being outcomes in Wave 2. There were 56 main outcomes considered, including 54 specific indicators of well-being across eight domains: psychological well-being (12 indicators), psychological distress (four indicators), social well-being (nine indicators), social distress (two indicators), social participation (five indicators), character & prosocial behavior (nine indicators), physical health & health behavior (six indicators), and socioeconomic outcomes (seven indicators). The other two main outcomes were composite indicators of individual well-being from existing measures, including the 12-item Secure Flourishing Index that consists of two items for each of six domains—happiness & life satisfaction, mental & physical health, meaning & purpose, character & virtue, close social relationships, and financial & material stability—and the 10-item Flourishing Index that excludes financial & material stability because this domain is sometimes considered a means of flourishing rather than an end in itself^14,15^. In these Wave 2 data of the GFS, estimated internal consistency for the Secure Flourishing Index in the overall sample was *α* = 0.88 (ranging from *α* = 0.75 in Nigeria to *α* = 0.94 in Japan). We followed prior outcome-wide studies by including forgivingness as a ‘benchmark outcome’ to support the interpretation of results^59^.

Although not part of the main outcomes that we preregistered, we also report results for 22 additional well-being and related outcomes in the Supplemental File for readers who may be interested and to support cumulative scientific progress. Of those outcomes, four were single-item measures of primary outcomes that combined two items (i.e., anxiety symptoms composite, depression symptoms composite) and six corresponded to the domains of the Secure Flourishing Index outlined above. The remaining 12 outcomes were indicators of religious/spiritual beliefs, experiences, and engagement.

Descriptions of each measure, response options, recoding decisions, and details about which outcomes were modeled as binary versus continuous variables can be found at https://osf.io/9kpd8 (see ‘W2-Core Team Analyses’ worksheet). Decisions about operationalizing the outcomes were made prior to conducting analyses. All outcomes assessed on an 11-point scale were modeled as continuous variables. Other outcomes were assessed using Likert-type response formats with four or fewer categories, which were coded into binary variables for a few reasons. First, the analyses reported herein are part of a coordinated set of outcome-wide analyses that differ only in focal exposure. Applying a consistent coding scheme to the outcomes enhances the comparability of results across various exposures and outcomes that use this coordinated analytic framework. Second, ordinal regression models for outcomes with Likert-type response formats were deemed less suitable because their coefficients are challenging to concisely summarize and interpret when the effect of an exposure varies across categories, which is especially challenging in the context of an outcome-wide analysis involving numerous outcomes. Decisions about the cut points for outcomes assessed using Likert-type response formats were based on substantive grounds to provide a meaningful binary grouping of each outcome, informed by the subject matter expertise of scholars involved in coordinated Wave 1 analyses of the GFS data. Weighted descriptive statistics for the outcomes are reported in Table S2 for the total sample and Tables S9b–S31b for each country by wave (see also Table S33).

### Statistical analysis

All analyses were performed using R 4.5^60^ and the *Rglobalflourishing* package^61^. Weighted descriptive statistics for the sample (*N* = 207,919) were estimated for each of the sociodemographic and outcome variables at both waves. All country-specific analyses, including imputation and attrition modeling described below, accounted for the complex survey design by incorporating weights, primary sampling units, and strata provided by Gallup Inc.^44,46^. Within each country, we conducted a series of weighted linear (for continuous outcomes) or weighted modified Poisson (for binary outcomes) regression analyses. A separate model was estimated for each of the 78 outcomes (though only the 56 main outcomes were considered in the primary meta-analyses). Two models were used for each outcome. In Model 1, we regressed each outcome on forgivingness while controlling only for Wave 1 variables (i.e., sociodemographic and childhood characteristics) that we could reasonably assume were not on the pathway (i.e., mediators) between forgivingness and one or more outcomes. However, there is a risk that Model 1 may not provide adequate confounding control^62^. To explore this possibility, we performed an alternative to Model 1 in which we controlled additionally for principal components extracted from all contemporaneous Wave 1 variables other than the exposure of forgivingness (Model 2). Although Model 2 carries the risk of attenuated associations because the set of covariates could include one or more mediators, it provides an opportunity to evaluate how the results may differ after more extensive adjustment for potential confounding^63^. Principal components were used to reduce the dimensionality of predictors to mitigate multicollinearity^64^, while accommodating complex survey weights and missing data. The first seven principal components were used, accounting for an average of 51.2% of the variability in all covariates across the countries^44^, with additional principal components each explaining only 1–2% of additional variance (see Tables S9f–S31f for principal components results by country).

All analyses were initially conducted by country. Random effects meta-analyses were used to pool estimates for the outcomes across countries and to estimate heterogeneity (tau), with our primary meta-analyses focusing on the 56 main outcomes that were preregistered. For each outcome, a global *p*-value for an omnibus test of evidence of association in any country is reported^65^. Bonferroni-corrected *p*-value thresholds for our primary meta-analyses are provided based on the number of main outcomes for which effect sizes were estimated: *p* = 0.05/56 = 0.00089. All meta-analyses were conducted using the *metafor* package^66^. We interpret results principally based on the magnitude of observed associations using guidelines provided by Funder and Ozer^67^.

We report *E*-values for all associations to evaluate the sensitivity of results to potential unmeasured confounding. An *E*-value is the minimum strength of the association (on the risk ratio scale) that an unmeasured confounder would need to have with both the exposure and the outcome, above and beyond all measured covariates, to explain away their association^68^. *E*-values range from a value of 1 to anything above 1. A high *E*-value signifies that an unmeasured confounder would need to have a strong association with both the exposure and the outcome to explain away the association observed between them. Approximate *E*-values can be obtained for continuous outcomes through scale conversions^68^.

The primary analyses use all participants from Wave 1, including those who did not respond to the Wave 2 survey, by imputing missing data^69^. The attrition rate was 38% in the total sample, ranging from 9% in China to 80% in Hong Kong (for further details, see ref. ^44^). The country-specific patterns of missing data for all sociodemographic characteristics and childhood factors in the observed sample are presented in Tables S9a–S31a (see also Table S32 for a cross-country summary). Country-specific patterns of missing data for the outcome variables in the observed sample for Waves 1 and 2 are reported in Tables S9b–S31b (see also Table S33 for a cross-country summary). Multiple imputation by chained equations^70,71^ was used to impute missing data on the exposure, covariates, and outcomes. Twenty imputed datasets were used, with results pooled using Rubin’s rule^72^. We chose to impute 20 datasets to meet the efficiency threshold for multiple imputation, using the highest country-level fraction of missing information as a conservative benchmark (for additional details, see ref. ^44^). Using multiple imputation with all participants aligns with Wave 1 analyses and will also be used in Wave 3 to maximize utilization of information on those who did not respond in Wave 2 but respond again in Wave 3, thereby aligning the analytic sample across years and facilitating comparison of results. For Wave 1 missing data, the imputation models used sampling weights, sociodemographic characteristics, and retrospectively recalled childhood variables; for Wave 2 missing data, the imputation models additionally included the exposure of interest and prior values of all the outcomes assessed in Wave 1. Imputation was conducted separately by country to account for variation in the assessment of certain variables across countries (e.g., racial/ethnic identity), thereby also reflecting country-specific contexts and assessment methods.

As a sensitivity analysis for possible misspecification of the imputation models^73^, analyses were conducted using only Wave 2 participants (semi-complete case analysis), with attrition weights multiplied by the sampling weights for use in the analysis^74^. Attrition weights were estimated separately for each country using logistic regression models for retention to calculate stabilized inverse probability of retention weights^75^. Attrition predictors included sampling weight, strata, mode of survey completion, age, gender, education, income, employment status, marital status, racial/ethnic identity, religious service attendance, urban/rural status of participants, the Big Five personality traits, days of exercise, depression symptoms, loneliness, and the six domains of the Secure Flourishing Index, covering a range of potentially important predictors^50,76^. A summary of attrition model results for each country is presented in Tables S9e–S31e.

## Results

Sociodemographic characteristics of the total observed sample at Wave 1, weighted to be nationally representative within each country, are reported in Table 1 (see Table S1 for the weighted distribution of the childhood characteristics). The most common age group was 30–39 years (20%), followed by 40–49 years (17%) and 50–59 years (16%). Gender was nearly evenly split between males (49%) and females (51%). A majority were married (53%), employed by an employer (39%) or self-employed (18%), had 9–15 years of education (57%), and were native-born (94%). Religious service attendance ranged from weekly (19%) or more often (13%) to never (37%).

**Table 1:** Weighted Sample Characteristics

Characteristic	Wave 1 (*N* = 207,919)	Wave 2 (*N* = 128,868)
Dispositional forgivingness, *n* (%)		
Always	61,254 (29.5%)	32,316 (25.1%)
Often	93,572 (45.0%)	61,616 (47.8%)
Rarely	43,112 (20.7%)	28,600 (22.2%)
Never	9266 (4.5%)	5874 (4.6%)
(Missing)	714 (0.3%)	461 (0.4%)
Year of birth (current age), *n* (%)		
1943 or earlier (current age: 80+ years)	4047 (1.9%)	3445 (2.7%)
1943–1953 (current age: 70–79 years)	16,902 (8.1%)	12,684 (9.8%)
1953–1963 (current age: 60–69 years)	29,031 (14.0%)	19,538 (15.2%)
1963–1973 (current age: 50–59 years)	32,409 (15.6%)	20,495 (15.9%)
1973–1983 (current age: 40–49 years)	34,970 (16.8%)	21,996 (17.1%)
1983–1993 (current age: 30–39 years)	40,297 (19.4%)	24,641 (19.1%)
1993–1998 (current age: 25–29 years)	20,325 (9.8%)	12,309 (9.6%)
1998–2005 (current age: 18–24 years)	29,920 (14.4%)	13,760 (10.7%)
(Missing)	18 ( < 0.1%)	1 ( < 0.1%)
Gender, *n* (%)		
Male	100,661 (48.4%)	62,160 (48.2%)
Female	106,349 (51.1%)	66,141 (51.3%)
Other	523 (0.3%)	370 (0.3%)
(Missing)	386 (0.2%)	197 (0.2%)
Education, *n* (%)		
Up to 8 years	46,842 (22.5%)	22,657 (17.6%)
9–15 years	116,015 (55.8%)	72,942 (56.6%)
16+ years	44,904 (21.6%)	33,258 (25.8%)
(Missing)	158 (0.1%)	11 ( < 0.1%)
Country of respondent, *n* (%)		
Argentina	6724 (3.2%)	2876 (2.2%)
Australia	3844 (1.8%)	2578 (2.0%)
Brazil	13,203 (6.4%)	4221 (3.3%)
China	5022 (2.4%)	4575 (3.6%)
Egypt	4729 (2.3%)	3044 (2.4%)
Germany	9506 (4.6%)	5585 (4.3%)
Hong Kong (S.A.R. of China)	3012 (1.4%)	608 (0.5%)
India	12,765 (6.1%)	6353 (4.9%)
Indonesia	6992 (3.4%)	2661 (2.1%)
Israel	3669 (1.8%)	2494 (1.9%)
Japan	20,543 (9.9%)	13,966 (10.8%)
Kenya	11,389 (5.5%)	7712 (6.0%)
Mexico	5776 (2.8%)	2264 (1.8%)
Nigeria	6827 (3.3%)	3144 (2.4%)
Philippines	5292 (2.5%)	2684 (2.1%)
Poland	10,389 (5.0%)	6545 (5.1%)
South Africa	2651 (1.3%)	962 (0.7%)
Spain	6290 (3.0%)	2917 (2.3%)
Sweden	15,068 (7.2%)	11,663 (9.1%)
Tanzania	9075 (4.4%)	5588 (4.3%)
Turkey	1473 (0.7%)	498 (0.4%)
United Kingdom	5368 (2.6%)	3621 (2.8%)
United States	38,312 (18.4%)	32,309 (25.1%)

*Note*. Table is based on non-imputed data; cumulative percentages for variables may not add up to 100% due to rounding; S.A.R., Special Administrative Region. Expanded summary tables of all sociodemographic characteristics, childhood factors, and outcome variables are provided the Tables S1–S2 aggregated over the total sample and Tables S9a–S31a and S9b–S31b by country.

Country sample sizes in Wave 1 were largest in the United States (18%), Japan (9.9%), and Sweden (7.2%), and smallest in Turkey (0.7%), South Africa (1.3%), and Hong Kong (1.4%). Weighted country-specific sociodemographic and childhood characteristics are reported in Tables S9a–S31a. There was some variability in sociodemographic composition across contexts. As one illustration, the percentage of the population in India who were under 30 years of age (36% versus 11%), had completed eight or fewer years of education (72% versus 0.3%), and were married (76% versus 57%) was considerably higher than in the United States (see Tables S16a and S31a).

### Cross-country associations of forgivingness with multidimensional well-being

Standardized results from the random effects meta-analyses for the main outcomes are reported in Table 2 (see Table S8 for unstandardized results), with country-specific results presented in Tables S9g–S31g. Pooled meta-analyzed results for Model 1 suggested very small associations between forgivingness and better well-being across the countries for both the Secure Flourishing Index (*β* = 0.07) and the Flourishing Index (*β* = 0.08); psychological well-being indicators of optimism, meaningful activities, understanding one’s purpose, and self-rated mental health (*β*s = 0.05–0.06); social well-being indicators of relationship contentment, relationship satisfaction, social support, and sense of belonging in country (*β*s = 0.05–0.06); as well as character & prosocial behavior indicators of orientation to promote good, delayed gratification, hope, gratitude, and showing love/care (*β*s = 0.06–0.08).

**Table 2:** Meta-Analyzed Associations of Wave 1 Dispositional Forgivingness With Wave 2 Outcomes

Outcome	Model 1					Model 2				
	RR	ES	95% CI	τ	Global *p*-value	RR	ES	95% CI	τ	Global *p*-value
*Human Flourishing*										
Secure Flourishing Index		0.07	(0.06, 0.09)	0.03	1.7e-15***		0.02	(0.01, 0.02)	0.01	2.07e-03**
Flourishing Index		0.08	(0.06, 0.09)	0.03	1.7e-15***		0.02	(0.01, 0.02)	0.01	5.07e-04***
*Psychological Well-Being*										
Happiness		0.04	(0.03, 0.06)	0.03	2.55e-15***		−0.00	(−0.01, 0.01)	< 0.01†	0.485
Life satisfaction		0.04	(0.03, 0.05)	0.03	2.55e-15***		−0.00	(−0.01, 0.00)	< 0.01†	0.441
Current life evaluation		0.03	(0.01, 0.04)	0.03	5.11e-15***		−0.01	(−0.02, 0.00)	0.02	4.74e-03**
Future life evaluation		0.04	(0.03, 0.05)	0.03	5.11e-15***		0.00	(−0.01, 0.02)	0.02	0.022*
Optimism		0.06	(0.05, 0.07)	0.03	2.19e-15***		0.02	(0.01, 0.03)	< 0.01†	2.54e-04***
Freedom to pursue what’s important		0.04	(0.02, 0.05)	0.02	5.11e-15***		−0.01	(−0.02, −0.00)	< 0.01†	0.009*
Inner peace	1.02		(1.02, 1.03)	0.02	5.08e-15***	1.01		(1.00, 1.01)	0.01	3.79e-05***
Life balance	1.02		(1.02, 1.03)	0.01	5.11e-15***	1.01		(1.00, 1.01)	< 0.01†	1.33e-04***
Sense of mastery	1.02		(1.01, 1.02)	0.01	5.11e-15***	1.00		(1.00, 1.01)	< 0.01†	0.034*
Meaningful activities		0.05	(0.03, 0.06)	0.03	2.55e-15***		−0.00	(−0.01, 0.00)	< 0.01†	0.163
Understanding purpose		0.06	(0.04, 0.07)	0.02	2.55e-15***		0.00	(−0.00, 0.01)	< 0.01†	0.012*
Self-rated mental health		0.05	(0.03, 0.06)	0.02	4.6e-15***		0.00	(−0.00, 0.01)	< 0.01†	0.324
*Psychological Distress*										
Traumatic distress	1.00		(0.99, 1.00)	< 0.01†	0.039*	1.00		(0.99, 1.00)	< 0.01†	0.296
Depression symptoms composite	0.99		(0.99, 1.00)	< 0.01†	1.44e-08***	1.00		(1.00, 1.00)	< 0.01†	0.708
Anxiety symptoms composite	0.99		(0.99, 1.00)	< 0.01†	2.58e-10***	1.00		(1.00, 1.00)	< 0.01†	0.240
Suffering	1.00		(0.99, 1.00)	0.01	8.83e-05***	1.00		(1.00, 1.01)	< 0.01†	0.646
*Social Well-Being*										
Relationship contentment		0.06	(0.05, 0.07)	0.02	5.04e-15***		0.01	(0.01, 0.02)	< 0.01†	0.073
Relationship satisfaction		0.05	(0.04, 0.07)	0.02	5.11e-15***		0.01	(0.00, 0.01)	< 0.01†	0.508
Social support		0.05	(0.03, 0.06)	0.03	2.13e-15***		0.02	(0.01, 0.03)	0.02	5.29e-05***
Intimate/close friend	1.02		(1.01, 1.02)	< 0.01†	5.11e-15***	1.00		(1.00, 1.01)	< 0.01†	0.031*
Government approval	1.01		(1.01, 1.02)	0.01	5.46e-06***	1.00		(1.00, 1.01)	< 0.01†	0.016*
Say in government	1.01		(1.01, 1.02)	0.01	1.23e-12***	1.00		(1.00, 1.01)	< 0.01†	0.174
Belonging in country		0.05	(0.04, 0.07)	0.02	4.88e-15***		0.02	(0.01, 0.02)	< 0.01†	0.013*
City/place satisfaction	1.01		(1.01, 1.02)	0.01	2.04e-14***	1.01		(1.00, 1.01)	< 0.01†	1.07e-05***
Trust within country	1.02		(1.01, 1.03)	0.01	5.03e-15***	1.01		(1.01, 1.02)	< 0.01†	2.2e-08***
*Social Participation*										
Ever been married	1.00		(1.00, 1.00)	< 0.01†	0.937	1.00		(1.00, 1.00)	< 0.01†	0.973
Currently divorced	1.00		(1.00, 1.00)	< 0.01†	0.989	1.00		(1.00, 1.00)	< 0.01†	0.991
Number of children		0.01	(0.00, 0.01)	< 0.01†	0.724		0.01	(−0.00, 0.01)	< 0.01†	0.250
Weekly+ community participation	1.00		(1.00, 1.01)	< 0.01†	0.387	1.00		(1.00, 1.00)	< 0.01†	0.986
Weekly+ religious attendance	1.00		(1.00, 1.00)	< 0.01†	0.020*	1.00		(1.00, 1.00)	< 0.01†	0.732
*Social Distress*										
Loneliness		−0.04	(−0.05, −0.02)	0.02	5.11e-15***		0.00	(−0.00, 0.01)	< 0.01†	0.717
Perceived discrimination	1.00		(1.00, 1.01)	< 0.01†	1.9e-07***	1.00		(1.00, 1.01)	< 0.01†	1.55e-05***
*Character & Prosocial Behavior*										
Orientation to promote good		0.08	(0.07, 0.10)	0.03	1.7e-15***		0.04	(0.03, 0.05)	0.02	1.54e-12***
Delayed gratification		0.06	(0.05, 0.08)	0.03	2.55e-15***		0.03	(0.02, 0.04)	0.02	5.33e-11***
Hope		0.07	(0.06, 0.09)	0.02	1.7e-15***		0.02	(0.01, 0.03)	< 0.01†	0.016*
Gratitude		0.08	(0.06, 0.09)	0.04	2.54e-15***		0.03	(0.02, 0.04)	0.01	2.04e-07***
Showing love/care		0.08	(0.07, 0.10)	0.03	2.54e-15***		0.04	(0.03, 0.05)	< 0.01†	4.77e-06***
Forgivingness	1.13		(1.10, 1.16)	0.07	3.4e-16***	1.12		(1.09, 1.15)	0.07	4.54e-16***
Charitable giving	1.02		(1.01, 1.02)	< 0.01†	6.56e-07***	1.01		(1.00, 1.01)	< 0.01†	0.030*
Helping strangers	1.02		(1.01, 1.03)	0.01	5.11e-15***	1.01		(1.01, 1.02)	< 0.01†	1.36e-05***
Volunteering	1.01		(1.00, 1.01)	< 0.01†	2.15e-04***	1.00		(1.00, 1.00)	< 0.01†	0.451
*Physical Health & Health Behavior*										
Self-rated physical health		0.03	(0.02, 0.04)	0.02	5.11e-15***		0.00	(−0.01, 0.01)	< 0.01†	0.783
Health problems	1.00		(0.99, 1.00)	< 0.01†	0.206	1.00		(1.00, 1.00)	< 0.01†	0.824
Pain in past 4 weeks	1.00		(0.99, 1.00)	< 0.01†	0.443	1.00		(1.00, 1.00)	< 0.01†	0.748
Daily smoker	1.00		(1.00, 1.00)	< 0.01†	0.339	1.00		(1.00, 1.00)	< 0.01†	0.052
Number of drinks per week		−0.01	(−0.02, −0.00)	< 0.01†	0.380		−0.00	(−0.01, 0.00)	< 0.01†	0.776
Days exercise per week		0.03	(0.02, 0.04)	0.01	3.39e-05***		0.01	(0.00, 0.02)	0.01	0.063
*Socioeconomic Outcomes*										
Financial security		0.02	(0.01, 0.03)	0.02	6.84e-13***		−0.00	(−0.01, 0.00)	< 0.01†	0.625
Material security		0.02	(0.01, 0.04)	0.02	5.11e-15***		0.00	(−0.00, 0.01)	< 0.01†	0.109
Educational attainment (16+ years)	1.00		(1.00, 1.00)	< 0.01†	0.821	1.00		(1.00, 1.00)	< 0.01†	0.854
Currently employed	1.00		(1.00, 1.00)	< 0.01†	0.971	1.00		(1.00, 1.00)	< 0.01†	0.969
Financially comfortable/getting by	1.00		(1.00, 1.01)	< 0.01†	4.55e-07***	1.00		(0.99, 1.00)	< 0.01†	0.574
Own home	1.00		(0.99, 1.00)	< 0.01†	0.016*	1.00		(0.99, 1.00)	0.01	0.033*
Income -- top quintile	1.00		(1.00, 1.00)	< 0.01†	0.896	1.00		(1.00, 1.00)	< 0.01†	0.756

*Note. N* = 207,919; Reference for dispositional forgivingness: Never/rarely; RR, risk-ratio, null effect is 1.00; ES, effect size measure for standardized regression coefficient, null effect is 0.00; CI, confidence interval; τ (tau, heterogeneity), estimated standard deviation of the distribution of effects; Global *p*-value, joint test of the null hypothesis that the country-specific Wald tests are null in all countries.

Multiple imputation was performed to impute missing data on the covariates, exposure, and outcomes. Model 1 controlled for sociodemographic and childhood factors assessed at Wave 1: relationship with mother growing up; relationship with father growing up; parent marital status around age 12; experienced abuse growing up (except for Israel); felt like an outsider in family growing up; self-rated health growing up; subjective financial status growing up; frequency of religious service attendance around age 12; year of birth; gender; education; employment status; marital status; immigration status; religious affiliation; frequency of religious service attendance; and racial/ethnic identity when available. Model 2 controlled for all variables included in Model 1, and additionally controlled for the first seven principal components of the entire set of Wave 1 outcomes (except for dispositional forgivingness). Selected outcome variables were not assessed in certain countries, including government approval in China and Egypt, belonging in country in China, and say in government in China.

An outcome-wide analytic approach was used, and a separate model was run for each outcome. A different type of model was run depending on the nature of the outcome: (1) for each binary outcome, a weighted generalized linear model (with a log link and Poisson distribution) was used to estimate a RR; and (2) for each continuous outcome, a weighted linear regression model was used to estimate an ES. All effect sizes were standardized. For binary outcomes, the RR represents the change in risk of being in the upper category compared to the lower category between the lower and upper categories of dispositional forgivingness. For continuous outcomes, the ES represents the change (in standard deviations) on the outcome between the lower and upper categories of dispositional forgivingness.

*p*-value significance thresholds: *p* < 0.05*, *p* < 0.005**, (Bonferroni) *p* < 0.00089***, correction for multiple testing to significant threshold; †: Estimate of τ (tau, heterogeneity) is likely unstable. See forest plots in the Supplemental File for more detail on heterogeneity of effects.

Slightly weaker evidence of association was found between forgivingness and better well-being for the psychological well-being indicators of happiness, life satisfaction, current life evaluation, future life evaluation, and freedom to pursue what’s important (*β*s = 0.03–0.04); the social distress indicator of loneliness (*β* = −0.04); and the physical health & health behavior indicators of self-rated physical health and days of exercise per week (*β*s = 0.03).

Associations between forgivingness and better well-being were more negligible for the psychological well-being indicators of inner peace, life balance, and sense of mastery (RRs = 1.02); the psychological distress indicators of depression symptoms and anxiety symptoms (RRs = 0.99); the social well-being indicators of intimate/close friend, government approval, say in government, city/place satisfaction, and trust within country (RRs = 1.01–1.02); the social participation indicator of number of children (*β* = 0.01); the character & prosocial behavior indicators of charitable giving, helping strangers, and volunteering (RRs = 1.01–1.02); the physical health & health behavior indicator of number of drinks per week (*β* = −0.01); and the socioeconomic indicators of financial security and material security (*β*s = 0.02). There was relatively little evidence of association between forgivingness and the other main outcomes in Model 1 when estimates were pooled across countries.

When applying a more conservative analytic approach to confounding control that additionally adjusted for the seven principal components extracted from Wave 1 outcomes (i.e., Model 2), we observed a general attenuation of the associations between forgivingness and the well-being outcomes (see Table 2). For example, forgivingness evidenced one of its strongest associations with orientation to promote good in Model 1. After adjusting for principal components extracted from Wave 1 outcomes, the association attenuated from *β* = 0.08 to 0.04. Although it is not possible to discern whether such attenuation is because adjustment for prior values of the outcomes controls for confounding that was not adequately addressed by the set of sociodemographic and childhood covariates adjusted for in Model 1 or because one or more of the prior values of the outcomes from Wave 1 are on the pathway from Wave 1 forgivingness to one or more of the Wave 2 outcomes^62^, some combination of these possibilities seems plausible. Even with a more conservative analytic approach and attenuated associations for Model 2, many outcomes for which there was some evidence of association in Model 1 also showed some evidence of association in Model 2. We tentatively suggest that the actual estimates of association for each outcome might be somewhere between the results for Model 1 and Model 2, in which case forgivingness is likely meaningfully associated with many outcomes for which there was evidence of an association in Model 1.

Results of the *E*-value sensitivity analysis for the primary random effects meta-analyses involving the main outcomes are reported in Table 3. For Model 1, *E*-values corresponding with the point estimates (not including the association of Wave 1 forgivingness with Wave 2 forgivingness) ranged from 1.01–1.37, with somewhat smaller *E*-values for the confidence interval limit (1.00–1.32). For example, to explain away the observed association between Wave 1 forgivingness and Wave 2 orientation to promote good, an unmeasured confounder that was jointly associated with both of these variables by risk ratios of 1.37-fold each (above and beyond the sociodemographic and childhood variables adjusted for in the model) could do so, but weaker joint confounder associations could not. For the limit of the confidence interval, unmeasured confounder risk ratio associations of 1.32 for Wave 1 forgivingness and Wave 2 orientation to promote good could shift the confidence interval to include the null, but weaker joint confounder associations could not.

**Table 3:** E-Value Sensitivity Analysis for Meta-Analyzed Associations of Wave 1 Dispositional Forgivingness With Wave 2 Outcomes

Outcome	Model 1		Model 2	
	*E*-value	*E*-value for CI	*E*-value	*E*-value for CI
*Human Flourishing*				
Secure Flourishing Index	1.34	1.29	1.14	1.09
Flourishing Index	1.35	1.31	1.14	1.10
*Psychological Well-Being*				
Happiness	1.24	1.18	1.02	1.00
Life satisfaction	1.23	1.18	1.05	1.00
Current life evaluation	1.18	1.12	1.08	1.00
Future life evaluation	1.23	1.18	1.07	1.00
Optimism	1.30	1.25	1.15	1.12
Freedom to pursue what’s important	1.22	1.17	1.09	1.01
Inner peace	1.17	1.14	1.10	1.06
Life balance	1.17	1.14	1.09	1.06
Sense of mastery	1.15	1.12	1.07	1.04
Meaningful activities	1.26	1.21	1.06	1.00
Understanding purpose	1.29	1.25	1.06	1.00
Self-rated mental health	1.26	1.21	1.06	1.00
*Psychological Distress*				
Traumatic distress	1.03	1.00	1.02	1.00
Depression symptoms composite	1.09	1.04	1.05	1.00
Anxiety symptoms composite	1.11	1.07	1.05	1.00
Suffering	1.07	1.00	1.05	1.00
*Social Well-Being*				
Relationship contentment	1.30	1.26	1.12	1.08
Relationship satisfaction	1.28	1.24	1.09	1.02
Social support	1.26	1.20	1.15	1.09
Intimate/close friend	1.14	1.11	1.07	1.05
Government approval	1.13	1.09	1.06	1.03
Say in government	1.13	1.09	1.06	1.00
Belonging in country	1.28	1.24	1.14	1.11
City/place satisfaction	1.13	1.10	1.09	1.04
Trust within country	1.16	1.13	1.12	1.09
*Social Participation*				
Ever been married	1.04	1.02	1.04	1.00
Currently divorced	1.01	1.00	1.01	1.00
Number of children	1.09	1.03	1.09	1.00
Weekly+ community participation	1.07	1.05	1.03	1.00
Weekly+ religious attendance	1.05	1.03	1.02	1.00
*Social Distress*				
Loneliness	1.22	1.17	1.04	1.00
Perceived discrimination	1.04	1.00	1.03	1.00
*Character & Prosocial Behavior*				
Orientation to promote good	1.37	1.32	1.24	1.20
Delayed gratification	1.30	1.25	1.20	1.15
Hope	1.34	1.30	1.16	1.12
Gratitude	1.35	1.29	1.20	1.16
Showing love/care	1.36	1.32	1.23	1.20
Forgivingness	1.51	1.43	1.49	1.42
Charitable giving	1.14	1.11	1.08	1.06
Helping strangers	1.17	1.14	1.12	1.08
Volunteering	1.10	1.06	1.02	1.00
*Physical Health & Health Behavior*				
Self-rated physical health	1.19	1.14	1.04	1.00
Health problems	1.05	1.00	1.03	1.00
Pain in past 4 weeks	1.05	1.00	1.02	1.00
Daily smoker	1.01	1.00	1.05	1.02
Number of drinks per week	1.11	1.07	1.06	1.00
Days exercise per week	1.18	1.13	1.11	1.04
*Socioeconomic Outcomes*				
Financial security	1.15	1.09	1.04	1.00
Material security	1.17	1.12	1.07	1.00
Educational attainment (16+ years)	1.00	1.00	1.00	1.00
Currently employed	1.01	1.00	1.03	1.00
Financially comfortable/getting by	1.04	1.00	1.06	1.01
Own home	1.06	1.01	1.06	1.00
Income -- top quintile	1.04	1.00	1.05	1.00

*Note*. CI, confidence interval. The formula for calculating *E*-values can be found in VanderWeele and Ding^68^. *E*-values for estimate are the minimum strength of association on the risk ratio scale that an unmeasured confounder would need to have with both the exposure and the outcome to fully explain away the observed association between them, conditional on the measured covariates. *E*-values for the 95% CI closest to the null denote the minimum strength of association on the risk ratio scale that an unmeasured confounder would need to have with both the exposure and the outcome to shift the CI to include the null value, conditional on the measured covariates.

As a reference point for considering the plausibility of unmeasured confounding, Model 1 results suggested that endorsing ‘often/always’ forgiving others at Wave 1 was associated with a 13% higher likelihood of endorsing ‘often/always’ forgiving others at Wave 2 on average across countries, after controlling for sociodemographic characteristics and childhood factors. This suggests that an unmeasured confounder would need to be jointly associated with forgivingness and the outcome of interest by risk ratios of at least 1.13 each to fully explain away the observed association—comparable in strength to the longitudinal association of forgivingness with itself. Many of the *E*-values for the effect estimates in Model 1 exceeded this threshold, suggesting that some of those observed associations may be potentially robust to unmeasured confounding; it seems unlikely that an unmeasured variable would be as strongly related to both Wave 1 forgivingness and an outcome as forgivingness is to itself over time. However, *E*-values for Model 2 (point estimate: 1.01–1.24, confidence interval limit: 1.00–1.20) were slightly smaller than those observed for Model 1, reflecting weaker evidence of associations in Model 2 after additionally adjusting for principal components extracted from Wave 1 outcomes and a higher susceptibility of Model 1 to unmeasured confounding because fewer covariates were adjusted for.

When we repeated the meta-analyses using a semi-complete case approach with attrition weights for both Models 1 and 2 (see Tables S5 and S6, respectively, as well as Table S7 for corresponding *E*-values), estimates of association were either the same or differed only marginally from those observed for the random effects meta-analyses with multiply imputed data. These findings suggest that the results are not highly sensitive to the approach used to handle missing data.

### Similarities and differences across countries

In addition to the meta-analytic pooled estimates of the associations, Table 2 also presents global *p*-values and tau (*τ*) values that provide evidence of cross-national heterogeneity in the associations between forgivingness and each subsequent outcome for Models 1 and 2. A more detailed description of the heterogeneity of these associations can be found in the forest plots displaying country-specific point estimates and corresponding 95% CIs for outcomes (see Figures S1–S78). Most global *p*-values for Model 1 passed the Bonferroni-corrected threshold, providing robust evidence of an association between forgivingness and a given outcome in at least one country (somewhat fewer global *p*-values passed the Bonferroni-corrected threshold for Model 2). Tau values for Model 1 were mostly below 0.03 (and were generally smaller for Model 2), suggesting modest heterogeneity across the samples.

Country-specific standardized results for associations between forgivingness and the subsequent outcomes are reported in Tables S9g–S31g (see also Tables S34 and S35), with corresponding *E*-values presented in Tables S9i–S31i. Accompanying forest plots provide a visual display of country-specific associations ordered on the y-axis by magnitude of association (see Figures S1–S78). Although we did not find clear evidence of an association between forgivingness and a main outcome that was universal across all samples in Model 1, forgivingness was associated with better well-being across at least half of the samples on the Flourishing Index (20/23 samples at *p* < 0.05, 13/23 samples at *p* < 0.00089), Secure Flourishing Index (19/23, 11/23), showing love/care (19/23, 12/23), orientation to promote good (17/23, 12/23), hope (16/23, 10/23), gratitude (16/23, 10/23), delayed gratification (15/23, 8/23), relationship satisfaction (15/23, 5/23), relationship contentment (14/23, 6/23), helping strangers (14/23, 7/23), understanding one’s purpose (13/23, 6/23), optimism (12/23, 7/23), meaningful activities (12/23, 6/23), future life evaluation (12/23, 4/23), and sense of belonging in country (12/22, 5/22). Many of these outcomes were among those most strongly associated with forgivingness in the meta-analyses. Even in these cases where the pattern of associations for a given outcome was more consistent across samples, estimates of association in each sample varied to some extent. The domains in which forgivingness tended to show the most consistent associations (*p* < 0.05) with better well-being on the outcomes across the samples included psychological well-being (ranging from seven samples for current life evaluation to 13 samples for understanding one’s purpose), social well-being (ranging from four samples for say in government to 15 samples for relationship satisfaction), and character & prosocial behavior (ranging from five samples for volunteering to 19 samples for showing love/care).

Most outcomes for which there was little evidence of association with forgivingness when estimates were pooled meta-analytically (e.g., educational attainment, currently employed) were not predicted by forgivingness in any of the samples. There were also instances in which pooled estimates across the samples did not suggest evidence of an association with a given outcome, but evidence of such an association was found in certain samples. For example, there were four countries in which forgivingness was associated with a very small decrease in subsequent suffering (i.e., Brazil, Japan, Sweden, the United States), three of which passed the Bonferroni-corrected threshold of *p* < 0.00089. There were only a few instances in which forgivingness was associated with worse subsequent well-being on selected outcomes in certain countries, such as higher perceived discrimination in China and Poland, higher traumatic distress in the Philippines, higher daily smoking in the United States, and a lower likelihood of owning a home in Sweden, although magnitudes of association were mostly very small and only one (i.e., perceived discrimination in China) passed the Bonferroni-corrected threshold of *p* < 0.00089.

The samples in which forgivingness showed evidence of associations with better well-being on the greatest number of outcomes (not including forgivingness itself) included the United States (46/55 outcomes at *p* < 0.05, 39/55 outcomes at *p* < 0.00089), Japan (41/55, 40/55), Sweden (36/55, 32/55), Brazil (30/55, 14/55), Germany (30/55, 13/55), and the United Kingdom (29/55, 16/55). The samples with the fewest associations included South Africa (1/55 outcomes at *p* < 0.05, 0/55 outcomes at *p* < 0.00089), Hong Kong (4/55, 0/55), Indonesia (5/55, 0/55), Nigeria (5/55, 0/55), Turkey (6/55, 0/55), and Egypt (6/54, 0/54). Country-specific results for Model 2 showed a similar pattern to Model 1, except that estimates of association were somewhat attenuated (see Tables S9g–S31g). Estimates of association using imputed data were also similar to those observed after repeating the country-specific analyses using a semi-complete case approach with attrition weights for both Models 1 and 2 (see Tables S9h–S31h).

## Discussion

Working with two waves of longitudinal data from a diverse set of 23 countries in the GFS, this study is among the first to estimate population-level associations of dispositional forgivingness with multidimensional well-being assessed approximately one year later. We observed two main findings. First, pooled estimates across the 23 samples provided some evidence of association between forgivingness and better well-being on composite indicators of well-being and many specific indicators of well-being on almost all domains that were examined, although stronger and more consistent evidence of association was observed for outcomes on some domains (e.g., psychological well-being) compared to others (e.g., socioeconomic outcomes). Second, we found evidence of cross-national similarities and differences in the pattern of associations between forgivingness and the well-being outcomes, with some outcomes showing more consistent evidence of association across the countries (e.g., the United States) than others (e.g., South Africa). By leveraging a multinational dataset with roughly nationally representative samples that collectively represent about two-thirds of the global population, our findings provide unique insights into how the potential benefits of forgivingness for well-being might generalize (or diverge) across a variety of socioecological contexts around the world.

Pooled estimates for the main outcomes in Model 1 of our primary analyses offered some evidence supporting associations between forgivingness and better well-being on both composite well-being indicators and a variety of specific well-being indicators across most domains. Although the observed associations were generally very small to more negligible (and attenuated when more extensive confounding control was applied in Model 2), this pattern of findings aligns with some theoretical and empirical work that suggests forgiveness of others may have implications for whole person functioning^4,16^. Our outcome-wide analytic approach contributes to a small but growing number of studies that have applied a holistic and integrative lens toward building a more complete understanding of how forgiving others might be related to well-being^10,20,25^, which we extend by considering a wider range of outcomes in a diverse multinational sample. For example, Chen et al.^20^ performed one of the most expansive outcome-wide studies on forgiving others to date, examining associations of spiritually-motivated forgivingness with 25 well-being outcomes in a sample of young United States adults; our study examined over twice as many, a number of which (e.g., life balance, showing love/care) have been underexplored in prior research. Our findings also help to fill a notable gap in the literature by shedding light on potential population-level implications of forgivingness for well-being in contexts (e.g., Nigeria, Tanzania) where relatively few rigorous empirical studies along these lines have been conducted previously, expanding the cultural inclusivity and representativeness of research on forgiveness.

Results of our primary analyses also point to heterogeneity in the pattern of associations across the outcomes, suggesting that forgivingness may be more strongly related to some aspects of well-being than others (at least over a timeframe of approximately one year). In particular, we observed slightly stronger (though still very small in magnitude) evidence of associations for outcomes on the domains of psychological well-being (e.g., optimism, meaningful activities), social well-being (e.g., relationship satisfaction, social support), and character & prosocial behavior (e.g., orientation to promote good, delayed gratification) compared to other domains (e.g., physical health & health behavior, socioeconomic outcomes). These findings share some common ground with existing literature, including robust evidence that suggests forgiving others tends to be more salient and immediately beneficial to psychological and social dimensions of functioning compared to the physical dimension—especially if the emphasis is on more distal physical well-being outcomes (e.g., general health problems) that usually take time to manifest^19,33^. Other findings were less consistent with trends in prior research, such as little evidence of association between forgivingness and psychological distress outcomes. Somewhat contrasting findings for psychological distress and psychological well-being might be understood through principles of broaden-and-build theory^77^, which suggests that the positive emotions associated with forgiving others (e.g., compassion, empathy) might contribute to building cognitive-emotional resources that enhance psychological well-being to a greater extent than reduce psychological distress.

Our findings also point to the utility of simultaneously considering a range of outcomes across different dimensions of well-being to understand the broad impact of forgivingness. For example, many of the associations for outcomes on the character & prosocial behavior domain were similar in magnitude to those found for outcomes on domains that tend to receive more widespread attention in the empirical literature on forgiveness of others (e.g., psychological well-being). While these findings align with theory and research that suggest forgiveness of others can contribute to or reinforce prosocial values, moral commitments, and virtues implicated in volitional well-being^6,78^, our outcome-wide approach points to the importance of affording these benefits equal consideration alongside more traditionally emphasized well-being outcomes. Although the outcome-wide approach has its limitations (e.g., it leans more toward hypothesis generation than hypothesis testing), it offers a powerful framework for identifying potential cross-domain effects that might otherwise be overlooked.

The United Nations has specified ‘good health and well-being’ as one of 17 Sustainable Development Goals. Progress toward this goal, both in the short term and beyond 2030, requires prevention and promotion efforts to support well-being. Although the associations we observed were generally modest in magnitude, our findings align with the assertion that forgiveness could play a role in promoting population-level well-being if evidence-based resources that encourage the general population to explore and practice forgiveness (when safe and appropriate) can be disseminated widely^7^. One promising option is the brief self-directed REACH Forgiveness workbook (https://www.discoverforgiveness.org/tools/the-reach-forgiveness-workbook), which demonstrated large effects on forgiveness, mental health, and composite well-being in a recent multisite randomized trial across five countries^22^. While this do-it-yourself workbook should not be considered a replacement for treatment from qualified healthcare professionals when needed and its benefits are likely to vary across individuals, this freely available resource has the potential to reach and provide support to many people around the world at low cost. Recent applied work in which forgiveness has been effectively promoted through awareness-raising campaigns within large communities (e.g., university campuses) may offer some possible templates for disseminating forgiveness resources within towns, cities, states, and perhaps even nationally^79,80^. Large-scale forgiveness campaigns could promote societal flourishing, and might even be embraced as part of a public health agenda^7^. However, efforts to disseminate and promote forgiveness resources should be sensitive to concerns of justice, accountability, and reconciliation that can complicate processing of forgiveness.

Probing country-specific results revealed some cross-national similarities and differences in the pattern of associations. When evidence of association was observed in a given country, forgivingness was associated with better well-being in almost every case (with only a few exceptions in certain countries). Outcomes that were most consistently associated with forgivingness across the countries included both composite well-being indicators, several indicators of character & prosocial behavior (e.g., showing love/care, orientation to promote good), as well as selected indicators of psychological well-being (e.g., understanding one’s purpose, optimism) and social well-being (e.g., relationship satisfaction, sense of belonging in country). These findings suggest that forgiveness may have a near-universal role in supporting aspects of well-being, perhaps reflecting its widespread cultural importance^81^.

However, our cross-national findings also suggested that the extent of benefits to well-being may not be consistent across all sociocultural contexts. To illustrate, forgivingness showed some evidence of association with a majority of well-being outcomes in the United Kingdom, but it showed evidence of association with very few outcomes in Nigeria. Although there may be several reasons for such cross-national variation (e.g., differences in population sociodemographics across countries), one possibility is that the implications of forgivingness for well-being might be reinforced or constrained depending on the broader socioecological context in which people live^16,38^. Many of the countries in which we observed more limited evidence of association between forgivingness and the outcomes (e.g., Egypt, South Africa) face social-structural vulnerabilities (e.g., political instability, economic inequality) that may blunt or overpower some of the benefits of forgiving others for well-being. Thus, although forgiveness typically provides a boost to psychological well-being (e.g., inner peace, greater balance), such benefits might be overwhelmed by chronic or new stressors that a person encounters within the sociocultural context in which they live^21,82^.

There was also a general trend toward more limited evidence of association between forgivingness and well-being in countries where endorsement of forgivingness tended to be higher, suggesting somewhat stronger linkages to well-being in countries where processing of forgiveness might be a less common strategy for dealing with interpersonal hurts or offenses. For example, very few outcomes showed evidence of association with forgivingness in the three countries with the highest Wave 1 forgivingness (i.e., Nigeria, Egypt, Indonesia), whereas several countries with comparatively low Wave 1 forgivingness (e.g., Japan, the United Kingdom) showed evidence of association with numerous outcomes. In cultures where forgiveness is highly normative and widely practiced, it is possible that forgiveness of others might show weaker population-average associations with well-being because its benefits are already saturated within the culture, variability in forgivingness is lower, and/or strong cultural norms tend to impose forgiveness as an obligation rather than a personal choice^83^. Although further work is needed to explore how different sociocultural features might influence when and why forgivingness is more (or less) strongly associated with well-being in particular contexts, our findings suggest that population-level efforts to promote forgiveness should carefully attend to the unique sociocultural landscape of each context.

A key strength of this study is the use of longitudinal samples from a substantially diverse set of 23 countries and territories that are roughly representative of the populations from which they were drawn. However, some cultures and contexts are not represented in the GFS. Some caution should be applied when attempting to generalize the findings beyond the countries in our analytic sample, and further research is needed in nations that are not currently represented in the GFS.

We used single items to assess all psychosocial covariates, the exposure of forgivingness, and almost all outcomes. While it is common for single items to be prioritized in large-scale epidemiologic research (such as the GFS) because they offer an efficient and uniform approach to assessment under the resource constraints of conducting multinational research, there are trade-offs to consider. For example, single items can effectively capture the essence of a construct, but they may lack conceptual breadth and cultural sensitivity^84^. The single-item measure of forgivingness used in this study is grounded in prior published work and offers a pragmatic approach to assessing the tendency to forgive in large-scale multinational research, yet it may not fully capture the multidimensional nature of forgiveness or be sufficiently sensitive to cross-cultural differences in how forgiveness is understood and expressed. There might also be variation in measurement error across the samples. For example, while some of the smallest estimates of association were observed in Nigeria, other analyses also showed that associations with Wave 2 flourishing tended to be smallest in Nigeria when averaged over all Wave 1 exposures, possibly pointing to issues with the quality of data in Nigeria^45^. Future research could build on our findings by employing measures that provide broader conceptual coverage of the psychosocial constructs that were assessed.

This study is based entirely on self-report data, which could be affected by different forms of response bias (e.g., socially desirable responding). Although our findings contribute to strengthening knowledge about linkages between forgivingness and subjective indicators of well-being at the population level, additional longitudinal research is needed using more objective indicators. For example, future work could consider physiological indicators of physical health (e.g., blood pressure, heart rate variability) or standardized clinical assessments of mental health to corroborate and extend the findings reported herein.

The attrition rate was nearly 40% overall, but was quite varied across the 23 samples (ranging from a low of 9% to a high of 80%). Our primary analytic models were based on multiply imputed data, which has its advantages (e.g., preservation of roughly nationally representative sample structure, reduced risk of attrition bias). However, factors that have the potential to bias results cannot be fully addressed by multiple imputation. For example, our results may be biased if participants in both waves differ systematically from Wave 2 non-respondents on unobserved characteristics. Although we did not observe substantial differences in the total sample between Wave 1 and 2 responders on the measured variables and the results from our semi-complete case analysis with attrition weights were similar to the results from our primary analyses, we cannot rule out the possibility that attrition may have biased the results and weakened the generalizability and cross-cultural comparability of our findings.

In Model 1 of our primary analyses, covariates included a set of sociodemographic and childhood factors that could reasonably be ruled out as not being on the pathway from Wave 1 forgivingness to Wave 2 outcomes. However, the results for Model 1 could still be biased away from the null because the set of covariates may not have provided adequate confounding control, though *E*-values suggested that many of the observed associations for Model 1 might be somewhat robust to unmeasured confounding. After applying a more conservative approach to confounding control in Model 2 when additional adjustment was made for seven principal components extracted from Wave 1 outcomes treated as covariates, we found that estimates of association were generally smaller than those observed for Model 1. With all covariates taken from the same wave in which forgivingness was assessed, we are unable to determine whether attenuation of associations in Model 2 is due to more extensive confounding control or because adjustment was made for variables that might be on the pathway between Wave 1 forgivingness and one or more Wave 2 outcomes^62^. Some combination of these possibilities seems likely, in which case the actual effect estimate between forgivingness and each outcome might be somewhere between the associations observed for Model 1 and Model 2. Longitudinal data with more than two waves would help clarify these associations by temporally disentangling covariates from the exposure and mitigating concerns about potential bidirectional effects.

About half of our main outcomes were analyzed as binary variables. A number of these were originally assessed using ordinal response scales with four or fewer response options. Recoding decisions were made a priori on the basis of several practical and interpretive reasons, including the goals of maintaining consistency with coding used in a coordinated set of Wave 1 analyses^85,86^ and facilitating comparability across other coordinated outcome-wide studies using the same longitudinal GFS data^44^. Although binary outcomes can produce effect estimates that are easier to interpret—particularly for non-technical audiences and when policy considerations are in view—dichotomization has its drawbacks. For example, dichotomizing an outcome reduces its variability, which can diminish its sensitivity to the effect of an exposure. This may have contributed to the generally weaker associations that we observed between forgivingness and the binary outcomes in this study.

The outcome-wide analytic approach used in this study involved estimating population-average associations between forgivingness and well-being outcomes assessed approximately one year later. While our findings take an important step forward toward developing a more integrative and well-rounded picture of the potential benefits of forgivingness for population-level well-being, further work is needed to identify the boundary conditions (i.e., when and for whom) under which associations might vary and the mediating mechanisms (i.e., how and why) that might explain the associations that were observed. Our exploration of potential cross-country heterogeneity in the associations between forgivingness and well-being outcomes lays the groundwork for subsequent work aimed at identifying sources of heterogeneity, including national-level factors (e.g., economic indicators, cultural values, national religiosity) that may explain variation in associations across countries.

In summary, this multinational study provided some population-level evidence suggesting that forgivingness is associated with better subsequent multidimensional well-being both within and across a set of culturally and geographically diverse countries. Although observed associations were generally very modest in magnitude and the two-wave design precludes definitive causal conclusions, our findings suggest that forgivingness may have important implications for population well-being. The population-average associations reported in this study set the stage for more granular follow-up studies to understand potential boundary conditions and mediating mechanisms that can inform the next frontier of forgiveness theory, research, and practice.

## Supplementary information

Supplemental File

## Data Availability

The data are publicly available through the Center for Open Science (https://www.cos.io/gfs). The research questions, variables, and analyses for the current study were preregistered with the Center for Open Science prior to accessing data (https://osf.io/kyvze). All code to reproduce analyses is openly available in an online repository (10.17605/osf.io/rbcmp)^61^.
