# Supplementary material for: Longitudinal associations of dispositional forgivingness with multidimensional well-being: a two-wave outcome-wide analysis in the Global Flourishing Study
Source: Npj Ment Health Res. 2026 Jan 21;5:3. doi: 10.1038/s44184-026-00187-5 (PMC12823676; doi:10.1038/s44184-026-00187-5)
Supplement: Supplementary file 1 — Supplemental File [file 44184_2026_187_MOESM1_ESM.pdf]

Longitudinal Associations of Dispositional Forgivingness with Multidimensional Well-being: A Two-Wave Outcome-Wide Analysis in the Global Flourishing Study

SUPPLEMENTAL FILE

- Summary statistics of demographic variables, childhood variables, and outcome variables by wave or attrition status (Tables S1-S4).
- Supplemental meta-analytic results (Tables S5-S8).
- Country-specific results—contains 9 tables per country:
  - Weighted summary statistics of demographic and childhood variables at Waves 1 & 2 (Tables S9a-S31a).
  - Weighted summary statistics of outcome variables at Waves 1 & 2 (Tables S9b-S31b).
  - Unweighted summary statistics of demographic and childhood variables at Wave 1 by retention status (Tables S9c-S31c).
  - Unweighted summary statistics of all outcomes at Wave 1 by retention status (Tables S9d-S31d).
  - Summary of fitted attrition model (Tables S9e-S31e).
  - Summary of principal components (Tables S9f-S31f).
  - Outcome-wide results using multiple imputation for Model 1 & Model 2 (Tables S9g-S31g).
  - Outcome-wide results using attrition weights for Model 1 & Model 2 (Tables S9h-S31h).
  - *E*-value sensitivity of results to unmeasured confounding (Tables S9i-S31i).
- Summary table of demographic and childhood variables (Table S32) and summary of outcomes across countries and waves for comparison (Table S33).
- Summary table of country-specific results combined into single summary tables for each model (Model 1 results in Table S34; Model 2 results in Table S35).
- Forest plots illustrating the heterogeneity in associations across countries (Figures S1-S78).

Table S1. Weighted summary statistics for demographic and childhood variables.

**Characteristic**	**Wave 1**<br>N = 207,919	**Wave 2**<br>N = 128,868
Forgivingness, n (%)		
Always	61,254 (29.5%)	32,316 (25.1%)
Often	93,572 (45.0%)	61,616 (47.8%)
Rarely	43,112 (20.7%)	28,600 (22.2%)
Never	9,266 (4.5%)	5,874 (4.6%)
(Missing)	714 (0.3%)	461 (0.4%)
Year of birth, n (%)		
1943 or earlier (current age: 80+ years)	4,047 (1.9%)	3,445 (2.7%)
1943-1953 (current age: 70-79 years)	16,902 (8.1%)	12,684 (9.8%)
1953-1963 (current age: 60-69 years)	29,031 (14.0%)	19,538 (15.2%)
1963-1973 (current age: 50-59 years)	32,409 (15.6%)	20,495 (15.9%)
1973-1983 (current age: 40-49 years)	34,970 (16.8%)	21,996 (17.1%)
1983-1993 (current age: 30-39 years)	40,297 (19.4%)	24,641 (19.1%)
1993-1998 (current age: 25-29 years)	20,325 (9.8%)	12,309 (9.6%)
1998-2005 (current age: 18-24 years)	29,920 (14.4%)	13,760 (10.7%)
(Missing)	18 (<0.0%)	1 (<0.0%)
Age of participant		
Mean	44.8	47.0
Standard Deviation	17.5	17.5
Min, Max	18.0, 99.0	18.0, 99.0
(Missing)	18 (<0.1%)	1 (<0.1%)
Gender, n (%)		
Male	100,661 (48.4%)	62,160 (48.2%)
Female	106,349 (51.1%)	66,141 (51.3%)
Other	523 (0.3%)	370 (0.3%)
(Missing)	386 (0.2%)	197 (0.2%)
Respondent marital status, n (%)		
Single/Never been married	53,367 (25.7%)	30,380 (23.6%)
Married	110,663 (53.2%)	70,924 (55.0%)
Separated	5,094 (2.4%)	3,171 (2.5%)
Divorced	11,940 (5.7%)	8,112 (6.3%)
Widowed	9,935 (4.8%)	6,476 (5.0%)
Domestic partner	15,112 (7.3%)	8,922 (6.9%)
(Missing)	1,808 (0.9%)	883 (0.7%)
Education (years), n (%)		
Up to 8	46,842 (22.5%)	22,657 (17.6%)
9-15	116,015 (55.8%)	72,942 (56.6%)
16+	44,904 (21.6%)	33,258 (25.8%)
(Missing)	158 (0.1%)	11 (<0.0%)
Employment status, n (%)		
Employed for an employer	81,157 (39.0%)	54,273 (42.1%)
Self-employed	36,901 (17.7%)	21,050 (16.3%)
Retired	30,456 (14.6%)	21,673 (16.8%)
Student	11,505 (5.5%)	5,024 (3.9%)
Homemaker	21,897 (10.5%)	11,073 (8.6%)
Unemployed and looking for a job	16,673 (8.0%)	8,872 (6.9%)
None of these/Other	8,534 (4.1%)	5,933 (4.6%)
(Missing)	796 (0.4%)	969 (0.8%)
Current religious service attendance, n (%)		
More than once a week	26,507 (12.7%)	14,615 (11.3%)
Once a week	39,833 (19.2%)	21,979 (17.1%)

Table S1. Weighted summary statistics for demographic and childhood variables.

**Characteristic**	**Wave 1**<br>N = 207,919	**Wave 2**<br>N = 128,868
One to three times a month	20,084 (9.7%)	11,278 (8.8%)
A few times a year	42,064 (20.2%)	26,286 (20.4%)
Never	78,707 (37.9%)	54,127 (42.0%)
(Missing)	723 (0.3%)	584 (0.5%)
Immigration status, n (%)		
Born in this country	196,161 (94.3%)	121,266 (94.1%)
Born in another country	9,687 (4.7%)	6,450 (5.0%)
(Missing)	2,072 (1.0%)	1,152 (0.9%)
Parental marital status around age 12, n (%)		
Parents were married	157,017 (75.5%)	99,322 (77.1%)
Parents were divorced	17,862 (8.6%)	11,800 (9.2%)
Parents were never married	15,193 (7.3%)	8,527 (6.6%)
One or both of them had died	7,933 (3.8%)	4,730 (3.7%)
Unsure	2,556 (1.2%)	1,269 (1.0%)
(Missing)	7,359 (3.5%)	3,221 (2.5%)
Religious service attendance around age 12, n (%)		
At least once a week	83,764 (40.3%)	50,167 (38.9%)
One to three times a month	33,771 (16.2%)	20,002 (15.5%)
Less than once a month	37,340 (18.0%)	23,647 (18.3%)
Never	51,069 (24.6%)	33,916 (26.3%)
(Missing)	1,975 (0.9%)	1,136 (0.9%)
Relationship with mother when growing up, n (%)		
Very good	130,141 (62.6%)	78,006 (60.5%)
Somewhat good	54,797 (26.4%)	35,852 (27.8%)
Somewhat bad	11,235 (5.4%)	7,775 (6.0%)
Very bad	4,435 (2.1%)	2,931 (2.3%)
(Does not apply)	6,353 (3.1%)	3,761 (2.9%)
(Missing)	958 (0.5%)	544 (0.4%)
Relationship with father when growing up, n (%)		
Very good	109,545 (52.7%)	64,862 (50.3%)
Somewhat good	58,172 (28.0%)	38,073 (29.5%)
Somewhat bad	16,097 (7.7%)	11,100 (8.6%)
Very bad	8,278 (4.0%)	5,380 (4.2%)
(Does not apply)	14,447 (6.9%)	8,739 (6.8%)
(Missing)	1,379 (0.7%)	715 (0.6%)
Felt like an outsider in family when growing up, n (%)		
Yes	28,936 (13.9%)	18,511 (14.4%)
No	175,363 (84.3%)	108,222 (84.0%)
(Missing)	3,620 (1.7%)	2,135 (1.7%)
Experienced abuse when growing up, n (%)		
Yes	29,156 (14.0%)	19,037 (14.8%)
No	172,270 (82.9%)	106,222 (82.4%)
(Missing)	6,493 (3.1%)	3,610 (2.8%)
Self-rated health when growing up, n (%)		
Excellent	68,598 (33.0%)	43,616 (33.8%)
Very good	65,141 (31.3%)	40,812 (31.7%)
Good	48,478 (23.3%)	29,209 (22.7%)
Fair	20,231 (9.7%)	12,049 (9.4%)
Poor	4,933 (2.4%)	2,856 (2.2%)
(Missing)	539 (0.3%)	325 (0.3%)
Subjective financial status of family growing up, n (%)		

Table S1. Weighted summary statistics for demographic and childhood variables.

Characteristic	Wave 1<br>N = 207,919	Wave 2<br>N = 128,868
Lived comfortably	72,541 (34.9%)	44,531 (34.6%)
Got by	85,508 (41.1%)	54,279 (42.1%)
Found it difficult	36,515 (17.6%)	22,213 (17.2%)
Found it very difficult	12,684 (6.1%)	7,487 (5.8%)
(Missing)	672 (0.3%)	358 (0.3%)
Religious affiliation growing up, n (%)		
Christianity	119,860 (57.6%)	75,376 (58.5%)
Taoism	224 (0.1%)	140 (0.1%)
Confucianism	73 (0.0%)	43 (0.0%)
Primal, Animist, or Folk religion	420 (0.2%)	198 (0.2%)
Spiritism	333 (0.2%)	86 (0.1%)
Umbanda, Candomblé, and other African-derived religions	262 (0.1%)	76 (0.1%)
Chinese folk/traditional religion	243 (0.1%)	157 (0.1%)
Islam	23,946 (11.5%)	12,369 (9.6%)
Hinduism	10,815 (5.2%)	5,414 (4.2%)
Buddhism	7,946 (3.8%)	5,410 (4.2%)
Judaism	3,956 (1.9%)	2,811 (2.2%)
Sikhism	233 (0.1%)	93 (0.1%)
Baha'i	35 (0.0%)	25 (0.0%)
Jainism	30 (0.0%)	20 (0.0%)
Shinto	427 (0.2%)	268 (0.2%)
Some other religion	746 (0.4%)	512 (0.4%)
No religion/Atheist/Agnostic	37,292 (17.9%)	25,301 (19.6%)
(Missing)	1,078 (0.5%)	569 (0.4%)

Note. N (%); this table is based on non-imputed data. Cumulative percentages for variables may not add up to 100% due to rounding. Wave 1 characteristics weighted using the Gallup provided sampling weight, ANNUAL_WEIGHT_R2; Wave 2 characteristics weighted accounting for attrition by using the adjusted Wave 1 weight, ANNUAL_WEIGHT_R2, multiplied by the created attrition weight to account for dropout, to maintain nationally representative estimates for Wave 2 characteristics.

Table S2. Weighted summary statistics for outcome variables by Wave.

Outcome	Wave 1<br>N = 207,919	Wave 2<br>N = 128,868
Secure flourishing index		
Mean	7.1	7.0
Standard Deviation	1.7	1.7
Min, Max	0.0, 10.0	0.0, 10.0
(Missing)	5,665 (2.7%)	3,831 (3.0%)
Flourishing index		
Mean	7.3	7.1
Standard Deviation	1.7	1.7
Min, Max	0.0, 10.0	0.0, 10.0
(Missing)	5,092 (2.4%)	3,542 (2.7%)
Happiness & life satisfaction		
Mean	6.9	6.7
Standard Deviation	2.2	2.2
Min, Max	0.0, 10.0	0.0, 10.0
(Missing)	1,048 (0.5%)	584 (0.5%)
Physical & mental health		
Mean	7.3	7.1
Standard Deviation	2.1	2.1
Min, Max	0.0, 10.0	0.0, 10.0
(Missing)	687 (0.3%)	1,301 (1.0%)
Meaning & purpose		
Mean	7.4	7.2
Standard Deviation	2.2	2.2
Min, Max	0.0, 10.0	0.0, 10.0
(Missing)	1,420 (0.7%)	687 (0.5%)
Character & virtue		
Mean	7.6	7.4
Standard Deviation	1.9	1.9
Min, Max	0.0, 10.0	0.0, 10.0
(Missing)	1,446 (0.7%)	736 (0.6%)
Close social relationships		
Mean	7.4	7.2
Standard Deviation	2.3	2.3
Min, Max	0.0, 10.0	0.0, 10.0
(Missing)	1,457 (0.7%)	666 (0.5%)
Financial & material security		
Mean	5.9	6.1
Standard Deviation	3.2	3.0
Min, Max	0.0, 10.0	0.0, 10.0
(Missing)	779 (0.4%)	371 (0.3%)
Happiness		
Mean	7.0	6.8
Standard Deviation	2.4	2.3
Min, Max	0.0, 10.0	0.0, 10.0
(Missing)	408 (0.2%)	214 (0.2%)
Life satisfaction		
Mean	6.9	6.6
Standard Deviation	2.6	2.5
Min, Max	0.0, 10.0	0.0, 10.0
(Missing)	708 (0.3%)	391 (0.3%)
Current life evaluation		

Table S2. Weighted summary statistics for outcome variables by Wave.

Outcome	Wave 1<br>N = 207,919	Wave 2<br>N = 128,868
Mean	6.4	6.3
Standard Deviation	2.4	2.3
Min, Max	0.0, 10.0	0.0, 10.0
(Missing)	519 (0.2%)	173 (0.1%)
Future life evaluation		
Mean	7.5	7.2
Standard Deviation	2.3	2.3
Min, Max	0.0, 10.0	0.0, 10.0
(Missing)	4,160 (2.0%)	1,493 (1.2%)
Optimism		
Mean	8.0	7.8
Standard Deviation	2.3	2.4
Min, Max	0.0, 10.0	0.0, 10.0
(Missing)	797 (0.4%)	507 (0.4%)
Freedom to pursue what's important		
Mean	7.7	7.4
Standard Deviation	2.4	2.5
Min, Max	0.0, 10.0	0.0, 10.0
(Missing)	492 (0.2%)	196 (0.2%)
Inner peace, n (%)		
Always	46,046 (22.1%)	24,520 (19.0%)
Often	104,743 (50.4%)	68,424 (53.1%)
Rarely	48,364 (23.3%)	30,493 (23.7%)
Never	7,973 (3.8%)	4,394 (3.4%)
(Missing)	793 (0.4%)	1,037 (0.8%)
Life balance, n (%)		
Always	35,653 (17.1%)	18,737 (14.5%)
Often	109,530 (52.7%)	69,935 (54.3%)
Rarely	53,712 (25.8%)	34,716 (26.9%)
Never	8,118 (3.9%)	4,890 (3.8%)
(Missing)	906 (0.4%)	590 (0.5%)
Sense of mastery, n (%)		
Always	54,526 (26.2%)	28,724 (22.3%)
Often	105,568 (50.8%)	68,835 (53.4%)
Rarely	40,071 (19.3%)	25,967 (20.2%)
Never	6,640 (3.2%)	4,633 (3.6%)
(Missing)	1,114 (0.5%)	708 (0.5%)
Meaningful activities		
Mean	7.3	7.1
Standard Deviation	2.4	2.4
Min, Max	0.0, 10.0	0.0, 10.0
(Missing)	636 (0.3%)	268 (0.2%)
Understanding purpose		
Mean	7.4	7.2
Standard Deviation	2.6	2.6
Min, Max	0.0, 10.0	0.0, 10.0
(Missing)	847 (0.4%)	440 (0.3%)
Self-rated mental health		
Mean	7.6	7.4
Standard Deviation	2.4	2.4
Min, Max	0.0, 10.0	0.0, 10.0

Table S2. Weighted summary statistics for outcome variables by Wave.

**Outcome**	**Wave 1**<br>N = 207,919	**Wave 2**<br>N = 128,868
(Missing)	400 (0.2%)	351 (0.3%)
Traumatic distress, n (%)		
A lot	23,079 (11.1%)	13,672 (10.6%)
Some	46,790 (22.5%)	29,449 (22.9%)
Not very much	62,122 (29.9%)	40,020 (31.1%)
Not at all	75,090 (36.1%)	45,219 (35.1%)
(Missing)	838 (0.4%)	509 (0.4%)
Depression symptoms composite, n (%)	59,658 (28.9%)	34,546 (27.0%)
(Missing)	1,516 (0.7%)	786 (0.6%)
Depression – feel hopeless, n (%)		
Nearly every day	17,965 (8.6%)	10,347 (8.0%)
More than half the days	25,765 (12.4%)	15,632 (12.1%)
Several days	62,656 (30.1%)	39,447 (30.6%)
Not at all	100,782 (48.5%)	63,103 (49.0%)
(Missing)	751 (0.4%)	339 (0.3%)
Depression – loss of interest, n (%)		
Nearly every day	23,215 (11.2%)	12,410 (9.6%)
More than half the days	31,916 (15.4%)	19,010 (14.8%)
Several days	62,593 (30.1%)	40,690 (31.6%)
Not at all	89,307 (43.0%)	56,255 (43.7%)
(Missing)	888 (0.4%)	502 (0.4%)
Anxiety symptoms composite, n (%)	56,239 (27.2%)	33,035 (25.8%)
(Missing)	1,341 (0.6%)	734 (0.6%)
Anxiety – feel on edge, n (%)		
Nearly every day	21,832 (10.5%)	12,187 (9.5%)
More than half the days	27,355 (13.2%)	16,917 (13.1%)
Several days	71,275 (34.3%)	45,069 (35.0%)
Not at all	86,676 (41.7%)	54,239 (42.1%)
(Missing)	781 (0.4%)	456 (0.4%)
Anxiety – cannot stop worrying, n (%)		
Nearly every day	21,818 (10.5%)	11,950 (9.3%)
More than half the days	24,587 (11.8%)	15,124 (11.7%)
Several days	56,222 (27.0%)	35,667 (27.7%)
Not at all	104,575 (50.3%)	65,791 (51.1%)
(Missing)	716 (0.3%)	337 (0.3%)
Suffering, n (%)		
A lot	21,048 (10.1%)	13,089 (10.2%)
Some	67,628 (32.5%)	43,926 (34.1%)
Not very much	69,657 (33.5%)	44,279 (34.4%)
Not at all	48,795 (23.5%)	27,113 (21.0%)
(Missing)	791 (0.4%)	461 (0.4%)
Relationship contentment		
Mean	7.6	7.4
Standard Deviation	2.5	2.5
Min, Max	0.0, 10.0	0.0, 10.0
(Missing)	719 (0.3%)	350 (0.3%)
Relationship satisfaction		
Mean	7.3	7.1
Standard Deviation	2.6	2.6
Min, Max	0.0, 10.0	0.0, 10.0
(Missing)	870 (0.4%)	396 (0.3%)

Table S2. Weighted summary statistics for outcome variables by Wave.

**Outcome**	**Wave 1**<br>N = 207,919	**Wave 2**<br>N = 128,868
Social support		
Mean	7.4	7.3
Standard Deviation	2.8	2.8
Min, Max	0.0, 10.0	0.0, 10.0
(Missing)	559 (0.3%)	1,950 (1.5%)
Intimate/close friend, n (%)		
Yes	171,664 (82.6%)	105,875 (82.2%)
No	35,306 (17.0%)	22,421 (17.4%)
(Missing)	949 (0.5%)	571 (0.4%)
Government approval, n (%)		
Strongly approve	29,231 (14.1%)	15,702 (12.2%)
Somewhat approve	46,939 (22.6%)	27,839 (21.6%)
Neither approve nor disapprove	42,455 (20.4%)	23,951 (18.6%)
Somewhat disapprove	35,836 (17.2%)	23,820 (18.5%)
Strongly disapprove	42,401 (20.4%)	29,150 (22.6%)
(Missing)	11,059 (5.3%)	8,406 (6.5%)
Say in government, n (%)		
Agree	71,922 (34.6%)	43,148 (33.5%)
Disagree	80,086 (38.5%)	51,182 (39.7%)
Unsure	49,976 (24.0%)	29,563 (22.9%)
(Missing)	5,934 (2.9%)	4,976 (3.9%)
Belonging in country		
Mean	7.6	7.4
Standard Deviation	2.6	2.6
Min, Max	0.0, 10.0	0.0, 10.0
(Missing)	6,320 (3.0%)	5,489 (4.3%)
City/place satisfaction, n (%)		
Satisfied	156,301 (75.2%)	94,405 (73.3%)
Dissatisfied	31,531 (15.2%)	20,447 (15.9%)
Unsure	19,171 (9.2%)	13,303 (10.3%)
(Missing)	916 (0.4%)	712 (0.6%)
Trust within country, n (%)		
All people	6,476 (3.1%)	2,841 (2.2%)
Most people	42,276 (20.3%)	26,158 (20.3%)
Some people	85,480 (41.1%)	54,365 (42.2%)
Not very many people	61,444 (29.6%)	39,009 (30.3%)
None	10,463 (5.0%)	5,773 (4.5%)
(Missing)	1,780 (0.9%)	723 (0.6%)
Number of children		
Mean	1.0	1.0
Standard Deviation	1.6	1.9
Min, Max	0.0, 97.0	0.0, 97.0
(Missing)	1,461 (0.7%)	13,909 (11%)
Community participation, n (%)		
More than once a week	16,107 (7.7%)	9,572 (7.4%)
Once a week	21,124 (10.2%)	12,454 (9.7%)
One to three times a month	25,085 (12.1%)	16,148 (12.5%)
A few times a year	47,457 (22.8%)	29,580 (23.0%)
Never	97,476 (46.9%)	60,783 (47.2%)
(Missing)	670 (0.3%)	332 (0.3%)
Religious attendance, n (%)		

Table S2. Weighted summary statistics for outcome variables by Wave.

**Outcome**	**Wave 1**<br>N = 207,919	**Wave 2**<br>N = 128,868
More than once a week	26,507 (12.7%)	14,615 (11.3%)
Once a week	39,833 (19.2%)	21,979 (17.1%)
One to three times a month	20,084 (9.7%)	11,278 (8.8%)
A few times a year	42,064 (20.2%)	26,286 (20.4%)
Never	78,707 (37.9%)	54,127 (42.0%)
(Missing)	723 (0.3%)	584 (0.5%)
Loneliness		
Mean	3.4	3.4
Standard Deviation	3.1	3.0
Min, Max	0.0, 10.0	0.0, 10.0
(Missing)	389 (0.2%)	212 (0.2%)
Perceived discrimination, n (%)		
Always	13,196 (6.3%)	6,979 (5.4%)
Often	32,806 (15.8%)	20,343 (15.8%)
Rarely	80,322 (38.6%)	53,536 (41.5%)
Never	80,672 (38.8%)	47,653 (37.0%)
(Missing)	923 (0.4%)	357 (0.3%)
Orientation to promote good		
Mean	7.9	7.7
Standard Deviation	2.1	2.1
Min, Max	0.0, 10.0	0.0, 10.0
(Missing)	699 (0.3%)	296 (0.2%)
Delayed gratification		
Mean	7.3	7.1
Standard Deviation	2.5	2.4
Min, Max	0.0, 10.0	0.0, 10.0
(Missing)	930 (0.4%)	504 (0.4%)
Hope		
Mean	7.9	7.6
Standard Deviation	2.3	2.3
Min, Max	0.0, 10.0	0.0, 10.0
(Missing)	2,752 (1.3%)	953 (0.7%)
Gratitude		
Mean	7.8	7.6
Standard Deviation	2.4	2.4
Min, Max	0.0, 10.0	0.0, 10.0
(Missing)	3,080 (1.5%)	1,049 (0.8%)
Showing love/care		
Mean	8.1	8.0
Standard Deviation	2.2	2.2
Min, Max	0.0, 10.0	0.0, 10.0
(Missing)	380 (0.2%)	508 (0.4%)
Forgivingness, n (%)		
Always	61,254 (29.5%)	32,316 (25.1%)
Often	93,572 (45.0%)	61,616 (47.8%)
Rarely	43,112 (20.7%)	28,600 (22.2%)
Never	9,266 (4.5%)	5,874 (4.6%)
(Missing)	714 (0.3%)	461 (0.4%)
Charitable giving, n (%)		
Yes	77,822 (37.4%)	54,505 (42.3%)
No	129,538 (62.3%)	73,625 (57.1%)

Table S2. Weighted summary statistics for outcome variables by Wave.

Outcome	Wave 1<br>N = 207,919	Wave 2<br>N = 128,868
(Missing)	559 (0.3%)	738 (0.6%)
Helping strangers, n (%)		
Yes	108,198 (52.0%)	67,049 (52.0%)
No	98,762 (47.5%)	61,278 (47.6%)
(Missing)	959 (0.5%)	541 (0.4%)
Volunteering, n (%)		
Yes	47,876 (23.0%)	33,736 (26.2%)
No	159,416 (76.7%)	94,624 (73.4%)
(Missing)	626 (0.3%)	508 (0.4%)
Self-rated physical health		
Mean	7.1	6.8
Standard Deviation	2.4	2.3
Min, Max	0.0, 10.0	0.0, 10.0
(Missing)	353 (0.2%)	1,147 (0.9%)
Health problems, n (%)		
Yes	43,515 (20.9%)	30,555 (23.7%)
No	162,901 (78.3%)	97,549 (75.7%)
(Missing)	1,503 (0.7%)	764 (0.6%)
Pain in past 4 weeks, n (%)		
A lot	25,137 (12.1%)	16,416 (12.7%)
Some	66,705 (32.1%)	42,749 (33.2%)
Not very much	66,696 (32.1%)	42,467 (33.0%)
None at all	49,011 (23.6%)	27,034 (21.0%)
(Missing)	370 (0.2%)	202 (0.2%)
Number of cigarettes per day		
Mean	2.0	1.8
Standard Deviation	5.7	5.4
Min, Max	0.0, 97.0	0.0, 97.0
(Missing)	3,357 (1.6%)	2,974 (2.3%)
Number of drinks per week		
Mean	2.1	2.0
Standard Deviation	5.6	5.4
Min, Max	0.0, 97.0	0.0, 97.0
(Missing)	2,979 (1.4%)	2,590 (2.0%)
Days exercise per week		
Mean	2.5	2.4
Standard Deviation	2.5	2.4
Min, Max	0.0, 7.0	0.0, 7.0
(Missing)	3,527 (1.7%)	1,262 (1.0%)
Financial security		
Mean	5.8	6.0
Standard Deviation	3.4	3.3
Min, Max	0.0, 10.0	0.0, 10.0
(Missing)	361 (0.2%)	141 (0.1%)
Material security		
Mean	6.0	6.3
Standard Deviation	3.4	3.2
Min, Max	0.0, 10.0	0.0, 10.0
(Missing)	492 (0.2%)	256 (0.2%)
Educational attainment (16+ years), n (%)		
Up to 8	46,842 (22.5%)	22,657 (17.6%)

Table S2. Weighted summary statistics for outcome variables by Wave.

Outcome	Wave 1<br>N = 207,919	Wave 2<br>N = 128,868
9-15	116,015 (55.8%)	72,942 (56.6%)
16+	44,904 (21.6%)	33,258 (25.8%)
(Missing)	158 (0.1%)	11 (<0.0%)
Currently employed, n (%)		
Employed for an employer	81,157 (39.0%)	54,273 (42.1%)
Self-employed	36,901 (17.7%)	21,050 (16.3%)
Retired	30,456 (14.6%)	21,673 (16.8%)
Student	11,505 (5.5%)	5,024 (3.9%)
Homemaker	21,897 (10.5%)	11,073 (8.6%)
Unemployed and looking for a job	16,673 (8.0%)	8,872 (6.9%)
None of these/Other	8,534 (4.1%)	5,933 (4.6%)
(Missing)	796 (0.4%)	969 (0.8%)
Financially comfortable/getting by, n (%)		
Living comfortably on present income	51,560 (24.8%)	35,832 (27.8%)
Getting by on present income	88,865 (42.7%)	56,021 (43.5%)
Finding it difficult on present income	43,512 (20.9%)	24,658 (19.1%)
Finding it very difficult on present income	21,711 (10.4%)	11,478 (8.9%)
(Missing)	2,270 (1.1%)	878 (0.7%)
Own home, n (%)		
Someone in this household owns this home	118,807 (57.1%)	75,403 (58.5%)
Someone in this household rents this home	41,752 (20.1%)	26,227 (20.4%)
Both	6,599 (3.2%)	3,999 (3.1%)
Neither	23,688 (11.4%)	10,685 (8.3%)
Rent	4,303 (2.1%)	3,300 (2.6%)
Own	10,513 (5.1%)	8,090 (6.3%)
Something else	218 (0.1%)	235 (0.2%)
(Missing)	2,039 (1.0%)	929 (0.7%)
Religious/spiritual connection, n (%)		
Always	64,962 (31.2%)	37,929 (29.4%)
Often	53,107 (25.5%)	30,496 (23.7%)
Rarely	52,151 (25.1%)	32,023 (24.8%)
Never	37,091 (17.8%)	28,106 (21.8%)
(Missing)	609 (0.3%)	314 (0.2%)
Belief in life after death, n (%)		
Yes	104,482 (50.3%)	61,723 (47.9%)
No	50,095 (24.1%)	31,871 (24.7%)
Unsure	52,291 (25.1%)	34,589 (26.8%)
(Missing)	1,051 (0.5%)	685 (0.5%)
Transformative religious experience, n (%)		
Yes	75,404 (36.3%)	42,975 (33.3%)
No	131,016 (63.0%)	85,204 (66.1%)
(Missing)	1,499 (0.7%)	689 (0.5%)
Religious reading or listening, n (%)		
More than once a day	22,763 (10.9%)	13,577 (10.5%)
About once a day	32,125 (15.5%)	17,871 (13.9%)
Sometimes	71,967 (34.6%)	40,377 (31.3%)
Never	79,998 (38.5%)	56,390 (43.8%)
(Missing)	1,066 (0.5%)	653 (0.5%)
Prayer or meditation, n (%)		
More than once a day	49,615 (23.9%)	28,312 (22.0%)
About once a day	42,335 (20.4%)	23,883 (18.5%)

Table S2. Weighted summary statistics for outcome variables by Wave.

Outcome	Wave 1<br>N = 207,919	Wave 2<br>N = 128,868
Sometimes	59,492 (28.6%)	35,582 (27.6%)
Never	55,784 (26.8%)	40,645 (31.5%)
(Missing)	694 (0.3%)	447 (0.3%)
Belief in God/gods/spiritual forces, n (%)		
One God	119,927 (57.7%)	69,501 (53.9%)
More than one god	11,428 (5.5%)	6,459 (5.0%)
An impersonal spiritual force	18,795 (9.0%)	12,451 (9.7%)
None of these	34,961 (16.8%)	25,494 (19.8%)
Unsure	22,140 (10.6%)	14,529 (11.3%)
(Missing)	668 (0.3%)	434 (0.3%)
Religious centrality, n (%)		
Agree	101,164 (48.7%)	57,917 (44.9%)
Disagree	33,096 (15.9%)	20,077 (15.6%)
Not relevant	49,906 (24.0%)	36,185 (28.1%)
Unsure	22,675 (10.9%)	14,187 (11.0%)
(Missing)	1,078 (0.5%)	503 (0.4%)
Religious/spiritual comfort, n (%)		
Agree	116,835 (56.2%)	66,622 (51.7%)
Disagree	26,191 (12.6%)	16,060 (12.5%)
Not relevant	45,732 (22.0%)	34,195 (26.5%)
Unsure	18,301 (8.8%)	11,535 (9.0%)
(Missing)	860 (0.4%)	456 (0.4%)
Feel loved by God, n (%)		
Agree	119,671 (57.6%)	68,452 (53.1%)
Disagree	23,787 (11.4%)	14,794 (11.5%)
Not relevant	43,623 (21.0%)	32,018 (24.8%)
Unsure	19,741 (9.5%)	12,879 (10.0%)
(Missing)	1,096 (0.5%)	725 (0.6%)
Feel punished by God, n (%)		
Agree	41,799 (20.1%)	23,627 (18.3%)
Disagree	94,280 (45.3%)	56,465 (43.8%)
Not relevant	46,192 (22.2%)	32,966 (25.6%)
Unsure	24,460 (11.8%)	15,129 (11.7%)
(Missing)	1,187 (0.6%)	681 (0.5%)
Experienced religious criticism, n (%)		
Agree	39,242 (18.9%)	21,256 (16.5%)
Disagree	76,613 (36.8%)	44,721 (34.7%)
Not relevant	65,876 (31.7%)	46,693 (36.2%)
Unsure	24,978 (12.0%)	15,568 (12.1%)
(Missing)	1,210 (0.6%)	630 (0.5%)
Faith-sharing, n (%)		
Agree	86,217 (41.5%)	48,768 (37.8%)
Disagree	50,925 (24.5%)	31,293 (24.3%)
Not relevant	54,296 (26.1%)	38,634 (30.0%)
Unsure	15,580 (7.5%)	9,694 (7.5%)
(Missing)	901 (0.4%)	478 (0.4%)

Note. N (%); this table is based on non-imputed data. Cumulative percentages for variables may not add up to 100% due to rounding. Wave 1 characteristics weighted using the Gallup provided sampling weight, ANNUAL_WEIGHT_R2; Wave 2 characteristics weighted accounting for attrition by using the adjusted Wave 1 weight, ANNUAL_WEIGHT_R2, multiplied by the created attrition weight to account for dropout, to maintain nationally representative estimates for Wave 2 characteristics.

Table S3. Unweighted summary statistics for demographic and childhood variables by retention status.

**Characteristic**	**Attritors–Not Observed in Wave 2** N = 79,051	**Retained–Observed in Wave 2** N = 128,868
Forgivingness, n (%)		
Always	26,300 (33.3%)	33,564 (26.0%)
Often	32,555 (41.2%)	63,071 (48.9%)
Rarely	16,049 (20.3%)	26,866 (20.8%)
Never	3,856 (4.9%)	4,975 (3.9%)
(Missing)	291 (0.4%)	392 (0.3%)
Year of birth, n (%)		
1943 or earlier (current age: 80+ years)	1,123 (1.4%)	3,662 (2.8%)
1943-1953 (current age: 70-79 years)	3,937 (5.0%)	15,976 (12.4%)
1953-1963 (current age: 60-69 years)	7,157 (9.1%)	21,995 (17.1%)
1963-1973 (current age: 50-59 years)	10,089 (12.8%)	21,336 (16.6%)
1973-1983 (current age: 40-49 years)	13,757 (17.4%)	20,932 (16.2%)
1983-1993 (current age: 30-39 years)	18,564 (23.5%)	23,250 (18.0%)
1993-1998 (current age: 25-29 years)	10,357 (13.1%)	10,525 (8.2%)
1998-2005 (current age: 18-24 years)	14,057 (17.8%)	11,182 (8.7%)
(Missing)	10 (0.0%)	10 (<0.0%)
Age of participant		
Mean	40.5	49.0
Standard Deviation	16.3	17.6
Min, Max	18.0, 99.0	18.0, 99.0
(Missing)	10 (<0.1%)	10 (<0.1%)
Gender, n (%)		
Male	34,938 (44.2%)	62,823 (48.7%)
Female	43,714 (55.3%)	65,617 (50.9%)
Other	168 (0.2%)	238 (0.2%)
(Missing)	231 (0.3%)	190 (0.1%)
Respondent marital status, n (%)		
Single/Never been married	22,924 (29.0%)	27,961 (21.7%)
Married	38,596 (48.8%)	74,144 (57.5%)
Separated	2,362 (3.0%)	2,867 (2.2%)
Divorced	3,758 (4.8%)	8,525 (6.6%)
Widowed	2,996 (3.8%)	6,327 (4.9%)
Domestic partner	7,371 (9.3%)	8,233 (6.4%)
(Missing)	1,044 (1.3%)	811 (0.6%)
Education (years), n (%)		
Up to 8	15,482 (19.6%)	18,321 (14.2%)
9-15	48,117 (60.9%)	70,512 (54.7%)
16+	15,391 (19.5%)	39,909 (31.0%)
(Missing)	61 (0.1%)	126 (0.1%)
Employment status, n (%)		
Employed for an employer	27,843 (35.2%)	48,161 (37.4%)
Self-employed	15,300 (19.4%)	20,685 (16.1%)
Retired	7,109 (9.0%)	27,606 (21.4%)
Student	5,498 (7.0%)	5,412 (4.2%)
Homemaker	10,959 (13.9%)	12,169 (9.4%)
Unemployed and looking for a job	8,339 (10.5%)	8,913 (6.9%)
None of these/Other	3,436 (4.3%)	5,665 (4.4%)
(Missing)	567 (0.7%)	257 (0.2%)
Current religious service attendance, n (%)		

Table S3. Unweighted summary statistics for demographic and childhood variables by retention status.

**Characteristic**	**Attritors–Not Observed in Wave 2** N = 79,051	**Retained–Observed in Wave 2** N = 128,868
More than once a week	11,796 (14.9%)	14,306 (11.1%)
Once a week	16,257 (20.6%)	23,844 (18.5%)
One to three times a month	8,970 (11.3%)	11,610 (9.0%)
A few times a year	16,380 (20.7%)	25,468 (19.8%)
Never	25,326 (32.0%)	53,239 (41.3%)
(Missing)	322 (0.4%)	401 (0.3%)
Immigration status, n (%)		
Born in this country	74,341 (94.0%)	122,628 (95.2%)
Born in another country	3,598 (4.6%)	5,290 (4.1%)
(Missing)	1,112 (1.4%)	950 (0.7%)
Parental marital status around age 12, n (%)		
Parents were married	56,922 (72.0%)	102,962 (79.9%)
Parents were divorced	6,492 (8.2%)	10,313 (8.0%)
Parents were never married	7,074 (8.9%)	6,780 (5.3%)
One or both of them had died	3,101 (3.9%)	4,625 (3.6%)
Unsure	1,326 (1.7%)	1,011 (0.8%)
(Missing)	4,136 (5.2%)	3,177 (2.5%)
Religious service attendance around age 12, n (%)		
At least once a week	33,367 (42.2%)	52,240 (40.5%)
One to three times a month	14,213 (18.0%)	19,918 (15.5%)
Less than once a month	13,970 (17.7%)	22,672 (17.6%)
Never	16,661 (21.1%)	32,943 (25.6%)
(Missing)	840 (1.1%)	1,095 (0.8%)
Relationship with mother when growing up, n (%)		
Very good	50,997 (64.5%)	78,870 (61.2%)
Somewhat good	19,685 (24.9%)	35,753 (27.7%)
Somewhat bad	3,772 (4.8%)	7,464 (5.8%)
Very bad	1,479 (1.9%)	2,555 (2.0%)
(Does not apply)	2,684 (3.4%)	3,689 (2.9%)
(Missing)	434 (0.5%)	537 (0.4%)
Relationship with father when growing up, n (%)		
Very good	43,258 (54.7%)	65,701 (51.0%)
Somewhat good	20,508 (25.9%)	38,627 (30.0%)
Somewhat bad	5,536 (7.0%)	10,665 (8.3%)
Very bad	3,122 (3.9%)	4,852 (3.8%)
(Does not apply)	5,987 (7.6%)	8,297 (6.4%)
(Missing)	640 (0.8%)	726 (0.6%)
Felt like an outsider in family when growing up, n (%)		
Yes	11,595 (14.7%)	17,312 (13.4%)
No	65,940 (83.4%)	109,621 (85.1%)
(Missing)	1,516 (1.9%)	1,935 (1.5%)
Experienced abuse when growing up, n (%)		
Yes	10,882 (13.8%)	17,725 (13.8%)
No	65,176 (82.4%)	107,610 (83.5%)
(Missing)	2,993 (3.8%)	3,533 (2.7%)
Self-rated health when growing up, n (%)		
Excellent	25,415 (32.2%)	45,818 (35.6%)

Table S3. Unweighted summary statistics for demographic and childhood variables by retention status.

**Characteristic**	**Attritors–Not Observed in Wave 2** N = 79,051	**Retained–Observed in Wave 2** N = 128,868
Very good	24,074 (30.5%)	40,639 (31.5%)
Good	19,079 (24.1%)	27,881 (21.6%)
Fair	8,247 (10.4%)	11,626 (9.0%)
Poor	2,035 (2.6%)	2,593 (2.0%)
(Missing)	201 (0.3%)	311 (0.2%)
Subjective financial status of family growing up, n (%)		
Lived comfortably	28,921 (36.6%)	46,076 (35.8%)
Got by	31,140 (39.4%)	54,847 (42.6%)
Found it difficult	13,944 (17.6%)	20,954 (16.3%)
Found it very difficult	4,739 (6.0%)	6,679 (5.2%)
(Missing)	307 (0.4%)	312 (0.2%)
Religious affiliation growing up, n (%)		
Christianity	44,656 (56.5%)	77,080 (59.8%)
Taoism	77 (0.1%)	144 (0.1%)
Confucianism	37 (0.0%)	41 (0.0%)
Primal, Animist, or Folk religion	197 (0.2%)	186 (0.1%)
Spiritism	242 (0.3%)	118 (0.1%)
Umbanda, Candomblé, and other African-derived religions	251 (0.3%)	78 (0.1%)
Chinese folk/traditional religion	104 (0.1%)	157 (0.1%)
Islam	11,061 (14.0%)	11,215 (8.7%)
Hinduism	5,617 (7.1%)	5,679 (4.4%)
Buddhism	2,396 (3.0%)	5,363 (4.2%)
Judaism	1,162 (1.5%)	3,390 (2.6%)
Sikhism	117 (0.1%)	89 (0.1%)
Baha'i	13 (0.0%)	17 (0.0%)
Jainism	12 (0.0%)	19 (0.0%)
Shinto	149 (0.2%)	259 (0.2%)
Some other religion	228 (0.3%)	397 (0.3%)
No religion/Atheist/Agnostic	12,249 (15.5%)	24,167 (18.8%)
(Missing)	483 (0.6%)	469 (0.4%)

Note. N (%); this table is based on non-imputed data. Cumulative percentages for variables may not add up to 100% due to rounding.

Table S4. Unweighted summary statistics for Wave 1 outcome variables by retention status.

**Outcome**	**Attritors–Not Observed in Wave 2** N = 79,051	**Retained–Observed in Wave 2** N = 128,868
Secure flourishing index		
Mean	7.1	7.2
Standard Deviation	1.7	1.7
Min, Max	0.0, 10.0	0.0, 10.0
(Missing)	2,386 (3.0%)	3,182 (2.5%)
Flourishing index		
Mean	7.4	7.3
Standard Deviation	1.7	1.7
Min, Max	0.0, 10.0	0.0, 10.0
(Missing)	2,115 (2.7%)	2,861 (2.2%)
Happiness & life satisfaction		
Mean	6.9	7.0
Standard Deviation	2.3	2.2
Min, Max	0.0, 10.0	0.0, 10.0
(Missing)	478 (0.6%)	601 (0.5%)
Physical & mental health		
Mean	7.4	7.4
Standard Deviation	2.2	2.0
Min, Max	0.0, 10.0	0.0, 10.0
(Missing)	279 (0.4%)	393 (0.3%)
Meaning & purpose		
Mean	7.5	7.4
Standard Deviation	2.2	2.2
Min, Max	0.0, 10.0	0.0, 10.0
(Missing)	576 (0.7%)	773 (0.6%)
Character & virtue		
Mean	7.7	7.5
Standard Deviation	2.0	1.9
Min, Max	0.0, 10.0	0.0, 10.0
(Missing)	590 (0.7%)	756 (0.6%)
Close social relationships		
Mean	7.5	7.4
Standard Deviation	2.4	2.3
Min, Max	0.0, 10.0	0.0, 10.0
(Missing)	649 (0.8%)	850 (0.7%)
Financial & material security		
Mean	5.5	6.3
Standard Deviation	3.3	3.1
Min, Max	0.0, 10.0	0.0, 10.0
(Missing)	394 (0.5%)	419 (0.3%)
Happiness		
Mean	7.0	7.0
Standard Deviation	2.5	2.3
Min, Max	0.0, 10.0	0.0, 10.0
(Missing)	206 (0.3%)	233 (0.2%)
Life satisfaction		
Mean	6.9	6.9
Standard Deviation	2.7	2.5
Min, Max	0.0, 10.0	0.0, 10.0

Table S4. Unweighted summary statistics for Wave 1 outcome variables by retention status.

**Outcome**	**Attritors–Not Observed in Wave 2** N = 79,051	**Retained–Observed in Wave 2** N = 128,868
(Missing)	313 (0.4%)	400 (0.3%)
Current life evaluation		
Mean	6.4	6.6
Standard Deviation	2.5	2.4
Min, Max	0.0, 10.0	0.0, 10.0
(Missing)	240 (0.3%)	247 (0.2%)
Future life evaluation		
Mean	7.7	7.4
Standard Deviation	2.3	2.2
Min, Max	0.0, 10.0	0.0, 10.0
(Missing)	1,830 (2.3%)	1,936 (1.5%)
Optimism		
Mean	8.2	7.9
Standard Deviation	2.3	2.3
Min, Max	0.0, 10.0	0.0, 10.0
(Missing)	322 (0.4%)	521 (0.4%)
Freedom to pursue what's important		
Mean	7.8	7.7
Standard Deviation	2.5	2.4
Min, Max	0.0, 10.0	0.0, 10.0
(Missing)	205 (0.3%)	257 (0.2%)
Inner peace, n (%)		
Always	18,612 (23.5%)	27,235 (21.1%)
Often	36,175 (45.8%)	70,274 (54.5%)
Rarely	20,325 (25.7%)	27,124 (21.0%)
Never	3,597 (4.6%)	3,801 (2.9%)
(Missing)	342 (0.4%)	434 (0.3%)
Life balance, n (%)		
Always	14,813 (18.7%)	20,283 (15.7%)
Often	38,337 (48.5%)	73,414 (57.0%)
Rarely	22,033 (27.9%)	30,722 (23.8%)
Never	3,491 (4.4%)	3,966 (3.1%)
(Missing)	377 (0.5%)	483 (0.4%)
Sense of mastery, n (%)		
Always	23,141 (29.3%)	30,915 (24.0%)
Often	36,783 (46.5%)	70,304 (54.6%)
Rarely	15,771 (20.0%)	23,268 (18.1%)
Never	2,868 (3.6%)	3,759 (2.9%)
(Missing)	488 (0.6%)	622 (0.5%)
Meaningful activities		
Mean	7.3	7.3
Standard Deviation	2.5	2.4
Min, Max	0.0, 10.0	0.0, 10.0
(Missing)	290 (0.4%)	309 (0.2%)
Understanding purpose		
Mean	7.6	7.4
Standard Deviation	2.6	2.5
Min, Max	0.0, 10.0	0.0, 10.0
(Missing)	320 (0.4%)	490 (0.4%)

Table S4. Unweighted summary statistics for Wave 1 outcome variables by retention status.

**Outcome**	**Attritors–Not Observed in Wave 2**<br>N = 79,051	**Retained–Observed in Wave 2**<br>N = 128,868
Self-rated mental health		
Mean	7.6	7.7
Standard Deviation	2.5	2.3
Min, Max	0.0, 10.0	0.0, 10.0
(Missing)	169 (0.2%)	224 (0.2%)
Traumatic distress, n (%)		
A lot	10,802 (13.7%)	11,965 (9.3%)
Some	18,781 (23.8%)	27,479 (21.3%)
Not very much	22,493 (28.5%)	39,625 (30.7%)
Not at all	26,596 (33.6%)	49,368 (38.3%)
(Missing)	379 (0.5%)	431 (0.3%)
Depression symptoms composite, n (%)	26,650 (34.0%)	31,431 (24.5%)
(Missing)	633 (0.8%)	837 (0.6%)
Depression – feel hopeless, n (%)		
Nearly every day	8,515 (10.8%)	9,249 (7.2%)
More than half the days	11,075 (14.0%)	13,847 (10.7%)
Several days	24,661 (31.2%)	38,451 (29.8%)
Not at all	34,496 (43.6%)	66,902 (51.9%)
(Missing)	304 (0.4%)	419 (0.3%)
Depression – loss of interest, n (%)		
Nearly every day	10,721 (13.6%)	11,726 (9.1%)
More than half the days	13,765 (17.4%)	17,189 (13.3%)
Several days	24,565 (31.1%)	38,324 (29.7%)
Not at all	29,602 (37.4%)	61,149 (47.5%)
(Missing)	398 (0.5%)	480 (0.4%)
Anxiety symptoms composite, n (%)	25,471 (32.5%)	29,159 (22.8%)
(Missing)	574 (0.7%)	732 (0.6%)
Anxiety – feel on edge, n (%)		
Nearly every day	10,571 (13.4%)	10,762 (8.4%)
More than half the days	11,454 (14.5%)	14,906 (11.6%)
Several days	27,326 (34.6%)	44,647 (34.6%)
Not at all	29,365 (37.1%)	58,118 (45.1%)
(Missing)	335 (0.4%)	435 (0.3%)
Anxiety – cannot stop worrying, n (%)		
Nearly every day	10,443 (13.2%)	10,539 (8.2%)
More than half the days	10,750 (13.6%)	13,022 (10.1%)
Several days	22,054 (27.9%)	34,415 (26.7%)
Not at all	35,482 (44.9%)	70,525 (54.7%)
(Missing)	322 (0.4%)	367 (0.3%)
Suffering, n (%)		
A lot	9,107 (11.5%)	10,994 (8.5%)
Some	26,409 (33.4%)	41,630 (32.3%)
Not very much	24,875 (31.5%)	45,704 (35.5%)
Not at all	18,310 (23.2%)	30,113 (23.4%)
(Missing)	350 (0.4%)	427 (0.3%)
Relationship contentment		
Mean	7.6	7.6
Standard Deviation	2.5	2.4
Min, Max	0.0, 10.0	0.0, 10.0

Table S4. Unweighted summary statistics for Wave 1 outcome variables by retention status.

**Outcome**	**Attritors–Not Observed in Wave 2** N = 79,051	**Retained–Observed in Wave 2** N = 128,868
(Missing)	334 (0.4%)	404 (0.3%)
Relationship satisfaction		
Mean	7.3	7.2
Standard Deviation	2.6	2.5
Min, Max	0.0, 10.0	0.0, 10.0
(Missing)	380 (0.5%)	519 (0.4%)
Social support		
Mean	7.3	7.5
Standard Deviation	2.9	2.7
Min, Max	0.0, 10.0	0.0, 10.0
(Missing)	254 (0.3%)	342 (0.3%)
Intimate/close friend, n (%)		
Yes	64,698 (81.8%)	107,220 (83.2%)
No	13,880 (17.6%)	21,220 (16.5%)
(Missing)	473 (0.6%)	428 (0.3%)
Government approval, n (%)		
Strongly approve	13,216 (16.7%)	15,284 (11.9%)
Somewhat approve	18,745 (23.7%)	29,590 (23.0%)
Neither approve nor disapprove	18,116 (22.9%)	22,741 (17.6%)
Somewhat disapprove	12,208 (15.4%)	23,658 (18.4%)
Strongly disapprove	14,017 (17.7%)	29,423 (22.8%)
(Missing)	2,749 (3.5%)	8,172 (6.3%)
Say in government, n (%)		
Agree	30,136 (38.1%)	43,054 (33.4%)
Disagree	28,121 (35.6%)	51,158 (39.7%)
Unsure	19,864 (25.1%)	29,693 (23.0%)
(Missing)	930 (1.2%)	4,963 (3.9%)
Belonging in country		
Mean	7.7	7.6
Standard Deviation	2.6	2.5
Min, Max	0.0, 10.0	0.0, 10.0
(Missing)	1,119 (1.4%)	5,140 (4.0%)
City/place satisfaction, n (%)		
Satisfied	58,598 (74.1%)	99,001 (76.8%)
Dissatisfied	12,487 (15.8%)	18,110 (14.1%)
Unsure	7,559 (9.6%)	11,251 (8.7%)
(Missing)	407 (0.5%)	506 (0.4%)
Trust within country, n (%)		
All people	3,221 (4.1%)	2,871 (2.2%)
Most people	14,977 (18.9%)	28,128 (21.8%)
Some people	31,856 (40.3%)	55,317 (42.9%)
Not very many people	23,233 (29.4%)	36,884 (28.6%)
None	5,048 (6.4%)	4,807 (3.7%)
(Missing)	716 (0.9%)	861 (0.7%)
Number of children		
Mean	1.1	0.9
Standard Deviation	1.7	1.5
Min, Max	0.0, 97.0	0.0, 97.0
(Missing)	939 (1.2%)	592 (0.5%)

Table S4. Unweighted summary statistics for Wave 1 outcome variables by retention status.

**Outcome**	**Attritors–Not Observed in Wave 2** N = 79,051	**Retained–Observed in Wave 2** N = 128,868
Community participation, n (%)		
More than once a week	6,837 (8.6%)	10,161 (7.9%)
Once a week	8,776 (11.1%)	12,486 (9.7%)
One to three times a month	9,681 (12.2%)	16,982 (13.2%)
A few times a year	17,544 (22.2%)	31,389 (24.4%)
Never	35,902 (45.4%)	57,531 (44.6%)
(Missing)	311 (0.4%)	319 (0.2%)
Religious attendance, n (%)		
More than once a week	11,796 (14.9%)	14,306 (11.1%)
Once a week	16,257 (20.6%)	23,844 (18.5%)
One to three times a month	8,970 (11.3%)	11,610 (9.0%)
A few times a year	16,380 (20.7%)	25,468 (19.8%)
Never	25,326 (32.0%)	53,239 (41.3%)
(Missing)	322 (0.4%)	401 (0.3%)
Loneliness		
Mean	3.5	3.2
Standard Deviation	3.1	2.9
Min, Max	0.0, 10.0	0.0, 10.0
(Missing)	202 (0.3%)	192 (0.1%)
Perceived discrimination, n (%)		
Always	6,330 (8.0%)	6,323 (4.9%)
Often	13,223 (16.7%)	19,022 (14.8%)
Rarely	29,003 (36.7%)	52,924 (41.1%)
Never	30,085 (38.1%)	50,167 (38.9%)
(Missing)	410 (0.5%)	432 (0.3%)
Orientation to promote good		
Mean	8.0	7.9
Standard Deviation	2.1	2.0
Min, Max	0.0, 10.0	0.0, 10.0
(Missing)	296 (0.4%)	381 (0.3%)
Delayed gratification		
Mean	7.4	7.2
Standard Deviation	2.5	2.4
Min, Max	0.0, 10.0	0.0, 10.0
(Missing)	371 (0.5%)	460 (0.4%)
Hope		
Mean	8.1	7.8
Standard Deviation	2.3	2.3
Min, Max	0.0, 10.0	0.0, 10.0
(Missing)	756 (1.0%)	2,384 (1.8%)
Gratitude		
Mean	7.8	7.8
Standard Deviation	2.4	2.4
Min, Max	0.0, 10.0	0.0, 10.0
(Missing)	891 (1.1%)	2,512 (1.9%)
Showing love/care		
Mean	8.1	8.1
Standard Deviation	2.3	2.2
Min, Max	0.0, 10.0	0.0, 10.0

Table S4. Unweighted summary statistics for Wave 1 outcome variables by retention status.

**Outcome**	**Attritors–Not Observed in Wave 2** N = 79,051	**Retained–Observed in Wave 2** N = 128,868
(Missing)	193 (0.2%)	191 (0.1%)
Forgivingness, n (%)		
Always	26,300 (33.3%)	33,564 (26.0%)
Often	32,555 (41.2%)	63,071 (48.9%)
Rarely	16,049 (20.3%)	26,866 (20.8%)
Never	3,856 (4.9%)	4,975 (3.9%)
(Missing)	291 (0.4%)	392 (0.3%)
Charitable giving, n (%)		
Yes	28,389 (35.9%)	52,756 (40.9%)
No	50,408 (63.8%)	75,765 (58.8%)
(Missing)	254 (0.3%)	347 (0.3%)
Helping strangers, n (%)		
Yes	45,226 (57.2%)	64,313 (49.9%)
No	33,364 (42.2%)	64,056 (49.7%)
(Missing)	461 (0.6%)	499 (0.4%)
Volunteering, n (%)		
Yes	19,434 (24.6%)	31,503 (24.4%)
No	59,355 (75.1%)	96,991 (75.3%)
(Missing)	262 (0.3%)	374 (0.3%)
Self-rated physical health		
Mean	7.2	7.1
Standard Deviation	2.4	2.3
Min, Max	0.0, 10.0	0.0, 10.0
(Missing)	140 (0.2%)	210 (0.2%)
Health problems, n (%)		
Yes	15,521 (19.6%)	27,488 (21.3%)
No	62,445 (79.0%)	100,885 (78.3%)
(Missing)	1,085 (1.4%)	495 (0.4%)
Pain in past 4 weeks, n (%)		
A lot	10,074 (12.7%)	14,140 (11.0%)
Some	25,050 (31.7%)	42,243 (32.8%)
Not very much	24,606 (31.1%)	43,278 (33.6%)
None at all	19,136 (24.2%)	29,035 (22.5%)
(Missing)	185 (0.2%)	172 (0.1%)
Number of cigarettes per day		
Mean	2.1	1.7
Standard Deviation	5.9	5.3
Min, Max	0.0, 97.0	0.0, 97.0
(Missing)	1,643 (2.1%)	1,802 (1.4%)
Number of drinks per week		
Mean	1.9	2.3
Standard Deviation	5.8	5.5
Min, Max	0.0, 97.0	0.0, 97.0
(Missing)	1,572 (2.0%)	1,451 (1.1%)
Days exercise per week		
Mean	2.5	2.5
Standard Deviation	2.5	2.5
Min, Max	0.0, 7.0	0.0, 7.0
(Missing)	1,660 (2.1%)	1,516 (1.2%)

Table S4. Unweighted summary statistics for Wave 1 outcome variables by retention status.

**Outcome**	**Attritors–Not Observed in Wave 2** N = 79,051	**Retained–Observed in Wave 2** N = 128,868
Financial security		
Mean	5.3	6.1
Standard Deviation	3.5	3.3
Min, Max	0.0, 10.0	0.0, 10.0
(Missing)	176 (0.2%)	200 (0.2%)
Material security		
Mean	5.6	6.4
Standard Deviation	3.6	3.3
Min, Max	0.0, 10.0	0.0, 10.0
(Missing)	258 (0.3%)	245 (0.2%)
Educational attainment (16+ years), n (%)		
Up to 8	15,482 (19.6%)	18,321 (14.2%)
9-15	48,117 (60.9%)	70,512 (54.7%)
16+	15,391 (19.5%)	39,909 (31.0%)
(Missing)	61 (0.1%)	126 (0.1%)
Currently employed, n (%)		
Employed for an employer	27,843 (35.2%)	48,161 (37.4%)
Self-employed	15,300 (19.4%)	20,685 (16.1%)
Retired	7,109 (9.0%)	27,606 (21.4%)
Student	5,498 (7.0%)	5,412 (4.2%)
Homemaker	10,959 (13.9%)	12,169 (9.4%)
Unemployed and looking for a job	8,339 (10.5%)	8,913 (6.9%)
None of these/Other	3,436 (4.3%)	5,665 (4.4%)
(Missing)	567 (0.7%)	257 (0.2%)
Financially comfortable/getting by, n (%)		
Living comfortably on present income	17,103 (21.6%)	37,978 (29.5%)
Getting by on present income	31,741 (40.2%)	55,032 (42.7%)
Finding it difficult on present income	19,169 (24.2%)	23,903 (18.5%)
Finding it very difficult on present income	9,435 (11.9%)	11,207 (8.7%)
(Missing)	1,603 (2.0%)	748 (0.6%)
Own home, n (%)		
Someone in this household owns this home	43,021 (54.4%)	79,173 (61.4%)
Someone in this household rents this home	17,543 (22.2%)	21,932 (17.0%)
Both	2,888 (3.7%)	3,386 (2.6%)
Neither	10,598 (13.4%)	12,205 (9.5%)
Rent	1,322 (1.7%)	3,543 (2.7%)
Own	2,000 (2.5%)	7,833 (6.1%)
Something else	128 (0.2%)	216 (0.2%)
(Missing)	1,551 (2.0%)	580 (0.5%)
Religious/spiritual connection, n (%)		
Always	27,522 (34.8%)	37,737 (29.3%)
Often	20,453 (25.9%)	32,485 (25.2%)
Rarely	18,745 (23.7%)	33,097 (25.7%)
Never	12,080 (15.3%)	25,277 (19.6%)
(Missing)	251 (0.3%)	272 (0.2%)
Belief in life after death, n (%)		
Yes	41,680 (52.7%)	62,130 (48.2%)
No	18,513 (23.4%)	32,920 (25.5%)
Unsure	18,381 (23.3%)	33,241 (25.8%)

Table S4. Unweighted summary statistics for Wave 1 outcome variables by retention status.

**Outcome**	**Attritors–Not Observed in Wave 2** N = 79,051	**Retained–Observed in Wave 2** N = 128,868
(Missing)	477 (0.6%)	577 (0.4%)
Transformative religious experience, n (%)		
Yes	32,505 (41.1%)	42,612 (33.1%)
No	45,841 (58.0%)	85,496 (66.3%)
(Missing)	705 (0.9%)	760 (0.6%)
Religious reading or listening, n (%)		
More than once a day	9,937 (12.6%)	12,497 (9.7%)
About once a day	13,686 (17.3%)	18,492 (14.3%)
Sometimes	29,404 (37.2%)	42,711 (33.1%)
Never	25,543 (32.3%)	54,543 (42.3%)
(Missing)	481 (0.6%)	625 (0.5%)
Prayer or meditation, n (%)		
More than once a day	20,831 (26.4%)	28,626 (22.2%)
About once a day	17,468 (22.1%)	25,213 (19.6%)
Sometimes	22,474 (28.4%)	36,302 (28.2%)
Never	17,883 (22.6%)	38,385 (29.8%)
(Missing)	395 (0.5%)	342 (0.3%)
Belief in God/gods/spiritual forces, n (%)		
One God	49,389 (62.5%)	69,540 (54.0%)
More than one god	5,132 (6.5%)	5,966 (4.6%)
An impersonal spiritual force	6,484 (8.2%)	12,934 (10.0%)
None of these	10,573 (13.4%)	25,579 (19.8%)
Unsure	7,106 (9.0%)	14,540 (11.3%)
(Missing)	367 (0.5%)	309 (0.2%)
Religious centrality, n (%)		
Agree	41,742 (52.8%)	59,171 (45.9%)
Disagree	11,609 (14.7%)	21,945 (17.0%)
Not relevant	16,351 (20.7%)	33,915 (26.3%)
Unsure	8,830 (11.2%)	13,324 (10.3%)
(Missing)	519 (0.7%)	513 (0.4%)
Religious/spiritual comfort, n (%)		
Agree	47,739 (60.4%)	68,221 (52.9%)
Disagree	9,327 (11.8%)	17,510 (13.6%)
Not relevant	14,604 (18.5%)	31,609 (24.5%)
Unsure	6,975 (8.8%)	11,079 (8.6%)
(Missing)	406 (0.5%)	449 (0.3%)
Feel loved by God, n (%)		
Agree	49,839 (63.0%)	68,520 (53.2%)
Disagree	8,303 (10.5%)	16,264 (12.6%)
Not relevant	13,621 (17.2%)	30,544 (23.7%)
Unsure	6,819 (8.6%)	12,921 (10.0%)
(Missing)	469 (0.6%)	619 (0.5%)
Feel punished by God, n (%)		
Agree	18,255 (23.1%)	21,804 (16.9%)
Disagree	34,804 (44.0%)	62,003 (48.1%)
Not relevant	15,665 (19.8%)	30,744 (23.9%)
Unsure	9,772 (12.4%)	13,691 (10.6%)
(Missing)	555 (0.7%)	626 (0.5%)
Experienced religious criticism, n (%)		

Table S4. Unweighted summary statistics for Wave 1 outcome variables by retention status.

**Outcome**	**Attritors–Not Observed in Wave 2** N = 79,051	**Retained–Observed in Wave 2** N = 128,868
Agree	17,704 (22.4%)	19,997 (15.5%)
Disagree	28,870 (36.5%)	49,432 (38.4%)
Not relevant	21,371 (27.0%)	45,354 (35.2%)
Unsure	10,585 (13.4%)	13,503 (10.5%)
(Missing)	521 (0.7%)	582 (0.5%)
Faith-sharing, n (%)		
Agree	36,150 (45.7%)	49,220 (38.2%)
Disagree	17,972 (22.7%)	33,992 (26.4%)
Not relevant	17,997 (22.8%)	36,545 (28.4%)
Unsure	6,526 (8.3%)	8,691 (6.7%)
(Missing)	406 (0.5%)	420 (0.3%)

Note. N (%); this table is based on non-imputed data. Cumulative percentages for variables may not add up to 100% due to rounding.

Table S5. Meta-analyzed associations of forgivingness at Wave 1 with well-being and other outcomes at Wave 2 for Model 1 by approach to address missingness (multiple imputation vs. complete case with attrition weights).

	Multiple Imputation					Complete Case with Attrition Weights				
Outcome	RR	ES	95% CI	τ	Global p-value	RR	ES	95% CI	τ	Global p-value
Human Flourishing										
Secure flourishing index		0.07	(0.06,0.09)	0.03	1.7e-15***		0.07	(0.05,0.09)	0.03	2.55e-15***
Flourishing index		0.08	(0.06,0.09)	0.03	1.7e-15***		0.07	(0.06,0.09)	0.03	2.55e-15***
Happiness & life satisfaction		0.04	(0.03,0.06)	0.03	2.55e-15***		0.04	(0.03,0.06)	0.04	3.4e-15***
Physical & mental health		0.04	(0.03,0.06)	0.02	2.55e-15***		0.04	(0.03,0.06)	0.02	5.05e-15***
Meaning & purpose		0.06	(0.05,0.07)	0.02	2.55e-15***		0.06	(0.04,0.08)	0.03	2.55e-15***
Character & virtue		0.09	(0.07,0.10)	0.04	1.28e-15***		0.09	(0.07,0.11)	0.05	1.57e-15***
Close social relationships		0.06	(0.05,0.08)	0.02	4.08e-15***		0.06	(0.05,0.08)	0.03	5.11e-15***
Financial & material security		0.02	(0.01,0.04)	0.03	5.11e-15***		0.03	(0.01,0.04)	0.02	2.81e-13***
Psychological Well-Being										
Happiness (a)		0.04	(0.03,0.06)	0.03	2.55e-15***		0.04	(0.02,0.06)	0.04	2.55e-15***
Life satisfaction (a)		0.04	(0.03,0.05)	0.03	2.55e-15***		0.04	(0.03,0.06)	0.03	5.1e-15***
Current life evaluation		0.03	(0.01,0.04)	0.03	5.11e-15***		0.03	(0.01,0.04)	0.03	5.11e-15***
Future life evaluation		0.04	(0.03,0.05)	0.03	5.11e-15***		0.04	(0.03,0.06)	0.03	5.11e-15***
Optimism		0.06	(0.05,0.07)	0.03	2.19e-15***		0.06	(0.05,0.08)	0.03	2.54e-15***
Freedom to pursue what's important		0.04	(0.02,0.05)	0.02	5.11e-15***		0.03	(0.02,0.05)	0.03	5.11e-15***
Inner peace	1.02		(1.02,1.03)	0.02	5.08e-15***	1.03		(1.02,1.03)	0.02	5.1e-15***
Life balance	1.02		(1.02,1.03)	0.01	5.11e-15***	1.02		(1.02,1.03)	0.01	5.11e-15***
Sense of mastery	1.02		(1.01,1.02)	0.01	5.11e-15***	1.02		(1.01,1.03)	0.01	5.11e-15***
Meaningful activities (c)		0.05	(0.03,0.06)	0.03	2.55e-15***		0.05	(0.03,0.07)	0.03	5.11e-15***
Understanding purpose (c)		0.06	(0.04,0.07)	0.02	2.55e-15***		0.06	(0.04,0.07)	0.02	2.55e-15***
Self-rated mental health (b)		0.05	(0.03,0.06)	0.02	4.6e-15***		0.05	(0.04,0.06)	0.02	3.83e-15***
Psychological Distress										
Traumatic distress	1.00		(0.99,1.00)	<0.01†	0.039*	1.00		(0.99,1.00)	0.01	2.35e-03**
Depression symptoms composite	0.99		(0.99,1.00)	<0.01†	1.44e-08***	1.00		(0.99,1.00)	0.01	1.39e-07***
Depression – feel hopeless	0.99		(0.99,1.00)	0.01	1.2e-07***	0.99		(0.99,1.00)	0.01	4.29e-07***
Depression – loss of interest	1.00		(0.99,1.00)	<0.01†	2.99e-06***	1.00		(0.99,1.00)	0.01	1.69e-06***
Anxiety symptoms composite	0.99		(0.99,1.00)	<0.01†	2.58e-10***	0.99		(0.98,1.00)	0.01	2e-07***
Anxiety – feel on edge	0.99		(0.99,1.00)	<0.01†	2.49e-07***	0.99		(0.99,1.00)	<0.01†	1.38e-06***
Anxiety – cannot stop worrying	0.99		(0.99,1.00)	0.01	1.76e-07***	0.99		(0.99,1.00)	0.01	8.78e-08***
Suffering	1.00		(0.99,1.00)	0.01	8.83e-05***	0.99		(0.99,1.00)	0.01	3.25e-05***
Social Well-Being										
Relationship contentment (e)		0.06	(0.05,0.07)	0.02	5.04e-15***		0.06	(0.05,0.08)	0.03	5.11e-15***
Relationship satisfaction (e)		0.05	(0.04,0.07)	0.02	5.11e-15***		0.06	(0.04,0.07)	0.03	5.11e-15***
Social support		0.05	(0.03,0.06)	0.03	2.13e-15***		0.05	(0.03,0.07)	0.04	2.54e-15***
Intimate/close friend	1.02		(1.01,1.02)	<0.01†	5.11e-15***	1.01		(1.01,1.02)	0.01	5.11e-15***
Government approval	1.01		(1.01,1.02)	0.01	5.46e-06***	1.02		(1.01,1.02)	0.02	1.4e-06***
Say in government	1.01		(1.01,1.02)	0.01	1.23e-12***	1.01		(1.01,1.02)	0.01	2.66e-11***
Belonging in country		0.05	(0.04,0.07)	0.02	4.88e-15***		0.06	(0.04,0.07)	0.02	4.88e-15***
City/place satisfaction	1.01		(1.01,1.02)	0.01	2.04e-14***	1.02		(1.01,1.02)	0.01	6.64e-14***
Trust within country	1.02		(1.01,1.03)	0.01	5.03e-15***	1.02		(1.01,1.03)	0.02	5.1e-15***
Social Participation										
Ever been married	1.00		(1.00,1.00)	<0.01†	0.937	1.00		(1.00,1.00)	<0.01†	0.555
Currently divorced	1.00		(1.00,1.00)	<0.01†	0.989	1.00		(1.00,1.00)	<0.01†	0.137
Number of children		0.01	(0.00,0.01)	<0.01†	0.724		0.01	(0.00,0.02)	<0.01†	0.171
Weekly+ community participation	1.00		(1.00,1.01)	<0.01†	0.387	1.00		(1.00,1.01)	<0.01†	0.360
Weekly+ religious attendance	1.00		(1.00,1.00)	<0.01†	0.020*	1.00		(1.00,1.00)	<0.01†	0.085
Social Distress										
Loneliness		-0.04	(-0.05,-0.02)	0.02	5.11e-15***		-0.03	(-0.05,-0.02)	0.03	5.11e-15***
Perceived discrimination	1.00		(1.00,1.01)	<0.01†	1.9e-07***	1.00		(0.99,1.01)	0.01	1.47e-07***
Character & Prosocial Behavior										
Orientation to promote good (d)		0.08	(0.07,0.10)	0.03	1.7e-15***		0.08	(0.06,0.10)	0.04	1.7e-15***
Delayed gratification (d)		0.06	(0.05,0.08)	0.03	2.55e-15***		0.07	(0.05,0.09)	0.04	2.55e-15***
Hope		0.07	(0.06,0.09)	0.02	1.7e-15***		0.07	(0.06,0.09)	0.03	2.32e-15***
Gratitude		0.08	(0.06,0.09)	0.04	2.54e-15***		0.08	(0.06,0.10)	0.04	2.55e-15***
Showing love/care		0.08	(0.07,0.10)	0.03	2.54e-15***		0.08	(0.07,0.10)	0.03	2.55e-15***
Forgivingness	1.13		(1.10,1.16)	0.07	3.4e-16***	1.14		(1.11,1.17)	0.07	2.84e-16***
Charitable giving	1.02		(1.01,1.02)	<0.01†	6.56e-07***	1.02		(1.01,1.02)	0.01	7.58e-07***
Helping strangers	1.02		(1.01,1.03)	0.01	5.11e-15***	1.02		(1.01,1.03)	0.02	1.71e-13***
Volunteering	1.01		(1.00,1.01)	<0.01†	2.15e-04***	1.01		(1.00,1.01)	<0.01†	1.63e-05***
Physical Health & Health Behavior										
Self-rated physical health (b)		0.03	(0.02,0.04)	0.02	5.11e-15***		0.03	(0.01,0.04)	0.02	2.73e-12***
Health problems	1.00		(0.99,1.00)	<0.01†	0.206	1.00		(0.99,1.00)	<0.01†	0.078
Pain in past 4 weeks	1.00		(0.99,1.00)	<0.01†	0.443	1.00		(0.99,1.01)	<0.01†	0.172
Daily smoker	1.00		(1.00,1.00)	<0.01†	0.339	1.00		(1.00,1.00)	<0.01†	0.380
Number of drinks per week		-0.01	(-0.02,-0.00)	<0.01†	0.380		-0.01	(-0.02,-0.00)	<0.01†	0.459
Days exercise per week		0.03	(0.02,0.04)	0.01	3.39e-05***		0.02	(0.01,0.04)	0.02	5.78e-05***
Socioeconomic Outcomes										
Financial security (f)		0.02	(0.01,0.03)	0.02	6.84e-13***		0.02	(0.01,0.04)	0.02	1.66e-09***
Material security (f)		0.02	(0.01,0.04)	0.02	5.11e-15***		0.03	(0.01,0.04)	0.03	2.04e-14***
Educational attainment (16+ years)	1.00		(1.00,1.00)	<0.01†	0.821	1.00		(1.00,1.00)	<0.01†	0.229
Currently employed	1.00		(1.00,1.00)	<0.01†	0.971	1.00		(1.00,1.00)	<0.01†	0.434
Financially comfortable/getting by	1.00		(1.00,1.01)	<0.01†	4.55e-07***	1.00		(1.00,1.01)	<0.01†	3.01e-07***
Own home	1.00		(0.99,1.00)	<0.01†	0.016*	1.00		(0.99,1.00)	0.01	0.016*
Income – top quintile	1.00		(1.00,1.00)	<0.01†	0.896	1.00		(0.99,1.00)	<0.01†	0.810
Religion & Spirituality										
Religious/spiritual connection	1.02		(1.02,1.03)	0.01	5.1e-15***	1.02		(1.02,1.03)	<0.01†	1.35e-11***
Belief in life after death	1.01		(1.01,1.02)	0.01	1.21e-05***	1.01		(1.00,1.02)	0.02	8.6e-06***
Transformative religious experience	1.02		(1.01,1.02)	0.01	5.22e-12***	1.02		(1.01,1.03)	0.02	1.29e-08***
Religious reading or listening	1.01		(1.01,1.02)	<0.01†	5.11e-15***	1.01		(1.01,1.02)	0.01	2.42e-10***
Prayer or meditation	1.02		(1.01,1.02)	0.01	5.11e-15***	1.02		(1.01,1.02)	0.01	1.53e-14***
Belief in God/gods/spiritual forces	1.01		(1.00,1.01)	0.01	7.17e-04**	1.01		(1.00,1.01)	<0.01†	1.99e-05***
Religious centrality	1.01		(1.01,1.02)	<0.01†	5.11e-15***	1.01		(1.01,1.02)	<0.01†	5.11e-15***
Religious/spiritual comfort	1.01		(1.01,1.02)	<0.01†	5.11e-15***	1.01		(1.01,1.02)	<0.01†	1.06e-10***
Feel loved by God	1.01		(1.01,1.02)	<0.01†	2.04e-11***	1.01		(1.01,1.02)	<0.01†	9.57e-10***
Feel punished by God	1.00		(0.99,1.00)	<0.01†	0.015*	1.00		(0.99,1.00)	0.01	0.009*
Experienced religious criticism	1.00		(1.00,1.00)	<0.01†	0.419	1.00		(0.99,1.00)	<0.01†	0.066
Faith-sharing	1.02		(1.01,1.02)	0.02	8.32e-13***	1.02		(1.01,1.03)	0.02	5.52e-13***

Notes. N(multiple imputation)=207919; N(complete-case)=128868; Reference for focal predictor: never/rarely; RR, risk-ratio, null effect is 1.00; ES, effect size measure for standardized regression coefficient, null effect is 0.00; CI, confidence interval; τ (tau, heterogeneity), estimated standard deviation of the distribution of effects; Global p-value, joint test of the null hypothesis that the country-specific Wald tests are null in all countries; (a) item part of the Happiness & Life Satisfaction domain of the Secure Flourishing Index; (b) item part of the Physical & Mental Health domain of the Secure Flourishing Index; (c) item part of the Meaning & Purpose domain of the Secure Flourishing Index; (d) item part of the Character & Virtue domain of the Secure Flourishing Index; (e) item part of the Subjective Social Connectedness domain of the Secure Flourishing Index; (f) item part of the Financial & Material Security domain of the Secure Flourishing Index.

Multiple imputation was performed to impute missing data on the covariates, exposure, and outcomes. All models controlled for sociodemographic and childhood factors assessed at Wave 1.

An outcome-wide analytic approach was used, and a separate model was run for each outcome. A different type of model was run depending on the nature of the outcome: (1) for each binary outcome, a weighted generalized linear model (with a log link and Poisson distribution) was used to estimate an RR; and (2) for each continuous outcome, a weighted linear regression model was used to estimate an ES. All effect sizes were standardized. For continuous outcomes, the ES represents the change in SD on the outcome between the lower and upper categories of the binary focal predictor. For binary outcomes, the RR represents the change in risk of being in the upper category compared to the lower category between the lower and upper categories of the binary focal predictor.

P-value significance thresholds: $p < 0.05$*, $p < 0.005$**, (Bonferroni) $p < 6.41e-04$***, correction for multiple testing to significant threshold; † Estimate of τ (tau, heterogeneity) is likely unstable.

Table S6. Meta-analyzed associations of forgivingness at Wave 1 with well-being and other outcomes at Wave 2 for Model 2 by approach to address missingness (multiple imputation vs. complete case with attrition weights).

	Multiple Imputation					Complete Case with Attrition Weights				
Outcome	RR	ES	95% CI	τ	Global p-value	RR	ES	95% CI	τ	Global p-value
Human Flourishing										
Secure flourishing index		0.02	(0.01,0.02)	0.01	2.07e-03**		0.01	(0.01,0.02)	0.01	0.031*
Flourishing index		0.02	(0.01,0.02)	0.01	5.07e-04***		0.01	(0.01,0.02)	0.01	0.032*
Happiness & life satisfaction		-0.00	(-0.01,0.01)	<0.01†	0.195		0.00	(-0.01,0.01)	0.02	0.104
Physical & mental health		0.00	(-0.00,0.01)	<0.01†	0.797		0.00	(-0.00,0.01)	<0.01†	0.619
Meaning & purpose		0.00	(-0.01,0.01)	<0.01†	2.26e-03**		0.00	(-0.01,0.01)	0.01	0.033*
Character & virtue		0.04	(0.03,0.06)	0.02	4.18e-11***		0.05	(0.03,0.06)	0.03	1.44e-10***
Close social relationships		0.01	(0.01,0.02)	<0.01†	0.042*		0.01	(0.00,0.02)	<0.01†	0.144
Financial & material security		0.00	(-0.01,0.01)	0.01	0.007*		0.00	(-0.01,0.01)	0.01	0.165
Psychological Well-Being										
Happiness (a)		-0.00	(-0.01,0.01)	<0.01†	0.485		-0.00	(-0.01,0.01)	0.02	0.161
Life satisfaction (a)		-0.00	(-0.01,0.00)	<0.01†	0.441		0.00	(-0.01,0.01)	0.02	0.192
Current life evaluation		-0.01	(-0.02,0.00)	0.02	4.74e-03**		-0.01	(-0.02,0.01)	0.02	0.018*
Future life evaluation		0.00	(-0.01,0.02)	0.02	0.022*		0.01	(-0.01,0.02)	0.02	0.036*
Optimism		0.02	(0.01,0.03)	<0.01†	2.54e-04***		0.02	(0.01,0.03)	0.01	0.005*
Freedom to pursue what's important		-0.01	(-0.02,-0.00)	<0.01†	0.009*		-0.01	(-0.02,0.00)	0.02	0.005*
Inner peace	1.01		(1.00,1.01)	0.01	3.79e-05***	1.01		(1.00,1.01)	0.01	7.11e-06***
Life balance	1.01		(1.00,1.01)	<0.01†	1.33e-04***	1.01		(1.00,1.01)	<0.01†	2.39e-05***
Sense of mastery	1.00		(1.00,1.01)	<0.01†	0.034*	1.01		(1.00,1.01)	<0.01†	0.090
Meaningful activities (c)		-0.00	(-0.01,0.00)	<0.01†	0.163		0.00	(-0.01,0.01)	0.02	0.082
Understanding purpose (c)		0.00	(-0.00,0.01)	<0.01†	0.012*		0.00	(-0.01,0.01)	0.01	0.045*
Self-rated mental health (b)		0.00	(-0.00,0.01)	<0.01†	0.324		0.01	(-0.00,0.01)	0.01	0.057
Psychological Distress										
Traumatic distress	1.00		(0.99,1.00)	<0.01†	0.296	1.00		(0.99,1.00)	0.01	3.65e-03**
Depression symptoms composite	1.00		(1.00,1.00)	<0.01†	0.708	1.00		(1.00,1.01)	<0.01†	0.079
Depression – feel hopeless	1.00		(1.00,1.00)	<0.01†	0.726	1.00		(1.00,1.01)	<0.01†	0.050
Depression – loss of interest	1.00		(1.00,1.00)	<0.01†	0.850	1.00		(1.00,1.01)	<0.01†	0.094
Anxiety symptoms composite	1.00		(1.00,1.00)	<0.01†	0.240	1.00		(0.99,1.00)	<0.01†	0.098
Anxiety – feel on edge	1.00		(1.00,1.00)	<0.01†	0.945	1.00		(1.00,1.00)	<0.01†	0.888
Anxiety – cannot stop worrying	1.00		(1.00,1.00)	<0.01†	0.413	1.00		(1.00,1.00)	<0.01†	0.092
Suffering	1.00		(1.00,1.01)	<0.01†	0.646	1.00		(1.00,1.01)	<0.01†	0.028*
Social Well-Being										
Relationship contentment (e)		0.01	(0.01,0.02)	<0.01†	0.073		0.02	(0.01,0.02)	0.01	0.162
Relationship satisfaction (e)		0.01	(0.00,0.01)	<0.01†	0.508		0.01	(0.00,0.02)	0.01	0.094
Social support		0.02	(0.01,0.03)	0.02	5.29e-05***		0.02	(0.01,0.03)	0.03	1.12e-05***
Intimate/close friend	1.00		(1.00,1.01)	<0.01†	0.031*	1.01		(1.00,1.01)	<0.01†	2.21e-03**
Government approval	1.00		(1.00,1.01)	<0.01†	0.016*	1.00		(1.00,1.01)	<0.01†	0.050*
Say in government	1.00		(1.00,1.01)	<0.01†	0.174	1.00		(1.00,1.00)	<0.01†	0.454
Belonging in country		0.02	(0.01,0.02)	<0.01†	0.013*		0.02	(0.01,0.03)	<0.01†	0.017*
City/place satisfaction	1.01		(1.00,1.01)	<0.01†	1.07e-05***	1.01		(1.00,1.01)	<0.01†	1.25e-05***
Trust within country	1.01		(1.01,1.02)	<0.01†	2.2e-08***	1.01		(1.01,1.02)	0.01	1.13e-08***
Social Participation										
Ever been married	1.00		(1.00,1.00)	<0.01†	0.973	1.00		(1.00,1.00)	<0.01†	0.899
Currently divorced	1.00		(1.00,1.00)	<0.01†	0.991	1.00		(1.00,1.00)	<0.01†	0.566
Number of children		0.01	(-0.00,0.01)	<0.01†	0.250		0.01	(-0.00,0.02)	0.01	0.053
Weekly+ community participation	1.00		(1.00,1.00)	<0.01†	0.986	1.00		(1.00,1.00)	<0.01†	0.926
Weekly+ religious attendance	1.00		(1.00,1.00)	<0.01†	0.732	1.00		(1.00,1.00)	<0.01†	0.849
Social Distress										
Loneliness		0.00	(-0.00,0.01)	<0.01†	0.717		0.00	(-0.01,0.01)	<0.01†	0.276
Perceived discrimination	1.00		(1.00,1.01)	<0.01†	1.55e-05***	1.00		(1.00,1.01)	<0.01†	4.67e-06***
Character & Prosocial Behavior										
Orientation to promote good (d)		0.04	(0.03,0.05)	0.02	1.54e-12***		0.04	(0.03,0.05)	0.02	5.36e-12***
Delayed gratification (d)		0.03	(0.02,0.04)	0.02	5.33e-11***		0.03	(0.02,0.05)	0.03	6.19e-12***
Hope		0.02	(0.01,0.03)	<0.01†	0.016*		0.02	(0.01,0.03)	<0.01†	0.007*
Gratitude		0.03	(0.02,0.04)	0.01	2.04e-07***		0.03	(0.02,0.04)	0.02	5.03e-06***
Showing love/care		0.04	(0.03,0.05)	<0.01†	4.77e-06***		0.04	(0.03,0.05)	0.01	1.44e-04***
Forgivingness	1.12		(1.09,1.15)	0.07	4.54e-16***	1.13		(1.11,1.16)	0.06	3.39e-16***
Charitable giving	1.01		(1.00,1.01)	<0.01†	0.030*	1.01		(1.00,1.01)	0.01	0.014*
Helping strangers	1.01		(1.01,1.02)	<0.01†	1.36e-05***	1.01		(1.01,1.02)	0.01	1.59e-04***
Volunteering	1.00		(1.00,1.00)	<0.01†	0.451	1.00		(0.99,1.01)	0.01	0.093
Physical Health & Health Behavior										
Self-rated physical health (b)		0.00	(-0.01,0.01)	<0.01†	0.783		0.00	(-0.01,0.01)	<0.01†	0.297
Health problems	1.00		(1.00,1.00)	<0.01†	0.824	1.00		(1.00,1.00)	<0.01†	0.357
Pain in past 4 weeks	1.00		(1.00,1.00)	<0.01†	0.748	1.00		(1.00,1.01)	<0.01†	0.199
Daily smoker	1.00		(1.00,1.00)	<0.01†	0.052	1.00		(1.00,1.01)	<0.01†	0.027*
Number of drinks per week		-0.00	(-0.01,0.00)	<0.01†	0.776		-0.00	(-0.01,0.01)	<0.01†	0.411
Days exercise per week		0.01	(0.00,0.02)	0.01	0.063		0.01	(-0.00,0.02)	0.02	0.256
Socioeconomic Outcomes										
Financial security (f)		-0.00	(-0.01,0.00)	<0.01†	0.625		0.00	(-0.01,0.01)	0.01	0.213
Material security (f)		0.00	(-0.00,0.01)	<0.01†	0.109		0.01	(-0.01,0.02)	0.02	0.197
Educational attainment (16+ years)	1.00		(1.00,1.00)	<0.01†	0.854	1.00		(1.00,1.00)	<0.01†	0.383
Currently employed	1.00		(1.00,1.00)	<0.01†	0.969	1.00		(1.00,1.00)	<0.01†	0.353
Financially comfortable/getting by	1.00		(0.99,1.00)	<0.01†	0.574	1.00		(0.99,1.00)	<0.01†	0.666
Own home	1.00		(0.99,1.00)	0.01	0.033*	1.00		(0.99,1.00)	<0.01†	0.104
Income – top quintile	1.00		(1.00,1.00)	<0.01†	0.756	1.00		(0.99,1.00)	<0.01†	0.684
Religion & Spirituality										
Religious/spiritual connection	1.00		(1.00,1.01)	<0.01†	0.289	1.00		(1.00,1.01)	<0.01†	0.103
Belief in life after death	1.00		(0.99,1.00)	<0.01†	0.071	1.00		(0.99,1.01)	0.01	0.027*
Transformative religious experience	1.00		(1.00,1.01)	<0.01†	0.157	1.01		(1.00,1.01)	0.01	0.022*
Religious reading or listening	1.00		(1.00,1.01)	<0.01†	3.11e-03**	1.00		(1.00,1.01)	<0.01†	0.027*
Prayer or meditation	1.00		(1.00,1.01)	<0.01†	0.034*	1.00		(1.00,1.01)	0.01	0.068
Belief in God/gods/spiritual forces	1.00		(1.00,1.00)	<0.01†	0.491	1.00		(0.99,1.00)	<0.01†	0.090
Religious centrality	1.00		(1.00,1.00)	<0.01†	0.142	1.00		(1.00,1.00)	<0.01†	0.374
Religious/spiritual comfort	1.00		(1.00,1.00)	<0.01†	0.663	1.00		(1.00,1.00)	<0.01†	0.340
Feel loved by God	1.00		(1.00,1.00)	<0.01†	0.737	1.00		(1.00,1.00)	<0.01†	0.904
Feel punished by God	1.00		(1.00,1.00)	<0.01†	0.131	1.00		(0.99,1.00)	0.01	0.048*
Experienced religious criticism	1.00		(1.00,1.00)	<0.01†	0.809	1.00		(1.00,1.00)	<0.01†	0.110
Faith-sharing	1.01		(1.00,1.01)	<0.01†	0.016*	1.01		(1.00,1.01)	0.01	3.55e-03**

Notes. N(multiple imputation)=207919; N(complete-case)=128868; Reference for focal predictor: never/rarely; RR, risk-ratio, null effect is 1.00; ES, effect size measure for standardized regression coefficient, null effect is 0.00; CI, confidence interval; τ (tau, heterogeneity), estimated standard deviation of the distribution of effects; Global p-value, joint test of the null hypothesis that the country-specific Wald tests are null in all countries; (a) item part of the Happiness & Life Satisfaction domain of the Secure Flourishing Index; (b) item part of the Physical & Mental Health domain of the Secure Flourishing Index; (c) item part of the Meaning & Purpose domain of the Secure Flourishing Index; (d) item part of the Character & Virtue domain of the Secure Flourishing Index; (e) item part of the Subjective Social Connectedness domain of the Secure Flourishing Index; (f) item part of the Financial & Material Security domain of the Secure Flourishing Index.

Multiple imputation was performed to impute missing data on the covariates, exposure, and outcomes. All models controlled for sociodemographic and childhood factors assessed at Wave 1. For the PC (principal components), the first seven principal components of the entire set of contemporaneous confounders assessed at Wave 1 were included as additional covariates of the outcomes at Wave 2.

An outcome-wide analytic approach was used, and a separate model was run for each outcome. A different type of model was run depending on the nature of the outcome: (1) for each binary outcome, a weighted generalized linear model (with a log link and Poisson distribution) was used to estimate an RR; and (2) for each continuous outcome, a weighted linear regression model was used to estimate an ES. All effect sizes were standardized. For continuous outcomes, the ES represents the change in SD on the outcome between the lower and upper categories of the binary focal predictor. For binary outcomes, the RR represents the change in risk of being in the upper category compared to the lower category between the lower and upper categories of the binary focal predictor.

P-value significance thresholds: $p < 0.05$*, $p < 0.005$**, (Bonferroni) $p < 6.41e-04$***, correction for multiple testing to significant threshold; † Estimate of τ (tau, heterogeneity) is likely unstable.

Table S7. Forgivingness for comparing estimated E-values across models and how missingness at Wave 2 was handled.

	Multiple Imputation				Complete Case w/ Attrition Weights			
	Model 1: Demographics and Childhood Variables as Covariates		Model 2: Demographics, Childhood, and Other Wave 1 Confounders (Via Principal Components) as Covariates		Model 1: Demographics and Childhood Variables as Covariates		Model 2: Demographics, Childhood, and Other Wave 1 Confounders (Via Principal Components) as Covariates	
Outcome	EE	ECI	EE	ECI	EE	ECI	EE	ECI
Human Flourishing								
Secure flourishing index	1.34	1.29	1.14	1.09	1.33	1.28	1.13	1.07
Flourishing index	1.35	1.31	1.14	1.10	1.34	1.29	1.13	1.08
Happiness & life satisfaction	1.25	1.19	1.02	1.00	1.24	1.18	1.00	1.00
Physical & mental health	1.24	1.20	1.05	1.00	1.25	1.20	1.04	1.00
Meaning & purpose	1.30	1.26	1.04	1.00	1.30	1.25	1.05	1.00
Character & virtue	1.38	1.33	1.25	1.20	1.38	1.32	1.25	1.19
Close social relationships	1.31	1.27	1.12	1.07	1.31	1.26	1.12	1.07
Financial & material security	1.17	1.11	1.05	1.00	1.18	1.12	1.06	1.00
Psychological Well-Being								
Happiness	1.24	1.18	1.02	1.00	1.23	1.16	1.05	1.00
Life satisfaction	1.23	1.18	1.05	1.00	1.24	1.18	1.03	1.00
Current life evaluation	1.18	1.12	1.08	1.00	1.19	1.12	1.08	1.00
Future life evaluation	1.23	1.18	1.07	1.00	1.25	1.19	1.08	1.00
Optimism	1.30	1.25	1.15	1.12	1.31	1.25	1.14	1.08
Freedom to pursue what's important	1.22	1.17	1.09	1.01	1.21	1.15	1.10	1.00
Inner peace	1.17	1.14	1.10	1.06	1.19	1.15	1.10	1.06
Life balance	1.17	1.14	1.09	1.06	1.18	1.15	1.09	1.06
Sense of mastery	1.15	1.12	1.07	1.04	1.16	1.13	1.08	1.03
Meaningful activities	1.26	1.21	1.06	1.00	1.27	1.21	1.03	1.00
Understanding purpose	1.29	1.25	1.06	1.00	1.29	1.25	1.06	1.00
Self-rated mental health	1.26	1.21	1.06	1.00	1.27	1.22	1.08	1.00
Psychological Distress								
Traumatic distress	1.03	1.00	1.02	1.00	1.05	1.00	1.04	1.00
Depression symptoms composite	1.09	1.04	1.05	1.00	1.07	1.00	1.06	1.00
Depression – feel hopeless	1.08	1.00	1.04	1.00	1.08	1.00	1.05	1.00
Depression – loss of interest	1.08	1.00	1.04	1.00	1.07	1.00	1.05	1.00
Anxiety symptoms composite	1.11	1.07	1.05	1.00	1.11	1.07	1.05	1.00
Anxiety – feel on edge	1.09	1.05	1.01	1.00	1.10	1.07	1.02	1.00
Anxiety – cannot stop worrying	1.09	1.03	1.03	1.00	1.09	1.04	1.04	1.00
Suffering	1.07	1.00	1.05	1.00	1.08	1.00	1.05	1.00
Social Well-Being								
Relationship contentment	1.30	1.26	1.12	1.08	1.31	1.26	1.13	1.08
Relationship satisfaction	1.28	1.24	1.09	1.02	1.28	1.24	1.10	1.01
Social support	1.26	1.20	1.15	1.09	1.27	1.20	1.16	1.08
Intimate/close friend	1.14	1.11	1.07	1.05	1.14	1.10	1.08	1.03
Government approval	1.13	1.09	1.06	1.03	1.14	1.10	1.06	1.00
Say in government	1.13	1.09	1.06	1.00	1.13	1.09	1.04	1.00
Belonging in country	1.28	1.24	1.14	1.11	1.29	1.25	1.15	1.11
City/place satisfaction	1.13	1.10	1.09	1.04	1.14	1.11	1.10	1.05
Trust within country	1.16	1.13	1.12	1.09	1.16	1.12	1.13	1.09
Social Participation								
Ever been married	1.04	1.02	1.04	1.00	1.05	1.03	1.05	1.02
Currently divorced	1.01	1.00	1.01	1.00	1.00	1.00	1.01	1.00
Number of children	1.09	1.03	1.09	1.00	1.11	1.05	1.10	1.00
Weekly+ community participation	1.07	1.05	1.03	1.00	1.07	1.05	1.04	1.00
Weekly+ religious attendance	1.05	1.03	1.02	1.00	1.05	1.03	1.01	1.00
Social Distress								
Loneliness	1.22	1.17	1.04	1.00	1.20	1.14	1.05	1.00
Perceived discrimination	1.04	1.00	1.03	1.00	1.03	1.00	1.03	1.00
Character & Prosocial Behavior								
Orientation to promote good	1.37	1.32	1.24	1.20	1.37	1.31	1.23	1.19
Delayed gratification	1.30	1.25	1.20	1.15	1.32	1.25	1.22	1.15
Hope	1.34	1.30	1.16	1.12	1.34	1.29	1.16	1.12
Gratitude	1.35	1.29	1.20	1.16	1.35	1.29	1.20	1.14
Showing love/care	1.36	1.32	1.23	1.20	1.37	1.33	1.23	1.19
Forgivingness	1.51	1.43	1.49	1.42	1.54	1.47	1.52	1.45
Charitable giving	1.14	1.11	1.08	1.06	1.14	1.11	1.08	1.00
Helping strangers	1.17	1.14	1.12	1.08	1.17	1.13	1.12	1.08
Volunteering	1.10	1.06	1.02	1.00	1.09	1.04	1.00	1.00
Physical Health & Health Behavior								
Self-rated physical health	1.19	1.14	1.04	1.00	1.18	1.13	1.01	1.00
Health problems	1.05	1.00	1.03	1.00	1.05	1.00	1.05	1.00
Pain in past 4 weeks	1.05	1.00	1.02	1.00	1.01	1.00	1.05	1.00
Daily smoker	1.01	1.00	1.05	1.02	1.03	1.00	1.06	1.04
Number of drinks per week	1.11	1.07	1.06	1.00	1.09	1.02	1.05	1.00
Days exercise per week	1.18	1.13	1.11	1.04	1.17	1.12	1.11	1.00
Socioeconomic Outcomes								
Financial security	1.15	1.09	1.04	1.00	1.17	1.11	1.05	1.00
Material security	1.17	1.12	1.07	1.00	1.18	1.12	1.07	1.00
Educational attainment (16+ years)	1.00	1.00	1.00	1.00	1.00	1.00	1.00	1.00
Currently employed	1.01	1.00	1.03	1.00	1.00	1.00	1.03	1.00
Financially comfortable/getting by	1.04	1.00	1.06	1.01	1.05	1.00	1.05	1.00
Own home	1.06	1.01	1.06	1.00	1.05	1.00	1.05	1.00
Income – top quintile	1.04	1.00	1.05	1.00	1.05	1.00	1.06	1.00
Religion & Spirituality								
Religious/spiritual connection	1.18	1.16	1.08	1.05	1.18	1.16	1.07	1.04
Belief in life after death	1.12	1.08	1.03	1.00	1.13	1.07	1.02	1.00
Transformative religious experience	1.15	1.11	1.07	1.00	1.16	1.11	1.09	1.03
Religious reading or listening	1.12	1.09	1.06	1.00	1.12	1.08	1.07	1.00
Prayer or meditation	1.15	1.11	1.07	1.00	1.14	1.10	1.06	1.00
Belief in God/gods/spiritual forces	1.09	1.05	1.00	1.00	1.10	1.06	1.02	1.00
Religious centrality	1.13	1.10	1.04	1.00	1.13	1.10	1.03	1.00
Religious/spiritual comfort	1.13	1.11	1.01	1.00	1.13	1.10	1.02	1.00
Feel loved by God	1.12	1.10	1.01	1.00	1.12	1.09	1.01	1.00
Feel punished by God	1.06	1.00	1.05	1.03	1.06	1.00	1.06	1.00
Experienced religious criticism	1.03	1.00	1.04	1.02	1.06	1.00	1.06	1.04
Faith-sharing	1.15	1.11	1.08	1.03	1.16	1.11	1.09	1.01

Notes. EE, E-value for estimate; ECI, E-value for the limit of the confidence interval. The formula for calculating E-values can be found in VanderWeele and Ding (2017). E-values for estimate are the minimum strength of association on the risk ratio scale that an unmeasured confounder would need to have with both the exposure and the outcome to fully explain away the observed association between the exposure and outcome, conditional on the measured covariates. E-values for the 95% CI closest to the null denote the minimum strength of association on the risk ratio scale that an unmeasured confounder would need to have with both the exposure and the outcome to shift the CI to include the null value, conditional on the measured covariates.

Table S8. Unstandardized effects sizes for the raw score of forgivingness (multiple imputation results only).

	Model 1: Demographic and Childhood Variables as Covariates					Model 2: Demographic, Childhood, and Other Wave 1 Confounding Variables (Via Principal Components) as Covariates				
Outcome	RR	ES	95% CI	τ	Global p-value	RR	ES	95% CI	τ	Global p-value
Human Flourishing										
Secure flourishing index		0.02	(0.01,0.02)	0.01	2.07e-03**		0.01	(0.01,0.02)	0.01	0.031*
Flourishing index		0.02	(0.01,0.02)	0.01	5.07e-04***		0.01	(0.01,0.02)	0.01	0.032*
Happiness & life satisfaction		-0.00	(-0.01,0.01)	<0.01†	0.195		0.00	(-0.01,0.01)	0.02	0.104
Physical & mental health		0.00	(-0.00,0.01)	<0.01†	0.797		0.00	(-0.00,0.01)	<0.01†	0.619
Meaning & purpose		0.00	(-0.01,0.01)	<0.01†	2.26e-03**		0.00	(-0.01,0.01)	0.01	0.033*
Character & virtue		0.04	(0.03,0.06)	0.02	4.18e-11***		0.05	(0.03,0.06)	0.03	1.44e-10***
Close social relationships		0.01	(0.01,0.02)	<0.01†	0.042*		0.01	(0.00,0.02)	<0.01†	0.144
Financial & material security		0.00	(-0.01,0.01)	0.01	0.007*		0.00	(-0.01,0.01)	0.01	0.165
Psychological Well-Being										
Happiness (a)		-0.00	(-0.01,0.01)	<0.01†	0.485		-0.00	(-0.01,0.01)	0.02	0.161
Life satisfaction (a)		-0.00	(-0.01,0.00)	<0.01†	0.441		0.00	(-0.01,0.01)	0.02	0.192
Current life evaluation		-0.01	(-0.02,0.00)	0.02	4.74e-03**		-0.01	(-0.02,0.01)	0.02	0.018*
Future life evaluation		0.00	(-0.01,0.02)	0.02	0.022*		0.01	(-0.01,0.02)	0.02	0.036*
Optimism		0.02	(0.01,0.03)	<0.01†	2.54e-04***		0.02	(0.01,0.03)	0.01	0.005*
Freedom to pursue what's important		-0.01	(-0.02,-0.00)	<0.01†	0.009*		-0.01	(-0.02,0.00)	0.02	0.005*
Inner peace	1.01		(1.00,1.01)	0.01	3.79e-05***	1.01		(1.00,1.01)	0.01	7.11e-06***
Life balance	1.01		(1.00,1.01)	<0.01†	1.33e-04***	1.01		(1.00,1.01)	<0.01†	2.39e-05***
Sense of mastery	1.00		(1.00,1.01)	<0.01†	0.034*	1.01		(1.00,1.01)	<0.01†	0.090
Meaningful activities (c)		-0.00	(-0.01,0.00)	<0.01†	0.163		0.00	(-0.01,0.01)	0.02	0.082
Understanding purpose (c)		0.00	(-0.00,0.01)	<0.01†	0.012*		0.00	(-0.01,0.01)	0.01	0.045*
Self-rated mental health (b)		0.00	(-0.00,0.01)	<0.01†	0.324		0.01	(-0.00,0.01)	0.01	0.057
Psychological Distress										
Traumatic distress	1.00		(0.99,1.00)	<0.01†	0.296	1.00		(0.99,1.00)	0.01	3.65e-03**
Depression symptoms composite	1.00		(1.00,1.00)	<0.01†	0.708	1.00		(1.00,1.01)	<0.01†	0.079
Depression – feel hopeless	1.00		(1.00,1.00)	<0.01†	0.726	1.00		(1.00,1.01)	<0.01†	0.050
Depression – loss of interest	1.00		(1.00,1.00)	<0.01†	0.850	1.00		(1.00,1.01)	<0.01†	0.094
Anxiety symptoms composite	1.00		(1.00,1.00)	<0.01†	0.240	1.00		(0.99,1.00)	<0.01†	0.098
Anxiety – feel on edge	1.00		(1.00,1.00)	<0.01†	0.945	1.00		(1.00,1.00)	<0.01†	0.888
Anxiety – cannot stop worrying	1.00		(1.00,1.00)	<0.01†	0.413	1.00		(1.00,1.00)	<0.01†	0.092
Suffering	1.00		(1.00,1.01)	<0.01†	0.646	1.00		(1.00,1.01)	<0.01†	0.028*
Social Well-Being										
Relationship contentment (e)		0.01	(0.01,0.02)	<0.01†	0.073		0.02	(0.01,0.02)	0.01	0.162
Relationship satisfaction (e)		0.01	(0.00,0.01)	<0.01†	0.508		0.01	(0.00,0.02)	0.01	0.094
Social support		0.02	(0.01,0.03)	0.02	5.29e-05***		0.02	(0.01,0.03)	0.03	1.12e-05***
Intimate/close friend	1.00		(1.00,1.01)	<0.01†	0.031*	1.01		(1.00,1.01)	<0.01†	2.21e-03**
Government approval	1.00		(1.00,1.01)	<0.01†	0.016*	1.00		(1.00,1.01)	<0.01†	0.050*
Say in government	1.00		(1.00,1.01)	<0.01†	0.174	1.00		(1.00,1.00)	<0.01†	0.454
Belonging in country		0.02	(0.01,0.02)	<0.01†	0.013*		0.02	(0.01,0.03)	<0.01†	0.017*
City/place satisfaction	1.01		(1.00,1.01)	<0.01†	1.07e-05***	1.01		(1.00,1.01)	<0.01†	1.25e-05***
Trust within country	1.01		(1.01,1.02)	<0.01†	2.2e-08***	1.01		(1.01,1.02)	0.01	1.13e-08***
Social Participation										
Ever been married	1.00		(1.00,1.00)	<0.01†	0.973	1.00		(1.00,1.00)	<0.01†	0.899
Currently divorced	1.00		(1.00,1.00)	<0.01†	0.991	1.00		(1.00,1.00)	<0.01†	0.566
Number of children		0.01	(-0.00,0.01)	<0.01†	0.250		0.01	(-0.00,0.02)	0.01	0.053
Weekly+ community participation	1.00		(1.00,1.00)	<0.01†	0.986	1.00		(1.00,1.00)	<0.01†	0.926
Weekly+ religious attendance	1.00		(1.00,1.00)	<0.01†	0.732	1.00		(1.00,1.00)	<0.01†	0.849
Social Distress										
Loneliness		0.00	(-0.00,0.01)	<0.01†	0.717		0.00	(-0.01,0.01)	<0.01†	0.276
Perceived discrimination	1.00		(1.00,1.01)	<0.01†	1.55e-05***	1.00		(1.00,1.01)	<0.01†	4.67e-06***
Character & Prosocial Behavior										
Orientation to promote good (d)		0.04	(0.03,0.05)	0.02	1.54e-12***		0.04	(0.03,0.05)	0.02	5.36e-12***
Delayed gratification (d)		0.03	(0.02,0.04)	0.02	5.33e-11***		0.03	(0.02,0.05)	0.03	6.19e-12***
Hope		0.02	(0.01,0.03)	<0.01†	0.016*		0.02	(0.01,0.03)	<0.01†	0.007*
Gratitude		0.03	(0.02,0.04)	0.01	2.04e-07***		0.03	(0.02,0.04)	0.02	5.03e-06***
Showing love/care		0.04	(0.03,0.05)	<0.01†	4.77e-06***		0.04	(0.03,0.05)	0.01	1.44e-04***
Forgivingness	1.12		(1.09,1.15)	0.07	4.54e-16***	1.13		(1.11,1.16)	0.06	3.39e-16***
Charitable giving	1.01		(1.00,1.01)	<0.01†	0.030*	1.01		(1.00,1.01)	0.01	0.014*
Helping strangers	1.01		(1.01,1.02)	<0.01†	1.36e-05***	1.01		(1.01,1.02)	0.01	1.59e-04***
Volunteering	1.00		(1.00,1.00)	<0.01†	0.451	1.00		(0.99,1.01)	0.01	0.093
Physical Health & Health Behavior										
Self-rated physical health (b)		0.00	(-0.01,0.01)	<0.01†	0.783		0.00	(-0.01,0.01)	<0.01†	0.297
Health problems	1.00		(1.00,1.00)	<0.01†	0.824	1.00		(1.00,1.00)	<0.01†	0.357
Pain in past 4 weeks	1.00		(1.00,1.00)	<0.01†	0.748	1.00		(1.00,1.01)	<0.01†	0.199
Daily smoker	1.00		(1.00,1.00)	<0.01†	0.052	1.00		(1.00,1.01)	<0.01†	0.027*
Number of drinks per week		-0.00	(-0.01,0.00)	<0.01†	0.776		-0.00	(-0.01,0.01)	<0.01†	0.411
Days exercise per week		0.01	(0.00,0.02)	0.01	0.063		0.01	(-0.00,0.02)	0.02	0.256
Socioeconomic Outcomes										
Financial security (f)		-0.00	(-0.01,0.00)	<0.01†	0.625		0.00	(-0.01,0.01)	0.01	0.213
Material security (f)		0.00	(-0.00,0.01)	<0.01†	0.109		0.01	(-0.01,0.02)	0.02	0.197
Educational attainment (16+ years)	1.00		(1.00,1.00)	<0.01†	0.854	1.00		(1.00,1.00)	<0.01†	0.383
Currently employed	1.00		(1.00,1.00)	<0.01†	0.969	1.00		(1.00,1.00)	<0.01†	0.353
Financially comfortable/getting by	1.00		(0.99,1.00)	<0.01†	0.574	1.00		(0.99,1.00)	<0.01†	0.666
Own home	1.00		(0.99,1.00)	0.01	0.033*	1.00		(0.99,1.00)	<0.01†	0.104
Income – top quintile	1.00		(1.00,1.00)	<0.01†	0.756	1.00		(0.99,1.00)	<0.01†	0.684
Religion & Spirituality										
Religious/spiritual connection	1.00		(1.00,1.01)	<0.01†	0.289	1.00		(1.00,1.01)	<0.01†	0.103
Belief in life after death	1.00		(0.99,1.00)	<0.01†	0.071	1.00		(0.99,1.01)	0.01	0.027*
Transformative religious experience	1.00		(1.00,1.01)	<0.01†	0.157	1.01		(1.00,1.01)	0.01	0.022*
Religious reading or listening	1.00		(1.00,1.01)	<0.01†	3.11e-03**	1.00		(1.00,1.01)	<0.01†	0.027*
Prayer or meditation	1.00		(1.00,1.01)	<0.01†	0.034*	1.00		(1.00,1.01)	0.01	0.068
Belief in God/gods/spiritual forces	1.00		(1.00,1.00)	<0.01†	0.491	1.00		(0.99,1.00)	<0.01†	0.090
Religious centrality	1.00		(1.00,1.00)	<0.01†	0.142	1.00		(1.00,1.00)	<0.01†	0.374
Religious/spiritual comfort	1.00		(1.00,1.00)	<0.01†	0.663	1.00		(1.00,1.00)	<0.01†	0.340
Feel loved by God	1.00		(1.00,1.00)	<0.01†	0.737	1.00		(1.00,1.00)	<0.01†	0.904
Feel punished by God	1.00		(1.00,1.00)	<0.01†	0.131	1.00		(0.99,1.00)	0.01	0.048*
Experienced religious criticism	1.00		(1.00,1.00)	<0.01†	0.809	1.00		(1.00,1.00)	<0.01†	0.110
Faith-sharing	1.01		(1.00,1.01)	<0.01†	0.016*	1.01		(1.00,1.01)	0.01	3.55e-03**

Notes. N(multiple imputation)=207919; N(complete-case)=128868; Reference for focal predictor: never/rarely; RR, risk-ratio, null effect is 1.00; ES, effect size measure for unstandardized regression coefficient, null effect is 0.00; CI, confidence interval; τ (tau, heterogeneity), estimated standard deviation of the distribution of effects; Global p-value, joint test of the null hypothesis that the country-specific Wald tests are null in all countries; (a) item part of the Happiness & Life Satisfaction domain of the Secure Flourishing Index; (b) item part of the Physical & Mental Health domain of the Secure Flourishing Index; (c) item part of the Meaning & Purpose domain of the Secure Flourishing Index; (d) item part of the Character & Virtue domain of the Secure Flourishing Index; (e) item part of the Subjective Social Connectedness domain of the Secure Flourishing Index; (f) item part of the Financial & Material Security domain of the Secure Flourishing Index.

Multiple imputation was performed to impute missing data on the covariates, exposure, and outcomes. All models controlled for sociodemographic and childhood factors assessed at Wave 1. For the PC (principal components), the first seven principal components of the entire set of contemporaneous confounders assessed at Wave 1 were included as additional covariates of the outcomes at Wave 2.

An outcome-wide analytic approach was used, and a separate model was run for each outcome. A different type of model was run depending on the nature of the outcome: (1) for each binary outcome, a weighted generalized linear model (with a log link and Poisson distribution) was used to estimate an RR; and (2) for each continuous outcome, a weighted linear regression model was used to estimate an ES, and no standardization was conducted prior to estimating the model.

P-value significance thresholds: p < 0.05*, p < 0.005**, (Bonferroni) p < 6.41e-04***, correction for multiple testing to significant threshold; † Estimate of τ (tau, heterogeneity) is likely unstable.

Table S9a. Weighted summary statistics for demographic and childhood variables in Argentina

**Characteristic**	**Wave 1** N = 6,724	**Wave 2** N = 2,889
Forgivingness, n (%)		
Always	2,446 (36.4%)	970 (33.6%)
Often	2,486 (37.0%)	1,221 (42.3%)
Rarely	1,336 (19.9%)	540 (18.7%)
Never	426 (6.3%)	154 (5.3%)
(Missing)	29 (0.4%)	5 (0.2%)
Year of birth, n (%)		
1943 or earlier (current age: 80+ years)	111 (1.6%)	60 (2.1%)
1943-1953 (current age: 70-79 years)	338 (5.0%)	167 (5.8%)
1953-1963 (current age: 60-69 years)	703 (10.5%)	331 (11.4%)
1963-1973 (current age: 50-59 years)	982 (14.6%)	455 (15.7%)
1973-1983 (current age: 40-49 years)	1,220 (18.1%)	573 (19.8%)
1983-1993 (current age: 30-39 years)	1,434 (21.3%)	615 (21.3%)
1993-1998 (current age: 25-29 years)	724 (10.8%)	298 (10.3%)
1998-2005 (current age: 18-24 years)	1,212 (18.0%)	390 (13.5%)
(Missing)	0 (0%)	0 (0%)
Age of participant		
Mean	41.8	43.8
Standard Deviation	16.7	16.6
Min, Max	18.0, 99.0	18.0, 96.0
Gender, n (%)		
Male	3,159 (47.0%)	1,331 (46.1%)
Female	3,526 (52.4%)	1,538 (53.2%)
Other	20 (0.3%)	7 (0.2%)
(Missing)	19 (0.3%)	13 (0.4%)
Respondent marital status, n (%)		
Single/Never been married	2,446 (36.4%)	1,012 (35.0%)
Married	1,523 (22.7%)	743 (25.7%)
Separated	445 (6.6%)	182 (6.3%)
Divorced	306 (4.6%)	135 (4.7%)
Widowed	395 (5.9%)	169 (5.9%)
Domestic partner	1,522 (22.6%)	623 (21.6%)
(Missing)	88 (1.3%)	25 (0.9%)
Education (years), n (%)		
Up to 8	2,258 (33.6%)	706 (24.5%)
9-15	3,856 (57.4%)	1,894 (65.6%)
16+	607 (9.0%)	289 (10.0%)
(Missing)	3 (0.0%)	0 (0%)
Employment status, n (%)		
Employed for an employer	2,499 (37.2%)	929 (32.1%)
Self-employed	1,751 (26.0%)	889 (30.8%)
Retired	734 (10.9%)	362 (12.5%)
Student	358 (5.3%)	143 (5.0%)
Homemaker	618 (9.2%)	261 (9.0%)
Unemployed and looking for a job	561 (8.3%)	187 (6.5%)
None of these/Other	180 (2.7%)	63 (2.2%)
(Missing)	22 (0.3%)	55 (1.9%)
Current religious service attendance, n (%)		
More than once a week	519 (7.7%)	241 (8.3%)
Once a week	771 (11.5%)	259 (9.0%)

Table S9a. Weighted summary statistics for demographic and childhood variables in Argentina

**Characteristic**	**Wave 1**<br>N = 6,724	**Wave 2**<br>N = 2,889
One to three times a month	464 (6.9%)	174 (6.0%)
A few times a year	1,927 (28.7%)	861 (29.8%)
Never	3,014 (44.8%)	1,347 (46.6%)
(Missing)	28 (0.4%)	7 (0.2%)
Immigration status, n (%)		
Born in this country	6,338 (94.3%)	2,733 (94.6%)
Born in another country	357 (5.3%)	152 (5.3%)
(Missing)	29 (0.4%)	4 (0.1%)
Parental marital status around age 12, n (%)		
Parents were married	4,071 (60.5%)	1,829 (63.3%)
Parents were divorced	636 (9.5%)	279 (9.7%)
Parents were never married	1,395 (20.7%)	557 (19.3%)
One or both of them had died	199 (3.0%)	107 (3.7%)
Unsure	238 (3.5%)	75 (2.6%)
(Missing)	186 (2.8%)	43 (1.5%)
Religious service attendance around age 12, n (%)		
At least once a week	2,594 (38.6%)	1,049 (36.3%)
One to three times a month	1,216 (18.1%)	522 (18.1%)
Less than once a month	1,056 (15.7%)	490 (17.0%)
Never	1,806 (26.9%)	805 (27.9%)
(Missing)	53 (0.8%)	23 (0.8%)
Relationship with mother when growing up, n (%)		
Very good	4,440 (66.0%)	1,850 (64.0%)
Somewhat good	1,445 (21.5%)	672 (23.3%)
Somewhat bad	310 (4.6%)	140 (4.9%)
Very bad	218 (3.2%)	85 (2.9%)
(Does not apply)	273 (4.1%)	125 (4.3%)
(Missing)	37 (0.6%)	17 (0.6%)
Relationship with father when growing up, n (%)		
Very good	3,557 (52.9%)	1,571 (54.4%)
Somewhat good	1,550 (23.0%)	651 (22.5%)
Somewhat bad	452 (6.7%)	213 (7.4%)
Very bad	417 (6.2%)	174 (6.0%)
(Does not apply)	711 (10.6%)	273 (9.4%)
(Missing)	37 (0.6%)	8 (0.3%)
Felt like an outsider in family when growing up, n (%)		
Yes	1,217 (18.1%)	491 (17.0%)
No	5,399 (80.3%)	2,351 (81.4%)
(Missing)	109 (1.6%)	47 (1.6%)
Experienced abuse when growing up, n (%)		
Yes	1,312 (19.5%)	576 (19.9%)
No	5,260 (78.2%)	2,284 (79.1%)
(Missing)	153 (2.3%)	29 (1.0%)
Self-rated health when growing up, n (%)		
Excellent	2,401 (35.7%)	1,003 (34.7%)
Very good	1,814 (27.0%)	802 (27.8%)
Good	1,827 (27.2%)	798 (27.6%)
Fair	515 (7.7%)	225 (7.8%)
Poor	153 (2.3%)	58 (2.0%)
(Missing)	14 (0.2%)	3 (0.1%)

Table S9a. Weighted summary statistics for demographic and childhood variables in Argentina

**Characteristic**	**Wave 1**<br>N = 6,724	**Wave 2**<br>N = 2,889
Subjective financial status of family growing up, n (%)		
Lived comfortably	2,037 (30.3%)	809 (28.0%)
Got by	2,305 (34.3%)	1,021 (35.3%)
Found it difficult	1,786 (26.6%)	798 (27.6%)
Found it very difficult	576 (8.6%)	252 (8.7%)
(Missing)	20 (0.3%)	9 (0.3%)
Religious affiliation growing up, n (%)		
Christianity	5,790 (86.1%)	2,533 (87.7%)
Taoism	1 (0.0%)	1 (0.0%)
Confucianism	0 (0%)	0 (0%)
Primal, Animist, or Folk religion	17 (0.2%)	5 (0.2%)
Spiritism	0 (0%)	0 (0%)
Umbanda, Candomblé, and other African-derived religions	0 (0%)	0 (0%)
Chinese folk/traditional religion	0 (0%)	0 (0%)
Islam	11 (0.2%)	0 (0.0%)
Hinduism	3 (0.0%)	0 (0%)
Buddhism	3 (0.0%)	1 (0.0%)
Judaism	49 (0.7%)	20 (0.7%)
Sikhism	4 (0.1%)	0 (0%)
Baha'i	0 (0%)	0 (0%)
Jainism	0 (0%)	0 (0%)
Shinto	0 (0%)	0 (0%)
Some other religion	11 (0.2%)	10 (0.4%)
No religion/Atheist/Agnostic	705 (10.5%)	275 (9.5%)
(Missing)	130 (1.9%)	43 (1.5%)

Note. N (%); this table is based on non-imputed data. Cumulative percentages for variables may not add up to 100% due to rounding. Wave 1 characteristics weighted using the Gallup provided sampling weight, ANNUAL_WEIGHT_R2; Wave 2 characteristics weighted accounting for attrition by using the adjusted Wave 1 weight, ANNUAL_WEIGHT_R2, multiplied by the created attrition weight to account for dropout, to maintain nationally representative estimates for Wave 2 characteristics.

Table S9b. Weighted summary statistics for outcome variables in Argentina

**Outcome**	**Wave 1** N = 6,724	**Wave 2** N = 2,889
Secure flourishing index		
Mean	7.1	7.1
Standard Deviation	1.5	1.4
Min, Max	0.4, 10.0	0.4, 10.0
(Missing)	207 (3.1%)	56 (1.9%)
Flourishing index		
Mean	7.8	7.7
Standard Deviation	1.5	1.4
Min, Max	0.5, 10.0	0.0, 10.0
(Missing)	191 (2.8%)	49 (1.7%)
Happiness & life satisfaction		
Mean	7.3	7.1
Standard Deviation	2.1	2.0
Min, Max	0.0, 10.0	0.0, 10.0
(Missing)	31 (0.5%)	5 (0.2%)
Physical & mental health		
Mean	7.6	7.3
Standard Deviation	1.9	1.8
Min, Max	0.0, 10.0	0.0, 10.0
(Missing)	22 (0.3%)	11 (0.4%)
Meaning & purpose		
Mean	7.9	7.8
Standard Deviation	2.0	2.0
Min, Max	0.0, 10.0	0.0, 10.0
(Missing)	45 (0.7%)	13 (0.4%)
Character & virtue		
Mean	8.4	8.2
Standard Deviation	1.6	1.6
Min, Max	0.0, 10.0	0.0, 10.0
(Missing)	68 (1.0%)	15 (0.5%)
Close social relationships		
Mean	7.8	7.8
Standard Deviation	2.2	2.1
Min, Max	0.0, 10.0	0.0, 10.0
(Missing)	40 (0.6%)	14 (0.5%)
Financial & material security		
Mean	3.9	4.1
Standard Deviation	3.5	3.3
Min, Max	0.0, 10.0	0.0, 10.0
(Missing)	22 (0.3%)	6 (0.2%)
Happiness		
Mean	7.4	7.1
Standard Deviation	2.1	2.0
Min, Max	0.0, 10.0	0.0, 10.0
(Missing)	16 (0.2%)	2 (<0.1%)
Life satisfaction		
Mean	7.2	7.1
Standard Deviation	2.4	2.3
Min, Max	0.0, 10.0	0.0, 10.0
(Missing)	15 (0.2%)	4 (0.1%)
Current life evaluation		

Table S9b. Weighted summary statistics for outcome variables in Argentina

Outcome	Wave 1<br>N = 6,724	Wave 2<br>N = 2,889
Mean	6.7	6.6
Standard Deviation	2.2	2.1
Min, Max	0.0, 10.0	0.0, 10.0
(Missing)	8 (0.1%)	1 (<0.1%)
Future life evaluation		
Mean	8.0	8.0
Standard Deviation	2.1	2.0
Min, Max	0.0, 10.0	0.0, 10.0
(Missing)	141 (2.1%)	44 (1.5%)
Optimism		
Mean	8.9	8.8
Standard Deviation	1.8	1.8
Min, Max	0.0, 10.0	0.0, 10.0
(Missing)	20 (0.3%)	5 (0.2%)
Freedom to pursue what's important		
Mean	8.3	8.3
Standard Deviation	2.3	2.1
Min, Max	0.0, 10.0	0.0, 10.0
(Missing)	21 (0.3%)	3 (0.1%)
Inner peace, n (%)		
Always	1,723 (25.6%)	777 (26.9%)
Often	2,809 (41.8%)	1,455 (50.4%)
Rarely	1,752 (26.1%)	538 (18.6%)
Never	427 (6.3%)	112 (3.9%)
(Missing)	14 (0.2%)	7 (0.3%)
Life balance, n (%)		
Always	1,399 (20.8%)	525 (18.2%)
Often	3,428 (51.0%)	1,610 (55.7%)
Rarely	1,619 (24.1%)	660 (22.9%)
Never	256 (3.8%)	88 (3.1%)
(Missing)	23 (0.3%)	5 (0.2%)
Sense of mastery, n (%)		
Always	2,778 (41.3%)	1,074 (37.2%)
Often	3,086 (45.9%)	1,559 (54.0%)
Rarely	692 (10.3%)	201 (7.0%)
Never	122 (1.8%)	45 (1.6%)
(Missing)	46 (0.7%)	10 (0.4%)
Meaningful activities		
Mean	7.9	7.9
Standard Deviation	2.2	2.1
Min, Max	0.0, 10.0	0.0, 10.0
(Missing)	14 (0.2%)	5 (0.2%)
Understanding purpose		
Mean	7.9	7.7
Standard Deviation	2.3	2.4
Min, Max	0.0, 10.0	0.0, 10.0
(Missing)	32 (0.5%)	7 (0.3%)
Self-rated mental health		
Mean	7.9	7.7
Standard Deviation	2.2	2.1
Min, Max	0.0, 10.0	0.0, 10.0

Table S9b. Weighted summary statistics for outcome variables in Argentina

**Outcome**	**Wave 1**<br>N = 6,724	**Wave 2**<br>N = 2,889
(Missing)	19 (0.3%)	2 (<0.1%)
Traumatic distress, n (%)		
A lot	1,125 (16.7%)	434 (15.0%)
Some	1,444 (21.5%)	660 (22.9%)
Not very much	1,645 (24.5%)	688 (23.8%)
Not at all	2,490 (37.0%)	1,103 (38.2%)
(Missing)	20 (0.3%)	4 (0.1%)
Depression symptoms composite, n (%)	2,426 (36.4%)	998 (35.0%)
(Missing)	54 (0.8%)	35 (1.2%)
Depression – feel hopeless, n (%)		
Nearly every day	931 (13.8%)	350 (12.1%)
More than half the days	934 (13.9%)	386 (13.4%)
Several days	2,076 (30.9%)	878 (30.4%)
Not at all	2,767 (41.2%)	1,256 (43.5%)
(Missing)	15 (0.2%)	18 (0.6%)
Depression – loss of interest, n (%)		
Nearly every day	998 (14.8%)	400 (13.8%)
More than half the days	1,179 (17.5%)	476 (16.5%)
Several days	2,291 (34.1%)	1,076 (37.2%)
Not at all	2,210 (32.9%)	914 (31.6%)
(Missing)	45 (0.7%)	24 (0.8%)
Anxiety symptoms composite, n (%)	2,745 (41.1%)	1,066 (37.1%)
(Missing)	54 (0.8%)	16 (0.6%)
Anxiety – feel on edge, n (%)		
Nearly every day	1,354 (20.1%)	469 (16.2%)
More than half the days	1,023 (15.2%)	449 (15.5%)
Several days	2,368 (35.2%)	946 (32.8%)
Not at all	1,951 (29.0%)	1,013 (35.1%)
(Missing)	28 (0.4%)	12 (0.4%)
Anxiety – cannot stop worrying, n (%)		
Nearly every day	1,242 (18.5%)	487 (16.9%)
More than half the days	958 (14.2%)	405 (14.0%)
Several days	1,885 (28.0%)	827 (28.6%)
Not at all	2,607 (38.8%)	1,161 (40.2%)
(Missing)	33 (0.5%)	9 (0.3%)
Suffering, n (%)		
A lot	920 (13.7%)	378 (13.1%)
Some	2,051 (30.5%)	967 (33.5%)
Not very much	2,049 (30.5%)	798 (27.6%)
Not at all	1,672 (24.9%)	738 (25.6%)
(Missing)	32 (0.5%)	7 (0.2%)
Relationship contentment		
Mean	8.0	8.1
Standard Deviation	2.4	2.2
Min, Max	0.0, 10.0	0.0, 10.0
(Missing)	15 (0.2%)	10 (0.4%)
Relationship satisfaction		
Mean	7.6	7.5
Standard Deviation	2.5	2.4
Min, Max	0.0, 10.0	0.0, 10.0
(Missing)	31 (0.5%)	7 (0.2%)

Table S9b. Weighted summary statistics for outcome variables in Argentina

Outcome	Wave 1<br>N = 6,724	Wave 2<br>N = 2,889
Social support		
Mean	8.2	8.1
Standard Deviation	2.5	2.5
Min, Max	0.0, 10.0	0.0, 10.0
(Missing)	15 (0.2%)	8 (0.3%)
Intimate/close friend, n (%)		
Yes	5,432 (80.8%)	2,321 (80.3%)
No	1,258 (18.7%)	547 (18.9%)
(Missing)	34 (0.5%)	21 (0.7%)
Government approval, n (%)		
Strongly approve	386 (5.7%)	378 (13.1%)
Somewhat approve	1,296 (19.3%)	814 (28.2%)
Neither approve nor disapprove	1,456 (21.7%)	682 (23.6%)
Somewhat disapprove	1,185 (17.6%)	325 (11.3%)
Strongly disapprove	2,326 (34.6%)	658 (22.8%)
(Missing)	75 (1.1%)	33 (1.1%)
Say in government, n (%)		
Agree	2,494 (37.1%)	1,691 (58.5%)
Disagree	2,197 (32.7%)	367 (12.7%)
Unsure	1,974 (29.4%)	807 (27.9%)
(Missing)	59 (0.9%)	23 (0.8%)
Belonging in country		
Mean	8.1	8.3
Standard Deviation	2.5	2.4
Min, Max	0.0, 10.0	0.0, 10.0
(Missing)	76 (1.1%)	38 (1.3%)
City/place satisfaction, n (%)		
Satisfied	4,751 (70.7%)	2,027 (70.2%)
Dissatisfied	1,201 (17.9%)	491 (17.0%)
Unsure	726 (10.8%)	347 (12.0%)
(Missing)	46 (0.7%)	24 (0.8%)
Trust within country, n (%)		
All people	136 (2.0%)	29 (1.0%)
Most people	765 (11.4%)	390 (13.5%)
Some people	2,787 (41.5%)	1,319 (45.7%)
Not very many people	2,406 (35.8%)	932 (32.3%)
None	561 (8.3%)	199 (6.9%)
(Missing)	70 (1.0%)	19 (0.7%)
Number of children		
Mean	1.1	0.9
Standard Deviation	1.5	1.3
Min, Max	0.0, 52.0	0.0, 8.0
(Missing)	97 (1.4%)	305 (11%)
Community participation, n (%)		
More than once a week	745 (11.1%)	276 (9.6%)
Once a week	556 (8.3%)	250 (8.6%)
One to three times a month	488 (7.3%)	178 (6.2%)
A few times a year	1,577 (23.4%)	714 (24.7%)
Never	3,327 (49.5%)	1,458 (50.5%)
(Missing)	32 (0.5%)	13 (0.4%)
Religious attendance, n (%)		

Table S9b. Weighted summary statistics for outcome variables in Argentina

**Outcome**	**Wave 1**<br>N = 6,724	**Wave 2**<br>N = 2,889
More than once a week	519 (7.7%)	241 (8.3%)
Once a week	771 (11.5%)	259 (9.0%)
One to three times a month	464 (6.9%)	174 (6.0%)
A few times a year	1,927 (28.7%)	861 (29.8%)
Never	3,014 (44.8%)	1,347 (46.6%)
(Missing)	28 (0.4%)	7 (0.2%)
Loneliness		
Mean	3.6	3.5
Standard Deviation	3.3	3.2
Min, Max	0.0, 10.0	0.0, 10.0
(Missing)	3 (<0.1%)	3 (0.1%)
Perceived discrimination, n (%)		
Always	537 (8.0%)	198 (6.9%)
Often	1,080 (16.1%)	454 (15.7%)
Rarely	2,229 (33.2%)	1,067 (36.9%)
Never	2,853 (42.4%)	1,165 (40.3%)
(Missing)	24 (0.4%)	5 (0.2%)
Orientation to promote good		
Mean	8.6	8.6
Standard Deviation	1.6	1.6
Min, Max	0.0, 10.0	0.0, 10.0
(Missing)	19 (0.3%)	1 (<0.1%)
Delayed gratification		
Mean	8.1	7.9
Standard Deviation	2.1	2.2
Min, Max	0.0, 10.0	0.0, 10.0
(Missing)	60 (0.9%)	14 (0.5%)
Hope		
Mean	8.9	8.8
Standard Deviation	1.7	1.8
Min, Max	0.0, 10.0	0.0, 10.0
(Missing)	16 (0.2%)	2 (<0.1%)
Gratitude		
Mean	8.5	8.3
Standard Deviation	2.0	2.1
Min, Max	0.0, 10.0	0.0, 10.0
(Missing)	15 (0.2%)	10 (0.4%)
Showing love/care		
Mean	8.5	8.4
Standard Deviation	2.1	2.1
Min, Max	0.0, 10.0	0.0, 10.0
(Missing)	14 (0.2%)	4 (0.1%)
Forgivingness, n (%)		
Always	2,446 (36.4%)	970 (33.6%)
Often	2,486 (37.0%)	1,221 (42.3%)
Rarely	1,336 (19.9%)	540 (18.7%)
Never	426 (6.3%)	154 (5.3%)
(Missing)	29 (0.4%)	5 (0.2%)
Charitable giving, n (%)		
Yes	1,354 (20.1%)	843 (29.2%)
No	5,357 (79.7%)	2,001 (69.3%)

Table S9b. Weighted summary statistics for outcome variables in Argentina

Outcome	Wave 1<br>N = 6,724	Wave 2<br>N = 2,889
(Missing)	13 (0.2%)	45 (1.5%)
Helping strangers, n (%)		
Yes	4,508 (67.0%)	1,895 (65.6%)
No	2,178 (32.4%)	965 (33.4%)
(Missing)	38 (0.6%)	28 (1.0%)
Volunteering, n (%)		
Yes	1,409 (21.0%)	526 (18.2%)
No	5,307 (78.9%)	2,332 (80.7%)
(Missing)	8 (0.1%)	30 (1.1%)
Self-rated physical health		
Mean	7.3	7.0
Standard Deviation	2.2	2.0
Min, Max	0.0, 10.0	0.0, 10.0
(Missing)	5 (<0.1%)	10 (0.3%)
Health problems, n (%)		
Yes	1,215 (18.1%)	525 (18.2%)
No	5,407 (80.4%)	2,324 (80.4%)
(Missing)	102 (1.5%)	40 (1.4%)
Pain in past 4 weeks, n (%)		
A lot	1,018 (15.1%)	407 (14.1%)
Some	1,927 (28.7%)	844 (29.2%)
Not very much	2,140 (31.8%)	991 (34.3%)
None at all	1,625 (24.2%)	637 (22.0%)
(Missing)	14 (0.2%)	11 (0.4%)
Number of cigarettes per day		
Mean	3.9	3.3
Standard Deviation	8.1	7.2
Min, Max	0.0, 96.0	0.0, 60.0
(Missing)	104 (1.6%)	51 (1.8%)
Number of drinks per week		
Mean	1.5	1.8
Standard Deviation	3.6	5.1
Min, Max	0.0, 97.0	0.0, 97.0
(Missing)	97 (1.4%)	42 (1.5%)
Days exercise per week		
Mean	2.0	2.1
Standard Deviation	2.3	2.3
Min, Max	0.0, 7.0	0.0, 7.0
(Missing)	33 (0.5%)	10 (0.4%)
Financial security		
Mean	3.9	4.1
Standard Deviation	3.7	3.5
Min, Max	0.0, 10.0	0.0, 10.0
(Missing)	15 (0.2%)	5 (0.2%)
Material security		
Mean	3.8	4.1
Standard Deviation	3.7	3.6
Min, Max	0.0, 10.0	0.0, 10.0
(Missing)	8 (0.1%)	2 (<0.1%)
Educational attainment (16+ years), n (%)		
Up to 8	2,258 (33.6%)	706 (24.5%)

Table S9b. Weighted summary statistics for outcome variables in Argentina

Outcome	Wave 1<br>N = 6,724	Wave 2<br>N = 2,889
9-15	3,856 (57.4%)	1,894 (65.6%)
16+	607 (9.0%)	289 (10.0%)
(Missing)	3 (0.0%)	0 (0%)
Currently employed, n (%)		
Employed for an employer	2,499 (37.2%)	929 (32.1%)
Self-employed	1,751 (26.0%)	889 (30.8%)
Retired	734 (10.9%)	362 (12.5%)
Student	358 (5.3%)	143 (5.0%)
Homemaker	618 (9.2%)	261 (9.0%)
Unemployed and looking for a job	561 (8.3%)	187 (6.5%)
None of these/Other	180 (2.7%)	63 (2.2%)
(Missing)	22 (0.3%)	55 (1.9%)
Financially comfortable/getting by, n (%)		
Living comfortably on present income	810 (12.1%)	396 (13.7%)
Getting by on present income	2,343 (34.8%)	1,104 (38.2%)
Finding it difficult on present income	2,257 (33.6%)	972 (33.6%)
Finding it very difficult on present income	1,148 (17.1%)	373 (12.9%)
(Missing)	165 (2.5%)	44 (1.5%)
Own home, n (%)		
Someone in this household owns this home	3,564 (53.0%)	1,630 (56.4%)
Someone in this household rents this home	1,131 (16.8%)	578 (20.0%)
Both	324 (4.8%)	177 (6.1%)
Neither	1,544 (23.0%)	465 (16.1%)
Rent	0 (0%)	0 (0%)
Own	0 (0%)	0 (0%)
Something else	0 (0%)	0 (0%)
(Missing)	161 (2.4%)	39 (1.4%)
Religious/spiritual connection, n (%)		
Always	2,190 (32.6%)	950 (32.9%)
Often	1,763 (26.2%)	825 (28.6%)
Rarely	1,746 (26.0%)	683 (23.6%)
Never	1,010 (15.0%)	424 (14.7%)
(Missing)	15 (0.2%)	6 (0.2%)
Belief in life after death, n (%)		
Yes	3,843 (57.2%)	1,714 (59.3%)
No	1,035 (15.4%)	508 (17.6%)
Unsure	1,787 (26.6%)	657 (22.7%)
(Missing)	59 (0.9%)	10 (0.4%)
Transformative religious experience, n (%)		
Yes	2,659 (39.5%)	1,142 (39.5%)
No	4,035 (60.0%)	1,732 (59.9%)
(Missing)	30 (0.4%)	15 (0.5%)
Religious reading or listening, n (%)		
More than once a day	628 (9.3%)	284 (9.8%)
About once a day	710 (10.6%)	265 (9.2%)
Sometimes	2,932 (43.6%)	1,194 (41.3%)
Never	2,408 (35.8%)	1,117 (38.7%)
(Missing)	47 (0.7%)	28 (1.0%)
Prayer or meditation, n (%)		
More than once a day	1,074 (16.0%)	467 (16.2%)
About once a day	1,397 (20.8%)	544 (18.8%)

Table S9b. Weighted summary statistics for outcome variables in Argentina

Outcome	Wave 1<br>N = 6,724	Wave 2<br>N = 2,889
Sometimes	2,666 (39.6%)	1,199 (41.5%)
Never	1,559 (23.2%)	669 (23.2%)
(Missing)	28 (0.4%)	10 (0.3%)
Belief in God/gods/spiritual forces, n (%)		
One God	4,742 (70.5%)	1,997 (69.1%)
More than one god	160 (2.4%)	79 (2.7%)
An impersonal spiritual force	740 (11.0%)	371 (12.9%)
None of these	533 (7.9%)	237 (8.2%)
Unsure	525 (7.8%)	191 (6.6%)
(Missing)	23 (0.3%)	13 (0.4%)
Religious centrality, n (%)		
Agree	3,048 (45.3%)	1,451 (50.2%)
Disagree	745 (11.1%)	297 (10.3%)
Not relevant	1,580 (23.5%)	685 (23.7%)
Unsure	1,285 (19.1%)	442 (15.3%)
(Missing)	66 (1.0%)	14 (0.5%)
Religious/spiritual comfort, n (%)		
Agree	3,852 (57.3%)	1,578 (54.6%)
Disagree	692 (10.3%)	301 (10.4%)
Not relevant	1,253 (18.6%)	641 (22.2%)
Unsure	885 (13.2%)	359 (12.4%)
(Missing)	43 (0.6%)	10 (0.3%)
Feel loved by God, n (%)		
Agree	4,503 (67.0%)	1,936 (67.0%)
Disagree	483 (7.2%)	213 (7.4%)
Not relevant	983 (14.6%)	443 (15.3%)
Unsure	716 (10.6%)	279 (9.7%)
(Missing)	39 (0.6%)	17 (0.6%)
Feel punished by God, n (%)		
Agree	922 (13.7%)	345 (11.9%)
Disagree	3,398 (50.5%)	1,407 (48.7%)
Not relevant	1,167 (17.4%)	600 (20.8%)
Unsure	1,187 (17.6%)	519 (18.0%)
(Missing)	50 (0.7%)	18 (0.6%)
Experienced religious criticism, n (%)		
Agree	976 (14.5%)	398 (13.8%)
Disagree	2,145 (31.9%)	887 (30.7%)
Not relevant	1,884 (28.0%)	917 (31.7%)
Unsure	1,641 (24.4%)	671 (23.2%)
(Missing)	78 (1.2%)	16 (0.6%)
Faith-sharing, n (%)		
Agree	3,316 (49.3%)	1,522 (52.7%)
Disagree	1,183 (17.6%)	406 (14.1%)
Not relevant	1,517 (22.6%)	679 (23.5%)
Unsure	664 (9.9%)	270 (9.3%)
(Missing)	44 (0.6%)	12 (0.4%)

Note. N (%); this table is based on non-imputed data. Cumulative percentages for variables may not add up to 100% due to rounding. Wave 1 characteristics weighted using the Gallup provided sampling weight, ANNUAL_WEIGHT_R2; Wave 2 characteristics weighted accounting for attrition by using the adjusted Wave 1 weight, ANNUAL_WEIGHT_R2, multiplied by the created attrition weight to account for dropout, to maintain nationally representative estimates for Wave 2 characteristics.

Table S9c. Unweighted summary statistics for demographic and childhood variables in Argentina by retention status

**Characteristic**	**Attritors–Not Observed in Wave 2**<br>N = 3,996	**Retained–Observed in Wave 2**<br>N = 2,709
Forgivingness, n (%)		
Always	1,527 (38.2%)	911 (33.6%)
Often	1,416 (35.4%)	1,064 (39.3%)
Rarely	772 (19.3%)	561 (20.7%)
Never	262 (6.6%)	162 (6.0%)
(Missing)	18 (0.5%)	11 (0.4%)
Year of birth, n (%)		
1943 or earlier (current age: 80+ years)	48 (1.2%)	63 (2.3%)
1943-1953 (current age: 70-79 years)	141 (3.5%)	197 (7.3%)
1953-1963 (current age: 60-69 years)	350 (8.8%)	353 (13.0%)
1963-1973 (current age: 50-59 years)	429 (10.7%)	554 (20.4%)
1973-1983 (current age: 40-49 years)	671 (16.8%)	547 (20.2%)
1983-1993 (current age: 30-39 years)	924 (23.1%)	504 (18.6%)
1993-1998 (current age: 25-29 years)	517 (12.9%)	203 (7.5%)
1998-2005 (current age: 18-24 years)	915 (22.9%)	289 (10.7%)
(Missing)	0 (0%)	0 (0%)
Age of participant		
Mean	38.7	46.5
Standard Deviation	16.1	16.5
Min, Max	18.0, 99.0	18.0, 95.0
Gender, n (%)		
Male	1,827 (45.7%)	1,324 (48.9%)
Female	2,143 (53.6%)	1,371 (50.6%)
Other	14 (0.3%)	6 (0.2%)
(Missing)	12 (0.3%)	7 (0.3%)
Respondent marital status, n (%)		
Single/Never been married	1,544 (38.6%)	893 (33.0%)
Married	829 (20.7%)	692 (25.5%)
Separated	246 (6.2%)	198 (7.3%)
Divorced	151 (3.8%)	155 (5.7%)
Widowed	172 (4.3%)	223 (8.2%)
Domestic partner	983 (24.6%)	533 (19.7%)
(Missing)	71 (1.8%)	16 (0.6%)
Education (years), n (%)		
Up to 8	1,405 (35.2%)	846 (31.2%)
9-15	2,305 (57.7%)	1,540 (56.8%)
16+	285 (7.1%)	322 (11.9%)
(Missing)	1 (0.0%)	1 (0.0%)
Employment status, n (%)		
Employed for an employer	1,517 (38.0%)	973 (35.9%)
Self-employed	934 (23.4%)	815 (30.1%)
Retired	345 (8.6%)	389 (14.4%)
Student	257 (6.4%)	99 (3.7%)
Homemaker	415 (10.4%)	200 (7.4%)
Unemployed and looking for a job	389 (9.7%)	169 (6.3%)
None of these/Other	120 (3.0%)	60 (2.2%)
(Missing)	18 (0.4%)	4 (0.1%)
Current religious service attendance, n (%)		
More than once a week	338 (8.4%)	180 (6.6%)

Table S9c. Unweighted summary statistics for demographic and childhood variables in Argentina by retention status

**Characteristic**	**Attritors–Not Observed in Wave 2**<br>N = 3,996	**Retained–Observed in Wave 2**<br>N = 2,709
Once a week	476 (11.9%)	293 (10.8%)
One to three times a month	288 (7.2%)	174 (6.4%)
A few times a year	1,113 (27.9%)	809 (29.9%)
Never	1,758 (44.0%)	1,249 (46.1%)
(Missing)	23 (0.6%)	5 (0.2%)
Immigration status, n (%)		
Born in this country	3,751 (93.9%)	2,570 (94.9%)
Born in another country	219 (5.5%)	136 (5.0%)
(Missing)	26 (0.6%)	3 (0.1%)
Parental marital status around age 12, n (%)		
Parents were married	2,234 (55.9%)	1,829 (67.5%)
Parents were divorced	384 (9.6%)	249 (9.2%)
Parents were never married	953 (23.9%)	434 (16.0%)
One or both of them had died	93 (2.3%)	106 (3.9%)
Unsure	181 (4.5%)	56 (2.0%)
(Missing)	150 (3.8%)	34 (1.3%)
Religious service attendance around age 12, n (%)		
At least once a week	1,561 (39.1%)	1,025 (37.8%)
One to three times a month	722 (18.1%)	490 (18.1%)
Less than once a month	611 (15.3%)	443 (16.3%)
Never	1,068 (26.7%)	733 (27.0%)
(Missing)	34 (0.9%)	19 (0.7%)
Relationship with mother when growing up, n (%)		
Very good	2,662 (66.6%)	1,764 (65.1%)
Somewhat good	813 (20.3%)	630 (23.3%)
Somewhat bad	187 (4.7%)	123 (4.5%)
Very bad	142 (3.5%)	75 (2.8%)
(Does not apply)	168 (4.2%)	104 (3.8%)
(Missing)	24 (0.6%)	13 (0.5%)
Relationship with father when growing up, n (%)		
Very good	2,027 (50.7%)	1,522 (56.2%)
Somewhat good	935 (23.4%)	610 (22.5%)
Somewhat bad	276 (6.9%)	174 (6.4%)
Very bad	272 (6.8%)	143 (5.3%)
(Does not apply)	457 (11.4%)	251 (9.3%)
(Missing)	28 (0.7%)	8 (0.3%)
Felt like an outsider in family when growing up, n (%)		
Yes	770 (19.3%)	442 (16.3%)
No	3,151 (78.9%)	2,234 (82.5%)
(Missing)	75 (1.9%)	33 (1.2%)
Experienced abuse when growing up, n (%)		
Yes	795 (19.9%)	513 (18.9%)
No	3,076 (77.0%)	2,170 (80.1%)
(Missing)	125 (3.1%)	26 (1.0%)
Self-rated health when growing up, n (%)		
Excellent	1,460 (36.5%)	933 (34.5%)
Very good	1,043 (26.1%)	766 (28.3%)

Table S9c. Unweighted summary statistics for demographic and childhood variables in Argentina by retention status

**Characteristic**	**Attritors–Not Observed in Wave 2** N = 3,996	**Retained–Observed in Wave 2** N = 2,709
Good	1,093 (27.4%)	728 (26.9%)
Fair	287 (7.2%)	228 (8.4%)
Poor	101 (2.5%)	51 (1.9%)
(Missing)	11 (0.3%)	3 (0.1%)
Subjective financial status of family growing up, n (%)		
Lived comfortably	1,240 (31.0%)	790 (29.2%)
Got by	1,374 (34.4%)	924 (34.1%)
Found it difficult	1,045 (26.2%)	736 (27.2%)
Found it very difficult	323 (8.1%)	252 (9.3%)
(Missing)	13 (0.3%)	7 (0.3%)
Religious affiliation growing up, n (%)		
Christianity	3,369 (84.3%)	2,405 (88.8%)
Taoism	1 (0.0%)	1 (0.0%)
Confucianism	0 (0%)	0 (0%)
Primal, Animist, or Folk religion	13 (0.3%)	3 (0.1%)
Spiritism	0 (0%)	0 (0%)
Umbanda, Candomblé, and other African-derived religions	0 (0%)	0 (0%)
Chinese folk/traditional religion	0 (0%)	0 (0%)
Islam	10 (0.3%)	0 (0.0%)
Hinduism	3 (0.1%)	0 (0%)
Buddhism	3 (0.1%)	1 (0.0%)
Judaism	29 (0.7%)	20 (0.7%)
Sikhism	4 (0.1%)	0 (0%)
Baha'i	0 (0%)	0 (0%)
Jainism	0 (0%)	0 (0%)
Shinto	0 (0%)	0 (0%)
Some other religion	5 (0.1%)	6 (0.2%)
No religion/Atheist/Agnostic	470 (11.8%)	232 (8.6%)
(Missing)	89 (2.2%)	40 (1.5%)

Note. N (%); this table is based on non-imputed data. Cumulative percentages for variables may not add up to 100% due to rounding.

Table S9d. Unweighted summary statistics for Wave 1 outcome variables in Argentina by retention status.

**Outcome**	**Attritors–Not Observed in Wave 2** N = 3,996	**Retained–Observed in Wave 2** N = 2,709
Secure flourishing index		
Mean	7.1	7.1
Standard Deviation	1.5	1.4
Min, Max	0.4, 10.0	1.4, 10.0
(Missing)	132 (3.3%)	74 (2.7%)
Flourishing index		
Mean	7.8	7.8
Standard Deviation	1.5	1.4
Min, Max	0.5, 10.0	1.7, 10.0
(Missing)	125 (3.1%)	65 (2.4%)
Happiness & life satisfaction		
Mean	7.3	7.2
Standard Deviation	2.2	2.0
Min, Max	0.0, 10.0	0.0, 10.0
(Missing)	22 (0.6%)	9 (0.3%)
Physical & mental health		
Mean	7.6	7.5
Standard Deviation	1.9	1.8
Min, Max	0.0, 10.0	0.0, 10.0
(Missing)	16 (0.4%)	7 (0.2%)
Meaning & purpose		
Mean	7.9	7.9
Standard Deviation	2.0	1.9
Min, Max	0.0, 10.0	0.0, 10.0
(Missing)	39 (1.0%)	6 (0.2%)
Character & virtue		
Mean	8.4	8.3
Standard Deviation	1.6	1.5
Min, Max	0.0, 10.0	0.0, 10.0
(Missing)	37 (0.9%)	31 (1.2%)
Close social relationships		
Mean	7.8	7.8
Standard Deviation	2.3	2.1
Min, Max	0.0, 10.0	0.0, 10.0
(Missing)	20 (0.5%)	20 (0.7%)
Financial & material security		
Mean	3.8	3.9
Standard Deviation	3.5	3.4
Min, Max	0.0, 10.0	0.0, 10.0
(Missing)	13 (0.3%)	10 (0.4%)
Happiness		
Mean	7.4	7.3
Standard Deviation	2.2	2.0
Min, Max	0.0, 10.0	0.0, 10.0
(Missing)	15 (0.4%)	2 (<0.1%)
Life satisfaction		
Mean	7.2	7.2
Standard Deviation	2.5	2.2
Min, Max	0.0, 10.0	0.0, 10.0

Table S9d. Unweighted summary statistics for Wave 1 outcome variables in Argentina by retention status.

**Outcome**	**Attritors–Not Observed in Wave 2**<br>N = 3,996	**Retained–Observed in Wave 2**<br>N = 2,709
(Missing)	8 (0.2%)	7 (0.3%)
Current life evaluation		
Mean	6.8	6.7
Standard Deviation	2.3	2.0
Min, Max	0.0, 10.0	0.0, 10.0
(Missing)	4 (0.1%)	4 (0.1%)
Future life evaluation		
Mean	8.2	7.8
Standard Deviation	2.2	2.1
Min, Max	0.0, 10.0	0.0, 10.0
(Missing)	79 (2.0%)	61 (2.3%)
Optimism		
Mean	8.9	8.8
Standard Deviation	1.8	1.8
Min, Max	0.0, 10.0	0.0, 10.0
(Missing)	13 (0.3%)	7 (0.2%)
Freedom to pursue what's important		
Mean	8.2	8.3
Standard Deviation	2.3	2.1
Min, Max	0.0, 10.0	0.0, 10.0
(Missing)	15 (0.4%)	6 (0.2%)
Inner peace, n (%)		
Always	985 (24.6%)	734 (27.1%)
Often	1,609 (40.3%)	1,193 (44.0%)
Rarely	1,103 (27.6%)	642 (23.7%)
Never	293 (7.3%)	131 (4.8%)
(Missing)	5 (0.1%)	9 (0.3%)
Life balance, n (%)		
Always	857 (21.5%)	537 (19.8%)
Often	1,954 (48.9%)	1,465 (54.1%)
Rarely	999 (25.0%)	615 (22.7%)
Never	173 (4.3%)	82 (3.0%)
(Missing)	13 (0.3%)	10 (0.4%)
Sense of mastery, n (%)		
Always	1,699 (42.5%)	1,071 (39.5%)
Often	1,699 (42.5%)	1,381 (51.0%)
Rarely	481 (12.0%)	208 (7.7%)
Never	92 (2.3%)	29 (1.1%)
(Missing)	25 (0.6%)	21 (0.8%)
Meaningful activities		
Mean	7.9	8.0
Standard Deviation	2.3	2.1
Min, Max	0.0, 10.0	0.0, 10.0
(Missing)	11 (0.3%)	3 (0.1%)
Understanding purpose		
Mean	7.9	7.9
Standard Deviation	2.4	2.2
Min, Max	0.0, 10.0	0.0, 10.0
(Missing)	28 (0.7%)	4 (0.1%)

Table S9d. Unweighted summary statistics for Wave 1 outcome variables in Argentina by retention status.

Outcome	Attritors–Not Observed in Wave 2<br>N = 3,996	Retained–Observed in Wave 2<br>N = 2,709
Self-rated mental health		
Mean	7.9	7.9
Standard Deviation	2.2	2.1
Min, Max	0.0, 10.0	0.0, 10.0
(Missing)	14 (0.3%)	5 (0.2%)
Traumatic distress, n (%)		
A lot	714 (17.9%)	407 (15.0%)
Some	919 (23.0%)	520 (19.2%)
Not very much	973 (24.3%)	667 (24.6%)
Not at all	1,374 (34.4%)	1,111 (41.0%)
(Missing)	16 (0.4%)	4 (0.1%)
Depression symptoms composite, n (%)	1,575 (39.7%)	841 (31.3%)
(Missing)	33 (0.8%)	21 (0.8%)
Depression – feel hopeless, n (%)		
Nearly every day	625 (15.7%)	301 (11.1%)
More than half the days	623 (15.6%)	307 (11.3%)
Several days	1,200 (30.0%)	871 (32.2%)
Not at all	1,538 (38.5%)	1,224 (45.2%)
(Missing)	9 (0.2%)	6 (0.2%)
Depression – loss of interest, n (%)		
Nearly every day	645 (16.1%)	349 (12.9%)
More than half the days	766 (19.2%)	408 (15.1%)
Several days	1,342 (33.6%)	943 (34.8%)
Not at all	1,213 (30.4%)	993 (36.6%)
(Missing)	29 (0.7%)	16 (0.6%)
Anxiety symptoms composite, n (%)	1,755 (44.3%)	979 (36.4%)
(Missing)	35 (0.9%)	19 (0.7%)
Anxiety – feel on edge, n (%)		
Nearly every day	919 (23.0%)	428 (15.8%)
More than half the days	622 (15.6%)	398 (14.7%)
Several days	1,385 (34.7%)	976 (36.0%)
Not at all	1,053 (26.3%)	896 (33.1%)
(Missing)	17 (0.4%)	11 (0.4%)
Anxiety – cannot stop worrying, n (%)		
Nearly every day	807 (20.2%)	429 (15.9%)
More than half the days	612 (15.3%)	342 (12.6%)
Several days	1,084 (27.1%)	796 (29.4%)
Not at all	1,470 (36.8%)	1,131 (41.8%)
(Missing)	23 (0.6%)	10 (0.4%)
Suffering, n (%)		
A lot	575 (14.4%)	341 (12.6%)
Some	1,202 (30.1%)	844 (31.2%)
Not very much	1,172 (29.3%)	872 (32.2%)
Not at all	1,025 (25.6%)	642 (23.7%)
(Missing)	22 (0.6%)	9 (0.3%)
Relationship contentment		
Mean	8.0	8.1
Standard Deviation	2.5	2.2
Min, Max	0.0, 10.0	0.0, 10.0

Table S9d. Unweighted summary statistics for Wave 1 outcome variables in Argentina by retention status.

Outcome	Attritors–Not Observed in Wave 2<br>N = 3,996	Retained–Observed in Wave 2<br>N = 2,709
(Missing)	11 (0.3%)	4 (0.1%)
Relationship satisfaction		
Mean	7.6	7.6
Standard Deviation	2.5	2.3
Min, Max	0.0, 10.0	0.0, 10.0
(Missing)	14 (0.4%)	16 (0.6%)
Social support		
Mean	8.2	8.2
Standard Deviation	2.6	2.4
Min, Max	0.0, 10.0	0.0, 10.0
(Missing)	7 (0.2%)	8 (0.3%)
Intimate/close friend, n (%)		
Yes	3,217 (80.5%)	2,200 (81.2%)
No	752 (18.8%)	502 (18.5%)
(Missing)	26 (0.7%)	7 (0.3%)
Government approval, n (%)		
Strongly approve	288 (7.2%)	95 (3.5%)
Somewhat approve	756 (18.9%)	537 (19.8%)
Neither approve nor disapprove	972 (24.3%)	477 (17.6%)
Somewhat disapprove	671 (16.8%)	512 (18.9%)
Strongly disapprove	1,267 (31.7%)	1,055 (38.9%)
(Missing)	43 (1.1%)	33 (1.2%)
Say in government, n (%)		
Agree	1,311 (32.8%)	1,179 (43.5%)
Disagree	1,394 (34.9%)	795 (29.4%)
Unsure	1,253 (31.4%)	713 (26.3%)
(Missing)	38 (0.9%)	21 (0.8%)
Belonging in country		
Mean	8.0	8.2
Standard Deviation	2.6	2.4
Min, Max	0.0, 10.0	0.0, 10.0
(Missing)	58 (1.5%)	17 (0.6%)
City/place satisfaction, n (%)		
Satisfied	2,763 (69.2%)	1,975 (72.9%)
Dissatisfied	730 (18.3%)	467 (17.2%)
Unsure	469 (11.7%)	254 (9.4%)
(Missing)	33 (0.8%)	12 (0.5%)
Trust within country, n (%)		
All people	107 (2.7%)	28 (1.0%)
Most people	444 (11.1%)	318 (11.8%)
Some people	1,621 (40.6%)	1,159 (42.8%)
Not very many people	1,439 (36.0%)	960 (35.5%)
None	341 (8.5%)	217 (8.0%)
(Missing)	44 (1.1%)	26 (1.0%)
Number of children		
Mean	1.2	0.9
Standard Deviation	1.6	1.3
Min, Max	0.0, 52.0	0.0, 9.0
(Missing)	74 (1.9%)	22 (0.8%)

Table S9d. Unweighted summary statistics for Wave 1 outcome variables in Argentina by retention status.

**Outcome**	**Attritors–Not Observed in Wave 2**<br>N = 3,996	**Retained–Observed in Wave 2**<br>N = 2,709
Community participation, n (%)		
More than once a week	462 (11.6%)	281 (10.4%)
Once a week	319 (8.0%)	236 (8.7%)
One to three times a month	325 (8.1%)	160 (5.9%)
A few times a year	916 (22.9%)	656 (24.2%)
Never	1,944 (48.7%)	1,374 (50.7%)
(Missing)	29 (0.7%)	3 (0.1%)
Religious attendance, n (%)		
More than once a week	338 (8.4%)	180 (6.6%)
Once a week	476 (11.9%)	293 (10.8%)
One to three times a month	288 (7.2%)	174 (6.4%)
A few times a year	1,113 (27.9%)	809 (29.9%)
Never	1,758 (44.0%)	1,249 (46.1%)
(Missing)	23 (0.6%)	5 (0.2%)
Loneliness		
Mean	3.7	3.4
Standard Deviation	3.4	3.2
Min, Max	0.0, 10.0	0.0, 10.0
(Missing)	3 (<0.1%)	0 (0%)
Perceived discrimination, n (%)		
Always	381 (9.5%)	153 (5.7%)
Often	679 (17.0%)	397 (14.7%)
Rarely	1,270 (31.8%)	954 (35.2%)
Never	1,653 (41.4%)	1,193 (44.0%)
(Missing)	12 (0.3%)	12 (0.4%)
Orientation to promote good		
Mean	8.6	8.6
Standard Deviation	1.7	1.5
Min, Max	0.0, 10.0	0.0, 10.0
(Missing)	15 (0.4%)	4 (0.2%)
Delayed gratification		
Mean	8.2	8.0
Standard Deviation	2.1	2.1
Min, Max	0.0, 10.0	0.0, 10.0
(Missing)	33 (0.8%)	27 (1.0%)
Hope		
Mean	9.0	8.9
Standard Deviation	1.7	1.6
Min, Max	0.0, 10.0	0.0, 10.0
(Missing)	9 (0.2%)	8 (0.3%)
Gratitude		
Mean	8.5	8.5
Standard Deviation	2.0	2.0
Min, Max	0.0, 10.0	0.0, 10.0
(Missing)	7 (0.2%)	7 (0.3%)
Showing love/care		
Mean	8.5	8.4
Standard Deviation	2.2	2.1
Min, Max	0.0, 10.0	0.0, 10.0

Table S9d. Unweighted summary statistics for Wave 1 outcome variables in Argentina by retention status.

Outcome	Attritors–Not Observed in Wave 2<br>N = 3,996	Retained–Observed in Wave 2<br>N = 2,709
(Missing)	7 (0.2%)	7 (0.3%)
Forgivingness, n (%)		
Always	1,527 (38.2%)	911 (33.6%)
Often	1,416 (35.4%)	1,064 (39.3%)
Rarely	772 (19.3%)	561 (20.7%)
Never	262 (6.6%)	162 (6.0%)
(Missing)	18 (0.5%)	11 (0.4%)
Charitable giving, n (%)		
Yes	759 (19.0%)	592 (21.9%)
No	3,227 (80.8%)	2,114 (78.0%)
(Missing)	10 (0.2%)	3 (0.1%)
Helping strangers, n (%)		
Yes	2,707 (67.8%)	1,787 (66.0%)
No	1,259 (31.5%)	913 (33.7%)
(Missing)	29 (0.7%)	9 (0.3%)
Volunteering, n (%)		
Yes	834 (20.9%)	571 (21.1%)
No	3,158 (79.0%)	2,133 (78.8%)
(Missing)	4 (0.1%)	5 (0.2%)
Self-rated physical health		
Mean	7.4	7.1
Standard Deviation	2.2	2.1
Min, Max	0.0, 10.0	0.0, 10.0
(Missing)	3 (<0.1%)	2 (<0.1%)
Health problems, n (%)		
Yes	698 (17.5%)	514 (19.0%)
No	3,207 (80.3%)	2,185 (80.6%)
(Missing)	91 (2.3%)	10 (0.4%)
Pain in past 4 weeks, n (%)		
A lot	621 (15.5%)	394 (14.5%)
Some	1,157 (29.0%)	764 (28.2%)
Not very much	1,234 (30.9%)	900 (33.2%)
None at all	972 (24.3%)	648 (23.9%)
(Missing)	11 (0.3%)	3 (0.1%)
Number of cigarettes per day		
Mean	4.2	3.5
Standard Deviation	8.2	7.9
Min, Max	0.0, 96.0	0.0, 60.0
(Missing)	59 (1.5%)	45 (1.7%)
Number of drinks per week		
Mean	1.4	1.7
Standard Deviation	3.6	3.4
Min, Max	0.0, 97.0	0.0, 56.0
(Missing)	67 (1.7%)	30 (1.1%)
Days exercise per week		
Mean	2.0	1.9
Standard Deviation	2.4	2.3
Min, Max	0.0, 7.0	0.0, 7.0
(Missing)	18 (0.5%)	15 (0.5%)

Table S9d. Unweighted summary statistics for Wave 1 outcome variables in Argentina by retention status.

**Outcome**	**Attritors–Not Observed in Wave 2** N = 3,996	**Retained–Observed in Wave 2** N = 2,709
Financial security		
Mean	3.9	4.0
Standard Deviation	3.7	3.6
Min, Max	0.0, 10.0	0.0, 10.0
(Missing)	10 (0.3%)	5 (0.2%)
Material security		
Mean	3.7	3.9
Standard Deviation	3.8	3.7
Min, Max	0.0, 10.0	0.0, 10.0
(Missing)	3 (<0.1%)	5 (0.2%)
Educational attainment (16+ years), n (%)		
Up to 8	1,405 (35.2%)	846 (31.2%)
9-15	2,305 (57.7%)	1,540 (56.8%)
16+	285 (7.1%)	322 (11.9%)
(Missing)	1 (0.0%)	1 (0.0%)
Currently employed, n (%)		
Employed for an employer	1,517 (38.0%)	973 (35.9%)
Self-employed	934 (23.4%)	815 (30.1%)
Retired	345 (8.6%)	389 (14.4%)
Student	257 (6.4%)	99 (3.7%)
Homemaker	415 (10.4%)	200 (7.4%)
Unemployed and looking for a job	389 (9.7%)	169 (6.3%)
None of these/Other	120 (3.0%)	60 (2.2%)
(Missing)	18 (0.4%)	4 (0.1%)
Financially comfortable/getting by, n (%)		
Living comfortably on present income	463 (11.6%)	345 (12.7%)
Getting by on present income	1,301 (32.6%)	1,038 (38.3%)
Finding it difficult on present income	1,316 (32.9%)	935 (34.5%)
Finding it very difficult on present income	773 (19.3%)	370 (13.7%)
(Missing)	143 (3.6%)	21 (0.8%)
Own home, n (%)		
Someone in this household owns this home	1,996 (49.9%)	1,561 (57.6%)
Someone in this household rents this home	656 (16.4%)	473 (17.5%)
Both	208 (5.2%)	115 (4.2%)
Neither	990 (24.8%)	548 (20.2%)
Rent	0 (0%)	0 (0%)
Own	0 (0%)	0 (0%)
Something else	0 (0%)	0 (0%)
(Missing)	147 (3.7%)	12 (0.4%)
Religious/spiritual connection, n (%)		
Always	1,270 (31.8%)	915 (33.8%)
Often	1,039 (26.0%)	719 (26.5%)
Rarely	1,051 (26.3%)	690 (25.5%)
Never	625 (15.6%)	382 (14.1%)
(Missing)	11 (0.3%)	4 (0.1%)
Belief in life after death, n (%)		
Yes	2,275 (56.9%)	1,557 (57.5%)
No	551 (13.8%)	482 (17.8%)
Unsure	1,123 (28.1%)	657 (24.3%)

Table S9d. Unweighted summary statistics for Wave 1 outcome variables in Argentina by retention status.

Outcome	Attritors–Not Observed in Wave 2<br>N = 3,996	Retained–Observed in Wave 2<br>N = 2,709
(Missing)	47 (1.2%)	12 (0.4%)
Transformative religious experience, n (%)		
Yes	1,604 (40.1%)	1,047 (38.7%)
No	2,369 (59.3%)	1,655 (61.1%)
(Missing)	23 (0.6%)	7 (0.3%)
Religious reading or listening, n (%)		
More than once a day	409 (10.2%)	216 (8.0%)
About once a day	411 (10.3%)	297 (11.0%)
Sometimes	1,764 (44.2%)	1,158 (42.8%)
Never	1,382 (34.6%)	1,020 (37.6%)
(Missing)	29 (0.7%)	18 (0.7%)
Prayer or meditation, n (%)		
More than once a day	635 (15.9%)	437 (16.1%)
About once a day	768 (19.2%)	626 (23.1%)
Sometimes	1,646 (41.2%)	1,010 (37.3%)
Never	929 (23.2%)	626 (23.1%)
(Missing)	18 (0.4%)	10 (0.4%)
Belief in God/gods/spiritual forces, n (%)		
One God	2,841 (71.1%)	1,887 (69.7%)
More than one god	100 (2.5%)	59 (2.2%)
An impersonal spiritual force	411 (10.3%)	327 (12.1%)
None of these	300 (7.5%)	232 (8.6%)
Unsure	325 (8.1%)	198 (7.3%)
(Missing)	19 (0.5%)	5 (0.2%)
Religious centrality, n (%)		
Agree	1,803 (45.1%)	1,237 (45.7%)
Disagree	452 (11.3%)	291 (10.7%)
Not relevant	901 (22.6%)	676 (24.9%)
Unsure	786 (19.7%)	495 (18.3%)
(Missing)	54 (1.4%)	11 (0.4%)
Religious/spiritual comfort, n (%)		
Agree	2,288 (57.3%)	1,553 (57.3%)
Disagree	403 (10.1%)	287 (10.6%)
Not relevant	717 (17.9%)	533 (19.7%)
Unsure	555 (13.9%)	327 (12.1%)
(Missing)	33 (0.8%)	9 (0.3%)
Feel loved by God, n (%)		
Agree	2,667 (66.7%)	1,824 (67.3%)
Disagree	284 (7.1%)	198 (7.3%)
Not relevant	584 (14.6%)	396 (14.6%)
Unsure	436 (10.9%)	278 (10.3%)
(Missing)	25 (0.6%)	14 (0.5%)
Feel punished by God, n (%)		
Agree	628 (15.7%)	290 (10.7%)
Disagree	1,898 (47.5%)	1,493 (55.1%)
Not relevant	690 (17.3%)	474 (17.5%)
Unsure	743 (18.6%)	439 (16.2%)
(Missing)	36 (0.9%)	13 (0.5%)
Experienced religious criticism, n (%)		

Table S9d. Unweighted summary statistics for Wave 1 outcome variables in Argentina by retention status.

Outcome	Attritors–Not Observed in Wave 2<br>N = 3,996	Retained–Observed in Wave 2<br>N = 2,709
Agree	593 (14.8%)	380 (14.0%)
Disagree	1,259 (31.5%)	880 (32.5%)
Not relevant	1,095 (27.4%)	784 (28.9%)
Unsure	990 (24.8%)	645 (23.8%)
(Missing)	57 (1.4%)	21 (0.8%)
Faith-sharing, n (%)		
Agree	1,912 (47.9%)	1,396 (51.5%)
Disagree	722 (18.1%)	457 (16.9%)
Not relevant	900 (22.5%)	612 (22.6%)
Unsure	423 (10.6%)	238 (8.8%)
(Missing)	38 (0.9%)	6 (0.2%)

Note. N (%); this table is based on non-imputed data. Cumulative percentages for variables may not add up to 100% due to rounding.

Table S9e. Summary of fitted attrition model in Argentina

Characteristic	Odds Ratio	95% CI	p-value
**ANNUAL_WEIGHT_R2**	0.88	0.80, 0.96	0.005
**Recruitment Survey Mode**			
CATI	—	—	
CAWI	0.51	0.43, 0.60	1.55e-14
CAPI	0.74	0.60, 0.91	0.004
**Happiness & life satisfaction**	0.93	0.83, 1.05	0.253
**Physical & mental health**	0.95	0.86, 1.04	0.271
**Meaning & purpose**	1.03	0.92, 1.15	0.627
**Character & virtue**	0.96	0.89, 1.04	0.299
**Close social relationships**	0.94	0.86, 1.04	0.249
**Financial & material security**	0.97	0.90, 1.05	0.416
**Extraversion**	1.02	0.95, 1.10	0.540
**Openness to experience**	1.06	0.98, 1.15	0.125
**Agreeableness**	1.00	0.92, 1.08	0.981
**Conscientiousness**	1.02	0.94, 1.10	0.699
**Neuroticism**	1.08	0.99, 1.17	0.071
**Depression symptoms composite**	0.98	0.90, 1.08	0.718
**Anxiety symptoms composite**	0.98	0.89, 1.07	0.603
**Loneliness**	0.94	0.86, 1.02	0.154
**Days exercise per week**	0.95	0.88, 1.02	0.156
**Year of birth (age group)**			
1983-1993 (current age: 30-39 years)	—	—	
1973-1983 (current age: 40-49 years)	1.35	1.09, 1.67	0.005
1963-1973 (current age: 50-59 years)	2.12	1.66, 2.71	1.50e-09
1998-2005 (current age: 18-24 years)	0.70	0.52, 0.93	0.015
1993-1998 (current age: 25-29 years)	0.80	0.62, 1.05	0.103
1953-1963 (current age: 60-69 years)	1.60	1.15, 2.24	0.006
1943-1953 (current age: 70-79 years)	2.22	1.32, 3.73	0.003
1943 or earlier (current age: 80+ years)	2.22	1.06, 4.63	0.035
**Gender of respondent**			
Female	—	—	
Male	1.05	0.90, 1.23	0.538
Other	1.16	0.31, 4.26	0.827
**Marital status**			
Single/Never been married	—	—	
Married	0.85	0.68, 1.06	0.150
Domestic partner	0.88	0.72, 1.07	0.190
Separated	1.01	0.75, 1.36	0.945
Divorced	0.92	0.67, 1.26	0.607
Widowed	1.25	0.84, 1.84	0.268
**Employment status**			
Employed for an employer	—	—	
Self-employed	1.11	0.93, 1.34	0.255
Retired	0.90	0.62, 1.32	0.603
Homemaker	0.99	0.74, 1.34	0.963
Unemployed and looking for a job	0.93	0.69, 1.26	0.639
Student	0.93	0.67, 1.31	0.685
None of these/Other	0.86	0.56, 1.33	0.508
**Religious attendance**			
Never	—	—	
A few times a year	1.01	0.85, 1.19	0.931
Once a week	0.90	0.70, 1.16	0.421

Table S9e. Summary of fitted attrition model in Argentina

**Characteristic**	**Odds Ratio**	**95% CI**	**p-value**
More than once a week	0.88	0.65, 1.20	0.430
One to three times a month	0.86	0.64, 1.16	0.326
**Educational attainment (16+ years)**			
9-15	—	—	
16+	1.09	0.89, 1.33	0.389
Up to 8	1.10	0.85, 1.43	0.476
**Born in This country**			
Born in this country	—	—	
Born in another country	0.90	0.64, 1.24	0.511
**Race plurality (prominent race/ethnic group [0] or not [1])**	0.93	0.87, 1.00	0.062
**Urbanicity**			
A large city	—	—	
A small town or village	1.08	0.91, 1.27	0.384
A suburb of a large city	1.10	0.90, 1.35	0.336
A rural area or on a farm	0.67	0.47, 0.95	0.024
**Monthly household income**			
Argentina: More than 200,000 pesos	—	—	
Argentina: 150,001 – 200,000 pesos	0.68	0.54, 0.86	0.001
Argentina: 120,001 – 150,000 pesos	0.92	0.69, 1.21	0.547
(None/No household income)	0.38	0.26, 0.56	6.83e-07
Argentina: 100,001 – 120,000 pesos	0.73	0.53, 1.02	0.066
Argentina: 90,001 – 100,000 pesos	0.75	0.54, 1.05	0.090
Argentina: 50,001 – 60,000 pesos	0.63	0.43, 0.92	0.016
Argentina: 40,001 – 50,000 pesos	0.51	0.35, 0.74	3.24e-04
Argentina: 70,001 – 80,000 pesos	0.79	0.55, 1.15	0.221
Argentina: 80,001 – 90,000 pesos	0.74	0.51, 1.08	0.119
Argentina: 60,001 – 70,000 pesos	0.62	0.42, 0.93	0.020
Argentina: 30,001 – 40,000 pesos	0.38	0.24, 0.59	1.91e-05
Argentina: 20,001 – 30,000 pesos	0.81	0.49, 1.35	0.423
Argentina: 10,001 – 20,000 pesos	0.59	0.37, 0.96	0.034
Argentina: 5,001 – 10,000 pesos	1.05	0.52, 2.11	0.891
Argentina: 1,000 pesos or less	0.17	0.06, 0.48	7.63e-04
Argentina: 1,001 – 5,000 pesos	0.55	0.28, 1.07	0.078

Abbreviations: CI = Confidence Interval, OR = Odds Ratio

Notes. N=6724; attrition weights were estimated using the 'survey::svyglm(family=quasibinomial('logit'))' function. All continuous predictors were standardized and all categorical predictors used the most common category as the reference group. Reported p-values are based on the fitted regression model and no adjustments for multiple testing were done within this table.

Table S9f. Summary of principal components in Argentina

PC	Percent Explained by each PC	Cumulative Percent Explained
1	34.57	34.57
2	5.52	40.09
3	4.75	44.84
4	2.49	47.33
5	2.22	49.54
6	1.81	51.35
**7**	**1.75**	**53.10**
8	1.47	54.57
9	1.40	55.98
10	1.34	57.32
11	1.27	58.59
12	1.21	59.80
13	1.18	60.98
14	1.16	62.14
15	1.13	63.27
16	1.12	64.39
17	1.09	65.48
18	1.05	66.53
19	1.01	67.54
20	0.98	68.52

Notes. N=6724; PCA was conducted using ’survey::svyprcomp(.)’ function using all available contemporaneous exposures at wave 1. All PCs were standardized prior to being used as predictors. The bolded row represented the number of retained components for analysis was 7.

Table S9g. Associations of forgivingness with adult well-being and other outcomes at Wave 2 in Argentina

Outcome	Model 1: Demographic and Childhood Variables as Covariates					Model 2: Demographic, Childhood, and Other Wave 1 Confounding Variables (Via Principal Components) as Covariates				
	RR	ES	95% CI	SE	p-value	RR	ES	95% CI	SE	p-value
Human Flourishing										
Secure flourishing index		0.05	(0.01,0.09)	0.02	0.009*		-0.00	(-0.03,0.03)	0.02	0.901
Flourishing index		0.06	(0.03,0.10)	0.02	2.44e-04***		0.00	(-0.03,0.03)	0.01	0.909
Happiness & life satisfaction		0.03	(-0.01,0.07)	0.02	0.192		-0.01	(-0.05,0.02)	0.02	0.432
Physical & mental health		0.01	(-0.03,0.05)	0.02	0.746		-0.02	(-0.06,0.01)	0.02	0.192
Meaning & purpose		0.04	(-0.00,0.08)	0.02	0.053		-0.02	(-0.06,0.02)	0.02	0.416
Character & virtue		0.09	(0.05,0.13)	0.02	4.7e-05***		0.05	(0.00,0.09)	0.02	0.031*
Close social relationships		0.07	(0.03,0.11)	0.02	1.67e-03**		0.02	(-0.02,0.06)	0.02	0.300
Financial & material security		-0.01	(-0.05,0.03)	0.02	0.543		-0.01	(-0.05,0.03)	0.02	0.694
Psychological Well-Being										
Happiness (a)		0.01	(-0.03,0.05)	0.02	0.644		-0.02	(-0.07,0.02)	0.02	0.251
Life satisfaction (a)		0.03	(-0.01,0.08)	0.02	0.168		-0.00	(-0.05,0.04)	0.02	0.932
Current life evaluation		0.02	(-0.03,0.07)	0.03	0.385		0.00	(-0.05,0.05)	0.02	0.941
Future life evaluation		0.04	(0.00,0.08)	0.02	0.043*		0.02	(-0.02,0.06)	0.02	0.313
Optimism		0.04	(-0.01,0.08)	0.02	0.106		0.00	(-0.05,0.05)	0.02	0.999
Freedom to pursue what's important		0.00	(-0.04,0.05)	0.02	0.840		-0.04	(-0.08,-0.00)	0.02	0.042*
Inner peace	1.02		(1.00,1.04)	0.01	0.042*	1.00		(0.99,1.02)	0.01	0.649
Life balance	1.01		(0.99,1.03)	0.01	0.177	1.00		(0.98,1.02)	0.01	0.986
Sense of mastery	1.01		(0.99,1.02)	0.01	0.299	1.00		(0.99,1.01)	0.01	0.979
Meaningful activities (c)		0.04	(-0.01,0.09)	0.02	0.126		-0.01	(-0.05,0.04)	0.02	0.709
Understanding purpose (c)		0.03	(-0.01,0.07)	0.02	0.123		-0.02	(-0.06,0.02)	0.02	0.371
Self-rated mental health (b)		0.02	(-0.02,0.06)	0.02	0.328		-0.02	(-0.06,0.02)	0.02	0.366
Psychological Distress										
Traumatic distress	1.01		(0.99,1.04)	0.01	0.247	1.01		(0.99,1.04)	0.01	0.328
Depression symptoms composite	1.00		(0.98,1.02)	0.01	0.958	1.01		(0.99,1.03)	0.01	0.165
Depression – feel hopeless	1.01		(0.99,1.02)	0.01	0.484	1.02		(1.00,1.04)	0.01	0.068
Depression – loss of interest	1.00		(0.98,1.02)	0.01	0.981	1.01		(0.99,1.03)	0.01	0.373
Anxiety symptoms composite	0.99		(0.97,1.01)	0.01	0.217	1.00		(0.98,1.02)	0.01	0.760
Anxiety – feel on edge	0.99		(0.98,1.01)	0.01	0.547	1.00		(0.98,1.02)	0.01	0.780
Anxiety – cannot stop worrying	0.99		(0.97,1.01)	0.01	0.267	1.00		(0.98,1.02)	0.01	0.811
Suffering	1.01		(0.99,1.04)	0.01	0.171	1.02		(1.00,1.04)	0.01	0.069
Social Well-Being										
Relationship contentment (e)		0.07	(0.03,0.12)	0.02	2.19e-03**		0.03	(-0.01,0.07)	0.02	0.172
Relationship satisfaction (e)		0.05	(0.00,0.09)	0.02	0.045*		0.01	(-0.04,0.05)	0.02	0.777
Social support		0.03	(-0.01,0.08)	0.02	0.170		0.00	(-0.04,0.05)	0.02	0.893
Intimate/close friend	1.02		(1.00,1.04)	0.01	0.053	1.01		(0.99,1.02)	0.01	0.530
Government approval	1.00		(0.98,1.02)	0.01	0.831	1.00		(0.98,1.02)	0.01	0.957
Say in government	1.02		(1.00,1.04)	0.01	0.081	1.01		(0.99,1.04)	0.01	0.267
Belonging in country		0.02	(-0.02,0.07)	0.02	0.311		-0.01	(-0.06,0.04)	0.02	0.749
City/place satisfaction	1.03		(1.01,1.05)	0.01	0.014*	1.02		(1.00,1.04)	0.01	0.070
Trust within country	1.02		(0.99,1.04)	0.01	0.137	1.01		(0.99,1.04)	0.01	0.330
Social Participation										
Ever been married	1.00		(0.98,1.01)	0.01	0.895	1.00		(0.98,1.01)	0.01	0.798
Currently divorced	1.00		(0.99,1.01)	0.00	0.896	1.00		(0.99,1.01)	0.00	0.888
Number of children		0.01	(-0.02,0.05)	0.02	0.468		0.03	(-0.00,0.07)	0.02	0.075
Weekly+ community participation	1.00		(0.99,1.02)	0.01	0.742	1.00		(0.98,1.02)	0.01	0.998
Weekly+ religious attendance	1.01		(0.99,1.02)	0.01	0.337	1.00		(0.99,1.02)	0.01	0.710
Social Distress										
Loneliness		-0.01	(-0.06,0.04)	0.02	0.661		0.02	(-0.03,0.07)	0.02	0.415
Perceived discrimination	1.00		(0.98,1.02)	0.01	0.815	1.01		(0.99,1.02)	0.01	0.483
Character & Prosocial Behavior										
Orientation to promote good (d)		0.10	(0.05,0.14)	0.02	3.79e-05***		0.05	(0.01,0.09)	0.02	0.030*
Delayed gratification (d)		0.05	(0.01,0.10)	0.02	0.028*		0.03	(-0.02,0.07)	0.02	0.254
Hope		0.06	(0.01,0.11)	0.03	0.028*		0.01	(-0.04,0.06)	0.03	0.602
Gratitude		0.05	(0.01,0.09)	0.02	0.022*		0.00	(-0.04,0.04)	0.02	0.972
Showing love/care		0.05	(0.00,0.10)	0.02	0.052		0.01	(-0.04,0.06)	0.03	0.818
Forgivingness	1.16		(1.14,1.18)	0.01	2.22e-16***	1.16		(1.13,1.18)	0.01	2.22e-16***
Charitable giving	1.00		(0.98,1.02)	0.01	0.772	0.99		(0.97,1.01)	0.01	0.482
Helping strangers	1.03		(1.00,1.05)	0.01	0.032*	1.01		(0.99,1.03)	0.01	0.455
Volunteering	1.02		(1.00,1.03)	0.01	0.052	1.01		(0.99,1.03)	0.01	0.249
Physical Health & Health Behavior										
Self-rated physical health (b)		-0.01	(-0.05,0.03)	0.02	0.671		-0.02	(-0.07,0.02)	0.02	0.306
Health problems	1.01		(0.99,1.03)	0.01	0.273	1.01		(0.99,1.02)	0.01	0.475
Pain in past 4 weeks	1.02		(1.00,1.04)	0.01	0.100	1.02		(0.99,1.04)	0.01	0.182
Daily smoker	1.00		(0.98,1.02)	0.01	0.776	1.00		(0.98,1.02)	0.01	0.854
Number of drinks per week		-0.04	(-0.10,0.01)	0.03	0.103		-0.03	(-0.08,0.02)	0.03	0.202
Days exercise per week		0.03	(-0.01,0.08)	0.02	0.140		0.03	(-0.01,0.07)	0.02	0.162
Socioeconomic Outcomes										
Financial security (f)		-0.01	(-0.05,0.03)	0.02	0.652		-0.01	(-0.05,0.03)	0.02	0.778
Material security (f)		-0.01	(-0.06,0.03)	0.02	0.615		-0.01	(-0.05,0.04)	0.02	0.737
Educational attainment (16+ years)	1.00		(1.00,1.01)	0.00	0.173	1.00		(1.00,1.01)	0.00	0.223
Currently employed	1.00		(0.98,1.02)	0.01	0.794	1.00		(0.98,1.02)	0.01	0.819
Financially comfortable/getting by	0.98		(0.95,1.00)	0.01	0.091	0.98		(0.95,1.00)	0.01	0.071
Own home	1.00		(0.97,1.02)	0.01	0.886	1.00		(0.97,1.02)	0.01	0.828
Income – top quintile	1.00		(0.99,1.02)	0.01	0.908	1.00		(0.99,1.02)	0.01	0.810
Religion & Spirituality										
Religious/spiritual connection	1.04		(1.02,1.07)	0.01	8.35e-05***	1.01		(0.99,1.03)	0.01	0.182
Belief in life after death	1.05		(1.03,1.07)	0.01	2.37e-05***	1.02		(1.00,1.05)	0.01	0.024*
Transformative religious experience	1.05		(1.02,1.07)	0.01	8.81e-05***	1.02		(1.00,1.05)	0.01	0.028*
Religious reading or listening	1.03		(1.01,1.04)	0.01	8.78e-04**	1.01		(1.00,1.03)	0.01	0.087
Prayer or meditation	1.05		(1.03,1.07)	0.01	4.88e-08***	1.03		(1.01,1.04)	0.01	2.46e-03**
Belief in God/gods/spiritual forces	1.01		(1.00,1.03)	0.01	0.056	1.00		(0.99,1.02)	0.01	0.682
Religious centrality	1.03		(1.01,1.05)	0.01	0.008*	1.00		(0.98,1.02)	0.01	0.778
Religious/spiritual comfort	1.03		(1.01,1.05)	0.01	4.77e-03**	1.00		(0.98,1.02)	0.01	0.951
Feel loved by God	1.03		(1.01,1.05)	0.01	0.007*	1.00		(0.98,1.02)	0.01	0.923
Feel punished by God	0.98		(0.97,1.00)	0.01	0.093	0.99		(0.97,1.01)	0.01	0.264
Experienced religious criticism	0.99		(0.97,1.01)	0.01	0.223	0.99		(0.97,1.01)	0.01	0.216
Faith-sharing	1.05		(1.03,1.07)	0.01	1.67e-05***	1.02		(1.00,1.04)	0.01	0.034*

Notes. N=6724; Reference for focal predictor: never/rarely. RR, risk-ratio, null effect is 1.00; ES, effect size measure for standardized regression coefficient, null effect is 0.00; SE, standard error, the SE reported for binary/Likert-type outcomes where risk-ratios are on the log(RR) scale; CI, confidence interval; p-value, a Wald-type test of the null hypothesis that the effect of the focal predictor is zero; (a) item part of the Happiness & Life Satisfaction domain of the Secure Flourishing Index; (b) item part of the Physical & Mental Health domain of the Secure Flourishing Index; (c) item part of the Meaning & Purpose domain of the Secure Flourishing Index; (d) item part of the Character & Virtue domain of the Secure Flourishing Index; (e) item part of the Subjective Social Connectedness domain of the Secure Flourishing Index; (f) item part of the Financial & Material Security domain of the Secure Flourishing Index.

Multiple imputation was performed to impute missing data on the covariates, exposure, and outcomes. All models controlled for sociodemographic and childhood factors assessed at Wave 1. For Model 2 with PC (principal components), the first seven principal components of the entire set of contemporaneous confounders assessed at Wave 1 were included as additional covariates of the outcomes at Wave 2.

An outcome-wide analytic approach was used, and a separate model was run for each outcome. A different type of model was run depending on the nature of the outcome: (1) for each binary outcome, a weighted generalized linear model (with a log link and Poisson distribution) was used to estimate an RR; and (2) for each continuous outcome, a weighted linear regression model was used to estimate a ES. All effect sizes were standardized. For continuous outcomes, the ES represents the change in SD on the outcome between the lower and upper categories of the binary focal predictor. For binary outcomes, the RR represents the change in risk of being in the upper category compared to the lower category between the lower and upper categories of the binary focal predictor.

P-value significance thresholds: $p < 0.05$*, $p < 0.005$**, (Bonferroni) $p < 6.41e-04$***, correction for multiple testing using Bonferroni adjusted significant threshold.

Table S9h. Associations of forgivingness with adult well-being and other outcomes at Wave 2 in Argentina using complete-case analyses with attrition weights.

	Model 1: Demographic and Childhood Variables as Covariates					Model 2: Demographic, Childhood, and Other Wave 1 Confounding Variables (Via Principal Components) as Covariates				
Outcome	RR	ES	95% CI	SE	p-value	RR	ES	95% CI	SE	p-value
Human Flourishing										
Secure flourishing index		0.06	(0.01,0.11)	0.03	0.023*		-0.01	(-0.05,0.04)	0.02	0.780
Flourishing index		0.07	(0.02,0.12)	0.03	4.26e-03**		-0.00	(-0.05,0.04)	0.02	0.835
Happiness & life satisfaction		0.03	(-0.03,0.08)	0.03	0.315		-0.03	(-0.07,0.02)	0.02	0.252
Physical & mental health		-0.00	(-0.05,0.05)	0.03	0.909		-0.05	(-0.10,-0.01)	0.02	0.026*
Meaning & purpose		0.07	(0.02,0.12)	0.03	0.010*		-0.00	(-0.05,0.04)	0.02	0.865
Character & virtue		0.10	(0.04,0.15)	0.03	7.68e-04**		0.04	(-0.01,0.09)	0.03	0.143
Close social relationships		0.09	(0.03,0.15)	0.03	1.64e-03**		0.03	(-0.02,0.09)	0.03	0.236
Financial & material security		-0.01	(-0.06,0.05)	0.03	0.807		-0.01	(-0.06,0.05)	0.03	0.808
Psychological Well-Being										
Happiness (a)		-0.01	(-0.06,0.05)	0.03	0.785		-0.05	(-0.10,-0.01)	0.02	0.025*
Life satisfaction (a)		0.05	(0.00,0.11)	0.03	0.045*		0.00	(-0.05,0.05)	0.02	0.994
Current life evaluation		0.03	(-0.02,0.09)	0.03	0.243		0.00	(-0.05,0.06)	0.03	0.920
Future life evaluation		0.06	(0.01,0.12)	0.03	0.029*		0.03	(-0.02,0.09)	0.03	0.261
Optimism		0.05	(-0.01,0.11)	0.03	0.101		-0.00	(-0.06,0.05)	0.03	0.920
Freedom to pursue what's important		0.00	(-0.05,0.05)	0.03	0.996		-0.06	(-0.11,-0.02)	0.02	0.009*
Inner peace	1.03		(1.00,1.05)	0.01	0.019*	1.00		(0.98,1.03)	0.01	0.680
Life balance	1.03		(1.00,1.06)	0.01	0.024*	1.00		(0.98,1.03)	0.01	0.786
Sense of mastery	1.01		(1.00,1.03)	0.01	0.079	1.00		(0.99,1.02)	0.01	0.679
Meaningful activities (c)		0.06	(0.01,0.12)	0.03	0.019*		-0.00	(-0.05,0.05)	0.02	0.994
Understanding purpose (c)		0.06	(0.00,0.11)	0.03	0.032*		-0.01	(-0.06,0.04)	0.03	0.797
Self-rated mental health (b)		0.03	(-0.02,0.08)	0.03	0.264		-0.03	(-0.08,0.02)	0.02	0.248
Psychological Distress										
Traumatic distress	1.02		(0.99,1.05)	0.01	0.203	1.02		(0.99,1.04)	0.01	0.249
Depression symptoms composite	1.01		(0.98,1.04)	0.01	0.396	1.03		(1.00,1.06)	0.01	0.020*
Depression – feel hopeless	1.02		(0.99,1.04)	0.01	0.132	1.04		(1.01,1.06)	0.01	3.31e-03**
Depression – loss of interest	1.01		(0.99,1.04)	0.01	0.392	1.02		(1.00,1.05)	0.01	0.061
Anxiety symptoms composite	0.98		(0.95,1.01)	0.01	0.187	1.00		(0.97,1.02)	0.01	0.800
Anxiety – feel on edge	0.99		(0.97,1.02)	0.01	0.561	1.01		(0.98,1.03)	0.01	0.642
Anxiety – cannot stop worrying	1.00		(0.97,1.02)	0.01	0.759	1.01		(0.99,1.04)	0.01	0.413
Suffering	1.02		(0.99,1.05)	0.01	0.117	1.03		(1.00,1.06)	0.01	0.024*
Social Well-Being										
Relationship contentment (e)		0.10	(0.04,0.16)	0.03	7.18e-04**		0.05	(-0.01,0.11)	0.03	0.080
Relationship satisfaction (e)		0.06	(0.01,0.12)	0.03	0.022*		0.01	(-0.04,0.06)	0.03	0.711
Social support		0.02	(-0.04,0.07)	0.03	0.487		-0.02	(-0.07,0.04)	0.03	0.569
Intimate/close friend	1.02		(0.99,1.04)	0.01	0.156	1.00		(0.98,1.03)	0.01	0.746
Government approval	1.01		(0.98,1.03)	0.01	0.714	1.00		(0.97,1.03)	0.01	0.940
Say in government	1.01		(0.98,1.03)	0.01	0.693	1.00		(0.97,1.02)	0.01	0.772
Belonging in country		0.01	(-0.05,0.06)	0.03	0.742		-0.03	(-0.08,0.02)	0.03	0.280
City/place satisfaction	1.04		(1.02,1.07)	0.01	9.95e-04**	1.03		(1.01,1.06)	0.01	0.011*
Trust within country	1.01		(0.99,1.04)	0.01	0.317	1.00		(0.98,1.03)	0.01	0.817
Social Participation										
Ever been married	1.01		(1.00,1.03)	0.01	0.171	1.01		(0.99,1.02)	0.01	0.425
Currently divorced	1.00		(1.00,1.01)	0.00	0.197	1.00		(1.00,1.01)	0.00	0.311
Number of children		0.01	(-0.05,0.06)	0.03	0.814		0.03	(-0.02,0.09)	0.03	0.192
Weekly+ community participation	0.99		(0.98,1.01)	0.01	0.613	0.99		(0.97,1.01)	0.01	0.291
Weekly+ religious attendance	1.01		(1.00,1.03)	0.01	0.133	1.01		(0.99,1.02)	0.01	0.398
Social Distress										
Loneliness		-0.01	(-0.06,0.05)	0.03	0.828		0.04	(-0.00,0.09)	0.02	0.078
Perceived discrimination	1.00		(0.97,1.02)	0.01	0.708	1.00		(0.98,1.03)	0.01	0.759
Character & Prosocial Behavior										
Orientation to promote good (d)		0.08	(0.03,0.14)	0.03	2.44e-03**		0.02	(-0.03,0.07)	0.03	0.448
Delayed gratification (d)		0.08	(0.02,0.13)	0.03	0.008*		0.04	(-0.01,0.10)	0.03	0.133
Hope		0.06	(0.00,0.12)	0.03	0.047*		0.01	(-0.05,0.07)	0.03	0.827
Gratitude		0.04	(-0.01,0.10)	0.03	0.130		-0.02	(-0.07,0.03)	0.03	0.380
Showing love/care		0.03	(-0.02,0.09)	0.03	0.232		-0.03	(-0.08,0.03)	0.03	0.356
Forgivingness	1.16		(1.13,1.19)	0.01	2.22e-16***	1.15		(1.13,1.18)	0.01	2.22e-16***
Charitable giving	1.01		(0.98,1.03)	0.01	0.541	0.99		(0.97,1.02)	0.01	0.620
Helping strangers	1.03		(1.01,1.06)	0.01	0.019*	1.01		(0.99,1.04)	0.01	0.378
Volunteering	1.01		(0.99,1.03)	0.01	0.207	1.00		(0.98,1.02)	0.01	0.802
Physical Health & Health Behavior										
Self-rated physical health (b)		-0.04	(-0.09,0.01)	0.03	0.156		-0.07	(-0.12,-0.02)	0.03	0.011*
Health problems	1.02		(1.00,1.04)	0.01	0.020*	1.02		(1.00,1.04)	0.01	0.019*
Pain in past 4 weeks	1.03		(1.00,1.06)	0.01	0.036*	1.03		(1.00,1.06)	0.01	0.033*
Daily smoker	0.98		(0.96,1.01)	0.01	0.218	0.99		(0.97,1.01)	0.01	0.362
Number of drinks per week		-0.09	(-0.19,0.01)	0.05	0.065		-0.08	(-0.16,0.01)	0.04	0.074
Days exercise per week		0.03	(-0.02,0.09)	0.03	0.238		0.02	(-0.04,0.07)	0.03	0.522
Socioeconomic Outcomes										
Financial security (f)		0.00	(-0.05,0.06)	0.03	0.888		0.00	(-0.05,0.06)	0.03	0.887
Material security (f)		-0.02	(-0.07,0.04)	0.03	0.565		-0.02	(-0.07,0.04)	0.03	0.576
Educational attainment (16+ years)	1.00		(1.00,1.01)	0.00	0.012*	1.00		(1.00,1.01)	0.00	0.027*
Currently employed	1.00		(0.98,1.02)	0.01	0.996	1.00		(0.98,1.03)	0.01	0.796
Financially comfortable/getting by	0.98		(0.96,1.01)	0.01	0.191	0.98		(0.95,1.00)	0.01	0.073
Own home	1.00		(0.97,1.03)	0.01	0.973	1.00		(0.98,1.03)	0.01	0.810
Income – top quintile	1.01		(0.98,1.03)	0.01	0.549	1.01		(0.99,1.04)	0.01	0.390
Religion & Spirituality										
Religious/spiritual connection	1.05		(1.03,1.08)	0.01	8.53e-05***	1.01		(0.99,1.03)	0.01	0.334
Belief in life after death	1.07		(1.04,1.10)	0.01	1.27e-06***	1.04		(1.01,1.07)	0.01	2.33e-03**
Transformative religious experience	1.05		(1.03,1.08)	0.01	4.51e-05***	1.03		(1.00,1.05)	0.01	0.027*
Religious reading or listening	1.03		(1.02,1.05)	0.01	1.69e-04***	1.01		(1.00,1.03)	0.01	0.076
Prayer or meditation	1.06		(1.04,1.08)	0.01	9.58e-08***	1.02		(1.00,1.04)	0.01	0.016*
Belief in God/gods/spiritual forces	1.03		(1.01,1.05)	0.01	3.94e-03**	1.02		(1.00,1.04)	0.01	0.101
Religious centrality	1.03		(1.01,1.06)	0.01	0.012*	0.99		(0.97,1.01)	0.01	0.486
Religious/spiritual comfort	1.03		(1.00,1.05)	0.01	0.042*	0.98		(0.96,1.01)	0.01	0.161
Feel loved by God	1.04		(1.02,1.07)	0.01	9.05e-04**	1.01		(0.99,1.03)	0.01	0.471
Feel punished by God	0.99		(0.97,1.01)	0.01	0.169	0.99		(0.97,1.01)	0.01	0.473
Experienced religious criticism	0.99		(0.97,1.01)	0.01	0.364	0.99		(0.97,1.01)	0.01	0.339
Faith-sharing	1.06		(1.03,1.09)	0.01	2e-05***	1.03		(1.00,1.05)	0.01	0.046*

Table S9h. Associations of forgivingness with adult well-being and other outcomes at Wave 2 in Argentina using complete-case analyses with attrition weights.

	Model 1: Demographic and Childhood Variables as Covariates					Model 2: Demographic, Childhood, and Other Wave 1 Confounding Variables (Via Principal Components) as Covariates				
Outcome	RR	ES	95% CI	SE	p-value	RR	ES	95% CI	SE	p-value

Notes. N=2932; Reference for focal predictor: never/rarely. RR, risk-ratio, null effect is 1.00; ES, effect size measure for standardized regression coefficient, null effect is 0.00; SE, standard error, the SE reported for binary/Likert-type outcomes where risk-ratios are on the log(RR) scale; CI, confidence interval; p-value, a Wald-type test of the null hypothesis that the effect of the focal predictor is zero; (a) item part of the Happiness & Life Satisfaction domain of the Secure Flourishing Index; (b) item part of the Physical & Mental Health domain of the Secure Flourishing Index; (c) item part of the Meaning & Purpose domain of the Secure Flourishing Index; (d) item part of the Character & Virtue domain of the Secure Flourishing Index; (e) item part of the Subjective Social Connectedness domain of the Secure Flourishing Index; (f) item part of the Financial & Material Security domain of the Secure Flourishing Index.

Attrition weights were computed to adjust the complete case data (those who responded at Wave 2 to at least 50% of the questions) and multiple imputation was used to impute missing data on all remaining within wave on the covariates, exposure, and outcomes. All models controlled for sociodemographic and childhood factors assessed at Wave 1. For Model 2 with PC (principal components), the first seven principal components of the entire set of contemporaneous confounders assessed at Wave 1 were included as additional covariates of the outcomes at Wave 2.

An outcome-wide analytic approach was used, and a separate model was run for each outcome. A different type of model was run depending on the nature of the outcome: (1) for each binary outcome, a weighted generalized linear model (with a log link and Poisson distribution) was used to estimate an RR; and (2) for each continuous outcome, a weighted linear regression model was used to estimate a ES. All effect sizes were standardized. For continuous outcomes, the ES represents the change in SD on the outcome between the lower and upper categories of the binary focal predictor. For binary outcomes, the RR represents the change in risk of being in the upper category compared to the lower category between the lower and upper categories of the binary focal predictor.

P-value significance thresholds: $p < 0.05$*, $p < 0.005$**, (Bonferroni) $p < 6.41e-04$***, correction for multiple testing using Bonferroni adjusted significant threshold.

Table S9i. Sensitivity analysis of forgivingness outcome-wide results to unmeasured confounding using E-values in Argentina

	Multiple Imputation				Complete Case w/ Attrition Weights			
	Model 1: Demographics and Childhood Variables as Covariates		Model 2: Demographics, Childhood, and Other Wave 1 Confounders (Via Principal Components) as Covariates		Model 1: Demographics and Childhood Variables as Covariates		Model 2: Demographics, Childhood, and Other Wave 1 Confounders (Via Principal Components) as Covariates	
Outcome	EE	ECI	EE	ECI	EE	ECI	EE	ECI
Human Flourishing								
Secure flourishing index	1.45	1.19	1.07	1.00	1.52	1.15	1.13	1.00
Flourishing index	1.54	1.33	1.06	1.00	1.60	1.27	1.11	1.00
Happiness & life satisfaction	1.30	1.00	1.21	1.00	1.30	1.00	1.30	1.00
Physical & mental health	1.13	1.00	1.29	1.00	1.08	1.00	1.48	1.13
Meaning & purpose	1.40	1.01	1.22	1.00	1.57	1.22	1.10	1.00
Character & virtue	1.69	1.44	1.43	1.11	1.73	1.39	1.39	1.00
Close social relationships	1.57	1.30	1.26	1.00	1.70	1.35	1.34	1.00
Financial & material security	1.19	1.00	1.15	1.00	1.13	1.00	1.13	1.00
Psychological Well-Being								
Happiness	1.17	1.00	1.28	1.00	1.14	1.00	1.48	1.13
Life satisfaction	1.35	1.00	1.07	1.00	1.48	1.06	1.02	1.00
Current life evaluation	1.27	1.00	1.07	1.00	1.35	1.00	1.08	1.00
Future life evaluation	1.40	1.06	1.25	1.00	1.52	1.13	1.33	1.00
Optimism	1.37	1.00	1.01	1.00	1.45	1.00	1.08	1.00
Freedom to pursue what's important	1.10	1.00	1.41	1.07	1.02	1.00	1.54	1.22
Inner peace	1.25	1.04	1.11	1.00	1.34	1.12	1.12	1.00
Life balance	1.21	1.00	1.02	1.00	1.33	1.10	1.10	1.00
Sense of mastery	1.15	1.00	1.02	1.00	1.21	1.00	1.09	1.00
Meaningful activities	1.38	1.00	1.15	1.00	1.54	1.17	1.02	1.00
Understanding purpose	1.34	1.00	1.24	1.00	1.50	1.11	1.13	1.00
Self-rated mental health	1.25	1.00	1.23	1.00	1.32	1.00	1.32	1.00
Psychological Distress								
Traumatic distress	1.22	1.00	1.20	1.00	1.24	1.00	1.23	1.00
Depression symptoms composite	1.04	1.00	1.21	1.00	1.19	1.00	1.34	1.11
Depression – feel hopeless	1.14	1.00	1.24	1.00	1.26	1.00	1.39	1.19
Depression – loss of interest	1.02	1.00	1.16	1.00	1.19	1.00	1.29	1.00
Anxiety symptoms composite	1.20	1.00	1.09	1.00	1.26	1.00	1.09	1.00
Anxiety – feel on edge	1.13	1.00	1.08	1.00	1.15	1.00	1.13	1.00
Anxiety – cannot stop worrying	1.18	1.00	1.08	1.00	1.11	1.00	1.18	1.00
Suffering	1.22	1.00	1.26	1.00	1.28	1.00	1.35	1.11
Social Well-Being								
Relationship contentment	1.59	1.30	1.33	1.00	1.78	1.42	1.46	1.00
Relationship satisfaction	1.44	1.07	1.13	1.00	1.54	1.16	1.17	1.00
Social support	1.34	1.00	1.09	1.00	1.25	1.00	1.22	1.00
Intimate/close friend	1.25	1.00	1.13	1.00	1.24	1.00	1.10	1.00
Government approval	1.08	1.00	1.04	1.00	1.12	1.00	1.05	1.00
Say in government	1.27	1.00	1.21	1.00	1.13	1.00	1.11	1.00
Belonging in country	1.28	1.00	1.14	1.00	1.16	1.00	1.32	1.00
City/place satisfaction	1.31	1.12	1.26	1.00	1.44	1.24	1.36	1.15
Trust within country	1.26	1.00	1.20	1.00	1.21	1.00	1.09	1.00
Social Participation								
Ever been married	1.05	1.00	1.07	1.00	1.19	1.00	1.14	1.00
Currently divorced	1.04	1.00	1.04	1.00	1.10	1.00	1.09	1.00
Number of children	1.20	1.00	1.33	1.00	1.13	1.00	1.35	1.00
Weekly+ community participation	1.09	1.00	1.01	1.00	1.12	1.00	1.18	1.00
Weekly+ religious attendance	1.14	1.00	1.08	1.00	1.21	1.00	1.14	1.00
Social Distress								
Loneliness	1.17	1.00	1.25	1.00	1.12	1.00	1.41	1.00
Perceived discrimination	1.08	1.00	1.14	1.00	1.11	1.00	1.10	1.00
Character & Prosocial Behavior								
Orientation to promote good	1.74	1.47	1.46	1.12	1.66	1.32	1.25	1.00
Delayed gratification	1.46	1.13	1.30	1.00	1.62	1.25	1.40	1.00
Hope	1.50	1.14	1.20	1.00	1.53	1.05	1.13	1.00
Gratitude	1.46	1.15	1.04	1.00	1.40	1.00	1.27	1.00
Showing love/care	1.45	1.03	1.12	1.00	1.35	1.00	1.29	1.00
Forgivingness	2.15	2.00	2.12	1.97	2.14	1.96	2.11	1.93
Charitable giving	1.09	1.00	1.15	1.00	1.15	1.00	1.13	1.00
Helping strangers	1.31	1.08	1.16	1.00	1.35	1.12	1.19	1.00
Volunteering	1.24	1.00	1.18	1.00	1.20	1.00	1.08	1.00
Physical Health & Health Behavior								
Self-rated physical health	1.16	1.00	1.28	1.00	1.37	1.00	1.55	1.21
Health problems	1.17	1.00	1.13	1.00	1.27	1.09	1.27	1.10
Pain in past 4 weeks	1.25	1.00	1.22	1.00	1.34	1.07	1.33	1.08
Daily smoker	1.09	1.00	1.07	1.00	1.23	1.00	1.19	1.00
Number of drinks per week	1.42	1.00	1.35	1.00	1.71	1.00	1.61	1.00
Days exercise per week	1.35	1.00	1.33	1.00	1.35	1.00	1.23	1.00
Socioeconomic Outcomes								
Financial security	1.17	1.00	1.12	1.00	1.10	1.00	1.10	1.00
Material security	1.19	1.00	1.15	1.00	1.22	1.00	1.22	1.00
Educational attainment (16+ years)	1.09	1.00	1.09	1.00	1.09	1.04	1.08	1.03
Currently employed	1.08	1.00	1.07	1.00	1.01	1.00	1.09	1.00
Financially comfortable/getting by	1.28	1.00	1.31	1.00	1.25	1.00	1.30	1.00
Own home	1.07	1.00	1.09	1.00	1.03	1.00	1.09	1.00
Income – top quintile	1.05	1.00	1.07	1.00	1.14	1.00	1.18	1.00
Religion & Spirituality								
Religious/spiritual connection	1.44	1.29	1.21	1.00	1.49	1.31	1.19	1.00
Belief in life after death	1.48	1.32	1.30	1.09	1.58	1.41	1.41	1.21
Transformative religious experience	1.45	1.29	1.30	1.08	1.50	1.33	1.32	1.09
Religious reading or listening	1.32	1.19	1.21	1.00	1.36	1.23	1.22	1.00
Prayer or meditation	1.48	1.36	1.31	1.17	1.53	1.39	1.30	1.11
Belief in God/gods/spiritual forces	1.21	1.00	1.09	1.00	1.34	1.17	1.24	1.00
Religious centrality	1.31	1.14	1.08	1.00	1.37	1.15	1.15	1.00
Religious/spiritual comfort	1.35	1.17	1.04	1.00	1.32	1.05	1.23	1.00
Feel loved by God	1.32	1.15	1.05	1.00	1.43	1.24	1.15	1.00
Feel punished by God	1.23	1.00	1.18	1.00	1.21	1.00	1.15	1.00
Experienced religious criticism	1.19	1.00	1.19	1.00	1.16	1.00	1.17	1.00
Faith-sharing	1.46	1.31	1.29	1.07	1.54	1.35	1.32	1.03

Notes. EE, E-value for estimate; ECI, E-value for the limit of the confidence interval. The formula for calculating E-values can be found in VanderWeele and Ding (2017). E-values for estimate are the minimum strength of association on the risk ratio scale that an unmeasured confounder would need to have with both the exposure and the outcome to fully explain away the observed association between the exposure and outcome, conditional on the measured covariates. E-values for the 95% CI closest to the null denote the minimum strength of association on the risk ratio scale that an unmeasured confounder would need to have with both the exposure and the outcome to shift the CI to include the null value, conditional on the measured covariates.

Table S10a. Weighted summary statistics for demographic and childhood variables in Australia

**Characteristic**	**Wave 1** N = 3,844	**Wave 2** N = 2,590
Forgivingness, n (%)		
Always	628 (16.3%)	431 (16.7%)
Often	2,374 (61.8%)	1,615 (62.3%)
Rarely	745 (19.4%)	478 (18.4%)
Never	89 (2.3%)	63 (2.4%)
(Missing)	8 (0.2%)	4 (0.1%)
Year of birth, n (%)		
1943 or earlier (current age: 80+ years)	167 (4.4%)	168 (6.5%)
1943-1953 (current age: 70-79 years)	459 (11.9%)	311 (12.0%)
1953-1963 (current age: 60-69 years)	532 (13.8%)	394 (15.2%)
1963-1973 (current age: 50-59 years)	659 (17.1%)	445 (17.2%)
1973-1983 (current age: 40-49 years)	607 (15.8%)	414 (16.0%)
1983-1993 (current age: 30-39 years)	674 (17.5%)	465 (18.0%)
1993-1998 (current age: 25-29 years)	313 (8.1%)	199 (7.7%)
1998-2005 (current age: 18-24 years)	431 (11.2%)	193 (7.4%)
(Missing)	1 (0.0%)	0 (<0.0%)
Age of participant		
Mean	48.3	50.4
Standard Deviation	18.3	18.3
Min, Max	18.0, 95.0	19.0, 95.0
(Missing)	1 (<0.1%)	0 (<0.1%)
Gender, n (%)		
Male	1,878 (48.9%)	1,270 (49.1%)
Female	1,920 (49.9%)	1,290 (49.8%)
Other	40 (1.0%)	27 (1.0%)
(Missing)	6 (0.2%)	3 (0.1%)
Respondent marital status, n (%)		
Single/Never been married	949 (24.7%)	582 (22.5%)
Married	1,708 (44.4%)	1,225 (47.3%)
Separated	157 (4.1%)	92 (3.6%)
Divorced	319 (8.3%)	214 (8.3%)
Widowed	206 (5.4%)	151 (5.8%)
Domestic partner	467 (12.1%)	315 (12.2%)
(Missing)	38 (1.0%)	10 (0.4%)
Education (years), n (%)		
Up to 8	75 (1.9%)	38 (1.5%)
9-15	2,528 (65.8%)	1,611 (62.2%)
16+	1,230 (32.0%)	940 (36.3%)
(Missing)	11 (0.3%)	0 (0%)
Employment status, n (%)		
Employed for an employer	1,904 (49.5%)	1,280 (49.4%)
Self-employed	360 (9.4%)	212 (8.2%)
Retired	846 (22.0%)	610 (23.6%)
Student	218 (5.7%)	115 (4.4%)
Homemaker	148 (3.9%)	85 (3.3%)
Unemployed and looking for a job	145 (3.8%)	104 (4.0%)
None of these/Other	218 (5.7%)	151 (5.8%)
(Missing)	4 (0.1%)	33 (1.3%)
Current religious service attendance, n (%)		
More than once a week	163 (4.3%)	114 (4.4%)

Table S10a. Weighted summary statistics for demographic and childhood variables in Australia

Characteristic	Wave 1<br>N = 3,844	Wave 2<br>N = 2,590
Once a week	292 (7.6%)	190 (7.3%)
One to three times a month	132 (3.4%)	76 (2.9%)
A few times a year	656 (17.1%)	424 (16.4%)
Never	2,594 (67.5%)	1,768 (68.3%)
(Missing)	7 (0.2%)	18 (0.7%)
Immigration status, n (%)		
Born in this country	2,971 (77.3%)	1,996 (77.1%)
Born in another country	866 (22.5%)	586 (22.6%)
(Missing)	7 (0.2%)	8 (0.3%)
Parental marital status around age 12, n (%)		
Parents were married	3,011 (78.3%)	2,061 (79.6%)
Parents were divorced	479 (12.5%)	335 (12.9%)
Parents were never married	208 (5.4%)	111 (4.3%)
One or both of them had died	92 (2.4%)	66 (2.6%)
Unsure	13 (0.3%)	7 (0.3%)
(Missing)	41 (1.1%)	10 (0.4%)
Religious service attendance around age 12, n (%)		
At least once a week	1,329 (34.6%)	935 (36.1%)
One to three times a month	480 (12.5%)	338 (13.0%)
Less than once a month	609 (15.8%)	393 (15.2%)
Never	1,333 (34.7%)	879 (33.9%)
(Missing)	93 (2.4%)	45 (1.7%)
Relationship with mother when growing up, n (%)		
Very good	2,551 (66.4%)	1,714 (66.2%)
Somewhat good	923 (24.0%)	638 (24.6%)
Somewhat bad	220 (5.7%)	144 (5.6%)
Very bad	110 (2.9%)	62 (2.4%)
(Does not apply)	33 (0.9%)	29 (1.1%)
(Missing)	6 (0.2%)	3 (0.1%)
Relationship with father when growing up, n (%)		
Very good	2,012 (52.3%)	1,343 (51.8%)
Somewhat good	1,144 (29.8%)	788 (30.4%)
Somewhat bad	319 (8.3%)	212 (8.2%)
Very bad	208 (5.4%)	143 (5.5%)
(Does not apply)	151 (3.9%)	99 (3.8%)
(Missing)	9 (0.2%)	5 (0.2%)
Felt like an outsider in family when growing up, n (%)		
Yes	778 (20.2%)	513 (19.8%)
No	3,039 (79.0%)	2,052 (79.2%)
(Missing)	28 (0.7%)	24 (0.9%)
Experienced abuse when growing up, n (%)		
Yes	999 (26.0%)	696 (26.9%)
No	2,787 (72.5%)	1,873 (72.3%)
(Missing)	58 (1.5%)	21 (0.8%)
Self-rated health when growing up, n (%)		
Excellent	1,691 (44.0%)	1,138 (43.9%)
Very good	1,088 (28.3%)	732 (28.3%)
Good	620 (16.1%)	426 (16.4%)
Fair	329 (8.6%)	233 (9.0%)
Poor	112 (2.9%)	60 (2.3%)

Table S10a. Weighted summary statistics for demographic and childhood variables in Australia

**Characteristic**	**Wave 1** N = 3,844	**Wave 2** N = 2,590
(Missing)	4 (0.1%)	1 (0.0%)
Subjective financial status of family growing up, n (%)		
Lived comfortably	1,745 (45.4%)	1,183 (45.7%)
Got by	1,495 (38.9%)	1,015 (39.2%)
Found it difficult	433 (11.3%)	282 (10.9%)
Found it very difficult	155 (4.0%)	104 (4.0%)
(Missing)	15 (0.4%)	5 (0.2%)
Religious affiliation growing up, n (%)		
Christianity	2,625 (68.3%)	1,785 (68.9%)
Taoism	1 (0.0%)	1 (0.0%)
Confucianism	0 (0%)	0 (0%)
Primal, Animist, or Folk religion	4 (0.1%)	1 (0.0%)
Spiritism	0 (0%)	0 (0%)
Umbanda, Candomblé, and other African-derived religions	0 (0%)	0 (0%)
Chinese folk/traditional religion	0 (0%)	0 (0%)
Islam	52 (1.4%)	37 (1.4%)
Hinduism	41 (1.1%)	22 (0.8%)
Buddhism	18 (0.5%)	4 (0.2%)
Judaism	28 (0.7%)	13 (0.5%)
Sikhism	7 (0.2%)	0 (0%)
Baha'i	4 (0.1%)	3 (0.1%)
Jainism	0 (0%)	0 (0%)
Shinto	0 (0%)	0 (0%)
Some other religion	8 (0.2%)	7 (0.3%)
No religion/Atheist/Agnostic	1,034 (26.9%)	705 (27.2%)
(Missing)	21 (0.5%)	12 (0.5%)

Note. N (%); this table is based on non-imputed data. Cumulative percentages for variables may not add up to 100% due to rounding. Wave 1 characteristics weighted using the Gallup provided sampling weight, ANNUAL_WEIGHT_R2; Wave 2 characteristics weighted accounting for attrition by using the adjusted Wave 1 weight, ANNUAL_WEIGHT_R2, multiplied by the created attrition weight to account for dropout, to maintain nationally representative estimates for Wave 2 characteristics.

Table S10b. Weighted summary statistics for outcome variables in Australia

Outcome	Wave 1<br>N = 3,844	Wave 2<br>N = 2,590
Secure flourishing index		
Mean	6.9	6.9
Standard Deviation	1.6	1.7
Min, Max	1.0, 10.0	0.5, 10.0
(Missing)	38 (1.0%)	93 (3.6%)
Flourishing index		
Mean	7.0	6.9
Standard Deviation	1.6	1.6
Min, Max	1.2, 10.0	0.6, 10.0
(Missing)	33 (0.9%)	89 (3.5%)
Happiness & life satisfaction		
Mean	6.7	6.7
Standard Deviation	1.9	1.9
Min, Max	0.0, 10.0	0.0, 10.0
(Missing)	10 (0.3%)	3 (<0.1%)
Physical & mental health		
Mean	6.6	6.5
Standard Deviation	1.9	1.9
Min, Max	0.0, 10.0	0.0, 10.0
(Missing)	7 (0.2%)	65 (2.5%)
Meaning & purpose		
Mean	7.0	6.9
Standard Deviation	2.1	2.2
Min, Max	0.0, 10.0	0.0, 10.0
(Missing)	4 (0.1%)	6 (0.2%)
Character & virtue		
Mean	7.5	7.5
Standard Deviation	1.5	1.5
Min, Max	0.0, 10.0	0.0, 10.0
(Missing)	10 (0.3%)	11 (0.4%)
Close social relationships		
Mean	7.0	7.1
Standard Deviation	2.3	2.3
Min, Max	0.0, 10.0	0.0, 10.0
(Missing)	7 (0.2%)	8 (0.3%)
Financial & material security		
Mean	6.9	6.8
Standard Deviation	2.8	2.8
Min, Max	0.0, 10.0	0.0, 10.0
(Missing)	6 (0.1%)	5 (0.2%)
Happiness		
Mean	6.8	6.8
Standard Deviation	1.8	1.9
Min, Max	0.0, 10.0	0.0, 10.0
(Missing)	5 (0.1%)	1 (<0.1%)
Life satisfaction		
Mean	6.7	6.7
Standard Deviation	2.1	2.2
Min, Max	0.0, 10.0	0.0, 10.0
(Missing)	9 (0.2%)	1 (<0.1%)
Current life evaluation		

Table S10b. Weighted summary statistics for outcome variables in Australia

Outcome	Wave 1<br>N = 3,844	Wave 2<br>N = 2,590
Mean	6.7	6.7
Standard Deviation	1.8	1.8
Min, Max	0.0, 10.0	0.0, 10.0
(Missing)	3 (<0.1%)	5 (0.2%)
Future life evaluation		
Mean	7.6	7.4
Standard Deviation	1.9	2.0
Min, Max	0.0, 10.0	0.0, 10.0
(Missing)	6 (0.2%)	13 (0.5%)
Optimism		
Mean	7.3	7.3
Standard Deviation	2.3	2.3
Min, Max	0.0, 10.0	0.0, 10.0
(Missing)	52 (1.3%)	24 (0.9%)
Freedom to pursue what's important		
Mean	7.1	7.0
Standard Deviation	2.6	2.5
Min, Max	0.0, 10.0	0.0, 10.0
(Missing)	1 (<0.1%)	3 (0.1%)
Inner peace, n (%)		
Always	446 (11.6%)	288 (11.1%)
Often	2,500 (65.0%)	1,641 (63.4%)
Rarely	826 (21.5%)	607 (23.4%)
Never	62 (1.6%)	24 (0.9%)
(Missing)	10 (0.2%)	30 (1.2%)
Life balance, n (%)		
Always	237 (6.2%)	170 (6.6%)
Often	2,424 (63.1%)	1,645 (63.5%)
Rarely	1,088 (28.3%)	712 (27.5%)
Never	90 (2.3%)	51 (2.0%)
(Missing)	5 (0.1%)	12 (0.5%)
Sense of mastery, n (%)		
Always	536 (13.9%)	297 (11.5%)
Often	2,691 (70.0%)	1,798 (69.4%)
Rarely	555 (14.4%)	457 (17.6%)
Never	34 (0.9%)	25 (1.0%)
(Missing)	28 (0.7%)	14 (0.5%)
Meaningful activities		
Mean	7.2	7.1
Standard Deviation	2.1	2.2
Min, Max	0.0, 10.0	0.0, 10.0
(Missing)	2 (<0.1%)	1 (<0.1%)
Understanding purpose		
Mean	6.8	6.7
Standard Deviation	2.6	2.6
Min, Max	0.0, 10.0	0.0, 10.0
(Missing)	3 (<0.1%)	5 (0.2%)
Self-rated mental health		
Mean	6.7	6.7
Standard Deviation	2.3	2.3
Min, Max	0.0, 10.0	0.0, 10.0

Table S10b. Weighted summary statistics for outcome variables in Australia

Outcome	Wave 1<br>N = 3,844	Wave 2<br>N = 2,590
(Missing)	4 (0.1%)	13 (0.5%)
Traumatic distress, n (%)		
A lot	332 (8.6%)	218 (8.4%)
Some	836 (21.7%)	554 (21.4%)
Not very much	1,178 (30.6%)	853 (32.9%)
Not at all	1,496 (38.9%)	959 (37.0%)
(Missing)	3 (0.1%)	6 (0.2%)
Depression symptoms composite, n (%)	977 (25.4%)	616 (23.8%)
(Missing)	7 (0.2%)	7 (0.3%)
Depression – feel hopeless, n (%)		
Nearly every day	316 (8.2%)	211 (8.1%)
More than half the days	389 (10.1%)	274 (10.6%)
Several days	1,314 (34.2%)	925 (35.7%)
Not at all	1,821 (47.4%)	1,179 (45.5%)
(Missing)	4 (0.1%)	2 (0.1%)
Depression – loss of interest, n (%)		
Nearly every day	305 (7.9%)	206 (7.9%)
More than half the days	567 (14.8%)	323 (12.5%)
Several days	1,326 (34.5%)	943 (36.4%)
Not at all	1,642 (42.7%)	1,112 (42.9%)
(Missing)	3 (0.1%)	6 (0.2%)
Anxiety symptoms composite, n (%)	927 (24.2%)	680 (26.3%)
(Missing)	11 (0.3%)	7 (0.3%)
Anxiety – feel on edge, n (%)		
Nearly every day	385 (10.0%)	258 (10.0%)
More than half the days	449 (11.7%)	350 (13.5%)
Several days	1,625 (42.3%)	1,040 (40.2%)
Not at all	1,380 (35.9%)	936 (36.2%)
(Missing)	6 (0.2%)	5 (0.2%)
Anxiety – cannot stop worrying, n (%)		
Nearly every day	333 (8.7%)	221 (8.5%)
More than half the days	402 (10.5%)	319 (12.3%)
Several days	1,319 (34.3%)	874 (33.7%)
Not at all	1,784 (46.4%)	1,174 (45.3%)
(Missing)	5 (0.1%)	3 (0.1%)
Suffering, n (%)		
A lot	501 (13.0%)	358 (13.8%)
Some	1,464 (38.1%)	1,005 (38.8%)
Not very much	1,334 (34.7%)	839 (32.4%)
Not at all	532 (13.8%)	347 (13.4%)
(Missing)	13 (0.3%)	41 (1.6%)
Relationship contentment		
Mean	7.2	7.3
Standard Deviation	2.3	2.3
Min, Max	0.0, 10.0	0.0, 10.0
(Missing)	5 (0.1%)	1 (<0.1%)
Relationship satisfaction		
Mean	6.8	6.9
Standard Deviation	2.5	2.5
Min, Max	0.0, 10.0	0.0, 10.0
(Missing)	3 (<0.1%)	7 (0.3%)

Table S10b. Weighted summary statistics for outcome variables in Australia

Outcome	Wave 1<br>N = 3,844	Wave 2<br>N = 2,590
Social support		
Mean	8.1	8.1
Standard Deviation	2.3	2.3
Min, Max	0.0, 10.0	0.0, 10.0
(Missing)	10 (0.3%)	57 (2.2%)
Intimate/close friend, n (%)		
Yes	3,186 (82.9%)	2,189 (84.5%)
No	566 (14.7%)	385 (14.9%)
(Missing)	91 (2.4%)	15 (0.6%)
Government approval, n (%)		
Strongly approve	341 (8.9%)	143 (5.5%)
Somewhat approve	1,252 (32.6%)	773 (29.9%)
Neither approve nor disapprove	990 (25.8%)	544 (21.0%)
Somewhat disapprove	827 (21.5%)	669 (25.8%)
Strongly disapprove	416 (10.8%)	452 (17.4%)
(Missing)	17 (0.4%)	8 (0.3%)
Say in government, n (%)		
Agree	1,434 (37.3%)	877 (33.9%)
Disagree	1,578 (41.1%)	1,182 (45.6%)
Unsure	820 (21.3%)	527 (20.4%)
(Missing)	12 (0.3%)	4 (0.1%)
Belonging in country		
Mean	7.7	7.6
Standard Deviation	2.3	2.4
Min, Max	0.0, 10.0	0.0, 10.0
(Missing)	31 (0.8%)	23 (0.9%)
City/place satisfaction, n (%)		
Satisfied	3,126 (81.3%)	2,014 (77.8%)
Dissatisfied	413 (10.7%)	301 (11.6%)
Unsure	215 (5.6%)	233 (9.0%)
(Missing)	90 (2.4%)	41 (1.6%)
Trust within country, n (%)		
All people	20 (0.5%)	1 (0.0%)
Most people	1,424 (37.1%)	965 (37.3%)
Some people	1,733 (45.1%)	1,165 (45.0%)
Not very many people	633 (16.5%)	438 (16.9%)
None	6 (0.2%)	19 (0.7%)
(Missing)	28 (0.7%)	2 (0.1%)
Number of children		
Mean	0.5	0.5
Standard Deviation	1.0	0.9
Min, Max	0.0, 6.0	0.0, 6.0
(Missing)	6 (0.1%)	576 (22%)
Community participation, n (%)		
More than once a week	563 (14.7%)	405 (15.6%)
Once a week	508 (13.2%)	323 (12.5%)
One to three times a month	460 (12.0%)	390 (15.1%)
A few times a year	904 (23.5%)	567 (21.9%)
Never	1,402 (36.5%)	900 (34.8%)
(Missing)	6 (0.2%)	5 (0.2%)
Religious attendance, n (%)		

Table S10b. Weighted summary statistics for outcome variables in Australia

**Outcome**	**Wave 1**<br>N = 3,844	**Wave 2**<br>N = 2,590
More than once a week	163 (4.3%)	114 (4.4%)
Once a week	292 (7.6%)	190 (7.3%)
One to three times a month	132 (3.4%)	76 (2.9%)
A few times a year	656 (17.1%)	424 (16.4%)
Never	2,594 (67.5%)	1,768 (68.3%)
(Missing)	7 (0.2%)	18 (0.7%)
Loneliness		
Mean	3.4	3.4
Standard Deviation	2.8	2.8
Min, Max	0.0, 10.0	0.0, 10.0
(Missing)	1 (<0.1%)	1 (<0.1%)
Perceived discrimination, n (%)		
Always	118 (3.1%)	73 (2.8%)
Often	582 (15.1%)	446 (17.2%)
Rarely	2,014 (52.4%)	1,300 (50.2%)
Never	1,125 (29.3%)	769 (29.7%)
(Missing)	5 (0.1%)	1 (0.0%)
Orientation to promote good		
Mean	7.8	7.9
Standard Deviation	1.6	1.6
Min, Max	0.0, 10.0	0.0, 10.0
(Missing)	6 (0.1%)	10 (0.4%)
Delayed gratification		
Mean	7.2	7.2
Standard Deviation	2.0	2.0
Min, Max	0.0, 10.0	0.0, 10.0
(Missing)	4 (0.1%)	1 (<0.1%)
Hope		
Mean	7.7	7.5
Standard Deviation	2.0	2.1
Min, Max	0.0, 10.0	0.0, 10.0
(Missing)	152 (4.0%)	63 (2.4%)
Gratitude		
Mean	7.8	7.8
Standard Deviation	2.3	2.2
Min, Max	0.0, 10.0	0.0, 10.0
(Missing)	147 (3.8%)	64 (2.5%)
Showing love/care		
Mean	8.3	8.3
Standard Deviation	1.8	1.8
Min, Max	0.0, 10.0	0.0, 10.0
(Missing)	1 (<0.1%)	16 (0.6%)
Forgivingness, n (%)		
Always	628 (16.3%)	431 (16.7%)
Often	2,374 (61.8%)	1,615 (62.3%)
Rarely	745 (19.4%)	478 (18.4%)
Never	89 (2.3%)	63 (2.4%)
(Missing)	8 (0.2%)	4 (0.1%)
Charitable giving, n (%)		
Yes	2,000 (52.0%)	1,380 (53.3%)
No	1,834 (47.7%)	1,188 (45.9%)

Table S10b. Weighted summary statistics for outcome variables in Australia

**Outcome**	**Wave 1** N = 3,844	**Wave 2** N = 2,590
(Missing)	9 (0.2%)	22 (0.8%)
Helping strangers, n (%)		
Yes	2,356 (61.3%)	1,554 (60.0%)
No	1,453 (37.8%)	1,030 (39.8%)
(Missing)	35 (0.9%)	6 (0.2%)
Volunteering, n (%)		
Yes	1,255 (32.7%)	869 (33.6%)
No	2,586 (67.3%)	1,714 (66.2%)
(Missing)	3 (0.1%)	7 (0.3%)
Self-rated physical health		
Mean	6.5	6.4
Standard Deviation	2.1	2.0
Min, Max	0.0, 10.0	0.0, 10.0
(Missing)	4 (0.1%)	64 (2.5%)
Health problems, n (%)		
Yes	1,055 (27.5%)	808 (31.2%)
No	2,747 (71.5%)	1,776 (68.6%)
(Missing)	42 (1.1%)	6 (0.3%)
Pain in past 4 weeks, n (%)		
A lot	617 (16.0%)	453 (17.5%)
Some	1,535 (39.9%)	1,035 (39.9%)
Not very much	1,246 (32.4%)	840 (32.4%)
None at all	438 (11.4%)	244 (9.4%)
(Missing)	8 (0.2%)	18 (0.7%)
Number of cigarettes per day		
Mean	1.3	1.2
Standard Deviation	4.6	4.3
Min, Max	0.0, 40.0	0.0, 40.0
(Missing)	42 (1.1%)	111 (4.3%)
Number of drinks per week		
Mean	4.6	4.0
Standard Deviation	8.4	7.3
Min, Max	0.0, 97.0	0.0, 80.0
(Missing)	29 (0.8%)	100 (3.9%)
Days exercise per week		
Mean	2.7	2.7
Standard Deviation	2.3	2.2
Min, Max	0.0, 7.0	0.0, 7.0
(Missing)	3 (<0.1%)	4 (0.2%)
Financial security		
Mean	6.6	6.5
Standard Deviation	3.0	3.0
Min, Max	0.0, 10.0	0.0, 10.0
(Missing)	2 (<0.1%)	1 (<0.1%)
Material security		
Mean	7.2	7.1
Standard Deviation	2.8	2.8
Min, Max	0.0, 10.0	0.0, 10.0
(Missing)	4 (0.1%)	4 (0.2%)
Educational attainment (16+ years), n (%)		
Up to 8	75 (1.9%)	38 (1.5%)

Table S10b. Weighted summary statistics for outcome variables in Australia

Outcome	Wave 1<br>N = 3,844	Wave 2<br>N = 2,590
9-15	2,528 (65.8%)	1,611 (62.2%)
16+	1,230 (32.0%)	940 (36.3%)
(Missing)	11 (0.3%)	0 (0%)
Currently employed, n (%)		
Employed for an employer	1,904 (49.5%)	1,280 (49.4%)
Self-employed	360 (9.4%)	212 (8.2%)
Retired	846 (22.0%)	610 (23.6%)
Student	218 (5.7%)	115 (4.4%)
Homemaker	148 (3.9%)	85 (3.3%)
Unemployed and looking for a job	145 (3.8%)	104 (4.0%)
None of these/Other	218 (5.7%)	151 (5.8%)
(Missing)	4 (0.1%)	33 (1.3%)
Financially comfortable/getting by, n (%)		
Living comfortably on present income	1,748 (45.5%)	1,037 (40.0%)
Getting by on present income	1,409 (36.6%)	1,016 (39.2%)
Finding it difficult on present income	470 (12.2%)	396 (15.3%)
Finding it very difficult on present income	170 (4.4%)	128 (4.9%)
(Missing)	47 (1.2%)	13 (0.5%)
Own home, n (%)		
Someone in this household owns this home	2,392 (62.2%)	1,675 (64.7%)
Someone in this household rents this home	1,027 (26.7%)	601 (23.2%)
Both	171 (4.4%)	141 (5.4%)
Neither	215 (5.6%)	153 (5.9%)
Rent	0 (0%)	0 (0%)
Own	0 (0%)	0 (0%)
Something else	0 (0%)	0 (0%)
(Missing)	39 (1.0%)	21 (0.8%)
Religious/spiritual connection, n (%)		
Always	601 (15.6%)	387 (14.9%)
Often	805 (20.9%)	487 (18.8%)
Rarely	1,215 (31.6%)	829 (32.0%)
Never	1,220 (31.7%)	884 (34.1%)
(Missing)	3 (0.1%)	2 (0.1%)
Belief in life after death, n (%)		
Yes	1,392 (36.2%)	889 (34.3%)
No	1,196 (31.1%)	852 (32.9%)
Unsure	1,234 (32.1%)	823 (31.8%)
(Missing)	22 (0.6%)	25 (1.0%)
Transformative religious experience, n (%)		
Yes	966 (25.1%)	572 (22.1%)
No	2,864 (74.5%)	2,007 (77.5%)
(Missing)	14 (0.4%)	12 (0.4%)
Religious reading or listening, n (%)		
More than once a day	103 (2.7%)	83 (3.2%)
About once a day	290 (7.5%)	188 (7.3%)
Sometimes	1,038 (27.0%)	622 (24.0%)
Never	2,381 (61.9%)	1,678 (64.8%)
(Missing)	32 (0.8%)	19 (0.7%)
Prayer or meditation, n (%)		
More than once a day	317 (8.2%)	223 (8.6%)
About once a day	487 (12.7%)	286 (11.0%)

Table S10b. Weighted summary statistics for outcome variables in Australia

**Outcome**	**Wave 1** N = 3,844	**Wave 2** N = 2,590
Sometimes	1,382 (35.9%)	899 (34.7%)
Never	1,642 (42.7%)	1,168 (45.1%)
(Missing)	17 (0.4%)	14 (0.5%)
Belief in God/gods/spiritual forces, n (%)		
One God	1,243 (32.3%)	805 (31.1%)
More than one god	65 (1.7%)	51 (2.0%)
An impersonal spiritual force	741 (19.3%)	480 (18.5%)
None of these	1,170 (30.4%)	874 (33.7%)
Unsure	613 (15.9%)	371 (14.3%)
(Missing)	12 (0.3%)	10 (0.4%)
Religious centrality, n (%)		
Agree	961 (25.0%)	611 (23.6%)
Disagree	724 (18.8%)	417 (16.1%)
Not relevant	1,812 (47.1%)	1,354 (52.3%)
Unsure	335 (8.7%)	200 (7.7%)
(Missing)	13 (0.3%)	9 (0.3%)
Religious/spiritual comfort, n (%)		
Agree	1,249 (32.5%)	774 (29.9%)
Disagree	524 (13.6%)	310 (12.0%)
Not relevant	1,771 (46.1%)	1,293 (49.9%)
Unsure	290 (7.5%)	208 (8.0%)
(Missing)	10 (0.3%)	4 (0.2%)
Feel loved by God, n (%)		
Agree	1,105 (28.7%)	705 (27.2%)
Disagree	543 (14.1%)	346 (13.4%)
Not relevant	1,764 (45.9%)	1,261 (48.7%)
Unsure	422 (11.0%)	265 (10.2%)
(Missing)	10 (0.3%)	13 (0.5%)
Feel punished by God, n (%)		
Agree	165 (4.3%)	110 (4.2%)
Disagree	1,794 (46.7%)	1,126 (43.5%)
Not relevant	1,624 (42.3%)	1,180 (45.6%)
Unsure	249 (6.5%)	168 (6.5%)
(Missing)	12 (0.3%)	5 (0.2%)
Experienced religious criticism, n (%)		
Agree	185 (4.8%)	118 (4.5%)
Disagree	971 (25.3%)	632 (24.4%)
Not relevant	2,418 (62.9%)	1,690 (65.3%)
Unsure	260 (6.8%)	142 (5.5%)
(Missing)	10 (0.3%)	8 (0.3%)
Faith-sharing, n (%)		
Agree	883 (23.0%)	595 (23.0%)
Disagree	855 (22.2%)	568 (21.9%)
Not relevant	1,925 (50.1%)	1,321 (51.0%)
Unsure	176 (4.6%)	98 (3.8%)
(Missing)	5 (0.1%)	8 (0.3%)

Note. N (%); this table is based on non-imputed data. Cumulative percentages for variables may not add up to 100% due to rounding. Wave 1 characteristics weighted using the Gallup provided sampling weight, ANNUAL_WEIGHT_R2; Wave 2 characteristics weighted accounting for attrition by using the adjusted Wave 1 weight, ANNUAL_WEIGHT_R2, multiplied by the created attrition weight to account for dropout, to maintain nationally representative estimates for Wave 2 characteristics.

Table S10c. Unweighted summary statistics for demographic and childhood variables in Australia by retention status

Characteristic	Attritors–Not Observed in Wave 2<br>N = 1,471	Retained–Observed in Wave 2<br>N = 2,383
Forgivingness, n (%)		
Always	239 (16.3%)	391 (16.4%)
Often	919 (62.5%)	1,460 (61.3%)
Rarely	265 (18.0%)	482 (20.2%)
Never	43 (3.0%)	46 (1.9%)
(Missing)	4 (0.3%)	4 (0.2%)
Year of birth, n (%)		
1943 or earlier (current age: 80+ years)	57 (3.9%)	111 (4.6%)
1943-1953 (current age: 70-79 years)	153 (10.4%)	308 (12.9%)
1953-1963 (current age: 60-69 years)	152 (10.3%)	383 (16.1%)
1963-1973 (current age: 50-59 years)	223 (15.2%)	438 (18.4%)
1973-1983 (current age: 40-49 years)	235 (16.0%)	373 (15.7%)
1983-1993 (current age: 30-39 years)	272 (18.5%)	403 (16.9%)
1993-1998 (current age: 25-29 years)	139 (9.5%)	174 (7.3%)
1998-2005 (current age: 18-24 years)	238 (16.2%)	193 (8.1%)
(Missing)	1 (0.1%)	0 (0.0%)
Age of participant		
Mean	45.1	50.4
Standard Deviation	18.7	17.8
Min, Max	18.0, 95.0	18.0, 94.0
Gender, n (%)		
Male	722 (49.1%)	1,161 (48.7%)
Female	729 (49.6%)	1,196 (50.2%)
Other	17 (1.1%)	23 (1.0%)
(Missing)	3 (0.2%)	3 (0.1%)
Respondent marital status, n (%)		
Single/Never been married	432 (29.4%)	518 (21.7%)
Married	565 (38.4%)	1,150 (48.3%)
Separated	65 (4.4%)	92 (3.9%)
Divorced	107 (7.3%)	213 (8.9%)
Widowed	73 (4.9%)	135 (5.6%)
Domestic partner	203 (13.8%)	265 (11.1%)
(Missing)	26 (1.8%)	11 (0.5%)
Education (years), n (%)		
Up to 8	31 (2.1%)	43 (1.8%)
9-15	1,006 (68.4%)	1,527 (64.1%)
16+	430 (29.2%)	804 (33.7%)
(Missing)	3 (0.2%)	8 (0.4%)
Employment status, n (%)		
Employed for an employer	753 (51.2%)	1,155 (48.5%)
Self-employed	122 (8.3%)	240 (10.1%)
Retired	259 (17.6%)	591 (24.8%)
Student	114 (7.8%)	103 (4.3%)
Homemaker	68 (4.6%)	81 (3.4%)
Unemployed and looking for a job	69 (4.7%)	77 (3.2%)
None of these/Other	85 (5.8%)	133 (5.6%)
(Missing)	1 (0.1%)	3 (0.1%)
Current religious service attendance, n (%)		
More than once a week	59 (4.0%)	105 (4.4%)

Table S10c. Unweighted summary statistics for demographic and childhood variables in Australia by retention status

**Characteristic**	**Attritors–Not Observed in Wave 2** N = 1,471	**Retained–Observed in Wave 2** N = 2,383
Once a week	106 (7.2%)	186 (7.8%)
One to three times a month	60 (4.1%)	72 (3.0%)
A few times a year	272 (18.5%)	385 (16.2%)
Never	973 (66.1%)	1,629 (68.3%)
(Missing)	0 (0.0%)	7 (0.3%)
Immigration status, n (%)		
Born in this country	1,119 (76.1%)	1,859 (78.0%)
Born in another country	351 (23.9%)	517 (21.7%)
(Missing)	0 (<0.0%)	7 (0.3%)
Parental marital status around age 12, n (%)		
Parents were married	1,100 (74.8%)	1,920 (80.6%)
Parents were divorced	188 (12.8%)	292 (12.2%)
Parents were never married	113 (7.7%)	95 (4.0%)
One or both of them had died	32 (2.2%)	60 (2.5%)
Unsure	6 (0.4%)	6 (0.3%)
(Missing)	31 (2.1%)	9 (0.4%)
Religious service attendance around age 12, n (%)		
At least once a week	461 (31.3%)	872 (36.6%)
One to three times a month	175 (11.9%)	307 (12.9%)
Less than once a month	249 (16.9%)	361 (15.1%)
Never	532 (36.2%)	804 (33.7%)
(Missing)	54 (3.7%)	39 (1.6%)
Relationship with mother when growing up, n (%)		
Very good	977 (66.4%)	1,580 (66.3%)
Somewhat good	346 (23.6%)	579 (24.3%)
Somewhat bad	85 (5.8%)	136 (5.7%)
Very bad	51 (3.5%)	60 (2.5%)
(Does not apply)	8 (0.5%)	25 (1.1%)
(Missing)	4 (0.2%)	3 (0.1%)
Relationship with father when growing up, n (%)		
Very good	759 (51.6%)	1,258 (52.8%)
Somewhat good	440 (29.9%)	707 (29.7%)
Somewhat bad	125 (8.5%)	195 (8.2%)
Very bad	81 (5.5%)	128 (5.4%)
(Does not apply)	61 (4.2%)	90 (3.8%)
(Missing)	5 (0.4%)	4 (0.2%)
Felt like an outsider in family when growing up, n (%)		
Yes	319 (21.7%)	460 (19.3%)
No	1,143 (77.7%)	1,904 (79.9%)
(Missing)	9 (0.6%)	19 (0.8%)
Experienced abuse when growing up, n (%)		
Yes	368 (25.1%)	633 (26.6%)
No	1,063 (72.3%)	1,730 (72.6%)
(Missing)	39 (2.6%)	20 (0.8%)
Self-rated health when growing up, n (%)		
Excellent	622 (42.3%)	1,073 (45.0%)
Very good	417 (28.3%)	674 (28.3%)

Table S10c. Unweighted summary statistics for demographic and childhood variables in Australia by retention status

**Characteristic**	**Attritors–Not Observed in Wave 2** N = 1,471	**Retained–Observed in Wave 2** N = 2,383
Good	243 (16.5%)	379 (15.9%)
Fair	128 (8.7%)	202 (8.5%)
Poor	58 (3.9%)	54 (2.3%)
(Missing)	3 (0.2%)	1 (0.0%)
Subjective financial status of family growing up, n (%)		
Lived comfortably	667 (45.3%)	1,083 (45.4%)
Got by	557 (37.9%)	942 (39.5%)
Found it difficult	175 (11.9%)	259 (10.9%)
Found it very difficult	61 (4.1%)	95 (4.0%)
(Missing)	11 (0.7%)	5 (0.2%)
Religious affiliation growing up, n (%)		
Christianity	953 (64.8%)	1,680 (70.5%)
Taoism	0 (0%)	1 (0.0%)
Confucianism	0 (0%)	0 (0%)
Primal, Animist, or Folk religion	3 (0.2%)	1 (0.0%)
Spiritism	0 (0%)	0 (0%)
Umbanda, Candomblé, and other African-derived religions	0 (0%)	0 (0%)
Chinese folk/traditional religion	0 (0%)	0 (0%)
Islam	24 (1.6%)	29 (1.2%)
Hinduism	24 (1.6%)	16 (0.7%)
Buddhism	15 (1.0%)	4 (0.2%)
Judaism	18 (1.2%)	10 (0.4%)
Sikhism	7 (0.5%)	0 (0%)
Baha'i	2 (0.2%)	2 (0.1%)
Jainism	0 (0%)	0 (0%)
Shinto	0 (0%)	0 (0%)
Some other religion	3 (0.2%)	6 (0.3%)
No religion/Atheist/Agnostic	414 (28.1%)	623 (26.1%)
(Missing)	9 (0.6%)	12 (0.5%)

Note. N (%); this table is based on non-imputed data. Cumulative percentages for variables may not add up to 100% due to rounding.

Table S10d. Unweighted summary statistics for Wave 1 outcome variables in Australia by retention status.

**Outcome**	**Attritors–Not Observed in Wave 2** N = 1,471	**Retained–Observed in Wave 2** N = 2,383
Secure flourishing index		
Mean	6.8	7.0
Standard Deviation	1.6	1.6
Min, Max	1.1, 10.0	1.0, 10.0
(Missing)	23 (1.6%)	15 (0.6%)
Flourishing index		
Mean	6.9	7.0
Standard Deviation	1.6	1.6
Min, Max	1.2, 10.0	1.2, 10.0
(Missing)	20 (1.3%)	13 (0.6%)
Happiness & life satisfaction		
Mean	6.6	6.8
Standard Deviation	1.9	1.9
Min, Max	0.0, 10.0	0.0, 10.0
(Missing)	5 (0.3%)	5 (0.2%)
Physical & mental health		
Mean	6.5	6.6
Standard Deviation	1.9	1.9
Min, Max	0.0, 10.0	0.0, 10.0
(Missing)	5 (0.4%)	2 (<0.1%)
Meaning & purpose		
Mean	6.8	7.0
Standard Deviation	2.2	2.1
Min, Max	0.0, 10.0	0.0, 10.0
(Missing)	3 (0.2%)	2 (<0.1%)
Character & virtue		
Mean	7.4	7.5
Standard Deviation	1.5	1.5
Min, Max	2.0, 10.0	0.0, 10.0
(Missing)	8 (0.6%)	1 (<0.1%)
Close social relationships		
Mean	6.9	7.0
Standard Deviation	2.3	2.3
Min, Max	0.0, 10.0	0.0, 10.0
(Missing)	4 (0.3%)	4 (0.1%)
Financial & material security		
Mean	6.5	7.1
Standard Deviation	2.9	2.7
Min, Max	0.0, 10.0	0.0, 10.0
(Missing)	4 (0.3%)	1 (<0.1%)
Happiness		
Mean	6.7	6.9
Standard Deviation	1.9	1.8
Min, Max	0.0, 10.0	0.0, 10.0
(Missing)	3 (0.2%)	2 (<0.1%)
Life satisfaction		
Mean	6.5	6.8
Standard Deviation	2.2	2.1
Min, Max	0.0, 10.0	0.0, 10.0

Table S10d. Unweighted summary statistics for Wave 1 outcome variables in Australia by retention status.

**Outcome**	**Attritors–Not Observed in Wave 2** N = 1,471	**Retained–Observed in Wave 2** N = 2,383
(Missing)	5 (0.3%)	4 (0.2%)
Current life evaluation		
Mean	6.5	6.9
Standard Deviation	1.8	1.7
Min, Max	0.0, 10.0	0.0, 10.0
(Missing)	1 (<0.1%)	2 (<0.1%)
Future life evaluation		
Mean	7.6	7.6
Standard Deviation	1.9	1.8
Min, Max	0.0, 10.0	0.0, 10.0
(Missing)	3 (0.2%)	3 (0.1%)
Optimism		
Mean	7.2	7.4
Standard Deviation	2.4	2.3
Min, Max	0.0, 10.0	0.0, 10.0
(Missing)	22 (1.5%)	29 (1.2%)
Freedom to pursue what's important		
Mean	6.9	7.2
Standard Deviation	2.7	2.5
Min, Max	0.0, 10.0	0.0, 10.0
Inner peace, n (%)		
Always	155 (10.5%)	293 (12.3%)
Often	949 (64.5%)	1,557 (65.3%)
Rarely	341 (23.2%)	486 (20.4%)
Never	23 (1.6%)	40 (1.7%)
(Missing)	2 (0.2%)	7 (0.3%)
Life balance, n (%)		
Always	74 (5.1%)	164 (6.9%)
Often	914 (62.1%)	1,517 (63.7%)
Rarely	450 (30.6%)	640 (26.9%)
Never	31 (2.1%)	59 (2.5%)
(Missing)	2 (0.1%)	3 (0.1%)
Sense of mastery, n (%)		
Always	183 (12.4%)	355 (14.9%)
Often	1,027 (69.8%)	1,671 (70.1%)
Rarely	230 (15.6%)	326 (13.7%)
Never	13 (0.9%)	21 (0.9%)
(Missing)	18 (1.2%)	10 (0.4%)
Meaningful activities		
Mean	7.1	7.2
Standard Deviation	2.2	2.1
Min, Max	0.0, 10.0	0.0, 10.0
(Missing)	1 (<0.1%)	0 (<0.1%)
Understanding purpose		
Mean	6.6	6.8
Standard Deviation	2.6	2.6
Min, Max	0.0, 10.0	0.0, 10.0
(Missing)	1 (<0.1%)	1 (<0.1%)
Self-rated mental health		

Table S10d. Unweighted summary statistics for Wave 1 outcome variables in Australia by retention status.

**Outcome**	**Attritors–Not Observed in Wave 2** N = 1,471	**Retained–Observed in Wave 2** N = 2,383
Mean	6.6	6.8
Standard Deviation	2.3	2.3
Min, Max	0.0, 10.0	0.0, 10.0
(Missing)	3 (0.2%)	1 (<0.1%)
Traumatic distress, n (%)		
A lot	148 (10.1%)	184 (7.7%)
Some	340 (23.1%)	497 (20.9%)
Not very much	445 (30.3%)	736 (30.9%)
Not at all	535 (36.4%)	965 (40.5%)
(Missing)	2 (0.1%)	1 (0.0%)
Depression symptoms composite, n (%)	390 (26.6%)	588 (24.7%)
(Missing)	4 (0.3%)	2 (<0.1%)
Depression – feel hopeless, n (%)		
Nearly every day	143 (9.7%)	174 (7.3%)
More than half the days	139 (9.4%)	251 (10.5%)
Several days	515 (35.0%)	803 (33.7%)
Not at all	672 (45.7%)	1,154 (48.4%)
(Missing)	2 (0.2%)	1 (0.1%)
Depression – loss of interest, n (%)		
Nearly every day	128 (8.7%)	177 (7.4%)
More than half the days	231 (15.7%)	338 (14.2%)
Several days	518 (35.2%)	811 (34.0%)
Not at all	592 (40.2%)	1,056 (44.3%)
(Missing)	2 (0.1%)	1 (0.0%)
Anxiety symptoms composite, n (%)	392 (26.7%)	536 (22.6%)
(Missing)	1 (<0.1%)	10 (0.4%)
Anxiety – feel on edge, n (%)		
Nearly every day	178 (12.1%)	208 (8.7%)
More than half the days	172 (11.7%)	278 (11.7%)
Several days	624 (42.5%)	1,004 (42.1%)
Not at all	497 (33.8%)	887 (37.2%)
(Missing)	0 (0.0%)	6 (0.2%)
Anxiety – cannot stop worrying, n (%)		
Nearly every day	165 (11.2%)	169 (7.1%)
More than half the days	160 (10.9%)	243 (10.2%)
Several days	482 (32.8%)	841 (35.3%)
Not at all	663 (45.1%)	1,126 (47.3%)
(Missing)	1 (0.0%)	5 (0.2%)
Suffering, n (%)		
A lot	219 (14.9%)	282 (11.8%)
Some	557 (37.9%)	911 (38.2%)
Not very much	497 (33.8%)	841 (35.3%)
Not at all	189 (12.8%)	345 (14.5%)
(Missing)	8 (0.6%)	5 (0.2%)
Relationship contentment		
Mean	7.1	7.3
Standard Deviation	2.4	2.3
Min, Max	0.0, 10.0	0.0, 10.0
(Missing)	2 (0.2%)	3 (0.1%)

Table S10d. Unweighted summary statistics for Wave 1 outcome variables in Australia by retention status.

**Outcome**	**Attritors–Not Observed in Wave 2** N = 1,471	**Retained–Observed in Wave 2** N = 2,383
Relationship satisfaction		
Mean	6.8	6.8
Standard Deviation	2.5	2.5
Min, Max	0.0, 10.0	0.0, 10.0
(Missing)	2 (0.1%)	1 (<0.1%)
Social support		
Mean	7.9	8.2
Standard Deviation	2.4	2.2
Min, Max	0.0, 10.0	0.0, 10.0
(Missing)	7 (0.5%)	3 (0.1%)
Intimate/close friend, n (%)		
Yes	1,169 (79.5%)	2,027 (85.1%)
No	245 (16.7%)	322 (13.5%)
(Missing)	57 (3.8%)	34 (1.4%)
Government approval, n (%)		
Strongly approve	106 (7.2%)	236 (9.9%)
Somewhat approve	446 (30.3%)	811 (34.0%)
Neither approve nor disapprove	422 (28.7%)	570 (23.9%)
Somewhat disapprove	329 (22.4%)	501 (21.0%)
Strongly disapprove	160 (10.9%)	257 (10.8%)
(Missing)	7 (0.5%)	10 (0.4%)
Say in government, n (%)		
Agree	504 (34.3%)	935 (39.2%)
Disagree	631 (42.9%)	951 (39.9%)
Unsure	328 (22.3%)	493 (20.7%)
(Missing)	7 (0.5%)	4 (0.2%)
Belonging in country		
Mean	7.6	7.8
Standard Deviation	2.4	2.2
Min, Max	0.0, 10.0	0.0, 10.0
(Missing)	23 (1.5%)	9 (0.4%)
City/place satisfaction, n (%)		
Satisfied	1,148 (78.1%)	1,987 (83.4%)
Dissatisfied	170 (11.5%)	244 (10.2%)
Unsure	100 (6.8%)	114 (4.8%)
(Missing)	52 (3.6%)	38 (1.6%)
Trust within country, n (%)		
All people	6 (0.4%)	15 (0.6%)
Most people	497 (33.8%)	932 (39.1%)
Some people	660 (44.9%)	1,077 (45.2%)
Not very many people	291 (19.8%)	342 (14.4%)
None	3 (0.2%)	3 (0.1%)
(Missing)	13 (0.9%)	14 (0.6%)
Number of children		
Mean	0.6	0.5
Standard Deviation	1.0	1.0
Min, Max	0.0, 6.0	0.0, 6.0
(Missing)	3 (0.2%)	2 (<0.1%)
Community participation, n (%)		

Table S10d. Unweighted summary statistics for Wave 1 outcome variables in Australia by retention status.

Outcome	Attritors–Not Observed in Wave 2<br>N = 1,471	Retained–Observed in Wave 2<br>N = 2,383
More than once a week	187 (12.7%)	379 (15.9%)
Once a week	174 (11.9%)	335 (14.1%)
One to three times a month	171 (11.6%)	291 (12.2%)
A few times a year	340 (23.2%)	566 (23.7%)
Never	595 (40.5%)	809 (34.0%)
(Missing)	3 (0.2%)	3 (0.1%)
Religious attendance, n (%)		
More than once a week	59 (4.0%)	105 (4.4%)
Once a week	106 (7.2%)	186 (7.8%)
One to three times a month	60 (4.1%)	72 (3.0%)
A few times a year	272 (18.5%)	385 (16.2%)
Never	973 (66.1%)	1,629 (68.3%)
(Missing)	0 (0.0%)	7 (0.3%)
Loneliness		
Mean	3.6	3.3
Standard Deviation	2.8	2.8
Min, Max	0.0, 10.0	0.0, 10.0
Perceived discrimination, n (%)		
Always	47 (3.2%)	72 (3.0%)
Often	232 (15.8%)	351 (14.7%)
Rarely	793 (54.0%)	1,225 (51.4%)
Never	396 (26.9%)	732 (30.7%)
(Missing)	2 (0.2%)	3 (0.1%)
Orientation to promote good		
Mean	7.8	7.9
Standard Deviation	1.7	1.6
Min, Max	0.0, 10.0	0.0, 10.0
(Missing)	5 (0.3%)	1 (<0.1%)
Delayed gratification		
Mean	7.1	7.2
Standard Deviation	2.0	2.0
Min, Max	0.0, 10.0	0.0, 10.0
(Missing)	4 (0.2%)	0 (<0.1%)
Hope		
Mean	7.7	7.6
Standard Deviation	2.1	2.0
Min, Max	0.0, 10.0	0.0, 10.0
(Missing)	59 (4.0%)	93 (3.9%)
Gratitude		
Mean	7.7	7.8
Standard Deviation	2.4	2.2
Min, Max	0.0, 10.0	0.0, 10.0
(Missing)	60 (4.1%)	88 (3.7%)
Showing love/care		
Mean	8.2	8.3
Standard Deviation	1.8	1.8
Min, Max	0.0, 10.0	0.0, 10.0
Forgivingness, n (%)		
Always	239 (16.3%)	391 (16.4%)

Table S10d. Unweighted summary statistics for Wave 1 outcome variables in Australia by retention status.

**Outcome**	**Attritors–Not Observed in Wave 2** N = 1,471	**Retained–Observed in Wave 2** N = 2,383
Often	919 (62.5%)	1,460 (61.3%)
Rarely	265 (18.0%)	482 (20.2%)
Never	43 (3.0%)	46 (1.9%)
(Missing)	4 (0.3%)	4 (0.2%)
Charitable giving, n (%)		
Yes	751 (51.1%)	1,255 (52.7%)
No	717 (48.7%)	1,122 (47.1%)
(Missing)	3 (0.2%)	6 (0.3%)
Helping strangers, n (%)		
Yes	905 (61.6%)	1,457 (61.1%)
No	557 (37.9%)	899 (37.7%)
(Missing)	8 (0.5%)	27 (1.1%)
Volunteering, n (%)		
Yes	420 (28.6%)	840 (35.2%)
No	1,050 (71.4%)	1,541 (64.6%)
(Missing)	0 (0%)	3 (0.1%)
Self-rated physical health		
Mean	6.4	6.5
Standard Deviation	2.1	2.1
Min, Max	0.0, 10.0	0.0, 10.0
(Missing)	3 (0.2%)	2 (<0.1%)
Health problems, n (%)		
Yes	390 (26.5%)	668 (28.0%)
No	1,060 (72.1%)	1,694 (71.1%)
(Missing)	20 (1.4%)	21 (0.9%)
Pain in past 4 weeks, n (%)		
A lot	238 (16.2%)	380 (16.0%)
Some	574 (39.0%)	966 (40.5%)
Not very much	464 (31.6%)	786 (33.0%)
None at all	190 (12.9%)	248 (10.4%)
(Missing)	5 (0.3%)	4 (0.2%)
Number of cigarettes per day		
Mean	1.8	1.1
Standard Deviation	5.3	4.2
Min, Max	0.0, 36.0	0.0, 40.0
(Missing)	18 (1.2%)	24 (1.0%)
Number of drinks per week		
Mean	4.6	4.6
Standard Deviation	8.3	8.5
Min, Max	0.0, 97.0	0.0, 97.0
(Missing)	16 (1.1%)	13 (0.5%)
Days exercise per week		
Mean	2.5	2.7
Standard Deviation	2.3	2.3
Min, Max	0.0, 7.0	0.0, 7.0
(Missing)	2 (0.1%)	1 (<0.1%)
Financial security		
Mean	6.2	6.8
Standard Deviation	3.1	2.9

Table S10d. Unweighted summary statistics for Wave 1 outcome variables in Australia by retention status.

**Outcome**	**Attritors–Not Observed in Wave 2** N = 1,471	**Retained–Observed in Wave 2** N = 2,383
Min, Max	0.0, 10.0	0.0, 10.0
(Missing)	0 (<0.1%)	1 (<0.1%)
Material security		
Mean	6.9	7.4
Standard Deviation	2.9	2.7
Min, Max	0.0, 10.0	0.0, 10.0
(Missing)	4 (0.3%)	0 (0%)
Educational attainment (16+ years), n (%)		
Up to 8	31 (2.1%)	43 (1.8%)
9-15	1,006 (68.4%)	1,527 (64.1%)
16+	430 (29.2%)	804 (33.7%)
(Missing)	3 (0.2%)	8 (0.4%)
Currently employed, n (%)		
Employed for an employer	753 (51.2%)	1,155 (48.5%)
Self-employed	122 (8.3%)	240 (10.1%)
Retired	259 (17.6%)	591 (24.8%)
Student	114 (7.8%)	103 (4.3%)
Homemaker	68 (4.6%)	81 (3.4%)
Unemployed and looking for a job	69 (4.7%)	77 (3.2%)
None of these/Other	85 (5.8%)	133 (5.6%)
(Missing)	1 (0.1%)	3 (0.1%)
Financially comfortable/getting by, n (%)		
Living comfortably on present income	577 (39.2%)	1,178 (49.4%)
Getting by on present income	573 (39.0%)	838 (35.2%)
Finding it difficult on present income	194 (13.2%)	277 (11.6%)
Finding it very difficult on present income	92 (6.2%)	78 (3.3%)
(Missing)	35 (2.4%)	12 (0.5%)
Own home, n (%)		
Someone in this household owns this home	814 (55.4%)	1,586 (66.6%)
Someone in this household rents this home	457 (31.1%)	571 (24.0%)
Both	63 (4.3%)	108 (4.5%)
Neither	104 (7.1%)	111 (4.6%)
Rent	0 (0%)	0 (0%)
Own	0 (0%)	0 (0%)
Something else	0 (0%)	0 (0%)
(Missing)	32 (2.1%)	7 (0.3%)
Religious/spiritual connection, n (%)		
Always	222 (15.1%)	381 (16.0%)
Often	345 (23.5%)	461 (19.3%)
Rarely	451 (30.7%)	768 (32.2%)
Never	452 (30.8%)	771 (32.4%)
(Missing)	1 (0.0%)	2 (0.1%)
Belief in life after death, n (%)		
Yes	556 (37.8%)	839 (35.2%)
No	411 (27.9%)	790 (33.1%)
Unsure	489 (33.2%)	748 (31.4%)
(Missing)	15 (1.0%)	7 (0.3%)
Transformative religious experience, n (%)		
Yes	416 (28.3%)	552 (23.1%)

Table S10d. Unweighted summary statistics for Wave 1 outcome variables in Australia by retention status.

**Outcome**	**Attritors–Not Observed in Wave 2** N = 1,471	**Retained–Observed in Wave 2** N = 2,383
No	1,051 (71.5%)	1,821 (76.4%)
(Missing)	4 (0.3%)	10 (0.4%)
Religious reading or listening, n (%)		
More than once a day	44 (3.0%)	59 (2.5%)
About once a day	108 (7.3%)	183 (7.7%)
Sometimes	426 (29.0%)	614 (25.7%)
Never	879 (59.8%)	1,509 (63.3%)
(Missing)	14 (1.0%)	18 (0.8%)
Prayer or meditation, n (%)		
More than once a day	123 (8.3%)	195 (8.2%)
About once a day	201 (13.6%)	287 (12.1%)
Sometimes	498 (33.9%)	888 (37.2%)
Never	644 (43.8%)	1,001 (42.0%)
(Missing)	5 (0.3%)	12 (0.5%)
Belief in God/gods/spiritual forces, n (%)		
One God	480 (32.7%)	766 (32.1%)
More than one god	26 (1.8%)	38 (1.6%)
An impersonal spiritual force	295 (20.1%)	448 (18.8%)
None of these	428 (29.1%)	745 (31.3%)
Unsure	232 (15.8%)	382 (16.0%)
(Missing)	8 (0.6%)	4 (0.2%)
Religious centrality, n (%)		
Agree	362 (24.6%)	602 (25.2%)
Disagree	272 (18.5%)	453 (19.0%)
Not relevant	687 (46.7%)	1,129 (47.4%)
Unsure	144 (9.8%)	192 (8.0%)
(Missing)	6 (0.4%)	7 (0.3%)
Religious/spiritual comfort, n (%)		
Agree	499 (34.0%)	752 (31.6%)
Disagree	187 (12.7%)	339 (14.2%)
Not relevant	652 (44.3%)	1,124 (47.2%)
Unsure	127 (8.6%)	164 (6.9%)
(Missing)	5 (0.4%)	5 (0.2%)
Feel loved by God, n (%)		
Agree	443 (30.2%)	664 (27.8%)
Disagree	211 (14.4%)	333 (14.0%)
Not relevant	656 (44.6%)	1,113 (46.7%)
Unsure	151 (10.3%)	272 (11.4%)
(Missing)	8 (0.6%)	2 (0.1%)
Feel punished by God, n (%)		
Agree	86 (5.9%)	78 (3.3%)
Disagree	660 (44.9%)	1,139 (47.8%)
Not relevant	606 (41.2%)	1,023 (42.9%)
Unsure	110 (7.5%)	139 (5.8%)
(Missing)	8 (0.6%)	4 (0.2%)
Experienced religious criticism, n (%)		
Agree	87 (5.9%)	98 (4.1%)
Disagree	376 (25.6%)	597 (25.1%)
Not relevant	889 (60.4%)	1,536 (64.5%)

Table S10d. Unweighted summary statistics for Wave 1 outcome variables in Australia by retention status.

Outcome	Attritors–Not Observed in Wave 2<br>N = 1,471	Retained–Observed in Wave 2<br>N = 2,383
Unsure	109 (7.4%)	151 (6.3%)
(Missing)	9 (0.6%)	1 (0.0%)
Faith-sharing, n (%)		
Agree	367 (24.9%)	518 (21.7%)
Disagree	308 (21.0%)	549 (23.0%)
Not relevant	712 (48.4%)	1,219 (51.2%)
Unsure	80 (5.4%)	96 (4.0%)
(Missing)	4 (0.3%)	1 (0.0%)

Note. N (%); this table is based on non-imputed data. Cumulative percentages for variables may not add up to 100% due to rounding.

Table S10e. Summary of fitted attrition model in Australia

Characteristic	Odds Ratio	95% CI	p-value
**ANNUAL_WEIGHT_R2**	0.79	0.67, 0.92	0.003
**Happiness & life satisfaction**	1.10	0.91, 1.32	0.331
**Physical & mental health**	0.92	0.78, 1.08	0.288
**Meaning & purpose**	0.98	0.83, 1.15	0.790
**Character & virtue**	0.94	0.84, 1.06	0.314
**Close social relationships**	0.92	0.80, 1.06	0.262
**Financial & material security**	1.17	1.04, 1.32	0.009
**Extraversion**	0.90	0.81, 0.99	0.027
**Openness to experience**	1.06	0.96, 1.17	0.227
**Agreeableness**	1.11	1.00, 1.23	0.053
**Conscientiousness**	1.13	1.01, 1.26	0.038
**Neuroticism**	0.95	0.84, 1.07	0.367
**Depression symptoms composite**	1.08	0.95, 1.23	0.241
**Anxiety symptoms composite**	0.96	0.84, 1.09	0.494
**Loneliness**	0.98	0.85, 1.12	0.758
**Days exercise per week**	1.09	0.99, 1.20	0.076
**Year of birth (age group)**			
1953-1963 (current age: 60-69 years)	—	—	
1963-1973 (current age: 50-59 years)	0.92	0.68, 1.26	0.615
1943-1953 (current age: 70-79 years)	0.75	0.53, 1.07	0.114
1973-1983 (current age: 40-49 years)	0.92	0.64, 1.32	0.640
1983-1993 (current age: 30-39 years)	1.03	0.67, 1.58	0.903
1943 or earlier (current age: 80+ years)	0.75	0.46, 1.25	0.272
1998-2005 (current age: 18-24 years)	0.75	0.43, 1.32	0.318
1993-1998 (current age: 25-29 years)	1.04	0.60, 1.82	0.882
**Gender of respondent**			
Female	—	—	
Male	1.01	0.83, 1.24	0.892
Other	1.31	0.47, 3.67	0.611
**Marital status**			
Married	—	—	
Single/Never been married	0.85	0.63, 1.13	0.262
Divorced	0.96	0.68, 1.35	0.813
Domestic partner	0.79	0.58, 1.08	0.143
Widowed	0.83	0.55, 1.26	0.388
Separated	0.90	0.56, 1.45	0.675
**Employment status**			
Employed for an employer	—	—	
Retired	1.38	0.96, 1.96	0.080
Self-employed	1.14	0.83, 1.58	0.425
None of these/Other	1.07	0.67, 1.70	0.790
Student	0.96	0.57, 1.63	0.893
Unemployed and looking for a job	0.92	0.52, 1.61	0.758
Homemaker	0.77	0.46, 1.29	0.324
**Religious attendance**			
Never	—	—	
A few times a year	0.87	0.69, 1.11	0.273
Once a week	0.99	0.70, 1.40	0.968
More than once a week	1.00	0.63, 1.58	0.998
One to three times a month	0.65	0.42, 1.03	0.066
**Educational attainment (16+ years)**			
16+	—	—	

Table S10e. Summary of fitted attrition model in Australia

Characteristic	Odds Ratio	95% CI	p-value
9-15	1.06	0.82, 1.37	0.654
Up to 8	0.90	0.39, 2.10	0.816
**Born in This country**			
Born in this country	—	—	
Born in another country	0.94	0.72, 1.22	0.625
**Race plurality (prominent race/ethnic group [0] or not [1])**	0.91	0.82, 1.02	0.107
**Urbanicity**			
A suburb of a large city	—	—	
A small town or village	1.31	1.01, 1.71	0.044
A large city	1.01	0.76, 1.34	0.947
A rural area or on a farm	1.00	0.71, 1.40	0.995
**Annual household income**			
Australia: 100,001 – 150,000 dollars	—	—	
Australia: 150,001 – 250,000 dollars	1.29	0.94, 1.77	0.116
Australia: 75,001 – 100,000 dollars	1.02	0.73, 1.41	0.912
Australia: 20,001 – 30,000 dollars	1.04	0.69, 1.59	0.844
Australia: 60,001 – 75,000 dollars	1.27	0.85, 1.89	0.246
Australia: More than 250,000 dollars	1.08	0.73, 1.61	0.700
Australia: 30,001 – 40,000 dollars	0.81	0.54, 1.23	0.330
Australia: 40,001 – 50,000 dollars	0.85	0.54, 1.33	0.472
Australia: 50,001 – 60,000 dollars	1.05	0.67, 1.64	0.829
Australia: 20,000 dollars or less	1.17	0.73, 1.88	0.508
(None/No household income)	0.88	0.46, 1.65	0.685

Abbreviations: CI = Confidence Interval, OR = Odds Ratio

Notes. N=3844; attrition weights were estimated using the 'survey::svyglm(family=quasibinomial('logit'))' function. All continuous predictors were standardized and all categorical predictors used the most common category as the reference group. Reported p-values are based on the fitted regression model and no adjustments for multiple testing were done within this table.

Table S10f. Summary of principal components in Australia

PC	Percent Explained by each PC	Cumulative Percent Explained
1	34.94	34.94
2	7.69	42.64
3	7.66	50.29
4	2.40	52.69
5	1.84	54.53
6	1.68	56.21
**7**	**1.67**	**57.88**
8	1.57	59.44
9	1.42	60.87
10	1.40	62.27
11	1.31	63.57
12	1.25	64.82
13	1.19	66.01
14	1.14	67.15
15	1.07	68.22
16	1.05	69.27
17	1.02	70.29
18	0.96	71.25
19	0.95	72.20
20	0.93	73.13

Notes. N=3844; PCA was conducted using 'survey::svyprcomp(.)' function using all available contemporaneous exposures at wave 1. All PCs were standardized prior to being used as predictors. The bolded row represented the number of retained components for analysis was 7.

Table S10g. Associations of forgivingness with adult well-being and other outcomes at Wave 2 in Australia

	Model 1: Demographic and Childhood Variables as Covariates					Model 2: Demographic, Childhood, and Other Wave 1 Confounding Variables (Via Principal Components) as Covariates				
Outcome	RR	ES	95% CI	SE	p-value	RR	ES	95% CI	SE	p-value
Human Flourishing										
Secure flourishing index		0.06	(0.02,0.10)	0.02	1.84e-03**		0.01	(-0.02,0.04)	0.02	0.620
Flourishing index		0.07	(0.03,0.11)	0.02	6.75e-04**		0.01	(-0.02,0.04)	0.02	0.628
Happiness & life satisfaction		0.02	(-0.02,0.07)	0.02	0.272		-0.02	(-0.06,0.01)	0.02	0.190
Physical & mental health		0.03	(-0.01,0.07)	0.02	0.176		-0.01	(-0.04,0.03)	0.02	0.736
Meaning & purpose		0.09	(0.04,0.13)	0.02	1.32e-04***		0.03	(-0.01,0.07)	0.02	0.184
Character & virtue		0.11	(0.06,0.16)	0.03	3.06e-05***		0.06	(0.01,0.11)	0.02	0.011*
Close social relationships		0.04	(-0.01,0.08)	0.02	0.086		-0.02	(-0.06,0.02)	0.02	0.441
Financial & material security		0.02	(-0.03,0.07)	0.02	0.411		0.00	(-0.04,0.05)	0.02	0.831
Psychological Well-Being										
Happiness (a)		0.02	(-0.03,0.07)	0.03	0.387		-0.02	(-0.07,0.02)	0.02	0.275
Life satisfaction (a)		0.02	(-0.02,0.07)	0.02	0.312		-0.02	(-0.06,0.02)	0.02	0.299
Current life evaluation		0.02	(-0.03,0.07)	0.02	0.396		-0.01	(-0.06,0.03)	0.02	0.511
Future life evaluation		0.04	(-0.01,0.08)	0.02	0.134		-0.01	(-0.05,0.03)	0.02	0.673
Optimism		0.08	(0.03,0.13)	0.03	1.65e-03**		0.03	(-0.02,0.07)	0.02	0.233
Freedom to pursue what's important		0.02	(-0.02,0.07)	0.02	0.293		-0.01	(-0.06,0.03)	0.02	0.516
Inner peace	1.02		(1.00,1.04)	0.01	0.083	1.00		(0.98,1.02)	0.01	0.789
Life balance	1.02		(0.99,1.04)	0.01	0.200	1.00		(0.98,1.02)	0.01	0.952
Sense of mastery	1.01		(0.99,1.03)	0.01	0.408	1.00		(0.98,1.02)	0.01	0.649
Meaningful activities (c)		0.07	(0.03,0.12)	0.02	2.52e-03**		0.02	(-0.03,0.06)	0.02	0.405
Understanding purpose (c)		0.08	(0.03,0.13)	0.02	8.05e-04**		0.03	(-0.01,0.07)	0.02	0.198
Self-rated mental health (b)		0.04	(-0.01,0.08)	0.02	0.101		-0.00	(-0.04,0.04)	0.02	0.909
Psychological Distress										
Traumatic distress	1.00		(0.98,1.03)	0.01	0.743	1.01		(0.98,1.03)	0.01	0.595
Depression symptoms composite	0.99		(0.97,1.01)	0.01	0.353	1.01		(0.99,1.03)	0.01	0.593
Depression – feel hopeless	0.99		(0.97,1.01)	0.01	0.427	1.00		(0.98,1.02)	0.01	0.685
Depression – loss of interest	0.99		(0.97,1.01)	0.01	0.345	1.00		(0.98,1.02)	0.01	0.816
Anxiety symptoms composite	0.99		(0.97,1.02)	0.01	0.552	1.00		(0.98,1.03)	0.01	0.673
Anxiety – feel on edge	0.99		(0.97,1.01)	0.01	0.544	1.00		(0.98,1.02)	0.01	0.717
Anxiety – cannot stop worrying	1.00		(0.97,1.02)	0.01	0.720	1.01		(0.99,1.03)	0.01	0.559
Suffering	0.99		(0.96,1.01)	0.01	0.281	0.99		(0.97,1.02)	0.01	0.673
Social Well-Being										
Relationship contentment (e)		0.03	(-0.01,0.08)	0.02	0.170		-0.02	(-0.06,0.03)	0.02	0.442
Relationship satisfaction (e)		0.04	(-0.01,0.09)	0.03	0.121		-0.01	(-0.06,0.03)	0.02	0.609
Social support		0.04	(-0.00,0.09)	0.02	0.056		0.00	(-0.04,0.05)	0.02	0.832
Intimate/close friend	1.03		(1.01,1.05)	0.01	0.009*	1.01		(0.99,1.03)	0.01	0.181
Government approval	1.03		(1.00,1.05)	0.01	0.021*	1.02		(0.99,1.04)	0.01	0.219
Say in government	1.03		(1.01,1.05)	0.01	0.010*	1.01		(0.99,1.03)	0.01	0.342
Belonging in country		0.05	(0.00,0.10)	0.02	0.036*		0.01	(-0.03,0.06)	0.02	0.588
City/place satisfaction	1.03		(1.00,1.05)	0.01	0.031*	1.02		(0.99,1.04)	0.01	0.170
Trust within country	1.03		(1.00,1.05)	0.01	0.034*	1.02		(1.00,1.04)	0.01	0.121
Social Participation										
Ever been married	1.00		(0.98,1.01)	0.01	0.863	1.00		(0.98,1.01)	0.01	0.765
Currently divorced	1.00		(0.99,1.01)	0.00	0.497	1.00		(0.99,1.01)	0.00	0.611
Number of children		0.01	(-0.03,0.05)	0.02	0.690		0.01	(-0.03,0.05)	0.02	0.672
Weekly+ community participation	1.01		(0.99,1.03)	0.01	0.527	1.00		(0.98,1.02)	0.01	0.863
Weekly+ religious attendance	1.00		(1.00,1.01)	0.00	0.451	1.00		(0.99,1.01)	0.00	0.722
Social Distress										
Loneliness		-0.02	(-0.07,0.03)	0.02	0.395		0.02	(-0.02,0.06)	0.02	0.370
Perceived discrimination	1.00		(0.98,1.02)	0.01	0.798	1.00		(0.98,1.02)	0.01	0.901
Character & Prosocial Behavior										
Orientation to promote good (d)		0.12	(0.07,0.17)	0.03	9.99e-06***		0.08	(0.03,0.13)	0.03	3.52e-03**
Delayed gratification (d)		0.06	(0.01,0.12)	0.03	0.019*		0.03	(-0.02,0.09)	0.03	0.232
Hope		0.06	(0.01,0.11)	0.03	0.013*		0.01	(-0.04,0.05)	0.02	0.742
Gratitude		0.10	(0.05,0.15)	0.03	9.13e-05***		0.04	(-0.00,0.09)	0.02	0.055
Showing love/care		0.11	(0.06,0.17)	0.03	8.06e-05***		0.07	(0.01,0.12)	0.03	0.014*
Forgivingness	1.19		(1.17,1.22)	0.01	2.22e-16***	1.19		(1.16,1.21)	0.01	2.22e-16***
Charitable giving	1.02		(0.99,1.05)	0.01	0.188	1.01		(0.98,1.03)	0.01	0.542
Helping strangers	1.01		(0.99,1.04)	0.01	0.227	1.00		(0.98,1.03)	0.01	0.848
Volunteering	1.01		(0.99,1.03)	0.01	0.244	1.00		(0.98,1.02)	0.01	0.798
Physical Health & Health Behavior										
Self-rated physical health (b)		0.01	(-0.03,0.05)	0.02	0.633		-0.01	(-0.05,0.03)	0.02	0.688
Health problems	1.01		(0.99,1.04)	0.01	0.261	1.01		(0.99,1.03)	0.01	0.232
Pain in past 4 weeks	0.99		(0.97,1.01)	0.01	0.335	0.99		(0.97,1.01)	0.01	0.311
Daily smoker	1.00		(0.98,1.01)	0.01	0.770	1.00		(0.98,1.01)	0.01	0.908
Number of drinks per week		-0.04	(-0.10,0.01)	0.03	0.122		-0.05	(-0.10,0.01)	0.03	0.091
Days exercise per week		0.02	(-0.03,0.07)	0.02	0.442		0.01	(-0.04,0.06)	0.02	0.737
Socioeconomic Outcomes										
Financial security (f)		0.02	(-0.02,0.07)	0.02	0.350		0.01	(-0.03,0.06)	0.02	0.566
Material security (f)		0.01	(-0.04,0.07)	0.03	0.631		-0.00	(-0.05,0.04)	0.03	0.848
Educational attainment (16+ years)	1.00		(0.98,1.01)	0.01	0.557	1.00		(0.98,1.01)	0.01	0.478
Currently employed	0.99		(0.97,1.01)	0.01	0.210	0.99		(0.97,1.01)	0.01	0.286
Financially comfortable/getting by	1.00		(0.98,1.02)	0.01	0.955	1.00		(0.98,1.02)	0.01	0.802
Own home	1.01		(0.99,1.03)	0.01	0.396	1.02		(0.99,1.04)	0.01	0.139
Income – top quintile	1.00		(0.98,1.02)	0.01	0.975	1.00		(0.98,1.02)	0.01	0.887
Religion & Spirituality										
Religious/spiritual connection	1.03		(1.01,1.05)	0.01	2.64e-03**	1.00		(0.99,1.02)	0.01	0.588
Belief in life after death	1.02		(1.00,1.04)	0.01	0.132	0.99		(0.98,1.01)	0.01	0.525
Transformative religious experience	1.01		(0.99,1.03)	0.01	0.280	0.99		(0.98,1.01)	0.01	0.358
Religious reading or listening	1.01		(1.00,1.02)	0.00	3.16e-03**	1.01		(1.00,1.01)	0.00	0.138
Prayer or meditation	1.02		(1.01,1.04)	0.01	0.006*	1.01		(0.99,1.02)	0.01	0.434
Belief in God/gods/spiritual forces	1.01		(0.99,1.03)	0.01	0.448	0.99		(0.97,1.01)	0.01	0.439
Religious centrality	1.01		(0.99,1.02)	0.01	0.272	0.99		(0.98,1.00)	0.01	0.207
Religious/spiritual comfort	1.01		(0.99,1.03)	0.01	0.372	0.98		(0.97,1.00)	0.01	0.045*
Feel loved by God	1.02		(1.00,1.04)	0.01	0.021*	1.00		(0.98,1.01)	0.01	0.732
Feel punished by God	1.00		(0.99,1.01)	0.00	0.601	1.00		(0.99,1.01)	0.00	0.624
Experienced religious criticism	0.99		(0.98,1.00)	0.01	0.253	0.99		(0.98,1.00)	0.01	0.141
Faith-sharing	1.01		(0.99,1.03)	0.01	0.400	0.99		(0.98,1.01)	0.01	0.457

Notes. N=3844; Reference for focal predictor: never/rarely. RR, risk-ratio, null effect is 1.00; ES, effect size measure for standardized regression coefficient, null effect is 0.00; SE, standard error, the SE reported for binary/Likert-type outcomes where risk-ratios are on the log(RR) scale; CI, confidence interval; p-value, a Wald-type test of the null hypothesis that the effect of the focal predictor is zero; (a) item part of the Happiness & Life Satisfaction domain of the Secure Flourishing Index; (b) item part of the Physical & Mental Health domain of the Secure Flourishing Index; (c) item part of the Meaning & Purpose domain of the Secure Flourishing Index; (d) item part of the Character & Virtue domain of the Secure Flourishing Index; (e) item part of the Subjective Social Connectedness domain of the Secure Flourishing Index; (f) item part of the Financial & Material Security domain of the Secure Flourishing Index.

Multiple imputation was performed to impute missing data on the covariates, exposure, and outcomes. All models controlled for sociodemographic and childhood factors assessed at Wave 1. For Model 2 with PC (principal components), the first seven principal components of the entire set of contemporaneous confounders assessed at Wave 1 were included as additional covariates of the outcomes at Wave 2.

An outcome-wide analytic approach was used, and a separate model was run for each outcome. A different type of model was run depending on the nature of the outcome: (1) for each binary outcome, a weighted generalized linear model (with a log link and Poisson distribution) was used to estimate an RR; and (2) for each continuous outcome, a weighted linear regression model was used to estimate a ES. All effect sizes were standardized. For continuous outcomes, the ES represents the change in SD on the outcome between the lower and upper categories of the binary focal predictor. For binary outcomes, the RR represents the change in risk of being in the upper category compared to the lower category between the lower and upper categories of the binary focal predictor.

P-value significance thresholds: $p < 0.05$*, $p < 0.005$**, (Bonferroni) $p < 6.41e-04$***, correction for multiple testing using Bonferroni adjusted significant threshold.

Table S10h. Associations of forgivingness with adult well-being and other outcomes at Wave 2 in Australia using complete-case analyses with attrition weights.

	Model 1: Demographic and Childhood Variables as Covariates					Model 2: Demographic, Childhood, and Other Wave 1 Confounding Variables (Via Principal Components) as Covariates				
Outcome	RR	ES	95% CI	SE	p-value	RR	ES	95% CI	SE	p-value
Human Flourishing										
Secure flourishing index		0.07	(0.02,0.12)	0.03	4.63e-03**		0.02	(-0.02,0.06)	0.02	0.389
Flourishing index		0.07	(0.02,0.12)	0.03	0.007*		0.01	(-0.03,0.06)	0.02	0.520
Happiness & life satisfaction		0.03	(-0.03,0.08)	0.03	0.300		-0.02	(-0.07,0.02)	0.02	0.287
Physical & mental health		0.04	(-0.01,0.09)	0.03	0.122		0.01	(-0.04,0.05)	0.02	0.802
Meaning & purpose		0.09	(0.03,0.14)	0.03	1.32e-03**		0.03	(-0.02,0.08)	0.03	0.175
Character & virtue		0.13	(0.07,0.19)	0.03	4.86e-05***		0.08	(0.03,0.14)	0.03	3.7e-03**
Close social relationships		0.03	(-0.03,0.09)	0.03	0.303		-0.02	(-0.07,0.03)	0.03	0.394
Financial & material security		0.05	(-0.01,0.11)	0.03	0.084		0.02	(-0.03,0.07)	0.02	0.408
Psychological Well-Being										
Happiness (a)		0.03	(-0.03,0.08)	0.03	0.298		-0.02	(-0.07,0.02)	0.02	0.315
Life satisfaction (a)		0.03	(-0.03,0.08)	0.03	0.339		-0.02	(-0.07,0.02)	0.02	0.320
Current life evaluation		0.03	(-0.02,0.08)	0.03	0.235		-0.01	(-0.06,0.03)	0.02	0.634
Future life evaluation		0.05	(-0.00,0.11)	0.03	0.056		0.01	(-0.04,0.06)	0.03	0.789
Optimism		0.09	(0.04,0.15)	0.03	1.69e-03**		0.04	(-0.01,0.10)	0.03	0.106
Freedom to pursue what's important		0.02	(-0.03,0.08)	0.03	0.383		-0.02	(-0.07,0.03)	0.02	0.411
Inner peace	1.02		(1.00,1.05)	0.01	0.110	1.00		(0.98,1.03)	0.01	0.839
Life balance	1.02		(0.99,1.04)	0.01	0.242	1.00		(0.97,1.02)	0.01	0.887
Sense of mastery	1.01		(0.99,1.03)	0.01	0.349	1.00		(0.98,1.02)	0.01	0.924
Meaningful activities (c)		0.09	(0.03,0.14)	0.03	2.61e-03**		0.03	(-0.02,0.08)	0.03	0.203
Understanding purpose (c)		0.08	(0.02,0.13)	0.03	4.52e-03**		0.03	(-0.02,0.08)	0.03	0.239
Self-rated mental health (b)		0.05	(-0.00,0.10)	0.03	0.075		0.01	(-0.04,0.05)	0.02	0.782
Psychological Distress										
Traumatic distress	1.01		(0.99,1.04)	0.01	0.262	1.02		(1.00,1.04)	0.01	0.105
Depression symptoms composite	0.99		(0.96,1.01)	0.01	0.367	1.00		(0.98,1.03)	0.01	0.774
Depression – feel hopeless	0.99		(0.97,1.02)	0.01	0.591	1.01		(0.99,1.03)	0.01	0.592
Depression – loss of interest	0.99		(0.96,1.01)	0.01	0.258	1.00		(0.98,1.02)	0.01	0.945
Anxiety symptoms composite	0.99		(0.96,1.01)	0.01	0.403	1.00		(0.98,1.02)	0.01	0.960
Anxiety – feel on edge	0.99		(0.96,1.01)	0.01	0.383	1.00		(0.98,1.02)	0.01	0.967
Anxiety – cannot stop worrying	0.99		(0.97,1.02)	0.01	0.570	1.00		(0.98,1.03)	0.01	0.749
Suffering	0.98		(0.95,1.00)	0.01	0.097	0.99		(0.96,1.01)	0.01	0.326
Social Well-Being										
Relationship contentment (e)		0.03	(-0.03,0.08)	0.03	0.328		-0.02	(-0.07,0.03)	0.03	0.400
Relationship satisfaction (e)		0.03	(-0.03,0.09)	0.03	0.314		-0.02	(-0.07,0.03)	0.03	0.429
Social support		0.05	(-0.01,0.10)	0.03	0.102		0.00	(-0.05,0.06)	0.03	0.861
Intimate/close friend	1.03		(1.00,1.05)	0.01	0.018*	1.01		(0.99,1.04)	0.01	0.202
Government approval	1.03		(1.01,1.06)	0.01	0.010*	1.02		(0.99,1.04)	0.01	0.157
Say in government	1.03		(1.01,1.06)	0.01	0.006*	1.01		(0.99,1.04)	0.01	0.237
Belonging in country		0.07	(0.01,0.12)	0.03	0.024*		0.02	(-0.04,0.07)	0.03	0.532
City/place satisfaction	1.03		(1.01,1.06)	0.01	0.009*	1.02		(0.99,1.04)	0.01	0.135
Trust within country	1.04		(1.01,1.06)	0.01	0.006*	1.03		(1.00,1.05)	0.01	0.034*
Social Participation										
Ever been married	1.00		(0.98,1.01)	0.01	0.622	0.99		(0.98,1.01)	0.01	0.517
Currently divorced	1.00		(0.98,1.01)	0.01	0.396	1.00		(0.99,1.01)	0.01	0.476
Number of children		-0.02	(-0.07,0.04)	0.03	0.551		-0.01	(-0.06,0.04)	0.03	0.661
Weekly+ community participation	1.01		(0.98,1.03)	0.01	0.594	1.00		(0.98,1.03)	0.01	0.810
Weekly+ religious attendance	1.00		(1.00,1.01)	0.00	0.377	1.00		(0.99,1.01)	0.00	0.529
Social Distress										
Loneliness		-0.02	(-0.07,0.04)	0.03	0.552		0.03	(-0.02,0.07)	0.02	0.217
Perceived discrimination	0.99		(0.97,1.02)	0.01	0.646	1.00		(0.98,1.03)	0.01	0.775
Character & Prosocial Behavior										
Orientation to promote good (d)		0.13	(0.06,0.19)	0.03	1.32e-04***		0.09	(0.03,0.15)	0.03	0.005*
Delayed gratification (d)		0.10	(0.04,0.15)	0.03	1.35e-03**		0.06	(0.01,0.12)	0.03	0.031*
Hope		0.07	(0.01,0.12)	0.03	0.017*		0.01	(-0.03,0.06)	0.02	0.581
Gratitude		0.12	(0.07,0.17)	0.03	1.31e-05***		0.07	(0.02,0.12)	0.03	0.008*
Showing love/care		0.09	(0.03,0.15)	0.03	2.82e-03**		0.06	(-0.00,0.11)	0.03	0.062
Forgivingness	1.19		(1.16,1.22)	0.01	2.22e-16***	1.19		(1.16,1.22)	0.01	2.22e-16***
Charitable giving	1.02		(0.99,1.05)	0.01	0.133	1.01		(0.99,1.04)	0.01	0.321
Helping strangers	1.01		(0.99,1.04)	0.01	0.315	1.01		(0.98,1.04)	0.01	0.498
Volunteering	1.02		(0.99,1.04)	0.01	0.212	1.01		(0.99,1.03)	0.01	0.291
Physical Health & Health Behavior										
Self-rated physical health (b)		0.02	(-0.03,0.07)	0.03	0.426		0.00	(-0.04,0.05)	0.02	0.902
Health problems	1.02		(1.00,1.04)	0.01	0.079	1.02		(1.00,1.04)	0.01	0.044*
Pain in past 4 weeks	0.98		(0.96,1.01)	0.01	0.220	0.99		(0.96,1.01)	0.01	0.263
Daily smoker	1.00		(0.98,1.02)	0.01	0.705	1.00		(0.98,1.02)	0.01	0.804
Number of drinks per week		-0.05	(-0.11,0.01)	0.03	0.125		-0.06	(-0.12,0.01)	0.03	0.083
Days exercise per week		0.02	(-0.04,0.08)	0.03	0.466		0.02	(-0.04,0.07)	0.03	0.584
Socioeconomic Outcomes										
Financial security (f)		0.06	(-0.00,0.11)	0.03	0.053		0.03	(-0.02,0.08)	0.03	0.219
Material security (f)		0.04	(-0.02,0.09)	0.03	0.207		0.01	(-0.05,0.06)	0.03	0.796
Educational attainment (16+ years)	1.00		(0.99,1.01)	0.00	0.793	1.00		(0.99,1.01)	0.00	0.608
Currently employed	0.98		(0.97,1.00)	0.01	0.082	0.98		(0.97,1.00)	0.01	0.076
Financially comfortable/getting by	1.02		(1.00,1.05)	0.01	0.096	1.01		(0.99,1.04)	0.01	0.337
Own home	1.02		(0.99,1.05)	0.01	0.112	1.02		(1.00,1.05)	0.01	0.113
Income – top quintile	1.00		(0.98,1.03)	0.01	0.886	1.00		(0.98,1.03)	0.01	0.818
Religion & Spirituality										
Religious/spiritual connection	1.03		(1.01,1.05)	0.01	4.74e-03**	1.01		(0.99,1.03)	0.01	0.226
Belief in life after death	1.01		(0.98,1.03)	0.01	0.609	0.99		(0.97,1.01)	0.01	0.191
Transformative religious experience	1.00		(0.98,1.02)	0.01	0.718	0.99		(0.97,1.01)	0.01	0.346
Religious reading or listening	1.01		(1.00,1.02)	0.00	0.005*	1.01		(1.00,1.02)	0.00	0.045*
Prayer or meditation	1.02		(1.01,1.04)	0.01	2.62e-03**	1.01		(1.00,1.03)	0.01	0.059
Belief in God/gods/spiritual forces	1.01		(0.99,1.04)	0.01	0.257	1.00		(0.98,1.02)	0.01	0.839
Religious centrality	1.01		(0.99,1.03)	0.01	0.298	0.99		(0.98,1.01)	0.01	0.366
Religious/spiritual comfort	1.00		(0.98,1.03)	0.01	0.822	0.98		(0.96,1.00)	0.01	0.032*
Feel loved by God	1.02		(1.00,1.04)	0.01	0.070	1.00		(0.98,1.01)	0.01	0.747
Feel punished by God	1.00		(0.99,1.01)	0.01	0.644	1.00		(0.99,1.01)	0.01	0.779
Experienced religious criticism	0.99		(0.98,1.01)	0.01	0.373	0.99		(0.98,1.01)	0.01	0.252
Faith-sharing	1.01		(0.98,1.03)	0.01	0.531	1.00		(0.98,1.02)	0.01	0.642

Table S10h. Associations of forgivingness with adult well-being and other outcomes at Wave 2 in Australia using complete-case analyses with attrition weights.

	Model 1: Demographic and Childhood Variables as Covariates					Model 2: Demographic, Childhood, and Other Wave 1 Confounding Variables (Via Principal Components) as Covariates				
Outcome	RR	ES	95% CI	SE	p-value	RR	ES	95% CI	SE	p-value

Notes. N=2582; Reference for focal predictor: never/rarely. RR, risk-ratio, null effect is 1.00; ES, effect size measure for standardized regression coefficient, null effect is 0.00; SE, standard error, the SE reported for binary/Likert-type outcomes where risk-ratios are on the log(RR) scale; CI, confidence interval; p-value, a Wald-type test of the null hypothesis that the effect of the focal predictor is zero; (a) item part of the Happiness & Life Satisfaction domain of the Secure Flourishing Index; (b) item part of the Physical & Mental Health domain of the Secure Flourishing Index; (c) item part of the Meaning & Purpose domain of the Secure Flourishing Index; (d) item part of the Character & Virtue domain of the Secure Flourishing Index; (e) item part of the Subjective Social Connectedness domain of the Secure Flourishing Index; (f) item part of the Financial & Material Security domain of the Secure Flourishing Index.

Attrition weights were computed to adjust the complete case data (those who responded at Wave 2 to at least 50% of the questions) and multiple imputation was used to impute missing data on all remaining within wave on the covariates, exposure, and outcomes. All models controlled for sociodemographic and childhood factors assessed at Wave 1. For Model 2 with PC (principal components), the first seven principal components of the entire set of contemporaneous confounders assessed at Wave 1 were included as additional covariates of the outcomes at Wave 2.

An outcome-wide analytic approach was used, and a separate model was run for each outcome. A different type of model was run depending on the nature of the outcome: (1) for each binary outcome, a weighted generalized linear model (with a log link and Poisson distribution) was used to estimate an RR; and (2) for each continuous outcome, a weighted linear regression model was used to estimate a ES. All effect sizes were standardized. For continuous outcomes, the ES represents the change in SD on the outcome between the lower and upper categories of the binary focal predictor. For binary outcomes, the RR represents the change in risk of being in the upper category compared to the lower category between the lower and upper categories of the binary focal predictor.

P-value significance thresholds: $p < 0.05$*, $p < 0.005$**, (Bonferroni) $p < 6.41e-04$***, correction for multiple testing using Bonferroni adjusted significant threshold.

Table S10i. Sensitivity analysis of forgivingness outcome-wide results to unmeasured confounding using E-values in Australia

	Multiple Imputation				Complete Case w/ Attrition Weights			
	Model 1: Demographics and Childhood Variables as Covariates		Model 2: Demographics, Childhood, and Other Wave 1 Confounders (Via Principal Components) as Covariates		Model 1: Demographics and Childhood Variables as Covariates		Model 2: Demographics, Childhood, and Other Wave 1 Confounders (Via Principal Components) as Covariates	
Outcome	EE	ECI	EE	ECI	EE	ECI	EE	ECI
Human Flourishing								
Secure flourishing index	1.55	1.29	1.15	1.00	1.62	1.28	1.24	1.00
Flourishing index	1.60	1.33	1.15	1.00	1.62	1.26	1.21	1.00
Happiness & life satisfaction	1.30	1.00	1.30	1.00	1.33	1.00	1.29	1.00
Physical & mental health	1.32	1.00	1.13	1.00	1.40	1.00	1.12	1.00
Meaning & purpose	1.71	1.42	1.31	1.00	1.72	1.37	1.37	1.00
Character & virtue	1.85	1.53	1.56	1.22	1.97	1.58	1.69	1.32
Close social relationships	1.41	1.00	1.23	1.00	1.33	1.00	1.28	1.00
Financial & material security	1.26	1.00	1.11	1.00	1.47	1.00	1.27	1.00
Psychological Well-Being								
Happiness	1.28	1.00	1.30	1.00	1.33	1.00	1.28	1.00
Life satisfaction	1.29	1.00	1.28	1.00	1.31	1.00	1.29	1.00
Current life evaluation	1.26	1.00	1.22	1.00	1.34	1.00	1.18	1.00
Future life evaluation	1.38	1.00	1.17	1.00	1.49	1.00	1.14	1.00
Optimism	1.67	1.34	1.33	1.00	1.76	1.38	1.44	1.00
Freedom to pursue what's important	1.30	1.00	1.21	1.00	1.29	1.00	1.26	1.00
Inner peace	1.27	1.00	1.09	1.00	1.28	1.00	1.08	1.00
Life balance	1.23	1.00	1.04	1.00	1.24	1.00	1.07	1.00
Sense of mastery	1.17	1.00	1.12	1.00	1.19	1.00	1.05	1.00
Meaningful activities	1.63	1.31	1.25	1.00	1.70	1.34	1.36	1.00
Understanding purpose	1.67	1.36	1.33	1.00	1.66	1.30	1.34	1.00
Self-rated mental health	1.38	1.00	1.07	1.00	1.45	1.00	1.13	1.00
Psychological Distress								
Traumatic distress	1.10	1.00	1.13	1.00	1.22	1.00	1.27	1.00
Depression symptoms composite	1.19	1.00	1.13	1.00	1.20	1.00	1.10	1.00
Depression – feel hopeless	1.17	1.00	1.11	1.00	1.14	1.00	1.13	1.00
Depression – loss of interest	1.19	1.00	1.09	1.00	1.22	1.00	1.05	1.00
Anxiety symptoms composite	1.15	1.00	1.12	1.00	1.19	1.00	1.04	1.00
Anxiety – feel on edge	1.14	1.00	1.10	1.00	1.19	1.00	1.04	1.00
Anxiety – cannot stop worrying	1.11	1.00	1.14	1.00	1.15	1.00	1.10	1.00
Suffering	1.22	1.00	1.12	1.00	1.30	1.00	1.21	1.00
Social Well-Being								
Relationship contentment	1.36	1.00	1.24	1.00	1.32	1.00	1.28	1.00
Relationship satisfaction	1.41	1.00	1.19	1.00	1.33	1.00	1.27	1.00
Social support	1.44	1.00	1.11	1.00	1.45	1.00	1.11	1.00
Intimate/close friend	1.33	1.14	1.21	1.00	1.32	1.11	1.22	1.00
Government approval	1.35	1.11	1.23	1.00	1.37	1.16	1.24	1.00
Say in government	1.35	1.15	1.19	1.00	1.39	1.18	1.22	1.00
Belonging in country	1.49	1.10	1.20	1.00	1.58	1.16	1.24	1.00
City/place satisfaction	1.33	1.08	1.24	1.00	1.39	1.16	1.27	1.00
Trust within country	1.32	1.07	1.27	1.00	1.39	1.18	1.33	1.07
Social Participation								
Ever been married	1.06	1.00	1.08	1.00	1.11	1.00	1.13	1.00
Currently divorced	1.09	1.00	1.08	1.00	1.12	1.00	1.11	1.00
Number of children	1.16	1.00	1.17	1.00	1.22	1.00	1.18	1.00
Weekly+ community participation	1.15	1.00	1.07	1.00	1.14	1.00	1.09	1.00
Weekly+ religious attendance	1.08	1.00	1.05	1.00	1.10	1.00	1.08	1.00
Social Distress								
Loneliness	1.27	1.00	1.26	1.00	1.23	1.00	1.32	1.00
Perceived discrimination	1.09	1.00	1.06	1.00	1.13	1.00	1.10	1.00
Character & Prosocial Behavior								
Orientation to promote good	1.94	1.60	1.65	1.31	1.97	1.55	1.71	1.30
Delayed gratification	1.58	1.19	1.36	1.00	1.76	1.39	1.54	1.13
Hope	1.57	1.21	1.15	1.00	1.59	1.20	1.21	1.00
Gratitude	1.80	1.48	1.44	1.00	1.92	1.58	1.58	1.24
Showing love/care	1.88	1.53	1.59	1.21	1.75	1.35	1.51	1.00
Forgivingness	2.43	2.26	2.39	2.22	2.43	2.23	2.39	2.19
Charitable giving	1.26	1.00	1.16	1.00	1.29	1.00	1.22	1.00
Helping strangers	1.23	1.00	1.08	1.00	1.22	1.00	1.17	1.00
Volunteering	1.20	1.00	1.08	1.00	1.23	1.00	1.20	1.00
Physical Health & Health Behavior								
Self-rated physical health	1.18	1.00	1.15	1.00	1.27	1.00	1.09	1.00
Health problems	1.21	1.00	1.21	1.00	1.28	1.00	1.28	1.04
Pain in past 4 weeks	1.20	1.00	1.20	1.00	1.25	1.00	1.23	1.00
Daily smoker	1.08	1.00	1.05	1.00	1.10	1.00	1.08	1.00
Number of drinks per week	1.43	1.00	1.46	1.00	1.47	1.00	1.51	1.00
Days exercise per week	1.25	1.00	1.15	1.00	1.27	1.00	1.22	1.00
Socioeconomic Outcomes								
Financial security	1.28	1.00	1.20	1.00	1.51	1.00	1.35	1.00
Material security	1.20	1.00	1.12	1.00	1.39	1.00	1.14	1.00
Educational attainment (16+ years)	1.10	1.00	1.11	1.00	1.06	1.00	1.08	1.00
Currently employed	1.20	1.00	1.19	1.00	1.24	1.00	1.24	1.00
Financially comfortable/getting by	1.04	1.00	1.08	1.00	1.28	1.00	1.20	1.00
Own home	1.19	1.00	1.25	1.00	1.29	1.00	1.28	1.00
Income – top quintile	1.03	1.00	1.06	1.00	1.07	1.00	1.09	1.00
Religion & Spirituality								
Religious/spiritual connection	1.34	1.18	1.11	1.00	1.36	1.18	1.18	1.00
Belief in life after death	1.24	1.00	1.14	1.00	1.14	1.00	1.22	1.00
Transformative religious experience	1.18	1.00	1.16	1.00	1.11	1.00	1.18	1.00
Religious reading or listening	1.21	1.11	1.14	1.00	1.22	1.11	1.17	1.02
Prayer or meditation	1.28	1.13	1.13	1.00	1.31	1.16	1.21	1.00
Belief in God/gods/spiritual forces	1.16	1.00	1.16	1.00	1.23	1.00	1.08	1.00
Religious centrality	1.17	1.00	1.17	1.00	1.18	1.00	1.15	1.00
Religious/spiritual comfort	1.16	1.00	1.24	1.03	1.09	1.00	1.26	1.07
Feel loved by God	1.27	1.09	1.08	1.00	1.26	1.00	1.08	1.00
Feel punished by God	1.09	1.00	1.08	1.00	1.09	1.00	1.07	1.00
Experienced religious criticism	1.14	1.00	1.16	1.00	1.14	1.00	1.16	1.00
Faith-sharing	1.16	1.00	1.15	1.00	1.15	1.00	1.12	1.00

Notes. EE, E-value for estimate; ECI, E-value for the limit of the confidence interval. The formula for calculating E-values can be found in VanderWeele and Ding (2017). E-values for estimate are the minimum strength of association on the risk ratio scale that an unmeasured confounder would need to have with both the exposure and the outcome to fully explain away the observed association between the exposure and outcome, conditional on the measured covariates. E-values for the 95% CI closest to the null denote the minimum strength of association on the risk ratio scale that an unmeasured confounder would need to have with both the exposure and the outcome to shift the CI to include the null value, conditional on the measured covariates.

Table S11a. Weighted summary statistics for demographic and childhood variables in Brazil

**Characteristic**	**Wave 1**<br>N = 13,203	**Wave 2**<br>N = 4,240
Forgivingness, n (%)		
Always	4,811 (36.4%)	1,478 (34.9%)
Often	4,737 (35.9%)	1,651 (38.9%)
Rarely	2,947 (22.3%)	937 (22.1%)
Never	675 (5.1%)	166 (3.9%)
(Missing)	32 (0.2%)	9 (0.2%)
Year of birth, n (%)		
1943 or earlier (current age: 80+ years)	138 (1.0%)	54 (1.3%)
1943-1953 (current age: 70-79 years)	566 (4.3%)	229 (5.4%)
1953-1963 (current age: 60-69 years)	1,514 (11.5%)	515 (12.1%)
1963-1973 (current age: 50-59 years)	2,031 (15.4%)	696 (16.4%)
1973-1983 (current age: 40-49 years)	2,514 (19.0%)	820 (19.3%)
1983-1993 (current age: 30-39 years)	2,827 (21.4%)	958 (22.6%)
1993-1998 (current age: 25-29 years)	1,464 (11.1%)	462 (10.9%)
1998-2005 (current age: 18-24 years)	2,150 (16.3%)	507 (12.0%)
(Missing)	0 (0%)	0 (0%)
Age of participant		
Mean	41.9	43.8
Standard Deviation	16.2	16.0
Min, Max	18.0, 93.0	19.0, 93.0
Gender, n (%)		
Male	6,337 (48.0%)	2,021 (47.7%)
Female	6,800 (51.5%)	2,204 (52.0%)
Other	35 (0.3%)	11 (0.2%)
(Missing)	31 (0.2%)	5 (0.1%)
Respondent marital status, n (%)		
Single/Never been married	4,406 (33.4%)	1,403 (33.1%)
Married	4,582 (34.7%)	1,531 (36.1%)
Separated	586 (4.4%)	176 (4.1%)
Divorced	856 (6.5%)	304 (7.2%)
Widowed	439 (3.3%)	151 (3.6%)
Domestic partner	2,066 (15.7%)	620 (14.6%)
(Missing)	267 (2.0%)	55 (1.3%)
Education (years), n (%)		
Up to 8	3,327 (25.2%)	805 (19.0%)
9-15	7,618 (57.7%)	2,548 (60.1%)
16+	2,248 (17.0%)	887 (20.9%)
(Missing)	11 (0.1%)	0 (0%)
Employment status, n (%)		
Employed for an employer	3,634 (27.5%)	1,377 (32.5%)
Self-employed	2,876 (21.8%)	931 (22.0%)
Retired	1,629 (12.3%)	565 (13.3%)
Student	664 (5.0%)	137 (3.2%)
Homemaker	1,319 (10.0%)	441 (10.4%)
Unemployed and looking for a job	2,430 (18.4%)	567 (13.4%)
None of these/Other	449 (3.4%)	140 (3.3%)
(Missing)	203 (1.5%)	83 (1.9%)
Current religious service attendance, n (%)		
More than once a week	2,399 (18.2%)	830 (19.6%)
Once a week	2,283 (17.3%)	735 (17.3%)

Table S11a. Weighted summary statistics for demographic and childhood variables in Brazil

**Characteristic**	**Wave 1** N = 13,203	**Wave 2** N = 4,240
One to three times a month	1,405 (10.6%)	391 (9.2%)
A few times a year	3,968 (30.1%)	1,251 (29.5%)
Never	3,086 (23.4%)	1,016 (24.0%)
(Missing)	62 (0.5%)	18 (0.4%)
Immigration status, n (%)		
Born in this country	12,679 (96.0%)	4,126 (97.3%)
Born in another country	153 (1.2%)	45 (1.1%)
(Missing)	371 (2.8%)	69 (1.6%)
Parental marital status around age 12, n (%)		
Parents were married	8,481 (64.2%)	2,847 (67.1%)
Parents were divorced	1,391 (10.5%)	380 (9.0%)
Parents were never married	2,017 (15.3%)	669 (15.8%)
One or both of them had died	513 (3.9%)	171 (4.0%)
Unsure	343 (2.6%)	109 (2.6%)
(Missing)	457 (3.5%)	64 (1.5%)
Religious service attendance around age 12, n (%)		
At least once a week	6,294 (47.7%)	2,136 (50.4%)
One to three times a month	2,511 (19.0%)	708 (16.7%)
Less than once a month	2,614 (19.8%)	856 (20.2%)
Never	1,712 (13.0%)	518 (12.2%)
(Missing)	73 (0.6%)	22 (0.5%)
Relationship with mother when growing up, n (%)		
Very good	8,355 (63.3%)	2,708 (63.9%)
Somewhat good	3,552 (26.9%)	1,112 (26.2%)
Somewhat bad	488 (3.7%)	151 (3.5%)
Very bad	216 (1.6%)	71 (1.7%)
(Does not apply)	517 (3.9%)	176 (4.2%)
(Missing)	75 (0.6%)	23 (0.5%)
Relationship with father when growing up, n (%)		
Very good	6,345 (48.1%)	2,020 (47.6%)
Somewhat good	3,638 (27.6%)	1,176 (27.7%)
Somewhat bad	1,039 (7.9%)	335 (7.9%)
Very bad	768 (5.8%)	259 (6.1%)
(Does not apply)	1,319 (10.0%)	411 (9.7%)
(Missing)	94 (0.7%)	40 (0.9%)
Felt like an outsider in family when growing up, n (%)		
Yes	1,690 (12.8%)	537 (12.7%)
No	11,198 (84.8%)	3,614 (85.2%)
(Missing)	315 (2.4%)	89 (2.1%)
Experienced abuse when growing up, n (%)		
Yes	2,600 (19.7%)	935 (22.1%)
No	10,145 (76.8%)	3,238 (76.4%)
(Missing)	458 (3.5%)	67 (1.6%)
Self-rated health when growing up, n (%)		
Excellent	5,280 (40.0%)	1,662 (39.2%)
Very good	3,396 (25.7%)	1,051 (24.8%)
Good	2,882 (21.8%)	975 (23.0%)
Fair	1,383 (10.5%)	462 (10.9%)
Poor	232 (1.8%)	81 (1.9%)
(Missing)	30 (0.2%)	10 (0.2%)

Table S11a. Weighted summary statistics for demographic and childhood variables in Brazil

**Characteristic**	**Wave 1** N = 13,203	**Wave 2** N = 4,240
Subjective financial status of family growing up, n (%)		
Lived comfortably	4,992 (37.8%)	1,567 (37.0%)
Got by	4,608 (34.9%)	1,473 (34.7%)
Found it difficult	2,486 (18.8%)	851 (20.1%)
Found it very difficult	1,037 (7.9%)	330 (7.8%)
(Missing)	80 (0.6%)	20 (0.5%)
Religious affiliation growing up, n (%)		
Christianity	11,390 (86.3%)	3,719 (87.7%)
Taoism	1 (<0.0%)	1 (0.0%)
Confucianism	7 (0.1%)	3 (0.1%)
Primal, Animist, or Folk religion	18 (0.1%)	7 (0.2%)
Spiritism	333 (2.5%)	86 (2.0%)
Umbanda, Candomblé, and other African-derived religions	262 (2.0%)	76 (1.8%)
Chinese folk/traditional religion	0 (0%)	0 (0%)
Islam	15 (0.1%)	5 (0.1%)
Hinduism	1 (<0.0%)	0 (<0.0%)
Buddhism	26 (0.2%)	7 (0.2%)
Judaism	42 (0.3%)	8 (0.2%)
Sikhism	0 (0%)	0 (0%)
Baha'i	1 (0.0%)	0 (0%)
Jainism	4 (0.0%)	1 (0.0%)
Shinto	4 (0.0%)	1 (0.0%)
Some other religion	90 (0.7%)	28 (0.7%)
No religion/Atheist/Agnostic	914 (6.9%)	270 (6.4%)
(Missing)	95 (0.7%)	27 (0.6%)

Note. N (%); this table is based on non-imputed data. Cumulative percentages for variables may not add up to 100% due to rounding. Wave 1 characteristics weighted using the Gallup provided sampling weight, ANNUAL_WEIGHT_R2; Wave 2 characteristics weighted accounting for attrition by using the adjusted Wave 1 weight, ANNUAL_WEIGHT_R2, multiplied by the created attrition weight to account for dropout, to maintain nationally representative estimates for Wave 2 characteristics.

Table S11b. Weighted summary statistics for outcome variables in Brazil

Outcome	Wave 1<br>N = 13,203	Wave 2<br>N = 4,240
Secure flourishing index		
Mean	7.0	6.9
Standard Deviation	1.7	1.7
Min, Max	0.0, 10.0	0.0, 10.0
(Missing)	319 (2.4%)	93 (2.2%)
Flourishing index		
Mean	7.6	7.5
Standard Deviation	1.7	1.7
Min, Max	0.0, 10.0	0.0, 10.0
(Missing)	291 (2.2%)	80 (1.9%)
Happiness & life satisfaction		
Mean	7.2	7.1
Standard Deviation	2.2	2.2
Min, Max	0.0, 10.0	0.0, 10.0
(Missing)	67 (0.5%)	17 (0.4%)
Physical & mental health		
Mean	7.3	7.0
Standard Deviation	2.1	2.2
Min, Max	0.0, 10.0	0.0, 10.0
(Missing)	51 (0.4%)	23 (0.5%)
Meaning & purpose		
Mean	7.8	7.8
Standard Deviation	2.1	2.2
Min, Max	0.0, 10.0	0.0, 10.0
(Missing)	63 (0.5%)	16 (0.4%)
Character & virtue		
Mean	8.3	8.3
Standard Deviation	1.7	1.7
Min, Max	0.0, 10.0	0.0, 10.0
(Missing)	87 (0.7%)	15 (0.3%)
Close social relationships		
Mean	7.5	7.4
Standard Deviation	2.6	2.6
Min, Max	0.0, 10.0	0.0, 10.0
(Missing)	85 (0.6%)	14 (0.3%)
Financial & material security		
Mean	4.0	4.1
Standard Deviation	3.6	3.5
Min, Max	0.0, 10.0	0.0, 10.0
(Missing)	48 (0.4%)	16 (0.4%)
Happiness		
Mean	7.3	7.2
Standard Deviation	2.3	2.3
Min, Max	0.0, 10.0	0.0, 10.0
(Missing)	38 (0.3%)	5 (0.1%)
Life satisfaction		
Mean	7.2	7.0
Standard Deviation	2.5	2.4
Min, Max	0.0, 10.0	0.0, 10.0
(Missing)	34 (0.3%)	12 (0.3%)
Current life evaluation		

Table S11b. Weighted summary statistics for outcome variables in Brazil

Outcome	Wave 1<br>N = 13,203	Wave 2<br>N = 4,240
Mean	6.6	6.6
Standard Deviation	2.3	2.2
Min, Max	0.0, 10.0	0.0, 10.0
(Missing)	19 (0.1%)	4 (<0.1%)
Future life evaluation		
Mean	8.6	8.5
Standard Deviation	1.9	1.9
Min, Max	0.0, 10.0	0.0, 10.0
(Missing)	149 (1.1%)	16 (0.4%)
Optimism		
Mean	9.2	9.1
Standard Deviation	1.6	1.7
Min, Max	0.0, 10.0	0.0, 10.0
(Missing)	28 (0.2%)	11 (0.3%)
Freedom to pursue what's important		
Mean	8.3	8.2
Standard Deviation	2.4	2.4
Min, Max	0.0, 10.0	0.0, 10.0
(Missing)	36 (0.3%)	5 (0.1%)
Inner peace, n (%)		
Always	3,268 (24.7%)	1,040 (24.5%)
Often	6,077 (46.0%)	2,043 (48.2%)
Rarely	3,358 (25.4%)	1,007 (23.7%)
Never	453 (3.4%)	130 (3.1%)
(Missing)	46 (0.4%)	20 (0.5%)
Life balance, n (%)		
Always	2,269 (17.2%)	680 (16.0%)
Often	6,568 (49.7%)	2,160 (50.9%)
Rarely	3,875 (29.3%)	1,263 (29.8%)
Never	458 (3.5%)	131 (3.1%)
(Missing)	33 (0.2%)	6 (0.1%)
Sense of mastery, n (%)		
Always	4,357 (33.0%)	1,342 (31.6%)
Often	6,219 (47.1%)	2,086 (49.2%)
Rarely	2,283 (17.3%)	718 (16.9%)
Never	253 (1.9%)	69 (1.6%)
(Missing)	91 (0.7%)	25 (0.6%)
Meaningful activities		
Mean	7.9	7.9
Standard Deviation	2.3	2.3
Min, Max	0.0, 10.0	0.0, 10.0
(Missing)	24 (0.2%)	3 (<0.1%)
Understanding purpose		
Mean	7.8	7.7
Standard Deviation	2.5	2.6
Min, Max	0.0, 10.0	0.0, 10.0
(Missing)	44 (0.3%)	12 (0.3%)
Self-rated mental health		
Mean	7.4	7.2
Standard Deviation	2.6	2.6
Min, Max	0.0, 10.0	0.0, 10.0

Table S11b. Weighted summary statistics for outcome variables in Brazil

**Outcome**	**Wave 1**<br>N = 13,203	**Wave 2**<br>N = 4,240
(Missing)	24 (0.2%)	12 (0.3%)
Traumatic distress, n (%)		
A lot	2,689 (20.4%)	859 (20.3%)
Some	4,059 (30.7%)	1,208 (28.5%)
Not very much	3,023 (22.9%)	1,075 (25.4%)
Not at all	3,365 (25.5%)	1,085 (25.6%)
(Missing)	67 (0.5%)	14 (0.3%)
Depression symptoms composite, n (%)	5,382 (41.1%)	1,561 (37.0%)
(Missing)	92 (0.7%)	27 (0.6%)
Depression – feel hopeless, n (%)		
Nearly every day	2,021 (15.3%)	539 (12.7%)
More than half the days	1,967 (14.9%)	602 (14.2%)
Several days	3,502 (26.5%)	1,162 (27.4%)
Not at all	5,671 (43.0%)	1,932 (45.6%)
(Missing)	42 (0.3%)	6 (0.1%)
Depression – loss of interest, n (%)		
Nearly every day	2,561 (19.4%)	735 (17.3%)
More than half the days	2,557 (19.4%)	800 (18.9%)
Several days	3,545 (26.8%)	1,242 (29.3%)
Not at all	4,479 (33.9%)	1,442 (34.0%)
(Missing)	61 (0.5%)	22 (0.5%)
Anxiety symptoms composite, n (%)	6,127 (46.7%)	1,869 (44.4%)
(Missing)	70 (0.5%)	32 (0.7%)
Anxiety – feel on edge, n (%)		
Nearly every day	3,199 (24.2%)	933 (22.0%)
More than half the days	2,140 (16.2%)	644 (15.2%)
Several days	4,072 (30.8%)	1,402 (33.1%)
Not at all	3,750 (28.4%)	1,242 (29.3%)
(Missing)	43 (0.3%)	20 (0.5%)
Anxiety – cannot stop worrying, n (%)		
Nearly every day	3,143 (23.8%)	889 (21.0%)
More than half the days	2,096 (15.9%)	632 (14.9%)
Several days	3,491 (26.4%)	1,223 (28.8%)
Not at all	4,429 (33.5%)	1,483 (35.0%)
(Missing)	44 (0.3%)	14 (0.3%)
Suffering, n (%)		
A lot	1,572 (11.9%)	498 (11.8%)
Some	5,737 (43.5%)	1,786 (42.1%)
Not very much	3,082 (23.3%)	1,079 (25.4%)
Not at all	2,744 (20.8%)	858 (20.2%)
(Missing)	68 (0.5%)	18 (0.4%)
Relationship contentment		
Mean	7.7	7.7
Standard Deviation	2.7	2.7
Min, Max	0.0, 10.0	0.0, 10.0
(Missing)	43 (0.3%)	10 (0.2%)
Relationship satisfaction		
Mean	7.2	7.1
Standard Deviation	2.9	2.9
Min, Max	0.0, 10.0	0.0, 10.0
(Missing)	55 (0.4%)	4 (0.1%)

Table S11b. Weighted summary statistics for outcome variables in Brazil

Outcome	Wave 1<br>N = 13,203	Wave 2<br>N = 4,240
Social support		
Mean	7.2	7.2
Standard Deviation	3.0	3.0
Min, Max	0.0, 10.0	0.0, 10.0
(Missing)	54 (0.4%)	12 (0.3%)
Intimate/close friend, n (%)		
Yes	10,752 (81.4%)	3,412 (80.5%)
No	2,338 (17.7%)	786 (18.5%)
(Missing)	113 (0.9%)	43 (1.0%)
Government approval, n (%)		
Strongly approve	1,648 (12.5%)	479 (11.3%)
Somewhat approve	2,978 (22.6%)	864 (20.4%)
Neither approve nor disapprove	3,261 (24.7%)	920 (21.7%)
Somewhat disapprove	2,084 (15.8%)	714 (16.8%)
Strongly disapprove	3,147 (23.8%)	1,221 (28.8%)
(Missing)	85 (0.6%)	43 (1.0%)
Say in government, n (%)		
Agree	3,573 (27.1%)	1,138 (26.8%)
Disagree	5,924 (44.9%)	2,110 (49.8%)
Unsure	3,612 (27.4%)	965 (22.8%)
(Missing)	94 (0.7%)	27 (0.6%)
Belonging in country		
Mean	7.8	7.6
Standard Deviation	2.6	2.6
Min, Max	0.0, 10.0	0.0, 10.0
(Missing)	170 (1.3%)	62 (1.5%)
City/place satisfaction, n (%)		
Satisfied	8,171 (61.9%)	2,518 (59.4%)
Dissatisfied	3,449 (26.1%)	1,205 (28.4%)
Unsure	1,478 (11.2%)	468 (11.0%)
(Missing)	105 (0.8%)	49 (1.2%)
Trust within country, n (%)		
All people	172 (1.3%)	45 (1.1%)
Most people	1,106 (8.4%)	329 (7.7%)
Some people	4,794 (36.3%)	1,539 (36.3%)
Not very many people	5,687 (43.1%)	1,870 (44.1%)
None	1,361 (10.3%)	446 (10.5%)
(Missing)	84 (0.6%)	11 (0.3%)
Number of children		
Mean	0.9	0.9
Standard Deviation	1.4	1.2
Min, Max	0.0, 97.0	0.0, 18.0
(Missing)	322 (2.4%)	501 (12%)
Community participation, n (%)		
More than once a week	1,204 (9.1%)	360 (8.5%)
Once a week	1,195 (9.1%)	325 (7.7%)
One to three times a month	955 (7.2%)	333 (7.9%)
A few times a year	3,244 (24.6%)	1,018 (24.0%)
Never	6,524 (49.4%)	2,189 (51.6%)
(Missing)	82 (0.6%)	14 (0.3%)
Religious attendance, n (%)		

Table S11b. Weighted summary statistics for outcome variables in Brazil

Outcome	Wave 1<br>N = 13,203	Wave 2<br>N = 4,240
More than once a week	2,399 (18.2%)	830 (19.6%)
Once a week	2,283 (17.3%)	735 (17.3%)
One to three times a month	1,405 (10.6%)	391 (9.2%)
A few times a year	3,968 (30.1%)	1,251 (29.5%)
Never	3,086 (23.4%)	1,016 (24.0%)
(Missing)	62 (0.5%)	18 (0.4%)
Loneliness		
Mean	3.9	3.8
Standard Deviation	3.4	3.4
Min, Max	0.0, 10.0	0.0, 10.0
(Missing)	23 (0.2%)	6 (0.1%)
Perceived discrimination, n (%)		
Always	1,429 (10.8%)	444 (10.5%)
Often	2,647 (20.1%)	799 (18.8%)
Rarely	5,007 (37.9%)	1,757 (41.4%)
Never	4,082 (30.9%)	1,232 (29.1%)
(Missing)	38 (0.3%)	8 (0.2%)
Orientation to promote good		
Mean	8.6	8.5
Standard Deviation	1.8	1.8
Min, Max	0.0, 10.0	0.0, 10.0
(Missing)	50 (0.4%)	6 (0.1%)
Delayed gratification		
Mean	8.1	8.0
Standard Deviation	2.3	2.2
Min, Max	0.0, 10.0	0.0, 10.0
(Missing)	40 (0.3%)	10 (0.2%)
Hope		
Mean	8.9	8.8
Standard Deviation	1.9	1.9
Min, Max	0.0, 10.0	0.0, 10.0
(Missing)	29 (0.2%)	11 (0.3%)
Gratitude		
Mean	8.6	8.5
Standard Deviation	2.1	2.1
Min, Max	0.0, 10.0	0.0, 10.0
(Missing)	36 (0.3%)	14 (0.3%)
Showing love/care		
Mean	8.5	8.4
Standard Deviation	2.2	2.2
Min, Max	0.0, 10.0	0.0, 10.0
(Missing)	26 (0.2%)	3 (<0.1%)
Forgivingness, n (%)		
Always	4,811 (36.4%)	1,478 (34.9%)
Often	4,737 (35.9%)	1,651 (38.9%)
Rarely	2,947 (22.3%)	937 (22.1%)
Never	675 (5.1%)	166 (3.9%)
(Missing)	32 (0.2%)	9 (0.2%)
Charitable giving, n (%)		
Yes	4,083 (30.9%)	1,376 (32.4%)
No	9,088 (68.8%)	2,803 (66.1%)

Table S11b. Weighted summary statistics for outcome variables in Brazil

**Outcome**	**Wave 1** N = 13,203	**Wave 2** N = 4,240
(Missing)	32 (0.2%)	62 (1.5%)
Helping strangers, n (%)		
Yes	9,018 (68.3%)	2,734 (64.5%)
No	4,109 (31.1%)	1,468 (34.6%)
(Missing)	76 (0.6%)	39 (0.9%)
Volunteering, n (%)		
Yes	2,449 (18.6%)	781 (18.4%)
No	10,713 (81.1%)	3,415 (80.5%)
(Missing)	41 (0.3%)	44 (1.0%)
Self-rated physical health		
Mean	7.2	6.9
Standard Deviation	2.3	2.3
Min, Max	0.0, 10.0	0.0, 10.0
(Missing)	32 (0.2%)	14 (0.3%)
Health problems, n (%)		
Yes	2,520 (19.1%)	903 (21.3%)
No	10,345 (78.4%)	3,269 (77.1%)
(Missing)	339 (2.6%)	69 (1.6%)
Pain in past 4 weeks, n (%)		
A lot	2,396 (18.1%)	823 (19.4%)
Some	5,385 (40.8%)	1,750 (41.3%)
Not very much	3,040 (23.0%)	989 (23.3%)
None at all	2,357 (17.9%)	678 (16.0%)
(Missing)	25 (0.2%)	0 (<0.0%)
Number of cigarettes per day		
Mean	2.4	2.3
Standard Deviation	6.5	6.5
Min, Max	0.0, 97.0	0.0, 90.0
(Missing)	243 (1.8%)	71 (1.7%)
Number of drinks per week		
Mean	2.0	2.0
Standard Deviation	5.7	5.5
Min, Max	0.0, 97.0	0.0, 97.0
(Missing)	223 (1.7%)	77 (1.8%)
Days exercise per week		
Mean	2.2	2.2
Standard Deviation	2.4	2.4
Min, Max	0.0, 7.0	0.0, 7.0
(Missing)	50 (0.4%)	14 (0.3%)
Financial security		
Mean	4.0	4.1
Standard Deviation	3.8	3.6
Min, Max	0.0, 10.0	0.0, 10.0
(Missing)	24 (0.2%)	3 (<0.1%)
Material security		
Mean	3.9	4.0
Standard Deviation	3.9	3.8
Min, Max	0.0, 10.0	0.0, 10.0
(Missing)	24 (0.2%)	13 (0.3%)
Educational attainment (16+ years), n (%)		
Up to 8	3,327 (25.2%)	805 (19.0%)

Table S11b. Weighted summary statistics for outcome variables in Brazil

Outcome	Wave 1<br>N = 13,203	Wave 2<br>N = 4,240
9-15	7,618 (57.7%)	2,548 (60.1%)
16+	2,248 (17.0%)	887 (20.9%)
(Missing)	11 (0.1%)	0 (0%)
Currently employed, n (%)		
Employed for an employer	3,634 (27.5%)	1,377 (32.5%)
Self-employed	2,876 (21.8%)	931 (22.0%)
Retired	1,629 (12.3%)	565 (13.3%)
Student	664 (5.0%)	137 (3.2%)
Homemaker	1,319 (10.0%)	441 (10.4%)
Unemployed and looking for a job	2,430 (18.4%)	567 (13.4%)
None of these/Other	449 (3.4%)	140 (3.3%)
(Missing)	203 (1.5%)	83 (1.9%)
Financially comfortable/getting by, n (%)		
Living comfortably on present income	1,998 (15.1%)	661 (15.6%)
Getting by on present income	5,718 (43.3%)	2,011 (47.4%)
Finding it difficult on present income	3,692 (28.0%)	1,109 (26.1%)
Finding it very difficult on present income	1,334 (10.1%)	392 (9.2%)
(Missing)	461 (3.5%)	68 (1.6%)
Own home, n (%)		
Someone in this household owns this home	7,302 (55.3%)	2,561 (60.4%)
Someone in this household rents this home	3,348 (25.4%)	1,039 (24.5%)
Both	268 (2.0%)	82 (1.9%)
Neither	1,822 (13.8%)	465 (11.0%)
Rent	0 (0%)	0 (0%)
Own	0 (0%)	0 (0%)
Something else	0 (0%)	0 (0%)
(Missing)	463 (3.5%)	93 (2.2%)
Religious/spiritual connection, n (%)		
Always	5,940 (45.0%)	2,080 (49.1%)
Often	4,230 (32.0%)	1,181 (27.9%)
Rarely	2,339 (17.7%)	720 (17.0%)
Never	664 (5.0%)	256 (6.0%)
(Missing)	30 (0.2%)	3 (0.1%)
Belief in life after death, n (%)		
Yes	7,981 (60.4%)	2,538 (59.8%)
No	2,856 (21.6%)	915 (21.6%)
Unsure	2,303 (17.4%)	756 (17.8%)
(Missing)	63 (0.5%)	32 (0.7%)
Transformative religious experience, n (%)		
Yes	7,804 (59.1%)	2,407 (56.8%)
No	5,308 (40.2%)	1,804 (42.6%)
(Missing)	91 (0.7%)	29 (0.7%)
Religious reading or listening, n (%)		
More than once a day	2,262 (17.1%)	769 (18.1%)
About once a day	2,888 (21.9%)	937 (22.1%)
Sometimes	6,333 (48.0%)	1,911 (45.1%)
Never	1,588 (12.0%)	584 (13.8%)
(Missing)	133 (1.0%)	39 (0.9%)
Prayer or meditation, n (%)		
More than once a day	4,050 (30.7%)	1,358 (32.0%)
About once a day	4,394 (33.3%)	1,307 (30.8%)

Table S11b. Weighted summary statistics for outcome variables in Brazil

Outcome	Wave 1<br>N = 13,203	Wave 2<br>N = 4,240
Sometimes	3,748 (28.4%)	1,217 (28.7%)
Never	870 (6.6%)	309 (7.3%)
(Missing)	142 (1.1%)	48 (1.1%)
Belief in God/gods/spiritual forces, n (%)		
One God	11,458 (86.8%)	3,645 (86.0%)
More than one god	330 (2.5%)	108 (2.6%)
An impersonal spiritual force	806 (6.1%)	278 (6.6%)
None of these	259 (2.0%)	88 (2.1%)
Unsure	268 (2.0%)	93 (2.2%)
(Missing)	81 (0.6%)	28 (0.7%)
Religious centrality, n (%)		
Agree	8,555 (64.8%)	2,740 (64.6%)
Disagree	1,384 (10.5%)	497 (11.7%)
Not relevant	1,658 (12.6%)	545 (12.8%)
Unsure	1,526 (11.6%)	447 (10.6%)
(Missing)	79 (0.6%)	11 (0.3%)
Religious/spiritual comfort, n (%)		
Agree	10,370 (78.5%)	3,269 (77.1%)
Disagree	817 (6.2%)	262 (6.2%)
Not relevant	1,116 (8.5%)	401 (9.5%)
Unsure	854 (6.5%)	305 (7.2%)
(Missing)	45 (0.3%)	4 (0.1%)
Feel loved by God, n (%)		
Agree	11,452 (86.7%)	3,675 (86.7%)
Disagree	459 (3.5%)	145 (3.4%)
Not relevant	679 (5.1%)	215 (5.1%)
Unsure	553 (4.2%)	200 (4.7%)
(Missing)	60 (0.5%)	6 (0.1%)
Feel punished by God, n (%)		
Agree	2,436 (18.4%)	645 (15.2%)
Disagree	7,782 (58.9%)	2,577 (60.8%)
Not relevant	1,542 (11.7%)	527 (12.4%)
Unsure	1,373 (10.4%)	485 (11.4%)
(Missing)	70 (0.5%)	6 (0.1%)
Experienced religious criticism, n (%)		
Agree	2,326 (17.6%)	670 (15.8%)
Disagree	6,345 (48.1%)	2,150 (50.7%)
Not relevant	2,525 (19.1%)	864 (20.4%)
Unsure	1,946 (14.7%)	546 (12.9%)
(Missing)	62 (0.5%)	11 (0.3%)
Faith-sharing, n (%)		
Agree	8,180 (62.0%)	2,630 (62.0%)
Disagree	2,200 (16.7%)	736 (17.4%)
Not relevant	2,105 (15.9%)	698 (16.5%)
Unsure	662 (5.0%)	166 (3.9%)
(Missing)	56 (0.4%)	10 (0.2%)

Note. N (%); this table is based on non-imputed data. Cumulative percentages for variables may not add up to 100% due to rounding. Wave 1 characteristics weighted using the Gallup provided sampling weight, ANNUAL_WEIGHT_R2; Wave 2 characteristics weighted accounting for attrition by using the adjusted Wave 1 weight, ANNUAL_WEIGHT_R2, multiplied by the created attrition weight to account for dropout, to maintain nationally representative estimates for Wave 2 characteristics.

Table S11c. Unweighted summary statistics for demographic and childhood variables in Brazil by retention status

Characteristic	Attritors–Not Observed in Wave 2 N = 8,500	Retained–Observed in Wave 2 N = 4,648
Forgivingness, n (%)		
Always	3,091 (36.4%)	1,701 (36.6%)
Often	2,948 (34.7%)	1,772 (38.1%)
Rarely	1,991 (23.4%)	942 (20.3%)
Never	444 (5.2%)	229 (4.9%)
(Missing)	27 (0.3%)	5 (0.1%)
Year of birth, n (%)		
1943 or earlier (current age: 80+ years)	65 (0.8%)	74 (1.6%)
1943-1953 (current age: 70-79 years)	237 (2.8%)	329 (7.1%)
1953-1963 (current age: 60-69 years)	816 (9.6%)	696 (15.0%)
1963-1973 (current age: 50-59 years)	1,137 (13.4%)	890 (19.1%)
1973-1983 (current age: 40-49 years)	1,534 (18.0%)	972 (20.9%)
1983-1993 (current age: 30-39 years)	1,920 (22.6%)	893 (19.2%)
1993-1998 (current age: 25-29 years)	1,092 (12.8%)	362 (7.8%)
1998-2005 (current age: 18-24 years)	1,700 (20.0%)	433 (9.3%)
(Missing)	0 (0%)	0 (0%)
Age of participant		
Mean	39.4	46.6
Standard Deviation	15.6	16.1
Min, Max	18.0, 93.0	18.0, 92.0
Gender, n (%)		
Male	4,028 (47.4%)	2,284 (49.1%)
Female	4,430 (52.1%)	2,341 (50.4%)
Other	17 (0.2%)	18 (0.4%)
(Missing)	26 (0.3%)	5 (0.1%)
Respondent marital status, n (%)		
Single/Never been married	3,027 (35.6%)	1,356 (29.2%)
Married	2,642 (31.1%)	1,928 (41.5%)
Separated	393 (4.6%)	190 (4.1%)
Divorced	540 (6.4%)	312 (6.7%)
Widowed	228 (2.7%)	211 (4.5%)
Domestic partner	1,453 (17.1%)	602 (13.0%)
(Missing)	217 (2.5%)	48 (1.0%)
Education (years), n (%)		
Up to 8	2,256 (26.5%)	1,054 (22.7%)
9-15	5,076 (59.7%)	2,506 (53.9%)
16+	1,160 (13.6%)	1,086 (23.4%)
(Missing)	8 (0.1%)	3 (0.1%)
Employment status, n (%)		
Employed for an employer	2,291 (27.0%)	1,329 (28.6%)
Self-employed	1,825 (21.5%)	1,040 (22.4%)
Retired	869 (10.2%)	758 (16.3%)
Student	499 (5.9%)	160 (3.4%)
Homemaker	878 (10.3%)	435 (9.4%)
Unemployed and looking for a job	1,683 (19.8%)	734 (15.8%)
None of these/Other	284 (3.3%)	163 (3.5%)
(Missing)	171 (2.0%)	30 (0.6%)
Current religious service attendance, n (%)		
More than once a week	1,479 (17.4%)	912 (19.6%)

Table S11c. Unweighted summary statistics for demographic and childhood variables in Brazil by retention status

**Characteristic**	**Attritors–Not Observed in Wave 2** N = 8,500	**Retained–Observed in Wave 2** N = 4,648
Once a week	1,446 (17.0%)	828 (17.8%)
One to three times a month	933 (11.0%)	465 (10.0%)
A few times a year	2,626 (30.9%)	1,324 (28.5%)
Never	1,974 (23.2%)	1,099 (23.6%)
(Missing)	41 (0.5%)	20 (0.4%)
Immigration status, n (%)		
Born in this country	8,097 (95.3%)	4,531 (97.5%)
Born in another country	97 (1.1%)	55 (1.2%)
(Missing)	306 (3.6%)	62 (1.3%)
Parental marital status around age 12, n (%)		
Parents were married	5,144 (60.5%)	3,311 (71.2%)
Parents were divorced	1,005 (11.8%)	377 (8.1%)
Parents were never married	1,378 (16.2%)	629 (13.5%)
One or both of them had died	328 (3.9%)	183 (3.9%)
Unsure	259 (3.0%)	81 (1.7%)
(Missing)	385 (4.5%)	68 (1.5%)
Religious service attendance around age 12, n (%)		
At least once a week	3,941 (46.4%)	2,329 (50.1%)
One to three times a month	1,723 (20.3%)	774 (16.7%)
Less than once a month	1,699 (20.0%)	904 (19.5%)
Never	1,091 (12.8%)	614 (13.2%)
(Missing)	46 (0.5%)	27 (0.6%)
Relationship with mother when growing up, n (%)		
Very good	5,311 (62.5%)	3,012 (64.8%)
Somewhat good	2,324 (27.3%)	1,213 (26.1%)
Somewhat bad	335 (3.9%)	150 (3.2%)
Very bad	144 (1.7%)	70 (1.5%)
(Does not apply)	337 (4.0%)	177 (3.8%)
(Missing)	49 (0.6%)	26 (0.6%)
Relationship with father when growing up, n (%)		
Very good	4,068 (47.9%)	2,251 (48.4%)
Somewhat good	2,303 (27.1%)	1,321 (28.4%)
Somewhat bad	681 (8.0%)	353 (7.6%)
Very bad	506 (5.9%)	259 (5.6%)
(Does not apply)	894 (10.5%)	419 (9.0%)
(Missing)	49 (0.6%)	45 (1.0%)
Felt like an outsider in family when growing up, n (%)		
Yes	1,139 (13.4%)	543 (11.7%)
No	7,136 (84.0%)	4,017 (86.4%)
(Missing)	224 (2.6%)	88 (1.9%)
Experienced abuse when growing up, n (%)		
Yes	1,644 (19.3%)	946 (20.4%)
No	6,469 (76.1%)	3,635 (78.2%)
(Missing)	387 (4.6%)	67 (1.4%)
Self-rated health when growing up, n (%)		
Excellent	3,433 (40.4%)	1,824 (39.2%)
Very good	2,246 (26.4%)	1,134 (24.4%)

Table S11c. Unweighted summary statistics for demographic and childhood variables in Brazil by retention status

**Characteristic**	**Attritors–Not Observed in Wave 2** N = 8,500	**Retained–Observed in Wave 2** N = 4,648
Good	1,764 (20.8%)	1,108 (23.8%)
Fair	902 (10.6%)	476 (10.2%)
Poor	136 (1.6%)	96 (2.1%)
(Missing)	20 (0.2%)	10 (0.2%)
Subjective financial status of family growing up, n (%)		
Lived comfortably	3,213 (37.8%)	1,758 (37.8%)
Got by	3,041 (35.8%)	1,546 (33.3%)
Found it difficult	1,548 (18.2%)	929 (20.0%)
Found it very difficult	643 (7.6%)	391 (8.4%)
(Missing)	55 (0.6%)	24 (0.5%)
Religious affiliation growing up, n (%)		
Christianity	7,232 (85.1%)	4,113 (88.5%)
Taoism	0 (<0.0%)	0 (<0.0%)
Confucianism	3 (0.0%)	4 (0.1%)
Primal, Animist, or Folk religion	13 (0.1%)	5 (0.1%)
Spiritism	224 (2.6%)	108 (2.3%)
Umbanda, Candomblé, and other African-derived religions	189 (2.2%)	72 (1.6%)
Chinese folk/traditional religion	0 (0%)	0 (0%)
Islam	10 (0.1%)	5 (0.1%)
Hinduism	0 (<0.0%)	0 (<0.0%)
Buddhism	19 (0.2%)	7 (0.2%)
Judaism	34 (0.4%)	8 (0.2%)
Sikhism	0 (0%)	0 (0%)
Baha'i	1 (0.0%)	0 (0%)
Jainism	3 (0.0%)	1 (0.0%)
Shinto	3 (0.0%)	1 (0.0%)
Some other religion	69 (0.8%)	21 (0.5%)
No religion/Atheist/Agnostic	636 (7.5%)	274 (5.9%)
(Missing)	65 (0.8%)	30 (0.6%)

Note. N (%); this table is based on non-imputed data. Cumulative percentages for variables may not add up to 100% due to rounding.

Table S11d. Unweighted summary statistics for Wave 1 outcome variables in Brazil by retention status.

**Outcome**	**Attritors–Not Observed in Wave 2** N = 8,500	**Retained–Observed in Wave 2** N = 4,648
Secure flourishing index		
Mean	7.0	7.1
Standard Deviation	1.7	1.6
Min, Max	0.0, 10.0	0.8, 10.0
(Missing)	221 (2.6%)	96 (2.1%)
Flourishing index		
Mean	7.6	7.7
Standard Deviation	1.8	1.7
Min, Max	0.0, 10.0	1.0, 10.0
(Missing)	202 (2.4%)	88 (1.9%)
Happiness & life satisfaction		
Mean	7.2	7.3
Standard Deviation	2.3	2.1
Min, Max	0.0, 10.0	0.0, 10.0
(Missing)	47 (0.6%)	19 (0.4%)
Physical & mental health		
Mean	7.3	7.3
Standard Deviation	2.2	2.0
Min, Max	0.0, 10.0	0.0, 10.0
(Missing)	35 (0.4%)	16 (0.3%)
Meaning & purpose		
Mean	7.8	7.9
Standard Deviation	2.1	2.1
Min, Max	0.0, 10.0	0.0, 10.0
(Missing)	34 (0.4%)	29 (0.6%)
Character & virtue		
Mean	8.3	8.3
Standard Deviation	1.8	1.7
Min, Max	0.0, 10.0	0.0, 10.0
(Missing)	59 (0.7%)	27 (0.6%)
Close social relationships		
Mean	7.4	7.5
Standard Deviation	2.6	2.5
Min, Max	0.0, 10.0	0.0, 10.0
(Missing)	60 (0.7%)	24 (0.5%)
Financial & material security		
Mean	4.0	4.0
Standard Deviation	3.6	3.5
Min, Max	0.0, 10.0	0.0, 10.0
(Missing)	30 (0.4%)	17 (0.4%)
Happiness		
Mean	7.3	7.4
Standard Deviation	2.4	2.2
Min, Max	0.0, 10.0	0.0, 10.0
(Missing)	31 (0.4%)	7 (0.1%)
Life satisfaction		
Mean	7.1	7.2
Standard Deviation	2.5	2.4
Min, Max	0.0, 10.0	0.0, 10.0

Table S11d. Unweighted summary statistics for Wave 1 outcome variables in Brazil by retention status.

Outcome	Attritors–Not Observed in Wave 2 N = 8,500	Retained–Observed in Wave 2 N = 4,648
(Missing)	21 (0.2%)	13 (0.3%)
Current life evaluation		
Mean	6.5	6.7
Standard Deviation	2.3	2.2
Min, Max	0.0, 10.0	0.0, 10.0
(Missing)	12 (0.1%)	6 (0.1%)
Future life evaluation		
Mean	8.7	8.5
Standard Deviation	1.8	1.9
Min, Max	0.0, 10.0	0.0, 10.0
(Missing)	95 (1.1%)	54 (1.2%)
Optimism		
Mean	9.2	9.2
Standard Deviation	1.6	1.6
Min, Max	0.0, 10.0	0.0, 10.0
(Missing)	16 (0.2%)	12 (0.3%)
Freedom to pursue what's important		
Mean	8.3	8.3
Standard Deviation	2.4	2.3
Min, Max	0.0, 10.0	0.0, 10.0
(Missing)	16 (0.2%)	20 (0.4%)
Inner peace, n (%)		
Always	2,083 (24.5%)	1,172 (25.2%)
Often	3,826 (45.0%)	2,228 (47.9%)
Rarely	2,265 (26.6%)	1,077 (23.2%)
Never	289 (3.4%)	163 (3.5%)
(Missing)	37 (0.4%)	9 (0.2%)
Life balance, n (%)		
Always	1,477 (17.4%)	783 (16.8%)
Often	4,141 (48.7%)	2,402 (51.7%)
Rarely	2,552 (30.0%)	1,305 (28.1%)
Never	307 (3.6%)	148 (3.2%)
(Missing)	23 (0.3%)	10 (0.2%)
Sense of mastery, n (%)		
Always	2,796 (32.9%)	1,542 (33.2%)
Often	3,935 (46.3%)	2,259 (48.6%)
Rarely	1,539 (18.1%)	734 (15.8%)
Never	162 (1.9%)	90 (1.9%)
(Missing)	68 (0.8%)	23 (0.5%)
Meaningful activities		
Mean	7.8	7.9
Standard Deviation	2.3	2.2
Min, Max	0.0, 10.0	0.0, 10.0
(Missing)	17 (0.2%)	7 (0.1%)
Understanding purpose		
Mean	7.8	7.8
Standard Deviation	2.5	2.5
Min, Max	0.0, 10.0	0.0, 10.0
(Missing)	22 (0.3%)	22 (0.5%)

Table S11d. Unweighted summary statistics for Wave 1 outcome variables in Brazil by retention status.

Outcome	Attritors–Not Observed in Wave 2<br>N = 8,500	Retained–Observed in Wave 2<br>N = 4,648
Self-rated mental health		
Mean	7.3	7.5
Standard Deviation	2.7	2.5
Min, Max	0.0, 10.0	0.0, 10.0
(Missing)	15 (0.2%)	8 (0.2%)
Traumatic distress, n (%)		
A lot	1,773 (20.9%)	904 (19.5%)
Some	2,642 (31.1%)	1,399 (30.1%)
Not very much	1,944 (22.9%)	1,067 (23.0%)
Not at all	2,095 (24.7%)	1,257 (27.0%)
(Missing)	46 (0.5%)	21 (0.5%)
Depression symptoms composite, n (%)	3,687 (43.7%)	1,666 (36.0%)
(Missing)	69 (0.8%)	23 (0.5%)
Depression – feel hopeless, n (%)		
Nearly every day	1,394 (16.4%)	616 (13.2%)
More than half the days	1,359 (16.0%)	598 (12.9%)
Several days	2,210 (26.0%)	1,279 (27.5%)
Not at all	3,508 (41.3%)	2,143 (46.1%)
(Missing)	29 (0.3%)	12 (0.3%)
Depression – loss of interest, n (%)		
Nearly every day	1,733 (20.4%)	814 (17.5%)
More than half the days	1,757 (20.7%)	787 (16.9%)
Several days	2,239 (26.3%)	1,292 (27.8%)
Not at all	2,724 (32.0%)	1,741 (37.5%)
(Missing)	47 (0.5%)	14 (0.3%)
Anxiety symptoms composite, n (%)	4,141 (49.0%)	1,955 (42.2%)
(Missing)	56 (0.7%)	13 (0.3%)
Anxiety – feel on edge, n (%)		
Nearly every day	2,182 (25.7%)	1,000 (21.5%)
More than half the days	1,408 (16.6%)	722 (15.5%)
Several days	2,578 (30.3%)	1,477 (31.8%)
Not at all	2,299 (27.0%)	1,438 (30.9%)
(Missing)	33 (0.4%)	11 (0.2%)
Anxiety – cannot stop worrying, n (%)		
Nearly every day	2,134 (25.1%)	994 (21.4%)
More than half the days	1,458 (17.2%)	626 (13.5%)
Several days	2,131 (25.1%)	1,348 (29.0%)
Not at all	2,738 (32.2%)	1,675 (36.0%)
(Missing)	38 (0.4%)	5 (0.1%)
Suffering, n (%)		
A lot	1,047 (12.3%)	518 (11.1%)
Some	3,678 (43.3%)	2,035 (43.8%)
Not very much	1,996 (23.5%)	1,073 (23.1%)
Not at all	1,727 (20.3%)	1,007 (21.7%)
(Missing)	52 (0.6%)	16 (0.3%)
Relationship contentment		
Mean	7.7	7.7
Standard Deviation	2.7	2.6
Min, Max	0.0, 10.0	0.0, 10.0

Table S11d. Unweighted summary statistics for Wave 1 outcome variables in Brazil by retention status.

Outcome	Attritors–Not Observed in Wave 2 N = 8,500	Retained–Observed in Wave 2 N = 4,648
(Missing)	29 (0.3%)	14 (0.3%)
Relationship satisfaction		
Mean	7.2	7.3
Standard Deviation	2.9	2.8
Min, Max	0.0, 10.0	0.0, 10.0
(Missing)	40 (0.5%)	15 (0.3%)
Social support		
Mean	7.2	7.3
Standard Deviation	3.0	2.9
Min, Max	0.0, 10.0	0.0, 10.0
(Missing)	43 (0.5%)	10 (0.2%)
Intimate/close friend, n (%)		
Yes	6,966 (82.0%)	3,740 (80.5%)
No	1,443 (17.0%)	887 (19.1%)
(Missing)	91 (1.1%)	21 (0.5%)
Government approval, n (%)		
Strongly approve	1,022 (12.0%)	621 (13.4%)
Somewhat approve	1,911 (22.5%)	1,055 (22.7%)
Neither approve nor disapprove	2,299 (27.0%)	943 (20.3%)
Somewhat disapprove	1,315 (15.5%)	761 (16.4%)
Strongly disapprove	1,893 (22.3%)	1,244 (26.8%)
(Missing)	61 (0.7%)	24 (0.5%)
Say in government, n (%)		
Agree	2,367 (27.8%)	1,190 (25.6%)
Disagree	3,608 (42.5%)	2,296 (49.4%)
Unsure	2,446 (28.8%)	1,148 (24.7%)
(Missing)	79 (0.9%)	14 (0.3%)
Belonging in country		
Mean	7.8	7.8
Standard Deviation	2.6	2.6
Min, Max	0.0, 10.0	0.0, 10.0
(Missing)	106 (1.2%)	63 (1.4%)
City/place satisfaction, n (%)		
Satisfied	5,165 (60.8%)	2,975 (64.0%)
Dissatisfied	2,213 (26.0%)	1,222 (26.3%)
Unsure	1,039 (12.2%)	430 (9.3%)
(Missing)	83 (1.0%)	21 (0.4%)
Trust within country, n (%)		
All people	128 (1.5%)	43 (0.9%)
Most people	669 (7.9%)	433 (9.3%)
Some people	3,066 (36.1%)	1,708 (36.8%)
Not very many people	3,674 (43.2%)	1,988 (42.8%)
None	898 (10.6%)	456 (9.8%)
(Missing)	64 (0.8%)	19 (0.4%)
Number of children		
Mean	1.0	0.8
Standard Deviation	1.5	1.1
Min, Max	0.0, 97.0	0.0, 26.0
(Missing)	259 (3.0%)	60 (1.3%)

Table S11d. Unweighted summary statistics for Wave 1 outcome variables in Brazil by retention status.

**Outcome**	**Attritors–Not Observed in Wave 2** N = 8,500	**Retained–Observed in Wave 2** N = 4,648
Community participation, n (%)		
More than once a week	780 (9.2%)	419 (9.0%)
Once a week	802 (9.4%)	387 (8.3%)
One to three times a month	626 (7.4%)	324 (7.0%)
A few times a year	2,052 (24.1%)	1,178 (25.4%)
Never	4,177 (49.1%)	2,320 (49.9%)
(Missing)	62 (0.7%)	19 (0.4%)
Religious attendance, n (%)		
More than once a week	1,479 (17.4%)	912 (19.6%)
Once a week	1,446 (17.0%)	828 (17.8%)
One to three times a month	933 (11.0%)	465 (10.0%)
A few times a year	2,626 (30.9%)	1,324 (28.5%)
Never	1,974 (23.2%)	1,099 (23.6%)
(Missing)	41 (0.5%)	20 (0.4%)
Loneliness		
Mean	4.0	3.8
Standard Deviation	3.5	3.4
Min, Max	0.0, 10.0	0.0, 10.0
(Missing)	14 (0.2%)	8 (0.2%)
Perceived discrimination, n (%)		
Always	954 (11.2%)	468 (10.1%)
Often	1,758 (20.7%)	877 (18.9%)
Rarely	3,160 (37.2%)	1,828 (39.3%)
Never	2,601 (30.6%)	1,464 (31.5%)
(Missing)	26 (0.3%)	12 (0.3%)
Orientation to promote good		
Mean	8.6	8.6
Standard Deviation	1.8	1.8
Min, Max	0.0, 10.0	0.0, 10.0
(Missing)	37 (0.4%)	13 (0.3%)
Delayed gratification		
Mean	8.1	8.1
Standard Deviation	2.3	2.2
Min, Max	0.0, 10.0	0.0, 10.0
(Missing)	25 (0.3%)	15 (0.3%)
Hope		
Mean	8.9	8.8
Standard Deviation	1.9	1.9
Min, Max	0.0, 10.0	0.0, 10.0
(Missing)	21 (0.2%)	8 (0.2%)
Gratitude		
Mean	8.6	8.6
Standard Deviation	2.1	2.0
Min, Max	0.0, 10.0	0.0, 10.0
(Missing)	21 (0.3%)	15 (0.3%)
Showing love/care		
Mean	8.5	8.5
Standard Deviation	2.2	2.1
Min, Max	0.0, 10.0	0.0, 10.0

Table S11d. Unweighted summary statistics for Wave 1 outcome variables in Brazil by retention status.

**Outcome**	**Attritors–Not Observed in Wave 2** N = 8,500	**Retained–Observed in Wave 2** N = 4,648
(Missing)	16 (0.2%)	10 (0.2%)
Forgivingness, n (%)		
Always	3,091 (36.4%)	1,701 (36.6%)
Often	2,948 (34.7%)	1,772 (38.1%)
Rarely	1,991 (23.4%)	942 (20.3%)
Never	444 (5.2%)	229 (4.9%)
(Missing)	27 (0.3%)	5 (0.1%)
Charitable giving, n (%)		
Yes	2,446 (28.8%)	1,625 (35.0%)
No	6,039 (71.1%)	3,006 (64.7%)
(Missing)	15 (0.2%)	17 (0.4%)
Helping strangers, n (%)		
Yes	5,843 (68.7%)	3,136 (67.5%)
No	2,605 (30.6%)	1,488 (32.0%)
(Missing)	51 (0.6%)	24 (0.5%)
Volunteering, n (%)		
Yes	1,585 (18.6%)	854 (18.4%)
No	6,889 (81.1%)	3,779 (81.3%)
(Missing)	26 (0.3%)	15 (0.3%)
Self-rated physical health		
Mean	7.2	7.1
Standard Deviation	2.4	2.2
Min, Max	0.0, 10.0	0.0, 10.0
(Missing)	21 (0.3%)	11 (0.2%)
Health problems, n (%)		
Yes	1,558 (18.3%)	953 (20.5%)
No	6,659 (78.3%)	3,642 (78.4%)
(Missing)	283 (3.3%)	53 (1.1%)
Pain in past 4 weeks, n (%)		
A lot	1,568 (18.5%)	817 (17.6%)
Some	3,440 (40.5%)	1,924 (41.4%)
Not very much	1,911 (22.5%)	1,117 (24.0%)
None at all	1,559 (18.3%)	787 (16.9%)
(Missing)	22 (0.3%)	3 (0.1%)
Number of cigarettes per day		
Mean	2.6	2.1
Standard Deviation	6.8	5.8
Min, Max	0.0, 97.0	0.0, 97.0
(Missing)	183 (2.2%)	58 (1.2%)
Number of drinks per week		
Mean	2.0	2.0
Standard Deviation	5.9	5.4
Min, Max	0.0, 97.0	0.0, 97.0
(Missing)	170 (2.0%)	51 (1.1%)
Days exercise per week		
Mean	2.1	2.2
Standard Deviation	2.4	2.4
Min, Max	0.0, 7.0	0.0, 7.0
(Missing)	33 (0.4%)	16 (0.3%)

Table S11d. Unweighted summary statistics for Wave 1 outcome variables in Brazil by retention status.

Outcome	Attritors–Not Observed in Wave 2 N = 8,500	Retained–Observed in Wave 2 N = 4,648
Financial security		
Mean	4.0	4.0
Standard Deviation	3.8	3.7
Min, Max	0.0, 10.0	0.0, 10.0
(Missing)	15 (0.2%)	9 (0.2%)
Material security		
Mean	3.9	3.9
Standard Deviation	3.9	3.8
Min, Max	0.0, 10.0	0.0, 10.0
(Missing)	16 (0.2%)	8 (0.2%)
Educational attainment (16+ years), n (%)		
Up to 8	2,256 (26.5%)	1,054 (22.7%)
9-15	5,076 (59.7%)	2,506 (53.9%)
16+	1,160 (13.6%)	1,086 (23.4%)
(Missing)	8 (0.1%)	3 (0.1%)
Currently employed, n (%)		
Employed for an employer	2,291 (27.0%)	1,329 (28.6%)
Self-employed	1,825 (21.5%)	1,040 (22.4%)
Retired	869 (10.2%)	758 (16.3%)
Student	499 (5.9%)	160 (3.4%)
Homemaker	878 (10.3%)	435 (9.4%)
Unemployed and looking for a job	1,683 (19.8%)	734 (15.8%)
None of these/Other	284 (3.3%)	163 (3.5%)
(Missing)	171 (2.0%)	30 (0.6%)
Financially comfortable/getting by, n (%)		
Living comfortably on present income	1,217 (14.3%)	774 (16.7%)
Getting by on present income	3,519 (41.4%)	2,179 (46.9%)
Finding it difficult on present income	2,502 (29.4%)	1,172 (25.2%)
Finding it very difficult on present income	871 (10.2%)	457 (9.8%)
(Missing)	391 (4.6%)	66 (1.4%)
Own home, n (%)		
Someone in this household owns this home	4,330 (50.9%)	2,951 (63.5%)
Someone in this household rents this home	2,286 (26.9%)	1,045 (22.5%)
Both	191 (2.2%)	75 (1.6%)
Neither	1,293 (15.2%)	518 (11.1%)
Rent	0 (0%)	0 (0%)
Own	0 (0%)	0 (0%)
Something else	0 (0%)	0 (0%)
(Missing)	400 (4.7%)	59 (1.3%)
Religious/spiritual connection, n (%)		
Always	3,699 (43.5%)	2,220 (47.8%)
Often	2,783 (32.7%)	1,428 (30.7%)
Rarely	1,572 (18.5%)	756 (16.3%)
Never	425 (5.0%)	236 (5.1%)
(Missing)	21 (0.2%)	9 (0.2%)
Belief in life after death, n (%)		
Yes	5,156 (60.7%)	2,791 (60.1%)
No	1,844 (21.7%)	1,001 (21.5%)
Unsure	1,456 (17.1%)	838 (18.0%)

Table S11d. Unweighted summary statistics for Wave 1 outcome variables in Brazil by retention status.

Outcome	Attritors–Not Observed in Wave 2<br>N = 8,500	Retained–Observed in Wave 2<br>N = 4,648
(Missing)	44 (0.5%)	18 (0.4%)
Transformative religious experience, n (%)		
Yes	5,078 (59.7%)	2,692 (57.9%)
No	3,352 (39.4%)	1,935 (41.6%)
(Missing)	69 (0.8%)	21 (0.5%)
Religious reading or listening, n (%)		
More than once a day	1,429 (16.8%)	824 (17.7%)
About once a day	1,760 (20.7%)	1,119 (24.1%)
Sometimes	4,210 (49.5%)	2,093 (45.0%)
Never	1,011 (11.9%)	570 (12.3%)
(Missing)	90 (1.1%)	42 (0.9%)
Prayer or meditation, n (%)		
More than once a day	2,515 (29.6%)	1,521 (32.7%)
About once a day	2,782 (32.7%)	1,595 (34.3%)
Sometimes	2,538 (29.9%)	1,191 (25.6%)
Never	566 (6.7%)	299 (6.4%)
(Missing)	100 (1.2%)	41 (0.9%)
Belief in God/gods/spiritual forces, n (%)		
One God	7,387 (86.9%)	4,023 (86.6%)
More than one god	233 (2.7%)	95 (2.0%)
An impersonal spiritual force	477 (5.6%)	327 (7.0%)
None of these	171 (2.0%)	87 (1.9%)
Unsure	172 (2.0%)	95 (2.1%)
(Missing)	61 (0.7%)	20 (0.4%)
Religious centrality, n (%)		
Agree	5,411 (63.7%)	3,111 (66.9%)
Disagree	892 (10.5%)	487 (10.5%)
Not relevant	1,074 (12.6%)	577 (12.4%)
Unsure	1,060 (12.5%)	458 (9.9%)
(Missing)	64 (0.7%)	15 (0.3%)
Religious/spiritual comfort, n (%)		
Agree	6,598 (77.6%)	3,731 (80.3%)
Disagree	541 (6.4%)	272 (5.9%)
Not relevant	732 (8.6%)	379 (8.2%)
Unsure	593 (7.0%)	257 (5.5%)
(Missing)	35 (0.4%)	10 (0.2%)
Feel loved by God, n (%)		
Agree	7,356 (86.5%)	4,050 (87.1%)
Disagree	274 (3.2%)	183 (3.9%)
Not relevant	465 (5.5%)	210 (4.5%)
Unsure	362 (4.3%)	189 (4.1%)
(Missing)	43 (0.5%)	16 (0.4%)
Feel punished by God, n (%)		
Agree	1,602 (18.9%)	822 (17.7%)
Disagree	4,871 (57.3%)	2,882 (62.0%)
Not relevant	1,009 (11.9%)	526 (11.3%)
Unsure	967 (11.4%)	399 (8.6%)
(Missing)	51 (0.6%)	19 (0.4%)
Experienced religious criticism, n (%)		

Table S11d. Unweighted summary statistics for Wave 1 outcome variables in Brazil by retention status.

Outcome	Attritors–Not Observed in Wave 2<br>N = 8,500	Retained–Observed in Wave 2<br>N = 4,648
Agree	1,568 (18.4%)	747 (16.1%)
Disagree	3,965 (46.6%)	2,357 (50.7%)
Not relevant	1,617 (19.0%)	897 (19.3%)
Unsure	1,299 (15.3%)	638 (13.7%)
(Missing)	52 (0.6%)	10 (0.2%)
Faith-sharing, n (%)		
Agree	5,213 (61.3%)	2,934 (63.1%)
Disagree	1,426 (16.8%)	764 (16.4%)
Not relevant	1,309 (15.4%)	788 (17.0%)
Unsure	511 (6.0%)	146 (3.1%)
(Missing)	41 (0.5%)	15 (0.3%)

Note. N (%); this table is based on non-imputed data. Cumulative percentages for variables may not add up to 100% due to rounding.

Table S11e. Summary of fitted attrition model in Brazil

Characteristic	Odds Ratio	95% CI	p-value
**ANNUAL_WEIGHT_R2**	1.02	0.96, 1.08	0.525
**Recruitment Survey Mode**			
CAWI	—	—	
CATI	1.99	1.77, 2.25	3.81e-29
CAPI	1.33	1.11, 1.60	0.002
**Happiness & life satisfaction**	1.02	0.94, 1.11	0.654
**Physical & mental health**	0.96	0.89, 1.03	0.286
**Meaning & purpose**	0.93	0.86, 1.01	0.108
**Character & virtue**	0.96	0.90, 1.02	0.165
**Close social relationships**	1.00	0.93, 1.08	0.966
**Financial & material security**	0.96	0.91, 1.01	0.146
**Extraversion**	0.95	0.90, 1.00	0.060
**Openness to experience**	0.98	0.92, 1.04	0.433
**Agreeableness**	1.03	0.98, 1.10	0.239
**Conscientiousness**	1.05	0.99, 1.11	0.122
**Neuroticism**	0.98	0.92, 1.05	0.556
**Depression symptoms composite**	1.0	0.93, 1.06	0.873
**Anxiety symptoms composite**	0.97	0.92, 1.04	0.429
**Loneliness**	1.00	0.94, 1.07	0.970
**Days exercise per week**	1.05	1.00, 1.11	0.064
**Year of birth (age group)**			
1983-1993 (current age: 30-39 years)	—	—	
1973-1983 (current age: 40-49 years)	1.33	1.15, 1.54	9.22e-05
1998-2005 (current age: 18-24 years)	0.57	0.48, 0.68	4.33e-10
1993-1998 (current age: 25-29 years)	0.73	0.62, 0.87	4.90e-04
1963-1973 (current age: 50-59 years)	1.68	1.42, 1.98	7.78e-10
1953-1963 (current age: 60-69 years)	1.76	1.37, 2.26	8.19e-06
1943-1953 (current age: 70-79 years)	2.58	1.73, 3.85	3.74e-06
1943 or earlier (current age: 80+ years)	2.20	1.13, 4.27	0.020
**Gender of respondent**			
Female	—	—	
Male	0.96	0.86, 1.08	0.537
Other	1.88	0.93, 3.81	0.078
**Marital status**			
Single/Never been married	—	—	
Married	1.07	0.94, 1.23	0.306
Domestic partner	0.89	0.76, 1.04	0.131
Divorced	0.80	0.63, 1.00	0.054
Separated	0.80	0.61, 1.06	0.123
Widowed	0.98	0.70, 1.39	0.924
**Employment status**			
Employed for an employer	—	—	
Self-employed	1.01	0.88, 1.16	0.877
Unemployed and looking for a job	1.11	0.95, 1.31	0.188
Homemaker	1.0	0.81, 1.23	0.962
Retired	0.78	0.61, 1.01	0.061
Student	1.21	0.92, 1.58	0.165
None of these/Other	0.88	0.66, 1.18	0.402
**Religious attendance**			
A few times a year	—	—	
Never	1.02	0.89, 1.17	0.781
More than once a week	1.15	0.99, 1.35	0.075

Table S11e. Summary of fitted attrition model in Brazil

Characteristic	Odds Ratio	95% CI	p-value
Once a week	1.04	0.89, 1.21	0.647
One to three times a month	0.98	0.83, 1.17	0.852
**Educational attainment (16+ years)**			
9-15	—	—	
16+	1.15	1.00, 1.32	0.057
Up to 8	0.88	0.76, 1.01	0.077
**Born in This country**			
Born in this country	—	—	
Born in another country	1.05	0.63, 1.76	0.854
**Race plurality (prominent race/ethnic group [0] or not [1])**	0.93	0.89, 0.99	0.013
**Urbanicity**			
A large city	—	—	
A small town or village	0.83	0.73, 0.93	0.002
A suburb of a large city	0.99	0.82, 1.18	0.876
A rural area or on a farm	0.94	0.77, 1.14	0.525
**Monthly household income**			
Brazil: 1,001 – 1,500 reals	—	—	
Brazil: 1,501 – 2,000 reals	1.16	0.94, 1.42	0.158
Brazil: 2,501 – 3,000 reals	1.32	1.06, 1.65	0.015
Brazil: 2,001 – 2,500 reals	1.18	0.95, 1.47	0.141
Brazil: 3,001 – 4,000 reals	1.52	1.22, 1.89	2.17e-04
(None/No household income)	0.82	0.63, 1.06	0.128
Brazil: 751 – 1,000 reals	1.03	0.81, 1.31	0.822
Brazil: 4,001 – 5,000 reals	1.59	1.24, 2.03	2.20e-04
Brazil: 501 – 750 reals	1.19	0.90, 1.59	0.226
Brazil: 5,001 – 6,000 reals	1.60	1.21, 2.13	0.001
Brazil: 101 – 500 reals	0.81	0.60, 1.09	0.165
Brazil: 6,001 – 8,000 reals	1.56	1.17, 2.08	0.003
Brazil: 10,001 – 15,000 reals	2.48	1.78, 3.45	6.84e-08
Brazil: 8,001 – 10,000 reals	1.78	1.30, 2.44	3.70e-04
Brazil: 100 reals or less	0.70	0.48, 1.03	0.074
Brazil: More than 15,000 reals	1.76	1.23, 2.52	0.002

Abbreviations: CI = Confidence Interval, OR = Odds Ratio

Notes. N=13203; attrition weights were estimated using the ’survey::svyglm(family=quasibinomial(‘logit’))’ function. All continuous predictors were standardized and all categorical predictors used the most common category as the reference group. Reported p-values are based on the fitted regression model and no adjustments for multiple testing were done within this table.

Table S11f. Summary of principal components in Brazil

PC	Percent Explained by each PC	Cumulative Percent Explained
1	34.42	34.42
2	6.04	40.45
3	3.60	44.06
4	2.39	46.45
5	2.20	48.65
6	1.69	50.34
**7**	**1.60**	**51.94**
8	1.57	53.50
9	1.51	55.02
10	1.38	56.39
11	1.28	57.68
12	1.26	58.94
13	1.22	60.16
14	1.19	61.35
15	1.13	62.49
16	1.10	63.58
17	1.06	64.64
18	1.05	65.69
19	1.03	66.72
20	1.00	67.72

Notes. N=13203; PCA was conducted using 'survey::svyprcomp(.)' function using all available contemporaneous exposures at wave 1. All PCs were standardized prior to being used as predictors. The bolded row represented the number of retained components for analysis was 7.

Table S11g. Associations of forgivingness with adult well-being and other outcomes at Wave 2 in Brazil

	Model 1: Demographic and Childhood Variables as Covariates					Model 2: Demographic, Childhood, and Other Wave 1 Confounding Variables (Via Principal Components) as Covariates				
Outcome	RR	ES	95% CI	SE	p-value	RR	ES	95% CI	SE	p-value
Human Flourishing										
Secure flourishing index		0.09	(0.06,0.11)	0.01	8.56e-11***		0.01	(-0.01,0.04)	0.01	0.192
Flourishing index		0.09	(0.06,0.11)	0.01	8.31e-11***		0.01	(-0.01,0.03)	0.01	0.379
Happiness & life satisfaction		0.04	(0.01,0.07)	0.02	0.007*		-0.02	(-0.04,0.01)	0.01	0.244
Physical & mental health		0.06	(0.03,0.10)	0.02	6.16e-04***		0.00	(-0.03,0.04)	0.02	0.766
Meaning & purpose		0.09	(0.06,0.12)	0.02	2.43e-07***		0.02	(-0.01,0.05)	0.01	0.112
Character & virtue		0.08	(0.05,0.11)	0.02	2.82e-07***		0.04	(0.01,0.07)	0.02	0.016*
Close social relationships		0.05	(0.02,0.07)	0.01	6.38e-04***		-0.01	(-0.03,0.02)	0.01	0.627
Financial & material security		0.04	(0.01,0.07)	0.02	0.026*		0.02	(-0.01,0.05)	0.02	0.274
Psychological Well-Being										
Happiness (a)		0.04	(0.00,0.07)	0.02	0.050*		-0.01	(-0.05,0.02)	0.02	0.463
Life satisfaction (a)		0.04	(0.00,0.07)	0.02	0.032*		-0.01	(-0.04,0.01)	0.02	0.324
Current life evaluation		-0.01	(-0.04,0.03)	0.02	0.740		-0.04	(-0.07,-0.01)	0.02	0.008*
Future life evaluation		0.00	(-0.03,0.04)	0.02	0.861		-0.03	(-0.06,0.01)	0.02	0.149
Optimism		0.04	(0.01,0.07)	0.02	0.013*		0.00	(-0.03,0.03)	0.02	0.977
Freedom to pursue what's important		0.03	(-0.00,0.07)	0.02	0.081		-0.01	(-0.05,0.02)	0.02	0.411
Inner peace	1.04		(1.02,1.05)	0.01	3.64e-06***	1.02		(1.00,1.03)	0.01	0.029*
Life balance	1.04		(1.02,1.05)	0.01	3.46e-05***	1.01		(1.00,1.03)	0.01	0.107
Sense of mastery	1.02		(1.01,1.03)	0.01	1.02e-03**	1.00		(0.99,1.02)	0.01	0.510
Meaningful activities (c)		0.07	(0.04,0.10)	0.02	2.35e-05***		0.02	(-0.01,0.05)	0.02	0.236
Understanding purpose (c)		0.08	(0.04,0.11)	0.02	5.21e-05***		0.02	(-0.01,0.05)	0.02	0.212
Self-rated mental health (b)		0.07	(0.04,0.10)	0.01	1.99e-06***		0.02	(-0.01,0.04)	0.01	0.161
Psychological Distress										
Traumatic distress	0.98		(0.96,1.00)	0.01	0.024*	0.98		(0.96,1.00)	0.01	0.056
Depression symptoms composite	0.98		(0.96,0.99)	0.01	0.008*	0.99		(0.98,1.01)	0.01	0.447
Depression – feel hopeless	0.98		(0.97,0.99)	0.01	4.6e-03**	0.99		(0.98,1.01)	0.01	0.257
Depression – loss of interest	0.98		(0.97,1.00)	0.01	0.040*	0.99		(0.98,1.01)	0.01	0.410
Anxiety symptoms composite	0.98		(0.97,0.99)	0.01	3.69e-03**	1.00		(0.98,1.01)	0.01	0.521
Anxiety – feel on edge	0.98		(0.97,1.00)	0.01	0.020*	1.00		(0.98,1.01)	0.01	0.595
Anxiety – cannot stop worrying	0.98		(0.97,1.00)	0.01	0.033*	1.00		(0.98,1.01)	0.01	0.641
Suffering	0.97		(0.96,0.98)	0.01	1.22e-04***	0.98		(0.97,1.00)	0.01	0.032*
Social Well-Being										
Relationship contentment (e)		0.04	(0.01,0.07)	0.02	0.026*		-0.01	(-0.04,0.02)	0.02	0.569
Relationship satisfaction (e)		0.04	(0.01,0.07)	0.02	0.011*		-0.00	(-0.03,0.03)	0.02	0.917
Social support		0.07	(0.03,0.10)	0.02	1.99e-04***		0.04	(0.00,0.07)	0.02	0.041*
Intimate/close friend	1.02		(1.00,1.03)	0.01	0.016*	1.01		(0.99,1.02)	0.01	0.357
Government approval	1.00		(0.99,1.02)	0.01	0.820	0.99		(0.98,1.01)	0.01	0.452
Say in government	1.00		(0.98,1.01)	0.01	0.609	0.99		(0.97,1.00)	0.01	0.160
Belonging in country		0.08	(0.05,0.11)	0.02	3.74e-06***		0.04	(0.01,0.07)	0.02	0.023*
City/place satisfaction	1.01		(0.99,1.03)	0.01	0.239	1.00		(0.98,1.02)	0.01	0.958
Trust within country	1.02		(1.01,1.04)	0.01	0.010*	1.01		(1.00,1.03)	0.01	0.147
Social Participation										
Ever been married	1.00		(0.99,1.01)	0.01	0.854	1.00		(0.99,1.01)	0.01	0.873
Currently divorced	1.00		(0.99,1.01)	0.00	0.944	1.00		(0.99,1.01)	0.00	0.912
Number of children		0.00	(-0.03,0.03)	0.01	0.863		0.00	(-0.03,0.03)	0.01	0.832
Weekly+ community participation	1.01		(0.99,1.02)	0.01	0.343	1.00		(0.99,1.01)	0.01	0.916
Weekly+ religious attendance	1.02		(1.01,1.04)	0.01	8.13e-04**	1.01		(1.00,1.03)	0.01	0.038*
Social Distress										
Loneliness		-0.05	(-0.09,-0.01)	0.02	0.015*		-0.01	(-0.05,0.03)	0.02	0.667
Perceived discrimination	0.99		(0.98,1.01)	0.01	0.297	0.99		(0.98,1.01)	0.01	0.437
Character & Prosocial Behavior										
Orientation to promote good (d)		0.09	(0.06,0.12)	0.02	3.88e-07***		0.05	(0.02,0.08)	0.02	2.93e-03**
Delayed gratification (d)		0.05	(0.01,0.08)	0.02	4.15e-03**		0.01	(-0.02,0.04)	0.02	0.451
Hope		0.07	(0.04,0.11)	0.02	2.05e-04***		0.02	(-0.01,0.06)	0.02	0.189
Gratitude		0.09	(0.05,0.12)	0.02	1.99e-06***		0.04	(0.01,0.07)	0.02	0.026*
Showing love/care		0.07	(0.04,0.10)	0.01	2.77e-06***		0.03	(0.00,0.06)	0.01	0.031*
Forgivingness	1.22		(1.20,1.24)	0.01	2.22e-16***	1.21		(1.19,1.24)	0.01	2.22e-16***
Charitable giving	1.01		(1.00,1.03)	0.01	0.030*	1.00		(0.99,1.01)	0.01	0.839
Helping strangers	1.03		(1.01,1.04)	0.01	1.22e-03**	1.01		(1.00,1.03)	0.01	0.126
Volunteering	1.01		(0.99,1.02)	0.01	0.398	1.00		(0.98,1.01)	0.01	0.505
Physical Health & Health Behavior										
Self-rated physical health (b)		0.03	(-0.01,0.07)	0.02	0.139		-0.01	(-0.05,0.03)	0.02	0.559
Health problems	1.00		(0.99,1.02)	0.01	0.619	1.01		(0.99,1.02)	0.01	0.438
Pain in past 4 weeks	1.00		(0.99,1.02)	0.01	0.868	1.01		(0.99,1.02)	0.01	0.304
Daily smoker	1.00		(0.99,1.01)	0.01	0.584	1.00		(0.99,1.01)	0.01	0.769
Number of drinks per week		-0.02	(-0.05,0.01)	0.02	0.176		-0.01	(-0.04,0.03)	0.02	0.762
Days exercise per week		0.01	(-0.03,0.05)	0.02	0.628		-0.02	(-0.05,0.02)	0.02	0.419
Socioeconomic Outcomes										
Financial security (f)		0.03	(-0.00,0.07)	0.02	0.069		0.01	(-0.02,0.05)	0.02	0.536
Material security (f)		0.03	(-0.00,0.06)	0.02	0.083		0.02	(-0.02,0.05)	0.02	0.303
Educational attainment (16+ years)	1.00		(1.00,1.01)	0.00	0.118	1.00		(1.00,1.01)	0.00	0.149
Currently employed	1.00		(0.99,1.01)	0.01	0.854	1.00		(0.99,1.01)	0.01	0.992
Financially comfortable/getting by	1.00		(0.98,1.01)	0.01	0.511	0.99		(0.97,1.00)	0.01	0.066
Own home	1.00		(0.98,1.02)	0.01	0.964	1.00		(0.98,1.02)	0.01	0.850
Income – top quintile	1.00		(0.99,1.02)	0.01	0.449	1.01		(0.99,1.02)	0.01	0.375
Religion & Spirituality										
Religious/spiritual connection	1.02		(1.01,1.04)	0.01	3.16e-04***	1.00		(0.99,1.01)	0.01	0.771
Belief in life after death	1.01		(0.99,1.02)	0.01	0.313	0.99		(0.98,1.00)	0.01	0.139
Transformative religious experience	1.04		(1.03,1.06)	0.01	2.59e-07***	1.02		(1.00,1.03)	0.01	0.015*
Religious reading or listening	1.05		(1.03,1.06)	0.01	6.85e-08***	1.02		(1.01,1.04)	0.01	3.17e-03**
Prayer or meditation	1.04		(1.02,1.05)	0.01	7.29e-06***	1.01		(0.99,1.02)	0.01	0.221
Belief in God/gods/spiritual forces	1.01		(1.00,1.01)	0.00	0.034*	1.00		(0.99,1.01)	0.00	0.746
Religious centrality	1.03		(1.01,1.05)	0.01	9.03e-04**	1.00		(0.99,1.02)	0.01	0.600
Religious/spiritual comfort	1.03		(1.01,1.04)	0.01	1.88e-05***	1.00		(0.99,1.01)	0.01	0.702
Feel loved by God	1.01		(1.00,1.02)	0.00	0.082	0.99		(0.98,1.00)	0.00	0.038*
Feel punished by God	0.98		(0.97,1.00)	0.01	0.014*	0.99		(0.98,1.00)	0.01	0.040*
Experienced religious criticism	0.99		(0.98,1.00)	0.01	0.150	0.99		(0.98,1.00)	0.01	0.238
Faith-sharing	1.02		(1.00,1.04)	0.01	0.021*	1.00		(0.98,1.02)	0.01	0.793

Notes. N=13203; Reference for focal predictor: never/rarely. RR, risk-ratio, null effect is 1.00; ES, effect size measure for standardized regression coefficient, null effect is 0.00; SE, standard error, the SE reported for binary/Likert-type outcomes where risk-ratios are on the log(RR) scale; CI, confidence interval; p-value, a Wald-type test of the null hypothesis that the effect of the focal predictor is zero; (a) item part of the Happiness & Life Satisfaction domain of the Secure Flourishing Index; (b) item part of the Physical & Mental Health domain of the Secure Flourishing Index; (c) item part of the Meaning & Purpose domain of the Secure Flourishing Index; (d) item part of the Character & Virtue domain of the Secure Flourishing Index; (e) item part of the Subjective Social Connectedness domain of the Secure Flourishing Index; (f) item part of the Financial & Material Security domain of the Secure Flourishing Index.

Multiple imputation was performed to impute missing data on the covariates, exposure, and outcomes. All models controlled for sociodemographic and childhood factors assessed at Wave 1. For Model 2 with PC (principal components), the first seven principal components of the entire set of contemporaneous confounders assessed at Wave 1 were included as additional covariates of the outcomes at Wave 2.

An outcome-wide analytic approach was used, and a separate model was run for each outcome. A different type of model was run depending on the nature of the outcome: (1) for each binary outcome, a weighted generalized linear model (with a log link and Poisson distribution) was used to estimate an RR; and (2) for each continuous outcome, a weighted linear regression model was used to estimate a ES. All effect sizes were standardized. For continuous outcomes, the ES represents the change in SD on the outcome between the lower and upper categories of the binary focal predictor. For binary outcomes, the RR represents the change in risk of being in the upper category compared to the lower category between the lower and upper categories of the binary focal predictor.

P-value significance thresholds: $p < 0.05$*, $p < 0.005$**, (Bonferroni) $p < 6.41\text{e-}04$***, correction for multiple testing using Bonferroni adjusted significant threshold.

Table S11h. Associations of forgivingness with adult well-being and other outcomes at Wave 2 in Brazil using complete-case analyses with attrition weights.

	Model 1: Demographic and Childhood Variables as Covariates					Model 2: Demographic, Childhood, and Other Wave 1 Confounding Variables (Via Principal Components) as Covariates				
Outcome	RR	ES	95% CI	SE	p-value	RR	ES	95% CI	SE	p-value
Human Flourishing										
Secure flourishing index		0.10	(0.06,0.14)	0.02	3.74e-06***		0.03	(-0.01,0.07)	0.02	0.122
Flourishing index		0.10	(0.05,0.14)	0.02	9.71e-06***		0.03	(-0.01,0.06)	0.02	0.198
Happiness & life satisfaction		0.06	(0.02,0.11)	0.02	3.91e-03**		0.00	(-0.04,0.05)	0.02	0.816
Physical & mental health		0.07	(0.03,0.11)	0.02	2.03e-03**		0.01	(-0.03,0.04)	0.02	0.757
Meaning & purpose		0.12	(0.07,0.16)	0.02	7.98e-08***		0.05	(0.01,0.09)	0.02	0.010*
Character & virtue		0.08	(0.04,0.13)	0.02	9.97e-05***		0.04	(-0.00,0.08)	0.02	0.071
Close social relationships		0.06	(0.02,0.10)	0.02	0.007*		0.01	(-0.03,0.05)	0.02	0.702
Financial & material security		0.04	(0.00,0.08)	0.02	0.039*		0.02	(-0.02,0.06)	0.02	0.278
Psychological Well-Being										
Happiness (a)		0.05	(0.01,0.10)	0.02	0.014*		-0.00	(-0.04,0.04)	0.02	0.985
Life satisfaction (a)		0.06	(0.02,0.11)	0.02	3.23e-03**		0.01	(-0.03,0.05)	0.02	0.654
Current life evaluation		0.01	(-0.03,0.05)	0.02	0.593		-0.02	(-0.06,0.02)	0.02	0.259
Future life evaluation		0.03	(-0.01,0.07)	0.02	0.185		-0.00	(-0.04,0.04)	0.02	0.878
Optimism		0.03	(-0.01,0.07)	0.02	0.098		-0.01	(-0.05,0.03)	0.02	0.675
Freedom to pursue what's important		0.04	(-0.00,0.09)	0.02	0.058		-0.01	(-0.05,0.03)	0.02	0.739
Inner peace	1.05		(1.03,1.07)	0.01	4.06e-08***	1.03		(1.01,1.05)	0.01	8.61e-04**
Life balance	1.05		(1.03,1.07)	0.01	5.42e-06***	1.02		(1.00,1.03)	0.01	0.082
Sense of mastery	1.03		(1.02,1.05)	0.01	7.72e-05***	1.02		(1.00,1.03)	0.01	0.068
Meaningful activities (c)		0.10	(0.06,0.15)	0.02	3.72e-06***		0.05	(0.01,0.09)	0.02	0.020*
Understanding purpose (c)		0.10	(0.06,0.14)	0.02	1.21e-06***		0.04	(0.00,0.08)	0.02	0.034*
Self-rated mental health (b)		0.08	(0.04,0.12)	0.02	2.56e-04***		0.02	(-0.02,0.06)	0.02	0.276
Psychological Distress										
Traumatic distress	0.96		(0.94,0.98)	0.01	1.3e-04***	0.96		(0.94,0.98)	0.01	1.61e-04***
Depression symptoms composite	0.97		(0.95,0.99)	0.01	4.79e-04***	0.98		(0.96,1.00)	0.01	0.052
Depression – feel hopeless	0.96		(0.95,0.98)	0.01	1.94e-04***	0.98		(0.96,0.99)	0.01	0.008*
Depression – loss of interest	0.98		(0.96,1.00)	0.01	0.015*	0.98		(0.97,1.00)	0.01	0.124
Anxiety symptoms composite	0.97		(0.95,0.99)	0.01	2.04e-03**	0.99		(0.97,1.00)	0.01	0.147
Anxiety – feel on edge	0.97		(0.95,0.99)	0.01	0.009*	0.99		(0.97,1.01)	0.01	0.217
Anxiety – cannot stop worrying	0.97		(0.96,0.99)	0.01	0.012*	0.99		(0.97,1.01)	0.01	0.282
Suffering	0.95		(0.94,0.97)	0.01	1.44e-06***	0.97		(0.95,0.99)	0.01	1.14e-03**
Social Well-Being										
Relationship contentment (e)		0.06	(0.01,0.10)	0.02	0.014*		0.01	(-0.04,0.05)	0.02	0.729
Relationship satisfaction (e)		0.05	(0.01,0.10)	0.02	0.011*		0.01	(-0.03,0.05)	0.02	0.719
Social support		0.08	(0.04,0.13)	0.02	9.68e-05***		0.06	(0.01,0.10)	0.02	0.009*
Intimate/close friend	1.03		(1.01,1.04)	0.01	3.19e-03**	1.02		(1.00,1.03)	0.01	0.078
Government approval	1.00		(0.98,1.02)	0.01	0.928	0.99		(0.97,1.01)	0.01	0.196
Say in government	1.00		(0.98,1.02)	0.01	0.812	0.99		(0.97,1.01)	0.01	0.398
Belonging in country		0.08	(0.04,0.12)	0.02	1.79e-04***		0.03	(-0.01,0.07)	0.02	0.122
City/place satisfaction	1.02		(1.00,1.04)	0.01	0.096	1.00		(0.98,1.02)	0.01	0.735
Trust within country	1.02		(1.00,1.04)	0.01	0.086	1.00		(0.98,1.02)	0.01	0.700
Social Participation										
Ever been married	1.00		(0.99,1.01)	0.01	0.946	1.00		(0.99,1.01)	0.01	0.970
Currently divorced	1.00		(1.00,1.01)	0.00	0.155	1.00		(1.00,1.01)	0.00	0.124
Number of children		0.02	(-0.02,0.07)	0.02	0.324		0.00	(-0.04,0.05)	0.02	0.824
Weekly+ community participation	1.00		(0.99,1.02)	0.01	0.675	0.99		(0.98,1.01)	0.01	0.326
Weekly+ religious attendance	1.03		(1.01,1.04)	0.01	3.33e-03**	1.01		(1.00,1.03)	0.01	0.135
Social Distress										
Loneliness		-0.06	(-0.10,-0.02)	0.02	0.006*		-0.02	(-0.06,0.02)	0.02	0.381
Perceived discrimination	0.98		(0.96,1.00)	0.01	0.064	0.98		(0.96,1.00)	0.01	0.078
Character & Prosocial Behavior										
Orientation to promote good (d)		0.08	(0.04,0.12)	0.02	7.97e-05***		0.04	(-0.00,0.08)	0.02	0.059
Delayed gratification (d)		0.06	(0.02,0.11)	0.02	3.91e-03**		0.03	(-0.02,0.07)	0.02	0.218
Hope		0.09	(0.04,0.14)	0.02	1.7e-04***		0.04	(-0.00,0.08)	0.02	0.078
Gratitude		0.09	(0.05,0.14)	0.02	8.65e-05***		0.04	(-0.01,0.08)	0.02	0.092
Showing love/care		0.08	(0.03,0.12)	0.02	8.18e-04**		0.03	(-0.01,0.08)	0.02	0.108
Forgivingness	1.22		(1.20,1.24)	0.01	2.22e-16***	1.21		(1.19,1.24)	0.01	2.22e-16***
Charitable giving	1.01		(0.99,1.03)	0.01	0.195	1.00		(0.98,1.01)	0.01	0.704
Helping strangers	1.03		(1.01,1.05)	0.01	3.94e-03**	1.01		(0.99,1.03)	0.01	0.148
Volunteering	1.01		(0.99,1.02)	0.01	0.238	1.00		(0.98,1.01)	0.01	0.495
Physical Health & Health Behavior										
Self-rated physical health (b)		0.04	(-0.00,0.08)	0.02	0.080		-0.01	(-0.05,0.03)	0.02	0.541
Health problems	0.99		(0.98,1.01)	0.01	0.524	1.00		(0.98,1.01)	0.01	0.669
Pain in past 4 weeks	1.00		(0.98,1.02)	0.01	0.961	1.01		(0.99,1.03)	0.01	0.602
Daily smoker	1.01		(0.99,1.02)	0.01	0.527	1.01		(1.00,1.03)	0.01	0.078
Number of drinks per week		-0.03	(-0.08,0.02)	0.03	0.199		-0.01	(-0.05,0.03)	0.02	0.678
Days exercise per week		0.02	(-0.03,0.06)	0.02	0.425		-0.01	(-0.06,0.03)	0.02	0.572
Socioeconomic Outcomes										
Financial security (f)		0.04	(-0.00,0.08)	0.02	0.050		0.02	(-0.02,0.05)	0.02	0.441
Material security (f)		0.04	(-0.00,0.08)	0.02	0.065		0.02	(-0.01,0.06)	0.02	0.230
Educational attainment (16+ years)	1.01		(1.00,1.01)	0.00	0.113	1.00		(1.00,1.01)	0.00	0.163
Currently employed	1.00		(0.98,1.02)	0.01	0.806	0.99		(0.98,1.01)	0.01	0.499
Financially comfortable/getting by	0.99		(0.97,1.01)	0.01	0.351	0.99		(0.97,1.00)	0.01	0.139
Own home	1.00		(0.98,1.02)	0.01	0.929	1.00		(0.98,1.02)	0.01	0.758
Income – top quintile	1.00		(0.98,1.02)	0.01	0.949	1.00		(0.99,1.02)	0.01	0.775
Religion & Spirituality										
Religious/spiritual connection	1.02		(1.01,1.04)	0.01	0.009*	0.99		(0.98,1.01)	0.01	0.454
Belief in life after death	1.01		(0.99,1.03)	0.01	0.196	0.99		(0.97,1.01)	0.01	0.558
Transformative religious experience	1.05		(1.03,1.07)	0.01	2.03e-06***	1.02		(1.00,1.04)	0.01	0.026*
Religious reading or listening	1.05		(1.04,1.07)	0.01	2.44e-09***	1.03		(1.01,1.05)	0.01	1.31e-03**
Prayer or meditation	1.05		(1.03,1.07)	0.01	1.22e-07***	1.02		(1.01,1.04)	0.01	0.009*
Belief in God/gods/spiritual forces	1.01		(1.00,1.02)	0.01	0.011*	1.01		(1.00,1.02)	0.01	0.199
Religious centrality	1.04		(1.02,1.06)	0.01	2.46e-05***	1.01		(0.99,1.03)	0.01	0.180
Religious/spiritual comfort	1.03		(1.02,1.05)	0.01	2.83e-04***	1.00		(0.99,1.02)	0.01	0.567
Feel loved by God	1.01		(1.00,1.03)	0.01	0.074	0.99		(0.98,1.01)	0.01	0.348
Feel punished by God	0.99		(0.97,1.00)	0.01	0.088	0.98		(0.97,1.00)	0.01	0.028*
Experienced religious criticism	0.98		(0.96,1.00)	0.01	0.013*	0.98		(0.96,0.99)	0.01	4.4e-03**
Faith-sharing	1.02		(1.00,1.04)	0.01	0.022*	1.00		(0.98,1.02)	0.01	0.825

Table S11h. Associations of forgivingness with adult well-being and other outcomes at Wave 2 in Brazil using complete-case analyses with attrition weights.

Outcome	Model 1: Demographic and Childhood Variables as Covariates					Model 2: Demographic, Childhood, and Other Wave 1 Confounding Variables (Via Principal Components) as Covariates				
	RR	ES	95% CI	SE	p-value	RR	ES	95% CI	SE	p-value

Notes. N=4274; Reference for focal predictor: never/rarely. RR, risk-ratio, null effect is 1.00; ES, effect size measure for standardized regression coefficient, null effect is 0.00; SE, standard error, the SE reported for binary/Likert-type outcomes where risk-ratios are on the log(RR) scale; CI, confidence interval; p-value, a Wald-type test of the null hypothesis that the effect of the focal predictor is zero; (a) item part of the Happiness & Life Satisfaction domain of the Secure Flourishing Index; (b) item part of the Physical & Mental Health domain of the Secure Flourishing Index; (c) item part of the Meaning & Purpose domain of the Secure Flourishing Index; (d) item part of the Character & Virtue domain of the Secure Flourishing Index; (e) item part of the Subjective Social Connectedness domain of the Secure Flourishing Index; (f) item part of the Financial & Material Security domain of the Secure Flourishing Index.

Attrition weights were computed to adjust the complete case data (those who responded at Wave 2 to at least 50% of the questions) and multiple imputation was used to impute missing data on all remaining within wave on the covariates, exposure, and outcomes. All models controlled for sociodemographic and childhood factors assessed at Wave 1. For Model 2 with PC (principal components), the first seven principal components of the entire set of contemporaneous confounders assessed at Wave 1 were included as additional covariates of the outcomes at Wave 2.

An outcome-wide analytic approach was used, and a separate model was run for each outcome. A different type of model was run depending on the nature of the outcome: (1) for each binary outcome, a weighted generalized linear model (with a log link and Poisson distribution) was used to estimate an RR; and (2) for each continuous outcome, a weighted linear regression model was used to estimate a ES. All effect sizes were standardized. For continuous outcomes, the ES represents the change in SD on the outcome between the lower and upper categories of the binary focal predictor. For binary outcomes, the RR represents the change in risk of being in the upper category compared to the lower category between the lower and upper categories of the binary focal predictor.

P-value significance thresholds: p < 0.05*, p < 0.005**, (Bonferroni) p < 6.41e-04***, correction for multiple testing using Bonferroni adjusted significant threshold.

Table S11i. Sensitivity analysis of forgivingness outcome-wide results to unmeasured confounding using E-values in Brazil

	Multiple Imputation				Complete Case w/ Attrition Weights			
	Model 1: Demographics and Childhood Variables as Covariates		Model 2: Demographics, Childhood, and Other Wave 1 Confounders (Via Principal Components) as Covariates		Model 1: Demographics and Childhood Variables as Covariates		Model 2: Demographics, Childhood, and Other Wave 1 Confounders (Via Principal Components) as Covariates	
Outcome	EE	ECI	EE	ECI	EE	ECI	EE	ECI
Human Flourishing								
Secure flourishing index	1.68	1.52	1.20	1.00	1.76	1.50	1.32	1.00
Flourishing index	1.67	1.52	1.16	1.00	1.75	1.48	1.29	1.00
Happiness & life satisfaction	1.40	1.18	1.22	1.00	1.54	1.26	1.11	1.00
Physical & mental health	1.53	1.31	1.11	1.00	1.58	1.29	1.13	1.00
Meaning & purpose	1.68	1.50	1.28	1.00	1.86	1.60	1.47	1.19
Character & virtue	1.65	1.47	1.37	1.14	1.66	1.40	1.37	1.00
Close social relationships	1.43	1.25	1.13	1.00	1.52	1.22	1.15	1.00
Financial & material security	1.38	1.12	1.23	1.00	1.40	1.07	1.26	1.00
Psychological Well-Being								
Happiness	1.37	1.05	1.19	1.00	1.49	1.18	1.03	1.00
Life satisfaction	1.36	1.10	1.21	1.00	1.54	1.26	1.16	1.00
Current life evaluation	1.11	1.00	1.40	1.18	1.18	1.00	1.28	1.00
Future life evaluation	1.09	1.00	1.30	1.00	1.30	1.00	1.09	1.00
Optimism	1.39	1.16	1.03	1.00	1.35	1.00	1.15	1.00
Freedom to pursue what's important	1.34	1.00	1.21	1.00	1.41	1.00	1.14	1.00
Inner peace	1.38	1.28	1.23	1.07	1.49	1.37	1.33	1.19
Life balance	1.39	1.27	1.20	1.00	1.45	1.31	1.23	1.00
Sense of mastery	1.26	1.15	1.10	1.00	1.36	1.24	1.22	1.00
Meaningful activities	1.58	1.39	1.24	1.00	1.79	1.52	1.46	1.15
Understanding purpose	1.61	1.40	1.26	1.00	1.77	1.52	1.40	1.09
Self-rated mental health	1.59	1.41	1.24	1.00	1.65	1.38	1.26	1.00
Psychological Distress								
Traumatic distress	1.28	1.09	1.25	1.00	1.41	1.26	1.41	1.25
Depression symptoms composite	1.29	1.13	1.14	1.00	1.38	1.23	1.26	1.00
Depression – feel hopeless	1.27	1.14	1.15	1.00	1.40	1.25	1.31	1.14
Depression – loss of interest	1.24	1.04	1.14	1.00	1.30	1.12	1.23	1.00
Anxiety symptoms composite	1.27	1.14	1.11	1.00	1.35	1.19	1.22	1.00
Anxiety – feel on edge	1.24	1.08	1.10	1.00	1.32	1.14	1.20	1.00
Anxiety – cannot stop worrying	1.24	1.06	1.10	1.00	1.31	1.13	1.18	1.00
Suffering	1.35	1.23	1.24	1.06	1.47	1.33	1.35	1.20
Social Well-Being								
Relationship contentment	1.38	1.12	1.16	1.00	1.50	1.18	1.15	1.00
Relationship satisfaction	1.40	1.17	1.06	1.00	1.49	1.19	1.14	1.00
Social support	1.55	1.35	1.37	1.08	1.67	1.41	1.50	1.20
Intimate/close friend	1.24	1.09	1.13	1.00	1.31	1.16	1.23	1.00
Government approval	1.07	1.00	1.13	1.00	1.05	1.00	1.20	1.00
Say in government	1.11	1.00	1.20	1.00	1.08	1.00	1.15	1.00
Belonging in country	1.62	1.43	1.37	1.12	1.65	1.38	1.34	1.00
City/place satisfaction	1.18	1.00	1.03	1.00	1.23	1.00	1.09	1.00
Trust within country	1.29	1.13	1.20	1.00	1.25	1.00	1.10	1.00
Social Participation								
Ever been married	1.05	1.00	1.05	1.00	1.03	1.00	1.02	1.00
Currently divorced	1.03	1.00	1.03	1.00	1.10	1.00	1.11	1.00
Number of children	1.08	1.00	1.09	1.00	1.27	1.00	1.11	1.00
Weekly+ community participation	1.12	1.00	1.04	1.00	1.09	1.00	1.14	1.00
Weekly+ religious attendance	1.28	1.17	1.21	1.04	1.31	1.16	1.20	1.00
Social Distress								
Loneliness	1.45	1.18	1.15	1.00	1.51	1.23	1.24	1.00
Perceived discrimination	1.15	1.00	1.13	1.00	1.25	1.00	1.25	1.00
Character & Prosocial Behavior								
Orientation to promote good	1.70	1.51	1.46	1.24	1.66	1.41	1.39	1.00
Delayed gratification	1.42	1.21	1.18	1.00	1.54	1.25	1.30	1.00
Hope	1.60	1.37	1.28	1.00	1.72	1.42	1.39	1.00
Gratitude	1.68	1.48	1.37	1.11	1.71	1.43	1.37	1.00
Showing love/care	1.58	1.41	1.34	1.09	1.62	1.34	1.36	1.00
Forgivingness	2.50	2.36	2.46	2.32	2.52	2.38	2.48	2.34
Charitable giving	1.22	1.06	1.06	1.00	1.20	1.00	1.10	1.00
Helping strangers	1.30	1.18	1.19	1.00	1.34	1.17	1.22	1.00
Volunteering	1.13	1.00	1.11	1.00	1.16	1.00	1.12	1.00
Physical Health & Health Behavior								
Self-rated physical health	1.33	1.00	1.18	1.00	1.39	1.00	1.19	1.00
Health problems	1.10	1.00	1.13	1.00	1.13	1.00	1.10	1.00
Pain in past 4 weeks	1.05	1.00	1.15	1.00	1.04	1.00	1.12	1.00
Daily smoker	1.09	1.00	1.06	1.00	1.12	1.00	1.21	1.00
Number of drinks per week	1.26	1.00	1.11	1.00	1.34	1.00	1.16	1.00
Days exercise per week	1.16	1.00	1.22	1.00	1.23	1.00	1.19	1.00
Socioeconomic Outcomes								
Financial security	1.35	1.00	1.17	1.00	1.40	1.01	1.21	1.00
Material security	1.32	1.00	1.23	1.00	1.38	1.00	1.28	1.00
Educational attainment (16+ years)	1.10	1.00	1.10	1.00	1.12	1.00	1.11	1.00
Currently employed	1.06	1.00	1.01	1.00	1.08	1.00	1.13	1.00
Financially comfortable/getting by	1.11	1.00	1.20	1.00	1.17	1.00	1.22	1.00
Own home	1.03	1.00	1.07	1.00	1.05	1.00	1.09	1.00
Income – top quintile	1.12	1.00	1.13	1.00	1.04	1.00	1.08	1.00
Religion & Spirituality								
Religious/spiritual connection	1.30	1.19	1.07	1.00	1.30	1.13	1.13	1.00
Belief in life after death	1.14	1.00	1.18	1.00	1.21	1.00	1.13	1.00
Transformative religious experience	1.42	1.32	1.26	1.10	1.46	1.33	1.28	1.08
Religious reading or listening	1.46	1.35	1.29	1.15	1.51	1.39	1.33	1.19
Prayer or meditation	1.38	1.27	1.16	1.00	1.49	1.36	1.30	1.13
Belief in God/gods/spiritual forces	1.15	1.04	1.05	1.00	1.21	1.09	1.14	1.00
Religious centrality	1.33	1.20	1.11	1.00	1.43	1.28	1.20	1.00
Religious/spiritual comfort	1.31	1.22	1.08	1.00	1.37	1.22	1.11	1.00
Feel loved by God	1.16	1.00	1.17	1.04	1.21	1.00	1.14	1.00
Feel punished by God	1.23	1.09	1.20	1.04	1.21	1.00	1.24	1.07
Experienced religious criticism	1.16	1.00	1.14	1.00	1.27	1.11	1.29	1.14
Faith-sharing	1.29	1.10	1.08	1.00	1.29	1.10	1.08	1.00

Notes. EE, E-value for estimate; ECI, E-value for the limit of the confidence interval. The formula for calculating E-values can be found in VanderWeele and Ding (2017). E-values for estimate are the minimum strength of association on the risk ratio scale that an unmeasured confounder would need to have with both the exposure and the outcome to fully explain away the observed association between the exposure and outcome, conditional on the measured covariates. E-values for the 95% CI closest to the null denote the minimum strength of association on the risk ratio scale that an unmeasured confounder would need to have with both the exposure and the outcome to shift the CI to include the null value, conditional on the measured covariates.

Table S12a. Weighted summary statistics for demographic and childhood variables in China

**Characteristic**	**Wave 1** N = 5,022	**Wave 2** N = 4,595
Forgivingness, n (%)		
Always	668 (13.3%)	461 (10.0%)
Often	2,094 (41.7%)	1,844 (40.1%)
Rarely	1,927 (38.4%)	1,927 (41.9%)
Never	330 (6.6%)	363 (7.9%)
(Missing)	2 (0.0%)	0 (0%)
Year of birth, n (%)		
1943 or earlier (current age: 80+ years)	2 (0.0%)	1 (0.0%)
1943-1953 (current age: 70-79 years)	145 (2.9%)	184 (4.0%)
1953-1963 (current age: 60-69 years)	968 (19.3%)	902 (19.6%)
1963-1973 (current age: 50-59 years)	843 (16.8%)	773 (16.8%)
1973-1983 (current age: 40-49 years)	1,019 (20.3%)	918 (20.0%)
1983-1993 (current age: 30-39 years)	899 (17.9%)	813 (17.7%)
1993-1998 (current age: 25-29 years)	610 (12.1%)	580 (12.6%)
1998-2005 (current age: 18-24 years)	536 (10.7%)	425 (9.2%)
(Missing)	0 (0%)	0 (0%)
Age of participant		
Mean	44.8	45.5
Standard Deviation	15.4	15.4
Min, Max	18.0, 83.0	18.0, 84.0
Gender, n (%)		
Male	2,541 (50.6%)	2,326 (50.6%)
Female	2,481 (49.4%)	2,269 (49.4%)
Other	0 (0%)	0 (0%)
(Missing)	0 (0%)	0 (0%)
Respondent marital status, n (%)		
Single/Never been married	1,008 (20.1%)	913 (19.9%)
Married	3,551 (70.7%)	3,261 (71.0%)
Separated	44 (0.9%)	57 (1.2%)
Divorced	119 (2.4%)	108 (2.4%)
Widowed	159 (3.2%)	155 (3.4%)
Domestic partner	137 (2.7%)	91 (2.0%)
(Missing)	5 (0.1%)	10 (0.2%)
Education (years), n (%)		
Up to 8	3,231 (64.3%)	2,955 (64.3%)
9-15	1,317 (26.2%)	1,206 (26.2%)
16+	474 (9.4%)	434 (9.4%)
(Missing)	0 (0%)	0 (0%)
Employment status, n (%)		
Employed for an employer	1,906 (38.0%)	1,822 (39.7%)
Self-employed	1,295 (25.8%)	1,159 (25.2%)
Retired	924 (18.4%)	806 (17.5%)
Student	191 (3.8%)	161 (3.5%)
Homemaker	295 (5.9%)	278 (6.1%)
Unemployed and looking for a job	166 (3.3%)	135 (2.9%)
None of these/Other	244 (4.9%)	226 (4.9%)
(Missing)	2 (0.0%)	7 (0.1%)
Current religious service attendance, n (%)		
More than once a week	48 (1.0%)	55 (1.2%)
Once a week	187 (3.7%)	174 (3.8%)

Table S12a. Weighted summary statistics for demographic and childhood variables in China

Characteristic	Wave 1<br>N = 5,022	Wave 2<br>N = 4,595
One to three times a month	296 (5.9%)	268 (5.8%)
A few times a year	583 (11.6%)	534 (11.6%)
Never	3,902 (77.7%)	3,563 (77.5%)
(Missing)	6 (0.1%)	2 (0.0%)
Immigration status, n (%)		
Born in this country	5,006 (99.7%)	4,581 (99.7%)
Born in another country	11 (0.2%)	10 (0.2%)
(Missing)	5 (0.1%)	5 (0.1%)
Parental marital status around age 12, n (%)		
Parents were married	4,662 (92.8%)	4,276 (93.1%)
Parents were divorced	125 (2.5%)	113 (2.4%)
Parents were never married	33 (0.7%)	29 (0.6%)
One or both of them had died	137 (2.7%)	126 (2.8%)
Unsure	64 (1.3%)	50 (1.1%)
(Missing)	1 (0.0%)	1 (0.0%)
Religious service attendance around age 12, n (%)		
At least once a week	85 (1.7%)	79 (1.7%)
One to three times a month	310 (6.2%)	292 (6.4%)
Less than once a month	624 (12.4%)	567 (12.3%)
Never	3,999 (79.6%)	3,655 (79.5%)
(Missing)	4 (0.1%)	2 (0.1%)
Relationship with mother when growing up, n (%)		
Very good	2,059 (41.0%)	1,908 (41.5%)
Somewhat good	2,342 (46.6%)	2,128 (46.3%)
Somewhat bad	194 (3.9%)	177 (3.9%)
Very bad	25 (0.5%)	24 (0.5%)
(Does not apply)	403 (8.0%)	357 (7.8%)
(Missing)	0 (0%)	0 (0%)
Relationship with father when growing up, n (%)		
Very good	1,660 (33.0%)	1,527 (33.2%)
Somewhat good	2,335 (46.5%)	2,139 (46.6%)
Somewhat bad	435 (8.7%)	386 (8.4%)
Very bad	48 (1.0%)	45 (1.0%)
(Does not apply)	544 (10.8%)	497 (10.8%)
(Missing)	1 (0.0%)	1 (0.0%)
Felt like an outsider in family when growing up, n (%)		
Yes	622 (12.4%)	556 (12.1%)
No	4,315 (85.9%)	3,965 (86.3%)
(Missing)	85 (1.7%)	74 (1.6%)
Experienced abuse when growing up, n (%)		
Yes	279 (5.6%)	249 (5.4%)
No	4,741 (94.4%)	4,344 (94.5%)
(Missing)	2 (0.0%)	2 (0.0%)
Self-rated health when growing up, n (%)		
Excellent	731 (14.6%)	684 (14.9%)
Very good	1,939 (38.6%)	1,785 (38.8%)
Good	1,644 (32.7%)	1,497 (32.6%)
Fair	657 (13.1%)	590 (12.8%)
Poor	51 (1.0%)	40 (0.9%)
(Missing)	0 (0%)	0 (0%)

Table S12a. Weighted summary statistics for demographic and childhood variables in China

**Characteristic**	**Wave 1**<br>N = 5,022	**Wave 2**<br>N = 4,595
Subjective financial status of family growing up, n (%)		
Lived comfortably	1,376 (27.4%)	1,276 (27.8%)
Got by	2,696 (53.7%)	2,471 (53.8%)
Found it difficult	768 (15.3%)	682 (14.8%)
Found it very difficult	181 (3.6%)	165 (3.6%)
(Missing)	1 (0.0%)	1 (0.0%)
Religious affiliation growing up, n (%)		
Christianity	107 (2.1%)	102 (2.2%)
Taoism	103 (2.1%)	93 (2.0%)
Confucianism	11 (0.2%)	8 (0.2%)
Primal, Animist, or Folk religion	14 (0.3%)	13 (0.3%)
Spiritism	0 (0%)	0 (0%)
Umbanda, Candomblé, and other African-derived religions	0 (0%)	0 (0%)
Chinese folk/traditional religion	133 (2.7%)	130 (2.8%)
Islam	44 (0.9%)	15 (0.3%)
Hinduism	0 (0%)	0 (0%)
Buddhism	646 (12.9%)	612 (13.3%)
Judaism	0 (0%)	0 (0%)
Sikhism	0 (0%)	0 (0%)
Baha'i	0 (0%)	0 (0%)
Jainism	0 (0%)	0 (0%)
Shinto	7 (0.1%)	6 (0.1%)
Some other religion	1 (0.0%)	1 (0.0%)
No religion/Atheist/Agnostic	3,934 (78.3%)	3,594 (78.2%)
(Missing)	22 (0.4%)	21 (0.5%)

Note. N (%); this table is based on non-imputed data. Cumulative percentages for variables may not add up to 100% due to rounding. Wave 1 characteristics weighted using the Gallup provided sampling weight, ANNUAL_WEIGHT_R2; Wave 2 characteristics weighted accounting for attrition by using the adjusted Wave 1 weight, ANNUAL_WEIGHT_R2, multiplied by the created attrition weight to account for dropout, to maintain nationally representative estimates for Wave 2 characteristics.

Table S12b. Weighted summary statistics for outcome variables in China

Outcome	Wave 1<br>N = 5,022	Wave 2<br>N = 4,595
Secure flourishing index		
Mean	7.1	7.2
Standard Deviation	1.5	1.4
Min, Max	0.3, 10.0	0.4, 10.0
(Missing)	35 (0.7%)	10 (0.2%)
Flourishing index		
Mean	7.2	7.2
Standard Deviation	1.5	1.4
Min, Max	0.0, 10.0	0.2, 10.0
(Missing)	31 (0.6%)	8 (0.2%)
Happiness & life satisfaction		
Mean	7.0	7.0
Standard Deviation	1.6	1.6
Min, Max	0.0, 10.0	0.0, 10.0
(Missing)	19 (0.4%)	5 (0.1%)
Physical & mental health		
Mean	7.4	7.5
Standard Deviation	1.7	1.5
Min, Max	0.0, 10.0	0.0, 10.0
(Missing)	3 (<0.1%)	1 (<0.1%)
Meaning & purpose		
Mean	7.2	7.3
Standard Deviation	1.7	1.6
Min, Max	0.0, 10.0	0.0, 10.0
(Missing)	3 (<0.1%)	0 (<0.1%)
Character & virtue		
Mean	7.0	7.1
Standard Deviation	1.7	1.6
Min, Max	0.0, 10.0	0.0, 10.0
(Missing)	2 (<0.1%)	1 (<0.1%)
Close social relationships		
Mean	7.2	7.3
Standard Deviation	1.9	1.8
Min, Max	0.0, 10.0	0.0, 10.0
(Missing)	12 (0.2%)	1 (<0.1%)
Financial & material security		
Mean	6.9	7.0
Standard Deviation	2.2	2.0
Min, Max	0.0, 10.0	0.0, 10.0
(Missing)	4 (<0.1%)	2 (<0.1%)
Happiness		
Mean	7.0	6.9
Standard Deviation	1.7	1.6
Min, Max	0.0, 10.0	0.0, 10.0
(Missing)	4 (<0.1%)	0 (0%)
Life satisfaction		
Mean	7.0	7.0
Standard Deviation	1.8	1.7
Min, Max	0.0, 10.0	0.0, 10.0
(Missing)	14 (0.3%)	5 (0.1%)
Current life evaluation		

Table S12b. Weighted summary statistics for outcome variables in China

Outcome	Wave 1<br>N = 5,022	Wave 2<br>N = 4,595
Mean	6.3	6.4
Standard Deviation	1.8	1.7
Min, Max	0.0, 10.0	0.0, 10.0
(Missing)	1 (<0.1%)	3 (<0.1%)
Future life evaluation		
Mean	7.4	7.4
Standard Deviation	1.7	1.6
Min, Max	0.0, 10.0	0.0, 10.0
(Missing)	14 (0.3%)	1 (<0.1%)
Optimism		
Mean	7.6	7.7
Standard Deviation	1.9	1.8
Min, Max	0.0, 10.0	0.0, 10.0
Freedom to pursue what's important		
Mean	6.9	7.0
Standard Deviation	2.2	2.0
Min, Max	0.0, 10.0	0.0, 10.0
(Missing)	2 (<0.1%)	0 (0%)
Inner peace, n (%)		
Always	1,030 (20.5%)	975 (21.2%)
Often	3,357 (66.8%)	3,009 (65.5%)
Rarely	604 (12.0%)	575 (12.5%)
Never	27 (0.5%)	36 (0.8%)
(Missing)	5 (0.1%)	1 (0.0%)
Life balance, n (%)		
Always	1,044 (20.8%)	940 (20.5%)
Often	3,229 (64.3%)	2,888 (62.8%)
Rarely	713 (14.2%)	721 (15.7%)
Never	34 (0.7%)	45 (1.0%)
(Missing)	2 (0.0%)	2 (0.0%)
Sense of mastery, n (%)		
Always	1,040 (20.7%)	974 (21.2%)
Often	3,349 (66.7%)	3,005 (65.4%)
Rarely	589 (11.7%)	546 (11.9%)
Never	42 (0.8%)	62 (1.3%)
(Missing)	3 (0.1%)	9 (0.2%)
Meaningful activities		
Mean	7.3	7.3
Standard Deviation	1.8	1.7
Min, Max	0.0, 10.0	0.0, 10.0
(Missing)	1 (<0.1%)	0 (<0.1%)
Understanding purpose		
Mean	7.2	7.3
Standard Deviation	2.1	1.9
Min, Max	0.0, 10.0	0.0, 10.0
(Missing)	2 (<0.1%)	0 (0%)
Self-rated mental health		
Mean	7.5	7.6
Standard Deviation	1.9	1.8
Min, Max	0.0, 10.0	0.0, 10.0
(Missing)	3 (<0.1%)	0 (<0.1%)

Table S12b. Weighted summary statistics for outcome variables in China

**Outcome**	**Wave 1**<br>N = 5,022	**Wave 2**<br>N = 4,595
Traumatic distress, n (%)		
A lot	203 (4.1%)	112 (2.4%)
Some	984 (19.6%)	864 (18.8%)
Not very much	1,993 (39.7%)	1,963 (42.7%)
Not at all	1,841 (36.7%)	1,658 (36.1%)
(Missing)	1 (0.0%)	0 (0%)
Depression symptoms composite, n (%)	1,520 (30.3%)	1,151 (25.1%)
(Missing)	9 (0.2%)	4 (<0.1%)
Depression – feel hopeless, n (%)		
Nearly every day	121 (2.4%)	81 (1.8%)
More than half the days	579 (11.5%)	423 (9.2%)
Several days	2,324 (46.3%)	2,245 (48.8%)
Not at all	1,998 (39.8%)	1,842 (40.1%)
(Missing)	0 (<0.0%)	4 (0.1%)
Depression – loss of interest, n (%)		
Nearly every day	288 (5.7%)	173 (3.8%)
More than half the days	1,267 (25.2%)	995 (21.7%)
Several days	2,031 (40.4%)	2,054 (44.7%)
Not at all	1,428 (28.4%)	1,373 (29.9%)
(Missing)	9 (0.2%)	0 (0%)
Anxiety symptoms composite, n (%)	911 (18.2%)	707 (15.4%)
(Missing)	2 (<0.1%)	5 (0.1%)
Anxiety – feel on edge, n (%)		
Nearly every day	177 (3.5%)	101 (2.2%)
More than half the days	570 (11.3%)	456 (9.9%)
Several days	2,335 (46.5%)	2,329 (50.7%)
Not at all	1,940 (38.6%)	1,706 (37.1%)
(Missing)	0 (<0.0%)	4 (0.1%)
Anxiety – cannot stop worrying, n (%)		
Nearly every day	211 (4.2%)	108 (2.3%)
More than half the days	526 (10.5%)	455 (9.9%)
Several days	1,595 (31.8%)	1,580 (34.4%)
Not at all	2,688 (53.5%)	2,452 (53.4%)
(Missing)	2 (0.0%)	1 (0.0%)
Suffering, n (%)		
A lot	111 (2.2%)	76 (1.6%)
Some	1,576 (31.4%)	1,396 (30.4%)
Not very much	2,394 (47.7%)	2,465 (53.6%)
Not at all	941 (18.7%)	659 (14.3%)
(Missing)	0 (0%)	0 (0%)
Relationship contentment		
Mean	7.2	7.3
Standard Deviation	1.9	1.9
Min, Max	0.0, 10.0	0.0, 10.0
(Missing)	10 (0.2%)	1 (<0.1%)
Relationship satisfaction		
Mean	7.1	7.2
Standard Deviation	2.0	1.9
Min, Max	0.0, 10.0	0.0, 10.0
(Missing)	3 (<0.1%)	1 (<0.1%)
Social support		

Table S12b. Weighted summary statistics for outcome variables in China

Outcome	Wave 1<br>N = 5,022	Wave 2<br>N = 4,595
Mean	6.6	6.6
Standard Deviation	2.2	2.1
Min, Max	0.0, 10.0	0.0, 10.0
(Missing)	4 (<0.1%)	0 (0%)
Intimate/close friend, n (%)		
Yes	4,523 (90.1%)	4,123 (89.7%)
No	498 (9.9%)	472 (10.3%)
(Missing)	1 (0.0%)	0 (0%)
Government approval, n (%)		
Strongly approve	0 (0%)	0 (0%)
Somewhat approve	0 (0%)	0 (0%)
Neither approve nor disapprove	0 (0%)	0 (0%)
Somewhat disapprove	0 (0%)	0 (0%)
Strongly disapprove	0 (0%)	0 (0%)
(Missing)	5,022 (100.0%)	4,595 (100.0%)
Say in government, n (%)		
Agree	0 (0%)	0 (0%)
Disagree	0 (0%)	0 (0%)
Unsure	0 (0%)	0 (0%)
(Missing)	5,022 (100.0%)	4,595 (100.0%)
Belonging in country		
Mean	NA	NA
Standard Deviation	NA	NA
Min, Max	NA, NA	NA, NA
(Missing)	5,022 (100%)	4,595 (100%)
City/place satisfaction, n (%)		
Satisfied	4,320 (86.0%)	3,928 (85.5%)
Dissatisfied	445 (8.9%)	361 (7.9%)
Unsure	250 (5.0%)	306 (6.7%)
(Missing)	7 (0.1%)	1 (0.0%)
Trust within country, n (%)		
All people	190 (3.8%)	126 (2.7%)
Most people	2,979 (59.3%)	2,661 (57.9%)
Some people	1,252 (24.9%)	1,253 (27.3%)
Not very many people	554 (11.0%)	522 (11.4%)
None	46 (0.9%)	30 (0.6%)
(Missing)	1 (0.0%)	3 (0.1%)
Number of children		
Mean	0.7	0.7
Standard Deviation	0.8	0.8
Min, Max	0.0, 5.0	0.0, 5.0
(Missing)	19 (0.4%)	3 (<0.1%)
Community participation, n (%)		
More than once a week	245 (4.9%)	147 (3.2%)
Once a week	503 (10.0%)	460 (10.0%)
One to three times a month	940 (18.7%)	889 (19.3%)
A few times a year	1,525 (30.4%)	1,435 (31.2%)
Never	1,808 (36.0%)	1,663 (36.2%)
(Missing)	1 (0.0%)	1 (0.0%)
Religious attendance, n (%)		
More than once a week	48 (1.0%)	55 (1.2%)

Table S12b. Weighted summary statistics for outcome variables in China

**Outcome**	**Wave 1**<br>N = 5,022	**Wave 2**<br>N = 4,595
Once a week	187 (3.7%)	174 (3.8%)
One to three times a month	296 (5.9%)	268 (5.8%)
A few times a year	583 (11.6%)	534 (11.6%)
Never	3,902 (77.7%)	3,563 (77.5%)
(Missing)	6 (0.1%)	2 (0.0%)
Loneliness		
Mean	3.3	3.3
Standard Deviation	2.4	2.3
Min, Max	0.0, 10.0	0.0, 10.0
Perceived discrimination, n (%)		
Always	230 (4.6%)	146 (3.2%)
Often	610 (12.2%)	498 (10.8%)
Rarely	1,930 (38.4%)	2,037 (44.3%)
Never	2,246 (44.7%)	1,915 (41.7%)
(Missing)	6 (0.1%)	0 (0%)
Orientation to promote good		
Mean	7.2	7.3
Standard Deviation	1.8	1.7
Min, Max	0.0, 10.0	0.0, 10.0
Delayed gratification		
Mean	6.7	6.9
Standard Deviation	2.1	1.9
Min, Max	0.0, 10.0	0.0, 10.0
(Missing)	2 (<0.1%)	1 (<0.1%)
Hope		
Mean	7.5	7.5
Standard Deviation	1.9	1.7
Min, Max	0.0, 10.0	0.0, 10.0
Gratitude		
Mean	7.3	7.3
Standard Deviation	2.0	1.8
Min, Max	0.0, 10.0	0.0, 10.0
(Missing)	3 (<0.1%)	0 (0%)
Showing love/care		
Mean	7.4	7.4
Standard Deviation	1.8	1.8
Min, Max	0.0, 10.0	0.0, 10.0
Forgivingness, n (%)		
Always	668 (13.3%)	461 (10.0%)
Often	2,094 (41.7%)	1,844 (40.1%)
Rarely	1,927 (38.4%)	1,927 (41.9%)
Never	330 (6.6%)	363 (7.9%)
(Missing)	2 (0.0%)	0 (0%)
Charitable giving, n (%)		
Yes	1,779 (35.4%)	1,572 (34.2%)
No	3,235 (64.4%)	3,023 (65.8%)
(Missing)	9 (0.2%)	0 (0%)
Helping strangers, n (%)		
Yes	3,498 (69.7%)	2,958 (64.4%)
No	1,519 (30.2%)	1,638 (35.6%)
(Missing)	5 (0.1%)	0 (0%)

Table S12b. Weighted summary statistics for outcome variables in China

**Outcome**	**Wave 1** N = 5,022	**Wave 2** N = 4,595
Volunteering, n (%)		
Yes	1,702 (33.9%)	1,387 (30.2%)
No	3,313 (66.0%)	3,209 (69.8%)
(Missing)	7 (0.1%)	0 (0%)
Self-rated physical health		
Mean	7.2	7.3
Standard Deviation	1.8	1.6
Min, Max	0.0, 10.0	0.0, 10.0
Health problems, n (%)		
Yes	750 (14.9%)	722 (15.7%)
No	4,269 (85.0%)	3,870 (84.2%)
(Missing)	3 (0.1%)	3 (0.1%)
Pain in past 4 weeks, n (%)		
A lot	197 (3.9%)	101 (2.2%)
Some	1,254 (25.0%)	1,111 (24.2%)
Not very much	2,294 (45.7%)	2,249 (48.9%)
None at all	1,277 (25.4%)	1,134 (24.7%)
(Missing)	0 (0%)	1 (0.0%)
Number of cigarettes per day		
Mean	2.1	2.0
Standard Deviation	5.0	4.6
Min, Max	0.0, 97.0	0.0, 49.0
(Missing)	63 (1.2%)	76 (1.7%)
Number of drinks per week		
Mean	1.5	1.3
Standard Deviation	3.7	2.3
Min, Max	0.0, 97.0	0.0, 31.0
(Missing)	50 (1.0%)	20 (0.4%)
Days exercise per week		
Mean	2.7	2.7
Standard Deviation	2.1	1.9
Min, Max	0.0, 7.0	0.0, 7.0
Financial security		
Mean	6.9	7.0
Standard Deviation	2.4	2.2
Min, Max	0.0, 10.0	0.0, 10.0
Material security		
Mean	7.0	7.1
Standard Deviation	2.3	2.2
Min, Max	0.0, 10.0	0.0, 10.0
(Missing)	4 (<0.1%)	2 (<0.1%)
Educational attainment (16+ years), n (%)		
Up to 8	3,231 (64.3%)	2,955 (64.3%)
9-15	1,317 (26.2%)	1,206 (26.2%)
16+	474 (9.4%)	434 (9.4%)
(Missing)	0 (0%)	0 (0%)
Currently employed, n (%)		
Employed for an employer	1,906 (38.0%)	1,822 (39.7%)
Self-employed	1,295 (25.8%)	1,159 (25.2%)
Retired	924 (18.4%)	806 (17.5%)
Student	191 (3.8%)	161 (3.5%)

Table S12b. Weighted summary statistics for outcome variables in China

**Outcome**	**Wave 1**<br>N = 5,022	**Wave 2**<br>N = 4,595
Homemaker	295 (5.9%)	278 (6.1%)
Unemployed and looking for a job	166 (3.3%)	135 (2.9%)
None of these/Other	244 (4.9%)	226 (4.9%)
(Missing)	2 (0.0%)	7 (0.1%)
Financially comfortable/getting by, n (%)		
Living comfortably on present income	851 (16.9%)	1,017 (22.1%)
Getting by on present income	3,146 (62.7%)	2,693 (58.6%)
Finding it difficult on present income	869 (17.3%)	770 (16.8%)
Finding it very difficult on present income	153 (3.1%)	110 (2.4%)
(Missing)	3 (0.1%)	6 (0.1%)
Own home, n (%)		
Someone in this household owns this home	4,107 (81.8%)	3,803 (82.8%)
Someone in this household rents this home	689 (13.7%)	613 (13.3%)
Both	107 (2.1%)	105 (2.3%)
Neither	114 (2.3%)	72 (1.6%)
Rent	0 (0%)	0 (0%)
Own	0 (0%)	0 (0%)
Something else	0 (0%)	0 (0%)
(Missing)	5 (0.1%)	3 (0.1%)
Religious/spiritual connection, n (%)		
Always	332 (6.6%)	264 (5.7%)
Often	822 (16.4%)	771 (16.8%)
Rarely	1,452 (28.9%)	1,521 (33.1%)
Never	2,411 (48.0%)	2,037 (44.3%)
(Missing)	5 (0.1%)	2 (0.0%)
Belief in life after death, n (%)		
Yes	1,187 (23.6%)	1,058 (23.0%)
No	2,106 (41.9%)	1,916 (41.7%)
Unsure	1,718 (34.2%)	1,621 (35.3%)
(Missing)	11 (0.2%)	1 (0.0%)
Transformative religious experience, n (%)		
Yes	1,127 (22.4%)	1,012 (22.0%)
No	3,885 (77.4%)	3,584 (78.0%)
(Missing)	10 (0.2%)	0 (0%)
Religious reading or listening, n (%)		
More than once a day	73 (1.5%)	58 (1.3%)
About once a day	228 (4.5%)	225 (4.9%)
Sometimes	1,012 (20.2%)	915 (19.9%)
Never	3,697 (73.6%)	3,397 (73.9%)
(Missing)	11 (0.2%)	1 (0.0%)
Prayer or meditation, n (%)		
More than once a day	119 (2.4%)	98 (2.1%)
About once a day	356 (7.1%)	299 (6.5%)
Sometimes	1,605 (32.0%)	1,557 (33.9%)
Never	2,938 (58.5%)	2,641 (57.5%)
(Missing)	4 (0.1%)	1 (0.0%)
Belief in God/gods/spiritual forces, n (%)		
One God	171 (3.4%)	177 (3.8%)
More than one god	468 (9.3%)	428 (9.3%)
An impersonal spiritual force	1,042 (20.7%)	923 (20.1%)
None of these	2,525 (50.3%)	2,319 (50.5%)

Table S12b. Weighted summary statistics for outcome variables in China

Outcome	Wave 1<br>N = 5,022	Wave 2<br>N = 4,595
Unsure	814 (16.2%)	745 (16.2%)
(Missing)	2 (0.0%)	2 (0.0%)
Religious centrality, n (%)		
Agree	658 (13.1%)	632 (13.8%)
Disagree	1,245 (24.8%)	937 (20.4%)
Not relevant	2,352 (46.8%)	2,298 (50.0%)
Unsure	759 (15.1%)	726 (15.8%)
(Missing)	8 (0.2%)	2 (0.0%)
Religious/spiritual comfort, n (%)		
Agree	778 (15.5%)	726 (15.8%)
Disagree	1,269 (25.3%)	1,038 (22.6%)
Not relevant	2,211 (44.0%)	2,187 (47.6%)
Unsure	753 (15.0%)	643 (14.0%)
(Missing)	10 (0.2%)	2 (0.0%)
Feel loved by God, n (%)		
Agree	673 (13.4%)	642 (14.0%)
Disagree	1,350 (26.9%)	1,097 (23.9%)
Not relevant	2,184 (43.5%)	2,171 (47.2%)
Unsure	804 (16.0%)	680 (14.8%)
(Missing)	11 (0.2%)	6 (0.1%)
Feel punished by God, n (%)		
Agree	390 (7.8%)	373 (8.1%)
Disagree	1,774 (35.3%)	1,448 (31.5%)
Not relevant	2,122 (42.3%)	2,130 (46.4%)
Unsure	727 (14.5%)	643 (14.0%)
(Missing)	9 (0.2%)	2 (0.0%)
Experienced religious criticism, n (%)		
Agree	227 (4.5%)	235 (5.1%)
Disagree	1,803 (35.9%)	1,525 (33.2%)
Not relevant	2,388 (47.5%)	2,266 (49.3%)
Unsure	597 (11.9%)	567 (12.3%)
(Missing)	8 (0.2%)	3 (0.1%)
Faith-sharing, n (%)		
Agree	590 (11.8%)	577 (12.5%)
Disagree	1,460 (29.1%)	1,202 (26.2%)
Not relevant	2,282 (45.4%)	2,215 (48.2%)
Unsure	680 (13.5%)	600 (13.1%)
(Missing)	10 (0.2%)	2 (0.0%)

Note. N (%); this table is based on non-imputed data. Cumulative percentages for variables may not add up to 100% due to rounding. Wave 1 characteristics weighted using the Gallup provided sampling weight, ANNUAL_WEIGHT_R2; Wave 2 characteristics weighted accounting for attrition by using the adjusted Wave 1 weight, ANNUAL_WEIGHT_R2, multiplied by the created attrition weight to account for dropout, to maintain nationally representative estimates for Wave 2 characteristics.

Table S12c. Unweighted summary statistics for demographic and childhood variables in China by retention status

Characteristic	Attritors–Not Observed in Wave 2 N = 468	Retained–Observed in Wave 2 N = 4,604
Forgivingness, n (%)		
Always	70 (15.0%)	605 (13.1%)
Often	198 (42.4%)	1,916 (41.6%)
Rarely	168 (36.0%)	1,779 (38.6%)
Never	31 (6.6%)	303 (6.6%)
(Missing)	0 (0%)	2 (0.0%)
Year of birth, n (%)		
1943 or earlier (current age: 80+ years)	0 (0%)	2 (0.0%)
1943-1953 (current age: 70-79 years)	12 (2.5%)	134 (2.9%)
1953-1963 (current age: 60-69 years)	111 (23.7%)	867 (18.8%)
1963-1973 (current age: 50-59 years)	75 (16.0%)	777 (16.9%)
1973-1983 (current age: 40-49 years)	93 (19.8%)	937 (20.3%)
1983-1993 (current age: 30-39 years)	72 (15.5%)	836 (18.2%)
1993-1998 (current age: 25-29 years)	53 (11.3%)	563 (12.2%)
1998-2005 (current age: 18-24 years)	53 (11.2%)	489 (10.6%)
(Missing)	0 (0%)	0 (0%)
Age of participant		
Mean	45.8	44.7
Standard Deviation	15.7	15.3
Min, Max	18.0, 74.0	18.0, 83.0
Gender, n (%)		
Male	262 (56.1%)	2,303 (50.0%)
Female	205 (43.9%)	2,301 (50.0%)
Other	0 (0%)	0 (0%)
(Missing)	0 (0%)	0 (0%)
Respondent marital status, n (%)		
Single/Never been married	91 (19.5%)	927 (20.1%)
Married	328 (70.1%)	3,258 (70.8%)
Separated	12 (2.6%)	32 (0.7%)
Divorced	15 (3.3%)	105 (2.3%)
Widowed	15 (3.3%)	146 (3.2%)
Domestic partner	4 (0.9%)	134 (2.9%)
(Missing)	2 (0.3%)	3 (0.1%)
Education (years), n (%)		
Up to 8	296 (63.3%)	2,968 (64.5%)
9-15	130 (27.7%)	1,200 (26.1%)
16+	42 (9.0%)	437 (9.5%)
(Missing)	0 (0%)	0 (0%)
Employment status, n (%)		
Employed for an employer	153 (32.8%)	1,772 (38.5%)
Self-employed	131 (28.0%)	1,177 (25.6%)
Retired	94 (20.1%)	839 (18.2%)
Student	15 (3.3%)	177 (3.9%)
Homemaker	31 (6.7%)	266 (5.8%)
Unemployed and looking for a job	19 (4.1%)	148 (3.2%)
None of these/Other	22 (4.7%)	224 (4.9%)
(Missing)	2 (0.3%)	0 (0%)
Current religious service attendance, n (%)		
More than once a week	1 (0.3%)	47 (1.0%)

Table S12c. Unweighted summary statistics for demographic and childhood variables in China by retention status

**Characteristic**	**Attritors–Not Observed in Wave 2** N = 468	**Retained–Observed in Wave 2** N = 4,604
Once a week	16 (3.4%)	173 (3.8%)
One to three times a month	30 (6.3%)	270 (5.9%)
A few times a year	48 (10.3%)	541 (11.7%)
Never	372 (79.4%)	3,569 (77.5%)
(Missing)	1 (0.3%)	4 (0.1%)
Immigration status, n (%)		
Born in this country	465 (99.5%)	4,591 (99.7%)
Born in another country	2 (0.5%)	9 (0.2%)
(Missing)	0 (0%)	5 (0.1%)
Parental marital status around age 12, n (%)		
Parents were married	421 (90.1%)	4,287 (93.1%)
Parents were divorced	13 (2.8%)	114 (2.5%)
Parents were never married	5 (1.1%)	28 (0.6%)
One or both of them had died	12 (2.7%)	126 (2.7%)
Unsure	16 (3.3%)	48 (1.1%)
(Missing)	0 (0%)	1 (0.0%)
Religious service attendance around age 12, n (%)		
At least once a week	5 (1.1%)	81 (1.8%)
One to three times a month	21 (4.4%)	293 (6.4%)
Less than once a month	59 (12.6%)	571 (12.4%)
Never	382 (81.6%)	3,657 (79.4%)
(Missing)	1 (0.3%)	2 (0.1%)
Relationship with mother when growing up, n (%)		
Very good	177 (37.9%)	1,902 (41.3%)
Somewhat good	222 (47.6%)	2,143 (46.5%)
Somewhat bad	16 (3.5%)	179 (3.9%)
Very bad	1 (0.1%)	25 (0.5%)
(Does not apply)	51 (10.9%)	355 (7.7%)
(Missing)	0 (0%)	0 (0%)
Relationship with father when growing up, n (%)		
Very good	156 (33.4%)	1,520 (33.0%)
Somewhat good	205 (43.9%)	2,153 (46.8%)
Somewhat bad	50 (10.7%)	389 (8.4%)
Very bad	2 (0.5%)	46 (1.0%)
(Does not apply)	53 (11.4%)	496 (10.8%)
(Missing)	0 (0%)	1 (0.0%)
Felt like an outsider in family when growing up, n (%)		
Yes	76 (16.3%)	551 (12.0%)
No	378 (80.9%)	3,980 (86.4%)
(Missing)	13 (2.8%)	73 (1.6%)
Experienced abuse when growing up, n (%)		
Yes	37 (7.9%)	245 (5.3%)
No	431 (92.1%)	4,357 (94.6%)
(Missing)	0 (0%)	2 (0.0%)
Self-rated health when growing up, n (%)		
Excellent	61 (13.0%)	678 (14.7%)
Very good	170 (36.4%)	1,788 (38.8%)

Table S12c. Unweighted summary statistics for demographic and childhood variables in China by retention status

**Characteristic**	**Attritors–Not Observed in Wave 2** N = 468	**Retained–Observed in Wave 2** N = 4,604
Good	156 (33.3%)	1,505 (32.7%)
Fair	70 (14.9%)	593 (12.9%)
Poor	11 (2.3%)	40 (0.9%)
(Missing)	0 (0%)	0 (0%)
Subjective financial status of family growing up, n (%)		
Lived comfortably	117 (25.0%)	1,273 (27.7%)
Got by	240 (51.4%)	2,484 (53.9%)
Found it difficult	91 (19.5%)	684 (14.8%)
Found it very difficult	20 (4.2%)	163 (3.5%)
(Missing)	0 (0%)	1 (0.0%)
Religious affiliation growing up, n (%)		
Christianity	4 (0.8%)	104 (2.3%)
Taoism	11 (2.3%)	94 (2.0%)
Confucianism	3 (0.6%)	8 (0.2%)
Primal, Animist, or Folk religion	2 (0.4%)	13 (0.3%)
Spiritism	0 (0%)	0 (0%)
Umbanda, Candomblé, and other African-derived religions	0 (0%)	0 (0%)
Chinese folk/traditional religion	6 (1.2%)	129 (2.8%)
Islam	29 (6.3%)	15 (0.3%)
Hinduism	0 (0%)	0 (0%)
Buddhism	34 (7.4%)	619 (13.4%)
Judaism	0 (0%)	0 (0%)
Sikhism	0 (0%)	0 (0%)
Baha'i	0 (0%)	0 (0%)
Jainism	0 (0%)	0 (0%)
Shinto	1 (0.2%)	6 (0.1%)
Some other religion	0 (0%)	1 (0.0%)
No religion/Atheist/Agnostic	377 (80.7%)	3,595 (78.1%)
(Missing)	1 (0.3%)	21 (0.4%)

Note. N (%); this table is based on non-imputed data. Cumulative percentages for variables may not add up to 100% due to rounding.

Table S12d. Unweighted summary statistics for Wave 1 outcome variables in China by retention status.

**Outcome**	**Attritors–Not Observed in Wave 2**<br>N = 468	**Retained–Observed in Wave 2**<br>N = 4,604
Secure flourishing index		
Mean	7.2	7.1
Standard Deviation	1.4	1.5
Min, Max	2.2, 10.0	0.3, 10.0
(Missing)	0 (0%)	36 (0.8%)
Flourishing index		
Mean	7.3	7.1
Standard Deviation	1.4	1.5
Min, Max	2.6, 10.0	0.0, 10.0
(Missing)	0 (0%)	32 (0.7%)
Happiness & life satisfaction		
Mean	7.2	7.0
Standard Deviation	1.6	1.7
Min, Max	2.0, 10.0	0.0, 10.0
(Missing)	0 (0%)	19 (0.4%)
Physical & mental health		
Mean	7.5	7.4
Standard Deviation	1.6	1.7
Min, Max	0.0, 10.0	0.0, 10.0
(Missing)	0 (0%)	3 (<0.1%)
Meaning & purpose		
Mean	7.4	7.2
Standard Deviation	1.6	1.7
Min, Max	1.5, 10.0	0.0, 10.0
(Missing)	0 (0%)	3 (<0.1%)
Character & virtue		
Mean	7.2	7.0
Standard Deviation	1.7	1.7
Min, Max	0.0, 10.0	0.0, 10.0
(Missing)	0 (0%)	2 (<0.1%)
Close social relationships		
Mean	7.4	7.1
Standard Deviation	1.8	1.9
Min, Max	0.0, 10.0	0.0, 10.0
(Missing)	0 (0%)	12 (0.3%)
Financial & material security		
Mean	6.9	6.9
Standard Deviation	2.3	2.2
Min, Max	0.0, 10.0	0.0, 10.0
(Missing)	0 (0%)	4 (<0.1%)
Happiness		
Mean	7.2	7.0
Standard Deviation	1.7	1.7
Min, Max	1.0, 10.0	0.0, 10.0
(Missing)	0 (0%)	4 (<0.1%)
Life satisfaction		
Mean	7.3	7.0
Standard Deviation	1.8	1.8
Min, Max	0.0, 10.0	0.0, 10.0

Table S12d. Unweighted summary statistics for Wave 1 outcome variables in China by retention status.

**Outcome**	**Attritors–Not Observed in Wave 2**<br>N = 468	**Retained–Observed in Wave 2**<br>N = 4,604
(Missing)	0 (0%)	14 (0.3%)
Current life evaluation		
Mean	6.6	6.3
Standard Deviation	1.9	1.7
Min, Max	0.0, 10.0	0.0, 10.0
Future life evaluation		
Mean	7.5	7.3
Standard Deviation	1.6	1.7
Min, Max	0.0, 10.0	0.0, 10.0
(Missing)	0 (0%)	15 (0.3%)
Optimism		
Mean	7.7	7.6
Standard Deviation	1.9	1.9
Min, Max	0.0, 10.0	0.0, 10.0
Freedom to pursue what's important		
Mean	7.0	6.9
Standard Deviation	2.2	2.2
Min, Max	0.0, 10.0	0.0, 10.0
(Missing)	0 (0%)	2 (<0.1%)
Inner peace, n (%)		
Always	93 (19.9%)	947 (20.6%)
Often	317 (67.8%)	3,073 (66.7%)
Rarely	56 (11.9%)	554 (12.0%)
Never	1 (0.3%)	26 (0.6%)
(Missing)	0 (0%)	5 (0.1%)
Life balance, n (%)		
Always	105 (22.4%)	950 (20.6%)
Often	277 (59.3%)	2,985 (64.8%)
Rarely	77 (16.4%)	643 (14.0%)
Never	9 (1.8%)	26 (0.6%)
(Missing)	1 (0.1%)	1 (0.0%)
Sense of mastery, n (%)		
Always	98 (20.9%)	952 (20.7%)
Often	316 (67.6%)	3,066 (66.6%)
Rarely	52 (11.0%)	543 (11.8%)
Never	2 (0.4%)	40 (0.9%)
(Missing)	0 (0%)	3 (0.1%)
Meaningful activities		
Mean	7.5	7.3
Standard Deviation	1.7	1.8
Min, Max	0.0, 10.0	0.0, 10.0
(Missing)	0 (0%)	1 (<0.1%)
Understanding purpose		
Mean	7.3	7.2
Standard Deviation	2.0	2.1
Min, Max	0.0, 10.0	0.0, 10.0
(Missing)	0 (0%)	2 (<0.1%)
Self-rated mental health		
Mean	7.7	7.5

Table S12d. Unweighted summary statistics for Wave 1 outcome variables in China by retention status.

**Outcome**	**Attritors–Not Observed in Wave 2** N = 468	**Retained–Observed in Wave 2** N = 4,604
Standard Deviation	1.7	1.9
Min, Max	0.0, 10.0	0.0, 10.0
(Missing)	0 (0%)	3 (<0.1%)
Traumatic distress, n (%)		
A lot	32 (6.9%)	173 (3.8%)
Some	109 (23.2%)	884 (19.2%)
Not very much	181 (38.7%)	1,832 (39.8%)
Not at all	146 (31.2%)	1,715 (37.2%)
(Missing)	0 (0%)	1 (0.0%)
Depression symptoms composite, n (%)	163 (35.1%)	1,371 (29.8%)
(Missing)	2 (0.5%)	7 (0.1%)
Depression – feel hopeless, n (%)		
Nearly every day	21 (4.6%)	100 (2.2%)
More than half the days	51 (10.8%)	534 (11.6%)
Several days	215 (45.9%)	2,133 (46.3%)
Not at all	181 (38.7%)	1,837 (39.9%)
(Missing)	0 (0%)	0 (0.0%)
Depression – loss of interest, n (%)		
Nearly every day	36 (7.8%)	254 (5.5%)
More than half the days	135 (28.9%)	1,144 (24.9%)
Several days	173 (37.0%)	1,878 (40.8%)
Not at all	121 (25.8%)	1,322 (28.7%)
(Missing)	2 (0.5%)	7 (0.1%)
Anxiety symptoms composite, n (%)	117 (25.0%)	803 (17.4%)
(Missing)	0 (0%)	2 (<0.1%)
Anxiety – feel on edge, n (%)		
Nearly every day	29 (6.3%)	149 (3.2%)
More than half the days	62 (13.2%)	513 (11.2%)
Several days	202 (43.3%)	2,156 (46.8%)
Not at all	174 (37.3%)	1,785 (38.8%)
(Missing)	0 (0%)	0 (0.0%)
Anxiety – cannot stop worrying, n (%)		
Nearly every day	23 (4.9%)	191 (4.1%)
More than half the days	71 (15.3%)	460 (10.0%)
Several days	139 (29.7%)	1,472 (32.0%)
Not at all	235 (50.2%)	2,480 (53.9%)
(Missing)	0 (0%)	2 (0.0%)
Suffering, n (%)		
A lot	13 (2.8%)	98 (2.1%)
Some	162 (34.7%)	1,429 (31.0%)
Not very much	226 (48.3%)	2,192 (47.6%)
Not at all	66 (14.2%)	885 (19.2%)
(Missing)	0 (0%)	0 (0%)
Relationship contentment		
Mean	7.5	7.2
Standard Deviation	1.8	2.0
Min, Max	0.0, 10.0	0.0, 10.0
(Missing)	0 (0%)	10 (0.2%)
Relationship satisfaction		

Table S12d. Unweighted summary statistics for Wave 1 outcome variables in China by retention status.

**Outcome**	**Attritors–Not Observed in Wave 2**<br>N = 468	**Retained–Observed in Wave 2**<br>N = 4,604
Mean	7.3	7.1
Standard Deviation	2.0	2.0
Min, Max	0.0, 10.0	0.0, 10.0
(Missing)	0 (0%)	3 (<0.1%)
Social support		
Mean	6.7	6.6
Standard Deviation	2.1	2.2
Min, Max	0.0, 10.0	0.0, 10.0
(Missing)	0 (0%)	4 (<0.1%)
Intimate/close friend, n (%)		
Yes	415 (88.7%)	4,153 (90.2%)
No	53 (11.3%)	450 (9.8%)
(Missing)	0 (0%)	1 (0.0%)
Government approval, n (%)		
Strongly approve	0 (0%)	0 (0%)
Somewhat approve	0 (0%)	0 (0%)
Neither approve nor disapprove	0 (0%)	0 (0%)
Somewhat disapprove	0 (0%)	0 (0%)
Strongly disapprove	0 (0%)	0 (0%)
(Missing)	468 (100.0%)	4,604 (100.0%)
Say in government, n (%)		
Agree	0 (0%)	0 (0%)
Disagree	0 (0%)	0 (0%)
Unsure	0 (0%)	0 (0%)
(Missing)	468 (100.0%)	4,604 (100.0%)
Belonging in country		
Mean	NA	NA
Standard Deviation	NA	NA
Min, Max	NA, NA	NA, NA
(Missing)	468 (100%)	4,604 (100%)
City/place satisfaction, n (%)		
Satisfied	379 (81.1%)	3,984 (86.5%)
Dissatisfied	70 (14.9%)	378 (8.2%)
Unsure	18 (3.9%)	235 (5.1%)
(Missing)	0 (0%)	7 (0.2%)
Trust within country, n (%)		
All people	19 (4.1%)	173 (3.8%)
Most people	279 (59.6%)	2,731 (59.3%)
Some people	113 (24.1%)	1,152 (25.0%)
Not very many people	53 (11.3%)	507 (11.0%)
None	5 (1.0%)	41 (0.9%)
(Missing)	0 (0%)	1 (0.0%)
Number of children		
Mean	0.8	0.7
Standard Deviation	0.9	0.8
Min, Max	0.0, 5.0	0.0, 5.0
(Missing)	2 (0.5%)	17 (0.4%)
Community participation, n (%)		
More than once a week	32 (6.9%)	215 (4.7%)

Table S12d. Unweighted summary statistics for Wave 1 outcome variables in China by retention status.

**Outcome**	**Attritors–Not Observed in Wave 2** N = 468	**Retained–Observed in Wave 2** N = 4,604
Once a week	45 (9.7%)	462 (10.0%)
One to three times a month	89 (19.0%)	860 (18.7%)
A few times a year	144 (30.9%)	1,396 (30.3%)
Never	157 (33.5%)	1,670 (36.3%)
(Missing)	0 (0%)	1 (0.0%)
Religious attendance, n (%)		
More than once a week	1 (0.3%)	47 (1.0%)
Once a week	16 (3.4%)	173 (3.8%)
One to three times a month	30 (6.3%)	270 (5.9%)
A few times a year	48 (10.3%)	541 (11.7%)
Never	372 (79.4%)	3,569 (77.5%)
(Missing)	1 (0.3%)	4 (0.1%)
Loneliness		
Mean	3.3	3.3
Standard Deviation	2.6	2.4
Min, Max	0.0, 10.0	0.0, 10.0
Perceived discrimination, n (%)		
Always	49 (10.5%)	182 (4.0%)
Often	63 (13.5%)	553 (12.0%)
Rarely	166 (35.4%)	1,784 (38.7%)
Never	189 (40.4%)	2,080 (45.2%)
(Missing)	1 (0.3%)	5 (0.1%)
Orientation to promote good		
Mean	7.4	7.2
Standard Deviation	1.7	1.8
Min, Max	0.0, 10.0	0.0, 10.0
Delayed gratification		
Mean	7.0	6.7
Standard Deviation	2.1	2.1
Min, Max	0.0, 10.0	0.0, 10.0
(Missing)	0 (0%)	2 (<0.1%)
Hope		
Mean	7.5	7.4
Standard Deviation	1.9	1.9
Min, Max	0.0, 10.0	0.0, 10.0
Gratitude		
Mean	7.4	7.2
Standard Deviation	2.0	2.0
Min, Max	0.0, 10.0	0.0, 10.0
(Missing)	0 (0%)	3 (<0.1%)
Showing love/care		
Mean	7.5	7.3
Standard Deviation	1.8	1.8
Min, Max	0.0, 10.0	0.0, 10.0
Forgivingness, n (%)		
Always	70 (15.0%)	605 (13.1%)
Often	198 (42.4%)	1,916 (41.6%)
Rarely	168 (36.0%)	1,779 (38.6%)
Never	31 (6.6%)	303 (6.6%)

Table S12d. Unweighted summary statistics for Wave 1 outcome variables in China by retention status.

**Outcome**	**Attritors–Not Observed in Wave 2** N = 468	**Retained–Observed in Wave 2** N = 4,604
(Missing)	0 (0%)	2 (0.0%)
Charitable giving, n (%)		
Yes	175 (37.5%)	1,621 (35.2%)
No	292 (62.3%)	2,976 (64.6%)
(Missing)	1 (0.2%)	8 (0.2%)
Helping strangers, n (%)		
Yes	312 (66.8%)	3,221 (70.0%)
No	154 (33.0%)	1,380 (30.0%)
(Missing)	1 (0.2%)	4 (0.1%)
Volunteering, n (%)		
Yes	174 (37.2%)	1,545 (33.5%)
No	293 (62.6%)	3,054 (66.3%)
(Missing)	1 (0.2%)	6 (0.1%)
Self-rated physical health		
Mean	7.3	7.2
Standard Deviation	1.8	1.8
Min, Max	0.0, 10.0	0.0, 10.0
Health problems, n (%)		
Yes	119 (25.5%)	636 (13.8%)
No	347 (74.2%)	3,966 (86.1%)
(Missing)	1 (0.2%)	2 (0.0%)
Pain in past 4 weeks, n (%)		
A lot	25 (5.3%)	174 (3.8%)
Some	151 (32.2%)	1,115 (24.2%)
Not very much	204 (43.6%)	2,114 (45.9%)
None at all	89 (19.0%)	1,202 (26.1%)
(Missing)	0 (0%)	0 (0%)
Number of cigarettes per day		
Mean	2.8	2.1
Standard Deviation	5.7	4.9
Min, Max	0.0, 40.0	0.0, 97.0
(Missing)	3 (0.7%)	60 (1.3%)
Number of drinks per week		
Mean	1.8	1.4
Standard Deviation	5.3	3.5
Min, Max	0.0, 97.0	0.0, 97.0
(Missing)	6 (1.2%)	45 (1.0%)
Days exercise per week		
Mean	2.8	2.7
Standard Deviation	2.0	2.1
Min, Max	0.0, 7.0	0.0, 7.0
Financial security		
Mean	6.8	6.9
Standard Deviation	2.4	2.4
Min, Max	0.0, 10.0	0.0, 10.0
Material security		
Mean	6.9	7.0
Standard Deviation	2.4	2.3
Min, Max	0.0, 10.0	0.0, 10.0

Table S12d. Unweighted summary statistics for Wave 1 outcome variables in China by retention status.

**Outcome**	**Attritors–Not Observed in Wave 2**<br>N = 468	**Retained–Observed in Wave 2**<br>N = 4,604
(Missing)	0 (0%)	4 (<0.1%)
Educational attainment (16+ years), n (%)		
Up to 8	296 (63.3%)	2,968 (64.5%)
9-15	130 (27.7%)	1,200 (26.1%)
16+	42 (9.0%)	437 (9.5%)
(Missing)	0 (0%)	0 (0%)
Currently employed, n (%)		
Employed for an employer	153 (32.8%)	1,772 (38.5%)
Self-employed	131 (28.0%)	1,177 (25.6%)
Retired	94 (20.1%)	839 (18.2%)
Student	15 (3.3%)	177 (3.9%)
Homemaker	31 (6.7%)	266 (5.8%)
Unemployed and looking for a job	19 (4.1%)	148 (3.2%)
None of these/Other	22 (4.7%)	224 (4.9%)
(Missing)	2 (0.3%)	0 (0%)
Financially comfortable/getting by, n (%)		
Living comfortably on present income	86 (18.3%)	773 (16.8%)
Getting by on present income	267 (57.1%)	2,911 (63.2%)
Finding it difficult on present income	90 (19.2%)	787 (17.1%)
Finding it very difficult on present income	24 (5.1%)	131 (2.8%)
(Missing)	1 (0.2%)	2 (0.0%)
Own home, n (%)		
Someone in this household owns this home	365 (78.1%)	3,783 (82.2%)
Someone in this household rents this home	73 (15.7%)	622 (13.5%)
Both	11 (2.3%)	97 (2.1%)
Neither	17 (3.7%)	98 (2.1%)
Rent	0 (0%)	0 (0%)
Own	0 (0%)	0 (0%)
Something else	0 (0%)	0 (0%)
(Missing)	1 (0.2%)	4 (0.1%)
Religious/spiritual connection, n (%)		
Always	44 (9.4%)	291 (6.3%)
Often	87 (18.7%)	743 (16.1%)
Rarely	108 (23.0%)	1,359 (29.5%)
Never	228 (48.7%)	2,208 (47.9%)
(Missing)	1 (0.3%)	4 (0.1%)
Belief in life after death, n (%)		
Yes	95 (20.3%)	1,104 (24.0%)
No	209 (44.6%)	1,918 (41.7%)
Unsure	163 (34.8%)	1,572 (34.1%)
(Missing)	1 (0.3%)	10 (0.2%)
Transformative religious experience, n (%)		
Yes	106 (22.6%)	1,032 (22.4%)
No	360 (76.9%)	3,564 (77.4%)
(Missing)	2 (0.5%)	8 (0.2%)
Religious reading or listening, n (%)		
More than once a day	7 (1.5%)	67 (1.4%)
About once a day	22 (4.8%)	208 (4.5%)
Sometimes	97 (20.7%)	926 (20.1%)

Table S12d. Unweighted summary statistics for Wave 1 outcome variables in China by retention status.

**Outcome**	**Attritors–Not Observed in Wave 2** N = 468	**Retained–Observed in Wave 2** N = 4,604
Never	338 (72.4%)	3,396 (73.8%)
(Missing)	3 (0.7%)	8 (0.2%)
Prayer or meditation, n (%)		
More than once a day	18 (3.9%)	102 (2.2%)
About once a day	43 (9.2%)	316 (6.9%)
Sometimes	126 (27.0%)	1,495 (32.5%)
Never	279 (59.6%)	2,688 (58.4%)
(Missing)	1 (0.3%)	3 (0.1%)
Belief in God/gods/spiritual forces, n (%)		
One God	14 (3.1%)	158 (3.4%)
More than one god	39 (8.3%)	434 (9.4%)
An impersonal spiritual force	95 (20.3%)	957 (20.8%)
None of these	255 (54.6%)	2,295 (49.8%)
Unsure	64 (13.7%)	759 (16.5%)
(Missing)	0 (0%)	2 (0.0%)
Religious centrality, n (%)		
Agree	56 (11.9%)	609 (13.2%)
Disagree	117 (25.0%)	1,141 (24.8%)
Not relevant	230 (49.1%)	2,145 (46.6%)
Unsure	65 (13.8%)	702 (15.2%)
(Missing)	1 (0.2%)	7 (0.2%)
Religious/spiritual comfort, n (%)		
Agree	58 (12.5%)	728 (15.8%)
Disagree	121 (25.9%)	1,161 (25.2%)
Not relevant	206 (44.0%)	2,028 (44.0%)
Unsure	81 (17.3%)	679 (14.7%)
(Missing)	1 (0.2%)	9 (0.2%)
Feel loved by God, n (%)		
Agree	49 (10.6%)	630 (13.7%)
Disagree	136 (29.0%)	1,228 (26.7%)
Not relevant	201 (42.9%)	2,005 (43.6%)
Unsure	80 (17.2%)	731 (15.9%)
(Missing)	2 (0.3%)	9 (0.2%)
Feel punished by God, n (%)		
Agree	33 (7.0%)	361 (7.8%)
Disagree	161 (34.3%)	1,631 (35.4%)
Not relevant	200 (42.7%)	1,943 (42.2%)
Unsure	71 (15.3%)	662 (14.4%)
(Missing)	3 (0.6%)	7 (0.1%)
Experienced religious criticism, n (%)		
Agree	19 (4.1%)	210 (4.6%)
Disagree	152 (32.4%)	1,669 (36.3%)
Not relevant	229 (48.9%)	2,183 (47.4%)
Unsure	67 (14.4%)	535 (11.6%)
(Missing)	1 (0.2%)	7 (0.1%)
Faith-sharing, n (%)		
Agree	51 (11.0%)	545 (11.8%)
Disagree	128 (27.4%)	1,347 (29.3%)
Not relevant	215 (46.0%)	2,089 (45.4%)

Table S12d. Unweighted summary statistics for Wave 1 outcome variables in China by retention status.

Outcome	Attritors–Not Observed in Wave 2<br>N = 468	Retained–Observed in Wave 2<br>N = 4,604
Unsure	70 (14.9%)	616 (13.4%)
(Missing)	3 (0.7%)	6 (0.1%)

Note. N (%); this table is based on non-imputed data. Cumulative percentages for variables may not add up to 100% due to rounding.

Table S12e. Summary of fitted attrition model in China

Characteristic	Odds Ratio	95% CI	p-value
**ANNUAL_WEIGHT_R2**	1.01	0.80, 1.29	0.912
**Recruitment Survey Mode**			
CAWI	—	—	
CATI	1.31	0.94, 1.82	0.111
**Happiness & life satisfaction**	0.83	0.69, 1.00	0.045
**Physical & mental health**	0.96	0.80, 1.14	0.613
**Meaning & purpose**	1.04	0.84, 1.28	0.731
**Character & virtue**	0.92	0.77, 1.10	0.356
**Close social relationships**	0.85	0.72, 1.02	0.075
**Financial & material security**	1.10	0.96, 1.26	0.190
**Extraversion**	1.07	0.95, 1.21	0.247
**Openness to experience**	1.02	0.90, 1.16	0.741
**Agreeableness**	1.01	0.89, 1.15	0.822
**Conscientiousness**	1.05	0.91, 1.21	0.545
**Neuroticism**	1.06	0.93, 1.22	0.378
**Depression symptoms composite**	0.98	0.87, 1.11	0.790
**Anxiety symptoms composite**	0.85	0.75, 0.95	0.006
**Loneliness**	0.99	0.86, 1.14	0.861
**Days exercise per week**	0.98	0.87, 1.09	0.676
**Year of birth (age group)**			
1973-1983 (current age: 40-49 years)	—	—	
1953-1963 (current age: 60-69 years)	0.81	0.53, 1.23	0.317
1983-1993 (current age: 30-39 years)	1.24	0.86, 1.77	0.245
1963-1973 (current age: 50-59 years)	1.04	0.72, 1.51	0.826
1993-1998 (current age: 25-29 years)	1.09	0.73, 1.61	0.676
1998-2005 (current age: 18-24 years)	0.90	0.51, 1.57	0.708
1943-1953 (current age: 70-79 years)	1.16	0.57, 2.35	0.684
1943 or earlier (current age: 80+ years)	27,059	6,009, 121,838	1.14e-39
**Gender of respondent**			
Male	—	—	
Female	1.26	1.01, 1.57	0.041
**Marital status**			
Married	—	—	
Single/Never been married	1.09	0.77, 1.56	0.618
Widowed	1.00	0.57, 1.77	1.000
Domestic partner	3.47	1.17, 10.3	0.025
Divorced	0.81	0.44, 1.49	0.497
Separated	0.45	0.20, 1.05	0.063
**Employment status**			
Employed for an employer	—	—	
Self-employed	0.92	0.68, 1.25	0.597
Retired	0.96	0.62, 1.50	0.874
Homemaker	0.92	0.58, 1.43	0.699
None of these/Other	1.15	0.72, 1.84	0.559
Student	1.39	0.72, 2.68	0.330
Unemployed and looking for a job	0.80	0.49, 1.31	0.369
**Religious attendance**			
Never	—	—	
A few times a year	1.23	0.89, 1.70	0.212
One to three times a month	1.21	0.79, 1.86	0.389
Once a week	1.47	0.86, 2.53	0.159
More than once a week	4.63	0.96, 22.3	0.056

Table S12e. Summary of fitted attrition model in China

Characteristic	Odds Ratio	95% CI	p-value
**Educational attainment (16+ years)**			
Up to 8	—	—	
9-15	0.89	0.65, 1.24	0.503
16+	0.88	0.59, 1.32	0.542
**Born in This country**			
Born in this country	—	—	
Born in another country	0.33	0.08, 1.36	0.126
**Urbanicity**			
A large city	—	—	
A small town or village	0.75	0.57, 0.99	0.040
A suburb of a large city	0.81	0.57, 1.15	0.243
A rural area or on a farm	0.89	0.63, 1.26	0.511
**Monthly household income**			
China: 10,001 – 12,000 RMB	—	—	
China: 16,001 – 20,000 RMB	2.22	1.29, 3.82	0.004
China: 20,001 – 30,000 RMB	1.51	0.88, 2.59	0.133
China: 5,001 – 6,000 RMB	1.16	0.70, 1.90	0.569
China: 14,001 – 16,000 RMB	1.22	0.73, 2.04	0.453
China: 9,001 – 10,000 RMB	1.36	0.79, 2.32	0.263
China: 12,001 – 14,000 RMB	1.57	0.91, 2.72	0.108
China: 4,001 – 5,000 RMB	0.71	0.43, 1.16	0.167
China: 6,001 – 7,000 RMB	1.24	0.73, 2.12	0.430
China: 7,001 – 8,000 RMB	0.81	0.49, 1.33	0.403
China: 2,501 – 4,000 RMB	0.92	0.53, 1.59	0.760
China: 8,001 – 9,000 RMB	0.72	0.45, 1.16	0.180
China: 30,001 – 50,000 RMB	1.53	0.78, 3.01	0.221
China: 1,001 – 2,500 RMB	0.70	0.33, 1.48	0.349
(None/No household income)	0.58	0.27, 1.27	0.174
China: More than 100,000 RMB	0.74	0.24, 2.29	0.601
China: 50,001 – 75,000 RMB	2.30	0.61, 8.67	0.217
China: 1,000 RMB or less	2.13	0.58, 7.86	0.258
China: 75,001 – 100,000 RMB	3.87	0.48, 31.1	0.204

Abbreviations: CI = Confidence Interval, OR = Odds Ratio

Notes. N=5022; attrition weights were estimated using the 'survey::svyglm(family=quasibinomial('logit'))' function. All continuous predictors were standardized and all categorical predictors used the most common category as the reference group. Reported p-values are based on the fitted regression model and no adjustments for multiple testing were done within this table.

Table S12f. Summary of principal components in China

PC	Percent Explained by each PC	Cumulative Percent Explained
1	25.60	25.60
2	9.55	35.15
3	6.21	41.36
4	3.45	44.81
5	2.36	47.17
6	1.98	49.15
**7**	**1.79**	**50.94**
8	1.64	52.57
9	1.58	54.15
10	1.50	55.66
11	1.40	57.06
12	1.36	58.42
13	1.32	59.75
14	1.30	61.04
15	1.27	62.31
16	1.18	63.48
17	1.14	64.62
18	1.08	65.70
19	1.06	66.76
20	1.03	67.79

Notes. N=5022; PCA was conducted using ’survey::svyprcomp(.)’ function using all available contemporaneous exposures at wave 1. All PCs were standardized prior to being used as predictors. The bolded row represented the number of retained components for analysis was 7.

Table S12g. Associations of forgivingness with adult well-being and other outcomes at Wave 2 in China

	Model 1: Demographic and Childhood Variables as Covariates					Model 2: Demographic, Childhood, and Other Wave 1 Confounding Variables (Via Principal Components) as Covariates				
Outcome	RR	ES	95% CI	SE	p-value	RR	ES	95% CI	SE	p-value
Human Flourishing										
Secure flourishing index		0.07	(0.04,0.10)	0.02	2.48e-06***		0.03	(-0.00,0.06)	0.02	0.084
Flourishing index		0.07	(0.04,0.10)	0.02	3.87e-06***		0.02	(-0.01,0.06)	0.02	0.107
Happiness & life satisfaction		0.05	(0.02,0.09)	0.02	5.97e-04***		0.02	(-0.01,0.05)	0.02	0.178
Physical & mental health		0.04	(0.01,0.08)	0.02	0.005*		0.01	(-0.02,0.04)	0.02	0.510
Meaning & purpose		0.06	(0.02,0.09)	0.02	4.26e-04***		0.01	(-0.02,0.04)	0.02	0.435
Character & virtue		0.08	(0.05,0.11)	0.02	7.07e-07***		0.04	(0.01,0.08)	0.02	0.010*
Close social relationships		0.06	(0.03,0.09)	0.02	3.7e-04***		0.02	(-0.02,0.05)	0.02	0.297
Financial & material security		0.05	(0.02,0.08)	0.02	2.18e-03**		0.02	(-0.01,0.06)	0.02	0.168
Psychological Well-Being										
Happiness (a)		0.05	(0.02,0.08)	0.02	8e-04**		0.02	(-0.01,0.05)	0.02	0.170
Life satisfaction (a)		0.05	(0.01,0.08)	0.02	0.006*		0.02	(-0.02,0.05)	0.02	0.297
Current life evaluation		0.04	(0.01,0.07)	0.02	0.014*		0.02	(-0.01,0.05)	0.02	0.289
Future life evaluation		0.04	(0.01,0.07)	0.02	0.009*		0.02	(-0.02,0.05)	0.02	0.327
Optimism		0.06	(0.02,0.09)	0.02	1.05e-03**		0.02	(-0.01,0.06)	0.02	0.177
Freedom to pursue what's important		0.06	(0.03,0.09)	0.02	4.46e-04***		0.02	(-0.02,0.05)	0.02	0.308
Inner peace	1.00		(0.99,1.01)	0.01	0.922	0.99		(0.98,1.00)	0.01	0.236
Life balance	1.01		(1.00,1.02)	0.01	0.211	1.00		(0.99,1.01)	0.01	0.853
Sense of mastery	1.00		(0.99,1.02)	0.01	0.477	1.00		(0.99,1.01)	0.01	0.889
Meaningful activities (c)		0.05	(0.02,0.08)	0.02	1.51e-03**		0.02	(-0.01,0.05)	0.02	0.271
Understanding purpose (c)		0.05	(0.01,0.08)	0.02	4.21e-03**		0.00	(-0.03,0.04)	0.02	0.781
Self-rated mental health (b)		0.05	(0.01,0.08)	0.02	0.006*		0.02	(-0.02,0.05)	0.02	0.322
Psychological Distress										
Traumatic distress	0.99		(0.98,1.00)	0.01	0.107	0.99		(0.98,1.00)	0.01	0.126
Depression symptoms composite	1.00		(0.99,1.01)	0.01	0.861	1.00		(0.99,1.01)	0.01	0.941
Depression – feel hopeless	1.00		(0.99,1.01)	0.01	0.818	1.00		(0.99,1.01)	0.01	0.385
Depression – loss of interest	1.00		(0.99,1.01)	0.01	0.924	1.00		(0.98,1.01)	0.01	0.862
Anxiety symptoms composite	0.99		(0.98,1.00)	0.01	0.148	0.99		(0.98,1.01)	0.01	0.301
Anxiety – feel on edge	1.00		(0.99,1.01)	0.01	0.726	1.00		(0.99,1.01)	0.01	0.908
Anxiety – cannot stop worrying	1.00		(0.98,1.01)	0.01	0.373	1.00		(0.99,1.01)	0.01	0.641
Suffering	1.00		(0.98,1.01)	0.01	0.545	1.00		(0.99,1.02)	0.01	0.908
Social Well-Being										
Relationship contentment (e)		0.06	(0.03,0.09)	0.02	4.27e-04***		0.02	(-0.01,0.06)	0.02	0.194
Relationship satisfaction (e)		0.05	(0.01,0.08)	0.02	0.005*		0.01	(-0.02,0.04)	0.02	0.550
Social support		0.04	(0.01,0.07)	0.02	0.013*		0.02	(-0.01,0.05)	0.02	0.283
Intimate/close friend	1.00		(0.99,1.01)	0.01	0.584	0.99		(0.98,1.00)	0.01	0.097
Government approval										
Say in government										
Belonging in country										
City/place satisfaction	1.01		(0.99,1.02)	0.01	0.400	1.00		(0.99,1.01)	0.01	0.842
Trust within country	1.00		(0.99,1.01)	0.01	0.611	1.00		(0.98,1.01)	0.01	0.445
Social Participation										
Ever been married	1.00		(1.00,1.01)	0.00	0.151	1.00		(1.00,1.01)	0.00	0.139
Currently divorced	1.00		(1.00,1.00)	0.00	0.881	1.00		(1.00,1.00)	0.00	0.738
Number of children		-0.01	(-0.04,0.02)	0.01	0.493		-0.03	(-0.05,-0.00)	0.01	0.049*
Weekly+ community participation	1.01		(1.00,1.02)	0.01	0.166	1.00		(0.99,1.01)	0.01	0.611
Weekly+ religious attendance	1.00		(0.99,1.00)	0.00	0.516	1.00		(0.99,1.00)	0.00	0.180
Social Distress										
Loneliness		-0.03	(-0.06,0.00)	0.02	0.051		-0.01	(-0.04,0.02)	0.02	0.497
Perceived discrimination	1.03		(1.02,1.04)	0.01	1.01e-07***	1.02		(1.01,1.03)	0.01	1.07e-04***
Character & Prosocial Behavior										
Orientation to promote good (d)		0.07	(0.03,0.10)	0.02	1.39e-04***		0.03	(-0.01,0.06)	0.02	0.117
Delayed gratification (d)		0.07	(0.04,0.11)	0.02	6.87e-06***		0.05	(0.01,0.08)	0.02	0.006*
Hope		0.08	(0.04,0.11)	0.02	2.4e-06***		0.04	(0.00,0.07)	0.02	0.027*
Gratitude		0.05	(0.02,0.09)	0.02	1.21e-03**		0.02	(-0.02,0.05)	0.02	0.288
Showing love/care		0.06	(0.03,0.10)	0.02	1.97e-04***		0.03	(-0.00,0.06)	0.02	0.088
Forgivingness	1.10		(1.08,1.12)	0.01	2.22e-16***	1.09		(1.08,1.11)	0.01	2.22e-16***
Charitable giving	1.01		(0.99,1.02)	0.01	0.366	0.99		(0.98,1.01)	0.01	0.205
Helping strangers	1.02		(1.00,1.03)	0.01	0.029*	1.01		(0.99,1.02)	0.01	0.446
Volunteering	1.01		(0.99,1.02)	0.01	0.388	0.99		(0.98,1.01)	0.01	0.250
Physical Health & Health Behavior										
Self-rated physical health (b)		0.03	(-0.00,0.06)	0.02	0.064		0.00	(-0.03,0.03)	0.02	0.957
Health problems	1.00		(0.99,1.01)	0.01	0.600	0.99		(0.98,1.00)	0.01	0.238
Pain in past 4 weeks	1.00		(0.99,1.02)	0.01	0.534	1.01		(0.99,1.02)	0.01	0.376
Daily smoker	1.00		(0.99,1.02)	0.01	0.925	1.00		(0.98,1.01)	0.01	0.619
Number of drinks per week		-0.02	(-0.06,0.01)	0.02	0.176		-0.04	(-0.07,-0.00)	0.02	0.040*
Days exercise per week		0.06	(0.03,0.10)	0.02	2.35e-04***		0.03	(-0.00,0.07)	0.02	0.056
Socioeconomic Outcomes										
Financial security (f)		0.04	(0.01,0.07)	0.02	0.012*		0.02	(-0.02,0.05)	0.02	0.337
Material security (f)		0.05	(0.01,0.08)	0.02	0.005*		0.02	(-0.01,0.06)	0.02	0.148
Educational attainment (16+ years)	1.00		(1.00,1.00)	0.00	0.848	1.00		(1.00,1.00)	0.00	0.932
Currently employed	1.00		(0.99,1.01)	0.01	0.979	1.00		(0.99,1.01)	0.01	0.562
Financially comfortable/getting by	1.00		(0.99,1.01)	0.01	0.754	0.99		(0.98,1.00)	0.01	0.116
Own home	0.99		(0.98,1.00)	0.01	0.080	0.98		(0.97,1.00)	0.01	0.006*
Income – top quintile	0.99		(0.98,1.01)	0.01	0.443	0.99		(0.98,1.00)	0.01	0.073
Religion & Spirituality										
Religious/spiritual connection	1.02		(1.01,1.03)	0.01	4.09e-03**	1.01		(0.99,1.02)	0.01	0.369
Belief in life after death	0.99		(0.98,1.01)	0.01	0.351	0.99		(0.98,1.00)	0.01	0.227
Transformative religious experience	1.00		(0.99,1.02)	0.01	0.409	1.00		(0.99,1.01)	0.01	0.609
Religious reading or listening	1.00		(0.99,1.01)	0.00	0.763	1.00		(0.99,1.00)	0.00	0.177
Prayer or meditation	1.00		(0.99,1.01)	0.00	0.784	1.00		(0.99,1.00)	0.00	0.359
Belief in God/gods/spiritual forces	0.99		(0.98,1.01)	0.01	0.283	0.99		(0.98,1.01)	0.01	0.339
Religious centrality	1.00		(0.99,1.01)	0.00	0.549	1.00		(0.99,1.01)	0.00	0.506
Religious/spiritual comfort	1.00		(0.99,1.01)	0.00	0.970	1.00		(0.99,1.01)	0.00	0.604
Feel loved by God	1.00		(0.99,1.01)	0.00	0.479	0.99		(0.99,1.00)	0.00	0.125
Feel punished by God	1.01		(1.01,1.02)	0.00	7.18e-04**	1.01		(1.00,1.02)	0.00	0.010*
Experienced religious criticism	1.00		(0.99,1.01)	0.00	0.734	1.00		(0.99,1.00)	0.00	0.275
Faith-sharing	1.00		(0.99,1.01)	0.00	0.555	1.00		(0.99,1.01)	0.00	0.860

Notes. N=5022; Reference for focal predictor: never/rarely. RR, risk-ratio, null effect is 1.00; ES, effect size measure for standardized regression coefficient, null effect is 0.00; SE, standard error, the SE reported for binary/Likert-type outcomes where risk-ratios are on the log(RR) scale; CI, confidence interval; p-value, a Wald-type test of the null hypothesis that the effect of the focal predictor is zero; (a) item part of the Happiness & Life Satisfaction domain of the Secure Flourishing Index; (b) item part of the Physical & Mental Health domain of the Secure Flourishing Index; (c) item part of the Meaning & Purpose domain of the Secure Flourishing Index; (d) item part of the Character & Virtue domain of the Secure Flourishing Index; (e) item part of the Subjective Social Connectedness domain of the Secure Flourishing Index; (f) item part of the Financial & Material Security domain of the Secure Flourishing Index.

Multiple imputation was performed to impute missing data on the covariates, exposure, and outcomes. All models controlled for sociodemographic and childhood factors assessed at Wave 1. For Model 2 with PC (principal components), the first seven principal components of the entire set of contemporaneous confounders assessed at Wave 1 were included as additional covariates of the outcomes at Wave 2.

An outcome-wide analytic approach was used, and a separate model was run for each outcome. A different type of model was run depending on the nature of the outcome: (1) for each binary outcome, a weighted generalized linear model (with a log link and Poisson distribution) was used to estimate an RR; and (2) for each continuous outcome, a weighted linear regression model was used to estimate a ES. All effect sizes were standardized. For continuous outcomes, the ES represents the change in SD on the outcome between the lower and upper categories of the binary focal predictor. For binary outcomes, the RR represents the change in risk of being in the upper category compared to the lower category between the lower and upper categories of the binary focal predictor.

P-value significance thresholds: p < 0.05*, p < 0.005**, (Bonferroni) p < 6.41e-04***, correction for multiple testing using Bonferroni adjusted significant threshold.

Table S12h. Associations of forgivingness with adult well-being and other outcomes at Wave 2 in China using complete-case analyses with attrition weights.

	Model 1: Demographic and Childhood Variables as Covariates					Model 2: Demographic, Childhood, and Other Wave 1 Confounding Variables (Via Principal Components) as Covariates				
Outcome	RR	ES	95% CI	SE	p-value	RR	ES	95% CI	SE	p-value
Human Flourishing										
Secure flourishing index		0.07	(0.04,0.10)	0.02	1.49e-05***		0.02	(-0.01,0.06)	0.02	0.145
Flourishing index		0.07	(0.04,0.10)	0.02	2.09e-05***		0.02	(-0.01,0.05)	0.02	0.175
Happiness & life satisfaction		0.05	(0.02,0.08)	0.02	1.53e-03**		0.02	(-0.01,0.05)	0.02	0.284
Physical & mental health		0.05	(0.01,0.08)	0.02	4.1e-03**		0.01	(-0.02,0.04)	0.02	0.558
Meaning & purpose		0.05	(0.02,0.09)	0.02	7.29e-04**		0.01	(-0.02,0.04)	0.02	0.545
Character & virtue		0.08	(0.05,0.11)	0.02	1.08e-06***		0.04	(0.01,0.07)	0.02	0.014*
Close social relationships		0.06	(0.03,0.09)	0.02	2.34e-04***		0.02	(-0.02,0.05)	0.02	0.339
Financial & material security		0.05	(0.02,0.08)	0.02	3.87e-03**		0.02	(-0.01,0.05)	0.02	0.221
Psychological Well-Being										
Happiness (a)		0.05	(0.02,0.08)	0.02	1.7e-03**		0.02	(-0.01,0.05)	0.02	0.251
Life satisfaction (a)		0.05	(0.01,0.08)	0.02	4.91e-03**		0.01	(-0.02,0.05)	0.02	0.383
Current life evaluation		0.04	(0.01,0.07)	0.02	0.018*		0.01	(-0.02,0.05)	0.02	0.403
Future life evaluation		0.04	(0.01,0.07)	0.02	0.017*		0.01	(-0.02,0.05)	0.02	0.449
Optimism		0.06	(0.02,0.09)	0.02	7.38e-04**		0.02	(-0.01,0.06)	0.02	0.162
Freedom to pursue what's important		0.06	(0.02,0.09)	0.02	5.32e-04***		0.01	(-0.02,0.05)	0.02	0.380
Inner peace	1.00		(0.99,1.01)	0.01	0.826	0.99		(0.98,1.00)	0.01	0.169
Life balance	1.01		(1.00,1.02)	0.01	0.214	1.00		(0.99,1.01)	0.01	0.809
Sense of mastery	1.00		(0.99,1.02)	0.01	0.404	1.00		(0.99,1.01)	0.01	0.994
Meaningful activities (c)		0.05	(0.02,0.08)	0.02	2.05e-03**		0.01	(-0.02,0.05)	0.02	0.385
Understanding purpose (c)		0.05	(0.02,0.08)	0.02	3.31e-03**		0.00	(-0.03,0.04)	0.02	0.814
Self-rated mental health (b)		0.05	(0.02,0.08)	0.02	2.75e-03**		0.02	(-0.02,0.05)	0.02	0.345
Psychological Distress										
Traumatic distress	0.99		(0.98,1.00)	0.01	0.076	0.99		(0.98,1.00)	0.01	0.101
Depression symptoms composite	1.00		(0.98,1.01)	0.01	0.818	1.00		(0.99,1.01)	0.01	0.992
Depression – feel hopeless	1.00		(0.99,1.01)	0.00	0.962	1.00		(0.99,1.01)	0.01	0.475
Depression – loss of interest	1.00		(0.99,1.01)	0.01	0.906	1.00		(0.98,1.01)	0.01	0.845
Anxiety symptoms composite	0.99		(0.98,1.00)	0.01	0.164	0.99		(0.98,1.01)	0.01	0.311
Anxiety – feel on edge	1.00		(0.99,1.01)	0.01	0.681	1.00		(0.99,1.01)	0.01	0.964
Anxiety – cannot stop worrying	1.00		(0.99,1.01)	0.01	0.409	1.00		(0.99,1.01)	0.01	0.694
Suffering	0.99		(0.98,1.01)	0.01	0.484	1.00		(0.99,1.02)	0.01	0.927
Social Well-Being										
Relationship contentment (e)		0.06	(0.03,0.09)	0.02	9.9e-05***		0.02	(-0.01,0.05)	0.02	0.190
Relationship satisfaction (e)		0.05	(0.02,0.08)	0.02	1.51e-03**		0.01	(-0.02,0.04)	0.02	0.594
Social support		0.04	(0.01,0.07)	0.02	0.023*		0.01	(-0.02,0.04)	0.02	0.463
Intimate/close friend	1.00		(0.99,1.01)	0.00	0.390	0.99		(0.98,1.00)	0.00	0.034*
Government approval										
Say in government										
Belonging in country										
City/place satisfaction	1.00		(0.99,1.02)	0.01	0.558	1.00		(0.98,1.01)	0.01	0.567
Trust within country	1.00		(0.99,1.01)	0.01	0.662	1.00		(0.98,1.01)	0.01	0.374
Social Participation										
Ever been married	1.00		(1.00,1.01)	0.00	0.142	1.00		(1.00,1.01)	0.00	0.113
Currently divorced	1.00		(1.00,1.00)	0.00	0.746	1.00		(1.00,1.00)	0.00	0.537
Number of children		-0.02	(-0.05,0.01)	0.02	0.165		-0.03	(-0.06,-0.00)	0.01	0.043*
Weekly+ community participation	1.01		(1.00,1.02)	0.01	0.172	1.00		(0.99,1.01)	0.01	0.480
Weekly+ religious attendance	1.00		(0.99,1.00)	0.00	0.543	1.00		(0.99,1.00)	0.00	0.172
Social Distress										
Loneliness		-0.03	(-0.06,0.00)	0.02	0.083		-0.01	(-0.04,0.03)	0.02	0.688
Perceived discrimination	1.03		(1.02,1.04)	0.01	9.93e-09***	1.02		(1.01,1.04)	0.01	5.02e-06***
Character & Prosocial Behavior										
Orientation to promote good (d)		0.07	(0.04,0.10)	0.02	1.92e-05***		0.03	(-0.00,0.06)	0.02	0.093
Delayed gratification (d)		0.07	(0.04,0.11)	0.02	1.15e-05***		0.04	(0.01,0.08)	0.02	0.009*
Hope		0.08	(0.05,0.11)	0.02	1.68e-06***		0.04	(0.00,0.07)	0.02	0.029*
Gratitude		0.05	(0.02,0.08)	0.02	1.03e-03**		0.02	(-0.02,0.05)	0.02	0.332
Showing love/care		0.06	(0.03,0.10)	0.02	7.51e-05***		0.03	(-0.00,0.06)	0.02	0.090
Forgivingness	1.10		(1.08,1.12)	0.01	2.22e-16***	1.10		(1.08,1.12)	0.01	2.22e-16***
Charitable giving	1.01		(0.99,1.02)	0.01	0.474	0.99		(0.97,1.00)	0.01	0.146
Helping strangers	1.02		(1.00,1.03)	0.01	0.036*	1.00		(0.99,1.02)	0.01	0.555
Volunteering	1.01		(0.99,1.02)	0.01	0.388	0.99		(0.98,1.01)	0.01	0.218
Physical Health & Health Behavior										
Self-rated physical health (b)		0.03	(0.00,0.06)	0.02	0.037*		0.00	(-0.03,0.03)	0.02	0.946
Health problems	1.00		(0.98,1.01)	0.01	0.564	0.99		(0.98,1.00)	0.01	0.241
Pain in past 4 weeks	1.00		(0.99,1.02)	0.01	0.625	1.01		(0.99,1.02)	0.01	0.382
Daily smoker	1.00		(0.99,1.01)	0.01	0.962	1.00		(0.98,1.01)	0.01	0.624
Number of drinks per week		-0.02	(-0.06,0.01)	0.02	0.124		-0.04	(-0.07,-0.01)	0.02	0.023*
Days exercise per week		0.06	(0.03,0.09)	0.02	3.23e-04***		0.03	(-0.00,0.06)	0.02	0.082
Socioeconomic Outcomes										
Financial security (f)		0.04	(0.01,0.07)	0.02	0.014*		0.01	(-0.02,0.05)	0.02	0.383
Material security (f)		0.05	(0.01,0.08)	0.02	4.81e-03**		0.02	(-0.01,0.06)	0.02	0.172
Educational attainment (16+ years)	1.00		(1.00,1.00)	0.00	0.994	1.00		(1.00,1.00)	0.00	0.981
Currently employed	1.00		(0.99,1.01)	0.00	0.815	1.00		(0.99,1.00)	0.00	0.347
Financially comfortable/getting by	1.00		(0.99,1.01)	0.01	0.766	0.99		(0.98,1.00)	0.01	0.118
Own home	0.99		(0.98,1.00)	0.01	0.113	0.99		(0.98,1.00)	0.01	0.014*
Income – top quintile	1.00		(0.98,1.01)	0.01	0.674	0.99		(0.98,1.00)	0.01	0.144
Religion & Spirituality										
Religious/spiritual connection	1.02		(1.01,1.03)	0.01	4.01e-03**	1.01		(1.00,1.02)	0.01	0.235
Belief in life after death	0.99		(0.98,1.01)	0.01	0.381	0.99		(0.98,1.01)	0.01	0.248
Transformative religious experience	1.00		(0.99,1.01)	0.01	0.431	1.00		(0.99,1.01)	0.00	0.522
Religious reading or listening	1.00		(0.99,1.00)	0.00	0.609	0.99		(0.99,1.00)	0.00	0.109
Prayer or meditation	1.00		(0.99,1.01)	0.00	0.784	1.00		(0.99,1.00)	0.00	0.360
Belief in God/gods/spiritual forces	0.99		(0.98,1.00)	0.01	0.194	0.99		(0.98,1.00)	0.01	0.266
Religious centrality	1.00		(0.99,1.01)	0.00	0.491	1.00		(0.99,1.01)	0.00	0.477
Religious/spiritual comfort	1.00		(0.99,1.01)	0.00	0.953	1.00		(0.99,1.00)	0.00	0.476
Feel loved by God	1.00		(0.99,1.01)	0.00	0.461	0.99		(0.99,1.00)	0.00	0.099
Feel punished by God	1.01		(1.01,1.02)	0.00	4.26e-04***	1.01		(1.00,1.02)	0.00	0.007*
Experienced religious criticism	1.00		(0.99,1.01)	0.00	0.829	1.00		(0.99,1.00)	0.00	0.171
Faith-sharing	1.00		(0.99,1.01)	0.00	0.452	1.00		(0.99,1.01)	0.00	0.992

Table S12h. Associations of forgivingness with adult well-being and other outcomes at Wave 2 in China using complete-case analyses with attrition weights.

	Model 1: Demographic and Childhood Variables as Covariates					Model 2: Demographic, Childhood, and Other Wave 1 Confounding Variables (Via Principal Components) as Covariates				
Outcome	RR	ES	95% CI	SE	p-value	RR	ES	95% CI	SE	p-value

Notes. N=4544; Reference for focal predictor: never/rarely. RR, risk-ratio, null effect is 1.00; ES, effect size measure for standardized regression coefficient, null effect is 0.00; SE, standard error, the SE reported for binary/Likert-type outcomes where risk-ratios are on the log(RR) scale; CI, confidence interval; p-value, a Wald-type test of the null hypothesis that the effect of the focal predictor is zero; (a) item part of the Happiness & Life Satisfaction domain of the Secure Flourishing Index; (b) item part of the Physical & Mental Health domain of the Secure Flourishing Index; (c) item part of the Meaning & Purpose domain of the Secure Flourishing Index; (d) item part of the Character & Virtue domain of the Secure Flourishing Index; (e) item part of the Subjective Social Connectedness domain of the Secure Flourishing Index; (f) item part of the Financial & Material Security domain of the Secure Flourishing Index.

Attrition weights were computed to adjust the complete case data (those who responded at Wave 2 to at least 50% of the questions) and multiple imputation was used to impute missing data on all remaining within wave on the covariates, exposure, and outcomes. All models controlled for sociodemographic and childhood factors assessed at Wave 1. For Model 2 with PC (principal components), the first seven principal components of the entire set of contemporaneous confounders assessed at Wave 1 were included as additional covariates of the outcomes at Wave 2.

An outcome-wide analytic approach was used, and a separate model was run for each outcome. A different type of model was run depending on the nature of the outcome: (1) for each binary outcome, a weighted generalized linear model (with a log link and Poisson distribution) was used to estimate an RR; and (2) for each continuous outcome, a weighted linear regression model was used to estimate a ES. All effect sizes were standardized. For continuous outcomes, the ES represents the change in SD on the outcome between the lower and upper categories of the binary focal predictor. For binary outcomes, the RR represents the change in risk of being in the upper category compared to the lower category between the lower and upper categories of the binary focal predictor.

P-value significance thresholds: $p < 0.05$*, $p < 0.005$**, (Bonferroni) $p < 6.41e-04$***, correction for multiple testing using Bonferroni adjusted significant threshold.

Table S12i. Sensitivity analysis of forgivingness outcome-wide results to unmeasured confounding using E-values in China

	Multiple Imputation				Complete Case w/ Attrition Weights			
	Model 1: Demographics and Childhood Variables as Covariates		Model 2: Demographics, Childhood, and Other Wave 1 Confounders (Via Principal Components) as Covariates		Model 1: Demographics and Childhood Variables as Covariates		Model 2: Demographics, Childhood, and Other Wave 1 Confounders (Via Principal Components) as Covariates	
Outcome	EE	ECI	EE	ECI	EE	ECI	EE	ECI
Human Flourishing								
Secure flourishing index	1.54	1.37	1.28	1.00	1.52	1.35	1.26	1.00
Flourishing index	1.53	1.37	1.27	1.00	1.52	1.34	1.25	1.00
Happiness & life satisfaction	1.45	1.26	1.25	1.00	1.43	1.23	1.22	1.00
Physical & mental health	1.39	1.19	1.16	1.00	1.40	1.19	1.15	1.00
Meaning & purpose	1.45	1.27	1.18	1.00	1.44	1.25	1.15	1.00
Character & virtue	1.59	1.42	1.38	1.16	1.59	1.41	1.37	1.14
Close social relationships	1.47	1.28	1.21	1.00	1.47	1.28	1.20	1.00
Financial & material security	1.41	1.22	1.26	1.00	1.40	1.20	1.24	1.00
Psychological Well-Being								
Happiness	1.44	1.25	1.25	1.00	1.42	1.23	1.22	1.00
Life satisfaction	1.40	1.19	1.22	1.00	1.40	1.19	1.19	1.00
Current life evaluation	1.37	1.14	1.22	1.00	1.35	1.12	1.19	1.00
Future life evaluation	1.37	1.16	1.21	1.00	1.36	1.13	1.18	1.00
Optimism	1.45	1.25	1.26	1.00	1.45	1.26	1.26	1.00
Freedom to pursue what's important	1.46	1.27	1.21	1.00	1.45	1.26	1.19	1.00
Inner peace	1.03	1.00	1.13	1.00	1.05	1.00	1.14	1.00
Life balance	1.14	1.00	1.05	1.00	1.14	1.00	1.06	1.00
Sense of mastery	1.10	1.00	1.04	1.00	1.11	1.00	1.01	1.00
Meaningful activities	1.43	1.23	1.22	1.00	1.42	1.22	1.19	1.00
Understanding purpose	1.40	1.19	1.10	1.00	1.40	1.20	1.09	1.00
Self-rated mental health	1.40	1.19	1.21	1.00	1.41	1.21	1.20	1.00
Psychological Distress								
Traumatic distress	1.17	1.00	1.17	1.00	1.18	1.00	1.17	1.00
Depression symptoms composite	1.05	1.00	1.03	1.00	1.06	1.00	1.01	1.00
Depression – feel hopeless	1.05	1.00	1.11	1.00	1.02	1.00	1.09	1.00
Depression – loss of interest	1.04	1.00	1.05	1.00	1.04	1.00	1.06	1.00
Anxiety symptoms composite	1.15	1.00	1.12	1.00	1.15	1.00	1.12	1.00
Anxiety – feel on edge	1.07	1.00	1.04	1.00	1.07	1.00	1.02	1.00
Anxiety – cannot stop worrying	1.11	1.00	1.08	1.00	1.10	1.00	1.07	1.00
Suffering	1.11	1.00	1.04	1.00	1.12	1.00	1.04	1.00
Social Well-Being								
Relationship contentment	1.48	1.28	1.25	1.00	1.49	1.31	1.24	1.00
Relationship satisfaction	1.41	1.19	1.16	1.00	1.42	1.23	1.14	1.00
Social support	1.37	1.14	1.22	1.00	1.34	1.11	1.17	1.00
Intimate/close friend	1.08	1.00	1.15	1.00	1.10	1.00	1.17	1.04
Government approval								
Say in government								
Belonging in country								
City/place satisfaction	1.11	1.00	1.05	1.00	1.09	1.00	1.09	1.00
Trust within country	1.08	1.00	1.10	1.00	1.07	1.00	1.11	1.00
Social Participation								
Ever been married	1.07	1.00	1.07	1.00	1.07	1.00	1.08	1.00
Currently divorced	1.03	1.00	1.04	1.00	1.03	1.00	1.04	1.00
Number of children	1.16	1.00	1.28	1.02	1.24	1.00	1.30	1.04
Weekly+ community participation	1.14	1.00	1.08	1.00	1.14	1.00	1.10	1.00
Weekly+ religious attendance	1.06	1.00	1.10	1.00	1.06	1.00	1.10	1.00
Social Distress								
Loneliness	1.32	1.00	1.17	1.00	1.29	1.00	1.12	1.00
Perceived discrimination	1.31	1.24	1.25	1.17	1.33	1.25	1.28	1.20
Character & Prosocial Behavior								
Orientation to promote good	1.52	1.32	1.29	1.00	1.54	1.35	1.29	1.00
Delayed gratification	1.56	1.38	1.40	1.18	1.55	1.36	1.38	1.17
Hope	1.56	1.39	1.34	1.10	1.57	1.39	1.34	1.09
Gratitude	1.44	1.24	1.22	1.00	1.44	1.24	1.20	1.00
Showing love/care	1.50	1.30	1.30	1.00	1.50	1.32	1.29	1.00
Forgivingness	1.71	1.61	1.69	1.59	1.72	1.63	1.70	1.61
Charitable giving	1.13	1.00	1.16	1.00	1.12	1.00	1.17	1.00
Helping strangers	1.22	1.06	1.12	1.00	1.22	1.05	1.11	1.00
Volunteering	1.13	1.00	1.15	1.00	1.13	1.00	1.15	1.00
Physical Health & Health Behavior								
Self-rated physical health	1.30	1.00	1.04	1.00	1.32	1.06	1.05	1.00
Health problems	1.09	1.00	1.14	1.00	1.09	1.00	1.14	1.00
Pain in past 4 weeks	1.11	1.00	1.13	1.00	1.09	1.00	1.13	1.00
Daily smoker	1.04	1.00	1.09	1.00	1.03	1.00	1.09	1.00
Number of drinks per week	1.26	1.00	1.33	1.06	1.27	1.00	1.34	1.11
Days exercise per week	1.49	1.30	1.32	1.00	1.47	1.28	1.30	1.00
Socioeconomic Outcomes								
Financial security	1.37	1.15	1.21	1.00	1.36	1.14	1.19	1.00
Material security	1.40	1.19	1.27	1.00	1.40	1.19	1.26	1.00
Educational attainment (16+ years)	1.00	1.00	1.00	1.00	1.00	1.00	1.00	1.00
Currently employed	1.02	1.00	1.09	1.00	1.05	1.00	1.11	1.00
Financially comfortable/getting by	1.07	1.00	1.17	1.00	1.07	1.00	1.17	1.00
Own home	1.17	1.00	1.21	1.10	1.15	1.00	1.20	1.08
Income – top quintile	1.11	1.00	1.18	1.00	1.08	1.00	1.17	1.00
Religion & Spirituality								
Religious/spiritual connection	1.22	1.11	1.11	1.00	1.22	1.11	1.13	1.00
Belief in life after death	1.12	1.00	1.14	1.00	1.12	1.00	1.14	1.00
Transformative religious experience	1.10	1.00	1.08	1.00	1.10	1.00	1.09	1.00
Religious reading or listening	1.05	1.00	1.11	1.00	1.06	1.00	1.12	1.00
Prayer or meditation	1.05	1.00	1.10	1.00	1.05	1.00	1.10	1.00
Belief in God/gods/spiritual forces	1.13	1.00	1.12	1.00	1.14	1.00	1.13	1.00
Religious centrality	1.08	1.00	1.08	1.00	1.09	1.00	1.08	1.00
Religious/spiritual comfort	1.02	1.00	1.07	1.00	1.02	1.00	1.08	1.00
Feel loved by God	1.09	1.00	1.13	1.00	1.09	1.00	1.13	1.00
Feel punished by God	1.19	1.12	1.16	1.08	1.19	1.12	1.16	1.08
Experienced religious criticism	1.05	1.00	1.09	1.00	1.04	1.00	1.10	1.00
Faith-sharing	1.08	1.00	1.04	1.00	1.09	1.00	1.01	1.00

Notes. EE, E-value for estimate; ECI, E-value for the limit of the confidence interval. The formula for calculating E-values can be found in VanderWeele and Ding (2017). E-values for estimate are the minimum strength of association on the risk ratio scale that an unmeasured confounder would need to have with both the exposure and the outcome to fully explain away the observed association between the exposure and outcome, conditional on the measured covariates. E-values for the 95% CI closest to the null denote the minimum strength of association on the risk ratio scale that an unmeasured confounder would need to have with both the exposure and the outcome to shift the CI to include the null value, conditional on the measured covariates.

Table S13a. Weighted summary statistics for demographic and childhood variables in Egypt

**Characteristic**	**Wave 1** N = 4,729	**Wave 2** N = 3,057
Forgivingness, n (%)		
Always	2,723 (57.6%)	1,738 (56.8%)
Often	1,379 (29.2%)	827 (27.1%)
Rarely	429 (9.1%)	302 (9.9%)
Never	196 (4.1%)	183 (6.0%)
(Missing)	3 (0.1%)	7 (0.2%)
Year of birth, n (%)		
1943 or earlier (current age: 80+ years)	7 (0.1%)	0 (0%)
1943-1953 (current age: 70-79 years)	52 (1.1%)	53 (1.7%)
1953-1963 (current age: 60-69 years)	389 (8.2%)	275 (9.0%)
1963-1973 (current age: 50-59 years)	568 (12.0%)	385 (12.6%)
1973-1983 (current age: 40-49 years)	814 (17.2%)	566 (18.5%)
1983-1993 (current age: 30-39 years)	1,139 (24.1%)	742 (24.3%)
1993-1998 (current age: 25-29 years)	603 (12.7%)	418 (13.7%)
1998-2005 (current age: 18-24 years)	1,157 (24.5%)	617 (20.2%)
(Missing)	0 (0%)	0 (0%)
Age of participant		
Mean	37.0	38.3
Standard Deviation	14.2	14.1
Min, Max	18.0, 85.0	19.0, 77.0
Gender, n (%)		
Male	2,427 (51.3%)	1,568 (51.3%)
Female	2,302 (48.7%)	1,489 (48.7%)
Other	0 (0%)	0 (0%)
(Missing)	0 (<0.0%)	0 (0.0%)
Respondent marital status, n (%)		
Single/Never been married	1,077 (22.8%)	640 (20.9%)
Married	3,265 (69.1%)	2,184 (71.4%)
Separated	37 (0.8%)	23 (0.8%)
Divorced	99 (2.1%)	54 (1.8%)
Widowed	233 (4.9%)	155 (5.1%)
Domestic partner	0 (0%)	0 (0%)
(Missing)	17 (0.4%)	2 (0.1%)
Education (years), n (%)		
Up to 8	2,625 (55.5%)	1,586 (51.9%)
9-15	1,508 (31.9%)	1,016 (33.2%)
16+	596 (12.6%)	456 (14.9%)
(Missing)	0 (<0.0%)	0 (0%)
Employment status, n (%)		
Employed for an employer	1,262 (26.7%)	918 (30.0%)
Self-employed	884 (18.7%)	659 (21.6%)
Retired	236 (5.0%)	143 (4.7%)
Student	366 (7.7%)	114 (3.7%)
Homemaker	1,726 (36.5%)	1,099 (36.0%)
Unemployed and looking for a job	231 (4.9%)	73 (2.4%)
None of these/Other	21 (0.4%)	43 (1.4%)
(Missing)	4 (0.1%)	9 (0.3%)
Current religious service attendance, n (%)		
More than once a week	866 (18.3%)	358 (11.7%)
Once a week	954 (20.2%)	328 (10.7%)

Table S13a. Weighted summary statistics for demographic and childhood variables in Egypt

**Characteristic**	**Wave 1** N = 4,729	**Wave 2** N = 3,057
One to three times a month	378 (8.0%)	232 (7.6%)
A few times a year	458 (9.7%)	326 (10.7%)
Never	2,061 (43.6%)	1,813 (59.3%)
(Missing)	12 (0.3%)	0 (0.0%)
Immigration status, n (%)		
Born in this country	4,712 (99.6%)	3,047 (99.7%)
Born in another country	16 (0.3%)	10 (0.3%)
(Missing)	1 (0.0%)	1 (0.0%)
Parental marital status around age 12, n (%)		
Parents were married	4,050 (85.6%)	2,633 (86.1%)
Parents were divorced	135 (2.9%)	85 (2.8%)
Parents were never married	9 (0.2%)	1 (0.0%)
One or both of them had died	476 (10.1%)	305 (10.0%)
Unsure	33 (0.7%)	22 (0.7%)
(Missing)	25 (0.5%)	10 (0.3%)
Religious service attendance around age 12, n (%)		
At least once a week	2,310 (48.8%)	1,450 (47.4%)
One to three times a month	567 (12.0%)	387 (12.7%)
Less than once a month	629 (13.3%)	409 (13.4%)
Never	1,166 (24.7%)	774 (25.3%)
(Missing)	57 (1.2%)	37 (1.2%)
Relationship with mother when growing up, n (%)		
Very good	4,114 (87.0%)	2,668 (87.3%)
Somewhat good	502 (10.6%)	317 (10.4%)
Somewhat bad	20 (0.4%)	12 (0.4%)
Very bad	10 (0.2%)	4 (0.1%)
(Does not apply)	83 (1.8%)	56 (1.8%)
(Missing)	0 (0%)	0 (0%)
Relationship with father when growing up, n (%)		
Very good	3,714 (78.5%)	2,381 (77.9%)
Somewhat good	687 (14.5%)	445 (14.6%)
Somewhat bad	58 (1.2%)	49 (1.6%)
Very bad	30 (0.6%)	22 (0.7%)
(Does not apply)	226 (4.8%)	151 (5.0%)
(Missing)	13 (0.3%)	8 (0.3%)
Felt like an outsider in family when growing up, n (%)		
Yes	256 (5.4%)	171 (5.6%)
No	4,460 (94.3%)	2,872 (93.9%)
(Missing)	14 (0.3%)	14 (0.5%)
Experienced abuse when growing up, n (%)		
Yes	410 (8.7%)	273 (8.9%)
No	4,289 (90.7%)	2,770 (90.6%)
(Missing)	30 (0.6%)	14 (0.5%)
Self-rated health when growing up, n (%)		
Excellent	2,679 (56.7%)	1,708 (55.9%)
Very good	1,171 (24.8%)	764 (25.0%)
Good	500 (10.6%)	331 (10.8%)
Fair	270 (5.7%)	186 (6.1%)
Poor	107 (2.3%)	69 (2.2%)
(Missing)	1 (0.0%)	1 (0.0%)

Table S13a. Weighted summary statistics for demographic and childhood variables in Egypt

**Characteristic**	**Wave 1**<br>N = 4,729	**Wave 2**<br>N = 3,057
Subjective financial status of family growing up, n (%)		
Lived comfortably	1,250 (26.4%)	809 (26.4%)
Got by	2,348 (49.6%)	1,492 (48.8%)
Found it difficult	862 (18.2%)	581 (19.0%)
Found it very difficult	269 (5.7%)	176 (5.8%)
(Missing)	1 (0.0%)	0 (<0.0%)
Religious affiliation growing up, n (%)		
Christianity	121 (2.6%)	80 (2.6%)
Taoism	0 (<0.0%)	0 (0.0%)
Confucianism	0 (0%)	0 (0%)
Primal, Animist, or Folk religion	0 (0%)	0 (0%)
Spiritism	0 (0%)	0 (0%)
Umbanda, Candomblé, and other African-derived religions	0 (0%)	0 (0%)
Chinese folk/traditional religion	0 (0%)	0 (0%)
Islam	4,605 (97.4%)	2,977 (97.4%)
Hinduism	0 (0%)	0 (0%)
Buddhism	0 (0%)	0 (0%)
Judaism	0 (0%)	0 (0%)
Sikhism	0 (0%)	0 (0%)
Baha'i	0 (0%)	0 (0%)
Jainism	0 (0.0%)	0 (0.0%)
Shinto	0 (0%)	0 (0%)
Some other religion	0 (0%)	0 (0%)
No religion/Atheist/Agnostic	0 (0%)	0 (0%)
(Missing)	3 (0.1%)	0 (0%)

Note. N (%); this table is based on non-imputed data. Cumulative percentages for variables may not add up to 100% due to rounding. Wave 1 characteristics weighted using the Gallup provided sampling weight, ANNUAL_WEIGHT_R2; Wave 2 characteristics weighted accounting for attrition by using the adjusted Wave 1 weight, ANNUAL_WEIGHT_R2, multiplied by the created attrition weight to account for dropout, to maintain nationally representative estimates for Wave 2 characteristics.

Table S13b. Weighted summary statistics for outcome variables in Egypt

Outcome	Wave 1<br>N = 4,729	Wave 2<br>N = 3,057
Secure flourishing index		
Mean	7.3	7.3
Standard Deviation	1.5	1.5
Min, Max	1.3, 10.0	0.7, 10.0
(Missing)	222 (4.7%)	142 (4.6%)
Flourishing index		
Mean	7.6	7.6
Standard Deviation	1.5	1.5
Min, Max	1.6, 10.0	0.4, 10.0
(Missing)	213 (4.5%)	132 (4.3%)
Happiness & life satisfaction		
Mean	6.9	7.0
Standard Deviation	2.5	2.5
Min, Max	0.0, 10.0	0.0, 10.0
(Missing)	49 (1.0%)	11 (0.4%)
Physical & mental health		
Mean	7.7	7.5
Standard Deviation	2.0	2.0
Min, Max	0.0, 10.0	0.0, 10.0
(Missing)	51 (1.1%)	35 (1.1%)
Meaning & purpose		
Mean	8.0	8.0
Standard Deviation	2.0	2.0
Min, Max	0.0, 10.0	0.0, 10.0
(Missing)	50 (1.1%)	28 (0.9%)
Character & virtue		
Mean	7.6	7.7
Standard Deviation	2.0	2.0
Min, Max	0.0, 10.0	0.0, 10.0
(Missing)	108 (2.3%)	57 (1.9%)
Close social relationships		
Mean	7.9	7.7
Standard Deviation	2.3	2.4
Min, Max	0.0, 10.0	0.0, 10.0
(Missing)	23 (0.5%)	16 (0.5%)
Financial & material security		
Mean	5.7	5.8
Standard Deviation	3.1	3.1
Min, Max	0.0, 10.0	0.0, 10.0
(Missing)	21 (0.4%)	11 (0.4%)
Happiness		
Mean	6.2	6.2
Standard Deviation	3.0	2.9
Min, Max	0.0, 10.0	0.0, 10.0
(Missing)	20 (0.4%)	6 (0.2%)
Life satisfaction		
Mean	7.7	7.8
Standard Deviation	2.9	2.8
Min, Max	0.0, 10.0	0.0, 10.0
(Missing)	29 (0.6%)	7 (0.2%)
Current life evaluation		

Table S13b. Weighted summary statistics for outcome variables in Egypt

Outcome	Wave 1<br>N = 4,729	Wave 2<br>N = 3,057
Mean	5.0	5.3
Standard Deviation	2.9	2.9
Min, Max	0.0, 10.0	0.0, 10.0
(Missing)	33 (0.7%)	9 (0.3%)
Future life evaluation		
Mean	7.1	7.2
Standard Deviation	2.8	2.9
Min, Max	0.0, 10.0	0.0, 10.0
(Missing)	826 (17%)	529 (17%)
Optimism		
Mean	7.6	7.5
Standard Deviation	2.6	2.5
Min, Max	0.0, 10.0	0.0, 10.0
(Missing)	68 (1.4%)	56 (1.8%)
Freedom to pursue what's important		
Mean	7.5	7.4
Standard Deviation	2.7	2.7
Min, Max	0.0, 10.0	0.0, 10.0
(Missing)	15 (0.3%)	2 (<0.1%)
Inner peace, n (%)		
Always	1,733 (36.7%)	1,103 (36.1%)
Often	2,311 (48.9%)	1,334 (43.6%)
Rarely	498 (10.5%)	436 (14.3%)
Never	179 (3.8%)	174 (5.7%)
(Missing)	8 (0.2%)	11 (0.4%)
Life balance, n (%)		
Always	1,194 (25.2%)	793 (25.9%)
Often	2,559 (54.1%)	1,471 (48.1%)
Rarely	722 (15.3%)	568 (18.6%)
Never	241 (5.1%)	214 (7.0%)
(Missing)	13 (0.3%)	12 (0.4%)
Sense of mastery, n (%)		
Always	2,242 (47.4%)	1,562 (51.1%)
Often	1,967 (41.6%)	1,145 (37.5%)
Rarely	384 (8.1%)	253 (8.3%)
Never	130 (2.8%)	85 (2.8%)
(Missing)	6 (0.1%)	12 (0.4%)
Meaningful activities		
Mean	8.0	8.0
Standard Deviation	2.4	2.4
Min, Max	0.0, 10.0	0.0, 10.0
(Missing)	21 (0.4%)	15 (0.5%)
Understanding purpose		
Mean	7.9	7.9
Standard Deviation	2.4	2.4
Min, Max	0.0, 10.0	0.0, 10.0
(Missing)	34 (0.7%)	14 (0.5%)
Self-rated mental health		
Mean	8.6	8.4
Standard Deviation	2.1	2.2
Min, Max	0.0, 10.0	0.0, 10.0

Table S13b. Weighted summary statistics for outcome variables in Egypt

Outcome	Wave 1<br>N = 4,729	Wave 2<br>N = 3,057
(Missing)	36 (0.8%)	20 (0.7%)
Traumatic distress, n (%)		
A lot	1,695 (35.8%)	1,086 (35.5%)
Some	810 (17.1%)	464 (15.2%)
Not very much	688 (14.6%)	432 (14.1%)
Not at all	1,523 (32.2%)	1,063 (34.8%)
(Missing)	13 (0.3%)	13 (0.4%)
Depression symptoms composite, n (%)	1,743 (37.1%)	1,111 (36.6%)
(Missing)	36 (0.8%)	26 (0.9%)
Depression – feel hopeless, n (%)		
Nearly every day	691 (14.6%)	499 (16.3%)
More than half the days	625 (13.2%)	385 (12.6%)
Several days	1,698 (35.9%)	1,010 (33.0%)
Not at all	1,707 (36.1%)	1,163 (38.0%)
(Missing)	7 (0.2%)	0 (<0.0%)
Depression – loss of interest, n (%)		
Nearly every day	651 (13.8%)	419 (13.7%)
More than half the days	767 (16.2%)	430 (14.1%)
Several days	1,753 (37.1%)	1,196 (39.1%)
Not at all	1,528 (32.3%)	987 (32.3%)
(Missing)	31 (0.7%)	26 (0.9%)
Anxiety symptoms composite, n (%)	2,084 (44.1%)	1,383 (45.2%)
(Missing)	4 (<0.1%)	1 (<0.1%)
Anxiety – feel on edge, n (%)		
Nearly every day	1,181 (25.0%)	843 (27.6%)
More than half the days	713 (15.1%)	413 (13.5%)
Several days	1,772 (37.5%)	1,138 (37.2%)
Not at all	1,060 (22.4%)	662 (21.7%)
(Missing)	3 (0.1%)	1 (0.0%)
Anxiety – cannot stop worrying, n (%)		
Nearly every day	824 (17.4%)	516 (16.9%)
More than half the days	681 (14.4%)	440 (14.4%)
Several days	1,710 (36.2%)	1,029 (33.6%)
Not at all	1,511 (31.9%)	1,072 (35.1%)
(Missing)	3 (0.1%)	0 (<0.0%)
Suffering, n (%)		
A lot	1,025 (21.7%)	765 (25.0%)
Some	1,329 (28.1%)	685 (22.4%)
Not very much	692 (14.6%)	485 (15.9%)
Not at all	1,678 (35.5%)	1,119 (36.6%)
(Missing)	5 (0.1%)	3 (0.1%)
Relationship contentment		
Mean	7.9	7.8
Standard Deviation	2.7	2.8
Min, Max	0.0, 10.0	0.0, 10.0
(Missing)	10 (0.2%)	6 (0.2%)
Relationship satisfaction		
Mean	7.8	7.6
Standard Deviation	2.6	2.7
Min, Max	0.0, 10.0	0.0, 10.0
(Missing)	16 (0.3%)	11 (0.4%)

Table S13b. Weighted summary statistics for outcome variables in Egypt

Outcome	Wave 1<br>N = 4,729	Wave 2<br>N = 3,057
Social support		
Mean	6.9	6.8
Standard Deviation	3.6	3.7
Min, Max	0.0, 10.0	0.0, 10.0
(Missing)	12 (0.3%)	6 (0.2%)
Intimate/close friend, n (%)		
Yes	4,131 (87.3%)	2,589 (84.7%)
No	585 (12.4%)	466 (15.3%)
(Missing)	13 (0.3%)	2 (0.1%)
Government approval, n (%)		
Strongly approve	0 (0%)	0 (0%)
Somewhat approve	0 (0%)	0 (0%)
Neither approve nor disapprove	0 (0%)	0 (0%)
Somewhat disapprove	0 (0%)	0 (0%)
Strongly disapprove	0 (0%)	0 (0%)
(Missing)	4,729 (100.0%)	3,057 (100.0%)
Say in government, n (%)		
Agree	2,250 (47.6%)	1,533 (50.1%)
Disagree	1,085 (22.9%)	823 (26.9%)
Unsure	1,284 (27.2%)	649 (21.2%)
(Missing)	110 (2.3%)	52 (1.7%)
Belonging in country		
Mean	8.8	8.9
Standard Deviation	2.4	2.2
Min, Max	0.0, 10.0	0.0, 10.0
(Missing)	16 (0.3%)	17 (0.5%)
City/place satisfaction, n (%)		
Satisfied	3,558 (75.2%)	2,428 (79.4%)
Dissatisfied	1,084 (22.9%)	562 (18.4%)
Unsure	84 (1.8%)	63 (2.1%)
(Missing)	2 (0.1%)	4 (0.1%)
Trust within country, n (%)		
All people	292 (6.2%)	142 (4.6%)
Most people	945 (20.0%)	645 (21.1%)
Some people	1,278 (27.0%)	708 (23.2%)
Not very many people	1,374 (29.1%)	951 (31.1%)
None	763 (16.1%)	553 (18.1%)
(Missing)	76 (1.6%)	58 (1.9%)
Number of children		
Mean	1.7	1.8
Standard Deviation	1.5	1.5
Min, Max	0.0, 20.0	0.0, 11.0
(Missing)	2 (<0.1%)	23 (0.8%)
Community participation, n (%)		
More than once a week	213 (4.5%)	115 (3.8%)
Once a week	138 (2.9%)	103 (3.4%)
One to three times a month	170 (3.6%)	82 (2.7%)
A few times a year	297 (6.3%)	155 (5.1%)
Never	3,905 (82.6%)	2,600 (85.0%)
(Missing)	6 (0.1%)	2 (0.1%)
Religious attendance, n (%)		

Table S13b. Weighted summary statistics for outcome variables in Egypt

Outcome	Wave 1<br>N = 4,729	Wave 2<br>N = 3,057
More than once a week	866 (18.3%)	358 (11.7%)
Once a week	954 (20.2%)	328 (10.7%)
One to three times a month	378 (8.0%)	232 (7.6%)
A few times a year	458 (9.7%)	326 (10.7%)
Never	2,061 (43.6%)	1,813 (59.3%)
(Missing)	12 (0.3%)	0 (0.0%)
Loneliness		
Mean	3.9	3.4
Standard Deviation	3.7	3.6
Min, Max	0.0, 10.0	0.0, 10.0
(Missing)	3 (<0.1%)	10 (0.3%)
Perceived discrimination, n (%)		
Always	731 (15.5%)	566 (18.5%)
Often	1,492 (31.6%)	779 (25.5%)
Rarely	784 (16.6%)	508 (16.6%)
Never	1,656 (35.0%)	1,154 (37.7%)
(Missing)	66 (1.4%)	51 (1.7%)
Orientation to promote good		
Mean	8.3	8.5
Standard Deviation	2.1	2.0
Min, Max	0.0, 10.0	0.0, 10.0
(Missing)	63 (1.3%)	28 (0.9%)
Delayed gratification		
Mean	7.0	6.8
Standard Deviation	3.0	3.1
Min, Max	0.0, 10.0	0.0, 10.0
(Missing)	58 (1.2%)	31 (1.0%)
Hope		
Mean	8.2	8.1
Standard Deviation	2.5	2.5
Min, Max	0.0, 10.0	0.0, 10.0
(Missing)	30 (0.6%)	23 (0.7%)
Gratitude		
Mean	7.7	7.6
Standard Deviation	2.6	2.6
Min, Max	0.0, 10.0	0.0, 10.0
(Missing)	49 (1.0%)	38 (1.2%)
Showing love/care		
Mean	8.2	8.3
Standard Deviation	2.7	2.7
Min, Max	0.0, 10.0	0.0, 10.0
(Missing)	12 (0.2%)	6 (0.2%)
Forgivingness, n (%)		
Always	2,723 (57.6%)	1,738 (56.8%)
Often	1,379 (29.2%)	827 (27.1%)
Rarely	429 (9.1%)	302 (9.9%)
Never	196 (4.1%)	183 (6.0%)
(Missing)	3 (0.1%)	7 (0.2%)
Charitable giving, n (%)		
Yes	2,689 (56.9%)	1,715 (56.1%)
No	2,007 (42.4%)	1,266 (41.4%)

Table S13b. Weighted summary statistics for outcome variables in Egypt

Outcome	Wave 1<br>N = 4,729	Wave 2<br>N = 3,057
(Missing)	33 (0.7%)	77 (2.5%)
Helping strangers, n (%)		
Yes	3,371 (71.3%)	2,212 (72.4%)
No	1,317 (27.8%)	776 (25.4%)
(Missing)	41 (0.9%)	69 (2.3%)
Volunteering, n (%)		
Yes	209 (4.4%)	260 (8.5%)
No	4,514 (95.4%)	2,782 (91.0%)
(Missing)	6 (0.1%)	16 (0.5%)
Self-rated physical health		
Mean	6.8	6.6
Standard Deviation	2.8	2.8
Min, Max	0.0, 10.0	0.0, 10.0
(Missing)	15 (0.3%)	17 (0.6%)
Health problems, n (%)		
Yes	1,164 (24.6%)	727 (23.8%)
No	3,551 (75.1%)	2,323 (76.0%)
(Missing)	14 (0.3%)	7 (0.2%)
Pain in past 4 weeks, n (%)		
A lot	1,703 (36.0%)	1,169 (38.2%)
Some	1,143 (24.2%)	698 (22.8%)
Not very much	769 (16.3%)	442 (14.5%)
None at all	1,109 (23.5%)	747 (24.4%)
(Missing)	5 (0.1%)	2 (0.1%)
Number of cigarettes per day		
Mean	3.4	4.2
Standard Deviation	7.7	9.3
Min, Max	0.0, 97.0	0.0, 97.0
(Missing)	33 (0.7%)	6 (0.2%)
Number of drinks per week		
Mean	0.0	0.0
Standard Deviation	0.2	0.3
Min, Max	0.0, 7.0	0.0, 20.0
(Missing)	35 (0.7%)	4 (0.1%)
Days exercise per week		
Mean	0.7	0.6
Standard Deviation	1.7	1.5
Min, Max	0.0, 7.0	0.0, 7.0
(Missing)	11 (0.2%)	6 (0.2%)
Financial security		
Mean	5.3	5.4
Standard Deviation	3.5	3.5
Min, Max	0.0, 10.0	0.0, 10.0
(Missing)	12 (0.3%)	9 (0.3%)
Material security		
Mean	6.2	6.3
Standard Deviation	3.4	3.4
Min, Max	0.0, 10.0	0.0, 10.0
(Missing)	12 (0.3%)	3 (0.1%)
Educational attainment (16+ years), n (%)		
Up to 8	2,625 (55.5%)	1,586 (51.9%)

Table S13b. Weighted summary statistics for outcome variables in Egypt

Outcome	Wave 1<br>N = 4,729	Wave 2<br>N = 3,057
9-15	1,508 (31.9%)	1,016 (33.2%)
16+	596 (12.6%)	456 (14.9%)
(Missing)	0 (<0.0%)	0 (0%)
Currently employed, n (%)		
Employed for an employer	1,262 (26.7%)	918 (30.0%)
Self-employed	884 (18.7%)	659 (21.6%)
Retired	236 (5.0%)	143 (4.7%)
Student	366 (7.7%)	114 (3.7%)
Homemaker	1,726 (36.5%)	1,099 (36.0%)
Unemployed and looking for a job	231 (4.9%)	73 (2.4%)
None of these/Other	21 (0.4%)	43 (1.4%)
(Missing)	4 (0.1%)	9 (0.3%)
Financially comfortable/getting by, n (%)		
Living comfortably on present income	328 (6.9%)	246 (8.0%)
Getting by on present income	2,355 (49.8%)	1,580 (51.7%)
Finding it difficult on present income	1,028 (21.7%)	645 (21.1%)
Finding it very difficult on present income	954 (20.2%)	574 (18.8%)
(Missing)	63 (1.3%)	13 (0.4%)
Own home, n (%)		
Someone in this household owns this home	3,418 (72.3%)	2,154 (70.5%)
Someone in this household rents this home	1,051 (22.2%)	685 (22.4%)
Both	20 (0.4%)	12 (0.4%)
Neither	218 (4.6%)	202 (6.6%)
Rent	0 (0%)	0 (0%)
Own	0 (0%)	0 (0%)
Something else	0 (0%)	0 (0%)
(Missing)	22 (0.5%)	4 (0.1%)
Religious/spiritual connection, n (%)		
Always	3,560 (75.3%)	2,274 (74.4%)
Often	723 (15.3%)	477 (15.6%)
Rarely	219 (4.6%)	149 (4.9%)
Never	219 (4.6%)	155 (5.1%)
(Missing)	8 (0.2%)	2 (0.1%)
Belief in life after death, n (%)		
Yes	2,993 (63.3%)	1,969 (64.4%)
No	1,080 (22.8%)	718 (23.5%)
Unsure	633 (13.4%)	359 (11.7%)
(Missing)	23 (0.5%)	12 (0.4%)
Transformative religious experience, n (%)		
Yes	1,653 (35.0%)	1,105 (36.2%)
No	3,037 (64.2%)	1,932 (63.2%)
(Missing)	40 (0.8%)	20 (0.7%)
Religious reading or listening, n (%)		
More than once a day	1,575 (33.3%)	1,082 (35.4%)
About once a day	1,152 (24.4%)	654 (21.4%)
Sometimes	1,604 (33.9%)	1,094 (35.8%)
Never	388 (8.2%)	223 (7.3%)
(Missing)	10 (0.2%)	5 (0.2%)
Prayer or meditation, n (%)		
More than once a day	3,503 (74.1%)	2,322 (76.0%)
About once a day	207 (4.4%)	90 (2.9%)

Table S13b. Weighted summary statistics for outcome variables in Egypt

Outcome	Wave 1<br>N = 4,729	Wave 2<br>N = 3,057
Sometimes	929 (19.6%)	601 (19.6%)
Never	79 (1.7%)	44 (1.5%)
(Missing)	11 (0.2%)	1 (0.0%)
Belief in God/gods/spiritual forces, n (%)		
One God	4,719 (99.8%)	3,057 (100.0%)
More than one god	0 (0%)	0 (0%)
An impersonal spiritual force	0 (0%)	0 (0%)
None of these	3 (0.1%)	0 (0%)
Unsure	0 (0%)	0 (0%)
(Missing)	8 (0.2%)	1 (0.0%)
Religious centrality, n (%)		
Agree	4,230 (89.4%)	2,762 (90.3%)
Disagree	232 (4.9%)	191 (6.2%)
Not relevant	50 (1.1%)	5 (0.2%)
Unsure	200 (4.2%)	98 (3.2%)
(Missing)	18 (0.4%)	0 (<0.0%)
Religious/spiritual comfort, n (%)		
Agree	4,592 (97.1%)	3,013 (98.6%)
Disagree	56 (1.2%)	27 (0.9%)
Not relevant	17 (0.4%)	1 (0.0%)
Unsure	46 (1.0%)	16 (0.5%)
(Missing)	18 (0.4%)	0 (0%)
Feel loved by God, n (%)		
Agree	4,631 (97.9%)	3,020 (98.8%)
Disagree	17 (0.4%)	7 (0.2%)
Not relevant	3 (0.1%)	1 (0.0%)
Unsure	67 (1.4%)	29 (1.0%)
(Missing)	12 (0.3%)	0 (0.0%)
Feel punished by God, n (%)		
Agree	4,126 (87.2%)	2,734 (89.4%)
Disagree	371 (7.8%)	241 (7.9%)
Not relevant	56 (1.2%)	8 (0.3%)
Unsure	149 (3.2%)	73 (2.4%)
(Missing)	27 (0.6%)	2 (0.1%)
Experienced religious criticism, n (%)		
Agree	1,360 (28.8%)	917 (30.0%)
Disagree	2,625 (55.5%)	1,876 (61.4%)
Not relevant	351 (7.4%)	98 (3.2%)
Unsure	376 (7.9%)	163 (5.3%)
(Missing)	17 (0.4%)	4 (0.1%)
Faith-sharing, n (%)		
Agree	1,968 (41.6%)	1,280 (41.9%)
Disagree	2,259 (47.8%)	1,602 (52.4%)
Not relevant	347 (7.3%)	115 (3.8%)
Unsure	135 (2.8%)	60 (2.0%)
(Missing)	21 (0.4%)	1 (0.0%)

Note. N (%); this table is based on non-imputed data. Cumulative percentages for variables may not add up to 100% due to rounding. Wave 1 characteristics weighted using the Gallup provided sampling weight, ANNUAL_WEIGHT_R2; Wave 2 characteristics weighted accounting for attrition by using the adjusted Wave 1 weight, ANNUAL_WEIGHT_R2, multiplied by the created attrition weight to account for dropout, to maintain nationally representative estimates for Wave 2 characteristics.

Table S13c. Unweighted summary statistics for demographic and childhood variables in Egypt by retention status

**Characteristic**	**Attritors–Not Observed in Wave 2** N = 1,716	**Retained–Observed in Wave 2** N = 3,027
Forgivingness, n (%)		
Always	997 (58.1%)	1,734 (57.3%)
Often	482 (28.1%)	902 (29.8%)
Rarely	163 (9.5%)	267 (8.8%)
Never	74 (4.3%)	122 (4.0%)
(Missing)	0 (0%)	3 (0.1%)
Year of birth, n (%)		
1943 or earlier (current age: 80+ years)	7 (0.4%)	0 (0%)
1943-1953 (current age: 70-79 years)	21 (1.2%)	31 (1.0%)
1953-1963 (current age: 60-69 years)	143 (8.3%)	247 (8.1%)
1963-1973 (current age: 50-59 years)	186 (10.8%)	385 (12.7%)
1973-1983 (current age: 40-49 years)	291 (16.9%)	526 (17.4%)
1983-1993 (current age: 30-39 years)	418 (24.3%)	725 (23.9%)
1993-1998 (current age: 25-29 years)	224 (13.1%)	380 (12.6%)
1998-2005 (current age: 18-24 years)	426 (24.8%)	734 (24.3%)
(Missing)	0 (0%)	0 (0%)
Age of participant		
Mean	36.8	37.1
Standard Deviation	14.4	14.1
Min, Max	18.0, 85.0	18.0, 75.0
Gender, n (%)		
Male	836 (48.7%)	1,599 (52.8%)
Female	880 (51.3%)	1,428 (47.2%)
Other	0 (0%)	0 (0%)
(Missing)	0 (0%)	0 (0.0%)
Respondent marital status, n (%)		
Single/Never been married	408 (23.8%)	672 (22.2%)
Married	1,163 (67.7%)	2,113 (69.8%)
Separated	16 (0.9%)	21 (0.7%)
Divorced	40 (2.3%)	60 (2.0%)
Widowed	80 (4.7%)	154 (5.1%)
Domestic partner	0 (0%)	0 (0%)
(Missing)	10 (0.6%)	7 (0.2%)
Education (years), n (%)		
Up to 8	971 (56.6%)	1,662 (54.9%)
9-15	521 (30.4%)	992 (32.8%)
16+	224 (13.0%)	374 (12.3%)
(Missing)	0 (0%)	0 (0.0%)
Employment status, n (%)		
Employed for an employer	434 (25.3%)	832 (27.5%)
Self-employed	341 (19.9%)	545 (18.0%)
Retired	81 (4.7%)	156 (5.1%)
Student	119 (6.9%)	248 (8.2%)
Homemaker	637 (37.1%)	1,094 (36.1%)
Unemployed and looking for a job	97 (5.6%)	135 (4.5%)
None of these/Other	5 (0.3%)	16 (0.5%)
(Missing)	3 (0.2%)	1 (0.0%)
Current religious service attendance, n (%)		
More than once a week	330 (19.2%)	538 (17.8%)

Table S13c. Unweighted summary statistics for demographic and childhood variables in Egypt by retention status

**Characteristic**	**Attritors–Not Observed in Wave 2**<br>N = 1,716	**Retained–Observed in Wave 2**<br>N = 3,027
Once a week	347 (20.2%)	610 (20.2%)
One to three times a month	143 (8.3%)	236 (7.8%)
A few times a year	115 (6.7%)	346 (11.4%)
Never	778 (45.3%)	1,289 (42.6%)
(Missing)	3 (0.2%)	9 (0.3%)
Immigration status, n (%)		
Born in this country	1,706 (99.4%)	3,020 (99.8%)
Born in another country	10 (0.6%)	6 (0.2%)
(Missing)	0 (0.0%)	1 (0.0%)
Parental marital status around age 12, n (%)		
Parents were married	1,454 (84.7%)	2,608 (86.2%)
Parents were divorced	52 (3.0%)	84 (2.8%)
Parents were never married	8 (0.5%)	1 (0.0%)
One or both of them had died	178 (10.3%)	300 (9.9%)
Unsure	10 (0.6%)	23 (0.8%)
(Missing)	15 (0.9%)	10 (0.3%)
Religious service attendance around age 12, n (%)		
At least once a week	865 (50.4%)	1,451 (47.9%)
One to three times a month	182 (10.6%)	387 (12.8%)
Less than once a month	231 (13.5%)	400 (13.2%)
Never	417 (24.3%)	753 (24.9%)
(Missing)	21 (1.2%)	36 (1.2%)
Relationship with mother when growing up, n (%)		
Very good	1,486 (86.6%)	2,642 (87.3%)
Somewhat good	189 (11.0%)	314 (10.4%)
Somewhat bad	8 (0.5%)	12 (0.4%)
Very bad	6 (0.4%)	4 (0.1%)
(Does not apply)	28 (1.6%)	56 (1.9%)
(Missing)	0 (0%)	0 (0%)
Relationship with father when growing up, n (%)		
Very good	1,362 (79.4%)	2,363 (78.1%)
Somewhat good	250 (14.6%)	439 (14.5%)
Somewhat bad	11 (0.6%)	48 (1.6%)
Very bad	9 (0.5%)	21 (0.7%)
(Does not apply)	80 (4.6%)	147 (4.9%)
(Missing)	4 (0.3%)	9 (0.3%)
Felt like an outsider in family when growing up, n (%)		
Yes	86 (5.0%)	170 (5.6%)
No	1,629 (94.9%)	2,844 (94.0%)
(Missing)	1 (0.1%)	13 (0.4%)
Experienced abuse when growing up, n (%)		
Yes	143 (8.3%)	268 (8.9%)
No	1,557 (90.7%)	2,745 (90.7%)
(Missing)	16 (0.9%)	14 (0.5%)
Self-rated health when growing up, n (%)		
Excellent	1,003 (58.4%)	1,684 (55.6%)
Very good	413 (24.1%)	762 (25.2%)

Table S13c. Unweighted summary statistics for demographic and childhood variables in Egypt by retention status

**Characteristic**	**Attritors–Not Observed in Wave 2**<br>N = 1,716	**Retained–Observed in Wave 2**<br>N = 3,027
Good	173 (10.1%)	329 (10.9%)
Fair	89 (5.2%)	183 (6.0%)
Poor	39 (2.3%)	69 (2.3%)
(Missing)	0 (0%)	1 (0.0%)
Subjective financial status of family growing up, n (%)		
Lived comfortably	448 (26.1%)	805 (26.6%)
Got by	875 (51.0%)	1,479 (48.9%)
Found it difficult	293 (17.1%)	572 (18.9%)
Found it very difficult	99 (5.8%)	170 (5.6%)
(Missing)	0 (0.0%)	0 (<0.0%)
Religious affiliation growing up, n (%)		
Christianity	42 (2.4%)	79 (2.6%)
Taoism	0 (0%)	0 (0.0%)
Confucianism	0 (0%)	0 (0%)
Primal, Animist, or Folk religion	0 (0%)	0 (0%)
Spiritism	0 (0%)	0 (0%)
Umbanda, Candomblé, and other African-derived religions	0 (0%)	0 (0%)
Chinese folk/traditional religion	0 (0%)	0 (0%)
Islam	1,672 (97.4%)	2,947 (97.3%)
Hinduism	0 (0%)	0 (0%)
Buddhism	0 (0%)	0 (0%)
Judaism	0 (0%)	0 (0%)
Sikhism	0 (0%)	0 (0%)
Baha'i	0 (0%)	0 (0%)
Jainism	0 (0%)	1 (0.0%)
Shinto	0 (0%)	0 (0%)
Some other religion	0 (0%)	0 (0%)
No religion/Atheist/Agnostic	0 (0%)	0 (0%)
(Missing)	3 (0.2%)	0 (0%)

Note. N (%); this table is based on non-imputed data. Cumulative percentages for variables may not add up to 100% due to rounding.

Table S13d. Unweighted summary statistics for Wave 1 outcome variables in Egypt by retention status.

Outcome	Attritors–Not Observed in Wave 2<br>N = 1,716	Retained–Observed in Wave 2<br>N = 3,027
Secure flourishing index		
Mean	7.3	7.3
Standard Deviation	1.5	1.5
Min, Max	1.6, 10.0	1.3, 10.0
(Missing)	84 (4.9%)	139 (4.6%)
Flourishing index		
Mean	7.6	7.6
Standard Deviation	1.5	1.5
Min, Max	1.7, 10.0	1.6, 10.0
(Missing)	83 (4.8%)	130 (4.3%)
Happiness & life satisfaction		
Mean	6.9	6.9
Standard Deviation	2.5	2.5
Min, Max	0.0, 10.0	0.0, 10.0
(Missing)	22 (1.3%)	27 (0.9%)
Physical & mental health		
Mean	7.8	7.7
Standard Deviation	2.0	2.0
Min, Max	0.0, 10.0	0.0, 10.0
(Missing)	15 (0.9%)	37 (1.2%)
Meaning & purpose		
Mean	8.0	8.0
Standard Deviation	2.0	2.0
Min, Max	0.0, 10.0	0.0, 10.0
(Missing)	21 (1.2%)	29 (1.0%)
Character & virtue		
Mean	7.7	7.6
Standard Deviation	2.0	2.0
Min, Max	0.0, 10.0	0.0, 10.0
(Missing)	45 (2.6%)	63 (2.1%)
Close social relationships		
Mean	7.8	7.9
Standard Deviation	2.4	2.3
Min, Max	0.0, 10.0	0.0, 10.0
(Missing)	9 (0.5%)	14 (0.4%)
Financial & material security		
Mean	5.7	5.7
Standard Deviation	3.1	3.1
Min, Max	0.0, 10.0	0.0, 10.0
(Missing)	11 (0.7%)	10 (0.3%)
Happiness		
Mean	6.2	6.2
Standard Deviation	3.0	2.9
Min, Max	0.0, 10.0	0.0, 10.0
(Missing)	11 (0.7%)	9 (0.3%)
Life satisfaction		
Mean	7.7	7.7
Standard Deviation	2.8	2.9
Min, Max	0.0, 10.0	0.0, 10.0

Table S13d. Unweighted summary statistics for Wave 1 outcome variables in Egypt by retention status.

**Outcome**	**Attritors–Not Observed in Wave 2** N = 1,716	**Retained–Observed in Wave 2** N = 3,027
(Missing)	10 (0.6%)	19 (0.6%)
Current life evaluation		
Mean	5.2	5.0
Standard Deviation	2.9	2.9
Min, Max	0.0, 10.0	0.0, 10.0
(Missing)	13 (0.8%)	20 (0.6%)
Future life evaluation		
Mean	7.0	7.2
Standard Deviation	2.8	2.8
Min, Max	0.0, 10.0	0.0, 10.0
(Missing)	316 (18%)	512 (17%)
Optimism		
Mean	7.6	7.6
Standard Deviation	2.6	2.6
Min, Max	0.0, 10.0	0.0, 10.0
(Missing)	25 (1.5%)	44 (1.4%)
Freedom to pursue what's important		
Mean	7.7	7.5
Standard Deviation	2.7	2.8
Min, Max	0.0, 10.0	0.0, 10.0
(Missing)	7 (0.4%)	9 (0.3%)
Inner peace, n (%)		
Always	643 (37.5%)	1,095 (36.2%)
Often	820 (47.8%)	1,498 (49.5%)
Rarely	186 (10.8%)	314 (10.4%)
Never	65 (3.8%)	115 (3.8%)
(Missing)	2 (0.1%)	6 (0.2%)
Life balance, n (%)		
Always	461 (26.9%)	735 (24.3%)
Often	887 (51.7%)	1,681 (55.5%)
Rarely	278 (16.2%)	446 (14.7%)
Never	87 (5.1%)	155 (5.1%)
(Missing)	3 (0.2%)	11 (0.3%)
Sense of mastery, n (%)		
Always	818 (47.7%)	1,431 (47.3%)
Often	693 (40.4%)	1,280 (42.3%)
Rarely	144 (8.4%)	240 (7.9%)
Never	58 (3.4%)	73 (2.4%)
(Missing)	3 (0.2%)	3 (0.1%)
Meaningful activities		
Mean	8.0	8.0
Standard Deviation	2.4	2.4
Min, Max	0.0, 10.0	0.0, 10.0
(Missing)	6 (0.3%)	15 (0.5%)
Understanding purpose		
Mean	8.0	7.9
Standard Deviation	2.4	2.4
Min, Max	0.0, 10.0	0.0, 10.0
(Missing)	18 (1.1%)	16 (0.5%)

Table S13d. Unweighted summary statistics for Wave 1 outcome variables in Egypt by retention status.

**Outcome**	**Attritors–Not Observed in Wave 2** N = 1,716	**Retained–Observed in Wave 2** N = 3,027
Self-rated mental health		
Mean	8.6	8.6
Standard Deviation	2.1	2.1
Min, Max	0.0, 10.0	0.0, 10.0
(Missing)	11 (0.6%)	26 (0.8%)
Traumatic distress, n (%)		
A lot	586 (34.1%)	1,115 (36.8%)
Some	278 (16.2%)	535 (17.7%)
Not very much	250 (14.5%)	441 (14.6%)
Not at all	596 (34.7%)	930 (30.7%)
(Missing)	6 (0.4%)	6 (0.2%)
Depression symptoms composite, n (%)	644 (37.8%)	1,104 (36.8%)
(Missing)	11 (0.6%)	25 (0.8%)
Depression – feel hopeless, n (%)		
Nearly every day	241 (14.0%)	453 (15.0%)
More than half the days	233 (13.6%)	394 (13.0%)
Several days	614 (35.8%)	1,089 (36.0%)
Not at all	626 (36.5%)	1,086 (35.9%)
(Missing)	2 (0.1%)	5 (0.2%)
Depression – loss of interest, n (%)		
Nearly every day	230 (13.4%)	423 (14.0%)
More than half the days	267 (15.6%)	502 (16.6%)
Several days	638 (37.2%)	1,119 (37.0%)
Not at all	572 (33.3%)	960 (31.7%)
(Missing)	9 (0.5%)	22 (0.7%)
Anxiety symptoms composite, n (%)	755 (44.0%)	1,335 (44.1%)
(Missing)	3 (0.2%)	2 (<0.1%)
Anxiety – feel on edge, n (%)		
Nearly every day	423 (24.6%)	762 (25.2%)
More than half the days	258 (15.1%)	457 (15.1%)
Several days	617 (36.0%)	1,161 (38.3%)
Not at all	416 (24.3%)	646 (21.4%)
(Missing)	2 (0.1%)	1 (0.0%)
Anxiety – cannot stop worrying, n (%)		
Nearly every day	289 (16.8%)	538 (17.8%)
More than half the days	262 (15.3%)	420 (13.9%)
Several days	599 (34.9%)	1,117 (36.9%)
Not at all	563 (32.8%)	951 (31.4%)
(Missing)	2 (0.1%)	1 (0.0%)
Suffering, n (%)		
A lot	361 (21.0%)	667 (22.0%)
Some	481 (28.0%)	852 (28.2%)
Not very much	231 (13.4%)	464 (15.3%)
Not at all	644 (37.5%)	1,039 (34.3%)
(Missing)	0 (0.0%)	5 (0.2%)
Relationship contentment		
Mean	7.9	8.0
Standard Deviation	2.8	2.7
Min, Max	0.0, 10.0	0.0, 10.0

Table S13d. Unweighted summary statistics for Wave 1 outcome variables in Egypt by retention status.

**Outcome**	**Attritors–Not Observed in Wave 2** N = 1,716	**Retained–Observed in Wave 2** N = 3,027
(Missing)	4 (0.2%)	7 (0.2%)
Relationship satisfaction		
Mean	7.8	7.8
Standard Deviation	2.6	2.6
Min, Max	0.0, 10.0	0.0, 10.0
(Missing)	9 (0.5%)	7 (0.2%)
Social support		
Mean	6.9	6.9
Standard Deviation	3.6	3.6
Min, Max	0.0, 10.0	0.0, 10.0
(Missing)	5 (0.3%)	7 (0.2%)
Intimate/close friend, n (%)		
Yes	1,502 (87.5%)	2,641 (87.3%)
No	212 (12.3%)	375 (12.4%)
(Missing)	2 (0.1%)	11 (0.4%)
Government approval, n (%)		
Strongly approve	0 (0%)	0 (0%)
Somewhat approve	0 (0%)	0 (0%)
Neither approve nor disapprove	0 (0%)	0 (0%)
Somewhat disapprove	0 (0%)	0 (0%)
Strongly disapprove	0 (0%)	0 (0%)
(Missing)	1,716 (100.0%)	3,027 (100.0%)
Say in government, n (%)		
Agree	850 (49.5%)	1,405 (46.4%)
Disagree	374 (21.8%)	715 (23.6%)
Unsure	443 (25.8%)	846 (28.0%)
(Missing)	49 (2.8%)	61 (2.0%)
Belonging in country		
Mean	8.9	8.7
Standard Deviation	2.2	2.4
Min, Max	0.0, 10.0	0.0, 10.0
(Missing)	10 (0.6%)	6 (0.2%)
City/place satisfaction, n (%)		
Satisfied	1,317 (76.7%)	2,252 (74.4%)
Dissatisfied	365 (21.3%)	723 (23.9%)
Unsure	35 (2.0%)	50 (1.6%)
(Missing)	0 (0%)	2 (0.1%)
Trust within country, n (%)		
All people	112 (6.5%)	181 (6.0%)
Most people	320 (18.6%)	629 (20.8%)
Some people	482 (28.1%)	799 (26.4%)
Not very many people	467 (27.2%)	912 (30.1%)
None	301 (17.5%)	463 (15.3%)
(Missing)	34 (2.0%)	43 (1.4%)
Number of children		
Mean	1.7	1.7
Standard Deviation	1.7	1.4
Min, Max	0.0, 20.0	0.0, 8.0
(Missing)	0 (0%)	2 (<0.1%)

Table S13d. Unweighted summary statistics for Wave 1 outcome variables in Egypt by retention status.

Outcome	Attritors–Not Observed in Wave 2<br>N = 1,716	Retained–Observed in Wave 2<br>N = 3,027
Community participation, n (%)		
More than once a week	62 (3.6%)	152 (5.0%)
Once a week	39 (2.3%)	99 (3.3%)
One to three times a month	68 (3.9%)	102 (3.4%)
A few times a year	88 (5.1%)	211 (7.0%)
Never	1,454 (84.7%)	2,462 (81.3%)
(Missing)	6 (0.3%)	1 (0.0%)
Religious attendance, n (%)		
More than once a week	330 (19.2%)	538 (17.8%)
Once a week	347 (20.2%)	610 (20.2%)
One to three times a month	143 (8.3%)	236 (7.8%)
A few times a year	115 (6.7%)	346 (11.4%)
Never	778 (45.3%)	1,289 (42.6%)
(Missing)	3 (0.2%)	9 (0.3%)
Loneliness		
Mean	3.9	3.8
Standard Deviation	3.7	3.7
Min, Max	0.0, 10.0	0.0, 10.0
(Missing)	3 (0.2%)	1 (<0.1%)
Perceived discrimination, n (%)		
Always	258 (15.0%)	476 (15.7%)
Often	538 (31.4%)	958 (31.7%)
Rarely	279 (16.3%)	507 (16.7%)
Never	609 (35.5%)	1,053 (34.8%)
(Missing)	32 (1.9%)	33 (1.1%)
Orientation to promote good		
Mean	8.3	8.3
Standard Deviation	2.1	2.1
Min, Max	0.0, 10.0	0.0, 10.0
(Missing)	29 (1.7%)	34 (1.1%)
Delayed gratification		
Mean	7.0	6.9
Standard Deviation	3.0	3.0
Min, Max	0.0, 10.0	0.0, 10.0
(Missing)	24 (1.4%)	34 (1.1%)
Hope		
Mean	8.2	8.2
Standard Deviation	2.5	2.4
Min, Max	0.0, 10.0	0.0, 10.0
(Missing)	14 (0.8%)	16 (0.5%)
Gratitude		
Mean	7.8	7.7
Standard Deviation	2.5	2.6
Min, Max	0.0, 10.0	0.0, 10.0
(Missing)	19 (1.1%)	30 (1.0%)
Showing love/care		
Mean	8.2	8.2
Standard Deviation	2.6	2.8
Min, Max	0.0, 10.0	0.0, 10.0

Table S13d. Unweighted summary statistics for Wave 1 outcome variables in Egypt by retention status.

**Outcome**	**Attritors–Not Observed in Wave 2**<br>N = 1,716	**Retained–Observed in Wave 2**<br>N = 3,027
(Missing)	4 (0.2%)	8 (0.3%)
Forgivingness, n (%)		
Always	997 (58.1%)	1,734 (57.3%)
Often	482 (28.1%)	902 (29.8%)
Rarely	163 (9.5%)	267 (8.8%)
Never	74 (4.3%)	122 (4.0%)
(Missing)	0 (0%)	3 (0.1%)
Charitable giving, n (%)		
Yes	967 (56.3%)	1,730 (57.2%)
No	735 (42.8%)	1,278 (42.2%)
(Missing)	14 (0.8%)	19 (0.6%)
Helping strangers, n (%)		
Yes	1,196 (69.7%)	2,187 (72.2%)
No	512 (29.8%)	808 (26.7%)
(Missing)	9 (0.5%)	33 (1.1%)
Volunteering, n (%)		
Yes	74 (4.3%)	135 (4.5%)
No	1,640 (95.5%)	2,888 (95.4%)
(Missing)	2 (0.1%)	4 (0.1%)
Self-rated physical health		
Mean	6.9	6.8
Standard Deviation	2.8	2.8
Min, Max	0.0, 10.0	0.0, 10.0
(Missing)	4 (0.2%)	11 (0.4%)
Health problems, n (%)		
Yes	413 (24.1%)	755 (24.9%)
No	1,295 (75.4%)	2,267 (74.9%)
(Missing)	9 (0.5%)	6 (0.2%)
Pain in past 4 weeks, n (%)		
A lot	609 (35.5%)	1,099 (36.3%)
Some	414 (24.1%)	732 (24.2%)
Not very much	284 (16.5%)	487 (16.1%)
None at all	407 (23.7%)	706 (23.3%)
(Missing)	2 (0.1%)	3 (0.1%)
Number of cigarettes per day		
Mean	3.0	3.7
Standard Deviation	7.3	8.0
Min, Max	0.0, 97.0	0.0, 60.0
(Missing)	14 (0.8%)	19 (0.6%)
Number of drinks per week		
Mean	0.0	0.0
Standard Deviation	0.1	0.3
Min, Max	0.0, 2.0	0.0, 7.0
(Missing)	12 (0.7%)	24 (0.8%)
Days exercise per week		
Mean	0.7	0.7
Standard Deviation	1.7	1.7
Min, Max	0.0, 7.0	0.0, 7.0
(Missing)	6 (0.4%)	5 (0.2%)

Table S13d. Unweighted summary statistics for Wave 1 outcome variables in Egypt by retention status.

**Outcome**	**Attritors–Not Observed in Wave 2** N = 1,716	**Retained–Observed in Wave 2** N = 3,027
Financial security		
Mean	5.3	5.2
Standard Deviation	3.5	3.4
Min, Max	0.0, 10.0	0.0, 10.0
(Missing)	6 (0.4%)	6 (0.2%)
Material security		
Mean	6.2	6.2
Standard Deviation	3.5	3.4
Min, Max	0.0, 10.0	0.0, 10.0
(Missing)	8 (0.5%)	4 (0.1%)
Educational attainment (16+ years), n (%)		
Up to 8	971 (56.6%)	1,662 (54.9%)
9-15	521 (30.4%)	992 (32.8%)
16+	224 (13.0%)	374 (12.3%)
(Missing)	0 (0%)	0 (0.0%)
Currently employed, n (%)		
Employed for an employer	434 (25.3%)	832 (27.5%)
Self-employed	341 (19.9%)	545 (18.0%)
Retired	81 (4.7%)	156 (5.1%)
Student	119 (6.9%)	248 (8.2%)
Homemaker	637 (37.1%)	1,094 (36.1%)
Unemployed and looking for a job	97 (5.6%)	135 (4.5%)
None of these/Other	5 (0.3%)	16 (0.5%)
(Missing)	3 (0.2%)	1 (0.0%)
Financially comfortable/getting by, n (%)		
Living comfortably on present income	136 (7.9%)	193 (6.4%)
Getting by on present income	884 (51.5%)	1,477 (48.8%)
Finding it difficult on present income	360 (21.0%)	671 (22.2%)
Finding it very difficult on present income	313 (18.3%)	644 (21.3%)
(Missing)	22 (1.3%)	41 (1.4%)
Own home, n (%)		
Someone in this household owns this home	1,208 (70.4%)	2,222 (73.4%)
Someone in this household rents this home	422 (24.6%)	632 (20.9%)
Both	7 (0.4%)	13 (0.4%)
Neither	68 (4.0%)	151 (5.0%)
Rent	0 (0%)	0 (0%)
Own	0 (0%)	0 (0%)
Something else	0 (0%)	0 (0%)
(Missing)	12 (0.7%)	9 (0.3%)
Religious/spiritual connection, n (%)		
Always	1,294 (75.4%)	2,277 (75.2%)
Often	275 (16.0%)	450 (14.8%)
Rarely	61 (3.6%)	159 (5.2%)
Never	82 (4.8%)	138 (4.6%)
(Missing)	4 (0.2%)	4 (0.1%)
Belief in life after death, n (%)		
Yes	1,087 (63.3%)	1,915 (63.3%)
No	401 (23.4%)	682 (22.5%)
Unsure	217 (12.6%)	419 (13.8%)

Table S13d. Unweighted summary statistics for Wave 1 outcome variables in Egypt by retention status.

Outcome	Attritors–Not Observed in Wave 2 N = 1,716	Retained–Observed in Wave 2 N = 3,027
(Missing)	11 (0.7%)	11 (0.4%)
Transformative religious experience, n (%)		
Yes	590 (34.4%)	1,068 (35.3%)
No	1,112 (64.8%)	1,934 (63.9%)
(Missing)	14 (0.8%)	26 (0.8%)
Religious reading or listening, n (%)		
More than once a day	595 (34.7%)	984 (32.5%)
About once a day	436 (25.4%)	719 (23.8%)
Sometimes	540 (31.5%)	1,069 (35.3%)
Never	142 (8.3%)	248 (8.2%)
(Missing)	3 (0.2%)	7 (0.2%)
Prayer or meditation, n (%)		
More than once a day	1,239 (72.2%)	2,275 (75.2%)
About once a day	96 (5.6%)	111 (3.7%)
Sometimes	333 (19.4%)	599 (19.8%)
Never	39 (2.3%)	41 (1.3%)
(Missing)	9 (0.5%)	1 (0.0%)
Belief in God/gods/spiritual forces, n (%)		
One God	1,709 (99.6%)	3,024 (99.9%)
More than one god	0 (0%)	0 (0%)
An impersonal spiritual force	0 (0%)	0 (0%)
None of these	0 (0%)	3 (0.1%)
Unsure	0 (0%)	0 (0%)
(Missing)	7 (0.4%)	0 (0.0%)
Religious centrality, n (%)		
Agree	1,538 (89.6%)	2,704 (89.3%)
Disagree	92 (5.4%)	140 (4.6%)
Not relevant	15 (0.9%)	35 (1.2%)
Unsure	63 (3.7%)	137 (4.5%)
(Missing)	8 (0.5%)	10 (0.3%)
Religious/spiritual comfort, n (%)		
Agree	1,663 (96.9%)	2,943 (97.2%)
Disagree	21 (1.2%)	35 (1.2%)
Not relevant	5 (0.3%)	12 (0.4%)
Unsure	16 (0.9%)	30 (1.0%)
(Missing)	11 (0.6%)	7 (0.2%)
Feel loved by God, n (%)		
Agree	1,670 (97.3%)	2,975 (98.3%)
Disagree	9 (0.5%)	8 (0.3%)
Not relevant	1 (0.0%)	2 (0.1%)
Unsure	29 (1.7%)	38 (1.3%)
(Missing)	8 (0.5%)	4 (0.1%)
Feel punished by God, n (%)		
Agree	1,491 (86.9%)	2,648 (87.5%)
Disagree	129 (7.5%)	244 (8.1%)
Not relevant	20 (1.2%)	36 (1.2%)
Unsure	63 (3.6%)	87 (2.9%)
(Missing)	14 (0.8%)	12 (0.4%)
Experienced religious criticism, n (%)		

Table S13d. Unweighted summary statistics for Wave 1 outcome variables in Egypt by retention status.

**Outcome**	**Attritors–Not Observed in Wave 2**<br>N = 1,716	**Retained–Observed in Wave 2**<br>N = 3,027
Agree	477 (27.8%)	888 (29.3%)
Disagree	970 (56.5%)	1,663 (54.9%)
Not relevant	123 (7.2%)	229 (7.6%)
Unsure	137 (8.0%)	240 (7.9%)
(Missing)	9 (0.5%)	7 (0.2%)
Faith-sharing, n (%)		
Agree	711 (41.5%)	1,263 (41.7%)
Disagree	830 (48.4%)	1,435 (47.4%)
Not relevant	109 (6.4%)	239 (7.9%)
Unsure	53 (3.1%)	82 (2.7%)
(Missing)	13 (0.7%)	8 (0.3%)

Note. N (%); this table is based on non-imputed data. Cumulative percentages for variables may not add up to 100% due to rounding.

Table S13e. Summary of fitted attrition model in Egypt

Characteristic	Odds Ratio	95% CI	p-value
**ANNUAL_WEIGHT_R2**	0.86	0.73, 1.02	0.076
**Happiness & life satisfaction**	1.00	0.92, 1.10	0.945
**Physical & mental health**	0.93	0.85, 1.02	0.115
**Meaning & purpose**	1.00	0.91, 1.10	0.971
**Character & virtue**	0.99	0.90, 1.09	0.883
**Close social relationships**	1.07	0.98, 1.17	0.118
**Financial & material security**	0.99	0.90, 1.09	0.866
**Extraversion**	0.95	0.87, 1.04	0.251
**Openness to experience**	1.05	0.97, 1.14	0.208
**Agreeableness**	0.97	0.89, 1.05	0.414
**Conscientiousness**	0.98	0.90, 1.07	0.718
**Neuroticism**	1.03	0.95, 1.12	0.458
**Depression symptoms composite**	0.98	0.91, 1.07	0.691
**Anxiety symptoms composite**	1.03	0.94, 1.13	0.536
**Loneliness**	0.99	0.90, 1.09	0.847
**Days exercise per week**	0.99	0.91, 1.09	0.862
**Year of birth (age group)**			
1983-1993 (current age: 30-39 years)	—	—	
1973-1983 (current age: 40-49 years)	0.98	0.80, 1.21	0.846
1998-2005 (current age: 18-24 years)	1.18	0.83, 1.68	0.357
1963-1973 (current age: 50-59 years)	1.09	0.84, 1.41	0.510
1993-1998 (current age: 25-29 years)	1.10	0.84, 1.44	0.473
1953-1963 (current age: 60-69 years)	0.97	0.64, 1.45	0.874
1943-1953 (current age: 70-79 years)	0.70	0.30, 1.59	0.385
1943 or earlier (current age: 80+ years)	0.00	0.00, 0.00	2.91e-29
**Gender of respondent**			
Female	—	—	
Male	1.50	1.15, 1.95	0.003
**Marital status**			
Married	—	—	
Single/Never been married	0.80	0.61, 1.06	0.124
Widowed	1.33	0.93, 1.89	0.113
Divorced	0.94	0.55, 1.59	0.800
Separated	0.78	0.35, 1.74	0.531
**Employment status**			
Homemaker	—	—	
Employed for an employer	0.87	0.65, 1.16	0.334
Self-employed	0.73	0.54, 0.97	0.034
Student	1.17	0.78, 1.75	0.450
Retired	0.94	0.54, 1.62	0.812
Unemployed and looking for a job	0.71	0.46, 1.11	0.135
None of these/Other	1.56	0.40, 6.01	0.511
**Religious attendance**			
Never	—	—	
Once a week	1.04	0.86, 1.26	0.659
More than once a week	0.98	0.79, 1.21	0.856
A few times a year	1.84	1.36, 2.50	2.00e-04
One to three times a month	1.05	0.77, 1.42	0.752
**Educational attainment (16+ years)**			
9-15	—	—	
Up to 8	1.13	0.88, 1.45	0.330
16+	1.04	0.77, 1.39	0.804

Table S13e. Summary of fitted attrition model in Egypt

Characteristic	Odds Ratio	95% CI	p-value
**Born in This country**			
Born in this country	—	—	
Born in another country	0.40	0.11, 1.48	0.165
**Race plurality (prominent race/ethnic group [0] or not [1])**	0.99	0.93, 1.05	0.655
**Urbanicity**			
A small town or village	—	—	
A large city	1.08	0.86, 1.34	0.503
A rural area or on a farm	1.09	0.89, 1.32	0.399
A suburb of a large city	1.09	0.77, 1.53	0.635
**Monthly household income**			
Egypt: 1,001 – 3,000 EGP	—	—	
Egypt: 3,001 – 5,000 EGP	1.16	0.94, 1.42	0.167
Egypt: 501 – 1,000 EGP	0.97	0.76, 1.25	0.820
Egypt: 5,001 – 7,000 EGP	0.93	0.67, 1.28	0.642
Egypt: 201 – 500 EGP	0.66	0.45, 0.98	0.041
(None/No household income)	1.17	0.73, 1.89	0.501
Egypt: 7,001 – 10,000 EGP	0.83	0.49, 1.41	0.483
Egypt: 200 EGP or less	1.20	0.51, 2.83	0.670
Egypt: 10,001 – 20,000 EGP	1.82	0.67, 4.96	0.237
Egypt: More than 20,000 EGP	1.39	0.15, 13.0	0.768

Abbreviations: CI = Confidence Interval, OR = Odds Ratio

Notes. N=4729; attrition weights were estimated using the 'survey::svyglm(family=quasibinomial('logit'))' function. All continuous predictors were standardized and all categorical predictors used the most common category as the reference group. Reported p-values are based on the fitted regression model and no adjustments for multiple testing were done within this table.

Table S13f. Summary of principal components in Egypt

PC	Percent Explained by each PC	Cumulative Percent Explained
1	35.72	35.72
2	4.80	40.52
3	2.48	43.00
4	1.99	44.99
5	1.89	46.87
6	1.70	48.58
**7**	**1.57**	**50.15**
8	1.54	51.69
9	1.44	53.13
10	1.37	54.50
11	1.32	55.82
12	1.29	57.11
13	1.24	58.35
14	1.18	59.53
15	1.14	60.67
16	1.12	61.79
17	1.07	62.85
18	1.06	63.91
19	1.04	64.95
20	1.01	65.96

Notes. N=4729; PCA was conducted using ’survey::svyprcomp(.)’ function using all available contemporaneous exposures at wave 1. All PCs were standardized prior to being used as predictors. The bolded row represented the number of retained components for analysis was 7.

Table S13g. Associations of forgivingness with adult well-being and other outcomes at Wave 2 in Egypt

	Model 1: Demographic and Childhood Variables as Covariates					Model 2: Demographic, Childhood, and Other Wave 1 Confounding Variables (Via Principal Components) as Covariates				
Outcome	RR	ES	95% CI	SE	p-value	RR	ES	95% CI	SE	p-value
Human Flourishing										
Secure flourishing index		0.05	(0.02,0.09)	0.02	0.005*		0.02	(-0.02,0.05)	0.02	0.362
Flourishing index		0.06	(0.02,0.10)	0.02	2.21e-03**		0.02	(-0.01,0.06)	0.02	0.172
Happiness & life satisfaction		0.05	(0.01,0.09)	0.02	0.011*		0.02	(-0.02,0.05)	0.02	0.370
Physical & mental health		0.03	(-0.01,0.08)	0.02	0.125		0.01	(-0.03,0.05)	0.02	0.524
Meaning & purpose		0.02	(-0.02,0.07)	0.02	0.354		-0.00	(-0.04,0.04)	0.02	0.981
Character & virtue		0.05	(0.00,0.10)	0.02	0.037*		0.04	(-0.01,0.09)	0.02	0.108
Close social relationships		0.03	(-0.01,0.08)	0.02	0.142		0.01	(-0.04,0.05)	0.02	0.752
Financial & material security		0.02	(-0.03,0.06)	0.02	0.443		-0.01	(-0.05,0.03)	0.02	0.600
Psychological Well-Being										
Happiness (a)		0.02	(-0.02,0.07)	0.02	0.250		-0.00	(-0.04,0.04)	0.02	0.854
Life satisfaction (a)		0.06	(0.01,0.10)	0.02	0.011*		0.03	(-0.01,0.07)	0.02	0.158
Current life evaluation		0.02	(-0.03,0.07)	0.02	0.452		-0.00	(-0.05,0.04)	0.02	0.841
Future life evaluation		0.07	(0.02,0.12)	0.02	4.05e-03**		0.05	(0.01,0.10)	0.02	0.027*
Optimism		0.02	(-0.04,0.07)	0.03	0.542		-0.00	(-0.05,0.05)	0.03	0.955
Freedom to pursue what's important		0.03	(-0.02,0.07)	0.02	0.217		0.01	(-0.03,0.05)	0.02	0.710
Inner peace	1.02		(1.00,1.04)	0.01	0.062	1.01		(1.00,1.03)	0.01	0.116
Life balance	1.01		(0.99,1.03)	0.01	0.546	1.00		(0.98,1.02)	0.01	0.946
Sense of mastery	1.00		(0.99,1.02)	0.01	0.780	1.00		(0.98,1.01)	0.01	0.875
Meaningful activities (c)		0.00	(-0.05,0.05)	0.02	0.982		-0.02	(-0.06,0.03)	0.02	0.477
Understanding purpose (c)		0.03	(-0.01,0.08)	0.02	0.120		0.02	(-0.03,0.06)	0.02	0.444
Self-rated mental health (b)		0.02	(-0.02,0.06)	0.02	0.368		0.00	(-0.04,0.04)	0.02	0.958
Psychological Distress										
Traumatic distress	1.00		(0.98,1.03)	0.01	0.901	1.01		(0.98,1.03)	0.01	0.485
Depression symptoms composite	0.99		(0.97,1.01)	0.01	0.344	1.00		(0.98,1.02)	0.01	0.900
Depression – feel hopeless	1.00		(0.98,1.02)	0.01	0.764	1.01		(0.99,1.03)	0.01	0.168
Depression – loss of interest	0.99		(0.97,1.01)	0.01	0.241	0.99		(0.97,1.01)	0.01	0.593
Anxiety symptoms composite	0.98		(0.96,1.00)	0.01	0.132	1.00		(0.98,1.02)	0.01	0.757
Anxiety – feel on edge	0.99		(0.97,1.01)	0.01	0.224	1.00		(0.98,1.02)	0.01	0.775
Anxiety – cannot stop worrying	0.99		(0.97,1.01)	0.01	0.214	1.00		(0.98,1.02)	0.01	0.948
Suffering	1.01		(0.99,1.03)	0.01	0.431	1.01		(0.99,1.03)	0.01	0.210
Social Well-Being										
Relationship contentment (e)		0.02	(-0.03,0.06)	0.02	0.449		-0.00	(-0.05,0.04)	0.02	0.868
Relationship satisfaction (e)		0.04	(-0.01,0.09)	0.03	0.113		0.02	(-0.03,0.06)	0.02	0.517
Social support		-0.02	(-0.06,0.02)	0.02	0.382		-0.04	(-0.08,-0.00)	0.02	0.038*
Intimate/close friend	1.02		(1.00,1.03)	0.01	0.078	1.01		(0.99,1.03)	0.01	0.185
Government approval										
Say in government	1.02		(0.99,1.04)	0.01	0.169	1.02		(0.99,1.04)	0.01	0.169
Belonging in country		0.04	(-0.00,0.09)	0.02	0.064		0.02	(-0.02,0.07)	0.02	0.341
City/place satisfaction	1.02		(1.00,1.03)	0.01	0.039*	1.01		(0.99,1.03)	0.01	0.271
Trust within country	1.01		(0.98,1.03)	0.01	0.474	1.00		(0.98,1.03)	0.01	0.911
Social Participation										
Ever been married	1.00		(0.99,1.01)	0.01	0.720	1.00		(0.99,1.01)	0.01	0.658
Currently divorced	1.00		(1.00,1.00)	0.00	0.910	1.00		(1.00,1.00)	0.00	0.932
Number of children		0.03	(-0.01,0.06)	0.02	0.114		0.04	(0.00,0.07)	0.02	0.028*
Weekly+ community participation	1.00		(0.98,1.01)	0.01	0.535	1.00		(0.99,1.01)	0.01	0.843
Weekly+ religious attendance	1.00		(0.99,1.02)	0.01	0.587	1.01		(0.99,1.02)	0.01	0.414
Social Distress										
Loneliness		-0.03	(-0.07,0.01)	0.02	0.134		-0.01	(-0.05,0.03)	0.02	0.594
Perceived discrimination	1.02		(0.99,1.04)	0.01	0.206	1.02		(0.99,1.04)	0.01	0.158
Character & Prosocial Behavior										
Orientation to promote good (d)		0.04	(-0.00,0.09)	0.02	0.070		0.03	(-0.01,0.08)	0.02	0.176
Delayed gratification (d)		0.03	(-0.01,0.08)	0.02	0.151		0.03	(-0.02,0.08)	0.02	0.243
Hope		0.06	(0.01,0.11)	0.03	0.015*		0.04	(-0.01,0.09)	0.02	0.106
Gratitude		0.04	(-0.01,0.09)	0.03	0.127		0.02	(-0.03,0.07)	0.02	0.356
Showing love/care		0.02	(-0.03,0.07)	0.03	0.370		0.01	(-0.04,0.06)	0.02	0.750
Forgivingness	1.06		(1.04,1.08)	0.01	2.82e-08***	1.06		(1.04,1.08)	0.01	1.19e-07***
Charitable giving	1.01		(0.99,1.04)	0.01	0.241	1.01		(0.99,1.04)	0.01	0.347
Helping strangers	1.02		(0.99,1.04)	0.01	0.205	1.01		(0.99,1.04)	0.01	0.234
Volunteering	1.01		(1.00,1.02)	0.01	0.135	1.01		(1.00,1.03)	0.01	0.095
Physical Health & Health Behavior										
Self-rated physical health (b)		0.03	(-0.01,0.08)	0.02	0.171		0.02	(-0.03,0.06)	0.02	0.428
Health problems	1.01		(0.99,1.02)	0.01	0.394	1.01		(0.99,1.02)	0.01	0.216
Pain in past 4 weeks	1.01		(0.99,1.03)	0.01	0.571	1.01		(0.99,1.03)	0.01	0.362
Daily smoker	0.99		(0.98,1.01)	0.01	0.509	1.00		(0.98,1.02)	0.01	0.894
Number of drinks per week		-0.00	(-0.04,0.03)	0.02	0.881		0.00	(-0.03,0.04)	0.02	0.875
Days exercise per week		0.02	(-0.02,0.06)	0.02	0.374		0.02	(-0.02,0.06)	0.02	0.279
Socioeconomic Outcomes										
Financial security (f)		0.02	(-0.03,0.06)	0.02	0.459		-0.01	(-0.05,0.04)	0.02	0.789
Material security (f)		0.01	(-0.03,0.06)	0.02	0.611		-0.01	(-0.06,0.03)	0.02	0.577
Educational attainment (16+ years)	1.00		(1.00,1.01)	0.00	0.496	1.00		(1.00,1.01)	0.00	0.547
Currently employed	1.01		(0.99,1.03)	0.01	0.172	1.01		(1.00,1.03)	0.01	0.147
Financially comfortable/getting by	1.00		(0.98,1.02)	0.01	0.989	0.99		(0.97,1.01)	0.01	0.458
Own home	1.01		(0.99,1.03)	0.01	0.484	1.00		(0.99,1.02)	0.01	0.656
Income – top quintile	1.00		(0.98,1.02)	0.01	0.828	1.00		(0.98,1.02)	0.01	0.763
Religion & Spirituality										
Religious/spiritual connection	1.01		(0.99,1.02)	0.01	0.238	1.01		(0.99,1.02)	0.01	0.356
Belief in life after death	0.99		(0.97,1.01)	0.01	0.160	0.98		(0.96,1.00)	0.01	0.066
Transformative religious experience	1.00		(0.98,1.02)	0.01	0.972	1.01		(0.98,1.03)	0.01	0.646
Religious reading or listening	1.00		(0.98,1.03)	0.01	0.786	1.00		(0.98,1.03)	0.01	0.949
Prayer or meditation	1.01		(0.99,1.03)	0.01	0.162	1.01		(0.99,1.03)	0.01	0.376
Belief in God/gods/spiritual forces										
Religious centrality	1.00		(0.99,1.02)	0.01	0.632	1.00		(0.99,1.02)	0.01	0.674
Religious/spiritual comfort	1.00		(1.00,1.01)	0.00	0.519	1.00		(0.99,1.01)	0.00	0.639
Feel loved by God	1.00		(1.00,1.01)	0.00	0.663	1.00		(1.00,1.01)	0.00	0.568
Feel punished by God	1.00		(0.98,1.01)	0.01	0.702	1.00		(0.98,1.01)	0.01	0.712
Experienced religious criticism	0.98		(0.96,1.00)	0.01	0.047*	0.99		(0.97,1.01)	0.01	0.189
Faith-sharing	0.99		(0.97,1.02)	0.01	0.661	1.00		(0.98,1.03)	0.01	0.966

Notes. N=4729; Reference for focal predictor: never/rarely. RR, risk-ratio, null effect is 1.00; ES, effect size measure for standardized regression coefficient, null effect is 0.00; SE, standard error, the SE reported for binary/Likert-type outcomes where risk-ratios are on the log(RR) scale; CI, confidence interval; p-value, a Wald-type test of the null hypothesis that the effect of the focal predictor is zero; (a) item part of the Happiness & Life Satisfaction domain of the Secure Flourishing Index; (b) item part of the Physical & Mental Health domain of the Secure Flourishing Index; (c) item part of the Meaning & Purpose domain of the Secure Flourishing Index; (d) item part of the Character & Virtue domain of the Secure Flourishing Index; (e) item part of the Subjective Social Connectedness domain of the Secure Flourishing Index; (f) item part of the Financial & Material Security domain of the Secure Flourishing Index.

Multiple imputation was performed to impute missing data on the covariates, exposure, and outcomes. All models controlled for sociodemographic and childhood factors assessed at Wave 1. For Model 2 with PC (principal components), the first seven principal components of the entire set of contemporaneous confounders assessed at Wave 1 were included as additional covariates of the outcomes at Wave 2.

An outcome-wide analytic approach was used, and a separate model was run for each outcome. A different type of model was run depending on the nature of the outcome: (1) for each binary outcome, a weighted generalized linear model (with a log link and Poisson distribution) was used to estimate an RR; and (2) for each continuous outcome, a weighted linear regression model was used to estimate a ES. All effect sizes were standardized. For continuous outcomes, the ES represents the change in SD on the outcome between the lower and upper categories of the binary focal predictor. For binary outcomes, the RR represents the change in risk of being in the upper category compared to the lower category between the lower and upper categories of the binary focal predictor.

P-value significance thresholds: $p < 0.05$*, $p < 0.005$**, (Bonferroni) $p < 6.41e-04$***, correction for multiple testing using Bonferroni adjusted significant threshold.

Table S13h. Associations of forgivingness with adult well-being and other outcomes at Wave 2 in Egypt using complete-case analyses with attrition weights.

	Model 1: Demographic and Childhood Variables as Covariates					Model 2: Demographic, Childhood, and Other Wave 1 Confounding Variables (Via Principal Components) as Covariates				
Outcome	RR	ES	95% CI	SE	p-value	RR	ES	95% CI	SE	p-value
Human Flourishing										
Secure flourishing index		0.04	(-0.00,0.08)	0.02	0.067		0.01	(-0.03,0.05)	0.02	0.511
Flourishing index		0.04	(-0.00,0.08)	0.02	0.067		0.02	(-0.02,0.05)	0.02	0.444
Happiness & life satisfaction		0.04	(0.01,0.08)	0.02	0.023*		0.02	(-0.02,0.06)	0.02	0.280
Physical & mental health		0.03	(-0.02,0.08)	0.02	0.225		0.02	(-0.03,0.06)	0.02	0.481
Meaning & purpose		-0.00	(-0.04,0.04)	0.02	0.980		-0.01	(-0.06,0.03)	0.02	0.502
Character & virtue		0.05	(0.00,0.10)	0.03	0.041*		0.04	(-0.00,0.09)	0.03	0.077
Close social relationships		0.00	(-0.04,0.05)	0.02	0.833		-0.01	(-0.06,0.03)	0.02	0.579
Financial & material security		0.02	(-0.02,0.06)	0.02	0.371		0.00	(-0.04,0.04)	0.02	0.944
Psychological Well-Being										
Happiness (a)		0.02	(-0.02,0.06)	0.02	0.344		0.00	(-0.04,0.04)	0.02	0.905
Life satisfaction (a)		0.05	(0.01,0.09)	0.02	0.010*		0.03	(-0.01,0.07)	0.02	0.125
Current life evaluation		0.00	(-0.04,0.04)	0.02	0.959		-0.01	(-0.05,0.02)	0.02	0.466
Future life evaluation		0.06	(0.01,0.10)	0.02	0.018*		0.04	(-0.00,0.09)	0.02	0.056
Optimism		0.01	(-0.04,0.06)	0.03	0.703		0.00	(-0.05,0.05)	0.02	0.964
Freedom to pursue what's important		0.04	(-0.01,0.09)	0.02	0.101		0.03	(-0.02,0.08)	0.02	0.210
Inner peace	1.03		(1.01,1.05)	0.01	0.008*	1.02		(1.00,1.05)	0.01	0.019*
Life balance	1.01		(0.98,1.03)	0.01	0.491	1.00		(0.98,1.03)	0.01	0.708
Sense of mastery	1.00		(0.98,1.01)	0.01	0.688	0.99		(0.98,1.01)	0.01	0.348
Meaningful activities (c)		-0.02	(-0.06,0.03)	0.02	0.414		-0.03	(-0.07,0.01)	0.02	0.193
Understanding purpose (c)		0.02	(-0.03,0.07)	0.02	0.449		0.01	(-0.04,0.05)	0.02	0.805
Self-rated mental health (b)		0.01	(-0.04,0.05)	0.02	0.721		-0.00	(-0.05,0.04)	0.02	0.887
Psychological Distress										
Traumatic distress	1.00		(0.98,1.02)	0.01	0.951	1.01		(0.98,1.03)	0.01	0.595
Depression symptoms composite	0.99		(0.97,1.01)	0.01	0.405	1.00		(0.98,1.02)	0.01	0.832
Depression – feel hopeless	1.01		(0.99,1.03)	0.01	0.201	1.02		(1.00,1.04)	0.01	0.041*
Depression – loss of interest	0.98		(0.96,1.01)	0.01	0.175	0.99		(0.97,1.01)	0.01	0.322
Anxiety symptoms composite	0.99		(0.97,1.01)	0.01	0.277	1.00		(0.98,1.02)	0.01	0.833
Anxiety – feel on edge	0.99		(0.97,1.01)	0.01	0.311	1.00		(0.98,1.02)	0.01	0.774
Anxiety – cannot stop worrying	0.99		(0.97,1.01)	0.01	0.358	1.00		(0.98,1.02)	0.01	0.942
Suffering	1.01		(0.99,1.03)	0.01	0.380	1.01		(0.99,1.03)	0.01	0.237
Social Well-Being										
Relationship contentment (e)		-0.01	(-0.05,0.03)	0.02	0.608		-0.03	(-0.07,0.02)	0.02	0.257
Relationship satisfaction (e)		0.02	(-0.03,0.07)	0.03	0.421		0.00	(-0.04,0.05)	0.02	0.877
Social support		-0.03	(-0.08,0.01)	0.02	0.131		-0.05	(-0.09,-0.01)	0.02	0.025*
Intimate/close friend	1.02		(1.00,1.04)	0.01	0.035*	1.02		(1.00,1.03)	0.01	0.066
Government approval										
Say in government	1.02		(1.00,1.04)	0.01	0.107	1.02		(1.00,1.04)	0.01	0.064
Belonging in country		0.03	(-0.02,0.08)	0.03	0.181		0.02	(-0.03,0.07)	0.03	0.482
City/place satisfaction	1.02		(1.00,1.05)	0.01	0.027*	1.02		(1.00,1.04)	0.01	0.127
Trust within country	1.01		(0.99,1.03)	0.01	0.462	1.00		(0.98,1.02)	0.01	0.905
Social Participation										
Ever been married	1.00		(0.99,1.02)	0.01	0.576	1.00		(0.99,1.02)	0.01	0.511
Currently divorced	1.00		(1.00,1.00)	0.00	0.745	1.00		(1.00,1.00)	0.00	0.836
Number of children		0.02	(-0.01,0.06)	0.02	0.241		0.03	(-0.01,0.07)	0.02	0.106
Weekly+ community participation	1.00		(0.98,1.01)	0.01	0.685	1.00		(0.99,1.01)	0.01	0.990
Weekly+ religious attendance	1.00		(0.99,1.02)	0.01	0.616	1.01		(0.99,1.03)	0.01	0.510
Social Distress										
Loneliness		-0.02	(-0.06,0.02)	0.02	0.312		-0.01	(-0.05,0.03)	0.02	0.727
Perceived discrimination	1.02		(1.00,1.05)	0.01	0.029*	1.03		(1.00,1.05)	0.01	0.019*
Character & Prosocial Behavior										
Orientation to promote good (d)		0.04	(-0.02,0.09)	0.03	0.181		0.03	(-0.02,0.08)	0.03	0.279
Delayed gratification (d)		0.04	(-0.00,0.09)	0.02	0.077		0.04	(-0.01,0.08)	0.02	0.107
Hope		0.07	(0.02,0.12)	0.03	0.011*		0.05	(0.00,0.10)	0.03	0.048*
Gratitude		0.03	(-0.02,0.09)	0.03	0.185		0.02	(-0.03,0.07)	0.02	0.356
Showing love/care		0.01	(-0.04,0.06)	0.02	0.644		0.00	(-0.04,0.05)	0.02	0.895
Forgivingness	1.07		(1.05,1.09)	0.01	5.38e-10***	1.07		(1.04,1.09)	0.01	3.27e-09***
Charitable giving	1.01		(0.99,1.04)	0.01	0.358	1.01		(0.98,1.04)	0.01	0.495
Helping strangers	1.02		(0.99,1.04)	0.01	0.196	1.01		(0.99,1.04)	0.01	0.200
Volunteering	1.01		(1.00,1.03)	0.01	0.022*	1.02		(1.00,1.03)	0.01	0.009*
Physical Health & Health Behavior										
Self-rated physical health (b)		0.04	(-0.01,0.09)	0.03	0.162		0.03	(-0.02,0.07)	0.02	0.281
Health problems	1.00		(0.99,1.02)	0.01	0.631	1.01		(0.99,1.02)	0.01	0.494
Pain in past 4 weeks	1.01		(0.99,1.03)	0.01	0.399	1.01		(0.99,1.03)	0.01	0.252
Daily smoker	1.00		(0.98,1.02)	0.01	0.832	1.00		(0.99,1.02)	0.01	0.752
Number of drinks per week		-0.00	(-0.03,0.03)	0.02	0.900		0.01	(-0.03,0.05)	0.02	0.752
Days exercise per week		0.03	(-0.01,0.08)	0.02	0.142		0.04	(-0.00,0.08)	0.02	0.077
Socioeconomic Outcomes										
Financial security (f)		0.02	(-0.03,0.06)	0.02	0.496		0.00	(-0.04,0.04)	0.02	0.988
Material security (f)		0.02	(-0.02,0.07)	0.02	0.367		0.00	(-0.04,0.05)	0.02	0.914
Educational attainment (16+ years)	1.00		(1.00,1.01)	0.00	0.359	1.00		(1.00,1.01)	0.00	0.403
Currently employed	1.02		(1.00,1.04)	0.01	0.020*	1.02		(1.00,1.04)	0.01	0.017*
Financially comfortable/getting by	1.00		(0.97,1.02)	0.01	0.772	0.99		(0.97,1.01)	0.01	0.390
Own home	1.00		(0.98,1.02)	0.01	0.880	1.00		(0.98,1.02)	0.01	0.768
Income – top quintile	0.99		(0.97,1.02)	0.01	0.681	0.99		(0.97,1.02)	0.01	0.670
Religion & Spirituality										
Religious/spiritual connection	1.01		(0.99,1.02)	0.01	0.390	1.01		(0.99,1.02)	0.01	0.550
Belief in life after death	0.99		(0.97,1.00)	0.01	0.118	0.98		(0.96,1.00)	0.01	0.035*
Transformative religious experience	1.00		(0.98,1.03)	0.01	0.933	1.01		(0.98,1.03)	0.01	0.587
Religious reading or listening	1.00		(0.97,1.02)	0.01	0.803	0.99		(0.97,1.02)	0.01	0.602
Prayer or meditation	1.01		(0.99,1.03)	0.01	0.407	1.00		(0.99,1.02)	0.01	0.648
Belief in God/gods/spiritual forces										
Religious centrality	1.01		(0.99,1.03)	0.01	0.419	1.01		(0.99,1.03)	0.01	0.381
Religious/spiritual comfort	1.00		(1.00,1.01)	0.00	0.476	1.00		(1.00,1.01)	0.00	0.599
Feel loved by God	1.00		(1.00,1.00)	0.00	0.850	1.00		(1.00,1.01)	0.00	0.635
Feel punished by God	0.99		(0.98,1.01)	0.01	0.255	0.99		(0.98,1.00)	0.01	0.235
Experienced religious criticism	0.98		(0.96,1.00)	0.01	0.057	0.99		(0.97,1.01)	0.01	0.179
Faith-sharing	1.00		(0.97,1.02)	0.01	0.671	1.00		(0.98,1.02)	0.01	0.991

Table S13h. Associations of forgivingness with adult well-being and other outcomes at Wave 2 in Egypt using complete-case analyses with attrition weights.

	Model 1: Demographic and Childhood Variables as Covariates					Model 2: Demographic, Childhood, and Other Wave 1 Confounding Variables (Via Principal Components) as Covariates				
Outcome	RR	ES	95% CI	SE	p-value	RR	ES	95% CI	SE	p-value

Notes. N=3040; Reference for focal predictor: never/rarely. RR, risk-ratio, null effect is 1.00; ES, effect size measure for standardized regression coefficient, null effect is 0.00; SE, standard error, the SE reported for binary/Likert-type outcomes where risk-ratios are on the log(RR) scale; CI, confidence interval; p-value, a Wald-type test of the null hypothesis that the effect of the focal predictor is zero; (a) item part of the Happiness & Life Satisfaction domain of the Secure Flourishing Index; (b) item part of the Physical & Mental Health domain of the Secure Flourishing Index; (c) item part of the Meaning & Purpose domain of the Secure Flourishing Index; (d) item part of the Character & Virtue domain of the Secure Flourishing Index; (e) item part of the Subjective Social Connectedness domain of the Secure Flourishing Index; (f) item part of the Financial & Material Security domain of the Secure Flourishing Index.

Attrition weights were computed to adjust the complete case data (those who responded at Wave 2 to at least 50% of the questions) and multiple imputation was used to impute missing data on all remaining within wave on the covariates, exposure, and outcomes. All models controlled for sociodemographic and childhood factors assessed at Wave 1. For Model 2 with PC (principal components), the first seven principal components of the entire set of contemporaneous confounders assessed at Wave 1 were included as additional covariates of the outcomes at Wave 2.

An outcome-wide analytic approach was used, and a separate model was run for each outcome. A different type of model was run depending on the nature of the outcome: (1) for each binary outcome, a weighted generalized linear model (with a log link and Poisson distribution) was used to estimate an RR; and (2) for each continuous outcome, a weighted linear regression model was used to estimate a ES. All effect sizes were standardized. For continuous outcomes, the ES represents the change in SD on the outcome between the lower and upper categories of the binary focal predictor. For binary outcomes, the RR represents the change in risk of being in the upper category compared to the lower category between the lower and upper categories of the binary focal predictor.

P-value significance thresholds: $p < 0.05$*, $p < 0.005$**, (Bonferroni) $p < 6.41e-04$***, correction for multiple testing using Bonferroni adjusted significant threshold.

Table S13i. Sensitivity analysis of forgivingness outcome-wide results to unmeasured confounding using E-values in Egypt

	Multiple Imputation				Complete Case w/ Attrition Weights			
	Model 1: Demographics and Childhood Variables as Covariates		Model 2: Demographics, Childhood, and Other Wave 1 Confounders (Via Principal Components) as Covariates		Model 1: Demographics and Childhood Variables as Covariates		Model 2: Demographics, Childhood, and Other Wave 1 Confounders (Via Principal Components) as Covariates	
Outcome	EE	ECI	EE	ECI	EE	ECI	EE	ECI
Human Flourishing								
Secure flourishing index	1.59	1.26	1.25	1.00	1.45	1.00	1.23	1.00
Flourishing index	1.62	1.31	1.33	1.00	1.45	1.00	1.25	1.00
Happiness & life satisfaction	1.54	1.21	1.26	1.00	1.49	1.14	1.30	1.00
Physical & mental health	1.41	1.00	1.22	1.00	1.38	1.00	1.25	1.00
Meaning & purpose	1.31	1.00	1.04	1.00	1.04	1.00	1.24	1.00
Character & virtue	1.55	1.11	1.46	1.00	1.56	1.08	1.51	1.00
Close social relationships	1.42	1.00	1.16	1.00	1.13	1.00	1.22	1.00
Financial & material security	1.27	1.00	1.20	1.00	1.30	1.00	1.07	1.00
Psychological Well-Being								
Happiness	1.33	1.00	1.11	1.00	1.30	1.00	1.09	1.00
Life satisfaction	1.60	1.23	1.39	1.00	1.58	1.23	1.41	1.00
Current life evaluation	1.28	1.00	1.13	1.00	1.06	1.00	1.24	1.00
Future life evaluation	1.70	1.33	1.56	1.16	1.59	1.19	1.50	1.00
Optimism	1.26	1.00	1.07	1.00	1.20	1.00	1.06	1.00
Freedom to pursue what's important	1.36	1.00	1.17	1.00	1.47	1.00	1.39	1.00
Inner peace	1.28	1.00	1.26	1.00	1.38	1.17	1.36	1.12
Life balance	1.16	1.00	1.05	1.00	1.18	1.00	1.13	1.00
Sense of mastery	1.08	1.00	1.06	1.00	1.10	1.00	1.16	1.00
Meaningful activities	1.04	1.00	1.26	1.00	1.29	1.00	1.38	1.00
Understanding purpose	1.42	1.00	1.26	1.00	1.28	1.00	1.14	1.00
Self-rated mental health	1.29	1.00	1.06	1.00	1.17	1.00	1.10	1.00
Psychological Distress								
Traumatic distress	1.07	1.00	1.19	1.00	1.05	1.00	1.16	1.00
Depression symptoms composite	1.21	1.00	1.06	1.00	1.20	1.00	1.09	1.00
Depression – feel hopeless	1.10	1.00	1.24	1.00	1.23	1.00	1.30	1.05
Depression – loss of interest	1.23	1.00	1.15	1.00	1.28	1.00	1.23	1.00
Anxiety symptoms composite	1.27	1.00	1.10	1.00	1.23	1.00	1.09	1.00
Anxiety – feel on edge	1.24	1.00	1.10	1.00	1.21	1.00	1.10	1.00
Anxiety – cannot stop worrying	1.24	1.00	1.05	1.00	1.21	1.00	1.05	1.00
Suffering	1.18	1.00	1.24	1.00	1.19	1.00	1.23	1.00
Social Well-Being								
Relationship contentment	1.27	1.00	1.11	1.00	1.21	1.00	1.34	1.00
Relationship satisfaction	1.47	1.00	1.26	1.00	1.30	1.00	1.11	1.00
Social support	1.29	1.00	1.49	1.09	1.43	1.00	1.55	1.15
Intimate/close friend	1.27	1.00	1.22	1.00	1.31	1.07	1.28	1.00
Government approval								
Say in government	1.28	1.00	1.28	1.00	1.31	1.00	1.32	1.00
Belonging in country	1.49	1.00	1.31	1.00	1.42	1.00	1.28	1.00
City/place satisfaction	1.29	1.05	1.20	1.00	1.35	1.10	1.27	1.00
Trust within country	1.19	1.00	1.07	1.00	1.19	1.00	1.07	1.00
Social Participation								
Ever been married	1.09	1.00	1.10	1.00	1.12	1.00	1.13	1.00
Currently divorced	1.03	1.00	1.02	1.00	1.04	1.00	1.03	1.00
Number of children	1.36	1.00	1.45	1.12	1.32	1.00	1.41	1.00
Weekly+ community participation	1.12	1.00	1.06	1.00	1.10	1.00	1.02	1.00
Weekly+ religious attendance	1.13	1.00	1.17	1.00	1.14	1.00	1.16	1.00
Social Distress								
Loneliness	1.41	1.00	1.20	1.00	1.31	1.00	1.16	1.00
Perceived discrimination	1.27	1.00	1.29	1.00	1.36	1.09	1.38	1.13
Character & Prosocial Behavior								
Orientation to promote good	1.51	1.00	1.41	1.00	1.44	1.00	1.38	1.00
Delayed gratification	1.42	1.00	1.37	1.00	1.49	1.00	1.46	1.00
Hope	1.64	1.22	1.47	1.00	1.70	1.25	1.56	1.04
Gratitude	1.46	1.00	1.32	1.00	1.42	1.00	1.33	1.00
Showing love/care	1.32	1.00	1.17	1.00	1.21	1.00	1.10	1.00
Forgivingness	1.64	1.47	1.62	1.45	1.74	1.56	1.72	1.53
Charitable giving	1.26	1.00	1.23	1.00	1.23	1.00	1.20	1.00
Helping strangers	1.27	1.00	1.25	1.00	1.26	1.00	1.26	1.00
Volunteering	1.21	1.00	1.23	1.00	1.26	1.09	1.28	1.13
Physical Health & Health Behavior								
Self-rated physical health	1.40	1.00	1.27	1.00	1.44	1.00	1.35	1.00
Health problems	1.16	1.00	1.19	1.00	1.12	1.00	1.15	1.00
Pain in past 4 weeks	1.15	1.00	1.20	1.00	1.19	1.00	1.24	1.00
Daily smoker	1.15	1.00	1.06	1.00	1.09	1.00	1.10	1.00
Number of drinks per week	1.09	1.00	1.10	1.00	1.08	1.00	1.15	1.00
Days exercise per week	1.29	1.00	1.32	1.00	1.41	1.00	1.46	1.00
Socioeconomic Outcomes								
Financial security	1.27	1.00	1.14	1.00	1.25	1.00	1.03	1.00
Material security	1.22	1.00	1.22	1.00	1.31	1.00	1.09	1.00
Educational attainment (16+ years)	1.09	1.00	1.08	1.00	1.11	1.00	1.11	1.00
Currently employed	1.24	1.00	1.24	1.00	1.33	1.11	1.32	1.12
Financially comfortable/getting by	1.02	1.00	1.18	1.00	1.11	1.00	1.21	1.00
Own home	1.17	1.00	1.13	1.00	1.07	1.00	1.10	1.00
Income – top quintile	1.09	1.00	1.11	1.00	1.15	1.00	1.15	1.00
Religion & Spirituality								
Religious/spiritual connection	1.19	1.00	1.17	1.00	1.17	1.00	1.14	1.00
Belief in life after death	1.25	1.00	1.30	1.00	1.26	1.00	1.32	1.07
Transformative religious experience	1.04	1.00	1.14	1.00	1.06	1.00	1.17	1.00
Religious reading or listening	1.11	1.00	1.05	1.00	1.10	1.00	1.15	1.00
Prayer or meditation	1.24	1.00	1.18	1.00	1.18	1.00	1.12	1.00
Belief in God/gods/spiritual forces								
Religious centrality	1.12	1.00	1.11	1.00	1.17	1.00	1.18	1.00
Religious/spiritual comfort	1.09	1.00	1.08	1.00	1.09	1.00	1.08	1.00
Feel loved by God	1.07	1.00	1.08	1.00	1.04	1.00	1.06	1.00
Feel punished by God	1.10	1.00	1.10	1.00	1.17	1.00	1.18	1.00
Experienced religious criticism	1.30	1.03	1.23	1.00	1.31	1.00	1.25	1.00
Faith-sharing	1.15	1.00	1.04	1.00	1.13	1.00	1.02	1.00

Notes. EE, E-value for estimate; ECI, E-value for the limit of the confidence interval. The formula for calculating E-values can be found in VanderWeele and Ding (2017). E-values for estimate are the minimum strength of association on the risk ratio scale that an unmeasured confounder would need to have with both the exposure and the outcome to fully explain away the observed association between the exposure and outcome, conditional on the measured covariates. E-values for the 95% CI closest to the null denote the minimum strength of association on the risk ratio scale that an unmeasured confounder would need to have with both the exposure and the outcome to shift the CI to include the null value, conditional on the measured covariates.

Table S14a. Weighted summary statistics for demographic and childhood variables in Germany

**Characteristic**	**Wave 1** N = 9,506	**Wave 2** N = 5,612
Forgivingness, n (%)		
Always	1,332 (14.0%)	842 (15.0%)
Often	5,742 (60.4%)	3,349 (59.7%)
Rarely	2,170 (22.8%)	1,258 (22.4%)
Never	242 (2.5%)	142 (2.5%)
(Missing)	20 (0.2%)	21 (0.4%)
Year of birth, n (%)		
1943 or earlier (current age: 80+ years)	179 (1.9%)	133 (2.4%)
1943-1953 (current age: 70-79 years)	1,085 (11.4%)	717 (12.8%)
1953-1963 (current age: 60-69 years)	1,857 (19.5%)	1,064 (19.0%)
1963-1973 (current age: 50-59 years)	1,746 (18.4%)	1,010 (18.0%)
1973-1983 (current age: 40-49 years)	1,495 (15.7%)	886 (15.8%)
1983-1993 (current age: 30-39 years)	1,406 (14.8%)	860 (15.3%)
1993-1998 (current age: 25-29 years)	775 (8.2%)	490 (8.7%)
1998-2005 (current age: 18-24 years)	963 (10.1%)	451 (8.0%)
(Missing)	0 (0%)	0 (0%)
Age of participant		
Mean	49.1	50.0
Standard Deviation	17.3	17.3
Min, Max	18.0, 99.0	19.0, 99.0
Gender, n (%)		
Male	4,623 (48.6%)	2,719 (48.5%)
Female	4,859 (51.1%)	2,881 (51.3%)
Other	12 (0.1%)	6 (0.1%)
(Missing)	11 (0.1%)	6 (0.1%)
Respondent marital status, n (%)		
Single/Never been married	2,682 (28.2%)	1,565 (27.9%)
Married	4,768 (50.2%)	2,833 (50.5%)
Separated	214 (2.3%)	104 (1.9%)
Divorced	741 (7.8%)	466 (8.3%)
Widowed	397 (4.2%)	222 (4.0%)
Domestic partner	631 (6.6%)	374 (6.7%)
(Missing)	74 (0.8%)	48 (0.9%)
Education (years), n (%)		
Up to 8	232 (2.4%)	105 (1.9%)
9-15	6,219 (65.4%)	3,560 (63.4%)
16+	3,043 (32.0%)	1,945 (34.7%)
(Missing)	12 (0.1%)	2 (0.0%)
Employment status, n (%)		
Employed for an employer	5,023 (52.8%)	2,957 (52.7%)
Self-employed	717 (7.5%)	364 (6.5%)
Retired	2,352 (24.7%)	1,453 (25.9%)
Student	653 (6.9%)	331 (5.9%)
Homemaker	247 (2.6%)	141 (2.5%)
Unemployed and looking for a job	288 (3.0%)	179 (3.2%)
None of these/Other	211 (2.2%)	129 (2.3%)
(Missing)	15 (0.2%)	57 (1.0%)
Current religious service attendance, n (%)		
More than once a week	282 (3.0%)	149 (2.7%)
Once a week	425 (4.5%)	252 (4.5%)

Table S14a. Weighted summary statistics for demographic and childhood variables in Germany

**Characteristic**	**Wave 1**<br>N = 9,506	**Wave 2**<br>N = 5,612
One to three times a month	551 (5.8%)	368 (6.6%)
A few times a year	2,350 (24.7%)	2,117 (37.7%)
Never	5,888 (61.9%)	2,714 (48.4%)
(Missing)	10 (0.1%)	11 (0.2%)
Immigration status, n (%)		
Born in this country	8,724 (91.8%)	5,147 (91.7%)
Born in another country	742 (7.8%)	438 (7.8%)
(Missing)	40 (0.4%)	27 (0.5%)
Parental marital status around age 12, n (%)		
Parents were married	7,606 (80.0%)	4,543 (81.0%)
Parents were divorced	931 (9.8%)	544 (9.7%)
Parents were never married	588 (6.2%)	342 (6.1%)
One or both of them had died	245 (2.6%)	129 (2.3%)
Unsure	66 (0.7%)	33 (0.6%)
(Missing)	70 (0.7%)	21 (0.4%)
Religious service attendance around age 12, n (%)		
At least once a week	1,929 (20.3%)	1,123 (20.0%)
One to three times a month	1,892 (19.9%)	1,153 (20.5%)
Less than once a month	2,904 (30.5%)	1,721 (30.7%)
Never	2,754 (29.0%)	1,600 (28.5%)
(Missing)	27 (0.3%)	15 (0.3%)
Relationship with mother when growing up, n (%)		
Very good	5,510 (58.0%)	3,174 (56.6%)
Somewhat good	3,009 (31.6%)	1,819 (32.4%)
Somewhat bad	502 (5.3%)	320 (5.7%)
Very bad	185 (1.9%)	110 (2.0%)
(Does not apply)	247 (2.6%)	146 (2.6%)
(Missing)	54 (0.6%)	42 (0.8%)
Relationship with father when growing up, n (%)		
Very good	4,668 (49.1%)	2,709 (48.3%)
Somewhat good	3,003 (31.6%)	1,804 (32.1%)
Somewhat bad	837 (8.8%)	515 (9.2%)
Very bad	385 (4.0%)	235 (4.2%)
(Does not apply)	540 (5.7%)	305 (5.4%)
(Missing)	73 (0.8%)	43 (0.8%)
Felt like an outsider in family when growing up, n (%)		
Yes	1,127 (11.9%)	688 (12.3%)
No	8,242 (86.7%)	4,842 (86.3%)
(Missing)	137 (1.4%)	82 (1.5%)
Experienced abuse when growing up, n (%)		
Yes	1,079 (11.4%)	687 (12.2%)
No	8,329 (87.6%)	4,891 (87.2%)
(Missing)	97 (1.0%)	34 (0.6%)
Self-rated health when growing up, n (%)		
Excellent	2,662 (28.0%)	1,539 (27.4%)
Very good	3,534 (37.2%)	2,088 (37.2%)
Good	2,543 (26.8%)	1,536 (27.4%)
Fair	609 (6.4%)	352 (6.3%)
Poor	133 (1.4%)	78 (1.4%)
(Missing)	26 (0.3%)	19 (0.3%)

Table S14a. Weighted summary statistics for demographic and childhood variables in Germany

Characteristic	Wave 1<br>N = 9,506	Wave 2<br>N = 5,612
Subjective financial status of family growing up, n (%)		
Lived comfortably	3,204 (33.7%)	1,853 (33.0%)
Got by	4,500 (47.3%)	2,664 (47.5%)
Found it difficult	1,460 (15.4%)	878 (15.6%)
Found it very difficult	315 (3.3%)	193 (3.4%)
(Missing)	26 (0.3%)	23 (0.4%)
Religious affiliation growing up, n (%)		
Christianity	5,735 (60.3%)	3,512 (62.6%)
Taoism	0 (0%)	0 (0%)
Confucianism	4 (0.0%)	6 (0.1%)
Primal, Animist, or Folk religion	19 (0.2%)	16 (0.3%)
Spiritism	0 (0%)	0 (0%)
Umbanda, Candomblé, and other African-derived religions	0 (0%)	0 (0%)
Chinese folk/traditional religion	0 (0%)	0 (0%)
Islam	359 (3.8%)	153 (2.7%)
Hinduism	15 (0.2%)	12 (0.2%)
Buddhism	26 (0.3%)	8 (0.1%)
Judaism	18 (0.2%)	7 (0.1%)
Sikhism	5 (0.1%)	6 (0.1%)
Baha'i	2 (0.0%)	1 (0.0%)
Jainism	1 (0.0%)	0 (0%)
Shinto	0 (0%)	0 (0%)
Some other religion	68 (0.7%)	42 (0.7%)
No religion/Atheist/Agnostic	3,168 (33.3%)	1,792 (31.9%)
(Missing)	86 (0.9%)	55 (1.0%)

Note. N (%); this table is based on non-imputed data. Cumulative percentages for variables may not add up to 100% due to rounding. Wave 1 characteristics weighted using the Gallup provided sampling weight, ANNUAL_WEIGHT_R2; Wave 2 characteristics weighted accounting for attrition by using the adjusted Wave 1 weight, ANNUAL_WEIGHT_R2, multiplied by the created attrition weight to account for dropout, to maintain nationally representative estimates for Wave 2 characteristics.

Table S14b. Weighted summary statistics for outcome variables in Germany

Outcome	Wave 1<br>N = 9,506	Wave 2<br>N = 5,612
Secure flourishing index		
Mean	7.0	6.9
Standard Deviation	1.4	1.4
Min, Max	0.0, 10.0	0.0, 10.0
(Missing)	163 (1.7%)	125 (2.2%)
Flourishing index		
Mean	7.1	7.0
Standard Deviation	1.4	1.4
Min, Max	0.0, 10.0	0.0, 10.0
(Missing)	158 (1.7%)	116 (2.1%)
Happiness & life satisfaction		
Mean	6.9	6.7
Standard Deviation	1.9	1.9
Min, Max	0.0, 10.0	0.0, 10.0
(Missing)	45 (0.5%)	25 (0.5%)
Physical & mental health		
Mean	6.8	6.7
Standard Deviation	1.9	1.9
Min, Max	0.0, 10.0	0.0, 10.0
(Missing)	26 (0.3%)	40 (0.7%)
Meaning & purpose		
Mean	7.2	7.1
Standard Deviation	1.8	1.9
Min, Max	0.0, 10.0	0.0, 10.0
(Missing)	32 (0.3%)	21 (0.4%)
Character & virtue		
Mean	7.3	7.2
Standard Deviation	1.6	1.6
Min, Max	0.0, 10.0	0.0, 10.0
(Missing)	24 (0.3%)	20 (0.4%)
Close social relationships		
Mean	7.3	7.2
Standard Deviation	2.2	2.2
Min, Max	0.0, 10.0	0.0, 10.0
(Missing)	58 (0.6%)	21 (0.4%)
Financial & material security		
Mean	6.5	6.6
Standard Deviation	2.7	2.6
Min, Max	0.0, 10.0	0.0, 10.0
(Missing)	12 (0.1%)	15 (0.3%)
Happiness		
Mean	6.9	6.7
Standard Deviation	1.9	1.9
Min, Max	0.0, 10.0	0.0, 10.0
(Missing)	15 (0.2%)	5 (<0.1%)
Life satisfaction		
Mean	6.9	6.7
Standard Deviation	2.1	2.1
Min, Max	0.0, 10.0	0.0, 10.0
(Missing)	32 (0.3%)	20 (0.4%)
Current life evaluation		

Table S14b. Weighted summary statistics for outcome variables in Germany

Outcome	Wave 1<br>N = 9,506	Wave 2<br>N = 5,612
Mean	6.7	6.5
Standard Deviation	1.8	1.8
Min, Max	0.0, 10.0	0.0, 10.0
(Missing)	8 (<0.1%)	18 (0.3%)
Future life evaluation		
Mean	7.2	6.9
Standard Deviation	1.9	2.0
Min, Max	0.0, 10.0	0.0, 10.0
(Missing)	17 (0.2%)	40 (0.7%)
Optimism		
Mean	7.5	7.4
Standard Deviation	2.1	2.2
Min, Max	0.0, 10.0	0.0, 10.0
(Missing)	21 (0.2%)	4 (<0.1%)
Freedom to pursue what's important		
Mean	7.5	7.4
Standard Deviation	2.1	2.1
Min, Max	0.0, 10.0	0.0, 10.0
(Missing)	7 (<0.1%)	8 (0.1%)
Inner peace, n (%)		
Always	1,211 (12.7%)	851 (15.2%)
Often	6,332 (66.6%)	3,622 (64.5%)
Rarely	1,800 (18.9%)	1,031 (18.4%)
Never	153 (1.6%)	79 (1.4%)
(Missing)	10 (0.1%)	28 (0.5%)
Life balance, n (%)		
Always	764 (8.0%)	611 (10.9%)
Often	6,509 (68.5%)	3,695 (65.8%)
Rarely	2,034 (21.4%)	1,173 (20.9%)
Never	177 (1.9%)	109 (1.9%)
(Missing)	22 (0.2%)	23 (0.4%)
Sense of mastery, n (%)		
Always	700 (7.4%)	558 (9.9%)
Often	7,246 (76.2%)	4,134 (73.7%)
Rarely	1,355 (14.3%)	809 (14.4%)
Never	172 (1.8%)	87 (1.5%)
(Missing)	33 (0.3%)	23 (0.4%)
Meaningful activities		
Mean	7.3	7.2
Standard Deviation	2.0	2.0
Min, Max	0.0, 10.0	0.0, 10.0
(Missing)	11 (0.1%)	8 (0.2%)
Understanding purpose		
Mean	7.1	7.0
Standard Deviation	2.4	2.4
Min, Max	0.0, 10.0	0.0, 10.0
(Missing)	21 (0.2%)	13 (0.2%)
Self-rated mental health		
Mean	7.0	6.9
Standard Deviation	2.2	2.2
Min, Max	0.0, 10.0	0.0, 10.0

Table S14b. Weighted summary statistics for outcome variables in Germany

**Outcome**	**Wave 1**<br>N = 9,506	**Wave 2**<br>N = 5,612
(Missing)	16 (0.2%)	12 (0.2%)
Traumatic distress, n (%)		
A lot	687 (7.2%)	421 (7.5%)
Some	2,095 (22.0%)	1,133 (20.2%)
Not very much	2,448 (25.8%)	1,578 (28.1%)
Not at all	4,241 (44.6%)	2,455 (43.7%)
(Missing)	34 (0.4%)	25 (0.4%)
Depression symptoms composite, n (%)	1,484 (15.7%)	888 (15.9%)
(Missing)	33 (0.3%)	20 (0.3%)
Depression – feel hopeless, n (%)		
Nearly every day	491 (5.2%)	277 (4.9%)
More than half the days	740 (7.8%)	501 (8.9%)
Several days	2,912 (30.6%)	1,897 (33.8%)
Not at all	5,342 (56.2%)	2,925 (52.1%)
(Missing)	21 (0.2%)	12 (0.2%)
Depression – loss of interest, n (%)		
Nearly every day	471 (5.0%)	240 (4.3%)
More than half the days	791 (8.3%)	505 (9.0%)
Several days	2,518 (26.5%)	1,590 (28.3%)
Not at all	5,712 (60.1%)	3,267 (58.2%)
(Missing)	14 (0.1%)	10 (0.2%)
Anxiety symptoms composite, n (%)	1,814 (19.2%)	1,158 (20.7%)
(Missing)	47 (0.5%)	26 (0.5%)
Anxiety – feel on edge, n (%)		
Nearly every day	547 (5.8%)	298 (5.3%)
More than half the days	826 (8.7%)	635 (11.3%)
Several days	3,743 (39.4%)	2,276 (40.6%)
Not at all	4,363 (45.9%)	2,384 (42.5%)
(Missing)	27 (0.3%)	19 (0.3%)
Anxiety – cannot stop worrying, n (%)		
Nearly every day	700 (7.4%)	371 (6.6%)
More than half the days	853 (9.0%)	655 (11.7%)
Several days	3,056 (32.1%)	1,819 (32.4%)
Not at all	4,875 (51.3%)	2,759 (49.2%)
(Missing)	21 (0.2%)	8 (0.1%)
Suffering, n (%)		
A lot	837 (8.8%)	639 (11.4%)
Some	3,825 (40.2%)	2,255 (40.2%)
Not very much	3,296 (34.7%)	1,874 (33.4%)
Not at all	1,520 (16.0%)	831 (14.8%)
(Missing)	28 (0.3%)	13 (0.2%)
Relationship contentment		
Mean	7.4	7.4
Standard Deviation	2.2	2.2
Min, Max	0.0, 10.0	0.0, 10.0
(Missing)	36 (0.4%)	7 (0.1%)
Relationship satisfaction		
Mean	7.1	7.0
Standard Deviation	2.4	2.4
Min, Max	0.0, 10.0	0.0, 10.0
(Missing)	30 (0.3%)	17 (0.3%)

Table S14b. Weighted summary statistics for outcome variables in Germany

Outcome	Wave 1<br>N = 9,506	Wave 2<br>N = 5,612
Social support		
Mean	8.1	8.0
Standard Deviation	2.1	2.2
Min, Max	0.0, 10.0	0.0, 10.0
(Missing)	4 (<0.1%)	20 (0.4%)
Intimate/close friend, n (%)		
Yes	8,101 (85.2%)	4,783 (85.2%)
No	1,372 (14.4%)	801 (14.3%)
(Missing)	33 (0.3%)	28 (0.5%)
Government approval, n (%)		
Strongly approve	507 (5.3%)	316 (5.6%)
Somewhat approve	2,688 (28.3%)	1,518 (27.1%)
Neither approve nor disapprove	2,903 (30.5%)	1,660 (29.6%)
Somewhat disapprove	2,128 (22.4%)	1,216 (21.7%)
Strongly disapprove	1,241 (13.1%)	883 (15.7%)
(Missing)	39 (0.4%)	17 (0.3%)
Say in government, n (%)		
Agree	4,000 (42.1%)	2,447 (43.6%)
Disagree	3,484 (36.6%)	2,076 (37.0%)
Unsure	2,017 (21.2%)	1,080 (19.2%)
(Missing)	6 (0.1%)	9 (0.2%)
Belonging in country		
Mean	7.0	6.9
Standard Deviation	2.4	2.4
Min, Max	0.0, 10.0	0.0, 10.0
(Missing)	34 (0.4%)	35 (0.6%)
City/place satisfaction, n (%)		
Satisfied	7,624 (80.2%)	4,371 (77.9%)
Dissatisfied	1,092 (11.5%)	734 (13.1%)
Unsure	748 (7.9%)	473 (8.4%)
(Missing)	42 (0.4%)	33 (0.6%)
Trust within country, n (%)		
All people	70 (0.7%)	133 (2.4%)
Most people	2,571 (27.0%)	1,475 (26.3%)
Some people	4,133 (43.5%)	2,412 (43.0%)
Not very many people	2,508 (26.4%)	1,472 (26.2%)
None	193 (2.0%)	97 (1.7%)
(Missing)	31 (0.3%)	24 (0.4%)
Number of children		
Mean	0.4	0.4
Standard Deviation	1.0	0.8
Min, Max	0.0, 64.0	0.0, 7.0
(Missing)	46 (0.5%)	539 (9.6%)
Community participation, n (%)		
More than once a week	970 (10.2%)	621 (11.1%)
Once a week	1,210 (12.7%)	719 (12.8%)
One to three times a month	1,181 (12.4%)	719 (12.8%)
A few times a year	2,019 (21.2%)	1,096 (19.5%)
Never	4,117 (43.3%)	2,445 (43.6%)
(Missing)	10 (0.1%)	11 (0.2%)
Religious attendance, n (%)		

Table S14b. Weighted summary statistics for outcome variables in Germany

**Outcome**	**Wave 1**<br>N = 9,506	**Wave 2**<br>N = 5,612
More than once a week	282 (3.0%)	149 (2.7%)
Once a week	425 (4.5%)	252 (4.5%)
One to three times a month	551 (5.8%)	368 (6.6%)
A few times a year	2,350 (24.7%)	2,117 (37.7%)
Never	5,888 (61.9%)	2,714 (48.4%)
(Missing)	10 (0.1%)	11 (0.2%)
Loneliness		
Mean	3.0	3.1
Standard Deviation	2.6	2.7
Min, Max	0.0, 10.0	0.0, 10.0
(Missing)	16 (0.2%)	12 (0.2%)
Perceived discrimination, n (%)		
Always	225 (2.4%)	133 (2.4%)
Often	1,179 (12.4%)	733 (13.1%)
Rarely	3,690 (38.8%)	2,179 (38.8%)
Never	4,393 (46.2%)	2,556 (45.6%)
(Missing)	19 (0.2%)	11 (0.2%)
Orientation to promote good		
Mean	7.7	7.6
Standard Deviation	1.8	1.8
Min, Max	0.0, 10.0	0.0, 10.0
(Missing)	14 (0.1%)	4 (<0.1%)
Delayed gratification		
Mean	6.9	6.8
Standard Deviation	2.1	2.1
Min, Max	0.0, 10.0	0.0, 10.0
(Missing)	14 (0.1%)	17 (0.3%)
Hope		
Mean	7.7	7.4
Standard Deviation	2.0	2.1
Min, Max	0.0, 10.0	0.0, 10.0
(Missing)	36 (0.4%)	20 (0.4%)
Gratitude		
Mean	7.6	7.5
Standard Deviation	2.2	2.2
Min, Max	0.0, 10.0	0.0, 10.0
(Missing)	53 (0.6%)	27 (0.5%)
Showing love/care		
Mean	7.8	7.6
Standard Deviation	2.0	2.1
Min, Max	0.0, 10.0	0.0, 10.0
(Missing)	7 (<0.1%)	8 (0.1%)
Forgivingness, n (%)		
Always	1,332 (14.0%)	842 (15.0%)
Often	5,742 (60.4%)	3,349 (59.7%)
Rarely	2,170 (22.8%)	1,258 (22.4%)
Never	242 (2.5%)	142 (2.5%)
(Missing)	20 (0.2%)	21 (0.4%)
Charitable giving, n (%)		
Yes	3,406 (35.8%)	1,931 (34.4%)
No	6,076 (63.9%)	3,643 (64.9%)

Table S14b. Weighted summary statistics for outcome variables in Germany

**Outcome**	**Wave 1** N = 9,506	**Wave 2** N = 5,612
(Missing)	24 (0.2%)	37 (0.7%)
Helping strangers, n (%)		
Yes	4,872 (51.3%)	2,714 (48.4%)
No	4,594 (48.3%)	2,871 (51.2%)
(Missing)	40 (0.4%)	26 (0.5%)
Volunteering, n (%)		
Yes	1,989 (20.9%)	1,287 (22.9%)
No	7,494 (78.8%)	4,295 (76.5%)
(Missing)	23 (0.2%)	30 (0.5%)
Self-rated physical health		
Mean	6.6	6.5
Standard Deviation	2.1	2.1
Min, Max	0.0, 10.0	0.0, 10.0
(Missing)	12 (0.1%)	35 (0.6%)
Health problems, n (%)		
Yes	2,766 (29.1%)	1,644 (29.3%)
No	6,674 (70.2%)	3,913 (69.7%)
(Missing)	66 (0.7%)	55 (1.0%)
Pain in past 4 weeks, n (%)		
A lot	977 (10.3%)	677 (12.1%)
Some	2,931 (30.8%)	1,753 (31.2%)
Not very much	2,960 (31.1%)	1,836 (32.7%)
None at all	2,618 (27.5%)	1,333 (23.8%)
(Missing)	19 (0.2%)	13 (0.2%)
Number of cigarettes per day		
Mean	3.6	3.2
Standard Deviation	7.7	7.1
Min, Max	0.0, 97.0	0.0, 71.0
(Missing)	80 (0.8%)	115 (2.0%)
Number of drinks per week		
Mean	3.9	3.9
Standard Deviation	8.9	9.3
Min, Max	0.0, 97.0	0.0, 90.0
(Missing)	80 (0.8%)	125 (2.2%)
Days exercise per week		
Mean	2.4	2.3
Standard Deviation	2.2	2.1
Min, Max	0.0, 7.0	0.0, 7.0
(Missing)	13 (0.1%)	10 (0.2%)
Financial security		
Mean	6.2	6.4
Standard Deviation	3.0	2.9
Min, Max	0.0, 10.0	0.0, 10.0
(Missing)	9 (<0.1%)	6 (0.1%)
Material security		
Mean	6.8	6.8
Standard Deviation	2.8	2.7
Min, Max	0.0, 10.0	0.0, 10.0
(Missing)	8 (<0.1%)	10 (0.2%)
Educational attainment (16+ years), n (%)		
Up to 8	232 (2.4%)	105 (1.9%)

Table S14b. Weighted summary statistics for outcome variables in Germany

Outcome	Wave 1<br>N = 9,506	Wave 2<br>N = 5,612
9-15	6,219 (65.4%)	3,560 (63.4%)
16+	3,043 (32.0%)	1,945 (34.7%)
(Missing)	12 (0.1%)	2 (0.0%)
Currently employed, n (%)		
Employed for an employer	5,023 (52.8%)	2,957 (52.7%)
Self-employed	717 (7.5%)	364 (6.5%)
Retired	2,352 (24.7%)	1,453 (25.9%)
Student	653 (6.9%)	331 (5.9%)
Homemaker	247 (2.6%)	141 (2.5%)
Unemployed and looking for a job	288 (3.0%)	179 (3.2%)
None of these/Other	211 (2.2%)	129 (2.3%)
(Missing)	15 (0.2%)	57 (1.0%)
Financially comfortable/getting by, n (%)		
Living comfortably on present income	2,961 (31.2%)	1,935 (34.5%)
Getting by on present income	4,449 (46.8%)	2,571 (45.8%)
Finding it difficult on present income	1,539 (16.2%)	837 (14.9%)
Finding it very difficult on present income	470 (4.9%)	230 (4.1%)
(Missing)	88 (0.9%)	38 (0.7%)
Own home, n (%)		
Someone in this household owns this home	3,879 (40.8%)	2,243 (40.0%)
Someone in this household rents this home	3,768 (39.6%)	2,323 (41.4%)
Both	693 (7.3%)	403 (7.2%)
Neither	1,075 (11.3%)	598 (10.7%)
Rent	0 (0%)	0 (0%)
Own	0 (0%)	0 (0%)
Something else	0 (0%)	0 (0%)
(Missing)	91 (1.0%)	44 (0.8%)
Religious/spiritual connection, n (%)		
Always	1,130 (11.9%)	776 (13.8%)
Often	2,378 (25.0%)	1,287 (22.9%)
Rarely	3,289 (34.6%)	1,895 (33.8%)
Never	2,700 (28.4%)	1,645 (29.3%)
(Missing)	8 (0.1%)	9 (0.2%)
Belief in life after death, n (%)		
Yes	3,262 (34.3%)	1,905 (34.0%)
No	3,332 (35.1%)	1,997 (35.6%)
Unsure	2,877 (30.3%)	1,697 (30.2%)
(Missing)	34 (0.4%)	12 (0.2%)
Transformative religious experience, n (%)		
Yes	2,000 (21.0%)	1,070 (19.1%)
No	7,484 (78.7%)	4,529 (80.7%)
(Missing)	22 (0.2%)	13 (0.2%)
Religious reading or listening, n (%)		
More than once a day	187 (2.0%)	86 (1.5%)
About once a day	477 (5.0%)	261 (4.6%)
Sometimes	3,181 (33.5%)	1,333 (23.8%)
Never	5,633 (59.3%)	3,887 (69.3%)
(Missing)	27 (0.3%)	44 (0.8%)
Prayer or meditation, n (%)		
More than once a day	525 (5.5%)	266 (4.7%)
About once a day	1,070 (11.3%)	494 (8.8%)

Table S14b. Weighted summary statistics for outcome variables in Germany

**Outcome**	**Wave 1**<br>N = 9,506	**Wave 2**<br>N = 5,612
Sometimes	3,579 (37.7%)	1,597 (28.5%)
Never	4,318 (45.4%)	3,214 (57.3%)
(Missing)	14 (0.1%)	41 (0.7%)
Belief in God/gods/spiritual forces, n (%)		
One God	3,121 (32.8%)	1,834 (32.7%)
More than one god	305 (3.2%)	166 (3.0%)
An impersonal spiritual force	1,815 (19.1%)	1,176 (21.0%)
None of these	2,824 (29.7%)	1,681 (30.0%)
Unsure	1,421 (14.9%)	745 (13.3%)
(Missing)	20 (0.2%)	10 (0.2%)
Religious centrality, n (%)		
Agree	1,680 (17.7%)	953 (17.0%)
Disagree	2,028 (21.3%)	1,153 (20.6%)
Not relevant	4,794 (50.4%)	2,886 (51.4%)
Unsure	960 (10.1%)	602 (10.7%)
(Missing)	44 (0.5%)	17 (0.3%)
Religious/spiritual comfort, n (%)		
Agree	2,510 (26.4%)	1,422 (25.3%)
Disagree	1,697 (17.9%)	943 (16.8%)
Not relevant	4,059 (42.7%)	2,504 (44.6%)
Unsure	1,190 (12.5%)	715 (12.7%)
(Missing)	50 (0.5%)	28 (0.5%)
Feel loved by God, n (%)		
Agree	2,515 (26.5%)	1,400 (25.0%)
Disagree	1,535 (16.1%)	871 (15.5%)
Not relevant	4,174 (43.9%)	2,542 (45.3%)
Unsure	1,228 (12.9%)	745 (13.3%)
(Missing)	55 (0.6%)	53 (1.0%)
Feel punished by God, n (%)		
Agree	621 (6.5%)	313 (5.6%)
Disagree	4,147 (43.6%)	2,460 (43.8%)
Not relevant	3,801 (40.0%)	2,266 (40.4%)
Unsure	901 (9.5%)	542 (9.7%)
(Missing)	36 (0.4%)	30 (0.5%)
Experienced religious criticism, n (%)		
Agree	399 (4.2%)	187 (3.3%)
Disagree	3,827 (40.3%)	2,279 (40.6%)
Not relevant	4,782 (50.3%)	2,870 (51.2%)
Unsure	450 (4.7%)	241 (4.3%)
(Missing)	47 (0.5%)	34 (0.6%)
Faith-sharing, n (%)		
Agree	1,968 (20.7%)	1,079 (19.2%)
Disagree	2,776 (29.2%)	1,617 (28.8%)
Not relevant	4,268 (44.9%)	2,607 (46.5%)
Unsure	456 (4.8%)	281 (5.0%)
(Missing)	38 (0.4%)	28 (0.5%)

Note. N (%); this table is based on non-imputed data. Cumulative percentages for variables may not add up to 100% due to rounding. Wave 1 characteristics weighted using the Gallup provided sampling weight, ANNUAL_WEIGHT_R2; Wave 2 characteristics weighted accounting for attrition by using the adjusted Wave 1 weight, ANNUAL_WEIGHT_R2, multiplied by the created attrition weight to account for dropout, to maintain nationally representative estimates for Wave 2 characteristics.

Table S14c. Unweighted summary statistics for demographic and childhood variables in Germany by retention status

Characteristic	Attritors–Not Observed in Wave 2<br>N = 3,808	Retained–Observed in Wave 2<br>N = 5,773
Forgivingness, n (%)		
Always	549 (14.4%)	792 (13.7%)
Often	2,324 (61.0%)	3,461 (60.0%)
Rarely	826 (21.7%)	1,365 (23.6%)
Never	101 (2.6%)	143 (2.5%)
(Missing)	8 (0.2%)	12 (0.2%)
Year of birth, n (%)		
1943 or earlier (current age: 80+ years)	72 (1.9%)	108 (1.9%)
1943-1953 (current age: 70-79 years)	331 (8.7%)	771 (13.3%)
1953-1963 (current age: 60-69 years)	642 (16.9%)	1,237 (21.4%)
1963-1973 (current age: 50-59 years)	626 (16.4%)	1,140 (19.7%)
1973-1983 (current age: 40-49 years)	597 (15.7%)	910 (15.8%)
1983-1993 (current age: 30-39 years)	611 (16.1%)	802 (13.9%)
1993-1998 (current age: 25-29 years)	367 (9.6%)	410 (7.1%)
1998-2005 (current age: 18-24 years)	561 (14.7%)	396 (6.9%)
(Missing)	0 (0%)	0 (0%)
Age of participant		
Mean	46.1	51.3
Standard Deviation	17.8	16.7
Min, Max	18.0, 93.0	18.0, 99.0
Gender, n (%)		
Male	1,851 (48.6%)	2,809 (48.7%)
Female	1,941 (51.0%)	2,957 (51.2%)
Other	8 (0.2%)	3 (0.1%)
(Missing)	8 (0.2%)	3 (0.1%)
Respondent marital status, n (%)		
Single/Never been married	1,199 (31.5%)	1,494 (25.9%)
Married	1,725 (45.3%)	3,095 (53.6%)
Separated	97 (2.5%)	118 (2.0%)
Divorced	274 (7.2%)	475 (8.2%)
Widowed	130 (3.4%)	271 (4.7%)
Domestic partner	337 (8.8%)	293 (5.1%)
(Missing)	46 (1.2%)	27 (0.5%)
Education (years), n (%)		
Up to 8	124 (3.3%)	107 (1.9%)
9-15	2,584 (67.9%)	3,677 (63.7%)
16+	1,094 (28.7%)	1,983 (34.3%)
(Missing)	6 (0.2%)	6 (0.1%)
Employment status, n (%)		
Employed for an employer	2,019 (53.0%)	3,043 (52.7%)
Self-employed	299 (7.8%)	424 (7.3%)
Retired	828 (21.8%)	1,551 (26.9%)
Student	331 (8.7%)	321 (5.6%)
Homemaker	89 (2.3%)	161 (2.8%)
Unemployed and looking for a job	141 (3.7%)	147 (2.5%)
None of these/Other	97 (2.5%)	115 (2.0%)
(Missing)	4 (0.1%)	11 (0.2%)
Current religious service attendance, n (%)		
More than once a week	147 (3.9%)	134 (2.3%)

Table S14c. Unweighted summary statistics for demographic and childhood variables in Germany by retention status

Characteristic	Attritors–Not Observed in Wave 2<br>N = 3,808	Retained–Observed in Wave 2<br>N = 5,773
Once a week	174 (4.6%)	254 (4.4%)
One to three times a month	227 (6.0%)	328 (5.7%)
A few times a year	895 (23.5%)	1,478 (25.6%)
Never	2,364 (62.1%)	3,571 (61.8%)
(Missing)	2 (0.0%)	9 (0.2%)
Immigration status, n (%)		
Born in this country	3,453 (90.7%)	5,343 (92.5%)
Born in another country	347 (9.1%)	397 (6.9%)
(Missing)	8 (0.2%)	33 (0.6%)
Parental marital status around age 12, n (%)		
Parents were married	2,931 (77.0%)	4,744 (82.2%)
Parents were divorced	419 (11.0%)	516 (8.9%)
Parents were never married	270 (7.1%)	320 (5.5%)
One or both of them had died	107 (2.8%)	139 (2.4%)
Unsure	31 (0.8%)	35 (0.6%)
(Missing)	50 (1.3%)	19 (0.3%)
Religious service attendance around age 12, n (%)		
At least once a week	771 (20.2%)	1,174 (20.3%)
One to three times a month	711 (18.7%)	1,199 (20.8%)
Less than once a month	1,191 (31.3%)	1,733 (30.0%)
Never	1,124 (29.5%)	1,650 (28.6%)
(Missing)	11 (0.3%)	16 (0.3%)
Relationship with mother when growing up, n (%)		
Very good	2,337 (61.4%)	3,207 (55.6%)
Somewhat good	1,116 (29.3%)	1,923 (33.3%)
Somewhat bad	172 (4.5%)	336 (5.8%)
Very bad	75 (2.0%)	111 (1.9%)
(Does not apply)	90 (2.4%)	160 (2.8%)
(Missing)	18 (0.5%)	36 (0.6%)
Relationship with father when growing up, n (%)		
Very good	1,919 (50.4%)	2,782 (48.2%)
Somewhat good	1,172 (30.8%)	1,857 (32.2%)
Somewhat bad	325 (8.5%)	520 (9.0%)
Very bad	160 (4.2%)	227 (3.9%)
(Does not apply)	196 (5.1%)	350 (6.1%)
(Missing)	37 (1.0%)	37 (0.6%)
Felt like an outsider in family when growing up, n (%)		
Yes	483 (12.7%)	650 (11.3%)
No	3,272 (85.9%)	5,038 (87.3%)
(Missing)	53 (1.4%)	85 (1.5%)
Experienced abuse when growing up, n (%)		
Yes	428 (11.2%)	660 (11.4%)
No	3,318 (87.1%)	5,079 (88.0%)
(Missing)	63 (1.6%)	34 (0.6%)
Self-rated health when growing up, n (%)		
Excellent	1,113 (29.2%)	1,566 (27.1%)
Very good	1,370 (36.0%)	2,196 (38.0%)

Table S14c. Unweighted summary statistics for demographic and childhood variables in Germany by retention status

**Characteristic**	**Attritors–Not Observed in Wave 2**<br>N = 3,808	**Retained–Observed in Wave 2**<br>N = 5,773
Good	998 (26.2%)	1,567 (27.1%)
Fair	260 (6.8%)	352 (6.1%)
Poor	61 (1.6%)	72 (1.3%)
(Missing)	6 (0.2%)	20 (0.4%)
Subjective financial status of family growing up, n (%)		
Lived comfortably	1,393 (36.6%)	1,829 (31.7%)
Got by	1,734 (45.5%)	2,807 (48.6%)
Found it difficult	554 (14.6%)	920 (15.9%)
Found it very difficult	120 (3.2%)	198 (3.4%)
(Missing)	7 (0.2%)	20 (0.3%)
Religious affiliation growing up, n (%)		
Christianity	2,113 (55.5%)	3,681 (63.8%)
Taoism	0 (0%)	0 (0%)
Confucianism	0 (0%)	4 (0.1%)
Primal, Animist, or Folk religion	3 (0.1%)	17 (0.3%)
Spiritism	0 (0%)	0 (0%)
Umbanda, Candomblé, and other African-derived religions	0 (0%)	0 (0%)
Chinese folk/traditional religion	0 (0%)	0 (0%)
Islam	214 (5.6%)	143 (2.5%)
Hinduism	4 (0.1%)	11 (0.2%)
Buddhism	18 (0.5%)	8 (0.1%)
Judaism	10 (0.3%)	7 (0.1%)
Sikhism	1 (0.0%)	4 (0.1%)
Baha'i	0 (0%)	2 (0.0%)
Jainism	1 (0.0%)	0 (0%)
Shinto	0 (0%)	0 (0%)
Some other religion	26 (0.7%)	42 (0.7%)
No religion/Atheist/Agnostic	1,379 (36.2%)	1,806 (31.3%)
(Missing)	40 (1.0%)	46 (0.8%)

Note. N (%); this table is based on non-imputed data. Cumulative percentages for variables may not add up to 100% due to rounding.

Table S14d. Unweighted summary statistics for Wave 1 outcome variables in Germany by retention status.

**Outcome**	**Attritors–Not Observed in Wave 2** N = 3,808	**Retained–Observed in Wave 2** N = 5,773
Secure flourishing index		
Mean	6.9	7.1
Standard Deviation	1.4	1.4
Min, Max	0.0, 10.0	0.0, 10.0
(Missing)	49 (1.3%)	117 (2.0%)
Flourishing index		
Mean	7.0	7.2
Standard Deviation	1.3	1.4
Min, Max	0.0, 10.0	0.0, 10.0
(Missing)	46 (1.2%)	114 (2.0%)
Happiness & life satisfaction		
Mean	6.8	7.0
Standard Deviation	2.0	1.9
Min, Max	0.0, 10.0	0.0, 10.0
(Missing)	23 (0.6%)	22 (0.4%)
Physical & mental health		
Mean	6.8	6.9
Standard Deviation	2.0	1.9
Min, Max	0.0, 10.0	0.0, 10.0
(Missing)	10 (0.3%)	16 (0.3%)
Meaning & purpose		
Mean	7.0	7.3
Standard Deviation	1.9	1.8
Min, Max	0.0, 10.0	0.0, 10.0
(Missing)	6 (0.2%)	27 (0.5%)
Character & virtue		
Mean	7.3	7.3
Standard Deviation	1.6	1.6
Min, Max	0.0, 10.0	0.0, 10.0
(Missing)	9 (0.2%)	15 (0.3%)
Close social relationships		
Mean	7.2	7.3
Standard Deviation	2.2	2.1
Min, Max	0.0, 10.0	0.0, 10.0
(Missing)	10 (0.3%)	50 (0.9%)
Financial & material security		
Mean	6.3	6.6
Standard Deviation	2.8	2.6
Min, Max	0.0, 10.0	0.0, 10.0
(Missing)	6 (0.1%)	6 (0.1%)
Happiness		
Mean	6.8	7.0
Standard Deviation	2.0	1.9
Min, Max	0.0, 10.0	0.0, 10.0
(Missing)	5 (0.1%)	10 (0.2%)
Life satisfaction		
Mean	6.8	7.0
Standard Deviation	2.2	2.0
Min, Max	0.0, 10.0	0.0, 10.0

Table S14d. Unweighted summary statistics for Wave 1 outcome variables in Germany by retention status.

Outcome	Attritors–Not Observed in Wave 2<br>N = 3,808	Retained–Observed in Wave 2<br>N = 5,773
(Missing)	19 (0.5%)	12 (0.2%)
Current life evaluation		
Mean	6.8	6.7
Standard Deviation	1.8	1.8
Min, Max	0.0, 10.0	0.0, 10.0
(Missing)	7 (0.2%)	1 (<0.1%)
Future life evaluation		
Mean	7.3	7.1
Standard Deviation	1.9	2.0
Min, Max	0.0, 10.0	0.0, 10.0
(Missing)	9 (0.2%)	8 (0.1%)
Optimism		
Mean	7.5	7.5
Standard Deviation	2.1	2.1
Min, Max	0.0, 10.0	0.0, 10.0
(Missing)	15 (0.4%)	6 (0.1%)
Freedom to pursue what's important		
Mean	7.5	7.6
Standard Deviation	2.2	2.1
Min, Max	0.0, 10.0	0.0, 10.0
(Missing)	3 (<0.1%)	4 (<0.1%)
Inner peace, n (%)		
Always	470 (12.3%)	752 (13.0%)
Often	2,456 (64.5%)	3,933 (68.1%)
Rarely	806 (21.2%)	1,002 (17.4%)
Never	73 (1.9%)	80 (1.4%)
(Missing)	4 (0.1%)	7 (0.1%)
Life balance, n (%)		
Always	266 (7.0%)	507 (8.8%)
Often	2,546 (66.9%)	4,019 (69.6%)
Rarely	894 (23.5%)	1,150 (19.9%)
Never	91 (2.4%)	86 (1.5%)
(Missing)	12 (0.3%)	11 (0.2%)
Sense of mastery, n (%)		
Always	262 (6.9%)	445 (7.7%)
Often	2,828 (74.3%)	4,481 (77.6%)
Rarely	607 (15.9%)	753 (13.1%)
Never	92 (2.4%)	80 (1.4%)
(Missing)	19 (0.5%)	14 (0.2%)
Meaningful activities		
Mean	7.2	7.4
Standard Deviation	2.0	1.9
Min, Max	0.0, 10.0	0.0, 10.0
(Missing)	5 (0.1%)	7 (0.1%)
Understanding purpose		
Mean	6.9	7.2
Standard Deviation	2.4	2.3
Min, Max	0.0, 10.0	0.0, 10.0
(Missing)	1 (<0.1%)	20 (0.4%)

Table S14d. Unweighted summary statistics for Wave 1 outcome variables in Germany by retention status.

**Outcome**	**Attritors–Not Observed in Wave 2** N = 3,808	**Retained–Observed in Wave 2** N = 5,773
Self-rated mental health		
Mean	6.9	7.2
Standard Deviation	2.3	2.2
Min, Max	0.0, 10.0	0.0, 10.0
(Missing)	6 (0.2%)	10 (0.2%)
Traumatic distress, n (%)		
A lot	325 (8.5%)	364 (6.3%)
Some	869 (22.8%)	1,241 (21.5%)
Not very much	995 (26.1%)	1,472 (25.5%)
Not at all	1,606 (42.2%)	2,676 (46.4%)
(Missing)	14 (0.4%)	20 (0.4%)
Depression symptoms composite, n (%)	678 (17.9%)	811 (14.1%)
(Missing)	10 (0.3%)	24 (0.4%)
Depression – feel hopeless, n (%)		
Nearly every day	236 (6.2%)	255 (4.4%)
More than half the days	316 (8.3%)	429 (7.4%)
Several days	1,237 (32.5%)	1,693 (29.3%)
Not at all	2,011 (52.8%)	3,383 (58.6%)
(Missing)	8 (0.2%)	13 (0.2%)
Depression – loss of interest, n (%)		
Nearly every day	235 (6.2%)	236 (4.1%)
More than half the days	350 (9.2%)	444 (7.7%)
Several days	1,072 (28.1%)	1,461 (25.3%)
Not at all	2,149 (56.4%)	3,619 (62.7%)
(Missing)	2 (0.0%)	12 (0.2%)
Anxiety symptoms composite, n (%)	837 (22.1%)	983 (17.1%)
(Missing)	12 (0.3%)	36 (0.6%)
Anxiety – feel on edge, n (%)		
Nearly every day	272 (7.2%)	275 (4.8%)
More than half the days	366 (9.6%)	464 (8.0%)
Several days	1,560 (41.0%)	2,209 (38.3%)
Not at all	1,604 (42.1%)	2,805 (48.6%)
(Missing)	7 (0.2%)	21 (0.4%)
Anxiety – cannot stop worrying, n (%)		
Nearly every day	322 (8.5%)	381 (6.6%)
More than half the days	388 (10.2%)	468 (8.1%)
Several days	1,278 (33.5%)	1,799 (31.2%)
Not at all	1,815 (47.7%)	3,109 (53.9%)
(Missing)	5 (0.1%)	16 (0.3%)
Suffering, n (%)		
A lot	390 (10.2%)	449 (7.8%)
Some	1,527 (40.1%)	2,329 (40.3%)
Not very much	1,336 (35.1%)	1,985 (34.4%)
Not at all	544 (14.3%)	993 (17.2%)
(Missing)	11 (0.3%)	17 (0.3%)
Relationship contentment		
Mean	7.4	7.5
Standard Deviation	2.3	2.2
Min, Max	0.0, 10.0	0.0, 10.0

Table S14d. Unweighted summary statistics for Wave 1 outcome variables in Germany by retention status.

Outcome	Attritors–Not Observed in Wave 2<br>N = 3,808	Retained–Observed in Wave 2<br>N = 5,773
(Missing)	8 (0.2%)	29 (0.5%)
Relationship satisfaction		
Mean	7.0	7.1
Standard Deviation	2.4	2.3
Min, Max	0.0, 10.0	0.0, 10.0
(Missing)	2 (<0.1%)	29 (0.5%)
Social support		
Mean	8.0	8.1
Standard Deviation	2.2	2.1
Min, Max	0.0, 10.0	0.0, 10.0
(Missing)	1 (<0.1%)	3 (<0.1%)
Intimate/close friend, n (%)		
Yes	3,256 (85.5%)	4,908 (85.0%)
No	538 (14.1%)	846 (14.6%)
(Missing)	14 (0.4%)	19 (0.3%)
Government approval, n (%)		
Strongly approve	224 (5.9%)	286 (4.9%)
Somewhat approve	1,048 (27.5%)	1,664 (28.8%)
Neither approve nor disapprove	1,213 (31.8%)	1,710 (29.6%)
Somewhat disapprove	840 (22.0%)	1,306 (22.6%)
Strongly disapprove	468 (12.3%)	785 (13.6%)
(Missing)	16 (0.4%)	22 (0.4%)
Say in government, n (%)		
Agree	1,621 (42.6%)	2,410 (41.7%)
Disagree	1,359 (35.7%)	2,155 (37.3%)
Unsure	827 (21.7%)	1,204 (20.9%)
(Missing)	1 (0.0%)	5 (0.1%)
Belonging in country		
Mean	6.8	7.1
Standard Deviation	2.5	2.3
Min, Max	0.0, 10.0	0.0, 10.0
(Missing)	16 (0.4%)	18 (0.3%)
City/place satisfaction, n (%)		
Satisfied	3,033 (79.6%)	4,653 (80.6%)
Dissatisfied	450 (11.8%)	650 (11.3%)
Unsure	310 (8.1%)	443 (7.7%)
(Missing)	15 (0.4%)	28 (0.5%)
Trust within country, n (%)		
All people	47 (1.2%)	22 (0.4%)
Most people	982 (25.8%)	1,614 (28.0%)
Some people	1,645 (43.2%)	2,522 (43.7%)
Not very many people	1,037 (27.2%)	1,488 (25.8%)
None	88 (2.3%)	106 (1.8%)
(Missing)	10 (0.3%)	21 (0.4%)
Number of children		
Mean	0.4	0.4
Standard Deviation	1.0	0.9
Min, Max	0.0, 15.0	0.0, 64.0
(Missing)	18 (0.5%)	29 (0.5%)

Table S14d. Unweighted summary statistics for Wave 1 outcome variables in Germany by retention status.

**Outcome**	**Attritors–Not Observed in Wave 2** N = 3,808	**Retained–Observed in Wave 2** N = 5,773
Community participation, n (%)		
More than once a week	410 (10.8%)	566 (9.8%)
Once a week	480 (12.6%)	740 (12.8%)
One to three times a month	533 (14.0%)	653 (11.3%)
A few times a year	812 (21.3%)	1,223 (21.2%)
Never	1,572 (41.3%)	2,583 (44.7%)
(Missing)	2 (0.0%)	8 (0.1%)
Religious attendance, n (%)		
More than once a week	147 (3.9%)	134 (2.3%)
Once a week	174 (4.6%)	254 (4.4%)
One to three times a month	227 (6.0%)	328 (5.7%)
A few times a year	895 (23.5%)	1,478 (25.6%)
Never	2,364 (62.1%)	3,571 (61.8%)
(Missing)	2 (0.0%)	9 (0.2%)
Loneliness		
Mean	3.2	2.9
Standard Deviation	2.7	2.6
Min, Max	0.0, 10.0	0.0, 10.0
(Missing)	7 (0.2%)	10 (0.2%)
Perceived discrimination, n (%)		
Always	95 (2.5%)	132 (2.3%)
Often	543 (14.3%)	640 (11.1%)
Rarely	1,523 (40.0%)	2,192 (38.0%)
Never	1,640 (43.1%)	2,797 (48.4%)
(Missing)	8 (0.2%)	12 (0.2%)
Orientation to promote good		
Mean	7.6	7.7
Standard Deviation	1.9	1.8
Min, Max	0.0, 10.0	0.0, 10.0
(Missing)	5 (0.1%)	9 (0.2%)
Delayed gratification		
Mean	7.0	6.9
Standard Deviation	2.2	2.1
Min, Max	0.0, 10.0	0.0, 10.0
(Missing)	8 (0.2%)	6 (0.1%)
Hope		
Mean	7.6	7.7
Standard Deviation	1.9	2.0
Min, Max	0.0, 10.0	0.0, 10.0
(Missing)	16 (0.4%)	20 (0.3%)
Gratitude		
Mean	7.6	7.6
Standard Deviation	2.2	2.2
Min, Max	0.0, 10.0	0.0, 10.0
(Missing)	20 (0.5%)	33 (0.6%)
Showing love/care		
Mean	7.7	7.9
Standard Deviation	2.1	1.9
Min, Max	0.0, 10.0	0.0, 10.0

Table S14d. Unweighted summary statistics for Wave 1 outcome variables in Germany by retention status.

**Outcome**	**Attritors–Not Observed in Wave 2** N = 3,808	**Retained–Observed in Wave 2** N = 5,773
(Missing)	1 (<0.1%)	6 (0.1%)
Forgivingness, n (%)		
Always	549 (14.4%)	792 (13.7%)
Often	2,324 (61.0%)	3,461 (60.0%)
Rarely	826 (21.7%)	1,365 (23.6%)
Never	101 (2.6%)	143 (2.5%)
(Missing)	8 (0.2%)	12 (0.2%)
Charitable giving, n (%)		
Yes	1,452 (38.1%)	1,975 (34.2%)
No	2,353 (61.8%)	3,777 (65.4%)
(Missing)	3 (0.1%)	21 (0.4%)
Helping strangers, n (%)		
Yes	2,093 (55.0%)	2,807 (48.6%)
No	1,705 (44.8%)	2,936 (50.9%)
(Missing)	11 (0.3%)	30 (0.5%)
Volunteering, n (%)		
Yes	795 (20.9%)	1,210 (21.0%)
No	3,008 (79.0%)	4,545 (78.7%)
(Missing)	5 (0.1%)	18 (0.3%)
Self-rated physical health		
Mean	6.7	6.6
Standard Deviation	2.1	2.1
Min, Max	0.0, 10.0	0.0, 10.0
(Missing)	6 (0.2%)	6 (<0.1%)
Health problems, n (%)		
Yes	1,097 (28.8%)	1,691 (29.3%)
No	2,667 (70.0%)	4,061 (70.3%)
(Missing)	44 (1.2%)	21 (0.4%)
Pain in past 4 weeks, n (%)		
A lot	443 (11.6%)	538 (9.3%)
Some	1,165 (30.6%)	1,790 (31.0%)
Not very much	1,119 (29.4%)	1,869 (32.4%)
None at all	1,070 (28.1%)	1,568 (27.2%)
(Missing)	11 (0.3%)	8 (0.1%)
Number of cigarettes per day		
Mean	4.2	3.3
Standard Deviation	8.5	7.1
Min, Max	0.0, 97.0	0.0, 90.0
(Missing)	29 (0.8%)	52 (0.9%)
Number of drinks per week		
Mean	4.3	3.6
Standard Deviation	10.4	7.6
Min, Max	0.0, 97.0	0.0, 90.0
(Missing)	34 (0.9%)	46 (0.8%)
Days exercise per week		
Mean	2.5	2.3
Standard Deviation	2.2	2.1
Min, Max	0.0, 7.0	0.0, 7.0
(Missing)	6 (0.2%)	7 (0.1%)

Table S14d. Unweighted summary statistics for Wave 1 outcome variables in Germany by retention status.

**Outcome**	**Attritors–Not Observed in Wave 2** N = 3,808	**Retained–Observed in Wave 2** N = 5,773
Financial security		
Mean	6.0	6.4
Standard Deviation	3.1	2.9
Min, Max	0.0, 10.0	0.0, 10.0
(Missing)	4 (0.1%)	5 (<0.1%)
Material security		
Mean	6.6	6.9
Standard Deviation	2.9	2.7
Min, Max	0.0, 10.0	0.0, 10.0
(Missing)	3 (<0.1%)	5 (<0.1%)
Educational attainment (16+ years), n (%)		
Up to 8	124 (3.3%)	107 (1.9%)
9-15	2,584 (67.9%)	3,677 (63.7%)
16+	1,094 (28.7%)	1,983 (34.3%)
(Missing)	6 (0.2%)	6 (0.1%)
Currently employed, n (%)		
Employed for an employer	2,019 (53.0%)	3,043 (52.7%)
Self-employed	299 (7.8%)	424 (7.3%)
Retired	828 (21.8%)	1,551 (26.9%)
Student	331 (8.7%)	321 (5.6%)
Homemaker	89 (2.3%)	161 (2.8%)
Unemployed and looking for a job	141 (3.7%)	147 (2.5%)
None of these/Other	97 (2.5%)	115 (2.0%)
(Missing)	4 (0.1%)	11 (0.2%)
Financially comfortable/getting by, n (%)		
Living comfortably on present income	1,204 (31.6%)	1,780 (30.8%)
Getting by on present income	1,696 (44.5%)	2,794 (48.4%)
Finding it difficult on present income	650 (17.1%)	899 (15.6%)
Finding it very difficult on present income	199 (5.2%)	274 (4.7%)
(Missing)	60 (1.6%)	26 (0.5%)
Own home, n (%)		
Someone in this household owns this home	1,523 (40.0%)	2,389 (41.4%)
Someone in this household rents this home	1,517 (39.8%)	2,281 (39.5%)
Both	272 (7.1%)	427 (7.4%)
Neither	434 (11.4%)	649 (11.2%)
Rent	0 (0%)	0 (0%)
Own	0 (0%)	0 (0%)
Something else	0 (0%)	0 (0%)
(Missing)	63 (1.6%)	27 (0.5%)
Religious/spiritual connection, n (%)		
Always	443 (11.6%)	697 (12.1%)
Often	929 (24.4%)	1,469 (25.5%)
Rarely	1,325 (34.8%)	1,989 (34.5%)
Never	1,108 (29.1%)	1,612 (27.9%)
(Missing)	3 (0.1%)	5 (0.1%)
Belief in life after death, n (%)		
Yes	1,337 (35.1%)	1,949 (33.8%)
No	1,307 (34.3%)	2,054 (35.6%)
Unsure	1,153 (30.3%)	1,747 (30.3%)

Table S14d. Unweighted summary statistics for Wave 1 outcome variables in Germany by retention status.

**Outcome**	**Attritors–Not Observed in Wave 2** N = 3,808	**Retained–Observed in Wave 2** N = 5,773
(Missing)	12 (0.3%)	23 (0.4%)
Transformative religious experience, n (%)		
Yes	869 (22.8%)	1,143 (19.8%)
No	2,933 (77.0%)	4,615 (79.9%)
(Missing)	7 (0.2%)	15 (0.3%)
Religious reading or listening, n (%)		
More than once a day	73 (1.9%)	116 (2.0%)
About once a day	220 (5.8%)	259 (4.5%)
Sometimes	1,256 (33.0%)	1,952 (33.8%)
Never	2,251 (59.1%)	3,427 (59.4%)
(Missing)	9 (0.2%)	19 (0.3%)
Prayer or meditation, n (%)		
More than once a day	212 (5.6%)	317 (5.5%)
About once a day	424 (11.1%)	654 (11.3%)
Sometimes	1,427 (37.5%)	2,181 (37.8%)
Never	1,738 (45.6%)	2,614 (45.3%)
(Missing)	7 (0.2%)	7 (0.1%)
Belief in God/gods/spiritual forces, n (%)		
One God	1,204 (31.6%)	1,945 (33.7%)
More than one god	140 (3.7%)	166 (2.9%)
An impersonal spiritual force	751 (19.7%)	1,077 (18.7%)
None of these	1,137 (29.9%)	1,709 (29.6%)
Unsure	564 (14.8%)	868 (15.0%)
(Missing)	12 (0.3%)	8 (0.1%)
Religious centrality, n (%)		
Agree	665 (17.5%)	1,029 (17.8%)
Disagree	813 (21.3%)	1,231 (21.3%)
Not relevant	1,968 (51.7%)	2,861 (49.6%)
Unsure	351 (9.2%)	619 (10.7%)
(Missing)	12 (0.3%)	32 (0.6%)
Religious/spiritual comfort, n (%)		
Agree	1,041 (27.3%)	1,485 (25.7%)
Disagree	625 (16.4%)	1,090 (18.9%)
Not relevant	1,633 (42.9%)	2,458 (42.6%)
Unsure	488 (12.8%)	710 (12.3%)
(Missing)	20 (0.5%)	30 (0.5%)
Feel loved by God, n (%)		
Agree	955 (25.1%)	1,584 (27.4%)
Disagree	611 (16.0%)	936 (16.2%)
Not relevant	1,721 (45.2%)	2,482 (43.0%)
Unsure	497 (13.1%)	740 (12.8%)
(Missing)	24 (0.6%)	31 (0.5%)
Feel punished by God, n (%)		
Agree	256 (6.7%)	370 (6.4%)
Disagree	1,611 (42.3%)	2,572 (44.6%)
Not relevant	1,553 (40.8%)	2,275 (39.4%)
Unsure	372 (9.8%)	536 (9.3%)
(Missing)	16 (0.4%)	20 (0.3%)
Experienced religious criticism, n (%)		

Table S14d. Unweighted summary statistics for Wave 1 outcome variables in Germany by retention status.

Outcome	Attritors–Not Observed in Wave 2<br>N = 3,808	Retained–Observed in Wave 2<br>N = 5,773
Agree	177 (4.6%)	224 (3.9%)
Disagree	1,447 (38.0%)	2,418 (41.9%)
Not relevant	1,967 (51.6%)	2,850 (49.4%)
Unsure	198 (5.2%)	254 (4.4%)
(Missing)	19 (0.5%)	28 (0.5%)
Faith-sharing, n (%)		
Agree	830 (21.8%)	1,150 (19.9%)
Disagree	1,050 (27.6%)	1,752 (30.3%)
Not relevant	1,718 (45.1%)	2,583 (44.7%)
Unsure	195 (5.1%)	264 (4.6%)
(Missing)	14 (0.4%)	24 (0.4%)

Note. N (%); this table is based on non-imputed data. Cumulative percentages for variables may not add up to 100% due to rounding.

Table S14e. Summary of fitted attrition model in Germany

Characteristic	Odds Ratio	95% CI	p-value
**ANNUAL_WEIGHT_R2**	0.98	0.92, 1.04	0.492
**Recruitment Survey Mode**			
CAWI	—	—	
CATI	0.51	0.45, 0.58	3.51e-26
**Happiness & life satisfaction**	0.97	0.89, 1.04	0.376
**Physical & mental health**	1.04	0.97, 1.12	0.256
**Meaning & purpose**	1.08	1.00, 1.17	0.045
**Character & virtue**	0.95	0.89, 1.01	0.098
**Close social relationships**	0.97	0.91, 1.04	0.362
**Financial & material security**	1.19	1.11, 1.28	1.01e-06
**Extraversion**	0.95	0.90, 1.01	0.114
**Openness to experience**	0.91	0.85, 0.96	0.002
**Agreeableness**	1.07	1.00, 1.13	0.034
**Conscientiousness**	1.17	1.10, 1.24	7.04e-07
**Neuroticism**	0.95	0.89, 1.01	0.088
**Depression symptoms composite**	0.97	0.90, 1.05	0.489
**Anxiety symptoms composite**	0.99	0.92, 1.06	0.702
**Loneliness**	0.98	0.91, 1.04	0.474
**Days exercise per week**	0.91	0.86, 0.96	9.03e-04
**Year of birth (age group)**			
1953-1963 (current age: 60-69 years)	—	—	
1983-1993 (current age: 30-39 years)	0.69	0.56, 0.85	5.07e-04
1963-1973 (current age: 50-59 years)	0.94	0.77, 1.15	0.537
1973-1983 (current age: 40-49 years)	0.83	0.67, 1.02	0.076
1943-1953 (current age: 70-79 years)	1.17	0.94, 1.46	0.155
1993-1998 (current age: 25-29 years)	0.53	0.41, 0.69	1.80e-06
1998-2005 (current age: 18-24 years)	0.33	0.24, 0.45	2.54e-12
1943 or earlier (current age: 80+ years)	0.72	0.48, 1.06	0.094
**Gender of respondent**			
Male	—	—	
Female	0.98	0.88, 1.10	0.779
Other	0.46	0.09, 2.28	0.340
**Marital status**			
Married	—	—	
Single/Never been married	0.98	0.85, 1.13	0.778
Divorced	0.94	0.77, 1.15	0.568
Domestic partner	0.63	0.50, 0.81	2.16e-04
Widowed	1.26	0.95, 1.67	0.108
Separated	0.93	0.65, 1.33	0.683
**Employment status**			
Employed for an employer	—	—	
Retired	0.86	0.71, 1.04	0.126
Self-employed	0.95	0.77, 1.17	0.634
Student	1.25	0.92, 1.70	0.157
Unemployed and looking for a job	0.81	0.59, 1.12	0.201
Homemaker	1.08	0.77, 1.51	0.656
None of these/Other	1.04	0.70, 1.53	0.857
**Religious attendance**			
Never	—	—	
A few times a year	0.99	0.87, 1.13	0.912
One to three times a month	0.93	0.72, 1.18	0.534
Once a week	0.90	0.69, 1.19	0.468

Table S14e. Summary of fitted attrition model in Germany

**Characteristic**	**Odds Ratio**	**95% CI**	**p-value**
More than once a week	0.67	0.49, 0.93	0.015
**Educational attainment (16+ years)**			
9-15	—	—	
16+	1.27	1.13, 1.44	1.40e-04
Up to 8	0.61	0.44, 0.85	0.004
**Born in This country**			
Born in this country	—	—	
Born in another country	0.83	0.66, 1.04	0.108
**Urbanicity**			
A small town or village	—	—	
A large city	0.99	0.86, 1.13	0.843
A rural area or on a farm	0.87	0.74, 1.02	0.079
A suburb of a large city	1.03	0.84, 1.27	0.753
**Monthly household income**			
Germany: 501 - 1,500 euros	—	—	
Germany: 4,001 - 5,000 euros	1.01	0.77, 1.31	0.951
Germany: 1,501 - 2,000 euros	0.81	0.63, 1.04	0.103
Germany: 3,001 - 3,500 euros	0.85	0.66, 1.11	0.228
Germany: 2,501 - 3,000 euros	0.97	0.75, 1.26	0.803
Germany: 2,001 - 2,500 euros	1.04	0.80, 1.34	0.784
Germany: 5,001 - 6,000 euros	0.85	0.64, 1.12	0.254
Germany: 3,501 - 4,000 euros	0.83	0.63, 1.10	0.191
Germany: 6,001 - 7,500 euros	0.60	0.45, 0.79	3.93e-04
Germany: 7,501 - 10,000 euros	0.59	0.44, 0.80	6.03e-04
Germany: More than 10,000 euros	0.64	0.45, 0.92	0.014
(None/No household income)	0.79	0.54, 1.14	0.210
Germany: 500 euros or less	0.82	0.54, 1.23	0.340

Abbreviations: CI = Confidence Interval, OR = Odds Ratio

Notes. N=9506; attrition weights were estimated using the 'survey::svyglm(family=quasibinomial('logit'))' function. All continuous predictors were standardized and all categorical predictors used the most common category as the reference group. Reported p-values are based on the fitted regression model and no adjustments for multiple testing were done within this table.

Table S14f. Summary of principal components in Germany

PC	Percent Explained by each PC	Cumulative Percent Explained
1	34.40	34.40
2	5.98	40.38
3	4.89	45.27
4	2.19	47.46
5	1.97	49.43
6	1.77	51.19
**7**	**1.55**	**52.74**
8	1.45	54.19
9	1.41	55.60
10	1.33	56.93
11	1.29	58.22
12	1.27	59.49
13	1.26	60.75
14	1.19	61.94
15	1.12	63.06
16	1.10	64.16
17	1.08	65.24
18	1.04	66.28
19	1.02	67.30
20	0.99	68.28

Notes. N=9506; PCA was conducted using ’survey::svyprcomp(.)’ function using all available contemporaneous exposures at wave 1. All PCs were standardized prior to being used as predictors. The bolded row represented the number of retained components for analysis was 7.

Table S14g. Associations of forgivingness with adult well-being and other outcomes at Wave 2 in Germany

	Model 1: Demographic and Childhood Variables as Covariates					Model 2: Demographic, Childhood, and Other Wave 1 Confounding Variables (Via Principal Components) as Covariates				
Outcome	RR	ES	95% CI	SE	p-value	RR	ES	95% CI	SE	p-value
Human Flourishing										
Secure flourishing index		0.07	(0.05,0.10)	0.01	1.76e-08***		0.01	(-0.01,0.03)	0.01	0.176
Flourishing index		0.08	(0.06,0.11)	0.01	7.31e-10***		0.01	(-0.01,0.03)	0.01	0.178
Happiness & life satisfaction		0.04	(0.02,0.07)	0.01	1.3e-03**		-0.00	(-0.02,0.02)	0.01	0.962
Physical & mental health		0.04	(0.01,0.06)	0.01	0.007*		0.00	(-0.02,0.02)	0.01	0.977
Meaning & purpose		0.06	(0.03,0.09)	0.01	7.01e-05***		-0.00	(-0.03,0.02)	0.01	0.914
Character & virtue		0.10	(0.07,0.14)	0.02	2.45e-10***		0.05	(0.02,0.08)	0.02	1.57e-03**
Close social relationships		0.05	(0.03,0.08)	0.01	1.37e-04***		0.01	(-0.02,0.03)	0.01	0.661
Financial & material security		0.02	(-0.01,0.05)	0.01	0.153		0.01	(-0.02,0.03)	0.01	0.588
Psychological Well-Being										
Happiness (a)		0.04	(0.01,0.07)	0.01	0.006*		-0.00	(-0.03,0.03)	0.01	0.954
Life satisfaction (a)		0.04	(0.01,0.07)	0.01	0.010*		-0.00	(-0.03,0.03)	0.01	0.986
Current life evaluation		0.03	(0.00,0.06)	0.02	0.048*		0.00	(-0.03,0.03)	0.02	0.779
Future life evaluation		0.04	(0.01,0.07)	0.02	0.016*		0.00	(-0.02,0.03)	0.01	0.814
Optimism		0.08	(0.05,0.11)	0.01	2.68e-07***		0.03	(0.01,0.06)	0.01	0.018*
Freedom to pursue what's important		0.03	(-0.00,0.06)	0.02	0.075		-0.00	(-0.04,0.03)	0.02	0.773
Inner peace	1.02		(1.01,1.03)	0.01	0.005*	1.01		(0.99,1.02)	0.01	0.415
Life balance	1.01		(1.00,1.03)	0.01	0.028*	1.00		(0.99,1.01)	0.01	0.980
Sense of mastery	1.01		(1.00,1.02)	0.01	0.019*	1.00		(0.99,1.01)	0.01	0.891
Meaningful activities (c)		0.04	(0.01,0.07)	0.01	0.014*		-0.01	(-0.04,0.02)	0.01	0.429
Understanding purpose (c)		0.06	(0.03,0.09)	0.02	1.3e-04***		0.01	(-0.02,0.04)	0.01	0.657
Self-rated mental health (b)		0.04	(0.01,0.08)	0.02	0.005*		0.01	(-0.02,0.04)	0.01	0.526
Psychological Distress										
Traumatic distress	1.00		(0.98,1.01)	0.01	0.639	0.99		(0.98,1.01)	0.01	0.433
Depression symptoms composite	1.00		(0.98,1.01)	0.01	0.458	1.00		(0.99,1.01)	0.01	0.943
Depression – feel hopeless	1.00		(0.99,1.01)	0.01	0.573	1.00		(0.99,1.01)	0.01	0.892
Depression – loss of interest	1.00		(0.99,1.01)	0.01	0.720	1.00		(0.99,1.01)	0.01	0.745
Anxiety symptoms composite	0.99		(0.98,1.00)	0.01	0.114	1.00		(0.99,1.01)	0.01	0.580
Anxiety – feel on edge	0.99		(0.98,1.00)	0.01	0.130	1.00		(0.99,1.01)	0.01	0.460
Anxiety – cannot stop worrying	0.99		(0.98,1.01)	0.01	0.398	1.00		(0.99,1.01)	0.01	0.889
Suffering	1.00		(0.98,1.01)	0.01	0.730	1.00		(0.99,1.02)	0.01	0.586
Social Well-Being										
Relationship contentment (e)		0.04	(0.02,0.07)	0.02	3.24e-03**		-0.00	(-0.03,0.03)	0.01	0.997
Relationship satisfaction (e)		0.05	(0.02,0.08)	0.02	8.21e-04**		0.01	(-0.02,0.04)	0.01	0.498
Social support		0.10	(0.06,0.14)	0.02	3.12e-06***		0.07	(0.03,0.10)	0.02	7.22e-04**
Intimate/close friend	1.03		(1.02,1.04)	0.01	1.73e-05***	1.02		(1.01,1.04)	0.01	1.31e-03**
Government approval	1.02		(1.00,1.03)	0.01	0.019*	1.01		(1.00,1.03)	0.01	0.109
Say in government	1.01		(1.00,1.03)	0.01	0.085	1.00		(0.99,1.02)	0.01	0.556
Belonging in country		0.04	(0.01,0.07)	0.02	0.020*		0.01	(-0.02,0.04)	0.01	0.509
City/place satisfaction	1.02		(1.00,1.03)	0.01	0.013*	1.01		(1.00,1.02)	0.01	0.115
Trust within country	1.03		(1.02,1.05)	0.01	1.56e-05***	1.03		(1.02,1.04)	0.01	9.95e-05***
Social Participation										
Ever been married	1.00		(0.99,1.01)	0.01	0.772	1.00		(0.99,1.01)	0.01	0.887
Currently divorced	1.00		(1.00,1.01)	0.00	0.433	1.00		(1.00,1.01)	0.00	0.530
Number of children		0.02	(-0.01,0.05)	0.01	0.132		0.01	(-0.02,0.03)	0.01	0.660
Weekly+ community participation	1.00		(0.99,1.02)	0.01	0.722	1.00		(0.99,1.01)	0.01	0.845
Weekly+ religious attendance	1.00		(1.00,1.01)	0.00	0.509	1.00		(0.99,1.01)	0.00	0.825
Social Distress										
Loneliness		-0.04	(-0.08,-0.01)	0.02	0.015*		-0.01	(-0.04,0.03)	0.02	0.692
Perceived discrimination	1.00		(0.99,1.01)	0.01	0.470	0.99		(0.98,1.00)	0.01	0.186
Character & Prosocial Behavior										
Orientation to promote good (d)		0.11	(0.07,0.14)	0.02	8.38e-10***		0.05	(0.02,0.08)	0.02	1.02e-03**
Delayed gratification (d)		0.06	(0.03,0.10)	0.02	2.26e-04***		0.03	(-0.01,0.06)	0.02	0.119
Hope		0.09	(0.06,0.12)	0.02	1.22e-08***		0.04	(0.01,0.07)	0.01	3.24e-03**
Gratitude		0.10	(0.07,0.13)	0.02	3.64e-10***		0.04	(0.02,0.07)	0.01	1.38e-03**
Showing love/care		0.08	(0.05,0.11)	0.02	4e-06***		0.04	(0.01,0.07)	0.02	0.010*
Forgivingness	1.15		(1.13,1.17)	0.01	2.22e-16***	1.14		(1.13,1.16)	0.01	2.22e-16***
Charitable giving	1.02		(1.00,1.03)	0.01	0.012*	1.01		(0.99,1.02)	0.01	0.396
Helping strangers	1.04		(1.03,1.06)	0.01	3.04e-08***	1.03		(1.01,1.04)	0.01	2.42e-04***
Volunteering	1.01		(1.00,1.02)	0.01	0.258	1.00		(0.99,1.01)	0.01	0.647
Physical Health & Health Behavior										
Self-rated physical health (b)		0.02	(-0.01,0.05)	0.01	0.230		-0.01	(-0.04,0.02)	0.01	0.489
Health problems	1.00		(0.98,1.01)	0.01	0.567	1.00		(0.99,1.01)	0.01	0.975
Pain in past 4 weeks	1.00		(0.98,1.01)	0.01	0.797	1.00		(0.99,1.02)	0.01	0.881
Daily smoker	1.00		(0.99,1.02)	0.01	0.673	0.99		(0.98,1.01)	0.01	0.431
Number of drinks per week		-0.02	(-0.05,0.01)	0.02	0.167		-0.02	(-0.05,0.02)	0.02	0.376
Days exercise per week		0.02	(-0.01,0.05)	0.02	0.108		0.01	(-0.02,0.03)	0.02	0.741
Socioeconomic Outcomes										
Financial security (f)		0.00	(-0.02,0.03)	0.02	0.745		-0.01	(-0.03,0.02)	0.01	0.683
Material security (f)		0.03	(0.00,0.06)	0.02	0.032*		0.02	(-0.01,0.05)	0.02	0.192
Educational attainment (16+ years)	1.00		(0.99,1.01)	0.00	0.969	1.00		(0.99,1.00)	0.00	0.771
Currently employed	1.00		(0.99,1.01)	0.00	0.970	1.00		(0.99,1.01)	0.00	0.818
Financially comfortable/getting by	1.00		(0.99,1.02)	0.01	0.651	1.00		(0.99,1.01)	0.01	0.940
Own home	1.00		(0.98,1.01)	0.01	0.566	0.99		(0.98,1.01)	0.01	0.505
Income – top quintile	1.00		(0.98,1.01)	0.01	0.505	1.00		(0.99,1.01)	0.01	0.635
Religion & Spirituality										
Religious/spiritual connection	1.02		(1.01,1.04)	0.01	1.7e-03**	1.00		(0.99,1.01)	0.01	0.975
Belief in life after death	1.01		(1.00,1.03)	0.01	0.094	0.99		(0.98,1.01)	0.01	0.330
Transformative religious experience	1.01		(0.99,1.02)	0.01	0.264	0.99		(0.98,1.00)	0.01	0.154
Religious reading or listening	1.01		(1.00,1.01)	0.00	0.029*	1.00		(0.99,1.00)	0.00	0.810
Prayer or meditation	1.00		(0.99,1.01)	0.00	0.442	0.99		(0.98,1.00)	0.00	0.015*
Belief in God/gods/spiritual forces	1.01		(0.99,1.02)	0.01	0.494	0.99		(0.97,1.00)	0.01	0.096
Religious centrality	1.01		(1.00,1.02)	0.01	0.020*	1.00		(0.99,1.01)	0.01	0.466
Religious/spiritual comfort	1.02		(1.00,1.03)	0.01	0.009*	1.00		(0.99,1.01)	0.01	0.659
Feel loved by God	1.02		(1.01,1.03)	0.01	2.89e-03**	1.00		(0.99,1.01)	0.01	0.724
Feel punished by God	1.00		(0.99,1.01)	0.00	0.678	0.99		(0.99,1.00)	0.00	0.107
Experienced religious criticism	1.00		(1.00,1.01)	0.00	0.394	1.00		(0.99,1.00)	0.00	0.494
Faith-sharing	1.02		(1.01,1.03)	0.01	5.87e-04***	1.01		(0.99,1.02)	0.01	0.353

Notes. N=9506; Reference for focal predictor: never/rarely. RR, risk-ratio, null effect is 1.00; ES, effect size measure for standardized regression coefficient, null effect is 0.00; SE, standard error, the SE reported for binary/Likert-type outcomes where risk-ratios are on the log(RR) scale; CI, confidence interval; p-value, a Wald-type test of the null hypothesis that the effect of the focal predictor is zero; (a) item part of the Happiness & Life Satisfaction domain of the Secure Flourishing Index; (b) item part of the Physical & Mental Health domain of the Secure Flourishing Index; (c) item part of the Meaning & Purpose domain of the Secure Flourishing Index; (d) item part of the Character & Virtue domain of the Secure Flourishing Index; (e) item part of the Subjective Social Connectedness domain of the Secure Flourishing Index; (f) item part of the Financial & Material Security domain of the Secure Flourishing Index.

Multiple imputation was performed to impute missing data on the covariates, exposure, and outcomes. All models controlled for sociodemographic and childhood factors assessed at Wave 1. For Model 2 with PC (principal components), the first seven principal components of the entire set of contemporaneous confounders assessed at Wave 1 were included as additional covariates of the outcomes at Wave 2.

An outcome-wide analytic approach was used, and a separate model was run for each outcome. A different type of model was run depending on the nature of the outcome: (1) for each binary outcome, a weighted generalized linear model (with a log link and Poisson distribution) was used to estimate an RR; and (2) for each continuous outcome, a weighted linear regression model was used to estimate a ES. All effect sizes were standardized. For continuous outcomes, the ES represents the change in SD on the outcome between the lower and upper categories of the binary focal predictor. For binary outcomes, the RR represents the change in risk of being in the upper category compared to the lower category between the lower and upper categories of the binary focal predictor.

P-value significance thresholds: $p < 0.05$*, $p < 0.005$**, (Bonferroni) $p < 6.41e-04$***, correction for multiple testing using Bonferroni adjusted significant threshold.

Table S14h. Associations of forgivingness with adult well-being and other outcomes at Wave 2 in Germany using complete-case analyses with attrition weights.

	Model 1: Demographic and Childhood Variables as Covariates					Model 2: Demographic, Childhood, and Other Wave 1 Confounding Variables (Via Principal Components) as Covariates				
Outcome	RR	ES	95% CI	SE	p-value	RR	ES	95% CI	SE	p-value
Human Flourishing										
Secure flourishing index		0.09	(0.05,0.12)	0.02	1.59e-06***		0.02	(-0.00,0.05)	0.01	0.088
Flourishing index		0.09	(0.06,0.13)	0.02	3.1e-07***		0.02	(-0.00,0.05)	0.01	0.078
Happiness & life satisfaction		0.05	(0.02,0.08)	0.02	3.78e-03**		0.00	(-0.03,0.03)	0.01	0.975
Physical & mental health		0.05	(0.02,0.09)	0.02	1.79e-03**		0.01	(-0.02,0.04)	0.01	0.412
Meaning & purpose		0.07	(0.04,0.11)	0.02	6.77e-05***		0.01	(-0.02,0.04)	0.02	0.409
Character & virtue		0.11	(0.08,0.15)	0.02	1.36e-09***		0.06	(0.02,0.09)	0.02	7.66e-04**
Close social relationships		0.06	(0.02,0.10)	0.02	1.64e-03**		0.01	(-0.02,0.04)	0.02	0.518
Financial & material security		0.03	(-0.00,0.07)	0.02	0.084		0.01	(-0.02,0.05)	0.02	0.476
Psychological Well-Being										
Happiness (a)		0.05	(0.01,0.08)	0.02	0.008*		-0.00	(-0.03,0.03)	0.02	0.981
Life satisfaction (a)		0.05	(0.01,0.08)	0.02	4.92e-03**		0.00	(-0.03,0.03)	0.01	0.936
Current life evaluation		0.04	(0.01,0.08)	0.02	0.015*		0.01	(-0.02,0.04)	0.02	0.692
Future life evaluation		0.04	(0.01,0.08)	0.02	0.017*		0.00	(-0.03,0.03)	0.02	0.952
Optimism		0.08	(0.05,0.12)	0.02	4.47e-06***		0.04	(0.01,0.07)	0.02	0.018*
Freedom to pursue what's important		0.05	(0.01,0.09)	0.02	0.008*		0.01	(-0.02,0.05)	0.02	0.431
Inner peace	1.02		(1.01,1.04)	0.01	0.006*	1.01		(0.99,1.02)	0.01	0.454
Life balance	1.02		(1.00,1.03)	0.01	0.042*	1.00		(0.99,1.01)	0.01	0.888
Sense of mastery	1.02		(1.00,1.03)	0.01	0.022*	1.00		(0.99,1.02)	0.01	0.675
Meaningful activities (c)		0.05	(0.01,0.08)	0.02	0.008*		-0.00	(-0.03,0.03)	0.02	0.933
Understanding purpose (c)		0.08	(0.04,0.11)	0.02	2.84e-05***		0.02	(-0.01,0.05)	0.02	0.184
Self-rated mental health (b)		0.06	(0.02,0.09)	0.02	1.34e-03**		0.02	(-0.01,0.05)	0.02	0.310
Psychological Distress										
Traumatic distress	0.99		(0.97,1.01)	0.01	0.188	0.99		(0.97,1.00)	0.01	0.100
Depression symptoms composite	1.00		(0.98,1.01)	0.01	0.588	1.00		(0.99,1.01)	0.01	0.747
Depression – feel hopeless	1.00		(0.98,1.01)	0.01	0.697	1.00		(0.99,1.01)	0.01	0.611
Depression – loss of interest	1.00		(0.99,1.01)	0.01	0.882	1.00		(0.99,1.02)	0.01	0.552
Anxiety symptoms composite	0.99		(0.97,1.00)	0.01	0.060	0.99		(0.98,1.01)	0.01	0.357
Anxiety – feel on edge	0.99		(0.97,1.00)	0.01	0.091	0.99		(0.98,1.01)	0.01	0.303
Anxiety – cannot stop worrying	0.99		(0.98,1.01)	0.01	0.402	1.00		(0.99,1.02)	0.01	0.840
Suffering	0.99		(0.98,1.01)	0.01	0.532	1.00		(0.99,1.02)	0.01	0.738
Social Well-Being										
Relationship contentment (e)		0.05	(0.01,0.08)	0.02	0.011*		0.00	(-0.03,0.03)	0.02	0.939
Relationship satisfaction (e)		0.06	(0.03,0.10)	0.02	7.73e-04**		0.02	(-0.01,0.05)	0.02	0.285
Social support		0.12	(0.08,0.16)	0.02	4.46e-10***		0.09	(0.05,0.12)	0.02	9.41e-07***
Intimate/close friend	1.04		(1.02,1.05)	0.01	4.38e-07***	1.03		(1.01,1.04)	0.01	9.53e-05***
Government approval	1.02		(1.01,1.04)	0.01	3.83e-03**	1.01		(1.00,1.03)	0.01	0.118
Say in government	1.01		(0.99,1.03)	0.01	0.171	1.00		(0.98,1.02)	0.01	0.954
Belonging in country		0.03	(-0.00,0.07)	0.02	0.052		0.01	(-0.03,0.04)	0.02	0.645
City/place satisfaction	1.03		(1.01,1.04)	0.01	7.25e-04**	1.02		(1.00,1.03)	0.01	0.017*
Trust within country	1.03		(1.02,1.05)	0.01	9.92e-05***	1.03		(1.01,1.05)	0.01	9.96e-04**
Social Participation										
Ever been married	1.01		(0.99,1.02)	0.01	0.276	1.01		(0.99,1.02)	0.01	0.407
Currently divorced	1.00		(1.00,1.01)	0.00	0.389	1.00		(1.00,1.01)	0.00	0.559
Number of children		0.01	(-0.02,0.04)	0.02	0.565		-0.01	(-0.04,0.03)	0.02	0.688
Weekly+ community participation	1.00		(0.99,1.02)	0.01	0.620	1.00		(0.98,1.01)	0.01	0.777
Weekly+ religious attendance	1.00		(1.00,1.01)	0.00	0.401	1.00		(0.99,1.01)	0.00	0.767
Social Distress										
Loneliness		-0.06	(-0.09,-0.03)	0.02	6.03e-04***		-0.02	(-0.05,0.01)	0.02	0.195
Perceived discrimination	0.99		(0.98,1.01)	0.01	0.416	0.99		(0.98,1.00)	0.01	0.222
Character & Prosocial Behavior										
Orientation to promote good (d)		0.12	(0.08,0.16)	0.02	5.36e-10***		0.07	(0.03,0.10)	0.02	1.73e-04***
Delayed gratification (d)		0.07	(0.03,0.10)	0.02	1.31e-04***		0.03	(-0.01,0.06)	0.02	0.097
Hope		0.11	(0.07,0.14)	0.02	1.32e-09***		0.05	(0.02,0.08)	0.02	3.07e-04***
Gratitude		0.11	(0.07,0.15)	0.02	1.81e-08***		0.06	(0.02,0.09)	0.02	9.49e-04**
Showing love/care		0.09	(0.05,0.12)	0.02	1.59e-06***		0.06	(0.02,0.09)	0.02	1.53e-03**
Forgivingness	1.14		(1.12,1.16)	0.01	2.22e-16***	1.14		(1.12,1.16)	0.01	2.22e-16***
Charitable giving	1.02		(1.01,1.04)	0.01	4.1e-03**	1.01		(0.99,1.02)	0.01	0.242
Helping strangers	1.05		(1.03,1.07)	0.01	6.33e-08***	1.03		(1.02,1.05)	0.01	8.86e-05***
Volunteering	1.01		(1.00,1.02)	0.01	0.157	1.00		(0.99,1.01)	0.01	0.891
Physical Health & Health Behavior										
Self-rated physical health (b)		0.04	(0.00,0.07)	0.02	0.030*		0.00	(-0.02,0.03)	0.01	0.739
Health problems	0.99		(0.98,1.01)	0.01	0.373	1.00		(0.98,1.01)	0.01	0.906
Pain in past 4 weeks	1.00		(0.98,1.01)	0.01	0.676	1.00		(0.98,1.02)	0.01	0.941
Daily smoker	1.00		(0.98,1.01)	0.01	0.940	1.00		(0.98,1.01)	0.01	0.627
Number of drinks per week		-0.02	(-0.07,0.02)	0.02	0.289		-0.02	(-0.06,0.03)	0.02	0.469
Days exercise per week		0.04	(0.00,0.07)	0.02	0.034*		0.01	(-0.02,0.05)	0.02	0.384
Socioeconomic Outcomes										
Financial security (f)		0.01	(-0.02,0.05)	0.02	0.523		-0.01	(-0.04,0.03)	0.02	0.750
Material security (f)		0.05	(0.01,0.09)	0.02	0.008*		0.03	(-0.01,0.06)	0.02	0.097
Educational attainment (16+ years)	1.00		(0.99,1.01)	0.00	0.949	1.00		(0.99,1.01)	0.00	0.735
Currently employed	1.00		(0.99,1.01)	0.00	0.519	1.00		(0.99,1.01)	0.00	0.553
Financially comfortable/getting by	1.01		(0.99,1.02)	0.01	0.293	1.00		(0.99,1.02)	0.01	0.734
Own home	1.00		(0.99,1.02)	0.01	0.796	1.00		(0.98,1.01)	0.01	0.769
Income – top quintile	1.00		(0.98,1.01)	0.01	0.898	1.00		(0.98,1.01)	0.01	0.732
Religion & Spirituality										
Religious/spiritual connection	1.01		(0.99,1.02)	0.01	0.428	0.99		(0.97,1.00)	0.01	0.087
Belief in life after death	0.99		(0.98,1.01)	0.01	0.453	0.98		(0.96,1.00)	0.01	0.011*
Transformative religious experience	1.00		(0.99,1.01)	0.01	0.823	0.99		(0.97,1.00)	0.01	0.032*
Religious reading or listening	1.01		(1.00,1.01)	0.00	0.074	1.00		(0.99,1.01)	0.00	0.946
Prayer or meditation	1.00		(0.99,1.01)	0.01	0.746	0.99		(0.98,1.00)	0.00	0.005*
Belief in God/gods/spiritual forces	0.99		(0.98,1.01)	0.01	0.405	0.98		(0.96,0.99)	0.01	0.007*
Religious centrality	1.01		(1.00,1.02)	0.01	0.137	1.00		(0.98,1.01)	0.01	0.464
Religious/spiritual comfort	1.01		(1.00,1.03)	0.01	0.091	1.00		(0.98,1.01)	0.01	0.603
Feel loved by God	1.01		(1.00,1.03)	0.01	0.041*	1.00		(0.98,1.01)	0.01	0.618
Feel punished by God	1.00		(0.99,1.00)	0.00	0.306	0.99		(0.98,1.00)	0.00	0.053
Experienced religious criticism	1.00		(1.00,1.01)	0.00	0.727	1.00		(0.99,1.00)	0.00	0.399
Faith-sharing	1.02		(1.01,1.03)	0.01	3.85e-03**	1.01		(0.99,1.02)	0.01	0.365

Table S14h. Associations of forgivingness with adult well-being and other outcomes at Wave 2 in Germany using complete-case analyses with attrition weights.

	Model 1: Demographic and Childhood Variables as Covariates					Model 2: Demographic, Childhood, and Other Wave 1 Confounding Variables (Via Principal Components) as Covariates				
Outcome	RR	ES	95% CI	SE	p-value	RR	ES	95% CI	SE	p-value

Notes. N=5529; Reference for focal predictor: never/rarely. RR, risk-ratio, null effect is 1.00; ES, effect size measure for standardized regression coefficient, null effect is 0.00; SE, standard error, the SE reported for binary/Likert-type outcomes where risk-ratios are on the log(RR) scale; CI, confidence interval; p-value, a Wald-type test of the null hypothesis that the effect of the focal predictor is zero; (a) item part of the Happiness & Life Satisfaction domain of the Secure Flourishing Index; (b) item part of the Physical & Mental Health domain of the Secure Flourishing Index; (c) item part of the Meaning & Purpose domain of the Secure Flourishing Index; (d) item part of the Character & Virtue domain of the Secure Flourishing Index; (e) item part of the Subjective Social Connectedness domain of the Secure Flourishing Index; (f) item part of the Financial & Material Security domain of the Secure Flourishing Index.

Attrition weights were computed to adjust the complete case data (those who responded at Wave 2 to at least 50% of the questions) and multiple imputation was used to impute missing data on all remaining within wave on the covariates, exposure, and outcomes. All models controlled for sociodemographic and childhood factors assessed at Wave 1. For Model 2 with PC (principal components), the first seven principal components of the entire set of contemporaneous confounders assessed at Wave 1 were included as additional covariates of the outcomes at Wave 2.

An outcome-wide analytic approach was used, and a separate model was run for each outcome. A different type of model was run depending on the nature of the outcome: (1) for each binary outcome, a weighted generalized linear model (with a log link and Poisson distribution) was used to estimate an RR; and (2) for each continuous outcome, a weighted linear regression model was used to estimate a ES. All effect sizes were standardized. For continuous outcomes, the ES represents the change in SD on the outcome between the lower and upper categories of the binary focal predictor. For binary outcomes, the RR represents the change in risk of being in the upper category compared to the lower category between the lower and upper categories of the binary focal predictor.

P-value significance thresholds: $p < 0.05$*, $p < 0.005$**, (Bonferroni) $p < 6.41e-04$***, correction for multiple testing using Bonferroni adjusted significant threshold.

Table S14i. Sensitivity analysis of forgivingness outcome-wide results to unmeasured confounding using E-values in Germany

	Multiple Imputation				Complete Case w/ Attrition Weights			
	Model 1: Demographics and Childhood Variables as Covariates		Model 2: Demographics, Childhood, and Other Wave 1 Confounders (Via Principal Components) as Covariates		Model 1: Demographics and Childhood Variables as Covariates		Model 2: Demographics, Childhood, and Other Wave 1 Confounders (Via Principal Components) as Covariates	
Outcome	EE	ECI	EE	ECI	EE	ECI	EE	ECI
Human Flourishing								
Secure flourishing index	1.61	1.45	1.20	1.00	1.68	1.46	1.27	1.00
Flourishing index	1.65	1.49	1.19	1.00	1.71	1.50	1.27	1.00
Happiness & life satisfaction	1.42	1.23	1.03	1.00	1.45	1.22	1.03	1.00
Physical & mental health	1.38	1.17	1.03	1.00	1.48	1.25	1.19	1.00
Meaning & purpose	1.52	1.33	1.06	1.00	1.60	1.37	1.19	1.00
Character & virtue	1.80	1.61	1.45	1.25	1.83	1.62	1.49	1.28
Close social relationships	1.49	1.31	1.12	1.00	1.51	1.27	1.17	1.00
Financial & material security	1.26	1.00	1.14	1.00	1.34	1.00	1.19	1.00
Psychological Well-Being								
Happiness	1.40	1.19	1.04	1.00	1.43	1.19	1.03	1.00
Life satisfaction	1.38	1.16	1.02	1.00	1.43	1.20	1.05	1.00
Current life evaluation	1.34	1.04	1.11	1.00	1.41	1.15	1.13	1.00
Future life evaluation	1.38	1.14	1.09	1.00	1.40	1.14	1.05	1.00
Optimism	1.63	1.45	1.34	1.12	1.66	1.44	1.38	1.13
Freedom to pursue what's important	1.33	1.00	1.11	1.00	1.45	1.20	1.20	1.00
Inner peace	1.25	1.12	1.12	1.00	1.27	1.13	1.12	1.00
Life balance	1.22	1.06	1.02	1.00	1.23	1.04	1.05	1.00
Sense of mastery	1.21	1.08	1.04	1.00	1.24	1.08	1.09	1.00
Meaningful activities	1.37	1.14	1.17	1.00	1.44	1.19	1.06	1.00
Understanding purpose	1.54	1.34	1.13	1.00	1.62	1.40	1.26	1.00
Self-rated mental health	1.43	1.20	1.16	1.00	1.50	1.27	1.22	1.00
Psychological Distress								
Traumatic distress	1.10	1.00	1.13	1.00	1.18	1.00	1.21	1.00
Depression symptoms composite	1.11	1.00	1.03	1.00	1.10	1.00	1.07	1.00
Depression – feel hopeless	1.09	1.00	1.04	1.00	1.08	1.00	1.09	1.00
Depression – loss of interest	1.07	1.00	1.07	1.00	1.05	1.00	1.10	1.00
Anxiety symptoms composite	1.18	1.00	1.09	1.00	1.22	1.00	1.14	1.00
Anxiety – feel on edge	1.16	1.00	1.10	1.00	1.20	1.00	1.15	1.00
Anxiety – cannot stop worrying	1.12	1.00	1.04	1.00	1.13	1.00	1.06	1.00
Suffering	1.08	1.00	1.10	1.00	1.13	1.00	1.09	1.00
Social Well-Being								
Relationship contentment	1.43	1.22	1.01	1.00	1.44	1.18	1.05	1.00
Relationship satisfaction	1.48	1.27	1.16	1.00	1.54	1.30	1.24	1.00
Social support	1.75	1.52	1.57	1.33	1.87	1.65	1.67	1.47
Intimate/close friend	1.35	1.24	1.29	1.17	1.39	1.28	1.32	1.21
Government approval	1.25	1.09	1.20	1.00	1.30	1.15	1.20	1.00
Say in government	1.22	1.00	1.12	1.00	1.20	1.00	1.04	1.00
Belonging in country	1.37	1.13	1.17	1.00	1.36	1.00	1.14	1.00
City/place satisfaction	1.24	1.10	1.18	1.00	1.33	1.19	1.26	1.10
Trust within country	1.37	1.25	1.34	1.22	1.37	1.24	1.33	1.19
Social Participation								
Ever been married	1.07	1.00	1.05	1.00	1.14	1.00	1.13	1.00
Currently divorced	1.08	1.00	1.07	1.00	1.09	1.00	1.07	1.00
Number of children	1.26	1.00	1.12	1.00	1.17	1.00	1.13	1.00
Weekly+ community participation	1.08	1.00	1.06	1.00	1.10	1.00	1.08	1.00
Weekly+ religious attendance	1.08	1.00	1.04	1.00	1.10	1.00	1.05	1.00
Social Distress								
Loneliness	1.41	1.16	1.13	1.00	1.52	1.29	1.26	1.00
Perceived discrimination	1.11	1.00	1.15	1.00	1.13	1.00	1.16	1.00
Character & Prosocial Behavior								
Orientation to promote good	1.81	1.61	1.48	1.27	1.88	1.65	1.55	1.33
Delayed gratification	1.54	1.33	1.30	1.00	1.57	1.35	1.31	1.00
Hope	1.72	1.54	1.42	1.21	1.81	1.60	1.48	1.29
Gratitude	1.76	1.57	1.43	1.24	1.81	1.58	1.49	1.27
Showing love/care	1.64	1.44	1.41	1.17	1.69	1.47	1.49	1.26
Forgivingness	2.10	2.00	2.06	1.95	2.05	1.94	2.01	1.90
Charitable giving	1.24	1.10	1.12	1.00	1.30	1.15	1.16	1.00
Helping strangers	1.42	1.32	1.32	1.20	1.47	1.35	1.37	1.24
Volunteering	1.14	1.00	1.08	1.00	1.18	1.00	1.05	1.00
Physical Health & Health Behavior								
Self-rated physical health	1.23	1.00	1.16	1.00	1.37	1.10	1.11	1.00
Health problems	1.11	1.00	1.02	1.00	1.14	1.00	1.05	1.00
Pain in past 4 weeks	1.07	1.00	1.05	1.00	1.10	1.00	1.04	1.00
Daily smoker	1.09	1.00	1.12	1.00	1.04	1.00	1.10	1.00
Number of drinks per week	1.27	1.00	1.21	1.00	1.27	1.00	1.22	1.00
Days exercise per week	1.29	1.00	1.11	1.00	1.37	1.08	1.21	1.00
Socioeconomic Outcomes								
Financial security	1.11	1.00	1.12	1.00	1.18	1.00	1.12	1.00
Material security	1.35	1.09	1.25	1.00	1.45	1.19	1.32	1.00
Educational attainment (16+ years)	1.02	1.00	1.05	1.00	1.02	1.00	1.05	1.00
Currently employed	1.02	1.00	1.05	1.00	1.09	1.00	1.09	1.00
Financially comfortable/getting by	1.09	1.00	1.03	1.00	1.16	1.00	1.08	1.00
Own home	1.11	1.00	1.12	1.00	1.08	1.00	1.08	1.00
Income – top quintile	1.10	1.00	1.09	1.00	1.05	1.00	1.08	1.00
Religion & Spirituality								
Religious/spiritual connection	1.29	1.16	1.02	1.00	1.14	1.00	1.20	1.00
Belief in life after death	1.20	1.00	1.14	1.00	1.14	1.00	1.28	1.12
Transformative religious experience	1.14	1.00	1.16	1.00	1.06	1.00	1.22	1.05
Religious reading or listening	1.14	1.04	1.04	1.00	1.13	1.00	1.02	1.00
Prayer or meditation	1.10	1.00	1.18	1.07	1.07	1.00	1.21	1.11
Belief in God/gods/spiritual forces	1.12	1.00	1.20	1.00	1.15	1.00	1.29	1.13
Religious centrality	1.20	1.07	1.10	1.00	1.16	1.00	1.11	1.00
Religious/spiritual comfort	1.24	1.11	1.08	1.00	1.20	1.00	1.10	1.00
Feel loved by God	1.26	1.14	1.08	1.00	1.22	1.04	1.10	1.00
Feel punished by God	1.06	1.00	1.13	1.00	1.11	1.00	1.16	1.00
Experienced religious criticism	1.08	1.00	1.07	1.00	1.05	1.00	1.08	1.00
Faith-sharing	1.27	1.16	1.12	1.00	1.26	1.13	1.13	1.00

Notes. EE, E-value for estimate; ECI, E-value for the limit of the confidence interval. The formula for calculating E-values can be found in VanderWeele and Ding (2017). E-values for estimate are the minimum strength of association on the risk ratio scale that an unmeasured confounder would need to have with both the exposure and the outcome to fully explain away the observed association between the exposure and outcome, conditional on the measured covariates. E-values for the 95% CI closest to the null denote the minimum strength of association on the risk ratio scale that an unmeasured confounder would need to have with both the exposure and the outcome to shift the CI to include the null value, conditional on the measured covariates.

Table S15a. Weighted summary statistics for demographic and childhood variables in Hong Kong

**Characteristic**	**Wave 1** N = 3,012	**Wave 2** N = 611
Forgivingness, n (%)		
Always	502 (16.7%)	68 (11.1%)
Often	1,351 (44.9%)	280 (45.8%)
Rarely	999 (33.2%)	246 (40.3%)
Never	145 (4.8%)	17 (2.7%)
(Missing)	14 (0.5%)	0 (0%)
Year of birth, n (%)		
1943 or earlier (current age: 80+ years)	13 (0.4%)	4 (0.7%)
1943-1953 (current age: 70-79 years)	144 (4.8%)	39 (6.3%)
1953-1963 (current age: 60-69 years)	586 (19.5%)	117 (19.1%)
1963-1973 (current age: 50-59 years)	703 (23.3%)	128 (21.0%)
1973-1983 (current age: 40-49 years)	577 (19.2%)	132 (21.6%)
1983-1993 (current age: 30-39 years)	533 (17.7%)	119 (19.4%)
1993-1998 (current age: 25-29 years)	206 (6.8%)	41 (6.7%)
1998-2005 (current age: 18-24 years)	250 (8.3%)	31 (5.1%)
(Missing)	0 (0%)	0 (0%)
Age of participant		
Mean	47.4	48.3
Standard Deviation	15.1	14.5
Min, Max	18.0, 85.0	20.0, 80.0
Gender, n (%)		
Male	1,380 (45.8%)	277 (45.3%)
Female	1,629 (54.1%)	334 (54.7%)
Other	2 (0.1%)	0 (0%)
(Missing)	0 (0%)	0 (0%)
Respondent marital status, n (%)		
Single/Never been married	757 (25.1%)	196 (32.1%)
Married	2,051 (68.1%)	359 (58.8%)
Separated	19 (0.6%)	3 (0.5%)
Divorced	103 (3.4%)	31 (5.0%)
Widowed	46 (1.5%)	12 (2.0%)
Domestic partner	37 (1.2%)	9 (1.5%)
(Missing)	1 (0.0%)	0 (0%)
Education (years), n (%)		
Up to 8	401 (13.3%)	41 (6.7%)
9-15	2,083 (69.2%)	399 (65.4%)
16+	528 (17.5%)	171 (27.9%)
(Missing)	0 (0%)	0 (0%)
Employment status, n (%)		
Employed for an employer	2,084 (69.2%)	434 (71.1%)
Self-employed	247 (8.2%)	43 (7.1%)
Retired	387 (12.8%)	81 (13.2%)
Student	62 (2.1%)	6 (1.0%)
Homemaker	115 (3.8%)	28 (4.5%)
Unemployed and looking for a job	62 (2.1%)	15 (2.5%)
None of these/Other	37 (1.2%)	3 (0.5%)
(Missing)	19 (0.6%)	0 (0%)
Current religious service attendance, n (%)		
More than once a week	237 (7.9%)	18 (3.0%)
Once a week	561 (18.6%)	89 (14.5%)

Table S15a. Weighted summary statistics for demographic and childhood variables in Hong Kong

**Characteristic**	**Wave 1** N = 3,012	**Wave 2** N = 611
One to three times a month	333 (11.1%)	20 (3.2%)
A few times a year	542 (18.0%)	114 (18.6%)
Never	1,338 (44.4%)	371 (60.7%)
(Missing)	1 (0.0%)	0 (0%)
Immigration status, n (%)		
Born in this country	2,650 (88.0%)	525 (85.9%)
Born in another country	308 (10.2%)	74 (12.1%)
(Missing)	54 (1.8%)	12 (2.0%)
Parental marital status around age 12, n (%)		
Parents were married	2,750 (91.3%)	552 (90.4%)
Parents were divorced	115 (3.8%)	25 (4.1%)
Parents were never married	43 (1.4%)	14 (2.3%)
One or both of them had died	50 (1.6%)	13 (2.1%)
Unsure	53 (1.8%)	6 (1.0%)
(Missing)	1 (0.0%)	0 (0%)
Religious service attendance around age 12, n (%)		
At least once a week	427 (14.2%)	58 (9.5%)
One to three times a month	535 (17.8%)	65 (10.6%)
Less than once a month	742 (24.6%)	187 (30.5%)
Never	1,303 (43.3%)	301 (49.3%)
(Missing)	4 (0.1%)	0 (0%)
Relationship with mother when growing up, n (%)		
Very good	1,070 (35.5%)	203 (33.2%)
Somewhat good	1,170 (38.8%)	227 (37.3%)
Somewhat bad	297 (9.8%)	78 (12.8%)
Very bad	49 (1.6%)	26 (4.3%)
(Does not apply)	423 (14.1%)	76 (12.4%)
(Missing)	3 (0.1%)	0 (0.0%)
Relationship with father when growing up, n (%)		
Very good	856 (28.4%)	149 (24.3%)
Somewhat good	1,101 (36.5%)	232 (38.0%)
Somewhat bad	396 (13.1%)	86 (14.2%)
Very bad	105 (3.5%)	41 (6.7%)
(Does not apply)	551 (18.3%)	103 (16.8%)
(Missing)	4 (0.1%)	0 (0%)
Felt like an outsider in family when growing up, n (%)		
Yes	671 (22.3%)	92 (15.0%)
No	2,216 (73.6%)	510 (83.6%)
(Missing)	126 (4.2%)	9 (1.4%)
Experienced abuse when growing up, n (%)		
Yes	321 (10.7%)	59 (9.6%)
No	2,687 (89.2%)	552 (90.4%)
(Missing)	4 (0.1%)	0 (0%)
Self-rated health when growing up, n (%)		
Excellent	548 (18.2%)	46 (7.6%)
Very good	1,073 (35.6%)	204 (33.4%)
Good	858 (28.5%)	226 (37.1%)
Fair	428 (14.2%)	106 (17.3%)
Poor	92 (3.1%)	27 (4.4%)
(Missing)	12 (0.4%)	2 (0.3%)

Table S15a. Weighted summary statistics for demographic and childhood variables in Hong Kong

**Characteristic**	**Wave 1**<br>N = 3,012	**Wave 2**<br>N = 611
Subjective financial status of family growing up, n (%)		
Lived comfortably	912 (30.3%)	94 (15.4%)
Got by	1,530 (50.8%)	340 (55.6%)
Found it difficult	466 (15.5%)	146 (23.9%)
Found it very difficult	82 (2.7%)	25 (4.1%)
(Missing)	23 (0.7%)	7 (1.1%)
Religious affiliation growing up, n (%)		
Christianity	711 (23.6%)	119 (19.5%)
Taoism	77 (2.6%)	23 (3.8%)
Confucianism	10 (0.3%)	0 (0%)
Primal, Animist, or Folk religion	17 (0.6%)	2 (0.3%)
Spiritism	0 (0%)	0 (0%)
Umbanda, Candomblé, and other African-derived religions	0 (0%)	0 (0%)
Chinese folk/traditional religion	110 (3.7%)	28 (4.5%)
Islam	88 (2.9%)	2 (0.4%)
Hinduism	28 (0.9%)	1 (0.1%)
Buddhism	324 (10.8%)	47 (7.7%)
Judaism	17 (0.6%)	1 (0.1%)
Sikhism	4 (0.1%)	0 (0%)
Baha'i	0 (0%)	0 (0%)
Jainism	1 (0.0%)	0 (0.1%)
Shinto	18 (0.6%)	0 (0%)
Some other religion	4 (0.1%)	3 (0.4%)
No religion/Atheist/Agnostic	1,602 (53.2%)	385 (63.0%)
(Missing)	1 (0.0%)	0 (0%)

Note. N (%); this table is based on non-imputed data. Cumulative percentages for variables may not add up to 100% due to rounding. Wave 1 characteristics weighted using the Gallup provided sampling weight, ANNUAL_WEIGHT_R2; Wave 2 characteristics weighted accounting for attrition by using the adjusted Wave 1 weight, ANNUAL_WEIGHT_R2, multiplied by the created attrition weight to account for dropout, to maintain nationally representative estimates for Wave 2 characteristics.

Table S15b. Weighted summary statistics for outcome variables in Hong Kong

Outcome	Wave 1<br>N = 3,012	Wave 2<br>N = 611
Secure flourishing index		
Mean	7.1	6.4
Standard Deviation	1.8	1.5
Min, Max	0.0, 10.0	1.4, 10.0
(Missing)	179 (5.9%)	25 (4.1%)
Flourishing index		
Mean	7.1	6.4
Standard Deviation	1.8	1.5
Min, Max	0.0, 10.0	1.2, 10.0
(Missing)	144 (4.8%)	24 (3.9%)
Happiness & life satisfaction		
Mean	7.1	6.3
Standard Deviation	2.0	1.7
Min, Max	0.0, 10.0	0.0, 10.0
(Missing)	54 (1.8%)	13 (2.2%)
Physical & mental health		
Mean	7.1	6.5
Standard Deviation	1.9	1.7
Min, Max	0.0, 10.0	0.0, 10.0
(Missing)	19 (0.6%)	8 (1.3%)
Meaning & purpose		
Mean	7.2	6.5
Standard Deviation	1.9	1.8
Min, Max	0.0, 10.0	0.0, 10.0
(Missing)	35 (1.2%)	7 (1.1%)
Character & virtue		
Mean	7.2	6.5
Standard Deviation	1.8	1.7
Min, Max	0.0, 10.0	0.0, 10.0
(Missing)	12 (0.4%)	5 (0.9%)
Close social relationships		
Mean	7.1	6.5
Standard Deviation	2.0	1.9
Min, Max	0.0, 10.0	0.0, 10.0
(Missing)	65 (2.2%)	15 (2.5%)
Financial & material security		
Mean	6.8	6.3
Standard Deviation	2.4	2.2
Min, Max	0.0, 10.0	0.0, 10.0
(Missing)	43 (1.4%)	3 (0.5%)
Happiness		
Mean	7.1	6.4
Standard Deviation	2.0	1.7
Min, Max	0.0, 10.0	0.0, 10.0
(Missing)	5 (0.2%)	0 (0%)
Life satisfaction		
Mean	7.0	6.2
Standard Deviation	2.1	1.9
Min, Max	0.0, 10.0	0.0, 10.0
(Missing)	51 (1.7%)	13 (2.2%)
Current life evaluation		

Table S15b. Weighted summary statistics for outcome variables in Hong Kong

Outcome	Wave 1<br>N = 3,012	Wave 2<br>N = 611
Mean	6.8	6.0
Standard Deviation	2.0	1.8
Min, Max	0.0, 10.0	0.0, 10.0
(Missing)	1 (<0.1%)	0 (0%)
Future life evaluation		
Mean	7.1	6.3
Standard Deviation	2.0	1.8
Min, Max	0.0, 10.0	0.0, 10.0
(Missing)	39 (1.3%)	13 (2.2%)
Optimism		
Mean	7.2	6.6
Standard Deviation	2.1	2.0
Min, Max	0.0, 10.0	0.0, 10.0
(Missing)	10 (0.3%)	1 (0.1%)
Freedom to pursue what's important		
Mean	7.3	6.7
Standard Deviation	2.1	1.8
Min, Max	0.0, 10.0	0.0, 10.0
(Missing)	15 (0.5%)	1 (0.2%)
Inner peace, n (%)		
Always	919 (30.5%)	140 (22.9%)
Often	1,735 (57.6%)	375 (61.3%)
Rarely	332 (11.0%)	92 (15.1%)
Never	24 (0.8%)	4 (0.7%)
(Missing)	2 (0.1%)	0 (0%)
Life balance, n (%)		
Always	720 (23.9%)	97 (15.9%)
Often	1,671 (55.5%)	390 (63.9%)
Rarely	581 (19.3%)	118 (19.3%)
Never	28 (0.9%)	4 (0.7%)
(Missing)	11 (0.4%)	1 (0.2%)
Sense of mastery, n (%)		
Always	810 (26.9%)	110 (18.0%)
Often	1,759 (58.4%)	400 (65.5%)
Rarely	404 (13.4%)	94 (15.5%)
Never	30 (1.0%)	6 (1.0%)
(Missing)	8 (0.3%)	0 (0%)
Meaningful activities		
Mean	7.2	6.6
Standard Deviation	2.0	1.8
Min, Max	0.0, 10.0	0.0, 10.0
(Missing)	34 (1.1%)	7 (1.1%)
Understanding purpose		
Mean	7.2	6.5
Standard Deviation	2.2	2.1
Min, Max	0.0, 10.0	0.0, 10.0
(Missing)	1 (<0.1%)	0 (0%)
Self-rated mental health		
Mean	7.2	6.6
Standard Deviation	2.1	1.9
Min, Max	0.0, 10.0	0.0, 10.0

Table S15b. Weighted summary statistics for outcome variables in Hong Kong

Outcome	Wave 1<br>N = 3,012	Wave 2<br>N = 611
(Missing)	19 (0.6%)	7 (1.2%)
Traumatic distress, n (%)		
A lot	319 (10.6%)	31 (5.1%)
Some	809 (26.9%)	156 (25.5%)
Not very much	971 (32.2%)	206 (33.8%)
Not at all	909 (30.2%)	215 (35.2%)
(Missing)	4 (0.1%)	3 (0.5%)
Depression symptoms composite, n (%)	1,372 (45.9%)	215 (35.5%)
(Missing)	22 (0.7%)	4 (0.6%)
Depression – feel hopeless, n (%)		
Nearly every day	230 (7.6%)	14 (2.3%)
More than half the days	495 (16.4%)	101 (16.5%)
Several days	1,434 (47.6%)	287 (46.9%)
Not at all	833 (27.7%)	206 (33.7%)
(Missing)	21 (0.7%)	4 (0.6%)
Depression – loss of interest, n (%)		
Nearly every day	410 (13.6%)	36 (5.8%)
More than half the days	913 (30.3%)	182 (29.8%)
Several days	1,152 (38.2%)	259 (42.4%)
Not at all	535 (17.8%)	134 (21.9%)
(Missing)	1 (0.0%)	0 (0%)
Anxiety symptoms composite, n (%)	859 (28.8%)	133 (21.8%)
(Missing)	32 (1.1%)	1 (0.1%)
Anxiety – feel on edge, n (%)		
Nearly every day	216 (7.2%)	25 (4.1%)
More than half the days	529 (17.6%)	97 (15.9%)
Several days	1,401 (46.5%)	303 (49.7%)
Not at all	842 (28.0%)	185 (30.3%)
(Missing)	23 (0.8%)	1 (0.1%)
Anxiety – cannot stop worrying, n (%)		
Nearly every day	225 (7.5%)	11 (1.8%)
More than half the days	462 (15.4%)	95 (15.6%)
Several days	1,181 (39.2%)	217 (35.6%)
Not at all	1,136 (37.7%)	286 (46.9%)
(Missing)	9 (0.3%)	1 (0.1%)
Suffering, n (%)		
A lot	365 (12.1%)	32 (5.2%)
Some	1,099 (36.5%)	230 (37.7%)
Not very much	1,124 (37.3%)	275 (45.1%)
Not at all	423 (14.0%)	74 (12.1%)
(Missing)	1 (0.0%)	0 (0%)
Relationship contentment		
Mean	7.2	6.6
Standard Deviation	2.1	1.9
Min, Max	0.0, 10.0	0.0, 10.0
(Missing)	27 (0.9%)	7 (1.1%)
Relationship satisfaction		
Mean	7.1	6.4
Standard Deviation	2.1	2.0
Min, Max	0.0, 10.0	0.0, 10.0
(Missing)	42 (1.4%)	9 (1.4%)

Table S15b. Weighted summary statistics for outcome variables in Hong Kong

**Outcome**	**Wave 1**<br>N = 3,012	**Wave 2**<br>N = 611
Social support		
Mean	6.4	5.5
Standard Deviation	2.6	2.4
Min, Max	0.0, 10.0	0.0, 10.0
(Missing)	1 (<0.1%)	0 (0%)
Intimate/close friend, n (%)		
Yes	2,508 (83.3%)	491 (80.5%)
No	499 (16.6%)	119 (19.5%)
(Missing)	5 (0.2%)	0 (0%)
Government approval, n (%)		
Strongly approve	608 (20.2%)	22 (3.7%)
Somewhat approve	973 (32.3%)	184 (30.1%)
Neither approve nor disapprove	728 (24.2%)	218 (35.8%)
Somewhat disapprove	388 (12.9%)	96 (15.8%)
Strongly disapprove	311 (10.3%)	87 (14.3%)
(Missing)	5 (0.2%)	3 (0.4%)
Say in government, n (%)		
Agree	1,413 (46.9%)	206 (33.8%)
Disagree	875 (29.1%)	228 (37.3%)
Unsure	717 (23.8%)	172 (28.1%)
(Missing)	6 (0.2%)	5 (0.8%)
Belonging in country		
Mean	7.4	6.9
Standard Deviation	2.2	2.1
Min, Max	0.0, 10.0	0.0, 10.0
(Missing)	5 (0.2%)	0 (0%)
City/place satisfaction, n (%)		
Satisfied	2,101 (69.8%)	357 (58.4%)
Dissatisfied	612 (20.3%)	158 (25.8%)
Unsure	297 (9.9%)	96 (15.7%)
(Missing)	2 (0.1%)	0 (0%)
Trust within country, n (%)		
All people	274 (9.1%)	8 (1.3%)
Most people	1,161 (38.6%)	212 (34.7%)
Some people	1,007 (33.4%)	249 (40.8%)
Not very many people	523 (17.4%)	137 (22.5%)
None	46 (1.5%)	4 (0.6%)
(Missing)	2 (0.1%)	0 (0.0%)
Number of children		
Mean	0.6	0.5
Standard Deviation	1.0	1.0
Min, Max	0.0, 12.0	0.0, 12.0
(Missing)	39 (1.3%)	93 (15%)
Community participation, n (%)		
More than once a week	238 (7.9%)	14 (2.4%)
Once a week	403 (13.4%)	43 (7.1%)
One to three times a month	401 (13.3%)	37 (6.1%)
A few times a year	614 (20.4%)	151 (24.8%)
Never	1,350 (44.8%)	364 (59.7%)
(Missing)	5 (0.2%)	0 (0%)
Religious attendance, n (%)		

Table S15b. Weighted summary statistics for outcome variables in Hong Kong

**Outcome**	**Wave 1**<br>N = 3,012	**Wave 2**<br>N = 611
More than once a week	237 (7.9%)	18 (3.0%)
Once a week	561 (18.6%)	89 (14.5%)
One to three times a month	333 (11.1%)	20 (3.2%)
A few times a year	542 (18.0%)	114 (18.6%)
Never	1,338 (44.4%)	371 (60.7%)
(Missing)	1 (0.0%)	0 (0%)
Loneliness		
Mean	3.3	3.7
Standard Deviation	2.6	2.4
Min, Max	0.0, 10.0	0.0, 10.0
(Missing)	20 (0.7%)	6 (1.0%)
Perceived discrimination, n (%)		
Always	266 (8.8%)	21 (3.4%)
Often	568 (18.9%)	90 (14.7%)
Rarely	1,251 (41.5%)	312 (51.2%)
Never	921 (30.6%)	188 (30.7%)
(Missing)	5 (0.2%)	0 (0%)
Orientation to promote good		
Mean	7.3	6.6
Standard Deviation	2.0	1.8
Min, Max	0.0, 10.0	0.0, 10.0
Delayed gratification		
Mean	7.1	6.4
Standard Deviation	2.0	1.9
Min, Max	0.0, 10.0	0.0, 10.0
(Missing)	12 (0.4%)	5 (0.9%)
Hope		
Mean	7.3	6.5
Standard Deviation	2.1	2.0
Min, Max	0.0, 10.0	0.0, 10.0
(Missing)	8 (0.3%)	0 (0%)
Gratitude		
Mean	6.9	6.3
Standard Deviation	2.2	2.0
Min, Max	0.0, 10.0	0.0, 10.0
(Missing)	11 (0.3%)	1 (0.2%)
Showing love/care		
Mean	6.5	5.7
Standard Deviation	2.6	2.3
Min, Max	0.0, 10.0	0.0, 10.0
(Missing)	4 (0.1%)	6 (0.9%)
Forgivingness, n (%)		
Always	502 (16.7%)	68 (11.1%)
Often	1,351 (44.9%)	280 (45.8%)
Rarely	999 (33.2%)	246 (40.3%)
Never	145 (4.8%)	17 (2.7%)
(Missing)	14 (0.5%)	0 (0%)
Charitable giving, n (%)		
Yes	1,526 (50.7%)	209 (34.2%)
No	1,476 (49.0%)	402 (65.8%)
(Missing)	10 (0.3%)	0 (0%)

Table S15b. Weighted summary statistics for outcome variables in Hong Kong

**Outcome**	**Wave 1** N = 3,012	**Wave 2** N = 611
Helping strangers, n (%)		
Yes	1,950 (64.7%)	277 (45.3%)
No	1,055 (35.0%)	334 (54.7%)
(Missing)	7 (0.2%)	0 (0%)
Volunteering, n (%)		
Yes	1,041 (34.6%)	79 (13.0%)
No	1,959 (65.0%)	531 (87.0%)
(Missing)	12 (0.4%)	0 (0%)
Self-rated physical health		
Mean	7.1	6.3
Standard Deviation	2.0	1.7
Min, Max	0.0, 10.0	0.0, 10.0
Health problems, n (%)		
Yes	622 (20.6%)	106 (17.3%)
No	2,388 (79.3%)	493 (80.8%)
(Missing)	2 (0.1%)	12 (1.9%)
Pain in past 4 weeks, n (%)		
A lot	324 (10.7%)	39 (6.4%)
Some	1,031 (34.2%)	231 (37.8%)
Not very much	1,095 (36.4%)	227 (37.2%)
None at all	549 (18.2%)	114 (18.6%)
(Missing)	13 (0.4%)	0 (0%)
Number of cigarettes per day		
Mean	2.0	1.0
Standard Deviation	4.2	3.1
Min, Max	0.0, 30.0	0.0, 20.0
(Missing)	29 (1.0%)	3 (0.5%)
Number of drinks per week		
Mean	1.7	1.0
Standard Deviation	2.9	2.7
Min, Max	0.0, 56.0	0.0, 35.0
(Missing)	29 (1.0%)	3 (0.5%)
Days exercise per week		
Mean	2.2	1.9
Standard Deviation	2.2	2.1
Min, Max	0.0, 7.0	0.0, 7.0
(Missing)	5 (0.2%)	2 (0.3%)
Financial security		
Mean	6.8	6.3
Standard Deviation	2.7	2.5
Min, Max	0.0, 10.0	0.0, 10.0
(Missing)	1 (<0.1%)	0 (0%)
Material security		
Mean	6.9	6.4
Standard Deviation	2.4	2.3
Min, Max	0.0, 10.0	0.0, 10.0
(Missing)	42 (1.4%)	3 (0.5%)
Educational attainment (16+ years), n (%)		
Up to 8	401 (13.3%)	41 (6.7%)
9-15	2,083 (69.2%)	399 (65.4%)
16+	528 (17.5%)	171 (27.9%)

Table S15b. Weighted summary statistics for outcome variables in Hong Kong

Outcome	Wave 1<br>N = 3,012	Wave 2<br>N = 611
(Missing)	0 (0%)	0 (0%)
Currently employed, n (%)		
Employed for an employer	2,084 (69.2%)	434 (71.1%)
Self-employed	247 (8.2%)	43 (7.1%)
Retired	387 (12.8%)	81 (13.2%)
Student	62 (2.1%)	6 (1.0%)
Homemaker	115 (3.8%)	28 (4.5%)
Unemployed and looking for a job	62 (2.1%)	15 (2.5%)
None of these/Other	37 (1.2%)	3 (0.5%)
(Missing)	19 (0.6%)	0 (0%)
Financially comfortable/getting by, n (%)		
Living comfortably on present income	852 (28.3%)	97 (15.9%)
Getting by on present income	1,595 (53.0%)	372 (60.9%)
Finding it difficult on present income	454 (15.1%)	117 (19.2%)
Finding it very difficult on present income	110 (3.6%)	22 (3.7%)
(Missing)	1 (0.0%)	2 (0.3%)
Own home, n (%)		
Someone in this household owns this home	1,913 (63.5%)	319 (52.2%)
Someone in this household rents this home	691 (23.0%)	152 (25.0%)
Both	71 (2.3%)	19 (3.0%)
Neither	313 (10.4%)	110 (18.0%)
Rent	0 (0%)	0 (0%)
Own	0 (0%)	0 (0%)
Something else	0 (0%)	0 (0%)
(Missing)	24 (0.8%)	11 (1.8%)
Religious/spiritual connection, n (%)		
Always	499 (16.6%)	61 (9.9%)
Often	884 (29.3%)	128 (21.0%)
Rarely	945 (31.4%)	248 (40.6%)
Never	683 (22.7%)	173 (28.4%)
(Missing)	1 (0.0%)	0 (0%)
Belief in life after death, n (%)		
Yes	1,378 (45.8%)	255 (41.8%)
No	757 (25.1%)	114 (18.7%)
Unsure	873 (29.0%)	235 (38.5%)
(Missing)	3 (0.1%)	6 (1.0%)
Transformative religious experience, n (%)		
Yes	1,272 (42.2%)	183 (29.9%)
No	1,735 (57.6%)	422 (69.1%)
(Missing)	5 (0.2%)	6 (1.0%)
Religious reading or listening, n (%)		
More than once a day	277 (9.2%)	11 (1.8%)
About once a day	515 (17.1%)	41 (6.6%)
Sometimes	943 (31.3%)	206 (33.8%)
Never	1,274 (42.3%)	352 (57.7%)
(Missing)	2 (0.1%)	0 (0.1%)
Prayer or meditation, n (%)		
More than once a day	376 (12.5%)	36 (5.9%)
About once a day	525 (17.4%)	69 (11.4%)
Sometimes	983 (32.6%)	191 (31.3%)
Never	1,121 (37.2%)	313 (51.3%)

Table S15b. Weighted summary statistics for outcome variables in Hong Kong

**Outcome**	**Wave 1**<br>N = 3,012	**Wave 2**<br>N = 611
(Missing)	6 (0.2%)	1 (0.2%)
Belief in God/gods/spiritual forces, n (%)		
One God	717 (23.8%)	143 (23.4%)
More than one god	434 (14.4%)	63 (10.3%)
An impersonal spiritual force	632 (21.0%)	98 (16.1%)
None of these	734 (24.4%)	164 (26.8%)
Unsure	490 (16.3%)	141 (23.1%)
(Missing)	5 (0.2%)	2 (0.3%)
Religious centrality, n (%)		
Agree	960 (31.9%)	148 (24.3%)
Disagree	597 (19.8%)	75 (12.4%)
Not relevant	1,048 (34.8%)	249 (40.7%)
Unsure	400 (13.3%)	138 (22.6%)
(Missing)	7 (0.2%)	0 (0%)
Religious/spiritual comfort, n (%)		
Agree	1,070 (35.5%)	196 (32.1%)
Disagree	539 (17.9%)	75 (12.2%)
Not relevant	1,004 (33.3%)	220 (36.1%)
Unsure	380 (12.6%)	118 (19.3%)
(Missing)	19 (0.6%)	1 (0.2%)
Feel loved by God, n (%)		
Agree	901 (29.9%)	149 (24.4%)
Disagree	686 (22.8%)	110 (18.0%)
Not relevant	961 (31.9%)	227 (37.2%)
Unsure	428 (14.2%)	124 (20.3%)
(Missing)	36 (1.2%)	0 (0.0%)
Feel punished by God, n (%)		
Agree	655 (21.8%)	91 (15.0%)
Disagree	882 (29.3%)	182 (29.9%)
Not relevant	987 (32.8%)	189 (30.9%)
Unsure	455 (15.1%)	143 (23.4%)
(Missing)	33 (1.1%)	5 (0.8%)
Experienced religious criticism, n (%)		
Agree	512 (17.0%)	69 (11.3%)
Disagree	904 (30.0%)	172 (28.2%)
Not relevant	1,154 (38.3%)	248 (40.6%)
Unsure	419 (13.9%)	119 (19.5%)
(Missing)	23 (0.8%)	2 (0.3%)
Faith-sharing, n (%)		
Agree	964 (32.0%)	148 (24.3%)
Disagree	720 (23.9%)	128 (20.9%)
Not relevant	912 (30.3%)	218 (35.7%)
Unsure	409 (13.6%)	117 (19.1%)
(Missing)	7 (0.2%)	0 (0%)

Note. N (%); this table is based on non-imputed data. Cumulative percentages for variables may not add up to 100% due to rounding. Wave 1 characteristics weighted using the Gallup provided sampling weight, ANNUAL_WEIGHT_R2; Wave 2 characteristics weighted accounting for attrition by using the adjusted Wave 1 weight, ANNUAL_WEIGHT_R2, multiplied by the created attrition weight to account for dropout, to maintain nationally representative estimates for Wave 2 characteristics.

Table S15c. Unweighted summary statistics for demographic and childhood variables in Hong Kong by retention status

**Characteristic**	**Attritors–Not Observed in Wave 2** N = 2,146	**Retained–Observed in Wave 2** N = 818
Forgivingness, n (%)		
Always	417 (19.4%)	73 (8.9%)
Often	954 (44.5%)	376 (45.9%)
Rarely	655 (30.5%)	332 (40.6%)
Never	107 (5.0%)	36 (4.4%)
(Missing)	12 (0.6%)	1 (0.2%)
Year of birth, n (%)		
1943 or earlier (current age: 80+ years)	13 (0.6%)	0 (0%)
1943-1953 (current age: 70-79 years)	87 (4.0%)	57 (6.9%)
1953-1963 (current age: 60-69 years)	417 (19.4%)	161 (19.7%)
1963-1973 (current age: 50-59 years)	511 (23.8%)	179 (21.9%)
1973-1983 (current age: 40-49 years)	377 (17.6%)	194 (23.7%)
1983-1993 (current age: 30-39 years)	352 (16.4%)	175 (21.4%)
1993-1998 (current age: 25-29 years)	169 (7.9%)	32 (4.0%)
1998-2005 (current age: 18-24 years)	222 (10.3%)	21 (2.5%)
(Missing)	0 (0%)	0 (0%)
Age of participant		
Mean	46.7	49.3
Standard Deviation	15.5	14.0
Min, Max	18.0, 85.0	20.0, 79.0
Gender, n (%)		
Male	1,010 (47.0%)	347 (42.4%)
Female	1,134 (52.9%)	471 (57.6%)
Other	2 (0.1%)	0 (0%)
(Missing)	0 (0%)	0 (0%)
Respondent marital status, n (%)		
Single/Never been married	491 (22.9%)	257 (31.4%)
Married	1,518 (70.7%)	496 (60.6%)
Separated	14 (0.6%)	5 (0.6%)
Divorced	73 (3.4%)	28 (3.4%)
Widowed	25 (1.2%)	21 (2.5%)
Domestic partner	25 (1.2%)	11 (1.4%)
(Missing)	1 (0.0%)	0 (0%)
Education (years), n (%)		
Up to 8	353 (16.4%)	36 (4.4%)
9-15	1,519 (70.8%)	529 (64.6%)
16+	274 (12.8%)	253 (31.0%)
(Missing)	0 (0%)	0 (0%)
Employment status, n (%)		
Employed for an employer	1,464 (68.2%)	589 (71.9%)
Self-employed	214 (10.0%)	26 (3.2%)
Retired	266 (12.4%)	116 (14.1%)
Student	54 (2.5%)	6 (0.7%)
Homemaker	74 (3.5%)	39 (4.8%)
Unemployed and looking for a job	35 (1.6%)	27 (3.3%)
None of these/Other	24 (1.1%)	13 (1.5%)
(Missing)	15 (0.7%)	4 (0.5%)
Current religious service attendance, n (%)		
More than once a week	215 (10.0%)	15 (1.9%)

Table S15c. Unweighted summary statistics for demographic and childhood variables in Hong Kong by retention status

**Characteristic**	**Attritors–Not Observed in Wave 2** N = 2,146	**Retained–Observed in Wave 2** N = 818
Once a week	465 (21.7%)	83 (10.1%)
One to three times a month	262 (12.2%)	63 (7.8%)
A few times a year	372 (17.3%)	162 (19.8%)
Never	831 (38.7%)	495 (60.4%)
(Missing)	1 (0.0%)	0 (0%)
Immigration status, n (%)		
Born in this country	1,884 (87.8%)	725 (88.5%)
Born in another country	230 (10.7%)	72 (8.8%)
(Missing)	32 (1.5%)	22 (2.6%)
Parental marital status around age 12, n (%)		
Parents were married	1,954 (91.1%)	753 (92.0%)
Parents were divorced	89 (4.2%)	24 (2.9%)
Parents were never married	29 (1.4%)	13 (1.6%)
One or both of them had died	31 (1.4%)	18 (2.2%)
Unsure	41 (1.9%)	11 (1.3%)
(Missing)	1 (0.0%)	0 (0%)
Religious service attendance around age 12, n (%)		
At least once a week	348 (16.2%)	69 (8.4%)
One to three times a month	464 (21.6%)	56 (6.9%)
Less than once a month	505 (23.5%)	228 (27.8%)
Never	825 (38.4%)	466 (56.9%)
(Missing)	4 (0.2%)	0 (0%)
Relationship with mother when growing up, n (%)		
Very good	801 (37.3%)	250 (30.5%)
Somewhat good	829 (38.6%)	323 (39.4%)
Somewhat bad	182 (8.5%)	112 (13.7%)
Very bad	20 (0.9%)	29 (3.6%)
(Does not apply)	311 (14.5%)	105 (12.8%)
(Missing)	3 (0.1%)	1 (0.1%)
Relationship with father when growing up, n (%)		
Very good	664 (30.9%)	175 (21.3%)
Somewhat good	755 (35.2%)	331 (40.4%)
Somewhat bad	264 (12.3%)	127 (15.5%)
Very bad	62 (2.9%)	42 (5.2%)
(Does not apply)	398 (18.5%)	144 (17.6%)
(Missing)	3 (0.2%)	0 (0%)
Felt like an outsider in family when growing up, n (%)		
Yes	539 (25.1%)	117 (14.3%)
No	1,498 (69.8%)	689 (84.2%)
(Missing)	110 (5.1%)	13 (1.6%)
Experienced abuse when growing up, n (%)		
Yes	254 (11.9%)	60 (7.3%)
No	1,888 (88.0%)	759 (92.7%)
(Missing)	4 (0.2%)	0 (0%)
Self-rated health when growing up, n (%)		
Excellent	475 (22.1%)	58 (7.1%)
Very good	798 (37.2%)	256 (31.3%)

Table S15c. Unweighted summary statistics for demographic and childhood variables in Hong Kong by retention status

**Characteristic**	**Attritors–Not Observed in Wave 2** N = 2,146	**Retained–Observed in Wave 2** N = 818
Good	534 (24.9%)	316 (38.6%)
Fair	271 (12.6%)	153 (18.6%)
Poor	59 (2.8%)	32 (3.9%)
(Missing)	9 (0.4%)	4 (0.5%)
Subjective financial status of family growing up, n (%)		
Lived comfortably	780 (36.3%)	108 (13.2%)
Got by	1,037 (48.3%)	473 (57.7%)
Found it difficult	264 (12.3%)	200 (24.4%)
Found it very difficult	49 (2.3%)	33 (4.0%)
(Missing)	17 (0.8%)	5 (0.7%)
Religious affiliation growing up, n (%)		
Christianity	550 (25.6%)	146 (17.8%)
Taoism	55 (2.6%)	21 (2.6%)
Confucianism	9 (0.4%)	0 (0%)
Primal, Animist, or Folk religion	12 (0.6%)	4 (0.5%)
Spiritism	0 (0%)	0 (0%)
Umbanda, Candomblé, and other African-derived religions	0 (0%)	0 (0%)
Chinese folk/traditional religion	78 (3.6%)	30 (3.7%)
Islam	82 (3.8%)	3 (0.4%)
Hinduism	25 (1.2%)	1 (0.2%)
Buddhism	270 (12.6%)	47 (5.7%)
Judaism	16 (0.8%)	0 (0.0%)
Sikhism	4 (0.2%)	0 (0%)
Baha'i	0 (0%)	0 (0%)
Jainism	0 (0%)	1 (0.1%)
Shinto	18 (0.8%)	0 (0%)
Some other religion	0 (0%)	4 (0.5%)
No religion/Atheist/Agnostic	1,026 (47.8%)	560 (68.4%)
(Missing)	1 (0.1%)	0 (0%)

Note. N (%); this table is based on non-imputed data. Cumulative percentages for variables may not add up to 100% due to rounding.

Table S15d. Unweighted summary statistics for Wave 1 outcome variables in Hong Kong by retention status.

**Outcome**	**Attritors–Not Observed in Wave 2**<br>N = 2,146	**Retained–Observed in Wave 2**<br>N = 818
Secure flourishing index		
Mean	7.3	6.5
Standard Deviation	1.8	1.6
Min, Max	0.0, 10.0	0.7, 10.0
(Missing)	143 (6.7%)	32 (3.9%)
Flourishing index		
Mean	7.4	6.5
Standard Deviation	1.8	1.6
Min, Max	0.0, 10.0	0.7, 10.0
(Missing)	112 (5.2%)	29 (3.6%)
Happiness & life satisfaction		
Mean	7.3	6.4
Standard Deviation	2.0	1.9
Min, Max	0.0, 10.0	0.0, 10.0
(Missing)	38 (1.8%)	16 (1.9%)
Physical & mental health		
Mean	7.4	6.5
Standard Deviation	2.0	1.7
Min, Max	0.0, 10.0	0.0, 10.0
(Missing)	12 (0.6%)	7 (0.8%)
Meaning & purpose		
Mean	7.4	6.6
Standard Deviation	1.9	1.8
Min, Max	0.0, 10.0	0.0, 10.0
(Missing)	26 (1.2%)	8 (1.0%)
Character & virtue		
Mean	7.4	6.6
Standard Deviation	1.8	1.7
Min, Max	0.0, 10.0	0.0, 10.0
(Missing)	9 (0.4%)	3 (0.3%)
Close social relationships		
Mean	7.3	6.5
Standard Deviation	2.0	1.9
Min, Max	0.0, 10.0	0.0, 10.0
(Missing)	51 (2.4%)	13 (1.6%)
Financial & material security		
Mean	7.0	6.3
Standard Deviation	2.4	2.4
Min, Max	0.0, 10.0	0.0, 10.0
(Missing)	38 (1.8%)	3 (0.4%)
Happiness		
Mean	7.4	6.5
Standard Deviation	2.0	1.8
Min, Max	0.0, 10.0	0.0, 10.0
(Missing)	3 (0.1%)	2 (0.3%)
Life satisfaction		
Mean	7.2	6.3
Standard Deviation	2.1	2.0
Min, Max	0.0, 10.0	0.0, 10.0

Table S15d. Unweighted summary statistics for Wave 1 outcome variables in Hong Kong by retention status.

**Outcome**	**Attritors–Not Observed in Wave 2** N = 2,146	**Retained–Observed in Wave 2** N = 818
(Missing)	35 (1.6%)	16 (1.9%)
Current life evaluation		
Mean	7.1	6.0
Standard Deviation	2.0	1.8
Min, Max	0.0, 10.0	0.0, 10.0
(Missing)	1 (<0.1%)	0 (0%)
Future life evaluation		
Mean	7.4	6.4
Standard Deviation	2.0	1.8
Min, Max	0.0, 10.0	0.0, 10.0
(Missing)	35 (1.6%)	3 (0.4%)
Optimism		
Mean	7.4	6.7
Standard Deviation	2.1	2.1
Min, Max	0.0, 10.0	0.0, 10.0
(Missing)	9 (0.4%)	1 (<0.1%)
Freedom to pursue what's important		
Mean	7.4	6.8
Standard Deviation	2.1	2.0
Min, Max	0.0, 10.0	0.0, 10.0
(Missing)	12 (0.5%)	3 (0.4%)
Inner peace, n (%)		
Always	729 (34.0%)	170 (20.8%)
Often	1,185 (55.2%)	527 (64.4%)
Rarely	208 (9.7%)	121 (14.8%)
Never	22 (1.0%)	1 (0.1%)
(Missing)	2 (0.1%)	0 (0%)
Life balance, n (%)		
Always	586 (27.3%)	118 (14.4%)
Often	1,136 (53.0%)	513 (62.6%)
Rarely	393 (18.3%)	181 (22.1%)
Never	21 (1.0%)	6 (0.7%)
(Missing)	10 (0.5%)	1 (0.1%)
Sense of mastery, n (%)		
Always	646 (30.1%)	147 (17.9%)
Often	1,200 (55.9%)	535 (65.3%)
Rarely	265 (12.3%)	135 (16.5%)
Never	29 (1.4%)	0 (0%)
(Missing)	6 (0.3%)	2 (0.2%)
Meaningful activities		
Mean	7.4	6.7
Standard Deviation	2.0	1.9
Min, Max	0.0, 10.0	0.0, 10.0
(Missing)	25 (1.2%)	8 (1.0%)
Understanding purpose		
Mean	7.4	6.5
Standard Deviation	2.2	2.0
Min, Max	0.0, 10.0	0.0, 10.0
(Missing)	1 (<0.1%)	0 (0%)

Table S15d. Unweighted summary statistics for Wave 1 outcome variables in Hong Kong by retention status.

**Outcome**	**Attritors–Not Observed in Wave 2** N = 2,146	**Retained–Observed in Wave 2** N = 818
Self-rated mental health		
Mean	7.3	6.6
Standard Deviation	2.2	2.0
Min, Max	0.0, 10.0	0.0, 10.0
(Missing)	12 (0.6%)	7 (0.8%)
Traumatic distress, n (%)		
A lot	259 (12.1%)	53 (6.5%)
Some	587 (27.4%)	208 (25.5%)
Not very much	691 (32.2%)	264 (32.3%)
Not at all	607 (28.3%)	291 (35.6%)
(Missing)	3 (0.1%)	2 (0.2%)
Depression symptoms composite, n (%)	1,062 (49.9%)	283 (34.8%)
(Missing)	17 (0.8%)	5 (0.7%)
Depression – feel hopeless, n (%)		
Nearly every day	194 (9.0%)	30 (3.6%)
More than half the days	375 (17.5%)	111 (13.5%)
Several days	999 (46.5%)	414 (50.6%)
Not at all	563 (26.3%)	259 (31.6%)
(Missing)	15 (0.7%)	5 (0.7%)
Depression – loss of interest, n (%)		
Nearly every day	355 (16.6%)	43 (5.3%)
More than half the days	679 (31.6%)	218 (26.6%)
Several days	760 (35.4%)	379 (46.3%)
Not at all	351 (16.3%)	178 (21.8%)
(Missing)	1 (0.1%)	0 (0%)
Anxiety symptoms composite, n (%)	665 (31.4%)	177 (21.6%)
(Missing)	31 (1.4%)	0 (0%)
Anxiety – feel on edge, n (%)		
Nearly every day	169 (7.9%)	43 (5.2%)
More than half the days	398 (18.5%)	122 (14.9%)
Several days	981 (45.7%)	399 (48.8%)
Not at all	576 (26.9%)	254 (31.1%)
(Missing)	23 (1.1%)	0 (0%)
Anxiety – cannot stop worrying, n (%)		
Nearly every day	180 (8.4%)	39 (4.8%)
More than half the days	355 (16.5%)	99 (12.1%)
Several days	848 (39.5%)	314 (38.3%)
Not at all	755 (35.2%)	367 (44.8%)
(Missing)	8 (0.4%)	0 (0%)
Suffering, n (%)		
A lot	293 (13.6%)	64 (7.8%)
Some	781 (36.4%)	301 (36.8%)
Not very much	768 (35.8%)	341 (41.6%)
Not at all	304 (14.2%)	112 (13.7%)
(Missing)	0 (0.0%)	1 (0.1%)
Relationship contentment		
Mean	7.4	6.6
Standard Deviation	2.1	2.0
Min, Max	0.0, 10.0	0.0, 10.0

Table S15d. Unweighted summary statistics for Wave 1 outcome variables in Hong Kong by retention status.

**Outcome**	**Attritors–Not Observed in Wave 2** N = 2,146	**Retained–Observed in Wave 2** N = 818
(Missing)	22 (1.0%)	4 (0.5%)
Relationship satisfaction		
Mean	7.3	6.5
Standard Deviation	2.2	2.0
Min, Max	0.0, 10.0	0.0, 10.0
(Missing)	32 (1.5%)	9 (1.1%)
Social support		
Mean	6.7	5.4
Standard Deviation	2.6	2.4
Min, Max	0.0, 10.0	0.0, 10.0
(Missing)	1 (<0.1%)	0 (0%)
Intimate/close friend, n (%)		
Yes	1,834 (85.5%)	631 (77.0%)
No	307 (14.3%)	188 (23.0%)
(Missing)	5 (0.2%)	0 (0%)
Government approval, n (%)		
Strongly approve	557 (26.0%)	31 (3.8%)
Somewhat approve	735 (34.2%)	219 (26.8%)
Neither approve nor disapprove	440 (20.5%)	282 (34.5%)
Somewhat disapprove	226 (10.5%)	160 (19.6%)
Strongly disapprove	183 (8.5%)	126 (15.4%)
(Missing)	5 (0.2%)	0 (0%)
Say in government, n (%)		
Agree	1,135 (52.9%)	246 (30.0%)
Disagree	558 (26.0%)	309 (37.8%)
Unsure	449 (20.9%)	262 (32.1%)
(Missing)	5 (0.2%)	1 (0.2%)
Belonging in country		
Mean	7.6	6.9
Standard Deviation	2.1	2.3
Min, Max	0.0, 10.0	0.0, 10.0
(Missing)	5 (0.2%)	0 (0%)
City/place satisfaction, n (%)		
Satisfied	1,569 (73.1%)	494 (60.3%)
Dissatisfied	410 (19.1%)	195 (23.8%)
Unsure	166 (7.7%)	130 (15.8%)
(Missing)	2 (0.1%)	0 (0%)
Trust within country, n (%)		
All people	261 (12.2%)	3 (0.4%)
Most people	879 (40.9%)	260 (31.8%)
Some people	645 (30.1%)	351 (42.9%)
Not very many people	324 (15.1%)	194 (23.7%)
None	35 (1.6%)	10 (1.2%)
(Missing)	2 (0.1%)	0 (0%)
Number of children		
Mean	0.6	0.4
Standard Deviation	1.0	0.8
Min, Max	0.0, 12.0	0.0, 11.0
(Missing)	34 (1.6%)	4 (0.5%)

Table S15d. Unweighted summary statistics for Wave 1 outcome variables in Hong Kong by retention status.

Outcome	Attritors–Not Observed in Wave 2<br>N = 2,146	Retained–Observed in Wave 2<br>N = 818
Community participation, n (%)		
More than once a week	219 (10.2%)	11 (1.4%)
Once a week	341 (15.9%)	51 (6.3%)
One to three times a month	332 (15.5%)	59 (7.2%)
A few times a year	408 (19.0%)	199 (24.3%)
Never	840 (39.2%)	498 (60.8%)
(Missing)	5 (0.2%)	0 (0%)
Religious attendance, n (%)		
More than once a week	215 (10.0%)	15 (1.9%)
Once a week	465 (21.7%)	83 (10.1%)
One to three times a month	262 (12.2%)	63 (7.8%)
A few times a year	372 (17.3%)	162 (19.8%)
Never	831 (38.7%)	495 (60.4%)
(Missing)	1 (0.0%)	0 (0%)
Loneliness		
Mean	3.2	3.6
Standard Deviation	2.6	2.5
Min, Max	0.0, 10.0	0.0, 10.0
(Missing)	18 (0.8%)	1 (0.1%)
Perceived discrimination, n (%)		
Always	238 (11.1%)	20 (2.5%)
Often	440 (20.5%)	116 (14.2%)
Rarely	817 (38.1%)	420 (51.3%)
Never	646 (30.1%)	262 (32.0%)
(Missing)	5 (0.2%)	0 (0%)
Orientation to promote good		
Mean	7.5	6.7
Standard Deviation	1.9	1.9
Min, Max	0.0, 10.0	0.0, 10.0
Delayed gratification		
Mean	7.3	6.5
Standard Deviation	2.0	1.8
Min, Max	0.0, 10.0	0.0, 10.0
(Missing)	9 (0.4%)	3 (0.3%)
Hope		
Mean	7.5	6.6
Standard Deviation	2.0	2.1
Min, Max	0.0, 10.0	0.0, 10.0
(Missing)	7 (0.3%)	0 (<0.1%)
Gratitude		
Mean	7.2	6.2
Standard Deviation	2.2	2.2
Min, Max	0.0, 10.0	0.0, 10.0
(Missing)	10 (0.4%)	1 (<0.1%)
Showing love/care		
Mean	6.8	5.5
Standard Deviation	2.5	2.5
Min, Max	0.0, 10.0	0.0, 10.0
(Missing)	2 (<0.1%)	2 (0.3%)

Table S15d. Unweighted summary statistics for Wave 1 outcome variables in Hong Kong by retention status.

**Outcome**	**Attritors–Not Observed in Wave 2** N = 2,146	**Retained–Observed in Wave 2** N = 818
Forgivingness, n (%)		
Always	417 (19.4%)	73 (8.9%)
Often	954 (44.5%)	376 (45.9%)
Rarely	655 (30.5%)	332 (40.6%)
Never	107 (5.0%)	36 (4.4%)
(Missing)	12 (0.6%)	1 (0.2%)
Charitable giving, n (%)		
Yes	1,206 (56.2%)	287 (35.0%)
No	930 (43.4%)	532 (65.0%)
(Missing)	10 (0.5%)	0 (0%)
Helping strangers, n (%)		
Yes	1,476 (68.8%)	437 (53.3%)
No	663 (30.9%)	382 (46.7%)
(Missing)	7 (0.3%)	0 (0%)
Volunteering, n (%)		
Yes	907 (42.2%)	106 (12.9%)
No	1,228 (57.2%)	713 (87.1%)
(Missing)	11 (0.5%)	0 (0%)
Self-rated physical health		
Mean	7.4	6.3
Standard Deviation	2.0	1.8
Min, Max	0.0, 10.0	0.0, 10.0
Health problems, n (%)		
Yes	470 (21.9%)	140 (17.1%)
No	1,674 (78.0%)	679 (82.9%)
(Missing)	2 (0.1%)	0 (0%)
Pain in past 4 weeks, n (%)		
A lot	256 (11.9%)	60 (7.3%)
Some	718 (33.5%)	298 (36.4%)
Not very much	764 (35.6%)	315 (38.5%)
None at all	395 (18.4%)	145 (17.8%)
(Missing)	13 (0.6%)	0 (0%)
Number of cigarettes per day		
Mean	2.4	0.9
Standard Deviation	4.4	3.2
Min, Max	0.0, 30.0	0.0, 20.0
(Missing)	22 (1.0%)	6 (0.8%)
Number of drinks per week		
Mean	1.9	0.9
Standard Deviation	3.1	2.1
Min, Max	0.0, 56.0	0.0, 31.0
(Missing)	18 (0.8%)	11 (1.4%)
Days exercise per week		
Mean	2.3	1.9
Standard Deviation	2.2	2.2
Min, Max	0.0, 7.0	0.0, 7.0
(Missing)	5 (0.2%)	0 (0%)
Financial security		
Mean	7.0	6.2

Table S15d. Unweighted summary statistics for Wave 1 outcome variables in Hong Kong by retention status.

**Outcome**	**Attritors–Not Observed in Wave 2** N = 2,146	**Retained–Observed in Wave 2** N = 818
Standard Deviation	2.7	2.7
Min, Max	0.0, 10.0	0.0, 10.0
Material security		
Mean	7.1	6.4
Standard Deviation	2.4	2.4
Min, Max	0.0, 10.0	0.0, 10.0
(Missing)	37 (1.7%)	3 (0.4%)
Educational attainment (16+ years), n (%)		
Up to 8	353 (16.4%)	36 (4.4%)
9-15	1,519 (70.8%)	529 (64.6%)
16+	274 (12.8%)	253 (31.0%)
(Missing)	0 (0%)	0 (0%)
Currently employed, n (%)		
Employed for an employer	1,464 (68.2%)	589 (71.9%)
Self-employed	214 (10.0%)	26 (3.2%)
Retired	266 (12.4%)	116 (14.1%)
Student	54 (2.5%)	6 (0.7%)
Homemaker	74 (3.5%)	39 (4.8%)
Unemployed and looking for a job	35 (1.6%)	27 (3.3%)
None of these/Other	24 (1.1%)	13 (1.5%)
(Missing)	15 (0.7%)	4 (0.5%)
Financially comfortable/getting by, n (%)		
Living comfortably on present income	711 (33.1%)	120 (14.7%)
Getting by on present income	1,079 (50.3%)	496 (60.6%)
Finding it difficult on present income	283 (13.2%)	167 (20.5%)
Finding it very difficult on present income	74 (3.4%)	35 (4.3%)
(Missing)	1 (0.0%)	0 (0%)
Own home, n (%)		
Someone in this household owns this home	1,422 (66.3%)	456 (55.7%)
Someone in this household rents this home	471 (21.9%)	212 (25.9%)
Both	55 (2.6%)	14 (1.7%)
Neither	177 (8.2%)	135 (16.5%)
Rent	0 (0%)	0 (0%)
Own	0 (0%)	0 (0%)
Something else	0 (0%)	0 (0%)
(Missing)	21 (1.0%)	2 (0.2%)
Religious/spiritual connection, n (%)		
Always	447 (20.8%)	37 (4.6%)
Often	673 (31.3%)	194 (23.7%)
Rarely	604 (28.2%)	331 (40.4%)
Never	422 (19.6%)	256 (31.3%)
(Missing)	1 (0.0%)	1 (0.1%)
Belief in life after death, n (%)		
Yes	1,033 (48.1%)	320 (39.1%)
No	607 (28.3%)	133 (16.3%)
Unsure	505 (23.6%)	363 (44.4%)
(Missing)	1 (0.0%)	2 (0.3%)
Transformative religious experience, n (%)		
Yes	1,013 (47.2%)	231 (28.3%)

Table S15d. Unweighted summary statistics for Wave 1 outcome variables in Hong Kong by retention status.

**Outcome**	**Attritors–Not Observed in Wave 2** N = 2,146	**Retained–Observed in Wave 2** N = 818
No	1,131 (52.7%)	585 (71.4%)
(Missing)	3 (0.1%)	2 (0.3%)
Religious reading or listening, n (%)		
More than once a day	254 (11.8%)	15 (1.8%)
About once a day	453 (21.1%)	48 (5.8%)
Sometimes	653 (30.4%)	277 (33.8%)
Never	785 (36.6%)	479 (58.5%)
(Missing)	2 (0.1%)	0 (0%)
Prayer or meditation, n (%)		
More than once a day	327 (15.2%)	39 (4.8%)
About once a day	437 (20.4%)	75 (9.1%)
Sometimes	665 (31.0%)	305 (37.3%)
Never	711 (33.1%)	400 (48.8%)
(Missing)	6 (0.3%)	0 (0%)
Belief in God/gods/spiritual forces, n (%)		
One God	538 (25.1%)	166 (20.2%)
More than one god	332 (15.4%)	94 (11.5%)
An impersonal spiritual force	484 (22.6%)	135 (16.5%)
None of these	516 (24.1%)	207 (25.3%)
Unsure	271 (12.6%)	217 (26.5%)
(Missing)	4 (0.2%)	0 (0%)
Religious centrality, n (%)		
Agree	771 (35.9%)	167 (20.4%)
Disagree	502 (23.4%)	80 (9.8%)
Not relevant	639 (29.8%)	400 (48.9%)
Unsure	229 (10.7%)	169 (20.6%)
(Missing)	5 (0.2%)	1 (0.2%)
Religious/spiritual comfort, n (%)		
Agree	799 (37.2%)	251 (30.7%)
Disagree	458 (21.4%)	67 (8.1%)
Not relevant	648 (30.2%)	345 (42.2%)
Unsure	225 (10.5%)	152 (18.6%)
(Missing)	15 (0.7%)	3 (0.4%)
Feel loved by God, n (%)		
Agree	697 (32.5%)	186 (22.7%)
Disagree	556 (25.9%)	114 (14.0%)
Not relevant	624 (29.1%)	327 (39.9%)
Unsure	244 (11.4%)	182 (22.2%)
(Missing)	25 (1.2%)	10 (1.2%)
Feel punished by God, n (%)		
Agree	543 (25.3%)	96 (11.8%)
Disagree	648 (30.2%)	218 (26.6%)
Not relevant	655 (30.5%)	320 (39.1%)
Unsure	277 (12.9%)	174 (21.2%)
(Missing)	23 (1.1%)	10 (1.2%)
Experienced religious criticism, n (%)		
Agree	437 (20.4%)	61 (7.5%)
Disagree	681 (31.7%)	206 (25.1%)
Not relevant	743 (34.6%)	399 (48.7%)

Table S15d. Unweighted summary statistics for Wave 1 outcome variables in Hong Kong by retention status.

**Outcome**	**Attritors–Not Observed in Wave 2**<br>N = 2,146	**Retained–Observed in Wave 2**<br>N = 818
Unsure	268 (12.5%)	147 (17.9%)
(Missing)	17 (0.8%)	6 (0.7%)
Faith-sharing, n (%)		
Agree	764 (35.6%)	179 (21.9%)
Disagree	548 (25.5%)	158 (19.3%)
Not relevant	568 (26.5%)	336 (41.0%)
Unsure	262 (12.2%)	143 (17.5%)
(Missing)	4 (0.2%)	2 (0.3%)

Note. N (%); this table is based on non-imputed data. Cumulative percentages for variables may not add up to 100% due to rounding.

Table S15e. Summary of fitted attrition model in Hong Kong

Characteristic	Odds Ratio	95% CI	p-value
**ANNUAL_WEIGHT_R2**	1.32	1.12, 1.55	0.001
**Happiness & life satisfaction**	0.86	0.67, 1.12	0.271
**Physical & mental health**	0.79	0.61, 1.03	0.087
**Meaning & purpose**	1.07	0.83, 1.38	0.591
**Character & virtue**	0.85	0.69, 1.05	0.132
**Close social relationships**	1.05	0.84, 1.31	0.685
**Financial & material security**	0.95	0.81, 1.12	0.577
**Extraversion**	0.80	0.70, 0.91	8.61e-04
**Openness to experience**	0.89	0.78, 1.02	0.097
**Agreeableness**	0.98	0.85, 1.13	0.760
**Conscientiousness**	1.17	1.01, 1.35	0.040
**Neuroticism**	0.97	0.80, 1.17	0.747
**Depression symptoms composite**	0.94	0.82, 1.09	0.422
**Anxiety symptoms composite**	0.83	0.71, 0.97	0.021
**Loneliness**	0.88	0.74, 1.04	0.123
**Days exercise per week**	0.93	0.81, 1.05	0.248
**Year of birth (age group)**			
1963-1973 (current age: 50-59 years)	—	—	
1973-1983 (current age: 40-49 years)	1.22	0.90, 1.66	0.194
1983-1993 (current age: 30-39 years)	0.90	0.64, 1.26	0.528
1953-1963 (current age: 60-69 years)	0.74	0.43, 1.28	0.282
1998-2005 (current age: 18-24 years)	0.21	0.11, 0.37	1.18e-07
1993-1998 (current age: 25-29 years)	0.37	0.22, 0.63	2.68e-04
1943-1953 (current age: 70-79 years)	1.21	0.42, 3.51	0.726
1943 or earlier (current age: 80+ years)	0.00	0.00, 0.00	6.64e-68
**Gender of respondent**			
Female	—	—	
Male	0.80	0.62, 1.04	0.097
Other	0.00	0.00, 0.00	3.63e-52
**Marital status**			
Married	—	—	
Single/Never been married	1.27	0.90, 1.79	0.169
Divorced	1.16	0.48, 2.81	0.741
Domestic partner	1.00	0.42, 2.39	1.000
Widowed	1.95	0.47, 8.12	0.361
Separated	1.38	0.23, 8.30	0.726
**Employment status**			
Employed for an employer	—	—	
Self-employed	0.34	0.17, 0.68	0.003
Retired	0.80	0.39, 1.66	0.555
Homemaker	1.16	0.59, 2.26	0.671
Student	0.85	0.34, 2.16	0.735
Unemployed and looking for a job	1.59	0.70, 3.63	0.266
None of these/Other	1.13	0.38, 3.39	0.827
**Religious attendance**			
Never	—	—	
A few times a year	0.83	0.60, 1.15	0.266
Once a week	0.54	0.35, 0.81	0.004
One to three times a month	0.88	0.57, 1.37	0.579
More than once a week	0.34	0.14, 0.83	0.017
**Educational attainment (16+ years)**			
9-15	—	—	

Table S15e. Summary of fitted attrition model in Hong Kong

**Characteristic**	**Odds Ratio**	**95% CI**	**p-value**
16+	2.45	1.81, 3.33	7.66e-09
Up to 8	0.22	0.11, 0.46	5.83e-05
**Born in This country**			
Born in this country	—	—	
Born in another country	0.76	0.48, 1.20	0.242
**Urbanicity**			
A large city	—	—	
A suburb of a large city	0.79	0.55, 1.13	0.203
A small town or village	0.72	0.45, 1.15	0.169
A rural area or on a farm	0.39	0.13, 1.15	0.088
**Monthly household income**			
Hong Kong: 30,001 – 40,000 dollars	—	—	
Hong Kong: 50,001 – 60,000 dollars	0.77	0.47, 1.25	0.287
Hong Kong: 20,001 – 30,000 dollars	0.68	0.41, 1.14	0.141
Hong Kong: 70,001 – 80,000 dollars	0.54	0.32, 0.89	0.017
Hong Kong: 60,001 – 70,000 dollars	0.82	0.50, 1.35	0.437
Hong Kong: 45,001 – 50,000 dollars	0.95	0.59, 1.54	0.841
Hong Kong: 40,001 – 45,000 dollars	0.64	0.38, 1.09	0.099
Hong Kong: 80,001 – 100,000 dollars	0.72	0.41, 1.24	0.236
Hong Kong: More than 100,000 dollars	0.69	0.38, 1.24	0.212
Hong Kong: 15,001 – 20,000 dollars	0.76	0.42, 1.35	0.343
Hong Kong: 10,001 – 15,000 dollars	1.44	0.68, 3.06	0.345
Hong Kong: 5,001 – 10,000 dollars	0.63	0.21, 1.87	0.401
Hong Kong: 5,000 dollars or less	0.61	0.18, 2.09	0.435
(None/No household income)	0.54	0.09, 3.12	0.493

Abbreviations: CI = Confidence Interval, OR = Odds Ratio

Notes. N=3012; attrition weights were estimated using the 'survey::svyglm(family=quasibinomial('logit'))' function. All continuous predictors were standardized and all categorical predictors used the most common category as the reference group. Reported p-values are based on the fitted regression model and no adjustments for multiple testing were done within this table.

Table S15f. Summary of principal components in Hong Kong

PC	Percent Explained by each PC	Cumulative Percent Explained
1	33.35	33.35
2	11.30	44.65
3	6.98	51.63
4	3.54	55.17
5	1.91	57.07
6	1.56	58.63
**7**	**1.50**	**60.14**
8	1.42	61.56
9	1.30	62.85
10	1.22	64.07
11	1.18	65.25
12	1.16	66.41
13	1.14	67.55
14	1.09	68.64
15	1.05	69.69
16	0.99	70.67
17	0.97	71.64
18	0.94	72.59
19	0.91	73.49
20	0.90	74.40

Notes. N=3012; PCA was conducted using 'survey::svyprcomp(.)' function using all available contemporaneous exposures at wave 1. All PCs were standardized prior to being used as predictors. The bolded row represented the number of retained components for analysis was 7.

Table S15g. Associations of forgivingness with adult well-being and other outcomes at Wave 2 in Hong Kong

Outcome	Model 1: Demographic and Childhood Variables as Covariates					Model 2: Demographic, Childhood, and Other Wave 1 Confounding Variables (Via Principal Components) as Covariates				
	RR	ES	95% CI	SE	p-value	RR	ES	95% CI	SE	p-value
Human Flourishing										
Secure flourishing index		0.06	(0.01,0.12)	0.03	0.032*		0.02	(-0.04,0.07)	0.03	0.580
Flourishing index		0.08	(0.02,0.14)	0.03	0.009*		0.03	(-0.03,0.09)	0.03	0.291
Happiness & life satisfaction		0.06	(-0.01,0.13)	0.03	0.083		0.02	(-0.04,0.09)	0.03	0.475
Physical & mental health		0.06	(-0.02,0.14)	0.04	0.137		0.03	(-0.05,0.10)	0.04	0.448
Meaning & purpose		0.04	(-0.03,0.12)	0.04	0.250		0.01	(-0.07,0.08)	0.04	0.853
Character & virtue		0.09	(0.01,0.16)	0.04	0.032*		0.05	(-0.02,0.13)	0.04	0.160
Close social relationships		0.04	(-0.04,0.11)	0.04	0.346		-0.00	(-0.08,0.08)	0.04	0.930
Financial & material security		-0.02	(-0.09,0.06)	0.04	0.664		-0.04	(-0.11,0.03)	0.04	0.302
Psychological Well-Being										
Happiness (a)		0.05	(-0.04,0.13)	0.04	0.284		0.02	(-0.06,0.10)	0.04	0.630
Life satisfaction (a)		0.05	(-0.02,0.12)	0.04	0.195		0.02	(-0.05,0.09)	0.04	0.612
Current life evaluation		0.01	(-0.07,0.09)	0.04	0.788		-0.02	(-0.10,0.06)	0.04	0.598
Future life evaluation		0.03	(-0.06,0.12)	0.04	0.513		-0.00	(-0.08,0.08)	0.04	0.996
Optimism		0.04	(-0.03,0.12)	0.04	0.236		0.01	(-0.05,0.08)	0.03	0.697
Freedom to pursue what's important		0.01	(-0.07,0.08)	0.04	0.887		-0.02	(-0.09,0.04)	0.03	0.463
Inner peace	1.02		(0.99,1.05)	0.02	0.248	1.01		(0.98,1.04)	0.01	0.434
Life balance	1.02		(0.99,1.05)	0.02	0.271	1.01		(0.98,1.04)	0.02	0.513
Sense of mastery	1.01		(0.98,1.04)	0.01	0.504	1.00		(0.98,1.03)	0.01	0.761
Meaningful activities (c)		0.03	(-0.06,0.12)	0.04	0.506		0.00	(-0.08,0.09)	0.04	0.944
Understanding purpose (c)		0.04	(-0.02,0.11)	0.03	0.215		0.01	(-0.06,0.07)	0.03	0.809
Self-rated mental health (b)		0.05	(-0.01,0.12)	0.03	0.108		0.03	(-0.04,0.09)	0.03	0.384
Psychological Distress										
Traumatic distress	1.01		(0.97,1.05)	0.02	0.773	1.01		(0.97,1.05)	0.02	0.734
Depression symptoms composite	0.99		(0.96,1.02)	0.01	0.572	1.00		(0.97,1.02)	0.01	0.807
Depression – feel hopeless	0.99		(0.97,1.02)	0.01	0.675	1.00		(0.97,1.02)	0.01	0.802
Depression – loss of interest	0.98		(0.96,1.01)	0.01	0.301	0.99		(0.96,1.02)	0.01	0.389
Anxiety symptoms composite	0.99		(0.97,1.02)	0.01	0.546	1.00		(0.97,1.02)	0.01	0.721
Anxiety – feel on edge	1.00		(0.96,1.03)	0.02	0.760	1.00		(0.97,1.03)	0.02	0.845
Anxiety – cannot stop worrying	0.99		(0.97,1.02)	0.01	0.686	1.00		(0.97,1.02)	0.01	0.832
Suffering	0.99		(0.96,1.02)	0.02	0.467	0.99		(0.96,1.02)	0.02	0.598
Social Well-Being										
Relationship contentment (e)		0.02	(-0.06,0.10)	0.04	0.635		-0.01	(-0.10,0.08)	0.05	0.764
Relationship satisfaction (e)		0.04	(-0.03,0.12)	0.04	0.298		0.01	(-0.07,0.08)	0.04	0.843
Social support		0.03	(-0.05,0.10)	0.04	0.510		0.00	(-0.07,0.07)	0.04	0.960
Intimate/close friend	1.01		(0.98,1.04)	0.02	0.374	1.01		(0.98,1.04)	0.01	0.479
Government approval	1.02		(0.99,1.06)	0.02	0.192	1.01		(0.98,1.04)	0.02	0.496
Say in government	1.01		(0.98,1.04)	0.02	0.634	1.00		(0.97,1.03)	0.02	0.895
Belonging in country		0.02	(-0.05,0.09)	0.04	0.493		0.00	(-0.06,0.07)	0.03	0.989
City/place satisfaction	1.01		(0.98,1.05)	0.02	0.517	1.00		(0.97,1.04)	0.02	0.824
Trust within country	1.01		(0.98,1.04)	0.02	0.553	1.00		(0.97,1.03)	0.01	0.865
Social Participation										
Ever been married	1.00		(0.98,1.02)	0.01	0.974	1.00		(0.98,1.02)	0.01	0.976
Currently divorced	1.00		(0.99,1.01)	0.00	0.966	1.00		(0.99,1.01)	0.00	0.880
Number of children		0.01	(-0.05,0.07)	0.03	0.669		-0.00	(-0.06,0.06)	0.03	0.984
Weekly+ community participation	1.00		(0.98,1.03)	0.01	0.803	1.00		(0.98,1.03)	0.01	0.989
Weekly+ religious attendance	1.02		(1.00,1.04)	0.01	0.124	1.02		(1.00,1.05)	0.01	0.089
Social Distress										
Loneliness		-0.00	(-0.08,0.07)	0.04	0.934		0.02	(-0.06,0.10)	0.04	0.611
Perceived discrimination	1.03		(1.00,1.06)	0.01	0.079	1.02		(0.99,1.06)	0.02	0.115
Character & Prosocial Behavior										
Orientation to promote good (d)		0.05	(-0.02,0.13)	0.04	0.169		0.02	(-0.05,0.09)	0.04	0.498
Delayed gratification (d)		0.09	(0.01,0.17)	0.04	0.025*		0.07	(-0.01,0.14)	0.04	0.108
Hope		0.06	(-0.01,0.13)	0.04	0.100		0.03	(-0.04,0.09)	0.04	0.480
Gratitude		0.08	(0.01,0.15)	0.03	0.023*		0.05	(-0.01,0.12)	0.03	0.115
Showing love/care		0.04	(-0.03,0.11)	0.04	0.307		0.01	(-0.06,0.08)	0.04	0.732
Forgivingness	1.13		(1.09,1.17)	0.02	6.47e-08***	1.13		(1.09,1.17)	0.02	1.33e-07***
Charitable giving	1.02		(0.99,1.06)	0.02	0.237	1.02		(0.98,1.05)	0.02	0.409
Helping strangers	1.01		(0.97,1.05)	0.02	0.778	1.00		(0.96,1.04)	0.02	0.875
Volunteering	1.00		(0.98,1.03)	0.01	0.745	1.00		(0.97,1.03)	0.01	0.871
Physical Health & Health Behavior										
Self-rated physical health (b)		0.04	(-0.05,0.13)	0.05	0.376		0.02	(-0.07,0.11)	0.05	0.694
Health problems	1.01		(0.98,1.04)	0.02	0.543	1.01		(0.98,1.04)	0.02	0.637
Pain in past 4 weeks	1.00		(0.96,1.04)	0.02	0.972	1.00		(0.96,1.04)	0.02	0.942
Daily smoker	1.01		(0.97,1.05)	0.02	0.622	1.00		(0.97,1.04)	0.02	0.902
Number of drinks per week		0.01	(-0.06,0.07)	0.03	0.851		-0.01	(-0.08,0.06)	0.04	0.803
Days exercise per week		0.04	(-0.04,0.12)	0.04	0.330		0.03	(-0.05,0.11)	0.04	0.496
Socioeconomic Outcomes										
Financial security (f)		-0.02	(-0.11,0.07)	0.05	0.650		-0.04	(-0.13,0.05)	0.04	0.353
Material security (f)		-0.00	(-0.08,0.07)	0.04	0.899		-0.02	(-0.08,0.05)	0.03	0.647
Educational attainment (16+ years)	1.01		(0.98,1.04)	0.01	0.512	1.01		(0.98,1.04)	0.01	0.522
Currently employed	1.00		(0.97,1.02)	0.01	0.744	1.00		(0.97,1.03)	0.01	0.928
Financially comfortable/getting by	1.01		(0.97,1.04)	0.02	0.753	1.00		(0.97,1.03)	0.02	0.827
Own home	1.01		(0.97,1.05)	0.02	0.644	1.01		(0.97,1.04)	0.02	0.638
Income – top quintile	1.01		(0.99,1.03)	0.01	0.283	1.01		(0.99,1.03)	0.01	0.388
Religion & Spirituality										
Religious/spiritual connection	1.04		(1.01,1.08)	0.02	0.012*	1.04		(1.01,1.07)	0.02	0.018*
Belief in life after death	1.00		(0.97,1.04)	0.02	0.868	1.00		(0.97,1.04)	0.02	0.903
Transformative religious experience	1.03		(1.00,1.07)	0.02	0.088	1.03		(0.99,1.06)	0.02	0.140
Religious reading or listening	1.02		(1.00,1.04)	0.01	0.090	1.02		(1.00,1.04)	0.01	0.116
Prayer or meditation	1.02		(0.99,1.05)	0.01	0.175	1.02		(0.99,1.04)	0.01	0.266
Belief in God/gods/spiritual forces	1.01		(0.98,1.04)	0.02	0.553	1.01		(0.98,1.04)	0.02	0.594
Religious centrality	1.02		(1.00,1.05)	0.01	0.063	1.02		(0.99,1.04)	0.01	0.149
Religious/spiritual comfort	1.01		(0.98,1.04)	0.02	0.544	1.01		(0.98,1.04)	0.02	0.585
Feel loved by God	1.02		(0.99,1.05)	0.02	0.225	1.02		(0.98,1.05)	0.02	0.313
Feel punished by God	1.01		(0.98,1.04)	0.02	0.552	1.01		(0.98,1.04)	0.02	0.574
Experienced religious criticism	1.01		(0.98,1.04)	0.02	0.551	1.01		(0.98,1.04)	0.02	0.722
Faith-sharing	1.00		(0.98,1.03)	0.01	0.924	1.00		(0.97,1.02)	0.01	0.883

Notes. N=3012; Reference for focal predictor: never/rarely. RR, risk-ratio, null effect is 1.00; ES, effect size measure for standardized regression coefficient, null effect is 0.00; SE, standard error, the SE reported for binary/Likert-type outcomes where risk-ratios are on the log(RR) scale; CI, confidence interval; p-value, a Wald-type test of the null hypothesis that the effect of the focal predictor is zero; (a) item part of the Happiness & Life Satisfaction domain of the Secure Flourishing Index; (b) item part of the Physical & Mental Health domain of the Secure Flourishing Index; (c) item part of the Meaning & Purpose domain of the Secure Flourishing Index; (d) item part of the Character & Virtue domain of the Secure Flourishing Index; (e) item part of the Subjective Social Connectedness domain of the Secure Flourishing Index; (f) item part of the Financial & Material Security domain of the Secure Flourishing Index.

Multiple imputation was performed to impute missing data on the covariates, exposure, and outcomes. All models controlled for sociodemographic and childhood factors assessed at Wave 1. For Model 2 with PC (principal components), the first seven principal components of the entire set of contemporaneous confounders assessed at Wave 1 were included as additional covariates of the outcomes at Wave 2.

An outcome-wide analytic approach was used, and a separate model was run for each outcome. A different type of model was run depending on the nature of the outcome: (1) for each binary outcome, a weighted generalized linear model (with a log link and Poisson distribution) was used to estimate an RR; and (2) for each continuous outcome, a weighted linear regression model was used to estimate a ES. All effect sizes were standardized. For continuous outcomes, the ES represents the change in SD on the outcome between the lower and upper categories of the binary focal predictor. For binary outcomes, the RR represents the change in risk of being in the upper category compared to the lower category between the lower and upper categories of the binary focal predictor.

P-value significance thresholds: $p < 0.05$*, $p < 0.005$**, (Bonferroni) $p < 6.41e-04$***, correction for multiple testing using Bonferroni adjusted significant threshold.

Table S15h. Associations of forgivingness with adult well-being and other outcomes at Wave 2 in Hong Kong using complete-case analyses with attrition weights.

	Model 1: Demographic and Childhood Variables as Covariates					Model 2: Demographic, Childhood, and Other Wave 1 Confounding Variables (Via Principal Components) as Covariates				
Outcome	RR	ES	95% CI	SE	p-value	RR	ES	95% CI	SE	p-value
Human Flourishing										
Secure flourishing index		0.06	(-0.02,0.15)	0.04	0.151		0.01	(-0.05,0.08)	0.03	0.737
Flourishing index		0.08	(-0.01,0.16)	0.04	0.066		0.03	(-0.04,0.10)	0.03	0.383
Happiness & life satisfaction		0.06	(-0.03,0.15)	0.05	0.223		0.02	(-0.06,0.09)	0.04	0.677
Physical & mental health		0.08	(-0.01,0.17)	0.04	0.067		0.04	(-0.03,0.11)	0.04	0.246
Meaning & purpose		0.05	(-0.04,0.14)	0.05	0.255		0.00	(-0.07,0.08)	0.04	0.963
Character & virtue		0.12	(0.04,0.21)	0.04	0.005*		0.08	(0.00,0.16)	0.04	0.039*
Close social relationships		0.04	(-0.04,0.12)	0.04	0.340		-0.01	(-0.08,0.06)	0.03	0.818
Financial & material security		-0.02	(-0.11,0.06)	0.04	0.577		-0.06	(-0.13,0.02)	0.04	0.154
Psychological Well-Being										
Happiness (a)		0.05	(-0.04,0.14)	0.05	0.292		0.01	(-0.07,0.08)	0.04	0.848
Life satisfaction (a)		0.06	(-0.03,0.15)	0.05	0.216		0.02	(-0.06,0.10)	0.04	0.590
Current life evaluation		0.03	(-0.06,0.11)	0.04	0.566		-0.01	(-0.09,0.06)	0.04	0.735
Future life evaluation		0.09	(-0.00,0.18)	0.05	0.064		0.04	(-0.04,0.13)	0.04	0.281
Optimism		0.12	(0.04,0.21)	0.04	0.006*		0.08	(0.00,0.15)	0.04	0.038*
Freedom to pursue what's important		0.04	(-0.04,0.13)	0.04	0.325		-0.01	(-0.09,0.07)	0.04	0.834
Inner peace	1.04		(1.00,1.07)	0.02	0.030*	1.03		(1.00,1.06)	0.01	0.078
Life balance	1.03		(1.00,1.07)	0.02	0.090	1.02		(0.99,1.05)	0.02	0.231
Sense of mastery	1.02		(0.98,1.05)	0.02	0.296	1.01		(0.98,1.04)	0.02	0.644
Meaningful activities (c)		0.05	(-0.04,0.14)	0.04	0.288		-0.00	(-0.08,0.07)	0.04	0.911
Understanding purpose (c)		0.05	(-0.04,0.14)	0.05	0.308		0.01	(-0.07,0.09)	0.04	0.873
Self-rated mental health (b)		0.08	(-0.01,0.17)	0.04	0.074		0.04	(-0.03,0.11)	0.03	0.250
Psychological Distress										
Traumatic distress	1.02		(0.98,1.06)	0.02	0.265	1.02		(0.98,1.06)	0.02	0.239
Depression symptoms composite	0.98		(0.95,1.02)	0.02	0.395	1.00		(0.97,1.03)	0.02	0.865
Depression – feel hopeless	0.96		(0.93,1.00)	0.02	0.025*	0.97		(0.94,1.00)	0.01	0.025*
Depression – loss of interest	1.00		(0.96,1.04)	0.02	0.869	1.01		(0.97,1.05)	0.02	0.550
Anxiety symptoms composite	0.99		(0.95,1.02)	0.02	0.423	0.99		(0.96,1.02)	0.02	0.480
Anxiety – feel on edge	0.99		(0.96,1.02)	0.02	0.539	0.99		(0.96,1.02)	0.01	0.578
Anxiety – cannot stop worrying	0.98		(0.95,1.01)	0.02	0.230	0.99		(0.96,1.01)	0.01	0.297
Suffering	0.97		(0.93,1.01)	0.02	0.138	0.97		(0.93,1.01)	0.02	0.182
Social Well-Being										
Relationship contentment (e)		0.03	(-0.06,0.11)	0.04	0.516		-0.02	(-0.09,0.06)	0.04	0.624
Relationship satisfaction (e)		0.05	(-0.04,0.13)	0.04	0.265		0.00	(-0.07,0.07)	0.04	0.949
Social support		0.06	(-0.02,0.15)	0.04	0.157		0.04	(-0.04,0.12)	0.04	0.335
Intimate/close friend	1.01		(0.97,1.04)	0.02	0.664	1.00		(0.97,1.04)	0.02	0.894
Government approval	1.02		(0.98,1.06)	0.02	0.242	1.00		(0.97,1.04)	0.02	0.940
Say in government	1.01		(0.97,1.05)	0.02	0.679	0.99		(0.95,1.03)	0.02	0.694
Belonging in country		0.10	(0.02,0.18)	0.04	0.016*		0.05	(-0.02,0.12)	0.04	0.134
City/place satisfaction	1.04		(1.00,1.09)	0.02	0.067	1.02		(0.98,1.06)	0.02	0.391
Trust within country	1.01		(0.97,1.04)	0.02	0.705	1.00		(0.96,1.03)	0.02	0.914
Social Participation										
Ever been married	1.00		(0.99,1.02)	0.01	0.360	1.00		(0.99,1.01)	0.00	0.603
Currently divorced	1.00		(0.99,1.01)	0.01	0.761	1.00		(0.99,1.01)	0.01	0.900
Number of children		-0.00	(-0.10,0.09)	0.05	0.932		0.00	(-0.08,0.09)	0.04	0.941
Weekly+ community participation	1.01		(0.99,1.04)	0.01	0.327	1.01		(0.99,1.03)	0.01	0.420
Weekly+ religious attendance	1.02		(0.99,1.04)	0.01	0.130	1.02		(1.00,1.04)	0.01	0.123
Social Distress										
Loneliness		0.03	(-0.06,0.12)	0.05	0.556		0.07	(-0.00,0.15)	0.04	0.063
Perceived discrimination	1.01		(0.98,1.05)	0.02	0.495	1.01		(0.98,1.04)	0.02	0.588
Character & Prosocial Behavior										
Orientation to promote good (d)		0.11	(0.02,0.19)	0.04	0.016*		0.07	(-0.01,0.14)	0.04	0.096
Delayed gratification (d)		0.12	(0.04,0.20)	0.04	0.006*		0.09	(0.00,0.17)	0.04	0.039*
Hope		0.12	(0.03,0.21)	0.05	0.010*		0.07	(-0.01,0.14)	0.04	0.074
Gratitude		0.16	(0.08,0.24)	0.04	7.75e-05***		0.12	(0.05,0.19)	0.04	1.05e-03**
Showing love/care		0.06	(-0.03,0.14)	0.04	0.191		0.02	(-0.06,0.11)	0.04	0.558
Forgivingness	1.20		(1.15,1.25)	0.02	2.22e-16***	1.20		(1.15,1.25)	0.02	2.22e-16***
Charitable giving	1.06		(1.01,1.10)	0.02	0.009*	1.03		(0.99,1.07)	0.02	0.132
Helping strangers	1.04		(0.99,1.08)	0.02	0.103	1.01		(0.97,1.06)	0.02	0.538
Volunteering	1.00		(0.97,1.03)	0.02	0.959	0.99		(0.96,1.02)	0.01	0.423
Physical Health & Health Behavior										
Self-rated physical health (b)		0.07	(-0.02,0.15)	0.04	0.111		0.04	(-0.04,0.11)	0.04	0.350
Health problems	0.99		(0.96,1.02)	0.02	0.515	0.99		(0.96,1.03)	0.02	0.617
Pain in past 4 weeks	1.02		(0.98,1.06)	0.02	0.303	1.02		(0.98,1.06)	0.02	0.395
Daily smoker	0.99		(0.96,1.02)	0.02	0.679	1.01		(0.98,1.03)	0.01	0.627
Number of drinks per week		-0.07	(-0.18,0.04)	0.05	0.189		-0.06	(-0.15,0.03)	0.05	0.192
Days exercise per week		0.04	(-0.04,0.13)	0.04	0.337		0.03	(-0.05,0.12)	0.04	0.454
Socioeconomic Outcomes										
Financial security (f)		-0.02	(-0.10,0.07)	0.04	0.714		-0.05	(-0.13,0.03)	0.04	0.235
Material security (f)		-0.03	(-0.11,0.06)	0.04	0.512		-0.05	(-0.13,0.02)	0.04	0.180
Educational attainment (16+ years)	1.02		(1.00,1.04)	0.01	0.054	1.02		(1.00,1.03)	0.01	0.107
Currently employed	1.01		(1.00,1.03)	0.01	0.051	1.02		(1.00,1.03)	0.01	0.033*
Financially comfortable/getting by	1.02		(0.98,1.06)	0.02	0.369	1.01		(0.97,1.05)	0.02	0.604
Own home	1.03		(0.99,1.08)	0.02	0.138	1.03		(0.99,1.08)	0.02	0.178
Income – top quintile	1.00		(0.97,1.03)	0.02	0.977	1.00		(0.97,1.03)	0.02	0.979
Religion & Spirituality										
Religious/spiritual connection	1.05		(1.02,1.09)	0.02	2.73e-03**	1.04		(1.00,1.07)	0.02	0.028*
Belief in life after death	1.02		(0.97,1.06)	0.02	0.445	1.01		(0.97,1.06)	0.02	0.656
Transformative religious experience	1.05		(1.02,1.09)	0.02	0.005*	1.04		(1.01,1.08)	0.02	0.023*
Religious reading or listening	1.02		(1.00,1.04)	0.01	0.120	1.01		(0.99,1.03)	0.01	0.252
Prayer or meditation	1.01		(0.98,1.04)	0.01	0.461	1.00		(0.97,1.02)	0.01	0.840
Belief in God/gods/spiritual forces	1.00		(0.96,1.04)	0.02	0.977	0.98		(0.95,1.02)	0.02	0.394
Religious centrality	1.04		(1.01,1.07)	0.01	0.007*	1.02		(0.99,1.05)	0.01	0.163
Religious/spiritual comfort	1.03		(0.99,1.06)	0.02	0.129	1.01		(0.98,1.04)	0.02	0.698
Feel loved by God	1.02		(0.99,1.05)	0.02	0.185	1.00		(0.97,1.03)	0.01	0.880
Feel punished by God	1.01		(0.98,1.04)	0.01	0.579	1.00		(0.97,1.03)	0.01	0.956
Experienced religious criticism	1.01		(0.98,1.03)	0.01	0.649	1.00		(0.98,1.02)	0.01	0.949
Faith-sharing	1.00		(0.97,1.03)	0.02	0.956	0.98		(0.95,1.01)	0.02	0.249

Table S15h. Associations of forgivingness with adult well-being and other outcomes at Wave 2 in Hong Kong using complete-case analyses with attrition weights.

	Model 1: Demographic and Childhood Variables as Covariates					Model 2: Demographic, Childhood, and Other Wave 1 Confounding Variables (Via Principal Components) as Covariates				
Outcome	RR	ES	95% CI	SE	p-value	RR	ES	95% CI	SE	p-value

Notes. N=707; Reference for focal predictor: never/rarely. RR, risk-ratio, null effect is 1.00; ES, effect size measure for standardized regression coefficient, null effect is 0.00; SE, standard error, the SE reported for binary/Likert-type outcomes where risk-ratios are on the log(RR) scale; CI, confidence interval; p-value, a Wald-type test of the null hypothesis that the effect of the focal predictor is zero; (a) item part of the Happiness & Life Satisfaction domain of the Secure Flourishing Index; (b) item part of the Physical & Mental Health domain of the Secure Flourishing Index; (c) item part of the Meaning & Purpose domain of the Secure Flourishing Index; (d) item part of the Character & Virtue domain of the Secure Flourishing Index; (e) item part of the Subjective Social Connectedness domain of the Secure Flourishing Index; (f) item part of the Financial & Material Security domain of the Secure Flourishing Index.

Attrition weights were computed to adjust the complete case data (those who responded at Wave 2 to at least 50% of the questions) and multiple imputation was used to impute missing data on all remaining within wave on the covariates, exposure, and outcomes. All models controlled for sociodemographic and childhood factors assessed at Wave 1. For Model 2 with PC (principal components), the first seven principal components of the entire set of contemporaneous confounders assessed at Wave 1 were included as additional covariates of the outcomes at Wave 2.

An outcome-wide analytic approach was used, and a separate model was run for each outcome. A different type of model was run depending on the nature of the outcome: (1) for each binary outcome, a weighted generalized linear model (with a log link and Poisson distribution) was used to estimate an RR; and (2) for each continuous outcome, a weighted linear regression model was used to estimate a ES. All effect sizes were standardized. For continuous outcomes, the ES represents the change in SD on the outcome between the lower and upper categories of the binary focal predictor. For binary outcomes, the RR represents the change in risk of being in the upper category compared to the lower category between the lower and upper categories of the binary focal predictor.

P-value significance thresholds: $p < 0.05$*, $p < 0.005$**, (Bonferroni) $p < 6.41e-04$***, correction for multiple testing using Bonferroni adjusted significant threshold.

Table S15i. Sensitivity analysis of forgivingness outcome-wide results to unmeasured confounding using E-values in Hong Kong

Outcome	Multiple Imputation				Complete Case w/ Attrition Weights			
	Model 1: Demographics and Childhood Variables as Covariates		Model 2: Demographics, Childhood, and Other Wave 1 Confounders (Via Principal Components) as Covariates		Model 1: Demographics and Childhood Variables as Covariates		Model 2: Demographics, Childhood, and Other Wave 1 Confounders (Via Principal Components) as Covariates	
	EE	ECI	EE	ECI	EE	ECI	EE	ECI
Human Flourishing								
Secure flourishing index	1.51	1.13	1.20	1.00	1.49	1.00	1.17	1.00
Flourishing index	1.60	1.25	1.31	1.00	1.59	1.00	1.30	1.00
Happiness & life satisfaction	1.49	1.00	1.27	1.00	1.46	1.00	1.20	1.00
Physical & mental health	1.48	1.00	1.30	1.00	1.59	1.00	1.37	1.00
Meaning & purpose	1.39	1.00	1.13	1.00	1.43	1.00	1.06	1.00
Character & virtue	1.64	1.16	1.45	1.00	1.81	1.34	1.60	1.10
Close social relationships	1.35	1.00	1.09	1.00	1.36	1.00	1.14	1.00
Financial & material security	1.21	1.00	1.35	1.00	1.26	1.00	1.45	1.00
Psychological Well-Being								
Happiness	1.40	1.00	1.24	1.00	1.42	1.00	1.13	1.00
Life satisfaction	1.43	1.00	1.23	1.00	1.47	1.00	1.25	1.00
Current life evaluation	1.17	1.00	1.24	1.00	1.27	1.00	1.19	1.00
Future life evaluation	1.30	1.00	1.02	1.00	1.62	1.00	1.39	1.00
Optimism	1.39	1.00	1.19	1.00	1.82	1.34	1.58	1.10
Freedom to pursue what's important	1.11	1.00	1.27	1.00	1.38	1.00	1.14	1.00
Inner peace	1.24	1.00	1.18	1.00	1.36	1.09	1.29	1.00
Life balance	1.24	1.00	1.17	1.00	1.32	1.00	1.25	1.00
Sense of mastery	1.17	1.00	1.10	1.00	1.24	1.00	1.14	1.00
Meaningful activities	1.30	1.00	1.08	1.00	1.41	1.00	1.10	1.00
Understanding purpose	1.38	1.00	1.14	1.00	1.40	1.00	1.12	1.00
Self-rated mental health	1.45	1.00	1.30	1.00	1.59	1.00	1.36	1.00
Psychological Distress								
Traumatic distress	1.12	1.00	1.13	1.00	1.26	1.00	1.27	1.00
Depression symptoms composite	1.15	1.00	1.09	1.00	1.22	1.00	1.08	1.00
Depression – feel hopeless	1.13	1.00	1.09	1.00	1.36	1.11	1.34	1.10
Depression – loss of interest	1.21	1.00	1.19	1.00	1.09	1.00	1.18	1.00
Anxiety symptoms composite	1.14	1.00	1.10	1.00	1.20	1.00	1.17	1.00
Anxiety – feel on edge	1.11	1.00	1.09	1.00	1.17	1.00	1.15	1.00
Anxiety – cannot stop worrying	1.12	1.00	1.08	1.00	1.24	1.00	1.21	1.00
Suffering	1.18	1.00	1.15	1.00	1.34	1.00	1.30	1.00
Social Well-Being								
Relationship contentment	1.24	1.00	1.19	1.00	1.29	1.00	1.22	1.00
Relationship satisfaction	1.37	1.00	1.14	1.00	1.40	1.00	1.07	1.00
Social support	1.27	1.00	1.06	1.00	1.49	1.00	1.37	1.00
Intimate/close friend	1.20	1.00	1.17	1.00	1.14	1.00	1.07	1.00
Government approval	1.26	1.00	1.17	1.00	1.27	1.00	1.05	1.00
Say in government	1.14	1.00	1.07	1.00	1.16	1.00	1.15	1.00
Belonging in country	1.27	1.00	1.03	1.00	1.70	1.23	1.44	1.00
City/place satisfaction	1.18	1.00	1.10	1.00	1.39	1.00	1.23	1.00
Trust within country	1.16	1.00	1.08	1.00	1.13	1.00	1.07	1.00
Social Participation								
Ever been married	1.03	1.00	1.03	1.00	1.11	1.00	1.08	1.00
Currently divorced	1.02	1.00	1.03	1.00	1.07	1.00	1.04	1.00
Number of children	1.18	1.00	1.04	1.00	1.10	1.00	1.08	1.00
Weekly+ community participation	1.09	1.00	1.02	1.00	1.18	1.00	1.16	1.00
Weekly+ religious attendance	1.24	1.00	1.26	1.00	1.23	1.00	1.22	1.00
Social Distress								
Loneliness	1.09	1.00	1.24	1.00	1.28	1.00	1.54	1.00
Perceived discrimination	1.30	1.00	1.28	1.00	1.18	1.00	1.16	1.00
Character & Prosocial Behavior								
Orientation to promote good	1.44	1.00	1.27	1.00	1.73	1.24	1.51	1.00
Delayed gratification	1.65	1.20	1.52	1.00	1.80	1.33	1.62	1.10
Hope	1.49	1.00	1.27	1.00	1.79	1.30	1.53	1.00
Gratitude	1.60	1.19	1.45	1.00	2.01	1.59	1.79	1.41
Showing love/care	1.35	1.00	1.18	1.00	1.47	1.00	1.26	1.00
Forgivingness	1.90	1.67	1.90	1.66	2.24	1.99	2.26	2.01
Charitable giving	1.27	1.00	1.21	1.00	1.47	1.20	1.32	1.00
Helping strangers	1.12	1.00	1.09	1.00	1.36	1.00	1.19	1.00
Volunteering	1.11	1.00	1.07	1.00	1.04	1.00	1.18	1.00
Physical Health & Health Behavior								
Self-rated physical health	1.38	1.00	1.22	1.00	1.53	1.00	1.34	1.00
Health problems	1.17	1.00	1.14	1.00	1.18	1.00	1.15	1.00
Pain in past 4 weeks	1.04	1.00	1.06	1.00	1.26	1.00	1.23	1.00
Daily smoker	1.16	1.00	1.08	1.00	1.13	1.00	1.13	1.00
Number of drinks per week	1.12	1.00	1.15	1.00	1.54	1.00	1.48	1.00
Days exercise per week	1.36	1.00	1.29	1.00	1.38	1.00	1.32	1.00
Socioeconomic Outcomes								
Financial security	1.24	1.00	1.38	1.00	1.20	1.00	1.41	1.00
Material security	1.10	1.00	1.21	1.00	1.29	1.00	1.44	1.00
Educational attainment (16+ years)	1.16	1.00	1.16	1.00	1.24	1.00	1.21	1.00
Currently employed	1.10	1.00	1.05	1.00	1.21	1.00	1.22	1.05
Financially comfortable/getting by	1.11	1.00	1.09	1.00	1.23	1.00	1.16	1.00
Own home	1.15	1.00	1.15	1.00	1.34	1.00	1.32	1.00
Income – top quintile	1.17	1.00	1.14	1.00	1.03	1.00	1.03	1.00
Religion & Spirituality								
Religious/spiritual connection	1.40	1.16	1.39	1.14	1.47	1.24	1.37	1.10
Belief in life after death	1.08	1.00	1.07	1.00	1.23	1.00	1.17	1.00
Transformative religious experience	1.34	1.00	1.30	1.00	1.47	1.21	1.41	1.12
Religious reading or listening	1.24	1.00	1.23	1.00	1.21	1.00	1.18	1.00
Prayer or meditation	1.24	1.00	1.22	1.00	1.17	1.00	1.08	1.00
Belief in God/gods/spiritual forces	1.16	1.00	1.15	1.00	1.04	1.00	1.22	1.00
Religious centrality	1.27	1.00	1.24	1.00	1.38	1.17	1.24	1.00
Religious/spiritual comfort	1.17	1.00	1.15	1.00	1.29	1.00	1.12	1.00
Feel loved by God	1.25	1.00	1.22	1.00	1.25	1.00	1.07	1.00
Feel punished by God	1.16	1.00	1.16	1.00	1.14	1.00	1.04	1.00
Experienced religious criticism	1.16	1.00	1.12	1.00	1.12	1.00	1.04	1.00
Faith-sharing	1.05	1.00	1.07	1.00	1.04	1.00	1.23	1.00

Notes. EE, E-value for estimate; ECI, E-value for the limit of the confidence interval. The formula for calculating E-values can be found in VanderWeele and Ding (2017). E-values for estimate are the minimum strength of association on the risk ratio scale that an unmeasured confounder would need to have with both the exposure and the outcome to fully explain away the observed association between the exposure and outcome, conditional on the measured covariates. E-values for the 95% CI closest to the null denote the minimum strength of association on the risk ratio scale that an unmeasured confounder would need to have with both the exposure and the outcome to shift the CI to include the null value, conditional on the measured covariates.

Table S16a. Weighted summary statistics for demographic and childhood variables in India

**Characteristic**	**Wave 1** N = 12,765	**Wave 2** N = 6,382
Forgivingness, n (%)		
Always	7,783 (61.0%)	3,736 (58.5%)
Often	1,692 (13.3%)	790 (12.4%)
Rarely	2,148 (16.8%)	1,147 (18.0%)
Never	1,027 (8.0%)	678 (10.6%)
(Missing)	115 (0.9%)	32 (0.5%)
Year of birth, n (%)		
1943 or earlier (current age: 80+ years)	67 (0.5%)	27 (0.4%)
1943-1953 (current age: 70-79 years)	359 (2.8%)	190 (3.0%)
1953-1963 (current age: 60-69 years)	1,161 (9.1%)	606 (9.5%)
1963-1973 (current age: 50-59 years)	1,523 (11.9%)	783 (12.3%)
1973-1983 (current age: 40-49 years)	2,160 (16.9%)	1,116 (17.5%)
1983-1993 (current age: 30-39 years)	2,928 (22.9%)	1,524 (23.9%)
1993-1998 (current age: 25-29 years)	1,508 (11.8%)	809 (12.7%)
1998-2005 (current age: 18-24 years)	3,060 (24.0%)	1,327 (20.8%)
(Missing)	0 (0%)	0 (0%)
Age of participant		
Mean	37.9	39.1
Standard Deviation	15.1	15.0
Min, Max	18.0, 96.0	19.0, 97.0
Gender, n (%)		
Male	6,500 (50.9%)	3,262 (51.1%)
Female	6,265 (49.1%)	3,120 (48.9%)
Other	0 (0%)	0 (0%)
(Missing)	0 (0%)	0 (0%)
Respondent marital status, n (%)		
Single/Never been married	2,242 (17.6%)	1,068 (16.7%)
Married	9,678 (75.8%)	5,014 (78.6%)
Separated	46 (0.4%)	38 (0.6%)
Divorced	24 (0.2%)	14 (0.2%)
Widowed	439 (3.4%)	210 (3.3%)
Domestic partner	259 (2.0%)	32 (0.5%)
(Missing)	77 (0.6%)	5 (0.1%)
Education (years), n (%)		
Up to 8	9,188 (72.0%)	4,123 (64.6%)
9-15	2,903 (22.7%)	1,835 (28.7%)
16+	671 (5.3%)	425 (6.7%)
(Missing)	3 (0.0%)	0 (0%)
Employment status, n (%)		
Employed for an employer	2,707 (21.2%)	1,550 (24.3%)
Self-employed	3,359 (26.3%)	1,667 (26.1%)
Retired	271 (2.1%)	107 (1.7%)
Student	545 (4.3%)	183 (2.9%)
Homemaker	4,191 (32.8%)	1,787 (28.0%)
Unemployed and looking for a job	921 (7.2%)	593 (9.3%)
None of these/Other	722 (5.7%)	439 (6.9%)
(Missing)	49 (0.4%)	57 (0.9%)
Current religious service attendance, n (%)		
More than once a week	2,853 (22.4%)	1,313 (20.6%)
Once a week	3,204 (25.1%)	1,600 (25.1%)

Table S16a. Weighted summary statistics for demographic and childhood variables in India

**Characteristic**	**Wave 1** N = 12,765	**Wave 2** N = 6,382
One to three times a month	2,700 (21.2%)	1,500 (23.5%)
A few times a year	2,051 (16.1%)	1,069 (16.7%)
Never	1,883 (14.7%)	882 (13.8%)
(Missing)	74 (0.6%)	18 (0.3%)
Immigration status, n (%)		
Born in this country	12,622 (98.9%)	6,310 (98.9%)
Born in another country	115 (0.9%)	59 (0.9%)
(Missing)	28 (0.2%)	13 (0.2%)
Parental marital status around age 12, n (%)		
Parents were married	5,515 (43.2%)	2,781 (43.6%)
Parents were divorced	232 (1.8%)	121 (1.9%)
Parents were never married	1,056 (8.3%)	558 (8.7%)
One or both of them had died	938 (7.4%)	509 (8.0%)
Unsure	83 (0.6%)	23 (0.4%)
(Missing)	4,941 (38.7%)	2,391 (37.5%)
Religious service attendance around age 12, n (%)		
At least once a week	5,267 (41.3%)	2,644 (41.4%)
One to three times a month	2,933 (23.0%)	1,473 (23.1%)
Less than once a month	2,756 (21.6%)	1,342 (21.0%)
Never	1,489 (11.7%)	760 (11.9%)
(Missing)	321 (2.5%)	164 (2.6%)
Relationship with mother when growing up, n (%)		
Very good	11,436 (89.6%)	5,716 (89.6%)
Somewhat good	795 (6.2%)	399 (6.3%)
Somewhat bad	92 (0.7%)	49 (0.8%)
Very bad	79 (0.6%)	32 (0.5%)
(Does not apply)	275 (2.2%)	145 (2.3%)
(Missing)	86 (0.7%)	41 (0.6%)
Relationship with father when growing up, n (%)		
Very good	10,897 (85.4%)	5,462 (85.6%)
Somewhat good	995 (7.8%)	482 (7.5%)
Somewhat bad	130 (1.0%)	80 (1.2%)
Very bad	110 (0.9%)	44 (0.7%)
(Does not apply)	483 (3.8%)	245 (3.8%)
(Missing)	150 (1.2%)	70 (1.1%)
Felt like an outsider in family when growing up, n (%)		
Yes	1,951 (15.3%)	981 (15.4%)
No	10,753 (84.2%)	5,371 (84.2%)
(Missing)	60 (0.5%)	30 (0.5%)
Experienced abuse when growing up, n (%)		
Yes	1,485 (11.6%)	787 (12.3%)
No	10,484 (82.1%)	5,183 (81.2%)
(Missing)	796 (6.2%)	412 (6.5%)
Self-rated health when growing up, n (%)		
Excellent	2,171 (17.0%)	1,126 (17.6%)
Very good	3,845 (30.1%)	1,909 (29.9%)
Good	4,047 (31.7%)	1,987 (31.1%)
Fair	2,216 (17.4%)	1,138 (17.8%)
Poor	436 (3.4%)	198 (3.1%)
(Missing)	51 (0.4%)	24 (0.4%)

Table S16a. Weighted summary statistics for demographic and childhood variables in India

Characteristic	**Wave 1**<br>N = 12,765	**Wave 2**<br>N = 6,382
Subjective financial status of family growing up, n (%)		
Lived comfortably	4,869 (38.1%)	2,359 (37.0%)
Got by	3,032 (23.7%)	1,557 (24.4%)
Found it difficult	2,720 (21.3%)	1,423 (22.3%)
Found it very difficult	2,072 (16.2%)	1,011 (15.8%)
(Missing)	72 (0.6%)	32 (0.5%)
Religious affiliation growing up, n (%)		
Christianity	253 (2.0%)	118 (1.8%)
Taoism	0 (0%)	0 (0%)
Confucianism	0 (0%)	0 (0%)
Primal, Animist, or Folk religion	28 (0.2%)	9 (0.1%)
Spiritism	0 (0%)	0 (0%)
Umbanda, Candomblé, and other African-derived religions	0 (0%)	0 (0%)
Chinese folk/traditional religion	0 (0%)	0 (0%)
Islam	1,613 (12.6%)	843 (13.2%)
Hinduism	10,357 (81.1%)	5,183 (81.2%)
Buddhism	167 (1.3%)	84 (1.3%)
Judaism	0 (0%)	0 (0%)
Sikhism	122 (1.0%)	46 (0.7%)
Baha'i	0 (0%)	0 (0%)
Jainism	9 (0.1%)	4 (0.1%)
Shinto	4 (0.0%)	0 (0%)
Some other religion	66 (0.5%)	29 (0.5%)
No religion/Atheist/Agnostic	8 (0.1%)	8 (0.1%)
(Missing)	138 (1.1%)	59 (0.9%)

Note. N (%); this table is based on non-imputed data. Cumulative percentages for variables may not add up to 100% due to rounding. Wave 1 characteristics weighted using the Gallup provided sampling weight, ANNUAL_WEIGHT_R2; Wave 2 characteristics weighted accounting for attrition by using the adjusted Wave 1 weight, ANNUAL_WEIGHT_R2, multiplied by the created attrition weight to account for dropout, to maintain nationally representative estimates for Wave 2 characteristics.

Table S16b. Weighted summary statistics for outcome variables in India

Outcome	Wave 1<br>N = 12,765	Wave 2<br>N = 6,382
Secure flourishing index		
Mean	6.9	6.6
Standard Deviation	1.9	1.9
Min, Max	0.0, 10.0	0.0, 10.0
(Missing)	533 (4.2%)	56 (0.9%)
Flourishing index		
Mean	7.5	7.0
Standard Deviation	2.0	2.0
Min, Max	0.0, 10.0	0.0, 10.0
(Missing)	505 (4.0%)	55 (0.9%)
Happiness & life satisfaction		
Mean	6.7	6.5
Standard Deviation	3.1	3.0
Min, Max	0.0, 10.0	0.0, 10.0
(Missing)	89 (0.7%)	13 (0.2%)
Physical & mental health		
Mean	7.2	6.9
Standard Deviation	3.0	2.8
Min, Max	0.0, 10.0	0.0, 10.0
(Missing)	66 (0.5%)	8 (0.1%)
Meaning & purpose		
Mean	7.5	7.1
Standard Deviation	2.6	2.6
Min, Max	0.0, 10.0	0.0, 10.0
(Missing)	248 (1.9%)	17 (0.3%)
Character & virtue		
Mean	7.8	7.5
Standard Deviation	2.5	2.5
Min, Max	0.0, 10.0	0.0, 10.0
(Missing)	166 (1.3%)	22 (0.3%)
Close social relationships		
Mean	8.0	7.3
Standard Deviation	2.7	2.8
Min, Max	0.0, 10.0	0.0, 10.0
(Missing)	107 (0.8%)	13 (0.2%)
Financial & material security		
Mean	4.0	4.6
Standard Deviation	3.7	3.5
Min, Max	0.0, 10.0	0.0, 10.0
(Missing)	44 (0.3%)	3 (<0.1%)
Happiness		
Mean	6.5	6.2
Standard Deviation	3.6	3.4
Min, Max	0.0, 10.0	0.0, 10.0
(Missing)	38 (0.3%)	9 (0.1%)
Life satisfaction		
Mean	7.0	6.7
Standard Deviation	3.5	3.4
Min, Max	0.0, 10.0	0.0, 10.0
(Missing)	62 (0.5%)	4 (<0.1%)
Current life evaluation		

Table S16b. Weighted summary statistics for outcome variables in India

Outcome	Wave 1<br>N = 12,765	Wave 2<br>N = 6,382
Mean	5.6	5.6
Standard Deviation	3.6	3.4
Min, Max	0.0, 10.0	0.0, 10.0
(Missing)	138 (1.1%)	7 (0.1%)
Future life evaluation		
Mean	7.3	6.9
Standard Deviation	3.1	3.1
Min, Max	0.0, 10.0	0.0, 10.0
(Missing)	1,218 (9.5%)	115 (1.8%)
Optimism		
Mean	8.1	7.7
Standard Deviation	2.9	3.0
Min, Max	0.0, 10.0	0.0, 10.0
(Missing)	65 (0.5%)	11 (0.2%)
Freedom to pursue what's important		
Mean	8.1	7.7
Standard Deviation	3.0	3.0
Min, Max	0.0, 10.0	0.0, 10.0
(Missing)	68 (0.5%)	8 (0.1%)
Inner peace, n (%)		
Always	5,582 (43.7%)	2,582 (40.5%)
Often	2,085 (16.3%)	1,127 (17.7%)
Rarely	3,738 (29.3%)	1,995 (31.3%)
Never	1,235 (9.7%)	663 (10.4%)
(Missing)	125 (1.0%)	15 (0.2%)
Life balance, n (%)		
Always	4,111 (32.2%)	1,831 (28.7%)
Often	2,299 (18.0%)	1,214 (19.0%)
Rarely	4,892 (38.3%)	2,535 (39.7%)
Never	1,277 (10.0%)	786 (12.3%)
(Missing)	186 (1.5%)	17 (0.3%)
Sense of mastery, n (%)		
Always	6,940 (54.4%)	3,194 (50.0%)
Often	1,883 (14.8%)	943 (14.8%)
Rarely	2,909 (22.8%)	1,612 (25.3%)
Never	938 (7.3%)	625 (9.8%)
(Missing)	95 (0.7%)	8 (0.1%)
Meaningful activities		
Mean	6.9	6.6
Standard Deviation	3.4	3.2
Min, Max	0.0, 10.0	0.0, 10.0
(Missing)	159 (1.2%)	6 (<0.1%)
Understanding purpose		
Mean	8.0	7.5
Standard Deviation	3.0	3.0
Min, Max	0.0, 10.0	0.0, 10.0
(Missing)	118 (0.9%)	16 (0.3%)
Self-rated mental health		
Mean	7.4	6.9
Standard Deviation	3.3	3.2
Min, Max	0.0, 10.0	0.0, 10.0

Table S16b. Weighted summary statistics for outcome variables in India

Outcome	Wave 1<br>N = 12,765	Wave 2<br>N = 6,382
(Missing)	41 (0.3%)	6 (<0.1%)
Traumatic distress, n (%)		
A lot	3,255 (25.5%)	1,959 (30.7%)
Some	2,850 (22.3%)	1,608 (25.2%)
Not very much	1,051 (8.2%)	420 (6.6%)
Not at all	5,525 (43.3%)	2,365 (37.1%)
(Missing)	84 (0.7%)	30 (0.5%)
Depression symptoms composite, n (%)	6,188 (49.2%)	3,263 (51.3%)
(Missing)	196 (1.5%)	27 (0.4%)
Depression – feel hopeless, n (%)		
Nearly every day	1,829 (14.3%)	920 (14.4%)
More than half the days	2,134 (16.7%)	1,452 (22.8%)
Several days	3,211 (25.2%)	1,482 (23.2%)
Not at all	5,481 (42.9%)	2,514 (39.4%)
(Missing)	111 (0.9%)	14 (0.2%)
Depression – loss of interest, n (%)		
Nearly every day	3,692 (28.9%)	1,730 (27.1%)
More than half the days	2,259 (17.7%)	1,336 (20.9%)
Several days	2,766 (21.7%)	1,356 (21.3%)
Not at all	3,935 (30.8%)	1,943 (30.4%)
(Missing)	113 (0.9%)	17 (0.3%)
Anxiety symptoms composite, n (%)	4,529 (36.0%)	2,560 (40.4%)
(Missing)	185 (1.4%)	41 (0.6%)
Anxiety – feel on edge, n (%)		
Nearly every day	1,991 (15.6%)	1,008 (15.8%)
More than half the days	2,031 (15.9%)	1,325 (20.8%)
Several days	2,988 (23.4%)	1,339 (21.0%)
Not at all	5,665 (44.4%)	2,687 (42.1%)
(Missing)	90 (0.7%)	24 (0.4%)
Anxiety – cannot stop worrying, n (%)		
Nearly every day	2,121 (16.6%)	1,119 (17.5%)
More than half the days	1,895 (14.8%)	1,248 (19.5%)
Several days	2,690 (21.1%)	1,193 (18.7%)
Not at all	5,924 (46.4%)	2,797 (43.8%)
(Missing)	135 (1.1%)	25 (0.4%)
Suffering, n (%)		
A lot	2,693 (21.1%)	1,519 (23.8%)
Some	3,775 (29.6%)	2,108 (33.0%)
Not very much	1,228 (9.6%)	562 (8.8%)
Not at all	4,971 (38.9%)	2,169 (34.0%)
(Missing)	98 (0.8%)	24 (0.4%)
Relationship contentment		
Mean	8.0	7.4
Standard Deviation	3.1	3.3
Min, Max	0.0, 10.0	0.0, 10.0
(Missing)	32 (0.3%)	7 (0.1%)
Relationship satisfaction		
Mean	7.9	7.2
Standard Deviation	3.1	3.2
Min, Max	0.0, 10.0	0.0, 10.0
(Missing)	79 (0.6%)	9 (0.1%)

Table S16b. Weighted summary statistics for outcome variables in India

Outcome	Wave 1<br>N = 12,765	Wave 2<br>N = 6,382
Social support		
Mean	6.5	6.1
Standard Deviation	3.9	3.7
Min, Max	0.0, 10.0	0.0, 10.0
(Missing)	52 (0.4%)	4 (<0.1%)
Intimate/close friend, n (%)		
Yes	10,406 (81.5%)	4,973 (77.9%)
No	2,333 (18.3%)	1,394 (21.8%)
(Missing)	27 (0.2%)	15 (0.2%)
Government approval, n (%)		
Strongly approve	7,069 (55.4%)	3,287 (51.5%)
Somewhat approve	3,465 (27.1%)	2,126 (33.3%)
Neither approve nor disapprove	506 (4.0%)	193 (3.0%)
Somewhat disapprove	645 (5.1%)	283 (4.4%)
Strongly disapprove	785 (6.1%)	414 (6.5%)
(Missing)	295 (2.3%)	80 (1.3%)
Say in government, n (%)		
Agree	9,906 (77.6%)	4,847 (75.9%)
Disagree	2,016 (15.8%)	1,169 (18.3%)
Unsure	769 (6.0%)	346 (5.4%)
(Missing)	74 (0.6%)	20 (0.3%)
Belonging in country		
Mean	8.4	8.1
Standard Deviation	2.9	2.9
Min, Max	0.0, 10.0	0.0, 10.0
(Missing)	119 (0.9%)	22 (0.3%)
City/place satisfaction, n (%)		
Satisfied	11,835 (92.7%)	5,695 (89.2%)
Dissatisfied	826 (6.5%)	586 (9.2%)
Unsure	102 (0.8%)	96 (1.5%)
(Missing)	2 (0.0%)	5 (0.1%)
Trust within country, n (%)		
All people	2,108 (16.5%)	894 (14.0%)
Most people	2,194 (17.2%)	998 (15.6%)
Some people	5,863 (45.9%)	3,147 (49.3%)
Not very many people	1,072 (8.4%)	623 (9.8%)
None	1,098 (8.6%)	648 (10.2%)
(Missing)	430 (3.4%)	72 (1.1%)
Number of children		
Mean	1.5	1.9
Standard Deviation	1.7	3.1
Min, Max	0.0, 60.0	0.0, 97.0
(Missing)	18 (0.1%)	28 (0.4%)
Community participation, n (%)		
More than once a week	588 (4.6%)	300 (4.7%)
Once a week	1,107 (8.7%)	556 (8.7%)
One to three times a month	1,491 (11.7%)	992 (15.5%)
A few times a year	1,877 (14.7%)	1,054 (16.5%)
Never	7,540 (59.1%)	3,449 (54.0%)
(Missing)	162 (1.3%)	32 (0.5%)
Religious attendance, n (%)		

Table S16b. Weighted summary statistics for outcome variables in India

**Outcome**	**Wave 1**<br>N = 12,765	**Wave 2**<br>N = 6,382
More than once a week	2,853 (22.4%)	1,313 (20.6%)
Once a week	3,204 (25.1%)	1,600 (25.1%)
One to three times a month	2,700 (21.2%)	1,500 (23.5%)
A few times a year	2,051 (16.1%)	1,069 (16.7%)
Never	1,883 (14.7%)	882 (13.8%)
(Missing)	74 (0.6%)	18 (0.3%)
Loneliness		
Mean	3.8	4.3
Standard Deviation	4.0	3.9
Min, Max	0.0, 10.0	0.0, 10.0
(Missing)	32 (0.3%)	2 (<0.1%)
Perceived discrimination, n (%)		
Always	1,681 (13.2%)	1,085 (17.0%)
Often	1,079 (8.5%)	680 (10.7%)
Rarely	2,640 (20.7%)	1,670 (26.2%)
Never	7,169 (56.2%)	2,920 (45.7%)
(Missing)	196 (1.5%)	28 (0.4%)
Orientation to promote good		
Mean	8.4	7.9
Standard Deviation	2.7	2.8
Min, Max	0.0, 10.0	0.0, 10.0
(Missing)	67 (0.5%)	7 (0.1%)
Delayed gratification		
Mean	7.2	7.1
Standard Deviation	3.5	3.1
Min, Max	0.0, 10.0	0.0, 10.0
(Missing)	115 (0.9%)	19 (0.3%)
Hope		
Mean	8.3	7.7
Standard Deviation	2.8	2.9
Min, Max	0.0, 10.0	0.0, 10.0
(Missing)	88 (0.7%)	3 (<0.1%)
Gratitude		
Mean	7.7	7.2
Standard Deviation	3.1	3.1
Min, Max	0.0, 10.0	0.0, 10.0
(Missing)	209 (1.6%)	8 (0.1%)
Showing love/care		
Mean	8.2	7.8
Standard Deviation	3.0	3.1
Min, Max	0.0, 10.0	0.0, 10.0
(Missing)	37 (0.3%)	1 (<0.1%)
Forgivingness, n (%)		
Always	7,783 (61.0%)	3,736 (58.5%)
Often	1,692 (13.3%)	790 (12.4%)
Rarely	2,148 (16.8%)	1,147 (18.0%)
Never	1,027 (8.0%)	678 (10.6%)
(Missing)	115 (0.9%)	32 (0.5%)
Charitable giving, n (%)		
Yes	4,936 (38.7%)	3,453 (54.1%)
No	7,778 (60.9%)	2,913 (45.6%)

Table S16b. Weighted summary statistics for outcome variables in India

Outcome	Wave 1<br>N = 12,765	Wave 2<br>N = 6,382
(Missing)	51 (0.4%)	16 (0.3%)
Helping strangers, n (%)		
Yes	7,078 (55.4%)	4,370 (68.5%)
No	5,548 (43.5%)	1,986 (31.1%)
(Missing)	139 (1.1%)	26 (0.4%)
Volunteering, n (%)		
Yes	3,688 (28.9%)	2,942 (46.1%)
No	8,995 (70.5%)	3,414 (53.5%)
(Missing)	82 (0.6%)	26 (0.4%)
Self-rated physical health		
Mean	7.0	6.8
Standard Deviation	3.4	3.2
Min, Max	0.0, 10.0	0.0, 10.0
(Missing)	32 (0.2%)	2 (<0.1%)
Health problems, n (%)		
Yes	3,579 (28.0%)	2,045 (32.0%)
No	9,079 (71.1%)	4,309 (67.5%)
(Missing)	107 (0.8%)	28 (0.4%)
Pain in past 4 weeks, n (%)		
A lot	2,586 (20.3%)	1,636 (25.6%)
Some	3,829 (30.0%)	2,071 (32.4%)
Not very much	1,126 (8.8%)	487 (7.6%)
None at all	5,188 (40.6%)	2,173 (34.0%)
(Missing)	36 (0.3%)	16 (0.3%)
Number of cigarettes per day		
Mean	0.7	0.7
Standard Deviation	4.9	4.0
Min, Max	0.0, 97.0	0.0, 97.0
(Missing)	503 (3.9%)	62 (1.0%)
Number of drinks per week		
Mean	0.4	0.3
Standard Deviation	4.5	2.8
Min, Max	0.0, 97.0	0.0, 97.0
(Missing)	518 (4.1%)	73 (1.1%)
Days exercise per week		
Mean	3.1	3.0
Standard Deviation	3.2	3.2
Min, Max	0.0, 7.0	0.0, 7.0
(Missing)	2,726 (21%)	823 (13%)
Financial security		
Mean	4.1	4.6
Standard Deviation	4.1	3.9
Min, Max	0.0, 10.0	0.0, 10.0
(Missing)	31 (0.2%)	2 (<0.1%)
Material security		
Mean	4.0	4.6
Standard Deviation	4.1	4.0
Min, Max	0.0, 10.0	0.0, 10.0
(Missing)	18 (0.1%)	1 (<0.1%)
Educational attainment (16+ years), n (%)		
Up to 8	9,188 (72.0%)	4,123 (64.6%)

Table S16b. Weighted summary statistics for outcome variables in India

Outcome	Wave 1<br>N = 12,765	Wave 2<br>N = 6,382
9-15	2,903 (22.7%)	1,835 (28.7%)
16+	671 (5.3%)	425 (6.7%)
(Missing)	3 (0.0%)	0 (0%)
Currently employed, n (%)		
Employed for an employer	2,707 (21.2%)	1,550 (24.3%)
Self-employed	3,359 (26.3%)	1,667 (26.1%)
Retired	271 (2.1%)	107 (1.7%)
Student	545 (4.3%)	183 (2.9%)
Homemaker	4,191 (32.8%)	1,787 (28.0%)
Unemployed and looking for a job	921 (7.2%)	593 (9.3%)
None of these/Other	722 (5.7%)	439 (6.9%)
(Missing)	49 (0.4%)	57 (0.9%)
Financially comfortable/getting by, n (%)		
Living comfortably on present income	2,827 (22.1%)	1,466 (23.0%)
Getting by on present income	3,441 (27.0%)	1,864 (29.2%)
Finding it difficult on present income	3,552 (27.8%)	1,663 (26.1%)
Finding it very difficult on present income	2,872 (22.5%)	1,369 (21.5%)
(Missing)	73 (0.6%)	19 (0.3%)
Own home, n (%)		
Someone in this household owns this home	10,235 (80.2%)	4,778 (74.9%)
Someone in this household rents this home	1,788 (14.0%)	995 (15.6%)
Both	148 (1.2%)	47 (0.7%)
Neither	480 (3.8%)	546 (8.6%)
Rent	0 (0%)	0 (0%)
Own	0 (0%)	0 (0%)
Something else	0 (0%)	0 (0%)
(Missing)	113 (0.9%)	15 (0.2%)
Religious/spiritual connection, n (%)		
Always	7,775 (60.9%)	3,553 (55.7%)
Often	1,343 (10.5%)	758 (11.9%)
Rarely	2,321 (18.2%)	1,336 (20.9%)
Never	1,227 (9.6%)	721 (11.3%)
(Missing)	99 (0.8%)	13 (0.2%)
Belief in life after death, n (%)		
Yes	6,129 (48.0%)	3,111 (48.7%)
No	5,314 (41.6%)	2,723 (42.7%)
Unsure	1,210 (9.5%)	536 (8.4%)
(Missing)	112 (0.9%)	12 (0.2%)
Transformative religious experience, n (%)		
Yes	7,167 (56.1%)	3,466 (54.3%)
No	5,214 (40.8%)	2,835 (44.4%)
(Missing)	384 (3.0%)	81 (1.3%)
Religious reading or listening, n (%)		
More than once a day	1,690 (13.2%)	950 (14.9%)
About once a day	2,890 (22.6%)	1,382 (21.7%)
Sometimes	5,442 (42.6%)	2,641 (41.4%)
Never	2,675 (21.0%)	1,393 (21.8%)
(Missing)	69 (0.5%)	16 (0.2%)
Prayer or meditation, n (%)		
More than once a day	4,299 (33.7%)	2,041 (32.0%)
About once a day	3,886 (30.4%)	2,046 (32.1%)

Table S16b. Weighted summary statistics for outcome variables in India

Outcome	Wave 1<br>N = 12,765	Wave 2<br>N = 6,382
Sometimes	3,262 (25.6%)	1,625 (25.5%)
Never	1,285 (10.1%)	667 (10.5%)
(Missing)	33 (0.3%)	2 (0.0%)
Belief in God/gods/spiritual forces, n (%)		
One God	7,160 (56.1%)	3,533 (55.4%)
More than one god	4,729 (37.1%)	2,400 (37.6%)
An impersonal spiritual force	259 (2.0%)	110 (1.7%)
None of these	387 (3.0%)	264 (4.1%)
Unsure	203 (1.6%)	69 (1.1%)
(Missing)	27 (0.2%)	6 (0.1%)
Religious centrality, n (%)		
Agree	10,872 (85.2%)	5,367 (84.1%)
Disagree	1,220 (9.6%)	664 (10.4%)
Not relevant	205 (1.6%)	100 (1.6%)
Unsure	418 (3.3%)	243 (3.8%)
(Missing)	50 (0.4%)	9 (0.1%)
Religious/spiritual comfort, n (%)		
Agree	11,261 (88.2%)	5,447 (85.4%)
Disagree	1,077 (8.4%)	641 (10.0%)
Not relevant	142 (1.1%)	81 (1.3%)
Unsure	254 (2.0%)	210 (3.3%)
(Missing)	30 (0.2%)	3 (0.1%)
Feel loved by God, n (%)		
Agree	11,512 (90.2%)	5,627 (88.2%)
Disagree	936 (7.3%)	546 (8.6%)
Not relevant	129 (1.0%)	66 (1.0%)
Unsure	172 (1.3%)	141 (2.2%)
(Missing)	17 (0.1%)	3 (0.0%)
Feel punished by God, n (%)		
Agree	8,102 (63.5%)	4,179 (65.5%)
Disagree	3,972 (31.1%)	1,788 (28.0%)
Not relevant	251 (2.0%)	113 (1.8%)
Unsure	408 (3.2%)	297 (4.7%)
(Missing)	32 (0.3%)	6 (0.1%)
Experienced religious criticism, n (%)		
Agree	7,933 (62.1%)	3,926 (61.5%)
Disagree	3,947 (30.9%)	1,921 (30.1%)
Not relevant	260 (2.0%)	112 (1.8%)
Unsure	566 (4.4%)	413 (6.5%)
(Missing)	60 (0.5%)	10 (0.2%)
Faith-sharing, n (%)		
Agree	8,892 (69.7%)	4,367 (68.4%)
Disagree	3,102 (24.3%)	1,615 (25.3%)
Not relevant	340 (2.7%)	107 (1.7%)
Unsure	376 (2.9%)	284 (4.4%)
(Missing)	56 (0.4%)	10 (0.2%)

Note. N (%); this table is based on non-imputed data. Cumulative percentages for variables may not add up to 100% due to rounding. Wave 1 characteristics weighted using the Gallup provided sampling weight, ANNUAL_WEIGHT_R2; Wave 2 characteristics weighted accounting for attrition by using the adjusted Wave 1 weight, ANNUAL_WEIGHT_R2, multiplied by the created attrition weight to account for dropout, to maintain nationally representative estimates for Wave 2 characteristics.

Table S16c. Unweighted summary statistics for demographic and childhood variables in India by retention status

Characteristic	Attritors–Not Observed in Wave 2<br>N = 6,696	Retained–Observed in Wave 2<br>N = 6,050
Forgivingness, n (%)		
Always	4,052 (60.5%)	3,722 (61.5%)
Often	882 (13.2%)	807 (13.3%)
Rarely	1,136 (17.0%)	1,009 (16.7%)
Never	558 (8.3%)	466 (7.7%)
(Missing)	69 (1.0%)	46 (0.8%)
Year of birth, n (%)		
1943 or earlier (current age: 80+ years)	49 (0.7%)	17 (0.3%)
1943-1953 (current age: 70-79 years)	213 (3.2%)	143 (2.4%)
1953-1963 (current age: 60-69 years)	669 (10.0%)	485 (8.0%)
1963-1973 (current age: 50-59 years)	835 (12.5%)	683 (11.3%)
1973-1983 (current age: 40-49 years)	1,090 (16.3%)	1,071 (17.7%)
1983-1993 (current age: 30-39 years)	1,468 (21.9%)	1,461 (24.1%)
1993-1998 (current age: 25-29 years)	736 (11.0%)	774 (12.8%)
1998-2005 (current age: 18-24 years)	1,637 (24.4%)	1,416 (23.4%)
(Missing)	0 (0%)	0 (0%)
Age of participant		
Mean	38.4	37.2
Standard Deviation	15.6	14.5
Min, Max	18.0, 95.0	18.0, 96.0
Gender, n (%)		
Male	3,173 (47.4%)	3,335 (55.1%)
Female	3,523 (52.6%)	2,715 (44.9%)
Other	0 (0%)	0 (0%)
(Missing)	0 (0%)	0 (0%)
Respondent marital status, n (%)		
Single/Never been married	1,154 (17.2%)	1,087 (18.0%)
Married	5,054 (75.5%)	4,612 (76.2%)
Separated	26 (0.4%)	20 (0.3%)
Divorced	14 (0.2%)	10 (0.2%)
Widowed	242 (3.6%)	196 (3.2%)
Domestic partner	153 (2.3%)	104 (1.7%)
(Missing)	54 (0.8%)	22 (0.4%)
Education (years), n (%)		
Up to 8	4,967 (74.2%)	4,196 (69.4%)
9-15	1,426 (21.3%)	1,480 (24.5%)
16+	301 (4.5%)	373 (6.2%)
(Missing)	3 (0.0%)	0 (0%)
Employment status, n (%)		
Employed for an employer	1,420 (21.2%)	1,284 (21.2%)
Self-employed	1,647 (24.6%)	1,717 (28.4%)
Retired	149 (2.2%)	121 (2.0%)
Student	275 (4.1%)	270 (4.5%)
Homemaker	2,369 (35.4%)	1,803 (29.8%)
Unemployed and looking for a job	449 (6.7%)	474 (7.8%)
None of these/Other	364 (5.4%)	358 (5.9%)
(Missing)	25 (0.4%)	23 (0.4%)
Current religious service attendance, n (%)		
More than once a week	1,450 (21.7%)	1,403 (23.2%)

Table S16c. Unweighted summary statistics for demographic and childhood variables in India by retention status

**Characteristic**	**Attritors–Not Observed in Wave 2** N = 6,696	**Retained–Observed in Wave 2** N = 6,050
Once a week	1,730 (25.8%)	1,467 (24.2%)
One to three times a month	1,356 (20.2%)	1,345 (22.2%)
A few times a year	1,073 (16.0%)	975 (16.1%)
Never	1,044 (15.6%)	832 (13.8%)
(Missing)	44 (0.7%)	29 (0.5%)
Immigration status, n (%)		
Born in this country	6,613 (98.8%)	5,991 (99.0%)
Born in another country	66 (1.0%)	48 (0.8%)
(Missing)	17 (0.3%)	11 (0.2%)
Parental marital status around age 12, n (%)		
Parents were married	2,874 (42.9%)	2,635 (43.5%)
Parents were divorced	114 (1.7%)	119 (2.0%)
Parents were never married	537 (8.0%)	519 (8.6%)
One or both of them had died	470 (7.0%)	468 (7.7%)
Unsure	58 (0.9%)	23 (0.4%)
(Missing)	2,643 (39.5%)	2,286 (37.8%)
Religious service attendance around age 12, n (%)		
At least once a week	2,745 (41.0%)	2,515 (41.6%)
One to three times a month	1,525 (22.8%)	1,405 (23.2%)
Less than once a month	1,477 (22.1%)	1,272 (21.0%)
Never	785 (11.7%)	702 (11.6%)
(Missing)	164 (2.5%)	156 (2.6%)
Relationship with mother when growing up, n (%)		
Very good	5,982 (89.3%)	5,439 (89.9%)
Somewhat good	429 (6.4%)	365 (6.0%)
Somewhat bad	48 (0.7%)	44 (0.7%)
Very bad	48 (0.7%)	30 (0.5%)
(Does not apply)	142 (2.1%)	134 (2.2%)
(Missing)	47 (0.7%)	39 (0.6%)
Relationship with father when growing up, n (%)		
Very good	5,684 (84.9%)	5,200 (85.9%)
Somewhat good	541 (8.1%)	451 (7.5%)
Somewhat bad	58 (0.9%)	72 (1.2%)
Very bad	70 (1.0%)	39 (0.6%)
(Does not apply)	256 (3.8%)	226 (3.7%)
(Missing)	88 (1.3%)	62 (1.0%)
Felt like an outsider in family when growing up, n (%)		
Yes	1,012 (15.1%)	938 (15.5%)
No	5,653 (84.4%)	5,084 (84.0%)
(Missing)	32 (0.5%)	29 (0.5%)
Experienced abuse when growing up, n (%)		
Yes	724 (10.8%)	763 (12.6%)
No	5,555 (83.0%)	4,910 (81.1%)
(Missing)	417 (6.2%)	378 (6.2%)
Self-rated health when growing up, n (%)		
Excellent	1,100 (16.4%)	1,071 (17.7%)
Very good	2,028 (30.3%)	1,810 (29.9%)

Table S16c. Unweighted summary statistics for demographic and childhood variables in India by retention status

**Characteristic**	**Attritors–Not Observed in Wave 2** N = 6,696	**Retained–Observed in Wave 2** N = 6,050
Good	2,158 (32.2%)	1,880 (31.1%)
Fair	1,139 (17.0%)	1,075 (17.8%)
Poor	243 (3.6%)	191 (3.2%)
(Missing)	28 (0.4%)	23 (0.4%)
Subjective financial status of family growing up, n (%)		
Lived comfortably	2,613 (39.0%)	2,244 (37.1%)
Got by	1,546 (23.1%)	1,484 (24.5%)
Found it difficult	1,370 (20.5%)	1,350 (22.3%)
Found it very difficult	1,124 (16.8%)	942 (15.6%)
(Missing)	43 (0.6%)	29 (0.5%)
Religious affiliation growing up, n (%)		
Christianity	140 (2.1%)	112 (1.9%)
Taoism	0 (0%)	0 (0%)
Confucianism	0 (0%)	0 (0%)
Primal, Animist, or Folk religion	19 (0.3%)	8 (0.1%)
Spiritism	0 (0%)	0 (0%)
Umbanda, Candomblé, and other African-derived religions	0 (0%)	0 (0%)
Chinese folk/traditional religion	0 (0%)	0 (0%)
Islam	862 (12.9%)	748 (12.4%)
Hinduism	5,380 (80.3%)	4,966 (82.1%)
Buddhism	87 (1.3%)	81 (1.3%)
Judaism	0 (0%)	0 (0%)
Sikhism	75 (1.1%)	45 (0.7%)
Baha'i	0 (0%)	0 (0%)
Jainism	5 (0.1%)	5 (0.1%)
Shinto	4 (0.1%)	0 (0%)
Some other religion	39 (0.6%)	27 (0.4%)
No religion/Atheist/Agnostic	2 (0.0%)	6 (0.1%)
(Missing)	84 (1.3%)	53 (0.9%)

Note. N (%); this table is based on non-imputed data. Cumulative percentages for variables may not add up to 100% due to rounding.

Table S16d. Unweighted summary statistics for Wave 1 outcome variables in India by retention status.

**Outcome**	**Attritors–Not Observed in Wave 2** N = 6,696	**Retained–Observed in Wave 2** N = 6,050
Secure flourishing index		
Mean	6.9	6.9
Standard Deviation	1.9	1.8
Min, Max	0.0, 10.0	0.0, 10.0
(Missing)	325 (4.9%)	203 (3.4%)
Flourishing index		
Mean	7.4	7.5
Standard Deviation	2.1	2.0
Min, Max	0.0, 10.0	0.0, 10.0
(Missing)	310 (4.6%)	191 (3.2%)
Happiness & life satisfaction		
Mean	6.7	6.7
Standard Deviation	3.2	3.1
Min, Max	0.0, 10.0	0.0, 10.0
(Missing)	54 (0.8%)	35 (0.6%)
Physical & mental health		
Mean	7.2	7.3
Standard Deviation	3.0	2.9
Min, Max	0.0, 10.0	0.0, 10.0
(Missing)	38 (0.6%)	28 (0.5%)
Meaning & purpose		
Mean	7.5	7.5
Standard Deviation	2.6	2.6
Min, Max	0.0, 10.0	0.0, 10.0
(Missing)	152 (2.3%)	94 (1.6%)
Character & virtue		
Mean	7.7	7.9
Standard Deviation	2.5	2.5
Min, Max	0.0, 10.0	0.0, 10.0
(Missing)	101 (1.5%)	64 (1.1%)
Close social relationships		
Mean	7.9	8.0
Standard Deviation	2.7	2.7
Min, Max	0.0, 10.0	0.0, 10.0
(Missing)	64 (1.0%)	42 (0.7%)
Financial & material security		
Mean	4.1	4.0
Standard Deviation	3.7	3.7
Min, Max	0.0, 10.0	0.0, 10.0
(Missing)	24 (0.4%)	20 (0.3%)
Happiness		
Mean	6.5	6.5
Standard Deviation	3.6	3.6
Min, Max	0.0, 10.0	0.0, 10.0
(Missing)	22 (0.3%)	16 (0.3%)
Life satisfaction		
Mean	7.0	7.0
Standard Deviation	3.5	3.5
Min, Max	0.0, 10.0	0.0, 10.0

Table S16d. Unweighted summary statistics for Wave 1 outcome variables in India by retention status.

**Outcome**	**Attritors–Not Observed in Wave 2** N = 6,696	**Retained–Observed in Wave 2** N = 6,050
(Missing)	40 (0.6%)	22 (0.4%)
Current life evaluation		
Mean	5.6	5.6
Standard Deviation	3.6	3.6
Min, Max	0.0, 10.0	0.0, 10.0
(Missing)	89 (1.3%)	48 (0.8%)
Future life evaluation		
Mean	7.2	7.4
Standard Deviation	3.1	3.0
Min, Max	0.0, 10.0	0.0, 10.0
(Missing)	702 (10%)	509 (8.4%)
Optimism		
Mean	8.1	8.1
Standard Deviation	2.9	2.9
Min, Max	0.0, 10.0	0.0, 10.0
(Missing)	46 (0.7%)	19 (0.3%)
Freedom to pursue what's important		
Mean	8.1	8.2
Standard Deviation	3.0	2.9
Min, Max	0.0, 10.0	0.0, 10.0
(Missing)	41 (0.6%)	27 (0.4%)
Inner peace, n (%)		
Always	2,935 (43.8%)	2,638 (43.6%)
Often	1,078 (16.1%)	1,005 (16.6%)
Rarely	1,925 (28.7%)	1,810 (29.9%)
Never	684 (10.2%)	547 (9.0%)
(Missing)	75 (1.1%)	50 (0.8%)
Life balance, n (%)		
Always	2,184 (32.6%)	1,919 (31.7%)
Often	1,191 (17.8%)	1,105 (18.3%)
Rarely	2,519 (37.6%)	2,369 (39.2%)
Never	692 (10.3%)	582 (9.6%)
(Missing)	110 (1.6%)	75 (1.2%)
Sense of mastery, n (%)		
Always	3,658 (54.6%)	3,271 (54.1%)
Often	956 (14.3%)	927 (15.3%)
Rarely	1,495 (22.3%)	1,412 (23.3%)
Never	519 (7.8%)	415 (6.9%)
(Missing)	68 (1.0%)	26 (0.4%)
Meaningful activities		
Mean	7.0	6.9
Standard Deviation	3.4	3.4
Min, Max	0.0, 10.0	0.0, 10.0
(Missing)	96 (1.4%)	62 (1.0%)
Understanding purpose		
Mean	8.0	8.0
Standard Deviation	3.0	3.0
Min, Max	0.0, 10.0	0.0, 10.0
(Missing)	75 (1.1%)	41 (0.7%)

Table S16d. Unweighted summary statistics for Wave 1 outcome variables in India by retention status.

Outcome	Attritors–Not Observed in Wave 2<br>N = 6,696	Retained–Observed in Wave 2<br>N = 6,050
Self-rated mental health		
Mean	7.4	7.5
Standard Deviation	3.3	3.3
Min, Max	0.0, 10.0	0.0, 10.0
(Missing)	25 (0.4%)	16 (0.3%)
Traumatic distress, n (%)		
A lot	1,710 (25.5%)	1,540 (25.5%)
Some	1,491 (22.3%)	1,355 (22.4%)
Not very much	525 (7.8%)	526 (8.7%)
Not at all	2,915 (43.5%)	2,601 (43.0%)
(Missing)	55 (0.8%)	28 (0.5%)
Depression symptoms composite, n (%)	3,215 (48.9%)	2,967 (49.6%)
(Missing)	122 (1.8%)	73 (1.2%)
Depression – feel hopeless, n (%)		
Nearly every day	957 (14.3%)	869 (14.4%)
More than half the days	1,090 (16.3%)	1,043 (17.2%)
Several days	1,615 (24.1%)	1,596 (26.4%)
Not at all	2,962 (44.2%)	2,504 (41.4%)
(Missing)	72 (1.1%)	37 (0.6%)
Depression – loss of interest, n (%)		
Nearly every day	1,950 (29.1%)	1,736 (28.7%)
More than half the days	1,159 (17.3%)	1,098 (18.2%)
Several days	1,419 (21.2%)	1,345 (22.2%)
Not at all	2,099 (31.3%)	1,828 (30.2%)
(Missing)	69 (1.0%)	43 (0.7%)
Anxiety symptoms composite, n (%)	2,367 (36.0%)	2,156 (36.0%)
(Missing)	122 (1.8%)	61 (1.0%)
Anxiety – feel on edge, n (%)		
Nearly every day	1,058 (15.8%)	929 (15.3%)
More than half the days	1,039 (15.5%)	991 (16.4%)
Several days	1,596 (23.8%)	1,385 (22.9%)
Not at all	2,933 (43.8%)	2,726 (45.1%)
(Missing)	70 (1.0%)	19 (0.3%)
Anxiety – cannot stop worrying, n (%)		
Nearly every day	1,109 (16.6%)	1,009 (16.7%)
More than half the days	962 (14.4%)	932 (15.4%)
Several days	1,432 (21.4%)	1,252 (20.7%)
Not at all	3,109 (46.4%)	2,806 (46.4%)
(Missing)	85 (1.3%)	50 (0.8%)
Suffering, n (%)		
A lot	1,404 (21.0%)	1,286 (21.3%)
Some	1,917 (28.6%)	1,857 (30.7%)
Not very much	677 (10.1%)	546 (9.0%)
Not at all	2,640 (39.4%)	2,322 (38.4%)
(Missing)	59 (0.9%)	39 (0.6%)
Relationship contentment		
Mean	8.0	8.0
Standard Deviation	3.2	3.1
Min, Max	0.0, 10.0	0.0, 10.0

Table S16d. Unweighted summary statistics for Wave 1 outcome variables in India by retention status.

**Outcome**	**Attritors–Not Observed in Wave 2** N = 6,696	**Retained–Observed in Wave 2** N = 6,050
(Missing)	17 (0.3%)	15 (0.2%)
Relationship satisfaction		
Mean	7.9	8.0
Standard Deviation	3.1	3.1
Min, Max	0.0, 10.0	0.0, 10.0
(Missing)	49 (0.7%)	29 (0.5%)
Social support		
Mean	6.5	6.5
Standard Deviation	3.9	3.9
Min, Max	0.0, 10.0	0.0, 10.0
(Missing)	33 (0.5%)	19 (0.3%)
Intimate/close friend, n (%)		
Yes	5,478 (81.8%)	4,911 (81.2%)
No	1,203 (18.0%)	1,128 (18.6%)
(Missing)	16 (0.2%)	11 (0.2%)
Government approval, n (%)		
Strongly approve	3,671 (54.8%)	3,391 (56.0%)
Somewhat approve	1,796 (26.8%)	1,666 (27.5%)
Neither approve nor disapprove	294 (4.4%)	209 (3.5%)
Somewhat disapprove	337 (5.0%)	308 (5.1%)
Strongly disapprove	420 (6.3%)	363 (6.0%)
(Missing)	179 (2.7%)	114 (1.9%)
Say in government, n (%)		
Agree	5,164 (77.1%)	4,730 (78.2%)
Disagree	1,070 (16.0%)	942 (15.6%)
Unsure	422 (6.3%)	344 (5.7%)
(Missing)	40 (0.6%)	34 (0.6%)
Belonging in country		
Mean	8.4	8.4
Standard Deviation	2.9	2.9
Min, Max	0.0, 10.0	0.0, 10.0
(Missing)	69 (1.0%)	50 (0.8%)
City/place satisfaction, n (%)		
Satisfied	6,215 (92.8%)	5,602 (92.6%)
Dissatisfied	425 (6.3%)	401 (6.6%)
Unsure	56 (0.8%)	46 (0.8%)
(Missing)	1 (0.0%)	1 (0.0%)
Trust within country, n (%)		
All people	1,137 (17.0%)	965 (16.0%)
Most people	1,087 (16.2%)	1,109 (18.3%)
Some people	3,047 (45.5%)	2,810 (46.4%)
Not very many people	549 (8.2%)	522 (8.6%)
None	612 (9.1%)	481 (7.9%)
(Missing)	264 (3.9%)	163 (2.7%)
Number of children		
Mean	1.5	1.5
Standard Deviation	1.8	1.7
Min, Max	0.0, 52.0	0.0, 60.0
(Missing)	10 (0.1%)	8 (0.1%)

Table S16d. Unweighted summary statistics for Wave 1 outcome variables in India by retention status.

**Outcome**	**Attritors–Not Observed in Wave 2** N = 6,696	**Retained–Observed in Wave 2** N = 6,050
Community participation, n (%)		
More than once a week	295 (4.4%)	292 (4.8%)
Once a week	573 (8.6%)	533 (8.8%)
One to three times a month	750 (11.2%)	742 (12.3%)
A few times a year	945 (14.1%)	932 (15.4%)
Never	4,041 (60.3%)	3,481 (57.5%)
(Missing)	92 (1.4%)	70 (1.1%)
Religious attendance, n (%)		
More than once a week	1,450 (21.7%)	1,403 (23.2%)
Once a week	1,730 (25.8%)	1,467 (24.2%)
One to three times a month	1,356 (20.2%)	1,345 (22.2%)
A few times a year	1,073 (16.0%)	975 (16.1%)
Never	1,044 (15.6%)	832 (13.8%)
(Missing)	44 (0.7%)	29 (0.5%)
Loneliness		
Mean	3.8	3.8
Standard Deviation	4.0	4.0
Min, Max	0.0, 10.0	0.0, 10.0
(Missing)	18 (0.3%)	14 (0.2%)
Perceived discrimination, n (%)		
Always	894 (13.4%)	784 (13.0%)
Often	564 (8.4%)	513 (8.5%)
Rarely	1,317 (19.7%)	1,324 (21.9%)
Never	3,806 (56.8%)	3,349 (55.3%)
(Missing)	114 (1.7%)	81 (1.3%)
Orientation to promote good		
Mean	8.3	8.4
Standard Deviation	2.7	2.7
Min, Max	0.0, 10.0	0.0, 10.0
(Missing)	47 (0.7%)	19 (0.3%)
Delayed gratification		
Mean	7.1	7.3
Standard Deviation	3.5	3.4
Min, Max	0.0, 10.0	0.0, 10.0
(Missing)	67 (1.0%)	47 (0.8%)
Hope		
Mean	8.3	8.4
Standard Deviation	2.8	2.8
Min, Max	0.0, 10.0	0.0, 10.0
(Missing)	59 (0.9%)	28 (0.5%)
Gratitude		
Mean	7.7	7.7
Standard Deviation	3.1	3.2
Min, Max	0.0, 10.0	0.0, 10.0
(Missing)	125 (1.9%)	83 (1.4%)
Showing love/care		
Mean	8.2	8.3
Standard Deviation	3.1	3.0
Min, Max	0.0, 10.0	0.0, 10.0

Table S16d. Unweighted summary statistics for Wave 1 outcome variables in India by retention status.

**Outcome**	**Attritors–Not Observed in Wave 2** N = 6,696	**Retained–Observed in Wave 2** N = 6,050
(Missing)	26 (0.4%)	11 (0.2%)
Forgivingness, n (%)		
Always	4,052 (60.5%)	3,722 (61.5%)
Often	882 (13.2%)	807 (13.3%)
Rarely	1,136 (17.0%)	1,009 (16.7%)
Never	558 (8.3%)	466 (7.7%)
(Missing)	69 (1.0%)	46 (0.8%)
Charitable giving, n (%)		
Yes	2,555 (38.2%)	2,377 (39.3%)
No	4,120 (61.5%)	3,644 (60.2%)
(Missing)	22 (0.3%)	30 (0.5%)
Helping strangers, n (%)		
Yes	3,653 (54.6%)	3,419 (56.5%)
No	2,966 (44.3%)	2,570 (42.5%)
(Missing)	77 (1.2%)	61 (1.0%)
Volunteering, n (%)		
Yes	1,886 (28.2%)	1,800 (29.8%)
No	4,765 (71.2%)	4,213 (69.6%)
(Missing)	45 (0.7%)	37 (0.6%)
Self-rated physical health		
Mean	7.0	7.1
Standard Deviation	3.5	3.4
Min, Max	0.0, 10.0	0.0, 10.0
(Missing)	18 (0.3%)	13 (0.2%)
Health problems, n (%)		
Yes	1,789 (26.7%)	1,791 (29.6%)
No	4,844 (72.3%)	4,215 (69.7%)
(Missing)	63 (0.9%)	44 (0.7%)
Pain in past 4 weeks, n (%)		
A lot	1,383 (20.6%)	1,198 (19.8%)
Some	1,967 (29.4%)	1,860 (30.7%)
Not very much	601 (9.0%)	522 (8.6%)
None at all	2,721 (40.6%)	2,459 (40.6%)
(Missing)	24 (0.4%)	11 (0.2%)
Number of cigarettes per day		
Mean	0.7	0.7
Standard Deviation	4.8	5.1
Min, Max	0.0, 97.0	0.0, 97.0
(Missing)	291 (4.3%)	210 (3.5%)
Number of drinks per week		
Mean	0.4	0.4
Standard Deviation	4.5	4.4
Min, Max	0.0, 97.0	0.0, 97.0
(Missing)	299 (4.5%)	217 (3.6%)
Days exercise per week		
Mean	3.0	3.3
Standard Deviation	3.2	3.2
Min, Max	0.0, 7.0	0.0, 7.0
(Missing)	1,468 (22%)	1,250 (21%)

Table S16d. Unweighted summary statistics for Wave 1 outcome variables in India by retention status.

**Outcome**	**Attritors–Not Observed in Wave 2** N = 6,696	**Retained–Observed in Wave 2** N = 6,050
Financial security		
Mean	4.1	4.1
Standard Deviation	4.1	4.1
Min, Max	0.0, 10.0	0.0, 10.0
(Missing)	16 (0.2%)	15 (0.2%)
Material security		
Mean	4.0	3.9
Standard Deviation	4.1	4.1
Min, Max	0.0, 10.0	0.0, 10.0
(Missing)	10 (0.2%)	7 (0.1%)
Educational attainment (16+ years), n (%)		
Up to 8	4,967 (74.2%)	4,196 (69.4%)
9-15	1,426 (21.3%)	1,480 (24.5%)
16+	301 (4.5%)	373 (6.2%)
(Missing)	3 (0.0%)	0 (0%)
Currently employed, n (%)		
Employed for an employer	1,420 (21.2%)	1,284 (21.2%)
Self-employed	1,647 (24.6%)	1,717 (28.4%)
Retired	149 (2.2%)	121 (2.0%)
Student	275 (4.1%)	270 (4.5%)
Homemaker	2,369 (35.4%)	1,803 (29.8%)
Unemployed and looking for a job	449 (6.7%)	474 (7.8%)
None of these/Other	364 (5.4%)	358 (5.9%)
(Missing)	25 (0.4%)	23 (0.4%)
Financially comfortable/getting by, n (%)		
Living comfortably on present income	1,470 (21.9%)	1,354 (22.4%)
Getting by on present income	1,774 (26.5%)	1,665 (27.5%)
Finding it difficult on present income	1,877 (28.0%)	1,669 (27.6%)
Finding it very difficult on present income	1,522 (22.7%)	1,345 (22.2%)
(Missing)	54 (0.8%)	18 (0.3%)
Own home, n (%)		
Someone in this household owns this home	5,298 (79.1%)	4,927 (81.4%)
Someone in this household rents this home	980 (14.6%)	803 (13.3%)
Both	72 (1.1%)	76 (1.3%)
Neither	268 (4.0%)	210 (3.5%)
Rent	0 (0%)	0 (0%)
Own	0 (0%)	0 (0%)
Something else	0 (0%)	0 (0%)
(Missing)	78 (1.2%)	33 (0.6%)
Religious/spiritual connection, n (%)		
Always	4,065 (60.7%)	3,699 (61.1%)
Often	699 (10.4%)	643 (10.6%)
Rarely	1,214 (18.1%)	1,103 (18.2%)
Never	652 (9.7%)	572 (9.5%)
(Missing)	66 (1.0%)	32 (0.5%)
Belief in life after death, n (%)		
Yes	3,171 (47.4%)	2,953 (48.8%)
No	2,885 (43.1%)	2,414 (39.9%)
Unsure	584 (8.7%)	628 (10.4%)

Table S16d. Unweighted summary statistics for Wave 1 outcome variables in India by retention status.

**Outcome**	**Attritors–Not Observed in Wave 2**<br>N = 6,696	**Retained–Observed in Wave 2**<br>N = 6,050
(Missing)	56 (0.8%)	56 (0.9%)
Transformative religious experience, n (%)		
Yes	3,680 (55.0%)	3,482 (57.6%)
No	2,807 (41.9%)	2,394 (39.6%)
(Missing)	209 (3.1%)	174 (2.9%)
Religious reading or listening, n (%)		
More than once a day	842 (12.6%)	849 (14.0%)
About once a day	1,529 (22.8%)	1,355 (22.4%)
Sometimes	2,827 (42.2%)	2,609 (43.1%)
Never	1,454 (21.7%)	1,213 (20.1%)
(Missing)	44 (0.7%)	24 (0.4%)
Prayer or meditation, n (%)		
More than once a day	2,274 (34.0%)	2,017 (33.3%)
About once a day	2,047 (30.6%)	1,833 (30.3%)
Sometimes	1,693 (25.3%)	1,566 (25.9%)
Never	659 (9.8%)	625 (10.3%)
(Missing)	23 (0.3%)	9 (0.1%)
Belief in God/gods/spiritual forces, n (%)		
One God	3,784 (56.5%)	3,363 (55.6%)
More than one god	2,454 (36.7%)	2,270 (37.5%)
An impersonal spiritual force	132 (2.0%)	126 (2.1%)
None of these	201 (3.0%)	186 (3.1%)
Unsure	111 (1.7%)	91 (1.5%)
(Missing)	14 (0.2%)	13 (0.2%)
Religious centrality, n (%)		
Agree	5,659 (84.5%)	5,201 (86.0%)
Disagree	644 (9.6%)	574 (9.5%)
Not relevant	105 (1.6%)	99 (1.6%)
Unsure	258 (3.8%)	157 (2.6%)
(Missing)	31 (0.5%)	19 (0.3%)
Religious/spiritual comfort, n (%)		
Agree	5,902 (88.1%)	5,343 (88.3%)
Disagree	565 (8.4%)	510 (8.4%)
Not relevant	75 (1.1%)	67 (1.1%)
Unsure	138 (2.1%)	115 (1.9%)
(Missing)	15 (0.2%)	15 (0.2%)
Feel loved by God, n (%)		
Agree	6,032 (90.1%)	5,463 (90.3%)
Disagree	502 (7.5%)	432 (7.1%)
Not relevant	57 (0.8%)	73 (1.2%)
Unsure	94 (1.4%)	77 (1.3%)
(Missing)	12 (0.2%)	5 (0.1%)
Feel punished by God, n (%)		
Agree	4,214 (62.9%)	3,878 (64.1%)
Disagree	2,118 (31.6%)	1,845 (30.5%)
Not relevant	142 (2.1%)	107 (1.8%)
Unsure	210 (3.1%)	199 (3.3%)
(Missing)	12 (0.2%)	20 (0.3%)
Experienced religious criticism, n (%)		

Table S16d. Unweighted summary statistics for Wave 1 outcome variables in India by retention status.

Outcome	Attritors–Not Observed in Wave 2<br>N = 6,696	Retained–Observed in Wave 2<br>N = 6,050
Agree	4,093 (61.1%)	3,833 (63.4%)
Disagree	2,143 (32.0%)	1,793 (29.6%)
Not relevant	132 (2.0%)	128 (2.1%)
Unsure	292 (4.4%)	273 (4.5%)
(Missing)	36 (0.5%)	24 (0.4%)
Faith-sharing, n (%)		
Agree	4,507 (67.3%)	4,383 (72.5%)
Disagree	1,765 (26.4%)	1,322 (21.8%)
Not relevant	184 (2.7%)	155 (2.6%)
Unsure	210 (3.1%)	164 (2.7%)
(Missing)	30 (0.4%)	26 (0.4%)

Note. N (%); this table is based on non-imputed data. Cumulative percentages for variables may not add up to 100% due to rounding.

Table S16e. Summary of fitted attrition model in India

Characteristic	Odds Ratio	95% CI	p-value
**ANNUAL_WEIGHT_R2**	0.82	0.77, 0.87	1.55e-10
**Happiness & life satisfaction**	1.00	0.94, 1.06	0.967
**Physical & mental health**	1.02	0.96, 1.08	0.611
**Meaning & purpose**	0.95	0.88, 1.01	0.120
**Character & virtue**	1.06	1.01, 1.12	0.022
**Close social relationships**	1.01	0.95, 1.06	0.797
**Financial & material security**	0.96	0.91, 1.01	0.146
**Extraversion**	1.05	1.00, 1.10	0.063
**Openness to experience**	1.00	0.96, 1.05	0.880
**Agreeableness**	1.01	0.96, 1.06	0.683
**Conscientiousness**	1.05	0.99, 1.11	0.092
**Neuroticism**	0.99	0.95, 1.04	0.796
**Depression symptoms composite**	1.03	0.98, 1.08	0.232
**Anxiety symptoms composite**	1.00	0.95, 1.05	0.982
**Loneliness**	0.98	0.93, 1.03	0.454
**Days exercise per week**	1.04	0.99, 1.10	0.112
**Year of birth (age group)**			
1983-1993 (current age: 30-39 years)	—	—	
1973-1983 (current age: 40-49 years)	0.99	0.88, 1.13	0.931
1998-2005 (current age: 18-24 years)	1.14	0.95, 1.36	0.150
1993-1998 (current age: 25-29 years)	1.05	0.91, 1.22	0.483
1963-1973 (current age: 50-59 years)	0.88	0.74, 1.04	0.132
1953-1963 (current age: 60-69 years)	0.81	0.67, 0.98	0.027
1943-1953 (current age: 70-79 years)	0.70	0.51, 0.95	0.022
1943 or earlier (current age: 80+ years)	0.36	0.17, 0.75	0.007
**Gender of respondent**			
Female	—	—	
Male	1.33	1.18, 1.49	4.07e-06
**Marital status**			
Married	—	—	
Single/Never been married	0.94	0.79, 1.11	0.449
Widowed	1.26	0.95, 1.66	0.113
Domestic partner	0.77	0.54, 1.11	0.167
Separated	1.01	0.45, 2.23	0.986
Divorced	0.85	0.29, 2.46	0.761
**Employment status**			
Homemaker	—	—	
Self-employed	1.18	1.02, 1.36	0.028
Employed for an employer	1.05	0.91, 1.21	0.536
Unemployed and looking for a job	1.14	0.92, 1.41	0.241
Student	1.02	0.78, 1.34	0.894
None of these/Other	1.13	0.91, 1.41	0.254
Retired	1.17	0.80, 1.70	0.421
**Religious attendance**			
Once a week	—	—	
More than once a week	1.13	0.99, 1.30	0.076
One to three times a month	1.13	0.98, 1.30	0.083
A few times a year	1.03	0.88, 1.21	0.683
Never	1.02	0.86, 1.21	0.833
**Educational attainment (16+ years)**			
Up to 8	—	—	
9-15	1.00	0.90, 1.12	0.975

Table S16e. Summary of fitted attrition model in India

**Characteristic**	**Odds Ratio**	**95% CI**	**p-value**
16+	1.09	0.92, 1.30	0.319
**Born in This country**			
Born in this country	—	—	
Born in another country	0.83	0.48, 1.42	0.487
**Race plurality (prominent race/ethnic group [0] or not [1])**	1.00	0.95, 1.05	0.944
**Urbanicity**			
A small town or village	—	—	
A rural area or on a farm	0.97	0.86, 1.08	0.570
A large city	0.87	0.77, 1.0	0.041
A suburb of a large city	0.86	0.69, 1.07	0.173
**Monthly household income**			
India: 7,501 – 10,000 rupees	—	—	
India: 10,001 – 15,000 rupees	1.16	0.98, 1.36	0.086
India: 4,001 – 5,000 rupees	1.09	0.90, 1.33	0.381
India: 5,001 – 6,000 rupees	0.91	0.76, 1.09	0.288
India: 15,001 – 20,000 rupees	1.18	0.96, 1.45	0.120
India: 1,001 – 2,000 rupees	1.08	0.88, 1.33	0.456
India: 2,001 – 3,000 rupees	0.96	0.78, 1.18	0.687
India: 20,001 – 35,000 rupees	1.02	0.82, 1.27	0.853
India: 3,001 – 4,000 rupees	1.06	0.86, 1.31	0.570
India: 6,001 – 7,500 rupees	1.19	0.93, 1.51	0.164
India: 1,000 rupees or less	0.95	0.75, 1.19	0.635
(None/No household income)	0.93	0.72, 1.22	0.610
India: 35,001 – 50,000 rupees	1.01	0.73, 1.39	0.960
India: More than 50,000 rupees	1.28	0.87, 1.89	0.203

Abbreviations: CI = Confidence Interval, OR = Odds Ratio

Notes. N=12765; attrition weights were estimated using the 'survey::svyglm(family=quasibinomial('logit'))' function. All continuous predictors were standardized and all categorical predictors used the most common category as the reference group. Reported p-values are based on the fitted regression model and no adjustments for multiple testing were done within this table.

Table S16f. Summary of principal components in India

PC	Percent Explained by each PC	Cumulative Percent Explained
1	30.50	30.50
2	4.59	35.09
3	2.75	37.84
4	2.31	40.15
5	1.89	42.04
6	1.75	43.79
**7**	**1.63**	**45.42**
8	1.53	46.95
9	1.44	48.39
10	1.41	49.81
11	1.31	51.12
12	1.30	52.42
13	1.22	53.63
14	1.21	54.84
15	1.17	56.01
16	1.14	57.16
17	1.09	58.25
18	1.07	59.32
19	1.06	60.38
20	1.03	61.41

Notes. N=12765; PCA was conducted using 'survey::svyprcomp(.)' function using all available contemporaneous exposures at wave 1. All PCs were standardized prior to being used as predictors. The bolded row represented the number of retained components for analysis was 7.

Table S16g. Associations of forgivingness with adult well-being and other outcomes at Wave 2 in India

Outcome	Model 1: Demographic and Childhood Variables as Covariates					Model 2: Demographic, Childhood, and Other Wave 1 Confounding Variables (Via Principal Components) as Covariates				
	RR	ES	95% CI	SE	p-value	RR	ES	95% CI	SE	p-value
Human Flourishing										
Secure flourishing index		0.01	(-0.01,0.04)	0.01	0.369		0.01	(-0.02,0.03)	0.01	0.635
Flourishing index		0.02	(-0.00,0.04)	0.01	0.112		0.01	(-0.02,0.03)	0.01	0.569
Happiness & life satisfaction		0.00	(-0.03,0.03)	0.01	0.982		-0.01	(-0.03,0.02)	0.01	0.554
Physical & mental health		-0.01	(-0.04,0.02)	0.01	0.449		-0.02	(-0.04,0.01)	0.01	0.205
Meaning & purpose		0.01	(-0.02,0.04)	0.02	0.461		0.00	(-0.03,0.03)	0.02	0.943
Character & virtue		0.02	(-0.01,0.04)	0.01	0.232		0.01	(-0.02,0.03)	0.01	0.537
Close social relationships		0.04	(0.01,0.08)	0.02	0.005*		0.04	(0.01,0.07)	0.02	0.019*
Financial & material security		-0.02	(-0.05,0.02)	0.02	0.357		0.00	(-0.03,0.03)	0.02	0.982
Psychological Well-Being										
Happiness (a)		-0.01	(-0.04,0.02)	0.02	0.510		-0.02	(-0.05,0.01)	0.01	0.212
Life satisfaction (a)		0.01	(-0.02,0.04)	0.01	0.489		0.01	(-0.02,0.04)	0.02	0.703
Current life evaluation		-0.00	(-0.04,0.03)	0.02	0.882		-0.00	(-0.04,0.03)	0.02	0.802
Future life evaluation		0.01	(-0.02,0.05)	0.02	0.378		0.01	(-0.02,0.04)	0.02	0.457
Optimism		0.01	(-0.02,0.05)	0.02	0.423		0.00	(-0.03,0.04)	0.02	0.862
Freedom to pursue what's important		0.01	(-0.02,0.04)	0.01	0.684		-0.00	(-0.03,0.03)	0.01	1.000
Inner peace	1.02		(1.00,1.03)	0.01	0.038*	1.01		(0.99,1.02)	0.01	0.216
Life balance	1.02		(1.01,1.03)	0.01	0.007*	1.01		(1.00,1.02)	0.01	0.188
Sense of mastery	1.02		(1.00,1.03)	0.01	0.010*	1.01		(1.00,1.02)	0.01	0.087
Meaningful activities (c)		0.00	(-0.03,0.03)	0.01	0.861		-0.01	(-0.03,0.02)	0.01	0.718
Understanding purpose (c)		0.02	(-0.01,0.05)	0.02	0.308		0.01	(-0.02,0.04)	0.02	0.637
Self-rated mental health (b)		-0.00	(-0.03,0.03)	0.02	0.809		-0.01	(-0.04,0.02)	0.02	0.527
Psychological Distress										
Traumatic distress	1.01		(1.00,1.03)	0.01	0.135	1.01		(1.00,1.03)	0.01	0.066
Depression symptoms composite	1.01		(0.99,1.03)	0.01	0.220	1.01		(0.99,1.03)	0.01	0.254
Depression – feel hopeless	1.01		(0.99,1.02)	0.01	0.303	1.01		(1.00,1.02)	0.01	0.201
Depression – loss of interest	1.00		(0.99,1.02)	0.01	0.701	1.00		(0.99,1.02)	0.01	0.733
Anxiety symptoms composite	1.01		(1.00,1.03)	0.01	0.078	1.02		(1.00,1.03)	0.01	0.044*
Anxiety – feel on edge	1.00		(0.99,1.02)	0.01	0.670	1.01		(0.99,1.02)	0.01	0.457
Anxiety – cannot stop worrying	1.01		(1.00,1.02)	0.01	0.118	1.01		(1.00,1.03)	0.01	0.075
Suffering	1.01		(0.99,1.02)	0.01	0.410	1.01		(0.99,1.02)	0.01	0.444
Social Well-Being										
Relationship contentment (e)		0.04	(0.01,0.07)	0.02	0.011*		0.03	(0.00,0.06)	0.02	0.031*
Relationship satisfaction (e)		0.03	(0.00,0.06)	0.02	0.033*		0.03	(-0.00,0.06)	0.02	0.081
Social support		0.03	(-0.01,0.06)	0.02	0.105		0.02	(-0.01,0.05)	0.02	0.180
Intimate/close friend	1.01		(0.99,1.02)	0.01	0.323	1.01		(0.99,1.02)	0.01	0.489
Government approval	1.00		(1.00,1.01)	0.00	0.332	1.01		(1.00,1.02)	0.00	0.203
Say in government	1.01		(1.00,1.03)	0.01	0.115	1.01		(1.00,1.03)	0.01	0.072
Belonging in country		0.03	(-0.00,0.06)	0.02	0.059		0.02	(-0.01,0.06)	0.02	0.144
City/place satisfaction	1.00		(0.99,1.01)	0.01	0.420	1.00		(0.99,1.01)	0.01	0.431
Trust within country	1.01		(1.00,1.02)	0.01	0.212	1.01		(0.99,1.02)	0.01	0.313
Social Participation										
Ever been married	1.00		(0.99,1.01)	0.00	0.558	1.00		(0.99,1.01)	0.00	0.492
Currently divorced	1.00		(1.00,1.00)	0.00	0.689	1.00		(1.00,1.00)	0.00	0.631
Number of children		0.01	(-0.02,0.03)	0.01	0.688		0.01	(-0.02,0.04)	0.01	0.395
Weekly+ community participation	1.00		(0.99,1.01)	0.01	0.709	1.00		(0.99,1.01)	0.01	0.916
Weekly+ religious attendance	1.01		(1.00,1.02)	0.01	0.199	1.00		(0.99,1.02)	0.01	0.571
Social Distress										
Loneliness		-0.01	(-0.05,0.03)	0.02	0.722		-0.01	(-0.05,0.03)	0.02	0.616
Perceived discrimination	1.01		(1.00,1.02)	0.01	0.126	1.01		(0.99,1.02)	0.01	0.399
Character & Prosocial Behavior										
Orientation to promote good (d)		0.02	(-0.01,0.06)	0.02	0.155		0.02	(-0.01,0.05)	0.02	0.273
Delayed gratification (d)		0.00	(-0.03,0.03)	0.02	0.875		-0.00	(-0.03,0.03)	0.02	0.795
Hope		0.02	(-0.01,0.05)	0.02	0.177		0.01	(-0.02,0.04)	0.02	0.424
Gratitude		0.00	(-0.03,0.03)	0.02	0.910		-0.00	(-0.03,0.03)	0.02	0.814
Showing love/care		0.05	(0.02,0.08)	0.02	2.32e-03**		0.04	(0.01,0.07)	0.02	0.009*
Forgivingness	1.04		(1.02,1.05)	0.01	2.46e-07***	1.04		(1.02,1.05)	0.01	8.63e-07***
Charitable giving	1.02		(1.00,1.03)	0.01	0.035*	1.01		(1.00,1.03)	0.01	0.062
Helping strangers	1.02		(1.01,1.04)	0.01	0.011*	1.02		(1.00,1.04)	0.01	0.029*
Volunteering	1.02		(1.00,1.04)	0.01	0.023*	1.02		(1.00,1.04)	0.01	0.042*
Physical Health & Health Behavior										
Self-rated physical health (b)		-0.01	(-0.04,0.01)	0.01	0.337		-0.02	(-0.05,0.01)	0.01	0.184
Health problems	0.99		(0.98,1.01)	0.01	0.384	0.99		(0.98,1.01)	0.01	0.307
Pain in past 4 weeks	1.00		(0.99,1.02)	0.01	0.577	1.01		(0.99,1.02)	0.01	0.292
Daily smoker	0.99		(0.98,1.00)	0.00	0.069	0.99		(0.98,1.00)	0.00	0.233
Number of drinks per week		0.00	(-0.03,0.04)	0.02	0.969		0.00	(-0.03,0.04)	0.02	0.890
Days exercise per week		0.04	(0.01,0.08)	0.02	0.019*		0.03	(-0.00,0.07)	0.02	0.071
Socioeconomic Outcomes										
Financial security (f)		-0.01	(-0.04,0.02)	0.02	0.609		0.00	(-0.03,0.04)	0.02	0.834
Material security (f)		-0.02	(-0.05,0.01)	0.02	0.320		-0.00	(-0.03,0.03)	0.01	0.849
Educational attainment (16+ years)	1.00		(1.00,1.00)	0.00	0.814	1.00		(1.00,1.00)	0.00	0.807
Currently employed	1.00		(0.99,1.02)	0.01	0.639	1.00		(0.99,1.02)	0.01	0.684
Financially comfortable/getting by	1.00		(0.99,1.02)	0.01	0.692	1.00		(0.99,1.02)	0.01	0.634
Own home	1.00		(0.99,1.02)	0.01	0.537	1.00		(0.99,1.02)	0.01	0.467
Income – top quintile	1.00		(0.98,1.01)	0.01	0.592	1.00		(0.98,1.01)	0.01	0.490
Religion & Spirituality										
Religious/spiritual connection	1.02		(1.00,1.03)	0.01	0.026*	1.01		(1.00,1.03)	0.01	0.127
Belief in life after death	1.01		(0.99,1.03)	0.01	0.447	1.01		(0.99,1.02)	0.01	0.505
Transformative religious experience	1.01		(0.99,1.03)	0.01	0.195	1.00		(0.99,1.02)	0.01	0.559
Religious reading or listening	1.02		(1.01,1.03)	0.01	1.43e-03**	1.01		(1.00,1.02)	0.01	0.098
Prayer or meditation	1.02		(1.00,1.03)	0.01	0.033*	1.01		(1.00,1.02)	0.01	0.192
Belief in God/gods/spiritual forces	1.00		(1.00,1.01)	0.00	0.597	1.00		(1.00,1.01)	0.00	0.652
Religious centrality	1.01		(0.99,1.02)	0.01	0.327	1.00		(0.99,1.01)	0.01	0.679
Religious/spiritual comfort	1.01		(1.00,1.02)	0.01	0.096	1.01		(1.00,1.02)	0.01	0.224
Feel loved by God	1.01		(0.99,1.02)	0.01	0.327	1.00		(0.99,1.02)	0.01	0.544
Feel punished by God	0.99		(0.97,1.00)	0.01	0.051	0.99		(0.98,1.00)	0.01	0.135
Experienced religious criticism	1.00		(0.99,1.02)	0.01	0.630	1.01		(0.99,1.02)	0.01	0.454
Faith-sharing	1.02		(1.01,1.04)	0.01	4.73e-03**	1.02		(1.01,1.04)	0.01	0.008*

Notes. N=12765; Reference for focal predictor: never/rarely. RR, risk-ratio, null effect is 1.00; ES, effect size measure for standardized regression coefficient, null effect is 0.00; SE, standard error, the SE reported for binary/Likert-type outcomes where risk-ratios are on the log(RR) scale; CI, confidence interval; p-value, a Wald-type test of the null hypothesis that the effect of the focal predictor is zero; (a) item part of the Happiness & Life Satisfaction domain of the Secure Flourishing Index; (b) item part of the Physical & Mental Health domain of the Secure Flourishing Index; (c) item part of the Meaning & Purpose domain of the Secure Flourishing Index; (d) item part of the Character & Virtue domain of the Secure Flourishing Index; (e) item part of the Subjective Social Connectedness domain of the Secure Flourishing Index; (f) item part of the Financial & Material Security domain of the Secure Flourishing Index.

Multiple imputation was performed to impute missing data on the covariates, exposure, and outcomes. All models controlled for sociodemographic and childhood factors assessed at Wave 1. For Model 2 with PC (principal components), the first seven principal components of the entire set of contemporaneous confounders assessed at Wave 1 were included as additional covariates of the outcomes at Wave 2.

An outcome-wide analytic approach was used, and a separate model was run for each outcome. A different type of model was run depending on the nature of the outcome: (1) for each binary outcome, a weighted generalized linear model (with a log link and Poisson distribution) was used to estimate an RR; and (2) for each continuous outcome, a weighted linear regression model was used to estimate a ES. All effect sizes were standardized. For continuous outcomes, the ES represents the change in SD on the outcome between the lower and upper categories of the binary focal predictor. For binary outcomes, the RR represents the change in risk of being in the upper category compared to the lower category between the lower and upper categories of the binary focal predictor.

P-value significance thresholds: $p < 0.05$*, $p < 0.005$**, (Bonferroni) $p < 6.41e-04$***, correction for multiple testing using Bonferroni adjusted significant threshold.

Table S16h. Associations of forgivingness with adult well-being and other outcomes at Wave 2 in India using complete-case analyses with attrition weights.

	Model 1: Demographic and Childhood Variables as Covariates					Model 2: Demographic, Childhood, and Other Wave 1 Confounding Variables (Via Principal Components) as Covariates				
Outcome	RR	ES	95% CI	SE	p-value	RR	ES	95% CI	SE	p-value
Human Flourishing										
Secure flourishing index		0.01	(-0.02,0.05)	0.02	0.405		0.01	(-0.03,0.04)	0.02	0.686
Flourishing index		0.02	(-0.02,0.05)	0.02	0.373		0.00	(-0.03,0.04)	0.02	0.839
Happiness & life satisfaction		0.00	(-0.03,0.04)	0.02	0.837		-0.01	(-0.04,0.02)	0.02	0.543
Physical & mental health		-0.01	(-0.05,0.03)	0.02	0.566		-0.02	(-0.05,0.02)	0.02	0.299
Meaning & purpose		0.02	(-0.02,0.05)	0.02	0.350		0.01	(-0.03,0.04)	0.02	0.761
Character & virtue		0.01	(-0.03,0.04)	0.02	0.654		0.00	(-0.03,0.04)	0.02	0.812
Close social relationships		0.04	(0.00,0.07)	0.02	0.026*		0.03	(-0.00,0.07)	0.02	0.072
Financial & material security		0.00	(-0.03,0.04)	0.02	0.882		0.01	(-0.02,0.05)	0.02	0.494
Psychological Well-Being										
Happiness (a)		-0.01	(-0.05,0.02)	0.02	0.456		-0.03	(-0.06,0.01)	0.02	0.124
Life satisfaction (a)		0.02	(-0.01,0.06)	0.02	0.261		0.01	(-0.03,0.04)	0.02	0.629
Current life evaluation		-0.01	(-0.04,0.03)	0.02	0.726		-0.01	(-0.04,0.02)	0.02	0.580
Future life evaluation		0.01	(-0.02,0.04)	0.02	0.503		0.01	(-0.03,0.04)	0.02	0.620
Optimism		0.02	(-0.02,0.05)	0.02	0.339		0.01	(-0.03,0.04)	0.02	0.737
Freedom to pursue what's important		-0.01	(-0.04,0.03)	0.02	0.640		-0.01	(-0.05,0.02)	0.02	0.453
Inner peace	1.03		(1.01,1.05)	0.01	1.47e-03**	1.02		(1.00,1.04)	0.01	0.040*
Life balance	1.02		(1.00,1.04)	0.01	0.029*	1.01		(0.99,1.02)	0.01	0.498
Sense of mastery	1.03		(1.01,1.05)	0.01	4.25e-04***	1.02		(1.00,1.04)	0.01	0.019*
Meaningful activities (c)		0.01	(-0.03,0.04)	0.02	0.655		-0.00	(-0.04,0.03)	0.02	0.835
Understanding purpose (c)		0.02	(-0.02,0.06)	0.02	0.271		0.01	(-0.02,0.05)	0.02	0.474
Self-rated mental health (b)		-0.00	(-0.04,0.04)	0.02	0.870		-0.01	(-0.05,0.02)	0.02	0.541
Psychological Distress										
Traumatic distress	1.01		(0.99,1.03)	0.01	0.371	1.01		(1.00,1.03)	0.01	0.132
Depression symptoms composite	1.02		(1.00,1.04)	0.01	0.027*	1.02		(1.00,1.04)	0.01	0.035*
Depression – feel hopeless	1.01		(1.00,1.03)	0.01	0.160	1.02		(1.00,1.03)	0.01	0.068
Depression – loss of interest	1.01		(0.99,1.02)	0.01	0.387	1.01		(0.99,1.03)	0.01	0.357
Anxiety symptoms composite	1.02		(1.00,1.04)	0.01	0.015*	1.03		(1.01,1.04)	0.01	3.65e-03**
Anxiety – feel on edge	1.00		(0.99,1.02)	0.01	0.803	1.01		(0.99,1.03)	0.01	0.337
Anxiety – cannot stop worrying	1.02		(1.00,1.04)	0.01	0.020*	1.02		(1.01,1.04)	0.01	3.55e-03**
Suffering	1.01		(0.99,1.03)	0.01	0.254	1.01		(0.99,1.03)	0.01	0.273
Social Well-Being										
Relationship contentment (e)		0.04	(0.00,0.08)	0.02	0.028*		0.03	(-0.00,0.07)	0.02	0.066
Relationship satisfaction (e)		0.03	(-0.01,0.06)	0.02	0.098		0.02	(-0.01,0.06)	0.02	0.195
Social support		0.05	(0.01,0.08)	0.02	0.009*		0.05	(0.01,0.08)	0.02	0.010*
Intimate/close friend	1.01		(0.99,1.02)	0.01	0.216	1.01		(0.99,1.02)	0.01	0.291
Government approval	1.00		(0.99,1.02)	0.01	0.495	1.01		(0.99,1.02)	0.01	0.303
Say in government	1.01		(1.00,1.03)	0.01	0.100	1.02		(1.00,1.03)	0.01	0.030*
Belonging in country		0.02	(-0.02,0.06)	0.02	0.292		0.02	(-0.02,0.05)	0.02	0.372
City/place satisfaction	1.01		(0.99,1.02)	0.01	0.309	1.01		(0.99,1.02)	0.01	0.374
Trust within country	1.01		(0.99,1.02)	0.01	0.361	1.01		(0.99,1.02)	0.01	0.361
Social Participation										
Ever been married	1.01		(1.00,1.02)	0.01	0.217	1.01		(1.00,1.02)	0.01	0.214
Currently divorced	1.00		(1.00,1.00)	0.00	0.359	1.00		(1.00,1.00)	0.00	0.329
Number of children		0.02	(-0.01,0.05)	0.01	0.181		0.03	(-0.00,0.05)	0.01	0.053
Weekly+ community participation	1.00		(0.99,1.01)	0.01	0.689	1.00		(0.99,1.01)	0.01	0.882
Weekly+ religious attendance	1.01		(0.99,1.03)	0.01	0.225	1.01		(0.99,1.02)	0.01	0.458
Social Distress										
Loneliness		-0.01	(-0.05,0.03)	0.02	0.486		-0.02	(-0.05,0.02)	0.02	0.419
Perceived discrimination	1.01		(1.00,1.03)	0.01	0.107	1.01		(0.99,1.02)	0.01	0.253
Character & Prosocial Behavior										
Orientation to promote good (d)		0.02	(-0.01,0.06)	0.02	0.212		0.02	(-0.02,0.06)	0.02	0.291
Delayed gratification (d)		-0.01	(-0.04,0.03)	0.02	0.625		-0.01	(-0.05,0.02)	0.02	0.527
Hope		0.01	(-0.02,0.04)	0.02	0.511		0.00	(-0.03,0.04)	0.02	0.788
Gratitude		-0.01	(-0.04,0.03)	0.02	0.728		-0.01	(-0.04,0.03)	0.02	0.677
Showing love/care		0.06	(0.02,0.10)	0.02	1.32e-03**		0.06	(0.02,0.09)	0.02	2.58e-03**
Forgivingness	1.08		(1.06,1.10)	0.01	2.22e-16***	1.07		(1.06,1.09)	0.01	2.22e-16***
Charitable giving	1.02		(1.00,1.04)	0.01	0.015*	1.02		(1.00,1.04)	0.01	0.015*
Helping strangers	1.03		(1.01,1.05)	0.01	9.09e-04**	1.03		(1.01,1.04)	0.01	4.25e-03**
Volunteering	1.03		(1.01,1.04)	0.01	4.65e-03**	1.02		(1.01,1.04)	0.01	0.008*
Physical Health & Health Behavior										
Self-rated physical health (b)		-0.02	(-0.05,0.02)	0.02	0.373		-0.02	(-0.06,0.01)	0.02	0.219
Health problems	0.99		(0.97,1.00)	0.01	0.109	0.99		(0.97,1.00)	0.01	0.092
Pain in past 4 weeks	1.00		(0.99,1.02)	0.01	0.664	1.01		(0.99,1.03)	0.01	0.284
Daily smoker	1.00		(0.99,1.01)	0.01	0.328	1.00		(0.99,1.01)	0.01	0.913
Number of drinks per week		0.00	(-0.03,0.03)	0.01	0.938		0.00	(-0.02,0.03)	0.01	0.877
Days exercise per week		0.05	(0.01,0.09)	0.02	0.022*		0.04	(-0.00,0.08)	0.02	0.055
Socioeconomic Outcomes										
Financial security (f)		0.01	(-0.03,0.04)	0.02	0.713		0.01	(-0.02,0.05)	0.02	0.421
Material security (f)		-0.00	(-0.04,0.03)	0.02	0.930		0.01	(-0.03,0.04)	0.02	0.685
Educational attainment (16+ years)	1.00		(1.00,1.00)	0.00	0.923	1.00		(1.00,1.00)	0.00	0.713
Currently employed	1.00		(0.99,1.02)	0.01	0.842	1.00		(0.99,1.02)	0.01	0.764
Financially comfortable/getting by	1.00		(0.99,1.02)	0.01	0.617	1.00		(0.99,1.02)	0.01	0.651
Own home	1.00		(0.99,1.02)	0.01	0.638	1.00		(0.99,1.02)	0.01	0.626
Income – top quintile	0.99		(0.97,1.01)	0.01	0.362	0.99		(0.97,1.01)	0.01	0.248
Religion & Spirituality										
Religious/spiritual connection	1.03		(1.02,1.05)	0.01	6.38e-05***	1.02		(1.01,1.04)	0.01	4.73e-03**
Belief in life after death	1.01		(0.99,1.02)	0.01	0.488	1.00		(0.99,1.02)	0.01	0.741
Transformative religious experience	1.01		(1.00,1.03)	0.01	0.114	1.01		(0.99,1.03)	0.01	0.371
Religious reading or listening	1.03		(1.01,1.05)	0.01	2.48e-04***	1.02		(1.00,1.03)	0.01	0.026*
Prayer or meditation	1.01		(1.00,1.03)	0.01	0.094	1.01		(0.99,1.03)	0.01	0.289
Belief in God/gods/spiritual forces	1.00		(0.99,1.01)	0.00	0.793	1.00		(0.99,1.01)	0.00	0.906
Religious centrality	1.01		(1.00,1.02)	0.01	0.189	1.01		(0.99,1.02)	0.01	0.366
Religious/spiritual comfort	1.02		(1.00,1.03)	0.01	0.015*	1.01		(1.00,1.03)	0.01	0.059
Feel loved by God	1.01		(1.00,1.02)	0.01	0.149	1.01		(0.99,1.02)	0.01	0.296
Feel punished by God	0.99		(0.97,1.00)	0.01	0.160	0.99		(0.98,1.01)	0.01	0.292
Experienced religious criticism	1.01		(0.99,1.02)	0.01	0.492	1.01		(0.99,1.03)	0.01	0.248
Faith-sharing	1.03		(1.01,1.05)	0.01	2.57e-04***	1.03		(1.01,1.05)	0.01	5.45e-04***

Table S16h. Associations of forgivingness with adult well-being and other outcomes at Wave 2 in India using complete-case analyses with attrition weights.

	Model 1: Demographic and Childhood Variables as Covariates					Model 2: Demographic, Childhood, and Other Wave 1 Confounding Variables (Via Principal Components) as Covariates				
Outcome	RR	ES	95% CI	SE	p-value	RR	ES	95% CI	SE	p-value

Notes. N=6374; Reference for focal predictor: never/rarely. RR, risk-ratio, null effect is 1.00; ES, effect size measure for standardized regression coefficient, null effect is 0.00; SE, standard error, the SE reported for binary/Likert-type outcomes where risk-ratios are on the log(RR) scale; CI, confidence interval; p-value, a Wald-type test of the null hypothesis that the effect of the focal predictor is zero; (a) item part of the Happiness & Life Satisfaction domain of the Secure Flourishing Index; (b) item part of the Physical & Mental Health domain of the Secure Flourishing Index; (c) item part of the Meaning & Purpose domain of the Secure Flourishing Index; (d) item part of the Character & Virtue domain of the Secure Flourishing Index; (e) item part of the Subjective Social Connectedness domain of the Secure Flourishing Index; (f) item part of the Financial & Material Security domain of the Secure Flourishing Index.

Attrition weights were computed to adjust the complete case data (those who responded at Wave 2 to at least 50% of the questions) and multiple imputation was used to impute missing data on all remaining within wave on the covariates, exposure, and outcomes. All models controlled for sociodemographic and childhood factors assessed at Wave 1. For Model 2 with PC (principal components), the first seven principal components of the entire set of contemporaneous confounders assessed at Wave 1 were included as additional covariates of the outcomes at Wave 2.

An outcome-wide analytic approach was used, and a separate model was run for each outcome. A different type of model was run depending on the nature of the outcome: (1) for each binary outcome, a weighted generalized linear model (with a log link and Poisson distribution) was used to estimate an RR; and (2) for each continuous outcome, a weighted linear regression model was used to estimate a ES. All effect sizes were standardized. For continuous outcomes, the ES represents the change in SD on the outcome between the lower and upper categories of the binary focal predictor. For binary outcomes, the RR represents the change in risk of being in the upper category compared to the lower category between the lower and upper categories of the binary focal predictor.

P-value significance thresholds: $p < 0.05$*, $p < 0.005$**, (Bonferroni) $p < 6.41e-04$***, correction for multiple testing using Bonferroni adjusted significant threshold.

Table S16i. Sensitivity analysis of forgivingness outcome-wide results to unmeasured confounding using E-values in India

	Multiple Imputation				Complete Case w/ Attrition Weights			
	Model 1: Demographics and Childhood Variables as Covariates		Model 2: Demographics, Childhood, and Other Wave 1 Confounders (Via Principal Components) as Covariates		Model 1: Demographics and Childhood Variables as Covariates		Model 2: Demographics, Childhood, and Other Wave 1 Confounders (Via Principal Components) as Covariates	
Outcome	EE	ECI	EE	ECI	EE	ECI	EE	ECI
Human Flourishing								
Secure flourishing index	1.18	1.00	1.12	1.00	1.21	1.00	1.13	1.00
Flourishing index	1.24	1.00	1.13	1.00	1.22	1.00	1.09	1.00
Happiness & life satisfaction	1.03	1.00	1.14	1.00	1.10	1.00	1.17	1.00
Physical & mental health	1.17	1.00	1.23	1.00	1.18	1.00	1.24	1.00
Meaning & purpose	1.19	1.00	1.05	1.00	1.23	1.00	1.12	1.00
Character & virtue	1.23	1.00	1.15	1.00	1.15	1.00	1.11	1.00
Close social relationships	1.43	1.21	1.38	1.13	1.40	1.11	1.35	1.00
Financial & material security	1.21	1.00	1.03	1.00	1.08	1.00	1.19	1.00
Psychological Well-Being								
Happiness	1.17	1.00	1.24	1.00	1.20	1.00	1.31	1.00
Life satisfaction	1.17	1.00	1.12	1.00	1.26	1.00	1.16	1.00
Current life evaluation	1.08	1.00	1.11	1.00	1.12	1.00	1.16	1.00
Future life evaluation	1.21	1.00	1.19	1.00	1.18	1.00	1.16	1.00
Optimism	1.20	1.00	1.09	1.00	1.23	1.00	1.13	1.00
Freedom to pursue what's important	1.13	1.00	1.00	1.00	1.15	1.00	1.20	1.00
Inner peace	1.23	1.05	1.17	1.00	1.34	1.19	1.26	1.05
Life balance	1.27	1.13	1.18	1.00	1.26	1.07	1.13	1.00
Sense of mastery	1.25	1.11	1.19	1.00	1.35	1.21	1.27	1.10
Meaningful activities	1.08	1.00	1.12	1.00	1.14	1.00	1.10	1.00
Understanding purpose	1.22	1.00	1.14	1.00	1.26	1.00	1.20	1.00
Self-rated mental health	1.10	1.00	1.17	1.00	1.09	1.00	1.18	1.00
Psychological Distress								
Traumatic distress	1.20	1.00	1.22	1.00	1.16	1.00	1.21	1.00
Depression symptoms composite	1.17	1.00	1.17	1.00	1.26	1.08	1.26	1.06
Depression – feel hopeless	1.15	1.00	1.17	1.00	1.20	1.00	1.23	1.00
Depression – loss of interest	1.09	1.00	1.09	1.00	1.15	1.00	1.16	1.00
Anxiety symptoms composite	1.21	1.00	1.23	1.03	1.28	1.11	1.31	1.16
Anxiety – feel on edge	1.09	1.00	1.13	1.00	1.08	1.00	1.16	1.00
Anxiety – cannot stop worrying	1.18	1.00	1.20	1.00	1.26	1.09	1.31	1.16
Suffering	1.13	1.00	1.12	1.00	1.18	1.00	1.17	1.00
Social Well-Being								
Relationship contentment	1.39	1.16	1.35	1.09	1.40	1.11	1.36	1.00
Relationship satisfaction	1.35	1.09	1.31	1.00	1.32	1.00	1.28	1.00
Social support	1.30	1.00	1.27	1.00	1.45	1.19	1.44	1.18
Intimate/close friend	1.15	1.00	1.13	1.00	1.17	1.00	1.16	1.00
Government approval	1.12	1.00	1.13	1.00	1.11	1.00	1.14	1.00
Say in government	1.20	1.00	1.22	1.00	1.21	1.00	1.25	1.07
Belonging in country	1.34	1.00	1.28	1.00	1.25	1.00	1.23	1.00
City/place satisfaction	1.11	1.00	1.11	1.00	1.13	1.00	1.12	1.00
Trust within country	1.15	1.00	1.14	1.00	1.14	1.00	1.14	1.00
Social Participation								
Ever been married	1.08	1.00	1.09	1.00	1.14	1.00	1.14	1.00
Currently divorced	1.03	1.00	1.03	1.00	1.04	1.00	1.05	1.00
Number of children	1.12	1.00	1.19	1.00	1.25	1.00	1.31	1.00
Weekly+ community participation	1.07	1.00	1.04	1.00	1.08	1.00	1.05	1.00
Weekly+ religious attendance	1.17	1.00	1.11	1.00	1.18	1.00	1.14	1.00
Social Distress								
Loneliness	1.14	1.00	1.17	1.00	1.20	1.00	1.22	1.00
Perceived discrimination	1.19	1.00	1.13	1.00	1.20	1.00	1.17	1.00
Character & Prosocial Behavior								
Orientation to promote good	1.29	1.00	1.24	1.00	1.29	1.00	1.26	1.00
Delayed gratification	1.08	1.00	1.10	1.00	1.16	1.00	1.18	1.00
Hope	1.26	1.00	1.19	1.00	1.18	1.00	1.11	1.00
Gratitude	1.06	1.00	1.10	1.00	1.13	1.00	1.14	1.00
Showing love/care	1.45	1.24	1.40	1.18	1.53	1.28	1.51	1.26
Forgivingness	1.41	1.31	1.40	1.29	1.68	1.56	1.64	1.53
Charitable giving	1.24	1.05	1.22	1.00	1.29	1.11	1.29	1.11
Helping strangers	1.30	1.13	1.28	1.08	1.35	1.20	1.32	1.16
Volunteering	1.27	1.09	1.25	1.04	1.31	1.15	1.30	1.14
Physical Health & Health Behavior								
Self-rated physical health	1.20	1.00	1.24	1.00	1.23	1.00	1.27	1.00
Health problems	1.14	1.00	1.16	1.00	1.22	1.00	1.23	1.00
Pain in past 4 weeks	1.11	1.00	1.15	1.00	1.10	1.00	1.17	1.00
Daily smoker	1.16	1.00	1.13	1.00	1.12	1.00	1.04	1.00
Number of drinks per week	1.04	1.00	1.08	1.00	1.05	1.00	1.07	1.00
Days exercise per week	1.41	1.15	1.34	1.00	1.44	1.14	1.40	1.00
Socioeconomic Outcomes								
Financial security	1.16	1.00	1.09	1.00	1.13	1.00	1.21	1.00
Material security	1.22	1.00	1.08	1.00	1.06	1.00	1.14	1.00
Educational attainment (16+ years)	1.03	1.00	1.03	1.00	1.02	1.00	1.03	1.00
Currently employed	1.09	1.00	1.09	1.00	1.07	1.00	1.08	1.00
Financially comfortable/getting by	1.09	1.00	1.10	1.00	1.11	1.00	1.11	1.00
Own home	1.11	1.00	1.12	1.00	1.10	1.00	1.11	1.00
Income – top quintile	1.10	1.00	1.11	1.00	1.16	1.00	1.19	1.00
Religion & Spirituality								
Religious/spiritual connection	1.24	1.07	1.19	1.00	1.37	1.24	1.30	1.15
Belief in life after death	1.15	1.00	1.14	1.00	1.14	1.00	1.09	1.00
Transformative religious experience	1.18	1.00	1.12	1.00	1.22	1.00	1.16	1.00
Religious reading or listening	1.27	1.15	1.18	1.00	1.35	1.22	1.26	1.08
Prayer or meditation	1.23	1.06	1.17	1.00	1.23	1.00	1.17	1.00
Belief in God/gods/spiritual forces	1.07	1.00	1.06	1.00	1.05	1.00	1.03	1.00
Religious centrality	1.13	1.00	1.08	1.00	1.16	1.00	1.13	1.00
Religious/spiritual comfort	1.19	1.00	1.15	1.00	1.24	1.09	1.20	1.00
Feel loved by God	1.13	1.00	1.10	1.00	1.17	1.00	1.14	1.00
Feel punished by God	1.21	1.00	1.18	1.00	1.19	1.00	1.16	1.00
Experienced religious criticism	1.10	1.00	1.13	1.00	1.14	1.00	1.18	1.00
Faith-sharing	1.29	1.15	1.28	1.13	1.37	1.23	1.35	1.21

Notes. EE, E-value for estimate; ECI, E-value for the limit of the confidence interval. The formula for calculating E-values can be found in VanderWeele and Ding (2017). E-values for estimate are the minimum strength of association on the risk ratio scale that an unmeasured confounder would need to have with both the exposure and the outcome to fully explain away the observed association between the exposure and outcome, conditional on the measured covariates. E-values for the 95% CI closest to the null denote the minimum strength of association on the risk ratio scale that an unmeasured confounder would need to have with both the exposure and the outcome to shift the CI to include the null value, conditional on the measured covariates.

Table S17a. Weighted summary statistics for demographic and childhood variables in Indonesia

**Characteristic**	**Wave 1** N = 6,992	**Wave 2** N = 2,674
Forgivingness, n (%)		
Always	3,559 (50.9%)	1,356 (50.7%)
Often	2,344 (33.5%)	889 (33.2%)
Rarely	683 (9.8%)	261 (9.8%)
Never	399 (5.7%)	166 (6.2%)
(Missing)	7 (0.1%)	2 (0.1%)
Year of birth, n (%)		
1943 or earlier (current age: 80+ years)	20 (0.3%)	12 (0.5%)
1943-1953 (current age: 70-79 years)	85 (1.2%)	42 (1.6%)
1953-1963 (current age: 60-69 years)	520 (7.4%)	214 (8.0%)
1963-1973 (current age: 50-59 years)	1,171 (16.8%)	475 (17.8%)
1973-1983 (current age: 40-49 years)	1,470 (21.0%)	602 (22.5%)
1983-1993 (current age: 30-39 years)	1,526 (21.8%)	557 (20.8%)
1993-1998 (current age: 25-29 years)	795 (11.4%)	332 (12.4%)
1998-2005 (current age: 18-24 years)	1,404 (20.1%)	441 (16.5%)
(Missing)	0 (0%)	0 (0%)
Age of participant		
Mean	38.9	40.3
Standard Deviation	14.2	14.2
Min, Max	18.0, 99.0	19.0, 93.0
Gender, n (%)		
Male	3,497 (50.0%)	1,345 (50.3%)
Female	3,477 (49.7%)	1,322 (49.4%)
Other	7 (0.1%)	3 (0.1%)
(Missing)	11 (0.2%)	4 (0.2%)
Respondent marital status, n (%)		
Single/Never been married	1,498 (21.4%)	543 (20.3%)
Married	4,715 (67.4%)	1,858 (69.5%)
Separated	83 (1.2%)	38 (1.4%)
Divorced	192 (2.7%)	58 (2.2%)
Widowed	442 (6.3%)	160 (6.0%)
Domestic partner	18 (0.3%)	14 (0.5%)
(Missing)	43 (0.6%)	2 (0.1%)
Education (years), n (%)		
Up to 8	3,509 (50.2%)	1,125 (42.1%)
9-15	3,146 (45.0%)	1,351 (50.5%)
16+	334 (4.8%)	198 (7.4%)
(Missing)	3 (0.0%)	0 (0%)
Employment status, n (%)		
Employed for an employer	1,308 (18.7%)	374 (14.0%)
Self-employed	2,157 (30.9%)	736 (27.5%)
Retired	67 (1.0%)	27 (1.0%)
Student	318 (4.5%)	55 (2.1%)
Homemaker	2,111 (30.2%)	816 (30.5%)
Unemployed and looking for a job	561 (8.0%)	260 (9.7%)
None of these/Other	450 (6.4%)	372 (13.9%)
(Missing)	20 (0.3%)	34 (1.3%)
Current religious service attendance, n (%)		
More than once a week	2,671 (38.2%)	928 (34.7%)
Once a week	2,540 (36.3%)	958 (35.8%)

Table S17a. Weighted summary statistics for demographic and childhood variables in Indonesia

**Characteristic**	**Wave 1** N = 6,992	**Wave 2** N = 2,674
One to three times a month	773 (11.1%)	352 (13.1%)
A few times a year	645 (9.2%)	276 (10.3%)
Never	345 (4.9%)	150 (5.6%)
(Missing)	18 (0.3%)	10 (0.4%)
Immigration status, n (%)		
Born in this country	6,958 (99.5%)	2,661 (99.5%)
Born in another country	34 (0.5%)	13 (0.5%)
(Missing)	0 (0%)	0 (0%)
Parental marital status around age 12, n (%)		
Parents were married	5,503 (78.7%)	2,080 (77.8%)
Parents were divorced	465 (6.7%)	196 (7.3%)
Parents were never married	49 (0.7%)	12 (0.5%)
One or both of them had died	762 (10.9%)	331 (12.4%)
Unsure	109 (1.6%)	30 (1.1%)
(Missing)	104 (1.5%)	24 (0.9%)
Religious service attendance around age 12, n (%)		
At least once a week	5,361 (76.7%)	2,093 (78.3%)
One to three times a month	982 (14.0%)	331 (12.4%)
Less than once a month	320 (4.6%)	114 (4.2%)
Never	278 (4.0%)	114 (4.3%)
(Missing)	51 (0.7%)	21 (0.8%)
Relationship with mother when growing up, n (%)		
Very good	6,217 (88.9%)	2,342 (87.6%)
Somewhat good	597 (8.5%)	239 (8.9%)
Somewhat bad	53 (0.8%)	32 (1.2%)
Very bad	28 (0.4%)	11 (0.4%)
(Does not apply)	69 (1.0%)	39 (1.5%)
(Missing)	28 (0.4%)	10 (0.4%)
Relationship with father when growing up, n (%)		
Very good	6,061 (86.7%)	2,305 (86.2%)
Somewhat good	640 (9.2%)	239 (8.9%)
Somewhat bad	67 (1.0%)	38 (1.4%)
Very bad	52 (0.7%)	19 (0.7%)
(Does not apply)	113 (1.6%)	46 (1.7%)
(Missing)	60 (0.9%)	27 (1.0%)
Felt like an outsider in family when growing up, n (%)		
Yes	356 (5.1%)	149 (5.6%)
No	6,625 (94.8%)	2,523 (94.4%)
(Missing)	11 (0.2%)	1 (0.1%)
Experienced abuse when growing up, n (%)		
Yes	504 (7.2%)	196 (7.3%)
No	6,412 (91.7%)	2,460 (92.0%)
(Missing)	76 (1.1%)	17 (0.6%)
Self-rated health when growing up, n (%)		
Excellent	1,272 (18.2%)	459 (17.2%)
Very good	1,957 (28.0%)	737 (27.6%)
Good	2,453 (35.1%)	927 (34.7%)
Fair	1,251 (17.9%)	528 (19.8%)
Poor	58 (0.8%)	21 (0.8%)
(Missing)	0 (<0.0%)	0 (<0.0%)

Table S17a. Weighted summary statistics for demographic and childhood variables in Indonesia

**Characteristic**	**Wave 1** N = 6,992	**Wave 2** N = 2,674
Subjective financial status of family growing up, n (%)		
Lived comfortably	3,373 (48.2%)	1,224 (45.8%)
Got by	2,963 (42.4%)	1,204 (45.0%)
Found it difficult	454 (6.5%)	174 (6.5%)
Found it very difficult	193 (2.8%)	69 (2.6%)
(Missing)	8 (0.1%)	4 (0.1%)
Religious affiliation growing up, n (%)		
Christianity	515 (7.4%)	216 (8.1%)
Taoism	0 (<0.0%)	0 (<0.0%)
Confucianism	1 (<0.0%)	0 (0%)
Primal, Animist, or Folk religion	0 (<0.0%)	0 (0.0%)
Spiritism	0 (0%)	0 (0%)
Umbanda, Candomblé, and other African-derived religions	0 (0%)	0 (0%)
Chinese folk/traditional religion	0 (0%)	0 (0%)
Islam	6,388 (91.4%)	2,426 (90.7%)
Hinduism	73 (1.0%)	22 (0.8%)
Buddhism	5 (0.1%)	6 (0.2%)
Judaism	0 (0%)	0 (0%)
Sikhism	0 (0%)	0 (0%)
Baha'i	0 (0%)	0 (0%)
Jainism	1 (<0.0%)	0 (0%)
Shinto	0 (0%)	0 (0%)
Some other religion	0 (0%)	0 (0%)
No religion/Atheist/Agnostic	2 (0.0%)	2 (0.1%)
(Missing)	7 (0.1%)	1 (0.0%)

Note. N (%); this table is based on non-imputed data. Cumulative percentages for variables may not add up to 100% due to rounding. Wave 1 characteristics weighted using the Gallup provided sampling weight, ANNUAL_WEIGHT_R2; Wave 2 characteristics weighted accounting for attrition by using the adjusted Wave 1 weight, ANNUAL_WEIGHT_R2, multiplied by the created attrition weight to account for dropout, to maintain nationally representative estimates for Wave 2 characteristics.

Table S17b. Weighted summary statistics for outcome variables in Indonesia

Outcome	Wave 1<br>N = 6,992	Wave 2<br>N = 2,674
Secure flourishing index		
Mean	8.1	7.9
Standard Deviation	1.4	1.4
Min, Max	0.8, 10.0	0.0, 10.0
(Missing)	164 (2.3%)	62 (2.3%)
Flourishing index		
Mean	8.5	8.3
Standard Deviation	1.3	1.4
Min, Max	0.5, 10.0	0.0, 10.0
(Missing)	149 (2.1%)	54 (2.0%)
Happiness & life satisfaction		
Mean	8.0	7.9
Standard Deviation	2.0	2.0
Min, Max	0.0, 10.0	0.0, 10.0
(Missing)	32 (0.5%)	15 (0.6%)
Physical & mental health		
Mean	8.4	8.1
Standard Deviation	1.7	1.9
Min, Max	0.0, 10.0	0.0, 10.0
(Missing)	37 (0.5%)	5 (0.2%)
Meaning & purpose		
Mean	8.7	8.5
Standard Deviation	1.5	1.6
Min, Max	0.0, 10.0	0.0, 10.0
(Missing)	46 (0.7%)	18 (0.7%)
Character & virtue		
Mean	8.5	8.4
Standard Deviation	1.7	1.7
Min, Max	0.0, 10.0	0.0, 10.0
(Missing)	50 (0.7%)	15 (0.6%)
Close social relationships		
Mean	8.7	8.6
Standard Deviation	1.7	1.7
Min, Max	0.0, 10.0	0.0, 10.0
(Missing)	46 (0.7%)	11 (0.4%)
Financial & material security		
Mean	6.3	6.2
Standard Deviation	3.1	3.0
Min, Max	0.0, 10.0	0.0, 10.0
(Missing)	28 (0.4%)	8 (0.3%)
Happiness		
Mean	8.1	7.9
Standard Deviation	2.2	2.2
Min, Max	0.0, 10.0	0.0, 10.0
(Missing)	21 (0.3%)	10 (0.4%)
Life satisfaction		
Mean	8.0	7.9
Standard Deviation	2.3	2.3
Min, Max	0.0, 10.0	0.0, 10.0
(Missing)	19 (0.3%)	7 (0.3%)
Current life evaluation		

Table S17b. Weighted summary statistics for outcome variables in Indonesia

Outcome	Wave 1<br>N = 6,992	Wave 2<br>N = 2,674
Mean	7.0	7.0
Standard Deviation	2.6	2.4
Min, Max	0.0, 10.0	0.0, 10.0
(Missing)	33 (0.5%)	3 (0.1%)
Future life evaluation		
Mean	8.4	8.3
Standard Deviation	2.1	2.0
Min, Max	0.0, 10.0	0.0, 10.0
(Missing)	82 (1.2%)	19 (0.7%)
Optimism		
Mean	9.1	9.1
Standard Deviation	1.7	1.7
Min, Max	0.0, 10.0	0.0, 10.0
(Missing)	24 (0.3%)	2 (<0.1%)
Freedom to pursue what's important		
Mean	8.8	8.6
Standard Deviation	1.8	1.8
Min, Max	0.0, 10.0	0.0, 10.0
(Missing)	20 (0.3%)	1 (<0.1%)
Inner peace, n (%)		
Always	2,485 (35.5%)	767 (28.7%)
Often	2,431 (34.8%)	983 (36.8%)
Rarely	1,472 (21.1%)	725 (27.1%)
Never	581 (8.3%)	188 (7.0%)
(Missing)	23 (0.3%)	11 (0.4%)
Life balance, n (%)		
Always	2,126 (30.4%)	668 (25.0%)
Often	2,631 (37.6%)	1,062 (39.7%)
Rarely	1,726 (24.7%)	714 (26.7%)
Never	474 (6.8%)	220 (8.2%)
(Missing)	35 (0.5%)	9 (0.3%)
Sense of mastery, n (%)		
Always	2,551 (36.5%)	842 (31.5%)
Often	2,819 (40.3%)	1,179 (44.1%)
Rarely	1,328 (19.0%)	504 (18.9%)
Never	262 (3.7%)	131 (4.9%)
(Missing)	32 (0.5%)	17 (0.6%)
Meaningful activities		
Mean	8.5	8.4
Standard Deviation	1.9	1.9
Min, Max	0.0, 10.0	0.0, 10.0
(Missing)	26 (0.4%)	3 (0.1%)
Understanding purpose		
Mean	8.9	8.6
Standard Deviation	1.7	1.8
Min, Max	0.0, 10.0	0.0, 10.0
(Missing)	25 (0.4%)	15 (0.6%)
Self-rated mental health		
Mean	8.6	8.3
Standard Deviation	1.9	2.1
Min, Max	0.0, 10.0	0.0, 10.0

Table S17b. Weighted summary statistics for outcome variables in Indonesia

Outcome	Wave 1<br>N = 6,992	Wave 2<br>N = 2,674
(Missing)	29 (0.4%)	3 (0.1%)
Traumatic distress, n (%)		
A lot	428 (6.1%)	214 (8.0%)
Some	1,143 (16.3%)	512 (19.2%)
Not very much	2,627 (37.6%)	1,128 (42.2%)
Not at all	2,770 (39.6%)	818 (30.6%)
(Missing)	23 (0.3%)	1 (0.1%)
Depression symptoms composite, n (%)	1,375 (19.8%)	571 (21.6%)
(Missing)	56 (0.8%)	28 (1.1%)
Depression – feel hopeless, n (%)		
Nearly every day	398 (5.7%)	176 (6.6%)
More than half the days	396 (5.7%)	149 (5.6%)
Several days	1,846 (26.4%)	797 (29.8%)
Not at all	4,333 (62.0%)	1,549 (57.9%)
(Missing)	19 (0.3%)	3 (0.1%)
Depression – loss of interest, n (%)		
Nearly every day	753 (10.8%)	265 (9.9%)
More than half the days	545 (7.8%)	264 (9.9%)
Several days	2,419 (34.6%)	1,093 (40.9%)
Not at all	3,230 (46.2%)	1,023 (38.3%)
(Missing)	44 (0.6%)	28 (1.1%)
Anxiety symptoms composite, n (%)	1,005 (14.4%)	456 (17.1%)
(Missing)	29 (0.4%)	8 (0.3%)
Anxiety – feel on edge, n (%)		
Nearly every day	501 (7.2%)	235 (8.8%)
More than half the days	415 (5.9%)	187 (7.0%)
Several days	2,034 (29.1%)	813 (30.4%)
Not at all	4,031 (57.6%)	1,432 (53.6%)
(Missing)	11 (0.2%)	6 (0.2%)
Anxiety – cannot stop worrying, n (%)		
Nearly every day	432 (6.2%)	200 (7.5%)
More than half the days	348 (5.0%)	181 (6.8%)
Several days	1,544 (22.1%)	659 (24.7%)
Not at all	4,645 (66.4%)	1,629 (60.9%)
(Missing)	22 (0.3%)	5 (0.2%)
Suffering, n (%)		
A lot	588 (8.4%)	279 (10.4%)
Some	1,217 (17.4%)	538 (20.1%)
Not very much	2,682 (38.4%)	1,095 (41.0%)
Not at all	2,459 (35.2%)	748 (28.0%)
(Missing)	46 (0.7%)	13 (0.5%)
Relationship contentment		
Mean	8.8	8.7
Standard Deviation	1.8	1.9
Min, Max	0.0, 10.0	0.0, 10.0
(Missing)	28 (0.4%)	6 (0.2%)
Relationship satisfaction		
Mean	8.6	8.4
Standard Deviation	1.9	2.0
Min, Max	0.0, 10.0	0.0, 10.0
(Missing)	28 (0.4%)	5 (0.2%)

Table S17b. Weighted summary statistics for outcome variables in Indonesia

**Outcome**	**Wave 1**<br>N = 6,992	**Wave 2**<br>N = 2,674
Social support		
Mean	7.5	7.2
Standard Deviation	2.9	2.9
Min, Max	0.0, 10.0	0.0, 10.0
(Missing)	9 (0.1%)	1 (<0.1%)
Intimate/close friend, n (%)		
Yes	5,380 (76.9%)	2,152 (80.5%)
No	1,601 (22.9%)	510 (19.1%)
(Missing)	11 (0.2%)	11 (0.4%)
Government approval, n (%)		
Strongly approve	1,800 (25.7%)	588 (22.0%)
Somewhat approve	1,116 (16.0%)	454 (17.0%)
Neither approve nor disapprove	3,586 (51.3%)	1,388 (51.9%)
Somewhat disapprove	277 (4.0%)	167 (6.2%)
Strongly disapprove	166 (2.4%)	58 (2.2%)
(Missing)	47 (0.7%)	18 (0.7%)
Say in government, n (%)		
Agree	5,540 (79.2%)	2,146 (80.3%)
Disagree	369 (5.3%)	113 (4.2%)
Unsure	1,032 (14.8%)	386 (14.4%)
(Missing)	51 (0.7%)	29 (1.1%)
Belonging in country		
Mean	8.8	8.8
Standard Deviation	1.8	1.8
Min, Max	0.0, 10.0	0.0, 10.0
(Missing)	43 (0.6%)	26 (1.0%)
City/place satisfaction, n (%)		
Satisfied	6,328 (90.5%)	2,299 (86.0%)
Dissatisfied	291 (4.2%)	204 (7.6%)
Unsure	368 (5.3%)	163 (6.1%)
(Missing)	5 (0.1%)	9 (0.3%)
Trust within country, n (%)		
All people	696 (10.0%)	254 (9.5%)
Most people	2,743 (39.2%)	1,071 (40.0%)
Some people	1,933 (27.7%)	714 (26.7%)
Not very many people	1,234 (17.6%)	480 (17.9%)
None	293 (4.2%)	131 (4.9%)
(Missing)	92 (1.3%)	24 (0.9%)
Number of children		
Mean	1.3	1.5
Standard Deviation	1.4	2.4
Min, Max	0.0, 97.0	0.0, 96.0
(Missing)	0 (<0.1%)	260 (9.7%)
Community participation, n (%)		
More than once a week	886 (12.7%)	356 (13.3%)
Once a week	1,379 (19.7%)	536 (20.1%)
One to three times a month	986 (14.1%)	408 (15.3%)
A few times a year	1,219 (17.4%)	550 (20.6%)
Never	2,499 (35.7%)	812 (30.4%)
(Missing)	23 (0.3%)	11 (0.4%)
Religious attendance, n (%)		

Table S17b. Weighted summary statistics for outcome variables in Indonesia

Outcome	Wave 1<br>N = 6,992	Wave 2<br>N = 2,674
More than once a week	2,671 (38.2%)	928 (34.7%)
Once a week	2,540 (36.3%)	958 (35.8%)
One to three times a month	773 (11.1%)	352 (13.1%)
A few times a year	645 (9.2%)	276 (10.3%)
Never	345 (4.9%)	150 (5.6%)
(Missing)	18 (0.3%)	10 (0.4%)
Loneliness		
Mean	2.5	2.8
Standard Deviation	3.0	3.0
Min, Max	0.0, 10.0	0.0, 10.0
(Missing)	15 (0.2%)	7 (0.3%)
Perceived discrimination, n (%)		
Always	611 (8.7%)	191 (7.1%)
Often	857 (12.3%)	366 (13.7%)
Rarely	2,037 (29.1%)	855 (32.0%)
Never	3,451 (49.4%)	1,257 (47.0%)
(Missing)	35 (0.5%)	4 (0.2%)
Orientation to promote good		
Mean	8.6	8.6
Standard Deviation	1.8	1.9
Min, Max	0.0, 10.0	0.0, 10.0
(Missing)	29 (0.4%)	13 (0.5%)
Delayed gratification		
Mean	8.4	8.2
Standard Deviation	2.0	2.0
Min, Max	0.0, 10.0	0.0, 10.0
(Missing)	41 (0.6%)	3 (0.1%)
Hope		
Mean	9.2	9.0
Standard Deviation	1.4	1.6
Min, Max	0.0, 10.0	0.0, 10.0
(Missing)	16 (0.2%)	4 (0.1%)
Gratitude		
Mean	8.9	8.8
Standard Deviation	1.8	1.8
Min, Max	0.0, 10.0	0.0, 10.0
(Missing)	43 (0.6%)	7 (0.3%)
Showing love/care		
Mean	8.9	8.7
Standard Deviation	1.8	1.9
Min, Max	0.0, 10.0	0.0, 10.0
(Missing)	11 (0.2%)	1 (<0.1%)
Forgivingness, n (%)		
Always	3,559 (50.9%)	1,356 (50.7%)
Often	2,344 (33.5%)	889 (33.2%)
Rarely	683 (9.8%)	261 (9.8%)
Never	399 (5.7%)	166 (6.2%)
(Missing)	7 (0.1%)	2 (0.1%)
Charitable giving, n (%)		
Yes	5,456 (78.0%)	2,385 (89.2%)
No	1,512 (21.6%)	268 (10.0%)

Table S17b. Weighted summary statistics for outcome variables in Indonesia

Outcome	Wave 1<br>N = 6,992	Wave 2<br>N = 2,674
(Missing)	24 (0.3%)	20 (0.8%)
Helping strangers, n (%)		
Yes	3,476 (49.7%)	1,731 (64.8%)
No	3,483 (49.8%)	924 (34.6%)
(Missing)	32 (0.5%)	18 (0.7%)
Volunteering, n (%)		
Yes	3,233 (46.2%)	1,777 (66.5%)
No	3,742 (53.5%)	875 (32.7%)
(Missing)	17 (0.2%)	22 (0.8%)
Self-rated physical health		
Mean	8.3	8.0
Standard Deviation	2.0	2.2
Min, Max	0.0, 10.0	0.0, 10.0
(Missing)	19 (0.3%)	4 (0.1%)
Health problems, n (%)		
Yes	1,053 (15.1%)	633 (23.7%)
No	5,875 (84.0%)	2,030 (75.9%)
(Missing)	64 (0.9%)	11 (0.4%)
Pain in past 4 weeks, n (%)		
A lot	707 (10.1%)	232 (8.7%)
Some	1,703 (24.4%)	562 (21.0%)
Not very much	2,866 (41.0%)	1,406 (52.6%)
None at all	1,695 (24.2%)	473 (17.7%)
(Missing)	20 (0.3%)	0 (<0.0%)
Number of cigarettes per day		
Mean	3.9	4.1
Standard Deviation	6.6	7.0
Min, Max	0.0, 97.0	0.0, 97.0
(Missing)	424 (6.1%)	98 (3.7%)
Number of drinks per week		
Mean	0.2	0.1
Standard Deviation	1.6	0.9
Min, Max	0.0, 97.0	0.0, 27.0
(Missing)	446 (6.4%)	109 (4.1%)
Days exercise per week		
Mean	3.0	2.9
Standard Deviation	2.8	2.7
Min, Max	0.0, 7.0	0.0, 7.0
(Missing)	80 (1.1%)	18 (0.7%)
Financial security		
Mean	6.1	6.0
Standard Deviation	3.4	3.3
Min, Max	0.0, 10.0	0.0, 10.0
(Missing)	15 (0.2%)	7 (0.2%)
Material security		
Mean	6.4	6.4
Standard Deviation	3.5	3.4
Min, Max	0.0, 10.0	0.0, 10.0
(Missing)	13 (0.2%)	1 (<0.1%)
Educational attainment (16+ years), n (%)		
Up to 8	3,509 (50.2%)	1,125 (42.1%)

Table S17b. Weighted summary statistics for outcome variables in Indonesia

Outcome	Wave 1<br>N = 6,992	Wave 2<br>N = 2,674
9-15	3,146 (45.0%)	1,351 (50.5%)
16+	334 (4.8%)	198 (7.4%)
(Missing)	3 (0.0%)	0 (0%)
Currently employed, n (%)		
Employed for an employer	1,308 (18.7%)	374 (14.0%)
Self-employed	2,157 (30.9%)	736 (27.5%)
Retired	67 (1.0%)	27 (1.0%)
Student	318 (4.5%)	55 (2.1%)
Homemaker	2,111 (30.2%)	816 (30.5%)
Unemployed and looking for a job	561 (8.0%)	260 (9.7%)
None of these/Other	450 (6.4%)	372 (13.9%)
(Missing)	20 (0.3%)	34 (1.3%)
Financially comfortable/getting by, n (%)		
Living comfortably on present income	2,266 (32.4%)	850 (31.8%)
Getting by on present income	3,194 (45.7%)	1,162 (43.5%)
Finding it difficult on present income	944 (13.5%)	461 (17.2%)
Finding it very difficult on present income	468 (6.7%)	169 (6.3%)
(Missing)	120 (1.7%)	32 (1.2%)
Own home, n (%)		
Someone in this household owns this home	4,269 (61.1%)	1,495 (55.9%)
Someone in this household rents this home	620 (8.9%)	279 (10.4%)
Both	281 (4.0%)	97 (3.6%)
Neither	1,710 (24.5%)	772 (28.9%)
Rent	0 (0%)	0 (0%)
Own	0 (0%)	0 (0%)
Something else	0 (0%)	0 (0%)
(Missing)	112 (1.6%)	30 (1.1%)
Religious/spiritual connection, n (%)		
Always	4,111 (58.8%)	1,514 (56.6%)
Often	1,954 (27.9%)	770 (28.8%)
Rarely	575 (8.2%)	225 (8.4%)
Never	323 (4.6%)	160 (6.0%)
(Missing)	30 (0.4%)	6 (0.2%)
Belief in life after death, n (%)		
Yes	6,602 (94.4%)	2,534 (94.8%)
No	151 (2.2%)	31 (1.2%)
Unsure	211 (3.0%)	86 (3.2%)
(Missing)	29 (0.4%)	22 (0.8%)
Transformative religious experience, n (%)		
Yes	3,791 (54.2%)	1,533 (57.3%)
No	3,135 (44.8%)	1,124 (42.0%)
(Missing)	66 (0.9%)	17 (0.6%)
Religious reading or listening, n (%)		
More than once a day	2,304 (32.9%)	879 (32.9%)
About once a day	1,804 (25.8%)	669 (25.0%)
Sometimes	2,264 (32.4%)	864 (32.3%)
Never	592 (8.5%)	236 (8.8%)
(Missing)	29 (0.4%)	25 (0.9%)
Prayer or meditation, n (%)		
More than once a day	4,616 (66.0%)	1,808 (67.6%)
About once a day	1,241 (17.7%)	437 (16.3%)

Table S17b. Weighted summary statistics for outcome variables in Indonesia

**Outcome**	**Wave 1**<br>N = 6,992	**Wave 2**<br>N = 2,674
Sometimes	924 (13.2%)	347 (13.0%)
Never	186 (2.7%)	64 (2.4%)
(Missing)	24 (0.3%)	18 (0.7%)
Belief in God/gods/spiritual forces, n (%)		
One God	6,611 (94.6%)	2,522 (94.3%)
More than one god	75 (1.1%)	24 (0.9%)
An impersonal spiritual force	70 (1.0%)	43 (1.6%)
None of these	68 (1.0%)	29 (1.1%)
Unsure	145 (2.1%)	48 (1.8%)
(Missing)	24 (0.3%)	8 (0.3%)
Religious centrality, n (%)		
Agree	6,511 (93.1%)	2,511 (93.9%)
Disagree	125 (1.8%)	47 (1.7%)
Not relevant	90 (1.3%)	44 (1.7%)
Unsure	226 (3.2%)	61 (2.3%)
(Missing)	39 (0.6%)	11 (0.4%)
Religious/spiritual comfort, n (%)		
Agree	6,691 (95.7%)	2,555 (95.6%)
Disagree	85 (1.2%)	29 (1.1%)
Not relevant	64 (0.9%)	40 (1.5%)
Unsure	132 (1.9%)	41 (1.5%)
(Missing)	20 (0.3%)	8 (0.3%)
Feel loved by God, n (%)		
Agree	6,318 (90.4%)	2,458 (91.9%)
Disagree	205 (2.9%)	57 (2.1%)
Not relevant	166 (2.4%)	70 (2.6%)
Unsure	269 (3.9%)	80 (3.0%)
(Missing)	34 (0.5%)	9 (0.3%)
Feel punished by God, n (%)		
Agree	1,965 (28.1%)	919 (34.4%)
Disagree	3,071 (43.9%)	981 (36.7%)
Not relevant	751 (10.7%)	278 (10.4%)
Unsure	1,161 (16.6%)	482 (18.0%)
(Missing)	44 (0.6%)	14 (0.5%)
Experienced religious criticism, n (%)		
Agree	1,282 (18.3%)	542 (20.3%)
Disagree	3,466 (49.6%)	1,198 (44.8%)
Not relevant	1,161 (16.6%)	447 (16.7%)
Unsure	1,050 (15.0%)	470 (17.6%)
(Missing)	33 (0.5%)	17 (0.7%)
Faith-sharing, n (%)		
Agree	3,801 (54.4%)	1,496 (55.9%)
Disagree	1,579 (22.6%)	541 (20.2%)
Not relevant	759 (10.9%)	327 (12.2%)
Unsure	807 (11.5%)	297 (11.1%)
(Missing)	46 (0.7%)	13 (0.5%)

Note. N (%); this table is based on non-imputed data. Cumulative percentages for variables may not add up to 100% due to rounding. Wave 1 characteristics weighted using the Gallup provided sampling weight, ANNUAL_WEIGHT_R2; Wave 2 characteristics weighted accounting for attrition by using the adjusted Wave 1 weight, ANNUAL_WEIGHT_R2, multiplied by the created attrition weight to account for dropout, to maintain nationally representative estimates for Wave 2 characteristics.

Table S17c. Unweighted summary statistics for demographic and childhood variables in Indonesia by retention status

Characteristic	Attritors–Not Observed in Wave 2<br>N = 4,344	Retained–Observed in Wave 2<br>N = 2,587
Forgivingness, n (%)		
Always	2,249 (51.8%)	1,277 (49.3%)
Often	1,409 (32.4%)	917 (35.5%)
Rarely	432 (10.0%)	244 (9.4%)
Never	248 (5.7%)	147 (5.7%)
(Missing)	5 (0.1%)	2 (0.1%)
Year of birth, n (%)		
1943 or earlier (current age: 80+ years)	12 (0.3%)	8 (0.3%)
1943-1953 (current age: 70-79 years)	53 (1.2%)	32 (1.2%)
1953-1963 (current age: 60-69 years)	353 (8.1%)	160 (6.2%)
1963-1973 (current age: 50-59 years)	789 (18.2%)	368 (14.2%)
1973-1983 (current age: 40-49 years)	927 (21.3%)	530 (20.5%)
1983-1993 (current age: 30-39 years)	912 (21.0%)	604 (23.3%)
1993-1998 (current age: 25-29 years)	467 (10.8%)	323 (12.5%)
1998-2005 (current age: 18-24 years)	832 (19.1%)	563 (21.8%)
(Missing)	0 (0%)	0 (0%)
Age of participant		
Mean	39.6	37.7
Standard Deviation	14.3	13.9
Min, Max	18.0, 99.0	18.0, 92.0
Gender, n (%)		
Male	2,108 (48.5%)	1,363 (52.7%)
Female	2,225 (51.2%)	1,217 (47.0%)
Other	5 (0.1%)	2 (0.1%)
(Missing)	6 (0.1%)	5 (0.2%)
Respondent marital status, n (%)		
Single/Never been married	877 (20.2%)	613 (23.7%)
Married	2,976 (68.5%)	1,694 (65.5%)
Separated	55 (1.3%)	28 (1.1%)
Divorced	118 (2.7%)	73 (2.8%)
Widowed	275 (6.3%)	163 (6.3%)
Domestic partner	10 (0.2%)	8 (0.3%)
(Missing)	33 (0.8%)	8 (0.3%)
Education (years), n (%)		
Up to 8	2,314 (53.3%)	1,153 (44.6%)
9-15	1,853 (42.7%)	1,274 (49.2%)
16+	175 (4.0%)	159 (6.2%)
(Missing)	2 (0.0%)	1 (0.0%)
Employment status, n (%)		
Employed for an employer	792 (18.2%)	506 (19.6%)
Self-employed	1,320 (30.4%)	820 (31.7%)
Retired	44 (1.0%)	22 (0.9%)
Student	164 (3.8%)	153 (5.9%)
Homemaker	1,386 (31.9%)	701 (27.1%)
Unemployed and looking for a job	327 (7.5%)	231 (8.9%)
None of these/Other	296 (6.8%)	149 (5.8%)
(Missing)	15 (0.3%)	5 (0.2%)
Current religious service attendance, n (%)		
More than once a week	1,632 (37.6%)	1,017 (39.3%)

Table S17c. Unweighted summary statistics for demographic and childhood variables in Indonesia by retention status

Characteristic	Attritors–Not Observed in Wave 2<br>N = 4,344	Retained–Observed in Wave 2<br>N = 2,587
Once a week	1,593 (36.7%)	924 (35.7%)
One to three times a month	477 (11.0%)	290 (11.2%)
A few times a year	418 (9.6%)	220 (8.5%)
Never	215 (5.0%)	126 (4.9%)
(Missing)	8 (0.2%)	11 (0.4%)
Immigration status, n (%)		
Born in this country	4,325 (99.6%)	2,572 (99.4%)
Born in another country	18 (0.4%)	15 (0.6%)
(Missing)	0 (0%)	0 (0%)
Parental marital status around age 12, n (%)		
Parents were married	3,445 (79.3%)	2,008 (77.6%)
Parents were divorced	267 (6.1%)	196 (7.6%)
Parents were never married	35 (0.8%)	13 (0.5%)
One or both of them had died	442 (10.2%)	315 (12.2%)
Unsure	74 (1.7%)	33 (1.3%)
(Missing)	80 (1.8%)	22 (0.9%)
Religious service attendance around age 12, n (%)		
At least once a week	3,290 (75.7%)	2,027 (78.4%)
One to three times a month	645 (14.9%)	326 (12.6%)
Less than once a month	206 (4.8%)	111 (4.3%)
Never	171 (3.9%)	104 (4.0%)
(Missing)	32 (0.7%)	19 (0.7%)
Relationship with mother when growing up, n (%)		
Very good	3,880 (89.3%)	2,281 (88.2%)
Somewhat good	369 (8.5%)	223 (8.6%)
Somewhat bad	22 (0.5%)	31 (1.2%)
Very bad	18 (0.4%)	9 (0.4%)
(Does not apply)	35 (0.8%)	34 (1.3%)
(Missing)	19 (0.4%)	9 (0.3%)
Relationship with father when growing up, n (%)		
Very good	3,768 (86.8%)	2,239 (86.5%)
Somewhat good	408 (9.4%)	226 (8.7%)
Somewhat bad	29 (0.7%)	38 (1.5%)
Very bad	34 (0.8%)	17 (0.7%)
(Does not apply)	71 (1.6%)	40 (1.6%)
(Missing)	32 (0.7%)	27 (1.1%)
Felt like an outsider in family when growing up, n (%)		
Yes	217 (5.0%)	137 (5.3%)
No	4,117 (94.8%)	2,449 (94.7%)
(Missing)	9 (0.2%)	1 (0.1%)
Experienced abuse when growing up, n (%)		
Yes	311 (7.2%)	189 (7.3%)
No	3,977 (91.6%)	2,379 (92.0%)
(Missing)	55 (1.3%)	20 (0.8%)
Self-rated health when growing up, n (%)		
Excellent	804 (18.5%)	456 (17.6%)
Very good	1,220 (28.1%)	719 (27.8%)

Table S17c. Unweighted summary statistics for demographic and childhood variables in Indonesia by retention status

Characteristic	Attritors–Not Observed in Wave 2<br>N = 4,344	Retained–Observed in Wave 2<br>N = 2,587
Good	1,537 (35.4%)	894 (34.6%)
Fair	745 (17.1%)	498 (19.2%)
Poor	37 (0.9%)	20 (0.8%)
(Missing)	0 (<0.0%)	0 (<0.0%)
Subjective financial status of family growing up, n (%)		
Lived comfortably	2,132 (49.1%)	1,209 (46.7%)
Got by	1,790 (41.2%)	1,151 (44.5%)
Found it difficult	288 (6.6%)	161 (6.2%)
Found it very difficult	129 (3.0%)	62 (2.4%)
(Missing)	5 (0.1%)	4 (0.1%)
Religious affiliation growing up, n (%)		
Christianity	281 (6.5%)	233 (9.0%)
Taoism	0 (0%)	0 (<0.0%)
Confucianism	1 (0.0%)	0 (0%)
Primal, Animist, or Folk religion	0 (0%)	0 (0.0%)
Spiritism	0 (0%)	0 (0%)
Umbanda, Candomblé, and other African-derived religions	0 (0%)	0 (0%)
Chinese folk/traditional religion	0 (0%)	0 (0%)
Islam	4,008 (92.3%)	2,321 (89.7%)
Hinduism	47 (1.1%)	25 (1.0%)
Buddhism	0 (<0.0%)	5 (0.2%)
Judaism	0 (0%)	0 (0%)
Sikhism	0 (0%)	0 (0%)
Baha'i	0 (0%)	0 (0%)
Jainism	1 (0.0%)	0 (0%)
Shinto	0 (0%)	0 (0%)
Some other religion	0 (0%)	0 (0%)
No religion/Atheist/Agnostic	0 (0%)	2 (0.1%)
(Missing)	6 (0.1%)	1 (0.0%)

Note. N (%); this table is based on non-imputed data. Cumulative percentages for variables may not add up to 100% due to rounding.

Table S17d. Unweighted summary statistics for Wave 1 outcome variables in Indonesia by retention status.

**Outcome**	**Attritors–Not Observed in Wave 2** N = 4,344	**Retained–Observed in Wave 2** N = 2,587
Secure flourishing index		
Mean	8.1	8.1
Standard Deviation	1.3	1.4
Min, Max	1.6, 10.0	0.8, 10.0
(Missing)	87 (2.0%)	76 (2.9%)
Flourishing index		
Mean	8.5	8.5
Standard Deviation	1.3	1.4
Min, Max	0.7, 10.0	0.5, 10.0
(Missing)	82 (1.9%)	66 (2.6%)
Happiness & life satisfaction		
Mean	8.1	8.0
Standard Deviation	2.0	2.0
Min, Max	0.0, 10.0	0.0, 10.0
(Missing)	19 (0.4%)	12 (0.5%)
Physical & mental health		
Mean	8.5	8.4
Standard Deviation	1.7	1.8
Min, Max	0.0, 10.0	0.0, 10.0
(Missing)	18 (0.4%)	19 (0.7%)
Meaning & purpose		
Mean	8.7	8.7
Standard Deviation	1.5	1.5
Min, Max	0.0, 10.0	0.0, 10.0
(Missing)	29 (0.7%)	16 (0.6%)
Character & virtue		
Mean	8.5	8.5
Standard Deviation	1.7	1.7
Min, Max	0.0, 10.0	0.0, 10.0
(Missing)	28 (0.7%)	21 (0.8%)
Close social relationships		
Mean	8.7	8.7
Standard Deviation	1.7	1.7
Min, Max	0.0, 10.0	0.0, 10.0
(Missing)	27 (0.6%)	18 (0.7%)
Financial & material security		
Mean	6.4	6.0
Standard Deviation	3.0	3.2
Min, Max	0.0, 10.0	0.0, 10.0
(Missing)	13 (0.3%)	15 (0.6%)
Happiness		
Mean	8.1	8.0
Standard Deviation	2.2	2.3
Min, Max	0.0, 10.0	0.0, 10.0
(Missing)	11 (0.3%)	10 (0.4%)
Life satisfaction		
Mean	8.0	8.0
Standard Deviation	2.3	2.3
Min, Max	0.0, 10.0	0.0, 10.0

Table S17d. Unweighted summary statistics for Wave 1 outcome variables in Indonesia by retention status.

**Outcome**	**Attritors–Not Observed in Wave 2** N = 4,344	**Retained–Observed in Wave 2** N = 2,587
(Missing)	14 (0.3%)	5 (0.2%)
Current life evaluation		
Mean	7.1	6.7
Standard Deviation	2.5	2.6
Min, Max	0.0, 10.0	0.0, 10.0
(Missing)	18 (0.4%)	16 (0.6%)
Future life evaluation		
Mean	8.4	8.3
Standard Deviation	2.0	2.1
Min, Max	0.0, 10.0	0.0, 10.0
(Missing)	46 (1.1%)	36 (1.4%)
Optimism		
Mean	9.1	9.2
Standard Deviation	1.7	1.7
Min, Max	0.0, 10.0	0.0, 10.0
(Missing)	14 (0.3%)	9 (0.4%)
Freedom to pursue what's important		
Mean	8.8	8.8
Standard Deviation	1.8	1.9
Min, Max	0.0, 10.0	0.0, 10.0
(Missing)	14 (0.3%)	5 (0.2%)
Inner peace, n (%)		
Always	1,631 (37.6%)	825 (31.9%)
Often	1,451 (33.4%)	962 (37.2%)
Rarely	915 (21.1%)	544 (21.0%)
Never	326 (7.5%)	253 (9.8%)
(Missing)	20 (0.5%)	3 (0.1%)
Life balance, n (%)		
Always	1,388 (32.0%)	714 (27.6%)
Often	1,610 (37.1%)	999 (38.6%)
Rarely	1,051 (24.2%)	661 (25.6%)
Never	271 (6.2%)	201 (7.8%)
(Missing)	23 (0.5%)	12 (0.5%)
Sense of mastery, n (%)		
Always	1,645 (37.9%)	880 (34.0%)
Often	1,741 (40.1%)	1,054 (40.7%)
Rarely	812 (18.7%)	505 (19.5%)
Never	129 (3.0%)	133 (5.1%)
(Missing)	16 (0.4%)	16 (0.6%)
Meaningful activities		
Mean	8.5	8.5
Standard Deviation	1.9	1.9
Min, Max	0.0, 10.0	0.0, 10.0
(Missing)	15 (0.3%)	10 (0.4%)
Understanding purpose		
Mean	8.9	8.9
Standard Deviation	1.7	1.7
Min, Max	0.0, 10.0	0.0, 10.0
(Missing)	16 (0.4%)	9 (0.3%)

Table S17d. Unweighted summary statistics for Wave 1 outcome variables in Indonesia by retention status.

**Outcome**	**Attritors–Not Observed in Wave 2** N = 4,344	**Retained–Observed in Wave 2** N = 2,587
Self-rated mental health		
Mean	8.6	8.5
Standard Deviation	1.9	2.0
Min, Max	0.0, 10.0	0.0, 10.0
(Missing)	11 (0.3%)	18 (0.7%)
Traumatic distress, n (%)		
A lot	272 (6.3%)	152 (5.9%)
Some	714 (16.4%)	419 (16.2%)
Not very much	1,601 (36.9%)	1,006 (38.9%)
Not at all	1,740 (40.1%)	1,004 (38.8%)
(Missing)	17 (0.4%)	6 (0.2%)
Depression symptoms composite, n (%)	853 (19.8%)	510 (19.8%)
(Missing)	38 (0.9%)	17 (0.6%)
Depression – feel hopeless, n (%)		
Nearly every day	244 (5.6%)	151 (5.8%)
More than half the days	228 (5.3%)	166 (6.4%)
Several days	1,134 (26.1%)	697 (26.9%)
Not at all	2,721 (62.6%)	1,571 (60.7%)
(Missing)	16 (0.4%)	2 (0.1%)
Depression – loss of interest, n (%)		
Nearly every day	479 (11.0%)	267 (10.3%)
More than half the days	346 (8.0%)	194 (7.5%)
Several days	1,430 (32.9%)	973 (37.6%)
Not at all	2,060 (47.4%)	1,137 (44.0%)
(Missing)	28 (0.7%)	15 (0.6%)
Anxiety symptoms composite, n (%)	645 (14.9%)	350 (13.6%)
(Missing)	21 (0.5%)	7 (0.3%)
Anxiety – feel on edge, n (%)		
Nearly every day	328 (7.6%)	167 (6.5%)
More than half the days	256 (5.9%)	156 (6.0%)
Several days	1,234 (28.4%)	785 (30.3%)
Not at all	2,518 (58.0%)	1,476 (57.0%)
(Missing)	7 (0.2%)	3 (0.1%)
Anxiety – cannot stop worrying, n (%)		
Nearly every day	273 (6.3%)	155 (6.0%)
More than half the days	229 (5.3%)	115 (4.5%)
Several days	900 (20.7%)	636 (24.6%)
Not at all	2,926 (67.4%)	1,675 (64.7%)
(Missing)	16 (0.4%)	6 (0.2%)
Suffering, n (%)		
A lot	371 (8.5%)	211 (8.2%)
Some	758 (17.4%)	448 (17.3%)
Not very much	1,636 (37.7%)	1,024 (39.6%)
Not at all	1,550 (35.7%)	886 (34.2%)
(Missing)	29 (0.7%)	17 (0.7%)
Relationship contentment		
Mean	8.8	8.8
Standard Deviation	1.8	1.8
Min, Max	0.0, 10.0	0.0, 10.0

Table S17d. Unweighted summary statistics for Wave 1 outcome variables in Indonesia by retention status.

**Outcome**	**Attritors–Not Observed in Wave 2** N = 4,344	**Retained–Observed in Wave 2** N = 2,587
(Missing)	18 (0.4%)	10 (0.4%)
Relationship satisfaction		
Mean	8.6	8.6
Standard Deviation	1.9	2.0
Min, Max	0.0, 10.0	0.0, 10.0
(Missing)	15 (0.3%)	13 (0.5%)
Social support		
Mean	7.5	7.5
Standard Deviation	2.8	2.9
Min, Max	0.0, 10.0	0.0, 10.0
(Missing)	5 (0.1%)	4 (0.2%)
Intimate/close friend, n (%)		
Yes	3,342 (76.9%)	1,990 (76.9%)
No	999 (23.0%)	588 (22.7%)
(Missing)	3 (0.1%)	9 (0.3%)
Government approval, n (%)		
Strongly approve	1,144 (26.3%)	638 (24.7%)
Somewhat approve	697 (16.1%)	409 (15.8%)
Neither approve nor disapprove	2,212 (50.9%)	1,344 (51.9%)
Somewhat disapprove	154 (3.5%)	121 (4.7%)
Strongly disapprove	108 (2.5%)	55 (2.1%)
(Missing)	27 (0.6%)	20 (0.8%)
Say in government, n (%)		
Agree	3,416 (78.6%)	2,078 (80.3%)
Disagree	239 (5.5%)	125 (4.8%)
Unsure	659 (15.2%)	363 (14.0%)
(Missing)	30 (0.7%)	21 (0.8%)
Belonging in country		
Mean	8.8	8.8
Standard Deviation	1.8	1.8
Min, Max	0.0, 10.0	0.0, 10.0
(Missing)	28 (0.6%)	15 (0.6%)
City/place satisfaction, n (%)		
Satisfied	3,980 (91.6%)	2,288 (88.4%)
Dissatisfied	160 (3.7%)	130 (5.0%)
Unsure	201 (4.6%)	165 (6.4%)
(Missing)	2 (0.0%)	4 (0.1%)
Trust within country, n (%)		
All people	440 (10.1%)	250 (9.7%)
Most people	1,699 (39.1%)	1,020 (39.4%)
Some people	1,201 (27.7%)	715 (27.7%)
Not very many people	763 (17.6%)	461 (17.8%)
None	187 (4.3%)	102 (4.0%)
(Missing)	54 (1.2%)	38 (1.5%)
Number of children		
Mean	1.3	1.4
Standard Deviation	1.4	1.6
Min, Max	0.0, 96.0	0.0, 97.0
Community participation, n (%)		

Table S17d. Unweighted summary statistics for Wave 1 outcome variables in Indonesia by retention status.

**Outcome**	**Attritors–Not Observed in Wave 2** N = 4,344	**Retained–Observed in Wave 2** N = 2,587
More than once a week	545 (12.6%)	333 (12.9%)
Once a week	881 (20.3%)	484 (18.7%)
One to three times a month	585 (13.5%)	394 (15.2%)
A few times a year	716 (16.5%)	496 (19.2%)
Never	1,606 (37.0%)	867 (33.5%)
(Missing)	10 (0.2%)	13 (0.5%)
Religious attendance, n (%)		
More than once a week	1,632 (37.6%)	1,017 (39.3%)
Once a week	1,593 (36.7%)	924 (35.7%)
One to three times a month	477 (11.0%)	290 (11.2%)
A few times a year	418 (9.6%)	220 (8.5%)
Never	215 (5.0%)	126 (4.9%)
(Missing)	8 (0.2%)	11 (0.4%)
Loneliness		
Mean	2.4	2.6
Standard Deviation	2.9	3.1
Min, Max	0.0, 10.0	0.0, 10.0
(Missing)	9 (0.2%)	6 (0.2%)
Perceived discrimination, n (%)		
Always	408 (9.4%)	196 (7.6%)
Often	506 (11.7%)	345 (13.3%)
Rarely	1,245 (28.7%)	776 (30.0%)
Never	2,160 (49.7%)	1,260 (48.7%)
(Missing)	25 (0.6%)	10 (0.4%)
Orientation to promote good		
Mean	8.6	8.7
Standard Deviation	1.8	1.8
Min, Max	0.0, 10.0	0.0, 10.0
(Missing)	13 (0.3%)	16 (0.6%)
Delayed gratification		
Mean	8.4	8.4
Standard Deviation	2.0	2.1
Min, Max	0.0, 10.0	0.0, 10.0
(Missing)	27 (0.6%)	14 (0.5%)
Hope		
Mean	9.1	9.3
Standard Deviation	1.5	1.4
Min, Max	0.0, 10.0	0.0, 10.0
(Missing)	5 (0.1%)	11 (0.4%)
Gratitude		
Mean	8.9	9.0
Standard Deviation	1.8	1.7
Min, Max	0.0, 10.0	0.0, 10.0
(Missing)	16 (0.4%)	28 (1.1%)
Showing love/care		
Mean	8.8	8.9
Standard Deviation	1.8	1.8
Min, Max	0.0, 10.0	0.0, 10.0
(Missing)	9 (0.2%)	2 (<0.1%)

Table S17d. Unweighted summary statistics for Wave 1 outcome variables in Indonesia by retention status.

**Outcome**	**Attritors–Not Observed in Wave 2** N = 4,344	**Retained–Observed in Wave 2** N = 2,587
Forgivingness, n (%)		
Always	2,249 (51.8%)	1,277 (49.3%)
Often	1,409 (32.4%)	917 (35.5%)
Rarely	432 (10.0%)	244 (9.4%)
Never	248 (5.7%)	147 (5.7%)
(Missing)	5 (0.1%)	2 (0.1%)
Charitable giving, n (%)		
Yes	3,356 (77.3%)	2,055 (79.4%)
No	976 (22.5%)	519 (20.1%)
(Missing)	12 (0.3%)	13 (0.5%)
Helping strangers, n (%)		
Yes	2,152 (49.5%)	1,294 (50.0%)
No	2,177 (50.1%)	1,275 (49.3%)
(Missing)	15 (0.3%)	17 (0.7%)
Volunteering, n (%)		
Yes	1,971 (45.4%)	1,236 (47.8%)
No	2,365 (54.5%)	1,341 (51.8%)
(Missing)	7 (0.2%)	10 (0.4%)
Self-rated physical health		
Mean	8.3	8.3
Standard Deviation	2.0	2.0
Min, Max	0.0, 10.0	0.0, 10.0
(Missing)	11 (0.3%)	8 (0.3%)
Health problems, n (%)		
Yes	638 (14.7%)	407 (15.7%)
No	3,661 (84.3%)	2,162 (83.6%)
(Missing)	45 (1.0%)	18 (0.7%)
Pain in past 4 weeks, n (%)		
A lot	453 (10.4%)	247 (9.5%)
Some	1,058 (24.4%)	631 (24.4%)
Not very much	1,746 (40.2%)	1,098 (42.4%)
None at all	1,070 (24.6%)	609 (23.5%)
(Missing)	16 (0.4%)	3 (0.1%)
Number of cigarettes per day		
Mean	3.9	4.1
Standard Deviation	6.5	6.8
Min, Max	0.0, 97.0	0.0, 60.0
(Missing)	248 (5.7%)	174 (6.7%)
Number of drinks per week		
Mean	0.2	0.1
Standard Deviation	1.8	1.1
Min, Max	0.0, 97.0	0.0, 35.0
(Missing)	270 (6.2%)	173 (6.7%)
Days exercise per week		
Mean	2.9	3.0
Standard Deviation	2.8	2.8
Min, Max	0.0, 7.0	0.0, 7.0
(Missing)	47 (1.1%)	32 (1.2%)
Financial security		

Table S17d. Unweighted summary statistics for Wave 1 outcome variables in Indonesia by retention status.

Outcome	Attritors–Not Observed in Wave 2<br>N = 4,344	Retained–Observed in Wave 2<br>N = 2,587
Mean	6.2	5.8
Standard Deviation	3.4	3.5
Min, Max	0.0, 10.0	0.0, 10.0
(Missing)	6 (0.1%)	9 (0.4%)
Material security		
Mean	6.6	6.2
Standard Deviation	3.4	3.6
Min, Max	0.0, 10.0	0.0, 10.0
(Missing)	7 (0.2%)	7 (0.3%)
Educational attainment (16+ years), n (%)		
Up to 8	2,314 (53.3%)	1,153 (44.6%)
9-15	1,853 (42.7%)	1,274 (49.2%)
16+	175 (4.0%)	159 (6.2%)
(Missing)	2 (0.0%)	1 (0.0%)
Currently employed, n (%)		
Employed for an employer	792 (18.2%)	506 (19.6%)
Self-employed	1,320 (30.4%)	820 (31.7%)
Retired	44 (1.0%)	22 (0.9%)
Student	164 (3.8%)	153 (5.9%)
Homemaker	1,386 (31.9%)	701 (27.1%)
Unemployed and looking for a job	327 (7.5%)	231 (8.9%)
None of these/Other	296 (6.8%)	149 (5.8%)
(Missing)	15 (0.3%)	5 (0.2%)
Financially comfortable/getting by, n (%)		
Living comfortably on present income	1,470 (33.8%)	772 (29.8%)
Getting by on present income	1,932 (44.5%)	1,237 (47.8%)
Finding it difficult on present income	561 (12.9%)	377 (14.6%)
Finding it very difficult on present income	291 (6.7%)	173 (6.7%)
(Missing)	90 (2.1%)	28 (1.1%)
Own home, n (%)		
Someone in this household owns this home	2,681 (61.7%)	1,548 (59.8%)
Someone in this household rents this home	391 (9.0%)	222 (8.6%)
Both	161 (3.7%)	119 (4.6%)
Neither	1,024 (23.6%)	674 (26.1%)
Rent	0 (0%)	0 (0%)
Own	0 (0%)	0 (0%)
Something else	0 (0%)	0 (0%)
(Missing)	86 (2.0%)	24 (0.9%)
Religious/spiritual connection, n (%)		
Always	2,558 (58.9%)	1,516 (58.6%)
Often	1,229 (28.3%)	707 (27.3%)
Rarely	343 (7.9%)	228 (8.8%)
Never	192 (4.4%)	128 (5.0%)
(Missing)	22 (0.5%)	8 (0.3%)
Belief in life after death, n (%)		
Yes	4,100 (94.4%)	2,444 (94.5%)
No	97 (2.2%)	52 (2.0%)
Unsure	129 (3.0%)	80 (3.1%)
(Missing)	17 (0.4%)	12 (0.4%)

Table S17d. Unweighted summary statistics for Wave 1 outcome variables in Indonesia by retention status.

**Outcome**	**Attritors–Not Observed in Wave 2** N = 4,344	**Retained–Observed in Wave 2** N = 2,587
Transformative religious experience, n (%)		
Yes	2,330 (53.6%)	1,429 (55.2%)
No	1,970 (45.4%)	1,135 (43.9%)
(Missing)	43 (1.0%)	23 (0.9%)
Religious reading or listening, n (%)		
More than once a day	1,372 (31.6%)	916 (35.4%)
About once a day	1,157 (26.6%)	628 (24.3%)
Sometimes	1,446 (33.3%)	795 (30.7%)
Never	355 (8.2%)	232 (9.0%)
(Missing)	14 (0.3%)	16 (0.6%)
Prayer or meditation, n (%)		
More than once a day	2,846 (65.5%)	1,732 (66.9%)
About once a day	771 (17.8%)	459 (17.7%)
Sometimes	583 (13.4%)	332 (12.9%)
Never	126 (2.9%)	57 (2.2%)
(Missing)	17 (0.4%)	7 (0.3%)
Belief in God/gods/spiritual forces, n (%)		
One God	4,098 (94.3%)	2,456 (94.9%)
More than one god	55 (1.3%)	18 (0.7%)
An impersonal spiritual force	52 (1.2%)	17 (0.6%)
None of these	37 (0.9%)	30 (1.2%)
Unsure	87 (2.0%)	57 (2.2%)
(Missing)	15 (0.3%)	9 (0.3%)
Religious centrality, n (%)		
Agree	4,026 (92.7%)	2,430 (93.9%)
Disagree	77 (1.8%)	47 (1.8%)
Not relevant	62 (1.4%)	27 (1.1%)
Unsure	148 (3.4%)	75 (2.9%)
(Missing)	31 (0.7%)	8 (0.3%)
Religious/spiritual comfort, n (%)		
Agree	4,145 (95.4%)	2,488 (96.2%)
Disagree	46 (1.1%)	39 (1.5%)
Not relevant	46 (1.1%)	17 (0.6%)
Unsure	87 (2.0%)	43 (1.7%)
(Missing)	18 (0.4%)	1 (0.0%)
Feel loved by God, n (%)		
Agree	3,933 (90.5%)	2,329 (90.0%)
Disagree	131 (3.0%)	72 (2.8%)
Not relevant	107 (2.5%)	57 (2.2%)
Unsure	153 (3.5%)	115 (4.4%)
(Missing)	20 (0.4%)	14 (0.5%)
Feel punished by God, n (%)		
Agree	1,223 (28.2%)	725 (28.0%)
Disagree	1,890 (43.5%)	1,155 (44.7%)
Not relevant	497 (11.4%)	245 (9.5%)
Unsure	708 (16.3%)	444 (17.1%)
(Missing)	26 (0.6%)	18 (0.7%)
Experienced religious criticism, n (%)		
Agree	807 (18.6%)	462 (17.9%)

Table S17d. Unweighted summary statistics for Wave 1 outcome variables in Indonesia by retention status.

**Outcome**	**Attritors–Not Observed in Wave 2** N = 4,344	**Retained–Observed in Wave 2** N = 2,587
Disagree	2,140 (49.3%)	1,296 (50.1%)
Not relevant	712 (16.4%)	440 (17.0%)
Unsure	659 (15.2%)	381 (14.7%)
(Missing)	25 (0.6%)	8 (0.3%)
Faith-sharing, n (%)		
Agree	2,374 (54.7%)	1,392 (53.8%)
Disagree	944 (21.7%)	624 (24.1%)
Not relevant	478 (11.0%)	274 (10.6%)
Unsure	511 (11.8%)	287 (11.1%)
(Missing)	36 (0.8%)	9 (0.3%)

Note. N (%); this table is based on non-imputed data. Cumulative percentages for variables may not add up to 100% due to rounding.

Table S17e. Summary of fitted attrition model in Indonesia

Characteristic	Odds Ratio	95% CI	p-value
**ANNUAL_WEIGHT_R2**	0.84	0.74, 0.95	0.004
**Recruitment Survey Mode**			
CAPI	—	—	
CATI	1.13	0.89, 1.42	0.317
**Happiness & life satisfaction**	0.96	0.87, 1.06	0.466
**Physical & mental health**	0.96	0.88, 1.05	0.352
**Meaning & purpose**	1.05	0.94, 1.18	0.374
**Character & virtue**	1.03	0.95, 1.12	0.486
**Close social relationships**	1.01	0.92, 1.11	0.779
**Financial & material security**	0.90	0.84, 0.97	0.005
**Extraversion**	1.05	0.98, 1.13	0.186
**Openness to experience**	1.09	1.01, 1.17	0.019
**Agreeableness**	1.02	0.94, 1.10	0.647
**Conscientiousness**	0.98	0.90, 1.07	0.683
**Neuroticism**	1.02	0.95, 1.09	0.646
**Depression symptoms composite**	0.99	0.92, 1.06	0.694
**Anxiety symptoms composite**	0.92	0.85, 0.99	0.023
**Loneliness**	1.03	0.96, 1.12	0.398
**Days exercise per week**	1.04	0.97, 1.12	0.296
**Year of birth (age group)**			
1983-1993 (current age: 30-39 years)	—	—	
1973-1983 (current age: 40-49 years)	0.87	0.72, 1.06	0.176
1998-2005 (current age: 18-24 years)	0.99	0.75, 1.31	0.967
1993-1998 (current age: 25-29 years)	1.01	0.80, 1.27	0.935
1963-1973 (current age: 50-59 years)	0.78	0.61, 1.01	0.060
1953-1963 (current age: 60-69 years)	0.80	0.55, 1.18	0.268
1943-1953 (current age: 70-79 years)	1.03	0.49, 2.16	0.934
1943 or earlier (current age: 80+ years)	0.90	0.12, 6.97	0.919
**Gender of respondent**			
Female	—	—	
Male	1.20	0.99, 1.46	0.059
Other	1.03	0.16, 6.67	0.978
**Marital status**			
Married	—	—	
Single/Never been married	0.88	0.70, 1.11	0.272
Widowed	1.20	0.86, 1.68	0.272
Divorced	1.01	0.67, 1.53	0.947
Separated	0.87	0.43, 1.75	0.701
Domestic partner	1.06	0.21, 5.46	0.944
**Employment status**			
Homemaker	—	—	
Self-employed	1.18	0.94, 1.47	0.154
Employed for an employer	1.17	0.91, 1.49	0.222
Unemployed and looking for a job	1.23	0.89, 1.70	0.200
None of these/Other	0.93	0.64, 1.36	0.720
Student	1.63	1.08, 2.46	0.020
Retired	0.97	0.47, 1.98	0.928
**Religious attendance**			
More than once a week	—	—	
Once a week	0.94	0.80, 1.10	0.434
One to three times a month	1.00	0.81, 1.24	0.999
A few times a year	0.85	0.67, 1.10	0.218

Table S17e. Summary of fitted attrition model in Indonesia

Characteristic	Odds Ratio	95% CI	p-value
Never	0.97	0.69, 1.36	0.840
**Educational attainment (16+ years)**			
9-15	—	—	
Up to 8	1.01	0.77, 1.33	0.921
16+	1.32	1.07, 1.64	0.011
**Born in This country**			
Born in this country	—	—	
Born in another country	1.32	0.58, 3.02	0.510
**Race plurality (prominent race/ethnic group [0] or not [1])**	1.02	0.95, 1.10	0.577
**Urbanicity**			
A rural area or on a farm	—	—	
A small town or village	1.04	0.87, 1.25	0.645
A large city	0.95	0.77, 1.18	0.654
A suburb of a large city	1.02	0.81, 1.30	0.846
**Monthly household income**			
Indonesia: 2,500,001 – 4,000,000 rupiah	—	—	
Indonesia: 1,000,001 – 1,500,000 rupiah	1.55	1.19, 2.01	9.98e-04
Indonesia: 1,500,001 – 2,000,000 rupiah	1.16	0.89, 1.50	0.282
Indonesia: 2,000,001 – 2,500,000 rupiah	1.35	1.02, 1.78	0.033
Indonesia: 600,001 – 1,000,000 rupiah	1.61	1.25, 2.07	2.20e-04
Indonesia: 300,001 – 600,000 rupiah	1.77	1.33, 2.36	9.00e-05
Indonesia: 4,000,001 - 8,000,000 rupiah	0.98	0.73, 1.32	0.915
(None/No household income)	1.43	1.06, 1.92	0.019
Indonesia: 150,000 rupiah or less	1.75	1.26, 2.44	9.19e-04
Indonesia: 150,001 – 300,000 rupiah	2.19	1.53, 3.14	2.03e-05
Indonesia: 8,000,001 - 14,000,000 rupiah	1.58	0.66, 3.79	0.308
Indonesia: More than 14,000,000 rupiah	0.51	0.28, 0.94	0.032

Abbreviations: CI = Confidence Interval, OR = Odds Ratio

Notes. N=6992; attrition weights were estimated using the ’survey::svyglm(family=quasibinomial(’logit’))’ function. All continuous predictors were standardized and all categorical predictors used the most common category as the reference group. Reported p-values are based on the fitted regression model and no adjustments for multiple testing were done within this table.

Table S17f. Summary of principal components in Indonesia

PC	Percent Explained by each PC	Cumulative Percent Explained
1	43.44	43.44
2	3.93	47.37
3	2.54	49.91
4	1.73	51.64
5	1.59	53.23
6	1.58	54.81
**7**	**1.46**	**56.27**
8	1.41	57.68
9	1.36	59.04
10	1.27	60.31
11	1.22	61.53
12	1.20	62.73
13	1.18	63.91
14	1.15	65.06
15	1.12	66.18
16	1.10	67.29
17	1.03	68.32
18	1.02	69.34
19	1.01	70.34
20	0.94	71.29

Notes. N=6992; PCA was conducted using 'survey::svyprcomp(.)' function using all available contemporaneous exposures at wave 1. All PCs were standardized prior to being used as predictors. The bolded row represented the number of retained components for analysis was 7.

Table S17g. Associations of forgivingness with adult well-being and other outcomes at Wave 2 in Indonesia

Outcome	Model 1: Demographic and Childhood Variables as Covariates					Model 2: Demographic, Childhood, and Other Wave 1 Confounding Variables (Via Principal Components) as Covariates				
	RR	ES	95% CI	SE	p-value	RR	ES	95% CI	SE	p-value
Human Flourishing										
Secure flourishing index		0.04	(0.00,0.07)	0.02	0.050		0.00	(-0.03,0.03)	0.02	0.866
Flourishing index		0.04	(-0.00,0.08)	0.02	0.067		0.00	(-0.03,0.04)	0.02	0.872
Happiness & life satisfaction		0.01	(-0.04,0.06)	0.02	0.729		-0.01	(-0.06,0.03)	0.02	0.629
Physical & mental health		0.02	(-0.03,0.06)	0.02	0.445		-0.00	(-0.05,0.04)	0.02	0.852
Meaning & purpose		0.04	(-0.01,0.09)	0.02	0.103		0.01	(-0.03,0.06)	0.02	0.603
Character & virtue		0.03	(-0.01,0.08)	0.02	0.173		0.01	(-0.04,0.05)	0.02	0.823
Close social relationships		0.03	(-0.01,0.08)	0.02	0.149		0.01	(-0.03,0.05)	0.02	0.607
Financial & material security		0.02	(-0.02,0.06)	0.02	0.361		0.00	(-0.04,0.04)	0.02	0.960
Psychological Well-Being										
Happiness (a)		-0.00	(-0.05,0.04)	0.02	0.877		-0.02	(-0.07,0.03)	0.02	0.402
Life satisfaction (a)		0.02	(-0.03,0.07)	0.03	0.500		0.00	(-0.05,0.05)	0.02	0.963
Current life evaluation		-0.01	(-0.05,0.04)	0.02	0.762		-0.03	(-0.07,0.02)	0.02	0.254
Future life evaluation		0.02	(-0.03,0.07)	0.03	0.364		0.00	(-0.04,0.05)	0.02	0.880
Optimism		0.03	(-0.01,0.08)	0.03	0.171		0.01	(-0.03,0.06)	0.02	0.577
Freedom to pursue what's important		-0.02	(-0.07,0.02)	0.02	0.323		-0.04	(-0.09,0.00)	0.02	0.054
Inner peace	1.02		(1.00,1.04)	0.01	0.064	1.01		(0.99,1.03)	0.01	0.329
Life balance	1.02		(0.99,1.04)	0.01	0.151	1.00		(0.98,1.02)	0.01	0.844
Sense of mastery	1.03		(1.00,1.05)	0.01	0.023*	1.02		(0.99,1.04)	0.01	0.158
Meaningful activities (c)		0.01	(-0.04,0.06)	0.03	0.697		-0.01	(-0.06,0.04)	0.03	0.757
Understanding purpose (c)		0.05	(0.00,0.10)	0.03	0.040*		0.03	(-0.02,0.08)	0.03	0.277
Self-rated mental health (b)		0.03	(-0.02,0.08)	0.02	0.195		0.01	(-0.03,0.06)	0.02	0.588
Psychological Distress										
Traumatic distress	1.01		(0.99,1.03)	0.01	0.527	1.00		(0.98,1.02)	0.01	0.830
Depression symptoms composite	1.00		(0.98,1.01)	0.01	0.966	1.00		(0.99,1.02)	0.01	0.912
Depression – feel hopeless	1.00		(0.99,1.01)	0.01	0.967	1.00		(0.99,1.01)	0.01	0.804
Depression – loss of interest	1.00		(0.98,1.01)	0.01	0.762	1.00		(0.98,1.01)	0.01	0.786
Anxiety symptoms composite	1.00		(0.98,1.02)	0.01	0.999	1.00		(0.99,1.02)	0.01	0.731
Anxiety – feel on edge	0.99		(0.98,1.01)	0.01	0.400	1.00		(0.98,1.01)	0.01	0.680
Anxiety – cannot stop worrying	1.00		(0.99,1.02)	0.01	0.890	1.00		(0.99,1.02)	0.01	0.772
Suffering	1.00		(0.98,1.02)	0.01	0.908	1.00		(0.98,1.02)	0.01	0.888
Social Well-Being										
Relationship contentment (e)		0.04	(-0.01,0.09)	0.02	0.084		0.02	(-0.02,0.07)	0.02	0.295
Relationship satisfaction (e)		0.01	(-0.04,0.06)	0.03	0.581		-0.00	(-0.05,0.04)	0.02	0.844
Social support		0.03	(-0.02,0.08)	0.02	0.245		0.02	(-0.03,0.07)	0.02	0.420
Intimate/close friend	1.01		(0.98,1.03)	0.01	0.586	1.00		(0.98,1.03)	0.01	0.747
Government approval	1.01		(0.98,1.03)	0.01	0.564	1.00		(0.98,1.02)	0.01	0.921
Say in government	1.01		(0.99,1.03)	0.01	0.306	1.00		(0.98,1.02)	0.01	0.825
Belonging in country		0.02	(-0.02,0.07)	0.02	0.297		-0.00	(-0.05,0.04)	0.02	0.887
City/place satisfaction	1.01		(0.99,1.02)	0.01	0.327	1.00		(0.99,1.02)	0.01	0.614
Trust within country	1.03		(1.01,1.06)	0.01	1.58e-03**	1.02		(1.00,1.04)	0.01	0.042*
Social Participation										
Ever been married	1.00		(0.99,1.01)	0.00	0.976	1.00		(0.99,1.01)	0.00	0.971
Currently divorced	1.00		(0.99,1.01)	0.00	0.583	1.00		(0.99,1.01)	0.00	0.613
Number of children		-0.01	(-0.07,0.04)	0.03	0.642		-0.01	(-0.07,0.04)	0.03	0.705
Weekly+ community participation	1.00		(0.98,1.02)	0.01	0.732	0.99		(0.97,1.01)	0.01	0.461
Weekly+ religious attendance	1.01		(0.99,1.03)	0.01	0.253	1.01		(0.99,1.03)	0.01	0.270
Social Distress										
Loneliness		-0.03	(-0.08,0.02)	0.02	0.251		-0.01	(-0.06,0.04)	0.03	0.617
Perceived discrimination	1.00		(0.98,1.02)	0.01	0.879	1.00		(0.98,1.01)	0.01	0.740
Character & Prosocial Behavior										
Orientation to promote good (d)		0.04	(-0.01,0.08)	0.02	0.154		0.01	(-0.03,0.06)	0.02	0.587
Delayed gratification (d)		0.02	(-0.03,0.06)	0.02	0.439		-0.00	(-0.05,0.04)	0.02	0.857
Hope		0.08	(0.02,0.13)	0.03	0.013*		0.06	(0.00,0.12)	0.03	0.050*
Gratitude		0.06	(-0.01,0.12)	0.03	0.085		0.04	(-0.03,0.10)	0.03	0.257
Showing love/care		0.07	(0.02,0.12)	0.02	0.005*		0.05	(0.00,0.10)	0.02	0.042*
Forgivingness	1.05		(1.03,1.07)	0.01	2e-05***	1.04		(1.02,1.07)	0.01	4.74e-05***
Charitable giving	1.00		(0.99,1.01)	0.01	0.953	1.00		(0.98,1.01)	0.01	0.659
Helping strangers	0.99		(0.97,1.01)	0.01	0.383	0.99		(0.97,1.01)	0.01	0.183
Volunteering	1.00		(0.97,1.03)	0.02	0.754	0.99		(0.96,1.02)	0.02	0.509
Physical Health & Health Behavior										
Self-rated physical health (b)		-0.00	(-0.05,0.04)	0.02	0.921		-0.02	(-0.07,0.03)	0.02	0.405
Health problems	1.00		(0.98,1.02)	0.01	0.815	1.01		(0.99,1.03)	0.01	0.537
Pain in past 4 weeks	1.01		(1.00,1.03)	0.01	0.139	1.01		(0.99,1.03)	0.01	0.165
Daily smoker	0.99		(0.97,1.01)	0.01	0.408	1.00		(0.98,1.02)	0.01	0.807
Number of drinks per week		-0.03	(-0.10,0.04)	0.04	0.392		-0.03	(-0.10,0.04)	0.04	0.445
Days exercise per week		-0.03	(-0.08,0.03)	0.03	0.324		-0.03	(-0.09,0.02)	0.03	0.202
Socioeconomic Outcomes										
Financial security (f)		0.02	(-0.03,0.06)	0.02	0.423		0.01	(-0.04,0.05)	0.02	0.815
Material security (f)		0.01	(-0.03,0.05)	0.02	0.547		-0.00	(-0.04,0.03)	0.02	0.864
Educational attainment (16+ years)	1.00		(0.99,1.00)	0.00	0.412	1.00		(0.99,1.00)	0.00	0.332
Currently employed	0.99		(0.97,1.02)	0.01	0.513	0.99		(0.97,1.02)	0.01	0.514
Financially comfortable/getting by	0.99		(0.98,1.01)	0.01	0.566	0.99		(0.97,1.01)	0.01	0.168
Own home	1.00		(0.98,1.03)	0.01	0.866	1.00		(0.97,1.02)	0.01	0.832
Income – top quintile	1.00		(0.99,1.02)	0.01	0.592	1.00		(0.99,1.02)	0.01	0.604
Religion & Spirituality										
Religious/spiritual connection	1.01		(1.00,1.03)	0.01	0.166	1.01		(0.99,1.02)	0.01	0.459
Belief in life after death	1.01		(1.00,1.02)	0.01	0.196	1.00		(0.99,1.02)	0.01	0.446
Transformative religious experience	1.02		(0.99,1.04)	0.01	0.167	1.01		(0.99,1.04)	0.01	0.305
Religious reading or listening	1.02		(0.99,1.04)	0.01	0.150	1.01		(0.98,1.03)	0.01	0.633
Prayer or meditation	1.02		(1.00,1.04)	0.01	0.035*	1.01		(0.99,1.03)	0.01	0.225
Belief in God/gods/spiritual forces	1.00		(0.99,1.01)	0.00	0.478	1.00		(0.99,1.01)	0.00	0.712
Religious centrality	1.02		(1.00,1.03)	0.01	0.027*	1.01		(1.00,1.03)	0.01	0.076
Religious/spiritual comfort	1.01		(1.00,1.02)	0.00	0.115	1.00		(0.99,1.01)	0.00	0.420
Feel loved by God	1.01		(0.99,1.02)	0.01	0.276	1.00		(0.99,1.02)	0.01	0.616
Feel punished by God	0.99		(0.97,1.01)	0.01	0.263	0.99		(0.97,1.01)	0.01	0.431
Experienced religious criticism	0.99		(0.97,1.01)	0.01	0.389	0.99		(0.97,1.02)	0.01	0.620
Faith-sharing	0.99		(0.97,1.01)	0.01	0.459	0.98		(0.96,1.01)	0.01	0.174

Notes. N=6992; Reference for focal predictor: never/rarely. RR, risk-ratio, null effect is 1.00; ES, effect size measure for standardized regression coefficient, null effect is 0.00; SE, standard error, the SE reported for binary/Likert-type outcomes where risk-ratios are on the log(RR) scale; CI, confidence interval; p-value, a Wald-type test of the null hypothesis that the effect of the focal predictor is zero; (a) item part of the Happiness & Life Satisfaction domain of the Secure Flourishing Index; (b) item part of the Physical & Mental Health domain of the Secure Flourishing Index; (c) item part of the Meaning & Purpose domain of the Secure Flourishing Index; (d) item part of the Character & Virtue domain of the Secure Flourishing Index; (e) item part of the Subjective Social Connectedness domain of the Secure Flourishing Index; (f) item part of the Financial & Material Security domain of the Secure Flourishing Index.

Multiple imputation was performed to impute missing data on the covariates, exposure, and outcomes. All models controlled for sociodemographic and childhood factors assessed at Wave 1. For Model 2 with PC (principal components), the first seven principal components of the entire set of contemporaneous confounders assessed at Wave 1 were included as additional covariates of the outcomes at Wave 2.

An outcome-wide analytic approach was used, and a separate model was run for each outcome. A different type of model was run depending on the nature of the outcome: (1) for each binary outcome, a weighted generalized linear model (with a log link and Poisson distribution) was used to estimate an RR; and (2) for each continuous outcome, a weighted linear regression model was used to estimate a ES. All effect sizes were standardized. For continuous outcomes, the ES represents the change in SD on the outcome between the lower and upper categories of the binary focal predictor. For binary outcomes, the RR represents the change in risk of being in the upper category compared to the lower category between the lower and upper categories of the binary focal predictor.

P-value significance thresholds: $p < 0.05$*, $p < 0.005$**, (Bonferroni) $p < 6.41e-04$***, correction for multiple testing using Bonferroni adjusted significant threshold.

Table S17h. Associations of forgivingness with adult well-being and other outcomes at Wave 2 in Indonesia using complete-case analyses with attrition weights.

	Model 1: Demographic and Childhood Variables as Covariates					Model 2: Demographic, Childhood, and Other Wave 1 Confounding Variables (Via Principal Components) as Covariates				
Outcome	RR	ES	95% CI	SE	p-value	RR	ES	95% CI	SE	p-value
Human Flourishing										
Secure flourishing index		0.02	(-0.04,0.08)	0.03	0.499		-0.02	(-0.07,0.04)	0.03	0.561
Flourishing index		0.01	(-0.05,0.07)	0.03	0.810		-0.03	(-0.08,0.02)	0.03	0.287
Happiness & life satisfaction		-0.01	(-0.06,0.03)	0.02	0.565		-0.04	(-0.08,0.01)	0.02	0.095
Physical & mental health		0.01	(-0.04,0.05)	0.02	0.806		-0.02	(-0.07,0.02)	0.02	0.289
Meaning & purpose		0.01	(-0.05,0.06)	0.03	0.770		-0.02	(-0.07,0.03)	0.03	0.354
Character & virtue		0.00	(-0.06,0.07)	0.03	0.947		-0.03	(-0.09,0.03)	0.03	0.333
Close social relationships		0.03	(-0.03,0.09)	0.03	0.360		0.00	(-0.05,0.06)	0.03	0.873
Financial & material security		0.04	(-0.01,0.09)	0.03	0.155		0.02	(-0.03,0.08)	0.03	0.428
Psychological Well-Being										
Happiness (a)		-0.02	(-0.07,0.02)	0.02	0.331		-0.04	(-0.09,-0.00)	0.02	0.049*
Life satisfaction (a)		-0.00	(-0.05,0.05)	0.03	0.934		-0.02	(-0.06,0.02)	0.02	0.380
Current life evaluation		-0.03	(-0.09,0.03)	0.03	0.319		-0.05	(-0.11,0.01)	0.03	0.077
Future life evaluation		0.00	(-0.06,0.07)	0.03	0.933		-0.02	(-0.08,0.04)	0.03	0.525
Optimism		0.00	(-0.05,0.06)	0.03	0.929		-0.02	(-0.08,0.03)	0.03	0.401
Freedom to pursue what's important		-0.06	(-0.10,-0.02)	0.02	0.006*		-0.08	(-0.12,-0.03)	0.02	8.49e-04**
Inner peace	1.03		(1.00,1.06)	0.01	0.050*	1.01		(0.99,1.04)	0.01	0.323
Life balance	1.02		(0.99,1.05)	0.01	0.158	1.00		(0.98,1.03)	0.01	0.749
Sense of mastery	1.04		(1.01,1.06)	0.01	4.85e-03**	1.02		(1.00,1.05)	0.01	0.089
Meaningful activities (c)		-0.02	(-0.08,0.03)	0.03	0.365		-0.05	(-0.10,0.00)	0.03	0.064
Understanding purpose (c)		0.04	(-0.01,0.09)	0.03	0.145		0.01	(-0.04,0.06)	0.03	0.730
Self-rated mental health (b)		0.03	(-0.02,0.08)	0.03	0.220		0.00	(-0.04,0.05)	0.02	0.853
Psychological Distress										
Traumatic distress	1.01		(0.99,1.03)	0.01	0.340	1.00		(0.98,1.03)	0.01	0.664
Depression symptoms composite	1.00		(0.98,1.02)	0.01	0.674	1.00		(0.98,1.02)	0.01	0.881
Depression – feel hopeless	1.00		(0.98,1.01)	0.01	0.722	1.00		(0.99,1.01)	0.01	0.922
Depression – loss of interest	0.99		(0.97,1.01)	0.01	0.364	0.99		(0.97,1.01)	0.01	0.414
Anxiety symptoms composite	1.00		(0.98,1.02)	0.01	0.758	1.01		(0.99,1.03)	0.01	0.472
Anxiety – feel on edge	0.99		(0.97,1.01)	0.01	0.554	1.00		(0.98,1.02)	0.01	0.897
Anxiety – cannot stop worrying	1.00		(0.99,1.02)	0.01	0.559	1.01		(0.99,1.02)	0.01	0.457
Suffering	1.01		(0.99,1.03)	0.01	0.410	1.00		(0.98,1.02)	0.01	0.840
Social Well-Being										
Relationship contentment (e)		0.05	(-0.01,0.12)	0.03	0.127		0.03	(-0.03,0.09)	0.03	0.332
Relationship satisfaction (e)		0.00	(-0.05,0.06)	0.03	0.920		-0.02	(-0.07,0.03)	0.03	0.436
Social support		0.03	(-0.03,0.08)	0.03	0.305		0.02	(-0.04,0.07)	0.03	0.553
Intimate/close friend	1.00		(0.98,1.02)	0.01	0.926	1.00		(0.98,1.02)	0.01	0.941
Government approval	1.02		(1.00,1.05)	0.01	0.104	1.01		(0.98,1.04)	0.01	0.444
Say in government	1.00		(0.98,1.02)	0.01	0.760	0.99		(0.97,1.01)	0.01	0.355
Belonging in country		0.01	(-0.04,0.06)	0.03	0.690		-0.02	(-0.07,0.03)	0.03	0.484
City/place satisfaction	1.01		(0.99,1.03)	0.01	0.250	1.01		(0.99,1.03)	0.01	0.507
Trust within country	1.04		(1.01,1.06)	0.01	3.15e-03**	1.02		(1.00,1.05)	0.01	0.048*
Social Participation										
Ever been married	1.00		(0.99,1.01)	0.01	0.930	1.00		(0.99,1.01)	0.01	0.825
Currently divorced	1.00		(0.99,1.00)	0.00	0.424	1.00		(0.99,1.00)	0.00	0.615
Number of children		-0.01	(-0.05,0.03)	0.02	0.684		-0.01	(-0.05,0.04)	0.02	0.767
Weekly+ community participation	1.00		(0.97,1.02)	0.01	0.917	0.99		(0.97,1.02)	0.01	0.633
Weekly+ religious attendance	1.01		(0.99,1.03)	0.01	0.460	1.01		(0.99,1.03)	0.01	0.467
Social Distress										
Loneliness		-0.02	(-0.07,0.03)	0.03	0.533		0.00	(-0.05,0.06)	0.03	0.854
Perceived discrimination	1.00		(0.98,1.01)	0.01	0.682	0.99		(0.98,1.01)	0.01	0.511
Character & Prosocial Behavior										
Orientation to promote good (d)		0.02	(-0.04,0.08)	0.03	0.465		-0.01	(-0.06,0.05)	0.03	0.861
Delayed gratification (d)		-0.02	(-0.08,0.05)	0.03	0.598		-0.05	(-0.10,0.01)	0.03	0.135
Hope		0.03	(-0.03,0.08)	0.03	0.304		0.01	(-0.05,0.07)	0.03	0.723
Gratitude		0.01	(-0.04,0.06)	0.03	0.773		-0.01	(-0.06,0.04)	0.03	0.712
Showing love/care		0.06	(0.01,0.12)	0.03	0.025*		0.04	(-0.01,0.10)	0.03	0.147
Forgivingness	1.08		(1.05,1.10)	0.01	5.14e-11***	1.08		(1.05,1.10)	0.01	1.06e-09***
Charitable giving	1.00		(0.98,1.01)	0.01	0.584	0.99		(0.98,1.01)	0.01	0.379
Helping strangers	0.98		(0.96,1.01)	0.01	0.184	0.98		(0.96,1.00)	0.01	0.100
Volunteering	1.00		(0.97,1.02)	0.01	0.782	0.99		(0.97,1.02)	0.01	0.519
Physical Health & Health Behavior										
Self-rated physical health (b)		-0.02	(-0.07,0.03)	0.02	0.400		-0.04	(-0.09,-0.00)	0.02	0.042*
Health problems	1.01		(0.99,1.03)	0.01	0.201	1.01		(1.00,1.03)	0.01	0.108
Pain in past 4 weeks	1.02		(1.00,1.05)	0.01	0.038*	1.02		(1.00,1.04)	0.01	0.045*
Daily smoker	0.99		(0.97,1.02)	0.01	0.598	1.00		(0.98,1.02)	0.01	0.761
Number of drinks per week		-0.03	(-0.09,0.04)	0.03	0.430		-0.02	(-0.09,0.04)	0.03	0.424
Days exercise per week		-0.05	(-0.11,0.01)	0.03	0.074		-0.07	(-0.12,-0.01)	0.03	0.022*
Socioeconomic Outcomes										
Financial security (f)		0.05	(-0.01,0.10)	0.03	0.085		0.03	(-0.02,0.09)	0.03	0.235
Material security (f)		0.02	(-0.03,0.08)	0.03	0.398		0.01	(-0.05,0.06)	0.03	0.799
Educational attainment (16+ years)	0.99		(0.99,1.00)	0.00	0.090	0.99		(0.99,1.00)	0.00	0.052
Currently employed	1.00		(0.97,1.02)	0.01	0.740	1.00		(0.97,1.02)	0.01	0.728
Financially comfortable/getting by	0.99		(0.97,1.01)	0.01	0.509	0.98		(0.96,1.01)	0.01	0.146
Own home	1.00		(0.98,1.03)	0.01	0.799	0.99		(0.97,1.02)	0.01	0.599
Income – top quintile	1.01		(0.98,1.03)	0.01	0.495	1.01		(0.98,1.03)	0.01	0.591
Religion & Spirituality										
Religious/spiritual connection	1.01		(0.99,1.03)	0.01	0.327	1.01		(0.98,1.03)	0.01	0.623
Belief in life after death	1.00		(0.99,1.01)	0.01	0.928	1.00		(0.99,1.01)	0.01	0.749
Transformative religious experience	1.01		(0.98,1.04)	0.02	0.451	1.01		(0.98,1.04)	0.02	0.616
Religious reading or listening	1.01		(0.98,1.04)	0.01	0.423	1.00		(0.97,1.03)	0.01	0.993
Prayer or meditation	1.02		(1.00,1.04)	0.01	0.092	1.01		(0.99,1.03)	0.01	0.278
Belief in God/gods/spiritual forces	1.00		(1.00,1.01)	0.00	0.398	1.00		(0.99,1.01)	0.00	0.678
Religious centrality	1.01		(1.00,1.03)	0.01	0.094	1.01		(0.99,1.03)	0.01	0.214
Religious/spiritual comfort	1.00		(0.99,1.02)	0.01	0.531	1.00		(0.99,1.01)	0.01	0.980
Feel loved by God	1.01		(0.99,1.02)	0.01	0.510	1.00		(0.99,1.02)	0.01	0.829
Feel punished by God	0.99		(0.96,1.01)	0.01	0.278	0.99		(0.96,1.01)	0.01	0.279
Experienced religious criticism	0.99		(0.97,1.01)	0.01	0.338	0.99		(0.97,1.01)	0.01	0.428
Faith-sharing	0.98		(0.96,1.01)	0.01	0.136	0.97		(0.95,1.00)	0.01	0.027*

Table S17h. Associations of forgivingness with adult well-being and other outcomes at Wave 2 in Indonesia using complete-case analyses with attrition weights.

	Model 1: Demographic and Childhood Variables as Covariates					Model 2: Demographic, Childhood, and Other Wave 1 Confounding Variables (Via Principal Components) as Covariates				
Outcome	RR	ES	95% CI	SE	p-value	RR	ES	95% CI	SE	p-value

Notes. N=2684; Reference for focal predictor: never/rarely. RR, risk-ratio, null effect is 1.00; ES, effect size measure for standardized regression coefficient, null effect is 0.00; SE, standard error, the SE reported for binary/Likert-type outcomes where risk-ratios are on the log(RR) scale; CI, confidence interval; p-value, a Wald-type test of the null hypothesis that the effect of the focal predictor is zero; (a) item part of the Happiness & Life Satisfaction domain of the Secure Flourishing Index; (b) item part of the Physical & Mental Health domain of the Secure Flourishing Index; (c) item part of the Meaning & Purpose domain of the Secure Flourishing Index; (d) item part of the Character & Virtue domain of the Secure Flourishing Index; (e) item part of the Subjective Social Connectedness domain of the Secure Flourishing Index; (f) item part of the Financial & Material Security domain of the Secure Flourishing Index.

Attrition weights were computed to adjust the complete case data (those who responded at Wave 2 to at least 50% of the questions) and multiple imputation was used to impute missing data on all remaining within wave on the covariates, exposure, and outcomes. All models controlled for sociodemographic and childhood factors assessed at Wave 1. For Model 2 with PC (principal components), the first seven principal components of the entire set of contemporaneous confounders assessed at Wave 1 were included as additional covariates of the outcomes at Wave 2.

An outcome-wide analytic approach was used, and a separate model was run for each outcome. A different type of model was run depending on the nature of the outcome: (1) for each binary outcome, a weighted generalized linear model (with a log link and Poisson distribution) was used to estimate an RR; and (2) for each continuous outcome, a weighted linear regression model was used to estimate a ES. All effect sizes were standardized. For continuous outcomes, the ES represents the change in SD on the outcome between the lower and upper categories of the binary focal predictor. For binary outcomes, the RR represents the change in risk of being in the upper category compared to the lower category between the lower and upper categories of the binary focal predictor.

P-value significance thresholds: $p < 0.05$*, $p < 0.005$**, (Bonferroni) $p < 6.41e-04$***, correction for multiple testing using Bonferroni adjusted significant threshold.

Table S17i. Sensitivity analysis of forgivingness outcome-wide results to unmeasured confounding using E-values in Indonesia

Outcome	Multiple Imputation: Model 1: Demographics and Childhood Variables as Covariates EE	ECI	Multiple Imputation: Model 2: Demographics, Childhood, and Other Wave 1 Confounders (Via Principal Components) as Covariates EE	ECI	Complete Case w/ Attrition Weights: Model 1: Demographics and Childhood Variables as Covariates EE	ECI	Complete Case w/ Attrition Weights: Model 2: Demographics, Childhood, and Other Wave 1 Confounders (Via Principal Components) as Covariates EE	ECI
Human Flourishing								
Secure flourishing index	1.43	1.02	1.09	1.00	1.29	1.00	1.24	1.00
Flourishing index	1.42	1.00	1.09	1.00	1.16	1.00	1.35	1.00
Happiness & life satisfaction	1.17	1.00	1.20	1.00	1.23	1.00	1.42	1.00
Physical & mental health	1.27	1.00	1.11	1.00	1.14	1.00	1.32	1.00
Meaning & purpose	1.45	1.00	1.21	1.00	1.17	1.00	1.32	1.00
Character & virtue	1.39	1.00	1.13	1.00	1.08	1.00	1.37	1.00
Close social relationships	1.40	1.00	1.20	1.00	1.37	1.00	1.12	1.00
Financial & material security	1.26	1.00	1.05	1.00	1.44	1.00	1.30	1.00
Psychological Well-Being								
Happiness	1.11	1.00	1.29	1.00	1.32	1.00	1.49	1.03
Life satisfaction	1.26	1.00	1.06	1.00	1.08	1.00	1.29	1.00
Current life evaluation	1.15	1.00	1.34	1.00	1.37	1.00	1.53	1.00
Future life evaluation	1.31	1.00	1.11	1.00	1.09	1.00	1.29	1.00
Optimism	1.41	1.00	1.23	1.00	1.09	1.00	1.31	1.00
Freedom to pursue what’s important	1.31	1.00	1.48	1.01	1.62	1.27	1.75	1.40
Inner peace	1.29	1.00	1.20	1.00	1.38	1.01	1.24	1.00
Life balance	1.25	1.00	1.08	1.00	1.30	1.00	1.13	1.00
Sense of mastery	1.35	1.11	1.26	1.00	1.44	1.21	1.31	1.00
Meaningful activities	1.19	1.00	1.16	1.00	1.33	1.00	1.51	1.00
Understanding purpose	1.55	1.10	1.35	1.00	1.46	1.00	1.17	1.00
Self-rated mental health	1.39	1.00	1.22	1.00	1.39	1.00	1.12	1.00
Psychological Distress								
Traumatic distress	1.15	1.00	1.08	1.00	1.20	1.00	1.13	1.00
Depression symptoms composite	1.03	1.00	1.05	1.00	1.12	1.00	1.07	1.00
Depression – feel hopeless	1.03	1.00	1.07	1.00	1.09	1.00	1.04	1.00
Depression – loss of interest	1.09	1.00	1.08	1.00	1.19	1.00	1.18	1.00
Anxiety symptoms composite	1.01	1.00	1.10	1.00	1.10	1.00	1.16	1.00
Anxiety – feel on edge	1.15	1.00	1.10	1.00	1.15	1.00	1.06	1.00
Anxiety – cannot stop worrying	1.06	1.00	1.09	1.00	1.13	1.00	1.16	1.00
Suffering	1.06	1.00	1.07	1.00	1.19	1.00	1.08	1.00
Social Well-Being								
Relationship contentment	1.47	1.00	1.32	1.00	1.55	1.00	1.38	1.00
Relationship satisfaction	1.23	1.00	1.12	1.00	1.09	1.00	1.29	1.00
Social support	1.36	1.00	1.28	1.00	1.37	1.00	1.26	1.00
Intimate/close friend	1.15	1.00	1.11	1.00	1.05	1.00	1.05	1.00
Government approval	1.16	1.00	1.06	1.00	1.31	1.00	1.20	1.00
Say in government	1.19	1.00	1.08	1.00	1.10	1.00	1.19	1.00
Belonging in country	1.32	1.00	1.10	1.00	1.20	1.00	1.27	1.00
City/place satisfaction	1.17	1.00	1.12	1.00	1.22	1.00	1.16	1.00
Trust within country	1.43	1.23	1.33	1.05	1.46	1.23	1.35	1.03
Social Participation								
Ever been married	1.02	1.00	1.02	1.00	1.04	1.00	1.07	1.00
Currently divorced	1.08	1.00	1.08	1.00	1.09	1.00	1.07	1.00
Number of children	1.22	1.00	1.20	1.00	1.18	1.00	1.16	1.00
Weekly+ community participation	1.11	1.00	1.17	1.00	1.06	1.00	1.15	1.00
Weekly+ religious attendance	1.22	1.00	1.22	1.00	1.19	1.00	1.18	1.00
Social Distress								
Loneliness	1.36	1.00	1.22	1.00	1.25	1.00	1.12	1.00
Perceived discrimination	1.06	1.00	1.10	1.00	1.12	1.00	1.15	1.00
Character & Prosocial Behavior								
Orientation to promote good	1.41	1.00	1.22	1.00	1.31	1.00	1.13	1.00
Delayed gratification	1.26	1.00	1.11	1.00	1.26	1.00	1.50	1.00
Hope	1.72	1.27	1.60	1.07	1.36	1.00	1.19	1.00
Gratitude	1.59	1.00	1.42	1.00	1.16	1.00	1.19	1.00
Showing love/care	1.68	1.31	1.53	1.08	1.63	1.17	1.47	1.00
Forgivingness	1.53	1.36	1.51	1.34	1.78	1.60	1.75	1.56
Charitable giving	1.03	1.00	1.10	1.00	1.12	1.00	1.16	1.00
Helping strangers	1.18	1.00	1.24	1.00	1.27	1.00	1.31	1.00
Volunteering	1.13	1.00	1.20	1.00	1.11	1.00	1.17	1.00
Physical Health & Health Behavior								
Self-rated physical health	1.08	1.00	1.28	1.00	1.29	1.00	1.48	1.07
Health problems	1.09	1.00	1.15	1.00	1.23	1.00	1.25	1.00
Pain in past 4 weeks	1.24	1.00	1.23	1.00	1.33	1.07	1.33	1.04
Daily smoker	1.18	1.00	1.09	1.00	1.15	1.00	1.11	1.00
Number of drinks per week	1.38	1.00	1.35	1.00	1.34	1.00	1.33	1.00
Days exercise per week	1.34	1.00	1.40	1.00	1.55	1.00	1.65	1.19
Socioeconomic Outcomes								
Financial security	1.26	1.00	1.13	1.00	1.51	1.00	1.40	1.00
Material security	1.20	1.00	1.10	1.00	1.32	1.00	1.16	1.00
Educational attainment (16+ years)	1.08	1.00	1.09	1.00	1.14	1.00	1.15	1.00
Currently employed	1.17	1.00	1.18	1.00	1.13	1.00	1.13	1.00
Financially comfortable/getting by	1.14	1.00	1.22	1.00	1.17	1.00	1.27	1.00
Own home	1.08	1.00	1.09	1.00	1.11	1.00	1.16	1.00
Income – top quintile	1.13	1.00	1.13	1.00	1.19	1.00	1.16	1.00
Religion & Spirituality								
Religious/spiritual connection	1.22	1.00	1.15	1.00	1.21	1.00	1.14	1.00
Belief in life after death	1.17	1.00	1.13	1.00	1.04	1.00	1.08	1.00
Transformative religious experience	1.28	1.00	1.23	1.00	1.22	1.00	1.17	1.00
Religious reading or listening	1.27	1.00	1.14	1.00	1.21	1.00	1.02	1.00
Prayer or meditation	1.28	1.06	1.21	1.00	1.29	1.00	1.22	1.00
Belief in God/gods/spiritual forces	1.11	1.00	1.08	1.00	1.11	1.00	1.08	1.00
Religious centrality	1.29	1.08	1.24	1.00	1.24	1.00	1.20	1.00
Religious/spiritual comfort	1.17	1.00	1.12	1.00	1.12	1.00	1.02	1.00
Feel loved by God	1.16	1.00	1.10	1.00	1.13	1.00	1.07	1.00
Feel punished by God	1.23	1.00	1.19	1.00	1.25	1.00	1.25	1.00
Experienced religious criticism	1.18	1.00	1.14	1.00	1.20	1.00	1.17	1.00
Faith-sharing	1.18	1.00	1.25	1.00	1.30	1.00	1.38	1.10

Notes. EE, E-value for estimate; ECI, E-value for the limit of the confidence interval. The formula for calculating E-values can be found in VanderWeele and Ding (2017). E-values for estimate are the minimum strength of association on the risk ratio scale that an unmeasured confounder would need to have with both the exposure and the outcome to fully explain away the observed association between the exposure and outcome, conditional on the measured covariates. E-values for the 95% CI closest to the null denote the minimum strength of association on the risk ratio scale that an unmeasured confounder would need to have with both the exposure and the outcome to shift the CI to include the null value, conditional on the measured covariates.

Table S18a. Weighted summary statistics for demographic and childhood variables in Israel

**Characteristic**	**Wave 1** N = 3,669	**Wave 2** N = 2,506
Forgivingness, n (%)		
Always	1,135 (30.9%)	601 (24.0%)
Often	1,558 (42.5%)	1,208 (48.2%)
Rarely	683 (18.6%)	457 (18.2%)
Never	271 (7.4%)	155 (6.2%)
(Missing)	21 (0.6%)	85 (3.4%)
Year of birth, n (%)		
1943 or earlier (current age: 80+ years)	80 (2.2%)	75 (3.0%)
1943-1953 (current age: 70-79 years)	330 (9.0%)	254 (10.2%)
1953-1963 (current age: 60-69 years)	453 (12.3%)	306 (12.2%)
1963-1973 (current age: 50-59 years)	509 (13.9%)	360 (14.4%)
1973-1983 (current age: 40-49 years)	613 (16.7%)	441 (17.6%)
1983-1993 (current age: 30-39 years)	672 (18.3%)	451 (18.0%)
1993-1998 (current age: 25-29 years)	387 (10.5%)	267 (10.7%)
1998-2005 (current age: 18-24 years)	624 (17.0%)	352 (14.0%)
(Missing)	0 (0%)	0 (0%)
Age of participant		
Mean	44.1	45.5
Standard Deviation	17.9	18.0
Min, Max	18.0, 94.0	18.0, 94.0
Gender, n (%)		
Male	1,782 (48.6%)	1,226 (48.9%)
Female	1,880 (51.2%)	1,279 (51.0%)
Other	0 (0.0%)	0 (0%)
(Missing)	6 (0.2%)	1 (0.1%)
Respondent marital status, n (%)		
Single/Never been married	874 (23.8%)	508 (20.3%)
Married	1,999 (54.5%)	1,467 (58.5%)
Separated	50 (1.4%)	29 (1.2%)
Divorced	266 (7.3%)	162 (6.4%)
Widowed	218 (5.9%)	148 (5.9%)
Domestic partner	192 (5.2%)	145 (5.8%)
(Missing)	69 (1.9%)	47 (1.9%)
Education (years), n (%)		
Up to 8	239 (6.5%)	141 (5.6%)
9-15	1,558 (42.5%)	1,029 (41.1%)
16+	1,870 (51.0%)	1,335 (53.3%)
(Missing)	2 (0.1%)	1 (0.0%)
Employment status, n (%)		
Employed for an employer	1,742 (47.5%)	1,297 (51.7%)
Self-employed	415 (11.3%)	258 (10.3%)
Retired	574 (15.7%)	425 (17.0%)
Student	420 (11.5%)	238 (9.5%)
Homemaker	227 (6.2%)	131 (5.2%)
Unemployed and looking for a job	156 (4.3%)	72 (2.9%)
None of these/Other	124 (3.4%)	57 (2.3%)
(Missing)	10 (0.3%)	28 (1.1%)
Current religious service attendance, n (%)		
More than once a week	671 (18.3%)	429 (17.1%)
Once a week	496 (13.5%)	328 (13.1%)

Table S18a. Weighted summary statistics for demographic and childhood variables in Israel

**Characteristic**	**Wave 1**<br>N = 3,669	**Wave 2**<br>N = 2,506
One to three times a month	374 (10.2%)	248 (9.9%)
A few times a year	1,005 (27.4%)	661 (26.4%)
Never	1,108 (30.2%)	825 (32.9%)
(Missing)	14 (0.4%)	15 (0.6%)
Immigration status, n (%)		
Born in this country	2,814 (76.7%)	1,922 (76.7%)
Born in another country	851 (23.2%)	580 (23.1%)
(Missing)	5 (0.1%)	4 (0.2%)
Parental marital status around age 12, n (%)		
Parents were married	3,175 (86.5%)	2,185 (87.2%)
Parents were divorced	285 (7.8%)	191 (7.6%)
Parents were never married	34 (0.9%)	19 (0.8%)
One or both of them had died	127 (3.5%)	79 (3.2%)
Unsure	16 (0.4%)	13 (0.5%)
(Missing)	31 (0.9%)	19 (0.8%)
Religious service attendance around age 12, n (%)		
At least once a week	895 (24.4%)	594 (23.7%)
One to three times a month	432 (11.8%)	289 (11.5%)
Less than once a month	800 (21.8%)	563 (22.5%)
Never	1,526 (41.6%)	1,048 (41.8%)
(Missing)	17 (0.5%)	12 (0.5%)
Relationship with mother when growing up, n (%)		
Very good	2,702 (73.6%)	1,843 (73.6%)
Somewhat good	780 (21.3%)	531 (21.2%)
Somewhat bad	106 (2.9%)	75 (3.0%)
Very bad	18 (0.5%)	11 (0.4%)
(Does not apply)	45 (1.2%)	33 (1.3%)
(Missing)	17 (0.5%)	13 (0.5%)
Relationship with father when growing up, n (%)		
Very good	2,307 (62.9%)	1,582 (63.1%)
Somewhat good	897 (24.5%)	586 (23.4%)
Somewhat bad	230 (6.3%)	181 (7.2%)
Very bad	38 (1.0%)	18 (0.7%)
(Does not apply)	172 (4.7%)	126 (5.0%)
(Missing)	25 (0.7%)	14 (0.5%)
Felt like an outsider in family when growing up, n (%)		
Yes	368 (10.0%)	236 (9.4%)
No	3,231 (88.0%)	2,223 (88.7%)
(Missing)	71 (1.9%)	47 (1.9%)
Experienced abuse when growing up, n (%)		
Yes	0 (0%)	0 (0%)
No	0 (0%)	0 (0%)
(Missing)	3,669 (100.0%)	2,506 (100.0%)
Self-rated health when growing up, n (%)		
Excellent	1,800 (49.1%)	1,207 (48.2%)
Very good	1,272 (34.7%)	884 (35.3%)
Good	479 (13.0%)	334 (13.3%)
Fair	104 (2.8%)	77 (3.1%)
Poor	6 (0.2%)	2 (0.1%)
(Missing)	8 (0.2%)	3 (0.1%)

Table S18a. Weighted summary statistics for demographic and childhood variables in Israel

**Characteristic**	**Wave 1**<br>N = 3,669	**Wave 2**<br>N = 2,506
Subjective financial status of family growing up, n (%)		
Lived comfortably	927 (25.3%)	620 (24.7%)
Got by	1,815 (49.5%)	1,249 (49.8%)
Found it difficult	673 (18.3%)	462 (18.4%)
Found it very difficult	237 (6.5%)	166 (6.6%)
(Missing)	16 (0.4%)	9 (0.4%)
Religious affiliation growing up, n (%)		
Christianity	57 (1.6%)	49 (2.0%)
Taoism	0 (0%)	0 (0%)
Confucianism	0 (0%)	0 (0%)
Primal, Animist, or Folk religion	4 (0.1%)	0 (0%)
Spiritism	0 (0%)	0 (0%)
Umbanda, Candomblé, and other African-derived religions	0 (0%)	0 (0%)
Chinese folk/traditional religion	0 (0%)	0 (0%)
Islam	688 (18.8%)	455 (18.1%)
Hinduism	0 (0%)	0 (0%)
Buddhism	0 (0%)	0 (0%)
Judaism	2,835 (77.3%)	1,945 (77.6%)
Sikhism	1 (0.0%)	0 (0%)
Baha'i	1 (0.0%)	1 (0.0%)
Jainism	0 (0%)	0 (0%)
Shinto	0 (0%)	0 (0%)
Some other religion	5 (0.1%)	3 (0.1%)
No religion/Atheist/Agnostic	68 (1.8%)	47 (1.9%)
(Missing)	9 (0.2%)	6 (0.2%)

Note. N (%); this table is based on non-imputed data. Cumulative percentages for variables may not add up to 100% due to rounding. Wave 1 characteristics weighted using the Gallup provided sampling weight, ANNUAL_WEIGHT_R2; Wave 2 characteristics weighted accounting for attrition by using the adjusted Wave 1 weight, ANNUAL_WEIGHT_R2, multiplied by the created attrition weight to account for dropout, to maintain nationally representative estimates for Wave 2 characteristics.

Table S18b. Weighted summary statistics for outcome variables in Israel

Outcome	Wave 1<br>N = 3,669	Wave 2<br>N = 2,506
Secure flourishing index		
Mean	7.9	7.9
Standard Deviation	1.4	1.3
Min, Max	1.3, 10.0	1.9, 10.0
(Missing)	99 (2.7%)	156 (6.2%)
Flourishing index		
Mean	8.0	8.0
Standard Deviation	1.3	1.3
Min, Max	1.6, 10.0	2.1, 10.0
(Missing)	81 (2.2%)	144 (5.8%)
Happiness & life satisfaction		
Mean	7.6	7.3
Standard Deviation	1.7	1.7
Min, Max	0.0, 10.0	0.0, 10.0
(Missing)	14 (0.4%)	19 (0.7%)
Physical & mental health		
Mean	8.3	8.4
Standard Deviation	1.7	1.6
Min, Max	0.0, 10.0	1.0, 10.0
(Missing)	4 (0.1%)	20 (0.8%)
Meaning & purpose		
Mean	8.3	8.4
Standard Deviation	1.7	1.6
Min, Max	0.0, 10.0	0.0, 10.0
(Missing)	30 (0.8%)	38 (1.5%)
Character & virtue		
Mean	7.9	8.0
Standard Deviation	1.6	1.6
Min, Max	0.0, 10.0	0.0, 10.0
(Missing)	28 (0.7%)	51 (2.0%)
Close social relationships		
Mean	7.9	7.9
Standard Deviation	2.0	1.9
Min, Max	0.0, 10.0	0.0, 10.0
(Missing)	16 (0.4%)	36 (1.4%)
Financial & material security		
Mean	7.2	7.2
Standard Deviation	2.4	2.3
Min, Max	0.0, 10.0	0.0, 10.0
(Missing)	17 (0.5%)	16 (0.6%)
Happiness		
Mean	7.8	7.5
Standard Deviation	1.7	1.7
Min, Max	0.0, 10.0	0.0, 10.0
(Missing)	4 (0.1%)	9 (0.4%)
Life satisfaction		
Mean	7.5	7.2
Standard Deviation	2.0	1.9
Min, Max	0.0, 10.0	0.0, 10.0
(Missing)	10 (0.3%)	10 (0.4%)
Current life evaluation		

Table S18b. Weighted summary statistics for outcome variables in Israel

**Outcome**	**Wave 1**<br>N = 3,669	**Wave 2**<br>N = 2,506
Mean	7.3	7.2
Standard Deviation	1.8	1.7
Min, Max	0.0, 10.0	0.0, 10.0
(Missing)	3 (<0.1%)	1 (<0.1%)
Future life evaluation		
Mean	8.1	8.0
Standard Deviation	1.9	1.9
Min, Max	0.0, 10.0	0.0, 10.0
(Missing)	74 (2.0%)	182 (7.3%)
Optimism		
Mean	8.4	8.1
Standard Deviation	1.8	1.9
Min, Max	0.0, 10.0	0.0, 10.0
(Missing)	43 (1.2%)	64 (2.5%)
Freedom to pursue what's important		
Mean	8.0	8.1
Standard Deviation	1.9	2.0
Min, Max	0.0, 10.0	0.0, 10.0
(Missing)	17 (0.5%)	17 (0.7%)
Inner peace, n (%)		
Always	1,382 (37.7%)	762 (30.4%)
Often	1,764 (48.1%)	1,282 (51.2%)
Rarely	447 (12.2%)	388 (15.5%)
Never	47 (1.3%)	30 (1.2%)
(Missing)	29 (0.8%)	44 (1.7%)
Life balance, n (%)		
Always	970 (26.4%)	472 (18.8%)
Often	2,010 (54.8%)	1,373 (54.8%)
Rarely	569 (15.5%)	530 (21.1%)
Never	74 (2.0%)	54 (2.2%)
(Missing)	46 (1.2%)	78 (3.1%)
Sense of mastery, n (%)		
Always	1,355 (36.9%)	774 (30.9%)
Often	1,820 (49.6%)	1,358 (54.2%)
Rarely	418 (11.4%)	306 (12.2%)
Never	56 (1.5%)	35 (1.4%)
(Missing)	20 (0.6%)	32 (1.3%)
Meaningful activities		
Mean	8.1	8.1
Standard Deviation	1.9	1.9
Min, Max	0.0, 10.0	0.0, 10.0
(Missing)	9 (0.3%)	12 (0.5%)
Understanding purpose		
Mean	8.4	8.6
Standard Deviation	1.8	1.6
Min, Max	0.0, 10.0	0.0, 10.0
(Missing)	22 (0.6%)	30 (1.2%)
Self-rated mental health		
Mean	8.5	8.5
Standard Deviation	1.8	1.8
Min, Max	0.0, 10.0	0.0, 10.0

Table S18b. Weighted summary statistics for outcome variables in Israel

**Outcome**	**Wave 1**<br>N = 3,669	**Wave 2**<br>N = 2,506
(Missing)	2 (<0.1%)	16 (0.6%)
Traumatic distress, n (%)		
A lot	432 (11.8%)	254 (10.1%)
Some	738 (20.1%)	452 (18.0%)
Not very much	1,096 (29.9%)	690 (27.5%)
Not at all	1,371 (37.4%)	1,070 (42.7%)
(Missing)	33 (0.9%)	41 (1.6%)
Depression symptoms composite, n (%)	793 (21.9%)	483 (19.6%)
(Missing)	43 (1.2%)	40 (1.6%)
Depression – feel hopeless, n (%)		
Nearly every day	172 (4.7%)	127 (5.1%)
More than half the days	381 (10.4%)	254 (10.2%)
Several days	1,183 (32.3%)	776 (31.0%)
Not at all	1,917 (52.3%)	1,324 (52.8%)
(Missing)	16 (0.4%)	24 (1.0%)
Depression – loss of interest, n (%)		
Nearly every day	249 (6.8%)	119 (4.7%)
More than half the days	474 (12.9%)	307 (12.2%)
Several days	1,226 (33.4%)	766 (30.6%)
Not at all	1,691 (46.1%)	1,299 (51.8%)
(Missing)	29 (0.8%)	16 (0.6%)
Anxiety symptoms composite, n (%)	585 (16.2%)	378 (15.4%)
(Missing)	54 (1.5%)	55 (2.2%)
Anxiety – feel on edge, n (%)		
Nearly every day	149 (4.1%)	88 (3.5%)
More than half the days	311 (8.5%)	209 (8.4%)
Several days	1,030 (28.1%)	654 (26.1%)
Not at all	2,146 (58.5%)	1,531 (61.1%)
(Missing)	33 (0.9%)	23 (0.9%)
Anxiety – cannot stop worrying, n (%)		
Nearly every day	156 (4.3%)	94 (3.7%)
More than half the days	357 (9.7%)	257 (10.2%)
Several days	1,079 (29.4%)	675 (26.9%)
Not at all	2,049 (55.9%)	1,443 (57.6%)
(Missing)	28 (0.8%)	38 (1.5%)
Suffering, n (%)		
A lot	212 (5.8%)	155 (6.2%)
Some	798 (21.7%)	518 (20.7%)
Not very much	1,211 (33.0%)	706 (28.2%)
Not at all	1,434 (39.1%)	1,109 (44.3%)
(Missing)	15 (0.4%)	18 (0.7%)
Relationship contentment		
Mean	8.1	8.1
Standard Deviation	2.0	1.9
Min, Max	0.0, 10.0	0.0, 10.0
(Missing)	2 (<0.1%)	24 (1.0%)
Relationship satisfaction		
Mean	7.7	7.8
Standard Deviation	2.2	2.1
Min, Max	0.0, 10.0	0.0, 10.0
(Missing)	14 (0.4%)	25 (1.0%)

Table S18b. Weighted summary statistics for outcome variables in Israel

Outcome	Wave 1<br>N = 3,669	Wave 2<br>N = 2,506
Social support		
Mean	8.5	8.8
Standard Deviation	2.1	1.8
Min, Max	0.0, 10.0	0.0, 10.0
(Missing)	15 (0.4%)	21 (0.8%)
Intimate/close friend, n (%)		
Yes	3,204 (87.3%)	2,097 (83.7%)
No	436 (11.9%)	355 (14.2%)
(Missing)	29 (0.8%)	53 (2.1%)
Government approval, n (%)		
Strongly approve	648 (17.7%)	570 (22.8%)
Somewhat approve	757 (20.6%)	401 (16.0%)
Neither approve nor disapprove	809 (22.1%)	407 (16.2%)
Somewhat disapprove	570 (15.5%)	336 (13.4%)
Strongly disapprove	782 (21.3%)	653 (26.1%)
(Missing)	103 (2.8%)	138 (5.5%)
Say in government, n (%)		
Agree	1,156 (31.5%)	809 (32.3%)
Disagree	1,620 (44.1%)	1,150 (45.9%)
Unsure	864 (23.6%)	541 (21.6%)
(Missing)	29 (0.8%)	7 (0.3%)
Belonging in country		
Mean	7.7	7.9
Standard Deviation	2.5	2.6
Min, Max	0.0, 10.0	0.0, 10.0
(Missing)	50 (1.4%)	103 (4.1%)
City/place satisfaction, n (%)		
Satisfied	3,164 (86.2%)	2,187 (87.3%)
Dissatisfied	404 (11.0%)	232 (9.3%)
Unsure	94 (2.6%)	70 (2.8%)
(Missing)	7 (0.2%)	17 (0.7%)
Trust within country, n (%)		
All people	101 (2.7%)	79 (3.1%)
Most people	778 (21.2%)	613 (24.4%)
Some people	1,390 (37.9%)	917 (36.6%)
Not very many people	1,065 (29.0%)	639 (25.5%)
None	271 (7.4%)	174 (6.9%)
(Missing)	64 (1.7%)	84 (3.4%)
Number of children		
Mean	1.0	1.1
Standard Deviation	1.5	3.4
Min, Max	0.0, 8.0	0.0, 97.0
(Missing)	13 (0.4%)	139 (5.5%)
Community participation, n (%)		
More than once a week	281 (7.7%)	165 (6.6%)
Once a week	439 (12.0%)	221 (8.8%)
One to three times a month	430 (11.7%)	263 (10.5%)
A few times a year	700 (19.1%)	671 (26.8%)
Never	1,814 (49.4%)	1,161 (46.3%)
(Missing)	6 (0.2%)	24 (1.0%)
Religious attendance, n (%)		

Table S18b. Weighted summary statistics for outcome variables in Israel

Outcome	Wave 1<br>N = 3,669	Wave 2<br>N = 2,506
More than once a week	671 (18.3%)	429 (17.1%)
Once a week	496 (13.5%)	328 (13.1%)
One to three times a month	374 (10.2%)	248 (9.9%)
A few times a year	1,005 (27.4%)	661 (26.4%)
Never	1,108 (30.2%)	825 (32.9%)
(Missing)	14 (0.4%)	15 (0.6%)
Loneliness		
Mean	2.3	2.1
Standard Deviation	2.4	2.4
Min, Max	0.0, 10.0	0.0, 10.0
(Missing)	13 (0.3%)	2 (<0.1%)
Perceived discrimination, n (%)		
Always	190 (5.2%)	87 (3.5%)
Often	574 (15.7%)	352 (14.0%)
Rarely	1,268 (34.6%)	792 (31.6%)
Never	1,613 (44.0%)	1,237 (49.4%)
(Missing)	23 (0.6%)	39 (1.6%)
Orientation to promote good		
Mean	8.4	8.5
Standard Deviation	1.6	1.5
Min, Max	0.0, 10.0	0.0, 10.0
(Missing)	8 (0.2%)	7 (0.3%)
Delayed gratification		
Mean	7.5	7.4
Standard Deviation	2.3	2.3
Min, Max	0.0, 10.0	0.0, 10.0
(Missing)	20 (0.5%)	45 (1.8%)
Hope		
Mean	8.4	8.2
Standard Deviation	1.7	1.8
Min, Max	0.0, 10.0	0.0, 10.0
(Missing)	22 (0.6%)	33 (1.3%)
Gratitude		
Mean	7.9	8.0
Standard Deviation	2.1	2.0
Min, Max	0.0, 10.0	0.0, 10.0
(Missing)	18 (0.5%)	54 (2.1%)
Showing love/care		
Mean	8.6	8.7
Standard Deviation	1.7	1.5
Min, Max	0.0, 10.0	0.0, 10.0
(Missing)	8 (0.2%)	8 (0.3%)
Forgivingness, n (%)		
Always	1,135 (30.9%)	601 (24.0%)
Often	1,558 (42.5%)	1,208 (48.2%)
Rarely	683 (18.6%)	457 (18.2%)
Never	271 (7.4%)	155 (6.2%)
(Missing)	21 (0.6%)	85 (3.4%)
Charitable giving, n (%)		
Yes	2,018 (55.0%)	1,355 (54.1%)
No	1,646 (44.8%)	1,142 (45.6%)

Table S18b. Weighted summary statistics for outcome variables in Israel

Outcome	Wave 1<br>N = 3,669	Wave 2<br>N = 2,506
(Missing)	6 (0.2%)	9 (0.4%)
Helping strangers, n (%)		
Yes	2,309 (62.9%)	1,344 (53.7%)
No	1,321 (36.0%)	1,116 (44.5%)
(Missing)	39 (1.0%)	45 (1.8%)
Volunteering, n (%)		
Yes	762 (20.8%)	511 (20.4%)
No	2,899 (79.0%)	1,983 (79.1%)
(Missing)	8 (0.2%)	12 (0.5%)
Self-rated physical health		
Mean	8.1	8.3
Standard Deviation	1.9	1.8
Min, Max	0.0, 10.0	0.0, 10.0
(Missing)	1 (<0.1%)	4 (0.2%)
Health problems, n (%)		
Yes	499 (13.6%)	297 (11.8%)
No	3,140 (85.6%)	2,166 (86.4%)
(Missing)	30 (0.8%)	43 (1.7%)
Pain in past 4 weeks, n (%)		
A lot	232 (6.3%)	112 (4.5%)
Some	672 (18.3%)	359 (14.3%)
Not very much	1,144 (31.2%)	675 (26.9%)
None at all	1,615 (44.0%)	1,339 (53.4%)
(Missing)	6 (0.2%)	20 (0.8%)
Number of cigarettes per day		
Mean	3.1	2.9
Standard Deviation	7.4	7.0
Min, Max	0.0, 97.0	0.0, 74.0
(Missing)	29 (0.8%)	66 (2.6%)
Number of drinks per week		
Mean	1.2	1.0
Standard Deviation	4.1	3.5
Min, Max	0.0, 97.0	0.0, 97.0
(Missing)	52 (1.4%)	92 (3.7%)
Days exercise per week		
Mean	1.3	1.2
Standard Deviation	1.6	1.5
Min, Max	0.0, 7.0	0.0, 7.0
(Missing)	14 (0.4%)	12 (0.5%)
Financial security		
Mean	7.1	7.5
Standard Deviation	2.5	2.4
Min, Max	0.0, 10.0	0.0, 10.0
(Missing)	15 (0.4%)	10 (0.4%)
Material security		
Mean	7.2	6.9
Standard Deviation	2.5	2.6
Min, Max	0.0, 10.0	0.0, 10.0
(Missing)	6 (0.2%)	7 (0.3%)
Educational attainment (16+ years), n (%)		
Up to 8	239 (6.5%)	141 (5.6%)

Table S18b. Weighted summary statistics for outcome variables in Israel

Outcome	Wave 1<br>N = 3,669	Wave 2<br>N = 2,506
9-15	1,558 (42.5%)	1,029 (41.1%)
16+	1,870 (51.0%)	1,335 (53.3%)
(Missing)	2 (0.1%)	1 (0.0%)
Currently employed, n (%)		
Employed for an employer	1,742 (47.5%)	1,297 (51.7%)
Self-employed	415 (11.3%)	258 (10.3%)
Retired	574 (15.7%)	425 (17.0%)
Student	420 (11.5%)	238 (9.5%)
Homemaker	227 (6.2%)	131 (5.2%)
Unemployed and looking for a job	156 (4.3%)	72 (2.9%)
None of these/Other	124 (3.4%)	57 (2.3%)
(Missing)	10 (0.3%)	28 (1.1%)
Financially comfortable/getting by, n (%)		
Living comfortably on present income	752 (20.5%)	465 (18.6%)
Getting by on present income	1,955 (53.3%)	1,428 (57.0%)
Finding it difficult on present income	699 (19.0%)	469 (18.7%)
Finding it very difficult on present income	193 (5.3%)	114 (4.6%)
(Missing)	70 (1.9%)	29 (1.2%)
Own home, n (%)		
Someone in this household owns this home	2,337 (63.7%)	1,680 (67.0%)
Someone in this household rents this home	821 (22.4%)	580 (23.1%)
Both	95 (2.6%)	61 (2.4%)
Neither	363 (9.9%)	138 (5.5%)
Rent	0 (0%)	0 (0%)
Own	0 (0%)	0 (0%)
Something else	0 (0%)	0 (0%)
(Missing)	53 (1.4%)	47 (1.9%)
Religious/spiritual connection, n (%)		
Always	1,305 (35.6%)	749 (29.9%)
Often	997 (27.2%)	698 (27.9%)
Rarely	804 (21.9%)	510 (20.3%)
Never	533 (14.5%)	521 (20.8%)
(Missing)	31 (0.8%)	28 (1.1%)
Belief in life after death, n (%)		
Yes	2,001 (54.5%)	1,403 (56.0%)
No	783 (21.3%)	579 (23.1%)
Unsure	844 (23.0%)	492 (19.6%)
(Missing)	41 (1.1%)	32 (1.3%)
Transformative religious experience, n (%)		
Yes	852 (23.2%)	483 (19.3%)
No	2,759 (75.2%)	1,921 (76.6%)
(Missing)	58 (1.6%)	102 (4.1%)
Religious reading or listening, n (%)		
More than once a day	586 (16.0%)	399 (15.9%)
About once a day	516 (14.1%)	416 (16.6%)
Sometimes	1,254 (34.2%)	745 (29.7%)
Never	1,298 (35.4%)	921 (36.8%)
(Missing)	15 (0.4%)	25 (1.0%)
Prayer or meditation, n (%)		
More than once a day	875 (23.9%)	612 (24.4%)
About once a day	507 (13.8%)	301 (12.0%)

Table S18b. Weighted summary statistics for outcome variables in Israel

Outcome	Wave 1<br>N = 3,669	Wave 2<br>N = 2,506
Sometimes	992 (27.0%)	625 (24.9%)
Never	1,283 (35.0%)	938 (37.4%)
(Missing)	12 (0.3%)	30 (1.2%)
Belief in God/gods/spiritual forces, n (%)		
One God	2,648 (72.2%)	1,780 (71.0%)
More than one god	0 (0%)	0 (0%)
An impersonal spiritual force	0 (0%)	0 (0%)
None of these	462 (12.6%)	380 (15.2%)
Unsure	540 (14.7%)	323 (12.9%)
(Missing)	19 (0.5%)	22 (0.9%)
Religious centrality, n (%)		
Agree	1,709 (46.6%)	1,116 (44.5%)
Disagree	741 (20.2%)	434 (17.3%)
Not relevant	824 (22.5%)	732 (29.2%)
Unsure	373 (10.2%)	206 (8.2%)
(Missing)	22 (0.6%)	18 (0.7%)
Religious/spiritual comfort, n (%)		
Agree	2,208 (60.2%)	1,428 (57.0%)
Disagree	526 (14.3%)	329 (13.1%)
Not relevant	679 (18.5%)	586 (23.4%)
Unsure	249 (6.8%)	135 (5.4%)
(Missing)	6 (0.2%)	27 (1.1%)
Feel loved by God, n (%)		
Agree	2,138 (58.3%)	1,301 (51.9%)
Disagree	379 (10.3%)	288 (11.5%)
Not relevant	648 (17.7%)	588 (23.5%)
Unsure	479 (13.1%)	243 (9.7%)
(Missing)	25 (0.7%)	86 (3.4%)
Feel punished by God, n (%)		
Agree	740 (20.2%)	448 (17.9%)
Disagree	1,689 (46.0%)	1,074 (42.8%)
Not relevant	697 (19.0%)	616 (24.6%)
Unsure	516 (14.1%)	278 (11.1%)
(Missing)	27 (0.7%)	90 (3.6%)
Experienced religious criticism, n (%)		
Agree	394 (10.7%)	197 (7.9%)
Disagree	1,808 (49.3%)	1,216 (48.5%)
Not relevant	1,125 (30.7%)	915 (36.5%)
Unsure	319 (8.7%)	175 (7.0%)
(Missing)	22 (0.6%)	2 (0.1%)
Faith-sharing, n (%)		
Agree	1,728 (47.1%)	1,080 (43.1%)
Disagree	845 (23.0%)	605 (24.1%)
Not relevant	906 (24.7%)	747 (29.8%)
Unsure	181 (4.9%)	65 (2.6%)
(Missing)	10 (0.3%)	8 (0.3%)

Note. N (%); this table is based on non-imputed data. Cumulative percentages for variables may not add up to 100% due to rounding. Wave 1 characteristics weighted using the Gallup provided sampling weight, ANNUAL_WEIGHT_R2; Wave 2 characteristics weighted accounting for attrition by using the adjusted Wave 1 weight, ANNUAL_WEIGHT_R2, multiplied by the created attrition weight to account for dropout, to maintain nationally representative estimates for Wave 2 characteristics.

Table S18c. Unweighted summary statistics for demographic and childhood variables in Israel by retention status

**Characteristic**	**Attritors–Not Observed in Wave 2**<br>N = 1,166	**Retained–Observed in Wave 2**<br>N = 2,555
Forgivingness, n (%)		
Always	321 (27.6%)	833 (32.6%)
Often	528 (45.3%)	1,050 (41.1%)
Rarely	241 (20.7%)	450 (17.6%)
Never	72 (6.2%)	204 (8.0%)
(Missing)	4 (0.3%)	18 (0.7%)
Year of birth, n (%)		
1943 or earlier (current age: 80+ years)	12 (1.0%)	70 (2.8%)
1943-1953 (current age: 70-79 years)	68 (5.8%)	270 (10.6%)
1953-1963 (current age: 60-69 years)	105 (9.0%)	357 (14.0%)
1963-1973 (current age: 50-59 years)	158 (13.6%)	359 (14.0%)
1973-1983 (current age: 40-49 years)	190 (16.3%)	432 (16.9%)
1983-1993 (current age: 30-39 years)	249 (21.4%)	430 (16.8%)
1993-1998 (current age: 25-29 years)	133 (11.4%)	259 (10.1%)
1998-2005 (current age: 18-24 years)	251 (21.5%)	378 (14.8%)
(Missing)	0 (0%)	0 (0%)
Age of participant		
Mean	40.6	45.9
Standard Deviation	16.6	18.3
Min, Max	18.0, 94.0	18.0, 93.0
Gender, n (%)		
Male	536 (46.0%)	1,274 (49.9%)
Female	625 (53.6%)	1,280 (50.1%)
Other	0 (0.0%)	0 (0%)
(Missing)	5 (0.4%)	1 (0.0%)
Respondent marital status, n (%)		
Single/Never been married	324 (27.8%)	558 (21.9%)
Married	590 (50.6%)	1,442 (56.4%)
Separated	26 (2.3%)	24 (0.9%)
Divorced	77 (6.6%)	193 (7.6%)
Widowed	51 (4.4%)	171 (6.7%)
Domestic partner	71 (6.1%)	122 (4.8%)
(Missing)	26 (2.2%)	44 (1.7%)
Education (years), n (%)		
Up to 8	66 (5.6%)	178 (7.0%)
9-15	467 (40.1%)	1,114 (43.6%)
16+	633 (54.3%)	1,261 (49.4%)
(Missing)	0 (0%)	2 (0.1%)
Employment status, n (%)		
Employed for an employer	555 (47.6%)	1,212 (47.4%)
Self-employed	138 (11.9%)	282 (11.0%)
Retired	119 (10.2%)	469 (18.3%)
Student	160 (13.7%)	264 (10.3%)
Homemaker	76 (6.5%)	155 (6.1%)
Unemployed and looking for a job	62 (5.3%)	96 (3.7%)
None of these/Other	52 (4.4%)	73 (2.9%)
(Missing)	4 (0.4%)	5 (0.2%)
Current religious service attendance, n (%)		
More than once a week	176 (15.1%)	508 (19.9%)

Table S18c. Unweighted summary statistics for demographic and childhood variables in Israel by retention status

Characteristic	Attritors–Not Observed in Wave 2<br>N = 1,166	Retained–Observed in Wave 2<br>N = 2,555
Once a week	156 (13.4%)	347 (13.6%)
One to three times a month	116 (10.0%)	263 (10.3%)
A few times a year	380 (32.6%)	634 (24.8%)
Never	329 (28.2%)	797 (31.2%)
(Missing)	8 (0.6%)	6 (0.3%)
Immigration status, n (%)		
Born in this country	917 (78.7%)	1,934 (75.7%)
Born in another country	248 (21.3%)	616 (24.1%)
(Missing)	0 (0%)	5 (0.2%)
Parental marital status around age 12, n (%)		
Parents were married	985 (84.5%)	2,237 (87.5%)
Parents were divorced	100 (8.6%)	189 (7.4%)
Parents were never married	15 (1.3%)	19 (0.7%)
One or both of them had died	46 (3.9%)	83 (3.2%)
Unsure	8 (0.7%)	8 (0.3%)
(Missing)	11 (1.0%)	20 (0.8%)
Religious service attendance around age 12, n (%)		
At least once a week	289 (24.8%)	618 (24.2%)
One to three times a month	138 (11.8%)	300 (11.7%)
Less than once a month	230 (19.7%)	583 (22.8%)
Never	503 (43.1%)	1,043 (40.8%)
(Missing)	6 (0.5%)	11 (0.4%)
Relationship with mother when growing up, n (%)		
Very good	856 (73.4%)	1,885 (73.7%)
Somewhat good	255 (21.8%)	536 (21.0%)
Somewhat bad	31 (2.7%)	77 (3.0%)
Very bad	8 (0.7%)	11 (0.4%)
(Does not apply)	11 (0.9%)	35 (1.4%)
(Missing)	5 (0.4%)	13 (0.5%)
Relationship with father when growing up, n (%)		
Very good	737 (63.2%)	1,603 (62.7%)
Somewhat good	301 (25.9%)	607 (23.8%)
Somewhat bad	56 (4.8%)	179 (7.0%)
Very bad	19 (1.6%)	19 (0.7%)
(Does not apply)	42 (3.6%)	133 (5.2%)
(Missing)	11 (0.9%)	14 (0.6%)
Felt like an outsider in family when growing up, n (%)		
Yes	129 (11.1%)	243 (9.5%)
No	1,014 (87.0%)	2,263 (88.6%)
(Missing)	23 (2.0%)	49 (1.9%)
Experienced abuse when growing up, n (%)		
Yes	0 (0%)	0 (0%)
No	0 (0%)	0 (0%)
(Missing)	1,166 (100.0%)	2,555 (100.0%)
Self-rated health when growing up, n (%)		
Excellent	616 (52.9%)	1,206 (47.2%)
Very good	376 (32.2%)	916 (35.9%)

Table S18c. Unweighted summary statistics for demographic and childhood variables in Israel by retention status

**Characteristic**	**Attritors–Not Observed in Wave 2** N = 1,166	**Retained–Observed in Wave 2** N = 2,555
Good	137 (11.8%)	350 (13.7%)
Fair	28 (2.4%)	79 (3.1%)
Poor	4 (0.4%)	2 (0.1%)
(Missing)	5 (0.5%)	3 (0.1%)
Subjective financial status of family growing up, n (%)		
Lived comfortably	322 (27.6%)	616 (24.1%)
Got by	572 (49.1%)	1,269 (49.7%)
Found it difficult	194 (16.7%)	490 (19.2%)
Found it very difficult	70 (6.0%)	171 (6.7%)
(Missing)	6 (0.5%)	10 (0.4%)
Religious affiliation growing up, n (%)		
Christianity	14 (1.2%)	44 (1.7%)
Taoism	0 (0%)	0 (0%)
Confucianism	0 (0%)	0 (0%)
Primal, Animist, or Folk religion	4 (0.3%)	0 (0%)
Spiritism	0 (0%)	0 (0%)
Umbanda, Candomblé, and other African-derived religions	0 (0%)	0 (0%)
Chinese folk/traditional religion	0 (0%)	0 (0%)
Islam	295 (25.3%)	398 (15.6%)
Hinduism	0 (0%)	0 (0%)
Buddhism	0 (0%)	0 (0%)
Judaism	822 (70.5%)	2,059 (80.6%)
Sikhism	1 (0.1%)	0 (0%)
Baha'i	0 (0%)	1 (0.1%)
Jainism	0 (0%)	0 (0%)
Shinto	0 (0%)	0 (0%)
Some other religion	3 (0.3%)	2 (0.1%)
No religion/Atheist/Agnostic	24 (2.0%)	45 (1.8%)
(Missing)	3 (0.3%)	6 (0.2%)

Note. N (%); this table is based on non-imputed data. Cumulative percentages for variables may not add up to 100% due to rounding.

Table S18d. Unweighted summary statistics for Wave 1 outcome variables in Israel by retention status.

**Outcome**	**Attritors–Not Observed in Wave 2** N = 1,166	**Retained–Observed in Wave 2** N = 2,555
Secure flourishing index		
Mean	7.7	8.0
Standard Deviation	1.4	1.4
Min, Max	1.3, 10.0	3.1, 10.0
(Missing)	19 (1.7%)	82 (3.2%)
Flourishing index		
Mean	7.9	8.1
Standard Deviation	1.4	1.3
Min, Max	1.6, 10.0	2.5, 10.0
(Missing)	15 (1.2%)	69 (2.7%)
Happiness & life satisfaction		
Mean	7.4	7.7
Standard Deviation	1.7	1.6
Min, Max	0.0, 10.0	0.0, 10.0
(Missing)	0 (<0.1%)	14 (0.6%)
Physical & mental health		
Mean	8.1	8.4
Standard Deviation	1.8	1.6
Min, Max	0.0, 10.0	1.5, 10.0
(Missing)	1 (<0.1%)	3 (0.1%)
Meaning & purpose		
Mean	8.1	8.3
Standard Deviation	1.7	1.7
Min, Max	0.0, 10.0	0.5, 10.0
(Missing)	6 (0.5%)	25 (1.0%)
Character & virtue		
Mean	8.0	7.9
Standard Deviation	1.6	1.6
Min, Max	1.0, 10.0	0.0, 10.0
(Missing)	6 (0.5%)	22 (0.9%)
Close social relationships		
Mean	7.8	8.0
Standard Deviation	2.0	2.0
Min, Max	0.0, 10.0	0.0, 10.0
(Missing)	3 (0.3%)	13 (0.5%)
Financial & material security		
Mean	6.8	7.3
Standard Deviation	2.5	2.3
Min, Max	0.0, 10.0	0.0, 10.0
(Missing)	5 (0.4%)	13 (0.5%)
Happiness		
Mean	7.5	7.9
Standard Deviation	1.7	1.6
Min, Max	0.0, 10.0	0.0, 10.0
(Missing)	0 (<0.1%)	4 (0.1%)
Life satisfaction		
Mean	7.3	7.6
Standard Deviation	2.0	2.0
Min, Max	0.0, 10.0	0.0, 10.0

Table S18d. Unweighted summary statistics for Wave 1 outcome variables in Israel by retention status.

**Outcome**	**Attritors–Not Observed in Wave 2** N = 1,166	**Retained–Observed in Wave 2** N = 2,555
(Missing)	0 (<0.1%)	10 (0.4%)
Current life evaluation		
Mean	7.2	7.4
Standard Deviation	1.8	1.8
Min, Max	0.0, 10.0	0.0, 10.0
(Missing)	2 (0.2%)	1 (<0.1%)
Future life evaluation		
Mean	8.1	8.1
Standard Deviation	1.8	1.9
Min, Max	0.0, 10.0	0.0, 10.0
(Missing)	8 (0.7%)	69 (2.7%)
Optimism		
Mean	8.4	8.4
Standard Deviation	1.8	1.8
Min, Max	0.0, 10.0	0.0, 10.0
(Missing)	10 (0.9%)	34 (1.3%)
Freedom to pursue what's important		
Mean	8.0	8.0
Standard Deviation	1.9	1.9
Min, Max	0.0, 10.0	0.0, 10.0
(Missing)	2 (0.2%)	15 (0.6%)
Inner peace, n (%)		
Always	382 (32.8%)	1,024 (40.1%)
Often	600 (51.5%)	1,186 (46.4%)
Rarely	161 (13.8%)	291 (11.4%)
Never	12 (1.0%)	35 (1.4%)
(Missing)	11 (0.9%)	19 (0.7%)
Life balance, n (%)		
Always	261 (22.4%)	726 (28.4%)
Often	683 (58.6%)	1,353 (52.9%)
Rarely	194 (16.6%)	383 (15.0%)
Never	22 (1.9%)	53 (2.1%)
(Missing)	7 (0.6%)	40 (1.6%)
Sense of mastery, n (%)		
Always	399 (34.2%)	978 (38.3%)
Often	607 (52.1%)	1,236 (48.4%)
Rarely	138 (11.8%)	285 (11.2%)
Never	20 (1.7%)	36 (1.4%)
(Missing)	2 (0.2%)	19 (0.7%)
Meaningful activities		
Mean	7.9	8.1
Standard Deviation	1.9	2.0
Min, Max	0.0, 10.0	0.0, 10.0
(Missing)	3 (0.3%)	6 (0.3%)
Understanding purpose		
Mean	8.2	8.5
Standard Deviation	2.0	1.8
Min, Max	0.0, 10.0	0.0, 10.0
(Missing)	3 (0.2%)	20 (0.8%)

Table S18d. Unweighted summary statistics for Wave 1 outcome variables in Israel by retention status.

**Outcome**	**Attritors–Not Observed in Wave 2**<br>N = 1,166	**Retained–Observed in Wave 2**<br>N = 2,555
Self-rated mental health		
Mean	8.2	8.6
Standard Deviation	1.9	1.7
Min, Max	0.0, 10.0	0.0, 10.0
(Missing)	0 (0%)	2 (<0.1%)
Traumatic distress, n (%)		
A lot	183 (15.7%)	251 (9.8%)
Some	272 (23.3%)	474 (18.5%)
Not very much	351 (30.1%)	761 (29.8%)
Not at all	349 (29.9%)	1,048 (41.0%)
(Missing)	11 (1.0%)	22 (0.9%)
Depression symptoms composite, n (%)	298 (25.9%)	502 (19.9%)
(Missing)	14 (1.2%)	29 (1.1%)
Depression – feel hopeless, n (%)		
Nearly every day	54 (4.7%)	120 (4.7%)
More than half the days	149 (12.8%)	235 (9.2%)
Several days	401 (34.4%)	797 (31.2%)
Not at all	554 (47.5%)	1,395 (54.6%)
(Missing)	7 (0.6%)	9 (0.4%)
Depression – loss of interest, n (%)		
Nearly every day	97 (8.3%)	154 (6.0%)
More than half the days	185 (15.8%)	293 (11.5%)
Several days	413 (35.5%)	828 (32.4%)
Not at all	463 (39.7%)	1,258 (49.2%)
(Missing)	8 (0.7%)	21 (0.8%)
Anxiety symptoms composite, n (%)	236 (20.5%)	354 (14.1%)
(Missing)	16 (1.4%)	39 (1.5%)
Anxiety – feel on edge, n (%)		
Nearly every day	59 (5.1%)	92 (3.6%)
More than half the days	129 (11.1%)	184 (7.2%)
Several days	366 (31.4%)	676 (26.4%)
Not at all	598 (51.3%)	1,585 (62.0%)
(Missing)	14 (1.2%)	19 (0.8%)
Anxiety – cannot stop worrying, n (%)		
Nearly every day	73 (6.2%)	84 (3.3%)
More than half the days	137 (11.8%)	223 (8.7%)
Several days	382 (32.8%)	709 (27.7%)
Not at all	570 (48.9%)	1,515 (59.3%)
(Missing)	4 (0.4%)	25 (1.0%)
Suffering, n (%)		
A lot	71 (6.1%)	144 (5.6%)
Some	257 (22.0%)	552 (21.6%)
Not very much	417 (35.8%)	809 (31.7%)
Not at all	416 (35.7%)	1,041 (40.7%)
(Missing)	5 (0.4%)	9 (0.4%)
Relationship contentment		
Mean	7.9	8.1
Standard Deviation	2.0	1.9
Min, Max	0.0, 10.0	0.0, 10.0

Table S18d. Unweighted summary statistics for Wave 1 outcome variables in Israel by retention status.

**Outcome**	**Attritors–Not Observed in Wave 2** N = 1,166	**Retained–Observed in Wave 2** N = 2,555
(Missing)	0 (0%)	2 (<0.1%)
Relationship satisfaction		
Mean	7.6	7.8
Standard Deviation	2.2	2.2
Min, Max	0.0, 10.0	0.0, 10.0
(Missing)	3 (0.3%)	11 (0.4%)
Social support		
Mean	8.2	8.7
Standard Deviation	2.2	2.0
Min, Max	0.0, 10.0	0.0, 10.0
(Missing)	7 (0.6%)	8 (0.3%)
Intimate/close friend, n (%)		
Yes	1,026 (88.0%)	2,222 (87.0%)
No	131 (11.2%)	312 (12.2%)
(Missing)	9 (0.8%)	21 (0.8%)
Government approval, n (%)		
Strongly approve	162 (13.9%)	499 (19.5%)
Somewhat approve	241 (20.7%)	526 (20.6%)
Neither approve nor disapprove	312 (26.7%)	505 (19.8%)
Somewhat disapprove	211 (18.1%)	364 (14.3%)
Strongly disapprove	217 (18.6%)	578 (22.6%)
(Missing)	22 (1.9%)	84 (3.3%)
Say in government, n (%)		
Agree	331 (28.4%)	843 (33.0%)
Disagree	491 (42.1%)	1,153 (45.1%)
Unsure	333 (28.6%)	539 (21.1%)
(Missing)	10 (0.9%)	20 (0.8%)
Belonging in country		
Mean	7.6	7.7
Standard Deviation	2.5	2.5
Min, Max	0.0, 10.0	0.0, 10.0
(Missing)	13 (1.1%)	39 (1.5%)
City/place satisfaction, n (%)		
Satisfied	991 (85.1%)	2,218 (86.8%)
Dissatisfied	134 (11.5%)	275 (10.8%)
Unsure	37 (3.1%)	58 (2.3%)
(Missing)	3 (0.3%)	4 (0.2%)
Trust within country, n (%)		
All people	21 (1.8%)	82 (3.2%)
Most people	227 (19.4%)	564 (22.1%)
Some people	465 (39.9%)	944 (36.9%)
Not very many people	351 (30.1%)	728 (28.5%)
None	88 (7.5%)	187 (7.3%)
(Missing)	14 (1.2%)	51 (2.0%)
Number of children		
Mean	1.1	1.0
Standard Deviation	1.5	1.5
Min, Max	0.0, 7.0	0.0, 8.0
(Missing)	10 (0.8%)	3 (0.1%)

Table S18d. Unweighted summary statistics for Wave 1 outcome variables in Israel by retention status.

Outcome	Attritors–Not Observed in Wave 2<br>N = 1,166	Retained–Observed in Wave 2<br>N = 2,555
Community participation, n (%)		
More than once a week	102 (8.8%)	182 (7.1%)
Once a week	155 (13.3%)	288 (11.3%)
One to three times a month	127 (10.9%)	310 (12.1%)
A few times a year	229 (19.6%)	480 (18.8%)
Never	550 (47.2%)	1,291 (50.5%)
(Missing)	2 (0.1%)	4 (0.2%)
Religious attendance, n (%)		
More than once a week	176 (15.1%)	508 (19.9%)
Once a week	156 (13.4%)	347 (13.6%)
One to three times a month	116 (10.0%)	263 (10.3%)
A few times a year	380 (32.6%)	634 (24.8%)
Never	329 (28.2%)	797 (31.2%)
(Missing)	8 (0.6%)	6 (0.3%)
Loneliness		
Mean	2.7	2.1
Standard Deviation	2.6	2.3
Min, Max	0.0, 10.0	0.0, 10.0
(Missing)	4 (0.3%)	9 (0.4%)
Perceived discrimination, n (%)		
Always	66 (5.6%)	127 (5.0%)
Often	200 (17.1%)	381 (14.9%)
Rarely	449 (38.5%)	834 (32.6%)
Never	443 (38.0%)	1,199 (46.9%)
(Missing)	9 (0.7%)	14 (0.6%)
Orientation to promote good		
Mean	8.4	8.4
Standard Deviation	1.6	1.6
Min, Max	0.0, 10.0	0.0, 10.0
(Missing)	3 (0.3%)	5 (0.2%)
Delayed gratification		
Mean	7.6	7.5
Standard Deviation	2.2	2.4
Min, Max	0.0, 10.0	0.0, 10.0
(Missing)	3 (0.2%)	18 (0.7%)
Hope		
Mean	8.4	8.4
Standard Deviation	1.7	1.7
Min, Max	0.0, 10.0	0.0, 10.0
(Missing)	9 (0.8%)	13 (0.5%)
Gratitude		
Mean	7.9	7.9
Standard Deviation	1.9	2.1
Min, Max	0.0, 10.0	0.0, 10.0
(Missing)	8 (0.7%)	10 (0.4%)
Showing love/care		
Mean	8.5	8.6
Standard Deviation	1.7	1.7
Min, Max	0.0, 10.0	0.0, 10.0

Table S18d. Unweighted summary statistics for Wave 1 outcome variables in Israel by retention status.

Outcome	Attritors–Not Observed in Wave 2<br>N = 1,166	Retained–Observed in Wave 2<br>N = 2,555
(Missing)	3 (0.3%)	5 (0.2%)
Forgivingness, n (%)		
Always	321 (27.6%)	833 (32.6%)
Often	528 (45.3%)	1,050 (41.1%)
Rarely	241 (20.7%)	450 (17.6%)
Never	72 (6.2%)	204 (8.0%)
(Missing)	4 (0.3%)	18 (0.7%)
Charitable giving, n (%)		
Yes	630 (54.0%)	1,417 (55.5%)
No	532 (45.7%)	1,136 (44.4%)
(Missing)	3 (0.3%)	2 (0.1%)
Helping strangers, n (%)		
Yes	784 (67.3%)	1,554 (60.8%)
No	368 (31.6%)	975 (38.2%)
(Missing)	13 (1.1%)	26 (1.0%)
Volunteering, n (%)		
Yes	275 (23.6%)	495 (19.4%)
No	890 (76.3%)	2,053 (80.3%)
(Missing)	1 (0.1%)	7 (0.3%)
Self-rated physical health		
Mean	7.9	8.2
Standard Deviation	2.0	1.8
Min, Max	0.0, 10.0	0.0, 10.0
Health problems, n (%)		
Yes	155 (13.3%)	352 (13.8%)
No	1,002 (85.9%)	2,182 (85.4%)
(Missing)	9 (0.8%)	21 (0.8%)
Pain in past 4 weeks, n (%)		
A lot	94 (8.1%)	140 (5.5%)
Some	241 (20.7%)	438 (17.1%)
Not very much	387 (33.2%)	772 (30.2%)
None at all	440 (37.7%)	1,204 (47.1%)
(Missing)	4 (0.4%)	2 (0.1%)
Number of cigarettes per day		
Mean	2.9	3.2
Standard Deviation	6.6	7.7
Min, Max	0.0, 40.0	0.0, 97.0
(Missing)	16 (1.3%)	13 (0.5%)
Number of drinks per week		
Mean	1.1	1.3
Standard Deviation	3.4	4.3
Min, Max	0.0, 97.0	0.0, 97.0
(Missing)	21 (1.8%)	32 (1.3%)
Days exercise per week		
Mean	1.4	1.2
Standard Deviation	1.7	1.6
Min, Max	0.0, 7.0	0.0, 7.0
(Missing)	5 (0.4%)	10 (0.4%)
Financial security		

Table S18d. Unweighted summary statistics for Wave 1 outcome variables in Israel by retention status.

Outcome	Attritors–Not Observed in Wave 2<br>N = 1,166	Retained–Observed in Wave 2<br>N = 2,555
Mean	6.7	7.3
Standard Deviation	2.6	2.4
Min, Max	0.0, 10.0	0.0, 10.0
(Missing)	4 (0.3%)	11 (0.4%)
Material security		
Mean	7.0	7.3
Standard Deviation	2.5	2.5
Min, Max	0.0, 10.0	0.0, 10.0
(Missing)	2 (0.1%)	4 (0.2%)
Educational attainment (16+ years), n (%)		
Up to 8	66 (5.6%)	178 (7.0%)
9-15	467 (40.1%)	1,114 (43.6%)
16+	633 (54.3%)	1,261 (49.4%)
(Missing)	0 (0%)	2 (0.1%)
Currently employed, n (%)		
Employed for an employer	555 (47.6%)	1,212 (47.4%)
Self-employed	138 (11.9%)	282 (11.0%)
Retired	119 (10.2%)	469 (18.3%)
Student	160 (13.7%)	264 (10.3%)
Homemaker	76 (6.5%)	155 (6.1%)
Unemployed and looking for a job	62 (5.3%)	96 (3.7%)
None of these/Other	52 (4.4%)	73 (2.9%)
(Missing)	4 (0.4%)	5 (0.2%)
Financially comfortable/getting by, n (%)		
Living comfortably on present income	253 (21.7%)	508 (19.9%)
Getting by on present income	574 (49.2%)	1,412 (55.3%)
Finding it difficult on present income	241 (20.7%)	466 (18.2%)
Finding it very difficult on present income	70 (6.0%)	125 (4.9%)
(Missing)	27 (2.3%)	44 (1.7%)
Own home, n (%)		
Someone in this household owns this home	663 (56.9%)	1,713 (67.0%)
Someone in this household rents this home	250 (21.5%)	583 (22.8%)
Both	38 (3.3%)	58 (2.3%)
Neither	191 (16.4%)	172 (6.7%)
Rent	0 (0%)	0 (0%)
Own	0 (0%)	0 (0%)
Something else	0 (0%)	0 (0%)
(Missing)	23 (2.0%)	29 (1.1%)
Religious/spiritual connection, n (%)		
Always	402 (34.5%)	922 (36.1%)
Often	338 (29.0%)	671 (26.3%)
Rarely	275 (23.6%)	539 (21.1%)
Never	142 (12.1%)	401 (15.7%)
(Missing)	9 (0.8%)	22 (0.9%)
Belief in life after death, n (%)		
Yes	590 (50.6%)	1,443 (56.5%)
No	240 (20.6%)	554 (21.7%)
Unsure	319 (27.4%)	533 (20.9%)
(Missing)	16 (1.4%)	25 (1.0%)

Table S18d. Unweighted summary statistics for Wave 1 outcome variables in Israel by retention status.

Outcome	Attritors–Not Observed in Wave 2 N = 1,166	Retained–Observed in Wave 2 N = 2,555
Transformative religious experience, n (%)		
Yes	335 (28.7%)	524 (20.5%)
No	819 (70.2%)	1,984 (77.7%)
(Missing)	12 (1.1%)	47 (1.8%)
Religious reading or listening, n (%)		
More than once a day	149 (12.8%)	448 (17.5%)
About once a day	174 (15.0%)	348 (13.6%)
Sometimes	440 (37.7%)	829 (32.4%)
Never	395 (33.9%)	923 (36.1%)
(Missing)	7 (0.6%)	8 (0.3%)
Prayer or meditation, n (%)		
More than once a day	275 (23.6%)	613 (24.0%)
About once a day	180 (15.5%)	332 (13.0%)
Sometimes	310 (26.6%)	696 (27.3%)
Never	395 (33.9%)	907 (35.5%)
(Missing)	5 (0.5%)	6 (0.3%)
Belief in God/gods/spiritual forces, n (%)		
One God	848 (72.7%)	1,838 (71.9%)
More than one god	0 (0%)	0 (0%)
An impersonal spiritual force	0 (0%)	0 (0%)
None of these	134 (11.5%)	336 (13.1%)
Unsure	181 (15.5%)	366 (14.3%)
(Missing)	3 (0.3%)	16 (0.6%)
Religious centrality, n (%)		
Agree	533 (45.7%)	1,201 (47.0%)
Disagree	234 (20.1%)	518 (20.3%)
Not relevant	264 (22.7%)	571 (22.4%)
Unsure	124 (10.7%)	254 (9.9%)
(Missing)	11 (0.9%)	11 (0.4%)
Religious/spiritual comfort, n (%)		
Agree	672 (57.6%)	1,570 (61.4%)
Disagree	172 (14.7%)	362 (14.1%)
Not relevant	217 (18.6%)	471 (18.4%)
Unsure	100 (8.6%)	151 (5.9%)
(Missing)	4 (0.4%)	2 (0.1%)
Feel loved by God, n (%)		
Agree	678 (58.2%)	1,490 (58.3%)
Disagree	107 (9.2%)	278 (10.9%)
Not relevant	201 (17.2%)	457 (17.9%)
Unsure	168 (14.4%)	317 (12.4%)
(Missing)	11 (1.0%)	14 (0.5%)
Feel punished by God, n (%)		
Agree	155 (13.3%)	601 (23.5%)
Disagree	574 (49.2%)	1,136 (44.4%)
Not relevant	237 (20.3%)	470 (18.4%)
Unsure	193 (16.5%)	329 (12.9%)
(Missing)	7 (0.6%)	20 (0.8%)
Experienced religious criticism, n (%)		
Agree	146 (12.5%)	252 (9.9%)

Table S18d. Unweighted summary statistics for Wave 1 outcome variables in Israel by retention status.

Outcome	Attritors–Not Observed in Wave 2<br>N = 1,166	Retained–Observed in Wave 2<br>N = 2,555
Disagree	530 (45.4%)	1,308 (51.2%)
Not relevant	361 (30.9%)	780 (30.5%)
Unsure	122 (10.5%)	200 (7.8%)
(Missing)	7 (0.6%)	15 (0.6%)
Faith-sharing, n (%)		
Agree	543 (46.5%)	1,210 (47.3%)
Disagree	222 (19.1%)	638 (25.0%)
Not relevant	320 (27.5%)	596 (23.3%)
Unsure	76 (6.5%)	106 (4.1%)
(Missing)	4 (0.4%)	6 (0.2%)

Note. N (%); this table is based on non-imputed data. Cumulative percentages for variables may not add up to 100% due to rounding.

Table S18e. Summary of fitted attrition model in Israel

Characteristic	Odds Ratio	95% CI	p-value
**ANNUAL_WEIGHT_R2**	0.96	0.83, 1.11	0.608
**Happiness & life satisfaction**	1.09	0.95, 1.26	0.210
**Physical & mental health**	1.26	1.08, 1.47	0.005
**Meaning & purpose**	0.96	0.82, 1.12	0.604
**Character & virtue**	0.84	0.74, 0.94	0.004
**Close social relationships**	0.92	0.80, 1.06	0.261
**Financial & material security**	0.98	0.86, 1.12	0.729
**Extraversion**	0.95	0.87, 1.03	0.187
**Openness to experience**	1.00	0.90, 1.10	0.936
**Agreeableness**	0.99	0.90, 1.09	0.814
**Conscientiousness**	0.97	0.87, 1.08	0.525
**Neuroticism**	1.06	0.96, 1.18	0.257
**Depression symptoms composite**	1.02	0.91, 1.15	0.742
**Anxiety symptoms composite**	0.97	0.85, 1.10	0.630
**Loneliness**	0.83	0.74, 0.94	0.005
**Days exercise per week**	0.97	0.88, 1.06	0.471
**Year of birth (age group)**			
1983-1993 (current age: 30-39 years)	—	—	
1973-1983 (current age: 40-49 years)	1.43	1.09, 1.89	0.012
1998-2005 (current age: 18-24 years)	0.83	0.58, 1.17	0.281
1963-1973 (current age: 50-59 years)	1.35	1.04, 1.76	0.024
1953-1963 (current age: 60-69 years)	1.98	1.33, 2.95	0.001
1993-1998 (current age: 25-29 years)	1.05	0.76, 1.45	0.773
1943-1953 (current age: 70-79 years)	2.57	1.52, 4.35	8.04e-04
1943 or earlier (current age: 80+ years)	3.82	1.63, 8.92	0.003
**Gender of respondent**			
Female	—	—	
Male	1.06	0.89, 1.26	0.496
Other	0.00	0.00, 0.00	4.48e-13
**Marital status**			
Married	—	—	
Single/Never been married	1.22	0.93, 1.61	0.148
Divorced	1.30	0.87, 1.94	0.196
Widowed	1.07	0.62, 1.85	0.805
Domestic partner	0.88	0.55, 1.40	0.587
Separated	0.48	0.23, 1.02	0.055
**Employment status**			
Employed for an employer	—	—	
Retired	1.14	0.75, 1.76	0.527
Self-employed	0.87	0.66, 1.16	0.339
Student	0.86	0.62, 1.20	0.362
Homemaker	1.24	0.75, 2.03	0.387
Unemployed and looking for a job	0.87	0.56, 1.35	0.530
None of these/Other	0.55	0.35, 0.87	0.011
**Religious attendance**			
Never	—	—	
A few times a year	0.69	0.54, 0.88	0.004
More than once a week	1.19	0.84, 1.67	0.321
Once a week	0.92	0.68, 1.24	0.566
One to three times a month	1.03	0.71, 1.49	0.872
**Educational attainment (16+ years)**			
9-15	—	—	

Table S18e. Summary of fitted attrition model in Israel

Characteristic	Odds Ratio	95% CI	p-value
16+	0.75	0.61, 0.92	0.008
Up to 8	1.02	0.57, 1.82	0.939
**Born in This country**			
Born in this country	—	—	
Born in another country	0.88	0.68, 1.15	0.347
**Race plurality (prominent race/ethnic group [0] or not [1])**	0.85	0.75, 0.96	0.011
**Urbanicity**			
A large city	—	—	
A small town or village	1.10	0.80, 1.51	0.539
A rural area or on a farm	0.93	0.62, 1.38	0.704
A suburb of a large city	0.90	0.39, 2.05	0.796
**Monthly household income**			
Israel: 12,001 – 15,000 Israeli shekels	—	—	
Israel: 11,001 - 12,000 Israeli shekels	0.94	0.64, 1.37	0.736
Israel: 15,001 – 16,000 Israeli shekels	0.80	0.55, 1.16	0.226
Israel: 7,001 - 8,000 Israeli shekels	0.66	0.44, 0.99	0.045
Israel: 6,001 - 7,000 Israeli shekels	0.70	0.48, 1.02	0.060
Israel: 9,001 - 10,000 Israeli shekels	0.72	0.48, 1.10	0.126
Israel: 8,001 - 9,000 Israeli shekels	0.60	0.38, 0.95	0.031
Israel: 18,001 – 20,000 Israeli shekels	0.97	0.67, 1.43	0.891
Israel: 16,001 – 18,000 Israeli shekels	0.80	0.52, 1.24	0.309
Israel: 10,001 - 11,000 Israeli shekels	0.79	0.49, 1.25	0.302
Israel: 5,001 - 6,000 Israeli shekels	0.56	0.36, 0.86	0.010
Israel: 20,001 - 40,000 Israeli shekels	0.72	0.46, 1.14	0.153
Israel: 3,001 - 5,000 Israeli shekels	0.62	0.40, 0.97	0.037
(None/No household income)	0.61	0.33, 1.13	0.113
Israel: 1,001 - 3,000 Israeli shekels	0.70	0.37, 1.32	0.264
Israel: More than 40,000 Israeli shekels	0.49	0.22, 1.09	0.080
Israel: 1,000 Israeli shekels or less	0.20	0.09, 0.45	3.26e-04

Abbreviations: CI = Confidence Interval, OR = Odds Ratio

Notes. N=3669; attrition weights were estimated using the 'survey::svyglm(family=quasibinomial('logit'))' function. All continuous predictors were standardized and all categorical predictors used the most common category as the reference group. Reported p-values are based on the fitted regression model and no adjustments for multiple testing were done within this table.

Table S18f. Summary of principal components in Israel

PC	Percent Explained by each PC	Cumulative Percent Explained
1	29.92	29.92
2	7.40	37.32
3	6.30	43.62
4	2.61	46.23
5	2.23	48.46
6	1.96	50.42
**7**	**1.77**	**52.20**
8	1.74	53.94
9	1.54	55.48
10	1.50	56.98
11	1.41	58.40
12	1.32	59.71
13	1.29	61.01
14	1.22	62.23
15	1.20	63.42
16	1.15	64.58
17	1.11	65.69
18	1.07	66.75
19	1.03	67.78
20	1.02	68.80

Notes. N=3669; PCA was conducted using ’survey::svyprcomp(.)’ function using all available contemporaneous exposures at wave 1. All PCs were standardized prior to being used as predictors. The bolded row represented the number of retained components for analysis was 7.

Table S18g. Associations of forgivingness with adult well-being and other outcomes at Wave 2 in Israel

	Model 1: Demographic and Childhood Variables as Covariates					Model 2: Demographic, Childhood, and Other Wave 1 Confounding Variables (Via Principal Components) as Covariates				
Outcome	RR	ES	95% CI	SE	p-value	RR	ES	95% CI	SE	p-value
Human Flourishing										
Secure flourishing index		0.12	(0.07,0.16)	0.02	5.94e-07***		0.03	(-0.01,0.06)	0.02	0.127
Flourishing index		0.12	(0.07,0.16)	0.02	5.43e-07***		0.03	(-0.01,0.07)	0.02	0.097
Happiness & life satisfaction		0.07	(0.02,0.12)	0.03	0.010*		0.01	(-0.03,0.06)	0.02	0.544
Physical & mental health		0.06	(0.01,0.11)	0.02	0.015*		0.01	(-0.03,0.05)	0.02	0.724
Meaning & purpose		0.11	(0.06,0.15)	0.02	6.55e-06***		0.02	(-0.02,0.06)	0.02	0.294
Character & virtue		0.13	(0.09,0.18)	0.02	6.81e-08***		0.06	(0.01,0.11)	0.02	0.013*
Close social relationships		0.08	(0.03,0.14)	0.03	1.88e-03**		0.02	(-0.03,0.06)	0.02	0.460
Financial & material security		0.07	(0.02,0.13)	0.03	0.009*		0.00	(-0.04,0.05)	0.02	0.890
Psychological Well-Being										
Happiness (a)		0.06	(0.01,0.11)	0.02	0.017*		0.01	(-0.04,0.05)	0.02	0.727
Life satisfaction (a)		0.06	(0.01,0.11)	0.03	0.025*		0.02	(-0.03,0.07)	0.03	0.513
Current life evaluation		-0.02	(-0.07,0.02)	0.02	0.306		-0.06	(-0.11,-0.02)	0.02	0.007*
Future life evaluation		0.01	(-0.04,0.06)	0.02	0.702		-0.05	(-0.09,-0.00)	0.02	0.047*
Optimism		0.08	(0.03,0.13)	0.03	2.19e-03**		0.02	(-0.03,0.07)	0.03	0.360
Freedom to pursue what's important		0.08	(0.03,0.13)	0.03	2.93e-03**		0.00	(-0.04,0.05)	0.02	0.866
Inner peace	1.02		(1.00,1.04)	0.01	0.092	1.00		(0.98,1.02)	0.01	0.976
Life balance	1.01		(0.99,1.03)	0.01	0.359	0.99		(0.97,1.01)	0.01	0.514
Sense of mastery	1.00		(0.98,1.01)	0.01	0.645	0.98		(0.96,1.00)	0.01	0.094
Meaningful activities (c)		0.10	(0.05,0.15)	0.02	6.44e-05***		0.02	(-0.02,0.07)	0.02	0.333
Understanding purpose (c)		0.08	(0.03,0.13)	0.03	1.08e-03**		0.01	(-0.03,0.06)	0.02	0.562
Self-rated mental health (b)		0.07	(0.02,0.12)	0.03	0.011*		0.02	(-0.03,0.07)	0.03	0.402
Psychological Distress										
Traumatic distress	1.00		(0.98,1.03)	0.01	0.835	1.00		(0.97,1.02)	0.01	0.851
Depression symptoms composite	1.00		(0.99,1.02)	0.01	0.683	1.02		(1.00,1.04)	0.01	0.031*
Depression – feel hopeless	1.00		(0.98,1.02)	0.01	0.822	1.01		(1.00,1.03)	0.01	0.148
Depression – loss of interest	1.01		(0.99,1.03)	0.01	0.548	1.02		(1.00,1.04)	0.01	0.045*
Anxiety symptoms composite	1.00		(0.98,1.01)	0.01	0.690	1.01		(0.99,1.03)	0.01	0.337
Anxiety – feel on edge	0.99		(0.98,1.01)	0.01	0.325	1.00		(0.99,1.02)	0.01	0.802
Anxiety – cannot stop worrying	1.01		(0.99,1.03)	0.01	0.293	1.02		(1.00,1.04)	0.01	0.050*
Suffering	1.00		(0.98,1.02)	0.01	0.804	1.01		(0.98,1.03)	0.01	0.638
Social Well-Being										
Relationship contentment (e)		0.09	(0.03,0.14)	0.03	3.17e-03**		0.02	(-0.04,0.07)	0.03	0.509
Relationship satisfaction (e)		0.07	(0.02,0.12)	0.03	0.010*		0.01	(-0.03,0.06)	0.02	0.549
Social support		0.03	(-0.01,0.08)	0.02	0.188		-0.00	(-0.05,0.05)	0.02	0.874
Intimate/close friend	1.00		(0.98,1.02)	0.01	0.897	1.00		(0.98,1.02)	0.01	0.924
Government approval	1.05		(1.03,1.08)	0.01	3.63e-06***	1.02		(1.00,1.04)	0.01	0.142
Say in government	1.04		(1.02,1.06)	0.01	7.16e-04**	1.01		(0.99,1.03)	0.01	0.326
Belonging in country		0.10	(0.05,0.14)	0.02	3.66e-05***		0.03	(-0.01,0.08)	0.02	0.171
City/place satisfaction	1.01		(1.00,1.03)	0.01	0.132	1.01		(0.99,1.02)	0.01	0.495
Trust within country	1.00		(0.97,1.02)	0.01	0.706	1.00		(0.98,1.03)	0.01	0.839
Social Participation										
Ever been married	1.00		(0.99,1.01)	0.01	0.594	1.00		(0.99,1.02)	0.01	0.515
Currently divorced	1.00		(0.99,1.01)	0.00	0.730	1.00		(0.99,1.01)	0.00	0.795
Number of children		-0.02	(-0.07,0.02)	0.02	0.270		-0.02	(-0.07,0.03)	0.02	0.403
Weekly+ community participation	0.99		(0.97,1.00)	0.01	0.150	1.00		(0.98,1.01)	0.01	0.623
Weekly+ religious attendance	1.00		(0.99,1.01)	0.01	0.672	1.00		(0.98,1.01)	0.01	0.560
Social Distress										
Loneliness		-0.06	(-0.11,-0.01)	0.02	0.019*		-0.02	(-0.06,0.03)	0.02	0.513
Perceived discrimination	0.99		(0.97,1.01)	0.01	0.209	1.00		(0.98,1.02)	0.01	0.985
Character & Prosocial Behavior										
Orientation to promote good (d)		0.11	(0.07,0.16)	0.02	2.07e-06***		0.05	(0.01,0.10)	0.02	0.029*
Delayed gratification (d)		0.11	(0.06,0.16)	0.03	7.81e-05***		0.05	(-0.01,0.10)	0.03	0.087
Hope		0.09	(0.04,0.15)	0.03	1.05e-03**		0.03	(-0.02,0.08)	0.03	0.274
Gratitude		0.12	(0.07,0.17)	0.03	1.34e-06***		0.04	(-0.01,0.08)	0.02	0.091
Showing love/care		0.10	(0.05,0.15)	0.03	9.45e-05***		0.05	(-0.00,0.11)	0.03	0.054
Forgivingness	1.14		(1.11,1.17)	0.01	2.22e-16***	1.11		(1.09,1.14)	0.01	5.77e-15***
Charitable giving	1.06		(1.03,1.09)	0.01	1.07e-05***	1.03		(1.00,1.06)	0.01	0.022*
Helping strangers	1.05		(1.02,1.07)	0.01	3.33e-04***	1.02		(1.00,1.05)	0.01	0.094
Volunteering	1.02		(1.00,1.04)	0.01	0.018*	1.02		(1.00,1.04)	0.01	0.048*
Physical Health & Health Behavior										
Self-rated physical health (b)		0.03	(-0.01,0.08)	0.02	0.170		-0.01	(-0.05,0.03)	0.02	0.656
Health problems	0.99		(0.98,1.00)	0.01	0.153	1.00		(0.99,1.02)	0.01	0.783
Pain in past 4 weeks	0.99		(0.97,1.01)	0.01	0.301	0.99		(0.98,1.01)	0.01	0.519
Daily smoker	0.98		(0.96,1.00)	0.01	0.041*	1.02		(1.00,1.04)	0.01	0.050
Number of drinks per week		-0.04	(-0.08,-0.01)	0.02	0.020*		-0.01	(-0.05,0.04)	0.02	0.721
Days exercise per week		0.01	(-0.04,0.06)	0.03	0.571		0.02	(-0.03,0.07)	0.03	0.340
Socioeconomic Outcomes										
Financial security (f)		0.07	(0.02,0.13)	0.03	0.010*		0.01	(-0.03,0.06)	0.03	0.564
Material security (f)		0.06	(0.00,0.11)	0.03	0.045*		-0.01	(-0.06,0.04)	0.03	0.772
Educational attainment (16+ years)	1.00		(0.99,1.01)	0.00	0.814	1.00		(0.99,1.01)	0.00	0.694
Currently employed	1.00		(0.99,1.02)	0.01	0.768	1.00		(0.98,1.02)	0.01	0.957
Financially comfortable/getting by	1.01		(0.99,1.03)	0.01	0.256	0.99		(0.97,1.02)	0.01	0.628
Own home	0.99		(0.97,1.00)	0.01	0.144	0.97		(0.96,0.99)	0.01	0.005*
Income – top quintile	0.98		(0.96,1.00)	0.01	0.088	0.98		(0.96,1.00)	0.01	0.068
Religion & Spirituality										
Religious/spiritual connection	1.03		(1.01,1.05)	0.01	8.41e-04**	1.01		(0.99,1.03)	0.01	0.441
Belief in life after death	1.05		(1.03,1.07)	0.01	1.33e-05***	1.03		(1.01,1.05)	0.01	3.97e-03**
Transformative religious experience	1.00		(0.98,1.02)	0.01	0.766	1.00		(0.99,1.02)	0.01	0.681
Religious reading or listening	1.00		(0.98,1.01)	0.01	0.581	0.99		(0.97,1.00)	0.01	0.143
Prayer or meditation	1.00		(0.98,1.02)	0.01	0.861	0.98		(0.97,1.00)	0.01	0.072
Belief in God/gods/spiritual forces	1.03		(1.02,1.05)	0.01	1.53e-04***	1.01		(0.99,1.02)	0.01	0.248
Religious centrality	1.01		(0.99,1.03)	0.01	0.220	0.99		(0.98,1.01)	0.01	0.603
Religious/spiritual comfort	1.04		(1.02,1.06)	0.01	1.1e-03**	1.01		(0.99,1.03)	0.01	0.383
Feel loved by God	1.05		(1.02,1.07)	0.01	9.84e-05***	1.01		(0.99,1.03)	0.01	0.200
Feel punished by God	1.01		(0.99,1.03)	0.01	0.368	1.02		(1.00,1.03)	0.01	0.114
Experienced religious criticism	0.99		(0.98,1.00)	0.01	0.137	1.00		(0.98,1.01)	0.01	0.655
Faith-sharing	1.07		(1.04,1.09)	0.01	1.17e-07***	1.03		(1.01,1.05)	0.01	3.1e-03**

Notes. N=3669; Reference for focal predictor: never/rarely. RR, risk-ratio, null effect is 1.00; ES, effect size measure for standardized regression coefficient, null effect is 0.00; SE, standard error, the SE reported for binary/Likert-type outcomes where risk-ratios are on the log(RR) scale; CI, confidence interval; p-value, a Wald-type test of the null hypothesis that the effect of the focal predictor is zero; (a) item part of the Happiness & Life Satisfaction domain of the Secure Flourishing Index; (b) item part of the Physical & Mental Health domain of the Secure Flourishing Index; (c) item part of the Meaning & Purpose domain of the Secure Flourishing Index; (d) item part of the Character & Virtue domain of the Secure Flourishing Index; (e) item part of the Subjective Social Connectedness domain of the Secure Flourishing Index; (f) item part of the Financial & Material Security domain of the Secure Flourishing Index.

Multiple imputation was performed to impute missing data on the covariates, exposure, and outcomes. All models controlled for sociodemographic and childhood factors assessed at Wave 1. For Model 2 with PC (principal components), the first seven principal components of the entire set of contemporaneous confounders assessed at Wave 1 were included as additional covariates of the outcomes at Wave 2.

An outcome-wide analytic approach was used, and a separate model was run for each outcome. A different type of model was run depending on the nature of the outcome: (1) for each binary outcome, a weighted generalized linear model (with a log link and Poisson distribution) was used to estimate an RR; and (2) for each continuous outcome, a weighted linear regression model was used to estimate a ES. All effect sizes were standardized. For continuous outcomes, the ES represents the change in SD on the outcome between the lower and upper categories of the binary focal predictor. For binary outcomes, the RR represents the change in risk of being in the upper category compared to the lower category between the lower and upper categories of the binary focal predictor.

P-value significance thresholds: $p < 0.05$*, $p < 0.005$**, (Bonferroni) $p < 6.41e-04$***, correction for multiple testing using Bonferroni adjusted significant threshold.

Table S18h. Associations of forgivingness with adult well-being and other outcomes at Wave 2 in Israel using complete-case analyses with attrition weights.

	Model 1: Demographic and Childhood Variables as Covariates					Model 2: Demographic, Childhood, and Other Wave 1 Confounding Variables (Via Principal Components) as Covariates				
Outcome	RR	ES	95% CI	SE	p-value	RR	ES	95% CI	SE	p-value
Human Flourishing										
Secure flourishing index		0.11	(0.06,0.16)	0.03	5.47e-05***		0.02	(-0.03,0.06)	0.02	0.514
Flourishing index		0.11	(0.06,0.16)	0.03	7.51e-05***		0.02	(-0.03,0.07)	0.02	0.416
Happiness & life satisfaction		0.06	(-0.00,0.12)	0.03	0.053		0.00	(-0.06,0.06)	0.03	0.978
Physical & mental health		0.07	(0.01,0.12)	0.03	0.015*		0.01	(-0.03,0.06)	0.02	0.572
Meaning & purpose		0.11	(0.05,0.16)	0.03	2.77e-04***		0.02	(-0.03,0.07)	0.03	0.484
Character & virtue		0.13	(0.07,0.18)	0.03	8.4e-06***		0.05	(-0.01,0.11)	0.03	0.131
Close social relationships		0.07	(0.01,0.14)	0.03	0.024*		0.00	(-0.05,0.06)	0.03	0.898
Financial & material security		0.07	(0.02,0.13)	0.03	0.012*		-0.00	(-0.06,0.05)	0.03	0.873
Psychological Well-Being										
Happiness (a)		0.05	(-0.00,0.11)	0.03	0.058		-0.00	(-0.06,0.05)	0.03	0.893
Life satisfaction (a)		0.06	(-0.00,0.11)	0.03	0.070		0.00	(-0.06,0.06)	0.03	0.880
Current life evaluation		-0.04	(-0.09,0.01)	0.03	0.151		-0.08	(-0.13,-0.03)	0.03	1.03e-03**
Future life evaluation		-0.00	(-0.06,0.05)	0.03	0.899		-0.06	(-0.12,-0.01)	0.03	0.030*
Optimism		0.08	(0.01,0.14)	0.03	0.019*		0.01	(-0.05,0.08)	0.03	0.639
Freedom to pursue what's important		0.06	(0.01,0.12)	0.03	0.034*		-0.02	(-0.08,0.04)	0.03	0.470
Inner peace	1.02		(1.00,1.04)	0.01	0.032*	1.00		(0.98,1.02)	0.01	0.764
Life balance	1.01		(0.99,1.03)	0.01	0.346	0.99		(0.97,1.02)	0.01	0.487
Sense of mastery	1.00		(0.98,1.01)	0.01	0.646	0.98		(0.96,1.00)	0.01	0.089
Meaningful activities (c)		0.10	(0.05,0.16)	0.03	3.03e-04***		0.02	(-0.04,0.08)	0.03	0.482
Understanding purpose (c)		0.09	(0.03,0.15)	0.03	0.006*		0.01	(-0.04,0.06)	0.02	0.643
Self-rated mental health (b)		0.08	(0.02,0.14)	0.03	0.009*		0.02	(-0.03,0.08)	0.03	0.399
Psychological Distress										
Traumatic distress	1.00		(0.97,1.02)	0.01	0.796	0.99		(0.97,1.02)	0.01	0.680
Depression symptoms composite	1.01		(0.99,1.03)	0.01	0.439	1.03		(1.01,1.05)	0.01	0.007*
Depression – feel hopeless	1.01		(0.99,1.03)	0.01	0.499	1.02		(1.00,1.04)	0.01	0.027*
Depression – loss of interest	1.01		(0.99,1.03)	0.01	0.285	1.03		(1.01,1.05)	0.01	3.82e-03**
Anxiety symptoms composite	0.99		(0.97,1.01)	0.01	0.188	1.00		(0.98,1.02)	0.01	0.708
Anxiety – feel on edge	0.99		(0.97,1.01)	0.01	0.214	1.00		(0.99,1.02)	0.01	0.704
Anxiety – cannot stop worrying	1.00		(0.98,1.03)	0.01	0.734	1.02		(0.99,1.04)	0.01	0.132
Suffering	0.99		(0.97,1.02)	0.01	0.527	1.00		(0.98,1.03)	0.01	0.787
Social Well-Being										
Relationship contentment (e)		0.08	(0.02,0.15)	0.03	0.013*		0.00	(-0.06,0.06)	0.03	0.882
Relationship satisfaction (e)		0.06	(-0.00,0.12)	0.03	0.052		0.00	(-0.05,0.06)	0.03	0.921
Social support		0.02	(-0.03,0.07)	0.03	0.406		-0.01	(-0.06,0.04)	0.03	0.621
Intimate/close friend	1.00		(0.98,1.02)	0.01	0.768	1.00		(0.98,1.02)	0.01	0.680
Government approval	1.06		(1.04,1.09)	0.01	1.06e-06***	1.01		(0.99,1.03)	0.01	0.225
Say in government	1.04		(1.02,1.06)	0.01	3.37e-04***	1.01		(0.99,1.03)	0.01	0.516
Belonging in country		0.09	(0.04,0.14)	0.03	2.14e-04***		0.02	(-0.03,0.07)	0.03	0.356
City/place satisfaction	1.01		(1.00,1.03)	0.01	0.108	1.01		(0.99,1.03)	0.01	0.308
Trust within country	0.99		(0.96,1.02)	0.02	0.602	1.00		(0.97,1.03)	0.02	0.865
Social Participation										
Ever been married	1.00		(0.99,1.02)	0.01	0.422	1.01		(0.99,1.02)	0.01	0.472
Currently divorced	1.00		(0.99,1.00)	0.00	0.259	1.00		(0.99,1.00)	0.00	0.228
Number of children		-0.04	(-0.09,0.02)	0.03	0.184		-0.03	(-0.09,0.03)	0.03	0.307
Weekly+ community participation	0.98		(0.96,1.00)	0.01	0.057	0.99		(0.97,1.01)	0.01	0.401
Weekly+ religious attendance	1.00		(0.98,1.01)	0.01	0.598	0.99		(0.98,1.01)	0.01	0.346
Social Distress										
Loneliness		-0.04	(-0.10,0.01)	0.03	0.120		-0.00	(-0.05,0.05)	0.03	0.959
Perceived discrimination	0.98		(0.96,1.00)	0.01	0.128	1.00		(0.98,1.02)	0.01	0.984
Character & Prosocial Behavior										
Orientation to promote good (d)		0.11	(0.06,0.16)	0.03	1.82e-05***		0.05	(-0.01,0.11)	0.03	0.106
Delayed gratification (d)		0.11	(0.04,0.17)	0.03	1.19e-03**		0.03	(-0.03,0.10)	0.03	0.313
Hope		0.09	(0.03,0.15)	0.03	4.46e-03**		0.02	(-0.05,0.08)	0.03	0.621
Gratitude		0.13	(0.08,0.18)	0.03	1.14e-06***		0.04	(-0.01,0.09)	0.03	0.168
Showing love/care		0.08	(0.03,0.14)	0.03	2.89e-03**		0.03	(-0.03,0.09)	0.03	0.273
Forgivingness	1.14		(1.10,1.17)	0.02	2.22e-16***	1.11		(1.08,1.14)	0.01	1.27e-12***
Charitable giving	1.06		(1.04,1.09)	0.01	7.13e-06***	1.03		(1.00,1.05)	0.01	0.045*
Helping strangers	1.05		(1.02,1.08)	0.01	1.16e-04***	1.02		(1.00,1.05)	0.01	0.101
Volunteering	1.02		(1.00,1.04)	0.01	0.060	1.01		(0.99,1.03)	0.01	0.185
Physical Health & Health Behavior										
Self-rated physical health (b)		0.04	(-0.01,0.10)	0.03	0.127		0.00	(-0.05,0.05)	0.02	0.969
Health problems	0.99		(0.97,1.01)	0.01	0.204	1.00		(0.99,1.02)	0.01	0.626
Pain in past 4 weeks	0.99		(0.96,1.01)	0.01	0.193	0.99		(0.97,1.01)	0.01	0.387
Daily smoker	0.97		(0.95,0.99)	0.01	0.015*	1.02		(1.00,1.04)	0.01	0.131
Number of drinks per week		-0.05	(-0.09,-0.00)	0.02	0.032*		-0.01	(-0.05,0.02)	0.02	0.483
Days exercise per week		0.02	(-0.04,0.07)	0.03	0.553		0.05	(-0.01,0.10)	0.03	0.092
Socioeconomic Outcomes										
Financial security (f)		0.08	(0.02,0.14)	0.03	0.011*		0.01	(-0.04,0.06)	0.03	0.706
Material security (f)		0.06	(0.00,0.12)	0.03	0.036*		-0.02	(-0.07,0.04)	0.03	0.560
Educational attainment (16+ years)	1.00		(0.99,1.00)	0.00	0.346	1.00		(0.99,1.00)	0.00	0.210
Currently employed	1.00		(0.99,1.02)	0.01	0.603	1.01		(0.99,1.02)	0.01	0.489
Financially comfortable/getting by	1.01		(0.99,1.03)	0.01	0.368	0.99		(0.97,1.01)	0.01	0.388
Own home	1.00		(0.98,1.02)	0.01	0.949	0.99		(0.97,1.01)	0.01	0.293
Income – top quintile	0.97		(0.95,1.00)	0.01	0.036*	0.97		(0.95,1.00)	0.01	0.032*
Religion & Spirituality										
Religious/spiritual connection	1.04		(1.01,1.06)	0.01	1.81e-03**	1.00		(0.98,1.02)	0.01	0.830
Belief in life after death	1.05		(1.03,1.08)	0.01	2.35e-06***	1.03		(1.01,1.04)	0.01	3.71e-03**
Transformative religious experience	1.01		(0.99,1.03)	0.01	0.373	1.01		(0.99,1.03)	0.01	0.388
Religious reading or listening	1.00		(0.98,1.01)	0.01	0.675	0.98		(0.97,1.00)	0.01	0.115
Prayer or meditation	1.00		(0.98,1.02)	0.01	0.721	0.98		(0.96,1.00)	0.01	0.037*
Belief in God/gods/spiritual forces	1.04		(1.03,1.06)	0.01	1.15e-06***	1.01		(1.00,1.03)	0.01	0.098
Religious centrality	1.02		(0.99,1.04)	0.01	0.162	0.99		(0.97,1.01)	0.01	0.521
Religious/spiritual comfort	1.05		(1.02,1.08)	0.01	8.41e-05***	1.01		(0.99,1.03)	0.01	0.324
Feel loved by God	1.06		(1.03,1.08)	0.01	2e-05***	1.02		(0.99,1.04)	0.01	0.191
Feel punished by God	1.01		(0.99,1.03)	0.01	0.212	1.02		(1.00,1.04)	0.01	0.051
Experienced religious criticism	0.99		(0.97,1.00)	0.01	0.098	1.00		(0.98,1.01)	0.01	0.581
Faith-sharing	1.07		(1.04,1.10)	0.02	1.68e-05***	1.03		(1.00,1.06)	0.01	0.053

Table S18h. Associations of forgivingness with adult well-being and other outcomes at Wave 2 in Israel using complete-case analyses with attrition weights.

Outcome	Model 1: Demographic and Childhood Variables as Covariates					Model 2: Demographic, Childhood, and Other Wave 1 Confounding Variables (Via Principal Components) as Covariates				
	RR	ES	95% CI	SE	p-value	RR	ES	95% CI	SE	p-value

Notes. N=2490; Reference for focal predictor: never/rarely. RR, risk-ratio, null effect is 1.00; ES, effect size measure for standardized regression coefficient, null effect is 0.00; SE, standard error, the SE reported for binary/Likert-type outcomes where risk-ratios are on the log(RR) scale; CI, confidence interval; p-value, a Wald-type test of the null hypothesis that the effect of the focal predictor is zero; (a) item part of the Happiness & Life Satisfaction domain of the Secure Flourishing Index; (b) item part of the Physical & Mental Health domain of the Secure Flourishing Index; (c) item part of the Meaning & Purpose domain of the Secure Flourishing Index; (d) item part of the Character & Virtue domain of the Secure Flourishing Index; (e) item part of the Subjective Social Connectedness domain of the Secure Flourishing Index; (f) item part of the Financial & Material Security domain of the Secure Flourishing Index.

Attrition weights were computed to adjust the complete case data (those who responded at Wave 2 to at least 50% of the questions) and multiple imputation was used to impute missing data on all remaining within wave on the covariates, exposure, and outcomes. All models controlled for sociodemographic and childhood factors assessed at Wave 1. For Model 2 with PC (principal components), the first seven principal components of the entire set of contemporaneous confounders assessed at Wave 1 were included as additional covariates of the outcomes at Wave 2.

An outcome-wide analytic approach was used, and a separate model was run for each outcome. A different type of model was run depending on the nature of the outcome: (1) for each binary outcome, a weighted generalized linear model (with a log link and Poisson distribution) was used to estimate an RR; and (2) for each continuous outcome, a weighted linear regression model was used to estimate a ES. All effect sizes were standardized. For continuous outcomes, the ES represents the change in SD on the outcome between the lower and upper categories of the binary focal predictor. For binary outcomes, the RR represents the change in risk of being in the upper category compared to the lower category between the lower and upper categories of the binary focal predictor.

P-value significance thresholds: $p < 0.05$*, $p < 0.005$**, (Bonferroni) $p < 6.41e-04$***, correction for multiple testing using Bonferroni adjusted significant threshold.

Table S18i. Sensitivity analysis of forgivingness outcome-wide results to unmeasured confounding using E-values in Israel

	Multiple Imputation				Complete Case w/ Attrition Weights			
	Model 1: Demographics and Childhood Variables as Covariates		Model 2: Demographics, Childhood, and Other Wave 1 Confounders (Via Principal Components) as Covariates		Model 1: Demographics and Childhood Variables as Covariates		Model 2: Demographics, Childhood, and Other Wave 1 Confounders (Via Principal Components) as Covariates	
Outcome	EE	ECI	EE	ECI	EE	ECI	EE	ECI
Human Flourishing								
Secure flourishing index	1.86	1.58	1.30	1.00	1.81	1.49	1.21	1.00
Flourishing index	1.86	1.59	1.33	1.00	1.81	1.49	1.25	1.00
Happiness & life satisfaction	1.56	1.22	1.21	1.00	1.50	1.00	1.04	1.00
Physical & mental health	1.51	1.18	1.14	1.00	1.56	1.19	1.20	1.00
Meaning & purpose	1.80	1.52	1.26	1.00	1.80	1.45	1.23	1.00
Character & virtue	1.96	1.67	1.51	1.19	1.93	1.59	1.43	1.00
Close social relationships	1.66	1.33	1.23	1.00	1.60	1.17	1.10	1.00
Financial & material security	1.59	1.24	1.09	1.00	1.60	1.22	1.10	1.00
Psychological Well-Being								
Happiness	1.51	1.17	1.15	1.00	1.47	1.00	1.10	1.00
Life satisfaction	1.52	1.14	1.23	1.00	1.49	1.00	1.11	1.00
Current life evaluation	1.28	1.00	1.54	1.24	1.37	1.00	1.65	1.35
Future life evaluation	1.16	1.00	1.43	1.05	1.09	1.00	1.54	1.13
Optimism	1.63	1.32	1.28	1.00	1.61	1.19	1.21	1.00
Freedom to pursue what's important	1.65	1.31	1.10	1.00	1.54	1.11	1.26	1.00
Inner peace	1.25	1.00	1.03	1.00	1.29	1.07	1.09	1.00
Life balance	1.18	1.00	1.14	1.00	1.18	1.00	1.16	1.00
Sense of mastery	1.11	1.00	1.24	1.00	1.10	1.00	1.24	1.00
Meaningful activities	1.76	1.47	1.27	1.00	1.79	1.44	1.25	1.00
Understanding purpose	1.66	1.35	1.19	1.00	1.67	1.29	1.18	1.00
Self-rated mental health	1.58	1.22	1.26	1.00	1.63	1.25	1.28	1.00
Psychological Distress								
Traumatic distress	1.08	1.00	1.08	1.00	1.10	1.00	1.12	1.00
Depression symptoms composite	1.10	1.00	1.26	1.07	1.15	1.00	1.32	1.15
Depression – feel hopeless	1.07	1.00	1.22	1.00	1.14	1.00	1.27	1.08
Depression – loss of interest	1.13	1.00	1.27	1.03	1.19	1.00	1.35	1.17
Anxiety symptoms composite	1.10	1.00	1.17	1.00	1.21	1.00	1.10	1.00
Anxiety – feel on edge	1.16	1.00	1.07	1.00	1.18	1.00	1.09	1.00
Anxiety – cannot stop worrying	1.17	1.00	1.26	1.01	1.10	1.00	1.24	1.00
Suffering	1.08	1.00	1.12	1.00	1.15	1.00	1.09	1.00
Social Well-Being								
Relationship contentment	1.68	1.32	1.24	1.00	1.65	1.23	1.11	1.00
Relationship satisfaction	1.56	1.22	1.21	1.00	1.52	1.00	1.08	1.00
Social support	1.33	1.00	1.10	1.00	1.26	1.00	1.19	1.00
Intimate/close friend	1.05	1.00	1.05	1.00	1.09	1.00	1.11	1.00
Government approval	1.50	1.35	1.23	1.00	1.54	1.38	1.20	1.00
Say in government	1.40	1.23	1.18	1.00	1.41	1.25	1.14	1.00
Belonging in country	1.73	1.46	1.34	1.00	1.73	1.42	1.28	1.00
City/place satisfaction	1.21	1.00	1.14	1.00	1.21	1.00	1.17	1.00
Trust within country	1.12	1.00	1.08	1.00	1.16	1.00	1.08	1.00
Social Participation								
Ever been married	1.09	1.00	1.10	1.00	1.12	1.00	1.12	1.00
Currently divorced	1.06	1.00	1.05	1.00	1.11	1.00	1.12	1.00
Number of children	1.28	1.00	1.25	1.00	1.37	1.00	1.33	1.00
Weekly+ community participation	1.21	1.00	1.11	1.00	1.28	1.00	1.17	1.00
Weekly+ religious attendance	1.08	1.00	1.10	1.00	1.10	1.00	1.15	1.00
Social Distress								
Loneliness	1.51	1.17	1.22	1.00	1.41	1.00	1.06	1.00
Perceived discrimination	1.20	1.00	1.02	1.00	1.24	1.00	1.02	1.00
Character & Prosocial Behavior								
Orientation to promote good	1.83	1.56	1.48	1.12	1.83	1.52	1.44	1.00
Delayed gratification	1.81	1.49	1.43	1.00	1.79	1.40	1.34	1.00
Hope	1.73	1.39	1.32	1.00	1.68	1.30	1.21	1.00
Gratitude	1.90	1.60	1.38	1.00	1.94	1.63	1.36	1.00
Showing love/care	1.78	1.47	1.47	1.00	1.66	1.31	1.34	1.00
Forgivingness	2.01	1.83	1.86	1.70	2.02	1.81	1.85	1.66
Charitable giving	1.55	1.37	1.35	1.11	1.56	1.38	1.32	1.04
Helping strangers	1.45	1.27	1.27	1.00	1.48	1.30	1.28	1.00
Volunteering	1.29	1.11	1.26	1.02	1.25	1.00	1.21	1.00
Physical Health & Health Behavior								
Self-rated physical health	1.33	1.00	1.16	1.00	1.40	1.00	1.05	1.00
Health problems	1.18	1.00	1.07	1.00	1.18	1.00	1.10	1.00
Pain in past 4 weeks	1.17	1.00	1.13	1.00	1.22	1.00	1.16	1.00
Daily smoker	1.26	1.04	1.26	1.00	1.32	1.12	1.24	1.00
Number of drinks per week	1.42	1.14	1.15	1.00	1.44	1.10	1.20	1.00
Days exercise per week	1.21	1.00	1.29	1.00	1.22	1.00	1.43	1.00
Socioeconomic Outcomes								
Financial security	1.61	1.24	1.21	1.00	1.62	1.23	1.17	1.00
Material security	1.50	1.06	1.14	1.00	1.53	1.10	1.23	1.00
Educational attainment (16+ years)	1.05	1.00	1.06	1.00	1.11	1.00	1.12	1.00
Currently employed	1.08	1.00	1.03	1.00	1.10	1.00	1.12	1.00
Financially comfortable/getting by	1.20	1.00	1.12	1.00	1.17	1.00	1.16	1.00
Own home	1.22	1.00	1.31	1.15	1.04	1.00	1.19	1.00
Income – top quintile	1.25	1.00	1.26	1.00	1.31	1.07	1.31	1.08
Religion & Spirituality								
Religious/spiritual connection	1.37	1.21	1.15	1.00	1.39	1.21	1.08	1.00
Belief in life after death	1.45	1.31	1.32	1.16	1.51	1.35	1.32	1.16
Transformative religious experience	1.09	1.00	1.10	1.00	1.16	1.00	1.15	1.00
Religious reading or listening	1.11	1.00	1.20	1.00	1.10	1.00	1.23	1.00
Prayer or meditation	1.06	1.00	1.23	1.00	1.10	1.00	1.27	1.06
Belief in God/gods/spiritual forces	1.36	1.23	1.16	1.00	1.44	1.31	1.21	1.00
Religious centrality	1.19	1.00	1.12	1.00	1.23	1.00	1.14	1.00
Religious/spiritual comfort	1.40	1.23	1.17	1.00	1.48	1.30	1.19	1.00
Feel loved by God	1.45	1.29	1.20	1.00	1.52	1.34	1.23	1.00
Feel punished by God	1.16	1.00	1.22	1.00	1.21	1.00	1.28	1.00
Experienced religious criticism	1.18	1.00	1.09	1.00	1.22	1.00	1.11	1.00
Faith-sharing	1.58	1.42	1.36	1.19	1.59	1.39	1.32	1.00

Notes. EE, E-value for estimate; ECI, E-value for the limit of the confidence interval. The formula for calculating E-values can be found in VanderWeele and Ding (2017). E-values for estimate are the minimum strength of association on the risk ratio scale that an unmeasured confounder would need to have with both the exposure and the outcome to fully explain away the observed association between the exposure and outcome, conditional on the measured covariates. E-values for the 95% CI closest to the null denote the minimum strength of association on the risk ratio scale that an unmeasured confounder would need to have with both the exposure and the outcome to shift the CI to include the null value, conditional on the measured covariates.

Table S19a. Weighted summary statistics for demographic and childhood variables in Japan

**Characteristic**	**Wave 1**<br>N = 20,543	**Wave 2**<br>N = 13,969
Forgivingness, n (%)		
Always	1,609 (7.8%)	837 (6.0%)
Often	10,069 (49.0%)	6,111 (43.7%)
Rarely	7,051 (34.3%)	5,400 (38.7%)
Never	1,708 (8.3%)	1,563 (11.2%)
(Missing)	105 (0.5%)	58 (0.4%)
Year of birth, n (%)		
1943 or earlier (current age: 80+ years)	491 (2.4%)	421 (3.0%)
1943-1953 (current age: 70-79 years)	3,498 (17.0%)	2,605 (18.7%)
1953-1963 (current age: 60-69 years)	3,981 (19.4%)	2,644 (18.9%)
1963-1973 (current age: 50-59 years)	3,621 (17.6%)	2,508 (18.0%)
1973-1983 (current age: 40-49 years)	3,388 (16.5%)	2,341 (16.8%)
1983-1993 (current age: 30-39 years)	2,907 (14.2%)	1,840 (13.2%)
1993-1998 (current age: 25-29 years)	816 (4.0%)	661 (4.7%)
1998-2005 (current age: 18-24 years)	1,841 (9.0%)	950 (6.8%)
(Missing)	0 (0%)	0 (0%)
Age of participant		
Mean	51.9	53.1
Standard Deviation	17.4	17.2
Min, Max	18.0, 99.0	19.0, 99.0
Gender, n (%)		
Male	9,772 (47.6%)	6,607 (47.3%)
Female	10,676 (52.0%)	7,310 (52.3%)
Other	28 (0.1%)	17 (0.1%)
(Missing)	67 (0.3%)	34 (0.2%)
Respondent marital status, n (%)		
Single/Never been married	4,934 (24.0%)	3,295 (23.6%)
Married	11,852 (57.7%)	8,121 (58.1%)
Separated	195 (1.0%)	114 (0.8%)
Divorced	2,139 (10.4%)	1,463 (10.5%)
Widowed	1,204 (5.9%)	828 (5.9%)
Domestic partner	155 (0.8%)	110 (0.8%)
(Missing)	63 (0.3%)	37 (0.3%)
Education (years), n (%)		
Up to 8	570 (2.8%)	272 (1.9%)
9-15	14,922 (72.6%)	10,225 (73.2%)
16+	5,051 (24.6%)	3,473 (24.9%)
(Missing)	0 (0%)	0 (0%)
Employment status, n (%)		
Employed for an employer	11,102 (54.0%)	7,443 (53.3%)
Self-employed	1,738 (8.5%)	1,114 (8.0%)
Retired	2,460 (12.0%)	1,857 (13.3%)
Student	556 (2.7%)	234 (1.7%)
Homemaker	1,209 (5.9%)	807 (5.8%)
Unemployed and looking for a job	572 (2.8%)	427 (3.1%)
None of these/Other	2,873 (14.0%)	2,054 (14.7%)
(Missing)	34 (0.2%)	32 (0.2%)
Current religious service attendance, n (%)		
More than once a week	315 (1.5%)	228 (1.6%)
Once a week	359 (1.7%)	293 (2.1%)

Table S19a. Weighted summary statistics for demographic and childhood variables in Japan

**Characteristic**	**Wave 1**<br>N = 20,543	**Wave 2**<br>N = 13,969
One to three times a month	878 (4.3%)	644 (4.6%)
A few times a year	3,131 (15.2%)	1,950 (14.0%)
Never	15,745 (76.6%)	10,789 (77.2%)
(Missing)	115 (0.6%)	66 (0.5%)
Immigration status, n (%)		
Born in this country	19,540 (95.1%)	13,276 (95.0%)
Born in another country	162 (0.8%)	106 (0.8%)
(Missing)	842 (4.1%)	587 (4.2%)
Parental marital status around age 12, n (%)		
Parents were married	17,659 (86.0%)	12,050 (86.3%)
Parents were divorced	1,158 (5.6%)	783 (5.6%)
Parents were never married	600 (2.9%)	416 (3.0%)
One or both of them had died	756 (3.7%)	496 (3.6%)
Unsure	320 (1.6%)	197 (1.4%)
(Missing)	49 (0.2%)	26 (0.2%)
Religious service attendance around age 12, n (%)		
At least once a week	399 (1.9%)	273 (2.0%)
One to three times a month	891 (4.3%)	595 (4.3%)
Less than once a month	5,033 (24.5%)	3,408 (24.4%)
Never	14,099 (68.6%)	9,627 (68.9%)
(Missing)	122 (0.6%)	67 (0.5%)
Relationship with mother when growing up, n (%)		
Very good	5,639 (27.4%)	3,841 (27.5%)
Somewhat good	9,416 (45.8%)	6,431 (46.0%)
Somewhat bad	2,765 (13.5%)	1,856 (13.3%)
Very bad	801 (3.9%)	548 (3.9%)
(Does not apply)	1,856 (9.0%)	1,251 (9.0%)
(Missing)	66 (0.3%)	41 (0.3%)
Relationship with father when growing up, n (%)		
Very good	4,165 (20.3%)	2,819 (20.2%)
Somewhat good	9,065 (44.1%)	6,183 (44.3%)
Somewhat bad	3,426 (16.7%)	2,322 (16.6%)
Very bad	1,228 (6.0%)	885 (6.3%)
(Does not apply)	2,602 (12.7%)	1,723 (12.3%)
(Missing)	57 (0.3%)	37 (0.3%)
Felt like an outsider in family when growing up, n (%)		
Yes	1,981 (9.6%)	1,338 (9.6%)
No	17,110 (83.3%)	11,665 (83.5%)
(Missing)	1,452 (7.1%)	967 (6.9%)
Experienced abuse when growing up, n (%)		
Yes	1,490 (7.3%)	1,010 (7.2%)
No	18,956 (92.3%)	12,899 (92.3%)
(Missing)	97 (0.5%)	61 (0.4%)
Self-rated health when growing up, n (%)		
Excellent	2,757 (13.4%)	1,823 (13.0%)
Very good	7,110 (34.6%)	4,847 (34.7%)
Good	6,668 (32.5%)	4,548 (32.6%)
Fair	3,177 (15.5%)	2,182 (15.6%)
Poor	754 (3.7%)	516 (3.7%)
(Missing)	77 (0.4%)	53 (0.4%)

Table S19a. Weighted summary statistics for demographic and childhood variables in Japan

**Characteristic**	**Wave 1** N = 20,543	**Wave 2** N = 13,969
Subjective financial status of family growing up, n (%)		
Lived comfortably	8,353 (40.7%)	5,595 (40.0%)
Got by	8,767 (42.7%)	5,980 (42.8%)
Found it difficult	2,397 (11.7%)	1,663 (11.9%)
Found it very difficult	974 (4.7%)	694 (5.0%)
(Missing)	52 (0.3%)	38 (0.3%)
Religious affiliation growing up, n (%)		
Christianity	346 (1.7%)	230 (1.6%)
Taoism	16 (0.1%)	6 (0.0%)
Confucianism	24 (0.1%)	16 (0.1%)
Primal, Animist, or Folk religion	13 (0.1%)	4 (0.0%)
Spiritism	0 (0%)	0 (0%)
Umbanda, Candomblé, and other African-derived religions	0 (0%)	0 (0%)
Chinese folk/traditional religion	0 (0%)	0 (0%)
Islam	9 (0.0%)	0 (0%)
Hinduism	4 (0.0%)	6 (0.0%)
Buddhism	6,487 (31.6%)	4,455 (31.9%)
Judaism	0 (0%)	0 (0%)
Sikhism	0 (0%)	0 (0%)
Baha'i	7 (0.0%)	6 (0.0%)
Jainism	1 (<0.0%)	2 (0.0%)
Shinto	382 (1.9%)	253 (1.8%)
Some other religion	47 (0.2%)	29 (0.2%)
No religion/Atheist/Agnostic	12,989 (63.2%)	8,832 (63.2%)
(Missing)	217 (1.1%)	132 (0.9%)

Note. N (%); this table is based on non-imputed data. Cumulative percentages for variables may not add up to 100% due to rounding. Wave 1 characteristics weighted using the Gallup provided sampling weight, ANNUAL_WEIGHT_R2; Wave 2 characteristics weighted accounting for attrition by using the adjusted Wave 1 weight, ANNUAL_WEIGHT_R2, multiplied by the created attrition weight to account for dropout, to maintain nationally representative estimates for Wave 2 characteristics.

Table S19b. Weighted summary statistics for outcome variables in Japan

Outcome	Wave 1<br>N = 20,543	Wave 2<br>N = 13,969
Secure flourishing index		
Mean	5.9	5.7
Standard Deviation	1.8	1.8
Min, Max	0.0, 10.0	0.0, 10.0
(Missing)	927 (4.5%)	617 (4.4%)
Flourishing index		
Mean	5.9	5.8
Standard Deviation	1.8	1.8
Min, Max	0.0, 10.0	0.0, 10.0
(Missing)	817 (4.0%)	568 (4.1%)
Happiness & life satisfaction		
Mean	6.2	5.9
Standard Deviation	2.1	2.2
Min, Max	0.0, 10.0	0.0, 10.0
(Missing)	190 (0.9%)	201 (1.4%)
Physical & mental health		
Mean	6.1	6.0
Standard Deviation	2.0	2.0
Min, Max	0.0, 10.0	0.0, 10.0
(Missing)	87 (0.4%)	70 (0.5%)
Meaning & purpose		
Mean	5.8	5.7
Standard Deviation	2.1	2.1
Min, Max	0.0, 10.0	0.0, 10.0
(Missing)	185 (0.9%)	108 (0.8%)
Character & virtue		
Mean	5.7	5.6
Standard Deviation	1.8	1.8
Min, Max	0.0, 10.0	0.0, 10.0
(Missing)	167 (0.8%)	82 (0.6%)
Close social relationships		
Mean	6.0	5.9
Standard Deviation	2.2	2.2
Min, Max	0.0, 10.0	0.0, 10.0
(Missing)	300 (1.5%)	199 (1.4%)
Financial & material security		
Mean	5.7	5.5
Standard Deviation	2.7	2.6
Min, Max	0.0, 10.0	0.0, 10.0
(Missing)	142 (0.7%)	69 (0.5%)
Happiness		
Mean	6.2	6.0
Standard Deviation	2.1	2.2
Min, Max	0.0, 10.0	0.0, 10.0
(Missing)	75 (0.4%)	46 (0.3%)
Life satisfaction		
Mean	6.1	5.8
Standard Deviation	2.3	2.3
Min, Max	0.0, 10.0	0.0, 10.0
(Missing)	122 (0.6%)	160 (1.1%)
Current life evaluation		

Table S19b. Weighted summary statistics for outcome variables in Japan

**Outcome**	**Wave 1**<br>N = 20,543	**Wave 2**<br>N = 13,969
Mean	5.9	5.6
Standard Deviation	2.1	2.1
Min, Max	0.0, 10.0	0.0, 10.0
(Missing)	80 (0.4%)	57 (0.4%)
Future life evaluation		
Mean	6.0	5.7
Standard Deviation	2.2	2.2
Min, Max	0.0, 10.0	0.0, 10.0
(Missing)	84 (0.4%)	70 (0.5%)
Optimism		
Mean	6.7	6.7
Standard Deviation	2.3	2.3
Min, Max	0.0, 10.0	0.0, 10.0
(Missing)	46 (0.2%)	27 (0.2%)
Freedom to pursue what's important		
Mean	6.4	6.4
Standard Deviation	2.1	2.1
Min, Max	0.0, 10.0	0.0, 10.0
(Missing)	68 (0.3%)	35 (0.3%)
Inner peace, n (%)		
Always	2,373 (11.6%)	1,603 (11.5%)
Often	11,523 (56.1%)	7,445 (53.3%)
Rarely	5,543 (27.0%)	4,069 (29.1%)
Never	998 (4.9%)	774 (5.5%)
(Missing)	106 (0.5%)	78 (0.6%)
Life balance, n (%)		
Always	2,786 (13.6%)	1,880 (13.5%)
Often	12,011 (58.5%)	7,668 (54.9%)
Rarely	4,688 (22.8%)	3,638 (26.0%)
Never	962 (4.7%)	728 (5.2%)
(Missing)	95 (0.5%)	56 (0.4%)
Sense of mastery, n (%)		
Always	608 (3.0%)	452 (3.2%)
Often	7,435 (36.2%)	4,723 (33.8%)
Rarely	9,870 (48.0%)	6,753 (48.3%)
Never	2,465 (12.0%)	1,945 (13.9%)
(Missing)	166 (0.8%)	96 (0.7%)
Meaningful activities		
Mean	6.0	5.8
Standard Deviation	2.2	2.3
Min, Max	0.0, 10.0	0.0, 10.0
(Missing)	90 (0.4%)	56 (0.4%)
Understanding purpose		
Mean	5.7	5.6
Standard Deviation	2.3	2.3
Min, Max	0.0, 10.0	0.0, 10.0
(Missing)	100 (0.5%)	51 (0.4%)
Self-rated mental health		
Mean	6.2	6.1
Standard Deviation	2.2	2.3
Min, Max	0.0, 10.0	0.0, 10.0

Table S19b. Weighted summary statistics for outcome variables in Japan

Outcome	Wave 1<br>N = 20,543	Wave 2<br>N = 13,969
(Missing)	33 (0.2%)	31 (0.2%)
Traumatic distress, n (%)		
A lot	1,213 (5.9%)	862 (6.2%)
Some	4,627 (22.5%)	3,363 (24.1%)
Not very much	9,020 (43.9%)	6,130 (43.9%)
Not at all	5,615 (27.3%)	3,587 (25.7%)
(Missing)	67 (0.3%)	28 (0.2%)
Depression symptoms composite, n (%)	4,390 (21.6%)	3,152 (22.8%)
(Missing)	188 (0.9%)	122 (0.9%)
Depression – feel hopeless, n (%)		
Nearly every day	1,141 (5.6%)	814 (5.8%)
More than half the days	2,303 (11.2%)	1,621 (11.6%)
Several days	6,052 (29.5%)	3,990 (28.6%)
Not at all	10,943 (53.3%)	7,477 (53.5%)
(Missing)	104 (0.5%)	66 (0.5%)
Depression – loss of interest, n (%)		
Nearly every day	1,202 (5.9%)	860 (6.2%)
More than half the days	3,040 (14.8%)	2,192 (15.7%)
Several days	5,753 (28.0%)	3,994 (28.6%)
Not at all	10,456 (50.9%)	6,863 (49.1%)
(Missing)	92 (0.4%)	61 (0.4%)
Anxiety symptoms composite, n (%)	3,814 (18.7%)	2,781 (20.1%)
(Missing)	173 (0.8%)	149 (1.1%)
Anxiety – feel on edge, n (%)		
Nearly every day	1,368 (6.7%)	986 (7.1%)
More than half the days	2,550 (12.4%)	1,854 (13.3%)
Several days	7,347 (35.8%)	4,979 (35.6%)
Not at all	9,151 (44.5%)	6,035 (43.2%)
(Missing)	127 (0.6%)	114 (0.8%)
Anxiety – cannot stop worrying, n (%)		
Nearly every day	821 (4.0%)	583 (4.2%)
More than half the days	1,844 (9.0%)	1,271 (9.1%)
Several days	4,606 (22.4%)	3,227 (23.1%)
Not at all	13,219 (64.3%)	8,848 (63.3%)
(Missing)	54 (0.3%)	39 (0.3%)
Suffering, n (%)		
A lot	1,289 (6.3%)	987 (7.1%)
Some	6,267 (30.5%)	4,534 (32.5%)
Not very much	9,683 (47.1%)	6,310 (45.2%)
Not at all	3,223 (15.7%)	2,064 (14.8%)
(Missing)	80 (0.4%)	73 (0.5%)
Relationship contentment		
Mean	6.0	5.9
Standard Deviation	2.3	2.3
Min, Max	0.0, 10.0	0.0, 10.0
(Missing)	139 (0.7%)	96 (0.7%)
Relationship satisfaction		
Mean	6.0	5.8
Standard Deviation	2.3	2.3
Min, Max	0.0, 10.0	0.0, 10.0
(Missing)	178 (0.9%)	115 (0.8%)

Table S19b. Weighted summary statistics for outcome variables in Japan

Outcome	Wave 1<br>N = 20,543	Wave 2<br>N = 13,969
Social support		
Mean	5.3	5.3
Standard Deviation	2.4	2.4
Min, Max	0.0, 10.0	0.0, 10.0
(Missing)	79 (0.4%)	58 (0.4%)
Intimate/close friend, n (%)		
Yes	13,815 (67.3%)	9,429 (67.5%)
No	6,619 (32.2%)	4,475 (32.0%)
(Missing)	109 (0.5%)	65 (0.5%)
Government approval, n (%)		
Strongly approve	251 (1.2%)	151 (1.1%)
Somewhat approve	3,700 (18.0%)	1,841 (13.2%)
Neither approve nor disapprove	8,450 (41.1%)	5,005 (35.8%)
Somewhat disapprove	5,407 (26.3%)	4,312 (30.9%)
Strongly disapprove	2,638 (12.8%)	2,605 (18.6%)
(Missing)	97 (0.5%)	56 (0.4%)
Say in government, n (%)		
Agree	4,448 (21.7%)	3,322 (23.8%)
Disagree	8,128 (39.6%)	5,878 (42.1%)
Unsure	7,885 (38.4%)	4,746 (34.0%)
(Missing)	82 (0.4%)	23 (0.2%)
Belonging in country		
Mean	6.0	6.1
Standard Deviation	2.3	2.3
Min, Max	0.0, 10.0	0.0, 10.0
(Missing)	118 (0.6%)	116 (0.8%)
City/place satisfaction, n (%)		
Satisfied	11,476 (55.9%)	7,450 (53.3%)
Dissatisfied	3,648 (17.8%)	2,902 (20.8%)
Unsure	5,259 (25.6%)	3,512 (25.1%)
(Missing)	161 (0.8%)	105 (0.8%)
Trust within country, n (%)		
All people	133 (0.6%)	65 (0.5%)
Most people	5,952 (29.0%)	3,806 (27.2%)
Some people	8,832 (43.0%)	5,985 (42.8%)
Not very many people	4,697 (22.9%)	3,362 (24.1%)
None	821 (4.0%)	696 (5.0%)
(Missing)	108 (0.5%)	54 (0.4%)
Number of children		
Mean	0.4	0.3
Standard Deviation	1.5	0.9
Min, Max	0.0, 97.0	0.0, 36.0
(Missing)	136 (0.7%)	719 (5.1%)
Community participation, n (%)		
More than once a week	873 (4.3%)	600 (4.3%)
Once a week	1,011 (4.9%)	732 (5.2%)
One to three times a month	1,538 (7.5%)	1,087 (7.8%)
A few times a year	2,238 (10.9%)	1,535 (11.0%)
Never	14,819 (72.1%)	9,967 (71.4%)
(Missing)	65 (0.3%)	49 (0.3%)
Religious attendance, n (%)		

Table S19b. Weighted summary statistics for outcome variables in Japan

Outcome	Wave 1<br>N = 20,543	Wave 2<br>N = 13,969
More than once a week	315 (1.5%)	228 (1.6%)
Once a week	359 (1.7%)	293 (2.1%)
One to three times a month	878 (4.3%)	644 (4.6%)
A few times a year	3,131 (15.2%)	1,950 (14.0%)
Never	15,745 (76.6%)	10,789 (77.2%)
(Missing)	115 (0.6%)	66 (0.5%)
Loneliness		
Mean	4.0	4.2
Standard Deviation	2.7	2.7
Min, Max	0.0, 10.0	0.0, 10.0
(Missing)	38 (0.2%)	20 (0.1%)
Perceived discrimination, n (%)		
Always	431 (2.1%)	289 (2.1%)
Often	2,923 (14.2%)	2,140 (15.3%)
Rarely	9,251 (45.0%)	6,527 (46.7%)
Never	7,837 (38.1%)	4,989 (35.7%)
(Missing)	101 (0.5%)	24 (0.2%)
Orientation to promote good		
Mean	6.1	6.0
Standard Deviation	2.1	2.1
Min, Max	0.0, 10.0	0.0, 10.0
(Missing)	106 (0.5%)	54 (0.4%)
Delayed gratification		
Mean	5.2	5.1
Standard Deviation	2.0	2.0
Min, Max	0.0, 10.0	0.0, 10.0
(Missing)	78 (0.4%)	38 (0.3%)
Hope		
Mean	6.0	5.9
Standard Deviation	2.2	2.3
Min, Max	0.0, 10.0	0.0, 10.0
(Missing)	110 (0.5%)	63 (0.5%)
Gratitude		
Mean	5.8	5.9
Standard Deviation	2.2	2.3
Min, Max	0.0, 10.0	0.0, 10.0
(Missing)	122 (0.6%)	80 (0.6%)
Showing love/care		
Mean	6.0	6.0
Standard Deviation	2.2	2.2
Min, Max	0.0, 10.0	0.0, 10.0
(Missing)	43 (0.2%)	33 (0.2%)
Forgivingness, n (%)		
Always	1,609 (7.8%)	837 (6.0%)
Often	10,069 (49.0%)	6,111 (43.7%)
Rarely	7,051 (34.3%)	5,400 (38.7%)
Never	1,708 (8.3%)	1,563 (11.2%)
(Missing)	105 (0.5%)	58 (0.4%)
Charitable giving, n (%)		
Yes	1,962 (9.6%)	1,633 (11.7%)
No	18,455 (89.8%)	12,242 (87.6%)

Table S19b. Weighted summary statistics for outcome variables in Japan

Outcome	Wave 1<br>N = 20,543	Wave 2<br>N = 13,969
(Missing)	126 (0.6%)	95 (0.7%)
Helping strangers, n (%)		
Yes	2,384 (11.6%)	1,678 (12.0%)
No	18,126 (88.2%)	12,261 (87.8%)
(Missing)	34 (0.2%)	30 (0.2%)
Volunteering, n (%)		
Yes	1,810 (8.8%)	1,246 (8.9%)
No	18,619 (90.6%)	12,662 (90.6%)
(Missing)	114 (0.6%)	62 (0.4%)
Self-rated physical health		
Mean	6.0	5.8
Standard Deviation	2.1	2.1
Min, Max	0.0, 10.0	0.0, 10.0
(Missing)	60 (0.3%)	44 (0.3%)
Health problems, n (%)		
Yes	3,572 (17.4%)	2,604 (18.6%)
No	16,925 (82.4%)	11,330 (81.1%)
(Missing)	46 (0.2%)	35 (0.2%)
Pain in past 4 weeks, n (%)		
A lot	641 (3.1%)	489 (3.5%)
Some	6,101 (29.7%)	4,429 (31.7%)
Not very much	9,080 (44.2%)	6,054 (43.3%)
None at all	4,659 (22.7%)	2,953 (21.1%)
(Missing)	63 (0.3%)	44 (0.3%)
Number of cigarettes per day		
Mean	3.0	3.0
Standard Deviation	6.8	6.7
Min, Max	0.0, 97.0	0.0, 94.0
(Missing)	252 (1.2%)	87 (0.6%)
Number of drinks per week		
Mean	3.1	3.3
Standard Deviation	7.2	7.0
Min, Max	0.0, 97.0	0.0, 97.0
(Missing)	153 (0.7%)	85 (0.6%)
Days exercise per week		
Mean	2.0	2.0
Standard Deviation	2.4	2.4
Min, Max	0.0, 7.0	0.0, 7.0
(Missing)	38 (0.2%)	8 (<0.1%)
Financial security		
Mean	5.5	5.3
Standard Deviation	3.0	2.9
Min, Max	0.0, 10.0	0.0, 10.0
(Missing)	69 (0.3%)	21 (0.1%)
Material security		
Mean	5.9	5.7
Standard Deviation	2.7	2.6
Min, Max	0.0, 10.0	0.0, 10.0
(Missing)	81 (0.4%)	49 (0.4%)
Educational attainment (16+ years), n (%)		
Up to 8	570 (2.8%)	272 (1.9%)

Table S19b. Weighted summary statistics for outcome variables in Japan

Outcome	Wave 1<br>N = 20,543	Wave 2<br>N = 13,969
9-15	14,922 (72.6%)	10,225 (73.2%)
16+	5,051 (24.6%)	3,473 (24.9%)
(Missing)	0 (0%)	0 (0%)
Currently employed, n (%)		
Employed for an employer	11,102 (54.0%)	7,443 (53.3%)
Self-employed	1,738 (8.5%)	1,114 (8.0%)
Retired	2,460 (12.0%)	1,857 (13.3%)
Student	556 (2.7%)	234 (1.7%)
Homemaker	1,209 (5.9%)	807 (5.8%)
Unemployed and looking for a job	572 (2.8%)	427 (3.1%)
None of these/Other	2,873 (14.0%)	2,054 (14.7%)
(Missing)	34 (0.2%)	32 (0.2%)
Financially comfortable/getting by, n (%)		
Living comfortably on present income	4,329 (21.1%)	2,913 (20.9%)
Getting by on present income	11,772 (57.3%)	7,965 (57.0%)
Finding it difficult on present income	2,927 (14.2%)	2,040 (14.6%)
Finding it very difficult on present income	1,419 (6.9%)	1,019 (7.3%)
(Missing)	96 (0.5%)	32 (0.2%)
Own home, n (%)		
Someone in this household owns this home	11,020 (53.6%)	8,436 (60.4%)
Someone in this household rents this home	2,995 (14.6%)	2,762 (19.8%)
Both	392 (1.9%)	377 (2.7%)
Neither	6,007 (29.2%)	2,367 (16.9%)
Rent	0 (0%)	0 (0%)
Own	0 (0%)	0 (0%)
Something else	0 (0%)	0 (0%)
(Missing)	128 (0.6%)	26 (0.2%)
Religious/spiritual connection, n (%)		
Always	852 (4.1%)	657 (4.7%)
Often	3,843 (18.7%)	2,473 (17.7%)
Rarely	8,583 (41.8%)	5,162 (37.0%)
Never	7,224 (35.2%)	5,645 (40.4%)
(Missing)	41 (0.2%)	31 (0.2%)
Belief in life after death, n (%)		
Yes	4,230 (20.6%)	2,805 (20.1%)
No	6,688 (32.6%)	4,940 (35.4%)
Unsure	9,514 (46.3%)	6,153 (44.0%)
(Missing)	111 (0.5%)	71 (0.5%)
Transformative religious experience, n (%)		
Yes	1,610 (7.8%)	1,133 (8.1%)
No	18,843 (91.7%)	12,783 (91.5%)
(Missing)	90 (0.4%)	53 (0.4%)
Religious reading or listening, n (%)		
More than once a day	149 (0.7%)	88 (0.6%)
About once a day	588 (2.9%)	405 (2.9%)
Sometimes	2,876 (14.0%)	1,837 (13.2%)
Never	16,801 (81.8%)	11,585 (82.9%)
(Missing)	128 (0.6%)	53 (0.4%)
Prayer or meditation, n (%)		
More than once a day	530 (2.6%)	426 (3.0%)
About once a day	1,474 (7.2%)	1,045 (7.5%)

Table S19b. Weighted summary statistics for outcome variables in Japan

**Outcome**	**Wave 1**<br>N = 20,543	**Wave 2**<br>N = 13,969
Sometimes	4,307 (21.0%)	2,682 (19.2%)
Never	14,170 (69.0%)	9,781 (70.0%)
(Missing)	62 (0.3%)	36 (0.3%)
Belief in God/gods/spiritual forces, n (%)		
One God	569 (2.8%)	420 (3.0%)
More than one god	1,923 (9.4%)	1,433 (10.3%)
An impersonal spiritual force	1,621 (7.9%)	1,179 (8.4%)
None of these	10,112 (49.2%)	7,154 (51.2%)
Unsure	6,269 (30.5%)	3,743 (26.8%)
(Missing)	50 (0.2%)	40 (0.3%)
Religious centrality, n (%)		
Agree	1,527 (7.4%)	1,083 (7.8%)
Disagree	4,997 (24.3%)	3,192 (22.9%)
Not relevant	10,060 (49.0%)	7,130 (51.0%)
Unsure	3,876 (18.9%)	2,516 (18.0%)
(Missing)	84 (0.4%)	47 (0.3%)
Religious/spiritual comfort, n (%)		
Agree	1,481 (7.2%)	1,054 (7.5%)
Disagree	5,725 (27.9%)	3,614 (25.9%)
Not relevant	9,770 (47.6%)	7,085 (50.7%)
Unsure	3,427 (16.7%)	2,145 (15.4%)
(Missing)	139 (0.7%)	71 (0.5%)
Feel loved by God, n (%)		
Agree	2,137 (10.4%)	1,599 (11.4%)
Disagree	5,241 (25.5%)	3,443 (24.6%)
Not relevant	9,126 (44.4%)	6,382 (45.7%)
Unsure	3,841 (18.7%)	2,420 (17.3%)
(Missing)	199 (1.0%)	126 (0.9%)
Feel punished by God, n (%)		
Agree	521 (2.5%)	390 (2.8%)
Disagree	7,181 (35.0%)	4,850 (34.7%)
Not relevant	9,300 (45.3%)	6,552 (46.9%)
Unsure	3,358 (16.3%)	2,048 (14.7%)
(Missing)	183 (0.9%)	129 (0.9%)
Experienced religious criticism, n (%)		
Agree	273 (1.3%)	206 (1.5%)
Disagree	5,777 (28.1%)	3,653 (26.1%)
Not relevant	11,473 (55.8%)	8,203 (58.7%)
Unsure	2,855 (13.9%)	1,805 (12.9%)
(Missing)	165 (0.8%)	103 (0.7%)
Faith-sharing, n (%)		
Agree	796 (3.9%)	601 (4.3%)
Disagree	5,874 (28.6%)	3,864 (27.7%)
Not relevant	10,901 (53.1%)	7,699 (55.1%)
Unsure	2,861 (13.9%)	1,761 (12.6%)
(Missing)	112 (0.5%)	44 (0.3%)

Note. N (%); this table is based on non-imputed data. Cumulative percentages for variables may not add up to 100% due to rounding. Wave 1 characteristics weighted using the Gallup provided sampling weight, ANNUAL_WEIGHT_R2; Wave 2 characteristics weighted accounting for attrition by using the adjusted Wave 1 weight, ANNUAL_WEIGHT_R2, multiplied by the created attrition weight to account for dropout, to maintain nationally representative estimates for Wave 2 characteristics.

Table S19c. Unweighted summary statistics for demographic and childhood variables in Japan by retention status

Characteristic	Attritors–Not Observed in Wave 2<br>N = 6,653	Retained–Observed in Wave 2<br>N = 13,953
Forgivingness, n (%)		
Always	603 (9.1%)	1,007 (7.2%)
Often	3,250 (48.8%)	6,852 (49.1%)
Rarely	2,177 (32.7%)	4,900 (35.1%)
Never	573 (8.6%)	1,140 (8.2%)
(Missing)	50 (0.8%)	55 (0.4%)
Year of birth, n (%)		
1943 or earlier (current age: 80+ years)	138 (2.1%)	355 (2.5%)
1943-1953 (current age: 70-79 years)	843 (12.7%)	2,677 (19.2%)
1953-1963 (current age: 60-69 years)	1,028 (15.4%)	2,976 (21.3%)
1963-1973 (current age: 50-59 years)	852 (12.8%)	2,793 (20.0%)
1973-1983 (current age: 40-49 years)	962 (14.5%)	2,442 (17.5%)
1983-1993 (current age: 30-39 years)	1,219 (18.3%)	1,686 (12.1%)
1993-1998 (current age: 25-29 years)	434 (6.5%)	377 (2.7%)
1998-2005 (current age: 18-24 years)	1,177 (17.7%)	647 (4.6%)
(Missing)	0 (0%)	0 (0%)
Age of participant		
Mean	46.3	54.7
Standard Deviation	18.7	15.9
Min, Max	18.0, 99.0	18.0, 99.0
Gender, n (%)		
Male	2,987 (44.9%)	6,822 (48.9%)
Female	3,618 (54.4%)	7,085 (50.8%)
Other	14 (0.2%)	14 (0.1%)
(Missing)	34 (0.5%)	33 (0.2%)
Respondent marital status, n (%)		
Single/Never been married	2,080 (31.3%)	2,850 (20.4%)
Married	3,419 (51.4%)	8,486 (60.8%)
Separated	75 (1.1%)	121 (0.9%)
Divorced	604 (9.1%)	1,546 (11.1%)
Widowed	377 (5.7%)	831 (6.0%)
Domestic partner	83 (1.2%)	72 (0.5%)
(Missing)	15 (0.2%)	48 (0.3%)
Education (years), n (%)		
Up to 8	201 (3.0%)	370 (2.7%)
9-15	4,612 (69.3%)	10,365 (74.3%)
16+	1,841 (27.7%)	3,218 (23.1%)
(Missing)	0 (0%)	0 (0%)
Employment status, n (%)		
Employed for an employer	3,757 (56.5%)	7,373 (52.8%)
Self-employed	510 (7.7%)	1,235 (8.9%)
Retired	537 (8.1%)	1,941 (13.9%)
Student	374 (5.6%)	175 (1.3%)
Homemaker	386 (5.8%)	827 (5.9%)
Unemployed and looking for a job	199 (3.0%)	374 (2.7%)
None of these/Other	875 (13.2%)	2,008 (14.4%)
(Missing)	16 (0.2%)	18 (0.1%)
Current religious service attendance, n (%)		
More than once a week	106 (1.6%)	210 (1.5%)

Table S19c. Unweighted summary statistics for demographic and childhood variables in Japan by retention status

**Characteristic**	**Attritors–Not Observed in Wave 2**<br>N = 6,653	**Retained–Observed in Wave 2**<br>N = 13,953
Once a week	157 (2.4%)	201 (1.4%)
One to three times a month	353 (5.3%)	526 (3.8%)
A few times a year	1,032 (15.5%)	2,107 (15.1%)
Never	4,972 (74.7%)	10,827 (77.6%)
(Missing)	34 (0.5%)	82 (0.6%)
Immigration status, n (%)		
Born in this country	6,217 (93.4%)	13,388 (95.9%)
Born in another country	71 (1.1%)	90 (0.6%)
(Missing)	365 (5.5%)	475 (3.4%)
Parental marital status around age 12, n (%)		
Parents were married	5,540 (83.3%)	12,181 (87.3%)
Parents were divorced	451 (6.8%)	708 (5.1%)
Parents were never married	244 (3.7%)	356 (2.6%)
One or both of them had died	248 (3.7%)	511 (3.7%)
Unsure	145 (2.2%)	174 (1.2%)
(Missing)	26 (0.4%)	24 (0.2%)
Religious service attendance around age 12, n (%)		
At least once a week	130 (1.9%)	271 (1.9%)
One to three times a month	332 (5.0%)	560 (4.0%)
Less than once a month	1,675 (25.2%)	3,371 (24.2%)
Never	4,465 (67.1%)	9,681 (69.4%)
(Missing)	52 (0.8%)	70 (0.5%)
Relationship with mother when growing up, n (%)		
Very good	1,798 (27.0%)	3,860 (27.7%)
Somewhat good	2,937 (44.2%)	6,512 (46.7%)
Somewhat bad	972 (14.6%)	1,798 (12.9%)
Very bad	268 (4.0%)	535 (3.8%)
(Does not apply)	652 (9.8%)	1,208 (8.7%)
(Missing)	25 (0.4%)	41 (0.3%)
Relationship with father when growing up, n (%)		
Very good	1,358 (20.4%)	2,820 (20.2%)
Somewhat good	2,847 (42.8%)	6,249 (44.8%)
Somewhat bad	1,118 (16.8%)	2,318 (16.6%)
Very bad	381 (5.7%)	851 (6.1%)
(Does not apply)	927 (13.9%)	1,679 (12.0%)
(Missing)	21 (0.3%)	36 (0.3%)
Felt like an outsider in family when growing up, n (%)		
Yes	721 (10.8%)	1,262 (9.0%)
No	5,420 (81.5%)	11,748 (84.2%)
(Missing)	512 (7.7%)	943 (6.8%)
Experienced abuse when growing up, n (%)		
Yes	527 (7.9%)	967 (6.9%)
No	6,091 (91.6%)	12,925 (92.6%)
(Missing)	35 (0.5%)	62 (0.4%)
Self-rated health when growing up, n (%)		
Excellent	988 (14.9%)	1,774 (12.7%)
Very good	2,211 (33.2%)	4,924 (35.3%)

Table S19c. Unweighted summary statistics for demographic and childhood variables in Japan by retention status

**Characteristic**	**Attritors–Not Observed in Wave 2** N = 6,653	**Retained–Observed in Wave 2** N = 13,953
Good	2,127 (32.0%)	4,563 (32.7%)
Fair	1,033 (15.5%)	2,154 (15.4%)
Poor	273 (4.1%)	482 (3.5%)
(Missing)	21 (0.3%)	57 (0.4%)
Subjective financial status of family growing up, n (%)		
Lived comfortably	2,930 (44.0%)	5,440 (39.0%)
Got by	2,718 (40.9%)	6,081 (43.6%)
Found it difficult	714 (10.7%)	1,693 (12.1%)
Found it very difficult	276 (4.1%)	703 (5.0%)
(Missing)	15 (0.2%)	37 (0.3%)
Religious affiliation growing up, n (%)		
Christianity	121 (1.8%)	226 (1.6%)
Taoism	9 (0.1%)	6 (0.0%)
Confucianism	9 (0.1%)	16 (0.1%)
Primal, Animist, or Folk religion	9 (0.1%)	4 (0.0%)
Spiritism	0 (0%)	0 (0%)
Umbanda, Candomblé, and other African-derived religions	0 (0%)	0 (0%)
Chinese folk/traditional religion	0 (0%)	0 (0%)
Islam	9 (0.1%)	0 (0%)
Hinduism	1 (0.0%)	3 (0.0%)
Buddhism	1,896 (28.5%)	4,619 (33.1%)
Judaism	0 (0%)	0 (0%)
Sikhism	0 (0%)	0 (0%)
Baha'i	4 (0.1%)	3 (0.0%)
Jainism	0 (<0.0%)	1 (<0.0%)
Shinto	138 (2.1%)	244 (1.7%)
Some other religion	19 (0.3%)	28 (0.2%)
No religion/Atheist/Agnostic	4,343 (65.3%)	8,681 (62.2%)
(Missing)	95 (1.4%)	122 (0.9%)

Note. N (%); this table is based on non-imputed data. Cumulative percentages for variables may not add up to 100% due to rounding.

Table S19d. Unweighted summary statistics for Wave 1 outcome variables in Japan by retention status.

**Outcome**	**Attritors–Not Observed in Wave 2** N = 6,653	**Retained–Observed in Wave 2** N = 13,953
Secure flourishing index		
Mean	5.9	5.9
Standard Deviation	1.8	1.8
Min, Max	0.0, 10.0	0.0, 10.0
(Missing)	255 (3.8%)	677 (4.8%)
Flourishing index		
Mean	6.0	5.9
Standard Deviation	1.8	1.8
Min, Max	0.0, 10.0	0.0, 10.0
(Missing)	219 (3.3%)	603 (4.3%)
Happiness & life satisfaction		
Mean	6.2	6.1
Standard Deviation	2.1	2.1
Min, Max	0.0, 10.0	0.0, 10.0
(Missing)	48 (0.7%)	143 (1.0%)
Physical & mental health		
Mean	6.1	6.1
Standard Deviation	2.1	2.0
Min, Max	0.0, 10.0	0.0, 10.0
(Missing)	24 (0.4%)	64 (0.5%)
Meaning & purpose		
Mean	5.8	5.8
Standard Deviation	2.1	2.1
Min, Max	0.0, 10.0	0.0, 10.0
(Missing)	53 (0.8%)	133 (1.0%)
Character & virtue		
Mean	5.6	5.7
Standard Deviation	1.8	1.7
Min, Max	0.0, 10.0	0.0, 10.0
(Missing)	45 (0.7%)	123 (0.9%)
Close social relationships		
Mean	6.0	6.0
Standard Deviation	2.2	2.2
Min, Max	0.0, 10.0	0.0, 10.0
(Missing)	81 (1.2%)	221 (1.6%)
Financial & material security		
Mean	5.6	5.8
Standard Deviation	2.7	2.7
Min, Max	0.0, 10.0	0.0, 10.0
(Missing)	50 (0.8%)	92 (0.7%)
Happiness		
Mean	6.3	6.2
Standard Deviation	2.1	2.1
Min, Max	0.0, 10.0	0.0, 10.0
(Missing)	22 (0.3%)	54 (0.4%)
Life satisfaction		
Mean	6.1	6.1
Standard Deviation	2.3	2.3
Min, Max	0.0, 10.0	0.0, 10.0

Table S19d. Unweighted summary statistics for Wave 1 outcome variables in Japan by retention status.

Outcome	Attritors–Not Observed in Wave 2<br>N = 6,653	Retained–Observed in Wave 2<br>N = 13,953
(Missing)	29 (0.4%)	94 (0.7%)
Current life evaluation		
Mean	5.9	5.9
Standard Deviation	2.1	2.1
Min, Max	0.0, 10.0	0.0, 10.0
(Missing)	26 (0.4%)	54 (0.4%)
Future life evaluation		
Mean	6.2	6.0
Standard Deviation	2.2	2.2
Min, Max	0.0, 10.0	0.0, 10.0
(Missing)	39 (0.6%)	45 (0.3%)
Optimism		
Mean	6.7	6.8
Standard Deviation	2.3	2.2
Min, Max	0.0, 10.0	0.0, 10.0
(Missing)	15 (0.2%)	32 (0.2%)
Freedom to pursue what's important		
Mean	6.4	6.4
Standard Deviation	2.2	2.1
Min, Max	0.0, 10.0	0.0, 10.0
(Missing)	22 (0.3%)	47 (0.3%)
Inner peace, n (%)		
Always	731 (11.0%)	1,652 (11.8%)
Often	3,744 (56.3%)	7,814 (56.0%)
Rarely	1,790 (26.9%)	3,771 (27.0%)
Never	355 (5.3%)	646 (4.6%)
(Missing)	34 (0.5%)	72 (0.5%)
Life balance, n (%)		
Always	840 (12.6%)	1,957 (14.0%)
Often	3,937 (59.2%)	8,109 (58.1%)
Rarely	1,521 (22.9%)	3,182 (22.8%)
Never	328 (4.9%)	637 (4.6%)
(Missing)	27 (0.4%)	69 (0.5%)
Sense of mastery, n (%)		
Always	244 (3.7%)	364 (2.6%)
Often	2,361 (35.5%)	5,098 (36.5%)
Rarely	3,161 (47.5%)	6,741 (48.3%)
Never	841 (12.6%)	1,630 (11.7%)
(Missing)	46 (0.7%)	120 (0.9%)
Meaningful activities		
Mean	6.0	6.0
Standard Deviation	2.2	2.2
Min, Max	0.0, 10.0	0.0, 10.0
(Missing)	27 (0.4%)	64 (0.5%)
Understanding purpose		
Mean	5.7	5.7
Standard Deviation	2.3	2.3
Min, Max	0.0, 10.0	0.0, 10.0
(Missing)	28 (0.4%)	73 (0.5%)

Table S19d. Unweighted summary statistics for Wave 1 outcome variables in Japan by retention status.

**Outcome**	**Attritors–Not Observed in Wave 2**<br>N = 6,653	**Retained–Observed in Wave 2**<br>N = 13,953
Self-rated mental health		
Mean	6.2	6.3
Standard Deviation	2.3	2.2
Min, Max	0.0, 10.0	0.0, 10.0
(Missing)	8 (0.1%)	25 (0.2%)
Traumatic distress, n (%)		
A lot	484 (7.3%)	730 (5.2%)
Some	1,606 (24.1%)	3,031 (21.7%)
Not very much	2,783 (41.8%)	6,271 (44.9%)
Not at all	1,749 (26.3%)	3,886 (27.8%)
(Missing)	31 (0.5%)	36 (0.3%)
Depression symptoms composite, n (%)	1,539 (23.3%)	2,860 (20.7%)
(Missing)	58 (0.9%)	130 (0.9%)
Depression – feel hopeless, n (%)		
Nearly every day	432 (6.5%)	710 (5.1%)
More than half the days	818 (12.3%)	1,490 (10.7%)
Several days	2,116 (31.8%)	3,948 (28.3%)
Not at all	3,257 (49.0%)	7,731 (55.4%)
(Missing)	30 (0.5%)	74 (0.5%)
Depression – loss of interest, n (%)		
Nearly every day	403 (6.1%)	802 (5.7%)
More than half the days	1,058 (15.9%)	1,988 (14.2%)
Several days	1,942 (29.2%)	3,827 (27.4%)
Not at all	3,220 (48.4%)	7,275 (52.1%)
(Missing)	30 (0.5%)	62 (0.4%)
Anxiety symptoms composite, n (%)	1,450 (22.0%)	2,367 (17.1%)
(Missing)	49 (0.7%)	125 (0.9%)
Anxiety – feel on edge, n (%)		
Nearly every day	522 (7.8%)	847 (6.1%)
More than half the days	937 (14.1%)	1,616 (11.6%)
Several days	2,496 (37.5%)	4,869 (34.9%)
Not at all	2,664 (40.0%)	6,528 (46.8%)
(Missing)	35 (0.5%)	93 (0.7%)
Anxiety – cannot stop worrying, n (%)		
Nearly every day	348 (5.2%)	472 (3.4%)
More than half the days	712 (10.7%)	1,133 (8.1%)
Several days	1,669 (25.1%)	2,944 (21.1%)
Not at all	3,908 (58.7%)	9,366 (67.1%)
(Missing)	17 (0.2%)	38 (0.3%)
Suffering, n (%)		
A lot	445 (6.7%)	846 (6.1%)
Some	2,053 (30.9%)	4,233 (30.3%)
Not very much	3,090 (46.4%)	6,625 (47.5%)
Not at all	1,045 (15.7%)	2,189 (15.7%)
(Missing)	21 (0.3%)	60 (0.4%)
Relationship contentment		
Mean	6.0	6.0
Standard Deviation	2.3	2.3
Min, Max	0.0, 10.0	0.0, 10.0

Table S19d. Unweighted summary statistics for Wave 1 outcome variables in Japan by retention status.

Outcome	Attritors–Not Observed in Wave 2<br>N = 6,653	Retained–Observed in Wave 2<br>N = 13,953
(Missing)	41 (0.6%)	99 (0.7%)
Relationship satisfaction		
Mean	6.0	5.9
Standard Deviation	2.3	2.2
Min, Max	0.0, 10.0	0.0, 10.0
(Missing)	46 (0.7%)	133 (1.0%)
Social support		
Mean	5.5	5.2
Standard Deviation	2.4	2.4
Min, Max	0.0, 10.0	0.0, 10.0
(Missing)	23 (0.4%)	56 (0.4%)
Intimate/close friend, n (%)		
Yes	4,590 (69.0%)	9,264 (66.4%)
No	2,018 (30.3%)	4,626 (33.2%)
(Missing)	45 (0.7%)	64 (0.5%)
Government approval, n (%)		
Strongly approve	101 (1.5%)	150 (1.1%)
Somewhat approve	1,142 (17.2%)	2,572 (18.4%)
Neither approve nor disapprove	2,906 (43.7%)	5,563 (39.9%)
Somewhat disapprove	1,607 (24.2%)	3,822 (27.4%)
Strongly disapprove	864 (13.0%)	1,782 (12.8%)
(Missing)	33 (0.5%)	64 (0.5%)
Say in government, n (%)		
Agree	1,344 (20.2%)	3,122 (22.4%)
Disagree	2,646 (39.8%)	5,507 (39.5%)
Unsure	2,632 (39.6%)	5,274 (37.8%)
(Missing)	31 (0.5%)	51 (0.4%)
Belonging in country		
Mean	6.0	6.1
Standard Deviation	2.3	2.3
Min, Max	0.0, 10.0	0.0, 10.0
(Missing)	41 (0.6%)	78 (0.6%)
City/place satisfaction, n (%)		
Satisfied	3,601 (54.1%)	7,915 (56.7%)
Dissatisfied	1,174 (17.6%)	2,485 (17.8%)
Unsure	1,824 (27.4%)	3,446 (24.7%)
(Missing)	55 (0.8%)	106 (0.8%)
Trust within country, n (%)		
All people	61 (0.9%)	71 (0.5%)
Most people	1,708 (25.7%)	4,271 (30.6%)
Some people	2,964 (44.5%)	5,892 (42.2%)
Not very many people	1,582 (23.8%)	3,127 (22.4%)
None	303 (4.6%)	519 (3.7%)
(Missing)	35 (0.5%)	74 (0.5%)
Number of children		
Mean	0.5	0.4
Standard Deviation	1.2	1.6
Min, Max	0.0, 21.0	0.0, 97.0
(Missing)	50 (0.8%)	86 (0.6%)

Table S19d. Unweighted summary statistics for Wave 1 outcome variables in Japan by retention status.

**Outcome**	**Attritors–Not Observed in Wave 2**<br>N = 6,653	**Retained–Observed in Wave 2**<br>N = 13,953
Community participation, n (%)		
More than once a week	305 (4.6%)	570 (4.1%)
Once a week	386 (5.8%)	625 (4.5%)
One to three times a month	540 (8.1%)	1,001 (7.2%)
A few times a year	759 (11.4%)	1,484 (10.6%)
Never	4,642 (69.8%)	10,229 (73.3%)
(Missing)	21 (0.3%)	44 (0.3%)
Religious attendance, n (%)		
More than once a week	106 (1.6%)	210 (1.5%)
Once a week	157 (2.4%)	201 (1.4%)
One to three times a month	353 (5.3%)	526 (3.8%)
A few times a year	1,032 (15.5%)	2,107 (15.1%)
Never	4,972 (74.7%)	10,827 (77.6%)
(Missing)	34 (0.5%)	82 (0.6%)
Loneliness		
Mean	4.1	4.0
Standard Deviation	2.6	2.7
Min, Max	0.0, 10.0	0.0, 10.0
(Missing)	7 (<0.1%)	32 (0.2%)
Perceived discrimination, n (%)		
Always	180 (2.7%)	251 (1.8%)
Often	1,010 (15.2%)	1,919 (13.8%)
Rarely	2,902 (43.6%)	6,381 (45.7%)
Never	2,526 (38.0%)	5,336 (38.2%)
(Missing)	35 (0.5%)	66 (0.5%)
Orientation to promote good		
Mean	6.1	6.1
Standard Deviation	2.1	2.0
Min, Max	0.0, 10.0	0.0, 10.0
(Missing)	25 (0.4%)	82 (0.6%)
Delayed gratification		
Mean	5.2	5.2
Standard Deviation	2.1	2.0
Min, Max	0.0, 10.0	0.0, 10.0
(Missing)	28 (0.4%)	51 (0.4%)
Hope		
Mean	6.0	5.9
Standard Deviation	2.2	2.2
Min, Max	0.0, 10.0	0.0, 10.0
(Missing)	35 (0.5%)	76 (0.5%)
Gratitude		
Mean	5.9	5.8
Standard Deviation	2.3	2.2
Min, Max	0.0, 10.0	0.0, 10.0
(Missing)	39 (0.6%)	84 (0.6%)
Showing love/care		
Mean	6.0	5.9
Standard Deviation	2.2	2.2
Min, Max	0.0, 10.0	0.0, 10.0

Table S19d. Unweighted summary statistics for Wave 1 outcome variables in Japan by retention status.

**Outcome**	**Attritors–Not Observed in Wave 2**<br>N = 6,653	**Retained–Observed in Wave 2**<br>N = 13,953
(Missing)	13 (0.2%)	31 (0.2%)
Forgivingness, n (%)		
Always	603 (9.1%)	1,007 (7.2%)
Often	3,250 (48.8%)	6,852 (49.1%)
Rarely	2,177 (32.7%)	4,900 (35.1%)
Never	573 (8.6%)	1,140 (8.2%)
(Missing)	50 (0.8%)	55 (0.4%)
Charitable giving, n (%)		
Yes	672 (10.1%)	1,295 (9.3%)
No	5,944 (89.3%)	12,569 (90.1%)
(Missing)	37 (0.6%)	90 (0.6%)
Helping strangers, n (%)		
Yes	880 (13.2%)	1,506 (10.8%)
No	5,762 (86.6%)	12,424 (89.0%)
(Missing)	11 (0.2%)	23 (0.2%)
Volunteering, n (%)		
Yes	613 (9.2%)	1,202 (8.6%)
No	6,005 (90.3%)	12,672 (90.8%)
(Missing)	35 (0.5%)	79 (0.6%)
Self-rated physical health		
Mean	6.0	6.0
Standard Deviation	2.1	2.1
Min, Max	0.0, 10.0	0.0, 10.0
(Missing)	17 (0.3%)	43 (0.3%)
Health problems, n (%)		
Yes	1,179 (17.7%)	2,403 (17.2%)
No	5,462 (82.1%)	11,516 (82.5%)
(Missing)	12 (0.2%)	35 (0.2%)
Pain in past 4 weeks, n (%)		
A lot	224 (3.4%)	419 (3.0%)
Some	1,917 (28.8%)	4,204 (30.1%)
Not very much	2,957 (44.4%)	6,150 (44.1%)
None at all	1,539 (23.1%)	3,133 (22.5%)
(Missing)	16 (0.2%)	47 (0.3%)
Number of cigarettes per day		
Mean	2.6	3.2
Standard Deviation	6.4	7.0
Min, Max	0.0, 97.0	0.0, 80.0
(Missing)	81 (1.2%)	172 (1.2%)
Number of drinks per week		
Mean	2.8	3.3
Standard Deviation	7.3	7.2
Min, Max	0.0, 97.0	0.0, 97.0
(Missing)	50 (0.8%)	103 (0.7%)
Days exercise per week		
Mean	2.0	2.1
Standard Deviation	2.4	2.5
Min, Max	0.0, 7.0	0.0, 7.0
(Missing)	14 (0.2%)	24 (0.2%)

Table S19d. Unweighted summary statistics for Wave 1 outcome variables in Japan by retention status.

**Outcome**	**Attritors–Not Observed in Wave 2** N = 6,653	**Retained–Observed in Wave 2** N = 13,953
Financial security		
Mean	5.4	5.6
Standard Deviation	3.0	3.0
Min, Max	0.0, 10.0	0.0, 10.0
(Missing)	26 (0.4%)	43 (0.3%)
Material security		
Mean	5.8	5.9
Standard Deviation	2.7	2.6
Min, Max	0.0, 10.0	0.0, 10.0
(Missing)	29 (0.4%)	51 (0.4%)
Educational attainment (16+ years), n (%)		
Up to 8	201 (3.0%)	370 (2.7%)
9-15	4,612 (69.3%)	10,365 (74.3%)
16+	1,841 (27.7%)	3,218 (23.1%)
(Missing)	0 (0%)	0 (0%)
Currently employed, n (%)		
Employed for an employer	3,757 (56.5%)	7,373 (52.8%)
Self-employed	510 (7.7%)	1,235 (8.9%)
Retired	537 (8.1%)	1,941 (13.9%)
Student	374 (5.6%)	175 (1.3%)
Homemaker	386 (5.8%)	827 (5.9%)
Unemployed and looking for a job	199 (3.0%)	374 (2.7%)
None of these/Other	875 (13.2%)	2,008 (14.4%)
(Missing)	16 (0.2%)	18 (0.1%)
Financially comfortable/getting by, n (%)		
Living comfortably on present income	1,492 (22.4%)	2,847 (20.4%)
Getting by on present income	3,682 (55.3%)	8,132 (58.3%)
Finding it difficult on present income	931 (14.0%)	2,006 (14.4%)
Finding it very difficult on present income	504 (7.6%)	917 (6.6%)
(Missing)	44 (0.7%)	52 (0.4%)
Own home, n (%)		
Someone in this household owns this home	3,224 (48.5%)	7,844 (56.2%)
Someone in this household rents this home	1,099 (16.5%)	1,901 (13.6%)
Both	158 (2.4%)	234 (1.7%)
Neither	2,115 (31.8%)	3,904 (28.0%)
Rent	0 (0%)	0 (0%)
Own	0 (0%)	0 (0%)
Something else	0 (0%)	0 (0%)
(Missing)	57 (0.8%)	71 (0.5%)
Religious/spiritual connection, n (%)		
Always	323 (4.9%)	530 (3.8%)
Often	1,270 (19.1%)	2,584 (18.5%)
Rarely	2,699 (40.6%)	5,914 (42.4%)
Never	2,345 (35.3%)	4,901 (35.1%)
(Missing)	17 (0.3%)	24 (0.2%)
Belief in life after death, n (%)		
Yes	1,428 (21.5%)	2,813 (20.2%)
No	1,988 (29.9%)	4,728 (33.9%)
Unsure	3,201 (48.1%)	6,338 (45.4%)

Table S19d. Unweighted summary statistics for Wave 1 outcome variables in Japan by retention status.

**Outcome**	**Attritors–Not Observed in Wave 2** N = 6,653	**Retained–Observed in Wave 2** N = 13,953
(Missing)	37 (0.6%)	74 (0.5%)
Transformative religious experience, n (%)		
Yes	576 (8.7%)	1,038 (7.4%)
No	6,041 (90.8%)	12,862 (92.2%)
(Missing)	36 (0.5%)	54 (0.4%)
Religious reading or listening, n (%)		
More than once a day	71 (1.1%)	78 (0.6%)
About once a day	255 (3.8%)	332 (2.4%)
Sometimes	947 (14.2%)	1,937 (13.9%)
Never	5,330 (80.1%)	11,527 (82.6%)
(Missing)	50 (0.7%)	79 (0.6%)
Prayer or meditation, n (%)		
More than once a day	168 (2.5%)	363 (2.6%)
About once a day	514 (7.7%)	963 (6.9%)
Sometimes	1,361 (20.5%)	2,961 (21.2%)
Never	4,586 (68.9%)	9,629 (69.0%)
(Missing)	24 (0.4%)	37 (0.3%)
Belief in God/gods/spiritual forces, n (%)		
One God	174 (2.6%)	397 (2.8%)
More than one god	677 (10.2%)	1,250 (9.0%)
An impersonal spiritual force	602 (9.0%)	1,021 (7.3%)
None of these	3,042 (45.7%)	7,111 (51.0%)
Unsure	2,136 (32.1%)	4,148 (29.7%)
(Missing)	23 (0.3%)	27 (0.2%)
Religious centrality, n (%)		
Agree	449 (6.8%)	1,084 (7.8%)
Disagree	1,582 (23.8%)	3,432 (24.6%)
Not relevant	3,193 (48.0%)	6,900 (49.5%)
Unsure	1,396 (21.0%)	2,486 (17.8%)
(Missing)	33 (0.5%)	51 (0.4%)
Religious/spiritual comfort, n (%)		
Agree	465 (7.0%)	1,022 (7.3%)
Disagree	1,833 (27.5%)	3,911 (28.0%)
Not relevant	3,122 (46.9%)	6,680 (47.9%)
Unsure	1,191 (17.9%)	2,244 (16.1%)
(Missing)	43 (0.6%)	96 (0.7%)
Feel loved by God, n (%)		
Agree	679 (10.2%)	1,466 (10.5%)
Disagree	1,650 (24.8%)	3,609 (25.9%)
Not relevant	2,924 (43.9%)	6,231 (44.7%)
Unsure	1,343 (20.2%)	2,506 (18.0%)
(Missing)	59 (0.9%)	141 (1.0%)
Feel punished by God, n (%)		
Agree	184 (2.8%)	337 (2.4%)
Disagree	2,214 (33.3%)	4,993 (35.8%)
Not relevant	2,993 (45.0%)	6,337 (45.4%)
Unsure	1,201 (18.1%)	2,163 (15.5%)
(Missing)	61 (0.9%)	123 (0.9%)
Experienced religious criticism, n (%)		

Table S19d. Unweighted summary statistics for Wave 1 outcome variables in Japan by retention status.

Outcome	Attritors–Not Observed in Wave 2<br>N = 6,653	Retained–Observed in Wave 2<br>N = 13,953
Agree	116 (1.7%)	157 (1.1%)
Disagree	1,767 (26.6%)	4,031 (28.9%)
Not relevant	3,684 (55.4%)	7,825 (56.1%)
Unsure	1,032 (15.5%)	1,828 (13.1%)
(Missing)	54 (0.8%)	112 (0.8%)
Faith-sharing, n (%)		
Agree	270 (4.1%)	527 (3.8%)
Disagree	1,826 (27.4%)	4,069 (29.2%)
Not relevant	3,516 (52.8%)	7,419 (53.2%)
Unsure	1,000 (15.0%)	1,867 (13.4%)
(Missing)	41 (0.6%)	71 (0.5%)

Note. N (%); this table is based on non-imputed data. Cumulative percentages for variables may not add up to 100% due to rounding.

Table S19e. Summary of fitted attrition model in Japan

Characteristic	Odds Ratio	95% CI	p-value
**ANNUAL_WEIGHT_R2**	0.92	0.86, 0.98	0.011
**Recruitment Survey Mode**			
CAWI	—	—	
CATI	0.55	0.44, 0.68	8.65e-08
**Happiness & life satisfaction**	0.87	0.81, 0.94	1.85e-04
**Physical & mental health**	1.08	1.02, 1.16	0.012
**Meaning & purpose**	0.98	0.91, 1.05	0.604
**Character & virtue**	1.02	0.96, 1.07	0.571
**Close social relationships**	0.98	0.92, 1.04	0.498
**Financial & material security**	1.05	1.00, 1.11	0.036
**Extraversion**	0.96	0.92, 1.00	0.048
**Openness to experience**	0.99	0.95, 1.03	0.517
**Agreeableness**	1.03	0.99, 1.08	0.136
**Conscientiousness**	1.03	0.98, 1.08	0.199
**Neuroticism**	0.98	0.93, 1.03	0.335
**Depression symptoms composite**	1.00	0.95, 1.06	0.858
**Anxiety symptoms composite**	0.97	0.92, 1.02	0.185
**Loneliness**	0.98	0.93, 1.03	0.411
**Days exercise per week**	0.99	0.95, 1.03	0.696
**Year of birth (age group)**			
1953-1963 (current age: 60-69 years)	—	—	
1963-1973 (current age: 50-59 years)	1.12	0.98, 1.27	0.097
1943-1953 (current age: 70-79 years)	1.12	0.99, 1.27	0.080
1973-1983 (current age: 40-49 years)	0.90	0.78, 1.03	0.132
1983-1993 (current age: 30-39 years)	0.49	0.42, 0.57	2.28e-22
1998-2005 (current age: 18-24 years)	0.22	0.18, 0.26	5.74e-49
1993-1998 (current age: 25-29 years)	0.31	0.25, 0.38	4.57e-30
1943 or earlier (current age: 80+ years)	1.02	0.78, 1.33	0.881
**Gender of respondent**			
Female	—	—	
Male	1.15	1.05, 1.25	0.001
Other	0.79	0.35, 1.75	0.555
**Marital status**			
Married	—	—	
Single/Never been married	1.06	0.95, 1.18	0.301
Divorced	0.99	0.86, 1.14	0.880
Widowed	0.83	0.69, 1.01	0.070
Separated	0.82	0.56, 1.22	0.331
Domestic partner	0.64	0.43, 0.97	0.037
**Employment status**			
Employed for an employer	—	—	
None of these/Other	0.92	0.82, 1.04	0.177
Retired	1.19	1.03, 1.38	0.018
Homemaker	0.88	0.76, 1.03	0.118
Self-employed	1.00	0.86, 1.16	0.988
Unemployed and looking for a job	1.01	0.84, 1.22	0.911
Student	0.73	0.56, 0.94	0.016
**Religious attendance**			
Never	—	—	
A few times a year	0.96	0.86, 1.07	0.463
One to three times a month	0.82	0.69, 0.98	0.032
More than once a week	0.84	0.63, 1.11	0.211

Table S19e. Summary of fitted attrition model in Japan

**Characteristic**	**Odds Ratio**	**95% CI**	**p-value**
Once a week	0.78	0.58, 1.05	0.097
**Educational attainment (16+ years)**			
9-15	—	—	
16+	0.95	0.86, 1.04	0.242
Up to 8	1.01	0.80, 1.27	0.951
**Born in This country**			
Born in this country	—	—	
Born in another country	0.89	0.59, 1.35	0.591
**Urbanicity**			
A small town or village	—	—	
A suburb of a large city	1.03	0.94, 1.13	0.471
A large city	1.08	0.98, 1.20	0.113
A rural area or on a farm	0.96	0.82, 1.12	0.563
**Monthly household income**			
Japan: 300,001 – 400,000 yen	—	—	
Japan: 200,001 – 250,000 yen	0.89	0.76, 1.03	0.125
Japan: 250,001 – 300,000 yen	0.91	0.78, 1.06	0.203
Japan: 150,001 – 200,000 yen	0.83	0.71, 0.97	0.020
(None/No household income)	0.72	0.60, 0.85	1.37e-04
Japan: 100,001 – 150,000 yen	0.97	0.81, 1.16	0.749
Japan: 500,001 – 600,000 yen	1.06	0.88, 1.27	0.544
Japan: 400,001 – 450,000 yen	0.87	0.72, 1.04	0.115
Japan: 450,001 – 500,000 yen	0.98	0.81, 1.19	0.839
Japan: More than 1,000,000 yen	0.81	0.67, 0.99	0.038
Japan: 50,001 – 100,000 yen	0.85	0.68, 1.05	0.135
Japan: 600,001 – 700,000 yen	0.91	0.74, 1.12	0.380
Japan: 50,000 yen or less	0.77	0.61, 0.99	0.041
Japan: 700,001 – 800,000 yen	1.11	0.87, 1.42	0.411
Japan: 800,001 – 900,000 yen	0.84	0.64, 1.10	0.199
Japan: 900,001 – 1,000,000 yen	0.70	0.52, 0.94	0.019

Abbreviations: CI = Confidence Interval, OR = Odds Ratio

Notes. N=20543; attrition weights were estimated using the 'survey::svyglm(family=quasibinomial('logit'))' function. All continuous predictors were standardized and all categorical predictors used the most common category as the reference group. Reported p-values are based on the fitted regression model and no adjustments for multiple testing were done within this table.

Table S19f. Summary of principal components in Japan

PC	Percent Explained by each PC	Cumulative Percent Explained
1	29.13	29.13
2	7.82	36.95
3	6.42	43.37
4	2.76	46.13
5	1.99	48.12
6	1.81	49.93
**7**	**1.70**	**51.62**
8	1.53	53.15
9	1.50	54.65
10	1.44	56.09
11	1.35	57.43
12	1.32	58.76
13	1.27	60.03
14	1.20	61.22
15	1.18	62.40
16	1.15	63.55
17	1.14	64.69
18	1.09	65.78
19	1.06	66.84
20	1.02	67.87

Notes. N=20543; PCA was conducted using 'survey::svyprcomp(.)' function using all available contemporaneous exposures at wave 1. All PCs were standardized prior to being used as predictors. The bolded row represented the number of retained components for analysis was 7.

Table S19g. Associations of forgivingness with adult well-being and other outcomes at Wave 2 in Japan

	Model 1: Demographic and Childhood Variables as Covariates					Model 2: Demographic, Childhood, and Other Wave 1 Confounding Variables (Via Principal Components) as Covariates				
Outcome	RR	ES	95% CI	SE	p-value	RR	ES	95% CI	SE	p-value
Human Flourishing										
Secure flourishing index		0.14	(0.13,0.16)	0.01	2.22e-16***		-0.01	(-0.02,0.00)	0.01	0.100
Flourishing index		0.15	(0.13,0.16)	0.01	2.22e-16***		-0.01	(-0.02,0.00)	0.01	0.101
Happiness & life satisfaction		0.11	(0.10,0.13)	0.01	2.22e-16***		-0.02	(-0.03,-0.00)	0.01	0.019*
Physical & mental health		0.11	(0.09,0.12)	0.01	2.22e-16***		-0.00	(-0.01,0.01)	0.01	0.769
Meaning & purpose		0.13	(0.11,0.14)	0.01	2.22e-16***		-0.03	(-0.04,-0.01)	0.01	9.69e-05***
Character & virtue		0.13	(0.12,0.15)	0.01	2.22e-16***		0.00	(-0.01,0.02)	0.01	0.599
Close social relationships		0.14	(0.13,0.16)	0.01	2.22e-16***		0.00	(-0.01,0.02)	0.01	0.480
Financial & material security		0.08	(0.06,0.10)	0.01	2.22e-16***		-0.01	(-0.02,0.01)	0.01	0.506
Psychological Well-Being										
Happiness (a)		0.11	(0.10,0.13)	0.01	2.22e-16***		-0.01	(-0.03,0.00)	0.01	0.177
Life satisfaction (a)		0.10	(0.09,0.12)	0.01	2.22e-16***		-0.02	(-0.03,-0.00)	0.01	0.017*
Current life evaluation		0.10	(0.08,0.12)	0.01	2.22e-16***		-0.02	(-0.03,-0.00)	0.01	0.009*
Future life evaluation		0.11	(0.10,0.13)	0.01	2.22e-16***		-0.02	(-0.03,-0.00)	0.01	0.040*
Optimism		0.12	(0.10,0.14)	0.01	2.22e-16***		0.00	(-0.02,0.02)	0.01	0.770
Freedom to pursue what's important		0.10	(0.08,0.12)	0.01	2.22e-16***		-0.03	(-0.04,-0.01)	0.01	4e-04***
Inner peace	1.07		(1.06,1.08)	0.00	2.22e-16***	1.02		(1.01,1.03)	0.00	1.73e-06***
Life balance	1.07		(1.06,1.07)	0.00	2.22e-16***	1.02		(1.01,1.03)	0.00	5.76e-06***
Sense of mastery	1.05		(1.04,1.06)	0.00	2.22e-16***	1.01		(1.00,1.01)	0.00	0.277
Meaningful activities (c)		0.12	(0.10,0.14)	0.01	2.22e-16***		-0.02	(-0.04,-0.01)	0.01	0.005*
Understanding purpose (c)		0.11	(0.09,0.13)	0.01	2.22e-16***		-0.03	(-0.04,-0.01)	0.01	5.09e-04***
Self-rated mental health (b)		0.11	(0.09,0.13)	0.01	2.22e-16***		0.00	(-0.01,0.01)	0.01	0.985
Psychological Distress										
Traumatic distress	0.99		(0.99,1.00)	0.00	0.245	1.00		(0.99,1.01)	0.00	0.946
Depression symptoms composite	0.97		(0.97,0.98)	0.00	6.29e-10***	1.00		(0.99,1.01)	0.00	0.526
Depression – feel hopeless	0.98		(0.97,0.98)	0.00	5.2e-09***	1.00		(0.99,1.00)	0.00	0.195
Depression – loss of interest	0.98		(0.97,0.99)	0.00	1.34e-07***	1.00		(0.99,1.01)	0.00	0.814
Anxiety symptoms composite	0.97		(0.97,0.98)	0.00	1.13e-11***	0.99		(0.98,1.00)	0.00	0.009*
Anxiety – feel on edge	0.98		(0.97,0.98)	0.00	1.08e-08***	0.99		(0.99,1.00)	0.00	0.097
Anxiety – cannot stop worrying	0.98		(0.98,0.99)	0.00	1.08e-07***	0.99		(0.99,1.00)	0.00	0.025*
Suffering	0.98		(0.97,0.99)	0.00	5.1e-04***	1.00		(0.99,1.01)	0.00	0.530
Social Well-Being										
Relationship contentment (e)		0.14	(0.12,0.15)	0.01	2.22e-16***		0.01	(-0.01,0.02)	0.01	0.311
Relationship satisfaction (e)		0.13	(0.11,0.15)	0.01	2.22e-16***		0.00	(-0.01,0.02)	0.01	0.831
Social support		0.11	(0.09,0.13)	0.01	2.22e-16***		0.01	(-0.01,0.02)	0.01	0.369
Intimate/close friend	1.04		(1.03,1.05)	0.00	2.22e-16***	1.00		(1.00,1.01)	0.00	0.306
Government approval	1.02		(1.01,1.02)	0.00	1.39e-06***	1.00		(0.99,1.01)	0.00	0.784
Say in government	1.03		(1.02,1.04)	0.00	5.6e-14***	1.01		(1.00,1.02)	0.00	0.006*
Belonging in country		0.12	(0.10,0.14)	0.01	2.22e-16***		0.01	(-0.01,0.02)	0.01	0.337
City/place satisfaction	1.04		(1.03,1.05)	0.00	8.88e-16***	1.01		(1.00,1.02)	0.00	0.008*
Trust within country	1.06		(1.05,1.07)	0.00	2.22e-16***	1.03		(1.02,1.04)	0.00	2.82e-08***
Social Participation										
Ever been married	1.00		(1.00,1.01)	0.00	0.071	1.00		(1.00,1.00)	0.00	0.276
Currently divorced	1.00		(1.00,1.00)	0.00	0.406	1.00		(1.00,1.00)	0.00	0.408
Number of children		0.01	(-0.01,0.03)	0.01	0.310		0.01	(-0.01,0.03)	0.01	0.480
Weekly+ community participation	1.01		(1.00,1.01)	0.00	0.028*	1.00		(0.99,1.00)	0.00	0.293
Weekly+ religious attendance	1.00		(1.00,1.01)	0.00	0.251	1.00		(1.00,1.00)	0.00	0.906
Social Distress										
Loneliness		-0.09	(-0.11,-0.07)	0.01	2.22e-16***		0.00	(-0.01,0.02)	0.01	0.681
Perceived discrimination	1.01		(1.00,1.01)	0.00	0.089	1.00		(0.99,1.01)	0.00	0.646
Character & Prosocial Behavior										
Orientation to promote good (d)		0.13	(0.12,0.15)	0.01	2.22e-16***		-0.00	(-0.02,0.01)	0.01	0.802
Delayed gratification (d)		0.09	(0.07,0.11)	0.01	2.22e-16***		0.01	(-0.01,0.03)	0.01	0.340
Hope		0.13	(0.12,0.15)	0.01	2.22e-16***		-0.01	(-0.03,0.00)	0.01	0.146
Gratitude		0.15	(0.14,0.17)	0.01	2.22e-16***		0.02	(0.01,0.04)	0.01	4.37e-03**
Showing love/care		0.16	(0.14,0.18)	0.01	2.22e-16***		0.02	(0.01,0.04)	0.01	0.006*
Forgivingness	1.19		(1.18,1.20)	0.00	2.22e-16***	1.17		(1.16,1.19)	0.01	2.22e-16***
Charitable giving	1.01		(1.01,1.02)	0.00	1.58e-04***	1.00		(1.00,1.01)	0.00	0.450
Helping strangers	1.02		(1.01,1.02)	0.00	1.09e-05***	1.00		(1.00,1.01)	0.00	0.386
Volunteering	1.01		(1.01,1.02)	0.00	1.06e-05***	1.00		(1.00,1.01)	0.00	0.267
Physical Health & Health Behavior										
Self-rated physical health (b)		0.08	(0.06,0.10)	0.01	2.22e-16***		-0.00	(-0.02,0.01)	0.01	0.630
Health problems	1.00		(0.99,1.00)	0.00	0.513	1.00		(0.99,1.01)	0.00	0.669
Pain in past 4 weeks	0.99		(0.98,1.00)	0.00	0.210	1.00		(0.99,1.01)	0.00	0.531
Daily smoker	1.00		(1.00,1.01)	0.00	0.378	1.00		(1.00,1.01)	0.00	0.247
Number of drinks per week		0.00	(-0.01,0.02)	0.01	0.643		-0.00	(-0.02,0.02)	0.01	0.665
Days exercise per week		0.04	(0.03,0.06)	0.01	1.7e-06***		0.01	(-0.01,0.03)	0.01	0.471
Socioeconomic Outcomes										
Financial security (f)		0.07	(0.05,0.08)	0.01	2.98e-14***		-0.01	(-0.02,0.01)	0.01	0.395
Material security (f)		0.08	(0.06,0.10)	0.01	2.22e-16***		-0.00	(-0.02,0.01)	0.01	0.793
Educational attainment (16+ years)	1.00		(1.00,1.00)	0.00	0.187	1.00		(1.00,1.00)	0.00	0.525
Currently employed	1.00		(1.00,1.01)	0.00	0.612	1.00		(0.99,1.01)	0.00	0.962
Financially comfortable/getting by	1.02		(1.02,1.03)	0.00	1.98e-08***	1.00		(1.00,1.01)	0.00	0.397
Own home	1.01		(1.00,1.02)	0.00	0.110	1.01		(1.00,1.02)	0.00	0.170
Income – top quintile	1.00		(1.00,1.01)	0.00	0.393	1.00		(0.99,1.01)	0.00	0.881
Religion & Spirituality										
Religious/spiritual connection	1.03		(1.02,1.04)	0.00	1.03e-12***	1.01		(1.00,1.01)	0.00	0.104
Belief in life after death	1.01		(1.01,1.02)	0.00	1.94e-04***	1.00		(0.99,1.01)	0.00	0.626
Transformative religious experience	1.01		(1.00,1.01)	0.00	0.055	1.00		(0.99,1.00)	0.00	0.065
Religious reading or listening	1.00		(1.00,1.01)	0.00	0.014*	1.00		(1.00,1.00)	0.00	0.827
Prayer or meditation	1.01		(1.00,1.02)	0.00	7.55e-04**	1.00		(1.00,1.01)	0.00	0.440
Belief in God/gods/spiritual forces	1.02		(1.01,1.03)	0.00	4.21e-05***	1.00		(0.99,1.01)	0.00	0.565
Religious centrality	1.01		(1.01,1.02)	0.00	2.59e-05***	1.00		(1.00,1.00)	0.00	0.997
Religious/spiritual comfort	1.01		(1.00,1.01)	0.00	7.71e-05***	1.00		(0.99,1.00)	0.00	0.451
Feel loved by God	1.02		(1.01,1.03)	0.00	3e-10***	1.00		(1.00,1.01)	0.00	0.506
Feel punished by God	1.00		(1.00,1.00)	0.00	0.478	1.00		(1.00,1.00)	0.00	0.549
Experienced religious criticism	1.00		(1.00,1.00)	0.00	0.947	1.00		(1.00,1.00)	0.00	0.782
Faith-sharing	1.01		(1.00,1.01)	0.00	1.28e-04***	1.00		(1.00,1.00)	0.00	0.627

Notes. N=20543; Reference for focal predictor: never/rarely. RR, risk-ratio, null effect is 1.00; ES, effect size measure for standardized regression coefficient, null effect is 0.00; SE, standard error, the SE reported for binary/Likert-type outcomes where risk-ratios are on the log(RR) scale; CI, confidence interval; p-value, a Wald-type test of the null hypothesis that the effect of the focal predictor is zero; (a) item part of the Happiness & Life Satisfaction domain of the Secure Flourishing Index; (b) item part of the Physical & Mental Health domain of the Secure Flourishing Index; (c) item part of the Meaning & Purpose domain of the Secure Flourishing Index; (d) item part of the Character & Virtue domain of the Secure Flourishing Index; (e) item part of the Subjective Social Connectedness domain of the Secure Flourishing Index; (f) item part of the Financial & Material Security domain of the Secure Flourishing Index.

Multiple imputation was performed to impute missing data on the covariates, exposure, and outcomes. All models controlled for sociodemographic and childhood factors assessed at Wave 1. For Model 2 with PC (principal components), the first seven principal components of the entire set of contemporaneous confounders assessed at Wave 1 were included as additional covariates of the outcomes at Wave 2.

An outcome-wide analytic approach was used, and a separate model was run for each outcome. A different type of model was run depending on the nature of the outcome: (1) for each binary outcome, a weighted generalized linear model (with a log link and Poisson distribution) was used to estimate an RR; and (2) for each continuous outcome, a weighted linear regression model was used to estimate a ES. All effect sizes were standardized. For continuous outcomes, the ES represents the change in SD on the outcome between the lower and upper categories of the binary focal predictor. For binary outcomes, the RR represents the change in risk of being in the upper category compared to the lower category between the lower and upper categories of the binary focal predictor.

P-value significance thresholds: $p < 0.05$*, $p < 0.005$**, (Bonferroni) $p < 6.41e-04$***, correction for multiple testing using Bonferroni adjusted significant threshold.

Table S19h. Associations of forgivingness with adult well-being and other outcomes at Wave 2 in Japan using complete-case analyses with attrition weights.

	Model 1: Demographic and Childhood Variables as Covariates					Model 2: Demographic, Childhood, and Other Wave 1 Confounding Variables (Via Principal Components) as Covariates				
Outcome	RR	ES	95% CI	SE	p-value	RR	ES	95% CI	SE	p-value
Human Flourishing										
Secure flourishing index		0.14	(0.12,0.16)	0.01	2.22e-16***		-0.01	(-0.02,0.01)	0.01	0.321
Flourishing index		0.14	(0.13,0.16)	0.01	2.22e-16***		-0.01	(-0.02,0.01)	0.01	0.377
Happiness & life satisfaction		0.11	(0.09,0.13)	0.01	2.22e-16***		-0.02	(-0.03,0.00)	0.01	0.058
Physical & mental health		0.10	(0.08,0.12)	0.01	2.22e-16***		-0.00	(-0.02,0.02)	0.01	0.840
Meaning & purpose		0.13	(0.11,0.15)	0.01	2.22e-16***		-0.02	(-0.04,-0.01)	0.01	1.63e-03**
Character & virtue		0.14	(0.12,0.16)	0.01	2.22e-16***		0.01	(-0.01,0.03)	0.01	0.337
Close social relationships		0.14	(0.12,0.16)	0.01	2.22e-16***		0.01	(-0.01,0.02)	0.01	0.367
Financial & material security		0.08	(0.06,0.10)	0.01	1.22e-14***		-0.01	(-0.03,0.01)	0.01	0.450
Psychological Well-Being										
Happiness (a)		0.12	(0.10,0.14)	0.01	2.22e-16***		-0.01	(-0.03,0.01)	0.01	0.182
Life satisfaction (a)		0.11	(0.09,0.13)	0.01	2.22e-16***		-0.02	(-0.03,-0.00)	0.01	0.029*
Current life evaluation		0.10	(0.08,0.12)	0.01	2.22e-16***		-0.02	(-0.04,-0.00)	0.01	0.016*
Future life evaluation		0.11	(0.09,0.13)	0.01	2.22e-16***		-0.02	(-0.04,-0.00)	0.01	0.032*
Optimism		0.12	(0.09,0.14)	0.01	2.22e-16***		-0.01	(-0.02,0.01)	0.01	0.556
Freedom to pursue what's important		0.10	(0.08,0.12)	0.01	2.22e-16***		-0.03	(-0.05,-0.01)	0.01	3.24e-04***
Inner peace	1.08		(1.06,1.09)	0.01	2.22e-16***	1.03		(1.02,1.03)	0.00	3.1e-07***
Life balance	1.07		(1.06,1.08)	0.00	2.22e-16***	1.02		(1.01,1.03)	0.00	1.04e-06***
Sense of mastery	1.05		(1.04,1.07)	0.01	2.22e-16***	1.00		(1.00,1.01)	0.00	0.326
Meaningful activities (c)		0.13	(0.11,0.15)	0.01	2.22e-16***		-0.02	(-0.04,-0.00)	0.01	0.025*
Understanding purpose (c)		0.11	(0.09,0.13)	0.01	2.22e-16***		-0.03	(-0.04,-0.01)	0.01	1.8e-03**
Self-rated mental health (b)		0.11	(0.09,0.13)	0.01	2.22e-16***		0.00	(-0.02,0.02)	0.01	0.922
Psychological Distress										
Traumatic distress	1.00		(0.99,1.01)	0.00	0.547	1.00		(0.99,1.01)	0.00	0.871
Depression symptoms composite	0.97		(0.96,0.98)	0.00	6.3e-09***	1.00		(0.99,1.00)	0.00	0.241
Depression – feel hopeless	0.98		(0.97,0.98)	0.00	1.88e-08***	0.99		(0.99,1.00)	0.00	0.116
Depression – loss of interest	0.98		(0.97,0.98)	0.00	7.38e-08***	1.00		(0.99,1.00)	0.00	0.266
Anxiety symptoms composite	0.98		(0.97,0.98)	0.00	1.16e-08***	0.99		(0.98,1.00)	0.00	0.027*
Anxiety – feel on edge	0.98		(0.97,0.98)	0.00	6.01e-08***	0.99		(0.99,1.00)	0.00	0.113
Anxiety – cannot stop worrying	0.98		(0.97,0.99)	0.00	1.42e-06***	0.99		(0.99,1.00)	0.00	0.031*
Suffering	0.99		(0.98,1.00)	0.01	0.012*	1.00		(1.00,1.01)	0.00	0.335
Social Well-Being										
Relationship contentment (e)		0.14	(0.12,0.16)	0.01	2.22e-16***		0.01	(-0.01,0.03)	0.01	0.185
Relationship satisfaction (e)		0.13	(0.11,0.15)	0.01	2.22e-16***		0.00	(-0.01,0.02)	0.01	0.704
Social support		0.12	(0.10,0.14)	0.01	2.22e-16***		0.01	(-0.01,0.03)	0.01	0.339
Intimate/close friend	1.04		(1.03,1.05)	0.00	2.22e-16***	1.00		(0.99,1.01)	0.00	0.648
Government approval	1.02		(1.01,1.02)	0.00	7.64e-06***	1.00		(0.99,1.00)	0.00	0.451
Say in government	1.03		(1.02,1.04)	0.00	1.21e-12***	1.01		(1.00,1.02)	0.00	0.066
Belonging in country		0.13	(0.11,0.15)	0.01	2.22e-16***		0.01	(-0.01,0.03)	0.01	0.212
City/place satisfaction	1.04		(1.03,1.05)	0.01	2.89e-15***	1.01		(1.00,1.02)	0.01	0.030*
Trust within country	1.06		(1.05,1.07)	0.00	2.22e-16***	1.03		(1.02,1.04)	0.00	1.09e-08***
Social Participation										
Ever been married	1.00		(1.00,1.01)	0.00	0.024*	1.00		(1.00,1.00)	0.00	0.278
Currently divorced	1.00		(1.00,1.00)	0.00	0.312	1.00		(1.00,1.01)	0.00	0.277
Number of children		0.02	(-0.00,0.04)	0.01	0.065		0.02	(-0.00,0.04)	0.01	0.135
Weekly+ community participation	1.01		(1.00,1.01)	0.00	0.045*	1.00		(0.99,1.00)	0.00	0.238
Weekly+ religious attendance	1.00		(1.00,1.01)	0.00	0.061	1.00		(1.00,1.00)	0.00	0.963
Social Distress										
Loneliness		-0.10	(-0.12,-0.08)	0.01	2.22e-16***		0.00	(-0.02,0.02)	0.01	0.929
Perceived discrimination	1.01		(1.00,1.02)	0.00	0.067	1.00		(0.99,1.01)	0.00	0.903
Character & Prosocial Behavior										
Orientation to promote good (d)		0.14	(0.12,0.16)	0.01	2.22e-16***		0.00	(-0.02,0.02)	0.01	0.809
Delayed gratification (d)		0.09	(0.07,0.12)	0.01	2.22e-16***		0.01	(-0.01,0.03)	0.01	0.223
Hope		0.14	(0.12,0.16)	0.01	2.22e-16***		-0.01	(-0.02,0.01)	0.01	0.520
Gratitude		0.16	(0.14,0.18)	0.01	2.22e-16***		0.03	(0.01,0.04)	0.01	0.006*
Showing love/care		0.16	(0.14,0.18)	0.01	2.22e-16***		0.02	(-0.00,0.04)	0.01	0.063
Forgivingness	1.20		(1.19,1.21)	0.01	2.22e-16***	1.18		(1.17,1.19)	0.01	2.22e-16***
Charitable giving	1.01		(1.01,1.02)	0.00	6.24e-05***	1.00		(0.99,1.01)	0.00	0.674
Helping strangers	1.02		(1.01,1.03)	0.00	1.09e-07***	1.00		(1.00,1.01)	0.00	0.268
Volunteering	1.01		(1.01,1.02)	0.00	7.42e-07***	1.00		(1.00,1.01)	0.00	0.222
Physical Health & Health Behavior										
Self-rated physical health (b)		0.08	(0.06,0.10)	0.01	1.19e-13***		-0.00	(-0.02,0.01)	0.01	0.652
Health problems	1.00		(0.99,1.01)	0.00	0.772	1.00		(0.99,1.00)	0.00	0.448
Pain in past 4 weeks	0.99		(0.98,1.00)	0.01	0.244	0.99		(0.98,1.00)	0.01	0.272
Daily smoker	1.00		(1.00,1.01)	0.00	0.394	1.00		(0.99,1.01)	0.00	0.551
Number of drinks per week		0.00	(-0.02,0.02)	0.01	0.916		-0.01	(-0.03,0.01)	0.01	0.426
Days exercise per week		0.05	(0.03,0.07)	0.01	4.47e-06***		0.01	(-0.01,0.03)	0.01	0.371
Socioeconomic Outcomes										
Financial security (f)		0.07	(0.05,0.09)	0.01	7.23e-11***		-0.01	(-0.03,0.01)	0.01	0.265
Material security (f)		0.09	(0.06,0.11)	0.01	8.88e-16***		-0.00	(-0.02,0.02)	0.01	0.825
Educational attainment (16+ years)	1.00		(1.00,1.00)	0.00	0.117	1.00		(1.00,1.00)	0.00	0.468
Currently employed	1.00		(1.00,1.01)	0.00	0.636	1.00		(0.99,1.00)	0.00	0.551
Financially comfortable/getting by	1.03		(1.02,1.03)	0.00	1.31e-08***	1.00		(1.00,1.01)	0.00	0.337
Own home	1.01		(1.00,1.02)	0.01	0.105	1.01		(1.00,1.02)	0.01	0.303
Income – top quintile	1.00		(0.99,1.01)	0.01	0.601	1.00		(0.99,1.01)	0.01	0.867
Religion & Spirituality										
Religious/spiritual connection	1.03		(1.02,1.04)	0.00	3.88e-11***	1.00		(1.00,1.01)	0.00	0.358
Belief in life after death	1.02		(1.01,1.03)	0.00	4.09e-05***	1.00		(0.99,1.01)	0.00	0.784
Transformative religious experience	1.01		(1.00,1.01)	0.00	0.090	0.99		(0.99,1.00)	0.00	0.043*
Religious reading or listening	1.00		(1.00,1.01)	0.00	0.013*	1.00		(1.00,1.00)	0.00	0.812
Prayer or meditation	1.01		(1.01,1.02)	0.00	1.4e-04***	1.00		(1.00,1.01)	0.00	0.572
Belief in God/gods/spiritual forces	1.02		(1.01,1.03)	0.00	4.16e-06***	1.00		(0.99,1.01)	0.00	0.790
Religious centrality	1.01		(1.01,1.02)	0.00	2.07e-05***	1.00		(0.99,1.00)	0.00	0.530
Religious/spiritual comfort	1.01		(1.00,1.01)	0.00	1.74e-04***	1.00		(0.99,1.00)	0.00	0.212
Feel loved by God	1.02		(1.02,1.03)	0.00	5.01e-11***	1.00		(1.00,1.01)	0.00	0.438
Feel punished by God	1.00		(1.00,1.01)	0.00	0.178	1.00		(1.00,1.00)	0.00	0.517
Experienced religious criticism	1.00		(1.00,1.00)	0.00	0.668	1.00		(1.00,1.00)	0.00	0.223
Faith-sharing	1.01		(1.00,1.01)	0.00	1.65e-04***	1.00		(1.00,1.00)	0.00	0.800

Table S19h. Associations of forgivingness with adult well-being and other outcomes at Wave 2 in Japan using complete-case analyses with attrition weights.

Outcome	Model 1: Demographic and Childhood Variables as Covariates					Model 2: Demographic, Childhood, and Other Wave 1 Confounding Variables (Via Principal Components) as Covariates				
	RR	ES	95% CI	SE	p-value	RR	ES	95% CI	SE	p-value

Notes. N=13972; Reference for focal predictor: never/rarely. RR, risk-ratio, null effect is 1.00; ES, effect size measure for standardized regression coefficient, null effect is 0.00; SE, standard error, the SE reported for binary/Likert-type outcomes where risk-ratios are on the log(RR) scale; CI, confidence interval; p-value, a Wald-type test of the null hypothesis that the effect of the focal predictor is zero; (a) item part of the Happiness & Life Satisfaction domain of the Secure Flourishing Index; (b) item part of the Physical & Mental Health domain of the Secure Flourishing Index; (c) item part of the Meaning & Purpose domain of the Secure Flourishing Index; (d) item part of the Character & Virtue domain of the Secure Flourishing Index; (e) item part of the Subjective Social Connectedness domain of the Secure Flourishing Index; (f) item part of the Financial & Material Security domain of the Secure Flourishing Index.

Attrition weights were computed to adjust the complete case data (those who responded at Wave 2 to at least 50% of the questions) and multiple imputation was used to impute missing data on all remaining within wave on the covariates, exposure, and outcomes. All models controlled for sociodemographic and childhood factors assessed at Wave 1. For Model 2 with PC (principal components), the first seven principal components of the entire set of contemporaneous confounders assessed at Wave 1 were included as additional covariates of the outcomes at Wave 2.

An outcome-wide analytic approach was used, and a separate model was run for each outcome. A different type of model was run depending on the nature of the outcome: (1) for each binary outcome, a weighted generalized linear model (with a log link and Poisson distribution) was used to estimate an RR; and (2) for each continuous outcome, a weighted linear regression model was used to estimate a ES. All effect sizes were standardized. For continuous outcomes, the ES represents the change in SD on the outcome between the lower and upper categories of the binary focal predictor. For binary outcomes, the RR represents the change in risk of being in the upper category compared to the lower category between the lower and upper categories of the binary focal predictor.

P-value significance thresholds: $p < 0.05$*, $p < 0.005$**, (Bonferroni) $p < 6.41e-04$***, correction for multiple testing using Bonferroni adjusted significant threshold.

Table S19i. Sensitivity analysis of forgivingness outcome-wide results to unmeasured confounding using E-values in Japan

Outcome	Multiple Imputation: Model 1: Demographics and Childhood Variables as Covariates EE	ECI	Multiple Imputation: Model 2: Demographics, Childhood, and Other Wave 1 Confounders (Via Principal Components) as Covariates EE	ECI	Complete Case w/ Attrition Weights: Model 1: Demographics and Childhood Variables as Covariates EE	ECI	Complete Case w/ Attrition Weights: Model 2: Demographics, Childhood, and Other Wave 1 Confounders (Via Principal Components) as Covariates EE	ECI
Human Flourishing								
Secure flourishing index	1.92	1.83	1.14	1.00	1.91	1.80	1.13	1.00
Flourishing index	1.94	1.86	1.15	1.00	1.93	1.83	1.12	1.00
Happiness & life satisfaction	1.77	1.69	1.20	1.07	1.77	1.67	1.20	1.00
Physical & mental health	1.73	1.64	1.06	1.00	1.71	1.60	1.06	1.00
Meaning & purpose	1.84	1.75	1.29	1.19	1.85	1.75	1.27	1.15
Character & virtue	1.87	1.78	1.09	1.00	1.88	1.77	1.15	1.00
Close social relationships	1.92	1.83	1.11	1.00	1.92	1.81	1.13	1.00
Financial & material security	1.59	1.50	1.11	1.00	1.59	1.48	1.13	1.00
Psychological Well-Being								
Happiness	1.77	1.67	1.16	1.00	1.78	1.67	1.16	1.00
Life satisfaction	1.72	1.62	1.22	1.09	1.73	1.62	1.22	1.06
Current life evaluation	1.69	1.60	1.23	1.10	1.70	1.59	1.24	1.09
Future life evaluation	1.76	1.67	1.20	1.04	1.76	1.65	1.22	1.06
Optimism	1.79	1.69	1.08	1.00	1.77	1.66	1.11	1.00
Freedom to pursue what’s important	1.70	1.61	1.29	1.18	1.70	1.59	1.32	1.19
Inner peace	1.56	1.51	1.26	1.19	1.58	1.52	1.28	1.21
Life balance	1.53	1.48	1.25	1.18	1.55	1.49	1.27	1.20
Sense of mastery	1.46	1.40	1.11	1.00	1.47	1.41	1.11	1.00
Meaningful activities	1.80	1.71	1.24	1.12	1.84	1.73	1.23	1.07
Understanding purpose	1.74	1.65	1.29	1.18	1.77	1.66	1.28	1.16
Self-rated mental health	1.75	1.66	1.02	1.00	1.76	1.65	1.04	1.00
Psychological Distress								
Traumatic distress	1.11	1.00	1.02	1.00	1.08	1.00	1.04	1.00
Depression symptoms composite	1.29	1.23	1.07	1.00	1.30	1.23	1.11	1.00
Depression – feel hopeless	1.27	1.21	1.11	1.00	1.27	1.21	1.12	1.00
Depression – loss of interest	1.26	1.20	1.05	1.00	1.28	1.21	1.11	1.00
Anxiety symptoms composite	1.29	1.24	1.16	1.07	1.29	1.22	1.16	1.05
Anxiety – feel on edge	1.28	1.22	1.13	1.00	1.28	1.21	1.13	1.00
Anxiety – cannot stop worrying	1.23	1.18	1.14	1.05	1.23	1.17	1.14	1.04
Suffering	1.22	1.14	1.08	1.00	1.19	1.08	1.11	1.00
Social Well-Being								
Relationship contentment	1.90	1.81	1.13	1.00	1.93	1.82	1.17	1.00
Relationship satisfaction	1.86	1.77	1.06	1.00	1.87	1.76	1.09	1.00
Social support	1.75	1.65	1.13	1.00	1.79	1.68	1.15	1.00
Intimate/close friend	1.40	1.35	1.10	1.00	1.40	1.34	1.07	1.00
Government approval	1.22	1.17	1.05	1.00	1.22	1.16	1.08	1.00
Say in government	1.33	1.27	1.17	1.09	1.33	1.27	1.15	1.00
Belonging in country	1.80	1.71	1.14	1.00	1.86	1.75	1.17	1.00
City/place satisfaction	1.40	1.33	1.19	1.09	1.40	1.33	1.18	1.05
Trust within country	1.48	1.42	1.31	1.24	1.48	1.42	1.31	1.24
Social Participation								
Ever been married	1.08	1.00	1.06	1.00	1.09	1.03	1.06	1.00
Currently divorced	1.05	1.00	1.05	1.00	1.06	1.00	1.06	1.00
Number of children	1.16	1.00	1.13	1.00	1.24	1.00	1.20	1.00
Weekly+ community participation	1.13	1.04	1.08	1.00	1.13	1.02	1.09	1.00
Weekly+ religious attendance	1.07	1.00	1.02	1.00	1.09	1.00	1.01	1.00
Social Distress								
Loneliness	1.65	1.55	1.09	1.00	1.68	1.57	1.04	1.00
Perceived discrimination	1.12	1.00	1.06	1.00	1.14	1.00	1.03	1.00
Character & Prosocial Behavior								
Orientation to promote good	1.88	1.78	1.07	1.00	1.91	1.80	1.07	1.00
Delayed gratification	1.64	1.54	1.15	1.00	1.66	1.55	1.18	1.00
Hope	1.88	1.79	1.16	1.00	1.92	1.81	1.11	1.00
Gratitude	1.98	1.89	1.26	1.13	2.01	1.90	1.27	1.13
Showing love/care	2.01	1.92	1.26	1.13	2.00	1.89	1.21	1.00
Forgivingness	2.21	2.15	2.10	2.04	2.24	2.18	2.13	2.06
Charitable giving	1.19	1.12	1.08	1.00	1.19	1.13	1.06	1.00
Helping strangers	1.22	1.16	1.08	1.00	1.24	1.18	1.10	1.00
Volunteering	1.19	1.13	1.08	1.00	1.20	1.15	1.09	1.00
Physical Health & Health Behavior								
Self-rated physical health	1.59	1.50	1.09	1.00	1.57	1.46	1.10	1.00
Health problems	1.08	1.00	1.06	1.00	1.05	1.00	1.08	1.00
Pain in past 4 weeks	1.12	1.00	1.08	1.00	1.12	1.00	1.12	1.00
Daily smoker	1.09	1.00	1.10	1.00	1.10	1.00	1.08	1.00
Number of drinks per week	1.10	1.00	1.10	1.00	1.05	1.00	1.15	1.00
Days exercise per week	1.39	1.28	1.13	1.00	1.41	1.29	1.15	1.00
Socioeconomic Outcomes								
Financial security	1.52	1.42	1.13	1.00	1.53	1.41	1.16	1.00
Material security	1.59	1.49	1.07	1.00	1.61	1.50	1.07	1.00
Educational attainment (16+ years)	1.04	1.00	1.03	1.00	1.03	1.00	1.02	1.00
Currently employed	1.06	1.00	1.02	1.00	1.06	1.00	1.06	1.00
Financially comfortable/getting by	1.27	1.21	1.09	1.00	1.29	1.22	1.10	1.00
Own home	1.14	1.00	1.13	1.00	1.15	1.00	1.12	1.00
Income – top quintile	1.09	1.00	1.04	1.00	1.08	1.00	1.05	1.00
Religion & Spirituality								
Religious/spiritual connection	1.32	1.26	1.13	1.00	1.32	1.25	1.10	1.00
Belief in life after death	1.21	1.14	1.07	1.00	1.23	1.16	1.05	1.00
Transformative religious experience	1.12	1.00	1.11	1.00	1.11	1.00	1.12	1.02
Religious reading or listening	1.10	1.04	1.03	1.00	1.11	1.05	1.03	1.00
Prayer or meditation	1.17	1.10	1.07	1.00	1.18	1.12	1.06	1.00
Belief in God/gods/spiritual forces	1.24	1.16	1.08	1.00	1.25	1.18	1.05	1.00
Religious centrality	1.17	1.12	1.00	1.00	1.17	1.12	1.06	1.00
Religious/spiritual comfort	1.16	1.11	1.06	1.00	1.16	1.11	1.09	1.00
Feel loved by God	1.24	1.19	1.07	1.00	1.26	1.21	1.07	1.00
Feel punished by God	1.05	1.00	1.05	1.00	1.07	1.00	1.05	1.00
Experienced religious criticism	1.01	1.00	1.03	1.00	1.04	1.00	1.06	1.00
Faith-sharing	1.13	1.09	1.04	1.00	1.14	1.09	1.03	1.00

Notes. EE, E-value for estimate; ECI, E-value for the limit of the confidence interval. The formula for calculating E-values can be found in VanderWeele and Ding (2017). E-values for estimate are the minimum strength of association on the risk ratio scale that an unmeasured confounder would need to have with both the exposure and the outcome to fully explain away the observed association between the exposure and outcome, conditional on the measured covariates. E-values for the 95% CI closest to the null denote the minimum strength of association on the risk ratio scale that an unmeasured confounder would need to have with both the exposure and the outcome to shift the CI to include the null value, conditional on the measured covariates.

Table S20a. Weighted summary statistics for demographic and childhood variables in Kenya

**Characteristic**	**Wave 1** N = 11,389	**Wave 2** N = 7,713
Forgivingness, n (%)		
Always	5,323 (46.7%)	3,535 (45.8%)
Often	4,321 (37.9%)	2,991 (38.8%)
Rarely	1,460 (12.8%)	1,012 (13.1%)
Never	278 (2.4%)	167 (2.2%)
(Missing)	8 (0.1%)	7 (0.1%)
Year of birth, n (%)		
1943 or earlier (current age: 80+ years)	63 (0.6%)	40 (0.5%)
1943-1953 (current age: 70-79 years)	327 (2.9%)	251 (3.3%)
1953-1963 (current age: 60-69 years)	667 (5.9%)	515 (6.7%)
1963-1973 (current age: 50-59 years)	1,010 (8.9%)	722 (9.4%)
1973-1983 (current age: 40-49 years)	1,619 (14.2%)	1,180 (15.3%)
1983-1993 (current age: 30-39 years)	2,429 (21.3%)	1,768 (22.9%)
1993-1998 (current age: 25-29 years)	1,866 (16.4%)	1,324 (17.2%)
1998-2005 (current age: 18-24 years)	3,402 (29.9%)	1,913 (24.8%)
(Missing)	5 (0.0%)	0 (0%)
Age of participant		
Mean	35.4	36.8
Standard Deviation	15.0	14.8
Min, Max	18.0, 97.0	19.0, 94.0
(Missing)	5 (<0.1%)	0 (0%)
Gender, n (%)		
Male	5,555 (48.8%)	3,770 (48.9%)
Female	5,824 (51.1%)	3,940 (51.1%)
Other	2 (0.0%)	1 (<0.0%)
(Missing)	7 (0.1%)	3 (0.0%)
Respondent marital status, n (%)		
Single/Never been married	3,783 (33.2%)	2,277 (29.5%)
Married	6,405 (56.2%)	4,392 (56.9%)
Separated	458 (4.0%)	498 (6.5%)
Divorced	106 (0.9%)	66 (0.9%)
Widowed	441 (3.9%)	319 (4.1%)
Domestic partner	151 (1.3%)	148 (1.9%)
(Missing)	46 (0.4%)	14 (0.2%)
Education (years), n (%)		
Up to 8	4,736 (41.6%)	2,904 (37.6%)
9-15	5,910 (51.9%)	4,225 (54.8%)
16+	739 (6.5%)	585 (7.6%)
(Missing)	5 (0.0%)	0 (0%)
Employment status, n (%)		
Employed for an employer	1,413 (12.4%)	1,193 (15.5%)
Self-employed	3,483 (30.6%)	2,358 (30.6%)
Retired	281 (2.5%)	161 (2.1%)
Student	1,325 (11.6%)	522 (6.8%)
Homemaker	1,514 (13.3%)	956 (12.4%)
Unemployed and looking for a job	3,224 (28.3%)	2,369 (30.7%)
None of these/Other	141 (1.2%)	136 (1.8%)
(Missing)	9 (0.1%)	17 (0.2%)
Current religious service attendance, n (%)		
More than once a week	2,733 (24.0%)	2,076 (26.9%)

Table S20a. Weighted summary statistics for demographic and childhood variables in Kenya

**Characteristic**	**Wave 1** N = 11,389	**Wave 2** N = 7,713
Once a week	6,069 (53.3%)	3,691 (47.9%)
One to three times a month	1,242 (10.9%)	782 (10.1%)
A few times a year	861 (7.6%)	776 (10.1%)
Never	471 (4.1%)	380 (4.9%)
(Missing)	13 (0.1%)	8 (0.1%)
Immigration status, n (%)		
Born in this country	11,268 (98.9%)	7,633 (99.0%)
Born in another country	119 (1.0%)	79 (1.0%)
(Missing)	2 (0.0%)	1 (<0.0%)
Parental marital status around age 12, n (%)		
Parents were married	9,225 (81.0%)	6,258 (81.1%)
Parents were divorced	713 (6.3%)	477 (6.2%)
Parents were never married	682 (6.0%)	445 (5.8%)
One or both of them had died	463 (4.1%)	338 (4.4%)
Unsure	110 (1.0%)	72 (0.9%)
(Missing)	196 (1.7%)	123 (1.6%)
Religious service attendance around age 12, n (%)		
At least once a week	9,157 (80.4%)	6,266 (81.2%)
One to three times a month	1,705 (15.0%)	1,105 (14.3%)
Less than once a month	245 (2.2%)	179 (2.3%)
Never	203 (1.8%)	122 (1.6%)
(Missing)	79 (0.7%)	41 (0.5%)
Relationship with mother when growing up, n (%)		
Very good	9,400 (82.5%)	6,403 (83.0%)
Somewhat good	1,445 (12.7%)	984 (12.8%)
Somewhat bad	134 (1.2%)	78 (1.0%)
Very bad	108 (0.9%)	64 (0.8%)
(Does not apply)	236 (2.1%)	147 (1.9%)
(Missing)	66 (0.6%)	37 (0.5%)
Relationship with father when growing up, n (%)		
Very good	7,938 (69.7%)	5,355 (69.4%)
Somewhat good	1,906 (16.7%)	1,318 (17.1%)
Somewhat bad	220 (1.9%)	145 (1.9%)
Very bad	225 (2.0%)	154 (2.0%)
(Does not apply)	970 (8.5%)	661 (8.6%)
(Missing)	131 (1.1%)	80 (1.0%)
Felt like an outsider in family when growing up, n (%)		
Yes	1,249 (11.0%)	797 (10.3%)
No	10,089 (88.6%)	6,877 (89.2%)
(Missing)	52 (0.5%)	39 (0.5%)
Experienced abuse when growing up, n (%)		
Yes	1,319 (11.6%)	881 (11.4%)
No	10,022 (88.0%)	6,799 (88.1%)
(Missing)	48 (0.4%)	33 (0.4%)
Self-rated health when growing up, n (%)		
Excellent	4,459 (39.1%)	3,019 (39.1%)
Very good	2,612 (22.9%)	1,735 (22.5%)
Good	2,553 (22.4%)	1,752 (22.7%)
Fair	1,385 (12.2%)	957 (12.4%)
Poor	357 (3.1%)	243 (3.1%)

Table S20a. Weighted summary statistics for demographic and childhood variables in Kenya

**Characteristic**	**Wave 1** N = 11,389	**Wave 2** N = 7,713
(Missing)	24 (0.2%)	8 (0.1%)
Subjective financial status of family growing up, n (%)		
Lived comfortably	3,039 (26.7%)	2,010 (26.1%)
Got by	3,249 (28.5%)	2,237 (29.0%)
Found it difficult	4,085 (35.9%)	2,839 (36.8%)
Found it very difficult	997 (8.7%)	617 (8.0%)
(Missing)	19 (0.2%)	9 (0.1%)
Religious affiliation growing up, n (%)		
Christianity	10,352 (90.9%)	7,051 (91.4%)
Taoism	0 (0%)	0 (0%)
Confucianism	0 (0%)	0 (0%)
Primal, Animist, or Folk religion	12 (0.1%)	6 (0.1%)
Spiritism	0 (0%)	0 (0%)
Umbanda, Candomblé, and other African-derived religions	0 (0%)	0 (0%)
Chinese folk/traditional religion	0 (0%)	0 (0%)
Islam	935 (8.2%)	596 (7.7%)
Hinduism	0 (0%)	0 (0%)
Buddhism	5 (0.0%)	1 (0.0%)
Judaism	6 (0.1%)	6 (0.1%)
Sikhism	0 (<0.0%)	1 (<0.0%)
Baha'i	3 (0.0%)	3 (0.0%)
Jainism	1 (<0.0%)	1 (0.0%)
Shinto	0 (0%)	0 (0%)
Some other religion	1 (<0.0%)	0 (0%)
No religion/Atheist/Agnostic	65 (0.6%)	41 (0.5%)
(Missing)	8 (0.1%)	9 (0.1%)

Note. N (%); this table is based on non-imputed data. Cumulative percentages for variables may not add up to 100% due to rounding. Wave 1 characteristics weighted using the Gallup provided sampling weight, ANNUAL_WEIGHT_R2; Wave 2 characteristics weighted accounting for attrition by using the adjusted Wave 1 weight, ANNUAL_WEIGHT_R2, multiplied by the created attrition weight to account for dropout, to maintain nationally representative estimates for Wave 2 characteristics.

Table S20b. Weighted summary statistics for outcome variables in Kenya

**Outcome**	**Wave 1**<br>N = 11,389	**Wave 2**<br>N = 7,713
Secure flourishing index		
Mean	7.3	7.0
Standard Deviation	1.6	1.7
Min, Max	0.0, 10.0	0.0, 10.0
(Missing)	164 (1.4%)	39 (0.5%)
Flourishing index		
Mean	7.8	7.4
Standard Deviation	1.6	1.7
Min, Max	0.0, 10.0	0.0, 10.0
(Missing)	145 (1.3%)	34 (0.4%)
Happiness & life satisfaction		
Mean	6.7	6.3
Standard Deviation	2.7	2.7
Min, Max	0.0, 10.0	0.0, 10.0
(Missing)	23 (0.2%)	12 (0.1%)
Physical & mental health		
Mean	8.4	8.0
Standard Deviation	2.1	2.2
Min, Max	0.0, 10.0	0.0, 10.0
(Missing)	27 (0.2%)	8 (0.1%)
Meaning & purpose		
Mean	7.9	7.4
Standard Deviation	2.2	2.4
Min, Max	0.0, 10.0	0.0, 10.0
(Missing)	46 (0.4%)	7 (<0.1%)
Character & virtue		
Mean	8.1	7.6
Standard Deviation	2.2	2.4
Min, Max	0.0, 10.0	0.0, 10.0
(Missing)	21 (0.2%)	9 (0.1%)
Close social relationships		
Mean	7.9	7.4
Standard Deviation	2.4	2.6
Min, Max	0.0, 10.0	0.0, 10.0
(Missing)	44 (0.4%)	10 (0.1%)
Financial & material security		
Mean	4.9	5.0
Standard Deviation	3.4	3.2
Min, Max	0.0, 10.0	0.0, 10.0
(Missing)	21 (0.2%)	8 (0.1%)
Happiness		
Mean	7.3	6.9
Standard Deviation	3.0	3.0
Min, Max	0.0, 10.0	0.0, 10.0
(Missing)	13 (0.1%)	7 (<0.1%)
Life satisfaction		
Mean	6.0	5.8
Standard Deviation	3.5	3.5
Min, Max	0.0, 10.0	0.0, 10.0
(Missing)	15 (0.1%)	5 (<0.1%)
Current life evaluation		

Table S20b. Weighted summary statistics for outcome variables in Kenya

Outcome	Wave 1<br>N = 11,389	Wave 2<br>N = 7,713
Mean	5.5	5.5
Standard Deviation	3.3	3.2
Min, Max	0.0, 10.0	0.0, 10.0
(Missing)	18 (0.2%)	2 (<0.1%)
Future life evaluation		
Mean	7.5	7.2
Standard Deviation	2.8	2.9
Min, Max	0.0, 10.0	0.0, 10.0
(Missing)	121 (1.1%)	20 (0.3%)
Optimism		
Mean	8.9	8.6
Standard Deviation	2.2	2.6
Min, Max	0.0, 10.0	0.0, 10.0
(Missing)	9 (<0.1%)	9 (0.1%)
Freedom to pursue what's important		
Mean	8.4	7.9
Standard Deviation	2.6	3.0
Min, Max	0.0, 10.0	0.0, 10.0
(Missing)	11 (<0.1%)	5 (<0.1%)
Inner peace, n (%)		
Always	2,967 (26.1%)	1,671 (21.7%)
Often	3,949 (34.7%)	2,573 (33.4%)
Rarely	3,804 (33.4%)	3,007 (39.0%)
Never	645 (5.7%)	461 (6.0%)
(Missing)	24 (0.2%)	2 (0.0%)
Life balance, n (%)		
Always	2,464 (21.6%)	1,392 (18.0%)
Often	3,665 (32.2%)	2,309 (29.9%)
Rarely	4,562 (40.1%)	3,565 (46.2%)
Never	684 (6.0%)	445 (5.8%)
(Missing)	14 (0.1%)	1 (0.0%)
Sense of mastery, n (%)		
Always	4,086 (35.9%)	2,662 (34.5%)
Often	3,801 (33.4%)	2,405 (31.2%)
Rarely	2,992 (26.3%)	2,299 (29.8%)
Never	486 (4.3%)	330 (4.3%)
(Missing)	24 (0.2%)	18 (0.2%)
Meaningful activities		
Mean	7.2	6.9
Standard Deviation	3.1	3.2
Min, Max	0.0, 10.0	0.0, 10.0
(Missing)	18 (0.2%)	2 (<0.1%)
Understanding purpose		
Mean	8.5	8.0
Standard Deviation	2.5	2.9
Min, Max	0.0, 10.0	0.0, 10.0
(Missing)	29 (0.3%)	5 (<0.1%)
Self-rated mental health		
Mean	8.7	8.4
Standard Deviation	2.3	2.5
Min, Max	0.0, 10.0	0.0, 10.0

Table S20b. Weighted summary statistics for outcome variables in Kenya

**Outcome**	**Wave 1**<br>N = 11,389	**Wave 2**<br>N = 7,713
(Missing)	17 (0.2%)	4 (<0.1%)
Traumatic distress, n (%)		
A lot	1,693 (14.9%)	1,397 (18.1%)
Some	3,602 (31.6%)	2,724 (35.3%)
Not very much	3,190 (28.0%)	1,872 (24.3%)
Not at all	2,889 (25.4%)	1,716 (22.3%)
(Missing)	15 (0.1%)	4 (0.1%)
Depression symptoms composite, n (%)	4,473 (39.4%)	2,831 (36.7%)
(Missing)	40 (0.4%)	8 (0.1%)
Depression – feel hopeless, n (%)		
Nearly every day	1,486 (13.0%)	1,130 (14.7%)
More than half the days	1,733 (15.2%)	925 (12.0%)
Several days	4,504 (39.5%)	3,169 (41.1%)
Not at all	3,648 (32.0%)	2,485 (32.2%)
(Missing)	18 (0.2%)	3 (0.0%)
Depression – loss of interest, n (%)		
Nearly every day	1,960 (17.2%)	1,312 (17.0%)
More than half the days	1,725 (15.2%)	949 (12.3%)
Several days	4,730 (41.5%)	3,392 (44.0%)
Not at all	2,952 (25.9%)	2,055 (26.6%)
(Missing)	22 (0.2%)	5 (0.1%)
Anxiety symptoms composite, n (%)	4,062 (35.8%)	2,673 (34.7%)
(Missing)	50 (0.4%)	7 (<0.1%)
Anxiety – feel on edge, n (%)		
Nearly every day	1,527 (13.4%)	1,022 (13.2%)
More than half the days	1,567 (13.8%)	975 (12.6%)
Several days	4,202 (36.9%)	2,883 (37.4%)
Not at all	4,060 (35.7%)	2,831 (36.7%)
(Missing)	33 (0.3%)	2 (0.0%)
Anxiety – cannot stop worrying, n (%)		
Nearly every day	1,700 (14.9%)	1,280 (16.6%)
More than half the days	1,582 (13.9%)	969 (12.6%)
Several days	4,055 (35.6%)	2,742 (35.6%)
Not at all	4,034 (35.4%)	2,716 (35.2%)
(Missing)	19 (0.2%)	5 (0.1%)
Suffering, n (%)		
A lot	1,234 (10.8%)	931 (12.1%)
Some	3,978 (34.9%)	2,963 (38.4%)
Not very much	3,449 (30.3%)	2,070 (26.8%)
Not at all	2,710 (23.8%)	1,743 (22.6%)
(Missing)	18 (0.2%)	6 (0.1%)
Relationship contentment		
Mean	8.1	7.6
Standard Deviation	2.7	3.0
Min, Max	0.0, 10.0	0.0, 10.0
(Missing)	27 (0.2%)	8 (0.1%)
Relationship satisfaction		
Mean	7.7	7.2
Standard Deviation	3.0	3.2
Min, Max	0.0, 10.0	0.0, 10.0
(Missing)	18 (0.2%)	4 (<0.1%)

Table S20b. Weighted summary statistics for outcome variables in Kenya

**Outcome**	**Wave 1**<br>N = 11,389	**Wave 2**<br>N = 7,713
Social support		
Mean	7.0	6.5
Standard Deviation	3.4	3.5
Min, Max	0.0, 10.0	0.0, 10.0
(Missing)	15 (0.1%)	3 (<0.1%)
Intimate/close friend, n (%)		
Yes	9,278 (81.5%)	6,232 (80.8%)
No	2,104 (18.5%)	1,469 (19.1%)
(Missing)	7 (0.1%)	11 (0.1%)
Government approval, n (%)		
Strongly approve	2,554 (22.4%)	1,247 (16.2%)
Somewhat approve	3,359 (29.5%)	2,409 (31.2%)
Neither approve nor disapprove	1,661 (14.6%)	740 (9.6%)
Somewhat disapprove	1,312 (11.5%)	1,143 (14.8%)
Strongly disapprove	2,478 (21.8%)	2,155 (27.9%)
(Missing)	24 (0.2%)	19 (0.2%)
Say in government, n (%)		
Agree	4,334 (38.1%)	2,511 (32.6%)
Disagree	3,466 (30.4%)	2,003 (26.0%)
Unsure	3,573 (31.4%)	3,194 (41.4%)
(Missing)	16 (0.1%)	4 (0.1%)
Belonging in country		
Mean	7.8	7.4
Standard Deviation	3.1	3.3
Min, Max	0.0, 10.0	0.0, 10.0
(Missing)	30 (0.3%)	15 (0.2%)
City/place satisfaction, n (%)		
Satisfied	9,080 (79.7%)	5,406 (70.1%)
Dissatisfied	1,246 (10.9%)	1,381 (17.9%)
Unsure	1,056 (9.3%)	919 (11.9%)
(Missing)	6 (0.1%)	7 (0.1%)
Trust within country, n (%)		
All people	301 (2.6%)	181 (2.3%)
Most people	1,111 (9.8%)	544 (7.1%)
Some people	4,517 (39.7%)	3,139 (40.7%)
Not very many people	4,563 (40.1%)	3,198 (41.5%)
None	853 (7.5%)	639 (8.3%)
(Missing)	43 (0.4%)	12 (0.2%)
Number of children		
Mean	2.0	2.6
Standard Deviation	1.9	2.5
Min, Max	0.0, 20.0	0.0, 97.0
(Missing)	3 (<0.1%)	232 (3.0%)
Community participation, n (%)		
More than once a week	919 (8.1%)	692 (9.0%)
Once a week	2,245 (19.7%)	1,458 (18.9%)
One to three times a month	1,878 (16.5%)	1,172 (15.2%)
A few times a year	2,498 (21.9%)	1,666 (21.6%)
Never	3,831 (33.6%)	2,719 (35.3%)
(Missing)	18 (0.2%)	5 (0.1%)
Religious attendance, n (%)		

Table S20b. Weighted summary statistics for outcome variables in Kenya

Outcome	Wave 1<br>N = 11,389	Wave 2<br>N = 7,713
More than once a week	2,733 (24.0%)	2,076 (26.9%)
Once a week	6,069 (53.3%)	3,691 (47.9%)
One to three times a month	1,242 (10.9%)	782 (10.1%)
A few times a year	861 (7.6%)	776 (10.1%)
Never	471 (4.1%)	380 (4.9%)
(Missing)	13 (0.1%)	8 (0.1%)
Loneliness		
Mean	3.5	4.0
Standard Deviation	3.5	3.6
Min, Max	0.0, 10.0	0.0, 10.0
(Missing)	20 (0.2%)	10 (0.1%)
Perceived discrimination, n (%)		
Always	1,179 (10.4%)	699 (9.1%)
Often	2,192 (19.2%)	1,564 (20.3%)
Rarely	4,657 (40.9%)	3,419 (44.3%)
Never	3,330 (29.2%)	2,021 (26.2%)
(Missing)	30 (0.3%)	10 (0.1%)
Orientation to promote good		
Mean	8.2	7.6
Standard Deviation	2.7	3.0
Min, Max	0.0, 10.0	0.0, 10.0
(Missing)	11 (0.1%)	3 (<0.1%)
Delayed gratification		
Mean	8.1	7.7
Standard Deviation	2.8	2.9
Min, Max	0.0, 10.0	0.0, 10.0
(Missing)	12 (0.1%)	7 (<0.1%)
Hope		
Mean	8.8	8.3
Standard Deviation	2.3	2.7
Min, Max	0.0, 10.0	0.0, 10.0
(Missing)	24 (0.2%)	2 (<0.1%)
Gratitude		
Mean	8.1	7.8
Standard Deviation	2.7	2.9
Min, Max	0.0, 10.0	0.0, 10.0
(Missing)	21 (0.2%)	5 (<0.1%)
Showing love/care		
Mean	8.6	8.3
Standard Deviation	2.4	2.7
Min, Max	0.0, 10.0	0.0, 10.0
(Missing)	13 (0.1%)	5 (<0.1%)
Forgivingness, n (%)		
Always	5,323 (46.7%)	3,535 (45.8%)
Often	4,321 (37.9%)	2,991 (38.8%)
Rarely	1,460 (12.8%)	1,012 (13.1%)
Never	278 (2.4%)	167 (2.2%)
(Missing)	8 (0.1%)	7 (0.1%)
Charitable giving, n (%)		
Yes	3,143 (27.6%)	2,906 (37.7%)
No	8,237 (72.3%)	4,798 (62.2%)

Table S20b. Weighted summary statistics for outcome variables in Kenya

Outcome	Wave 1<br>N = 11,389	Wave 2<br>N = 7,713
(Missing)	9 (0.1%)	10 (0.1%)
Helping strangers, n (%)		
Yes	7,423 (65.2%)	6,256 (81.1%)
No	3,943 (34.6%)	1,442 (18.7%)
(Missing)	23 (0.2%)	15 (0.2%)
Volunteering, n (%)		
Yes	4,467 (39.2%)	4,083 (52.9%)
No	6,900 (60.6%)	3,622 (47.0%)
(Missing)	22 (0.2%)	9 (0.1%)
Self-rated physical health		
Mean	8.1	7.6
Standard Deviation	2.7	2.9
Min, Max	0.0, 10.0	0.0, 10.0
(Missing)	13 (0.1%)	6 (<0.1%)
Health problems, n (%)		
Yes	1,647 (14.5%)	1,615 (20.9%)
No	9,705 (85.2%)	6,086 (78.9%)
(Missing)	37 (0.3%)	12 (0.2%)
Pain in past 4 weeks, n (%)		
A lot	1,164 (10.2%)	1,003 (13.0%)
Some	3,905 (34.3%)	3,051 (39.6%)
Not very much	2,957 (26.0%)	1,634 (21.2%)
None at all	3,356 (29.5%)	2,023 (26.2%)
(Missing)	6 (0.1%)	2 (0.0%)
Number of cigarettes per day		
Mean	0.3	0.3
Standard Deviation	1.9	2.4
Min, Max	0.0, 97.0	0.0, 97.0
(Missing)	115 (1.0%)	37 (0.5%)
Number of drinks per week		
Mean	0.4	0.4
Standard Deviation	2.8	2.5
Min, Max	0.0, 97.0	0.0, 97.0
(Missing)	120 (1.1%)	30 (0.4%)
Days exercise per week		
Mean	2.6	2.7
Standard Deviation	2.7	2.7
Min, Max	0.0, 7.0	0.0, 7.0
(Missing)	28 (0.2%)	13 (0.2%)
Financial security		
Mean	4.8	4.9
Standard Deviation	3.8	3.7
Min, Max	0.0, 10.0	0.0, 10.0
(Missing)	10 (<0.1%)	2 (<0.1%)
Material security		
Mean	5.0	5.0
Standard Deviation	3.9	3.7
Min, Max	0.0, 10.0	0.0, 10.0
(Missing)	11 (<0.1%)	7 (<0.1%)
Educational attainment (16+ years), n (%)		
Up to 8	4,736 (41.6%)	2,904 (37.6%)

Table S20b. Weighted summary statistics for outcome variables in Kenya

Outcome	Wave 1<br>N = 11,389	Wave 2<br>N = 7,713
9-15	5,910 (51.9%)	4,225 (54.8%)
16+	739 (6.5%)	585 (7.6%)
(Missing)	5 (0.0%)	0 (0%)
Currently employed, n (%)		
Employed for an employer	1,413 (12.4%)	1,193 (15.5%)
Self-employed	3,483 (30.6%)	2,358 (30.6%)
Retired	281 (2.5%)	161 (2.1%)
Student	1,325 (11.6%)	522 (6.8%)
Homemaker	1,514 (13.3%)	956 (12.4%)
Unemployed and looking for a job	3,224 (28.3%)	2,369 (30.7%)
None of these/Other	141 (1.2%)	136 (1.8%)
(Missing)	9 (0.1%)	17 (0.2%)
Financially comfortable/getting by, n (%)		
Living comfortably on present income	705 (6.2%)	461 (6.0%)
Getting by on present income	2,321 (20.4%)	1,494 (19.4%)
Finding it difficult on present income	5,205 (45.7%)	3,833 (49.7%)
Finding it very difficult on present income	3,130 (27.5%)	1,910 (24.8%)
(Missing)	27 (0.2%)	17 (0.2%)
Own home, n (%)		
Someone in this household owns this home	5,704 (50.1%)	3,670 (47.6%)
Someone in this household rents this home	4,021 (35.3%)	3,086 (40.0%)
Both	505 (4.4%)	366 (4.8%)
Neither	1,136 (10.0%)	574 (7.4%)
Rent	0 (0%)	0 (0%)
Own	0 (0%)	0 (0%)
Something else	0 (0%)	0 (0%)
(Missing)	23 (0.2%)	17 (0.2%)
Religious/spiritual connection, n (%)		
Always	6,077 (53.4%)	4,207 (54.5%)
Often	3,086 (27.1%)	2,029 (26.3%)
Rarely	1,770 (15.5%)	1,145 (14.8%)
Never	450 (4.0%)	327 (4.2%)
(Missing)	6 (0.1%)	5 (0.1%)
Belief in life after death, n (%)		
Yes	8,112 (71.2%)	5,290 (68.6%)
No	1,515 (13.3%)	865 (11.2%)
Unsure	1,738 (15.3%)	1,548 (20.1%)
(Missing)	24 (0.2%)	11 (0.1%)
Transformative religious experience, n (%)		
Yes	7,344 (64.5%)	5,056 (65.5%)
No	3,947 (34.7%)	2,628 (34.1%)
(Missing)	98 (0.9%)	30 (0.4%)
Religious reading or listening, n (%)		
More than once a day	3,239 (28.4%)	2,315 (30.0%)
About once a day	4,051 (35.6%)	2,600 (33.7%)
Sometimes	3,567 (31.3%)	2,446 (31.7%)
Never	512 (4.5%)	346 (4.5%)
(Missing)	20 (0.2%)	6 (0.1%)
Prayer or meditation, n (%)		
More than once a day	6,027 (52.9%)	4,097 (53.1%)
About once a day	3,511 (30.8%)	2,349 (30.5%)

Table S20b. Weighted summary statistics for outcome variables in Kenya

**Outcome**	**Wave 1**<br>N = 11,389	**Wave 2**<br>N = 7,713
Sometimes	1,601 (14.1%)	1,087 (14.1%)
Never	233 (2.0%)	174 (2.3%)
(Missing)	18 (0.2%)	6 (0.1%)
Belief in God/gods/spiritual forces, n (%)		
One God	10,895 (95.7%)	7,414 (96.1%)
More than one god	158 (1.4%)	57 (0.7%)
An impersonal spiritual force	148 (1.3%)	66 (0.9%)
None of these	57 (0.5%)	45 (0.6%)
Unsure	120 (1.1%)	126 (1.6%)
(Missing)	11 (0.1%)	5 (0.1%)
Religious centrality, n (%)		
Agree	9,204 (80.8%)	6,132 (79.5%)
Disagree	777 (6.8%)	414 (5.4%)
Not relevant	374 (3.3%)	187 (2.4%)
Unsure	1,025 (9.0%)	978 (12.7%)
(Missing)	8 (0.1%)	2 (0.0%)
Religious/spiritual comfort, n (%)		
Agree	9,975 (87.6%)	6,790 (88.0%)
Disagree	551 (4.8%)	248 (3.2%)
Not relevant	343 (3.0%)	125 (1.6%)
Unsure	514 (4.5%)	548 (7.1%)
(Missing)	6 (0.1%)	2 (0.0%)
Feel loved by God, n (%)		
Agree	10,528 (92.4%)	7,328 (95.0%)
Disagree	347 (3.0%)	81 (1.0%)
Not relevant	247 (2.2%)	70 (0.9%)
Unsure	260 (2.3%)	230 (3.0%)
(Missing)	7 (0.1%)	5 (0.1%)
Feel punished by God, n (%)		
Agree	4,867 (42.7%)	3,660 (47.4%)
Disagree	4,311 (37.8%)	2,452 (31.8%)
Not relevant	731 (6.4%)	301 (3.9%)
Unsure	1,460 (12.8%)	1,293 (16.8%)
(Missing)	20 (0.2%)	7 (0.1%)
Experienced religious criticism, n (%)		
Agree	5,881 (51.6%)	3,920 (50.8%)
Disagree	3,070 (27.0%)	1,830 (23.7%)
Not relevant	787 (6.9%)	450 (5.8%)
Unsure	1,637 (14.4%)	1,509 (19.6%)
(Missing)	15 (0.1%)	4 (0.1%)
Faith-sharing, n (%)		
Agree	9,244 (81.2%)	6,331 (82.1%)
Disagree	1,037 (9.1%)	504 (6.5%)
Not relevant	585 (5.1%)	327 (4.2%)
Unsure	516 (4.5%)	547 (7.1%)
(Missing)	7 (0.1%)	4 (0.1%)

Note. N (%); this table is based on non-imputed data. Cumulative percentages for variables may not add up to 100% due to rounding. Wave 1 characteristics weighted using the Gallup provided sampling weight, ANNUAL_WEIGHT_R2; Wave 2 characteristics weighted accounting for attrition by using the adjusted Wave 1 weight, ANNUAL_WEIGHT_R2, multiplied by the created attrition weight to account for dropout, to maintain nationally representative estimates for Wave 2 characteristics.

Table S20c. Unweighted summary statistics for demographic and childhood variables in Kenya by retention status

Characteristic	Attritors–Not Observed in Wave 2<br>N = 3,696	Retained–Observed in Wave 2<br>N = 7,728
Forgivingness, n (%)		
Always	1,659 (44.9%)	3,682 (47.6%)
Often	1,421 (38.4%)	2,913 (37.7%)
Rarely	508 (13.8%)	955 (12.4%)
Never	102 (2.8%)	176 (2.3%)
(Missing)	5 (0.1%)	2 (0.0%)
Year of birth, n (%)		
1943 or earlier (current age: 80+ years)	43 (1.2%)	20 (0.3%)
1943-1953 (current age: 70-79 years)	138 (3.7%)	189 (2.4%)
1953-1963 (current age: 60-69 years)	198 (5.3%)	472 (6.1%)
1963-1973 (current age: 50-59 years)	314 (8.5%)	700 (9.1%)
1973-1983 (current age: 40-49 years)	413 (11.2%)	1,216 (15.7%)
1983-1993 (current age: 30-39 years)	752 (20.4%)	1,686 (21.8%)
1993-1998 (current age: 25-29 years)	583 (15.8%)	1,290 (16.7%)
1998-2005 (current age: 18-24 years)	1,256 (34.0%)	2,151 (27.8%)
(Missing)	0 (0%)	5 (0.1%)
Age of participant		
Mean	35.0	35.6
Standard Deviation	16.0	14.4
Min, Max	18.0, 97.0	18.0, 92.0
(Missing)	0 (0%)	5 (<0.1%)
Gender, n (%)		
Male	1,648 (44.6%)	3,930 (50.9%)
Female	2,042 (55.3%)	3,794 (49.1%)
Other	2 (0.0%)	0 (<0.0%)
(Missing)	4 (0.1%)	3 (0.0%)
Respondent marital status, n (%)		
Single/Never been married	1,298 (35.1%)	2,493 (32.3%)
Married	1,988 (53.8%)	4,440 (57.5%)
Separated	145 (3.9%)	315 (4.1%)
Divorced	47 (1.3%)	59 (0.8%)
Widowed	140 (3.8%)	302 (3.9%)
Domestic partner	59 (1.6%)	92 (1.2%)
(Missing)	19 (0.5%)	27 (0.3%)
Education (years), n (%)		
Up to 8	1,746 (47.3%)	2,995 (38.8%)
9-15	1,738 (47.0%)	4,197 (54.3%)
16+	206 (5.6%)	536 (6.9%)
(Missing)	5 (0.1%)	0 (<0.0%)
Employment status, n (%)		
Employed for an employer	448 (12.1%)	970 (12.5%)
Self-employed	1,094 (29.6%)	2,401 (31.1%)
Retired	89 (2.4%)	193 (2.5%)
Student	441 (11.9%)	888 (11.5%)
Homemaker	551 (14.9%)	965 (12.5%)
Unemployed and looking for a job	1,002 (27.1%)	2,233 (28.9%)
None of these/Other	63 (1.7%)	77 (1.0%)
(Missing)	7 (0.2%)	2 (0.0%)
Current religious service attendance, n (%)		

Table S20c. Unweighted summary statistics for demographic and childhood variables in Kenya by retention status

**Characteristic**	**Attritors–Not Observed in Wave 2** N = 3,696	**Retained–Observed in Wave 2** N = 7,728
More than once a week	868 (23.5%)	1,874 (24.3%)
Once a week	1,912 (51.7%)	4,179 (54.1%)
One to three times a month	422 (11.4%)	824 (10.7%)
A few times a year	296 (8.0%)	566 (7.3%)
Never	193 (5.2%)	278 (3.6%)
(Missing)	5 (0.1%)	8 (0.1%)
Immigration status, n (%)		
Born in this country	3,634 (98.3%)	7,670 (99.2%)
Born in another country	61 (1.7%)	58 (0.7%)
(Missing)	1 (0.0%)	1 (<0.0%)
Parental marital status around age 12, n (%)		
Parents were married	2,977 (80.5%)	6,277 (81.2%)
Parents were divorced	245 (6.6%)	469 (6.1%)
Parents were never married	237 (6.4%)	446 (5.8%)
One or both of them had died	124 (3.4%)	341 (4.4%)
Unsure	38 (1.0%)	72 (0.9%)
(Missing)	74 (2.0%)	122 (1.6%)
Religious service attendance around age 12, n (%)		
At least once a week	2,881 (77.9%)	6,308 (81.6%)
One to three times a month	617 (16.7%)	1,091 (14.1%)
Less than once a month	72 (1.9%)	175 (2.3%)
Never	86 (2.3%)	116 (1.5%)
(Missing)	40 (1.1%)	39 (0.5%)
Relationship with mother when growing up, n (%)		
Very good	2,991 (80.9%)	6,440 (83.3%)
Somewhat good	481 (13.0%)	969 (12.5%)
Somewhat bad	59 (1.6%)	75 (1.0%)
Very bad	47 (1.3%)	60 (0.8%)
(Does not apply)	87 (2.4%)	149 (1.9%)
(Missing)	30 (0.8%)	36 (0.5%)
Relationship with father when growing up, n (%)		
Very good	2,580 (69.8%)	5,382 (69.6%)
Somewhat good	597 (16.2%)	1,316 (17.0%)
Somewhat bad	71 (1.9%)	149 (1.9%)
Very bad	76 (2.1%)	149 (1.9%)
(Does not apply)	323 (8.7%)	649 (8.4%)
(Missing)	48 (1.3%)	83 (1.1%)
Felt like an outsider in family when growing up, n (%)		
Yes	498 (13.5%)	751 (9.7%)
No	3,181 (86.1%)	6,942 (89.8%)
(Missing)	17 (0.5%)	35 (0.5%)
Experienced abuse when growing up, n (%)		
Yes	452 (12.2%)	870 (11.3%)
No	3,228 (87.3%)	6,826 (88.3%)
(Missing)	16 (0.4%)	32 (0.4%)
Self-rated health when growing up, n (%)		
Excellent	1,444 (39.1%)	3,028 (39.2%)

Table S20c. Unweighted summary statistics for demographic and childhood variables in Kenya by retention status

Characteristic	Attritors–Not Observed in Wave 2<br>N = 3,696	Retained–Observed in Wave 2<br>N = 7,728
Very good	887 (24.0%)	1,731 (22.4%)
Good	804 (21.7%)	1,758 (22.7%)
Fair	419 (11.3%)	971 (12.6%)
Poor	126 (3.4%)	231 (3.0%)
(Missing)	15 (0.4%)	9 (0.1%)
Subjective financial status of family growing up, n (%)		
Lived comfortably	1,032 (27.9%)	2,014 (26.1%)
Got by	984 (26.6%)	2,278 (29.5%)
Found it difficult	1,277 (34.5%)	2,823 (36.5%)
Found it very difficult	393 (10.6%)	604 (7.8%)
(Missing)	10 (0.3%)	8 (0.1%)
Religious affiliation growing up, n (%)		
Christianity	3,287 (88.9%)	7,099 (91.9%)
Taoism	0 (0%)	0 (0%)
Confucianism	0 (0%)	0 (0%)
Primal, Animist, or Folk religion	6 (0.2%)	6 (0.1%)
Spiritism	0 (0%)	0 (0%)
Umbanda, Candomblé, and other African-derived religions	0 (0%)	0 (0%)
Chinese folk/traditional religion	0 (0%)	0 (0%)
Islam	373 (10.1%)	562 (7.3%)
Hinduism	0 (0%)	0 (0%)
Buddhism	4 (0.1%)	1 (0.0%)
Judaism	0 (0%)	6 (0.1%)
Sikhism	0 (0%)	0 (<0.0%)
Baha'i	0 (0%)	4 (0.0%)
Jainism	0 (0%)	1 (0.0%)
Shinto	0 (0%)	0 (0%)
Some other religion	1 (0.0%)	0 (0%)
No religion/Atheist/Agnostic	24 (0.6%)	41 (0.5%)
(Missing)	2 (0.0%)	7 (0.1%)

Note. N (%); this table is based on non-imputed data. Cumulative percentages for variables may not add up to 100% due to rounding.

Table S20d. Unweighted summary statistics for Wave 1 outcome variables in Kenya by retention status.

**Outcome**	**Attritors–Not Observed in Wave 2** N = 3,696	**Retained–Observed in Wave 2** N = 7,728
Secure flourishing index		
Mean	7.4	7.3
Standard Deviation	1.6	1.6
Min, Max	0.8, 10.0	0.0, 10.0
(Missing)	46 (1.2%)	119 (1.5%)
Flourishing index		
Mean	7.8	7.8
Standard Deviation	1.7	1.6
Min, Max	0.7, 10.0	0.0, 10.0
(Missing)	40 (1.1%)	106 (1.4%)
Happiness & life satisfaction		
Mean	6.8	6.6
Standard Deviation	2.7	2.7
Min, Max	0.0, 10.0	0.0, 10.0
(Missing)	8 (0.2%)	15 (0.2%)
Physical & mental health		
Mean	8.4	8.4
Standard Deviation	2.1	2.0
Min, Max	0.0, 10.0	0.0, 10.0
(Missing)	5 (0.1%)	22 (0.3%)
Meaning & purpose		
Mean	7.9	7.9
Standard Deviation	2.3	2.2
Min, Max	0.0, 10.0	0.0, 10.0
(Missing)	8 (0.2%)	38 (0.5%)
Character & virtue		
Mean	8.1	8.1
Standard Deviation	2.3	2.2
Min, Max	0.0, 10.0	0.0, 10.0
(Missing)	7 (0.2%)	14 (0.2%)
Close social relationships		
Mean	8.0	7.8
Standard Deviation	2.4	2.4
Min, Max	0.0, 10.0	0.0, 10.0
(Missing)	14 (0.4%)	30 (0.4%)
Financial & material security		
Mean	5.1	4.8
Standard Deviation	3.4	3.4
Min, Max	0.0, 10.0	0.0, 10.0
(Missing)	6 (0.2%)	15 (0.2%)
Happiness		
Mean	7.5	7.2
Standard Deviation	3.0	2.9
Min, Max	0.0, 10.0	0.0, 10.0
(Missing)	3 (<0.1%)	9 (0.1%)
Life satisfaction		
Mean	6.1	5.9
Standard Deviation	3.6	3.5
Min, Max	0.0, 10.0	0.0, 10.0

Table S20d. Unweighted summary statistics for Wave 1 outcome variables in Kenya by retention status.

Outcome	Attritors–Not Observed in Wave 2<br>N = 3,696	Retained–Observed in Wave 2<br>N = 7,728
(Missing)	5 (0.1%)	10 (0.1%)
Current life evaluation		
Mean	5.6	5.5
Standard Deviation	3.4	3.2
Min, Max	0.0, 10.0	0.0, 10.0
(Missing)	12 (0.3%)	6 (<0.1%)
Future life evaluation		
Mean	7.5	7.5
Standard Deviation	2.9	2.8
Min, Max	0.0, 10.0	0.0, 10.0
(Missing)	47 (1.3%)	74 (1.0%)
Optimism		
Mean	8.9	8.9
Standard Deviation	2.3	2.2
Min, Max	0.0, 10.0	0.0, 10.0
(Missing)	3 (<0.1%)	7 (<0.1%)
Freedom to pursue what's important		
Mean	8.5	8.4
Standard Deviation	2.5	2.6
Min, Max	0.0, 10.0	0.0, 10.0
(Missing)	2 (<0.1%)	9 (0.1%)
Inner peace, n (%)		
Always	977 (26.4%)	1,999 (25.9%)
Often	1,274 (34.5%)	2,688 (34.8%)
Rarely	1,208 (32.7%)	2,608 (33.7%)
Never	229 (6.2%)	418 (5.4%)
(Missing)	8 (0.2%)	16 (0.2%)
Life balance, n (%)		
Always	825 (22.3%)	1,645 (21.3%)
Often	1,175 (31.8%)	2,502 (32.4%)
Rarely	1,463 (39.6%)	3,114 (40.3%)
Never	227 (6.2%)	459 (5.9%)
(Missing)	5 (0.1%)	9 (0.1%)
Sense of mastery, n (%)		
Always	1,337 (36.2%)	2,762 (35.7%)
Often	1,242 (33.6%)	2,571 (33.3%)
Rarely	935 (25.3%)	2,068 (26.8%)
Never	177 (4.8%)	310 (4.0%)
(Missing)	6 (0.2%)	19 (0.2%)
Meaningful activities		
Mean	7.3	7.2
Standard Deviation	3.2	3.1
Min, Max	0.0, 10.0	0.0, 10.0
(Missing)	5 (0.1%)	13 (0.2%)
Understanding purpose		
Mean	8.4	8.5
Standard Deviation	2.6	2.5
Min, Max	0.0, 10.0	0.0, 10.0
(Missing)	3 (<0.1%)	26 (0.3%)

Table S20d. Unweighted summary statistics for Wave 1 outcome variables in Kenya by retention status.

Outcome	Attritors–Not Observed in Wave 2<br>N = 3,696	Retained–Observed in Wave 2<br>N = 7,728
Self-rated mental health		
Mean	8.8	8.7
Standard Deviation	2.3	2.2
Min, Max	0.0, 10.0	0.0, 10.0
(Missing)	2 (<0.1%)	15 (0.2%)
Traumatic distress, n (%)		
A lot	529 (14.3%)	1,171 (15.1%)
Some	1,065 (28.8%)	2,552 (33.0%)
Not very much	1,076 (29.1%)	2,123 (27.5%)
Not at all	1,024 (27.7%)	1,870 (24.2%)
(Missing)	2 (0.1%)	13 (0.2%)
Depression symptoms composite, n (%)	1,364 (37.0%)	3,127 (40.6%)
(Missing)	8 (0.2%)	32 (0.4%)
Depression – feel hopeless, n (%)		
Nearly every day	446 (12.1%)	1,045 (13.5%)
More than half the days	522 (14.1%)	1,218 (15.8%)
Several days	1,471 (39.8%)	3,046 (39.4%)
Not at all	1,255 (34.0%)	2,402 (31.1%)
(Missing)	2 (0.0%)	17 (0.2%)
Depression – loss of interest, n (%)		
Nearly every day	576 (15.6%)	1,392 (18.0%)
More than half the days	518 (14.0%)	1,215 (15.7%)
Several days	1,549 (41.9%)	3,195 (41.3%)
Not at all	1,046 (28.3%)	1,911 (24.7%)
(Missing)	7 (0.2%)	15 (0.2%)
Anxiety symptoms composite, n (%)	1,264 (34.4%)	2,812 (36.5%)
(Missing)	20 (0.6%)	29 (0.4%)
Anxiety – feel on edge, n (%)		
Nearly every day	463 (12.5%)	1,069 (13.8%)
More than half the days	469 (12.7%)	1,104 (14.3%)
Several days	1,403 (38.0%)	2,810 (36.4%)
Not at all	1,347 (36.4%)	2,725 (35.3%)
(Missing)	14 (0.4%)	19 (0.2%)
Anxiety – cannot stop worrying, n (%)		
Nearly every day	516 (14.0%)	1,191 (15.4%)
More than half the days	530 (14.3%)	1,057 (13.7%)
Several days	1,304 (35.3%)	2,764 (35.8%)
Not at all	1,339 (36.2%)	2,706 (35.0%)
(Missing)	8 (0.2%)	11 (0.1%)
Suffering, n (%)		
A lot	425 (11.5%)	812 (10.5%)
Some	1,165 (31.5%)	2,830 (36.6%)
Not very much	1,127 (30.5%)	2,332 (30.2%)
Not at all	969 (26.2%)	1,746 (22.6%)
(Missing)	10 (0.3%)	8 (0.1%)
Relationship contentment		
Mean	8.1	8.1
Standard Deviation	2.7	2.7
Min, Max	0.0, 10.0	0.0, 10.0

Table S20d. Unweighted summary statistics for Wave 1 outcome variables in Kenya by retention status.

**Outcome**	**Attritors–Not Observed in Wave 2** N = 3,696	**Retained–Observed in Wave 2** N = 7,728
(Missing)	10 (0.3%)	16 (0.2%)
Relationship satisfaction		
Mean	7.8	7.6
Standard Deviation	3.0	3.0
Min, Max	0.0, 10.0	0.0, 10.0
(Missing)	4 (0.1%)	15 (0.2%)
Social support		
Mean	7.1	6.9
Standard Deviation	3.4	3.4
Min, Max	0.0, 10.0	0.0, 10.0
(Missing)	4 (<0.1%)	12 (0.2%)
Intimate/close friend, n (%)		
Yes	2,922 (79.1%)	6,388 (82.7%)
No	771 (20.9%)	1,336 (17.3%)
(Missing)	2 (0.1%)	5 (0.1%)
Government approval, n (%)		
Strongly approve	896 (24.2%)	1,664 (21.5%)
Somewhat approve	1,033 (27.9%)	2,339 (30.3%)
Neither approve nor disapprove	571 (15.4%)	1,094 (14.2%)
Somewhat disapprove	371 (10.0%)	947 (12.3%)
Strongly disapprove	819 (22.2%)	1,665 (21.6%)
(Missing)	6 (0.2%)	19 (0.2%)
Say in government, n (%)		
Agree	1,402 (37.9%)	2,945 (38.1%)
Disagree	1,091 (29.5%)	2,387 (30.9%)
Unsure	1,196 (32.3%)	2,387 (30.9%)
(Missing)	7 (0.2%)	9 (0.1%)
Belonging in country		
Mean	7.8	7.8
Standard Deviation	3.1	3.0
Min, Max	0.0, 10.0	0.0, 10.0
(Missing)	14 (0.4%)	15 (0.2%)
City/place satisfaction, n (%)		
Satisfied	2,950 (79.8%)	6,158 (79.7%)
Dissatisfied	387 (10.5%)	864 (11.2%)
Unsure	357 (9.7%)	702 (9.1%)
(Missing)	2 (0.0%)	5 (0.1%)
Trust within country, n (%)		
All people	116 (3.1%)	185 (2.4%)
Most people	402 (10.9%)	711 (9.2%)
Some people	1,424 (38.5%)	3,109 (40.2%)
Not very many people	1,467 (39.7%)	3,111 (40.3%)
None	278 (7.5%)	577 (7.5%)
(Missing)	9 (0.3%)	34 (0.4%)
Number of children		
Mean	2.0	2.0
Standard Deviation	1.9	1.8
Min, Max	0.0, 15.0	0.0, 20.0
(Missing)	1 (<0.1%)	2 (<0.1%)

Table S20d. Unweighted summary statistics for Wave 1 outcome variables in Kenya by retention status.

**Outcome**	**Attritors–Not Observed in Wave 2** N = 3,696	**Retained–Observed in Wave 2** N = 7,728
Community participation, n (%)		
More than once a week	293 (7.9%)	628 (8.1%)
Once a week	694 (18.8%)	1,560 (20.2%)
One to three times a month	583 (15.8%)	1,301 (16.8%)
A few times a year	809 (21.9%)	1,697 (22.0%)
Never	1,310 (35.4%)	2,530 (32.7%)
(Missing)	7 (0.2%)	11 (0.1%)
Religious attendance, n (%)		
More than once a week	868 (23.5%)	1,874 (24.3%)
Once a week	1,912 (51.7%)	4,179 (54.1%)
One to three times a month	422 (11.4%)	824 (10.7%)
A few times a year	296 (8.0%)	566 (7.3%)
Never	193 (5.2%)	278 (3.6%)
(Missing)	5 (0.1%)	8 (0.1%)
Loneliness		
Mean	3.4	3.6
Standard Deviation	3.5	3.5
Min, Max	0.0, 10.0	0.0, 10.0
(Missing)	9 (0.3%)	10 (0.1%)
Perceived discrimination, n (%)		
Always	405 (11.0%)	777 (10.1%)
Often	707 (19.1%)	1,493 (19.3%)
Rarely	1,458 (39.4%)	3,215 (41.6%)
Never	1,120 (30.3%)	2,219 (28.7%)
(Missing)	7 (0.2%)	24 (0.3%)
Orientation to promote good		
Mean	8.2	8.2
Standard Deviation	2.7	2.6
Min, Max	0.0, 10.0	0.0, 10.0
(Missing)	1 (<0.1%)	11 (0.1%)
Delayed gratification		
Mean	8.0	8.1
Standard Deviation	2.9	2.7
Min, Max	0.0, 10.0	0.0, 10.0
(Missing)	6 (0.2%)	6 (<0.1%)
Hope		
Mean	8.7	8.8
Standard Deviation	2.5	2.3
Min, Max	0.0, 10.0	0.0, 10.0
(Missing)	4 (0.1%)	21 (0.3%)
Gratitude		
Mean	8.0	8.1
Standard Deviation	2.8	2.7
Min, Max	0.0, 10.0	0.0, 10.0
(Missing)	7 (0.2%)	14 (0.2%)
Showing love/care		
Mean	8.5	8.6
Standard Deviation	2.6	2.4
Min, Max	0.0, 10.0	0.0, 10.0

Table S20d. Unweighted summary statistics for Wave 1 outcome variables in Kenya by retention status.

**Outcome**	**Attritors–Not Observed in Wave 2** N = 3,696	**Retained–Observed in Wave 2** N = 7,728
(Missing)	5 (0.1%)	7 (<0.1%)
Forgivingness, n (%)		
Always	1,659 (44.9%)	3,682 (47.6%)
Often	1,421 (38.4%)	2,913 (37.7%)
Rarely	508 (13.8%)	955 (12.4%)
Never	102 (2.8%)	176 (2.3%)
(Missing)	5 (0.1%)	2 (0.0%)
Charitable giving, n (%)		
Yes	1,034 (28.0%)	2,118 (27.4%)
No	2,658 (71.9%)	5,605 (72.5%)
(Missing)	4 (0.1%)	5 (0.1%)
Helping strangers, n (%)		
Yes	2,313 (62.6%)	5,137 (66.5%)
No	1,375 (37.2%)	2,577 (33.3%)
(Missing)	8 (0.2%)	14 (0.2%)
Volunteering, n (%)		
Yes	1,417 (38.3%)	3,065 (39.7%)
No	2,273 (61.5%)	4,647 (60.1%)
(Missing)	6 (0.2%)	16 (0.2%)
Self-rated physical health		
Mean	8.1	8.1
Standard Deviation	2.7	2.6
Min, Max	0.0, 10.0	0.0, 10.0
(Missing)	3 (<0.1%)	10 (0.1%)
Health problems, n (%)		
Yes	567 (15.3%)	1,084 (14.0%)
No	3,112 (84.2%)	6,624 (85.7%)
(Missing)	17 (0.5%)	20 (0.3%)
Pain in past 4 weeks, n (%)		
A lot	353 (9.6%)	815 (10.5%)
Some	1,228 (33.2%)	2,691 (34.8%)
Not very much	955 (25.9%)	2,011 (26.0%)
None at all	1,157 (31.3%)	2,207 (28.6%)
(Missing)	2 (0.1%)	4 (0.1%)
Number of cigarettes per day		
Mean	0.3	0.3
Standard Deviation	2.0	1.8
Min, Max	0.0, 97.0	0.0, 97.0
(Missing)	45 (1.2%)	70 (0.9%)
Number of drinks per week		
Mean	0.5	0.4
Standard Deviation	3.4	2.5
Min, Max	0.0, 97.0	0.0, 97.0
(Missing)	46 (1.2%)	74 (1.0%)
Days exercise per week		
Mean	2.6	2.7
Standard Deviation	2.7	2.7
Min, Max	0.0, 7.0	0.0, 7.0
(Missing)	10 (0.3%)	18 (0.2%)

Table S20d. Unweighted summary statistics for Wave 1 outcome variables in Kenya by retention status.

**Outcome**	**Attritors–Not Observed in Wave 2** N = 3,696	**Retained–Observed in Wave 2** N = 7,728
Financial security		
Mean	5.0	4.7
Standard Deviation	3.9	3.8
Min, Max	0.0, 10.0	0.0, 10.0
(Missing)	6 (0.2%)	4 (<0.1%)
Material security		
Mean	5.2	4.9
Standard Deviation	3.9	3.8
Min, Max	0.0, 10.0	0.0, 10.0
(Missing)	0 (<0.1%)	11 (0.1%)
Educational attainment (16+ years), n (%)		
Up to 8	1,746 (47.3%)	2,995 (38.8%)
9-15	1,738 (47.0%)	4,197 (54.3%)
16+	206 (5.6%)	536 (6.9%)
(Missing)	5 (0.1%)	0 (<0.0%)
Currently employed, n (%)		
Employed for an employer	448 (12.1%)	970 (12.5%)
Self-employed	1,094 (29.6%)	2,401 (31.1%)
Retired	89 (2.4%)	193 (2.5%)
Student	441 (11.9%)	888 (11.5%)
Homemaker	551 (14.9%)	965 (12.5%)
Unemployed and looking for a job	1,002 (27.1%)	2,233 (28.9%)
None of these/Other	63 (1.7%)	77 (1.0%)
(Missing)	7 (0.2%)	2 (0.0%)
Financially comfortable/getting by, n (%)		
Living comfortably on present income	266 (7.2%)	440 (5.7%)
Getting by on present income	705 (19.1%)	1,625 (21.0%)
Finding it difficult on present income	1,720 (46.5%)	3,500 (45.3%)
Finding it very difficult on present income	992 (26.8%)	2,149 (27.8%)
(Missing)	13 (0.4%)	14 (0.2%)
Own home, n (%)		
Someone in this household owns this home	1,830 (49.5%)	3,893 (50.4%)
Someone in this household rents this home	1,291 (34.9%)	2,742 (35.5%)
Both	165 (4.5%)	341 (4.4%)
Neither	401 (10.8%)	738 (9.5%)
Rent	0 (0%)	0 (0%)
Own	0 (0%)	0 (0%)
Something else	0 (0%)	0 (0%)
(Missing)	9 (0.2%)	14 (0.2%)
Religious/spiritual connection, n (%)		
Always	1,902 (51.5%)	4,197 (54.3%)
Often	993 (26.9%)	2,102 (27.2%)
Rarely	630 (17.0%)	1,143 (14.8%)
Never	171 (4.6%)	279 (3.6%)
(Missing)	0 (<0.0%)	6 (0.1%)
Belief in life after death, n (%)		
Yes	2,544 (68.8%)	5,597 (72.4%)
No	508 (13.7%)	1,012 (13.1%)
Unsure	632 (17.1%)	1,108 (14.3%)

Table S20d. Unweighted summary statistics for Wave 1 outcome variables in Kenya by retention status.

Outcome	Attritors–Not Observed in Wave 2<br>N = 3,696	Retained–Observed in Wave 2<br>N = 7,728
(Missing)	12 (0.3%)	12 (0.2%)
Transformative religious experience, n (%)		
Yes	2,329 (63.0%)	5,039 (65.2%)
No	1,334 (36.1%)	2,624 (33.9%)
(Missing)	33 (0.9%)	65 (0.8%)
Religious reading or listening, n (%)		
More than once a day	994 (26.9%)	2,257 (29.2%)
About once a day	1,331 (36.0%)	2,731 (35.3%)
Sometimes	1,162 (31.4%)	2,415 (31.3%)
Never	205 (5.6%)	307 (4.0%)
(Missing)	3 (0.1%)	17 (0.2%)
Prayer or meditation, n (%)		
More than once a day	1,850 (50.0%)	4,200 (54.3%)
About once a day	1,166 (31.5%)	2,354 (30.5%)
Sometimes	583 (15.8%)	1,020 (13.2%)
Never	92 (2.5%)	141 (1.8%)
(Missing)	5 (0.1%)	13 (0.2%)
Belief in God/gods/spiritual forces, n (%)		
One God	3,510 (95.0%)	7,419 (96.0%)
More than one god	66 (1.8%)	92 (1.2%)
An impersonal spiritual force	52 (1.4%)	96 (1.2%)
None of these	18 (0.5%)	39 (0.5%)
Unsure	48 (1.3%)	72 (0.9%)
(Missing)	1 (0.0%)	9 (0.1%)
Religious centrality, n (%)		
Agree	2,943 (79.6%)	6,291 (81.4%)
Disagree	246 (6.7%)	533 (6.9%)
Not relevant	142 (3.8%)	232 (3.0%)
Unsure	362 (9.8%)	665 (8.6%)
(Missing)	2 (0.1%)	7 (0.1%)
Religious/spiritual comfort, n (%)		
Agree	3,220 (87.1%)	6,787 (87.8%)
Disagree	184 (5.0%)	368 (4.8%)
Not relevant	125 (3.4%)	218 (2.8%)
Unsure	167 (4.5%)	349 (4.5%)
(Missing)	0 (0%)	6 (0.1%)
Feel loved by God, n (%)		
Agree	3,398 (91.9%)	7,164 (92.7%)
Disagree	122 (3.3%)	226 (2.9%)
Not relevant	91 (2.5%)	157 (2.0%)
Unsure	83 (2.3%)	178 (2.3%)
(Missing)	2 (0.1%)	4 (0.1%)
Feel punished by God, n (%)		
Agree	1,613 (43.7%)	3,267 (42.3%)
Disagree	1,378 (37.3%)	2,946 (38.1%)
Not relevant	257 (6.9%)	476 (6.2%)
Unsure	444 (12.0%)	1,022 (13.2%)
(Missing)	4 (0.1%)	16 (0.2%)
Experienced religious criticism, n (%)		

Table S20d. Unweighted summary statistics for Wave 1 outcome variables in Kenya by retention status.

Outcome	Attritors–Not Observed in Wave 2<br>N = 3,696	Retained–Observed in Wave 2<br>N = 7,728
Agree	1,984 (53.7%)	3,912 (50.6%)
Disagree	951 (25.7%)	2,129 (27.6%)
Not relevant	270 (7.3%)	519 (6.7%)
Unsure	488 (13.2%)	1,156 (15.0%)
(Missing)	4 (0.1%)	12 (0.2%)
Faith-sharing, n (%)		
Agree	2,923 (79.1%)	6,352 (82.2%)
Disagree	366 (9.9%)	673 (8.7%)
Not relevant	218 (5.9%)	367 (4.8%)
Unsure	188 (5.1%)	329 (4.3%)
(Missing)	1 (0.0%)	7 (0.1%)

Note. N (%); this table is based on non-imputed data. Cumulative percentages for variables may not add up to 100% due to rounding.

Table S20e. Summary of fitted attrition model in Kenya

Characteristic	Odds Ratio	95% CI	p-value
**ANNUAL_WEIGHT_R2**	0.92	0.84, 1.00	0.045
**Happiness & life satisfaction**	1.00	0.94, 1.06	0.990
**Physical & mental health**	0.98	0.93, 1.05	0.597
**Meaning & purpose**	1.03	0.97, 1.09	0.316
**Character & virtue**	1.05	1.00, 1.11	0.076
**Close social relationships**	0.93	0.88, 0.99	0.026
**Financial & material security**	0.95	0.90, 1.01	0.088
**Extraversion**	0.98	0.92, 1.03	0.367
**Openness to experience**	1.13	1.07, 1.19	1.00e-05
**Agreeableness**	0.99	0.94, 1.05	0.797
**Conscientiousness**	1.14	1.08, 1.21	6.76e-06
**Neuroticism**	1.07	1.01, 1.13	0.024
**Depression symptoms composite**	1.06	1.00, 1.12	0.042
**Anxiety symptoms composite**	1.00	0.95, 1.06	0.985
**Loneliness**	1.07	1.01, 1.12	0.012
**Days exercise per week**	0.99	0.94, 1.05	0.744
**Year of birth (age group)**			
1998-2005 (current age: 18-24 years)	—	—	
1983-1993 (current age: 30-39 years)	1.45	1.23, 1.71	1.41e-05
1993-1998 (current age: 25-29 years)	1.32	1.14, 1.54	3.94e-04
1973-1983 (current age: 40-49 years)	2.10	1.73, 2.56	3.39e-12
1963-1973 (current age: 50-59 years)	1.66	1.30, 2.13	8.64e-05
1953-1963 (current age: 60-69 years)	1.87	1.30, 2.68	8.02e-04
1943-1953 (current age: 70-79 years)	1.14	0.74, 1.77	0.541
1943 or earlier (current age: 80+ years)	0.33	0.15, 0.75	0.008
**Gender of respondent**			
Female	—	—	
Male	1.28	1.14, 1.44	4.56e-05
Other	0.09	0.01, 1.56	0.098
**Marital status**			
Married	—	—	
Single/Never been married	0.91	0.79, 1.04	0.162
Separated	1.0	0.76, 1.29	0.970
Widowed	1.31	0.95, 1.80	0.101
Domestic partner	0.76	0.51, 1.14	0.189
Divorced	0.53	0.33, 0.85	0.009
**Employment status**			
Self-employed	—	—	
Unemployed and looking for a job	1.20	1.05, 1.38	0.009
Employed for an employer	0.98	0.83, 1.16	0.799
Homemaker	0.99	0.84, 1.18	0.944
Student	1.19	0.97, 1.45	0.091
Retired	1.20	0.76, 1.89	0.427
None of these/Other	0.70	0.41, 1.20	0.191
**Religious attendance**			
Once a week	—	—	
More than once a week	1.01	0.89, 1.15	0.837
One to three times a month	0.92	0.78, 1.08	0.309
A few times a year	0.89	0.73, 1.09	0.263
Never	0.68	0.52, 0.89	0.006
**Educational attainment (16+ years)**			
9-15	—	—	

Table S20e. Summary of fitted attrition model in Kenya

Characteristic	Odds Ratio	95% CI	p-value
Up to 8	0.68	0.60, 0.77	8.07e-09
16+	0.97	0.79, 1.20	0.807
**Born in This country**			
Born in this country	—	—	
Born in another country	0.46	0.28, 0.75	0.002
**Race plurality (prominent race/ethnic group [0] or not [1])**	1.07	1.01, 1.14	0.028
**Urbanicity**			
A rural area or on a farm	—	—	
A small town or village	0.85	0.75, 0.97	0.020
A suburb of a large city	0.94	0.76, 1.17	0.582
A large city	1.07	0.68, 1.70	0.758
**Monthly household income**			
Kenya: 8,001 – 10,000 Kshs/month	—	—	
(None/No household income)	0.93	0.74, 1.15	0.485
Kenya: 4,001 – 5,000 Kshs/month	0.92	0.74, 1.15	0.463
Kenya: 5,001 – 6,000 Kshs/month	1.01	0.80, 1.27	0.936
Kenya: 12,001 – 18,000 Kshs/month	1.28	1.03, 1.60	0.027
Kenya: 2,001 – 3,000 Kshs/month	1.26	0.99, 1.61	0.059
Kenya: 10,001 – 12,000 Kshs/month	1.06	0.84, 1.33	0.630
Kenya: 6,001 – 8,000 Kshs/month	1.12	0.89, 1.41	0.344
Kenya: 18,001 – 25,000 Kshs/month	1.26	1.01, 1.57	0.041
Kenya: 1,001 – 2,000 Kshs/month	1.21	0.94, 1.56	0.140
Kenya: 25,001 – 40,000 Kshs/month	1.24	0.93, 1.65	0.140
Kenya: 3,001 – 4,000 Kshs/month	0.89	0.67, 1.18	0.418
Kenya: 500 Kshs/month or less	1.45	1.10, 1.91	0.009
Kenya: 551 – 1,000 Kshs/month	0.99	0.75, 1.30	0.928
Kenya: 40,001 – 80,000 Kshs/month	1.09	0.76, 1.57	0.628
Kenya: More than 80,000 Kshs/month	0.70	0.37, 1.33	0.276

Abbreviations: CI = Confidence Interval, OR = Odds Ratio

Notes. N=11389; attrition weights were estimated using the 'survey::svyglm(family=quasibinomial('logit'))' function. All continuous predictors were standardized and all categorical predictors used the most common category as the reference group. Reported p-values are based on the fitted regression model and no adjustments for multiple testing were done within this table.

Table S20f. Summary of principal components in Kenya

PC	Percent Explained by each PC	Cumulative Percent Explained
1	27.46	27.46
2	4.12	31.58
3	2.62	34.20
4	2.19	36.39
5	2.13	38.52
6	1.90	40.41
**7**	**1.74**	**42.15**
8	1.62	43.78
9	1.51	45.29
10	1.38	46.67
11	1.34	48.01
12	1.28	49.28
13	1.25	50.53
14	1.22	51.76
15	1.19	52.94
16	1.17	54.12
17	1.14	55.26
18	1.13	56.39
19	1.09	57.48
20	1.08	58.57

Notes. N=11389; PCA was conducted using 'survey::svyprcomp(.)' function using all available contemporaneous exposures at wave 1. All PCs were standardized prior to being used as predictors. The bolded row represented the number of retained components for analysis was 7.

Table S20g. Associations of forgivingness with adult well-being and other outcomes at Wave 2 in Kenya

Outcome	Model 1: Demographic and Childhood Variables as Covariates					Model 2: Demographic, Childhood, and Other Wave 1 Confounding Variables (Via Principal Components) as Covariates				
	RR	ES	95% CI	SE	p-value	RR	ES	95% CI	SE	p-value
Human Flourishing										
Secure flourishing index		0.05	(0.03,0.07)	0.01	3.21e-05***		0.02	(-0.01,0.04)	0.01	0.147
Flourishing index		0.05	(0.03,0.07)	0.01	1.38e-05***		0.02	(-0.00,0.04)	0.01	0.098
Happiness & life satisfaction		0.02	(-0.01,0.04)	0.01	0.184		0.00	(-0.02,0.03)	0.01	0.786
Physical & mental health		0.04	(0.01,0.06)	0.01	0.008*		0.01	(-0.01,0.04)	0.01	0.283
Meaning & purpose		0.02	(-0.00,0.05)	0.01	0.073		-0.00	(-0.03,0.02)	0.01	0.811
Character & virtue		0.05	(0.02,0.07)	0.01	1.55e-04***		0.02	(-0.00,0.05)	0.01	0.067
Close social relationships		0.04	(0.02,0.07)	0.01	1.89e-03**		0.02	(-0.00,0.05)	0.01	0.098
Financial & material security		0.01	(-0.01,0.04)	0.01	0.205		-0.00	(-0.02,0.02)	0.01	0.962
Psychological Well-Being										
Happiness (a)		0.00	(-0.02,0.03)	0.01	0.731		-0.01	(-0.03,0.02)	0.01	0.639
Life satisfaction (a)		0.02	(-0.00,0.05)	0.01	0.091		0.01	(-0.01,0.04)	0.01	0.397
Current life evaluation		0.01	(-0.02,0.04)	0.01	0.406		0.00	(-0.03,0.03)	0.01	0.938
Future life evaluation		0.03	(0.00,0.05)	0.01	0.037*		0.01	(-0.02,0.04)	0.01	0.426
Optimism		0.02	(-0.00,0.05)	0.01	0.106		0.01	(-0.02,0.03)	0.01	0.722
Freedom to pursue what's important		0.03	(0.00,0.06)	0.02	0.039*		0.01	(-0.02,0.04)	0.02	0.402
Inner peace	1.02		(1.00,1.03)	0.01	0.016*	1.01		(0.99,1.02)	0.01	0.288
Life balance	1.01		(1.00,1.03)	0.01	0.076	1.00		(0.99,1.02)	0.01	0.486
Sense of mastery	1.03		(1.02,1.04)	0.01	3.41e-05***	1.02		(1.00,1.03)	0.01	0.014*
Meaningful activities (c)		0.01	(-0.01,0.04)	0.01	0.386		-0.01	(-0.03,0.02)	0.01	0.604
Understanding purpose (c)		0.03	(-0.00,0.06)	0.01	0.086		0.00	(-0.03,0.03)	0.02	0.857
Self-rated mental health (b)		0.04	(0.01,0.07)	0.02	0.024*		0.02	(-0.01,0.05)	0.02	0.255
Psychological Distress										
Traumatic distress	1.00		(0.99,1.01)	0.01	0.582	1.00		(0.99,1.01)	0.01	0.837
Depression symptoms composite	1.00		(0.99,1.02)	0.01	0.514	1.01		(0.99,1.02)	0.01	0.298
Depression – feel hopeless	1.00		(0.99,1.01)	0.01	0.587	1.01		(0.99,1.02)	0.01	0.338
Depression – loss of interest	1.00		(0.99,1.01)	0.01	0.725	1.00		(0.99,1.02)	0.01	0.552
Anxiety symptoms composite	1.00		(0.98,1.01)	0.01	0.509	1.00		(0.99,1.01)	0.01	0.965
Anxiety – feel on edge	1.00		(0.99,1.01)	0.01	0.908	1.00		(0.99,1.01)	0.01	0.650
Anxiety – cannot stop worrying	1.00		(0.98,1.01)	0.01	0.631	1.00		(0.99,1.01)	0.01	0.866
Suffering	1.00		(0.99,1.01)	0.01	0.866	1.00		(0.99,1.02)	0.01	0.483
Social Well-Being										
Relationship contentment (e)		0.04	(0.02,0.07)	0.01	1.74e-03**		0.03	(-0.00,0.06)	0.01	0.053
Relationship satisfaction (e)		0.03	(-0.00,0.05)	0.01	0.056		0.01	(-0.02,0.04)	0.01	0.455
Social support		0.05	(0.02,0.08)	0.01	4.46e-04***		0.04	(0.01,0.07)	0.01	3.33e-03**
Intimate/close friend	1.00		(0.99,1.01)	0.01	0.587	1.00		(0.99,1.01)	0.01	0.554
Government approval	1.00		(0.99,1.02)	0.01	0.451	1.00		(0.99,1.02)	0.01	0.709
Say in government	1.00		(0.99,1.01)	0.01	0.994	1.00		(0.98,1.01)	0.01	0.501
Belonging in country		0.04	(0.01,0.07)	0.02	0.013*		0.03	(-0.00,0.06)	0.02	0.097
City/place satisfaction	1.00		(0.99,1.01)	0.01	0.679	0.99		(0.98,1.01)	0.01	0.386
Trust within country	1.00		(0.98,1.01)	0.01	0.770	0.99		(0.98,1.01)	0.01	0.473
Social Participation										
Ever been married	1.00		(0.99,1.01)	0.00	0.827	1.00		(0.99,1.01)	0.00	0.775
Currently divorced	1.00		(1.00,1.00)	0.00	0.289	1.00		(1.00,1.00)	0.00	0.413
Number of children		0.02	(-0.00,0.04)	0.01	0.107		0.03	(0.00,0.05)	0.01	0.024*
Weekly+ community participation	1.00		(0.99,1.01)	0.01	0.626	1.00		(0.99,1.01)	0.01	0.745
Weekly+ religious attendance	1.00		(0.99,1.01)	0.01	0.736	1.00		(0.98,1.01)	0.01	0.519
Social Distress										
Loneliness		-0.03	(-0.06,-0.00)	0.01	0.024*		-0.01	(-0.04,0.01)	0.01	0.320
Perceived discrimination	1.01		(0.99,1.02)	0.01	0.378	1.01		(0.99,1.02)	0.01	0.296
Character & Prosocial Behavior										
Orientation to promote good (d)		0.04	(0.02,0.07)	0.01	2.54e-03**		0.02	(-0.01,0.05)	0.01	0.118
Delayed gratification (d)		0.03	(0.01,0.06)	0.01	0.020*		0.02	(-0.01,0.04)	0.01	0.298
Hope		0.06	(0.03,0.08)	0.01	1.9e-04***		0.03	(0.01,0.06)	0.01	0.022*
Gratitude		0.03	(0.01,0.06)	0.01	0.019*		0.01	(-0.02,0.04)	0.01	0.387
Showing love/care		0.08	(0.05,0.11)	0.02	5.71e-06***		0.06	(0.03,0.10)	0.02	2.4e-04***
Forgivingness	1.05		(1.04,1.06)	0.01	2.22e-16***	1.05		(1.03,1.06)	0.01	2.03e-14***
Charitable giving	1.01		(0.99,1.02)	0.01	0.386	1.00		(0.99,1.02)	0.01	0.761
Helping strangers	1.01		(1.00,1.02)	0.01	0.017*	1.01		(1.00,1.02)	0.01	0.094
Volunteering	1.01		(1.00,1.02)	0.01	0.144	1.00		(0.99,1.02)	0.01	0.518
Physical Health & Health Behavior										
Self-rated physical health (b)		0.02	(-0.00,0.05)	0.01	0.058		0.01	(-0.02,0.03)	0.01	0.621
Health problems	1.00		(0.99,1.01)	0.01	0.347	1.00		(0.99,1.01)	0.01	0.863
Pain in past 4 weeks	1.00		(0.99,1.01)	0.01	0.882	1.00		(0.99,1.02)	0.01	0.594
Daily smoker	1.00		(1.00,1.01)	0.00	0.560	1.01		(1.00,1.02)	0.00	0.029*
Number of drinks per week		-0.03	(-0.07,0.01)	0.02	0.147		-0.02	(-0.06,0.02)	0.02	0.326
Days exercise per week		0.03	(0.00,0.05)	0.01	0.023*		0.02	(-0.00,0.05)	0.01	0.099
Socioeconomic Outcomes										
Financial security (f)		0.01	(-0.02,0.03)	0.01	0.511		-0.00	(-0.03,0.02)	0.01	0.875
Material security (f)		0.02	(-0.01,0.04)	0.01	0.196		0.00	(-0.02,0.03)	0.01	0.934
Educational attainment (16+ years)	1.00		(1.00,1.00)	0.00	0.539	1.00		(1.00,1.00)	0.00	0.513
Currently employed	1.00		(0.99,1.02)	0.01	0.694	1.00		(0.99,1.01)	0.01	0.802
Financially comfortable/getting by	1.01		(1.00,1.02)	0.01	0.017*	1.01		(1.00,1.02)	0.01	0.236
Own home	1.00		(0.99,1.02)	0.01	0.412	1.01		(1.00,1.02)	0.01	0.226
Income – top quintile	1.00		(0.99,1.01)	0.01	0.781	0.99		(0.98,1.01)	0.01	0.392
Religion & Spirituality										
Religious/spiritual connection	1.02		(1.01,1.03)	0.01	1.21e-03**	1.01		(1.00,1.02)	0.01	0.196
Belief in life after death	1.01		(0.99,1.02)	0.01	0.272	1.00		(0.98,1.01)	0.01	0.733
Transformative religious experience	1.02		(1.01,1.04)	0.01	5.98e-04***	1.02		(1.00,1.03)	0.01	0.026*
Religious reading or listening	1.02		(1.01,1.03)	0.01	0.006*	1.01		(1.00,1.02)	0.01	0.135
Prayer or meditation	1.01		(1.00,1.02)	0.01	0.094	1.00		(0.99,1.01)	0.01	0.686
Belief in God/gods/spiritual forces	1.00		(1.00,1.01)	0.00	0.154	1.00		(1.00,1.01)	0.00	0.594
Religious centrality	1.01		(1.00,1.02)	0.01	0.013*	1.00		(0.99,1.01)	0.01	0.512
Religious/spiritual comfort	1.01		(1.00,1.02)	0.00	0.086	1.00		(0.99,1.01)	0.00	0.931
Feel loved by God	1.00		(1.00,1.01)	0.00	0.233	1.00		(0.99,1.01)	0.00	0.916
Feel punished by God	1.00		(0.99,1.01)	0.01	0.842	1.00		(0.99,1.02)	0.01	0.680
Experienced religious criticism	1.00		(0.99,1.01)	0.01	0.864	1.00		(0.99,1.01)	0.01	0.817
Faith-sharing	1.02		(1.01,1.03)	0.01	3.09e-04***	1.01		(1.00,1.03)	0.01	0.021*

Notes. N=11389; Reference for focal predictor: never/rarely. RR, risk-ratio, null effect is 1.00; ES, effect size measure for standardized regression coefficient, null effect is 0.00; SE, standard error, the SE reported for binary/Likert-type outcomes where risk-ratios are on the log(RR) scale; CI, confidence interval; p-value, a Wald-type test of the null hypothesis that the effect of the focal predictor is zero; (a) item part of the Happiness & Life Satisfaction domain of the Secure Flourishing Index; (b) item part of the Physical & Mental Health domain of the Secure Flourishing Index; (c) item part of the Meaning & Purpose domain of the Secure Flourishing Index; (d) item part of the Character & Virtue domain of the Secure Flourishing Index; (e) item part of the Subjective Social Connectedness domain of the Secure Flourishing Index; (f) item part of the Financial & Material Security domain of the Secure Flourishing Index.

Multiple imputation was performed to impute missing data on the covariates, exposure, and outcomes. All models controlled for sociodemographic and childhood factors assessed at Wave 1. For Model 2 with PC (principal components), the first seven principal components of the entire set of contemporaneous confounders assessed at Wave 1 were included as additional covariates of the outcomes at Wave 2.

An outcome-wide analytic approach was used, and a separate model was run for each outcome. A different type of model was run depending on the nature of the outcome: (1) for each binary outcome, a weighted generalized linear model (with a log link and Poisson distribution) was used to estimate an RR; and (2) for each continuous outcome, a weighted linear regression model was used to estimate a ES. All effect sizes were standardized. For continuous outcomes, the ES represents the change in SD on the outcome between the lower and upper categories of the binary focal predictor. For binary outcomes, the RR represents the change in risk of being in the upper category compared to the lower category between the lower and upper categories of the binary focal predictor.

P-value significance thresholds: $p < 0.05$*, $p < 0.005$**, (Bonferroni) $p < 6.41e-04$***, correction for multiple testing using Bonferroni adjusted significant threshold.

Table S20h. Associations of forgivingness with adult well-being and other outcomes at Wave 2 in Kenya using complete-case analyses with attrition weights.

Outcome	Model 1: Demographic and Childhood Variables as Covariates					Model 2: Demographic, Childhood, and Other Wave 1 Confounding Variables (Via Principal Components) as Covariates				
	RR	ES	95% CI	SE	p-value	RR	ES	95% CI	SE	p-value
Human Flourishing										
Secure flourishing index		0.04	(0.01,0.06)	0.01	2.27e-03**		0.01	(-0.01,0.03)	0.01	0.390
Flourishing index		0.04	(0.02,0.06)	0.01	1.23e-03**		0.01	(-0.01,0.03)	0.01	0.365
Happiness & life satisfaction		0.01	(-0.02,0.04)	0.01	0.461		-0.00	(-0.03,0.02)	0.01	0.980
Physical & mental health		0.03	(0.01,0.06)	0.01	0.011*		0.01	(-0.02,0.03)	0.01	0.480
Meaning & purpose		0.02	(-0.01,0.04)	0.01	0.154		-0.01	(-0.03,0.02)	0.01	0.660
Character & virtue		0.04	(0.01,0.06)	0.01	2.31e-03**		0.01	(-0.01,0.04)	0.01	0.261
Close social relationships		0.04	(0.01,0.06)	0.01	0.007*		0.02	(-0.01,0.05)	0.01	0.166
Financial & material security		0.01	(-0.01,0.04)	0.01	0.300		0.00	(-0.02,0.03)	0.01	0.821
Psychological Well-Being										
Happiness (a)		-0.00	(-0.03,0.02)	0.01	0.844		-0.01	(-0.04,0.02)	0.01	0.443
Life satisfaction (a)		0.02	(-0.01,0.05)	0.01	0.202		0.01	(-0.02,0.03)	0.01	0.523
Current life evaluation		0.01	(-0.02,0.03)	0.01	0.582		0.00	(-0.03,0.03)	0.01	0.994
Future life evaluation		0.03	(-0.00,0.05)	0.01	0.071		0.01	(-0.02,0.04)	0.01	0.386
Optimism		0.01	(-0.01,0.04)	0.01	0.304		-0.01	(-0.03,0.02)	0.01	0.686
Freedom to pursue what's important		0.03	(0.00,0.06)	0.01	0.022*		0.01	(-0.01,0.04)	0.01	0.383
Inner peace	1.02		(1.01,1.03)	0.01	0.006*	1.01		(0.99,1.02)	0.01	0.251
Life balance	1.02		(1.00,1.03)	0.01	0.020*	1.01		(0.99,1.02)	0.01	0.241
Sense of mastery	1.03		(1.02,1.04)	0.01	1.85e-05***	1.02		(1.00,1.03)	0.01	0.013*
Meaningful activities (c)		0.01	(-0.02,0.04)	0.01	0.381		-0.00	(-0.03,0.03)	0.01	0.807
Understanding purpose (c)		0.02	(-0.01,0.04)	0.01	0.186		-0.01	(-0.03,0.02)	0.01	0.661
Self-rated mental health (b)		0.03	(0.00,0.06)	0.02	0.035*		0.01	(-0.02,0.04)	0.02	0.421
Psychological Distress										
Traumatic distress	0.99		(0.98,1.01)	0.01	0.311	0.99		(0.98,1.01)	0.01	0.421
Depression symptoms composite	1.01		(0.99,1.02)	0.01	0.259	1.01		(1.00,1.02)	0.01	0.209
Depression – feel hopeless	1.01		(0.99,1.02)	0.01	0.337	1.01		(1.00,1.02)	0.01	0.250
Depression – loss of interest	1.00		(0.99,1.02)	0.01	0.474	1.01		(0.99,1.02)	0.01	0.401
Anxiety symptoms composite	1.00		(0.98,1.01)	0.01	0.603	1.00		(0.99,1.01)	0.01	0.969
Anxiety – feel on edge	1.00		(0.99,1.01)	0.01	0.866	1.00		(0.99,1.02)	0.01	0.555
Anxiety – cannot stop worrying	1.00		(0.98,1.01)	0.01	0.802	1.00		(0.99,1.02)	0.01	0.836
Suffering	1.00		(0.98,1.01)	0.01	0.609	1.00		(0.99,1.02)	0.01	0.811
Social Well-Being										
Relationship contentment (e)		0.04	(0.02,0.07)	0.01	2.06e-03**		0.03	(-0.00,0.06)	0.01	0.068
Relationship satisfaction (e)		0.02	(-0.01,0.04)	0.01	0.141		0.01	(-0.02,0.03)	0.01	0.672
Social support		0.05	(0.03,0.08)	0.01	7.99e-05***		0.04	(0.02,0.07)	0.01	8.71e-04**
Intimate/close friend	1.00		(0.99,1.02)	0.01	0.537	1.00		(0.99,1.01)	0.01	0.546
Government approval	1.00		(0.99,1.02)	0.01	0.575	1.00		(0.99,1.01)	0.01	0.840
Say in government	1.00		(0.99,1.01)	0.01	0.786	1.00		(0.98,1.01)	0.01	0.719
Belonging in country		0.04	(0.02,0.07)	0.01	2.7e-03**		0.03	(0.00,0.06)	0.01	0.021*
City/place satisfaction	1.00		(0.99,1.01)	0.01	0.725	0.99		(0.98,1.01)	0.01	0.391
Trust within country	1.00		(0.98,1.01)	0.01	0.576	0.99		(0.98,1.01)	0.01	0.254
Social Participation										
Ever been married	1.00		(0.99,1.01)	0.00	0.945	1.00		(0.99,1.01)	0.00	0.941
Currently divorced	1.00		(1.00,1.00)	0.00	4.51e-03**	1.00		(1.00,1.00)	0.00	0.023*
Number of children		0.03	(0.01,0.05)	0.01	0.007*		0.04	(0.01,0.06)	0.01	2.35e-03**
Weekly+ community participation	1.01		(0.99,1.02)	0.01	0.352	1.00		(0.99,1.02)	0.01	0.527
Weekly+ religious attendance	1.00		(0.99,1.01)	0.01	0.799	0.99		(0.98,1.01)	0.01	0.301
Social Distress										
Loneliness		-0.03	(-0.06,-0.00)	0.01	0.023*		-0.02	(-0.04,0.01)	0.01	0.309
Perceived discrimination	1.01		(1.00,1.02)	0.01	0.176	1.01		(1.00,1.02)	0.01	0.134
Character & Prosocial Behavior										
Orientation to promote good (d)		0.03	(0.01,0.06)	0.01	0.010*		0.01	(-0.01,0.04)	0.01	0.348
Delayed gratification (d)		0.03	(0.00,0.06)	0.01	0.030*		0.01	(-0.02,0.04)	0.01	0.417
Hope		0.04	(0.01,0.07)	0.02	3.89e-03**		0.02	(-0.01,0.05)	0.02	0.129
Gratitude		0.02	(-0.01,0.05)	0.01	0.116		0.00	(-0.03,0.03)	0.01	0.944
Showing love/care		0.08	(0.05,0.11)	0.02	7.3e-07***		0.06	(0.03,0.09)	0.02	7.47e-05***
Forgivingness	1.06		(1.04,1.07)	0.01	2.22e-16***	1.05		(1.04,1.07)	0.01	7.88e-15***
Charitable giving	1.00		(0.99,1.02)	0.01	0.514	1.00		(0.99,1.01)	0.01	0.809
Helping strangers	1.01		(1.00,1.03)	0.01	0.009*	1.01		(1.00,1.02)	0.01	0.045*
Volunteering	1.01		(1.00,1.02)	0.01	0.207	1.00		(0.99,1.02)	0.01	0.670
Physical Health & Health Behavior										
Self-rated physical health (b)		0.02	(-0.00,0.05)	0.01	0.058		0.00	(-0.02,0.03)	0.01	0.790
Health problems	0.99		(0.98,1.00)	0.01	0.291	1.00		(0.99,1.01)	0.01	0.757
Pain in past 4 weeks	1.00		(0.98,1.01)	0.01	0.657	1.00		(0.99,1.01)	0.01	0.745
Daily smoker	1.01		(1.00,1.01)	0.00	0.136	1.01		(1.00,1.02)	0.00	1.7e-03**
Number of drinks per week		-0.03	(-0.06,0.01)	0.02	0.106		-0.02	(-0.05,0.02)	0.02	0.356
Days exercise per week		0.03	(0.01,0.06)	0.01	0.014*		0.03	(-0.00,0.05)	0.01	0.078
Socioeconomic Outcomes										
Financial security (f)		0.00	(-0.02,0.03)	0.01	0.740		-0.00	(-0.03,0.02)	0.01	0.880
Material security (f)		0.02	(-0.01,0.04)	0.01	0.179		0.01	(-0.02,0.03)	0.01	0.602
Educational attainment (16+ years)	1.00		(1.00,1.00)	0.00	0.422	1.00		(1.00,1.00)	0.00	0.409
Currently employed	1.00		(0.99,1.02)	0.01	0.754	1.00		(0.99,1.02)	0.01	0.824
Financially comfortable/getting by	1.01		(1.00,1.02)	0.01	0.037*	1.01		(1.00,1.02)	0.01	0.201
Own home	1.01		(0.99,1.02)	0.01	0.419	1.01		(1.00,1.02)	0.01	0.195
Income – top quintile	1.00		(0.98,1.01)	0.01	0.471	0.99		(0.98,1.00)	0.01	0.193
Religion & Spirituality										
Religious/spiritual connection	1.02		(1.01,1.03)	0.01	3.86e-04***	1.01		(1.00,1.02)	0.01	0.089
Belief in life after death	1.00		(0.99,1.02)	0.01	0.564	0.99		(0.98,1.01)	0.01	0.365
Transformative religious experience	1.02		(1.01,1.04)	0.01	9.33e-05***	1.02		(1.00,1.03)	0.01	0.011*
Religious reading or listening	1.02		(1.01,1.03)	0.01	3.94e-03**	1.01		(1.00,1.03)	0.01	0.085
Prayer or meditation	1.01		(1.00,1.02)	0.00	0.099	1.00		(0.99,1.01)	0.01	0.654
Belief in God/gods/spiritual forces	1.00		(1.00,1.01)	0.00	0.092	1.00		(1.00,1.01)	0.00	0.390
Religious centrality	1.01		(1.00,1.03)	0.01	0.009*	1.01		(0.99,1.02)	0.01	0.291
Religious/spiritual comfort	1.01		(1.00,1.02)	0.00	0.093	1.00		(0.99,1.01)	0.00	0.923
Feel loved by God	1.01		(1.00,1.01)	0.00	0.112	1.00		(0.99,1.01)	0.00	0.833
Feel punished by God	1.00		(0.99,1.01)	0.01	0.950	1.00		(0.99,1.01)	0.01	0.946
Experienced religious criticism	1.00		(0.98,1.01)	0.01	0.655	1.00		(0.98,1.01)	0.01	0.664
Faith-sharing	1.02		(1.01,1.03)	0.01	1e-03**	1.01		(1.00,1.02)	0.01	0.063

Table S20h. Associations of forgivingness with adult well-being and other outcomes at Wave 2 in Kenya using complete-case analyses with attrition weights.

	Model 1: Demographic and Childhood Variables as Covariates					Model 2: Demographic, Childhood, and Other Wave 1 Confounding Variables (Via Principal Components) as Covariates				
Outcome	RR	ES	95% CI	SE	p-value	RR	ES	95% CI	SE	p-value

Notes. N=7698; Reference for focal predictor: never/rarely. RR, risk-ratio, null effect is 1.00; ES, effect size measure for standardized regression coefficient, null effect is 0.00; SE, standard error, the SE reported for binary/Likert-type outcomes where risk-ratios are on the log(RR) scale; CI, confidence interval; p-value, a Wald-type test of the null hypothesis that the effect of the focal predictor is zero; (a) item part of the Happiness & Life Satisfaction domain of the Secure Flourishing Index; (b) item part of the Physical & Mental Health domain of the Secure Flourishing Index; (c) item part of the Meaning & Purpose domain of the Secure Flourishing Index; (d) item part of the Character & Virtue domain of the Secure Flourishing Index; (e) item part of the Subjective Social Connectedness domain of the Secure Flourishing Index; (f) item part of the Financial & Material Security domain of the Secure Flourishing Index.

Attrition weights were computed to adjust the complete case data (those who responded at Wave 2 to at least 50% of the questions) and multiple imputation was used to impute missing data on all remaining within wave on the covariates, exposure, and outcomes. All models controlled for sociodemographic and childhood factors assessed at Wave 1. For Model 2 with PC (principal components), the first seven principal components of the entire set of contemporaneous confounders assessed at Wave 1 were included as additional covariates of the outcomes at Wave 2.

An outcome-wide analytic approach was used, and a separate model was run for each outcome. A different type of model was run depending on the nature of the outcome: (1) for each binary outcome, a weighted generalized linear model (with a log link and Poisson distribution) was used to estimate an RR; and (2) for each continuous outcome, a weighted linear regression model was used to estimate a ES. All effect sizes were standardized. For continuous outcomes, the ES represents the change in SD on the outcome between the lower and upper categories of the binary focal predictor. For binary outcomes, the RR represents the change in risk of being in the upper category compared to the lower category between the lower and upper categories of the binary focal predictor.

P-value significance thresholds: $p < 0.05$*, $p < 0.005$**, (Bonferroni) $p < 6.41e-04$***, correction for multiple testing using Bonferroni adjusted significant threshold.

Table S20i. Sensitivity analysis of forgivingness outcome-wide results to unmeasured confounding using E-values in Kenya

Outcome	Multiple Imputation				Complete Case w/ Attrition Weights			
	Model 1: Demographics and Childhood Variables as Covariates		Model 2: Demographics, Childhood, and Other Wave 1 Confounders (Via Principal Components) as Covariates		Model 1: Demographics and Childhood Variables as Covariates		Model 2: Demographics, Childhood, and Other Wave 1 Confounders (Via Principal Components) as Covariates	
	EE	ECI	EE	ECI	EE	ECI	EE	ECI
Human Flourishing								
Secure flourishing index	1.51	1.34	1.25	1.00	1.42	1.22	1.18	1.00
Flourishing index	1.54	1.36	1.28	1.00	1.44	1.24	1.19	1.00
Happiness & life satisfaction	1.26	1.00	1.10	1.00	1.19	1.00	1.03	1.00
Physical & mental health	1.43	1.19	1.24	1.00	1.40	1.16	1.18	1.00
Meaning & purpose	1.32	1.00	1.10	1.00	1.27	1.00	1.14	1.00
Character & virtue	1.52	1.32	1.32	1.00	1.44	1.23	1.24	1.00
Close social relationships	1.48	1.26	1.32	1.00	1.42	1.19	1.28	1.00
Financial & material security	1.23	1.00	1.04	1.00	1.22	1.00	1.09	1.00
Psychological Well-Being								
Happiness	1.12	1.00	1.14	1.00	1.09	1.00	1.19	1.00
Life satisfaction	1.30	1.00	1.19	1.00	1.27	1.00	1.17	1.00
Current life evaluation	1.20	1.00	1.05	1.00	1.16	1.00	1.02	1.00
Future life evaluation	1.35	1.07	1.19	1.00	1.33	1.00	1.21	1.00
Optimism	1.32	1.00	1.13	1.00	1.23	1.00	1.14	1.00
Freedom to pursue what's important	1.39	1.08	1.22	1.00	1.38	1.12	1.21	1.00
Inner peace	1.27	1.10	1.17	1.00	1.29	1.14	1.17	1.00
Life balance	1.22	1.00	1.13	1.00	1.26	1.09	1.17	1.00
Sense of mastery	1.38	1.26	1.27	1.11	1.38	1.26	1.26	1.11
Meaningful activities	1.21	1.00	1.16	1.00	1.22	1.00	1.11	1.00
Understanding purpose	1.34	1.00	1.09	1.00	1.26	1.00	1.14	1.00
Self-rated mental health	1.42	1.13	1.27	1.00	1.40	1.09	1.21	1.00
Psychological Distress								
Traumatic distress	1.11	1.00	1.06	1.00	1.16	1.00	1.13	1.00
Depression symptoms composite	1.12	1.00	1.15	1.00	1.16	1.00	1.18	1.00
Depression – feel hopeless	1.10	1.00	1.14	1.00	1.14	1.00	1.16	1.00
Depression – loss of interest	1.08	1.00	1.11	1.00	1.13	1.00	1.14	1.00
Anxiety symptoms composite	1.12	1.00	1.03	1.00	1.11	1.00	1.03	1.00
Anxiety – feel on edge	1.04	1.00	1.09	1.00	1.06	1.00	1.11	1.00
Anxiety – cannot stop worrying	1.10	1.00	1.06	1.00	1.07	1.00	1.07	1.00
Suffering	1.06	1.00	1.13	1.00	1.11	1.00	1.07	1.00
Social Well-Being								
Relationship contentment	1.49	1.26	1.35	1.00	1.48	1.25	1.35	1.00
Relationship satisfaction	1.35	1.00	1.20	1.00	1.28	1.00	1.13	1.00
Social support	1.54	1.32	1.47	1.24	1.56	1.35	1.49	1.27
Intimate/close friend	1.10	1.00	1.11	1.00	1.11	1.00	1.11	1.00
Government approval	1.13	1.00	1.09	1.00	1.11	1.00	1.06	1.00
Say in government	1.01	1.00	1.12	1.00	1.08	1.00	1.09	1.00
Belonging in country	1.45	1.18	1.34	1.00	1.49	1.25	1.40	1.13
City/place satisfaction	1.09	1.00	1.14	1.00	1.08	1.00	1.14	1.00
Trust within country	1.08	1.00	1.14	1.00	1.12	1.00	1.18	1.00
Social Participation								
Ever been married	1.05	1.00	1.06	1.00	1.03	1.00	1.03	1.00
Currently divorced	1.06	1.00	1.06	1.00	1.08	1.04	1.07	1.02
Number of children	1.27	1.00	1.34	1.10	1.38	1.18	1.43	1.22
Weekly+ community participation	1.10	1.00	1.08	1.00	1.14	1.00	1.11	1.00
Weekly+ religious attendance	1.08	1.00	1.12	1.00	1.07	1.00	1.16	1.00
Social Distress								
Loneliness	1.40	1.12	1.24	1.00	1.40	1.12	1.24	1.00
Perceived discrimination	1.15	1.00	1.17	1.00	1.19	1.00	1.20	1.00
Character & Prosocial Behavior								
Orientation to promote good	1.48	1.25	1.31	1.00	1.40	1.17	1.22	1.00
Delayed gratification	1.39	1.13	1.24	1.00	1.36	1.09	1.20	1.00
Hope	1.57	1.34	1.40	1.13	1.48	1.23	1.32	1.00
Gratitude	1.39	1.13	1.21	1.00	1.29	1.00	1.05	1.00
Showing love/care	1.75	1.51	1.63	1.38	1.74	1.51	1.63	1.39
Forgivingness	1.55	1.46	1.53	1.43	1.61	1.51	1.59	1.48
Charitable giving	1.15	1.00	1.08	1.00	1.12	1.00	1.07	1.00
Helping strangers	1.23	1.09	1.19	1.00	1.25	1.11	1.21	1.03
Volunteering	1.19	1.00	1.12	1.00	1.18	1.00	1.10	1.00
Physical Health & Health Behavior								
Self-rated physical health	1.33	1.00	1.15	1.00	1.32	1.00	1.10	1.00
Health problems	1.13	1.00	1.05	1.00	1.14	1.00	1.07	1.00
Pain in past 4 weeks	1.06	1.00	1.12	1.00	1.10	1.00	1.08	1.00
Daily smoker	1.08	1.00	1.17	1.05	1.14	1.00	1.21	1.12
Number of drinks per week	1.37	1.00	1.28	1.00	1.35	1.00	1.25	1.00
Days exercise per week	1.36	1.11	1.30	1.00	1.40	1.15	1.33	1.00
Socioeconomic Outcomes								
Financial security	1.17	1.00	1.08	1.00	1.12	1.00	1.08	1.00
Material security	1.25	1.00	1.05	1.00	1.27	1.00	1.15	1.00
Educational attainment (16+ years)	1.05	1.00	1.05	1.00	1.07	1.00	1.07	1.00
Currently employed	1.09	1.00	1.07	1.00	1.08	1.00	1.07	1.00
Financially comfortable/getting by	1.23	1.09	1.15	1.00	1.22	1.05	1.17	1.00
Own home	1.13	1.00	1.17	1.00	1.14	1.00	1.18	1.00
Income – top quintile	1.07	1.00	1.14	1.00	1.13	1.00	1.18	1.00
Religion & Spirituality								
Religious/spiritual connection	1.29	1.17	1.17	1.00	1.32	1.20	1.20	1.00
Belief in life after death	1.18	1.00	1.09	1.00	1.11	1.00	1.15	1.00
Transformative religious experience	1.34	1.20	1.26	1.08	1.34	1.22	1.27	1.11
Religious reading or listening	1.29	1.14	1.20	1.00	1.30	1.15	1.22	1.00
Prayer or meditation	1.19	1.00	1.08	1.00	1.18	1.00	1.09	1.00
Belief in God/gods/spiritual forces	1.10	1.00	1.06	1.00	1.12	1.00	1.08	1.00
Religious centrality	1.23	1.10	1.11	1.00	1.25	1.11	1.15	1.00
Religious/spiritual comfort	1.17	1.00	1.03	1.00	1.18	1.00	1.04	1.00
Feel loved by God	1.12	1.00	1.03	1.00	1.13	1.00	1.05	1.00
Feel punished by God	1.06	1.00	1.10	1.00	1.03	1.00	1.04	1.00
Experienced religious criticism	1.06	1.00	1.07	1.00	1.10	1.00	1.10	1.00
Faith-sharing	1.32	1.20	1.24	1.08	1.30	1.17	1.22	1.00

Notes. EE, E-value for estimate; ECI, E-value for the limit of the confidence interval. The formula for calculating E-values can be found in VanderWeele and Ding (2017). E-values for estimate are the minimum strength of association on the risk ratio scale that an unmeasured confounder would need to have with both the exposure and the outcome to fully explain away the observed association between the exposure and outcome, conditional on the measured covariates. E-values for the 95% CI closest to the null denote the minimum strength of association on the risk ratio scale that an unmeasured confounder would need to have with both the exposure and the outcome to shift the CI to include the null value, conditional on the measured covariates.

Table S21a. Weighted summary statistics for demographic and childhood variables in Mexico

**Characteristic**	**Wave 1** N = 5,776	**Wave 2** N = 2,265
Forgivingness, n (%)		
Always	2,244 (38.8%)	807 (35.6%)
Often	2,175 (37.7%)	988 (43.6%)
Rarely	1,024 (17.7%)	363 (16.0%)
Never	305 (5.3%)	102 (4.5%)
(Missing)	29 (0.5%)	6 (0.3%)
Year of birth, n (%)		
1943 or earlier (current age: 80+ years)	65 (1.1%)	39 (1.7%)
1943-1953 (current age: 70-79 years)	262 (4.5%)	117 (5.2%)
1953-1963 (current age: 60-69 years)	576 (10.0%)	248 (11.0%)
1963-1973 (current age: 50-59 years)	854 (14.8%)	354 (15.6%)
1973-1983 (current age: 40-49 years)	1,009 (17.5%)	407 (18.0%)
1983-1993 (current age: 30-39 years)	1,263 (21.9%)	514 (22.7%)
1993-1998 (current age: 25-29 years)	605 (10.5%)	234 (10.3%)
1998-2005 (current age: 18-24 years)	1,141 (19.8%)	351 (15.5%)
(Missing)	0 (0%)	0 (0%)
Age of participant		
Mean	41.0	42.8
Standard Deviation	16.3	16.3
Min, Max	18.0, 99.0	19.0, 93.0
Gender, n (%)		
Male	2,757 (47.7%)	1,088 (48.0%)
Female	2,994 (51.8%)	1,169 (51.6%)
Other	3 (0.1%)	1 (0.0%)
(Missing)	22 (0.4%)	7 (0.3%)
Respondent marital status, n (%)		
Single/Never been married	1,481 (25.6%)	596 (26.3%)
Married	2,044 (35.4%)	842 (37.2%)
Separated	398 (6.9%)	149 (6.6%)
Divorced	221 (3.8%)	83 (3.6%)
Widowed	335 (5.8%)	143 (6.3%)
Domestic partner	1,126 (19.5%)	416 (18.4%)
(Missing)	171 (3.0%)	36 (1.6%)
Education (years), n (%)		
Up to 8	1,311 (22.7%)	397 (17.5%)
9-15	3,229 (55.9%)	1,292 (57.0%)
16+	1,234 (21.4%)	576 (25.4%)
(Missing)	1 (0.0%)	0 (0%)
Employment status, n (%)		
Employed for an employer	1,880 (32.5%)	812 (35.9%)
Self-employed	1,048 (18.1%)	466 (20.6%)
Retired	381 (6.6%)	155 (6.9%)
Student	283 (4.9%)	61 (2.7%)
Homemaker	1,285 (22.2%)	437 (19.3%)
Unemployed and looking for a job	581 (10.1%)	149 (6.6%)
None of these/Other	174 (3.0%)	100 (4.4%)
(Missing)	145 (2.5%)	85 (3.8%)
Current religious service attendance, n (%)		
More than once a week	604 (10.5%)	198 (8.7%)
Once a week	1,249 (21.6%)	496 (21.9%)

Table S21a. Weighted summary statistics for demographic and childhood variables in Mexico

Characteristic	Wave 1<br>N = 5,776	Wave 2<br>N = 2,265
One to three times a month	673 (11.7%)	222 (9.8%)
A few times a year	2,060 (35.7%)	861 (38.0%)
Never	1,146 (19.8%)	481 (21.2%)
(Missing)	44 (0.8%)	7 (0.3%)
Immigration status, n (%)		
Born in this country	5,510 (95.4%)	2,192 (96.8%)
Born in another country	109 (1.9%)	39 (1.7%)
(Missing)	157 (2.7%)	34 (1.5%)
Parental marital status around age 12, n (%)		
Parents were married	3,966 (68.7%)	1,617 (71.4%)
Parents were divorced	345 (6.0%)	115 (5.1%)
Parents were never married	846 (14.6%)	350 (15.5%)
One or both of them had died	175 (3.0%)	68 (3.0%)
Unsure	137 (2.4%)	49 (2.2%)
(Missing)	307 (5.3%)	65 (2.9%)
Religious service attendance around age 12, n (%)		
At least once a week	2,498 (43.2%)	988 (43.6%)
One to three times a month	1,166 (20.2%)	452 (20.0%)
Less than once a month	1,092 (18.9%)	455 (20.1%)
Never	951 (16.5%)	336 (14.8%)
(Missing)	69 (1.2%)	35 (1.5%)
Relationship with mother when growing up, n (%)		
Very good	3,899 (67.5%)	1,491 (65.8%)
Somewhat good	1,351 (23.4%)	565 (24.9%)
Somewhat bad	174 (3.0%)	82 (3.6%)
Very bad	91 (1.6%)	33 (1.5%)
(Does not apply)	181 (3.1%)	64 (2.8%)
(Missing)	80 (1.4%)	30 (1.3%)
Relationship with father when growing up, n (%)		
Very good	3,074 (53.2%)	1,154 (51.0%)
Somewhat good	1,563 (27.1%)	654 (28.9%)
Somewhat bad	334 (5.8%)	145 (6.4%)
Very bad	272 (4.7%)	102 (4.5%)
(Does not apply)	474 (8.2%)	186 (8.2%)
(Missing)	59 (1.0%)	23 (1.0%)
Felt like an outsider in family when growing up, n (%)		
Yes	779 (13.5%)	313 (13.8%)
No	4,887 (84.6%)	1,926 (85.0%)
(Missing)	110 (1.9%)	26 (1.2%)
Experienced abuse when growing up, n (%)		
Yes	907 (15.7%)	404 (17.9%)
No	4,592 (79.5%)	1,807 (79.8%)
(Missing)	277 (4.8%)	54 (2.4%)
Self-rated health when growing up, n (%)		
Excellent	1,853 (32.1%)	709 (31.3%)
Very good	1,346 (23.3%)	560 (24.7%)
Good	1,681 (29.1%)	656 (29.0%)
Fair	746 (12.9%)	286 (12.6%)
Poor	135 (2.3%)	44 (1.9%)
(Missing)	14 (0.2%)	10 (0.4%)

Table S21a. Weighted summary statistics for demographic and childhood variables in Mexico

Characteristic	Wave 1<br>N = 5,776	Wave 2<br>N = 2,265
Subjective financial status of family growing up, n (%)		
Lived comfortably	1,773 (30.7%)	691 (30.5%)
Got by	1,876 (32.5%)	723 (31.9%)
Found it difficult	1,713 (29.7%)	700 (30.9%)
Found it very difficult	367 (6.3%)	134 (5.9%)
(Missing)	47 (0.8%)	16 (0.7%)
Religious affiliation growing up, n (%)		
Christianity	5,321 (92.1%)	2,118 (93.5%)
Taoism	4 (0.1%)	0 (0%)
Confucianism	0 (0%)	0 (0%)
Primal, Animist, or Folk religion	2 (0.0%)	0 (0.0%)
Spiritism	0 (0%)	0 (0%)
Umbanda, Candomblé, and other African-derived religions	0 (0%)	0 (0%)
Chinese folk/traditional religion	0 (0%)	0 (0%)
Islam	5 (0.1%)	1 (0.1%)
Hinduism	1 (0.0%)	1 (0.0%)
Buddhism	1 (0.0%)	0 (0%)
Judaism	9 (0.2%)	7 (0.3%)
Sikhism	4 (0.1%)	2 (0.1%)
Baha'i	2 (0.0%)	0 (0%)
Jainism	0 (0%)	0 (0%)
Shinto	2 (0.0%)	0 (0%)
Some other religion	7 (0.1%)	6 (0.2%)
No religion/Atheist/Agnostic	341 (5.9%)	96 (4.3%)
(Missing)	76 (1.3%)	34 (1.5%)

Note. N (%); this table is based on non-imputed data. Cumulative percentages for variables may not add up to 100% due to rounding. Wave 1 characteristics weighted using the Gallup provided sampling weight, ANNUAL_WEIGHT_R2; Wave 2 characteristics weighted accounting for attrition by using the adjusted Wave 1 weight, ANNUAL_WEIGHT_R2, multiplied by the created attrition weight to account for dropout, to maintain nationally representative estimates for Wave 2 characteristics.

Table S21b. Weighted summary statistics for outcome variables in Mexico

Outcome	Wave 1<br>N = 5,776	Wave 2<br>N = 2,265
Secure flourishing index		
Mean	7.6	7.6
Standard Deviation	1.4	1.3
Min, Max	0.0, 10.0	0.8, 10.0
(Missing)	191 (3.3%)	59 (2.6%)
Flourishing index		
Mean	8.2	8.1
Standard Deviation	1.4	1.3
Min, Max	0.0, 10.0	0.9, 10.0
(Missing)	171 (3.0%)	49 (2.1%)
Happiness & life satisfaction		
Mean	7.8	7.7
Standard Deviation	1.9	1.8
Min, Max	0.0, 10.0	0.0, 10.0
(Missing)	30 (0.5%)	12 (0.5%)
Physical & mental health		
Mean	8.1	7.9
Standard Deviation	1.7	1.6
Min, Max	0.0, 10.0	0.0, 10.0
(Missing)	28 (0.5%)	7 (0.3%)
Meaning & purpose		
Mean	8.4	8.3
Standard Deviation	1.7	1.7
Min, Max	0.0, 10.0	0.0, 10.0
(Missing)	33 (0.6%)	9 (0.4%)
Character & virtue		
Mean	8.4	8.5
Standard Deviation	1.5	1.4
Min, Max	0.0, 10.0	0.0, 10.0
(Missing)	77 (1.3%)	10 (0.5%)
Close social relationships		
Mean	8.2	8.1
Standard Deviation	1.9	1.8
Min, Max	0.0, 10.0	0.0, 10.0
(Missing)	37 (0.6%)	15 (0.7%)
Financial & material security		
Mean	4.8	5.2
Standard Deviation	3.5	3.4
Min, Max	0.0, 10.0	0.0, 10.0
(Missing)	26 (0.5%)	11 (0.5%)
Happiness		
Mean	7.8	7.7
Standard Deviation	2.0	1.9
Min, Max	0.0, 10.0	0.0, 10.0
(Missing)	19 (0.3%)	7 (0.3%)
Life satisfaction		
Mean	7.8	7.7
Standard Deviation	2.2	2.1
Min, Max	0.0, 10.0	0.0, 10.0
(Missing)	15 (0.3%)	7 (0.3%)
Current life evaluation		

Table S21b. Weighted summary statistics for outcome variables in Mexico

Outcome	Wave 1<br>N = 5,776	Wave 2<br>N = 2,265
Mean	7.1	7.1
Standard Deviation	2.1	2.0
Min, Max	0.0, 10.0	0.0, 10.0
(Missing)	13 (0.2%)	2 (0.1%)
Future life evaluation		
Mean	8.3	8.2
Standard Deviation	2.1	1.9
Min, Max	0.0, 10.0	0.0, 10.0
(Missing)	170 (2.9%)	12 (0.5%)
Optimism		
Mean	9.1	9.0
Standard Deviation	1.7	1.7
Min, Max	0.0, 10.0	0.0, 10.0
(Missing)	20 (0.3%)	5 (0.2%)
Freedom to pursue what's important		
Mean	8.5	8.5
Standard Deviation	2.0	2.0
Min, Max	0.0, 10.0	0.0, 10.0
(Missing)	14 (0.2%)	7 (0.3%)
Inner peace, n (%)		
Always	1,597 (27.6%)	670 (29.6%)
Often	2,255 (39.0%)	1,142 (50.4%)
Rarely	1,453 (25.2%)	365 (16.1%)
Never	427 (7.4%)	72 (3.2%)
(Missing)	44 (0.8%)	17 (0.7%)
Life balance, n (%)		
Always	1,455 (25.2%)	505 (22.3%)
Often	2,910 (50.4%)	1,210 (53.4%)
Rarely	1,136 (19.7%)	455 (20.1%)
Never	238 (4.1%)	87 (3.8%)
(Missing)	37 (0.6%)	9 (0.4%)
Sense of mastery, n (%)		
Always	2,512 (43.5%)	865 (38.2%)
Often	2,605 (45.1%)	1,153 (50.9%)
Rarely	509 (8.8%)	195 (8.6%)
Never	88 (1.5%)	36 (1.6%)
(Missing)	63 (1.1%)	16 (0.7%)
Meaningful activities		
Mean	8.4	8.3
Standard Deviation	1.9	1.9
Min, Max	0.0, 10.0	0.0, 10.0
(Missing)	11 (0.2%)	1 (<0.1%)
Understanding purpose		
Mean	8.4	8.3
Standard Deviation	2.0	2.0
Min, Max	0.0, 10.0	0.0, 10.0
(Missing)	23 (0.4%)	8 (0.3%)
Self-rated mental health		
Mean	8.3	8.2
Standard Deviation	1.9	1.8
Min, Max	0.0, 10.0	0.0, 10.0

Table S21b. Weighted summary statistics for outcome variables in Mexico

**Outcome**	**Wave 1**<br>N = 5,776	**Wave 2**<br>N = 2,265
(Missing)	14 (0.2%)	1 (<0.1%)
Traumatic distress, n (%)		
A lot	771 (13.4%)	274 (12.1%)
Some	1,404 (24.3%)	604 (26.7%)
Not very much	1,711 (29.6%)	662 (29.2%)
Not at all	1,864 (32.3%)	716 (31.6%)
(Missing)	26 (0.4%)	9 (0.4%)
Depression symptoms composite, n (%)	1,613 (28.2%)	648 (28.8%)
(Missing)	64 (1.1%)	17 (0.8%)
Depression – feel hopeless, n (%)		
Nearly every day	539 (9.3%)	232 (10.3%)
More than half the days	674 (11.7%)	288 (12.7%)
Several days	1,867 (32.3%)	721 (31.8%)
Not at all	2,669 (46.2%)	1,018 (45.0%)
(Missing)	27 (0.5%)	6 (0.3%)
Depression – loss of interest, n (%)		
Nearly every day	637 (11.0%)	239 (10.6%)
More than half the days	842 (14.6%)	335 (14.8%)
Several days	1,932 (33.4%)	799 (35.3%)
Not at all	2,322 (40.2%)	879 (38.8%)
(Missing)	43 (0.7%)	11 (0.5%)
Anxiety symptoms composite, n (%)	1,706 (29.8%)	697 (31.0%)
(Missing)	54 (0.9%)	15 (0.7%)
Anxiety – feel on edge, n (%)		
Nearly every day	671 (11.6%)	216 (9.5%)
More than half the days	675 (11.7%)	312 (13.8%)
Several days	2,016 (34.9%)	762 (33.6%)
Not at all	2,388 (41.3%)	968 (42.7%)
(Missing)	26 (0.4%)	7 (0.3%)
Anxiety – cannot stop worrying, n (%)		
Nearly every day	738 (12.8%)	275 (12.1%)
More than half the days	707 (12.2%)	310 (13.7%)
Several days	1,633 (28.3%)	696 (30.7%)
Not at all	2,661 (46.1%)	975 (43.1%)
(Missing)	38 (0.6%)	9 (0.4%)
Suffering, n (%)		
A lot	492 (8.5%)	176 (7.8%)
Some	1,748 (30.3%)	748 (33.0%)
Not very much	1,845 (31.9%)	747 (33.0%)
Not at all	1,665 (28.8%)	584 (25.8%)
(Missing)	25 (0.4%)	10 (0.5%)
Relationship contentment		
Mean	8.4	8.4
Standard Deviation	2.0	1.9
Min, Max	0.0, 10.0	0.0, 10.0
(Missing)	22 (0.4%)	8 (0.4%)
Relationship satisfaction		
Mean	8.0	7.9
Standard Deviation	2.2	2.0
Min, Max	0.0, 10.0	0.0, 10.0
(Missing)	18 (0.3%)	11 (0.5%)

Table S21b. Weighted summary statistics for outcome variables in Mexico

Outcome	Wave 1<br>N = 5,776	Wave 2<br>N = 2,265
Social support		
Mean	8.3	8.2
Standard Deviation	2.4	2.4
Min, Max	0.0, 10.0	0.0, 10.0
(Missing)	30 (0.5%)	10 (0.4%)
Intimate/close friend, n (%)		
Yes	4,532 (78.5%)	1,774 (78.3%)
No	1,192 (20.6%)	472 (20.8%)
(Missing)	52 (0.9%)	19 (0.9%)
Government approval, n (%)		
Strongly approve	1,290 (22.3%)	538 (23.7%)
Somewhat approve	1,900 (32.9%)	702 (31.0%)
Neither approve nor disapprove	1,385 (24.0%)	530 (23.4%)
Somewhat disapprove	652 (11.3%)	235 (10.4%)
Strongly disapprove	484 (8.4%)	226 (10.0%)
(Missing)	64 (1.1%)	35 (1.5%)
Say in government, n (%)		
Agree	2,459 (42.6%)	1,403 (62.0%)
Disagree	1,437 (24.9%)	252 (11.1%)
Unsure	1,794 (31.1%)	593 (26.2%)
(Missing)	87 (1.5%)	17 (0.8%)
Belonging in country		
Mean	8.5	8.5
Standard Deviation	2.0	2.0
Min, Max	0.0, 10.0	0.0, 10.0
(Missing)	92 (1.6%)	51 (2.3%)
City/place satisfaction, n (%)		
Satisfied	4,275 (74.0%)	1,577 (69.6%)
Dissatisfied	830 (14.4%)	385 (17.0%)
Unsure	620 (10.7%)	275 (12.1%)
(Missing)	51 (0.9%)	28 (1.2%)
Trust within country, n (%)		
All people	157 (2.7%)	61 (2.7%)
Most people	983 (17.0%)	408 (18.0%)
Some people	2,603 (45.1%)	1,068 (47.1%)
Not very many people	1,689 (29.2%)	581 (25.6%)
None	297 (5.1%)	135 (6.0%)
(Missing)	47 (0.8%)	13 (0.6%)
Number of children		
Mean	1.2	1.1
Standard Deviation	1.4	1.2
Min, Max	0.0, 18.0	0.0, 10.0
(Missing)	156 (2.7%)	155 (6.8%)
Community participation, n (%)		
More than once a week	383 (6.6%)	122 (5.4%)
Once a week	525 (9.1%)	187 (8.3%)
One to three times a month	519 (9.0%)	165 (7.3%)
A few times a year	1,751 (30.3%)	707 (31.2%)
Never	2,568 (44.5%)	1,074 (47.4%)
(Missing)	29 (0.5%)	10 (0.4%)
Religious attendance, n (%)		

Table S21b. Weighted summary statistics for outcome variables in Mexico

Outcome	Wave 1<br>N = 5,776	Wave 2<br>N = 2,265
More than once a week	604 (10.5%)	198 (8.7%)
Once a week	1,249 (21.6%)	496 (21.9%)
One to three times a month	673 (11.7%)	222 (9.8%)
A few times a year	2,060 (35.7%)	861 (38.0%)
Never	1,146 (19.8%)	481 (21.2%)
(Missing)	44 (0.8%)	7 (0.3%)
Loneliness		
Mean	3.3	3.4
Standard Deviation	3.2	3.2
Min, Max	0.0, 10.0	0.0, 10.0
(Missing)	15 (0.3%)	10 (0.5%)
Perceived discrimination, n (%)		
Always	438 (7.6%)	161 (7.1%)
Often	888 (15.4%)	414 (18.3%)
Rarely	2,078 (36.0%)	879 (38.8%)
Never	2,331 (40.4%)	807 (35.7%)
(Missing)	42 (0.7%)	2 (0.1%)
Orientation to promote good		
Mean	8.6	8.6
Standard Deviation	1.6	1.6
Min, Max	0.0, 10.0	0.0, 10.0
(Missing)	33 (0.6%)	3 (0.1%)
Delayed gratification		
Mean	8.3	8.3
Standard Deviation	2.0	1.8
Min, Max	0.0, 10.0	0.0, 10.0
(Missing)	50 (0.9%)	7 (0.3%)
Hope		
Mean	9.0	9.0
Standard Deviation	1.6	1.5
Min, Max	0.0, 10.0	0.0, 10.0
(Missing)	31 (0.5%)	11 (0.5%)
Gratitude		
Mean	8.8	8.7
Standard Deviation	1.8	1.8
Min, Max	0.0, 10.0	0.0, 10.0
(Missing)	23 (0.4%)	12 (0.5%)
Showing love/care		
Mean	8.7	8.5
Standard Deviation	1.9	1.9
Min, Max	0.0, 10.0	0.0, 10.0
(Missing)	22 (0.4%)	5 (0.2%)
Forgivingness, n (%)		
Always	2,244 (38.8%)	807 (35.6%)
Often	2,175 (37.7%)	988 (43.6%)
Rarely	1,024 (17.7%)	363 (16.0%)
Never	305 (5.3%)	102 (4.5%)
(Missing)	29 (0.5%)	6 (0.3%)
Charitable giving, n (%)		
Yes	1,162 (20.1%)	684 (30.2%)
No	4,604 (79.7%)	1,551 (68.5%)

Table S21b. Weighted summary statistics for outcome variables in Mexico

Outcome	Wave 1<br>N = 5,776	Wave 2<br>N = 2,265
(Missing)	10 (0.2%)	31 (1.3%)
Helping strangers, n (%)		
Yes	3,617 (62.6%)	1,461 (64.5%)
No	2,129 (36.9%)	781 (34.5%)
(Missing)	29 (0.5%)	23 (1.0%)
Volunteering, n (%)		
Yes	1,225 (21.2%)	430 (19.0%)
No	4,530 (78.4%)	1,814 (80.1%)
(Missing)	21 (0.4%)	21 (0.9%)
Self-rated physical health		
Mean	7.8	7.6
Standard Deviation	2.0	1.9
Min, Max	0.0, 10.0	0.0, 10.0
(Missing)	16 (0.3%)	7 (0.3%)
Health problems, n (%)		
Yes	979 (17.0%)	389 (17.2%)
No	4,583 (79.3%)	1,828 (80.7%)
(Missing)	214 (3.7%)	47 (2.1%)
Pain in past 4 weeks, n (%)		
A lot	571 (9.9%)	216 (9.6%)
Some	1,604 (27.8%)	654 (28.9%)
Not very much	1,989 (34.4%)	874 (38.6%)
None at all	1,605 (27.8%)	519 (22.9%)
(Missing)	7 (0.1%)	2 (0.1%)
Number of cigarettes per day		
Mean	1.2	1.2
Standard Deviation	4.3	4.0
Min, Max	0.0, 97.0	0.0, 59.0
(Missing)	124 (2.1%)	72 (3.2%)
Number of drinks per week		
Mean	1.4	1.4
Standard Deviation	3.5	3.8
Min, Max	0.0, 97.0	0.0, 75.0
(Missing)	105 (1.8%)	63 (2.8%)
Days exercise per week		
Mean	2.4	2.5
Standard Deviation	2.5	2.5
Min, Max	0.0, 7.0	0.0, 7.0
(Missing)	33 (0.6%)	15 (0.7%)
Financial security		
Mean	4.9	5.3
Standard Deviation	3.7	3.5
Min, Max	0.0, 10.0	0.0, 10.0
(Missing)	8 (0.1%)	6 (0.3%)
Material security		
Mean	4.8	5.0
Standard Deviation	3.8	3.6
Min, Max	0.0, 10.0	0.0, 10.0
(Missing)	21 (0.4%)	8 (0.4%)
Educational attainment (16+ years), n (%)		
Up to 8	1,311 (22.7%)	397 (17.5%)

Table S21b. Weighted summary statistics for outcome variables in Mexico

Outcome	Wave 1<br>N = 5,776	Wave 2<br>N = 2,265
9-15	3,229 (55.9%)	1,292 (57.0%)
16+	1,234 (21.4%)	576 (25.4%)
(Missing)	1 (0.0%)	0 (0%)
Currently employed, n (%)		
Employed for an employer	1,880 (32.5%)	812 (35.9%)
Self-employed	1,048 (18.1%)	466 (20.6%)
Retired	381 (6.6%)	155 (6.9%)
Student	283 (4.9%)	61 (2.7%)
Homemaker	1,285 (22.2%)	437 (19.3%)
Unemployed and looking for a job	581 (10.1%)	149 (6.6%)
None of these/Other	174 (3.0%)	100 (4.4%)
(Missing)	145 (2.5%)	85 (3.8%)
Financially comfortable/getting by, n (%)		
Living comfortably on present income	775 (13.4%)	346 (15.3%)
Getting by on present income	1,942 (33.6%)	850 (37.5%)
Finding it difficult on present income	2,051 (35.5%)	732 (32.3%)
Finding it very difficult on present income	687 (11.9%)	257 (11.3%)
(Missing)	321 (5.6%)	80 (3.6%)
Own home, n (%)		
Someone in this household owns this home	2,978 (51.6%)	1,225 (54.1%)
Someone in this household rents this home	921 (16.0%)	386 (17.0%)
Both	372 (6.4%)	211 (9.3%)
Neither	1,201 (20.8%)	374 (16.5%)
Rent	0 (0%)	0 (0%)
Own	0 (0%)	0 (0%)
Something else	0 (0%)	0 (0%)
(Missing)	303 (5.2%)	69 (3.1%)
Religious/spiritual connection, n (%)		
Always	1,990 (34.5%)	794 (35.0%)
Often	1,837 (31.8%)	816 (36.0%)
Rarely	1,418 (24.6%)	481 (21.2%)
Never	501 (8.7%)	173 (7.6%)
(Missing)	30 (0.5%)	1 (0.0%)
Belief in life after death, n (%)		
Yes	3,429 (59.4%)	1,339 (59.1%)
No	844 (14.6%)	342 (15.1%)
Unsure	1,426 (24.7%)	551 (24.3%)
(Missing)	77 (1.3%)	33 (1.5%)
Transformative religious experience, n (%)		
Yes	2,551 (44.2%)	926 (40.9%)
No	3,173 (54.9%)	1,307 (57.7%)
(Missing)	52 (0.9%)	31 (1.4%)
Religious reading or listening, n (%)		
More than once a day	494 (8.5%)	205 (9.1%)
About once a day	803 (13.9%)	297 (13.1%)
Sometimes	3,124 (54.1%)	1,172 (51.7%)
Never	1,294 (22.4%)	563 (24.9%)
(Missing)	61 (1.1%)	27 (1.2%)
Prayer or meditation, n (%)		
More than once a day	994 (17.2%)	397 (17.5%)
About once a day	1,699 (29.4%)	622 (27.5%)

Table S21b. Weighted summary statistics for outcome variables in Mexico

Outcome	Wave 1<br>N = 5,776	Wave 2<br>N = 2,265
Sometimes	2,386 (41.3%)	964 (42.5%)
Never	650 (11.2%)	265 (11.7%)
(Missing)	46 (0.8%)	17 (0.8%)
Belief in God/gods/spiritual forces, n (%)		
One God	4,520 (78.3%)	1,763 (77.8%)
More than one god	237 (4.1%)	80 (3.6%)
An impersonal spiritual force	465 (8.0%)	222 (9.8%)
None of these	207 (3.6%)	83 (3.6%)
Unsure	303 (5.2%)	105 (4.7%)
(Missing)	45 (0.8%)	11 (0.5%)
Religious centrality, n (%)		
Agree	3,076 (53.3%)	1,260 (55.7%)
Disagree	634 (11.0%)	238 (10.5%)
Not relevant	997 (17.3%)	414 (18.3%)
Unsure	972 (16.8%)	344 (15.2%)
(Missing)	97 (1.7%)	8 (0.4%)
Religious/spiritual comfort, n (%)		
Agree	3,816 (66.1%)	1,467 (64.8%)
Disagree	400 (6.9%)	183 (8.1%)
Not relevant	811 (14.0%)	362 (16.0%)
Unsure	714 (12.4%)	241 (10.7%)
(Missing)	35 (0.6%)	12 (0.5%)
Feel loved by God, n (%)		
Agree	4,459 (77.2%)	1,743 (76.9%)
Disagree	277 (4.8%)	130 (5.7%)
Not relevant	573 (9.9%)	215 (9.5%)
Unsure	426 (7.4%)	169 (7.5%)
(Missing)	41 (0.7%)	8 (0.4%)
Feel punished by God, n (%)		
Agree	866 (15.0%)	317 (14.0%)
Disagree	3,001 (52.0%)	1,165 (51.5%)
Not relevant	976 (16.9%)	407 (18.0%)
Unsure	872 (15.1%)	362 (16.0%)
(Missing)	61 (1.1%)	14 (0.6%)
Experienced religious criticism, n (%)		
Agree	1,334 (23.1%)	518 (22.9%)
Disagree	1,636 (28.3%)	597 (26.4%)
Not relevant	1,299 (22.5%)	548 (24.2%)
Unsure	1,434 (24.8%)	593 (26.2%)
(Missing)	73 (1.3%)	9 (0.4%)
Faith-sharing, n (%)		
Agree	2,638 (45.7%)	1,154 (51.0%)
Disagree	1,217 (21.1%)	343 (15.2%)
Not relevant	1,218 (21.1%)	558 (24.6%)
Unsure	644 (11.2%)	198 (8.7%)
(Missing)	59 (1.0%)	12 (0.5%)

Note. N (%); this table is based on non-imputed data. Cumulative percentages for variables may not add up to 100% due to rounding. Wave 1 characteristics weighted using the Gallup provided sampling weight, ANNUAL_WEIGHT_R2; Wave 2 characteristics weighted accounting for attrition by using the adjusted Wave 1 weight, ANNUAL_WEIGHT_R2, multiplied by the created attrition weight to account for dropout, to maintain nationally representative estimates for Wave 2 characteristics.

Table S21c. Unweighted summary statistics for demographic and childhood variables in Mexico by retention status

Characteristic	Attritors–Not Observed in Wave 2<br>N = 3,274	Retained–Observed in Wave 2<br>N = 2,465
Forgivingness, n (%)		
Always	1,300 (39.7%)	928 (37.6%)
Often	1,154 (35.2%)	1,010 (41.0%)
Rarely	624 (19.0%)	392 (15.9%)
Never	182 (5.6%)	120 (4.9%)
(Missing)	14 (0.4%)	15 (0.6%)
Year of birth, n (%)		
1943 or earlier (current age: 80+ years)	33 (1.0%)	33 (1.3%)
1943-1953 (current age: 70-79 years)	114 (3.5%)	147 (6.0%)
1953-1963 (current age: 60-69 years)	249 (7.6%)	326 (13.2%)
1963-1973 (current age: 50-59 years)	387 (11.8%)	466 (18.9%)
1973-1983 (current age: 40-49 years)	538 (16.4%)	466 (18.9%)
1983-1993 (current age: 30-39 years)	745 (22.8%)	509 (20.6%)
1993-1998 (current age: 25-29 years)	393 (12.0%)	207 (8.4%)
1998-2005 (current age: 18-24 years)	816 (24.9%)	311 (12.6%)
(Missing)	0 (0%)	0 (0%)
Age of participant		
Mean	38.2	44.9
Standard Deviation	15.9	16.1
Min, Max	18.0, 99.0	18.0, 92.0
Gender, n (%)		
Male	1,571 (48.0%)	1,168 (47.4%)
Female	1,685 (51.5%)	1,290 (52.3%)
Other	2 (0.1%)	1 (0.0%)
(Missing)	15 (0.5%)	6 (0.2%)
Respondent marital status, n (%)		
Single/Never been married	887 (27.1%)	582 (23.6%)
Married	1,008 (30.8%)	1,029 (41.7%)
Separated	233 (7.1%)	162 (6.6%)
Divorced	108 (3.3%)	112 (4.6%)
Widowed	176 (5.4%)	157 (6.4%)
Domestic partner	722 (22.1%)	393 (15.9%)
(Missing)	139 (4.2%)	30 (1.2%)
Education (years), n (%)		
Up to 8	795 (24.3%)	506 (20.5%)
9-15	1,934 (59.1%)	1,270 (51.5%)
16+	543 (16.6%)	689 (28.0%)
(Missing)	1 (0.0%)	0 (0%)
Employment status, n (%)		
Employed for an employer	1,047 (32.0%)	821 (33.3%)
Self-employed	536 (16.4%)	507 (20.6%)
Retired	174 (5.3%)	207 (8.4%)
Student	182 (5.6%)	98 (4.0%)
Homemaker	718 (21.9%)	559 (22.7%)
Unemployed and looking for a job	388 (11.8%)	187 (7.6%)
None of these/Other	109 (3.3%)	64 (2.6%)
(Missing)	120 (3.7%)	23 (0.9%)
Current religious service attendance, n (%)		
More than once a week	316 (9.7%)	285 (11.6%)

Table S21c. Unweighted summary statistics for demographic and childhood variables in Mexico by retention status

**Characteristic**	**Attritors–Not Observed in Wave 2** N = 3,274	**Retained–Observed in Wave 2** N = 2,465
Once a week	701 (21.4%)	540 (21.9%)
One to three times a month	404 (12.4%)	263 (10.7%)
A few times a year	1,172 (35.8%)	874 (35.5%)
Never	659 (20.1%)	479 (19.4%)
(Missing)	20 (0.6%)	23 (0.9%)
Immigration status, n (%)		
Born in this country	3,081 (94.1%)	2,395 (97.2%)
Born in another country	62 (1.9%)	46 (1.9%)
(Missing)	131 (4.0%)	24 (1.0%)
Parental marital status around age 12, n (%)		
Parents were married	2,123 (64.8%)	1,822 (73.9%)
Parents were divorced	217 (6.6%)	125 (5.1%)
Parents were never married	499 (15.2%)	341 (13.9%)
One or both of them had died	99 (3.0%)	75 (3.0%)
Unsure	94 (2.9%)	42 (1.7%)
(Missing)	242 (7.4%)	60 (2.4%)
Religious service attendance around age 12, n (%)		
At least once a week	1,369 (41.8%)	1,114 (45.2%)
One to three times a month	683 (20.9%)	475 (19.3%)
Less than once a month	608 (18.6%)	477 (19.3%)
Never	580 (17.7%)	364 (14.8%)
(Missing)	34 (1.0%)	35 (1.4%)
Relationship with mother when growing up, n (%)		
Very good	2,225 (68.0%)	1,648 (66.9%)
Somewhat good	745 (22.7%)	599 (24.3%)
Somewhat bad	82 (2.5%)	91 (3.7%)
Very bad	52 (1.6%)	38 (1.6%)
(Does not apply)	121 (3.7%)	58 (2.3%)
(Missing)	48 (1.5%)	31 (1.3%)
Relationship with father when growing up, n (%)		
Very good	1,769 (54.0%)	1,284 (52.1%)
Somewhat good	853 (26.1%)	701 (28.4%)
Somewhat bad	181 (5.5%)	151 (6.1%)
Very bad	150 (4.6%)	120 (4.9%)
(Does not apply)	288 (8.8%)	182 (7.4%)
(Missing)	32 (1.0%)	26 (1.1%)
Felt like an outsider in family when growing up, n (%)		
Yes	447 (13.6%)	327 (13.3%)
No	2,744 (83.8%)	2,111 (85.7%)
(Missing)	82 (2.5%)	27 (1.1%)
Experienced abuse when growing up, n (%)		
Yes	467 (14.3%)	436 (17.7%)
No	2,576 (78.7%)	1,987 (80.6%)
(Missing)	230 (7.0%)	42 (1.7%)
Self-rated health when growing up, n (%)		
Excellent	1,077 (32.9%)	763 (31.0%)
Very good	738 (22.5%)	600 (24.3%)

Table S21c. Unweighted summary statistics for demographic and childhood variables in Mexico by retention status

Characteristic	Attritors–Not Observed in Wave 2<br>N = 3,274	Retained–Observed in Wave 2<br>N = 2,465
Good	949 (29.0%)	722 (29.3%)
Fair	421 (12.9%)	320 (13.0%)
Poor	84 (2.6%)	50 (2.0%)
(Missing)	4 (0.1%)	10 (0.4%)
Subjective financial status of family growing up, n (%)		
Lived comfortably	1,021 (31.2%)	740 (30.0%)
Got by	1,065 (32.5%)	799 (32.4%)
Found it difficult	942 (28.8%)	761 (30.9%)
Found it very difficult	217 (6.6%)	146 (5.9%)
(Missing)	28 (0.9%)	19 (0.8%)
Religious affiliation growing up, n (%)		
Christianity	2,969 (90.7%)	2,319 (94.1%)
Taoism	4 (0.1%)	0 (0%)
Confucianism	0 (0%)	0 (0%)
Primal, Animist, or Folk religion	2 (0.1%)	0 (<0.0%)
Spiritism	0 (0%)	0 (0%)
Umbanda, Candomblé, and other African-derived religions	0 (0%)	0 (0%)
Chinese folk/traditional religion	0 (0%)	0 (0%)
Islam	5 (0.1%)	1 (0.0%)
Hinduism	0 (0%)	1 (0.0%)
Buddhism	1 (0.0%)	0 (0%)
Judaism	5 (0.1%)	4 (0.2%)
Sikhism	3 (0.1%)	1 (0.0%)
Baha'i	2 (0.0%)	0 (0%)
Jainism	0 (0%)	0 (0%)
Shinto	2 (0.1%)	0 (0%)
Some other religion	4 (0.1%)	4 (0.2%)
No religion/Atheist/Agnostic	237 (7.2%)	100 (4.1%)
(Missing)	40 (1.2%)	35 (1.4%)

Note. N (%); this table is based on non-imputed data. Cumulative percentages for variables may not add up to 100% due to rounding.

Table S21d. Unweighted summary statistics for Wave 1 outcome variables in Mexico by retention status.

**Outcome**	**Attritors–Not Observed in Wave 2** N = 3,274	**Retained–Observed in Wave 2** N = 2,465
Secure flourishing index		
Mean	7.6	7.7
Standard Deviation	1.4	1.3
Min, Max	0.4, 10.0	0.0, 10.0
(Missing)	121 (3.7%)	68 (2.8%)
Flourishing index		
Mean	8.2	8.2
Standard Deviation	1.4	1.3
Min, Max	0.5, 10.0	0.0, 10.0
(Missing)	107 (3.3%)	63 (2.5%)
Happiness & life satisfaction		
Mean	7.8	7.8
Standard Deviation	2.0	1.8
Min, Max	0.0, 10.0	0.0, 10.0
(Missing)	20 (0.6%)	9 (0.4%)
Physical & mental health		
Mean	8.1	8.0
Standard Deviation	1.8	1.6
Min, Max	0.0, 10.0	0.0, 10.0
(Missing)	18 (0.6%)	9 (0.4%)
Meaning & purpose		
Mean	8.4	8.5
Standard Deviation	1.7	1.6
Min, Max	0.0, 10.0	0.0, 10.0
(Missing)	22 (0.7%)	11 (0.4%)
Character & virtue		
Mean	8.4	8.5
Standard Deviation	1.6	1.4
Min, Max	0.0, 10.0	0.0, 10.0
(Missing)	41 (1.3%)	35 (1.4%)
Close social relationships		
Mean	8.2	8.2
Standard Deviation	2.0	1.8
Min, Max	0.0, 10.0	0.0, 10.0
(Missing)	30 (0.9%)	6 (0.2%)
Financial & material security		
Mean	4.8	4.9
Standard Deviation	3.5	3.4
Min, Max	0.0, 10.0	0.0, 10.0
(Missing)	16 (0.5%)	10 (0.4%)
Happiness		
Mean	7.8	7.8
Standard Deviation	2.1	1.9
Min, Max	0.0, 10.0	0.0, 10.0
(Missing)	13 (0.4%)	5 (0.2%)
Life satisfaction		
Mean	7.9	7.8
Standard Deviation	2.2	2.0
Min, Max	0.0, 10.0	0.0, 10.0

Table S21d. Unweighted summary statistics for Wave 1 outcome variables in Mexico by retention status.

**Outcome**	**Attritors–Not Observed in Wave 2** N = 3,274	**Retained–Observed in Wave 2** N = 2,465
(Missing)	11 (0.3%)	4 (0.1%)
Current life evaluation		
Mean	7.1	7.1
Standard Deviation	2.2	2.0
Min, Max	0.0, 10.0	0.0, 10.0
(Missing)	5 (0.2%)	7 (0.3%)
Future life evaluation		
Mean	8.4	8.2
Standard Deviation	2.1	2.0
Min, Max	0.0, 10.0	0.0, 10.0
(Missing)	104 (3.2%)	64 (2.6%)
Optimism		
Mean	9.0	9.1
Standard Deviation	1.7	1.6
Min, Max	0.0, 10.0	0.0, 10.0
(Missing)	11 (0.3%)	9 (0.4%)
Freedom to pursue what's important		
Mean	8.5	8.6
Standard Deviation	2.0	1.9
Min, Max	0.0, 10.0	0.0, 10.0
(Missing)	7 (0.2%)	7 (0.3%)
Inner peace, n (%)		
Always	891 (27.2%)	696 (28.2%)
Often	1,189 (36.3%)	1,055 (42.8%)
Rarely	900 (27.5%)	541 (21.9%)
Never	273 (8.3%)	150 (6.1%)
(Missing)	20 (0.6%)	24 (1.0%)
Life balance, n (%)		
Always	856 (26.1%)	589 (23.9%)
Often	1,553 (47.4%)	1,342 (54.4%)
Rarely	693 (21.2%)	433 (17.6%)
Never	151 (4.6%)	84 (3.4%)
(Missing)	20 (0.6%)	17 (0.7%)
Sense of mastery, n (%)		
Always	1,462 (44.7%)	1,032 (41.8%)
Often	1,411 (43.1%)	1,180 (47.9%)
Rarely	310 (9.5%)	194 (7.9%)
Never	52 (1.6%)	35 (1.4%)
(Missing)	38 (1.2%)	24 (1.0%)
Meaningful activities		
Mean	8.4	8.5
Standard Deviation	2.0	1.7
Min, Max	0.0, 10.0	0.0, 10.0
(Missing)	9 (0.3%)	2 (<0.1%)
Understanding purpose		
Mean	8.4	8.4
Standard Deviation	2.0	1.9
Min, Max	0.0, 10.0	0.0, 10.0
(Missing)	14 (0.4%)	9 (0.4%)

Table S21d. Unweighted summary statistics for Wave 1 outcome variables in Mexico by retention status.

**Outcome**	**Attritors–Not Observed in Wave 2** N = 3,274	**Retained–Observed in Wave 2** N = 2,465
Self-rated mental health		
Mean	8.3	8.3
Standard Deviation	1.9	1.7
Min, Max	0.0, 10.0	0.0, 10.0
(Missing)	10 (0.3%)	4 (0.2%)
Traumatic distress, n (%)		
A lot	458 (14.0%)	307 (12.5%)
Some	795 (24.3%)	600 (24.3%)
Not very much	958 (29.3%)	742 (30.1%)
Not at all	1,047 (32.0%)	805 (32.7%)
(Missing)	15 (0.5%)	10 (0.4%)
Depression symptoms composite, n (%)	1,005 (31.1%)	593 (24.3%)
(Missing)	42 (1.3%)	22 (0.9%)
Depression – feel hopeless, n (%)		
Nearly every day	357 (10.9%)	176 (7.1%)
More than half the days	428 (13.1%)	240 (9.7%)
Several days	994 (30.4%)	863 (35.0%)
Not at all	1,478 (45.2%)	1,175 (47.7%)
(Missing)	15 (0.5%)	11 (0.4%)
Depression – loss of interest, n (%)		
Nearly every day	401 (12.3%)	230 (9.3%)
More than half the days	520 (15.9%)	316 (12.8%)
Several days	1,014 (31.0%)	909 (36.9%)
Not at all	1,309 (40.0%)	998 (40.5%)
(Missing)	30 (0.9%)	13 (0.5%)
Anxiety symptoms composite, n (%)	1,051 (32.4%)	640 (26.2%)
(Missing)	28 (0.9%)	26 (1.0%)
Anxiety – feel on edge, n (%)		
Nearly every day	462 (14.1%)	202 (8.2%)
More than half the days	422 (12.9%)	247 (10.0%)
Several days	1,066 (32.6%)	940 (38.1%)
Not at all	1,313 (40.1%)	1,060 (43.0%)
(Missing)	10 (0.3%)	16 (0.6%)
Anxiety – cannot stop worrying, n (%)		
Nearly every day	449 (13.7%)	283 (11.5%)
More than half the days	442 (13.5%)	258 (10.5%)
Several days	892 (27.2%)	732 (29.7%)
Not at all	1,467 (44.8%)	1,178 (47.8%)
(Missing)	23 (0.7%)	14 (0.6%)
Suffering, n (%)		
A lot	307 (9.4%)	181 (7.4%)
Some	963 (29.4%)	775 (31.4%)
Not very much	999 (30.5%)	836 (33.9%)
Not at all	983 (30.0%)	670 (27.2%)
(Missing)	21 (0.7%)	3 (0.1%)
Relationship contentment		
Mean	8.3	8.5
Standard Deviation	2.1	1.9
Min, Max	0.0, 10.0	0.0, 10.0

Table S21d. Unweighted summary statistics for Wave 1 outcome variables in Mexico by retention status.

**Outcome**	**Attritors–Not Observed in Wave 2** N = 3,274	**Retained–Observed in Wave 2** N = 2,465
(Missing)	18 (0.5%)	4 (0.2%)
Relationship satisfaction		
Mean	8.0	8.0
Standard Deviation	2.2	2.1
Min, Max	0.0, 10.0	0.0, 10.0
(Missing)	15 (0.4%)	3 (0.1%)
Social support		
Mean	8.2	8.4
Standard Deviation	2.5	2.2
Min, Max	0.0, 10.0	0.0, 10.0
(Missing)	20 (0.6%)	10 (0.4%)
Intimate/close friend, n (%)		
Yes	2,563 (78.3%)	1,940 (78.7%)
No	681 (20.8%)	503 (20.4%)
(Missing)	30 (0.9%)	22 (0.9%)
Government approval, n (%)		
Strongly approve	728 (22.3%)	553 (22.4%)
Somewhat approve	1,113 (34.0%)	773 (31.4%)
Neither approve nor disapprove	849 (25.9%)	525 (21.3%)
Somewhat disapprove	331 (10.1%)	319 (12.9%)
Strongly disapprove	216 (6.6%)	267 (10.8%)
(Missing)	36 (1.1%)	28 (1.2%)
Say in government, n (%)		
Agree	1,263 (38.6%)	1,184 (48.0%)
Disagree	830 (25.3%)	597 (24.2%)
Unsure	1,130 (34.5%)	648 (26.3%)
(Missing)	51 (1.5%)	35 (1.4%)
Belonging in country		
Mean	8.5	8.5
Standard Deviation	2.0	2.0
Min, Max	0.0, 10.0	0.0, 10.0
(Missing)	50 (1.5%)	41 (1.7%)
City/place satisfaction, n (%)		
Satisfied	2,435 (74.4%)	1,812 (73.5%)
Dissatisfied	442 (13.5%)	384 (15.6%)
Unsure	360 (11.0%)	256 (10.4%)
(Missing)	37 (1.1%)	13 (0.5%)
Trust within country, n (%)		
All people	109 (3.3%)	46 (1.9%)
Most people	553 (16.9%)	424 (17.2%)
Some people	1,452 (44.4%)	1,135 (46.0%)
Not very many people	956 (29.2%)	722 (29.3%)
None	182 (5.6%)	112 (4.6%)
(Missing)	21 (0.6%)	25 (1.0%)
Number of children		
Mean	1.2	1.1
Standard Deviation	1.5	1.4
Min, Max	0.0, 18.0	0.0, 16.0
(Missing)	131 (4.0%)	22 (0.9%)

Table S21d. Unweighted summary statistics for Wave 1 outcome variables in Mexico by retention status.

**Outcome**	**Attritors–Not Observed in Wave 2**<br>N = 3,274	**Retained–Observed in Wave 2**<br>N = 2,465
Community participation, n (%)		
More than once a week	235 (7.2%)	145 (5.9%)
Once a week	320 (9.8%)	201 (8.2%)
One to three times a month	296 (9.0%)	220 (8.9%)
A few times a year	967 (29.5%)	773 (31.4%)
Never	1,441 (44.0%)	1,112 (45.1%)
(Missing)	15 (0.5%)	14 (0.6%)
Religious attendance, n (%)		
More than once a week	316 (9.7%)	285 (11.6%)
Once a week	701 (21.4%)	540 (21.9%)
One to three times a month	404 (12.4%)	263 (10.7%)
A few times a year	1,172 (35.8%)	874 (35.5%)
Never	659 (20.1%)	479 (19.4%)
(Missing)	20 (0.6%)	23 (0.9%)
Loneliness		
Mean	3.4	3.3
Standard Deviation	3.3	3.1
Min, Max	0.0, 10.0	0.0, 10.0
(Missing)	8 (0.3%)	7 (0.3%)
Perceived discrimination, n (%)		
Always	289 (8.8%)	144 (5.9%)
Often	507 (15.5%)	375 (15.2%)
Rarely	1,092 (33.3%)	976 (39.6%)
Never	1,364 (41.7%)	950 (38.5%)
(Missing)	22 (0.7%)	20 (0.8%)
Orientation to promote good		
Mean	8.6	8.6
Standard Deviation	1.6	1.5
Min, Max	0.0, 10.0	0.0, 10.0
(Missing)	20 (0.6%)	13 (0.5%)
Delayed gratification		
Mean	8.2	8.3
Standard Deviation	2.1	2.0
Min, Max	0.0, 10.0	0.0, 10.0
(Missing)	26 (0.8%)	23 (0.9%)
Hope		
Mean	9.0	9.0
Standard Deviation	1.6	1.5
Min, Max	0.0, 10.0	0.0, 10.0
(Missing)	20 (0.6%)	11 (0.4%)
Gratitude		
Mean	8.8	8.9
Standard Deviation	1.9	1.6
Min, Max	0.0, 10.0	0.0, 10.0
(Missing)	13 (0.4%)	10 (0.4%)
Showing love/care		
Mean	8.7	8.7
Standard Deviation	1.9	1.8
Min, Max	0.0, 10.0	0.0, 10.0

Table S21d. Unweighted summary statistics for Wave 1 outcome variables in Mexico by retention status.

**Outcome**	**Attritors–Not Observed in Wave 2** N = 3,274	**Retained–Observed in Wave 2** N = 2,465
(Missing)	11 (0.3%)	10 (0.4%)
Forgivingness, n (%)		
Always	1,300 (39.7%)	928 (37.6%)
Often	1,154 (35.2%)	1,010 (41.0%)
Rarely	624 (19.0%)	392 (15.9%)
Never	182 (5.6%)	120 (4.9%)
(Missing)	14 (0.4%)	15 (0.6%)
Charitable giving, n (%)		
Yes	599 (18.3%)	558 (22.6%)
No	2,669 (81.5%)	1,902 (77.2%)
(Missing)	6 (0.2%)	4 (0.2%)
Helping strangers, n (%)		
Yes	2,058 (62.9%)	1,535 (62.3%)
No	1,200 (36.6%)	916 (37.2%)
(Missing)	16 (0.5%)	14 (0.6%)
Volunteering, n (%)		
Yes	674 (20.6%)	544 (22.1%)
No	2,583 (78.9%)	1,916 (77.7%)
(Missing)	16 (0.5%)	5 (0.2%)
Self-rated physical health		
Mean	7.8	7.7
Standard Deviation	2.1	1.8
Min, Max	0.0, 10.0	0.0, 10.0
(Missing)	11 (0.3%)	5 (0.2%)
Health problems, n (%)		
Yes	528 (16.1%)	446 (18.1%)
No	2,568 (78.5%)	1,986 (80.6%)
(Missing)	177 (5.4%)	33 (1.4%)
Pain in past 4 weeks, n (%)		
A lot	341 (10.4%)	226 (9.1%)
Some	857 (26.2%)	739 (30.0%)
Not very much	1,077 (32.9%)	900 (36.5%)
None at all	992 (30.3%)	599 (24.3%)
(Missing)	7 (0.2%)	1 (0.0%)
Number of cigarettes per day		
Mean	1.2	1.2
Standard Deviation	4.6	3.9
Min, Max	0.0, 97.0	0.0, 50.0
(Missing)	85 (2.6%)	37 (1.5%)
Number of drinks per week		
Mean	1.3	1.5
Standard Deviation	3.4	3.7
Min, Max	0.0, 70.0	0.0, 97.0
(Missing)	72 (2.2%)	32 (1.3%)
Days exercise per week		
Mean	2.4	2.5
Standard Deviation	2.6	2.5
Min, Max	0.0, 7.0	0.0, 7.0
(Missing)	19 (0.6%)	13 (0.5%)

Table S21d. Unweighted summary statistics for Wave 1 outcome variables in Mexico by retention status.

Outcome	Attritors–Not Observed in Wave 2 N = 3,274	Retained–Observed in Wave 2 N = 2,465
Financial security		
Mean	4.8	4.9
Standard Deviation	3.7	3.6
Min, Max	0.0, 10.0	0.0, 10.0
(Missing)	6 (0.2%)	2 (<0.1%)
Material security		
Mean	4.7	4.9
Standard Deviation	3.8	3.7
Min, Max	0.0, 10.0	0.0, 10.0
(Missing)	12 (0.4%)	9 (0.4%)
Educational attainment (16+ years), n (%)		
Up to 8	795 (24.3%)	506 (20.5%)
9-15	1,934 (59.1%)	1,270 (51.5%)
16+	543 (16.6%)	689 (28.0%)
(Missing)	1 (0.0%)	0 (0%)
Currently employed, n (%)		
Employed for an employer	1,047 (32.0%)	821 (33.3%)
Self-employed	536 (16.4%)	507 (20.6%)
Retired	174 (5.3%)	207 (8.4%)
Student	182 (5.6%)	98 (4.0%)
Homemaker	718 (21.9%)	559 (22.7%)
Unemployed and looking for a job	388 (11.8%)	187 (7.6%)
None of these/Other	109 (3.3%)	64 (2.6%)
(Missing)	120 (3.7%)	23 (0.9%)
Financially comfortable/getting by, n (%)		
Living comfortably on present income	443 (13.5%)	327 (13.3%)
Getting by on present income	1,021 (31.2%)	912 (37.0%)
Finding it difficult on present income	1,170 (35.8%)	867 (35.2%)
Finding it very difficult on present income	398 (12.2%)	284 (11.5%)
(Missing)	241 (7.4%)	75 (3.1%)
Own home, n (%)		
Someone in this household owns this home	1,513 (46.2%)	1,452 (58.9%)
Someone in this household rents this home	555 (17.0%)	359 (14.6%)
Both	217 (6.6%)	153 (6.2%)
Neither	740 (22.6%)	451 (18.3%)
Rent	0 (0%)	0 (0%)
Own	0 (0%)	0 (0%)
Something else	0 (0%)	0 (0%)
(Missing)	248 (7.6%)	50 (2.0%)
Religious/spiritual connection, n (%)		
Always	1,099 (33.6%)	879 (35.7%)
Often	1,022 (31.2%)	804 (32.6%)
Rarely	846 (25.8%)	562 (22.8%)
Never	291 (8.9%)	206 (8.4%)
(Missing)	15 (0.5%)	15 (0.6%)
Belief in life after death, n (%)		
Yes	1,915 (58.5%)	1,492 (60.5%)
No	492 (15.0%)	346 (14.0%)
Unsure	827 (25.3%)	589 (23.9%)

Table S21d. Unweighted summary statistics for Wave 1 outcome variables in Mexico by retention status.

**Outcome**	**Attritors–Not Observed in Wave 2** N = 3,274	**Retained–Observed in Wave 2** N = 2,465
(Missing)	39 (1.2%)	38 (1.5%)
Transformative religious experience, n (%)		
Yes	1,388 (42.4%)	1,148 (46.6%)
No	1,858 (56.7%)	1,293 (52.5%)
(Missing)	28 (0.9%)	24 (1.0%)
Religious reading or listening, n (%)		
More than once a day	278 (8.5%)	212 (8.6%)
About once a day	419 (12.8%)	381 (15.4%)
Sometimes	1,781 (54.4%)	1,322 (53.6%)
Never	762 (23.3%)	522 (21.2%)
(Missing)	33 (1.0%)	29 (1.2%)
Prayer or meditation, n (%)		
More than once a day	509 (15.5%)	482 (19.5%)
About once a day	944 (28.8%)	745 (30.2%)
Sometimes	1,398 (42.7%)	970 (39.4%)
Never	391 (12.0%)	253 (10.3%)
(Missing)	31 (1.0%)	15 (0.6%)
Belief in God/gods/spiritual forces, n (%)		
One God	2,543 (77.7%)	1,948 (79.0%)
More than one god	148 (4.5%)	87 (3.5%)
An impersonal spiritual force	230 (7.0%)	233 (9.4%)
None of these	115 (3.5%)	91 (3.7%)
Unsure	205 (6.3%)	94 (3.8%)
(Missing)	32 (1.0%)	12 (0.5%)
Religious centrality, n (%)		
Agree	1,665 (50.9%)	1,394 (56.6%)
Disagree	369 (11.3%)	260 (10.5%)
Not relevant	572 (17.5%)	418 (17.0%)
Unsure	597 (18.2%)	367 (14.9%)
(Missing)	70 (2.1%)	25 (1.0%)
Religious/spiritual comfort, n (%)		
Agree	2,072 (63.3%)	1,723 (69.9%)
Disagree	215 (6.6%)	183 (7.4%)
Not relevant	487 (14.9%)	318 (12.9%)
Unsure	471 (14.4%)	236 (9.6%)
(Missing)	29 (0.9%)	6 (0.2%)
Feel loved by God, n (%)		
Agree	2,482 (75.8%)	1,950 (79.1%)
Disagree	152 (4.7%)	123 (5.0%)
Not relevant	344 (10.5%)	225 (9.1%)
Unsure	265 (8.1%)	157 (6.4%)
(Missing)	31 (0.9%)	10 (0.4%)
Feel punished by God, n (%)		
Agree	548 (16.7%)	311 (12.6%)
Disagree	1,594 (48.7%)	1,391 (56.5%)
Not relevant	561 (17.1%)	408 (16.6%)
Unsure	523 (16.0%)	342 (13.9%)
(Missing)	48 (1.5%)	13 (0.5%)
Experienced religious criticism, n (%)		

Table S21d. Unweighted summary statistics for Wave 1 outcome variables in Mexico by retention status.

**Outcome**	**Attritors–Not Observed in Wave 2** N = 3,274	**Retained–Observed in Wave 2** N = 2,465
Agree	798 (24.4%)	525 (21.3%)
Disagree	944 (28.8%)	681 (27.6%)
Not relevant	677 (20.7%)	617 (25.0%)
Unsure	812 (24.8%)	612 (24.8%)
(Missing)	43 (1.3%)	30 (1.2%)
Faith-sharing, n (%)		
Agree	1,417 (43.3%)	1,207 (49.0%)
Disagree	717 (21.9%)	491 (19.9%)
Not relevant	695 (21.2%)	515 (20.9%)
Unsure	405 (12.4%)	234 (9.5%)
(Missing)	40 (1.2%)	18 (0.7%)

Note. N (%); this table is based on non-imputed data. Cumulative percentages for variables may not add up to 100% due to rounding.

Table S21e. Summary of fitted attrition model in Mexico

**Characteristic**	**Odds Ratio**	**95% CI**	**p-value**
**ANNUAL_WEIGHT_R2**	0.97	0.89, 1.06	0.473
**Recruitment Survey Mode**			
CAWI	—	—	
CATI	1.93	1.59, 2.35	4.78e-11
CAPI	1.60	1.30, 1.97	8.69e-06
**Happiness & life satisfaction**	0.99	0.89, 1.11	0.916
**Physical & mental health**	0.97	0.88, 1.07	0.557
**Meaning & purpose**	1.04	0.93, 1.16	0.463
**Character & virtue**	1.02	0.94, 1.10	0.713
**Close social relationships**	0.93	0.84, 1.02	0.126
**Financial & material security**	0.99	0.92, 1.07	0.825
**Extraversion**	0.92	0.85, 0.99	0.025
**Openness to experience**	1.11	1.03, 1.20	0.007
**Agreeableness**	1.02	0.94, 1.11	0.650
**Conscientiousness**	0.97	0.89, 1.05	0.394
**Neuroticism**	1.05	0.97, 1.14	0.233
**Depression symptoms composite**	0.94	0.86, 1.03	0.190
**Anxiety symptoms composite**	0.99	0.90, 1.08	0.809
**Loneliness**	1.03	0.94, 1.12	0.571
**Days exercise per week**	0.98	0.91, 1.06	0.605
**Year of birth (age group)**			
1983-1993 (current age: 30-39 years)	—	—	
1998-2005 (current age: 18-24 years)	0.62	0.48, 0.81	3.48e-04
1973-1983 (current age: 40-49 years)	1.25	1.01, 1.56	0.044
1993-1998 (current age: 25-29 years)	0.86	0.67, 1.09	0.210
1963-1973 (current age: 50-59 years)	1.91	1.50, 2.43	1.54e-07
1953-1963 (current age: 60-69 years)	2.17	1.58, 2.97	1.53e-06
1943-1953 (current age: 70-79 years)	2.60	1.61, 4.21	9.79e-05
1943 or earlier (current age: 80+ years)	2.34	1.03, 5.32	0.043
**Gender of respondent**			
Female	—	—	
Male	0.89	0.76, 1.06	0.194
Other	0.99	0.22, 4.52	0.988
**Marital status**			
Married	—	—	
Single/Never been married	0.98	0.78, 1.22	0.824
Domestic partner	0.81	0.65, 1.01	0.060
Separated	0.89	0.67, 1.20	0.451
Widowed	0.69	0.48, 1.00	0.050
Divorced	0.93	0.65, 1.34	0.710
**Employment status**			
Employed for an employer	—	—	
Homemaker	1.09	0.87, 1.37	0.433
Self-employed	1.08	0.87, 1.33	0.497
Unemployed and looking for a job	0.77	0.58, 1.01	0.061
Retired	0.69	0.46, 1.04	0.080
Student	1.18	0.77, 1.80	0.450
None of these/Other	0.79	0.51, 1.24	0.304
**Religious attendance**			
A few times a year	—	—	
Once a week	0.95	0.77, 1.16	0.595
Never	1.00	0.81, 1.24	0.988

Table S21e. Summary of fitted attrition model in Mexico

**Characteristic**	**Odds Ratio**	**95% CI**	**p-value**
One to three times a month	0.88	0.69, 1.13	0.317
More than once a week	1.18	0.91, 1.52	0.213
**Educational attainment (16+ years)**			
9-15	—	—	
Up to 8	0.73	0.60, 0.89	0.002
16+	1.35	1.10, 1.66	0.004
**Born in This country**			
Born in this country	—	—	
Born in another country	1.29	0.79, 2.13	0.312
**Race plurality (prominent race/ethnic group [0] or not [1])**	0.89	0.83, 0.96	0.003
**Urbanicity**			
A small town or village	—	—	
A large city	1.18	1.00, 1.40	0.050
A rural area or on a farm	1.19	0.94, 1.50	0.147
A suburb of a large city	1.66	1.23, 2.24	8.78e-04
**Monthly household income**			
Mexico: 7.001 – 10.000 Mexican pesos	—	—	
Mexico: 5.001 – 7.000 Mexican pesos	1.30	0.95, 1.77	0.100
Mexico: 10.001 – 15.000 Mexican pesos	1.21	0.88, 1.66	0.248
Mexico: 4.001 – 5.000 Mexican pesos	1.04	0.74, 1.47	0.800
(None/No household income)	0.90	0.63, 1.29	0.567
Mexico: 15.001 – 20.000 Mexican pesos	1.46	1.01, 2.10	0.043
Mexico: 3.001 – 4.000 Mexican pesos	0.80	0.55, 1.15	0.233
Mexico: 1.001 – 1.500 Mexican pesos	1.13	0.75, 1.69	0.567
Mexico: 1.501 – 2.000 Mexican pesos	1.19	0.83, 1.70	0.356
Mexico: 20.001 – 30.000 Mexican pesos	1.70	1.16, 2.49	0.007
Mexico: 2.501 – 3.000 Mexican pesos	0.95	0.65, 1.39	0.777
Mexico: 551 – 1.000 Mexican pesos	0.95	0.63, 1.43	0.816
Mexico: 2.001 – 2.500 Mexican pesos	0.77	0.52, 1.14	0.186
Mexico: 550 Mexican pesos or less	1.16	0.74, 1.83	0.520
Mexico: 30.001 – 40.000 Mexican pesos	2.20	1.30, 3.71	0.003
Mexico: More than 70.000 Mexican pesos	1.32	0.72, 2.42	0.364
Mexico: 40.001 – 50.000 Mexican pesos	3.00	1.59, 5.66	6.99e-04
Mexico: 50.001 – 70.000 Mexican pesos	1.10	0.58, 2.08	0.781

Abbreviations: CI = Confidence Interval, OR = Odds Ratio

Notes. N=5776; attrition weights were estimated using the ’survey::svyglm(family=quasibinomial(’logit’))’ function. All continuous predictors were standardized and all categorical predictors used the most common category as the reference group. Reported p-values are based on the fitted regression model and no adjustments for multiple testing were done within this table.

Table S21f. Summary of principal components in Mexico

PC	Percent Explained by each PC	Cumulative Percent Explained
1	34.16	34.16
2	5.24	39.39
3	4.48	43.87
4	2.76	46.62
5	2.11	48.74
6	1.92	50.65
**7**	**1.68**	**52.33**
8	1.51	53.84
9	1.40	55.24
10	1.36	56.60
11	1.31	57.91
12	1.27	59.17
13	1.21	60.39
14	1.17	61.55
15	1.13	62.68
16	1.10	63.77
17	1.04	64.82
18	1.03	65.85
19	1.01	66.86
20	0.97	67.83

Notes. N=5776; PCA was conducted using 'survey::svyprcomp(.)' function using all available contemporaneous exposures at wave 1. All PCs were standardized prior to being used as predictors. The bolded row represented the number of retained components for analysis was 7.

Table S21g. Associations of forgivingness with adult well-being and other outcomes at Wave 2 in Mexico

	Model 1: Demographic and Childhood Variables as Covariates					Model 2: Demographic, Childhood, and Other Wave 1 Confounding Variables (Via Principal Components) as Covariates				
Outcome	RR	ES	95% CI	SE	p-value	RR	ES	95% CI	SE	p-value
Human Flourishing										
Secure flourishing index		0.11	(0.07,0.14)	0.02	2.48e-09***		0.06	(0.03,0.09)	0.02	1.27e-04***
Flourishing index		0.09	(0.05,0.12)	0.02	4.23e-06***		0.04	(0.01,0.07)	0.02	0.015*
Happiness & life satisfaction		0.07	(0.03,0.11)	0.02	1.68e-03**		0.03	(-0.01,0.07)	0.02	0.099
Physical & mental health		0.06	(0.02,0.10)	0.02	2.2e-03**		0.03	(-0.01,0.06)	0.02	0.121
Meaning & purpose		0.05	(0.01,0.10)	0.02	0.024*		0.01	(-0.03,0.06)	0.02	0.550
Character & virtue		0.08	(0.03,0.13)	0.03	4.05e-03**		0.05	(0.00,0.10)	0.03	0.050*
Close social relationships		0.06	(0.02,0.10)	0.02	0.006*		0.03	(-0.01,0.07)	0.02	0.212
Financial & material security		0.08	(0.05,0.12)	0.02	1.19e-05***		0.06	(0.03,0.09)	0.02	3.18e-04***
Psychological Well-Being										
Happiness (a)		0.07	(0.02,0.11)	0.02	0.006*		0.04	(-0.00,0.09)	0.02	0.082
Life satisfaction (a)		0.05	(-0.00,0.09)	0.02	0.054		0.02	(-0.03,0.06)	0.02	0.480
Current life evaluation		0.02	(-0.02,0.07)	0.02	0.246		-0.01	(-0.05,0.03)	0.02	0.800
Future life evaluation		-0.01	(-0.06,0.03)	0.02	0.514		-0.04	(-0.08,0.01)	0.02	0.094
Optimism		0.00	(-0.04,0.05)	0.02	0.877		-0.03	(-0.07,0.02)	0.02	0.283
Freedom to pursue what's important		0.06	(0.01,0.10)	0.02	0.024*		0.02	(-0.02,0.07)	0.02	0.300
Inner peace	1.01		(0.99,1.03)	0.01	0.327	1.00		(0.99,1.02)	0.01	0.713
Life balance	1.02		(0.99,1.04)	0.01	0.179	1.00		(0.98,1.02)	0.01	0.934
Sense of mastery	1.01		(1.00,1.03)	0.01	0.050	1.01		(0.99,1.02)	0.01	0.395
Meaningful activities (c)		0.04	(-0.00,0.08)	0.02	0.073		0.01	(-0.04,0.05)	0.02	0.714
Understanding purpose (c)		0.05	(0.00,0.09)	0.02	0.050		0.01	(-0.03,0.06)	0.02	0.556
Self-rated mental health (b)		0.04	(-0.01,0.09)	0.03	0.085		0.01	(-0.04,0.06)	0.03	0.630
Psychological Distress										
Traumatic distress	1.00		(0.97,1.02)	0.01	0.784	1.00		(0.97,1.02)	0.01	0.786
Depression symptoms composite	0.98		(0.96,1.00)	0.01	0.018*	0.99		(0.97,1.01)	0.01	0.267
Depression – feel hopeless	0.98		(0.96,0.99)	0.01	0.014*	0.99		(0.97,1.00)	0.01	0.155
Depression – loss of interest	0.99		(0.97,1.01)	0.01	0.202	1.00		(0.98,1.02)	0.01	0.815
Anxiety symptoms composite	0.97		(0.95,0.99)	0.01	4.97e-03**	0.98		(0.96,1.00)	0.01	0.049*
Anxiety – feel on edge	0.98		(0.95,1.00)	0.01	0.032*	0.98		(0.96,1.01)	0.01	0.176
Anxiety – cannot stop worrying	0.98		(0.96,1.00)	0.01	0.026*	0.98		(0.96,1.00)	0.01	0.090
Suffering	0.99		(0.97,1.01)	0.01	0.241	0.99		(0.97,1.01)	0.01	0.443
Social Well-Being										
Relationship contentment (e)		0.05	(0.01,0.09)	0.02	0.017*		0.02	(-0.02,0.07)	0.02	0.257
Relationship satisfaction (e)		0.05	(0.00,0.10)	0.02	0.040*		0.02	(-0.03,0.07)	0.02	0.400
Social support		0.03	(-0.01,0.07)	0.02	0.171		0.01	(-0.03,0.05)	0.02	0.699
Intimate/close friend	1.03		(1.01,1.06)	0.01	1.44e-03**	1.02		(1.00,1.05)	0.01	0.030*
Government approval	1.01		(0.99,1.04)	0.01	0.191	1.01		(0.99,1.03)	0.01	0.459
Say in government	1.00		(0.98,1.02)	0.01	0.904	0.99		(0.97,1.01)	0.01	0.495
Belonging in country		0.04	(-0.01,0.10)	0.03	0.124		0.02	(-0.03,0.07)	0.03	0.462
City/place satisfaction	1.00		(0.98,1.02)	0.01	0.882	0.99		(0.97,1.01)	0.01	0.437
Trust within country	1.02		(0.99,1.05)	0.01	0.178	1.02		(0.99,1.04)	0.01	0.252
Social Participation										
Ever been married	1.00		(0.98,1.02)	0.01	0.964	1.00		(0.98,1.02)	0.01	0.907
Currently divorced	1.00		(0.99,1.01)	0.00	0.863	1.00		(0.99,1.01)	0.00	0.936
Number of children		-0.03	(-0.06,0.01)	0.02	0.163		-0.01	(-0.05,0.02)	0.02	0.513
Weekly+ community participation	1.00		(0.99,1.02)	0.01	0.499	1.00		(0.99,1.02)	0.01	0.832
Weekly+ religious attendance	1.00		(0.98,1.02)	0.01	0.973	1.00		(0.98,1.01)	0.01	0.622
Social Distress										
Loneliness		-0.07	(-0.12,-0.02)	0.03	0.006*		-0.05	(-0.10,0.00)	0.03	0.061
Perceived discrimination	1.00		(0.98,1.02)	0.01	0.897	1.00		(0.98,1.03)	0.01	0.678
Character & Prosocial Behavior										
Orientation to promote good (d)		0.07	(0.02,0.12)	0.02	4.66e-03**		0.05	(0.00,0.10)	0.02	0.052
Delayed gratification (d)		0.05	(0.00,0.10)	0.03	0.048*		0.03	(-0.02,0.08)	0.03	0.200
Hope		0.03	(-0.02,0.08)	0.03	0.262		0.00	(-0.05,0.06)	0.03	0.880
Gratitude		0.06	(0.01,0.11)	0.02	0.020*		0.03	(-0.02,0.07)	0.02	0.303
Showing love/care		0.06	(0.01,0.10)	0.02	0.022*		0.03	(-0.02,0.08)	0.02	0.221
Forgivingness	1.12		(1.09,1.14)	0.01	4.21e-13***	1.11		(1.09,1.14)	0.01	1.83e-12***
Charitable giving	1.02		(1.00,1.04)	0.01	0.110	1.01		(0.99,1.03)	0.01	0.532
Helping strangers	1.02		(0.99,1.04)	0.01	0.159	1.01		(0.98,1.03)	0.01	0.579
Volunteering	1.01		(1.00,1.03)	0.01	0.171	1.01		(0.99,1.02)	0.01	0.436
Physical Health & Health Behavior										
Self-rated physical health (b)		0.05	(0.01,0.09)	0.02	0.008*		0.03	(-0.00,0.07)	0.02	0.068
Health problems	1.00		(0.98,1.02)	0.01	0.827	1.00		(0.98,1.01)	0.01	0.711
Pain in past 4 weeks	0.98		(0.96,1.01)	0.01	0.135	0.98		(0.96,1.01)	0.01	0.160
Daily smoker	1.00		(0.98,1.01)	0.01	0.585	1.00		(0.98,1.01)	0.01	0.629
Number of drinks per week		-0.01	(-0.05,0.03)	0.02	0.672		-0.00	(-0.05,0.04)	0.02	0.821
Days exercise per week		-0.02	(-0.06,0.02)	0.02	0.401		-0.03	(-0.07,0.01)	0.02	0.129
Socioeconomic Outcomes										
Financial security (f)		0.06	(0.02,0.10)	0.02	0.006*		0.04	(-0.00,0.08)	0.02	0.057
Material security (f)		0.08	(0.04,0.12)	0.02	5.62e-04***		0.06	(0.02,0.11)	0.02	3.83e-03**
Educational attainment (16+ years)	0.99		(0.98,1.00)	0.00	0.109	0.99		(0.98,1.00)	0.00	0.104
Currently employed	0.99		(0.97,1.01)	0.01	0.501	0.99		(0.97,1.01)	0.01	0.422
Financially comfortable/getting by	0.99		(0.97,1.02)	0.01	0.629	0.99		(0.96,1.01)	0.01	0.300
Own home	0.99		(0.97,1.00)	0.01	0.120	0.98		(0.97,1.00)	0.01	0.093
Income – top quintile	1.00		(0.98,1.02)	0.01	0.730	1.00		(0.98,1.02)	0.01	0.774
Religion & Spirituality										
Religious/spiritual connection	1.03		(1.01,1.05)	0.01	1.04e-03**	1.02		(1.00,1.03)	0.01	0.104
Belief in life after death	1.04		(1.02,1.06)	0.01	4.57e-04***	1.02		(1.00,1.04)	0.01	0.040*
Transformative religious experience	1.04		(1.02,1.06)	0.01	1.96e-04***	1.02		(1.00,1.04)	0.01	0.033*
Religious reading or listening	1.02		(1.00,1.03)	0.01	0.052	1.00		(0.99,1.02)	0.01	0.550
Prayer or meditation	1.03		(1.01,1.04)	0.01	0.007*	1.01		(0.99,1.02)	0.01	0.395
Belief in God/gods/spiritual forces	1.01		(1.00,1.02)	0.01	0.236	1.00		(0.99,1.01)	0.01	0.861
Religious centrality	1.03		(1.01,1.05)	0.01	0.007*	1.01		(0.99,1.03)	0.01	0.261
Religious/spiritual comfort	1.03		(1.00,1.05)	0.01	0.028*	1.01		(0.99,1.03)	0.01	0.468
Feel loved by God	1.01		(0.99,1.02)	0.01	0.453	0.99		(0.98,1.01)	0.01	0.286
Feel punished by God	1.00		(0.98,1.01)	0.01	0.644	1.00		(0.98,1.02)	0.01	0.851
Experienced religious criticism	1.00		(0.98,1.02)	0.01	0.887	1.00		(0.98,1.02)	0.01	0.697
Faith-sharing	1.03		(1.01,1.05)	0.01	0.007*	1.01		(0.99,1.03)	0.01	0.247

Notes. N=5776; Reference for focal predictor: never/rarely. RR, risk-ratio, null effect is 1.00; ES, effect size measure for standardized regression coefficient, null effect is 0.00; SE, standard error, the SE reported for binary/Likert-type outcomes where risk-ratios are on the log(RR) scale; CI, confidence interval; p-value, a Wald-type test of the null hypothesis that the effect of the focal predictor is zero; (a) item part of the Happiness & Life Satisfaction domain of the Secure Flourishing Index; (b) item part of the Physical & Mental Health domain of the Secure Flourishing Index; (c) item part of the Meaning & Purpose domain of the Secure Flourishing Index; (d) item part of the Character & Virtue domain of the Secure Flourishing Index; (e) item part of the Subjective Social Connectedness domain of the Secure Flourishing Index; (f) item part of the Financial & Material Security domain of the Secure Flourishing Index.

Multiple imputation was performed to impute missing data on the covariates, exposure, and outcomes. All models controlled for sociodemographic and childhood factors assessed at Wave 1. For Model 2 with PC (principal components), the first seven principal components of the entire set of contemporaneous confounders assessed at Wave 1 were included as additional covariates of the outcomes at Wave 2.

An outcome-wide analytic approach was used, and a separate model was run for each outcome. A different type of model was run depending on the nature of the outcome: (1) for each binary outcome, a weighted generalized linear model (with a log link and Poisson distribution) was used to estimate an RR; and (2) for each continuous outcome, a weighted linear regression model was used to estimate a ES. All effect sizes were standardized. For continuous outcomes, the ES represents the change in SD on the outcome between the lower and upper categories of the binary focal predictor. For binary outcomes, the RR represents the change in risk of being in the upper category compared to the lower category between the lower and upper categories of the binary focal predictor.

P-value significance thresholds: $p < 0.05$*, $p < 0.005$**, (Bonferroni) $p < 6.41e-04$***, correction for multiple testing using Bonferroni adjusted significant threshold.

Table S21h. Associations of forgivingness with adult well-being and other outcomes at Wave 2 in Mexico using complete-case analyses with attrition weights.

	Model 1: Demographic and Childhood Variables as Covariates					Model 2: Demographic, Childhood, and Other Wave 1 Confounding Variables (Via Principal Components) as Covariates				
Outcome	RR	ES	95% CI	SE	p-value	RR	ES	95% CI	SE	p-value
Human Flourishing										
Secure flourishing index		0.11	(0.05,0.16)	0.03	6.64e-05***		0.07	(0.02,0.12)	0.02	2.86e-03**
Flourishing index		0.10	(0.04,0.15)	0.03	5.64e-04***		0.05	(0.01,0.10)	0.02	0.029*
Happiness & life satisfaction		0.09	(0.04,0.14)	0.03	7.04e-04**		0.06	(0.02,0.11)	0.02	0.009*
Physical & mental health		0.05	(0.00,0.11)	0.03	0.049*		0.02	(-0.03,0.07)	0.02	0.367
Meaning & purpose		0.07	(0.01,0.12)	0.03	0.019*		0.03	(-0.03,0.08)	0.03	0.317
Character & virtue		0.09	(0.04,0.15)	0.03	1.32e-03**		0.06	(0.01,0.12)	0.03	0.024*
Close social relationships		0.08	(0.02,0.13)	0.03	0.006*		0.04	(-0.01,0.09)	0.03	0.132
Financial & material security		0.07	(0.02,0.11)	0.03	0.009*		0.06	(0.02,0.11)	0.02	0.008*
Psychological Well-Being										
Happiness (a)		0.08	(0.03,0.13)	0.03	1.29e-03**		0.06	(0.01,0.11)	0.03	0.012*
Life satisfaction (a)		0.08	(0.02,0.13)	0.03	0.006*		0.05	(0.00,0.10)	0.03	0.043*
Current life evaluation		0.02	(-0.03,0.08)	0.03	0.418		-0.00	(-0.05,0.05)	0.03	0.946
Future life evaluation		0.00	(-0.05,0.05)	0.02	0.950		-0.02	(-0.07,0.03)	0.02	0.475
Optimism		0.02	(-0.03,0.08)	0.03	0.430		-0.01	(-0.07,0.04)	0.03	0.713
Freedom to pursue what's important		0.08	(0.02,0.14)	0.03	0.012*		0.04	(-0.02,0.10)	0.03	0.166
Inner peace	1.01		(0.99,1.04)	0.01	0.227	1.00		(0.98,1.03)	0.01	0.723
Life balance	1.01		(0.98,1.03)	0.01	0.510	0.99		(0.97,1.01)	0.01	0.498
Sense of mastery	1.01		(0.99,1.03)	0.01	0.366	1.00		(0.98,1.02)	0.01	0.924
Meaningful activities (c)		0.07	(0.01,0.12)	0.03	0.021*		0.03	(-0.02,0.09)	0.03	0.220
Understanding purpose (c)		0.05	(-0.00,0.11)	0.03	0.057		0.01	(-0.04,0.07)	0.03	0.578
Self-rated mental health (b)		0.05	(-0.00,0.10)	0.03	0.072		0.01	(-0.04,0.06)	0.02	0.603
Psychological Distress										
Traumatic distress	0.99		(0.97,1.02)	0.01	0.553	0.99		(0.96,1.01)	0.01	0.333
Depression symptoms composite	0.99		(0.97,1.01)	0.01	0.413	1.00		(0.98,1.02)	0.01	0.878
Depression – feel hopeless	0.99		(0.97,1.01)	0.01	0.260	0.99		(0.97,1.02)	0.01	0.621
Depression – loss of interest	0.99		(0.97,1.02)	0.01	0.518	1.00		(0.98,1.02)	0.01	0.893
Anxiety symptoms composite	0.98		(0.96,1.00)	0.01	0.090	0.99		(0.96,1.01)	0.01	0.193
Anxiety – feel on edge	0.98		(0.95,1.00)	0.01	0.035*	0.98		(0.96,1.01)	0.01	0.133
Anxiety – cannot stop worrying	0.99		(0.96,1.01)	0.01	0.221	0.99		(0.97,1.01)	0.01	0.395
Suffering	1.00		(0.97,1.02)	0.01	0.892	1.00		(0.97,1.02)	0.01	0.828
Social Well-Being										
Relationship contentment (e)		0.08	(0.03,0.14)	0.03	3.73e-03**		0.05	(-0.00,0.10)	0.03	0.067
Relationship satisfaction (e)		0.06	(0.01,0.12)	0.03	0.030*		0.02	(-0.03,0.08)	0.03	0.346
Social support		0.03	(-0.02,0.08)	0.03	0.298		0.01	(-0.05,0.06)	0.03	0.834
Intimate/close friend	1.04		(1.01,1.06)	0.01	0.006*	1.02		(1.00,1.05)	0.01	0.054
Government approval	1.02		(1.00,1.05)	0.01	0.112	1.01		(0.98,1.03)	0.01	0.645
Say in government	0.99		(0.97,1.02)	0.01	0.513	0.98		(0.96,1.00)	0.01	0.079
Belonging in country		0.05	(-0.00,0.11)	0.03	0.053		0.02	(-0.03,0.08)	0.03	0.371
City/place satisfaction	1.00		(0.97,1.02)	0.01	0.706	0.99		(0.97,1.01)	0.01	0.362
Trust within country	1.02		(0.99,1.04)	0.01	0.187	1.01		(0.99,1.04)	0.01	0.302
Social Participation										
Ever been married	1.01		(0.99,1.03)	0.01	0.246	1.01		(0.99,1.03)	0.01	0.347
Currently divorced	1.00		(0.99,1.01)	0.00	0.619	1.00		(0.99,1.01)	0.00	0.802
Number of children		-0.03	(-0.09,0.03)	0.03	0.282		-0.04	(-0.09,0.02)	0.03	0.182
Weekly+ community participation	1.00		(0.98,1.02)	0.01	0.778	0.99		(0.98,1.01)	0.01	0.569
Weekly+ religious attendance	1.00		(0.97,1.02)	0.01	0.746	0.99		(0.97,1.02)	0.01	0.536
Social Distress										
Loneliness		-0.06	(-0.12,-0.01)	0.03	0.018*		-0.04	(-0.09,0.01)	0.03	0.092
Perceived discrimination	1.00		(0.98,1.03)	0.01	0.875	1.01		(0.98,1.03)	0.01	0.603
Character & Prosocial Behavior										
Orientation to promote good (d)		0.08	(0.03,0.14)	0.03	4.11e-03**		0.06	(0.00,0.11)	0.03	0.043*
Delayed gratification (d)		0.07	(0.01,0.13)	0.03	0.014*		0.05	(-0.01,0.11)	0.03	0.095
Hope		0.05	(-0.01,0.10)	0.03	0.080		0.02	(-0.04,0.07)	0.03	0.551
Gratitude		0.07	(0.02,0.13)	0.03	0.011*		0.04	(-0.01,0.10)	0.03	0.147
Showing love/care		0.07	(0.01,0.12)	0.03	0.016*		0.04	(-0.02,0.09)	0.03	0.191
Forgivingness	1.14		(1.11,1.17)	0.01	2.22e-16***	1.13		(1.10,1.16)	0.01	2.22e-16***
Charitable giving	1.01		(0.99,1.03)	0.01	0.420	1.01		(0.98,1.03)	0.01	0.625
Helping strangers	1.02		(1.00,1.05)	0.01	0.083	1.01		(0.99,1.04)	0.01	0.408
Volunteering	1.01		(0.99,1.03)	0.01	0.306	1.01		(0.99,1.03)	0.01	0.350
Physical Health & Health Behavior										
Self-rated physical health (b)		0.05	(-0.01,0.10)	0.03	0.094		0.03	(-0.02,0.08)	0.03	0.309
Health problems	1.00		(0.99,1.02)	0.01	0.638	1.00		(0.98,1.02)	0.01	0.989
Pain in past 4 weeks	0.98		(0.96,1.01)	0.01	0.203	0.98		(0.96,1.01)	0.01	0.152
Daily smoker	1.00		(0.97,1.02)	0.01	0.704	0.99		(0.97,1.02)	0.01	0.621
Number of drinks per week		0.02	(-0.03,0.06)	0.02	0.500		0.01	(-0.03,0.06)	0.02	0.601
Days exercise per week		-0.03	(-0.09,0.02)	0.03	0.256		-0.05	(-0.10,0.00)	0.03	0.073
Socioeconomic Outcomes										
Financial security (f)		0.06	(0.01,0.11)	0.02	0.019*		0.05	(0.01,0.10)	0.02	0.023*
Material security (f)		0.07	(0.01,0.12)	0.03	0.013*		0.06	(0.01,0.11)	0.02	0.010*
Educational attainment (16+ years)	0.99		(0.98,1.00)	0.01	0.134	0.99		(0.98,1.00)	0.01	0.081
Currently employed	0.99		(0.97,1.01)	0.01	0.439	0.99		(0.96,1.01)	0.01	0.229
Financially comfortable/getting by	1.00		(0.97,1.03)	0.01	0.894	1.00		(0.97,1.03)	0.01	0.919
Own home	0.97		(0.95,1.00)	0.01	0.037*	0.97		(0.95,1.00)	0.01	0.034*
Income – top quintile	1.00		(0.98,1.03)	0.01	0.725	1.01		(0.98,1.03)	0.01	0.644
Religion & Spirituality										
Religious/spiritual connection	1.03		(1.01,1.05)	0.01	0.016*	1.01		(0.99,1.04)	0.01	0.195
Belief in life after death	1.04		(1.01,1.07)	0.01	2.91e-03**	1.03		(1.00,1.05)	0.01	0.057
Transformative religious experience	1.05		(1.03,1.08)	0.01	3.2e-05***	1.04		(1.02,1.07)	0.01	1.14e-03**
Religious reading or listening	1.01		(0.99,1.03)	0.01	0.174	1.01		(0.99,1.03)	0.01	0.419
Prayer or meditation	1.02		(0.99,1.04)	0.01	0.157	1.00		(0.98,1.03)	0.01	0.725
Belief in God/gods/spiritual forces	1.02		(1.00,1.03)	0.01	0.059	1.01		(0.99,1.02)	0.01	0.299
Religious centrality	1.03		(1.00,1.05)	0.01	0.052	1.01		(0.98,1.03)	0.01	0.677
Religious/spiritual comfort	1.02		(0.99,1.04)	0.01	0.121	1.00		(0.98,1.02)	0.01	0.884
Feel loved by God	1.01		(0.99,1.03)	0.01	0.449	0.99		(0.97,1.01)	0.01	0.484
Feel punished by God	1.00		(0.98,1.02)	0.01	0.730	1.00		(0.98,1.02)	0.01	0.747
Experienced religious criticism	1.01		(0.99,1.04)	0.01	0.284	1.01		(0.98,1.03)	0.01	0.491
Faith-sharing	1.03		(1.00,1.06)	0.01	0.025*	1.01		(0.99,1.04)	0.01	0.331

Table S21h. Associations of forgivingness with adult well-being and other outcomes at Wave 2 in Mexico using complete-case analyses with attrition weights.

	Model 1: Demographic and Childhood Variables as Covariates					Model 2: Demographic, Childhood, and Other Wave 1 Confounding Variables (Via Principal Components) as Covariates				
Outcome	RR	ES	95% CI	SE	p-value	RR	ES	95% CI	SE	p-value

Notes. N=2278; Reference for focal predictor: never/rarely. RR, risk-ratio, null effect is 1.00; ES, effect size measure for standardized regression coefficient, null effect is 0.00; SE, standard error, the SE reported for binary/Likert-type outcomes where risk-ratios are on the log(RR) scale; CI, confidence interval; p-value, a Wald-type test of the null hypothesis that the effect of the focal predictor is zero; (a) item part of the Happiness & Life Satisfaction domain of the Secure Flourishing Index; (b) item part of the Physical & Mental Health domain of the Secure Flourishing Index; (c) item part of the Meaning & Purpose domain of the Secure Flourishing Index; (d) item part of the Character & Virtue domain of the Secure Flourishing Index; (e) item part of the Subjective Social Connectedness domain of the Secure Flourishing Index; (f) item part of the Financial & Material Security domain of the Secure Flourishing Index.

Attrition weights were computed to adjust the complete case data (those who responded at Wave 2 to at least 50% of the questions) and multiple imputation was used to impute missing data on all remaining within wave on the covariates, exposure, and outcomes. All models controlled for sociodemographic and childhood factors assessed at Wave 1. For Model 2 with PC (principal components), the first seven principal components of the entire set of contemporaneous confounders assessed at Wave 1 were included as additional covariates of the outcomes at Wave 2.

An outcome-wide analytic approach was used, and a separate model was run for each outcome. A different type of model was run depending on the nature of the outcome: (1) for each binary outcome, a weighted generalized linear model (with a log link and Poisson distribution) was used to estimate an RR; and (2) for each continuous outcome, a weighted linear regression model was used to estimate a ES. All effect sizes were standardized. For continuous outcomes, the ES represents the change in SD on the outcome between the lower and upper categories of the binary focal predictor. For binary outcomes, the RR represents the change in risk of being in the upper category compared to the lower category between the lower and upper categories of the binary focal predictor.

P-value significance thresholds: $p < 0.05$*, $p < 0.005$**, (Bonferroni) $p < 6.41e-04$***, correction for multiple testing using Bonferroni adjusted significant threshold.

Table S21i. Sensitivity analysis of forgivingness outcome-wide results to unmeasured confounding using E-values in Mexico

	Multiple Imputation				Complete Case w/ Attrition Weights			
	Model 1: Demographics and Childhood Variables as Covariates		Model 2: Demographics, Childhood, and Other Wave 1 Confounders (Via Principal Components) as Covariates		Model 1: Demographics and Childhood Variables as Covariates		Model 2: Demographics, Childhood, and Other Wave 1 Confounders (Via Principal Components) as Covariates	
Outcome	EE	ECI	EE	ECI	EE	ECI	EE	ECI
Human Flourishing								
Secure flourishing index	1.82	1.61	1.54	1.33	1.83	1.50	1.60	1.29
Flourishing index	1.70	1.47	1.41	1.15	1.77	1.42	1.50	1.13
Happiness & life satisfaction	1.57	1.30	1.35	1.00	1.71	1.38	1.56	1.23
Physical & mental health	1.53	1.27	1.32	1.00	1.50	1.02	1.28	1.00
Meaning & purpose	1.49	1.15	1.20	1.00	1.59	1.18	1.31	1.00
Character & virtue	1.63	1.30	1.48	1.05	1.75	1.38	1.56	1.16
Close social relationships	1.54	1.25	1.31	1.00	1.65	1.28	1.41	1.00
Financial & material security	1.67	1.44	1.54	1.32	1.57	1.23	1.55	1.23
Psychological Well-Being								
Happiness	1.58	1.26	1.41	1.00	1.69	1.36	1.57	1.21
Life satisfaction	1.44	1.00	1.23	1.00	1.64	1.27	1.48	1.06
Current life evaluation	1.29	1.00	1.12	1.00	1.28	1.00	1.07	1.00
Future life evaluation	1.21	1.00	1.38	1.00	1.06	1.00	1.24	1.00
Optimism	1.10	1.00	1.30	1.00	1.28	1.00	1.18	1.00
Freedom to pursue what's important	1.51	1.15	1.30	1.00	1.64	1.23	1.41	1.00
Inner peace	1.17	1.00	1.10	1.00	1.22	1.00	1.11	1.00
Life balance	1.24	1.00	1.05	1.00	1.16	1.00	1.16	1.00
Sense of mastery	1.23	1.00	1.14	1.00	1.16	1.00	1.05	1.00
Meaningful activities	1.41	1.00	1.15	1.00	1.58	1.17	1.36	1.00
Understanding purpose	1.45	1.04	1.20	1.00	1.50	1.00	1.21	1.00
Self-rated mental health	1.43	1.00	1.19	1.00	1.47	1.00	1.20	1.00
Psychological Distress								
Traumatic distress	1.10	1.00	1.10	1.00	1.16	1.00	1.21	1.00
Depression symptoms composite	1.30	1.11	1.19	1.00	1.18	1.00	1.07	1.00
Depression – feel hopeless	1.30	1.12	1.22	1.00	1.21	1.00	1.13	1.00
Depression – loss of interest	1.20	1.00	1.08	1.00	1.16	1.00	1.07	1.00
Anxiety symptoms composite	1.37	1.18	1.29	1.02	1.29	1.00	1.23	1.00
Anxiety – feel on edge	1.31	1.08	1.23	1.00	1.32	1.07	1.25	1.00
Anxiety – cannot stop worrying	1.31	1.09	1.26	1.00	1.23	1.00	1.18	1.00
Suffering	1.21	1.00	1.16	1.00	1.07	1.00	1.09	1.00
Social Well-Being								
Relationship contentment	1.48	1.17	1.29	1.00	1.69	1.31	1.48	1.00
Relationship satisfaction	1.47	1.09	1.26	1.00	1.55	1.13	1.30	1.00
Social support	1.33	1.00	1.15	1.00	1.32	1.00	1.13	1.00
Intimate/close friend	1.39	1.22	1.30	1.08	1.39	1.18	1.31	1.00
Government approval	1.23	1.00	1.16	1.00	1.29	1.00	1.14	1.00
Say in government	1.06	1.00	1.16	1.00	1.16	1.00	1.29	1.00
Belonging in country	1.43	1.00	1.26	1.00	1.51	1.00	1.30	1.00
City/place satisfaction	1.06	1.00	1.16	1.00	1.12	1.00	1.19	1.00
Trust within country	1.26	1.00	1.24	1.00	1.25	1.00	1.22	1.00
Social Participation								
Ever been married	1.03	1.00	1.05	1.00	1.19	1.00	1.17	1.00
Currently divorced	1.05	1.00	1.03	1.00	1.07	1.00	1.05	1.00
Number of children	1.30	1.00	1.19	1.00	1.35	1.00	1.39	1.00
Weekly+ community participation	1.12	1.00	1.07	1.00	1.09	1.00	1.13	1.00
Weekly+ religious attendance	1.03	1.00	1.11	1.00	1.10	1.00	1.15	1.00
Social Distress								
Loneliness	1.62	1.28	1.47	1.00	1.56	1.18	1.42	1.00
Perceived discrimination	1.06	1.00	1.12	1.00	1.07	1.00	1.14	1.00
Character & Prosocial Behavior								
Orientation to promote good	1.59	1.28	1.46	1.03	1.69	1.31	1.52	1.07
Delayed gratification	1.47	1.06	1.35	1.00	1.62	1.22	1.46	1.00
Hope	1.34	1.00	1.10	1.00	1.47	1.00	1.22	1.00
Gratitude	1.52	1.17	1.30	1.00	1.62	1.24	1.41	1.00
Showing love/care	1.50	1.16	1.33	1.00	1.59	1.20	1.38	1.00
Forgivingness	1.92	1.76	1.89	1.72	2.08	1.90	2.03	1.85
Charitable giving	1.24	1.00	1.14	1.00	1.18	1.00	1.13	1.00
Helping strangers	1.25	1.00	1.15	1.00	1.30	1.00	1.19	1.00
Volunteering	1.19	1.00	1.14	1.00	1.18	1.00	1.17	1.00
Physical Health & Health Behavior								
Self-rated physical health	1.48	1.21	1.36	1.00	1.44	1.00	1.30	1.00
Health problems	1.07	1.00	1.10	1.00	1.12	1.00	1.02	1.00
Pain in past 4 weeks	1.27	1.00	1.26	1.00	1.25	1.00	1.27	1.00
Daily smoker	1.12	1.00	1.11	1.00	1.11	1.00	1.13	1.00
Number of drinks per week	1.16	1.00	1.11	1.00	1.23	1.00	1.19	1.00
Days exercise per week	1.24	1.00	1.35	1.00	1.35	1.00	1.47	1.00
Socioeconomic Outcomes								
Financial security	1.54	1.24	1.41	1.00	1.53	1.17	1.50	1.15
Material security	1.65	1.37	1.56	1.27	1.57	1.21	1.56	1.22
Educational attainment (16+ years)	1.15	1.00	1.16	1.00	1.18	1.00	1.20	1.00
Currently employed	1.16	1.00	1.17	1.00	1.17	1.00	1.22	1.00
Financially comfortable/getting by	1.13	1.00	1.21	1.00	1.07	1.00	1.06	1.00
Own home	1.22	1.00	1.23	1.00	1.32	1.06	1.32	1.07
Income – top quintile	1.10	1.00	1.09	1.00	1.12	1.00	1.14	1.00
Religion & Spirituality								
Religious/spiritual connection	1.38	1.22	1.23	1.00	1.34	1.13	1.23	1.00
Belief in life after death	1.41	1.24	1.28	1.05	1.44	1.22	1.33	1.00
Transformative religious experience	1.44	1.28	1.30	1.07	1.52	1.34	1.44	1.24
Religious reading or listening	1.23	1.00	1.12	1.00	1.21	1.00	1.15	1.00
Prayer or meditation	1.32	1.15	1.15	1.00	1.25	1.00	1.11	1.00
Belief in God/gods/spiritual forces	1.14	1.00	1.05	1.00	1.24	1.00	1.16	1.00
Religious centrality	1.36	1.16	1.20	1.00	1.32	1.00	1.12	1.00
Religious/spiritual comfort	1.34	1.09	1.17	1.00	1.27	1.00	1.07	1.00
Feel loved by God	1.14	1.00	1.16	1.00	1.16	1.00	1.15	1.00
Feel punished by God	1.11	1.00	1.07	1.00	1.10	1.00	1.10	1.00
Experienced religious criticism	1.06	1.00	1.10	1.00	1.21	1.00	1.16	1.00
Faith-sharing	1.36	1.17	1.21	1.00	1.35	1.10	1.21	1.00

Notes. EE, E-value for estimate; ECI, E-value for the limit of the confidence interval. The formula for calculating E-values can be found in VanderWeele and Ding (2017). E-values for estimate are the minimum strength of association on the risk ratio scale that an unmeasured confounder would need to have with both the exposure and the outcome to fully explain away the observed association between the exposure and outcome, conditional on the measured covariates. E-values for the 95% CI closest to the null denote the minimum strength of association on the risk ratio scale that an unmeasured confounder would need to have with both the exposure and the outcome to shift the CI to include the null value, conditional on the measured covariates.

Table S22a. Weighted summary statistics for demographic and childhood variables in Nigeria

Characteristic	Wave 1<br>N = 6,827	Wave 2<br>N = 3,145
Forgivingness, n (%)		
Always	4,276 (62.6%)	1,844 (58.6%)
Often	1,983 (29.0%)	1,013 (32.2%)
Rarely	472 (6.9%)	242 (7.7%)
Never	81 (1.2%)	45 (1.4%)
(Missing)	16 (0.2%)	1 (0.0%)
Year of birth, n (%)		
1943 or earlier (current age: 80+ years)	40 (0.6%)	23 (0.7%)
1943-1953 (current age: 70-79 years)	114 (1.7%)	60 (1.9%)
1953-1963 (current age: 60-69 years)	274 (4.0%)	138 (4.4%)
1963-1973 (current age: 50-59 years)	565 (8.3%)	259 (8.2%)
1973-1983 (current age: 40-49 years)	993 (14.6%)	502 (16.0%)
1983-1993 (current age: 30-39 years)	1,931 (28.3%)	920 (29.3%)
1993-1998 (current age: 25-29 years)	1,212 (17.8%)	552 (17.5%)
1998-2005 (current age: 18-24 years)	1,698 (24.9%)	691 (22.0%)
(Missing)	0 (0%)	0 (0%)
Age of participant		
Mean	34.5	35.9
Standard Deviation	13.3	13.4
Min, Max	18.0, 93.0	19.0, 94.0
Gender, n (%)		
Male	3,416 (50.0%)	1,586 (50.4%)
Female	3,410 (50.0%)	1,558 (49.6%)
Other	0 (<0.0%)	0 (0%)
(Missing)	0 (0%)	0 (0%)
Respondent marital status, n (%)		
Single/Never been married	2,423 (35.5%)	1,028 (32.7%)
Married	3,974 (58.2%)	1,939 (61.7%)
Separated	110 (1.6%)	54 (1.7%)
Divorced	66 (1.0%)	24 (0.8%)
Widowed	199 (2.9%)	87 (2.8%)
Domestic partner	14 (0.2%)	8 (0.3%)
(Missing)	42 (0.6%)	5 (0.1%)
Education (years), n (%)		
Up to 8	2,595 (38.0%)	1,138 (36.2%)
9-15	4,102 (60.1%)	1,873 (59.6%)
16+	129 (1.9%)	133 (4.2%)
(Missing)	2 (0.0%)	0 (0%)
Employment status, n (%)		
Employed for an employer	699 (10.2%)	506 (16.1%)
Self-employed	3,879 (56.8%)	1,828 (58.1%)
Retired	164 (2.4%)	50 (1.6%)
Student	692 (10.1%)	183 (5.8%)
Homemaker	487 (7.1%)	88 (2.8%)
Unemployed and looking for a job	698 (10.2%)	383 (12.2%)
None of these/Other	201 (3.0%)	99 (3.2%)
(Missing)	7 (0.1%)	8 (0.2%)
Current religious service attendance, n (%)		
More than once a week	4,005 (58.7%)	1,934 (61.5%)
Once a week	1,939 (28.4%)	774 (24.6%)

Table S22a. Weighted summary statistics for demographic and childhood variables in Nigeria

**Characteristic**	**Wave 1** N = 6,827	**Wave 2** N = 3,145
One to three times a month	529 (7.7%)	190 (6.0%)
A few times a year	255 (3.7%)	176 (5.6%)
Never	80 (1.2%)	67 (2.1%)
(Missing)	20 (0.3%)	4 (0.1%)
Immigration status, n (%)		
Born in this country	6,779 (99.3%)	3,125 (99.4%)
Born in another country	47 (0.7%)	20 (0.6%)
(Missing)	1 (0.0%)	0 (0%)
Parental marital status around age 12, n (%)		
Parents were married	5,558 (81.4%)	2,563 (81.5%)
Parents were divorced	309 (4.5%)	136 (4.3%)
Parents were never married	351 (5.1%)	169 (5.4%)
One or both of them had died	455 (6.7%)	207 (6.6%)
Unsure	58 (0.9%)	33 (1.1%)
(Missing)	95 (1.4%)	37 (1.2%)
Religious service attendance around age 12, n (%)		
At least once a week	5,903 (86.5%)	2,734 (87.0%)
One to three times a month	612 (9.0%)	267 (8.5%)
Less than once a month	134 (2.0%)	62 (2.0%)
Never	135 (2.0%)	61 (1.9%)
(Missing)	43 (0.6%)	21 (0.7%)
Relationship with mother when growing up, n (%)		
Very good	5,989 (87.7%)	2,728 (86.8%)
Somewhat good	648 (9.5%)	300 (9.5%)
Somewhat bad	61 (0.9%)	39 (1.2%)
Very bad	19 (0.3%)	10 (0.3%)
(Does not apply)	101 (1.5%)	59 (1.9%)
(Missing)	9 (0.1%)	8 (0.3%)
Relationship with father when growing up, n (%)		
Very good	5,575 (81.7%)	2,550 (81.1%)
Somewhat good	926 (13.6%)	453 (14.4%)
Somewhat bad	75 (1.1%)	38 (1.2%)
Very bad	43 (0.6%)	11 (0.4%)
(Does not apply)	178 (2.6%)	85 (2.7%)
(Missing)	29 (0.4%)	7 (0.2%)
Felt like an outsider in family when growing up, n (%)		
Yes	685 (10.0%)	285 (9.1%)
No	6,046 (88.6%)	2,824 (89.8%)
(Missing)	96 (1.4%)	36 (1.1%)
Experienced abuse when growing up, n (%)		
Yes	892 (13.1%)	392 (12.5%)
No	5,842 (85.6%)	2,730 (86.8%)
(Missing)	93 (1.4%)	24 (0.7%)
Self-rated health when growing up, n (%)		
Excellent	2,668 (39.1%)	1,245 (39.6%)
Very good	2,587 (37.9%)	1,174 (37.3%)
Good	1,152 (16.9%)	561 (17.8%)
Fair	302 (4.4%)	123 (3.9%)
Poor	105 (1.5%)	35 (1.1%)
(Missing)	15 (0.2%)	6 (0.2%)

Table S22a. Weighted summary statistics for demographic and childhood variables in Nigeria

**Characteristic**	**Wave 1** N = 6,827	**Wave 2** N = 3,145
Subjective financial status of family growing up, n (%)		
Lived comfortably	2,207 (32.3%)	993 (31.6%)
Got by	2,392 (35.0%)	1,106 (35.2%)
Found it difficult	1,636 (24.0%)	771 (24.5%)
Found it very difficult	559 (8.2%)	256 (8.1%)
(Missing)	32 (0.5%)	18 (0.6%)
Religious affiliation growing up, n (%)		
Christianity	3,502 (51.3%)	1,617 (51.4%)
Taoism	0 (0%)	0 (0%)
Confucianism	1 (<0.0%)	0 (0%)
Primal, Animist, or Folk religion	19 (0.3%)	9 (0.3%)
Spiritism	0 (0%)	0 (0%)
Umbanda, Candomblé, and other African-derived religions	0 (0%)	0 (0%)
Chinese folk/traditional religion	0 (0%)	0 (0%)
Islam	3,272 (47.9%)	1,503 (47.8%)
Hinduism	0 (0%)	0 (0%)
Buddhism	0 (<0.0%)	0 (0.0%)
Judaism	0 (0%)	0 (0%)
Sikhism	0 (0%)	0 (0%)
Baha’i	0 (0%)	0 (0%)
Jainism	0 (0%)	0 (0%)
Shinto	0 (0%)	0 (0%)
Some other religion	0 (0%)	0 (0%)
No religion/Atheist/Agnostic	18 (0.3%)	8 (0.3%)
(Missing)	15 (0.2%)	7 (0.2%)

Note. N (%); this table is based on non-imputed data. Cumulative percentages for variables may not add up to 100% due to rounding. Wave 1 characteristics weighted using the Gallup provided sampling weight, ANNUAL_WEIGHT_R2; Wave 2 characteristics weighted accounting for attrition by using the adjusted Wave 1 weight, ANNUAL_WEIGHT_R2, multiplied by the created attrition weight to account for dropout, to maintain nationally representative estimates for Wave 2 characteristics.

Table S22b. Weighted summary statistics for outcome variables in Nigeria

Outcome	Wave 1<br>N = 6,827	Wave 2<br>N = 3,145
Secure flourishing index		
Mean	7.4	6.6
Standard Deviation	1.4	1.4
Min, Max	1.3, 10.0	1.1, 10.0
(Missing)	181 (2.6%)	35 (1.1%)
Flourishing index		
Mean	7.8	7.1
Standard Deviation	1.5	1.5
Min, Max	0.6, 10.0	0.4, 10.0
(Missing)	164 (2.4%)	33 (1.1%)
Happiness & life satisfaction		
Mean	6.8	5.9
Standard Deviation	2.4	2.5
Min, Max	0.0, 10.0	0.0, 10.0
(Missing)	27 (0.4%)	3 (0.1%)
Physical & mental health		
Mean	8.5	7.6
Standard Deviation	1.8	2.1
Min, Max	0.0, 10.0	0.0, 10.0
(Missing)	26 (0.4%)	3 (<0.1%)
Meaning & purpose		
Mean	7.9	7.3
Standard Deviation	1.9	1.9
Min, Max	0.0, 10.0	0.0, 10.0
(Missing)	50 (0.7%)	12 (0.4%)
Character & virtue		
Mean	8.3	7.5
Standard Deviation	1.8	2.1
Min, Max	0.0, 10.0	0.0, 10.0
(Missing)	58 (0.8%)	3 (0.1%)
Close social relationships		
Mean	7.7	6.9
Standard Deviation	2.2	2.4
Min, Max	0.0, 10.0	0.0, 10.0
(Missing)	29 (0.4%)	15 (0.5%)
Financial & material security		
Mean	5.1	4.5
Standard Deviation	2.9	2.7
Min, Max	0.0, 10.0	0.0, 10.0
(Missing)	21 (0.3%)	9 (0.3%)
Happiness		
Mean	7.1	6.1
Standard Deviation	2.6	3.0
Min, Max	0.0, 10.0	0.0, 10.0
(Missing)	12 (0.2%)	0 (0%)
Life satisfaction		
Mean	6.5	5.7
Standard Deviation	2.9	3.0
Min, Max	0.0, 10.0	0.0, 10.0
(Missing)	16 (0.2%)	3 (0.1%)
Current life evaluation		

Table S22b. Weighted summary statistics for outcome variables in Nigeria

Outcome	Wave 1<br>N = 6,827	Wave 2<br>N = 3,145
Mean	5.7	4.9
Standard Deviation	2.8	2.8
Min, Max	0.0, 10.0	0.0, 10.0
(Missing)	19 (0.3%)	2 (<0.1%)
Future life evaluation		
Mean	8.3	7.7
Standard Deviation	2.1	2.4
Min, Max	0.0, 10.0	0.0, 10.0
(Missing)	142 (2.1%)	35 (1.1%)
Optimism		
Mean	8.9	8.7
Standard Deviation	1.8	2.1
Min, Max	0.0, 10.0	0.0, 10.0
(Missing)	19 (0.3%)	2 (<0.1%)
Freedom to pursue what's important		
Mean	8.2	7.5
Standard Deviation	2.3	2.5
Min, Max	0.0, 10.0	0.0, 10.0
(Missing)	12 (0.2%)	1 (<0.1%)
Inner peace, n (%)		
Always	2,572 (37.7%)	927 (29.5%)
Often	2,784 (40.8%)	1,333 (42.4%)
Rarely	1,250 (18.3%)	742 (23.6%)
Never	195 (2.9%)	134 (4.3%)
(Missing)	26 (0.4%)	8 (0.3%)
Life balance, n (%)		
Always	2,241 (32.8%)	1,266 (40.3%)
Often	2,632 (38.6%)	1,046 (33.3%)
Rarely	1,515 (22.2%)	623 (19.8%)
Never	408 (6.0%)	198 (6.3%)
(Missing)	31 (0.5%)	11 (0.4%)
Sense of mastery, n (%)		
Always	2,598 (38.1%)	1,227 (39.0%)
Often	2,894 (42.4%)	1,356 (43.1%)
Rarely	1,189 (17.4%)	476 (15.1%)
Never	124 (1.8%)	84 (2.7%)
(Missing)	21 (0.3%)	2 (0.1%)
Meaningful activities		
Mean	7.4	6.7
Standard Deviation	2.5	2.7
Min, Max	0.0, 10.0	0.0, 10.0
(Missing)	24 (0.4%)	9 (0.3%)
Understanding purpose		
Mean	8.4	7.9
Standard Deviation	2.1	2.3
Min, Max	0.0, 10.0	0.0, 10.0
(Missing)	27 (0.4%)	4 (0.1%)
Self-rated mental health		
Mean	8.7	8.0
Standard Deviation	2.1	2.4
Min, Max	0.0, 10.0	0.0, 10.0

Table S22b. Weighted summary statistics for outcome variables in Nigeria

**Outcome**	**Wave 1**<br>N = 6,827	**Wave 2**<br>N = 3,145
(Missing)	18 (0.3%)	1 (<0.1%)
Traumatic distress, n (%)		
A lot	975 (14.3%)	857 (27.3%)
Some	1,705 (25.0%)	706 (22.5%)
Not very much	2,433 (35.6%)	1,049 (33.3%)
Not at all	1,687 (24.7%)	530 (16.9%)
(Missing)	28 (0.4%)	3 (0.1%)
Depression symptoms composite, n (%)	3,047 (45.0%)	1,576 (50.5%)
(Missing)	61 (0.9%)	23 (0.7%)
Depression – feel hopeless, n (%)		
Nearly every day	894 (13.1%)	456 (14.5%)
More than half the days	1,232 (18.0%)	730 (23.2%)
Several days	2,184 (32.0%)	981 (31.2%)
Not at all	2,487 (36.4%)	963 (30.6%)
(Missing)	31 (0.4%)	15 (0.5%)
Depression – loss of interest, n (%)		
Nearly every day	1,389 (20.4%)	680 (21.6%)
More than half the days	1,424 (20.9%)	699 (22.2%)
Several days	2,211 (32.4%)	1,084 (34.5%)
Not at all	1,766 (25.9%)	673 (21.4%)
(Missing)	36 (0.5%)	8 (0.3%)
Anxiety symptoms composite, n (%)	2,498 (37.0%)	1,263 (40.7%)
(Missing)	81 (1.2%)	37 (1.2%)
Anxiety – feel on edge, n (%)		
Nearly every day	780 (11.4%)	353 (11.2%)
More than half the days	1,220 (17.9%)	634 (20.2%)
Several days	2,218 (32.5%)	977 (31.1%)
Not at all	2,557 (37.4%)	1,161 (36.9%)
(Missing)	52 (0.8%)	19 (0.6%)
Anxiety – cannot stop worrying, n (%)		
Nearly every day	877 (12.8%)	513 (16.3%)
More than half the days	1,206 (17.7%)	608 (19.3%)
Several days	2,295 (33.6%)	976 (31.1%)
Not at all	2,416 (35.4%)	1,027 (32.7%)
(Missing)	33 (0.5%)	20 (0.6%)
Suffering, n (%)		
A lot	1,036 (15.2%)	661 (21.0%)
Some	1,769 (25.9%)	733 (23.3%)
Not very much	2,514 (36.8%)	1,235 (39.3%)
Not at all	1,473 (21.6%)	515 (16.4%)
(Missing)	36 (0.5%)	2 (0.0%)
Relationship contentment		
Mean	7.8	7.1
Standard Deviation	2.4	2.7
Min, Max	0.0, 10.0	0.0, 10.0
(Missing)	18 (0.3%)	6 (0.2%)
Relationship satisfaction		
Mean	7.6	6.6
Standard Deviation	2.5	2.9
Min, Max	0.0, 10.0	0.0, 10.0
(Missing)	13 (0.2%)	8 (0.3%)

Table S22b. Weighted summary statistics for outcome variables in Nigeria

Outcome	Wave 1<br>N = 6,827	Wave 2<br>N = 3,145
Social support		
Mean	6.6	5.6
Standard Deviation	2.9	3.2
Min, Max	0.0, 10.0	0.0, 10.0
(Missing)	29 (0.4%)	8 (0.3%)
Intimate/close friend, n (%)		
Yes	6,319 (92.6%)	2,799 (89.0%)
No	502 (7.4%)	337 (10.7%)
(Missing)	6 (0.1%)	8 (0.3%)
Government approval, n (%)		
Strongly approve	1,186 (17.4%)	443 (14.1%)
Somewhat approve	1,495 (21.9%)	741 (23.6%)
Neither approve nor disapprove	900 (13.2%)	339 (10.8%)
Somewhat disapprove	1,424 (20.9%)	592 (18.8%)
Strongly disapprove	1,744 (25.5%)	996 (31.7%)
(Missing)	78 (1.1%)	33 (1.0%)
Say in government, n (%)		
Agree	2,791 (40.9%)	1,485 (47.2%)
Disagree	3,113 (45.6%)	1,240 (39.4%)
Unsure	906 (13.3%)	414 (13.2%)
(Missing)	16 (0.2%)	5 (0.2%)
Belonging in country		
Mean	7.3	6.2
Standard Deviation	2.8	3.0
Min, Max	0.0, 10.0	0.0, 10.0
(Missing)	74 (1.1%)	4 (0.1%)
City/place satisfaction, n (%)		
Satisfied	4,905 (71.9%)	2,290 (72.8%)
Dissatisfied	1,658 (24.3%)	681 (21.7%)
Unsure	258 (3.8%)	168 (5.3%)
(Missing)	6 (0.1%)	5 (0.2%)
Trust within country, n (%)		
All people	163 (2.4%)	28 (0.9%)
Most people	985 (14.4%)	389 (12.4%)
Some people	2,284 (33.5%)	1,174 (37.3%)
Not very many people	2,502 (36.7%)	1,219 (38.8%)
None	761 (11.1%)	300 (9.5%)
(Missing)	132 (1.9%)	34 (1.1%)
Number of children		
Mean	2.8	3.3
Standard Deviation	2.6	2.8
Min, Max	0.0, 19.0	0.0, 20.0
(Missing)	29 (0.4%)	74 (2.4%)
Community participation, n (%)		
More than once a week	1,040 (15.2%)	297 (9.4%)
Once a week	1,194 (17.5%)	590 (18.8%)
One to three times a month	1,246 (18.3%)	580 (18.4%)
A few times a year	1,656 (24.3%)	733 (23.3%)
Never	1,677 (24.6%)	939 (29.9%)
(Missing)	15 (0.2%)	6 (0.2%)
Religious attendance, n (%)		

Table S22b. Weighted summary statistics for outcome variables in Nigeria

Outcome	Wave 1<br>N = 6,827	Wave 2<br>N = 3,145
More than once a week	4,005 (58.7%)	1,934 (61.5%)
Once a week	1,939 (28.4%)	774 (24.6%)
One to three times a month	529 (7.7%)	190 (6.0%)
A few times a year	255 (3.7%)	176 (5.6%)
Never	80 (1.2%)	67 (2.1%)
(Missing)	20 (0.3%)	4 (0.1%)
Loneliness		
Mean	3.2	4.3
Standard Deviation	2.9	3.1
Min, Max	0.0, 10.0	0.0, 10.0
(Missing)	23 (0.3%)	8 (0.2%)
Perceived discrimination, n (%)		
Always	924 (13.5%)	386 (12.3%)
Often	1,375 (20.1%)	721 (22.9%)
Rarely	2,458 (36.0%)	1,211 (38.5%)
Never	2,012 (29.5%)	819 (26.0%)
(Missing)	58 (0.9%)	8 (0.2%)
Orientation to promote good		
Mean	8.2	7.5
Standard Deviation	2.1	2.4
Min, Max	0.0, 10.0	0.0, 10.0
(Missing)	23 (0.3%)	1 (<0.1%)
Delayed gratification		
Mean	8.3	7.5
Standard Deviation	2.1	2.5
Min, Max	0.0, 10.0	0.0, 10.0
(Missing)	40 (0.6%)	2 (<0.1%)
Hope		
Mean	8.7	8.1
Standard Deviation	2.0	2.3
Min, Max	0.0, 10.0	0.0, 10.0
(Missing)	10 (0.2%)	1 (<0.1%)
Gratitude		
Mean	8.4	7.8
Standard Deviation	2.1	2.4
Min, Max	0.0, 10.0	0.0, 10.0
(Missing)	27 (0.4%)	1 (<0.1%)
Showing love/care		
Mean	8.4	7.9
Standard Deviation	2.0	2.3
Min, Max	0.0, 10.0	0.0, 10.0
(Missing)	9 (0.1%)	1 (<0.1%)
Forgivingness, n (%)		
Always	4,276 (62.6%)	1,844 (58.6%)
Often	1,983 (29.0%)	1,013 (32.2%)
Rarely	472 (6.9%)	242 (7.7%)
Never	81 (1.2%)	45 (1.4%)
(Missing)	16 (0.2%)	1 (0.0%)
Charitable giving, n (%)		
Yes	3,205 (47.0%)	1,579 (50.2%)
No	3,607 (52.8%)	1,563 (49.7%)

Table S22b. Weighted summary statistics for outcome variables in Nigeria

**Outcome**	**Wave 1**<br>N = 6,827	**Wave 2**<br>N = 3,145
(Missing)	14 (0.2%)	3 (0.1%)
Helping strangers, n (%)		
Yes	5,608 (82.2%)	2,800 (89.0%)
No	1,201 (17.6%)	338 (10.8%)
(Missing)	18 (0.3%)	7 (0.2%)
Volunteering, n (%)		
Yes	3,410 (50.0%)	1,833 (58.3%)
No	3,402 (49.8%)	1,296 (41.2%)
(Missing)	15 (0.2%)	16 (0.5%)
Self-rated physical health		
Mean	8.3	7.3
Standard Deviation	2.2	2.7
Min, Max	0.0, 10.0	0.0, 10.0
(Missing)	10 (0.2%)	3 (<0.1%)
Health problems, n (%)		
Yes	925 (13.6%)	650 (20.7%)
No	5,887 (86.2%)	2,487 (79.1%)
(Missing)	14 (0.2%)	8 (0.3%)
Pain in past 4 weeks, n (%)		
A lot	828 (12.1%)	613 (19.5%)
Some	1,820 (26.7%)	790 (25.1%)
Not very much	2,663 (39.0%)	1,235 (39.3%)
None at all	1,506 (22.1%)	506 (16.1%)
(Missing)	10 (0.2%)	0 (0.0%)
Number of cigarettes per day		
Mean	0.3	0.2
Standard Deviation	2.5	1.6
Min, Max	0.0, 97.0	0.0, 97.0
(Missing)	64 (0.9%)	11 (0.3%)
Number of drinks per week		
Mean	0.6	0.4
Standard Deviation	3.4	1.9
Min, Max	0.0, 97.0	0.0, 80.0
(Missing)	64 (0.9%)	7 (0.2%)
Days exercise per week		
Mean	3.0	3.8
Standard Deviation	2.6	2.7
Min, Max	0.0, 7.0	0.0, 7.0
(Missing)	132 (1.9%)	35 (1.1%)
Financial security		
Mean	5.0	4.4
Standard Deviation	3.1	3.0
Min, Max	0.0, 10.0	0.0, 10.0
(Missing)	13 (0.2%)	1 (<0.1%)
Material security		
Mean	5.2	4.7
Standard Deviation	3.2	3.1
Min, Max	0.0, 10.0	0.0, 10.0
(Missing)	9 (0.1%)	9 (0.3%)
Educational attainment (16+ years), n (%)		
Up to 8	2,595 (38.0%)	1,138 (36.2%)

Table S22b. Weighted summary statistics for outcome variables in Nigeria

Outcome	Wave 1<br>N = 6,827	Wave 2<br>N = 3,145
9-15	4,102 (60.1%)	1,873 (59.6%)
16+	129 (1.9%)	133 (4.2%)
(Missing)	2 (0.0%)	0 (0%)
Currently employed, n (%)		
Employed for an employer	699 (10.2%)	506 (16.1%)
Self-employed	3,879 (56.8%)	1,828 (58.1%)
Retired	164 (2.4%)	50 (1.6%)
Student	692 (10.1%)	183 (5.8%)
Homemaker	487 (7.1%)	88 (2.8%)
Unemployed and looking for a job	698 (10.2%)	383 (12.2%)
None of these/Other	201 (3.0%)	99 (3.2%)
(Missing)	7 (0.1%)	8 (0.2%)
Financially comfortable/getting by, n (%)		
Living comfortably on present income	551 (8.1%)	445 (14.1%)
Getting by on present income	2,090 (30.6%)	1,140 (36.3%)
Finding it difficult on present income	2,457 (36.0%)	890 (28.3%)
Finding it very difficult on present income	1,641 (24.0%)	647 (20.6%)
(Missing)	87 (1.3%)	23 (0.7%)
Own home, n (%)		
Someone in this household owns this home	2,958 (43.3%)	1,536 (48.8%)
Someone in this household rents this home	3,133 (45.9%)	1,337 (42.5%)
Both	345 (5.0%)	114 (3.6%)
Neither	358 (5.2%)	138 (4.4%)
Rent	0 (0%)	0 (0%)
Own	0 (0%)	0 (0%)
Something else	0 (0%)	0 (0%)
(Missing)	33 (0.5%)	20 (0.6%)
Religious/spiritual connection, n (%)		
Always	4,256 (62.3%)	2,147 (68.3%)
Often	1,805 (26.4%)	614 (19.5%)
Rarely	578 (8.5%)	250 (7.9%)
Never	170 (2.5%)	128 (4.1%)
(Missing)	18 (0.3%)	6 (0.2%)
Belief in life after death, n (%)		
Yes	4,943 (72.4%)	2,355 (74.9%)
No	1,461 (21.4%)	501 (15.9%)
Unsure	413 (6.1%)	281 (8.9%)
(Missing)	10 (0.1%)	7 (0.2%)
Transformative religious experience, n (%)		
Yes	4,013 (58.8%)	2,012 (64.0%)
No	2,715 (39.8%)	1,116 (35.5%)
(Missing)	99 (1.4%)	17 (0.5%)
Religious reading or listening, n (%)		
More than once a day	2,286 (33.5%)	1,179 (37.5%)
About once a day	1,976 (28.9%)	769 (24.4%)
Sometimes	2,204 (32.3%)	1,011 (32.1%)
Never	335 (4.9%)	182 (5.8%)
(Missing)	25 (0.4%)	4 (0.1%)
Prayer or meditation, n (%)		
More than once a day	4,932 (72.2%)	2,352 (74.8%)
About once a day	1,307 (19.1%)	509 (16.2%)

Table S22b. Weighted summary statistics for outcome variables in Nigeria

Outcome	Wave 1<br>N = 6,827	Wave 2<br>N = 3,145
Sometimes	530 (7.8%)	256 (8.1%)
Never	44 (0.6%)	23 (0.7%)
(Missing)	15 (0.2%)	5 (0.2%)
Belief in God/gods/spiritual forces, n (%)		
One God	6,656 (97.5%)	3,109 (98.9%)
More than one god	101 (1.5%)	10 (0.3%)
An impersonal spiritual force	27 (0.4%)	8 (0.3%)
None of these	20 (0.3%)	5 (0.2%)
Unsure	13 (0.2%)	9 (0.3%)
(Missing)	10 (0.1%)	3 (0.1%)
Religious centrality, n (%)		
Agree	6,074 (89.0%)	2,789 (88.7%)
Disagree	484 (7.1%)	168 (5.3%)
Not relevant	119 (1.7%)	76 (2.4%)
Unsure	146 (2.1%)	106 (3.4%)
(Missing)	5 (0.1%)	5 (0.2%)
Religious/spiritual comfort, n (%)		
Agree	6,386 (93.5%)	2,913 (92.6%)
Disagree	303 (4.4%)	117 (3.7%)
Not relevant	64 (0.9%)	52 (1.7%)
Unsure	69 (1.0%)	60 (1.9%)
(Missing)	5 (0.1%)	3 (0.1%)
Feel loved by God, n (%)		
Agree	6,452 (94.5%)	3,014 (95.9%)
Disagree	214 (3.1%)	54 (1.7%)
Not relevant	83 (1.2%)	33 (1.0%)
Unsure	67 (1.0%)	42 (1.3%)
(Missing)	10 (0.1%)	1 (0.0%)
Feel punished by God, n (%)		
Agree	1,768 (25.9%)	718 (22.8%)
Disagree	3,970 (58.2%)	1,986 (63.1%)
Not relevant	549 (8.0%)	222 (7.1%)
Unsure	510 (7.5%)	217 (6.9%)
(Missing)	30 (0.4%)	2 (0.1%)
Experienced religious criticism, n (%)		
Agree	3,371 (49.4%)	1,464 (46.5%)
Disagree	2,319 (34.0%)	1,058 (33.7%)
Not relevant	564 (8.3%)	300 (9.5%)
Unsure	553 (8.1%)	319 (10.1%)
(Missing)	20 (0.3%)	4 (0.1%)
Faith-sharing, n (%)		
Agree	5,233 (76.7%)	2,484 (79.0%)
Disagree	1,098 (16.1%)	374 (11.9%)
Not relevant	342 (5.0%)	171 (5.4%)
Unsure	148 (2.2%)	99 (3.1%)
(Missing)	6 (0.1%)	16 (0.5%)

Note. N (%); this table is based on non-imputed data. Cumulative percentages for variables may not add up to 100% due to rounding. Wave 1 characteristics weighted using the Gallup provided sampling weight, ANNUAL_WEIGHT_R2; Wave 2 characteristics weighted accounting for attrition by using the adjusted Wave 1 weight, ANNUAL_WEIGHT_R2, multiplied by the created attrition weight to account for dropout, to maintain nationally representative estimates for Wave 2 characteristics.

Table S22c. Unweighted summary statistics for demographic and childhood variables in Nigeria by retention status

Characteristic	Attritors–Not Observed in Wave 2<br>N = 3,816	Retained–Observed in Wave 2<br>N = 2,968
Forgivingness, n (%)		
Always	2,409 (63.1%)	1,839 (62.0%)
Often	1,106 (29.0%)	865 (29.1%)
Rarely	240 (6.3%)	229 (7.7%)
Never	51 (1.3%)	29 (1.0%)
(Missing)	10 (0.3%)	5 (0.2%)
Year of birth, n (%)		
1943 or earlier (current age: 80+ years)	4 (0.1%)	37 (1.2%)
1943-1953 (current age: 70-79 years)	40 (1.0%)	74 (2.5%)
1953-1963 (current age: 60-69 years)	131 (3.4%)	143 (4.8%)
1963-1973 (current age: 50-59 years)	288 (7.5%)	275 (9.3%)
1973-1983 (current age: 40-49 years)	525 (13.8%)	463 (15.6%)
1983-1993 (current age: 30-39 years)	1,080 (28.3%)	839 (28.3%)
1993-1998 (current age: 25-29 years)	719 (18.8%)	484 (16.3%)
1998-2005 (current age: 18-24 years)	1,030 (27.0%)	654 (22.0%)
(Missing)	0 (0%)	0 (0%)
Age of participant		
Mean	33.1	36.3
Standard Deviation	12.3	14.5
Min, Max	18.0, 82.0	18.0, 93.0
Gender, n (%)		
Male	1,866 (48.9%)	1,531 (51.6%)
Female	1,950 (51.1%)	1,438 (48.4%)
Other	0 (0.0%)	0 (0%)
(Missing)	0 (0%)	0 (0%)
Respondent marital status, n (%)		
Single/Never been married	1,397 (36.6%)	1,009 (34.0%)
Married	2,222 (58.2%)	1,728 (58.2%)
Separated	50 (1.3%)	60 (2.0%)
Divorced	34 (0.9%)	32 (1.1%)
Widowed	77 (2.0%)	122 (4.1%)
Domestic partner	10 (0.3%)	3 (0.1%)
(Missing)	26 (0.7%)	15 (0.5%)
Education (years), n (%)		
Up to 8	1,486 (38.9%)	1,091 (36.8%)
9-15	2,265 (59.3%)	1,813 (61.1%)
16+	65 (1.7%)	63 (2.1%)
(Missing)	0 (0.0%)	1 (0.0%)
Employment status, n (%)		
Employed for an employer	384 (10.1%)	310 (10.5%)
Self-employed	2,197 (57.6%)	1,656 (55.8%)
Retired	73 (1.9%)	90 (3.0%)
Student	403 (10.6%)	284 (9.6%)
Homemaker	254 (6.7%)	231 (7.8%)
Unemployed and looking for a job	408 (10.7%)	285 (9.6%)
None of these/Other	94 (2.5%)	107 (3.6%)
(Missing)	3 (0.1%)	4 (0.1%)
Current religious service attendance, n (%)		
More than once a week	2,117 (55.5%)	1,868 (62.9%)

Table S22c. Unweighted summary statistics for demographic and childhood variables in Nigeria by retention status

Characteristic	Attritors–Not Observed in Wave 2<br>N = 3,816	Retained–Observed in Wave 2<br>N = 2,968
Once a week	1,148 (30.1%)	777 (26.2%)
One to three times a month	324 (8.5%)	200 (6.8%)
A few times a year	155 (4.1%)	97 (3.3%)
Never	61 (1.6%)	18 (0.6%)
(Missing)	11 (0.3%)	8 (0.3%)
Immigration status, n (%)		
Born in this country	3,790 (99.3%)	2,947 (99.3%)
Born in another country	25 (0.7%)	21 (0.7%)
(Missing)	1 (0.0%)	0 (0%)
Parental marital status around age 12, n (%)		
Parents were married	3,104 (81.3%)	2,419 (81.5%)
Parents were divorced	178 (4.7%)	129 (4.3%)
Parents were never married	200 (5.2%)	149 (5.0%)
One or both of them had died	255 (6.7%)	197 (6.6%)
Unsure	24 (0.6%)	34 (1.2%)
(Missing)	55 (1.4%)	39 (1.3%)
Religious service attendance around age 12, n (%)		
At least once a week	3,289 (86.2%)	2,577 (86.8%)
One to three times a month	355 (9.3%)	253 (8.5%)
Less than once a month	74 (1.9%)	59 (2.0%)
Never	75 (2.0%)	60 (2.0%)
(Missing)	24 (0.6%)	19 (0.6%)
Relationship with mother when growing up, n (%)		
Very good	3,376 (88.4%)	2,575 (86.7%)
Somewhat good	359 (9.4%)	285 (9.6%)
Somewhat bad	23 (0.6%)	38 (1.3%)
Very bad	9 (0.2%)	10 (0.4%)
(Does not apply)	48 (1.2%)	53 (1.8%)
(Missing)	2 (0.0%)	8 (0.3%)
Relationship with father when growing up, n (%)		
Very good	3,135 (82.2%)	2,404 (81.0%)
Somewhat good	493 (12.9%)	429 (14.5%)
Somewhat bad	41 (1.1%)	34 (1.1%)
Very bad	31 (0.8%)	12 (0.4%)
(Does not apply)	96 (2.5%)	81 (2.7%)
(Missing)	20 (0.5%)	9 (0.3%)
Felt like an outsider in family when growing up, n (%)		
Yes	411 (10.8%)	269 (9.1%)
No	3,344 (87.6%)	2,666 (89.8%)
(Missing)	61 (1.6%)	34 (1.1%)
Experienced abuse when growing up, n (%)		
Yes	525 (13.8%)	360 (12.1%)
No	3,221 (84.4%)	2,586 (87.1%)
(Missing)	70 (1.8%)	22 (0.7%)
Self-rated health when growing up, n (%)		
Excellent	1,489 (39.0%)	1,162 (39.2%)
Very good	1,447 (37.9%)	1,123 (37.8%)

Table S22c. Unweighted summary statistics for demographic and childhood variables in Nigeria by retention status

**Characteristic**	**Attritors–Not Observed in Wave 2**<br>N = 3,816	**Retained–Observed in Wave 2**<br>N = 2,968
Good	625 (16.4%)	520 (17.5%)
Fair	179 (4.7%)	120 (4.1%)
Poor	66 (1.7%)	38 (1.3%)
(Missing)	10 (0.3%)	5 (0.2%)
Subjective financial status of family growing up, n (%)		
Lived comfortably	1,254 (32.9%)	939 (31.6%)
Got by	1,347 (35.3%)	1,030 (34.7%)
Found it difficult	891 (23.3%)	736 (24.8%)
Found it very difficult	309 (8.1%)	247 (8.3%)
(Missing)	15 (0.4%)	17 (0.6%)
Religious affiliation growing up, n (%)		
Christianity	1,967 (51.5%)	1,513 (51.0%)
Taoism	0 (0%)	0 (0%)
Confucianism	1 (0.0%)	0 (0%)
Primal, Animist, or Folk religion	11 (0.3%)	8 (0.3%)
Spiritism	0 (0%)	0 (0%)
Umbanda, Candomblé, and other African-derived religions	0 (0%)	0 (0%)
Chinese folk/traditional religion	0 (0%)	0 (0%)
Islam	1,820 (47.7%)	1,432 (48.2%)
Hinduism	0 (0%)	0 (0%)
Buddhism	0 (0%)	0 (0.0%)
Judaism	0 (0%)	0 (0%)
Sikhism	0 (0%)	0 (0%)
Baha’i	0 (0%)	0 (0%)
Jainism	0 (0%)	0 (0%)
Shinto	0 (0%)	0 (0%)
Some other religion	0 (0%)	0 (0%)
No religion/Atheist/Agnostic	8 (0.2%)	9 (0.3%)
(Missing)	10 (0.3%)	6 (0.2%)

Note. N (%); this table is based on non-imputed data. Cumulative percentages for variables may not add up to 100% due to rounding.

Table S22d. Unweighted summary statistics for Wave 1 outcome variables in Nigeria by retention status.

Outcome	Attritors–Not Observed in Wave 2 N = 3,816	Retained–Observed in Wave 2 N = 2,968
Secure flourishing index		
Mean	7.4	7.3
Standard Deviation	1.4	1.4
Min, Max	1.7, 10.0	1.3, 10.0
(Missing)	104 (2.7%)	76 (2.6%)
Flourishing index		
Mean	7.9	7.8
Standard Deviation	1.4	1.5
Min, Max	1.6, 10.0	0.6, 10.0
(Missing)	97 (2.5%)	66 (2.2%)
Happiness & life satisfaction		
Mean	6.9	6.7
Standard Deviation	2.3	2.4
Min, Max	0.0, 10.0	0.0, 10.0
(Missing)	16 (0.4%)	11 (0.4%)
Physical & mental health		
Mean	8.6	8.4
Standard Deviation	1.8	1.8
Min, Max	0.0, 10.0	0.0, 10.0
(Missing)	14 (0.4%)	12 (0.4%)
Meaning & purpose		
Mean	8.0	7.8
Standard Deviation	1.9	1.9
Min, Max	0.0, 10.0	0.0, 10.0
(Missing)	28 (0.7%)	22 (0.7%)
Character & virtue		
Mean	8.3	8.3
Standard Deviation	1.8	1.8
Min, Max	0.0, 10.0	0.0, 10.0
(Missing)	40 (1.0%)	17 (0.6%)
Close social relationships		
Mean	7.7	7.7
Standard Deviation	2.2	2.2
Min, Max	0.0, 10.0	0.0, 10.0
(Missing)	14 (0.4%)	15 (0.5%)
Financial & material security		
Mean	5.3	4.9
Standard Deviation	2.8	2.9
Min, Max	0.0, 10.0	0.0, 10.0
(Missing)	11 (0.3%)	10 (0.3%)
Happiness		
Mean	7.2	7.0
Standard Deviation	2.6	2.6
Min, Max	0.0, 10.0	0.0, 10.0
(Missing)	8 (0.2%)	4 (0.1%)
Life satisfaction		
Mean	6.6	6.4
Standard Deviation	2.9	2.9
Min, Max	0.0, 10.0	0.0, 10.0

Table S22d. Unweighted summary statistics for Wave 1 outcome variables in Nigeria by retention status.

Outcome	Attritors–Not Observed in Wave 2<br>N = 3,816	Retained–Observed in Wave 2<br>N = 2,968
(Missing)	8 (0.2%)	8 (0.3%)
Current life evaluation		
Mean	5.7	5.8
Standard Deviation	2.8	2.8
Min, Max	0.0, 10.0	0.0, 10.0
(Missing)	10 (0.3%)	9 (0.3%)
Future life evaluation		
Mean	8.3	8.3
Standard Deviation	2.1	2.1
Min, Max	0.0, 10.0	0.0, 10.0
(Missing)	87 (2.3%)	53 (1.8%)
Optimism		
Mean	8.9	9.0
Standard Deviation	1.8	1.7
Min, Max	0.0, 10.0	0.0, 10.0
(Missing)	7 (0.2%)	12 (0.4%)
Freedom to pursue what's important		
Mean	8.2	8.1
Standard Deviation	2.3	2.3
Min, Max	0.0, 10.0	0.0, 10.0
(Missing)	8 (0.2%)	3 (<0.1%)
Inner peace, n (%)		
Always	1,447 (37.9%)	1,109 (37.3%)
Often	1,530 (40.1%)	1,238 (41.7%)
Rarely	706 (18.5%)	537 (18.1%)
Never	123 (3.2%)	70 (2.4%)
(Missing)	11 (0.3%)	16 (0.5%)
Life balance, n (%)		
Always	1,279 (33.5%)	947 (31.9%)
Often	1,463 (38.3%)	1,152 (38.8%)
Rarely	841 (22.0%)	665 (22.4%)
Never	211 (5.5%)	195 (6.6%)
(Missing)	23 (0.6%)	8 (0.3%)
Sense of mastery, n (%)		
Always	1,486 (38.9%)	1,095 (36.9%)
Often	1,573 (41.2%)	1,305 (44.0%)
Rarely	678 (17.8%)	503 (17.0%)
Never	69 (1.8%)	54 (1.8%)
(Missing)	10 (0.3%)	11 (0.4%)
Meaningful activities		
Mean	7.5	7.3
Standard Deviation	2.5	2.6
Min, Max	0.0, 10.0	0.0, 10.0
(Missing)	10 (0.3%)	14 (0.5%)
Understanding purpose		
Mean	8.4	8.3
Standard Deviation	2.1	2.1
Min, Max	0.0, 10.0	0.0, 10.0
(Missing)	19 (0.5%)	8 (0.3%)

Table S22d. Unweighted summary statistics for Wave 1 outcome variables in Nigeria by retention status.

**Outcome**	**Attritors–Not Observed in Wave 2** N = 3,816	**Retained–Observed in Wave 2** N = 2,968
Self-rated mental health		
Mean	8.7	8.6
Standard Deviation	2.1	2.0
Min, Max	0.0, 10.0	0.0, 10.0
(Missing)	11 (0.3%)	6 (0.2%)
Traumatic distress, n (%)		
A lot	526 (13.8%)	444 (15.0%)
Some	941 (24.6%)	754 (25.4%)
Not very much	1,399 (36.7%)	1,017 (34.3%)
Not at all	937 (24.5%)	740 (24.9%)
(Missing)	14 (0.4%)	14 (0.5%)
Depression symptoms composite, n (%)	1,754 (46.4%)	1,272 (43.3%)
(Missing)	32 (0.8%)	28 (1.0%)
Depression – feel hopeless, n (%)		
Nearly every day	518 (13.6%)	369 (12.4%)
More than half the days	709 (18.6%)	514 (17.3%)
Several days	1,227 (32.2%)	943 (31.8%)
Not at all	1,347 (35.3%)	1,126 (37.9%)
(Missing)	15 (0.4%)	15 (0.5%)
Depression – loss of interest, n (%)		
Nearly every day	808 (21.2%)	572 (19.3%)
More than half the days	813 (21.3%)	602 (20.3%)
Several days	1,239 (32.5%)	958 (32.3%)
Not at all	935 (24.5%)	823 (27.7%)
(Missing)	22 (0.6%)	14 (0.5%)
Anxiety symptoms composite, n (%)	1,427 (37.8%)	1,054 (36.0%)
(Missing)	42 (1.1%)	39 (1.3%)
Anxiety – feel on edge, n (%)		
Nearly every day	435 (11.4%)	340 (11.4%)
More than half the days	693 (18.2%)	520 (17.5%)
Several days	1,237 (32.4%)	968 (32.6%)
Not at all	1,422 (37.3%)	1,119 (37.7%)
(Missing)	29 (0.8%)	23 (0.8%)
Anxiety – cannot stop worrying, n (%)		
Nearly every day	493 (12.9%)	379 (12.8%)
More than half the days	694 (18.2%)	504 (17.0%)
Several days	1,316 (34.5%)	964 (32.5%)
Not at all	1,299 (34.0%)	1,104 (37.2%)
(Missing)	16 (0.4%)	17 (0.6%)
Suffering, n (%)		
A lot	547 (14.3%)	484 (16.3%)
Some	1,050 (27.5%)	706 (23.8%)
Not very much	1,370 (35.9%)	1,130 (38.1%)
Not at all	828 (21.7%)	635 (21.4%)
(Missing)	22 (0.6%)	14 (0.5%)
Relationship contentment		
Mean	7.8	7.8
Standard Deviation	2.4	2.4
Min, Max	0.0, 10.0	0.0, 10.0

Table S22d. Unweighted summary statistics for Wave 1 outcome variables in Nigeria by retention status.

Outcome	Attritors–Not Observed in Wave 2<br>N = 3,816	Retained–Observed in Wave 2<br>N = 2,968
(Missing)	7 (0.2%)	11 (0.4%)
Relationship satisfaction		
Mean	7.6	7.6
Standard Deviation	2.5	2.4
Min, Max	0.0, 10.0	0.0, 10.0
(Missing)	7 (0.2%)	6 (0.2%)
Social support		
Mean	6.7	6.5
Standard Deviation	2.9	2.9
Min, Max	0.0, 10.0	0.0, 10.0
(Missing)	16 (0.4%)	13 (0.4%)
Intimate/close friend, n (%)		
Yes	3,524 (92.3%)	2,756 (92.9%)
No	288 (7.5%)	211 (7.1%)
(Missing)	5 (0.1%)	1 (0.0%)
Government approval, n (%)		
Strongly approve	675 (17.7%)	503 (16.9%)
Somewhat approve	845 (22.1%)	640 (21.6%)
Neither approve nor disapprove	507 (13.3%)	388 (13.1%)
Somewhat disapprove	775 (20.3%)	640 (21.6%)
Strongly disapprove	962 (25.2%)	772 (26.0%)
(Missing)	52 (1.4%)	26 (0.9%)
Say in government, n (%)		
Agree	1,575 (41.3%)	1,199 (40.4%)
Disagree	1,722 (45.1%)	1,373 (46.3%)
Unsure	505 (13.2%)	395 (13.3%)
(Missing)	14 (0.4%)	2 (0.1%)
Belonging in country		
Mean	7.4	7.1
Standard Deviation	2.8	2.8
Min, Max	0.0, 10.0	0.0, 10.0
(Missing)	39 (1.0%)	35 (1.2%)
City/place satisfaction, n (%)		
Satisfied	2,729 (71.5%)	2,147 (72.3%)
Dissatisfied	945 (24.8%)	702 (23.6%)
Unsure	138 (3.6%)	119 (4.0%)
(Missing)	5 (0.1%)	1 (0.0%)
Trust within country, n (%)		
All people	86 (2.3%)	75 (2.5%)
Most people	563 (14.8%)	415 (14.0%)
Some people	1,310 (34.3%)	959 (32.3%)
Not very many people	1,351 (35.4%)	1,137 (38.3%)
None	434 (11.4%)	322 (10.8%)
(Missing)	71 (1.9%)	60 (2.0%)
Number of children		
Mean	2.8	2.8
Standard Deviation	2.7	2.5
Min, Max	0.0, 19.0	0.0, 18.0
(Missing)	20 (0.5%)	9 (0.3%)

Table S22d. Unweighted summary statistics for Wave 1 outcome variables in Nigeria by retention status.

**Outcome**	**Attritors–Not Observed in Wave 2** N = 3,816	**Retained–Observed in Wave 2** N = 2,968
Community participation, n (%)		
More than once a week	576 (15.1%)	458 (15.4%)
Once a week	656 (17.2%)	530 (17.9%)
One to three times a month	672 (17.6%)	567 (19.1%)
A few times a year	904 (23.7%)	742 (25.0%)
Never	1,002 (26.3%)	662 (22.3%)
(Missing)	7 (0.2%)	8 (0.3%)
Religious attendance, n (%)		
More than once a week	2,117 (55.5%)	1,868 (62.9%)
Once a week	1,148 (30.1%)	777 (26.2%)
One to three times a month	324 (8.5%)	200 (6.8%)
A few times a year	155 (4.1%)	97 (3.3%)
Never	61 (1.6%)	18 (0.6%)
(Missing)	11 (0.3%)	8 (0.3%)
Loneliness		
Mean	3.1	3.4
Standard Deviation	2.9	2.9
Min, Max	0.0, 10.0	0.0, 10.0
(Missing)	11 (0.3%)	12 (0.4%)
Perceived discrimination, n (%)		
Always	537 (14.1%)	380 (12.8%)
Often	758 (19.9%)	609 (20.5%)
Rarely	1,387 (36.3%)	1,055 (35.5%)
Never	1,104 (28.9%)	897 (30.2%)
(Missing)	31 (0.8%)	27 (0.9%)
Orientation to promote good		
Mean	8.2	8.3
Standard Deviation	2.2	2.0
Min, Max	0.0, 10.0	0.0, 10.0
(Missing)	11 (0.3%)	13 (0.4%)
Delayed gratification		
Mean	8.4	8.3
Standard Deviation	2.0	2.1
Min, Max	0.0, 10.0	0.0, 10.0
(Missing)	31 (0.8%)	8 (0.3%)
Hope		
Mean	8.6	8.7
Standard Deviation	2.0	1.9
Min, Max	0.0, 10.0	0.0, 10.0
(Missing)	4 (<0.1%)	7 (0.2%)
Gratitude		
Mean	8.4	8.4
Standard Deviation	2.1	2.1
Min, Max	0.0, 10.0	0.0, 10.0
(Missing)	11 (0.3%)	16 (0.5%)
Showing love/care		
Mean	8.4	8.4
Standard Deviation	2.0	2.0
Min, Max	0.0, 10.0	0.0, 10.0

Table S22d. Unweighted summary statistics for Wave 1 outcome variables in Nigeria by retention status.

**Outcome**	**Attritors–Not Observed in Wave 2** N = 3,816	**Retained–Observed in Wave 2** N = 2,968
(Missing)	3 (<0.1%)	6 (0.2%)
Forgivingness, n (%)		
Always	2,409 (63.1%)	1,839 (62.0%)
Often	1,106 (29.0%)	865 (29.1%)
Rarely	240 (6.3%)	229 (7.7%)
Never	51 (1.3%)	29 (1.0%)
(Missing)	10 (0.3%)	5 (0.2%)
Charitable giving, n (%)		
Yes	1,792 (46.9%)	1,394 (47.0%)
No	2,012 (52.7%)	1,573 (53.0%)
(Missing)	12 (0.3%)	2 (0.1%)
Helping strangers, n (%)		
Yes	3,116 (81.6%)	2,459 (82.8%)
No	689 (18.1%)	504 (17.0%)
(Missing)	11 (0.3%)	6 (0.2%)
Volunteering, n (%)		
Yes	1,892 (49.6%)	1,498 (50.5%)
No	1,912 (50.1%)	1,468 (49.5%)
(Missing)	12 (0.3%)	2 (0.1%)
Self-rated physical health		
Mean	8.4	8.1
Standard Deviation	2.1	2.3
Min, Max	0.0, 10.0	0.0, 10.0
(Missing)	3 (<0.1%)	7 (0.3%)
Health problems, n (%)		
Yes	482 (12.6%)	439 (14.8%)
No	3,324 (87.1%)	2,525 (85.1%)
(Missing)	10 (0.3%)	4 (0.1%)
Pain in past 4 weeks, n (%)		
A lot	446 (11.7%)	377 (12.7%)
Some	1,042 (27.3%)	767 (25.8%)
Not very much	1,491 (39.1%)	1,155 (38.9%)
None at all	830 (21.8%)	667 (22.5%)
(Missing)	7 (0.2%)	3 (0.1%)
Number of cigarettes per day		
Mean	0.2	0.3
Standard Deviation	2.1	2.9
Min, Max	0.0, 97.0	0.0, 97.0
(Missing)	31 (0.8%)	32 (1.1%)
Number of drinks per week		
Mean	0.6	0.5
Standard Deviation	3.8	3.0
Min, Max	0.0, 97.0	0.0, 97.0
(Missing)	33 (0.9%)	31 (1.1%)
Days exercise per week		
Mean	3.0	3.1
Standard Deviation	2.6	2.6
Min, Max	0.0, 7.0	0.0, 7.0
(Missing)	66 (1.7%)	65 (2.2%)

Table S22d. Unweighted summary statistics for Wave 1 outcome variables in Nigeria by retention status.

**Outcome**	**Attritors–Not Observed in Wave 2** N = 3,816	**Retained–Observed in Wave 2** N = 2,968
Financial security		
Mean	5.2	4.8
Standard Deviation	3.1	3.1
Min, Max	0.0, 10.0	0.0, 10.0
(Missing)	9 (0.2%)	3 (0.1%)
Material security		
Mean	5.4	5.1
Standard Deviation	3.2	3.2
Min, Max	0.0, 10.0	0.0, 10.0
(Missing)	2 (<0.1%)	7 (0.2%)
Educational attainment (16+ years), n (%)		
Up to 8	1,486 (38.9%)	1,091 (36.8%)
9-15	2,265 (59.3%)	1,813 (61.1%)
16+	65 (1.7%)	63 (2.1%)
(Missing)	0 (0.0%)	1 (0.0%)
Currently employed, n (%)		
Employed for an employer	384 (10.1%)	310 (10.5%)
Self-employed	2,197 (57.6%)	1,656 (55.8%)
Retired	73 (1.9%)	90 (3.0%)
Student	403 (10.6%)	284 (9.6%)
Homemaker	254 (6.7%)	231 (7.8%)
Unemployed and looking for a job	408 (10.7%)	285 (9.6%)
None of these/Other	94 (2.5%)	107 (3.6%)
(Missing)	3 (0.1%)	4 (0.1%)
Financially comfortable/getting by, n (%)		
Living comfortably on present income	297 (7.8%)	252 (8.5%)
Getting by on present income	1,110 (29.1%)	970 (32.7%)
Finding it difficult on present income	1,456 (38.2%)	983 (33.1%)
Finding it very difficult on present income	889 (23.3%)	743 (25.0%)
(Missing)	64 (1.7%)	21 (0.7%)
Own home, n (%)		
Someone in this household owns this home	1,597 (41.9%)	1,345 (45.3%)
Someone in this household rents this home	1,790 (46.9%)	1,322 (44.5%)
Both	197 (5.2%)	146 (4.9%)
Neither	213 (5.6%)	142 (4.8%)
Rent	0 (0%)	0 (0%)
Own	0 (0%)	0 (0%)
Something else	0 (0%)	0 (0%)
(Missing)	20 (0.5%)	13 (0.4%)
Religious/spiritual connection, n (%)		
Always	2,368 (62.0%)	1,862 (62.7%)
Often	1,006 (26.4%)	787 (26.5%)
Rarely	354 (9.3%)	220 (7.4%)
Never	77 (2.0%)	93 (3.1%)
(Missing)	11 (0.3%)	6 (0.2%)
Belief in life after death, n (%)		
Yes	2,734 (71.6%)	2,179 (73.4%)
No	835 (21.9%)	616 (20.8%)
Unsure	244 (6.4%)	167 (5.6%)

Table S22d. Unweighted summary statistics for Wave 1 outcome variables in Nigeria by retention status.

Outcome	Attritors–Not Observed in Wave 2<br>N = 3,816	Retained–Observed in Wave 2<br>N = 2,968
(Missing)	4 (0.1%)	6 (0.2%)
Transformative religious experience, n (%)		
Yes	2,221 (58.2%)	1,769 (59.6%)
No	1,538 (40.3%)	1,159 (39.0%)
(Missing)	58 (1.5%)	41 (1.4%)
Religious reading or listening, n (%)		
More than once a day	1,230 (32.2%)	1,043 (35.1%)
About once a day	1,151 (30.2%)	811 (27.3%)
Sometimes	1,217 (31.9%)	974 (32.8%)
Never	203 (5.3%)	129 (4.4%)
(Missing)	15 (0.4%)	10 (0.4%)
Prayer or meditation, n (%)		
More than once a day	2,703 (70.8%)	2,200 (74.1%)
About once a day	767 (20.1%)	530 (17.9%)
Sometimes	309 (8.1%)	217 (7.3%)
Never	26 (0.7%)	17 (0.6%)
(Missing)	11 (0.3%)	4 (0.1%)
Belief in God/gods/spiritual forces, n (%)		
One God	3,725 (97.6%)	2,889 (97.3%)
More than one god	57 (1.5%)	43 (1.5%)
An impersonal spiritual force	13 (0.3%)	14 (0.5%)
None of these	5 (0.1%)	15 (0.5%)
Unsure	8 (0.2%)	6 (0.2%)
(Missing)	8 (0.2%)	2 (0.1%)
Religious centrality, n (%)		
Agree	3,385 (88.7%)	2,652 (89.3%)
Disagree	293 (7.7%)	187 (6.3%)
Not relevant	52 (1.4%)	67 (2.3%)
Unsure	85 (2.2%)	59 (2.0%)
(Missing)	2 (0.1%)	3 (0.1%)
Religious/spiritual comfort, n (%)		
Agree	3,557 (93.2%)	2,791 (94.0%)
Disagree	180 (4.7%)	120 (4.1%)
Not relevant	36 (1.0%)	27 (0.9%)
Unsure	39 (1.0%)	29 (1.0%)
(Missing)	4 (0.1%)	1 (0.0%)
Feel loved by God, n (%)		
Agree	3,605 (94.5%)	2,807 (94.6%)
Disagree	130 (3.4%)	82 (2.8%)
Not relevant	43 (1.1%)	39 (1.3%)
Unsure	31 (0.8%)	36 (1.2%)
(Missing)	6 (0.2%)	4 (0.1%)
Feel punished by God, n (%)		
Agree	1,027 (26.9%)	728 (24.5%)
Disagree	2,172 (56.9%)	1,775 (59.8%)
Not relevant	320 (8.4%)	225 (7.6%)
Unsure	278 (7.3%)	229 (7.7%)
(Missing)	19 (0.5%)	11 (0.4%)
Experienced religious criticism, n (%)		

Table S22d. Unweighted summary statistics for Wave 1 outcome variables in Nigeria by retention status.

Outcome	**Attritors–Not Observed in Wave 2** N = 3,816	**Retained–Observed in Wave 2** N = 2,968
Agree	1,896 (49.7%)	1,454 (49.0%)
Disagree	1,299 (34.0%)	1,006 (33.9%)
Not relevant	318 (8.3%)	242 (8.2%)
Unsure	293 (7.7%)	258 (8.7%)
(Missing)	10 (0.3%)	9 (0.3%)
Faith-sharing, n (%)		
Agree	2,947 (77.2%)	2,253 (75.9%)
Disagree	621 (16.3%)	470 (15.8%)
Not relevant	170 (4.5%)	170 (5.7%)
Unsure	74 (1.9%)	74 (2.5%)
(Missing)	4 (0.1%)	2 (0.1%)

Note. N (%); this table is based on non-imputed data. Cumulative percentages for variables may not add up to 100% due to rounding.

Table S22e. Summary of fitted attrition model in Nigeria

**Characteristic**	**Odds Ratio**	**95% CI**	**p-value**
**ANNUAL_WEIGHT_R2**	0.68	0.59, 0.77	5.65e-08
**Happiness & life satisfaction**	0.95	0.87, 1.04	0.266
**Physical & mental health**	0.94	0.87, 1.03	0.175
**Meaning & purpose**	0.93	0.84, 1.02	0.131
**Character & virtue**	1.09	1.00, 1.18	0.043
**Close social relationships**	1.06	0.98, 1.15	0.145
**Financial & material security**	0.92	0.85, 0.99	0.024
**Extraversion**	1.00	0.93, 1.08	0.973
**Openness to experience**	0.96	0.88, 1.06	0.424
**Agreeableness**	0.98	0.90, 1.08	0.706
**Conscientiousness**	1.03	0.95, 1.12	0.451
**Neuroticism**	0.95	0.88, 1.03	0.188
**Depression symptoms composite**	0.94	0.87, 1.02	0.157
**Anxiety symptoms composite**	0.97	0.89, 1.05	0.458
**Loneliness**	1.08	1.00, 1.17	0.065
**Days exercise per week**	1.04	0.97, 1.12	0.272
**Year of birth (age group)**			
1983-1993 (current age: 30-39 years)	—	—	
1998-2005 (current age: 18-24 years)	0.80	0.66, 0.98	0.027
1993-1998 (current age: 25-29 years)	0.82	0.68, 0.97	0.024
1973-1983 (current age: 40-49 years)	1.36	1.10, 1.68	0.005
1963-1973 (current age: 50-59 years)	1.84	1.30, 2.61	7.20e-04
1953-1963 (current age: 60-69 years)	2.57	1.41, 4.66	0.002
1943-1953 (current age: 70-79 years)	4.26	1.60, 11.4	0.004
1943 or earlier (current age: 80+ years)	21.8	3.00, 159	0.003
**Gender of respondent**			
Male	—	—	
Female	0.97	0.83, 1.14	0.719
Other	0.00	0.00, 0.00	1.27e-18
**Marital status**			
Married	—	—	
Single/Never been married	1.08	0.93, 1.26	0.312
Separated	1.41	0.82, 2.41	0.212
Widowed	1.06	0.63, 1.81	0.820
Divorced	0.90	0.43, 1.87	0.776
Domestic partner	0.50	0.12, 2.02	0.328
**Employment status**			
Self-employed	—	—	
Employed for an employer	1.05	0.84, 1.30	0.683
Student	1.09	0.88, 1.36	0.420
Unemployed and looking for a job	0.98	0.76, 1.27	0.897
Homemaker	1.43	1.05, 1.95	0.023
None of these/Other	1.46	0.93, 2.30	0.098
Retired	1.04	0.54, 2.00	0.916
**Religious attendance**			
More than once a week	—	—	
Once a week	0.79	0.67, 0.93	0.005
One to three times a month	0.74	0.57, 0.95	0.018
A few times a year	0.65	0.48, 0.88	0.006
Never	0.29	0.14, 0.58	6.31e-04
**Educational attainment (16+ years)**			
9-15	—	—	

Table S22e. Summary of fitted attrition model in Nigeria

**Characteristic**	**Odds Ratio**	**95% CI**	**p-value**
Up to 8	1.66	1.19, 2.34	0.003
16+	1.02	0.76, 1.37	0.892
**Born in This country**			
Born in this country	—	—	
Born in another country	0.92	0.41, 2.06	0.840
**Race plurality (prominent race/ethnic group [0] or not [1])**	0.89	0.80, 1.0	0.040
**Urbanicity**			
A small town or village	—	—	
A large city	1.16	0.93, 1.44	0.189
A rural area or on a farm	1.05	0.82, 1.34	0.715
A suburb of a large city	0.96	0.71, 1.29	0.776
**Monthly household income**			
Nigeria: 47,001 – 57,000 naira/month	—	—	
Nigeria: 24,001 – 30,000 naira/month	1.00	0.74, 1.35	0.996
Nigeria: 57,001 – 67,000 naira/month	0.96	0.70, 1.32	0.810
Nigeria: 30,001 – 37,000 naira/month	1.04	0.75, 1.44	0.800
Nigeria: 37,001 – 47,000 naira/month	1.00	0.70, 1.43	0.983
Nigeria: 18,001 – 24,000 naira/month	1.31	0.94, 1.83	0.108
Nigeria: 77,001 – 100,000 naira/month	1.02	0.73, 1.43	0.899
Nigeria: 67,001 – 77,000 naira/month	0.80	0.58, 1.11	0.181
(None/No household income)	1.25	0.85, 1.84	0.247
Nigeria: 100,001 – 150,000 naira/month	0.85	0.58, 1.25	0.411
Nigeria: More than 150,000 naira/month	1.27	0.90, 1.78	0.166
Nigeria: 13,001 – 18,000 naira/month	1.56	1.05, 2.32	0.027
Nigeria: 9,001 – 13,000 naira/month	1.00	0.68, 1.46	0.998
Nigeria: 3,701 – 6,000 naira/month	2.15	1.27, 3.64	0.005
Nigeria: 6,001 – 9,000 naira/month	0.91	0.51, 1.65	0.766
Nigeria: 3,700 naira/month or less	1.10	0.61, 1.98	0.757

Abbreviations: CI = Confidence Interval, OR = Odds Ratio

Notes. N=6827; attrition weights were estimated using the 'survey::svyglm(family=quasibinomial('logit'))' function. All continuous predictors were standardized and all categorical predictors used the most common category as the reference group. Reported p-values are based on the fitted regression model and no adjustments for multiple testing were done within this table.

Table S22f. Summary of principal components in Nigeria

PC	Percent Explained by each PC	Cumulative Percent Explained
1	38.82	38.82
2	4.37	43.19
3	2.54	45.74
4	2.03	47.76
5	1.85	49.61
6	1.76	51.37
**7**	**1.52**	**52.89**
8	1.42	54.31
9	1.37	55.68
10	1.32	57.00
11	1.29	58.29
12	1.25	59.54
13	1.22	60.76
14	1.18	61.94
15	1.16	63.10
16	1.10	64.20
17	1.09	65.28
18	1.06	66.35
19	1.02	67.37
20	1.01	68.38

Notes. N=6827; PCA was conducted using 'survey::svyprcomp(.)' function using all available contemporaneous exposures at wave 1. All PCs were standardized prior to being used as predictors. The bolded row represented the number of retained components for analysis was 7.

Table S22g. Associations of forgivingness with adult well-being and other outcomes at Wave 2 in Nigeria

	Model 1: Demographic and Childhood Variables as Covariates					Model 2: Demographic, Childhood, and Other Wave 1 Confounding Variables (Via Principal Components) as Covariates				
Outcome	RR	ES	95% CI	SE	p-value	RR	ES	95% CI	SE	p-value
Human Flourishing										
Secure flourishing index		0.06	(0.01,0.10)	0.02	0.011*		0.04	(-0.01,0.08)	0.02	0.089
Flourishing index		0.06	(0.01,0.10)	0.02	0.009*		0.04	(-0.01,0.08)	0.02	0.088
Happiness & life satisfaction		0.01	(-0.03,0.06)	0.02	0.563		0.01	(-0.04,0.05)	0.02	0.823
Physical & mental health		0.01	(-0.03,0.06)	0.02	0.582		0.00	(-0.04,0.05)	0.02	0.895
Meaning & purpose		0.05	(0.01,0.10)	0.02	0.024*		0.04	(-0.01,0.09)	0.02	0.098
Character & virtue		0.07	(0.03,0.11)	0.02	1.63e-03**		0.06	(0.02,0.10)	0.02	0.005*
Close social relationships		0.02	(-0.02,0.06)	0.02	0.365		0.01	(-0.04,0.05)	0.02	0.806
Financial & material security		0.02	(-0.03,0.07)	0.03	0.409		0.02	(-0.03,0.07)	0.03	0.514
Psychological Well-Being										
Happiness (a)		0.02	(-0.03,0.07)	0.03	0.499		0.01	(-0.04,0.06)	0.03	0.687
Life satisfaction (a)		0.00	(-0.04,0.05)	0.02	0.829		-0.00	(-0.05,0.04)	0.02	0.935
Current life evaluation		0.02	(-0.01,0.05)	0.02	0.263		0.02	(-0.01,0.06)	0.02	0.230
Future life evaluation		0.05	(0.01,0.10)	0.02	0.023*		0.05	(0.00,0.09)	0.02	0.030*
Optimism		0.04	(-0.01,0.10)	0.03	0.148		0.04	(-0.02,0.10)	0.03	0.203
Freedom to pursue what's important		0.04	(-0.01,0.09)	0.03	0.095		0.04	(-0.01,0.09)	0.03	0.161
Inner peace	1.01		(0.99,1.02)	0.01	0.457	1.01		(0.99,1.02)	0.01	0.508
Life balance	1.01		(0.99,1.02)	0.01	0.576	1.00		(0.98,1.02)	0.01	0.768
Sense of mastery	1.01		(0.99,1.03)	0.01	0.418	1.00		(0.99,1.02)	0.01	0.584
Meaningful activities (c)		0.05	(0.01,0.10)	0.02	0.028*		0.05	(-0.00,0.09)	0.02	0.066
Understanding purpose (c)		0.03	(-0.02,0.08)	0.03	0.315		0.01	(-0.04,0.07)	0.03	0.610
Self-rated mental health (b)		0.03	(-0.02,0.08)	0.03	0.288		0.02	(-0.03,0.07)	0.03	0.511
Psychological Distress										
Traumatic distress	0.99		(0.97,1.01)	0.01	0.362	1.00		(0.97,1.02)	0.01	0.710
Depression symptoms composite	1.00		(0.98,1.03)	0.01	0.708	1.01		(0.99,1.03)	0.01	0.524
Depression – feel hopeless	1.00		(0.98,1.02)	0.01	0.770	1.00		(0.98,1.02)	0.01	0.955
Depression – loss of interest	1.00		(0.98,1.02)	0.01	0.801	1.01		(0.98,1.03)	0.01	0.605
Anxiety symptoms composite	0.99		(0.97,1.01)	0.01	0.347	0.99		(0.98,1.01)	0.01	0.607
Anxiety – feel on edge	1.00		(0.98,1.01)	0.01	0.687	1.00		(0.98,1.02)	0.01	0.930
Anxiety – cannot stop worrying	0.99		(0.97,1.01)	0.01	0.273	0.99		(0.97,1.01)	0.01	0.440
Suffering	1.00		(0.98,1.02)	0.01	0.875	1.00		(0.98,1.02)	0.01	0.772
Social Well-Being										
Relationship contentment (e)		0.03	(-0.02,0.08)	0.03	0.218		0.02	(-0.04,0.07)	0.03	0.530
Relationship satisfaction (e)		0.00	(-0.04,0.04)	0.02	0.973		-0.01	(-0.05,0.04)	0.02	0.742
Social support		-0.03	(-0.07,0.01)	0.02	0.127		-0.04	(-0.08,0.00)	0.02	0.071
Intimate/close friend	1.01		(0.99,1.03)	0.01	0.169	1.01		(0.99,1.03)	0.01	0.256
Government approval	1.00		(0.98,1.02)	0.01	0.883	1.00		(0.98,1.02)	0.01	0.872
Say in government	1.01		(0.99,1.03)	0.01	0.355	1.01		(0.99,1.03)	0.01	0.353
Belonging in country		0.02	(-0.02,0.05)	0.02	0.443		0.01	(-0.03,0.05)	0.02	0.551
City/place satisfaction	0.99		(0.98,1.01)	0.01	0.542	0.99		(0.98,1.01)	0.01	0.374
Trust within country	1.00		(0.98,1.03)	0.01	0.636	1.00		(0.98,1.02)	0.01	0.814
Social Participation										
Ever been married	1.00		(0.98,1.01)	0.01	0.807	1.00		(0.98,1.01)	0.01	0.697
Currently divorced	1.00		(0.99,1.00)	0.00	0.799	1.00		(0.99,1.00)	0.00	0.871
Number of children		-0.02	(-0.06,0.02)	0.02	0.384		-0.02	(-0.06,0.02)	0.02	0.281
Weekly+ community participation	0.99		(0.98,1.01)	0.01	0.504	1.00		(0.98,1.02)	0.01	0.832
Weekly+ religious attendance	1.00		(0.98,1.01)	0.01	0.729	1.00		(0.98,1.01)	0.01	0.657
Social Distress										
Loneliness		0.02	(-0.02,0.07)	0.02	0.338		0.03	(-0.01,0.08)	0.02	0.153
Perceived discrimination	0.99		(0.97,1.01)	0.01	0.470	1.00		(0.98,1.02)	0.01	0.712
Character & Prosocial Behavior										
Orientation to promote good (d)		0.06	(0.01,0.11)	0.03	0.020*		0.05	(0.00,0.10)	0.02	0.043*
Delayed gratification (d)		0.05	(-0.00,0.09)	0.02	0.057		0.04	(-0.01,0.09)	0.02	0.100
Hope		0.03	(-0.02,0.09)	0.03	0.204		0.03	(-0.03,0.08)	0.03	0.320
Gratitude		0.01	(-0.03,0.05)	0.02	0.568		0.01	(-0.03,0.05)	0.02	0.763
Showing love/care		0.04	(-0.01,0.09)	0.02	0.084		0.03	(-0.02,0.08)	0.03	0.216
Forgivingness	1.02		(1.01,1.03)	0.01	4.36e-03**	1.02		(1.01,1.03)	0.01	0.006*
Charitable giving	0.99		(0.97,1.02)	0.01	0.495	0.99		(0.97,1.02)	0.01	0.689
Helping strangers	1.00		(0.98,1.01)	0.01	0.678	1.00		(0.98,1.01)	0.01	0.812
Volunteering	0.99		(0.97,1.01)	0.01	0.403	0.99		(0.98,1.01)	0.01	0.586
Physical Health & Health Behavior										
Self-rated physical health (b)		-0.01	(-0.05,0.04)	0.02	0.772		-0.01	(-0.06,0.03)	0.02	0.621
Health problems	1.01		(0.99,1.03)	0.01	0.343	1.01		(0.99,1.03)	0.01	0.242
Pain in past 4 weeks	1.02		(0.99,1.04)	0.01	0.186	1.02		(0.99,1.04)	0.01	0.146
Daily smoker	1.00		(0.99,1.01)	0.00	0.890	1.00		(0.99,1.01)	0.00	0.768
Number of drinks per week		0.00	(-0.04,0.05)	0.02	0.925		0.02	(-0.04,0.07)	0.03	0.558
Days exercise per week		0.02	(-0.02,0.06)	0.02	0.278		0.02	(-0.02,0.06)	0.02	0.249
Socioeconomic Outcomes										
Financial security (f)		-0.01	(-0.06,0.04)	0.03	0.761		-0.01	(-0.06,0.04)	0.03	0.741
Material security (f)		0.04	(-0.01,0.09)	0.03	0.119		0.04	(-0.02,0.09)	0.03	0.189
Educational attainment (16+ years)	1.00		(0.99,1.01)	0.00	0.736	1.00		(1.00,1.01)	0.00	0.541
Currently employed	1.00		(0.98,1.02)	0.01	0.947	1.00		(0.98,1.02)	0.01	0.792
Financially comfortable/getting by	0.99		(0.98,1.01)	0.01	0.508	0.99		(0.98,1.01)	0.01	0.409
Own home	0.99		(0.97,1.01)	0.01	0.470	0.99		(0.97,1.01)	0.01	0.328
Income – top quintile	1.01		(0.99,1.02)	0.01	0.534	1.01		(0.99,1.02)	0.01	0.562
Religion & Spirituality										
Religious/spiritual connection	1.00		(0.99,1.01)	0.01	0.955	1.00		(0.98,1.01)	0.01	0.629
Belief in life after death	0.99		(0.97,1.01)	0.01	0.370	0.99		(0.97,1.01)	0.01	0.406
Transformative religious experience	0.99		(0.97,1.01)	0.01	0.254	0.99		(0.97,1.01)	0.01	0.421
Religious reading or listening	1.00		(0.98,1.02)	0.01	0.648	0.99		(0.97,1.01)	0.01	0.515
Prayer or meditation	1.00		(0.98,1.01)	0.01	0.670	1.00		(0.98,1.01)	0.01	0.475
Belief in God/gods/spiritual forces	1.00		(1.00,1.00)	0.00	0.850	1.00		(1.00,1.00)	0.00	0.855
Religious centrality	1.00		(0.99,1.02)	0.01	0.794	1.00		(0.99,1.01)	0.01	0.976
Religious/spiritual comfort	1.00		(0.99,1.01)	0.01	0.983	1.00		(0.99,1.01)	0.01	0.632
Feel loved by God	1.00		(0.99,1.01)	0.00	0.372	1.00		(0.99,1.01)	0.00	0.433
Feel punished by God	0.99		(0.97,1.00)	0.01	0.157	0.99		(0.97,1.01)	0.01	0.246
Experienced religious criticism	0.99		(0.97,1.01)	0.01	0.240	0.99		(0.97,1.01)	0.01	0.353
Faith-sharing	1.01		(0.99,1.03)	0.01	0.288	1.01		(0.99,1.03)	0.01	0.313

Notes. N=6827; Reference for focal predictor: never/rarely. RR, risk-ratio, null effect is 1.00; ES, effect size measure for standardized regression coefficient, null effect is 0.00; SE, standard error, the SE reported for binary/Likert-type outcomes where risk-ratios are on the log(RR) scale; CI, confidence interval; p-value, a Wald-type test of the null hypothesis that the effect of the focal predictor is zero; (a) item part of the Happiness & Life Satisfaction domain of the Secure Flourishing Index; (b) item part of the Physical & Mental Health domain of the Secure Flourishing Index; (c) item part of the Meaning & Purpose domain of the Secure Flourishing Index; (d) item part of the Character & Virtue domain of the Secure Flourishing Index; (e) item part of the Subjective Social Connectedness domain of the Secure Flourishing Index; (f) item part of the Financial & Material Security domain of the Secure Flourishing Index.

Multiple imputation was performed to impute missing data on the covariates, exposure, and outcomes. All models controlled for sociodemographic and childhood factors assessed at Wave 1. For Model 2 with PC (principal components), the first seven principal components of the entire set of contemporaneous confounders assessed at Wave 1 were included as additional covariates of the outcomes at Wave 2.

An outcome-wide analytic approach was used, and a separate model was run for each outcome. A different type of model was run depending on the nature of the outcome: (1) for each binary outcome, a weighted generalized linear model (with a log link and Poisson distribution) was used to estimate an RR; and (2) for each continuous outcome, a weighted linear regression model was used to estimate a ES. All effect sizes were standardized. For continuous outcomes, the ES represents the change in SD on the outcome between the lower and upper categories of the binary focal predictor. For binary outcomes, the RR represents the change in risk of being in the upper category compared to the lower category between the lower and upper categories of the binary focal predictor.

P-value significance thresholds: $p < 0.05$*, $p < 0.005$**, (Bonferroni) $p < 6.41e-04$***, correction for multiple testing using Bonferroni adjusted significant threshold.

Table S22h. Associations of forgivingness with adult well-being and other outcomes at Wave 2 in Nigeria using complete-case analyses with attrition weights.

	Model 1: Demographic and Childhood Variables as Covariates					Model 2: Demographic, Childhood, and Other Wave 1 Confounding Variables (Via Principal Components) as Covariates				
Outcome	RR	ES	95% CI	SE	p-value	RR	ES	95% CI	SE	p-value
Human Flourishing										
Secure flourishing index		0.08	(0.02,0.13)	0.03	0.012*		0.06	(-0.00,0.12)	0.03	0.052
Flourishing index		0.07	(0.01,0.13)	0.03	0.016*		0.06	(-0.00,0.12)	0.03	0.063
Happiness & life satisfaction		0.04	(-0.02,0.10)	0.03	0.177		0.03	(-0.03,0.09)	0.03	0.280
Physical & mental health		0.02	(-0.04,0.07)	0.03	0.549		0.01	(-0.05,0.06)	0.03	0.816
Meaning & purpose		0.09	(0.03,0.15)	0.03	3.63e-03**		0.08	(0.02,0.14)	0.03	0.013*
Character & virtue		0.08	(0.02,0.14)	0.03	0.008*		0.08	(0.02,0.14)	0.03	0.013*
Close social relationships		0.02	(-0.04,0.07)	0.03	0.525		0.00	(-0.06,0.06)	0.03	0.949
Financial & material security		0.04	(-0.02,0.10)	0.03	0.172		0.04	(-0.03,0.10)	0.03	0.265
Psychological Well-Being										
Happiness (a)		0.03	(-0.03,0.09)	0.03	0.316		0.03	(-0.03,0.08)	0.03	0.356
Life satisfaction (a)		0.04	(-0.01,0.09)	0.03	0.159		0.03	(-0.03,0.08)	0.03	0.321
Current life evaluation		0.05	(0.01,0.10)	0.02	0.016*		0.06	(0.01,0.10)	0.02	0.011*
Future life evaluation		0.10	(0.04,0.17)	0.03	1.83e-03**		0.10	(0.03,0.17)	0.03	2.83e-03**
Optimism		0.05	(-0.01,0.12)	0.03	0.120		0.05	(-0.02,0.12)	0.04	0.147
Freedom to pursue what's important		0.09	(0.03,0.16)	0.03	0.005*		0.09	(0.02,0.16)	0.04	0.012*
Inner peace	1.01		(0.98,1.03)	0.01	0.551	1.01		(0.98,1.03)	0.01	0.625
Life balance	1.02		(1.00,1.05)	0.01	0.062	1.02		(0.99,1.05)	0.01	0.126
Sense of mastery	1.01		(0.99,1.04)	0.01	0.295	1.01		(0.98,1.04)	0.01	0.478
Meaningful activities (c)		0.09	(0.04,0.14)	0.03	1.3e-03**		0.08	(0.02,0.14)	0.03	4.77e-03**
Understanding purpose (c)		0.05	(-0.01,0.11)	0.03	0.127		0.04	(-0.03,0.10)	0.03	0.252
Self-rated mental health (b)		0.04	(-0.02,0.09)	0.03	0.173		0.03	(-0.03,0.09)	0.03	0.343
Psychological Distress										
Traumatic distress	0.99		(0.96,1.01)	0.01	0.274	0.99		(0.97,1.02)	0.01	0.566
Depression symptoms composite	1.00		(0.97,1.02)	0.01	0.743	1.00		(0.97,1.03)	0.01	0.932
Depression – feel hopeless	0.98		(0.96,1.01)	0.01	0.176	0.99		(0.96,1.01)	0.01	0.298
Depression – loss of interest	1.00		(0.97,1.03)	0.02	0.837	1.00		(0.97,1.03)	0.02	0.886
Anxiety symptoms composite	0.99		(0.96,1.01)	0.01	0.393	0.99		(0.96,1.02)	0.01	0.428
Anxiety – feel on edge	1.00		(0.98,1.02)	0.01	0.963	1.00		(0.98,1.03)	0.01	0.713
Anxiety – cannot stop worrying	0.99		(0.96,1.02)	0.01	0.452	0.99		(0.97,1.02)	0.01	0.560
Suffering	1.00		(0.97,1.03)	0.01	0.867	1.01		(0.98,1.03)	0.01	0.640
Social Well-Being										
Relationship contentment (e)		0.03	(-0.02,0.08)	0.03	0.184		0.02	(-0.03,0.07)	0.03	0.541
Relationship satisfaction (e)		-0.00	(-0.06,0.06)	0.03	0.938		-0.01	(-0.07,0.05)	0.03	0.709
Social support		-0.04	(-0.08,0.01)	0.02	0.088		-0.04	(-0.09,0.00)	0.02	0.076
Intimate/close friend	1.01		(1.00,1.03)	0.01	0.138	1.01		(0.99,1.03)	0.01	0.212
Government approval	1.00		(0.97,1.02)	0.01	0.724	0.99		(0.96,1.02)	0.01	0.509
Say in government	1.02		(0.99,1.05)	0.01	0.202	1.02		(0.99,1.05)	0.02	0.322
Belonging in country		0.05	(-0.01,0.10)	0.03	0.107		0.04	(-0.02,0.10)	0.03	0.199
City/place satisfaction	1.00		(0.98,1.02)	0.01	0.876	0.99		(0.97,1.01)	0.01	0.540
Trust within country	1.01		(0.99,1.04)	0.01	0.393	1.00		(0.98,1.03)	0.01	0.715
Social Participation										
Ever been married	1.00		(0.98,1.03)	0.01	0.759	1.00		(0.98,1.02)	0.01	0.989
Currently divorced	1.00		(1.00,1.01)	0.00	0.494	1.00		(1.00,1.01)	0.00	0.376
Number of children		-0.01	(-0.06,0.04)	0.02	0.660		-0.02	(-0.06,0.03)	0.02	0.497
Weekly+ community participation	0.99		(0.96,1.01)	0.01	0.318	0.99		(0.97,1.02)	0.01	0.491
Weekly+ religious attendance	1.00		(0.98,1.01)	0.01	0.611	0.99		(0.98,1.01)	0.01	0.559
Social Distress										
Loneliness		0.01	(-0.04,0.07)	0.03	0.589		0.03	(-0.02,0.09)	0.03	0.268
Perceived discrimination	0.99		(0.96,1.01)	0.01	0.349	0.99		(0.97,1.02)	0.01	0.484
Character & Prosocial Behavior										
Orientation to promote good (d)		0.06	(0.00,0.12)	0.03	0.044*		0.06	(-0.00,0.12)	0.03	0.063
Delayed gratification (d)		0.08	(0.01,0.15)	0.03	0.019*		0.08	(0.01,0.15)	0.04	0.024*
Hope		0.07	(0.01,0.13)	0.03	0.014*		0.07	(0.01,0.12)	0.03	0.022*
Gratitude		0.03	(-0.03,0.09)	0.03	0.346		0.02	(-0.04,0.09)	0.03	0.447
Showing love/care		0.08	(0.01,0.14)	0.03	0.017*		0.07	(0.01,0.13)	0.03	0.030*
Forgivingness	1.02		(1.01,1.04)	0.01	2.69e-03**	1.02		(1.01,1.04)	0.01	3.5e-03**
Charitable giving	0.98		(0.96,1.01)	0.01	0.206	0.98		(0.96,1.01)	0.01	0.195
Helping strangers	0.99		(0.98,1.00)	0.01	0.240	1.00		(0.98,1.01)	0.01	0.530
Volunteering	0.98		(0.95,1.00)	0.01	0.063	0.98		(0.96,1.00)	0.01	0.112
Physical Health & Health Behavior										
Self-rated physical health (b)		-0.01	(-0.06,0.04)	0.03	0.745		-0.01	(-0.07,0.04)	0.03	0.575
Health problems	1.00		(0.98,1.03)	0.01	0.683	1.01		(0.98,1.03)	0.01	0.564
Pain in past 4 weeks	1.02		(0.99,1.05)	0.01	0.153	1.02		(1.00,1.05)	0.01	0.096
Daily smoker	1.00		(1.00,1.01)	0.00	0.592	1.01		(1.00,1.01)	0.00	0.095
Number of drinks per week		0.00	(-0.03,0.04)	0.02	0.984		0.02	(-0.02,0.06)	0.02	0.343
Days exercise per week		0.03	(-0.02,0.08)	0.03	0.259		0.03	(-0.02,0.08)	0.03	0.257
Socioeconomic Outcomes										
Financial security (f)		0.02	(-0.04,0.08)	0.03	0.588		0.01	(-0.05,0.08)	0.03	0.666
Material security (f)		0.06	(-0.00,0.12)	0.03	0.057		0.05	(-0.01,0.11)	0.03	0.119
Educational attainment (16+ years)	1.00		(1.00,1.00)	0.00	0.399	1.00		(1.00,1.00)	0.00	0.331
Currently employed	1.00		(0.98,1.03)	0.01	0.722	1.01		(0.98,1.03)	0.01	0.539
Financially comfortable/getting by	1.01		(0.98,1.03)	0.01	0.685	1.00		(0.98,1.03)	0.01	0.850
Own home	0.98		(0.96,1.01)	0.01	0.206	0.98		(0.96,1.01)	0.01	0.163
Income – top quintile	1.02		(0.99,1.04)	0.01	0.210	1.02		(0.99,1.04)	0.01	0.199
Religion & Spirituality										
Religious/spiritual connection	1.01		(0.99,1.03)	0.01	0.304	1.01		(0.99,1.02)	0.01	0.540
Belief in life after death	1.00		(0.98,1.02)	0.01	0.944	1.00		(0.98,1.02)	0.01	0.884
Transformative religious experience	0.99		(0.97,1.02)	0.01	0.531	0.99		(0.97,1.02)	0.01	0.495
Religious reading or listening	0.99		(0.97,1.01)	0.01	0.458	0.99		(0.97,1.01)	0.01	0.381
Prayer or meditation	0.99		(0.98,1.01)	0.01	0.303	0.99		(0.98,1.00)	0.01	0.119
Belief in God/gods/spiritual forces	1.00		(1.00,1.00)	0.00	0.864	1.00		(1.00,1.00)	0.00	0.822
Religious centrality	1.00		(0.99,1.02)	0.01	0.769	1.00		(0.98,1.02)	0.01	0.895
Religious/spiritual comfort	1.00		(0.99,1.02)	0.01	0.556	1.00		(0.99,1.02)	0.01	0.860
Feel loved by God	1.01		(0.99,1.02)	0.01	0.359	1.01		(0.99,1.02)	0.01	0.419
Feel punished by God	0.98		(0.96,1.00)	0.01	0.077	0.98		(0.96,1.00)	0.01	0.106
Experienced religious criticism	0.99		(0.97,1.01)	0.01	0.381	0.99		(0.97,1.02)	0.01	0.573
Faith-sharing	1.02		(1.00,1.04)	0.01	0.108	1.02		(1.00,1.05)	0.01	0.116

Table S22h. Associations of forgivingness with adult well-being and other outcomes at Wave 2 in Nigeria using complete-case analyses with attrition weights.

Outcome	Model 1: Demographic and Childhood Variables as Covariates					Model 2: Demographic, Childhood, and Other Wave 1 Confounding Variables (Via Principal Components) as Covariates				
	RR	ES	95% CI	SE	p-value	RR	ES	95% CI	SE	p-value

Notes. N=3146; Reference for focal predictor: never/rarely. RR, risk-ratio, null effect is 1.00; ES, effect size measure for standardized regression coefficient, null effect is 0.00; SE, standard error, the SE reported for binary/Likert-type outcomes where risk-ratios are on the log(RR) scale; CI, confidence interval; p-value, a Wald-type test of the null hypothesis that the effect of the focal predictor is zero; (a) item part of the Happiness & Life Satisfaction domain of the Secure Flourishing Index; (b) item part of the Physical & Mental Health domain of the Secure Flourishing Index; (c) item part of the Meaning & Purpose domain of the Secure Flourishing Index; (d) item part of the Character & Virtue domain of the Secure Flourishing Index; (e) item part of the Subjective Social Connectedness domain of the Secure Flourishing Index; (f) item part of the Financial & Material Security domain of the Secure Flourishing Index.

Attrition weights were computed to adjust the complete case data (those who responded at Wave 2 to at least 50% of the questions) and multiple imputation was used to impute missing data on all remaining within wave on the covariates, exposure, and outcomes. All models controlled for sociodemographic and childhood factors assessed at Wave 1. For Model 2 with PC (principal components), the first seven principal components of the entire set of contemporaneous confounders assessed at Wave 1 were included as additional covariates of the outcomes at Wave 2.

An outcome-wide analytic approach was used, and a separate model was run for each outcome. A different type of model was run depending on the nature of the outcome: (1) for each binary outcome, a weighted generalized linear model (with a log link and Poisson distribution) was used to estimate an RR; and (2) for each continuous outcome, a weighted linear regression model was used to estimate a ES. All effect sizes were standardized. For continuous outcomes, the ES represents the change in SD on the outcome between the lower and upper categories of the binary focal predictor. For binary outcomes, the RR represents the change in risk of being in the upper category compared to the lower category between the lower and upper categories of the binary focal predictor.

P-value significance thresholds: p < 0.05*, p < 0.005**, (Bonferroni) p < 6.41e-04***, correction for multiple testing using Bonferroni adjusted significant threshold.

Table S22i. Sensitivity analysis of forgivingness outcome-wide results to unmeasured confounding using E-values in Nigeria

	Multiple Imputation				Complete Case w/ Attrition Weights			
	Model 1: Demographics and Childhood Variables as Covariates		Model 2: Demographics, Childhood, and Other Wave 1 Confounders (Via Principal Components) as Covariates		Model 1: Demographics and Childhood Variables as Covariates		Model 2: Demographics, Childhood, and Other Wave 1 Confounders (Via Principal Components) as Covariates	
Outcome	EE	ECI	EE	ECI	EE	ECI	EE	ECI
Human Flourishing								
Secure flourishing index	1.70	1.26	1.53	1.00	1.87	1.29	1.72	1.00
Flourishing index	1.70	1.28	1.51	1.00	1.83	1.26	1.70	1.00
Happiness & life satisfaction	1.27	1.00	1.16	1.00	1.54	1.00	1.46	1.00
Physical & mental health	1.26	1.00	1.11	1.00	1.30	1.00	1.17	1.00
Meaning & purpose	1.69	1.19	1.56	1.00	2.02	1.44	1.89	1.29
Character & virtue	1.82	1.41	1.72	1.31	1.94	1.35	1.92	1.30
Close social relationships	1.34	1.00	1.16	1.00	1.31	1.00	1.08	1.00
Financial & material security	1.36	1.00	1.31	1.00	1.56	1.00	1.49	1.00
Psychological Well-Being								
Happiness	1.31	1.00	1.23	1.00	1.43	1.00	1.41	1.00
Life satisfaction	1.14	1.00	1.08	1.00	1.52	1.00	1.41	1.00
Current life evaluation	1.33	1.00	1.35	1.00	1.67	1.22	1.69	1.26
Future life evaluation	1.66	1.19	1.62	1.15	2.13	1.51	2.10	1.48
Optimism	1.59	1.00	1.54	1.00	1.66	1.00	1.64	1.00
Freedom to pursue what’s important	1.57	1.00	1.50	1.00	2.04	1.41	2.01	1.34
Inner peace	1.18	1.00	1.17	1.00	1.19	1.00	1.17	1.00
Life balance	1.16	1.00	1.11	1.00	1.40	1.00	1.35	1.00
Sense of mastery	1.20	1.00	1.16	1.00	1.28	1.00	1.22	1.00
Meaningful activities	1.67	1.17	1.60	1.00	2.00	1.49	1.91	1.38
Understanding purpose	1.41	1.00	1.27	1.00	1.63	1.00	1.52	1.00
Self-rated mental health	1.42	1.00	1.31	1.00	1.51	1.00	1.42	1.00
Psychological Distress								
Traumatic distress	1.24	1.00	1.14	1.00	1.29	1.00	1.20	1.00
Depression symptoms composite	1.14	1.00	1.19	1.00	1.15	1.00	1.07	1.00
Depression – feel hopeless	1.12	1.00	1.05	1.00	1.34	1.00	1.28	1.00
Depression – loss of interest	1.11	1.00	1.17	1.00	1.12	1.00	1.10	1.00
Anxiety symptoms composite	1.22	1.00	1.16	1.00	1.25	1.00	1.24	1.00
Anxiety – feel on edge	1.13	1.00	1.06	1.00	1.04	1.00	1.14	1.00
Anxiety – cannot stop worrying	1.24	1.00	1.20	1.00	1.23	1.00	1.20	1.00
Suffering	1.08	1.00	1.12	1.00	1.10	1.00	1.18	1.00
Social Well-Being								
Relationship contentment	1.46	1.00	1.30	1.00	1.47	1.00	1.28	1.00
Relationship satisfaction	1.05	1.00	1.18	1.00	1.09	1.00	1.24	1.00
Social support	1.47	1.00	1.54	1.00	1.51	1.00	1.55	1.00
Intimate/close friend	1.28	1.00	1.25	1.00	1.28	1.00	1.26	1.00
Government approval	1.08	1.00	1.09	1.00	1.15	1.00	1.22	1.00
Say in government	1.24	1.00	1.24	1.00	1.34	1.00	1.30	1.00
Belonging in country	1.29	1.00	1.25	1.00	1.59	1.00	1.51	1.00
City/place satisfaction	1.16	1.00	1.20	1.00	1.08	1.00	1.18	1.00
Trust within country	1.15	1.00	1.10	1.00	1.25	1.00	1.15	1.00
Social Participation								
Ever been married	1.09	1.00	1.12	1.00	1.12	1.00	1.02	1.00
Currently divorced	1.05	1.00	1.04	1.00	1.08	1.00	1.10	1.00
Number of children	1.32	1.00	1.36	1.00	1.23	1.00	1.28	1.00
Weekly+ community participation	1.18	1.00	1.09	1.00	1.27	1.00	1.21	1.00
Weekly+ religious attendance	1.10	1.00	1.12	1.00	1.14	1.00	1.15	1.00
Social Distress								
Loneliness	1.36	1.00	1.49	1.00	1.28	1.00	1.45	1.00
Perceived discrimination	1.20	1.00	1.14	1.00	1.27	1.00	1.22	1.00
Character & Prosocial Behavior								
Orientation to promote good	1.73	1.22	1.64	1.09	1.71	1.08	1.69	1.00
Delayed gratification	1.60	1.00	1.54	1.00	1.91	1.26	1.90	1.22
Hope	1.49	1.00	1.41	1.00	1.84	1.28	1.78	1.21
Gratitude	1.24	1.00	1.17	1.00	1.43	1.00	1.38	1.00
Showing love/care	1.58	1.00	1.46	1.00	1.88	1.27	1.82	1.18
Forgivingness	1.35	1.17	1.35	1.16	1.40	1.21	1.39	1.20
Charitable giving	1.21	1.00	1.16	1.00	1.31	1.00	1.32	1.00
Helping strangers	1.11	1.00	1.08	1.00	1.19	1.00	1.13	1.00
Volunteering	1.21	1.00	1.16	1.00	1.39	1.00	1.35	1.00
Physical Health & Health Behavior								
Self-rated physical health	1.17	1.00	1.24	1.00	1.20	1.00	1.28	1.00
Health problems	1.22	1.00	1.25	1.00	1.15	1.00	1.18	1.00
Pain in past 4 weeks	1.30	1.00	1.33	1.00	1.35	1.00	1.40	1.00
Daily smoker	1.05	1.00	1.07	1.00	1.08	1.00	1.18	1.00
Number of drinks per week	1.09	1.00	1.29	1.00	1.04	1.00	1.31	1.00
Days exercise per week	1.35	1.00	1.37	1.00	1.42	1.00	1.43	1.00
Socioeconomic Outcomes								
Financial security	1.19	1.00	1.20	1.00	1.30	1.00	1.26	1.00
Material security	1.56	1.00	1.50	1.00	1.70	1.00	1.61	1.00
Educational attainment (16+ years)	1.07	1.00	1.09	1.00	1.08	1.00	1.08	1.00
Currently employed	1.05	1.00	1.11	1.00	1.15	1.00	1.20	1.00
Financially comfortable/getting by	1.17	1.00	1.19	1.00	1.16	1.00	1.10	1.00
Own home	1.20	1.00	1.25	1.00	1.30	1.00	1.34	1.00
Income – top quintile	1.16	1.00	1.16	1.00	1.30	1.00	1.29	1.00
Religion & Spirituality								
Religious/spiritual connection	1.04	1.00	1.12	1.00	1.22	1.00	1.17	1.00
Belief in life after death	1.22	1.00	1.21	1.00	1.06	1.00	1.08	1.00
Transformative religious experience	1.26	1.00	1.21	1.00	1.20	1.00	1.21	1.00
Religious reading or listening	1.15	1.00	1.18	1.00	1.21	1.00	1.23	1.00
Prayer or meditation	1.11	1.00	1.15	1.00	1.17	1.00	1.23	1.00
Belief in God/gods/spiritual forces	1.04	1.00	1.04	1.00	1.03	1.00	1.03	1.00
Religious centrality	1.09	1.00	1.03	1.00	1.10	1.00	1.07	1.00
Religious/spiritual comfort	1.02	1.00	1.12	1.00	1.13	1.00	1.07	1.00
Feel loved by God	1.14	1.00	1.13	1.00	1.17	1.00	1.17	1.00
Feel punished by God	1.27	1.00	1.24	1.00	1.36	1.00	1.35	1.00
Experienced religious criticism	1.26	1.00	1.23	1.00	1.24	1.00	1.19	1.00
Faith-sharing	1.26	1.00	1.26	1.00	1.35	1.00	1.35	1.00

Notes. EE, E-value for estimate; ECI, E-value for the limit of the confidence interval. The formula for calculating E-values can be found in VanderWeele and Ding (2017). E-values for estimate are the minimum strength of association on the risk ratio scale that an unmeasured confounder would need to have with both the exposure and the outcome to fully explain away the observed association between the exposure and outcome, conditional on the measured covariates. E-values for the 95% CI closest to the null denote the minimum strength of association on the risk ratio scale that an unmeasured confounder would need to have with both the exposure and the outcome to shift the CI to include the null value, conditional on the measured covariates.

Table S23a. Weighted summary statistics for demographic and childhood variables in Philippines

**Characteristic**	**Wave 1** N = 5,292	**Wave 2** N = 2,684
Forgivingness, n (%)		
Always	2,524 (47.7%)	1,345 (50.1%)
Often	1,405 (26.6%)	728 (27.1%)
Rarely	1,134 (21.4%)	538 (20.0%)
Never	227 (4.3%)	73 (2.7%)
(Missing)	2 (0.0%)	0 (0.0%)
Year of birth, n (%)		
1943 or earlier (current age: 80+ years)	22 (0.4%)	13 (0.5%)
1943-1953 (current age: 70-79 years)	136 (2.6%)	100 (3.7%)
1953-1963 (current age: 60-69 years)	469 (8.9%)	260 (9.7%)
1963-1973 (current age: 50-59 years)	684 (12.9%)	352 (13.1%)
1973-1983 (current age: 40-49 years)	927 (17.5%)	476 (17.7%)
1983-1993 (current age: 30-39 years)	1,154 (21.8%)	622 (23.2%)
1993-1998 (current age: 25-29 years)	696 (13.1%)	354 (13.2%)
1998-2005 (current age: 18-24 years)	1,205 (22.8%)	507 (18.9%)
(Missing)	0 (0%)	0 (0%)
Age of participant		
Mean	38.4	39.9
Standard Deviation	15.3	15.3
Min, Max	18.0, 89.0	19.0, 90.0
Gender, n (%)		
Male	2,600 (49.1%)	1,312 (48.9%)
Female	2,668 (50.4%)	1,359 (50.6%)
Other	13 (0.2%)	7 (0.3%)
(Missing)	11 (0.2%)	6 (0.2%)
Respondent marital status, n (%)		
Single/Never been married	1,260 (23.8%)	624 (23.2%)
Married	2,320 (43.8%)	1,153 (42.9%)
Separated	240 (4.5%)	136 (5.1%)
Divorced	9 (0.2%)	0 (0.0%)
Widowed	263 (5.0%)	152 (5.7%)
Domestic partner	1,182 (22.3%)	617 (23.0%)
(Missing)	17 (0.3%)	2 (0.1%)
Education (years), n (%)		
Up to 8	1,240 (23.4%)	555 (20.7%)
9-15	3,702 (70.0%)	1,784 (66.5%)
16+	349 (6.6%)	345 (12.8%)
(Missing)	1 (0.0%)	0 (0%)
Employment status, n (%)		
Employed for an employer	1,331 (25.1%)	823 (30.6%)
Self-employed	1,334 (25.2%)	624 (23.3%)
Retired	146 (2.7%)	78 (2.9%)
Student	650 (12.3%)	209 (7.8%)
Homemaker	1,057 (20.0%)	450 (16.8%)
Unemployed and looking for a job	664 (12.6%)	401 (14.9%)
None of these/Other	111 (2.1%)	92 (3.4%)
(Missing)	0 (0%)	7 (0.3%)
Current religious service attendance, n (%)		
More than once a week	842 (15.9%)	395 (14.7%)
Once a week	1,928 (36.4%)	1,045 (38.9%)

Table S23a. Weighted summary statistics for demographic and childhood variables in Philippines

**Characteristic**	**Wave 1**<br>N = 5,292	**Wave 2**<br>N = 2,684
One to three times a month	1,368 (25.9%)	659 (24.6%)
A few times a year	930 (17.6%)	451 (16.8%)
Never	218 (4.1%)	129 (4.8%)
(Missing)	6 (0.1%)	6 (0.2%)
Immigration status, n (%)		
Born in this country	5,285 (99.9%)	2,681 (99.9%)
Born in another country	7 (0.1%)	3 (0.1%)
(Missing)	0 (0%)	0 (0%)
Parental marital status around age 12, n (%)		
Parents were married	4,538 (85.8%)	2,346 (87.4%)
Parents were divorced	64 (1.2%)	26 (1.0%)
Parents were never married	552 (10.4%)	237 (8.8%)
One or both of them had died	50 (0.9%)	29 (1.1%)
Unsure	56 (1.1%)	35 (1.3%)
(Missing)	32 (0.6%)	12 (0.4%)
Religious service attendance around age 12, n (%)		
At least once a week	2,436 (46.0%)	1,269 (47.3%)
One to three times a month	1,701 (32.1%)	835 (31.1%)
Less than once a month	901 (17.0%)	461 (17.2%)
Never	205 (3.9%)	102 (3.8%)
(Missing)	50 (0.9%)	17 (0.6%)
Relationship with mother when growing up, n (%)		
Very good	3,304 (62.4%)	1,671 (62.2%)
Somewhat good	1,727 (32.6%)	867 (32.3%)
Somewhat bad	129 (2.4%)	69 (2.6%)
Very bad	41 (0.8%)	26 (1.0%)
(Does not apply)	56 (1.1%)	29 (1.1%)
(Missing)	34 (0.6%)	22 (0.8%)
Relationship with father when growing up, n (%)		
Very good	3,415 (64.5%)	1,750 (65.2%)
Somewhat good	1,454 (27.5%)	721 (26.9%)
Somewhat bad	159 (3.0%)	82 (3.1%)
Very bad	60 (1.1%)	35 (1.3%)
(Does not apply)	106 (2.0%)	52 (1.9%)
(Missing)	97 (1.8%)	43 (1.6%)
Felt like an outsider in family when growing up, n (%)		
Yes	400 (7.5%)	188 (7.0%)
No	4,880 (92.2%)	2,494 (92.9%)
(Missing)	12 (0.2%)	3 (0.1%)
Experienced abuse when growing up, n (%)		
Yes	428 (8.1%)	222 (8.3%)
No	4,831 (91.3%)	2,460 (91.7%)
(Missing)	34 (0.6%)	2 (0.1%)
Self-rated health when growing up, n (%)		
Excellent	1,039 (19.6%)	483 (18.0%)
Very good	560 (10.6%)	276 (10.3%)
Good	2,178 (41.2%)	1,130 (42.1%)
Fair	1,244 (23.5%)	660 (24.6%)
Poor	271 (5.1%)	135 (5.0%)
(Missing)	0 (<0.0%)	0 (0%)

Table S23a. Weighted summary statistics for demographic and childhood variables in Philippines

**Characteristic**	**Wave 1**<br>N = 5,292	**Wave 2**<br>N = 2,684
Subjective financial status of family growing up, n (%)		
Lived comfortably	935 (17.7%)	445 (16.6%)
Got by	3,005 (56.8%)	1,512 (56.3%)
Found it difficult	1,055 (19.9%)	558 (20.8%)
Found it very difficult	294 (5.6%)	168 (6.3%)
(Missing)	2 (0.0%)	1 (0.0%)
Religious affiliation growing up, n (%)		
Christianity	4,972 (93.9%)	2,497 (93.0%)
Taoism	0 (0%)	0 (0%)
Confucianism	0 (0%)	0 (0%)
Primal, Animist, or Folk religion	13 (0.2%)	4 (0.2%)
Spiritism	0 (0%)	0 (0%)
Umbanda, Candomblé, and other African-derived religions	0 (0%)	0 (0%)
Chinese folk/traditional religion	0 (0%)	0 (0%)
Islam	273 (5.2%)	166 (6.2%)
Hinduism	0 (0%)	0 (0%)
Buddhism	1 (0.0%)	1 (0.0%)
Judaism	0 (0%)	0 (0%)
Sikhism	4 (0.1%)	5 (0.2%)
Baha'i	1 (0.0%)	0 (0%)
Jainism	0 (0%)	0 (0%)
Shinto	0 (0%)	0 (0%)
Some other religion	8 (0.2%)	6 (0.2%)
No religion/Atheist/Agnostic	9 (0.2%)	1 (0.0%)
(Missing)	11 (0.2%)	4 (0.2%)

Note. N (%); this table is based on non-imputed data. Cumulative percentages for variables may not add up to 100% due to rounding. Wave 1 characteristics weighted using the Gallup provided sampling weight, ANNUAL_WEIGHT_R2; Wave 2 characteristics weighted accounting for attrition by using the adjusted Wave 1 weight, ANNUAL_WEIGHT_R2, multiplied by the created attrition weight to account for dropout, to maintain nationally representative estimates for Wave 2 characteristics.

Table S23b. Weighted summary statistics for outcome variables in Philippines

Outcome	Wave 1<br>N = 5,292	Wave 2<br>N = 2,684
Secure flourishing index		
Mean	7.7	7.6
Standard Deviation	1.4	1.4
Min, Max	0.6, 10.0	0.8, 10.0
(Missing)	53 (1.0%)	26 (1.0%)
Flourishing index		
Mean	8.1	8.0
Standard Deviation	1.4	1.4
Min, Max	0.5, 10.0	1.0, 10.0
(Missing)	43 (0.8%)	25 (0.9%)
Happiness & life satisfaction		
Mean	7.4	7.4
Standard Deviation	2.1	2.0
Min, Max	0.0, 10.0	0.0, 10.0
(Missing)	7 (0.1%)	14 (0.5%)
Physical & mental health		
Mean	7.9	7.7
Standard Deviation	1.8	1.8
Min, Max	0.0, 10.0	0.0, 10.0
(Missing)	8 (0.2%)	9 (0.3%)
Meaning & purpose		
Mean	8.2	8.1
Standard Deviation	1.8	1.8
Min, Max	0.0, 10.0	0.0, 10.0
(Missing)	8 (0.2%)	11 (0.4%)
Character & virtue		
Mean	8.5	8.4
Standard Deviation	1.8	1.7
Min, Max	0.0, 10.0	0.0, 10.0
(Missing)	11 (0.2%)	11 (0.4%)
Close social relationships		
Mean	8.6	8.5
Standard Deviation	1.8	1.8
Min, Max	0.0, 10.0	0.0, 10.0
(Missing)	19 (0.4%)	7 (0.3%)
Financial & material security		
Mean	5.7	5.7
Standard Deviation	3.0	2.9
Min, Max	0.0, 10.0	0.0, 10.0
(Missing)	13 (0.2%)	7 (0.2%)
Happiness		
Mean	7.3	7.3
Standard Deviation	2.3	2.2
Min, Max	0.0, 10.0	0.0, 10.0
(Missing)	2 (<0.1%)	7 (0.3%)
Life satisfaction		
Mean	7.5	7.5
Standard Deviation	2.4	2.3
Min, Max	0.0, 10.0	0.0, 10.0
(Missing)	5 (<0.1%)	7 (0.2%)
Current life evaluation		

Table S23b. Weighted summary statistics for outcome variables in Philippines

Outcome	Wave 1<br>N = 5,292	Wave 2<br>N = 2,684
Mean	6.4	6.8
Standard Deviation	2.4	2.2
Min, Max	0.0, 10.0	0.0, 10.0
(Missing)	4 (<0.1%)	4 (0.2%)
Future life evaluation		
Mean	7.6	7.6
Standard Deviation	2.2	2.1
Min, Max	0.0, 10.0	0.0, 10.0
(Missing)	10 (0.2%)	8 (0.3%)
Optimism		
Mean	8.8	8.7
Standard Deviation	2.0	1.9
Min, Max	0.0, 10.0	0.0, 10.0
(Missing)	10 (0.2%)	4 (0.2%)
Freedom to pursue what's important		
Mean	8.6	8.6
Standard Deviation	2.0	2.0
Min, Max	0.0, 10.0	0.0, 10.0
(Missing)	7 (0.1%)	5 (0.2%)
Inner peace, n (%)		
Always	1,176 (22.2%)	622 (23.2%)
Often	1,486 (28.1%)	841 (31.3%)
Rarely	2,156 (40.7%)	1,029 (38.3%)
Never	471 (8.9%)	190 (7.1%)
(Missing)	3 (0.1%)	3 (0.1%)
Life balance, n (%)		
Always	1,047 (19.8%)	548 (20.4%)
Often	1,617 (30.6%)	803 (29.9%)
Rarely	2,182 (41.2%)	1,180 (43.9%)
Never	446 (8.4%)	152 (5.7%)
(Missing)	0 (<0.0%)	1 (0.0%)
Sense of mastery, n (%)		
Always	1,634 (30.9%)	870 (32.4%)
Often	1,667 (31.5%)	847 (31.5%)
Rarely	1,752 (33.1%)	860 (32.1%)
Never	236 (4.5%)	104 (3.9%)
(Missing)	2 (0.0%)	3 (0.1%)
Meaningful activities		
Mean	8.0	8.0
Standard Deviation	2.4	2.2
Min, Max	0.0, 10.0	0.0, 10.0
(Missing)	3 (<0.1%)	7 (0.3%)
Understanding purpose		
Mean	8.4	8.3
Standard Deviation	2.0	2.1
Min, Max	0.0, 10.0	0.0, 10.0
(Missing)	6 (0.1%)	4 (0.2%)
Self-rated mental health		
Mean	8.0	7.9
Standard Deviation	2.0	2.0
Min, Max	0.0, 10.0	0.0, 10.0

Table S23b. Weighted summary statistics for outcome variables in Philippines

**Outcome**	**Wave 1**<br>N = 5,292	**Wave 2**<br>N = 2,684
(Missing)	6 (0.1%)	5 (0.2%)
Traumatic distress, n (%)		
A lot	781 (14.8%)	365 (13.6%)
Some	1,674 (31.6%)	888 (33.1%)
Not very much	1,966 (37.2%)	975 (36.3%)
Not at all	866 (16.4%)	456 (17.0%)
(Missing)	4 (0.1%)	1 (0.0%)
Depression symptoms composite, n (%)	2,697 (51.1%)	1,273 (47.6%)
(Missing)	15 (0.3%)	8 (0.3%)
Depression – feel hopeless, n (%)		
Nearly every day	591 (11.2%)	319 (11.9%)
More than half the days	1,314 (24.8%)	566 (21.1%)
Several days	1,428 (27.0%)	765 (28.5%)
Not at all	1,949 (36.8%)	1,030 (38.4%)
(Missing)	9 (0.2%)	4 (0.2%)
Depression – loss of interest, n (%)		
Nearly every day	1,036 (19.6%)	534 (19.9%)
More than half the days	1,580 (29.9%)	760 (28.3%)
Several days	1,436 (27.1%)	781 (29.1%)
Not at all	1,233 (23.3%)	605 (22.5%)
(Missing)	7 (0.1%)	6 (0.2%)
Anxiety symptoms composite, n (%)	2,568 (48.7%)	1,265 (47.2%)
(Missing)	20 (0.4%)	2 (<0.1%)
Anxiety – feel on edge, n (%)		
Nearly every day	633 (12.0%)	333 (12.4%)
More than half the days	1,249 (23.6%)	558 (20.8%)
Several days	1,333 (25.2%)	704 (26.2%)
Not at all	2,067 (39.1%)	1,090 (40.6%)
(Missing)	10 (0.2%)	0 (0%)
Anxiety – cannot stop worrying, n (%)		
Nearly every day	1,334 (25.2%)	717 (26.7%)
More than half the days	1,177 (22.2%)	509 (19.0%)
Several days	1,344 (25.4%)	687 (25.6%)
Not at all	1,423 (26.9%)	770 (28.7%)
(Missing)	13 (0.3%)	2 (0.1%)
Suffering, n (%)		
A lot	700 (13.2%)	427 (15.9%)
Some	2,433 (46.0%)	1,185 (44.2%)
Not very much	1,893 (35.8%)	948 (35.3%)
Not at all	256 (4.8%)	123 (4.6%)
(Missing)	9 (0.2%)	1 (0.0%)
Relationship contentment		
Mean	8.7	8.6
Standard Deviation	2.0	2.0
Min, Max	0.0, 10.0	0.0, 10.0
(Missing)	8 (0.1%)	5 (0.2%)
Relationship satisfaction		
Mean	8.5	8.4
Standard Deviation	2.0	2.0
Min, Max	0.0, 10.0	0.0, 10.0
(Missing)	14 (0.3%)	6 (0.2%)

Table S23b. Weighted summary statistics for outcome variables in Philippines

Outcome	Wave 1<br>N = 5,292	Wave 2<br>N = 2,684
Social support		
Mean	8.1	7.8
Standard Deviation	2.3	2.4
Min, Max	0.0, 10.0	0.0, 10.0
(Missing)	5 (0.1%)	2 (<0.1%)
Intimate/close friend, n (%)		
Yes	4,756 (89.9%)	2,331 (86.8%)
No	528 (10.0%)	343 (12.8%)
(Missing)	8 (0.1%)	11 (0.4%)
Government approval, n (%)		
Strongly approve	1,554 (29.4%)	668 (24.9%)
Somewhat approve	2,136 (40.4%)	1,058 (39.4%)
Neither approve nor disapprove	762 (14.4%)	423 (15.8%)
Somewhat disapprove	497 (9.4%)	321 (12.0%)
Strongly disapprove	329 (6.2%)	197 (7.4%)
(Missing)	14 (0.3%)	17 (0.6%)
Say in government, n (%)		
Agree	2,418 (45.7%)	1,296 (48.3%)
Disagree	400 (7.6%)	157 (5.8%)
Unsure	2,459 (46.5%)	1,224 (45.6%)
(Missing)	15 (0.3%)	8 (0.3%)
Belonging in country		
Mean	8.1	8.0
Standard Deviation	2.2	2.1
Min, Max	0.0, 10.0	0.0, 10.0
(Missing)	20 (0.4%)	16 (0.6%)
City/place satisfaction, n (%)		
Satisfied	4,562 (86.2%)	2,124 (79.1%)
Dissatisfied	176 (3.3%)	125 (4.7%)
Unsure	553 (10.5%)	428 (15.9%)
(Missing)	1 (0.0%)	8 (0.3%)
Trust within country, n (%)		
All people	497 (9.4%)	211 (7.9%)
Most people	2,078 (39.3%)	1,094 (40.8%)
Some people	1,914 (36.2%)	937 (34.9%)
Not very many people	686 (13.0%)	385 (14.3%)
None	108 (2.0%)	54 (2.0%)
(Missing)	9 (0.2%)	3 (0.1%)
Number of children		
Mean	1.8	2.1
Standard Deviation	1.6	3.4
Min, Max	0.0, 33.0	0.0, 97.0
(Missing)	0 (0%)	81 (3.0%)
Community participation, n (%)		
More than once a week	291 (5.5%)	116 (4.3%)
Once a week	701 (13.2%)	341 (12.7%)
One to three times a month	886 (16.7%)	481 (17.9%)
A few times a year	1,486 (28.1%)	748 (27.9%)
Never	1,923 (36.3%)	995 (37.1%)
(Missing)	6 (0.1%)	2 (0.1%)
Religious attendance, n (%)		

Table S23b. Weighted summary statistics for outcome variables in Philippines

**Outcome**	**Wave 1**<br>N = 5,292	**Wave 2**<br>N = 2,684
More than once a week	842 (15.9%)	395 (14.7%)
Once a week	1,928 (36.4%)	1,045 (38.9%)
One to three times a month	1,368 (25.9%)	659 (24.6%)
A few times a year	930 (17.6%)	451 (16.8%)
Never	218 (4.1%)	129 (4.8%)
(Missing)	6 (0.1%)	6 (0.2%)
Loneliness		
Mean	3.5	3.7
Standard Deviation	3.2	3.1
Min, Max	0.0, 10.0	0.0, 10.0
(Missing)	3 (<0.1%)	7 (0.3%)
Perceived discrimination, n (%)		
Always	513 (9.7%)	286 (10.6%)
Often	877 (16.6%)	403 (15.0%)
Rarely	2,607 (49.3%)	1,360 (50.7%)
Never	1,288 (24.3%)	635 (23.7%)
(Missing)	7 (0.1%)	0 (0.0%)
Orientation to promote good		
Mean	8.5	8.5
Standard Deviation	2.0	1.8
Min, Max	0.0, 10.0	0.0, 10.0
(Missing)	2 (<0.1%)	5 (0.2%)
Delayed gratification		
Mean	8.4	8.3
Standard Deviation	2.0	1.9
Min, Max	0.0, 10.0	0.0, 10.0
(Missing)	9 (0.2%)	6 (0.2%)
Hope		
Mean	8.9	8.9
Standard Deviation	1.8	1.8
Min, Max	0.0, 10.0	0.0, 10.0
(Missing)	8 (0.1%)	1 (<0.1%)
Gratitude		
Mean	8.6	8.6
Standard Deviation	2.0	1.9
Min, Max	0.0, 10.0	0.0, 10.0
(Missing)	9 (0.2%)	12 (0.5%)
Showing love/care		
Mean	9.0	9.0
Standard Deviation	1.7	1.6
Min, Max	0.0, 10.0	0.0, 10.0
(Missing)	8 (0.1%)	8 (0.3%)
Forgivingness, n (%)		
Always	2,524 (47.7%)	1,345 (50.1%)
Often	1,405 (26.6%)	728 (27.1%)
Rarely	1,134 (21.4%)	538 (20.0%)
Never	227 (4.3%)	73 (2.7%)
(Missing)	2 (0.0%)	0 (0.0%)
Charitable giving, n (%)		
Yes	720 (13.6%)	778 (29.0%)
No	4,568 (86.3%)	1,895 (70.6%)

Table S23b. Weighted summary statistics for outcome variables in Philippines

Outcome	Wave 1<br>N = 5,292	Wave 2<br>N = 2,684
(Missing)	4 (0.1%)	12 (0.4%)
Helping strangers, n (%)		
Yes	3,340 (63.1%)	1,906 (71.0%)
No	1,929 (36.5%)	768 (28.6%)
(Missing)	23 (0.4%)	11 (0.4%)
Volunteering, n (%)		
Yes	1,330 (25.1%)	810 (30.2%)
No	3,960 (74.8%)	1,865 (69.5%)
(Missing)	2 (0.0%)	9 (0.3%)
Self-rated physical health		
Mean	7.7	7.6
Standard Deviation	2.1	2.1
Min, Max	0.0, 10.0	0.0, 10.0
(Missing)	5 (<0.1%)	4 (0.2%)
Health problems, n (%)		
Yes	1,797 (34.0%)	950 (35.4%)
No	3,478 (65.7%)	1,730 (64.4%)
(Missing)	17 (0.3%)	5 (0.2%)
Pain in past 4 weeks, n (%)		
A lot	582 (11.0%)	367 (13.7%)
Some	1,699 (32.1%)	905 (33.7%)
Not very much	2,251 (42.5%)	1,051 (39.1%)
None at all	756 (14.3%)	361 (13.5%)
(Missing)	4 (0.1%)	1 (0.0%)
Number of cigarettes per day		
Mean	1.7	1.7
Standard Deviation	4.5	4.5
Min, Max	0.0, 60.0	0.0, 97.0
(Missing)	84 (1.6%)	19 (0.7%)
Number of drinks per week		
Mean	1.0	0.7
Standard Deviation	4.1	3.1
Min, Max	0.0, 97.0	0.0, 90.0
(Missing)	100 (1.9%)	18 (0.7%)
Days exercise per week		
Mean	3.8	4.0
Standard Deviation	2.8	2.8
Min, Max	0.0, 7.0	0.0, 7.0
(Missing)	8 (0.1%)	1 (<0.1%)
Financial security		
Mean	5.3	5.4
Standard Deviation	3.3	3.2
Min, Max	0.0, 10.0	0.0, 10.0
(Missing)	2 (<0.1%)	0 (<0.1%)
Material security		
Mean	6.0	6.0
Standard Deviation	3.3	3.2
Min, Max	0.0, 10.0	0.0, 10.0
(Missing)	10 (0.2%)	6 (0.2%)
Educational attainment (16+ years), n (%)		
Up to 8	1,240 (23.4%)	555 (20.7%)

Table S23b. Weighted summary statistics for outcome variables in Philippines

Outcome	Wave 1<br>N = 5,292	Wave 2<br>N = 2,684
9-15	3,702 (70.0%)	1,784 (66.5%)
16+	349 (6.6%)	345 (12.8%)
(Missing)	1 (0.0%)	0 (0%)
Currently employed, n (%)		
Employed for an employer	1,331 (25.1%)	823 (30.6%)
Self-employed	1,334 (25.2%)	624 (23.3%)
Retired	146 (2.7%)	78 (2.9%)
Student	650 (12.3%)	209 (7.8%)
Homemaker	1,057 (20.0%)	450 (16.8%)
Unemployed and looking for a job	664 (12.6%)	401 (14.9%)
None of these/Other	111 (2.1%)	92 (3.4%)
(Missing)	0 (0%)	7 (0.3%)
Financially comfortable/getting by, n (%)		
Living comfortably on present income	422 (8.0%)	225 (8.4%)
Getting by on present income	2,149 (40.6%)	1,143 (42.6%)
Finding it difficult on present income	1,956 (37.0%)	955 (35.6%)
Finding it very difficult on present income	748 (14.1%)	358 (13.3%)
(Missing)	18 (0.3%)	4 (0.1%)
Own home, n (%)		
Someone in this household owns this home	2,830 (53.5%)	1,651 (61.5%)
Someone in this household rents this home	700 (13.2%)	373 (13.9%)
Both	419 (7.9%)	121 (4.5%)
Neither	1,332 (25.2%)	526 (19.6%)
Rent	0 (0%)	0 (0%)
Own	0 (0%)	0 (0%)
Something else	0 (0%)	0 (0%)
(Missing)	10 (0.2%)	13 (0.5%)
Religious/spiritual connection, n (%)		
Always	2,083 (39.4%)	1,120 (41.7%)
Often	1,291 (24.4%)	647 (24.1%)
Rarely	1,561 (29.5%)	740 (27.6%)
Never	355 (6.7%)	178 (6.6%)
(Missing)	1 (0.0%)	0 (0%)
Belief in life after death, n (%)		
Yes	2,819 (53.3%)	1,418 (52.8%)
No	446 (8.4%)	154 (5.7%)
Unsure	2,014 (38.1%)	1,103 (41.1%)
(Missing)	13 (0.3%)	10 (0.4%)
Transformative religious experience, n (%)		
Yes	3,265 (61.7%)	1,643 (61.2%)
No	2,017 (38.1%)	1,032 (38.4%)
(Missing)	10 (0.2%)	9 (0.3%)
Religious reading or listening, n (%)		
More than once a day	580 (11.0%)	288 (10.7%)
About once a day	767 (14.5%)	408 (15.2%)
Sometimes	3,592 (67.9%)	1,845 (68.7%)
Never	336 (6.3%)	134 (5.0%)
(Missing)	18 (0.3%)	9 (0.3%)
Prayer or meditation, n (%)		
More than once a day	1,946 (36.8%)	979 (36.5%)
About once a day	1,774 (33.5%)	937 (34.9%)

Table S23b. Weighted summary statistics for outcome variables in Philippines

**Outcome**	**Wave 1**<br>N = 5,292	**Wave 2**<br>N = 2,684
Sometimes	1,453 (27.5%)	704 (26.2%)
Never	95 (1.8%)	59 (2.2%)
(Missing)	24 (0.5%)	4 (0.2%)
Belief in God/gods/spiritual forces, n (%)		
One God	4,529 (85.6%)	2,271 (84.6%)
More than one god	276 (5.2%)	146 (5.4%)
An impersonal spiritual force	85 (1.6%)	45 (1.7%)
None of these	77 (1.5%)	17 (0.6%)
Unsure	318 (6.0%)	200 (7.5%)
(Missing)	7 (0.1%)	6 (0.2%)
Religious centrality, n (%)		
Agree	3,892 (73.5%)	2,014 (75.0%)
Disagree	290 (5.5%)	94 (3.5%)
Not relevant	186 (3.5%)	75 (2.8%)
Unsure	916 (17.3%)	495 (18.4%)
(Missing)	8 (0.2%)	7 (0.3%)
Religious/spiritual comfort, n (%)		
Agree	4,212 (79.6%)	2,165 (80.7%)
Disagree	273 (5.2%)	103 (3.8%)
Not relevant	188 (3.6%)	58 (2.2%)
Unsure	614 (11.6%)	355 (13.2%)
(Missing)	4 (0.1%)	3 (0.1%)
Feel loved by God, n (%)		
Agree	4,780 (90.3%)	2,477 (92.3%)
Disagree	150 (2.8%)	28 (1.0%)
Not relevant	86 (1.6%)	31 (1.2%)
Unsure	270 (5.1%)	139 (5.2%)
(Missing)	6 (0.1%)	9 (0.3%)
Feel punished by God, n (%)		
Agree	2,378 (44.9%)	1,254 (46.7%)
Disagree	1,290 (24.4%)	611 (22.8%)
Not relevant	355 (6.7%)	124 (4.6%)
Unsure	1,258 (23.8%)	692 (25.8%)
(Missing)	12 (0.2%)	4 (0.1%)
Experienced religious criticism, n (%)		
Agree	1,802 (34.1%)	1,006 (37.5%)
Disagree	1,353 (25.6%)	523 (19.5%)
Not relevant	475 (9.0%)	182 (6.8%)
Unsure	1,652 (31.2%)	971 (36.2%)
(Missing)	10 (0.2%)	4 (0.1%)
Faith-sharing, n (%)		
Agree	2,944 (55.6%)	1,477 (55.0%)
Disagree	781 (14.8%)	331 (12.3%)
Not relevant	430 (8.1%)	214 (8.0%)
Unsure	1,132 (21.4%)	657 (24.5%)
(Missing)	5 (0.1%)	5 (0.2%)

Note. N (%); this table is based on non-imputed data. Cumulative percentages for variables may not add up to 100% due to rounding. Wave 1 characteristics weighted using the Gallup provided sampling weight, ANNUAL_WEIGHT_R2; Wave 2 characteristics weighted accounting for attrition by using the adjusted Wave 1 weight, ANNUAL_WEIGHT_R2, multiplied by the created attrition weight to account for dropout, to maintain nationally representative estimates for Wave 2 characteristics.

Table S23c. Unweighted summary statistics for demographic and childhood variables in Philippines by retention status

Characteristic	Attritors–Not Observed in Wave 2<br>N = 2,612	Retained–Observed in Wave 2<br>N = 2,661
Forgivingness, n (%)		
Always	1,224 (46.9%)	1,292 (48.5%)
Often	705 (27.0%)	695 (26.1%)
Rarely	556 (21.3%)	574 (21.6%)
Never	126 (4.8%)	99 (3.7%)
(Missing)	1 (0.0%)	2 (0.1%)
Year of birth, n (%)		
1943 or earlier (current age: 80+ years)	9 (0.4%)	13 (0.5%)
1943-1953 (current age: 70-79 years)	60 (2.3%)	76 (2.8%)
1953-1963 (current age: 60-69 years)	236 (9.0%)	231 (8.7%)
1963-1973 (current age: 50-59 years)	306 (11.7%)	377 (14.2%)
1973-1983 (current age: 40-49 years)	448 (17.2%)	476 (17.9%)
1983-1993 (current age: 30-39 years)	540 (20.7%)	611 (23.0%)
1993-1998 (current age: 25-29 years)	369 (14.1%)	324 (12.2%)
1998-2005 (current age: 18-24 years)	644 (24.7%)	554 (20.8%)
(Missing)	0 (0%)	0 (0%)
Age of participant		
Mean	37.7	39.2
Standard Deviation	15.3	15.3
Min, Max	18.0, 85.0	18.0, 89.0
Gender, n (%)		
Male	1,300 (49.8%)	1,290 (48.5%)
Female	1,299 (49.7%)	1,360 (51.1%)
Other	8 (0.3%)	5 (0.2%)
(Missing)	5 (0.2%)	6 (0.2%)
Respondent marital status, n (%)		
Single/Never been married	629 (24.1%)	626 (23.5%)
Married	1,125 (43.1%)	1,187 (44.6%)
Separated	106 (4.1%)	134 (5.0%)
Divorced	5 (0.2%)	4 (0.1%)
Widowed	134 (5.1%)	128 (4.8%)
Domestic partner	604 (23.1%)	573 (21.5%)
(Missing)	8 (0.3%)	9 (0.3%)
Education (years), n (%)		
Up to 8	617 (23.6%)	619 (23.3%)
9-15	1,832 (70.1%)	1,857 (69.8%)
16+	163 (6.2%)	185 (6.9%)
(Missing)	0 (0%)	1 (0.0%)
Employment status, n (%)		
Employed for an employer	642 (24.6%)	685 (25.7%)
Self-employed	667 (25.5%)	661 (24.8%)
Retired	61 (2.3%)	85 (3.2%)
Student	331 (12.7%)	316 (11.9%)
Homemaker	522 (20.0%)	531 (20.0%)
Unemployed and looking for a job	336 (12.9%)	325 (12.2%)
None of these/Other	52 (2.0%)	59 (2.2%)
(Missing)	0 (0%)	0 (0%)
Current religious service attendance, n (%)		
More than once a week	404 (15.5%)	435 (16.4%)

Table S23c. Unweighted summary statistics for demographic and childhood variables in Philippines by retention status

**Characteristic**	**Attritors–Not Observed in Wave 2**<br>N = 2,612	**Retained–Observed in Wave 2**<br>N = 2,661
Once a week	941 (36.0%)	980 (36.8%)
One to three times a month	694 (26.6%)	668 (25.1%)
A few times a year	470 (18.0%)	456 (17.1%)
Never	99 (3.8%)	118 (4.4%)
(Missing)	2 (0.1%)	4 (0.2%)
Immigration status, n (%)		
Born in this country	2,607 (99.8%)	2,658 (99.9%)
Born in another country	5 (0.2%)	3 (0.1%)
(Missing)	0 (0%)	0 (0%)
Parental marital status around age 12, n (%)		
Parents were married	2,182 (83.5%)	2,342 (88.0%)
Parents were divorced	38 (1.5%)	26 (1.0%)
Parents were never married	325 (12.4%)	222 (8.4%)
One or both of them had died	23 (0.9%)	27 (1.0%)
Unsure	25 (0.9%)	32 (1.2%)
(Missing)	20 (0.8%)	12 (0.4%)
Religious service attendance around age 12, n (%)		
At least once a week	1,165 (44.6%)	1,264 (47.5%)
One to three times a month	870 (33.3%)	824 (31.0%)
Less than once a month	442 (16.9%)	457 (17.2%)
Never	105 (4.0%)	99 (3.7%)
(Missing)	31 (1.2%)	18 (0.7%)
Relationship with mother when growing up, n (%)		
Very good	1,622 (62.1%)	1,670 (62.8%)
Somewhat good	875 (33.5%)	845 (31.8%)
Somewhat bad	60 (2.3%)	69 (2.6%)
Very bad	16 (0.6%)	25 (1.0%)
(Does not apply)	26 (1.0%)	30 (1.1%)
(Missing)	13 (0.5%)	21 (0.8%)
Relationship with father when growing up, n (%)		
Very good	1,660 (63.6%)	1,744 (65.5%)
Somewhat good	742 (28.4%)	706 (26.5%)
Somewhat bad	78 (3.0%)	81 (3.0%)
Very bad	27 (1.0%)	34 (1.3%)
(Does not apply)	53 (2.0%)	54 (2.0%)
(Missing)	53 (2.0%)	43 (1.6%)
Felt like an outsider in family when growing up, n (%)		
Yes	210 (8.0%)	187 (7.0%)
No	2,392 (91.6%)	2,471 (92.9%)
(Missing)	9 (0.4%)	3 (0.1%)
Experienced abuse when growing up, n (%)		
Yes	212 (8.1%)	215 (8.1%)
No	2,369 (90.7%)	2,444 (91.9%)
(Missing)	31 (1.2%)	2 (0.1%)
Self-rated health when growing up, n (%)		
Excellent	557 (21.3%)	476 (17.9%)
Very good	283 (10.8%)	275 (10.3%)

Table S23c. Unweighted summary statistics for demographic and childhood variables in Philippines by retention status

**Characteristic**	**Attritors–Not Observed in Wave 2** N = 2,612	**Retained–Observed in Wave 2** N = 2,661
Good	1,052 (40.3%)	1,120 (42.1%)
Fair	587 (22.5%)	653 (24.5%)
Poor	132 (5.1%)	138 (5.2%)
(Missing)	0 (0.0%)	0 (0%)
Subjective financial status of family growing up, n (%)		
Lived comfortably	498 (19.0%)	433 (16.3%)
Got by	1,491 (57.1%)	1,503 (56.5%)
Found it difficult	496 (19.0%)	556 (20.9%)
Found it very difficult	125 (4.8%)	169 (6.3%)
(Missing)	1 (0.0%)	1 (0.0%)
Religious affiliation growing up, n (%)		
Christianity	2,481 (95.0%)	2,472 (92.9%)
Taoism	0 (0%)	0 (0%)
Confucianism	0 (0%)	0 (0%)
Primal, Animist, or Folk religion	9 (0.3%)	4 (0.1%)
Spiritism	0 (0%)	0 (0%)
Umbanda, Candomblé, and other African-derived religions	0 (0%)	0 (0%)
Chinese folk/traditional religion	0 (0%)	0 (0%)
Islam	105 (4.0%)	169 (6.3%)
Hinduism	0 (0%)	0 (0%)
Buddhism	0 (0%)	1 (0.0%)
Judaism	0 (0%)	0 (0%)
Sikhism	0 (0%)	4 (0.1%)
Baha'i	1 (0.0%)	0 (0%)
Jainism	0 (0%)	0 (0%)
Shinto	0 (0%)	0 (0%)
Some other religion	2 (0.1%)	6 (0.2%)
No religion/Atheist/Agnostic	8 (0.3%)	1 (0.1%)
(Missing)	6 (0.2%)	4 (0.2%)

Note. N (%); this table is based on non-imputed data. Cumulative percentages for variables may not add up to 100% due to rounding.

Table S23d. Unweighted summary statistics for Wave 1 outcome variables in Philippines by retention status.

**Outcome**	**Attritors–Not Observed in Wave 2**<br>N = 2,612	**Retained–Observed in Wave 2**<br>N = 2,661
Secure flourishing index		
Mean	7.7	7.7
Standard Deviation	1.4	1.4
Min, Max	0.6, 10.0	1.8, 10.0
(Missing)	24 (0.9%)	28 (1.1%)
Flourishing index		
Mean	8.1	8.1
Standard Deviation	1.5	1.4
Min, Max	0.5, 10.0	2.2, 10.0
(Missing)	18 (0.7%)	25 (0.9%)
Happiness & life satisfaction		
Mean	7.5	7.4
Standard Deviation	2.1	2.1
Min, Max	0.0, 10.0	0.0, 10.0
(Missing)	3 (0.1%)	4 (0.2%)
Physical & mental health		
Mean	7.9	7.9
Standard Deviation	1.8	1.9
Min, Max	0.0, 10.0	0.0, 10.0
(Missing)	5 (0.2%)	3 (0.1%)
Meaning & purpose		
Mean	8.2	8.2
Standard Deviation	1.8	1.9
Min, Max	0.0, 10.0	0.0, 10.0
(Missing)	3 (0.1%)	5 (0.2%)
Character & virtue		
Mean	8.5	8.5
Standard Deviation	1.7	1.8
Min, Max	0.0, 10.0	0.0, 10.0
(Missing)	6 (0.2%)	5 (0.2%)
Close social relationships		
Mean	8.6	8.5
Standard Deviation	1.8	1.9
Min, Max	0.0, 10.0	0.0, 10.0
(Missing)	5 (0.2%)	14 (0.5%)
Financial & material security		
Mean	5.7	5.6
Standard Deviation	3.0	2.9
Min, Max	0.0, 10.0	0.0, 10.0
(Missing)	9 (0.3%)	4 (0.1%)
Happiness		
Mean	7.3	7.3
Standard Deviation	2.4	2.3
Min, Max	0.0, 10.0	0.0, 10.0
(Missing)	2 (<0.1%)	0 (0%)
Life satisfaction		
Mean	7.6	7.4
Standard Deviation	2.4	2.5
Min, Max	0.0, 10.0	0.0, 10.0

Table S23d. Unweighted summary statistics for Wave 1 outcome variables in Philippines by retention status.

**Outcome**	**Attritors–Not Observed in Wave 2** N = 2,612	**Retained–Observed in Wave 2** N = 2,661
(Missing)	1 (<0.1%)	4 (0.2%)
Current life evaluation		
Mean	6.5	6.3
Standard Deviation	2.4	2.4
Min, Max	0.0, 10.0	0.0, 10.0
(Missing)	3 (0.1%)	1 (<0.1%)
Future life evaluation		
Mean	7.6	7.5
Standard Deviation	2.2	2.2
Min, Max	0.0, 10.0	0.0, 10.0
(Missing)	5 (0.2%)	4 (0.2%)
Optimism		
Mean	8.8	8.8
Standard Deviation	2.0	2.0
Min, Max	0.0, 10.0	0.0, 10.0
(Missing)	7 (0.3%)	3 (<0.1%)
Freedom to pursue what's important		
Mean	8.6	8.6
Standard Deviation	2.0	2.0
Min, Max	0.0, 10.0	0.0, 10.0
(Missing)	3 (<0.1%)	5 (0.2%)
Inner peace, n (%)		
Always	617 (23.6%)	554 (20.8%)
Often	724 (27.7%)	757 (28.4%)
Rarely	1,070 (41.0%)	1,078 (40.5%)
Never	199 (7.6%)	271 (10.2%)
(Missing)	1 (0.0%)	2 (0.1%)
Life balance, n (%)		
Always	526 (20.1%)	517 (19.4%)
Often	765 (29.3%)	847 (31.8%)
Rarely	1,105 (42.3%)	1,068 (40.1%)
Never	215 (8.2%)	229 (8.6%)
(Missing)	0 (0%)	0 (0.0%)
Sense of mastery, n (%)		
Always	789 (30.2%)	840 (31.6%)
Often	833 (31.9%)	828 (31.1%)
Rarely	879 (33.7%)	866 (32.5%)
Never	110 (4.2%)	126 (4.7%)
(Missing)	1 (0.0%)	1 (0.0%)
Meaningful activities		
Mean	8.0	8.0
Standard Deviation	2.3	2.4
Min, Max	0.0, 10.0	0.0, 10.0
(Missing)	1 (<0.1%)	2 (<0.1%)
Understanding purpose		
Mean	8.5	8.4
Standard Deviation	2.0	2.1
Min, Max	0.0, 10.0	0.0, 10.0
(Missing)	2 (<0.1%)	4 (0.1%)

Table S23d. Unweighted summary statistics for Wave 1 outcome variables in Philippines by retention status.

**Outcome**	**Attritors–Not Observed in Wave 2**<br>N = 2,612	**Retained–Observed in Wave 2**<br>N = 2,661
Self-rated mental health		
Mean	8.0	8.1
Standard Deviation	2.0	2.0
Min, Max	0.0, 10.0	0.0, 10.0
(Missing)	5 (0.2%)	1 (<0.1%)
Traumatic distress, n (%)		
A lot	403 (15.4%)	375 (14.1%)
Some	804 (30.8%)	865 (32.5%)
Not very much	967 (37.0%)	992 (37.3%)
Not at all	435 (16.7%)	428 (16.1%)
(Missing)	3 (0.1%)	1 (0.0%)
Depression symptoms composite, n (%)	1,334 (51.3%)	1,353 (50.9%)
(Missing)	10 (0.4%)	5 (0.2%)
Depression – feel hopeless, n (%)		
Nearly every day	309 (11.8%)	280 (10.5%)
More than half the days	647 (24.8%)	663 (24.9%)
Several days	684 (26.2%)	740 (27.8%)
Not at all	967 (37.0%)	975 (36.6%)
(Missing)	6 (0.2%)	4 (0.1%)
Depression – loss of interest, n (%)		
Nearly every day	522 (20.0%)	510 (19.2%)
More than half the days	781 (29.9%)	793 (29.8%)
Several days	697 (26.7%)	734 (27.6%)
Not at all	607 (23.2%)	622 (23.4%)
(Missing)	5 (0.2%)	2 (0.1%)
Anxiety symptoms composite, n (%)	1,267 (48.7%)	1,292 (48.8%)
(Missing)	9 (0.3%)	11 (0.4%)
Anxiety – feel on edge, n (%)		
Nearly every day	303 (11.6%)	328 (12.3%)
More than half the days	629 (24.1%)	615 (23.1%)
Several days	626 (24.0%)	704 (26.5%)
Not at all	1,047 (40.1%)	1,011 (38.0%)
(Missing)	7 (0.3%)	3 (0.1%)
Anxiety – cannot stop worrying, n (%)		
Nearly every day	627 (24.0%)	703 (26.4%)
More than half the days	585 (22.4%)	588 (22.1%)
Several days	669 (25.6%)	670 (25.2%)
Not at all	728 (27.9%)	689 (25.9%)
(Missing)	3 (0.1%)	11 (0.4%)
Suffering, n (%)		
A lot	340 (13.0%)	358 (13.5%)
Some	1,198 (45.9%)	1,227 (46.1%)
Not very much	937 (35.9%)	949 (35.7%)
Not at all	130 (5.0%)	126 (4.7%)
(Missing)	7 (0.3%)	1 (0.1%)
Relationship contentment		
Mean	8.7	8.6
Standard Deviation	2.0	2.1
Min, Max	0.0, 10.0	0.0, 10.0

Table S23d. Unweighted summary statistics for Wave 1 outcome variables in Philippines by retention status.

Outcome	Attritors–Not Observed in Wave 2<br>N = 2,612	Retained–Observed in Wave 2<br>N = 2,661
(Missing)	3 (0.1%)	5 (0.2%)
Relationship satisfaction		
Mean	8.5	8.4
Standard Deviation	2.0	2.1
Min, Max	0.0, 10.0	0.0, 10.0
(Missing)	3 (0.1%)	11 (0.4%)
Social support		
Mean	8.2	8.1
Standard Deviation	2.3	2.3
Min, Max	0.0, 10.0	0.0, 10.0
(Missing)	4 (0.1%)	2 (<0.1%)
Intimate/close friend, n (%)		
Yes	2,337 (89.5%)	2,403 (90.3%)
No	269 (10.3%)	257 (9.7%)
(Missing)	6 (0.2%)	1 (0.0%)
Government approval, n (%)		
Strongly approve	779 (29.8%)	769 (28.9%)
Somewhat approve	1,029 (39.4%)	1,100 (41.3%)
Neither approve nor disapprove	401 (15.4%)	357 (13.4%)
Somewhat disapprove	240 (9.2%)	255 (9.6%)
Strongly disapprove	156 (6.0%)	173 (6.5%)
(Missing)	7 (0.3%)	7 (0.3%)
Say in government, n (%)		
Agree	1,191 (45.6%)	1,218 (45.8%)
Disagree	205 (7.8%)	193 (7.3%)
Unsure	1,206 (46.2%)	1,245 (46.8%)
(Missing)	10 (0.4%)	5 (0.2%)
Belonging in country		
Mean	8.1	8.2
Standard Deviation	2.2	2.2
Min, Max	0.0, 10.0	0.0, 10.0
(Missing)	16 (0.6%)	4 (0.1%)
City/place satisfaction, n (%)		
Satisfied	2,247 (86.0%)	2,299 (86.4%)
Dissatisfied	89 (3.4%)	86 (3.2%)
Unsure	277 (10.6%)	275 (10.3%)
(Missing)	0 (0%)	1 (0.0%)
Trust within country, n (%)		
All people	250 (9.6%)	245 (9.2%)
Most people	1,006 (38.5%)	1,065 (40.0%)
Some people	946 (36.2%)	961 (36.1%)
Not very many people	336 (12.8%)	348 (13.1%)
None	66 (2.5%)	41 (1.5%)
(Missing)	8 (0.3%)	0 (0.0%)
Number of children		
Mean	1.8	1.9
Standard Deviation	1.6	1.6
Min, Max	0.0, 33.0	0.0, 28.0
Community participation, n (%)		

Table S23d. Unweighted summary statistics for Wave 1 outcome variables in Philippines by retention status.

**Outcome**	**Attritors–Not Observed in Wave 2** N = 2,612	**Retained–Observed in Wave 2** N = 2,661
More than once a week	147 (5.6%)	143 (5.4%)
Once a week	352 (13.5%)	346 (13.0%)
One to three times a month	450 (17.2%)	432 (16.2%)
A few times a year	723 (27.7%)	758 (28.5%)
Never	938 (35.9%)	979 (36.8%)
(Missing)	2 (0.1%)	4 (0.2%)
Religious attendance, n (%)		
More than once a week	404 (15.5%)	435 (16.4%)
Once a week	941 (36.0%)	980 (36.8%)
One to three times a month	694 (26.6%)	668 (25.1%)
A few times a year	470 (18.0%)	456 (17.1%)
Never	99 (3.8%)	118 (4.4%)
(Missing)	2 (0.1%)	4 (0.2%)
Loneliness		
Mean	3.6	3.5
Standard Deviation	3.2	3.2
Min, Max	0.0, 10.0	0.0, 10.0
(Missing)	2 (<0.1%)	1 (<0.1%)
Perceived discrimination, n (%)		
Always	253 (9.7%)	258 (9.7%)
Often	429 (16.4%)	446 (16.7%)
Rarely	1,274 (48.8%)	1,324 (49.8%)
Never	653 (25.0%)	630 (23.7%)
(Missing)	3 (0.1%)	4 (0.2%)
Orientation to promote good		
Mean	8.5	8.5
Standard Deviation	1.9	2.0
Min, Max	0.0, 10.0	0.0, 10.0
(Missing)	1 (<0.1%)	1 (<0.1%)
Delayed gratification		
Mean	8.4	8.5
Standard Deviation	2.0	2.0
Min, Max	0.0, 10.0	0.0, 10.0
(Missing)	4 (0.2%)	5 (0.2%)
Hope		
Mean	8.8	9.0
Standard Deviation	1.9	1.8
Min, Max	0.0, 10.0	0.0, 10.0
(Missing)	7 (0.3%)	1 (<0.1%)
Gratitude		
Mean	8.6	8.7
Standard Deviation	2.1	1.9
Min, Max	0.0, 10.0	0.0, 10.0
(Missing)	7 (0.3%)	2 (<0.1%)
Showing love/care		
Mean	9.0	9.1
Standard Deviation	1.8	1.6
Min, Max	0.0, 10.0	0.0, 10.0
(Missing)	5 (0.2%)	3 (0.1%)

Table S23d. Unweighted summary statistics for Wave 1 outcome variables in Philippines by retention status.

Outcome	Attritors–Not Observed in Wave 2<br>N = 2,612	Retained–Observed in Wave 2<br>N = 2,661
Forgivingness, n (%)		
Always	1,224 (46.9%)	1,292 (48.5%)
Often	705 (27.0%)	695 (26.1%)
Rarely	556 (21.3%)	574 (21.6%)
Never	126 (4.8%)	99 (3.7%)
(Missing)	1 (0.0%)	2 (0.1%)
Charitable giving, n (%)		
Yes	341 (13.1%)	376 (14.1%)
No	2,269 (86.9%)	2,282 (85.8%)
(Missing)	1 (0.0%)	3 (0.1%)
Helping strangers, n (%)		
Yes	1,661 (63.6%)	1,666 (62.6%)
No	938 (35.9%)	985 (37.0%)
(Missing)	12 (0.5%)	10 (0.4%)
Volunteering, n (%)		
Yes	657 (25.2%)	668 (25.1%)
No	1,954 (74.8%)	1,992 (74.8%)
(Missing)	0 (0.0%)	2 (0.1%)
Self-rated physical health		
Mean	7.7	7.7
Standard Deviation	2.1	2.2
Min, Max	0.0, 10.0	0.0, 10.0
(Missing)	1 (<0.1%)	3 (0.1%)
Health problems, n (%)		
Yes	843 (32.3%)	949 (35.7%)
No	1,754 (67.2%)	1,710 (64.2%)
(Missing)	14 (0.5%)	2 (0.1%)
Pain in past 4 weeks, n (%)		
A lot	266 (10.2%)	315 (11.8%)
Some	871 (33.3%)	821 (30.8%)
Not very much	1,112 (42.6%)	1,131 (42.5%)
None at all	360 (13.8%)	394 (14.8%)
(Missing)	2 (0.1%)	2 (0.1%)
Number of cigarettes per day		
Mean	1.8	1.6
Standard Deviation	4.6	4.5
Min, Max	0.0, 40.0	0.0, 60.0
(Missing)	44 (1.7%)	39 (1.5%)
Number of drinks per week		
Mean	1.1	0.8
Standard Deviation	4.6	3.6
Min, Max	0.0, 97.0	0.0, 97.0
(Missing)	50 (1.9%)	50 (1.9%)
Days exercise per week		
Mean	3.8	3.8
Standard Deviation	2.8	2.8
Min, Max	0.0, 7.0	0.0, 7.0
(Missing)	5 (0.2%)	3 (0.1%)
Financial security		

Table S23d. Unweighted summary statistics for Wave 1 outcome variables in Philippines by retention status.

Outcome	Attritors–Not Observed in Wave 2<br>N = 2,612	Retained–Observed in Wave 2<br>N = 2,661
Mean	5.4	5.3
Standard Deviation	3.3	3.3
Min, Max	0.0, 10.0	0.0, 10.0
(Missing)	1 (<0.1%)	1 (<0.1%)
Material security		
Mean	6.0	5.9
Standard Deviation	3.3	3.3
Min, Max	0.0, 10.0	0.0, 10.0
(Missing)	7 (0.3%)	3 (0.1%)
Educational attainment (16+ years), n (%)		
Up to 8	617 (23.6%)	619 (23.3%)
9-15	1,832 (70.1%)	1,857 (69.8%)
16+	163 (6.2%)	185 (6.9%)
(Missing)	0 (0%)	1 (0.0%)
Currently employed, n (%)		
Employed for an employer	642 (24.6%)	685 (25.7%)
Self-employed	667 (25.5%)	661 (24.8%)
Retired	61 (2.3%)	85 (3.2%)
Student	331 (12.7%)	316 (11.9%)
Homemaker	522 (20.0%)	531 (20.0%)
Unemployed and looking for a job	336 (12.9%)	325 (12.2%)
None of these/Other	52 (2.0%)	59 (2.2%)
(Missing)	0 (0%)	0 (0%)
Financially comfortable/getting by, n (%)		
Living comfortably on present income	221 (8.5%)	198 (7.4%)
Getting by on present income	1,066 (40.8%)	1,075 (40.4%)
Finding it difficult on present income	927 (35.5%)	1,023 (38.4%)
Finding it very difficult on present income	383 (14.7%)	361 (13.6%)
(Missing)	14 (0.5%)	3 (0.1%)
Own home, n (%)		
Someone in this household owns this home	1,393 (53.4%)	1,427 (53.6%)
Someone in this household rents this home	366 (14.0%)	330 (12.4%)
Both	234 (9.0%)	182 (6.9%)
Neither	610 (23.4%)	719 (27.0%)
Rent	0 (0%)	0 (0%)
Own	0 (0%)	0 (0%)
Something else	0 (0%)	0 (0%)
(Missing)	8 (0.3%)	2 (0.1%)
Religious/spiritual connection, n (%)		
Always	988 (37.8%)	1,090 (40.9%)
Often	638 (24.4%)	648 (24.4%)
Rarely	803 (30.7%)	751 (28.2%)
Never	182 (7.0%)	171 (6.4%)
(Missing)	1 (0.0%)	1 (0.0%)
Belief in life after death, n (%)		
Yes	1,363 (52.2%)	1,446 (54.4%)
No	244 (9.4%)	199 (7.5%)
Unsure	993 (38.0%)	1,014 (38.1%)
(Missing)	11 (0.4%)	2 (0.1%)

Table S23d. Unweighted summary statistics for Wave 1 outcome variables in Philippines by retention status.

Outcome	Attritors–Not Observed in Wave 2 N = 2,612	Retained–Observed in Wave 2 N = 2,661
Transformative religious experience, n (%)		
Yes	1,604 (61.4%)	1,650 (62.0%)
No	1,000 (38.3%)	1,009 (37.9%)
(Missing)	8 (0.3%)	2 (0.1%)
Religious reading or listening, n (%)		
More than once a day	294 (11.3%)	284 (10.7%)
About once a day	338 (12.9%)	428 (16.1%)
Sometimes	1,782 (68.2%)	1,797 (67.5%)
Never	184 (7.1%)	149 (5.6%)
(Missing)	14 (0.5%)	3 (0.1%)
Prayer or meditation, n (%)		
More than once a day	963 (36.9%)	977 (36.7%)
About once a day	868 (33.2%)	900 (33.8%)
Sometimes	710 (27.2%)	738 (27.7%)
Never	56 (2.1%)	39 (1.5%)
(Missing)	16 (0.6%)	8 (0.3%)
Belief in God/gods/spiritual forces, n (%)		
One God	2,224 (85.1%)	2,290 (86.0%)
More than one god	140 (5.4%)	135 (5.1%)
An impersonal spiritual force	40 (1.5%)	44 (1.7%)
None of these	41 (1.6%)	36 (1.3%)
Unsure	162 (6.2%)	155 (5.8%)
(Missing)	5 (0.2%)	2 (0.1%)
Religious centrality, n (%)		
Agree	1,877 (71.9%)	2,003 (75.3%)
Disagree	167 (6.4%)	121 (4.6%)
Not relevant	102 (3.9%)	83 (3.1%)
Unsure	460 (17.6%)	452 (17.0%)
(Missing)	6 (0.2%)	2 (0.1%)
Religious/spiritual comfort, n (%)		
Agree	2,065 (79.1%)	2,132 (80.1%)
Disagree	142 (5.4%)	129 (4.9%)
Not relevant	102 (3.9%)	85 (3.2%)
Unsure	298 (11.4%)	314 (11.8%)
(Missing)	4 (0.2%)	0 (0%)
Feel loved by God, n (%)		
Agree	2,337 (89.5%)	2,427 (91.2%)
Disagree	83 (3.2%)	66 (2.5%)
Not relevant	59 (2.3%)	26 (1.0%)
Unsure	130 (5.0%)	139 (5.2%)
(Missing)	4 (0.1%)	3 (0.1%)
Feel punished by God, n (%)		
Agree	1,171 (44.8%)	1,199 (45.0%)
Disagree	642 (24.6%)	643 (24.1%)
Not relevant	187 (7.2%)	166 (6.2%)
Unsure	605 (23.2%)	649 (24.4%)
(Missing)	7 (0.2%)	5 (0.2%)
Experienced religious criticism, n (%)		
Agree	923 (35.4%)	871 (32.7%)

Table S23d. Unweighted summary statistics for Wave 1 outcome variables in Philippines by retention status.

**Outcome**	**Attritors–Not Observed in Wave 2** N = 2,612	**Retained–Observed in Wave 2** N = 2,661
Disagree	649 (24.8%)	700 (26.3%)
Not relevant	233 (8.9%)	240 (9.0%)
Unsure	797 (30.5%)	850 (31.9%)
(Missing)	10 (0.4%)	0 (0%)
Faith-sharing, n (%)		
Agree	1,432 (54.8%)	1,502 (56.5%)
Disagree	377 (14.4%)	402 (15.1%)
Not relevant	220 (8.4%)	208 (7.8%)
Unsure	578 (22.1%)	548 (20.6%)
(Missing)	4 (0.2%)	1 (0.0%)

Note. N (%); this table is based on non-imputed data. Cumulative percentages for variables may not add up to 100% due to rounding.

Table S23e. Summary of fitted attrition model in Philippines

Characteristic	Odds Ratio	95% CI	p-value
**ANNUAL_WEIGHT_R2**	1.00	0.89, 1.11	0.975
**Recruitment Survey Mode**			
CAPI	—	—	
CATI	0.79	0.64, 0.98	0.033
**Happiness & life satisfaction**	0.96	0.88, 1.05	0.377
**Physical & mental health**	1.02	0.94, 1.11	0.646
**Meaning & purpose**	0.95	0.87, 1.05	0.322
**Character & virtue**	1.03	0.95, 1.12	0.496
**Close social relationships**	1.00	0.92, 1.10	0.913
**Financial & material security**	0.93	0.86, 1.00	0.056
**Extraversion**	1.03	0.96, 1.11	0.401
**Openness to experience**	1.04	0.98, 1.10	0.227
**Agreeableness**	1.04	0.97, 1.13	0.256
**Conscientiousness**	1.04	0.97, 1.12	0.283
**Neuroticism**	0.94	0.88, 1.01	0.114
**Depression symptoms composite**	1.01	0.94, 1.09	0.783
**Anxiety symptoms composite**	1.00	0.93, 1.08	0.990
**Loneliness**	0.96	0.89, 1.04	0.298
**Days exercise per week**	1.01	0.94, 1.08	0.773
**Year of birth (age group)**			
1983-1993 (current age: 30-39 years)	—	—	
1973-1983 (current age: 40-49 years)	0.94	0.77, 1.13	0.489
1998-2005 (current age: 18-24 years)	0.64	0.47, 0.88	0.005
1963-1973 (current age: 50-59 years)	1.10	0.89, 1.37	0.370
1993-1998 (current age: 25-29 years)	0.73	0.57, 0.94	0.016
1953-1963 (current age: 60-69 years)	0.90	0.66, 1.21	0.480
1943-1953 (current age: 70-79 years)	1.13	0.68, 1.85	0.641
1943 or earlier (current age: 80+ years)	1.22	0.45, 3.34	0.694
**Gender of respondent**			
Female	—	—	
Male	0.90	0.75, 1.07	0.235
Other	0.68	0.21, 2.21	0.526
**Marital status**			
Married	—	—	
Domestic partner	1.01	0.84, 1.22	0.885
Single/Never been married	1.19	0.95, 1.50	0.136
Widowed	0.86	0.64, 1.16	0.331
Separated	1.28	0.95, 1.72	0.105
Divorced	0.77	0.23, 2.57	0.666
**Employment status**			
Homemaker	—	—	
Self-employed	0.98	0.81, 1.19	0.875
Employed for an employer	1.10	0.89, 1.36	0.363
Unemployed and looking for a job	1.08	0.83, 1.39	0.561
Student	1.22	0.86, 1.73	0.269
Retired	1.55	0.93, 2.59	0.095
None of these/Other	1.10	0.68, 1.76	0.703
**Religious attendance**			
Once a week	—	—	
One to three times a month	0.94	0.79, 1.11	0.458
A few times a year	0.92	0.76, 1.11	0.372
More than once a week	1.06	0.87, 1.29	0.550

Table S23e. Summary of fitted attrition model in Philippines

**Characteristic**	**Odds Ratio**	**95% CI**	**p-value**
Never	1.25	0.88, 1.79	0.216
**Educational attainment (16+ years)**			
9-15	—	—	
Up to 8	0.98	0.81, 1.17	0.792
16+	1.00	0.78, 1.30	0.976
**Born in This country**			
Born in this country	—	—	
Born in another country	0.51	0.11, 2.29	0.382
**Race plurality (prominent race/ethnic group [0] or not [1])**	1.03	0.97, 1.10	0.382
**Urbanicity**			
A rural area or on a farm	—	—	
A large city	0.89	0.75, 1.07	0.223
A small town or village	1.28	1.08, 1.52	0.005
A suburb of a large city	0.83	0.67, 1.04	0.100
**Monthly household income**			
Philippines: 4,001 – 6,000 pesos	—	—	
Philippines: 1,501 – 3,000 pesos	1.03	0.80, 1.32	0.815
Philippines: 8,001 – 10,000 pesos	1.13	0.87, 1.46	0.367
Philippines: 10,001 – 15,000 pesos	0.97	0.74, 1.27	0.833
Philippines: 6,001 – 8,000 pesos	1.25	0.95, 1.65	0.108
Philippines: 3,001 – 4,000 pesos	1.08	0.80, 1.44	0.626
Philippines: 701 – 1,500 pesos	0.86	0.63, 1.17	0.339
Philippines: 700 pesos or less	0.76	0.55, 1.05	0.097
Philippines: 15,001 – 20,000 pesos	1.40	0.99, 1.99	0.056
(None/No household income)	1.16	0.79, 1.68	0.452
Philippines: 20,001 – 25,000 pesos	1.47	0.95, 2.27	0.086
Philippines: 25,001 – 30,000 pesos	1.43	0.83, 2.46	0.196
Philippines: 30,001 – 40,000 pesos	1.09	0.52, 2.26	0.823
Philippines: 50,001 – 90,000 pesos	2.17	1.03, 4.61	0.043
Philippines: 40,001 – 50,000 pesos	1.13	0.51, 2.51	0.760
Philippines: More than 90,000 pesos	1.19	0.50, 2.87	0.695

Abbreviations: CI = Confidence Interval, OR = Odds Ratio

Notes. N=5292; attrition weights were estimated using the 'survey::svyglm(family=quasibinomial('logit'))' function. All continuous predictors were standardized and all categorical predictors used the most common category as the reference group. Reported p-values are based on the fitted regression model and no adjustments for multiple testing were done within this table.

Table S23f. Summary of principal components in Philippines

PC	Percent Explained by each PC	Cumulative Percent Explained
1	33.60	33.60
2	3.48	37.08
3	3.05	40.12
4	2.03	42.15
5	1.92	44.08
6	1.74	45.82
**7**	**1.63**	**47.45**
8	1.61	49.06
9	1.51	50.57
10	1.46	52.03
11	1.33	53.36
12	1.27	54.63
13	1.22	55.85
14	1.20	57.05
15	1.17	58.22
16	1.14	59.35
17	1.10	60.45
18	1.08	61.54
19	1.06	62.60
20	1.03	63.63

Notes. N=5292; PCA was conducted using ’survey::svyprcomp(.)’ function using all available contemporaneous exposures at wave 1. All PCs were standardized prior to being used as predictors. The bolded row represented the number of retained components for analysis was 7.

Table S23g. Associations of forgivingness with adult well-being and other outcomes at Wave 2 in Philippines

Outcome	Model 1: Demographic and Childhood Variables as Covariates					Model 2: Demographic, Childhood, and Other Wave 1 Confounding Variables (Via Principal Components) as Covariates				
	RR	ES	95% CI	SE	p-value	RR	ES	95% CI	SE	p-value
Human Flourishing										
Secure flourishing index		0.04	(-0.00,0.08)	0.02	0.062		0.01	(-0.02,0.05)	0.02	0.507
Flourishing index		0.06	(0.02,0.10)	0.02	0.006*		0.03	(-0.01,0.06)	0.02	0.178
Happiness & life satisfaction		0.02	(-0.02,0.07)	0.02	0.261		0.01	(-0.03,0.05)	0.02	0.647
Physical & mental health		-0.00	(-0.04,0.04)	0.02	0.949		-0.02	(-0.06,0.02)	0.02	0.436
Meaning & purpose		0.06	(0.02,0.10)	0.02	4.51e-03**		0.03	(-0.01,0.07)	0.02	0.181
Character & virtue		0.06	(0.01,0.10)	0.02	0.013*		0.02	(-0.02,0.07)	0.02	0.254
Close social relationships		0.06	(0.02,0.11)	0.02	3.66e-03**		0.05	(0.00,0.09)	0.02	0.031*
Financial & material security		-0.03	(-0.07,0.01)	0.02	0.210		-0.03	(-0.07,0.02)	0.02	0.228
Psychological Well-Being										
Happiness (a)		0.04	(-0.00,0.08)	0.02	0.066		0.03	(-0.01,0.07)	0.02	0.195
Life satisfaction (a)		0.00	(-0.04,0.05)	0.02	0.941		-0.01	(-0.06,0.04)	0.02	0.659
Current life evaluation		0.00	(-0.04,0.04)	0.02	0.995		-0.01	(-0.05,0.03)	0.02	0.671
Future life evaluation		0.03	(-0.02,0.07)	0.02	0.222		0.01	(-0.04,0.05)	0.02	0.817
Optimism		0.03	(-0.01,0.07)	0.02	0.120		0.00	(-0.04,0.04)	0.02	0.986
Freedom to pursue what's important		0.04	(-0.00,0.08)	0.02	0.075		0.02	(-0.02,0.06)	0.02	0.389
Inner peace	1.00		(0.98,1.02)	0.01	0.894	0.99		(0.98,1.01)	0.01	0.593
Life balance	1.03		(1.01,1.05)	0.01	0.009*	1.02		(0.99,1.04)	0.01	0.156
Sense of mastery	1.02		(1.00,1.04)	0.01	0.068	1.01		(0.99,1.03)	0.01	0.422
Meaningful activities (c)		0.05	(0.00,0.09)	0.02	0.038*		0.02	(-0.02,0.06)	0.02	0.327
Understanding purpose (c)		0.05	(0.00,0.10)	0.02	0.043*		0.02	(-0.03,0.07)	0.02	0.368
Self-rated mental health (b)		0.02	(-0.03,0.06)	0.02	0.483		-0.00	(-0.05,0.04)	0.02	0.870
Psychological Distress										
Traumatic distress	1.03		(1.01,1.05)	0.01	4.83e-03**	1.03		(1.00,1.05)	0.01	0.020*
Depression symptoms composite	1.01		(0.99,1.03)	0.01	0.437	1.00		(0.98,1.02)	0.01	0.819
Depression – feel hopeless	1.01		(0.99,1.03)	0.01	0.528	1.00		(0.98,1.02)	0.01	0.707
Depression – loss of interest	1.00		(0.98,1.02)	0.01	0.870	0.99		(0.97,1.01)	0.01	0.538
Anxiety symptoms composite	1.00		(0.98,1.03)	0.01	0.820	1.00		(0.98,1.02)	0.01	0.891
Anxiety – feel on edge	1.00		(0.98,1.02)	0.01	0.713	1.00		(0.98,1.02)	0.01	0.952
Anxiety – cannot stop worrying	1.00		(0.98,1.03)	0.01	0.749	1.00		(0.98,1.03)	0.01	0.878
Suffering	1.01		(0.99,1.03)	0.01	0.295	1.00		(0.98,1.03)	0.01	0.662
Social Well-Being										
Relationship contentment (e)		0.06	(0.01,0.11)	0.02	0.019*		0.04	(-0.01,0.09)	0.02	0.088
Relationship satisfaction (e)		0.05	(0.01,0.09)	0.02	0.029*		0.04	(-0.01,0.08)	0.02	0.102
Social support		0.00	(-0.04,0.04)	0.02	0.920		-0.01	(-0.05,0.03)	0.02	0.638
Intimate/close friend	1.01		(0.99,1.03)	0.01	0.213	1.01		(0.99,1.03)	0.01	0.422
Government approval	1.01		(0.99,1.02)	0.01	0.416	1.00		(0.99,1.02)	0.01	0.704
Say in government	1.02		(1.00,1.04)	0.01	0.124	1.00		(0.98,1.02)	0.01	0.839
Belonging in country		0.07	(0.02,0.11)	0.02	0.007*		0.04	(-0.01,0.08)	0.02	0.107
City/place satisfaction	1.01		(0.99,1.03)	0.01	0.187	1.01		(0.99,1.03)	0.01	0.357
Trust within country	1.02		(1.01,1.04)	0.01	0.008*	1.02		(1.00,1.03)	0.01	0.064
Social Participation										
Ever been married	1.00		(0.98,1.01)	0.01	0.667	1.00		(0.98,1.01)	0.01	0.804
Currently divorced	1.00		(1.00,1.00)	0.00	0.547	1.00		(1.00,1.00)	0.00	0.544
Number of children		-0.03	(-0.08,0.02)	0.03	0.264		-0.03	(-0.08,0.02)	0.02	0.295
Weekly+ community participation	1.00		(0.99,1.02)	0.01	0.803	1.00		(0.98,1.01)	0.01	0.826
Weekly+ religious attendance	1.01		(1.00,1.04)	0.01	0.146	1.01		(0.99,1.03)	0.01	0.391
Social Distress										
Loneliness		0.02	(-0.02,0.06)	0.02	0.371		0.03	(-0.01,0.07)	0.02	0.143
Perceived discrimination	1.01		(0.99,1.03)	0.01	0.419	1.00		(0.98,1.02)	0.01	0.968
Character & Prosocial Behavior										
Orientation to promote good (d)		0.05	(0.01,0.09)	0.02	0.025*		0.02	(-0.03,0.06)	0.02	0.438
Delayed gratification (d)		0.05	(-0.00,0.09)	0.02	0.071		0.02	(-0.02,0.07)	0.02	0.322
Hope		0.05	(0.01,0.09)	0.02	0.010*		0.02	(-0.02,0.06)	0.02	0.239
Gratitude		0.06	(0.01,0.11)	0.02	0.011*		0.03	(-0.01,0.07)	0.02	0.191
Showing love/care		0.07	(0.02,0.11)	0.02	0.005*		0.05	(0.00,0.09)	0.02	0.052
Forgivingness	1.10		(1.08,1.12)	0.01	2.22e-16***	1.09		(1.07,1.11)	0.01	1.22e-15***
Charitable giving	1.01		(0.99,1.03)	0.01	0.424	1.00		(0.98,1.03)	0.01	0.722
Helping strangers	1.02		(1.00,1.04)	0.01	0.034*	1.01		(0.99,1.03)	0.01	0.174
Volunteering	0.99		(0.97,1.01)	0.01	0.322	0.99		(0.97,1.00)	0.01	0.130
Physical Health & Health Behavior										
Self-rated physical health (b)		-0.02	(-0.06,0.03)	0.02	0.427		-0.02	(-0.07,0.02)	0.02	0.327
Health problems	1.01		(0.99,1.03)	0.01	0.313	1.01		(0.99,1.03)	0.01	0.450
Pain in past 4 weeks	1.01		(0.99,1.03)	0.01	0.180	1.01		(0.99,1.03)	0.01	0.381
Daily smoker	1.00		(0.99,1.02)	0.01	0.803	1.01		(0.99,1.02)	0.01	0.442
Number of drinks per week		0.00	(-0.04,0.04)	0.02	0.923		0.01	(-0.04,0.05)	0.02	0.760
Days exercise per week		-0.02	(-0.06,0.02)	0.02	0.402		-0.01	(-0.05,0.03)	0.02	0.690
Socioeconomic Outcomes										
Financial security (f)		-0.02	(-0.06,0.03)	0.02	0.438		-0.02	(-0.07,0.02)	0.02	0.382
Material security (f)		-0.03	(-0.07,0.02)	0.02	0.226		-0.02	(-0.07,0.02)	0.02	0.324
Educational attainment (16+ years)	1.00		(0.99,1.01)	0.01	0.972	1.00		(0.98,1.01)	0.01	0.794
Currently employed	1.00		(0.98,1.02)	0.01	0.975	1.00		(0.98,1.02)	0.01	0.950
Financially comfortable/getting by	0.99		(0.97,1.01)	0.01	0.370	0.99		(0.97,1.01)	0.01	0.292
Own home	0.99		(0.97,1.01)	0.01	0.309	0.99		(0.97,1.01)	0.01	0.341
Income – top quintile	1.00		(0.98,1.02)	0.01	0.898	1.00		(0.98,1.02)	0.01	0.850
Religion & Spirituality										
Religious/spiritual connection	1.04		(1.02,1.07)	0.01	2.56e-04***	1.02		(1.00,1.04)	0.01	0.091
Belief in life after death	1.02		(1.00,1.04)	0.01	0.064	1.00		(0.97,1.02)	0.01	0.731
Transformative religious experience	1.02		(1.00,1.04)	0.01	0.022*	1.00		(0.98,1.02)	0.01	0.769
Religious reading or listening	1.00		(0.98,1.02)	0.01	0.746	0.99		(0.97,1.01)	0.01	0.198
Prayer or meditation	1.03		(1.01,1.05)	0.01	0.006*	1.01		(0.99,1.04)	0.01	0.228
Belief in God/gods/spiritual forces	1.01		(1.00,1.02)	0.01	0.198	1.00		(0.99,1.02)	0.01	0.611
Religious centrality	1.00		(0.98,1.02)	0.01	0.900	0.98		(0.96,1.00)	0.01	0.096
Religious/spiritual comfort	1.02		(1.00,1.05)	0.01	0.021*	1.01		(0.99,1.03)	0.01	0.506
Feel loved by God	1.00		(0.99,1.02)	0.01	0.626	1.00		(0.98,1.01)	0.01	0.556
Feel punished by God	1.00		(0.98,1.02)	0.01	0.909	1.00		(0.98,1.02)	0.01	0.867
Experienced religious criticism	1.01		(0.99,1.03)	0.01	0.469	1.00		(0.98,1.03)	0.01	0.641
Faith-sharing	1.03		(1.01,1.06)	0.01	0.007*	1.01		(0.99,1.04)	0.01	0.257

Notes. N=5292; Reference for focal predictor: never/rarely. RR, risk-ratio, null effect is 1.00; ES, effect size measure for standardized regression coefficient, null effect is 0.00; SE, standard error, the SE reported for binary/Likert-type outcomes where risk-ratios are on the log(RR) scale; CI, confidence interval; p-value, a Wald-type test of the null hypothesis that the effect of the focal predictor is zero; (a) item part of the Happiness & Life Satisfaction domain of the Secure Flourishing Index; (b) item part of the Physical & Mental Health domain of the Secure Flourishing Index; (c) item part of the Meaning & Purpose domain of the Secure Flourishing Index; (d) item part of the Character & Virtue domain of the Secure Flourishing Index; (e) item part of the Subjective Social Connectedness domain of the Secure Flourishing Index; (f) item part of the Financial & Material Security domain of the Secure Flourishing Index.

Multiple imputation was performed to impute missing data on the covariates, exposure, and outcomes. All models controlled for sociodemographic and childhood factors assessed at Wave 1. For Model 2 with PC (principal components), the first seven principal components of the entire set of contemporaneous confounders assessed at Wave 1 were included as additional covariates of the outcomes at Wave 2.

An outcome-wide analytic approach was used, and a separate model was run for each outcome. A different type of model was run depending on the nature of the outcome: (1) for each binary outcome, a weighted generalized linear model (with a log link and Poisson distribution) was used to estimate an RR; and (2) for each continuous outcome, a weighted linear regression model was used to estimate a ES. All effect sizes were standardized. For continuous outcomes, the ES represents the change in SD on the outcome between the lower and upper categories of the binary focal predictor. For binary outcomes, the RR represents the change in risk of being in the upper category compared to the lower category between the lower and upper categories of the binary focal predictor.

P-value significance thresholds: $p < 0.05$*, $p < 0.005$**, (Bonferroni) $p < 6.41e-04$***, correction for multiple testing using Bonferroni adjusted significant threshold.

Table S23h. Associations of forgivingness with adult well-being and other outcomes at Wave 2 in Philippines using complete-case analyses with attrition weights.

	Model 1: Demographic and Childhood Variables as Covariates					Model 2: Demographic, Childhood, and Other Wave 1 Confounding Variables (Via Principal Components) as Covariates				
Outcome	RR	ES	95% CI	SE	p-value	RR	ES	95% CI	SE	p-value
Human Flourishing										
Secure flourishing index		0.01	(-0.04,0.06)	0.03	0.635		-0.01	(-0.06,0.04)	0.02	0.701
Flourishing index		0.03	(-0.01,0.08)	0.02	0.172		0.01	(-0.04,0.06)	0.02	0.684
Happiness & life satisfaction		0.02	(-0.03,0.06)	0.02	0.403		0.01	(-0.04,0.05)	0.02	0.711
Physical & mental health		-0.01	(-0.06,0.04)	0.03	0.747		-0.02	(-0.07,0.03)	0.03	0.472
Meaning & purpose		0.04	(-0.01,0.09)	0.02	0.095		0.01	(-0.04,0.05)	0.02	0.782
Character & virtue		0.04	(-0.00,0.09)	0.02	0.059		0.01	(-0.03,0.06)	0.02	0.514
Close social relationships		0.04	(-0.01,0.09)	0.03	0.148		0.03	(-0.02,0.08)	0.03	0.299
Financial & material security		-0.05	(-0.09,-0.00)	0.02	0.048*		-0.05	(-0.10,-0.00)	0.02	0.033*
Psychological Well-Being										
Happiness (a)		0.04	(-0.01,0.08)	0.02	0.115		0.03	(-0.02,0.08)	0.02	0.204
Life satisfaction (a)		-0.00	(-0.05,0.04)	0.02	0.928		-0.01	(-0.06,0.03)	0.02	0.539
Current life evaluation		-0.00	(-0.05,0.04)	0.02	0.971		-0.01	(-0.06,0.04)	0.02	0.686
Future life evaluation		0.01	(-0.04,0.06)	0.02	0.709		-0.02	(-0.06,0.03)	0.02	0.498
Optimism		0.03	(-0.01,0.07)	0.02	0.165		-0.00	(-0.04,0.04)	0.02	0.979
Freedom to pursue what's important		0.03	(-0.02,0.08)	0.03	0.209		0.01	(-0.04,0.06)	0.03	0.682
Inner peace	1.00		(0.98,1.02)	0.01	0.985	0.99		(0.97,1.01)	0.01	0.237
Life balance	1.04		(1.02,1.06)	0.01	1e-03**	1.02		(1.00,1.04)	0.01	0.070
Sense of mastery	1.03		(1.01,1.05)	0.01	0.015*	1.01		(0.99,1.04)	0.01	0.285
Meaningful activities (c)		0.03	(-0.02,0.08)	0.02	0.223		0.00	(-0.05,0.05)	0.03	0.870
Understanding purpose (c)		0.04	(-0.01,0.08)	0.02	0.118		0.01	(-0.04,0.05)	0.02	0.767
Self-rated mental health (b)		0.01	(-0.04,0.06)	0.03	0.767		-0.01	(-0.06,0.04)	0.03	0.646
Psychological Distress										
Traumatic distress	1.04		(1.02,1.06)	0.01	5.15e-04***	1.03		(1.01,1.05)	0.01	0.005*
Depression symptoms composite	1.01		(0.99,1.03)	0.01	0.337	1.00		(0.98,1.02)	0.01	0.875
Depression – feel hopeless	1.01		(0.99,1.03)	0.01	0.544	1.00		(0.98,1.02)	0.01	0.893
Depression – loss of interest	1.00		(0.98,1.02)	0.01	0.942	0.99		(0.97,1.01)	0.01	0.441
Anxiety symptoms composite	1.01		(0.98,1.03)	0.01	0.594	1.00		(0.98,1.02)	0.01	0.873
Anxiety – feel on edge	1.01		(0.99,1.03)	0.01	0.331	1.01		(0.99,1.03)	0.01	0.538
Anxiety – cannot stop worrying	1.01		(0.98,1.03)	0.01	0.666	1.00		(0.98,1.03)	0.01	0.797
Suffering	1.01		(0.99,1.03)	0.01	0.499	1.00		(0.98,1.02)	0.01	0.922
Social Well-Being										
Relationship contentment (e)		0.04	(-0.01,0.09)	0.03	0.116		0.03	(-0.02,0.08)	0.03	0.257
Relationship satisfaction (e)		0.03	(-0.02,0.08)	0.03	0.253		0.02	(-0.03,0.07)	0.02	0.422
Social support		-0.01	(-0.06,0.04)	0.02	0.745		-0.02	(-0.07,0.03)	0.03	0.403
Intimate/close friend	1.01		(0.99,1.02)	0.01	0.287	1.01		(0.99,1.02)	0.01	0.474
Government approval	1.00		(0.98,1.03)	0.01	0.644	1.00		(0.98,1.02)	0.01	0.963
Say in government	1.01		(0.99,1.03)	0.01	0.248	0.99		(0.97,1.01)	0.01	0.524
Belonging in country		0.06	(0.02,0.11)	0.02	0.007*		0.03	(-0.01,0.07)	0.02	0.190
City/place satisfaction	1.01		(0.99,1.03)	0.01	0.344	1.01		(0.99,1.03)	0.01	0.404
Trust within country	1.02		(1.01,1.04)	0.01	0.008*	1.02		(1.00,1.03)	0.01	0.078
Social Participation										
Ever been married	0.99		(0.98,1.01)	0.01	0.297	0.99		(0.98,1.01)	0.01	0.481
Currently divorced	1.00		(1.00,1.00)	0.00	0.323	1.00		(1.00,1.00)	0.00	0.327
Number of children		-0.04	(-0.11,0.02)	0.03	0.186		-0.05	(-0.11,0.02)	0.03	0.149
Weekly+ community participation	1.01		(0.99,1.03)	0.01	0.453	1.00		(0.98,1.02)	0.01	0.815
Weekly+ religious attendance	1.02		(1.00,1.04)	0.01	0.088	1.01		(0.99,1.03)	0.01	0.327
Social Distress										
Loneliness		0.05	(0.00,0.09)	0.02	0.045*		0.05	(0.01,0.10)	0.02	0.015*
Perceived discrimination	1.01		(0.99,1.03)	0.01	0.501	1.00		(0.98,1.02)	0.01	0.850
Character & Prosocial Behavior										
Orientation to promote good (d)		0.04	(-0.01,0.09)	0.02	0.105		0.01	(-0.04,0.05)	0.02	0.760
Delayed gratification (d)		0.04	(-0.01,0.09)	0.02	0.100		0.02	(-0.03,0.07)	0.02	0.426
Hope		0.06	(0.00,0.11)	0.03	0.039*		0.03	(-0.02,0.09)	0.03	0.241
Gratitude		0.07	(0.02,0.11)	0.02	2.78e-03**		0.04	(-0.01,0.08)	0.02	0.102
Showing love/care		0.08	(0.04,0.13)	0.03	7.18e-04**		0.06	(0.02,0.11)	0.02	0.009*
Forgivingness	1.11		(1.09,1.14)	0.01	2.22e-16***	1.10		(1.08,1.13)	0.01	2.22e-16***
Charitable giving	1.00		(0.98,1.02)	0.01	0.785	0.99		(0.97,1.01)	0.01	0.270
Helping strangers	1.02		(1.00,1.04)	0.01	0.086	1.01		(0.99,1.03)	0.01	0.431
Volunteering	0.98		(0.96,1.01)	0.01	0.137	0.98		(0.96,1.00)	0.01	0.044*
Physical Health & Health Behavior										
Self-rated physical health (b)		-0.02	(-0.07,0.03)	0.03	0.413		-0.02	(-0.07,0.03)	0.03	0.408
Health problems	1.01		(0.98,1.03)	0.01	0.662	1.00		(0.98,1.02)	0.01	0.977
Pain in past 4 weeks	1.03		(1.00,1.05)	0.01	0.032*	1.02		(1.00,1.04)	0.01	0.113
Daily smoker	1.00		(0.98,1.02)	0.01	0.824	1.00		(0.98,1.02)	0.01	0.827
Number of drinks per week		0.00	(-0.03,0.03)	0.02	0.973		0.00	(-0.03,0.03)	0.02	0.862
Days exercise per week		-0.03	(-0.08,0.02)	0.02	0.181		-0.02	(-0.06,0.03)	0.02	0.469
Socioeconomic Outcomes										
Financial security (f)		-0.03	(-0.08,0.01)	0.02	0.173		-0.04	(-0.08,0.01)	0.02	0.097
Material security (f)		-0.05	(-0.10,-0.01)	0.02	0.026*		-0.05	(-0.10,-0.00)	0.02	0.031*
Educational attainment (16+ years)	1.00		(0.99,1.01)	0.01	0.876	1.00		(0.99,1.01)	0.01	0.827
Currently employed	0.99		(0.97,1.01)	0.01	0.437	0.99		(0.97,1.01)	0.01	0.478
Financially comfortable/getting by	0.99		(0.96,1.01)	0.01	0.259	0.99		(0.96,1.01)	0.01	0.232
Own home	0.98		(0.96,1.00)	0.01	0.043*	0.98		(0.96,1.00)	0.01	0.056
Income – top quintile	1.01		(0.98,1.03)	0.01	0.626	1.01		(0.99,1.03)	0.01	0.433
Religion & Spirituality										
Religious/spiritual connection	1.04		(1.02,1.07)	0.01	5.29e-04***	1.02		(1.00,1.04)	0.01	0.119
Belief in life after death	1.02		(1.00,1.04)	0.01	0.086	1.00		(0.97,1.02)	0.01	0.652
Transformative religious experience	1.03		(1.01,1.05)	0.01	0.007*	1.01		(0.98,1.03)	0.01	0.573
Religious reading or listening	1.00		(0.98,1.02)	0.01	0.834	0.99		(0.97,1.00)	0.01	0.141
Prayer or meditation	1.03		(1.01,1.06)	0.01	0.007*	1.02		(0.99,1.04)	0.01	0.176
Belief in God/gods/spiritual forces	1.01		(0.99,1.02)	0.01	0.300	1.00		(0.99,1.02)	0.01	0.763
Religious centrality	1.00		(0.98,1.02)	0.01	0.777	0.99		(0.96,1.01)	0.01	0.167
Religious/spiritual comfort	1.03		(1.00,1.05)	0.01	0.016*	1.01		(0.99,1.03)	0.01	0.522
Feel loved by God	1.00		(0.99,1.02)	0.01	0.734	1.00		(0.98,1.01)	0.01	0.481
Feel punished by God	1.01		(0.99,1.03)	0.01	0.489	1.00		(0.98,1.03)	0.01	0.754
Experienced religious criticism	1.02		(1.00,1.04)	0.01	0.124	1.01		(0.99,1.03)	0.01	0.480
Faith-sharing	1.03		(1.00,1.05)	0.01	0.043*	1.00		(0.98,1.03)	0.01	0.762

Table S23h. Associations of forgivingness with adult well-being and other outcomes at Wave 2 in Philippines using complete-case analyses with attrition weights.

	Model 1: Demographic and Childhood Variables as Covariates					Model 2: Demographic, Childhood, and Other Wave 1 Confounding Variables (Via Principal Components) as Covariates				
Outcome	RR	ES	95% CI	SE	p-value	RR	ES	95% CI	SE	p-value

Notes. N=2682; Reference for focal predictor: never/rarely. RR, risk-ratio, null effect is 1.00; ES, effect size measure for standardized regression coefficient, null effect is 0.00; SE, standard error, the SE reported for binary/Likert-type outcomes where risk-ratios are on the log(RR) scale; CI, confidence interval; p-value, a Wald-type test of the null hypothesis that the effect of the focal predictor is zero; (a) item part of the Happiness & Life Satisfaction domain of the Secure Flourishing Index; (b) item part of the Physical & Mental Health domain of the Secure Flourishing Index; (c) item part of the Meaning & Purpose domain of the Secure Flourishing Index; (d) item part of the Character & Virtue domain of the Secure Flourishing Index; (e) item part of the Subjective Social Connectedness domain of the Secure Flourishing Index; (f) item part of the Financial & Material Security domain of the Secure Flourishing Index.

Attrition weights were computed to adjust the complete case data (those who responded at Wave 2 to at least 50% of the questions) and multiple imputation was used to impute missing data on all remaining within wave on the covariates, exposure, and outcomes. All models controlled for sociodemographic and childhood factors assessed at Wave 1. For Model 2 with PC (principal components), the first seven principal components of the entire set of contemporaneous confounders assessed at Wave 1 were included as additional covariates of the outcomes at Wave 2.

An outcome-wide analytic approach was used, and a separate model was run for each outcome. A different type of model was run depending on the nature of the outcome: (1) for each binary outcome, a weighted generalized linear model (with a log link and Poisson distribution) was used to estimate an RR; and (2) for each continuous outcome, a weighted linear regression model was used to estimate a ES. All effect sizes were standardized. For continuous outcomes, the ES represents the change in SD on the outcome between the lower and upper categories of the binary focal predictor. For binary outcomes, the RR represents the change in risk of being in the upper category compared to the lower category between the lower and upper categories of the binary focal predictor.

P-value significance thresholds: $p < 0.05$*, $p < 0.005$**, (Bonferroni) $p < 6.41e-04$***, correction for multiple testing using Bonferroni adjusted significant threshold.

Table S23i. Sensitivity analysis of forgivingness outcome-wide results to unmeasured confounding using E-values in Philippines

	Multiple Imputation				Complete Case w/ Attrition Weights			
	Model 1: Demographics and Childhood Variables as Covariates		Model 2: Demographics, Childhood, and Other Wave 1 Confounders (Via Principal Components) as Covariates		Model 1: Demographics and Childhood Variables as Covariates		Model 2: Demographics, Childhood, and Other Wave 1 Confounders (Via Principal Components) as Covariates	
Outcome	EE	ECI	EE	ECI	EE	ECI	EE	ECI
Human Flourishing								
Secure flourishing index	1.38	1.00	1.19	1.00	1.19	1.00	1.16	1.00
Flourishing index	1.50	1.23	1.30	1.00	1.35	1.00	1.16	1.00
Happiness & life satisfaction	1.28	1.00	1.17	1.00	1.25	1.00	1.15	1.00
Physical & mental health	1.05	1.00	1.22	1.00	1.15	1.00	1.25	1.00
Meaning & purpose	1.52	1.25	1.31	1.00	1.39	1.00	1.13	1.00
Character & virtue	1.51	1.20	1.29	1.00	1.43	1.00	1.21	1.00
Close social relationships	1.55	1.27	1.44	1.11	1.38	1.00	1.30	1.00
Financial & material security	1.30	1.00	1.29	1.00	1.44	1.03	1.46	1.10
Psychological Well-Being								
Happiness	1.39	1.00	1.32	1.00	1.38	1.00	1.33	1.00
Life satisfaction	1.06	1.00	1.18	1.00	1.07	1.00	1.21	1.00
Current life evaluation	1.02	1.00	1.16	1.00	1.04	1.00	1.16	1.00
Future life evaluation	1.32	1.00	1.12	1.00	1.16	1.00	1.22	1.00
Optimism	1.35	1.00	1.03	1.00	1.33	1.00	1.04	1.00
Freedom to pursue what's important	1.39	1.00	1.25	1.00	1.34	1.00	1.18	1.00
Inner peace	1.06	1.00	1.12	1.00	1.02	1.00	1.21	1.00
Life balance	1.33	1.15	1.23	1.00	1.40	1.23	1.28	1.00
Sense of mastery	1.26	1.00	1.16	1.00	1.33	1.12	1.20	1.00
Meaningful activities	1.43	1.09	1.26	1.00	1.32	1.00	1.10	1.00
Understanding purpose	1.46	1.08	1.27	1.00	1.38	1.00	1.14	1.00
Self-rated mental health	1.23	1.00	1.10	1.00	1.14	1.00	1.18	1.00
Psychological Distress								
Traumatic distress	1.36	1.18	1.32	1.11	1.42	1.25	1.36	1.17
Depression symptoms composite	1.16	1.00	1.08	1.00	1.19	1.00	1.07	1.00
Depression – feel hopeless	1.13	1.00	1.10	1.00	1.14	1.00	1.06	1.00
Depression – loss of interest	1.07	1.00	1.14	1.00	1.05	1.00	1.17	1.00
Anxiety symptoms composite	1.08	1.00	1.06	1.00	1.14	1.00	1.07	1.00
Anxiety – feel on edge	1.10	1.00	1.04	1.00	1.18	1.00	1.14	1.00
Anxiety – cannot stop worrying	1.10	1.00	1.07	1.00	1.12	1.00	1.09	1.00
Suffering	1.19	1.00	1.11	1.00	1.15	1.00	1.05	1.00
Social Well-Being								
Relationship contentment	1.51	1.17	1.40	1.00	1.40	1.00	1.32	1.00
Relationship satisfaction	1.46	1.12	1.38	1.00	1.32	1.00	1.25	1.00
Social support	1.07	1.00	1.16	1.00	1.15	1.00	1.26	1.00
Intimate/close friend	1.20	1.00	1.15	1.00	1.16	1.00	1.13	1.00
Government approval	1.14	1.00	1.10	1.00	1.12	1.00	1.04	1.00
Say in government	1.24	1.00	1.07	1.00	1.20	1.00	1.15	1.00
Belonging in country	1.57	1.25	1.38	1.00	1.53	1.23	1.32	1.00
City/place satisfaction	1.19	1.00	1.16	1.00	1.17	1.00	1.16	1.00
Trust within country	1.28	1.13	1.23	1.00	1.28	1.13	1.23	1.00
Social Participation								
Ever been married	1.09	1.00	1.07	1.00	1.16	1.00	1.13	1.00
Currently divorced	1.02	1.00	1.02	1.00	1.02	1.00	1.02	1.00
Number of children	1.32	1.00	1.30	1.00	1.42	1.00	1.44	1.00
Weekly+ community participation	1.07	1.00	1.07	1.00	1.15	1.00	1.08	1.00
Weekly+ religious attendance	1.22	1.00	1.17	1.00	1.26	1.00	1.19	1.00
Social Distress								
Loneliness	1.23	1.00	1.32	1.00	1.44	1.05	1.49	1.17
Perceived discrimination	1.15	1.00	1.03	1.00	1.15	1.00	1.08	1.00
Character & Prosocial Behavior								
Orientation to promote good	1.46	1.13	1.23	1.00	1.40	1.00	1.14	1.00
Delayed gratification	1.43	1.00	1.28	1.00	1.39	1.00	1.25	1.00
Hope	1.46	1.19	1.28	1.00	1.50	1.09	1.35	1.00
Gratitude	1.52	1.21	1.33	1.00	1.57	1.28	1.38	1.00
Showing love/care	1.56	1.26	1.44	1.03	1.68	1.37	1.53	1.22
Forgivingness	1.79	1.66	1.75	1.62	1.88	1.72	1.82	1.67
Charitable giving	1.16	1.00	1.10	1.00	1.09	1.00	1.19	1.00
Helping strangers	1.28	1.06	1.21	1.00	1.26	1.00	1.16	1.00
Volunteering	1.17	1.00	1.22	1.00	1.24	1.00	1.29	1.04
Physical Health & Health Behavior								
Self-rated physical health	1.24	1.00	1.27	1.00	1.27	1.00	1.27	1.00
Health problems	1.19	1.00	1.16	1.00	1.12	1.00	1.03	1.00
Pain in past 4 weeks	1.21	1.00	1.17	1.00	1.31	1.08	1.26	1.00
Daily smoker	1.07	1.00	1.14	1.00	1.08	1.00	1.07	1.00
Number of drinks per week	1.07	1.00	1.14	1.00	1.03	1.00	1.08	1.00
Days exercise per week	1.23	1.00	1.15	1.00	1.35	1.00	1.23	1.00
Socioeconomic Outcomes								
Financial security	1.23	1.00	1.26	1.00	1.35	1.00	1.39	1.00
Material security	1.30	1.00	1.27	1.00	1.49	1.13	1.47	1.11
Educational attainment (16+ years)	1.02	1.00	1.07	1.00	1.05	1.00	1.06	1.00
Currently employed	1.03	1.00	1.04	1.00	1.16	1.00	1.15	1.00
Financially comfortable/getting by	1.18	1.00	1.19	1.00	1.21	1.00	1.21	1.00
Own home	1.17	1.00	1.17	1.00	1.30	1.04	1.29	1.00
Income – top quintile	1.05	1.00	1.07	1.00	1.12	1.00	1.16	1.00
Religion & Spirituality								
Religious/spiritual connection	1.43	1.27	1.25	1.00	1.43	1.25	1.25	1.00
Belief in life after death	1.27	1.00	1.10	1.00	1.26	1.00	1.12	1.00
Transformative religious experience	1.30	1.10	1.09	1.00	1.34	1.16	1.14	1.00
Religious reading or listening	1.09	1.00	1.20	1.00	1.08	1.00	1.23	1.00
Prayer or meditation	1.34	1.16	1.21	1.00	1.36	1.16	1.23	1.00
Belief in God/gods/spiritual forces	1.16	1.00	1.10	1.00	1.16	1.00	1.08	1.00
Religious centrality	1.06	1.00	1.25	1.00	1.09	1.00	1.22	1.00
Religious/spiritual comfort	1.31	1.10	1.14	1.00	1.31	1.11	1.14	1.00
Feel loved by God	1.09	1.00	1.10	1.00	1.08	1.00	1.11	1.00
Feel punished by God	1.05	1.00	1.06	1.00	1.16	1.00	1.10	1.00
Experienced religious criticism	1.15	1.00	1.12	1.00	1.24	1.00	1.15	1.00
Faith-sharing	1.37	1.17	1.21	1.00	1.31	1.04	1.10	1.00

Notes. EE, E-value for estimate; ECI, E-value for the limit of the confidence interval. The formula for calculating E-values can be found in VanderWeele and Ding (2017). E-values for estimate are the minimum strength of association on the risk ratio scale that an unmeasured confounder would need to have with both the exposure and the outcome to fully explain away the observed association between the exposure and outcome, conditional on the measured covariates. E-values for the 95% CI closest to the null denote the minimum strength of association on the risk ratio scale that an unmeasured confounder would need to have with both the exposure and the outcome to shift the CI to include the null value, conditional on the measured covariates.

Table S24a. Weighted summary statistics for demographic and childhood variables in Poland

Characteristic	Wave 1<br>N = 10,389	Wave 2<br>N = 6,516
Forgivingness, n (%)		
Always	1,706 (16.4%)	952 (14.6%)
Often	5,036 (48.5%)	3,094 (47.5%)
Rarely	2,823 (27.2%)	1,987 (30.5%)
Never	761 (7.3%)	450 (6.9%)
(Missing)	63 (0.6%)	32 (0.5%)
Year of birth, n (%)		
1943 or earlier (current age: 80+ years)	129 (1.2%)	106 (1.6%)
1943-1953 (current age: 70-79 years)	789 (7.6%)	623 (9.6%)
1953-1963 (current age: 60-69 years)	1,970 (19.0%)	1,244 (19.1%)
1963-1973 (current age: 50-59 years)	1,576 (15.2%)	976 (15.0%)
1973-1983 (current age: 40-49 years)	1,896 (18.3%)	1,251 (19.2%)
1983-1993 (current age: 30-39 years)	2,133 (20.5%)	1,291 (19.8%)
1993-1998 (current age: 25-29 years)	795 (7.7%)	480 (7.4%)
1998-2005 (current age: 18-24 years)	1,101 (10.6%)	544 (8.3%)
(Missing)	1 (<0.0%)	1 (0.0%)
Age of participant		
Mean	46.5	48.0
Standard Deviation	16.6	16.7
Min, Max	18.0, 92.0	19.0, 93.0
Gender, n (%)		
Male	4,949 (47.6%)	3,084 (47.3%)
Female	5,411 (52.1%)	3,412 (52.4%)
Other	4 (0.0%)	1 (0.0%)
(Missing)	25 (0.2%)	19 (0.3%)
Respondent marital status, n (%)		
Single/Never been married	1,910 (18.4%)	1,070 (16.4%)
Married	6,011 (57.9%)	3,900 (59.9%)
Separated	107 (1.0%)	79 (1.2%)
Divorced	517 (5.0%)	309 (4.7%)
Widowed	930 (9.0%)	503 (7.7%)
Domestic partner	544 (5.2%)	352 (5.4%)
(Missing)	369 (3.6%)	304 (4.7%)
Education (years), n (%)		
Up to 8	742 (7.1%)	171 (2.6%)
9-15	6,711 (64.6%)	4,347 (66.7%)
16+	2,934 (28.2%)	1,998 (30.7%)
(Missing)	1 (0.0%)	0 (0%)
Employment status, n (%)		
Employed for an employer	5,689 (54.8%)	3,654 (56.1%)
Self-employed	652 (6.3%)	348 (5.3%)
Retired	2,494 (24.0%)	1,571 (24.1%)
Student	561 (5.4%)	295 (4.5%)
Homemaker	371 (3.6%)	169 (2.6%)
Unemployed and looking for a job	294 (2.8%)	114 (1.7%)
None of these/Other	204 (2.0%)	175 (2.7%)
(Missing)	124 (1.2%)	191 (2.9%)
Current religious service attendance, n (%)		
More than once a week	280 (2.7%)	123 (1.9%)
Once a week	3,277 (31.5%)	2,014 (30.9%)

Table S24a. Weighted summary statistics for demographic and childhood variables in Poland

**Characteristic**	**Wave 1** N = 10,389	**Wave 2** N = 6,516
One to three times a month	2,061 (19.8%)	1,192 (18.3%)
A few times a year	3,084 (29.7%)	2,019 (31.0%)
Never	1,609 (15.5%)	1,073 (16.5%)
(Missing)	78 (0.7%)	95 (1.5%)
Immigration status, n (%)		
Born in this country	10,257 (98.7%)	6,452 (99.0%)
Born in another country	111 (1.1%)	58 (0.9%)
(Missing)	20 (0.2%)	7 (0.1%)
Parental marital status around age 12, n (%)		
Parents were married	8,979 (86.4%)	5,748 (88.2%)
Parents were divorced	595 (5.7%)	397 (6.1%)
Parents were never married	200 (1.9%)	94 (1.4%)
One or both of them had died	303 (2.9%)	149 (2.3%)
Unsure	223 (2.2%)	95 (1.5%)
(Missing)	87 (0.8%)	33 (0.5%)
Religious service attendance around age 12, n (%)		
At least once a week	4,730 (45.5%)	2,908 (44.6%)
One to three times a month	2,709 (26.1%)	1,766 (27.1%)
Less than once a month	2,184 (21.0%)	1,349 (20.7%)
Never	350 (3.4%)	209 (3.2%)
(Missing)	417 (4.0%)	284 (4.4%)
Relationship with mother when growing up, n (%)		
Very good	4,914 (47.3%)	3,196 (49.0%)
Somewhat good	4,971 (47.8%)	3,051 (46.8%)
Somewhat bad	278 (2.7%)	149 (2.3%)
Very bad	54 (0.5%)	27 (0.4%)
(Does not apply)	77 (0.7%)	33 (0.5%)
(Missing)	94 (0.9%)	60 (0.9%)
Relationship with father when growing up, n (%)		
Very good	4,238 (40.8%)	2,795 (42.9%)
Somewhat good	4,994 (48.1%)	3,023 (46.4%)
Somewhat bad	507 (4.9%)	294 (4.5%)
Very bad	88 (0.8%)	63 (1.0%)
(Does not apply)	398 (3.8%)	234 (3.6%)
(Missing)	164 (1.6%)	107 (1.6%)
Felt like an outsider in family when growing up, n (%)		
Yes	484 (4.7%)	314 (4.8%)
No	9,646 (92.9%)	6,067 (93.1%)
(Missing)	259 (2.5%)	136 (2.1%)
Experienced abuse when growing up, n (%)		
Yes	316 (3.0%)	206 (3.2%)
No	10,022 (96.5%)	6,287 (96.5%)
(Missing)	51 (0.5%)	23 (0.4%)
Self-rated health when growing up, n (%)		
Excellent	2,694 (25.9%)	1,777 (27.3%)
Very good	5,370 (51.7%)	3,284 (50.4%)
Good	1,783 (17.2%)	1,114 (17.1%)
Fair	399 (3.8%)	255 (3.9%)
Poor	109 (1.0%)	64 (1.0%)
(Missing)	34 (0.3%)	22 (0.3%)

Table S24a. Weighted summary statistics for demographic and childhood variables in Poland

**Characteristic**	**Wave 1**<br>N = 10,389	**Wave 2**<br>N = 6,516
Subjective financial status of family growing up, n (%)		
Lived comfortably	1,412 (13.6%)	803 (12.3%)
Got by	6,279 (60.4%)	4,015 (61.6%)
Found it difficult	2,142 (20.6%)	1,353 (20.8%)
Found it very difficult	461 (4.4%)	286 (4.4%)
(Missing)	95 (0.9%)	60 (0.9%)
Religious affiliation growing up, n (%)		
Christianity	9,867 (95.0%)	6,180 (94.8%)
Taoism	0 (0%)	0 (0%)
Confucianism	0 (0%)	0 (0%)
Primal, Animist, or Folk religion	5 (0.0%)	2 (0.0%)
Spiritism	0 (0%)	0 (0%)
Umbanda, Candomblé, and other African-derived religions	0 (0%)	0 (0%)
Chinese folk/traditional religion	0 (0%)	0 (0%)
Islam	3 (0.0%)	1 (0.0%)
Hinduism	0 (0%)	0 (0%)
Buddhism	2 (0.0%)	3 (0.0%)
Judaism	0 (0%)	0 (0%)
Sikhism	1 (0.0%)	1 (0.0%)
Baha'i	0 (0%)	0 (0%)
Jainism	0 (0%)	0 (0%)
Shinto	0 (0%)	0 (0%)
Some other religion	0 (0%)	0 (0%)
No religion/Atheist/Agnostic	483 (4.6%)	321 (4.9%)
(Missing)	28 (0.3%)	8 (0.1%)

Note. N (%); this table is based on non-imputed data. Cumulative percentages for variables may not add up to 100% due to rounding. Wave 1 characteristics weighted using the Gallup provided sampling weight, ANNUAL_WEIGHT_R2; Wave 2 characteristics weighted accounting for attrition by using the adjusted Wave 1 weight, ANNUAL_WEIGHT_R2, multiplied by the created attrition weight to account for dropout, to maintain nationally representative estimates for Wave 2 characteristics.

Table S24b. Weighted summary statistics for outcome variables in Poland

Outcome	Wave 1<br>N = 10,389	Wave 2<br>N = 6,516
Secure flourishing index		
Mean	7.6	7.7
Standard Deviation	1.3	1.2
Min, Max	0.3, 10.0	0.7, 10.0
(Missing)	525 (5.1%)	239 (3.7%)
Flourishing index		
Mean	7.7	7.8
Standard Deviation	1.3	1.2
Min, Max	0.3, 10.0	0.8, 10.0
(Missing)	462 (4.4%)	211 (3.2%)
Happiness & life satisfaction		
Mean	7.6	7.7
Standard Deviation	1.5	1.4
Min, Max	0.0, 10.0	0.0, 10.0
(Missing)	114 (1.1%)	33 (0.5%)
Physical & mental health		
Mean	7.9	8.0
Standard Deviation	1.7	1.6
Min, Max	0.0, 10.0	0.0, 10.0
(Missing)	40 (0.4%)	47 (0.7%)
Meaning & purpose		
Mean	7.8	7.9
Standard Deviation	1.6	1.5
Min, Max	0.0, 10.0	0.0, 10.0
(Missing)	92 (0.9%)	39 (0.6%)
Character & virtue		
Mean	7.4	7.6
Standard Deviation	1.6	1.4
Min, Max	0.0, 10.0	1.0, 10.0
(Missing)	94 (0.9%)	43 (0.7%)
Close social relationships		
Mean	7.7	7.7
Standard Deviation	1.7	1.6
Min, Max	0.0, 10.0	0.0, 10.0
(Missing)	209 (2.0%)	66 (1.0%)
Financial & material security		
Mean	7.2	7.6
Standard Deviation	2.2	2.0
Min, Max	0.0, 10.0	0.0, 10.0
(Missing)	99 (1.0%)	31 (0.5%)
Happiness		
Mean	7.6	7.7
Standard Deviation	1.6	1.5
Min, Max	0.0, 10.0	0.0, 10.0
(Missing)	14 (0.1%)	14 (0.2%)
Life satisfaction		
Mean	7.5	7.7
Standard Deviation	1.7	1.6
Min, Max	0.0, 10.0	0.0, 10.0
(Missing)	100 (1.0%)	21 (0.3%)
Current life evaluation		

Table S24b. Weighted summary statistics for outcome variables in Poland

Outcome	Wave 1<br>N = 10,389	Wave 2<br>N = 6,516
Mean	7.1	7.4
Standard Deviation	1.6	1.5
Min, Max	0.0, 10.0	0.0, 10.0
(Missing)	19 (0.2%)	6 (<0.1%)
Future life evaluation		
Mean	7.4	7.5
Standard Deviation	1.8	1.6
Min, Max	0.0, 10.0	0.0, 10.0
(Missing)	148 (1.4%)	100 (1.5%)
Optimism		
Mean	7.7	7.8
Standard Deviation	1.8	1.7
Min, Max	0.0, 10.0	0.0, 10.0
(Missing)	35 (0.3%)	29 (0.4%)
Freedom to pursue what's important		
Mean	7.6	7.6
Standard Deviation	1.9	1.7
Min, Max	0.0, 10.0	0.0, 10.0
(Missing)	59 (0.6%)	23 (0.4%)
Inner peace, n (%)		
Always	1,665 (16.0%)	861 (13.2%)
Often	6,448 (62.1%)	4,208 (64.6%)
Rarely	1,942 (18.7%)	1,263 (19.4%)
Never	255 (2.4%)	145 (2.2%)
(Missing)	79 (0.8%)	39 (0.6%)
Life balance, n (%)		
Always	1,644 (15.8%)	872 (13.4%)
Often	6,509 (62.7%)	4,119 (63.2%)
Rarely	1,915 (18.4%)	1,282 (19.7%)
Never	259 (2.5%)	193 (3.0%)
(Missing)	62 (0.6%)	49 (0.8%)
Sense of mastery, n (%)		
Always	2,927 (28.2%)	1,732 (26.6%)
Often	5,817 (56.0%)	3,718 (57.1%)
Rarely	1,293 (12.4%)	869 (13.3%)
Never	259 (2.5%)	145 (2.2%)
(Missing)	93 (0.9%)	53 (0.8%)
Meaningful activities		
Mean	7.7	7.8
Standard Deviation	1.7	1.6
Min, Max	0.0, 10.0	0.0, 10.0
(Missing)	57 (0.6%)	19 (0.3%)
Understanding purpose		
Mean	7.8	7.9
Standard Deviation	2.0	1.7
Min, Max	0.0, 10.0	0.0, 10.0
(Missing)	36 (0.4%)	20 (0.3%)
Self-rated mental health		
Mean	8.1	8.1
Standard Deviation	1.8	1.8
Min, Max	0.0, 10.0	0.0, 10.0

Table S24b. Weighted summary statistics for outcome variables in Poland

Outcome	Wave 1<br>N = 10,389	Wave 2<br>N = 6,516
(Missing)	20 (0.2%)	14 (0.2%)
Traumatic distress, n (%)		
A lot	308 (3.0%)	180 (2.8%)
Some	1,335 (12.9%)	859 (13.2%)
Not very much	3,013 (29.0%)	1,878 (28.8%)
Not at all	5,683 (54.7%)	3,574 (54.9%)
(Missing)	49 (0.5%)	26 (0.4%)
Depression symptoms composite, n (%)	1,415 (13.8%)	772 (12.0%)
(Missing)	136 (1.3%)	65 (1.0%)
Depression – feel hopeless, n (%)		
Nearly every day	235 (2.3%)	151 (2.3%)
More than half the days	656 (6.3%)	365 (5.6%)
Several days	2,495 (24.0%)	1,592 (24.4%)
Not at all	6,921 (66.6%)	4,373 (67.1%)
(Missing)	82 (0.8%)	36 (0.6%)
Depression – loss of interest, n (%)		
Nearly every day	372 (3.6%)	176 (2.7%)
More than half the days	1,148 (11.0%)	605 (9.3%)
Several days	3,197 (30.8%)	2,198 (33.7%)
Not at all	5,611 (54.0%)	3,505 (53.8%)
(Missing)	61 (0.6%)	33 (0.5%)
Anxiety symptoms composite, n (%)	1,264 (12.3%)	668 (10.4%)
(Missing)	101 (1.0%)	88 (1.3%)
Anxiety – feel on edge, n (%)		
Nearly every day	314 (3.0%)	173 (2.7%)
More than half the days	869 (8.4%)	458 (7.0%)
Several days	4,129 (39.7%)	2,543 (39.0%)
Not at all	5,019 (48.3%)	3,296 (50.6%)
(Missing)	59 (0.6%)	46 (0.7%)
Anxiety – cannot stop worrying, n (%)		
Nearly every day	248 (2.4%)	152 (2.3%)
More than half the days	666 (6.4%)	349 (5.4%)
Several days	2,313 (22.3%)	1,557 (23.9%)
Not at all	7,113 (68.5%)	4,413 (67.7%)
(Missing)	50 (0.5%)	45 (0.7%)
Suffering, n (%)		
A lot	221 (2.1%)	177 (2.7%)
Some	2,280 (21.9%)	1,698 (26.1%)
Not very much	3,728 (35.9%)	2,204 (33.8%)
Not at all	4,117 (39.6%)	2,406 (36.9%)
(Missing)	43 (0.4%)	31 (0.5%)
Relationship contentment		
Mean	7.8	7.8
Standard Deviation	1.8	1.7
Min, Max	0.0, 10.0	0.0, 10.0
(Missing)	119 (1.1%)	29 (0.4%)
Relationship satisfaction		
Mean	7.6	7.6
Standard Deviation	1.8	1.7
Min, Max	0.0, 10.0	0.0, 10.0
(Missing)	111 (1.1%)	41 (0.6%)

Table S24b. Weighted summary statistics for outcome variables in Poland

**Outcome**	**Wave 1**<br>N = 10,389	**Wave 2**<br>N = 6,516
Social support		
Mean	8.0	8.1
Standard Deviation	1.8	1.7
Min, Max	0.0, 10.0	0.0, 10.0
(Missing)	50 (0.5%)	45 (0.7%)
Intimate/close friend, n (%)		
Yes	8,908 (85.7%)	5,051 (77.5%)
No	1,314 (12.7%)	1,432 (22.0%)
(Missing)	167 (1.6%)	33 (0.5%)
Government approval, n (%)		
Strongly approve	1,360 (13.1%)	750 (11.5%)
Somewhat approve	2,002 (19.3%)	2,227 (34.2%)
Neither approve nor disapprove	2,103 (20.2%)	1,682 (25.8%)
Somewhat disapprove	1,948 (18.8%)	949 (14.6%)
Strongly disapprove	2,932 (28.2%)	854 (13.1%)
(Missing)	44 (0.4%)	54 (0.8%)
Say in government, n (%)		
Agree	2,701 (26.0%)	2,453 (37.6%)
Disagree	5,839 (56.2%)	3,179 (48.8%)
Unsure	1,792 (17.2%)	878 (13.5%)
(Missing)	57 (0.5%)	6 (0.1%)
Belonging in country		
Mean	7.7	7.9
Standard Deviation	2.2	1.7
Min, Max	0.0, 10.0	0.0, 10.0
(Missing)	39 (0.4%)	31 (0.5%)
City/place satisfaction, n (%)		
Satisfied	9,104 (87.6%)	5,741 (88.1%)
Dissatisfied	658 (6.3%)	347 (5.3%)
Unsure	614 (5.9%)	401 (6.1%)
(Missing)	13 (0.1%)	27 (0.4%)
Trust within country, n (%)		
All people	87 (0.8%)	42 (0.6%)
Most people	2,274 (21.9%)	1,472 (22.6%)
Some people	4,315 (41.5%)	2,832 (43.5%)
Not very many people	3,259 (31.4%)	1,890 (29.0%)
None	337 (3.2%)	239 (3.7%)
(Missing)	116 (1.1%)	41 (0.6%)
Number of children		
Mean	0.5	0.5
Standard Deviation	0.9	1.1
Min, Max	0.0, 11.0	0.0, 59.0
(Missing)	155 (1.5%)	969 (15%)
Community participation, n (%)		
More than once a week	270 (2.6%)	95 (1.5%)
Once a week	738 (7.1%)	394 (6.0%)
One to three times a month	1,157 (11.1%)	620 (9.5%)
A few times a year	3,051 (29.4%)	1,699 (26.1%)
Never	5,111 (49.2%)	3,692 (56.7%)
(Missing)	61 (0.6%)	17 (0.3%)
Religious attendance, n (%)		

Table S24b. Weighted summary statistics for outcome variables in Poland

Outcome	Wave 1<br>N = 10,389	Wave 2<br>N = 6,516
More than once a week	280 (2.7%)	123 (1.9%)
Once a week	3,277 (31.5%)	2,014 (30.9%)
One to three times a month	2,061 (19.8%)	1,192 (18.3%)
A few times a year	3,084 (29.7%)	2,019 (31.0%)
Never	1,609 (15.5%)	1,073 (16.5%)
(Missing)	78 (0.7%)	95 (1.5%)
Loneliness		
Mean	2.4	2.3
Standard Deviation	2.2	2.2
Min, Max	0.0, 10.0	0.0, 10.0
(Missing)	64 (0.6%)	17 (0.3%)
Perceived discrimination, n (%)		
Always	191 (1.8%)	158 (2.4%)
Often	1,108 (10.7%)	634 (9.7%)
Rarely	2,765 (26.6%)	1,473 (22.6%)
Never	6,302 (60.7%)	4,229 (64.9%)
(Missing)	23 (0.2%)	23 (0.3%)
Orientation to promote good		
Mean	7.9	8.1
Standard Deviation	1.6	1.5
Min, Max	0.0, 10.0	1.0, 10.0
(Missing)	58 (0.6%)	6 (<0.1%)
Delayed gratification		
Mean	6.8	7.1
Standard Deviation	2.3	2.0
Min, Max	0.0, 10.0	0.0, 10.0
(Missing)	56 (0.5%)	39 (0.6%)
Hope		
Mean	8.0	8.0
Standard Deviation	1.7	1.6
Min, Max	0.0, 10.0	0.0, 10.0
(Missing)	47 (0.5%)	39 (0.6%)
Gratitude		
Mean	7.4	7.5
Standard Deviation	1.8	1.7
Min, Max	0.0, 10.0	0.0, 10.0
(Missing)	119 (1.1%)	61 (0.9%)
Showing love/care		
Mean	7.8	7.8
Standard Deviation	2.0	1.9
Min, Max	0.0, 10.0	0.0, 10.0
(Missing)	40 (0.4%)	17 (0.3%)
Forgivingness, n (%)		
Always	1,706 (16.4%)	952 (14.6%)
Often	5,036 (48.5%)	3,094 (47.5%)
Rarely	2,823 (27.2%)	1,987 (30.5%)
Never	761 (7.3%)	450 (6.9%)
(Missing)	63 (0.6%)	32 (0.5%)
Charitable giving, n (%)		
Yes	2,100 (20.2%)	1,264 (19.4%)
No	8,234 (79.3%)	5,232 (80.3%)

Table S24b. Weighted summary statistics for outcome variables in Poland

Outcome	Wave 1<br>N = 10,389	Wave 2<br>N = 6,516
(Missing)	56 (0.5%)	21 (0.3%)
Helping strangers, n (%)		
Yes	2,638 (25.4%)	1,585 (24.3%)
No	7,546 (72.6%)	4,907 (75.3%)
(Missing)	205 (2.0%)	25 (0.4%)
Volunteering, n (%)		
Yes	871 (8.4%)	431 (6.6%)
No	9,443 (90.9%)	6,066 (93.1%)
(Missing)	75 (0.7%)	19 (0.3%)
Self-rated physical health		
Mean	7.7	7.8
Standard Deviation	1.9	1.7
Min, Max	0.0, 10.0	0.0, 10.0
(Missing)	20 (0.2%)	35 (0.5%)
Health problems, n (%)		
Yes	1,341 (12.9%)	921 (14.1%)
No	8,976 (86.4%)	5,469 (83.9%)
(Missing)	71 (0.7%)	127 (1.9%)
Pain in past 4 weeks, n (%)		
A lot	490 (4.7%)	270 (4.1%)
Some	2,810 (27.0%)	1,859 (28.5%)
Not very much	3,211 (30.9%)	1,983 (30.4%)
None at all	3,854 (37.1%)	2,397 (36.8%)
(Missing)	24 (0.2%)	7 (0.1%)
Number of cigarettes per day		
Mean	3.6	3.4
Standard Deviation	6.8	6.8
Min, Max	0.0, 97.0	0.0, 80.0
(Missing)	106 (1.0%)	224 (3.4%)
Number of drinks per week		
Mean	2.0	1.9
Standard Deviation	3.4	3.1
Min, Max	0.0, 97.0	0.0, 97.0
(Missing)	130 (1.3%)	222 (3.4%)
Days exercise per week		
Mean	1.4	1.3
Standard Deviation	1.9	1.8
Min, Max	0.0, 7.0	0.0, 7.0
(Missing)	108 (1.0%)	108 (1.7%)
Financial security		
Mean	7.0	7.5
Standard Deviation	2.4	2.1
Min, Max	0.0, 10.0	0.0, 10.0
(Missing)	36 (0.3%)	16 (0.2%)
Material security		
Mean	7.4	7.7
Standard Deviation	2.4	2.1
Min, Max	0.0, 10.0	0.0, 10.0
(Missing)	81 (0.8%)	16 (0.2%)
Educational attainment (16+ years), n (%)		
Up to 8	742 (7.1%)	171 (2.6%)

Table S24b. Weighted summary statistics for outcome variables in Poland

Outcome	Wave 1<br>N = 10,389	Wave 2<br>N = 6,516
9-15	6,711 (64.6%)	4,347 (66.7%)
16+	2,934 (28.2%)	1,998 (30.7%)
(Missing)	1 (0.0%)	0 (0%)
Currently employed, n (%)		
Employed for an employer	5,689 (54.8%)	3,654 (56.1%)
Self-employed	652 (6.3%)	348 (5.3%)
Retired	2,494 (24.0%)	1,571 (24.1%)
Student	561 (5.4%)	295 (4.5%)
Homemaker	371 (3.6%)	169 (2.6%)
Unemployed and looking for a job	294 (2.8%)	114 (1.7%)
None of these/Other	204 (2.0%)	175 (2.7%)
(Missing)	124 (1.2%)	191 (2.9%)
Financially comfortable/getting by, n (%)		
Living comfortably on present income	1,798 (17.3%)	1,013 (15.5%)
Getting by on present income	6,332 (60.9%)	4,283 (65.7%)
Finding it difficult on present income	1,786 (17.2%)	981 (15.1%)
Finding it very difficult on present income	350 (3.4%)	163 (2.5%)
(Missing)	123 (1.2%)	77 (1.2%)
Own home, n (%)		
Someone in this household owns this home	7,497 (72.2%)	5,516 (84.7%)
Someone in this household rents this home	1,334 (12.8%)	484 (7.4%)
Both	265 (2.6%)	101 (1.5%)
Neither	1,207 (11.6%)	300 (4.6%)
Rent	0 (0%)	0 (0%)
Own	0 (0%)	0 (0%)
Something else	0 (0%)	0 (0%)
(Missing)	87 (0.8%)	115 (1.8%)
Religious/spiritual connection, n (%)		
Always	2,013 (19.4%)	1,132 (17.4%)
Often	3,731 (35.9%)	2,122 (32.6%)
Rarely	3,042 (29.3%)	2,028 (31.1%)
Never	1,537 (14.8%)	1,165 (17.9%)
(Missing)	66 (0.6%)	70 (1.1%)
Belief in life after death, n (%)		
Yes	5,884 (56.6%)	3,754 (57.6%)
No	2,186 (21.0%)	1,274 (19.6%)
Unsure	2,254 (21.7%)	1,422 (21.8%)
(Missing)	65 (0.6%)	66 (1.0%)
Transformative religious experience, n (%)		
Yes	854 (8.2%)	502 (7.7%)
No	9,495 (91.4%)	5,985 (91.8%)
(Missing)	40 (0.4%)	29 (0.4%)
Religious reading or listening, n (%)		
More than once a day	94 (0.9%)	71 (1.1%)
About once a day	734 (7.1%)	368 (5.6%)
Sometimes	5,096 (49.1%)	3,105 (47.6%)
Never	4,385 (42.2%)	2,888 (44.3%)
(Missing)	80 (0.8%)	84 (1.3%)
Prayer or meditation, n (%)		
More than once a day	700 (6.7%)	313 (4.8%)
About once a day	2,207 (21.2%)	1,327 (20.4%)

Table S24b. Weighted summary statistics for outcome variables in Poland

**Outcome**	**Wave 1**<br>N = 10,389	**Wave 2**<br>N = 6,516
Sometimes	4,746 (45.7%)	2,985 (45.8%)
Never	2,684 (25.8%)	1,828 (28.1%)
(Missing)	52 (0.5%)	63 (1.0%)
Belief in God/gods/spiritual forces, n (%)		
One God	7,938 (76.4%)	4,985 (76.5%)
More than one god	99 (1.0%)	61 (0.9%)
An impersonal spiritual force	448 (4.3%)	348 (5.3%)
None of these	899 (8.7%)	481 (7.4%)
Unsure	939 (9.0%)	561 (8.6%)
(Missing)	65 (0.6%)	80 (1.2%)
Religious centrality, n (%)		
Agree	4,500 (43.3%)	2,753 (42.2%)
Disagree	2,421 (23.3%)	1,594 (24.5%)
Not relevant	1,235 (11.9%)	794 (12.2%)
Unsure	2,126 (20.5%)	1,338 (20.5%)
(Missing)	107 (1.0%)	37 (0.6%)
Religious/spiritual comfort, n (%)		
Agree	4,909 (47.3%)	3,003 (46.1%)
Disagree	2,006 (19.3%)	1,392 (21.4%)
Not relevant	1,195 (11.5%)	781 (12.0%)
Unsure	2,158 (20.8%)	1,297 (19.9%)
(Missing)	121 (1.2%)	43 (0.7%)
Feel loved by God, n (%)		
Agree	4,935 (47.5%)	2,940 (45.1%)
Disagree	1,569 (15.1%)	1,039 (15.9%)
Not relevant	1,176 (11.3%)	772 (11.9%)
Unsure	2,557 (24.6%)	1,707 (26.2%)
(Missing)	152 (1.5%)	59 (0.9%)
Feel punished by God, n (%)		
Agree	1,756 (16.9%)	1,016 (15.6%)
Disagree	4,620 (44.5%)	2,934 (45.0%)
Not relevant	1,266 (12.2%)	796 (12.2%)
Unsure	2,591 (24.9%)	1,710 (26.2%)
(Missing)	156 (1.5%)	61 (0.9%)
Experienced religious criticism, n (%)		
Agree	1,257 (12.1%)	706 (10.8%)
Disagree	4,970 (47.8%)	2,996 (46.0%)
Not relevant	2,001 (19.3%)	1,276 (19.6%)
Unsure	2,013 (19.4%)	1,475 (22.6%)
(Missing)	148 (1.4%)	64 (1.0%)
Faith-sharing, n (%)		
Agree	3,036 (29.2%)	1,577 (24.2%)
Disagree	3,798 (36.6%)	2,806 (43.1%)
Not relevant	1,655 (15.9%)	943 (14.5%)
Unsure	1,810 (17.4%)	1,158 (17.8%)
(Missing)	90 (0.9%)	33 (0.5%)

Note. N (%); this table is based on non-imputed data. Cumulative percentages for variables may not add up to 100% due to rounding. Wave 1 characteristics weighted using the Gallup provided sampling weight, ANNUAL_WEIGHT_R2; Wave 2 characteristics weighted accounting for attrition by using the adjusted Wave 1 weight, ANNUAL_WEIGHT_R2, multiplied by the created attrition weight to account for dropout, to maintain nationally representative estimates for Wave 2 characteristics.

Table S24c. Unweighted summary statistics for demographic and childhood variables in Poland by retention status

Characteristic	Attritors–Not Observed in Wave 2<br>N = 4,035	Retained–Observed in Wave 2<br>N = 6,366
Forgivingness, n (%)		
Always	483 (12.0%)	1,218 (19.1%)
Often	2,019 (50.0%)	3,026 (47.5%)
Rarely	1,188 (29.4%)	1,642 (25.8%)
Never	298 (7.4%)	463 (7.3%)
(Missing)	47 (1.2%)	17 (0.3%)
Year of birth, n (%)		
1943 or earlier (current age: 80+ years)	49 (1.2%)	80 (1.3%)
1943-1953 (current age: 70-79 years)	238 (5.9%)	549 (8.6%)
1953-1963 (current age: 60-69 years)	616 (15.3%)	1,350 (21.2%)
1963-1973 (current age: 50-59 years)	616 (15.3%)	962 (15.1%)
1973-1983 (current age: 40-49 years)	821 (20.3%)	1,081 (17.0%)
1983-1993 (current age: 30-39 years)	895 (22.2%)	1,243 (19.5%)
1993-1998 (current age: 25-29 years)	328 (8.1%)	469 (7.4%)
1998-2005 (current age: 18-24 years)	474 (11.7%)	630 (9.9%)
(Missing)	0 (0%)	1 (0.0%)
Age of participant		
Mean	44.7	47.6
Standard Deviation	16.1	16.9
Min, Max	18.0, 90.0	18.0, 92.0
Gender, n (%)		
Male	1,988 (49.3%)	2,969 (46.6%)
Female	2,036 (50.5%)	3,378 (53.1%)
Other	3 (0.1%)	1 (0.0%)
(Missing)	8 (0.2%)	17 (0.3%)
Respondent marital status, n (%)		
Single/Never been married	803 (19.9%)	1,112 (17.5%)
Married	2,125 (52.7%)	3,885 (61.0%)
Separated	56 (1.4%)	52 (0.8%)
Divorced	252 (6.2%)	268 (4.2%)
Widowed	413 (10.2%)	520 (8.2%)
Domestic partner	278 (6.9%)	269 (4.2%)
(Missing)	108 (2.7%)	260 (4.1%)
Education (years), n (%)		
Up to 8	346 (8.6%)	400 (6.3%)
9-15	2,609 (64.7%)	4,110 (64.6%)
16+	1,079 (26.7%)	1,856 (29.2%)
(Missing)	1 (0.0%)	0 (0%)
Employment status, n (%)		
Employed for an employer	2,284 (56.6%)	3,414 (53.6%)
Self-employed	283 (7.0%)	371 (5.8%)
Retired	812 (20.1%)	1,679 (26.4%)
Student	222 (5.5%)	340 (5.3%)
Homemaker	155 (3.8%)	217 (3.4%)
Unemployed and looking for a job	132 (3.3%)	162 (2.6%)
None of these/Other	124 (3.1%)	82 (1.3%)
(Missing)	24 (0.6%)	100 (1.6%)
Current religious service attendance, n (%)		
More than once a week	117 (2.9%)	163 (2.6%)

Table S24c. Unweighted summary statistics for demographic and childhood variables in Poland by retention status

Characteristic	Attritors–Not Observed in Wave 2<br>N = 4,035	Retained–Observed in Wave 2<br>N = 6,366
Once a week	984 (24.4%)	2,285 (35.9%)
One to three times a month	756 (18.7%)	1,306 (20.5%)
A few times a year	1,344 (33.3%)	1,749 (27.5%)
Never	797 (19.8%)	821 (12.9%)
(Missing)	37 (0.9%)	41 (0.6%)
Immigration status, n (%)		
Born in this country	3,947 (97.8%)	6,320 (99.3%)
Born in another country	73 (1.8%)	40 (0.6%)
(Missing)	15 (0.4%)	6 (0.1%)
Parental marital status around age 12, n (%)		
Parents were married	3,269 (81.0%)	5,711 (89.7%)
Parents were divorced	272 (6.7%)	326 (5.1%)
Parents were never married	121 (3.0%)	82 (1.3%)
One or both of them had died	166 (4.1%)	139 (2.2%)
Unsure	145 (3.6%)	82 (1.3%)
(Missing)	63 (1.5%)	26 (0.4%)
Religious service attendance around age 12, n (%)		
At least once a week	1,642 (40.7%)	3,085 (48.5%)
One to three times a month	1,069 (26.5%)	1,643 (25.8%)
Less than once a month	1,001 (24.8%)	1,191 (18.7%)
Never	188 (4.7%)	165 (2.6%)
(Missing)	135 (3.4%)	281 (4.4%)
Relationship with mother when growing up, n (%)		
Very good	1,586 (39.3%)	3,321 (52.2%)
Somewhat good	2,167 (53.7%)	2,819 (44.3%)
Somewhat bad	154 (3.8%)	127 (2.0%)
Very bad	35 (0.9%)	20 (0.3%)
(Does not apply)	54 (1.3%)	24 (0.4%)
(Missing)	39 (1.0%)	55 (0.9%)
Relationship with father when growing up, n (%)		
Very good	1,310 (32.5%)	2,919 (45.9%)
Somewhat good	2,147 (53.2%)	2,861 (44.9%)
Somewhat bad	268 (6.6%)	243 (3.8%)
Very bad	45 (1.1%)	43 (0.7%)
(Does not apply)	196 (4.9%)	204 (3.2%)
(Missing)	69 (1.7%)	96 (1.5%)
Felt like an outsider in family when growing up, n (%)		
Yes	223 (5.5%)	262 (4.1%)
No	3,659 (90.7%)	5,995 (94.2%)
(Missing)	153 (3.8%)	108 (1.7%)
Experienced abuse when growing up, n (%)		
Yes	144 (3.6%)	173 (2.7%)
No	3,862 (95.7%)	6,170 (96.9%)
(Missing)	29 (0.7%)	23 (0.4%)
Self-rated health when growing up, n (%)		
Excellent	845 (20.9%)	1,844 (29.0%)
Very good	2,155 (53.4%)	3,224 (50.6%)

Table S24c. Unweighted summary statistics for demographic and childhood variables in Poland by retention status

Characteristic	Attritors–Not Observed in Wave 2<br>N = 4,035	Retained–Observed in Wave 2<br>N = 6,366
Good	775 (19.2%)	1,014 (15.9%)
Fair	190 (4.7%)	210 (3.3%)
Poor	54 (1.3%)	55 (0.9%)
(Missing)	15 (0.4%)	18 (0.3%)
Subjective financial status of family growing up, n (%)		
Lived comfortably	630 (15.6%)	786 (12.4%)
Got by	2,366 (58.6%)	3,917 (61.5%)
Found it difficult	797 (19.8%)	1,346 (21.1%)
Found it very difficult	195 (4.8%)	267 (4.2%)
(Missing)	47 (1.2%)	49 (0.8%)
Religious affiliation growing up, n (%)		
Christianity	3,828 (94.9%)	6,049 (95.0%)
Taoism	0 (0%)	0 (0%)
Confucianism	0 (0%)	0 (0%)
Primal, Animist, or Folk religion	3 (0.1%)	2 (0.0%)
Spiritism	0 (0%)	0 (0%)
Umbanda, Candomblé, and other African-derived religions	0 (0%)	0 (0%)
Chinese folk/traditional religion	0 (0%)	0 (0%)
Islam	1 (0.0%)	1 (0.0%)
Hinduism	0 (0%)	0 (0%)
Buddhism	1 (0.0%)	1 (0.0%)
Judaism	0 (0%)	0 (0%)
Sikhism	0 (0%)	1 (0.0%)
Baha'i	0 (0%)	0 (0%)
Jainism	0 (0%)	0 (0%)
Shinto	0 (0%)	0 (0%)
Some other religion	0 (0%)	0 (0%)
No religion/Atheist/Agnostic	179 (4.4%)	304 (4.8%)
(Missing)	22 (0.5%)	7 (0.1%)

Note. N (%); this table is based on non-imputed data. Cumulative percentages for variables may not add up to 100% due to rounding.

Table S24d. Unweighted summary statistics for Wave 1 outcome variables in Poland by retention status.

**Outcome**	**Attritors–Not Observed in Wave 2** N = 4,035	**Retained–Observed in Wave 2** N = 6,366
Secure flourishing index		
Mean	7.3	7.7
Standard Deviation	1.4	1.2
Min, Max	1.3, 10.0	0.3, 10.0
(Missing)	313 (7.8%)	217 (3.4%)
Flourishing index		
Mean	7.4	7.8
Standard Deviation	1.4	1.2
Min, Max	1.4, 10.0	0.3, 10.0
(Missing)	278 (6.9%)	189 (3.0%)
Happiness & life satisfaction		
Mean	7.2	7.8
Standard Deviation	1.6	1.5
Min, Max	0.0, 10.0	0.0, 10.0
(Missing)	72 (1.8%)	43 (0.7%)
Physical & mental health		
Mean	7.6	8.1
Standard Deviation	1.8	1.6
Min, Max	0.0, 10.0	0.0, 10.0
(Missing)	24 (0.6%)	16 (0.3%)
Meaning & purpose		
Mean	7.6	7.9
Standard Deviation	1.7	1.6
Min, Max	0.0, 10.0	0.0, 10.0
(Missing)	53 (1.3%)	39 (0.6%)
Character & virtue		
Mean	7.3	7.4
Standard Deviation	1.7	1.6
Min, Max	0.0, 10.0	0.0, 10.0
(Missing)	72 (1.8%)	24 (0.4%)
Close social relationships		
Mean	7.5	7.8
Standard Deviation	1.8	1.6
Min, Max	0.0, 10.0	0.0, 10.0
(Missing)	116 (2.9%)	95 (1.5%)
Financial & material security		
Mean	6.8	7.4
Standard Deviation	2.3	2.2
Min, Max	0.0, 10.0	0.0, 10.0
(Missing)	62 (1.5%)	38 (0.6%)
Happiness		
Mean	7.3	7.8
Standard Deviation	1.6	1.5
Min, Max	0.0, 10.0	0.0, 10.0
(Missing)	11 (0.3%)	4 (<0.1%)
Life satisfaction		
Mean	7.2	7.7
Standard Deviation	1.8	1.6
Min, Max	0.0, 10.0	0.0, 10.0

Table S24d. Unweighted summary statistics for Wave 1 outcome variables in Poland by retention status.

**Outcome**	**Attritors–Not Observed in Wave 2** N = 4,035	**Retained–Observed in Wave 2** N = 6,366
(Missing)	62 (1.5%)	39 (0.6%)
Current life evaluation		
Mean	7.0	7.2
Standard Deviation	1.7	1.6
Min, Max	0.0, 10.0	0.0, 10.0
(Missing)	4 (0.1%)	14 (0.2%)
Future life evaluation		
Mean	7.4	7.4
Standard Deviation	1.8	1.7
Min, Max	0.0, 10.0	0.0, 10.0
(Missing)	80 (2.0%)	69 (1.1%)
Optimism		
Mean	7.6	7.8
Standard Deviation	1.9	1.7
Min, Max	0.0, 10.0	0.0, 10.0
(Missing)	29 (0.7%)	7 (0.1%)
Freedom to pursue what's important		
Mean	7.4	7.8
Standard Deviation	2.0	1.8
Min, Max	0.0, 10.0	0.0, 10.0
(Missing)	32 (0.8%)	27 (0.4%)
Inner peace, n (%)		
Always	455 (11.3%)	1,204 (18.9%)
Often	2,473 (61.3%)	3,981 (62.5%)
Rarely	925 (22.9%)	1,026 (16.1%)
Never	134 (3.3%)	122 (1.9%)
(Missing)	48 (1.2%)	32 (0.5%)
Life balance, n (%)		
Always	491 (12.2%)	1,149 (18.1%)
Often	2,466 (61.1%)	4,048 (63.6%)
Rarely	911 (22.6%)	1,013 (15.9%)
Never	129 (3.2%)	132 (2.1%)
(Missing)	39 (1.0%)	24 (0.4%)
Sense of mastery, n (%)		
Always	932 (23.1%)	1,990 (31.3%)
Often	2,239 (55.5%)	3,584 (56.3%)
Rarely	687 (17.0%)	615 (9.7%)
Never	127 (3.2%)	133 (2.1%)
(Missing)	49 (1.2%)	44 (0.7%)
Meaningful activities		
Mean	7.5	7.9
Standard Deviation	1.8	1.6
Min, Max	0.0, 10.0	0.0, 10.0
(Missing)	43 (1.1%)	15 (0.2%)
Understanding purpose		
Mean	7.6	7.9
Standard Deviation	2.0	2.0
Min, Max	0.0, 10.0	0.0, 10.0
(Missing)	12 (0.3%)	24 (0.4%)

Table S24d. Unweighted summary statistics for Wave 1 outcome variables in Poland by retention status.

Outcome	Attritors–Not Observed in Wave 2<br>N = 4,035	Retained–Observed in Wave 2<br>N = 6,366
Self-rated mental health		
Mean	7.8	8.3
Standard Deviation	1.9	1.7
Min, Max	0.0, 10.0	0.0, 10.0
(Missing)	14 (0.4%)	6 (<0.1%)
Traumatic distress, n (%)		
A lot	138 (3.4%)	170 (2.7%)
Some	606 (15.0%)	734 (11.5%)
Not very much	1,363 (33.8%)	1,661 (26.1%)
Not at all	1,902 (47.1%)	3,776 (59.3%)
(Missing)	26 (0.6%)	24 (0.4%)
Depression symptoms composite, n (%)	781 (19.7%)	645 (10.2%)
(Missing)	64 (1.6%)	72 (1.1%)
Depression – feel hopeless, n (%)		
Nearly every day	138 (3.4%)	99 (1.6%)
More than half the days	390 (9.7%)	272 (4.3%)
Several days	1,073 (26.6%)	1,429 (22.4%)
Not at all	2,394 (59.3%)	4,523 (71.1%)
(Missing)	40 (1.0%)	42 (0.7%)
Depression – loss of interest, n (%)		
Nearly every day	196 (4.9%)	178 (2.8%)
More than half the days	601 (14.9%)	555 (8.7%)
Several days	1,396 (34.6%)	1,811 (28.5%)
Not at all	1,813 (44.9%)	3,790 (59.5%)
(Missing)	30 (0.7%)	31 (0.5%)
Anxiety symptoms composite, n (%)	678 (17.0%)	595 (9.4%)
(Missing)	49 (1.2%)	53 (0.8%)
Anxiety – feel on edge, n (%)		
Nearly every day	171 (4.2%)	146 (2.3%)
More than half the days	465 (11.5%)	410 (6.4%)
Several days	1,727 (42.8%)	2,411 (37.9%)
Not at all	1,641 (40.7%)	3,370 (52.9%)
(Missing)	30 (0.8%)	29 (0.5%)
Anxiety – cannot stop worrying, n (%)		
Nearly every day	135 (3.3%)	115 (1.8%)
More than half the days	360 (8.9%)	310 (4.9%)
Several days	1,060 (26.3%)	1,262 (19.8%)
Not at all	2,456 (60.9%)	4,653 (73.1%)
(Missing)	24 (0.6%)	26 (0.4%)
Suffering, n (%)		
A lot	122 (3.0%)	101 (1.6%)
Some	892 (22.1%)	1,391 (21.8%)
Not very much	1,633 (40.5%)	2,107 (33.1%)
Not at all	1,372 (34.0%)	2,740 (43.0%)
(Missing)	16 (0.4%)	27 (0.4%)
Relationship contentment		
Mean	7.6	7.9
Standard Deviation	1.9	1.7
Min, Max	0.0, 10.0	0.0, 10.0

Table S24d. Unweighted summary statistics for Wave 1 outcome variables in Poland by retention status.

Outcome	Attritors–Not Observed in Wave 2 N = 4,035	Retained–Observed in Wave 2 N = 6,366
(Missing)	66 (1.6%)	54 (0.8%)
Relationship satisfaction		
Mean	7.5	7.7
Standard Deviation	2.0	1.8
Min, Max	0.0, 10.0	0.0, 10.0
(Missing)	66 (1.6%)	45 (0.7%)
Social support		
Mean	7.7	8.3
Standard Deviation	1.9	1.7
Min, Max	0.0, 10.0	0.0, 10.0
(Missing)	20 (0.5%)	30 (0.5%)
Intimate/close friend, n (%)		
Yes	3,313 (82.1%)	5,599 (88.0%)
No	625 (15.5%)	695 (10.9%)
(Missing)	96 (2.4%)	72 (1.1%)
Government approval, n (%)		
Strongly approve	409 (10.1%)	947 (14.9%)
Somewhat approve	793 (19.6%)	1,212 (19.0%)
Neither approve nor disapprove	874 (21.7%)	1,233 (19.4%)
Somewhat disapprove	835 (20.7%)	1,119 (17.6%)
Strongly disapprove	1,104 (27.4%)	1,830 (28.7%)
(Missing)	21 (0.5%)	24 (0.4%)
Say in government, n (%)		
Agree	1,002 (24.8%)	1,700 (26.7%)
Disagree	2,167 (53.7%)	3,675 (57.7%)
Unsure	834 (20.7%)	965 (15.2%)
(Missing)	32 (0.8%)	25 (0.4%)
Belonging in country		
Mean	7.3	8.0
Standard Deviation	2.4	2.0
Min, Max	0.0, 10.0	0.0, 10.0
(Missing)	25 (0.6%)	15 (0.2%)
City/place satisfaction, n (%)		
Satisfied	3,418 (84.7%)	5,692 (89.4%)
Dissatisfied	313 (7.8%)	347 (5.5%)
Unsure	296 (7.3%)	322 (5.1%)
(Missing)	8 (0.2%)	4 (0.1%)
Trust within country, n (%)		
All people	45 (1.1%)	43 (0.7%)
Most people	806 (20.0%)	1,467 (23.1%)
Some people	1,725 (42.8%)	2,597 (40.8%)
Not very many people	1,242 (30.8%)	2,020 (31.7%)
None	153 (3.8%)	186 (2.9%)
(Missing)	64 (1.6%)	53 (0.8%)
Number of children		
Mean	0.5	0.5
Standard Deviation	0.9	0.9
Min, Max	0.0, 6.0	0.0, 11.0
(Missing)	38 (0.9%)	117 (1.8%)

Table S24d. Unweighted summary statistics for Wave 1 outcome variables in Poland by retention status.

**Outcome**	**Attritors–Not Observed in Wave 2**<br>N = 4,035	**Retained–Observed in Wave 2**<br>N = 6,366
Community participation, n (%)		
More than once a week	143 (3.5%)	129 (2.0%)
Once a week	405 (10.0%)	338 (5.3%)
One to three times a month	473 (11.7%)	687 (10.8%)
A few times a year	1,112 (27.6%)	1,939 (30.5%)
Never	1,865 (46.2%)	3,248 (51.0%)
(Missing)	38 (0.9%)	24 (0.4%)
Religious attendance, n (%)		
More than once a week	117 (2.9%)	163 (2.6%)
Once a week	984 (24.4%)	2,285 (35.9%)
One to three times a month	756 (18.7%)	1,306 (20.5%)
A few times a year	1,344 (33.3%)	1,749 (27.5%)
Never	797 (19.8%)	821 (12.9%)
(Missing)	37 (0.9%)	41 (0.6%)
Loneliness		
Mean	2.9	2.1
Standard Deviation	2.3	2.1
Min, Max	0.0, 10.0	0.0, 10.0
(Missing)	51 (1.3%)	14 (0.2%)
Perceived discrimination, n (%)		
Always	92 (2.3%)	99 (1.6%)
Often	634 (15.7%)	483 (7.6%)
Rarely	1,254 (31.1%)	1,522 (23.9%)
Never	2,039 (50.5%)	4,254 (66.8%)
(Missing)	15 (0.4%)	8 (0.1%)
Orientation to promote good		
Mean	7.7	8.1
Standard Deviation	1.7	1.5
Min, Max	0.0, 10.0	0.0, 10.0
(Missing)	46 (1.1%)	13 (0.2%)
Delayed gratification		
Mean	6.9	6.7
Standard Deviation	2.2	2.4
Min, Max	0.0, 10.0	0.0, 10.0
(Missing)	43 (1.1%)	13 (0.2%)
Hope		
Mean	7.7	8.1
Standard Deviation	1.9	1.7
Min, Max	0.0, 10.0	0.0, 10.0
(Missing)	35 (0.9%)	12 (0.2%)
Gratitude		
Mean	7.2	7.5
Standard Deviation	2.0	1.7
Min, Max	0.0, 10.0	0.0, 10.0
(Missing)	95 (2.4%)	26 (0.4%)
Showing love/care		
Mean	7.6	8.0
Standard Deviation	2.0	1.9
Min, Max	0.0, 10.0	0.0, 10.0

Table S24d. Unweighted summary statistics for Wave 1 outcome variables in Poland by retention status.

Outcome	Attritors–Not Observed in Wave 2<br>N = 4,035	Retained–Observed in Wave 2<br>N = 6,366
(Missing)	33 (0.8%)	7 (0.1%)
Forgivingness, n (%)		
Always	483 (12.0%)	1,218 (19.1%)
Often	2,019 (50.0%)	3,026 (47.5%)
Rarely	1,188 (29.4%)	1,642 (25.8%)
Never	298 (7.4%)	463 (7.3%)
(Missing)	47 (1.2%)	17 (0.3%)
Charitable giving, n (%)		
Yes	859 (21.3%)	1,245 (19.6%)
No	3,140 (77.8%)	5,101 (80.1%)
(Missing)	36 (0.9%)	20 (0.3%)
Helping strangers, n (%)		
Yes	1,187 (29.4%)	1,461 (22.9%)
No	2,692 (66.7%)	4,853 (76.2%)
(Missing)	156 (3.9%)	52 (0.8%)
Volunteering, n (%)		
Yes	387 (9.6%)	486 (7.6%)
No	3,601 (89.2%)	5,850 (91.9%)
(Missing)	47 (1.2%)	29 (0.5%)
Self-rated physical health		
Mean	7.4	7.9
Standard Deviation	2.0	1.8
Min, Max	0.0, 10.0	0.0, 10.0
(Missing)	10 (0.2%)	10 (0.2%)
Health problems, n (%)		
Yes	574 (14.2%)	771 (12.1%)
No	3,428 (84.9%)	5,556 (87.3%)
(Missing)	33 (0.8%)	39 (0.6%)
Pain in past 4 weeks, n (%)		
A lot	240 (6.0%)	252 (4.0%)
Some	1,068 (26.5%)	1,744 (27.4%)
Not very much	1,437 (35.6%)	1,785 (28.0%)
None at all	1,278 (31.7%)	2,571 (40.4%)
(Missing)	11 (0.3%)	13 (0.2%)
Number of cigarettes per day		
Mean	3.8	3.4
Standard Deviation	6.5	6.9
Min, Max	0.0, 96.0	0.0, 97.0
(Missing)	63 (1.6%)	43 (0.7%)
Number of drinks per week		
Mean	2.4	1.8
Standard Deviation	4.2	2.8
Min, Max	0.0, 97.0	0.0, 50.0
(Missing)	89 (2.2%)	43 (0.7%)
Days exercise per week		
Mean	1.6	1.3
Standard Deviation	2.0	1.8
Min, Max	0.0, 7.0	0.0, 7.0
(Missing)	52 (1.3%)	56 (0.9%)

Table S24d. Unweighted summary statistics for Wave 1 outcome variables in Poland by retention status.

**Outcome**	**Attritors–Not Observed in Wave 2** N = 4,035	**Retained–Observed in Wave 2** N = 6,366
Financial security		
Mean	6.7	7.2
Standard Deviation	2.4	2.3
Min, Max	0.0, 10.0	0.0, 10.0
(Missing)	22 (0.6%)	14 (0.2%)
Material security		
Mean	7.0	7.6
Standard Deviation	2.4	2.3
Min, Max	0.0, 10.0	0.0, 10.0
(Missing)	57 (1.4%)	25 (0.4%)
Educational attainment (16+ years), n (%)		
Up to 8	346 (8.6%)	400 (6.3%)
9-15	2,609 (64.7%)	4,110 (64.6%)
16+	1,079 (26.7%)	1,856 (29.2%)
(Missing)	1 (0.0%)	0 (0%)
Currently employed, n (%)		
Employed for an employer	2,284 (56.6%)	3,414 (53.6%)
Self-employed	283 (7.0%)	371 (5.8%)
Retired	812 (20.1%)	1,679 (26.4%)
Student	222 (5.5%)	340 (5.3%)
Homemaker	155 (3.8%)	217 (3.4%)
Unemployed and looking for a job	132 (3.3%)	162 (2.6%)
None of these/Other	124 (3.1%)	82 (1.3%)
(Missing)	24 (0.6%)	100 (1.6%)
Financially comfortable/getting by, n (%)		
Living comfortably on present income	757 (18.8%)	1,046 (16.4%)
Getting by on present income	2,245 (55.6%)	4,085 (64.2%)
Finding it difficult on present income	743 (18.4%)	1,047 (16.4%)
Finding it very difficult on present income	219 (5.4%)	135 (2.1%)
(Missing)	71 (1.8%)	53 (0.8%)
Own home, n (%)		
Someone in this household owns this home	2,626 (65.1%)	4,868 (76.5%)
Someone in this household rents this home	698 (17.3%)	644 (10.1%)
Both	179 (4.4%)	90 (1.4%)
Neither	481 (11.9%)	728 (11.4%)
Rent	0 (0%)	0 (0%)
Own	0 (0%)	0 (0%)
Something else	0 (0%)	0 (0%)
(Missing)	51 (1.3%)	36 (0.6%)
Religious/spiritual connection, n (%)		
Always	553 (13.7%)	1,453 (22.8%)
Often	1,416 (35.1%)	2,318 (36.4%)
Rarely	1,360 (33.7%)	1,693 (26.6%)
Never	667 (16.5%)	875 (13.7%)
(Missing)	40 (1.0%)	27 (0.4%)
Belief in life after death, n (%)		
Yes	1,833 (45.4%)	4,039 (63.4%)
No	1,157 (28.7%)	1,045 (16.4%)
Unsure	1,008 (25.0%)	1,254 (19.7%)

Table S24d. Unweighted summary statistics for Wave 1 outcome variables in Poland by retention status.

Outcome	Attritors–Not Observed in Wave 2<br>N = 4,035	Retained–Observed in Wave 2<br>N = 6,366
(Missing)	37 (0.9%)	29 (0.5%)
Transformative religious experience, n (%)		
Yes	385 (9.5%)	472 (7.4%)
No	3,628 (89.9%)	5,876 (92.3%)
(Missing)	22 (0.6%)	18 (0.3%)
Religious reading or listening, n (%)		
More than once a day	51 (1.3%)	44 (0.7%)
About once a day	355 (8.8%)	383 (6.0%)
Sometimes	1,782 (44.2%)	3,312 (52.0%)
Never	1,809 (44.8%)	2,585 (40.6%)
(Missing)	39 (1.0%)	42 (0.7%)
Prayer or meditation, n (%)		
More than once a day	183 (4.5%)	514 (8.1%)
About once a day	700 (17.3%)	1,504 (23.6%)
Sometimes	1,881 (46.6%)	2,872 (45.1%)
Never	1,243 (30.8%)	1,452 (22.8%)
(Missing)	28 (0.7%)	24 (0.4%)
Belief in God/gods/spiritual forces, n (%)		
One God	2,721 (67.4%)	5,211 (81.9%)
More than one god	72 (1.8%)	29 (0.5%)
An impersonal spiritual force	241 (6.0%)	210 (3.3%)
None of these	500 (12.4%)	407 (6.4%)
Unsure	463 (11.5%)	480 (7.5%)
(Missing)	37 (0.9%)	29 (0.4%)
Religious centrality, n (%)		
Agree	1,383 (34.3%)	3,107 (48.8%)
Disagree	1,032 (25.6%)	1,395 (21.9%)
Not relevant	625 (15.5%)	618 (9.7%)
Unsure	922 (22.8%)	1,210 (19.0%)
(Missing)	73 (1.8%)	36 (0.6%)
Religious/spiritual comfort, n (%)		
Agree	1,542 (38.2%)	3,358 (52.7%)
Disagree	915 (22.7%)	1,098 (17.3%)
Not relevant	591 (14.6%)	610 (9.6%)
Unsure	907 (22.5%)	1,256 (19.7%)
(Missing)	79 (2.0%)	43 (0.7%)
Feel loved by God, n (%)		
Agree	1,584 (39.3%)	3,343 (52.5%)
Disagree	804 (19.9%)	775 (12.2%)
Not relevant	580 (14.4%)	602 (9.5%)
Unsure	970 (24.0%)	1,589 (25.0%)
(Missing)	97 (2.4%)	57 (0.9%)
Feel punished by God, n (%)		
Agree	579 (14.4%)	1,174 (18.4%)
Disagree	1,748 (43.3%)	2,875 (45.2%)
Not relevant	609 (15.1%)	663 (10.4%)
Unsure	1,006 (24.9%)	1,589 (25.0%)
(Missing)	93 (2.3%)	65 (1.0%)
Experienced religious criticism, n (%)		

Table S24d. Unweighted summary statistics for Wave 1 outcome variables in Poland by retention status.

Outcome	Attritors–Not Observed in Wave 2<br>N = 4,035	Retained–Observed in Wave 2<br>N = 6,366
Agree	425 (10.5%)	831 (13.1%)
Disagree	1,816 (45.0%)	3,155 (49.6%)
Not relevant	828 (20.5%)	1,178 (18.5%)
Unsure	874 (21.7%)	1,144 (18.0%)
(Missing)	91 (2.3%)	58 (0.9%)
Faith-sharing, n (%)		
Agree	1,116 (27.7%)	1,921 (30.2%)
Disagree	1,245 (30.9%)	2,549 (40.0%)
Not relevant	754 (18.7%)	907 (14.2%)
Unsure	854 (21.2%)	965 (15.2%)
(Missing)	67 (1.7%)	24 (0.4%)

Note. N (%); this table is based on non-imputed data. Cumulative percentages for variables may not add up to 100% due to rounding.

Table S24e. Summary of fitted attrition model in Poland

Characteristic	Odds Ratio	95% CI	p-value
**ANNUAL_WEIGHT_R2**	0.84	0.75, 0.94	0.002
**Recruitment Survey Mode**			
CAPI	—	—	
CATI	1.50	1.17, 1.93	0.001
**Happiness & life satisfaction**	1.23	1.10, 1.38	4.32e-04
**Physical & mental health**	1.21	1.08, 1.36	0.001
**Meaning & purpose**	0.93	0.81, 1.05	0.235
**Character & virtue**	0.95	0.86, 1.05	0.301
**Close social relationships**	0.81	0.74, 0.90	4.03e-05
**Financial & material security**	0.95	0.84, 1.07	0.381
**Extraversion**	1.11	1.03, 1.20	0.007
**Openness to experience**	0.86	0.78, 0.95	0.002
**Agreeableness**	1.22	1.12, 1.33	8.91e-06
**Conscientiousness**	1.05	0.96, 1.15	0.293
**Neuroticism**	0.98	0.90, 1.06	0.633
**Depression symptoms composite**	0.89	0.83, 0.97	0.007
**Anxiety symptoms composite**	0.98	0.90, 1.07	0.625
**Loneliness**	0.85	0.77, 0.94	0.002
**Days exercise per week**	0.86	0.79, 0.94	0.001
**Year of birth (age group)**			
1983-1993 (current age: 30-39 years)	—	—	
1973-1983 (current age: 40-49 years)	0.97	0.82, 1.14	0.693
1963-1973 (current age: 50-59 years)	1.20	1.01, 1.44	0.043
1953-1963 (current age: 60-69 years)	2.13	1.53, 2.95	6.76e-06
1993-1998 (current age: 25-29 years)	1.04	0.86, 1.24	0.695
1998-2005 (current age: 18-24 years)	1.14	0.84, 1.55	0.398
1943-1953 (current age: 70-79 years)	2.83	1.81, 4.43	5.19e-06
1943 or earlier (current age: 80+ years)	1.95	0.97, 3.94	0.062
**Gender of respondent**			
Female	—	—	
Male	0.93	0.82, 1.06	0.286
Other	0.40	0.05, 3.31	0.398
**Marital status**			
Married	—	—	
Single/Never been married	1.21	0.97, 1.50	0.090
Domestic partner	0.86	0.66, 1.12	0.265
Widowed	0.80	0.59, 1.08	0.142
Divorced	0.93	0.73, 1.20	0.587
Separated	0.89	0.54, 1.49	0.666
**Employment status**			
Employed for an employer	—	—	
Retired	1.15	0.82, 1.61	0.410
Self-employed	0.91	0.71, 1.16	0.442
Student	1.19	0.80, 1.78	0.390
Homemaker	1.19	0.87, 1.63	0.287
Unemployed and looking for a job	1.53	0.94, 2.49	0.087
None of these/Other	0.94	0.54, 1.64	0.829
**Religious attendance**			
A few times a year	—	—	
Once a week	1.39	1.07, 1.80	0.013
One to three times a month	1.28	1.04, 1.58	0.019
Never	0.83	0.65, 1.06	0.129

Table S24e. Summary of fitted attrition model in Poland

Characteristic	Odds Ratio	95% CI	p-value
More than once a week	1.10	0.72, 1.68	0.651
**Educational attainment (16+ years)**			
9-15	—	—	
16+	1.13	0.97, 1.32	0.114
Up to 8	1.27	0.79, 2.02	0.320
**Born in This country**			
Born in this country	—	—	
Born in another country	0.60	0.32, 1.13	0.112
**Race plurality (prominent race/ethnic group [0] or not [1])**	0.94	0.89, 0.99	0.021
**Urbanicity**			
A small town or village	—	—	
A large city	1.26	0.90, 1.77	0.176
A rural area or on a farm	1.64	1.09, 2.45	0.017
A suburb of a large city	0.41	0.08, 2.01	0.270
**Monthly household income**			
Poland: 6,001 – 8,000 PLN	—	—	
Poland: 5,001 – 6,000 PLN	1.00	0.83, 1.20	0.974
Poland: 4,001 – 5,000 PLN	0.88	0.70, 1.10	0.273
Poland: 8,001 – 10,000 PLN	1.06	0.85, 1.31	0.602
Poland: 3,501 – 4,000 PLN	0.85	0.65, 1.12	0.249
Poland: 10,001 – 15,000 PLN	1.77	1.24, 2.52	0.002
Poland: 3,001 – 3,500 PLN	0.74	0.54, 1.01	0.057
Poland: 2,501 – 3,000 PLN	0.60	0.43, 0.85	0.004
Poland: 2,001 – 2,500 PLN	0.47	0.32, 0.69	1.36e-04
(None/No household income)	0.69	0.34, 1.39	0.298
Poland: 15,001 – 20,000 PLN	1.05	0.64, 1.71	0.850
Poland: 1,501 – 2,000 PLN	0.48	0.28, 0.84	0.010
Poland: More than 20,000 PLN	0.73	0.40, 1.36	0.328
Poland: 1,001 – 1,500 PLN	0.26	0.12, 0.53	2.67e-04
Poland: 501 – 1,000 PLN	0.45	0.16, 1.30	0.142
Poland: 100 PLN or less	0.70	0.04, 12.4	0.810
Poland: 101 – 500 PLN	4.27	0.24, 76.9	0.325

Abbreviations: CI = Confidence Interval, OR = Odds Ratio

Notes. N=10389; attrition weights were estimated using the 'survey::svyglm(family=quasibinomial('logit'))' function. All continuous predictors were standardized and all categorical predictors used the most common category as the reference group. Reported p-values are based on the fitted regression model and no adjustments for multiple testing were done within this table.

Table S24f. Summary of principal components in Poland

PC	Percent Explained by each PC	Cumulative Percent Explained
1	29.86	29.86
2	6.77	36.63
3	5.75	42.38
4	2.44	44.82
5	2.23	47.05
6	1.91	48.95
**7**	**1.87**	**50.83**
8	1.63	52.45
9	1.53	53.98
10	1.46	55.44
11	1.37	56.81
12	1.34	58.15
13	1.28	59.43
14	1.26	60.69
15	1.21	61.90
16	1.19	63.09
17	1.14	64.23
18	1.06	65.29
19	1.04	66.33
20	1.02	67.35

Notes. N=10389; PCA was conducted using 'survey::svyprcomp(.)' function using all available contemporaneous exposures at wave 1. All PCs were standardized prior to being used as predictors. The bolded row represented the number of retained components for analysis was 7.

Table S24g. Associations of forgivingness with adult well-being and other outcomes at Wave 2 in Poland

Outcome	Model 1: Demographic and Childhood Variables as Covariates					Model 2: Demographic, Childhood, and Other Wave 1 Confounding Variables (Via Principal Components) as Covariates				
	RR	ES	95% CI	SE	p-value	RR	ES	95% CI	SE	p-value
Human Flourishing										
Secure flourishing index		0.09	(0.06,0.12)	0.02	2.42e-09***		0.04	(0.02,0.06)	0.01	1.02e-03**
Flourishing index		0.11	(0.08,0.14)	0.02	4.75e-12***		0.05	(0.03,0.08)	0.01	2.24e-05***
Happiness & life satisfaction		0.06	(0.03,0.09)	0.02	3.9e-04***		0.02	(-0.01,0.04)	0.01	0.174
Physical & mental health		0.04	(0.01,0.08)	0.02	0.024*		0.02	(-0.01,0.05)	0.01	0.137
Meaning & purpose		0.03	(0.00,0.06)	0.02	0.046*		-0.02	(-0.05,0.00)	0.01	0.092
Character & virtue		0.18	(0.14,0.22)	0.02	2.22e-16***		0.14	(0.10,0.18)	0.02	2.92e-12***
Close social relationships		0.10	(0.07,0.13)	0.02	3.83e-09***		0.04	(0.02,0.07)	0.01	1.87e-03**
Financial & material security		0.01	(-0.03,0.05)	0.02	0.506		-0.02	(-0.05,0.02)	0.02	0.403
Psychological Well-Being										
Happiness (a)		0.05	(0.02,0.08)	0.02	1.49e-03**		0.02	(-0.01,0.05)	0.01	0.279
Life satisfaction (a)		0.05	(0.02,0.08)	0.02	3.95e-03**		0.02	(-0.01,0.05)	0.02	0.285
Current life evaluation		0.10	(0.06,0.13)	0.02	8.56e-08***		0.07	(0.03,0.10)	0.02	2.17e-04***
Future life evaluation		0.09	(0.06,0.12)	0.02	2.64e-07***		0.06	(0.02,0.09)	0.02	9.61e-04**
Optimism		0.07	(0.03,0.11)	0.02	2.08e-04***		0.02	(-0.02,0.05)	0.02	0.321
Freedom to pursue what's important		0.03	(-0.01,0.06)	0.02	0.120		-0.01	(-0.04,0.02)	0.02	0.390
Inner peace	1.04		(1.02,1.05)	0.01	2.96e-06***	1.02		(1.01,1.03)	0.01	0.007*
Life balance	1.03		(1.02,1.04)	0.01	7.34e-05***	1.01		(1.00,1.03)	0.01	0.074
Sense of mastery	1.03		(1.02,1.05)	0.01	1.89e-05***	1.02		(1.01,1.03)	0.01	1.54e-03**
Meaningful activities (c)		0.04	(0.00,0.07)	0.02	0.033*		-0.01	(-0.04,0.02)	0.02	0.490
Understanding purpose (c)		0.02	(-0.01,0.05)	0.02	0.196		-0.03	(-0.06,-0.00)	0.02	0.043*
Self-rated mental health (b)		0.04	(0.00,0.08)	0.02	0.039*		0.02	(-0.01,0.05)	0.02	0.170
Psychological Distress										
Traumatic distress	1.01		(1.00,1.02)	0.01	0.068	1.01		(1.00,1.02)	0.01	0.071
Depression symptoms composite	1.00		(0.99,1.01)	0.01	0.463	1.00		(0.99,1.01)	0.01	0.912
Depression – feel hopeless	0.99		(0.99,1.00)	0.00	0.233	1.00		(0.99,1.01)	0.00	0.592
Depression – loss of interest	1.00		(0.99,1.01)	0.01	0.743	1.00		(0.99,1.01)	0.01	0.913
Anxiety symptoms composite	1.00		(0.99,1.01)	0.00	0.638	1.00		(0.99,1.01)	0.00	0.967
Anxiety – feel on edge	1.00		(0.99,1.01)	0.00	0.549	1.00		(0.99,1.01)	0.00	0.983
Anxiety – cannot stop worrying	1.00		(0.99,1.01)	0.00	0.958	1.00		(0.99,1.01)	0.00	0.634
Suffering	1.01		(0.99,1.02)	0.01	0.398	1.00		(0.99,1.02)	0.01	0.470
Social Well-Being										
Relationship contentment (e)		0.09	(0.06,0.12)	0.02	5.26e-08***		0.04	(0.01,0.07)	0.01	3.06e-03**
Relationship satisfaction (e)		0.08	(0.05,0.11)	0.02	2.39e-06***		0.04	(0.00,0.07)	0.02	0.025*
Social support		0.07	(0.04,0.10)	0.02	5.26e-05***		0.02	(-0.01,0.05)	0.02	0.276
Intimate/close friend	1.00		(0.99,1.02)	0.01	0.684	1.00		(0.99,1.01)	0.01	0.836
Government approval	1.03		(1.02,1.05)	0.01	1.88e-04***	1.03		(1.01,1.04)	0.01	7.25e-04**
Say in government	1.00		(0.98,1.02)	0.01	0.952	0.99		(0.98,1.01)	0.01	0.327
Belonging in country		0.03	(-0.00,0.06)	0.02	0.091		0.00	(-0.03,0.03)	0.02	0.769
City/place satisfaction	1.02		(1.01,1.03)	0.01	2.87e-04***	1.02		(1.01,1.03)	0.01	1.84e-03**
Trust within country	0.99		(0.98,1.01)	0.01	0.497	0.99		(0.97,1.00)	0.01	0.072
Social Participation										
Ever been married	1.00		(0.99,1.01)	0.00	0.735	1.00		(0.99,1.01)	0.00	0.706
Currently divorced	1.00		(0.99,1.01)	0.00	0.762	1.00		(1.00,1.01)	0.00	0.658
Number of children		0.02	(-0.01,0.05)	0.02	0.248		0.01	(-0.02,0.05)	0.02	0.386
Weekly+ community participation	1.01		(1.00,1.01)	0.00	0.124	1.00		(0.99,1.01)	0.00	0.657
Weekly+ religious attendance	1.00		(0.99,1.02)	0.01	0.456	1.00		(0.99,1.01)	0.01	0.516
Social Distress										
Loneliness		-0.04	(-0.08,-0.01)	0.02	0.014*		-0.01	(-0.03,0.02)	0.01	0.675
Perceived discrimination	1.01		(1.00,1.02)	0.01	0.013*	1.01		(1.00,1.02)	0.01	0.103
Character & Prosocial Behavior										
Orientation to promote good (d)		0.09	(0.06,0.12)	0.02	6.55e-08***		0.05	(0.02,0.08)	0.02	1.45e-03**
Delayed gratification (d)		0.18	(0.13,0.23)	0.02	3.33e-13***		0.15	(0.11,0.19)	0.02	2.32e-12***
Hope		0.06	(0.03,0.10)	0.02	7.75e-05***		0.01	(-0.02,0.04)	0.02	0.560
Gratitude		0.10	(0.07,0.13)	0.02	1.84e-09***		0.05	(0.02,0.08)	0.02	5e-04***
Showing love/care		0.08	(0.04,0.11)	0.02	1.03e-05***		0.03	(-0.00,0.06)	0.02	0.065
Forgivingness	1.12		(1.10,1.14)	0.01	2.22e-16***	1.11		(1.09,1.13)	0.01	2.22e-16***
Charitable giving	1.03		(1.01,1.04)	0.01	1.88e-05***	1.01		(1.00,1.02)	0.01	0.086
Helping strangers	1.03		(1.02,1.05)	0.01	6.96e-05***	1.03		(1.01,1.04)	0.01	7.14e-04**
Volunteering	1.00		(0.99,1.01)	0.00	0.659	0.99		(0.98,1.00)	0.00	0.056
Physical Health & Health Behavior										
Self-rated physical health (b)		0.03	(0.00,0.07)	0.02	0.049*		0.02	(-0.01,0.05)	0.02	0.260
Health problems	1.00		(0.99,1.02)	0.01	0.423	1.01		(0.99,1.02)	0.01	0.312
Pain in past 4 weeks	0.99		(0.98,1.01)	0.01	0.409	1.00		(0.98,1.01)	0.01	0.552
Daily smoker	1.00		(0.98,1.01)	0.01	0.832	1.01		(1.00,1.02)	0.01	0.176
Number of drinks per week		-0.00	(-0.04,0.03)	0.02	0.871		0.02	(-0.02,0.05)	0.02	0.297
Days exercise per week		0.07	(0.04,0.10)	0.02	2.44e-05***		0.05	(0.02,0.08)	0.02	2.51e-03**
Socioeconomic Outcomes										
Financial security (f)		0.01	(-0.03,0.04)	0.02	0.709		-0.02	(-0.05,0.02)	0.02	0.378
Material security (f)		0.02	(-0.02,0.05)	0.02	0.408		-0.01	(-0.05,0.02)	0.02	0.552
Educational attainment (16+ years)	1.00		(1.00,1.01)	0.00	0.523	1.00		(1.00,1.00)	0.00	1.000
Currently employed	1.00		(0.99,1.01)	0.01	0.733	1.00		(0.99,1.01)	0.01	0.449
Financially comfortable/getting by	1.00		(0.99,1.01)	0.01	0.570	0.99		(0.98,1.00)	0.01	0.056
Own home	1.00		(0.99,1.02)	0.01	0.438	1.01		(1.00,1.02)	0.01	0.295
Income – top quintile	0.99		(0.98,1.01)	0.01	0.280	0.99		(0.98,1.00)	0.01	0.198
Religion & Spirituality										
Religious/spiritual connection	1.04		(1.02,1.06)	0.01	4.75e-06***	1.02		(1.00,1.03)	0.01	0.048*
Belief in life after death	0.99		(0.97,1.00)	0.01	0.106	0.98		(0.97,1.00)	0.01	0.015*
Transformative religious experience	1.00		(1.00,1.01)	0.00	0.275	1.00		(0.99,1.01)	0.00	0.789
Religious reading or listening	1.01		(1.00,1.01)	0.00	0.076	1.00		(1.00,1.01)	0.00	0.442
Prayer or meditation	1.02		(1.01,1.03)	0.01	1.49e-03**	1.01		(1.00,1.02)	0.01	0.263
Belief in God/gods/spiritual forces	1.00		(0.99,1.01)	0.01	0.752	1.00		(0.99,1.01)	0.01	0.465
Religious centrality	1.04		(1.02,1.06)	0.01	5.09e-06***	1.02		(1.00,1.04)	0.01	0.016*
Religious/spiritual comfort	1.03		(1.01,1.05)	0.01	7.83e-04**	1.01		(1.00,1.03)	0.01	0.122
Feel loved by God	1.02		(1.01,1.03)	0.01	4.64e-03**	1.01		(0.99,1.02)	0.01	0.317
Feel punished by God	1.00		(0.99,1.01)	0.01	0.734	1.00		(0.99,1.01)	0.01	0.513
Experienced religious criticism	1.00		(0.99,1.01)	0.01	0.482	1.00		(0.99,1.01)	0.00	0.823
Faith-sharing	1.03		(1.01,1.04)	0.01	3.71e-04***	1.02		(1.00,1.03)	0.01	0.010*

Notes. N=10389; Reference for focal predictor: never/rarely. RR, risk-ratio, null effect is 1.00; ES, effect size measure for standardized regression coefficient, null effect is 0.00; SE, standard error, the SE reported for binary/Likert-type outcomes where risk-ratios are on the log(RR) scale; CI, confidence interval; p-value, a Wald-type test of the null hypothesis that the effect of the focal predictor is zero; (a) item part of the Happiness & Life Satisfaction domain of the Secure Flourishing Index; (b) item part of the Physical & Mental Health domain of the Secure Flourishing Index; (c) item part of the Meaning & Purpose domain of the Secure Flourishing Index; (d) item part of the Character & Virtue domain of the Secure Flourishing Index; (e) item part of the Subjective Social Connectedness domain of the Secure Flourishing Index; (f) item part of the Financial & Material Security domain of the Secure Flourishing Index.

Multiple imputation was performed to impute missing data on the covariates, exposure, and outcomes. All models controlled for sociodemographic and childhood factors assessed at Wave 1. For Model 2 with PC (principal components), the first seven principal components of the entire set of contemporaneous confounders assessed at Wave 1 were included as additional covariates of the outcomes at Wave 2.

An outcome-wide analytic approach was used, and a separate model was run for each outcome. A different type of model was run depending on the nature of the outcome: (1) for each binary outcome, a weighted generalized linear model (with a log link and Poisson distribution) was used to estimate an RR; and (2) for each continuous outcome, a weighted linear regression model was used to estimate a ES. All effect sizes were standardized. For continuous outcomes, the ES represents the change in SD on the outcome between the lower and upper categories of the binary focal predictor. For binary outcomes, the RR represents the change in risk of being in the upper category compared to the lower category between the lower and upper categories of the binary focal predictor.

P-value significance thresholds: $p < 0.05$*, $p < 0.005$**, (Bonferroni) $p < 6.41e-04$***, correction for multiple testing using Bonferroni adjusted significant threshold.

Table S24h. Associations of forgivingness with adult well-being and other outcomes at Wave 2 in Poland using complete-case analyses with attrition weights.

	Model 1: Demographic and Childhood Variables as Covariates					Model 2: Demographic, Childhood, and Other Wave 1 Confounding Variables (Via Principal Components) as Covariates				
Outcome	RR	ES	95% CI	SE	p-value	RR	ES	95% CI	SE	p-value
Human Flourishing										
Secure flourishing index		0.08	(0.05,0.12)	0.02	1.42e-05***		0.04	(0.00,0.07)	0.02	0.026*
Flourishing index		0.10	(0.06,0.14)	0.02	2.09e-07***		0.05	(0.02,0.08)	0.02	2.04e-03**
Happiness & life satisfaction		0.06	(0.02,0.09)	0.02	3.24e-03**		0.02	(-0.01,0.06)	0.02	0.183
Physical & mental health		0.03	(-0.01,0.07)	0.02	0.135		0.02	(-0.01,0.05)	0.02	0.258
Meaning & purpose		0.03	(-0.01,0.07)	0.02	0.117		-0.03	(-0.06,0.01)	0.02	0.150
Character & virtue		0.20	(0.15,0.24)	0.02	8.88e-16***		0.14	(0.10,0.18)	0.02	7.2e-12***
Close social relationships		0.10	(0.06,0.14)	0.02	8.6e-07***		0.05	(0.01,0.08)	0.02	0.011*
Financial & material security		-0.00	(-0.05,0.04)	0.02	0.942		-0.02	(-0.06,0.02)	0.02	0.319
Psychological Well-Being										
Happiness (a)		0.06	(0.02,0.10)	0.02	2.44e-03**		0.03	(-0.01,0.06)	0.02	0.144
Life satisfaction (a)		0.05	(0.01,0.09)	0.02	0.011*		0.02	(-0.02,0.05)	0.02	0.315
Current life evaluation		0.10	(0.06,0.14)	0.02	3.76e-06***		0.06	(0.02,0.10)	0.02	3.75e-03**
Future life evaluation		0.10	(0.06,0.14)	0.02	1.83e-06***		0.06	(0.02,0.10)	0.02	3.31e-03**
Optimism		0.07	(0.03,0.11)	0.02	6.17e-04***		0.01	(-0.03,0.05)	0.02	0.565
Freedom to pursue what's important		0.03	(-0.01,0.07)	0.02	0.093		-0.01	(-0.05,0.02)	0.02	0.408
Inner peace	1.03		(1.01,1.05)	0.01	4.63e-04***	1.01		(1.00,1.03)	0.01	0.119
Life balance	1.03		(1.01,1.05)	0.01	3.63e-04***	1.01		(1.00,1.03)	0.01	0.110
Sense of mastery	1.03		(1.02,1.05)	0.01	9.78e-05***	1.02		(1.01,1.04)	0.01	0.007*
Meaningful activities (c)		0.04	(-0.00,0.08)	0.02	0.069		-0.01	(-0.05,0.02)	0.02	0.550
Understanding purpose (c)		0.02	(-0.02,0.06)	0.02	0.276		-0.03	(-0.07,0.00)	0.02	0.053
Self-rated mental health (b)		0.03	(-0.01,0.07)	0.02	0.174		0.02	(-0.02,0.05)	0.02	0.313
Psychological Distress										
Traumatic distress	1.01		(0.99,1.02)	0.01	0.212	1.01		(0.99,1.02)	0.01	0.216
Depression symptoms composite	0.99		(0.98,1.00)	0.01	0.231	1.00		(0.99,1.01)	0.01	0.923
Depression – feel hopeless	0.99		(0.98,1.00)	0.01	0.108	1.00		(0.99,1.01)	0.01	0.636
Depression – loss of interest	1.00		(0.98,1.01)	0.01	0.595	1.00		(0.99,1.01)	0.01	0.798
Anxiety symptoms composite	0.99		(0.98,1.01)	0.01	0.281	1.00		(0.99,1.01)	0.01	0.914
Anxiety – feel on edge	0.99		(0.98,1.00)	0.01	0.217	1.00		(0.99,1.01)	0.01	0.830
Anxiety – cannot stop worrying	1.00		(0.99,1.01)	0.01	0.737	1.00		(0.99,1.01)	0.00	0.556
Suffering	1.00		(0.99,1.02)	0.01	0.804	1.00		(0.99,1.02)	0.01	0.826
Social Well-Being										
Relationship contentment (e)		0.10	(0.06,0.15)	0.02	6.18e-07***		0.05	(0.01,0.09)	0.02	0.008*
Relationship satisfaction (e)		0.09	(0.05,0.13)	0.02	1.24e-05***		0.04	(0.00,0.07)	0.02	0.036*
Social support		0.08	(0.04,0.12)	0.02	2.18e-04***		0.02	(-0.02,0.06)	0.02	0.272
Intimate/close friend	1.01		(0.99,1.03)	0.01	0.198	1.01		(0.99,1.02)	0.01	0.321
Government approval	1.05		(1.03,1.07)	0.01	1.12e-05***	1.03		(1.01,1.05)	0.01	2.35e-03**
Say in government	1.01		(0.99,1.03)	0.01	0.355	1.00		(0.98,1.01)	0.01	0.587
Belonging in country		0.03	(-0.00,0.07)	0.02	0.070		0.01	(-0.02,0.05)	0.02	0.422
City/place satisfaction	1.02		(1.01,1.04)	0.01	4e-04***	1.02		(1.01,1.03)	0.01	2.71e-03**
Trust within country	1.00		(0.98,1.02)	0.01	0.829	0.99		(0.97,1.01)	0.01	0.377
Social Participation										
Ever been married	1.01		(0.99,1.02)	0.01	0.269	1.01		(1.00,1.02)	0.01	0.211
Currently divorced	1.00		(0.99,1.00)	0.00	0.309	1.00		(0.99,1.00)	0.00	0.350
Number of children		0.02	(-0.01,0.05)	0.01	0.136		0.03	(0.00,0.06)	0.01	0.032*
Weekly+ community participation	1.00		(0.99,1.01)	0.00	0.544	1.00		(0.99,1.01)	0.00	0.534
Weekly+ religious attendance	1.00		(0.98,1.01)	0.01	0.842	0.99		(0.98,1.01)	0.01	0.246
Social Distress										
Loneliness		-0.05	(-0.09,-0.01)	0.02	0.018*		-0.01	(-0.05,0.02)	0.02	0.443
Perceived discrimination	1.01		(1.00,1.03)	0.01	0.047*	1.01		(0.99,1.02)	0.01	0.359
Character & Prosocial Behavior										
Orientation to promote good (d)		0.10	(0.06,0.14)	0.02	4e-06***		0.06	(0.02,0.10)	0.02	4.76e-03**
Delayed gratification (d)		0.20	(0.15,0.26)	0.03	8.15e-14***		0.16	(0.12,0.20)	0.02	2.69e-13***
Hope		0.06	(0.02,0.10)	0.02	3.37e-03**		-0.00	(-0.04,0.03)	0.02	0.898
Gratitude		0.12	(0.08,0.15)	0.02	8.18e-10***		0.06	(0.03,0.10)	0.02	1.01e-03**
Showing love/care		0.10	(0.05,0.14)	0.02	2.37e-05***		0.04	(0.00,0.08)	0.02	0.027*
Forgivingness	1.13		(1.11,1.15)	0.01	2.22e-16***	1.12		(1.10,1.14)	0.01	2.22e-16***
Charitable giving	1.03		(1.01,1.04)	0.01	1.26e-04***	1.00		(0.99,1.02)	0.01	0.502
Helping strangers	1.03		(1.01,1.05)	0.01	7.28e-04**	1.02		(1.01,1.04)	0.01	0.008*
Volunteering	1.00		(0.99,1.01)	0.00	0.392	0.99		(0.98,1.00)	0.01	0.022*
Physical Health & Health Behavior										
Self-rated physical health (b)		0.03	(-0.01,0.06)	0.02	0.157		0.02	(-0.02,0.05)	0.02	0.314
Health problems	1.00		(0.99,1.01)	0.01	0.782	1.00		(0.99,1.01)	0.01	0.938
Pain in past 4 weeks	0.99		(0.98,1.01)	0.01	0.283	0.99		(0.98,1.01)	0.01	0.336
Daily smoker	1.01		(0.99,1.02)	0.01	0.314	1.02		(1.01,1.04)	0.01	2.99e-03**
Number of drinks per week		0.01	(-0.03,0.05)	0.02	0.769		0.03	(-0.00,0.07)	0.02	0.067
Days exercise per week		0.06	(0.02,0.10)	0.02	2.43e-03**		0.03	(-0.01,0.07)	0.02	0.122
Socioeconomic Outcomes										
Financial security (f)		-0.00	(-0.05,0.04)	0.02	0.840		-0.02	(-0.06,0.02)	0.02	0.359
Material security (f)		0.00	(-0.04,0.05)	0.02	0.954		-0.02	(-0.06,0.02)	0.02	0.328
Educational attainment (16+ years)	1.00		(1.00,1.01)	0.00	0.607	1.00		(1.00,1.00)	0.00	0.844
Currently employed	1.00		(0.99,1.01)	0.01	0.627	1.00		(0.99,1.01)	0.01	0.781
Financially comfortable/getting by	1.00		(0.99,1.01)	0.01	0.911	1.00		(0.98,1.01)	0.01	0.609
Own home	1.00		(0.99,1.01)	0.01	0.742	1.01		(0.99,1.02)	0.01	0.305
Income – top quintile	0.99		(0.98,1.01)	0.01	0.406	1.00		(0.98,1.01)	0.01	0.637
Religion & Spirituality										
Religious/spiritual connection	1.03		(1.01,1.05)	0.01	0.007*	1.01		(0.99,1.03)	0.01	0.514
Belief in life after death	0.98		(0.96,1.00)	0.01	0.033*	0.98		(0.96,1.00)	0.01	0.012*
Transformative religious experience	1.01		(1.00,1.02)	0.00	0.214	1.00		(0.99,1.01)	0.00	0.870
Religious reading or listening	1.00		(0.99,1.01)	0.00	0.887	1.00		(0.99,1.01)	0.00	0.723
Prayer or meditation	1.01		(1.00,1.03)	0.01	0.067	1.00		(0.99,1.02)	0.01	0.546
Belief in God/gods/spiritual forces	1.00		(0.98,1.01)	0.01	0.441	0.99		(0.98,1.01)	0.01	0.247
Religious centrality	1.03		(1.01,1.05)	0.01	1.75e-03**	1.02		(1.00,1.04)	0.01	0.119
Religious/spiritual comfort	1.02		(1.00,1.04)	0.01	0.036*	1.01		(0.99,1.03)	0.01	0.356
Feel loved by God	1.02		(1.00,1.03)	0.01	0.062	1.01		(0.99,1.02)	0.01	0.364
Feel punished by God	1.00		(0.99,1.02)	0.01	0.686	1.01		(0.99,1.02)	0.01	0.388
Experienced religious criticism	1.00		(0.99,1.01)	0.01	0.966	0.99		(0.98,1.00)	0.00	0.162
Faith-sharing	1.04		(1.02,1.05)	0.01	6.57e-06***	1.03		(1.01,1.04)	0.01	4.22e-04***

Table S24h. Associations of forgivingness with adult well-being and other outcomes at Wave 2 in Poland using complete-case analyses with attrition weights.

	Model 1: Demographic and Childhood Variables as Covariates					Model 2: Demographic, Childhood, and Other Wave 1 Confounding Variables (Via Principal Components) as Covariates				
Outcome	RR	ES	95% CI	SE	p-value	RR	ES	95% CI	SE	p-value

Notes. N=6478; Reference for focal predictor: never/rarely. RR, risk-ratio, null effect is 1.00; ES, effect size measure for standardized regression coefficient, null effect is 0.00; SE, standard error, the SE reported for binary/Likert-type outcomes where risk-ratios are on the log(RR) scale; CI, confidence interval; p-value, a Wald-type test of the null hypothesis that the effect of the focal predictor is zero; (a) item part of the Happiness & Life Satisfaction domain of the Secure Flourishing Index; (b) item part of the Physical & Mental Health domain of the Secure Flourishing Index; (c) item part of the Meaning & Purpose domain of the Secure Flourishing Index; (d) item part of the Character & Virtue domain of the Secure Flourishing Index; (e) item part of the Subjective Social Connectedness domain of the Secure Flourishing Index; (f) item part of the Financial & Material Security domain of the Secure Flourishing Index.

Attrition weights were computed to adjust the complete case data (those who responded at Wave 2 to at least 50% of the questions) and multiple imputation was used to impute missing data on all remaining within wave on the covariates, exposure, and outcomes. All models controlled for sociodemographic and childhood factors assessed at Wave 1. For Model 2 with PC (principal components), the first seven principal components of the entire set of contemporaneous confounders assessed at Wave 1 were included as additional covariates of the outcomes at Wave 2.

An outcome-wide analytic approach was used, and a separate model was run for each outcome. A different type of model was run depending on the nature of the outcome: (1) for each binary outcome, a weighted generalized linear model (with a log link and Poisson distribution) was used to estimate an RR; and (2) for each continuous outcome, a weighted linear regression model was used to estimate a ES. All effect sizes were standardized. For continuous outcomes, the ES represents the change in SD on the outcome between the lower and upper categories of the binary focal predictor. For binary outcomes, the RR represents the change in risk of being in the upper category compared to the lower category between the lower and upper categories of the binary focal predictor.

P-value significance thresholds: p < 0.05*, p < 0.005**, (Bonferroni) p < 6.41e-04***, correction for multiple testing using Bonferroni adjusted significant threshold.

Table S24i. Sensitivity analysis of forgivingness outcome-wide results to unmeasured confounding using E-values in Poland

Outcome	Multiple Imputation				Complete Case w/ Attrition Weights			
	Model 1: Demographics and Childhood Variables as Covariates		Model 2: Demographics, Childhood, and Other Wave 1 Confounders (Via Principal Components) as Covariates		Model 1: Demographics and Childhood Variables as Covariates		Model 2: Demographics, Childhood, and Other Wave 1 Confounders (Via Principal Components) as Covariates	
	EE	ECI	EE	ECI	EE	ECI	EE	ECI
Human Flourishing								
Secure flourishing index	1.67	1.50	1.37	1.21	1.63	1.41	1.35	1.10
Flourishing index	1.75	1.58	1.44	1.29	1.73	1.51	1.43	1.23
Happiness & life satisfaction	1.47	1.28	1.23	1.00	1.47	1.23	1.26	1.00
Physical & mental health	1.39	1.12	1.26	1.00	1.32	1.00	1.24	1.00
Meaning & purpose	1.33	1.04	1.27	1.00	1.33	1.00	1.28	1.00
Character & virtue	2.19	1.94	1.95	1.73	2.28	2.00	1.96	1.73
Close social relationships	1.70	1.52	1.40	1.22	1.73	1.50	1.41	1.17
Financial & material security	1.19	1.00	1.20	1.00	1.06	1.00	1.25	1.00
Psychological Well-Being								
Happiness	1.45	1.24	1.21	1.00	1.48	1.25	1.28	1.00
Life satisfaction	1.43	1.21	1.21	1.00	1.42	1.17	1.22	1.00
Current life evaluation	1.70	1.50	1.54	1.33	1.70	1.47	1.50	1.24
Future life evaluation	1.66	1.47	1.48	1.27	1.70	1.48	1.49	1.24
Optimism	1.54	1.33	1.23	1.00	1.55	1.31	1.17	1.00
Freedom to pursue what's important	1.29	1.00	1.19	1.00	1.33	1.00	1.20	1.00
Inner peace	1.37	1.27	1.24	1.11	1.35	1.21	1.19	1.00
Life balance	1.32	1.21	1.19	1.00	1.34	1.21	1.19	1.00
Sense of mastery	1.35	1.24	1.26	1.15	1.36	1.23	1.26	1.12
Meaningful activities	1.35	1.08	1.17	1.00	1.36	1.00	1.17	1.00
Understanding purpose	1.25	1.00	1.32	1.05	1.26	1.00	1.34	1.00
Self-rated mental health	1.37	1.07	1.25	1.00	1.31	1.00	1.23	1.00
Psychological Distress								
Traumatic distress	1.18	1.00	1.18	1.00	1.16	1.00	1.16	1.00
Depression symptoms composite	1.10	1.00	1.04	1.00	1.14	1.00	1.04	1.00
Depression – feel hopeless	1.12	1.00	1.07	1.00	1.16	1.00	1.08	1.00
Depression – loss of interest	1.06	1.00	1.04	1.00	1.09	1.00	1.06	1.00
Anxiety symptoms composite	1.08	1.00	1.02	1.00	1.13	1.00	1.04	1.00
Anxiety – feel on edge	1.08	1.00	1.01	1.00	1.14	1.00	1.05	1.00
Anxiety – cannot stop worrying	1.02	1.00	1.07	1.00	1.07	1.00	1.09	1.00
Suffering	1.12	1.00	1.11	1.00	1.07	1.00	1.06	1.00
Social Well-Being								
Relationship contentment	1.67	1.49	1.39	1.20	1.74	1.51	1.43	1.19
Relationship satisfaction	1.61	1.42	1.35	1.11	1.65	1.43	1.36	1.08
Social support	1.54	1.35	1.22	1.00	1.58	1.35	1.24	1.00
Intimate/close friend	1.08	1.00	1.06	1.00	1.17	1.00	1.14	1.00
Government approval	1.36	1.22	1.31	1.18	1.44	1.30	1.32	1.17
Say in government	1.03	1.00	1.14	1.00	1.16	1.00	1.11	1.00
Belonging in country	1.29	1.00	1.10	1.00	1.32	1.00	1.20	1.00
City/place satisfaction	1.25	1.16	1.23	1.13	1.29	1.18	1.26	1.14
Trust within country	1.12	1.00	1.20	1.00	1.07	1.00	1.15	1.00
Social Participation								
Ever been married	1.06	1.00	1.07	1.00	1.13	1.00	1.15	1.00
Currently divorced	1.05	1.00	1.06	1.00	1.08	1.00	1.08	1.00
Number of children	1.22	1.00	1.19	1.00	1.25	1.00	1.30	1.07
Weekly+ community participation	1.13	1.00	1.06	1.00	1.08	1.00	1.08	1.00
Weekly+ religious attendance	1.10	1.00	1.10	1.00	1.06	1.00	1.16	1.00
Social Distress								
Loneliness	1.39	1.15	1.12	1.00	1.42	1.14	1.18	1.00
Perceived discrimination	1.20	1.09	1.16	1.00	1.19	1.02	1.12	1.00
Character & Prosocial Behavior								
Orientation to promote good	1.67	1.49	1.45	1.24	1.72	1.48	1.48	1.22
Delayed gratification	2.16	1.89	2.01	1.78	2.31	2.00	2.04	1.80
Hope	1.52	1.33	1.15	1.00	1.51	1.25	1.07	1.00
Gratitude	1.72	1.54	1.46	1.27	1.81	1.60	1.51	1.28
Showing love/care	1.59	1.39	1.30	1.00	1.70	1.44	1.39	1.11
Forgivingness	1.84	1.74	1.78	1.68	1.92	1.81	1.85	1.74
Charitable giving	1.30	1.21	1.17	1.00	1.30	1.20	1.11	1.00
Helping strangers	1.33	1.22	1.30	1.18	1.34	1.20	1.28	1.13
Volunteering	1.07	1.00	1.15	1.00	1.10	1.00	1.19	1.07
Physical Health & Health Behavior								
Self-rated physical health	1.34	1.03	1.22	1.00	1.29	1.00	1.22	1.00
Health problems	1.11	1.00	1.12	1.00	1.06	1.00	1.03	1.00
Pain in past 4 weeks	1.13	1.00	1.10	1.00	1.15	1.00	1.14	1.00
Daily smoker	1.06	1.00	1.17	1.00	1.15	1.00	1.28	1.15
Number of drinks per week	1.08	1.00	1.23	1.00	1.12	1.00	1.34	1.00
Days exercise per week	1.56	1.37	1.43	1.22	1.49	1.25	1.31	1.00
Socioeconomic Outcomes								
Financial security	1.13	1.00	1.21	1.00	1.10	1.00	1.23	1.00
Material security	1.21	1.00	1.16	1.00	1.05	1.00	1.24	1.00
Educational attainment (16+ years)	1.06	1.00	1.00	1.00	1.05	1.00	1.03	1.00
Currently employed	1.07	1.00	1.10	1.00	1.08	1.00	1.06	1.00
Financially comfortable/getting by	1.09	1.00	1.18	1.00	1.04	1.00	1.10	1.00
Own home	1.11	1.00	1.12	1.00	1.07	1.00	1.13	1.00
Income – top quintile	1.13	1.00	1.14	1.00	1.13	1.00	1.10	1.00
Religion & Spirituality								
Religious/spiritual connection	1.38	1.27	1.22	1.02	1.31	1.14	1.13	1.00
Belief in life after death	1.20	1.00	1.25	1.10	1.25	1.06	1.27	1.11
Transformative religious experience	1.11	1.00	1.05	1.00	1.13	1.00	1.04	1.00
Religious reading or listening	1.13	1.00	1.08	1.00	1.04	1.00	1.06	1.00
Prayer or meditation	1.24	1.14	1.13	1.00	1.19	1.00	1.10	1.00
Belief in God/gods/spiritual forces	1.07	1.00	1.10	1.00	1.11	1.00	1.14	1.00
Religious centrality	1.40	1.28	1.26	1.10	1.35	1.19	1.22	1.00
Religious/spiritual comfort	1.32	1.19	1.20	1.00	1.27	1.06	1.16	1.00
Feel loved by God	1.25	1.13	1.14	1.00	1.22	1.00	1.15	1.00
Feel punished by God	1.07	1.00	1.10	1.00	1.08	1.00	1.12	1.00
Experienced religious criticism	1.09	1.00	1.05	1.00	1.02	1.00	1.13	1.00
Faith-sharing	1.30	1.18	1.24	1.10	1.37	1.26	1.29	1.18

Notes. EE, E-value for estimate; ECI, E-value for the limit of the confidence interval. The formula for calculating E-values can be found in VanderWeele and Ding (2017). E-values for estimate are the minimum strength of association on the risk ratio scale that an unmeasured confounder would need to have with both the exposure and the outcome to fully explain away the observed association between the exposure and outcome, conditional on the measured covariates. E-values for the 95% CI closest to the null denote the minimum strength of association on the risk ratio scale that an unmeasured confounder would need to have with both the exposure and the outcome to shift the CI to include the null value, conditional on the measured covariates.

Table S25a. Weighted summary statistics for demographic and childhood variables in South Africa

**Characteristic**	**Wave 1** N = 2,651	**Wave 2** N = 957
Forgivingness, n (%)		
Always	1,595 (60.2%)	500 (52.2%)
Often	633 (23.9%)	272 (28.4%)
Rarely	257 (9.7%)	128 (13.4%)
Never	166 (6.3%)	56 (5.9%)
(Missing)	0 (0.0%)	1 (0.2%)
Year of birth, n (%)		
1943 or earlier (current age: 80+ years)	16 (0.6%)	3 (0.3%)
1943-1953 (current age: 70-79 years)	112 (4.2%)	40 (4.2%)
1953-1963 (current age: 60-69 years)	192 (7.2%)	89 (9.3%)
1963-1973 (current age: 50-59 years)	310 (11.7%)	93 (9.8%)
1973-1983 (current age: 40-49 years)	485 (18.3%)	196 (20.5%)
1983-1993 (current age: 30-39 years)	634 (23.9%)	238 (24.8%)
1993-1998 (current age: 25-29 years)	356 (13.4%)	133 (13.9%)
1998-2005 (current age: 18-24 years)	538 (20.3%)	165 (17.3%)
(Missing)	8 (0.3%)	0 (0%)
Age of participant		
Mean	38.8	39.6
Standard Deviation	15.3	14.8
Min, Max	18.0, 86.0	18.0, 84.0
(Missing)	8 (0.3%)	0 (0%)
Gender, n (%)		
Male	1,292 (48.8%)	459 (48.0%)
Female	1,352 (51.0%)	497 (51.9%)
Other	2 (0.1%)	0 (0%)
(Missing)	5 (0.2%)	1 (0.1%)
Respondent marital status, n (%)		
Single/Never been married	1,582 (59.7%)	525 (54.8%)
Married	527 (19.9%)	194 (20.3%)
Separated	73 (2.8%)	28 (2.9%)
Divorced	48 (1.8%)	23 (2.4%)
Widowed	132 (5.0%)	74 (7.7%)
Domestic partner	262 (9.9%)	105 (10.9%)
(Missing)	27 (1.0%)	9 (0.9%)
Education (years), n (%)		
Up to 8	697 (26.3%)	220 (23.0%)
9-15	1,797 (67.8%)	638 (66.6%)
16+	153 (5.8%)	99 (10.4%)
(Missing)	4 (0.1%)	0 (0%)
Employment status, n (%)		
Employed for an employer	549 (20.7%)	200 (20.9%)
Self-employed	401 (15.1%)	120 (12.6%)
Retired	234 (8.8%)	41 (4.3%)
Student	230 (8.7%)	70 (7.3%)
Homemaker	141 (5.3%)	53 (5.5%)
Unemployed and looking for a job	1,020 (38.5%)	403 (42.1%)
None of these/Other	72 (2.7%)	65 (6.8%)
(Missing)	3 (0.1%)	5 (0.5%)
Current religious service attendance, n (%)		
More than once a week	424 (16.0%)	127 (13.3%)

Table S25a. Weighted summary statistics for demographic and childhood variables in South Africa

**Characteristic**	**Wave 1** N = 2,651	**Wave 2** N = 957
Once a week	889 (33.5%)	295 (30.9%)
One to three times a month	568 (21.4%)	206 (21.5%)
A few times a year	427 (16.1%)	186 (19.4%)
Never	337 (12.7%)	140 (14.6%)
(Missing)	7 (0.2%)	3 (0.4%)
Immigration status, n (%)		
Born in this country	2,506 (94.5%)	902 (94.2%)
Born in another country	143 (5.4%)	55 (5.8%)
(Missing)	1 (0.1%)	0 (0%)
Parental marital status around age 12, n (%)		
Parents were married	1,311 (49.5%)	470 (49.1%)
Parents were divorced	133 (5.0%)	35 (3.7%)
Parents were never married	915 (34.5%)	358 (37.4%)
One or both of them had died	139 (5.2%)	43 (4.5%)
Unsure	34 (1.3%)	13 (1.4%)
(Missing)	119 (4.5%)	38 (4.0%)
Religious service attendance around age 12, n (%)		
At least once a week	1,672 (63.1%)	632 (66.1%)
One to three times a month	557 (21.0%)	173 (18.1%)
Less than once a month	173 (6.5%)	67 (7.0%)
Never	222 (8.4%)	80 (8.3%)
(Missing)	27 (1.0%)	6 (0.6%)
Relationship with mother when growing up, n (%)		
Very good	2,184 (82.4%)	765 (79.9%)
Somewhat good	263 (9.9%)	114 (11.9%)
Somewhat bad	51 (1.9%)	25 (2.6%)
Very bad	41 (1.5%)	7 (0.8%)
(Does not apply)	91 (3.4%)	39 (4.1%)
(Missing)	22 (0.8%)	7 (0.8%)
Relationship with father when growing up, n (%)		
Very good	1,647 (62.1%)	559 (58.4%)
Somewhat good	331 (12.5%)	143 (14.9%)
Somewhat bad	88 (3.3%)	40 (4.2%)
Very bad	167 (6.3%)	54 (5.7%)
(Does not apply)	331 (12.5%)	132 (13.7%)
(Missing)	86 (3.3%)	30 (3.1%)
Felt like an outsider in family when growing up, n (%)		
Yes	438 (16.5%)	151 (15.8%)
No	2,208 (83.3%)	804 (83.9%)
(Missing)	6 (0.2%)	3 (0.3%)
Experienced abuse when growing up, n (%)		
Yes	456 (17.2%)	169 (17.7%)
No	2,143 (80.8%)	779 (81.4%)
(Missing)	52 (1.9%)	9 (1.0%)
Self-rated health when growing up, n (%)		
Excellent	1,225 (46.2%)	455 (47.5%)
Very good	584 (22.0%)	201 (21.0%)
Good	365 (13.8%)	106 (11.1%)
Fair	275 (10.4%)	130 (13.6%)
Poor	186 (7.0%)	56 (5.8%)

Table S25a. Weighted summary statistics for demographic and childhood variables in South Africa

**Characteristic**	**Wave 1**<br>N = 2,651	**Wave 2**<br>N = 957
(Missing)	17 (0.6%)	9 (1.0%)
Subjective financial status of family growing up, n (%)		
Lived comfortably	1,048 (39.5%)	352 (36.7%)
Got by	875 (33.0%)	312 (32.6%)
Found it difficult	432 (16.3%)	172 (18.0%)
Found it very difficult	291 (11.0%)	121 (12.6%)
(Missing)	5 (0.2%)	1 (0.1%)
Religious affiliation growing up, n (%)		
Christianity	2,320 (87.5%)	831 (86.8%)
Taoism	1 (0.0%)	1 (0.1%)
Confucianism	0 (0%)	0 (0%)
Primal, Animist, or Folk religion	118 (4.5%)	37 (3.9%)
Spiritism	0 (0%)	0 (0%)
Umbanda, Candomblé, and other African-derived religions	0 (0%)	0 (0%)
Chinese folk/traditional religion	0 (0%)	0 (0%)
Islam	53 (2.0%)	24 (2.5%)
Hinduism	2 (0.1%)	1 (0.1%)
Buddhism	11 (0.4%)	3 (0.3%)
Judaism	0 (0%)	0 (0%)
Sikhism	0 (0%)	0 (0%)
Baha'i	0 (0%)	0 (0%)
Jainism	0 (0%)	0 (0%)
Shinto	2 (0.1%)	2 (0.2%)
Some other religion	7 (0.3%)	5 (0.5%)
No religion/Atheist/Agnostic	110 (4.1%)	46 (4.8%)
(Missing)	27 (1.0%)	8 (0.8%)

Note. N (%); this table is based on non-imputed data. Cumulative percentages for variables may not add up to 100% due to rounding. Wave 1 characteristics weighted using the Gallup provided sampling weight, ANNUAL_WEIGHT_R2; Wave 2 characteristics weighted accounting for attrition by using the adjusted Wave 1 weight, ANNUAL_WEIGHT_R2, multiplied by the created attrition weight to account for dropout, to maintain nationally representative estimates for Wave 2 characteristics.

Table S25b. Weighted summary statistics for outcome variables in South Africa

Outcome	Wave 1<br>N = 2,651	Wave 2<br>N = 957
Secure flourishing index		
Mean	7.1	6.9
Standard Deviation	1.5	1.6
Min, Max	0.8, 10.0	0.0, 10.0
(Missing)	62 (2.3%)	19 (2.0%)
Flourishing index		
Mean	7.4	7.3
Standard Deviation	1.6	1.6
Min, Max	1.0, 10.0	0.0, 10.0
(Missing)	51 (1.9%)	12 (1.3%)
Happiness & life satisfaction		
Mean	6.7	6.2
Standard Deviation	2.4	2.7
Min, Max	0.0, 10.0	0.0, 10.0
(Missing)	10 (0.4%)	7 (0.8%)
Physical & mental health		
Mean	7.9	7.5
Standard Deviation	2.2	2.3
Min, Max	0.0, 10.0	0.0, 10.0
(Missing)	11 (0.4%)	0 (<0.1%)
Meaning & purpose		
Mean	7.4	7.5
Standard Deviation	2.1	2.1
Min, Max	0.0, 10.0	0.0, 10.0
(Missing)	17 (0.6%)	3 (0.3%)
Character & virtue		
Mean	7.8	7.8
Standard Deviation	2.0	2.2
Min, Max	0.0, 10.0	0.0, 10.0
(Missing)	12 (0.5%)	1 (0.1%)
Close social relationships		
Mean	7.4	7.5
Standard Deviation	2.3	2.4
Min, Max	0.0, 10.0	0.0, 10.0
(Missing)	9 (0.3%)	1 (<0.1%)
Financial & material security		
Mean	5.4	4.7
Standard Deviation	3.0	3.1
Min, Max	0.0, 10.0	0.0, 10.0
(Missing)	13 (0.5%)	7 (0.7%)
Happiness		
Mean	7.0	6.5
Standard Deviation	2.7	3.0
Min, Max	0.0, 10.0	0.0, 10.0
(Missing)	1 (<0.1%)	1 (0.1%)
Life satisfaction		
Mean	6.4	6.0
Standard Deviation	2.8	3.1
Min, Max	0.0, 10.0	0.0, 10.0
(Missing)	10 (0.4%)	6 (0.6%)
Current life evaluation		

Table S25b. Weighted summary statistics for outcome variables in South Africa

**Outcome**	**Wave 1** N = 2,651	**Wave 2** N = 957
Mean	6.1	5.9
Standard Deviation	2.9	3.1
Min, Max	0.0, 10.0	0.0, 10.0
(Missing)	6 (0.2%)	0 (0%)
Future life evaluation		
Mean	7.6	7.5
Standard Deviation	2.5	2.8
Min, Max	0.0, 10.0	0.0, 10.0
(Missing)	70 (2.6%)	18 (1.9%)
Optimism		
Mean	8.2	8.4
Standard Deviation	2.2	2.5
Min, Max	0.0, 10.0	0.0, 10.0
(Missing)	9 (0.4%)	0 (0%)
Freedom to pursue what's important		
Mean	8.0	7.8
Standard Deviation	2.4	2.8
Min, Max	0.0, 10.0	0.0, 10.0
(Missing)	5 (0.2%)	0 (0%)
Inner peace, n (%)		
Always	1,170 (44.1%)	268 (28.0%)
Often	940 (35.5%)	363 (37.9%)
Rarely	340 (12.8%)	207 (21.7%)
Never	182 (6.9%)	112 (11.7%)
(Missing)	19 (0.7%)	7 (0.7%)
Life balance, n (%)		
Always	842 (31.8%)	168 (17.6%)
Often	1,014 (38.2%)	348 (36.3%)
Rarely	492 (18.6%)	298 (31.1%)
Never	286 (10.8%)	139 (14.5%)
(Missing)	17 (0.6%)	5 (0.5%)
Sense of mastery, n (%)		
Always	1,448 (54.6%)	403 (42.1%)
Often	783 (29.5%)	318 (33.2%)
Rarely	283 (10.7%)	168 (17.6%)
Never	128 (4.8%)	67 (7.0%)
(Missing)	10 (0.4%)	1 (0.1%)
Meaningful activities		
Mean	6.7	6.9
Standard Deviation	2.8	2.9
Min, Max	0.0, 10.0	0.0, 10.0
(Missing)	12 (0.5%)	3 (0.3%)
Understanding purpose		
Mean	8.1	8.1
Standard Deviation	2.4	2.6
Min, Max	0.0, 10.0	0.0, 10.0
(Missing)	4 (0.2%)	0 (<0.1%)
Self-rated mental health		
Mean	8.2	7.8
Standard Deviation	2.5	2.7
Min, Max	0.0, 10.0	0.0, 10.0

Table S25b. Weighted summary statistics for outcome variables in South Africa

Outcome	Wave 1<br>N = 2,651	Wave 2<br>N = 957
(Missing)	10 (0.4%)	0 (0%)
Traumatic distress, n (%)		
A lot	432 (16.3%)	215 (22.5%)
Some	462 (17.4%)	182 (19.0%)
Not very much	657 (24.8%)	296 (30.9%)
Not at all	1,014 (38.3%)	262 (27.4%)
(Missing)	86 (3.2%)	3 (0.3%)
Depression symptoms composite, n (%)	858 (32.6%)	375 (39.3%)
(Missing)	23 (0.9%)	4 (0.4%)
Depression – feel hopeless, n (%)		
Nearly every day	272 (10.3%)	166 (17.3%)
More than half the days	354 (13.4%)	149 (15.6%)
Several days	1,000 (37.7%)	334 (34.9%)
Not at all	1,009 (38.1%)	308 (32.2%)
(Missing)	16 (0.6%)	0 (0.0%)
Depression – loss of interest, n (%)		
Nearly every day	359 (13.6%)	133 (13.9%)
More than half the days	382 (14.4%)	145 (15.2%)
Several days	925 (34.9%)	355 (37.1%)
Not at all	973 (36.7%)	320 (33.4%)
(Missing)	12 (0.5%)	4 (0.4%)
Anxiety symptoms composite, n (%)	774 (29.5%)	407 (42.5%)
(Missing)	26 (1.0%)	1 (0.1%)
Anxiety – feel on edge, n (%)		
Nearly every day	236 (8.9%)	127 (13.3%)
More than half the days	352 (13.3%)	158 (16.5%)
Several days	925 (34.9%)	321 (33.5%)
Not at all	1,130 (42.6%)	352 (36.7%)
(Missing)	8 (0.3%)	0 (0%)
Anxiety – cannot stop worrying, n (%)		
Nearly every day	400 (15.1%)	188 (19.6%)
More than half the days	303 (11.4%)	170 (17.7%)
Several days	880 (33.2%)	314 (32.8%)
Not at all	1,048 (39.5%)	285 (29.8%)
(Missing)	21 (0.8%)	1 (0.1%)
Suffering, n (%)		
A lot	321 (12.1%)	172 (17.9%)
Some	471 (17.8%)	177 (18.5%)
Not very much	701 (26.5%)	357 (37.3%)
Not at all	1,140 (43.0%)	242 (25.3%)
(Missing)	18 (0.7%)	10 (1.0%)
Relationship contentment		
Mean	7.6	7.7
Standard Deviation	2.5	2.7
Min, Max	0.0, 10.0	0.0, 10.0
(Missing)	6 (0.2%)	0 (<0.1%)
Relationship satisfaction		
Mean	7.2	7.2
Standard Deviation	2.7	2.9
Min, Max	0.0, 10.0	0.0, 10.0
(Missing)	5 (0.2%)	1 (<0.1%)

Table S25b. Weighted summary statistics for outcome variables in South Africa

Outcome	Wave 1<br>N = 2,651	Wave 2<br>N = 957
Social support		
Mean	7.2	6.5
Standard Deviation	2.8	3.4
Min, Max	0.0, 10.0	0.0, 10.0
(Missing)	7 (0.3%)	1 (0.1%)
Intimate/close friend, n (%)		
Yes	2,199 (83.0%)	837 (87.4%)
No	450 (17.0%)	120 (12.6%)
(Missing)	2 (0.1%)	0 (0.0%)
Government approval, n (%)		
Strongly approve	580 (21.9%)	173 (18.1%)
Somewhat approve	424 (16.0%)	177 (18.5%)
Neither approve nor disapprove	580 (21.9%)	239 (25.0%)
Somewhat disapprove	294 (11.1%)	165 (17.3%)
Strongly disapprove	744 (28.1%)	198 (20.7%)
(Missing)	31 (1.2%)	5 (0.5%)
Say in government, n (%)		
Agree	1,317 (49.7%)	388 (40.5%)
Disagree	816 (30.8%)	269 (28.1%)
Unsure	508 (19.2%)	296 (31.0%)
(Missing)	10 (0.4%)	4 (0.4%)
Belonging in country		
Mean	7.7	7.5
Standard Deviation	2.7	2.9
Min, Max	0.0, 10.0	0.0, 10.0
(Missing)	17 (0.6%)	0 (<0.1%)
City/place satisfaction, n (%)		
Satisfied	1,465 (55.3%)	505 (52.8%)
Dissatisfied	873 (32.9%)	323 (33.7%)
Unsure	312 (11.8%)	126 (13.2%)
(Missing)	2 (0.1%)	3 (0.3%)
Trust within country, n (%)		
All people	89 (3.4%)	19 (2.0%)
Most people	194 (7.3%)	77 (8.0%)
Some people	679 (25.6%)	320 (33.4%)
Not very many people	903 (34.1%)	368 (38.4%)
None	743 (28.0%)	162 (16.9%)
(Missing)	42 (1.6%)	12 (1.2%)
Number of children		
Mean	1.9	2.6
Standard Deviation	1.9	3.0
Min, Max	0.0, 18.0	0.0, 44.0
(Missing)	3 (0.1%)	15 (1.6%)
Community participation, n (%)		
More than once a week	272 (10.3%)	59 (6.1%)
Once a week	380 (14.4%)	153 (15.9%)
One to three times a month	612 (23.1%)	179 (18.7%)
A few times a year	531 (20.0%)	258 (27.0%)
Never	846 (31.9%)	304 (31.7%)
(Missing)	9 (0.3%)	5 (0.5%)
Religious attendance, n (%)		

Table S25b. Weighted summary statistics for outcome variables in South Africa

**Outcome**	**Wave 1** N = 2,651	**Wave 2** N = 957
More than once a week	424 (16.0%)	127 (13.3%)
Once a week	889 (33.5%)	295 (30.9%)
One to three times a month	568 (21.4%)	206 (21.5%)
A few times a year	427 (16.1%)	186 (19.4%)
Never	337 (12.7%)	140 (14.6%)
(Missing)	7 (0.2%)	3 (0.4%)
Loneliness		
Mean	3.7	4.0
Standard Deviation	3.1	3.5
Min, Max	0.0, 10.0	0.0, 10.0
(Missing)	2 (<0.1%)	0 (0%)
Perceived discrimination, n (%)		
Always	427 (16.1%)	139 (14.5%)
Often	544 (20.5%)	194 (20.2%)
Rarely	497 (18.7%)	275 (28.8%)
Never	1,133 (42.8%)	345 (36.1%)
(Missing)	49 (1.9%)	4 (0.4%)
Orientation to promote good		
Mean	7.8	8.0
Standard Deviation	2.4	2.4
Min, Max	0.0, 10.0	0.0, 10.0
(Missing)	2 (<0.1%)	1 (<0.1%)
Delayed gratification		
Mean	7.7	7.5
Standard Deviation	2.4	2.9
Min, Max	0.0, 10.0	0.0, 10.0
(Missing)	10 (0.4%)	1 (<0.1%)
Hope		
Mean	8.6	8.7
Standard Deviation	2.1	2.1
Min, Max	0.0, 10.0	0.0, 10.0
(Missing)	5 (0.2%)	2 (0.2%)
Gratitude		
Mean	7.8	7.7
Standard Deviation	2.4	2.7
Min, Max	0.0, 10.0	0.0, 10.0
(Missing)	8 (0.3%)	2 (0.2%)
Showing love/care		
Mean	8.6	8.7
Standard Deviation	2.0	2.2
Min, Max	0.0, 10.0	0.0, 10.0
(Missing)	6 (0.2%)	0 (0%)
Forgivingness, n (%)		
Always	1,595 (60.2%)	500 (52.2%)
Often	633 (23.9%)	272 (28.4%)
Rarely	257 (9.7%)	128 (13.4%)
Never	166 (6.3%)	56 (5.9%)
(Missing)	0 (0.0%)	1 (0.2%)
Charitable giving, n (%)		
Yes	505 (19.1%)	246 (25.7%)
No	2,145 (80.9%)	706 (73.7%)

Table S25b. Weighted summary statistics for outcome variables in South Africa

Outcome	Wave 1<br>N = 2,651	Wave 2<br>N = 957
(Missing)	1 (0.0%)	6 (0.6%)
Helping strangers, n (%)		
Yes	1,520 (57.3%)	746 (77.9%)
No	1,119 (42.2%)	204 (21.3%)
(Missing)	12 (0.4%)	7 (0.8%)
Volunteering, n (%)		
Yes	649 (24.5%)	331 (34.6%)
No	2,002 (75.5%)	618 (64.6%)
(Missing)	0 (0%)	8 (0.9%)
Self-rated physical health		
Mean	7.5	7.2
Standard Deviation	2.7	2.8
Min, Max	0.0, 10.0	0.0, 10.0
(Missing)	3 (0.1%)	0 (<0.1%)
Health problems, n (%)		
Yes	575 (21.7%)	230 (24.0%)
No	2,044 (77.1%)	720 (75.2%)
(Missing)	31 (1.2%)	7 (0.8%)
Pain in past 4 weeks, n (%)		
A lot	290 (10.9%)	158 (16.5%)
Some	471 (17.8%)	180 (18.8%)
Not very much	753 (28.4%)	353 (36.8%)
None at all	1,129 (42.6%)	267 (27.8%)
(Missing)	8 (0.3%)	0 (0%)
Number of cigarettes per day		
Mean	1.7	1.6
Standard Deviation	4.5	4.2
Min, Max	0.0, 60.0	0.0, 67.0
(Missing)	47 (1.8%)	10 (1.0%)
Number of drinks per week		
Mean	2.4	1.7
Standard Deviation	6.1	3.4
Min, Max	0.0, 97.0	0.0, 24.0
(Missing)	51 (1.9%)	8 (0.9%)
Days exercise per week		
Mean	2.4	2.5
Standard Deviation	2.4	2.5
Min, Max	0.0, 7.0	0.0, 7.0
(Missing)	37 (1.4%)	7 (0.8%)
Financial security		
Mean	5.2	4.5
Standard Deviation	3.3	3.6
Min, Max	0.0, 10.0	0.0, 10.0
(Missing)	10 (0.4%)	1 (0.1%)
Material security		
Mean	5.6	4.9
Standard Deviation	3.4	3.7
Min, Max	0.0, 10.0	0.0, 10.0
(Missing)	5 (0.2%)	6 (0.6%)
Educational attainment (16+ years), n (%)		
Up to 8	697 (26.3%)	220 (23.0%)

Table S25b. Weighted summary statistics for outcome variables in South Africa

Outcome	Wave 1<br>N = 2,651	Wave 2<br>N = 957
9-15	1,797 (67.8%)	638 (66.6%)
16+	153 (5.8%)	99 (10.4%)
(Missing)	4 (0.1%)	0 (0%)
Currently employed, n (%)		
Employed for an employer	549 (20.7%)	200 (20.9%)
Self-employed	401 (15.1%)	120 (12.6%)
Retired	234 (8.8%)	41 (4.3%)
Student	230 (8.7%)	70 (7.3%)
Homemaker	141 (5.3%)	53 (5.5%)
Unemployed and looking for a job	1,020 (38.5%)	403 (42.1%)
None of these/Other	72 (2.7%)	65 (6.8%)
(Missing)	3 (0.1%)	5 (0.5%)
Financially comfortable/getting by, n (%)		
Living comfortably on present income	335 (12.6%)	94 (9.8%)
Getting by on present income	940 (35.4%)	220 (23.0%)
Finding it difficult on present income	850 (32.0%)	315 (32.9%)
Finding it very difficult on present income	460 (17.4%)	321 (33.5%)
(Missing)	67 (2.5%)	8 (0.8%)
Own home, n (%)		
Someone in this household owns this home	1,699 (64.1%)	511 (53.4%)
Someone in this household rents this home	382 (14.4%)	112 (11.7%)
Both	165 (6.2%)	73 (7.6%)
Neither	358 (13.5%)	255 (26.6%)
Rent	0 (0%)	0 (0%)
Own	0 (0%)	0 (0%)
Something else	0 (0%)	0 (0%)
(Missing)	47 (1.8%)	6 (0.7%)
Religious/spiritual connection, n (%)		
Always	1,658 (62.5%)	463 (48.4%)
Often	573 (21.6%)	246 (25.7%)
Rarely	243 (9.2%)	166 (17.4%)
Never	157 (5.9%)	80 (8.4%)
(Missing)	20 (0.7%)	2 (0.2%)
Belief in life after death, n (%)		
Yes	1,390 (52.4%)	526 (54.9%)
No	759 (28.6%)	171 (17.8%)
Unsure	487 (18.4%)	255 (26.6%)
(Missing)	14 (0.5%)	6 (0.6%)
Transformative religious experience, n (%)		
Yes	1,542 (58.2%)	556 (58.1%)
No	1,036 (39.1%)	396 (41.4%)
(Missing)	74 (2.8%)	6 (0.6%)
Religious reading or listening, n (%)		
More than once a day	442 (16.7%)	145 (15.1%)
About once a day	476 (18.0%)	197 (20.6%)
Sometimes	1,392 (52.5%)	507 (53.0%)
Never	331 (12.5%)	104 (10.9%)
(Missing)	11 (0.4%)	5 (0.5%)
Prayer or meditation, n (%)		
More than once a day	1,160 (43.7%)	396 (41.4%)
About once a day	726 (27.4%)	280 (29.3%)

Table S25b. Weighted summary statistics for outcome variables in South Africa

Outcome	Wave 1<br>N = 2,651	Wave 2<br>N = 957
Sometimes	686 (25.9%)	242 (25.3%)
Never	76 (2.9%)	35 (3.6%)
(Missing)	3 (0.1%)	4 (0.5%)
Belief in God/gods/spiritual forces, n (%)		
One God	2,128 (80.3%)	780 (81.4%)
More than one god	266 (10.0%)	70 (7.4%)
An impersonal spiritual force	135 (5.1%)	49 (5.1%)
None of these	60 (2.3%)	13 (1.4%)
Unsure	57 (2.1%)	41 (4.3%)
(Missing)	6 (0.2%)	3 (0.4%)
Religious centrality, n (%)		
Agree	2,072 (78.2%)	675 (70.5%)
Disagree	252 (9.5%)	60 (6.3%)
Not relevant	135 (5.1%)	76 (8.0%)
Unsure	186 (7.0%)	144 (15.0%)
(Missing)	5 (0.2%)	2 (0.2%)
Religious/spiritual comfort, n (%)		
Agree	2,313 (87.3%)	809 (84.5%)
Disagree	157 (5.9%)	21 (2.2%)
Not relevant	100 (3.8%)	54 (5.7%)
Unsure	73 (2.7%)	71 (7.4%)
(Missing)	8 (0.3%)	2 (0.2%)
Feel loved by God, n (%)		
Agree	2,421 (91.3%)	844 (88.1%)
Disagree	86 (3.3%)	22 (2.3%)
Not relevant	78 (2.9%)	28 (2.9%)
Unsure	58 (2.2%)	60 (6.3%)
(Missing)	8 (0.3%)	3 (0.3%)
Feel punished by God, n (%)		
Agree	1,088 (41.0%)	289 (30.2%)
Disagree	1,156 (43.6%)	360 (37.6%)
Not relevant	215 (8.1%)	107 (11.2%)
Unsure	183 (6.9%)	199 (20.8%)
(Missing)	9 (0.3%)	2 (0.2%)
Experienced religious criticism, n (%)		
Agree	1,039 (39.2%)	292 (30.5%)
Disagree	903 (34.1%)	269 (28.1%)
Not relevant	329 (12.4%)	129 (13.5%)
Unsure	371 (14.0%)	264 (27.6%)
(Missing)	9 (0.3%)	4 (0.4%)
Faith-sharing, n (%)		
Agree	1,908 (72.0%)	662 (69.1%)
Disagree	464 (17.5%)	127 (13.2%)
Not relevant	171 (6.5%)	90 (9.4%)
Unsure	95 (3.6%)	77 (8.0%)
(Missing)	12 (0.4%)	2 (0.2%)

Note. N (%); this table is based on non-imputed data. Cumulative percentages for variables may not add up to 100% due to rounding. Wave 1 characteristics weighted using the Gallup provided sampling weight, ANNUAL_WEIGHT_R2; Wave 2 characteristics weighted accounting for attrition by using the adjusted Wave 1 weight, ANNUAL_WEIGHT_R2, multiplied by the created attrition weight to account for dropout, to maintain nationally representative estimates for Wave 2 characteristics.

Table S25c. Unweighted summary statistics for demographic and childhood variables in South Africa by retention status

Characteristic	Attritors–Not Observed in Wave 2<br>N = 1,786	Retained–Observed in Wave 2<br>N = 899
Forgivingness, n (%)		
Always	1,064 (59.6%)	551 (61.3%)
Often	430 (24.1%)	211 (23.4%)
Rarely	182 (10.2%)	78 (8.7%)
Never	110 (6.1%)	59 (6.5%)
(Missing)	0 (0.0%)	0 (0%)
Year of birth, n (%)		
1943 or earlier (current age: 80+ years)	16 (0.9%)	0 (0.0%)
1943-1953 (current age: 70-79 years)	81 (4.5%)	33 (3.6%)
1953-1963 (current age: 60-69 years)	107 (6.0%)	87 (9.6%)
1963-1973 (current age: 50-59 years)	221 (12.4%)	94 (10.4%)
1973-1983 (current age: 40-49 years)	338 (18.9%)	154 (17.2%)
1983-1993 (current age: 30-39 years)	423 (23.7%)	218 (24.3%)
1993-1998 (current age: 25-29 years)	243 (13.6%)	118 (13.1%)
1998-2005 (current age: 18-24 years)	351 (19.7%)	193 (21.5%)
(Missing)	7 (0.4%)	1 (0.1%)
Age of participant		
Mean	38.9	38.6
Standard Deviation	15.4	15.3
Min, Max	18.0, 86.0	18.0, 83.0
(Missing)	7 (0.4%)	1 (0.1%)
Gender, n (%)		
Male	884 (49.5%)	425 (47.3%)
Female	896 (50.2%)	472 (52.5%)
Other	2 (0.1%)	0 (0%)
(Missing)	3 (0.2%)	1 (0.2%)
Respondent marital status, n (%)		
Single/Never been married	1,064 (59.6%)	538 (59.8%)
Married	341 (19.1%)	191 (21.3%)
Separated	55 (3.1%)	19 (2.1%)
Divorced	39 (2.2%)	10 (1.1%)
Widowed	80 (4.5%)	53 (5.9%)
Domestic partner	185 (10.4%)	81 (9.0%)
(Missing)	21 (1.2%)	7 (0.8%)
Education (years), n (%)		
Up to 8	470 (26.3%)	236 (26.3%)
9-15	1,211 (67.8%)	609 (67.7%)
16+	101 (5.7%)	54 (6.0%)
(Missing)	4 (0.2%)	0 (0%)
Employment status, n (%)		
Employed for an employer	401 (22.5%)	156 (17.3%)
Self-employed	276 (15.5%)	130 (14.5%)
Retired	149 (8.3%)	88 (9.8%)
Student	143 (8.0%)	89 (9.9%)
Homemaker	93 (5.2%)	50 (5.5%)
Unemployed and looking for a job	673 (37.7%)	360 (40.0%)
None of these/Other	48 (2.7%)	25 (2.7%)
(Missing)	2 (0.1%)	2 (0.2%)
Current religious service attendance, n (%)		

Table S25c. Unweighted summary statistics for demographic and childhood variables in South Africa by retention status

Characteristic	Attritors–Not Observed in Wave 2<br>N = 1,786	Retained–Observed in Wave 2<br>N = 899
More than once a week	298 (16.7%)	132 (14.7%)
Once a week	582 (32.6%)	317 (35.3%)
One to three times a month	379 (21.2%)	196 (21.8%)
A few times a year	301 (16.8%)	132 (14.7%)
Never	221 (12.4%)	120 (13.3%)
(Missing)	5 (0.3%)	2 (0.2%)
Immigration status, n (%)		
Born in this country	1,680 (94.1%)	857 (95.4%)
Born in another country	104 (5.8%)	41 (4.6%)
(Missing)	1 (0.1%)	0 (0%)
Parental marital status around age 12, n (%)		
Parents were married	884 (49.5%)	444 (49.4%)
Parents were divorced	100 (5.6%)	35 (3.9%)
Parents were never married	592 (33.2%)	334 (37.1%)
One or both of them had died	101 (5.7%)	40 (4.4%)
Unsure	21 (1.2%)	13 (1.5%)
(Missing)	87 (4.9%)	33 (3.7%)
Religious service attendance around age 12, n (%)		
At least once a week	1,094 (61.3%)	597 (66.5%)
One to three times a month	405 (22.7%)	160 (17.8%)
Less than once a month	111 (6.2%)	64 (7.1%)
Never	154 (8.6%)	72 (8.0%)
(Missing)	22 (1.2%)	5 (0.6%)
Relationship with mother when growing up, n (%)		
Very good	1,495 (83.7%)	718 (79.8%)
Somewhat good	162 (9.1%)	103 (11.5%)
Somewhat bad	26 (1.5%)	25 (2.7%)
Very bad	34 (1.9%)	8 (0.9%)
(Does not apply)	54 (3.0%)	38 (4.2%)
(Missing)	14 (0.8%)	8 (0.9%)
Relationship with father when growing up, n (%)		
Very good	1,143 (64.0%)	526 (58.5%)
Somewhat good	206 (11.5%)	129 (14.4%)
Somewhat bad	49 (2.7%)	39 (4.4%)
Very bad	115 (6.4%)	55 (6.1%)
(Does not apply)	212 (11.9%)	123 (13.7%)
(Missing)	61 (3.4%)	27 (3.0%)
Felt like an outsider in family when growing up, n (%)		
Yes	300 (16.8%)	143 (16.0%)
No	1,482 (83.0%)	753 (83.8%)
(Missing)	3 (0.2%)	3 (0.3%)
Experienced abuse when growing up, n (%)		
Yes	306 (17.1%)	156 (17.3%)
No	1,436 (80.4%)	734 (81.6%)
(Missing)	43 (2.4%)	9 (1.0%)
Self-rated health when growing up, n (%)		
Excellent	807 (45.2%)	432 (48.1%)

Table S25c. Unweighted summary statistics for demographic and childhood variables in South Africa by retention status

**Characteristic**	**Attritors–Not Observed in Wave 2** N = 1,786	**Retained–Observed in Wave 2** N = 899
Very good	403 (22.6%)	188 (20.9%)
Good	272 (15.2%)	99 (11.0%)
Fair	161 (9.0%)	116 (12.9%)
Poor	135 (7.6%)	54 (6.0%)
(Missing)	7 (0.4%)	10 (1.1%)
Subjective financial status of family growing up, n (%)		
Lived comfortably	723 (40.5%)	338 (37.6%)
Got by	599 (33.5%)	288 (32.0%)
Found it difficult	282 (15.8%)	156 (17.3%)
Found it very difficult	178 (10.0%)	116 (12.9%)
(Missing)	4 (0.2%)	1 (0.2%)
Religious affiliation growing up, n (%)		
Christianity	1,567 (87.7%)	782 (87.0%)
Taoism	0 (0%)	1 (0.1%)
Confucianism	0 (0%)	0 (0%)
Primal, Animist, or Folk religion	80 (4.5%)	40 (4.4%)
Spiritism	0 (0%)	0 (0%)
Umbanda, Candomblé, and other African-derived religions	0 (0%)	0 (0%)
Chinese folk/traditional religion	0 (0%)	0 (0%)
Islam	33 (1.8%)	21 (2.3%)
Hinduism	1 (0.1%)	1 (0.1%)
Buddhism	8 (0.5%)	3 (0.3%)
Judaism	0 (0%)	0 (0%)
Sikhism	0 (0%)	0 (0%)
Baha'i	0 (0%)	0 (0%)
Jainism	0 (0%)	0 (0%)
Shinto	0 (0%)	2 (0.2%)
Some other religion	4 (0.3%)	2 (0.3%)
No religion/Atheist/Agnostic	72 (4.0%)	39 (4.4%)
(Missing)	21 (1.2%)	7 (0.8%)

Note. N (%); this table is based on non-imputed data. Cumulative percentages for variables may not add up to 100% due to rounding.

Table S25d. Unweighted summary statistics for Wave 1 outcome variables in South Africa by retention status.

Outcome	Attritors–Not Observed in Wave 2<br>N = 1,786	Retained–Observed in Wave 2<br>N = 899
Secure flourishing index		
Mean	7.0	7.2
Standard Deviation	1.5	1.6
Min, Max	1.0, 10.0	0.8, 10.0
(Missing)	47 (2.6%)	16 (1.8%)
Flourishing index		
Mean	7.4	7.5
Standard Deviation	1.6	1.6
Min, Max	1.2, 10.0	1.0, 10.0
(Missing)	37 (2.1%)	14 (1.6%)
Happiness & life satisfaction		
Mean	6.7	6.7
Standard Deviation	2.3	2.5
Min, Max	0.0, 10.0	0.0, 10.0
(Missing)	8 (0.5%)	2 (0.2%)
Physical & mental health		
Mean	7.8	7.9
Standard Deviation	2.2	2.3
Min, Max	0.0, 10.0	0.0, 10.0
(Missing)	8 (0.4%)	4 (0.4%)
Meaning & purpose		
Mean	7.3	7.5
Standard Deviation	2.1	2.1
Min, Max	0.0, 10.0	0.0, 10.0
(Missing)	8 (0.5%)	8 (0.9%)
Character & virtue		
Mean	7.6	8.0
Standard Deviation	2.1	1.9
Min, Max	0.0, 10.0	0.0, 10.0
(Missing)	11 (0.6%)	2 (0.2%)
Close social relationships		
Mean	7.3	7.6
Standard Deviation	2.2	2.4
Min, Max	0.0, 10.0	0.0, 10.0
(Missing)	5 (0.3%)	4 (0.5%)
Financial & material security		
Mean	5.3	5.6
Standard Deviation	2.9	3.1
Min, Max	0.0, 10.0	0.0, 10.0
(Missing)	11 (0.6%)	3 (0.3%)
Happiness		
Mean	6.9	7.0
Standard Deviation	2.6	2.7
Min, Max	0.0, 10.0	0.0, 10.0
(Missing)	1 (<0.1%)	0 (0%)
Life satisfaction		
Mean	6.4	6.3
Standard Deviation	2.8	2.9
Min, Max	0.0, 10.0	0.0, 10.0

Table S25d. Unweighted summary statistics for Wave 1 outcome variables in South Africa by retention status.

**Outcome**	**Attritors–Not Observed in Wave 2** N = 1,786	**Retained–Observed in Wave 2** N = 899
(Missing)	8 (0.5%)	2 (0.2%)
Current life evaluation		
Mean	6.1	6.1
Standard Deviation	2.8	2.9
Min, Max	0.0, 10.0	0.0, 10.0
(Missing)	3 (0.2%)	3 (0.3%)
Future life evaluation		
Mean	7.5	7.8
Standard Deviation	2.5	2.4
Min, Max	0.0, 10.0	0.0, 10.0
(Missing)	45 (2.5%)	25 (2.8%)
Optimism		
Mean	8.2	8.3
Standard Deviation	2.2	2.3
Min, Max	0.0, 10.0	0.0, 10.0
(Missing)	6 (0.3%)	4 (0.4%)
Freedom to pursue what's important		
Mean	7.9	8.2
Standard Deviation	2.4	2.4
Min, Max	0.0, 10.0	0.0, 10.0
(Missing)	2 (0.1%)	3 (0.3%)
Inner peace, n (%)		
Always	791 (44.3%)	394 (43.9%)
Often	631 (35.4%)	320 (35.6%)
Rarely	221 (12.4%)	123 (13.7%)
Never	128 (7.2%)	56 (6.3%)
(Missing)	14 (0.8%)	5 (0.5%)
Life balance, n (%)		
Always	592 (33.2%)	261 (29.1%)
Often	676 (37.9%)	350 (39.0%)
Rarely	311 (17.4%)	186 (20.7%)
Never	194 (10.9%)	96 (10.6%)
(Missing)	12 (0.7%)	6 (0.6%)
Sense of mastery, n (%)		
Always	971 (54.4%)	495 (55.1%)
Often	528 (29.5%)	265 (29.5%)
Rarely	193 (10.8%)	94 (10.4%)
Never	88 (5.0%)	41 (4.6%)
(Missing)	6 (0.3%)	4 (0.5%)
Meaningful activities		
Mean	6.6	6.8
Standard Deviation	2.8	2.8
Min, Max	0.0, 10.0	0.0, 10.0
(Missing)	6 (0.3%)	7 (0.8%)
Understanding purpose		
Mean	8.0	8.2
Standard Deviation	2.4	2.4
Min, Max	0.0, 10.0	0.0, 10.0
(Missing)	3 (0.2%)	1 (0.2%)

Table S25d. Unweighted summary statistics for Wave 1 outcome variables in South Africa by retention status.

**Outcome**	**Attritors–Not Observed in Wave 2** N = 1,786	**Retained–Observed in Wave 2** N = 899
Self-rated mental health		
Mean	8.2	8.2
Standard Deviation	2.4	2.6
Min, Max	0.0, 10.0	0.0, 10.0
(Missing)	6 (0.3%)	4 (0.4%)
Traumatic distress, n (%)		
A lot	277 (15.5%)	160 (17.8%)
Some	310 (17.3%)	158 (17.6%)
Not very much	455 (25.5%)	211 (23.4%)
Not at all	685 (38.4%)	342 (38.0%)
(Missing)	59 (3.3%)	28 (3.1%)
Depression symptoms composite, n (%)	585 (33.2%)	283 (31.6%)
(Missing)	22 (1.2%)	2 (0.2%)
Depression – feel hopeless, n (%)		
Nearly every day	177 (9.9%)	98 (10.9%)
More than half the days	242 (13.6%)	116 (12.9%)
Several days	686 (38.4%)	327 (36.4%)
Not at all	664 (37.2%)	357 (39.7%)
(Missing)	15 (0.9%)	1 (0.1%)
Depression – loss of interest, n (%)		
Nearly every day	247 (13.9%)	117 (13.0%)
More than half the days	255 (14.3%)	131 (14.6%)
Several days	635 (35.6%)	302 (33.6%)
Not at all	637 (35.7%)	347 (38.6%)
(Missing)	11 (0.6%)	2 (0.2%)
Anxiety symptoms composite, n (%)	547 (31.0%)	238 (26.7%)
(Missing)	21 (1.2%)	5 (0.6%)
Anxiety – feel on edge, n (%)		
Nearly every day	158 (8.8%)	81 (9.0%)
More than half the days	255 (14.3%)	102 (11.4%)
Several days	615 (34.4%)	321 (35.7%)
Not at all	751 (42.0%)	393 (43.7%)
(Missing)	7 (0.4%)	1 (0.1%)
Anxiety – cannot stop worrying, n (%)		
Nearly every day	286 (16.0%)	119 (13.3%)
More than half the days	202 (11.3%)	105 (11.7%)
Several days	595 (33.3%)	296 (32.9%)
Not at all	686 (38.4%)	374 (41.6%)
(Missing)	16 (0.9%)	5 (0.6%)
Suffering, n (%)		
A lot	222 (12.4%)	103 (11.5%)
Some	315 (17.7%)	161 (17.9%)
Not very much	490 (27.4%)	221 (24.6%)
Not at all	742 (41.5%)	411 (45.8%)
(Missing)	17 (1.0%)	2 (0.2%)
Relationship contentment		
Mean	7.5	7.7
Standard Deviation	2.5	2.7
Min, Max	0.0, 10.0	0.0, 10.0

Table S25d. Unweighted summary statistics for Wave 1 outcome variables in South Africa by retention status.

**Outcome**	**Attritors–Not Observed in Wave 2** N = 1,786	**Retained–Observed in Wave 2** N = 899
(Missing)	4 (0.2%)	2 (0.3%)
Relationship satisfaction		
Mean	7.1	7.4
Standard Deviation	2.7	2.8
Min, Max	0.0, 10.0	0.0, 10.0
(Missing)	1 (<0.1%)	4 (0.5%)
Social support		
Mean	7.2	7.1
Standard Deviation	2.8	2.9
Min, Max	0.0, 10.0	0.0, 10.0
(Missing)	5 (0.3%)	2 (0.3%)
Intimate/close friend, n (%)		
Yes	1,480 (82.9%)	747 (83.1%)
No	305 (17.1%)	151 (16.8%)
(Missing)	1 (0.1%)	1 (0.1%)
Government approval, n (%)		
Strongly approve	357 (20.0%)	229 (25.4%)
Somewhat approve	296 (16.6%)	133 (14.8%)
Neither approve nor disapprove	409 (22.9%)	179 (19.9%)
Somewhat disapprove	206 (11.5%)	92 (10.2%)
Strongly disapprove	495 (27.7%)	258 (28.7%)
(Missing)	23 (1.3%)	8 (0.9%)
Say in government, n (%)		
Agree	871 (48.8%)	462 (51.4%)
Disagree	552 (30.9%)	275 (30.5%)
Unsure	356 (19.9%)	159 (17.7%)
(Missing)	7 (0.4%)	3 (0.3%)
Belonging in country		
Mean	7.6	7.9
Standard Deviation	2.7	2.7
Min, Max	0.0, 10.0	0.0, 10.0
(Missing)	9 (0.5%)	7 (0.8%)
City/place satisfaction, n (%)		
Satisfied	978 (54.8%)	505 (56.2%)
Dissatisfied	599 (33.6%)	285 (31.7%)
Unsure	208 (11.7%)	107 (12.0%)
(Missing)	0 (0%)	2 (0.2%)
Trust within country, n (%)		
All people	60 (3.3%)	30 (3.4%)
Most people	134 (7.5%)	63 (7.0%)
Some people	466 (26.1%)	222 (24.7%)
Not very many people	606 (33.9%)	308 (34.3%)
None	486 (27.2%)	266 (29.6%)
(Missing)	34 (1.9%)	9 (1.0%)
Number of children		
Mean	1.8	2.0
Standard Deviation	1.9	1.9
Min, Max	0.0, 18.0	0.0, 13.0
(Missing)	1 (<0.1%)	2 (0.2%)

Table S25d. Unweighted summary statistics for Wave 1 outcome variables in South Africa by retention status.

**Outcome**	**Attritors–Not Observed in Wave 2** N = 1,786	**Retained–Observed in Wave 2** N = 899
Community participation, n (%)		
More than once a week	179 (10.0%)	97 (10.7%)
Once a week	287 (16.1%)	100 (11.1%)
One to three times a month	391 (21.9%)	228 (25.3%)
A few times a year	356 (20.0%)	182 (20.2%)
Never	570 (31.9%)	287 (32.0%)
(Missing)	3 (0.2%)	6 (0.7%)
Religious attendance, n (%)		
More than once a week	298 (16.7%)	132 (14.7%)
Once a week	582 (32.6%)	317 (35.3%)
One to three times a month	379 (21.2%)	196 (21.8%)
A few times a year	301 (16.8%)	132 (14.7%)
Never	221 (12.4%)	120 (13.3%)
(Missing)	5 (0.3%)	2 (0.2%)
Loneliness		
Mean	3.7	3.6
Standard Deviation	3.1	3.1
Min, Max	0.0, 10.0	0.0, 10.0
(Missing)	1 (<0.1%)	1 (0.1%)
Perceived discrimination, n (%)		
Always	294 (16.5%)	139 (15.5%)
Often	347 (19.4%)	203 (22.6%)
Rarely	338 (19.0%)	165 (18.3%)
Never	764 (42.8%)	384 (42.7%)
(Missing)	42 (2.4%)	8 (0.9%)
Orientation to promote good		
Mean	7.7	8.0
Standard Deviation	2.4	2.3
Min, Max	0.0, 10.0	0.0, 10.0
(Missing)	2 (0.1%)	0 (0%)
Delayed gratification		
Mean	7.6	7.9
Standard Deviation	2.5	2.4
Min, Max	0.0, 10.0	0.0, 10.0
(Missing)	9 (0.5%)	2 (0.2%)
Hope		
Mean	8.5	8.8
Standard Deviation	2.1	2.0
Min, Max	0.0, 10.0	0.0, 10.0
(Missing)	1 (<0.1%)	4 (0.4%)
Gratitude		
Mean	7.7	8.0
Standard Deviation	2.4	2.4
Min, Max	0.0, 10.0	0.0, 10.0
(Missing)	4 (0.2%)	4 (0.5%)
Showing love/care		
Mean	8.5	8.8
Standard Deviation	2.0	2.1
Min, Max	0.0, 10.0	0.0, 10.0

Table S25d. Unweighted summary statistics for Wave 1 outcome variables in South Africa by retention status.

Outcome	Attritors–Not Observed in Wave 2<br>N = 1,786	Retained–Observed in Wave 2<br>N = 899
(Missing)	2 (0.1%)	4 (0.4%)
Forgivingness, n (%)		
Always	1,064 (59.6%)	551 (61.3%)
Often	430 (24.1%)	211 (23.4%)
Rarely	182 (10.2%)	78 (8.7%)
Never	110 (6.1%)	59 (6.5%)
(Missing)	0 (0.0%)	0 (0%)
Charitable giving, n (%)		
Yes	345 (19.3%)	166 (18.5%)
No	1,440 (80.7%)	732 (81.4%)
(Missing)	0 (0%)	1 (0.1%)
Helping strangers, n (%)		
Yes	995 (55.7%)	543 (60.5%)
No	783 (43.9%)	351 (39.1%)
(Missing)	8 (0.4%)	4 (0.5%)
Volunteering, n (%)		
Yes	445 (24.9%)	212 (23.6%)
No	1,340 (75.1%)	686 (76.4%)
(Missing)	0 (0%)	0 (0%)
Self-rated physical health		
Mean	7.5	7.6
Standard Deviation	2.8	2.7
Min, Max	0.0, 10.0	0.0, 10.0
(Missing)	1 (<0.1%)	1 (0.2%)
Health problems, n (%)		
Yes	423 (23.7%)	161 (17.9%)
No	1,344 (75.3%)	725 (80.6%)
(Missing)	18 (1.0%)	13 (1.5%)
Pain in past 4 weeks, n (%)		
A lot	179 (10.0%)	114 (12.6%)
Some	322 (18.0%)	155 (17.2%)
Not very much	515 (28.9%)	247 (27.5%)
None at all	763 (42.7%)	381 (42.4%)
(Missing)	6 (0.3%)	3 (0.3%)
Number of cigarettes per day		
Mean	1.7	1.6
Standard Deviation	4.1	5.3
Min, Max	0.0, 40.0	0.0, 60.0
(Missing)	35 (2.0%)	12 (1.4%)
Number of drinks per week		
Mean	2.5	2.2
Standard Deviation	6.2	6.0
Min, Max	0.0, 97.0	0.0, 97.0
(Missing)	38 (2.1%)	14 (1.5%)
Days exercise per week		
Mean	2.3	2.5
Standard Deviation	2.4	2.5
Min, Max	0.0, 7.0	0.0, 7.0
(Missing)	21 (1.2%)	16 (1.7%)

Table S25d. Unweighted summary statistics for Wave 1 outcome variables in South Africa by retention status.

Outcome	Attritors–Not Observed in Wave 2<br>N = 1,786	Retained–Observed in Wave 2<br>N = 899
Financial security		
Mean	5.2	5.3
Standard Deviation	3.2	3.3
Min, Max	0.0, 10.0	0.0, 10.0
(Missing)	7 (0.4%)	3 (0.3%)
Material security		
Mean	5.4	5.8
Standard Deviation	3.3	3.5
Min, Max	0.0, 10.0	0.0, 10.0
(Missing)	4 (0.2%)	1 (0.1%)
Educational attainment (16+ years), n (%)		
Up to 8	470 (26.3%)	236 (26.3%)
9-15	1,211 (67.8%)	609 (67.7%)
16+	101 (5.7%)	54 (6.0%)
(Missing)	4 (0.2%)	0 (0%)
Currently employed, n (%)		
Employed for an employer	401 (22.5%)	156 (17.3%)
Self-employed	276 (15.5%)	130 (14.5%)
Retired	149 (8.3%)	88 (9.8%)
Student	143 (8.0%)	89 (9.9%)
Homemaker	93 (5.2%)	50 (5.5%)
Unemployed and looking for a job	673 (37.7%)	360 (40.0%)
None of these/Other	48 (2.7%)	25 (2.7%)
(Missing)	2 (0.1%)	2 (0.2%)
Financially comfortable/getting by, n (%)		
Living comfortably on present income	232 (13.0%)	108 (12.0%)
Getting by on present income	636 (35.6%)	315 (35.1%)
Finding it difficult on present income	548 (30.7%)	311 (34.6%)
Finding it very difficult on present income	322 (18.0%)	145 (16.1%)
(Missing)	48 (2.7%)	20 (2.2%)
Own home, n (%)		
Someone in this household owns this home	1,133 (63.5%)	587 (65.3%)
Someone in this household rents this home	276 (15.5%)	111 (12.4%)
Both	113 (6.3%)	54 (6.0%)
Neither	225 (12.6%)	137 (15.2%)
Rent	0 (0%)	0 (0%)
Own	0 (0%)	0 (0%)
Something else	0 (0%)	0 (0%)
(Missing)	38 (2.1%)	10 (1.1%)
Religious/spiritual connection, n (%)		
Always	1,113 (62.3%)	566 (62.9%)
Often	385 (21.6%)	195 (21.7%)
Rarely	166 (9.3%)	80 (8.9%)
Never	107 (6.0%)	52 (5.8%)
(Missing)	14 (0.8%)	6 (0.7%)
Belief in life after death, n (%)		
Yes	934 (52.3%)	473 (52.7%)
No	503 (28.2%)	265 (29.5%)
Unsure	338 (18.9%)	156 (17.3%)

Table S25d. Unweighted summary statistics for Wave 1 outcome variables in South Africa by retention status.

Outcome	Attritors–Not Observed in Wave 2<br>N = 1,786	Retained–Observed in Wave 2<br>N = 899
(Missing)	10 (0.6%)	4 (0.5%)
Transformative religious experience, n (%)		
Yes	1,001 (56.1%)	558 (62.1%)
No	738 (41.3%)	313 (34.8%)
(Missing)	46 (2.6%)	28 (3.1%)
Religious reading or listening, n (%)		
More than once a day	280 (15.7%)	167 (18.6%)
About once a day	344 (19.3%)	139 (15.5%)
Sometimes	919 (51.5%)	489 (54.4%)
Never	237 (13.3%)	98 (11.0%)
(Missing)	6 (0.4%)	4 (0.5%)
Prayer or meditation, n (%)		
More than once a day	760 (42.6%)	413 (46.0%)
About once a day	483 (27.1%)	251 (27.9%)
Sometimes	486 (27.2%)	210 (23.3%)
Never	53 (3.0%)	24 (2.6%)
(Missing)	3 (0.2%)	1 (0.1%)
Belief in God/gods/spiritual forces, n (%)		
One God	1,405 (78.7%)	749 (83.3%)
More than one god	189 (10.6%)	81 (9.0%)
An impersonal spiritual force	98 (5.5%)	39 (4.3%)
None of these	49 (2.7%)	12 (1.3%)
Unsure	42 (2.3%)	16 (1.8%)
(Missing)	3 (0.2%)	2 (0.3%)
Religious centrality, n (%)		
Agree	1,379 (77.2%)	719 (80.0%)
Disagree	186 (10.4%)	70 (7.8%)
Not relevant	92 (5.2%)	44 (4.9%)
Unsure	125 (7.0%)	64 (7.1%)
(Missing)	4 (0.2%)	2 (0.2%)
Religious/spiritual comfort, n (%)		
Agree	1,551 (86.9%)	791 (88.0%)
Disagree	104 (5.8%)	55 (6.2%)
Not relevant	77 (4.3%)	25 (2.7%)
Unsure	48 (2.7%)	25 (2.8%)
(Missing)	5 (0.3%)	3 (0.3%)
Feel loved by God, n (%)		
Agree	1,614 (90.4%)	837 (93.1%)
Disagree	57 (3.2%)	30 (3.4%)
Not relevant	68 (3.8%)	12 (1.3%)
Unsure	42 (2.3%)	17 (1.9%)
(Missing)	4 (0.3%)	3 (0.4%)
Feel punished by God, n (%)		
Agree	736 (41.2%)	366 (40.7%)
Disagree	771 (43.2%)	399 (44.4%)
Not relevant	159 (8.9%)	59 (6.6%)
Unsure	116 (6.5%)	69 (7.7%)
(Missing)	5 (0.3%)	5 (0.5%)
Experienced religious criticism, n (%)		

Table S25d. Unweighted summary statistics for Wave 1 outcome variables in South Africa by retention status.

Outcome	Attritors–Not Observed in Wave 2<br>N = 1,786	Retained–Observed in Wave 2<br>N = 899
Agree	728 (40.8%)	325 (36.1%)
Disagree	574 (32.1%)	339 (37.7%)
Not relevant	229 (12.8%)	104 (11.6%)
Unsure	248 (13.9%)	129 (14.3%)
(Missing)	7 (0.4%)	3 (0.3%)
Faith-sharing, n (%)		
Agree	1,274 (71.3%)	658 (73.2%)
Disagree	310 (17.3%)	160 (17.8%)
Not relevant	126 (7.1%)	48 (5.3%)
Unsure	66 (3.7%)	30 (3.4%)
(Missing)	10 (0.5%)	2 (0.3%)

Note. N (%); this table is based on non-imputed data. Cumulative percentages for variables may not add up to 100% due to rounding.

Table S25e. Summary of fitted attrition model in South Africa

Characteristic	Odds Ratio	95% CI	p-value
**ANNUAL_WEIGHT_R2**	0.85	0.74, 0.96	0.012
**Happiness & life satisfaction**	0.87	0.75, 1.01	0.059
**Physical & mental health**	0.96	0.84, 1.10	0.567
**Meaning & purpose**	1.06	0.91, 1.23	0.453
**Character & virtue**	1.19	1.02, 1.39	0.023
**Close social relationships**	1.05	0.92, 1.19	0.507
**Financial & material security**	1.13	1.00, 1.28	0.053
**Extraversion**	1.03	0.93, 1.15	0.570
**Openness to experience**	0.97	0.85, 1.09	0.576
**Agreeableness**	0.98	0.86, 1.10	0.700
**Conscientiousness**	1.02	0.91, 1.14	0.706
**Neuroticism**	1.07	0.95, 1.20	0.269
**Depression symptoms composite**	1.0	0.87, 1.13	0.934
**Anxiety symptoms composite**	0.90	0.80, 1.01	0.062
**Loneliness**	1.02	0.92, 1.14	0.652
**Days exercise per week**	1.12	1.00, 1.27	0.052
**Year of birth (age group)**			
1983-1993 (current age: 30-39 years)	—	—	
1973-1983 (current age: 40-49 years)	0.92	0.67, 1.26	0.595
1998-2005 (current age: 18-24 years)	1.00	0.72, 1.39	0.999
1993-1998 (current age: 25-29 years)	0.92	0.68, 1.24	0.577
1963-1973 (current age: 50-59 years)	0.81	0.52, 1.28	0.362
1953-1963 (current age: 60-69 years)	1.57	0.79, 3.14	0.197
1943-1953 (current age: 70-79 years)	0.77	0.28, 2.06	0.594
1943 or earlier (current age: 80+ years)	0.03	0.00, 0.27	0.002
**Gender of respondent**			
Female	—	—	
Male	0.95	0.75, 1.21	0.687
Other	0.00	0.00, 0.00	1.54e-43
**Marital status**			
Single/Never been married	—	—	
Married	1.23	0.87, 1.74	0.238
Domestic partner	0.95	0.66, 1.37	0.787
Widowed	1.49	0.82, 2.70	0.189
Separated	0.78	0.39, 1.53	0.462
Divorced	0.60	0.27, 1.35	0.217
**Employment status**			
Unemployed and looking for a job	—	—	
Employed for an employer	0.86	0.64, 1.16	0.316
Self-employed	0.95	0.67, 1.34	0.778
Student	1.27	0.82, 1.96	0.279
Retired	0.97	0.46, 2.04	0.929
Homemaker	1.12	0.64, 1.96	0.700
None of these/Other	0.87	0.40, 1.87	0.714
**Religious attendance**			
Once a week	—	—	
One to three times a month	0.98	0.73, 1.30	0.874
A few times a year	0.88	0.65, 1.19	0.410
More than once a week	0.82	0.58, 1.16	0.252
Never	1.12	0.79, 1.60	0.511
**Educational attainment (16+ years)**			
9-15	—	—	

Table S25e. Summary of fitted attrition model in South Africa

Characteristic	Odds Ratio	95% CI	p-value
Up to 8	1.23	0.81, 1.88	0.334
16+	1.08	0.76, 1.53	0.679
**Born in This country**			
Born in this country	—	—	
Born in another country	0.85	0.41, 1.74	0.650
**Race plurality (prominent race/ethnic group [0] or not [1])**	0.93	0.83, 1.03	0.164
**Urbanicity**			
A small town or village	—	—	
A rural area or on a farm	0.94	0.71, 1.26	0.689
A suburb of a large city	1.08	0.81, 1.44	0.588
A large city	1.00	0.68, 1.46	0.997
**Monthly household income**			
South Africa: 1,001 – 2,000 rand/month	—	—	
South Africa: 2,001 – 3,000 rand/month	0.88	0.61, 1.26	0.483
South Africa: 351 – 1,000 rand/month	1.18	0.79, 1.76	0.413
(None/No household income)	1.27	0.84, 1.90	0.254
South Africa: 201 – 350 rand/month	1.00	0.66, 1.53	0.985
South Africa: 3,001 – 4,000 rand/month	0.55	0.35, 0.85	0.007
South Africa: 4,001 – 5,000 rand/month	1.60	0.96, 2.69	0.074
South Africa: 5,001 – 6,000 rand/month	0.86	0.45, 1.63	0.632
South Africa: 6,001 – 8,000 rand/month	0.80	0.43, 1.47	0.466
South Africa: 10,001 – 15,000 rand/month	0.66	0.34, 1.29	0.224
South Africa: 8,001 – 10,000 rand/month	0.96	0.51, 1.83	0.910
South Africa: 15,001 – 20,000 rand/month	0.47	0.21, 1.09	0.080
South Africa: 200 rand/month or less	0.82	0.41, 1.63	0.574
South Africa: 20,001 – 30,000 rand/month	0.61	0.27, 1.39	0.238
South Africa: More than 30,000 rand/month	1.13	0.46, 2.78	0.791

Abbreviations: CI = Confidence Interval, OR = Odds Ratio

Notes. N=2651; attrition weights were estimated using the 'survey::svyglm(family=quasibinomial('logit'))' function. All continuous predictors were standardized and all categorical predictors used the most common category as the reference group. Reported p-values are based on the fitted regression model and no adjustments for multiple testing were done within this table.

Table S25f. Summary of principal components in South Africa

PC	Percent Explained by each PC	Cumulative Percent Explained
1	31.57	31.57
2	4.22	35.78
3	2.85	38.64
4	2.13	40.77
5	1.92	42.69
6	1.87	44.56
**7**	**1.80**	**46.35**
8	1.61	47.96
9	1.53	49.49
10	1.44	50.93
11	1.37	52.30
12	1.34	53.64
13	1.31	54.95
14	1.27	56.22
15	1.19	57.41
16	1.17	58.58
17	1.14	59.73
18	1.13	60.86
19	1.11	61.97
20	1.07	63.04

Notes. N=2651; PCA was conducted using 'survey::svyprcomp(.)' function using all available contemporaneous exposures at wave 1. All PCs were standardized prior to being used as predictors. The bolded row represented the number of retained components for analysis was 7.

Table S25g. Associations of forgivingness with adult well-being and other outcomes at Wave 2 in South Africa

Outcome	Model 1: Demographic and Childhood Variables as Covariates					Model 2: Demographic, Childhood, and Other Wave 1 Confounding Variables (Via Principal Components) as Covariates				
	RR	ES	95% CI	SE	p-value	RR	ES	95% CI	SE	p-value
Human Flourishing										
Secure flourishing index		0.02	(-0.04,0.09)	0.03	0.512		-0.02	(-0.08,0.05)	0.03	0.643
Flourishing index		0.03	(-0.03,0.09)	0.03	0.361		-0.01	(-0.07,0.05)	0.03	0.803
Happiness & life satisfaction		-0.04	(-0.11,0.02)	0.03	0.226		-0.06	(-0.12,0.01)	0.03	0.081
Physical & mental health		0.05	(-0.02,0.12)	0.04	0.191		0.03	(-0.04,0.10)	0.04	0.353
Meaning & purpose		0.03	(-0.05,0.10)	0.04	0.503		-0.00	(-0.07,0.07)	0.04	0.990
Character & virtue		0.00	(-0.07,0.08)	0.04	0.899		-0.02	(-0.09,0.06)	0.04	0.674
Close social relationships		0.06	(-0.02,0.13)	0.04	0.169		0.02	(-0.05,0.10)	0.04	0.537
Financial & material security		-0.01	(-0.08,0.07)	0.04	0.876		-0.02	(-0.09,0.04)	0.04	0.493
Psychological Well-Being										
Happiness (a)		-0.02	(-0.09,0.04)	0.03	0.471		-0.04	(-0.10,0.02)	0.03	0.249
Life satisfaction (a)		-0.04	(-0.12,0.04)	0.04	0.300		-0.06	(-0.13,0.02)	0.04	0.154
Current life evaluation		-0.03	(-0.09,0.04)	0.03	0.467		-0.04	(-0.11,0.03)	0.04	0.263
Future life evaluation		-0.01	(-0.08,0.05)	0.03	0.686		-0.03	(-0.10,0.04)	0.04	0.459
Optimism		0.01	(-0.07,0.09)	0.04	0.796		0.00	(-0.08,0.08)	0.04	0.977
Freedom to pursue what's important		0.00	(-0.08,0.08)	0.04	0.982		-0.03	(-0.10,0.05)	0.04	0.519
Inner peace	1.01		(0.98,1.05)	0.02	0.393	1.01		(0.98,1.04)	0.02	0.544
Life balance	1.02		(0.99,1.05)	0.02	0.276	1.02		(0.98,1.05)	0.02	0.360
Sense of mastery	1.01		(0.98,1.04)	0.01	0.595	1.01		(0.98,1.04)	0.01	0.586
Meaningful activities (c)		0.03	(-0.04,0.10)	0.04	0.425		0.01	(-0.06,0.08)	0.04	0.736
Understanding purpose (c)		0.01	(-0.08,0.10)	0.05	0.853		-0.01	(-0.11,0.08)	0.05	0.768
Self-rated mental health (b)		0.06	(-0.00,0.13)	0.03	0.066		0.05	(-0.02,0.12)	0.04	0.192
Psychological Distress										
Traumatic distress	0.99		(0.96,1.03)	0.02	0.740	0.99		(0.95,1.03)	0.02	0.619
Depression symptoms composite	1.02		(0.99,1.05)	0.02	0.259	1.02		(0.99,1.05)	0.02	0.183
Depression – feel hopeless	1.01		(0.97,1.04)	0.02	0.697	1.01		(0.98,1.04)	0.02	0.702
Depression – loss of interest	1.01		(0.98,1.04)	0.02	0.437	1.02		(0.99,1.05)	0.02	0.277
Anxiety symptoms composite	1.01		(0.97,1.04)	0.02	0.733	1.01		(0.98,1.05)	0.02	0.512
Anxiety – feel on edge	0.99		(0.96,1.03)	0.02	0.711	1.00		(0.96,1.04)	0.02	0.914
Anxiety – cannot stop worrying	1.01		(0.98,1.04)	0.02	0.555	1.01		(0.98,1.04)	0.01	0.370
Suffering	1.00		(0.97,1.04)	0.02	0.956	1.00		(0.97,1.04)	0.02	0.909
Social Well-Being										
Relationship contentment (e)		0.06	(-0.02,0.15)	0.04	0.160		0.05	(-0.04,0.13)	0.04	0.302
Relationship satisfaction (e)		0.02	(-0.06,0.10)	0.04	0.556		-0.01	(-0.08,0.07)	0.04	0.871
Social support		0.01	(-0.06,0.07)	0.03	0.784		0.00	(-0.06,0.07)	0.03	0.936
Intimate/close friend	1.01		(0.98,1.03)	0.01	0.540	1.00		(0.98,1.03)	0.01	0.704
Government approval	1.01		(0.98,1.05)	0.02	0.373	1.01		(0.98,1.04)	0.02	0.548
Say in government	1.01		(0.98,1.04)	0.02	0.403	1.01		(0.98,1.04)	0.02	0.740
Belonging in country		0.01	(-0.06,0.07)	0.03	0.862		-0.01	(-0.08,0.05)	0.03	0.676
City/place satisfaction	1.00		(0.97,1.04)	0.02	0.844	1.00		(0.96,1.04)	0.02	0.981
Trust within country	1.02		(0.98,1.05)	0.02	0.402	1.01		(0.97,1.05)	0.02	0.614
Social Participation										
Ever been married	1.00		(0.97,1.02)	0.01	0.762	1.00		(0.97,1.02)	0.01	0.743
Currently divorced	1.00		(0.99,1.01)	0.01	0.797	1.00		(0.99,1.01)	0.01	0.774
Number of children		-0.01	(-0.07,0.04)	0.03	0.610		-0.02	(-0.07,0.04)	0.03	0.528
Weekly+ community participation	1.01		(0.98,1.05)	0.02	0.395	1.01		(0.98,1.04)	0.02	0.500
Weekly+ religious attendance	1.01		(0.98,1.05)	0.02	0.458	1.01		(0.98,1.05)	0.02	0.569
Social Distress										
Loneliness		-0.04	(-0.11,0.02)	0.03	0.190		-0.03	(-0.09,0.04)	0.03	0.406
Perceived discrimination	1.00		(0.97,1.03)	0.02	0.960	0.99		(0.96,1.03)	0.02	0.732
Character & Prosocial Behavior										
Orientation to promote good (d)		-0.01	(-0.08,0.06)	0.03	0.788		-0.02	(-0.10,0.05)	0.04	0.541
Delayed gratification (d)		0.01	(-0.05,0.08)	0.03	0.687		-0.00	(-0.07,0.07)	0.03	0.934
Hope		0.06	(-0.03,0.15)	0.05	0.175		0.04	(-0.05,0.12)	0.05	0.428
Gratitude		0.04	(-0.04,0.12)	0.04	0.317		0.02	(-0.06,0.10)	0.04	0.608
Showing love/care		0.09	(0.01,0.18)	0.04	0.035*		0.08	(-0.00,0.16)	0.04	0.060
Forgivingness	1.06		(1.02,1.10)	0.02	1.45e-03**	1.05		(1.02,1.09)	0.02	4.12e-03**
Charitable giving	1.00		(0.96,1.04)	0.02	0.854	0.99		(0.96,1.03)	0.02	0.753
Helping strangers	1.01		(0.98,1.04)	0.02	0.531	1.01		(0.98,1.04)	0.02	0.562
Volunteering	1.01		(0.98,1.04)	0.02	0.532	1.00		(0.97,1.04)	0.02	0.808
Physical Health & Health Behavior										
Self-rated physical health (b)		0.01	(-0.06,0.08)	0.04	0.777		0.00	(-0.07,0.07)	0.04	0.916
Health problems	0.99		(0.96,1.02)	0.02	0.550	0.99		(0.95,1.02)	0.02	0.478
Pain in past 4 weeks	1.02		(0.98,1.05)	0.02	0.371	1.02		(0.99,1.06)	0.02	0.264
Daily smoker	0.99		(0.96,1.02)	0.01	0.602	1.00		(0.97,1.03)	0.02	0.986
Number of drinks per week		-0.03	(-0.10,0.04)	0.04	0.385		-0.00	(-0.08,0.07)	0.04	0.951
Days exercise per week		0.00	(-0.07,0.08)	0.04	0.896		-0.01	(-0.08,0.06)	0.04	0.769
Socioeconomic Outcomes										
Financial security (f)		0.01	(-0.06,0.08)	0.04	0.849		-0.01	(-0.08,0.06)	0.04	0.818
Material security (f)		-0.02	(-0.09,0.06)	0.04	0.699		-0.03	(-0.10,0.04)	0.04	0.437
Educational attainment (16+ years)	1.00		(0.98,1.02)	0.01	0.663	1.00		(0.98,1.02)	0.01	0.700
Currently employed	1.01		(0.97,1.04)	0.02	0.726	1.01		(0.97,1.04)	0.02	0.760
Financially comfortable/getting by	1.01		(0.98,1.05)	0.02	0.548	1.01		(0.98,1.05)	0.02	0.547
Own home	1.01		(0.98,1.05)	0.02	0.450	1.01		(0.98,1.05)	0.02	0.426
Income – top quintile	0.99		(0.96,1.01)	0.01	0.355	0.99		(0.97,1.02)	0.01	0.601
Religion & Spirituality										
Religious/spiritual connection	1.03		(0.99,1.06)	0.02	0.163	1.02		(0.98,1.06)	0.02	0.301
Belief in life after death	1.02		(0.99,1.05)	0.02	0.235	1.01		(0.98,1.05)	0.02	0.394
Transformative religious experience	1.03		(0.99,1.07)	0.02	0.199	1.01		(0.97,1.05)	0.02	0.543
Religious reading or listening	1.01		(0.98,1.03)	0.01	0.721	1.00		(0.97,1.03)	0.01	0.800
Prayer or meditation	1.03		(1.00,1.07)	0.02	0.084	1.03		(0.99,1.07)	0.02	0.130
Belief in God/gods/spiritual forces	1.00		(0.98,1.02)	0.01	0.805	1.00		(0.98,1.02)	0.01	0.861
Religious centrality	1.00		(0.97,1.03)	0.02	0.827	0.99		(0.96,1.02)	0.02	0.484
Religious/spiritual comfort	1.02		(0.99,1.06)	0.02	0.247	1.02		(0.98,1.05)	0.02	0.385
Feel loved by God	1.02		(0.99,1.05)	0.01	0.133	1.02		(0.99,1.04)	0.01	0.276
Feel punished by God	0.99		(0.96,1.02)	0.01	0.595	0.99		(0.96,1.02)	0.02	0.429
Experienced religious criticism	0.99		(0.96,1.03)	0.02	0.725	0.99		(0.96,1.02)	0.02	0.534
Faith-sharing	1.04		(1.00,1.08)	0.02	0.092	1.03		(0.99,1.07)	0.02	0.218

Notes. N=2651; Reference for focal predictor: never/rarely. RR, risk-ratio, null effect is 1.00; ES, effect size measure for standardized regression coefficient, null effect is 0.00; SE, standard error, the SE reported for binary/Likert-type outcomes where risk-ratios are on the log(RR) scale; CI, confidence interval; p-value, a Wald-type test of the null hypothesis that the effect of the focal predictor is zero; (a) item part of the Happiness & Life Satisfaction domain of the Secure Flourishing Index; (b) item part of the Physical & Mental Health domain of the Secure Flourishing Index; (c) item part of the Meaning & Purpose domain of the Secure Flourishing Index; (d) item part of the Character & Virtue domain of the Secure Flourishing Index; (e) item part of the Subjective Social Connectedness domain of the Secure Flourishing Index; (f) item part of the Financial & Material Security domain of the Secure Flourishing Index.

Multiple imputation was performed to impute missing data on the covariates, exposure, and outcomes. All models controlled for sociodemographic and childhood factors assessed at Wave 1. For Model 2 with PC (principal components), the first seven principal components of the entire set of contemporaneous confounders assessed at Wave 1 were included as additional covariates of the outcomes at Wave 2.

An outcome-wide analytic approach was used, and a separate model was run for each outcome. A different type of model was run depending on the nature of the outcome: (1) for each binary outcome, a weighted generalized linear model (with a log link and Poisson distribution) was used to estimate an RR; and (2) for each continuous outcome, a weighted linear regression model was used to estimate a ES. All effect sizes were standardized. For continuous outcomes, the ES represents the change in SD on the outcome between the lower and upper categories of the binary focal predictor. For binary outcomes, the RR represents the change in risk of being in the upper category compared to the lower category between the lower and upper categories of the binary focal predictor.

P-value significance thresholds: $p < 0.05$*, $p < 0.005$**, (Bonferroni) $p < 6.41e-04$***, correction for multiple testing using Bonferroni adjusted significant threshold.

Table S25h. Associations of forgivingness with adult well-being and other outcomes at Wave 2 in South Africa using complete-case analyses with attrition weights.

	Model 1: Demographic and Childhood Variables as Covariates					Model 2: Demographic, Childhood, and Other Wave 1 Confounding Variables (Via Principal Components) as Covariates				
Outcome	RR	ES	95% CI	SE	p-value	RR	ES	95% CI	SE	p-value
Human Flourishing										
Secure flourishing index		-0.01	(-0.10,0.07)	0.04	0.741		-0.03	(-0.12,0.05)	0.04	0.476
Flourishing index		-0.02	(-0.10,0.06)	0.04	0.668		-0.03	(-0.11,0.05)	0.04	0.485
Happiness & life satisfaction		-0.08	(-0.15,0.00)	0.04	0.055		-0.09	(-0.17,-0.01)	0.04	0.023*
Physical & mental health		0.03	(-0.04,0.11)	0.04	0.390		0.03	(-0.05,0.10)	0.04	0.487
Meaning & purpose		-0.00	(-0.08,0.07)	0.04	0.967		-0.01	(-0.09,0.07)	0.04	0.783
Character & virtue		-0.04	(-0.11,0.03)	0.04	0.276		-0.04	(-0.11,0.03)	0.04	0.286
Close social relationships		0.03	(-0.05,0.11)	0.04	0.434		0.02	(-0.06,0.10)	0.04	0.619
Financial & material security		0.00	(-0.08,0.09)	0.04	0.952		-0.02	(-0.10,0.06)	0.04	0.668
Psychological Well-Being										
Happiness (a)		-0.06	(-0.13,0.01)	0.04	0.106		-0.07	(-0.14,-0.00)	0.04	0.048*
Life satisfaction (a)		-0.07	(-0.15,0.01)	0.04	0.083		-0.09	(-0.17,-0.00)	0.04	0.042*
Current life evaluation		-0.02	(-0.10,0.06)	0.04	0.586		-0.04	(-0.12,0.04)	0.04	0.305
Future life evaluation		-0.03	(-0.11,0.04)	0.04	0.417		-0.04	(-0.12,0.04)	0.04	0.338
Optimism		0.02	(-0.07,0.10)	0.04	0.722		0.02	(-0.06,0.10)	0.04	0.647
Freedom to pursue what's important		-0.04	(-0.11,0.03)	0.03	0.225		-0.05	(-0.12,0.02)	0.04	0.137
Inner peace	1.00		(0.96,1.04)	0.02	0.921	1.00		(0.96,1.04)	0.02	0.927
Life balance	1.02		(0.98,1.06)	0.02	0.389	1.01		(0.97,1.05)	0.02	0.580
Sense of mastery	0.99		(0.96,1.03)	0.02	0.614	0.99		(0.96,1.03)	0.02	0.691
Meaningful activities (c)		0.01	(-0.05,0.08)	0.03	0.710		0.01	(-0.06,0.08)	0.04	0.867
Understanding purpose (c)		-0.02	(-0.10,0.07)	0.04	0.705		-0.02	(-0.11,0.06)	0.04	0.571
Self-rated mental health (b)		0.06	(-0.02,0.15)	0.04	0.149		0.06	(-0.02,0.14)	0.04	0.151
Psychological Distress										
Traumatic distress	1.00		(0.96,1.04)	0.02	0.973	1.00		(0.96,1.04)	0.02	0.947
Depression symptoms composite	1.04		(1.01,1.08)	0.02	0.013*	1.05		(1.01,1.08)	0.02	0.007*
Depression – feel hopeless	1.02		(0.99,1.06)	0.02	0.184	1.02		(0.99,1.06)	0.02	0.173
Depression – loss of interest	1.03		(1.00,1.06)	0.02	0.054	1.03		(1.00,1.07)	0.02	0.031*
Anxiety symptoms composite	1.00		(0.96,1.04)	0.02	0.985	1.01		(0.97,1.05)	0.02	0.696
Anxiety – feel on edge	0.98		(0.95,1.02)	0.02	0.372	0.99		(0.95,1.03)	0.02	0.568
Anxiety – cannot stop worrying	1.00		(0.96,1.03)	0.02	0.870	1.00		(0.97,1.04)	0.02	0.836
Suffering	1.02		(0.99,1.06)	0.02	0.239	1.03		(0.99,1.06)	0.02	0.112
Social Well-Being										
Relationship contentment (e)		0.06	(-0.02,0.15)	0.04	0.145		0.06	(-0.03,0.14)	0.04	0.200
Relationship satisfaction (e)		-0.01	(-0.08,0.07)	0.04	0.844		-0.02	(-0.10,0.06)	0.04	0.639
Social support		0.02	(-0.07,0.10)	0.04	0.705		-0.01	(-0.09,0.07)	0.04	0.862
Intimate/close friend	0.99		(0.97,1.01)	0.01	0.349	0.99		(0.97,1.01)	0.01	0.315
Government approval	1.02		(0.99,1.06)	0.02	0.234	1.01		(0.98,1.05)	0.02	0.478
Say in government	1.00		(0.96,1.05)	0.02	0.860	1.00		(0.96,1.05)	0.02	0.829
Belonging in country		0.02	(-0.06,0.10)	0.04	0.632		0.02	(-0.07,0.10)	0.04	0.716
City/place satisfaction	1.00		(0.96,1.04)	0.02	0.922	0.99		(0.95,1.04)	0.02	0.786
Trust within country	1.01		(0.96,1.06)	0.02	0.647	1.01		(0.96,1.06)	0.02	0.758
Social Participation										
Ever been married	1.00		(0.98,1.03)	0.01	0.930	1.00		(0.98,1.03)	0.01	0.901
Currently divorced	1.00		(0.99,1.01)	0.00	0.897	1.00		(0.99,1.01)	0.00	0.730
Number of children		0.02	(-0.03,0.07)	0.03	0.407		0.03	(-0.03,0.09)	0.03	0.269
Weekly+ community participation	1.02		(0.99,1.06)	0.02	0.123	1.02		(0.99,1.05)	0.02	0.178
Weekly+ religious attendance	1.02		(0.98,1.06)	0.02	0.358	1.01		(0.97,1.05)	0.02	0.636
Social Distress										
Loneliness		0.01	(-0.07,0.09)	0.04	0.753		0.02	(-0.06,0.10)	0.04	0.622
Perceived discrimination	0.99		(0.96,1.03)	0.02	0.752	1.00		(0.96,1.03)	0.02	0.821
Character & Prosocial Behavior										
Orientation to promote good (d)		-0.04	(-0.12,0.03)	0.04	0.249		-0.04	(-0.11,0.03)	0.04	0.301
Delayed gratification (d)		-0.02	(-0.10,0.05)	0.04	0.505		-0.03	(-0.10,0.04)	0.04	0.469
Hope		0.02	(-0.05,0.09)	0.04	0.513		0.03	(-0.05,0.10)	0.04	0.492
Gratitude		0.00	(-0.07,0.08)	0.04	0.964		-0.01	(-0.08,0.07)	0.04	0.835
Showing love/care		0.08	(-0.02,0.17)	0.05	0.125		0.08	(-0.02,0.18)	0.05	0.105
Forgivingness	1.06		(1.02,1.10)	0.02	4.21e-03**	1.06		(1.02,1.09)	0.02	4.54e-03**
Charitable giving	0.99		(0.95,1.03)	0.02	0.729	1.00		(0.96,1.03)	0.02	0.885
Helping strangers	1.02		(0.98,1.06)	0.02	0.400	1.02		(0.98,1.07)	0.02	0.290
Volunteering	1.01		(0.97,1.04)	0.02	0.694	1.01		(0.97,1.04)	0.02	0.759
Physical Health & Health Behavior										
Self-rated physical health (b)		-0.00	(-0.07,0.06)	0.03	0.903		-0.01	(-0.08,0.05)	0.03	0.700
Health problems	1.00		(0.97,1.04)	0.02	0.767	1.00		(0.97,1.03)	0.02	0.977
Pain in past 4 weeks	1.04		(1.00,1.08)	0.02	0.053	1.04		(1.01,1.08)	0.02	0.015*
Daily smoker	1.01		(0.98,1.05)	0.02	0.431	1.02		(0.99,1.05)	0.02	0.169
Number of drinks per week		-0.01	(-0.09,0.08)	0.04	0.903		0.00	(-0.08,0.08)	0.04	0.965
Days exercise per week		0.01	(-0.08,0.11)	0.05	0.758		0.01	(-0.08,0.10)	0.05	0.842
Socioeconomic Outcomes										
Financial security (f)		0.02	(-0.06,0.10)	0.04	0.624		0.01	(-0.07,0.09)	0.04	0.836
Material security (f)		-0.02	(-0.09,0.06)	0.04	0.699		-0.04	(-0.11,0.04)	0.04	0.327
Educational attainment (16+ years)	0.99		(0.97,1.01)	0.01	0.340	0.99		(0.97,1.01)	0.01	0.352
Currently employed	1.00		(0.97,1.03)	0.02	0.976	1.00		(0.97,1.04)	0.02	0.826
Financially comfortable/getting by	0.99		(0.95,1.03)	0.02	0.678	0.98		(0.94,1.03)	0.02	0.480
Own home	1.02		(0.98,1.06)	0.02	0.294	1.02		(0.98,1.06)	0.02	0.391
Income – top quintile	0.99		(0.95,1.03)	0.02	0.571	0.99		(0.96,1.03)	0.02	0.726
Religion & Spirituality										
Religious/spiritual connection	1.01		(0.97,1.04)	0.02	0.716	1.01		(0.97,1.04)	0.02	0.699
Belief in life after death	1.05		(1.01,1.09)	0.02	0.028*	1.05		(1.01,1.09)	0.02	0.020*
Transformative religious experience	1.04		(1.00,1.09)	0.02	0.040*	1.03		(1.00,1.07)	0.02	0.078
Religious reading or listening	1.03		(0.99,1.07)	0.02	0.153	1.02		(0.99,1.06)	0.02	0.200
Prayer or meditation	1.03		(0.99,1.06)	0.02	0.112	1.02		(0.99,1.06)	0.02	0.179
Belief in God/gods/spiritual forces	1.01		(0.99,1.03)	0.01	0.415	1.01		(0.99,1.03)	0.01	0.414
Religious centrality	0.99		(0.96,1.03)	0.02	0.628	0.99		(0.95,1.02)	0.02	0.422
Religious/spiritual comfort	1.04		(1.00,1.07)	0.02	0.044*	1.03		(1.00,1.07)	0.02	0.050*
Feel loved by God	1.01		(0.98,1.04)	0.01	0.398	1.01		(0.98,1.04)	0.01	0.448
Feel punished by God	0.98		(0.95,1.01)	0.02	0.249	0.97		(0.94,1.01)	0.02	0.105
Experienced religious criticism	0.99		(0.96,1.03)	0.02	0.765	0.99		(0.95,1.02)	0.02	0.520
Faith-sharing	1.08		(1.03,1.12)	0.02	4.29e-04***	1.07		(1.03,1.11)	0.02	7.44e-04**

Table S25h. Associations of forgivingness with adult well-being and other outcomes at Wave 2 in South Africa using complete-case analyses with attrition weights.

	Model 1: Demographic and Childhood Variables as Covariates					Model 2: Demographic, Childhood, and Other Wave 1 Confounding Variables (Via Principal Components) as Covariates				
Outcome	RR	ES	95% CI	SE	p-value	RR	ES	95% CI	SE	p-value

Notes. N=978; Reference for focal predictor: never/rarely. RR, risk-ratio, null effect is 1.00; ES, effect size measure for standardized regression coefficient, null effect is 0.00; SE, standard error, the SE reported for binary/Likert-type outcomes where risk-ratios are on the log(RR) scale; CI, confidence interval; p-value, a Wald-type test of the null hypothesis that the effect of the focal predictor is zero; (a) item part of the Happiness & Life Satisfaction domain of the Secure Flourishing Index; (b) item part of the Physical & Mental Health domain of the Secure Flourishing Index; (c) item part of the Meaning & Purpose domain of the Secure Flourishing Index; (d) item part of the Character & Virtue domain of the Secure Flourishing Index; (e) item part of the Subjective Social Connectedness domain of the Secure Flourishing Index; (f) item part of the Financial & Material Security domain of the Secure Flourishing Index.

Attrition weights were computed to adjust the complete case data (those who responded at Wave 2 to at least 50% of the questions) and multiple imputation was used to impute missing data on all remaining within wave on the covariates, exposure, and outcomes. All models controlled for sociodemographic and childhood factors assessed at Wave 1. For Model 2 with PC (principal components), the first seven principal components of the entire set of contemporaneous confounders assessed at Wave 1 were included as additional covariates of the outcomes at Wave 2.

An outcome-wide analytic approach was used, and a separate model was run for each outcome. A different type of model was run depending on the nature of the outcome: (1) for each binary outcome, a weighted generalized linear model (with a log link and Poisson distribution) was used to estimate an RR; and (2) for each continuous outcome, a weighted linear regression model was used to estimate a ES. All effect sizes were standardized. For continuous outcomes, the ES represents the change in SD on the outcome between the lower and upper categories of the binary focal predictor. For binary outcomes, the RR represents the change in risk of being in the upper category compared to the lower category between the lower and upper categories of the binary focal predictor.

P-value significance thresholds: p < 0.05*, p < 0.005**, (Bonferroni) p < 6.41e-04***, correction for multiple testing using Bonferroni adjusted significant threshold.

Table S25i. Sensitivity analysis of forgivingness outcome-wide results to unmeasured confounding using E-values in South Africa

	Multiple Imputation				Complete Case w/ Attrition Weights			
	Model 1: Demographics and Childhood Variables as Covariates		Model 2: Demographics, Childhood, and Other Wave 1 Confounders (Via Principal Components) as Covariates		Model 1: Demographics and Childhood Variables as Covariates		Model 2: Demographics, Childhood, and Other Wave 1 Confounders (Via Principal Components) as Covariates	
Outcome	EE	ECI	EE	ECI	EE	ECI	EE	ECI
Human Flourishing								
Secure flourishing index	1.31	1.00	1.24	1.00	1.23	1.00	1.37	1.00
Flourishing index	1.36	1.00	1.16	1.00	1.26	1.00	1.36	1.00
Happiness & life satisfaction	1.45	1.00	1.58	1.00	1.71	1.00	1.81	1.21
Physical & mental health	1.50	1.00	1.39	1.00	1.40	1.00	1.34	1.00
Meaning & purpose	1.33	1.00	1.04	1.00	1.07	1.00	1.20	1.00
Character & virtue	1.12	1.00	1.25	1.00	1.45	1.00	1.44	1.00
Close social relationships	1.56	1.00	1.31	1.00	1.37	1.00	1.28	1.00
Financial & material security	1.13	1.00	1.32	1.00	1.09	1.00	1.26	1.00
Psychological Well-Being								
Happiness	1.31	1.00	1.41	1.00	1.59	1.00	1.67	1.05
Life satisfaction	1.45	1.00	1.56	1.00	1.68	1.00	1.77	1.10
Current life evaluation	1.33	1.00	1.44	1.00	1.29	1.00	1.47	1.00
Future life evaluation	1.22	1.00	1.34	1.00	1.38	1.00	1.43	1.00
Optimism	1.19	1.00	1.06	1.00	1.24	1.00	1.27	1.00
Freedom to pursue what's important	1.05	1.00	1.33	1.00	1.45	1.00	1.54	1.00
Inner peace	1.24	1.00	1.20	1.00	1.08	1.00	1.08	1.00
Life balance	1.29	1.00	1.26	1.00	1.29	1.00	1.22	1.00
Sense of mastery	1.17	1.00	1.17	1.00	1.19	1.00	1.16	1.00
Meaningful activities	1.36	1.00	1.21	1.00	1.21	1.00	1.14	1.00
Understanding purpose	1.17	1.00	1.23	1.00	1.25	1.00	1.32	1.00
Self-rated mental health	1.63	1.00	1.51	1.00	1.61	1.00	1.59	1.00
Psychological Distress								
Traumatic distress	1.15	1.00	1.19	1.00	1.04	1.00	1.06	1.00
Depression symptoms composite	1.28	1.00	1.30	1.00	1.50	1.18	1.52	1.22
Depression – feel hopeless	1.15	1.00	1.15	1.00	1.32	1.00	1.32	1.00
Depression – loss of interest	1.22	1.00	1.27	1.00	1.40	1.00	1.43	1.10
Anxiety symptoms composite	1.14	1.00	1.21	1.00	1.03	1.00	1.17	1.00
Anxiety – feel on edge	1.16	1.00	1.08	1.00	1.27	1.00	1.21	1.00
Anxiety – cannot stop worrying	1.19	1.00	1.23	1.00	1.10	1.00	1.11	1.00
Suffering	1.05	1.00	1.08	1.00	1.32	1.00	1.37	1.00
Social Well-Being								
Relationship contentment	1.62	1.00	1.49	1.00	1.62	1.00	1.57	1.00
Relationship satisfaction	1.32	1.00	1.15	1.00	1.16	1.00	1.27	1.00
Social support	1.18	1.00	1.09	1.00	1.24	1.00	1.15	1.00
Intimate/close friend	1.17	1.00	1.13	1.00	1.20	1.00	1.21	1.00
Government approval	1.25	1.00	1.19	1.00	1.31	1.00	1.23	1.00
Say in government	1.23	1.00	1.13	1.00	1.12	1.00	1.13	1.00
Belonging in country	1.14	1.00	1.22	1.00	1.28	1.00	1.25	1.00
City/place satisfaction	1.12	1.00	1.04	1.00	1.08	1.00	1.15	1.00
Trust within country	1.26	1.00	1.19	1.00	1.21	1.00	1.17	1.00
Social Participation								
Ever been married	1.11	1.00	1.12	1.00	1.06	1.00	1.07	1.00
Currently divorced	1.06	1.00	1.07	1.00	1.04	1.00	1.07	1.00
Number of children	1.23	1.00	1.27	1.00	1.30	1.00	1.40	1.00
Weekly+ community participation	1.24	1.00	1.20	1.00	1.34	1.00	1.31	1.00
Weekly+ religious attendance	1.23	1.00	1.20	1.00	1.29	1.00	1.19	1.00
Social Distress								
Loneliness	1.48	1.00	1.35	1.00	1.21	1.00	1.28	1.00
Perceived discrimination	1.05	1.00	1.14	1.00	1.15	1.00	1.12	1.00
Character & Prosocial Behavior								
Orientation to promote good	1.18	1.00	1.31	1.00	1.46	1.00	1.43	1.00
Delayed gratification	1.23	1.00	1.09	1.00	1.32	1.00	1.34	1.00
Hope	1.62	1.00	1.41	1.00	1.31	1.00	1.33	1.00
Gratitude	1.45	1.00	1.29	1.00	1.07	1.00	1.16	1.00
Showing love/care	1.83	1.18	1.75	1.00	1.71	1.00	1.74	1.00
Forgivingness	1.63	1.33	1.57	1.27	1.60	1.27	1.59	1.27
Charitable giving	1.11	1.00	1.15	1.00	1.16	1.00	1.09	1.00
Helping strangers	1.19	1.00	1.18	1.00	1.28	1.00	1.32	1.00
Volunteering	1.20	1.00	1.12	1.00	1.16	1.00	1.14	1.00
Physical Health & Health Behavior								
Self-rated physical health	1.19	1.00	1.11	1.00	1.11	1.00	1.22	1.00
Health problems	1.20	1.00	1.23	1.00	1.13	1.00	1.04	1.00
Pain in past 4 weeks	1.27	1.00	1.30	1.00	1.45	1.00	1.50	1.18
Daily smoker	1.17	1.00	1.03	1.00	1.23	1.00	1.31	1.00
Number of drinks per week	1.38	1.00	1.08	1.00	1.13	1.00	1.07	1.00
Days exercise per week	1.12	1.00	1.20	1.00	1.23	1.00	1.18	1.00
Socioeconomic Outcomes								
Financial security	1.15	1.00	1.17	1.00	1.29	1.00	1.17	1.00
Material security	1.24	1.00	1.36	1.00	1.24	1.00	1.42	1.00
Educational attainment (16+ years)	1.12	1.00	1.12	1.00	1.18	1.00	1.18	1.00
Currently employed	1.15	1.00	1.14	1.00	1.04	1.00	1.11	1.00
Financially comfortable/getting by	1.20	1.00	1.20	1.00	1.18	1.00	1.26	1.00
Own home	1.23	1.00	1.23	1.00	1.30	1.00	1.27	1.00
Income – top quintile	1.23	1.00	1.16	1.00	1.21	1.00	1.16	1.00
Religion & Spirituality								
Religious/spiritual connection	1.35	1.00	1.29	1.00	1.15	1.00	1.15	1.00
Belief in life after death	1.30	1.00	1.24	1.00	1.54	1.13	1.54	1.17
Transformative religious experience	1.36	1.00	1.23	1.00	1.49	1.08	1.42	1.00
Religious reading or listening	1.14	1.00	1.11	1.00	1.37	1.00	1.33	1.00
Prayer or meditation	1.42	1.00	1.37	1.00	1.36	1.00	1.32	1.00
Belief in God/gods/spiritual forces	1.08	1.00	1.07	1.00	1.18	1.00	1.18	1.00
Religious centrality	1.11	1.00	1.20	1.00	1.18	1.00	1.24	1.00
Religious/spiritual comfort	1.31	1.00	1.25	1.00	1.43	1.05	1.42	1.01
Feel loved by God	1.30	1.00	1.25	1.00	1.22	1.00	1.20	1.00
Feel punished by God	1.17	1.00	1.22	1.00	1.28	1.00	1.36	1.00
Experienced religious criticism	1.14	1.00	1.20	1.00	1.14	1.00	1.22	1.00
Faith-sharing	1.43	1.00	1.35	1.00	1.74	1.41	1.70	1.38

Notes. EE, E-value for estimate; ECI, E-value for the limit of the confidence interval. The formula for calculating E-values can be found in VanderWeele and Ding (2017). E-values for estimate are the minimum strength of association on the risk ratio scale that an unmeasured confounder would need to have with both the exposure and the outcome to fully explain away the observed association between the exposure and outcome, conditional on the measured covariates. E-values for the 95% CI closest to the null denote the minimum strength of association on the risk ratio scale that an unmeasured confounder would need to have with both the exposure and the outcome to shift the CI to include the null value, conditional on the measured covariates.

Table S26a. Weighted summary statistics for demographic and childhood variables in Spain

**Characteristic**	**Wave 1** N = 6,290	**Wave 2** N = 2,904
Forgivingness, n (%)		
Always	1,414 (22.5%)	648 (22.3%)
Often	3,471 (55.2%)	1,618 (55.7%)
Rarely	1,219 (19.4%)	566 (19.5%)
Never	179 (2.8%)	70 (2.4%)
(Missing)	7 (0.1%)	2 (0.1%)
Year of birth, n (%)		
1943 or earlier (current age: 80+ years)	40 (0.6%)	21 (0.7%)
1943-1953 (current age: 70-79 years)	492 (7.8%)	268 (9.2%)
1953-1963 (current age: 60-69 years)	950 (15.1%)	448 (15.4%)
1963-1973 (current age: 50-59 years)	1,196 (19.0%)	598 (20.6%)
1973-1983 (current age: 40-49 years)	1,373 (21.8%)	625 (21.5%)
1983-1993 (current age: 30-39 years)	1,127 (17.9%)	510 (17.5%)
1993-1998 (current age: 25-29 years)	472 (7.5%)	216 (7.4%)
1998-2005 (current age: 18-24 years)	639 (10.2%)	218 (7.5%)
(Missing)	0 (0%)	0 (0%)
Age of participant		
Mean	46.4	47.9
Standard Deviation	15.9	15.6
Min, Max	18.0, 88.0	19.0, 88.0
Gender, n (%)		
Male	3,133 (49.8%)	1,444 (49.7%)
Female	3,128 (49.7%)	1,450 (49.9%)
Other	6 (0.1%)	1 (0.0%)
(Missing)	23 (0.4%)	9 (0.3%)
Respondent marital status, n (%)		
Single/Never been married	1,782 (28.3%)	796 (27.4%)
Married	2,930 (46.6%)	1,400 (48.2%)
Separated	236 (3.8%)	88 (3.0%)
Divorced	509 (8.1%)	252 (8.7%)
Widowed	188 (3.0%)	74 (2.6%)
Domestic partner	586 (9.3%)	257 (8.9%)
(Missing)	58 (0.9%)	36 (1.2%)
Education (years), n (%)		
Up to 8	918 (14.6%)	305 (10.5%)
9-15	4,085 (64.9%)	1,911 (65.8%)
16+	1,284 (20.4%)	688 (23.7%)
(Missing)	3 (0.1%)	0 (0%)
Employment status, n (%)		
Employed for an employer	2,844 (45.2%)	1,339 (46.1%)
Self-employed	577 (9.2%)	232 (8.0%)
Retired	1,268 (20.2%)	607 (20.9%)
Student	466 (7.4%)	170 (5.9%)
Homemaker	362 (5.7%)	130 (4.5%)
Unemployed and looking for a job	644 (10.2%)	299 (10.3%)
None of these/Other	120 (1.9%)	81 (2.8%)
(Missing)	9 (0.1%)	48 (1.6%)
Current religious service attendance, n (%)		
More than once a week	323 (5.1%)	150 (5.2%)
Once a week	672 (10.7%)	241 (8.3%)

Table S26a. Weighted summary statistics for demographic and childhood variables in Spain

**Characteristic**	**Wave 1**<br>N = 6,290	**Wave 2**<br>N = 2,904
One to three times a month	438 (7.0%)	184 (6.3%)
A few times a year	1,968 (31.3%)	1,102 (37.9%)
Never	2,863 (45.5%)	1,215 (41.8%)
(Missing)	27 (0.4%)	11 (0.4%)
Immigration status, n (%)		
Born in this country	5,459 (86.8%)	2,540 (87.5%)
Born in another country	811 (12.9%)	356 (12.3%)
(Missing)	20 (0.3%)	8 (0.3%)
Parental marital status around age 12, n (%)		
Parents were married	5,268 (83.7%)	2,524 (86.9%)
Parents were divorced	391 (6.2%)	152 (5.2%)
Parents were never married	321 (5.1%)	119 (4.1%)
One or both of them had died	130 (2.1%)	56 (1.9%)
Unsure	74 (1.2%)	20 (0.7%)
(Missing)	106 (1.7%)	34 (1.2%)
Religious service attendance around age 12, n (%)		
At least once a week	2,370 (37.7%)	1,132 (39.0%)
One to three times a month	1,132 (18.0%)	519 (17.9%)
Less than once a month	1,302 (20.7%)	568 (19.6%)
Never	1,448 (23.0%)	667 (23.0%)
(Missing)	38 (0.6%)	17 (0.6%)
Relationship with mother when growing up, n (%)		
Very good	4,547 (72.3%)	2,074 (71.4%)
Somewhat good	1,264 (20.1%)	586 (20.2%)
Somewhat bad	245 (3.9%)	114 (3.9%)
Very bad	96 (1.5%)	51 (1.7%)
(Does not apply)	109 (1.7%)	67 (2.3%)
(Missing)	28 (0.4%)	13 (0.4%)
Relationship with father when growing up, n (%)		
Very good	4,114 (65.4%)	1,906 (65.6%)
Somewhat good	1,402 (22.3%)	648 (22.3%)
Somewhat bad	315 (5.0%)	136 (4.7%)
Very bad	179 (2.8%)	90 (3.1%)
(Does not apply)	249 (4.0%)	110 (3.8%)
(Missing)	31 (0.5%)	14 (0.5%)
Felt like an outsider in family when growing up, n (%)		
Yes	601 (9.6%)	282 (9.7%)
No	5,617 (89.3%)	2,586 (89.0%)
(Missing)	72 (1.1%)	36 (1.3%)
Experienced abuse when growing up, n (%)		
Yes	663 (10.5%)	308 (10.6%)
No	5,517 (87.7%)	2,563 (88.3%)
(Missing)	110 (1.8%)	33 (1.2%)
Self-rated health when growing up, n (%)		
Excellent	2,440 (38.8%)	1,080 (37.2%)
Very good	2,312 (36.8%)	1,105 (38.1%)
Good	1,224 (19.5%)	560 (19.3%)
Fair	164 (2.6%)	77 (2.6%)
Poor	132 (2.1%)	71 (2.4%)
(Missing)	18 (0.3%)	12 (0.4%)

Table S26a. Weighted summary statistics for demographic and childhood variables in Spain

**Characteristic**	**Wave 1**<br>N = 6,290	**Wave 2**<br>N = 2,904
Subjective financial status of family growing up, n (%)		
Lived comfortably	2,048 (32.6%)	920 (31.7%)
Got by	2,938 (46.7%)	1,327 (45.7%)
Found it difficult	1,167 (18.6%)	577 (19.9%)
Found it very difficult	110 (1.7%)	61 (2.1%)
(Missing)	26 (0.4%)	18 (0.6%)
Religious affiliation growing up, n (%)		
Christianity	5,101 (81.1%)	2,369 (81.6%)
Taoism	0 (0%)	0 (0%)
Confucianism	1 (0.0%)	1 (0.0%)
Primal, Animist, or Folk religion	4 (0.1%)	0 (0.0%)
Spiritism	0 (0%)	0 (0%)
Umbanda, Candomblé, and other African-derived religions	0 (0%)	0 (0%)
Chinese folk/traditional religion	0 (0%)	0 (0%)
Islam	134 (2.1%)	44 (1.5%)
Hinduism	5 (0.1%)	2 (0.1%)
Buddhism	8 (0.1%)	5 (0.2%)
Judaism	5 (0.1%)	1 (0.0%)
Sikhism	2 (0.0%)	1 (0.0%)
Baha'i	0 (0%)	0 (0%)
Jainism	0 (0%)	0 (0%)
Shinto	0 (0%)	0 (0%)
Some other religion	13 (0.2%)	4 (0.2%)
No religion/Atheist/Agnostic	989 (15.7%)	462 (15.9%)
(Missing)	27 (0.4%)	14 (0.5%)

Note. N (%); this table is based on non-imputed data. Cumulative percentages for variables may not add up to 100% due to rounding. Wave 1 characteristics weighted using the Gallup provided sampling weight, ANNUAL_WEIGHT_R2; Wave 2 characteristics weighted accounting for attrition by using the adjusted Wave 1 weight, ANNUAL_WEIGHT_R2, multiplied by the created attrition weight to account for dropout, to maintain nationally representative estimates for Wave 2 characteristics.

Table S26b. Weighted summary statistics for outcome variables in Spain

Outcome	Wave 1<br>N = 6,290	Wave 2<br>N = 2,904
Secure flourishing index		
Mean	6.9	6.8
Standard Deviation	1.4	1.4
Min, Max	0.0, 10.0	0.8, 10.0
(Missing)	146 (2.3%)	50 (1.7%)
Flourishing index		
Mean	7.3	7.2
Standard Deviation	1.4	1.5
Min, Max	0.0, 10.0	0.0, 10.0
(Missing)	137 (2.2%)	43 (1.5%)
Happiness & life satisfaction		
Mean	6.9	6.7
Standard Deviation	2.0	2.0
Min, Max	0.0, 10.0	0.0, 10.0
(Missing)	27 (0.4%)	7 (0.2%)
Physical & mental health		
Mean	7.1	6.9
Standard Deviation	1.9	1.9
Min, Max	0.0, 10.0	0.0, 10.0
(Missing)	28 (0.4%)	11 (0.4%)
Meaning & purpose		
Mean	7.3	7.2
Standard Deviation	1.9	2.0
Min, Max	0.0, 10.0	0.0, 10.0
(Missing)	41 (0.7%)	13 (0.4%)
Character & virtue		
Mean	7.8	7.7
Standard Deviation	1.5	1.6
Min, Max	0.0, 10.0	0.0, 10.0
(Missing)	22 (0.4%)	7 (0.2%)
Close social relationships		
Mean	7.4	7.2
Standard Deviation	2.0	2.1
Min, Max	0.0, 10.0	0.0, 10.0
(Missing)	38 (0.6%)	8 (0.3%)
Financial & material security		
Mean	4.8	5.1
Standard Deviation	3.0	3.0
Min, Max	0.0, 10.0	0.0, 10.0
(Missing)	13 (0.2%)	8 (0.3%)
Happiness		
Mean	6.9	6.8
Standard Deviation	2.0	2.0
Min, Max	0.0, 10.0	0.0, 10.0
(Missing)	16 (0.2%)	3 (0.1%)
Life satisfaction		
Mean	6.8	6.7
Standard Deviation	2.2	2.2
Min, Max	0.0, 10.0	0.0, 10.0
(Missing)	15 (0.2%)	4 (0.1%)
Current life evaluation		

Table S26b. Weighted summary statistics for outcome variables in Spain

**Outcome**	**Wave 1**<br>N = 6,290	**Wave 2**<br>N = 2,904
Mean	6.7	6.5
Standard Deviation	1.9	1.9
Min, Max	0.0, 10.0	0.0, 10.0
(Missing)	7 (0.1%)	7 (0.2%)
Future life evaluation		
Mean	7.6	7.4
Standard Deviation	1.9	2.0
Min, Max	0.0, 10.0	0.0, 10.0
(Missing)	16 (0.2%)	20 (0.7%)
Optimism		
Mean	8.3	8.1
Standard Deviation	2.0	2.0
Min, Max	0.0, 10.0	0.0, 10.0
(Missing)	16 (0.3%)	6 (0.2%)
Freedom to pursue what's important		
Mean	7.4	7.4
Standard Deviation	2.3	2.3
Min, Max	0.0, 10.0	0.0, 10.0
(Missing)	3 (<0.1%)	4 (0.1%)
Inner peace, n (%)		
Always	777 (12.3%)	457 (15.7%)
Often	2,710 (43.1%)	1,201 (41.3%)
Rarely	2,352 (37.4%)	1,062 (36.6%)
Never	428 (6.8%)	167 (5.7%)
(Missing)	24 (0.4%)	18 (0.6%)
Life balance, n (%)		
Always	985 (15.7%)	495 (17.0%)
Often	3,829 (60.9%)	1,731 (59.6%)
Rarely	1,337 (21.3%)	614 (21.2%)
Never	130 (2.1%)	63 (2.2%)
(Missing)	8 (0.1%)	2 (0.1%)
Sense of mastery, n (%)		
Always	1,530 (24.3%)	734 (25.3%)
Often	3,828 (60.9%)	1,744 (60.0%)
Rarely	815 (12.9%)	344 (11.8%)
Never	82 (1.3%)	55 (1.9%)
(Missing)	36 (0.6%)	28 (1.0%)
Meaningful activities		
Mean	7.3	7.2
Standard Deviation	2.2	2.2
Min, Max	0.0, 10.0	0.0, 10.0
(Missing)	14 (0.2%)	5 (0.2%)
Understanding purpose		
Mean	7.3	7.2
Standard Deviation	2.2	2.3
Min, Max	0.0, 10.0	0.0, 10.0
(Missing)	27 (0.4%)	8 (0.3%)
Self-rated mental health		
Mean	7.4	7.2
Standard Deviation	2.2	2.3
Min, Max	0.0, 10.0	0.0, 10.0

Table S26b. Weighted summary statistics for outcome variables in Spain

Outcome	Wave 1<br>N = 6,290	Wave 2<br>N = 2,904
(Missing)	9 (0.1%)	1 (<0.1%)
Traumatic distress, n (%)		
A lot	724 (11.5%)	338 (11.6%)
Some	1,618 (25.7%)	753 (25.9%)
Not very much	1,791 (28.5%)	836 (28.8%)
Not at all	2,149 (34.2%)	973 (33.5%)
(Missing)	9 (0.2%)	4 (0.2%)
Depression symptoms composite, n (%)	1,856 (29.7%)	895 (30.9%)
(Missing)	39 (0.6%)	7 (0.3%)
Depression – feel hopeless, n (%)		
Nearly every day	576 (9.2%)	300 (10.3%)
More than half the days	844 (13.4%)	405 (13.9%)
Several days	2,211 (35.2%)	1,016 (35.0%)
Not at all	2,639 (42.0%)	1,181 (40.7%)
(Missing)	20 (0.3%)	3 (0.1%)
Depression – loss of interest, n (%)		
Nearly every day	522 (8.3%)	276 (9.5%)
More than half the days	1,131 (18.0%)	507 (17.5%)
Several days	2,411 (38.3%)	1,162 (40.0%)
Not at all	2,202 (35.0%)	953 (32.8%)
(Missing)	23 (0.4%)	5 (0.2%)
Anxiety symptoms composite, n (%)	2,042 (32.6%)	1,002 (34.7%)
(Missing)	32 (0.5%)	15 (0.5%)
Anxiety – feel on edge, n (%)		
Nearly every day	797 (12.7%)	438 (15.1%)
More than half the days	982 (15.6%)	469 (16.1%)
Several days	2,783 (44.2%)	1,266 (43.6%)
Not at all	1,704 (27.1%)	719 (24.8%)
(Missing)	25 (0.4%)	12 (0.4%)
Anxiety – cannot stop worrying, n (%)		
Nearly every day	695 (11.0%)	329 (11.3%)
More than half the days	872 (13.9%)	458 (15.8%)
Several days	2,045 (32.5%)	938 (32.3%)
Not at all	2,667 (42.4%)	1,176 (40.5%)
(Missing)	11 (0.2%)	4 (0.1%)
Suffering, n (%)		
A lot	737 (11.7%)	432 (14.9%)
Some	2,301 (36.6%)	1,179 (40.6%)
Not very much	2,146 (34.1%)	970 (33.4%)
Not at all	1,073 (17.1%)	304 (10.5%)
(Missing)	32 (0.5%)	19 (0.7%)
Relationship contentment		
Mean	7.7	7.5
Standard Deviation	2.0	2.2
Min, Max	0.0, 10.0	0.0, 10.0
(Missing)	19 (0.3%)	6 (0.2%)
Relationship satisfaction		
Mean	7.1	6.9
Standard Deviation	2.3	2.4
Min, Max	0.0, 10.0	0.0, 10.0
(Missing)	24 (0.4%)	5 (0.2%)

Table S26b. Weighted summary statistics for outcome variables in Spain

**Outcome**	**Wave 1**<br>N = 6,290	**Wave 2**<br>N = 2,904
Social support		
Mean	8.1	8.0
Standard Deviation	2.2	2.2
Min, Max	0.0, 10.0	0.0, 10.0
(Missing)	35 (0.6%)	16 (0.6%)
Intimate/close friend, n (%)		
Yes	5,454 (86.7%)	2,505 (86.3%)
No	810 (12.9%)	385 (13.3%)
(Missing)	26 (0.4%)	14 (0.5%)
Government approval, n (%)		
Strongly approve	419 (6.7%)	223 (7.7%)
Somewhat approve	1,797 (28.6%)	798 (27.5%)
Neither approve nor disapprove	1,430 (22.7%)	579 (19.9%)
Somewhat disapprove	1,478 (23.5%)	679 (23.4%)
Strongly disapprove	1,134 (18.0%)	616 (21.2%)
(Missing)	32 (0.5%)	10 (0.3%)
Say in government, n (%)		
Agree	897 (14.3%)	479 (16.5%)
Disagree	3,805 (60.5%)	1,785 (61.5%)
Unsure	1,565 (24.9%)	638 (22.0%)
(Missing)	23 (0.4%)	3 (0.1%)
Belonging in country		
Mean	7.5	7.4
Standard Deviation	2.5	2.5
Min, Max	0.0, 10.0	0.0, 10.0
(Missing)	50 (0.8%)	17 (0.6%)
City/place satisfaction, n (%)		
Satisfied	5,049 (80.3%)	2,289 (78.8%)
Dissatisfied	767 (12.2%)	380 (13.1%)
Unsure	418 (6.6%)	199 (6.8%)
(Missing)	56 (0.9%)	37 (1.3%)
Trust within country, n (%)		
All people	104 (1.6%)	64 (2.2%)
Most people	987 (15.7%)	489 (16.8%)
Some people	2,736 (43.5%)	1,290 (44.4%)
Not very many people	2,199 (35.0%)	964 (33.2%)
None	223 (3.6%)	89 (3.1%)
(Missing)	40 (0.6%)	9 (0.3%)
Number of children		
Mean	0.6	0.5
Standard Deviation	1.3	0.9
Min, Max	0.0, 23.0	0.0, 16.0
(Missing)	52 (0.8%)	414 (14%)
Community participation, n (%)		
More than once a week	732 (11.6%)	396 (13.6%)
Once a week	716 (11.4%)	279 (9.6%)
One to three times a month	621 (9.9%)	311 (10.7%)
A few times a year	1,589 (25.3%)	706 (24.3%)
Never	2,617 (41.6%)	1,210 (41.7%)
(Missing)	14 (0.2%)	3 (0.1%)
Religious attendance, n (%)		

Table S26b. Weighted summary statistics for outcome variables in Spain

**Outcome**	**Wave 1**<br>N = 6,290	**Wave 2**<br>N = 2,904
More than once a week	323 (5.1%)	150 (5.2%)
Once a week	672 (10.7%)	241 (8.3%)
One to three times a month	438 (7.0%)	184 (6.3%)
A few times a year	1,968 (31.3%)	1,102 (37.9%)
Never	2,863 (45.5%)	1,215 (41.8%)
(Missing)	27 (0.4%)	11 (0.4%)
Loneliness		
Mean	3.3	3.4
Standard Deviation	2.7	2.7
Min, Max	0.0, 10.0	0.0, 10.0
(Missing)	8 (0.1%)	4 (0.1%)
Perceived discrimination, n (%)		
Always	281 (4.5%)	139 (4.8%)
Often	959 (15.2%)	462 (15.9%)
Rarely	2,225 (35.4%)	1,139 (39.2%)
Never	2,817 (44.8%)	1,160 (39.9%)
(Missing)	8 (0.1%)	3 (0.1%)
Orientation to promote good		
Mean	8.1	8.0
Standard Deviation	1.7	1.7
Min, Max	0.0, 10.0	0.0, 10.0
(Missing)	17 (0.3%)	5 (0.2%)
Delayed gratification		
Mean	7.5	7.4
Standard Deviation	2.0	2.1
Min, Max	0.0, 10.0	0.0, 10.0
(Missing)	8 (0.1%)	2 (<0.1%)
Hope		
Mean	8.1	7.9
Standard Deviation	1.9	1.9
Min, Max	0.0, 10.0	0.0, 10.0
(Missing)	23 (0.4%)	19 (0.6%)
Gratitude		
Mean	7.8	7.6
Standard Deviation	2.1	2.1
Min, Max	0.0, 10.0	0.0, 10.0
(Missing)	20 (0.3%)	18 (0.6%)
Showing love/care		
Mean	8.3	8.2
Standard Deviation	2.0	2.0
Min, Max	0.0, 10.0	0.0, 10.0
(Missing)	15 (0.2%)	1 (<0.1%)
Forgivingness, n (%)		
Always	1,414 (22.5%)	648 (22.3%)
Often	3,471 (55.2%)	1,618 (55.7%)
Rarely	1,219 (19.4%)	566 (19.5%)
Never	179 (2.8%)	70 (2.4%)
(Missing)	7 (0.1%)	2 (0.1%)
Charitable giving, n (%)		
Yes	2,210 (35.1%)	915 (31.5%)
No	4,061 (64.6%)	1,961 (67.5%)

Table S26b. Weighted summary statistics for outcome variables in Spain

**Outcome**	**Wave 1**<br>N = 6,290	**Wave 2**<br>N = 2,904
(Missing)	19 (0.3%)	28 (1.0%)
Helping strangers, n (%)		
Yes	3,381 (53.8%)	1,512 (52.1%)
No	2,895 (46.0%)	1,375 (47.4%)
(Missing)	14 (0.2%)	17 (0.6%)
Volunteering, n (%)		
Yes	1,088 (17.3%)	525 (18.1%)
No	5,180 (82.4%)	2,360 (81.2%)
(Missing)	22 (0.3%)	20 (0.7%)
Self-rated physical health		
Mean	6.8	6.7
Standard Deviation	2.1	2.1
Min, Max	0.0, 10.0	0.0, 10.0
(Missing)	20 (0.3%)	11 (0.4%)
Health problems, n (%)		
Yes	1,223 (19.4%)	602 (20.7%)
No	4,984 (79.2%)	2,263 (77.9%)
(Missing)	83 (1.3%)	39 (1.4%)
Pain in past 4 weeks, n (%)		
A lot	815 (13.0%)	410 (14.1%)
Some	2,136 (34.0%)	1,061 (36.5%)
Not very much	2,096 (33.3%)	927 (31.9%)
None at all	1,238 (19.7%)	505 (17.4%)
(Missing)	4 (0.1%)	1 (0.0%)
Number of cigarettes per day		
Mean	3.5	3.6
Standard Deviation	6.7	7.8
Min, Max	0.0, 75.0	0.0, 97.0
(Missing)	76 (1.2%)	55 (1.9%)
Number of drinks per week		
Mean	3.4	3.6
Standard Deviation	7.4	7.9
Min, Max	0.0, 95.0	0.0, 85.0
(Missing)	68 (1.1%)	48 (1.7%)
Days exercise per week		
Mean	2.7	2.7
Standard Deviation	2.4	2.4
Min, Max	0.0, 7.0	0.0, 7.0
(Missing)	6 (<0.1%)	6 (0.2%)
Financial security		
Mean	4.7	4.9
Standard Deviation	3.3	3.2
Min, Max	0.0, 10.0	0.0, 10.0
(Missing)	7 (0.1%)	2 (<0.1%)
Material security		
Mean	5.0	5.2
Standard Deviation	3.3	3.2
Min, Max	0.0, 10.0	0.0, 10.0
(Missing)	6 (<0.1%)	6 (0.2%)
Educational attainment (16+ years), n (%)		
Up to 8	918 (14.6%)	305 (10.5%)

Table S26b. Weighted summary statistics for outcome variables in Spain

Outcome	Wave 1<br>N = 6,290	Wave 2<br>N = 2,904
9-15	4,085 (64.9%)	1,911 (65.8%)
16+	1,284 (20.4%)	688 (23.7%)
(Missing)	3 (0.1%)	0 (0%)
Currently employed, n (%)		
Employed for an employer	2,844 (45.2%)	1,339 (46.1%)
Self-employed	577 (9.2%)	232 (8.0%)
Retired	1,268 (20.2%)	607 (20.9%)
Student	466 (7.4%)	170 (5.9%)
Homemaker	362 (5.7%)	130 (4.5%)
Unemployed and looking for a job	644 (10.2%)	299 (10.3%)
None of these/Other	120 (1.9%)	81 (2.8%)
(Missing)	9 (0.1%)	48 (1.6%)
Financially comfortable/getting by, n (%)		
Living comfortably on present income	1,178 (18.7%)	604 (20.8%)
Getting by on present income	2,703 (43.0%)	1,249 (43.0%)
Finding it difficult on present income	1,730 (27.5%)	770 (26.5%)
Finding it very difficult on present income	547 (8.7%)	218 (7.5%)
(Missing)	132 (2.1%)	63 (2.2%)
Own home, n (%)		
Someone in this household owns this home	3,554 (56.5%)	1,769 (60.9%)
Someone in this household rents this home	1,182 (18.8%)	478 (16.4%)
Both	178 (2.8%)	94 (3.3%)
Neither	1,246 (19.8%)	513 (17.7%)
Rent	0 (0%)	0 (0%)
Own	0 (0%)	0 (0%)
Something else	0 (0%)	0 (0%)
(Missing)	131 (2.1%)	50 (1.7%)
Religious/spiritual connection, n (%)		
Always	1,089 (17.3%)	528 (18.2%)
Often	1,629 (25.9%)	668 (23.0%)
Rarely	1,964 (31.2%)	869 (29.9%)
Never	1,598 (25.4%)	837 (28.8%)
(Missing)	9 (0.1%)	1 (0.0%)
Belief in life after death, n (%)		
Yes	2,516 (40.0%)	1,063 (36.6%)
No	1,712 (27.2%)	840 (28.9%)
Unsure	2,013 (32.0%)	990 (34.1%)
(Missing)	49 (0.8%)	11 (0.4%)
Transformative religious experience, n (%)		
Yes	1,333 (21.2%)	546 (18.8%)
No	4,925 (78.3%)	2,353 (81.0%)
(Missing)	32 (0.5%)	5 (0.2%)
Religious reading or listening, n (%)		
More than once a day	273 (4.3%)	95 (3.3%)
About once a day	477 (7.6%)	150 (5.2%)
Sometimes	2,230 (35.5%)	807 (27.8%)
Never	3,266 (51.9%)	1,833 (63.1%)
(Missing)	44 (0.7%)	20 (0.7%)
Prayer or meditation, n (%)		
More than once a day	601 (9.6%)	193 (6.7%)
About once a day	1,060 (16.8%)	392 (13.5%)

Table S26b. Weighted summary statistics for outcome variables in Spain

**Outcome**	**Wave 1** N = 6,290	**Wave 2** N = 2,904
Sometimes	2,415 (38.4%)	946 (32.6%)
Never	2,195 (34.9%)	1,368 (47.1%)
(Missing)	19 (0.3%)	5 (0.2%)
Belief in God/gods/spiritual forces, n (%)		
One God	2,807 (44.6%)	1,207 (41.6%)
More than one god	135 (2.1%)	67 (2.3%)
An impersonal spiritual force	1,074 (17.1%)	503 (17.3%)
None of these	1,418 (22.5%)	705 (24.3%)
Unsure	831 (13.2%)	414 (14.3%)
(Missing)	25 (0.4%)	9 (0.3%)
Religious centrality, n (%)		
Agree	1,669 (26.5%)	734 (25.3%)
Disagree	1,286 (20.4%)	605 (20.8%)
Not relevant	2,457 (39.1%)	1,194 (41.1%)
Unsure	796 (12.7%)	345 (11.9%)
(Missing)	82 (1.3%)	26 (0.9%)
Religious/spiritual comfort, n (%)		
Agree	2,306 (36.7%)	973 (33.5%)
Disagree	1,174 (18.7%)	552 (19.0%)
Not relevant	2,103 (33.4%)	1,096 (37.7%)
Unsure	669 (10.6%)	282 (9.7%)
(Missing)	38 (0.6%)	1 (0.0%)
Feel loved by God, n (%)		
Agree	2,300 (36.6%)	967 (33.3%)
Disagree	1,178 (18.7%)	556 (19.1%)
Not relevant	2,017 (32.1%)	1,000 (34.4%)
Unsure	756 (12.0%)	370 (12.7%)
(Missing)	40 (0.6%)	12 (0.4%)
Feel punished by God, n (%)		
Agree	601 (9.6%)	241 (8.3%)
Disagree	3,026 (48.1%)	1,387 (47.7%)
Not relevant	1,934 (30.7%)	946 (32.6%)
Unsure	689 (11.0%)	322 (11.1%)
(Missing)	40 (0.6%)	9 (0.3%)
Experienced religious criticism, n (%)		
Agree	404 (6.4%)	170 (5.9%)
Disagree	2,692 (42.8%)	1,174 (40.4%)
Not relevant	2,397 (38.1%)	1,204 (41.5%)
Unsure	747 (11.9%)	345 (11.9%)
(Missing)	50 (0.8%)	10 (0.3%)
Faith-sharing, n (%)		
Agree	1,657 (26.3%)	697 (24.0%)
Disagree	2,086 (33.2%)	936 (32.2%)
Not relevant	2,163 (34.4%)	1,099 (37.8%)
Unsure	349 (5.5%)	169 (5.8%)
(Missing)	36 (0.6%)	3 (0.1%)

Note. N (%); this table is based on non-imputed data. Cumulative percentages for variables may not add up to 100% due to rounding. Wave 1 characteristics weighted using the Gallup provided sampling weight, ANNUAL_WEIGHT_R2; Wave 2 characteristics weighted accounting for attrition by using the adjusted Wave 1 weight, ANNUAL_WEIGHT_R2, multiplied by the created attrition weight to account for dropout, to maintain nationally representative estimates for Wave 2 characteristics.

Table S26c. Unweighted summary statistics for demographic and childhood variables in Spain by retention status

Characteristic	Attritors–Not Observed in Wave 2<br>N = 3,121	Retained–Observed in Wave 2<br>N = 3,203
Forgivingness, n (%)		
Always	734 (23.5%)	689 (21.5%)
Often	1,655 (53.0%)	1,832 (57.2%)
Rarely	619 (19.8%)	608 (19.0%)
Never	110 (3.5%)	71 (2.2%)
(Missing)	3 (0.1%)	4 (0.1%)
Year of birth, n (%)		
1943 or earlier (current age: 80+ years)	12 (0.4%)	27 (0.9%)
1943-1953 (current age: 70-79 years)	203 (6.5%)	290 (9.1%)
1953-1963 (current age: 60-69 years)	286 (9.2%)	661 (20.6%)
1963-1973 (current age: 50-59 years)	484 (15.5%)	714 (22.3%)
1973-1983 (current age: 40-49 years)	673 (21.6%)	707 (22.1%)
1983-1993 (current age: 30-39 years)	651 (20.8%)	486 (15.2%)
1993-1998 (current age: 25-29 years)	319 (10.2%)	159 (5.0%)
1998-2005 (current age: 18-24 years)	492 (15.8%)	158 (4.9%)
(Missing)	0 (0%)	0 (0%)
Age of participant		
Mean	42.1	50.3
Standard Deviation	16.0	14.8
Min, Max	18.0, 88.0	18.0, 86.0
Gender, n (%)		
Male	1,550 (49.7%)	1,600 (50.0%)
Female	1,551 (49.7%)	1,594 (49.8%)
Other	6 (0.2%)	0 (0.0%)
(Missing)	15 (0.5%)	8 (0.3%)
Respondent marital status, n (%)		
Single/Never been married	1,092 (35.0%)	708 (22.1%)
Married	1,190 (38.1%)	1,746 (54.5%)
Separated	141 (4.5%)	98 (3.0%)
Divorced	249 (8.0%)	263 (8.2%)
Widowed	78 (2.5%)	110 (3.4%)
Domestic partner	335 (10.7%)	256 (8.0%)
(Missing)	36 (1.2%)	23 (0.7%)
Education (years), n (%)		
Up to 8	565 (18.1%)	362 (11.3%)
9-15	1,984 (63.6%)	2,121 (66.2%)
16+	571 (18.3%)	717 (22.4%)
(Missing)	0 (0%)	3 (0.1%)
Employment status, n (%)		
Employed for an employer	1,352 (43.3%)	1,505 (47.0%)
Self-employed	320 (10.3%)	262 (8.2%)
Retired	486 (15.6%)	784 (24.5%)
Student	329 (10.5%)	143 (4.5%)
Homemaker	163 (5.2%)	200 (6.2%)
Unemployed and looking for a job	387 (12.4%)	263 (8.2%)
None of these/Other	76 (2.4%)	45 (1.4%)
(Missing)	8 (0.2%)	1 (0.0%)
Current religious service attendance, n (%)		
More than once a week	209 (6.7%)	117 (3.7%)

Table S26c. Unweighted summary statistics for demographic and childhood variables in Spain by retention status

Characteristic	Attritors–Not Observed in Wave 2<br>N = 3,121	Retained–Observed in Wave 2<br>N = 3,203
Once a week	343 (11.0%)	334 (10.4%)
One to three times a month	249 (8.0%)	192 (6.0%)
A few times a year	927 (29.7%)	1,049 (32.8%)
Never	1,376 (44.1%)	1,501 (46.8%)
(Missing)	17 (0.5%)	10 (0.3%)
Immigration status, n (%)		
Born in this country	2,581 (82.7%)	2,902 (90.6%)
Born in another country	526 (16.9%)	294 (9.2%)
(Missing)	14 (0.4%)	7 (0.2%)
Parental marital status around age 12, n (%)		
Parents were married	2,422 (77.6%)	2,866 (89.5%)
Parents were divorced	261 (8.4%)	135 (4.2%)
Parents were never married	226 (7.2%)	100 (3.1%)
One or both of them had died	74 (2.4%)	58 (1.8%)
Unsure	59 (1.9%)	17 (0.5%)
(Missing)	80 (2.6%)	27 (0.9%)
Religious service attendance around age 12, n (%)		
At least once a week	1,068 (34.2%)	1,311 (40.9%)
One to three times a month	591 (18.9%)	548 (17.1%)
Less than once a month	681 (21.8%)	629 (19.6%)
Never	762 (24.4%)	696 (21.7%)
(Missing)	19 (0.6%)	19 (0.6%)
Relationship with mother when growing up, n (%)		
Very good	2,227 (71.3%)	2,344 (73.2%)
Somewhat good	642 (20.6%)	630 (19.7%)
Somewhat bad	133 (4.3%)	115 (3.6%)
Very bad	49 (1.6%)	48 (1.5%)
(Does not apply)	53 (1.7%)	56 (1.8%)
(Missing)	18 (0.6%)	10 (0.3%)
Relationship with father when growing up, n (%)		
Very good	1,991 (63.8%)	2,144 (66.9%)
Somewhat good	697 (22.3%)	713 (22.2%)
Somewhat bad	176 (5.6%)	141 (4.4%)
Very bad	97 (3.1%)	83 (2.6%)
(Does not apply)	140 (4.5%)	111 (3.5%)
(Missing)	19 (0.6%)	12 (0.4%)
Felt like an outsider in family when growing up, n (%)		
Yes	329 (10.5%)	277 (8.6%)
No	2,751 (88.1%)	2,895 (90.4%)
(Missing)	41 (1.3%)	31 (1.0%)
Experienced abuse when growing up, n (%)		
Yes	350 (11.2%)	317 (9.9%)
No	2,691 (86.2%)	2,854 (89.1%)
(Missing)	81 (2.6%)	31 (1.0%)
Self-rated health when growing up, n (%)		
Excellent	1,289 (41.3%)	1,167 (36.4%)
Very good	1,067 (34.2%)	1,254 (39.2%)

Table S26c. Unweighted summary statistics for demographic and childhood variables in Spain by retention status

**Characteristic**	**Attritors–Not Observed in Wave 2**<br>N = 3,121	**Retained–Observed in Wave 2**<br>N = 3,203
Good	612 (19.6%)	618 (19.3%)
Fair	76 (2.4%)	89 (2.8%)
Poor	72 (2.3%)	61 (1.9%)
(Missing)	5 (0.2%)	13 (0.4%)
Subjective financial status of family growing up, n (%)		
Lived comfortably	1,084 (34.7%)	977 (30.5%)
Got by	1,416 (45.4%)	1,536 (48.0%)
Found it difficult	554 (17.8%)	618 (19.3%)
Found it very difficult	52 (1.7%)	59 (1.8%)
(Missing)	15 (0.5%)	12 (0.4%)
Religious affiliation growing up, n (%)		
Christianity	2,439 (78.2%)	2,686 (83.9%)
Taoism	0 (0%)	0 (0%)
Confucianism	0 (0%)	1 (0.0%)
Primal, Animist, or Folk religion	4 (0.1%)	0 (0.0%)
Spiritism	0 (0%)	0 (0%)
Umbanda, Candomblé, and other African-derived religions	0 (0%)	0 (0%)
Chinese folk/traditional religion	0 (0%)	0 (0%)
Islam	106 (3.4%)	30 (0.9%)
Hinduism	2 (0.1%)	3 (0.1%)
Buddhism	5 (0.2%)	3 (0.1%)
Judaism	5 (0.2%)	0 (0.0%)
Sikhism	1 (0.0%)	1 (0.0%)
Baha'i	0 (0%)	0 (0%)
Jainism	0 (0%)	0 (0%)
Shinto	0 (0%)	0 (0%)
Some other religion	9 (0.3%)	4 (0.1%)
No religion/Atheist/Agnostic	538 (17.2%)	459 (14.3%)
(Missing)	12 (0.4%)	15 (0.5%)

Note. N (%); this table is based on non-imputed data. Cumulative percentages for variables may not add up to 100% due to rounding.

Table S26d. Unweighted summary statistics for Wave 1 outcome variables in Spain by retention status.

**Outcome**	**Attritors–Not Observed in Wave 2** N = 3,121	**Retained–Observed in Wave 2** N = 3,203
Secure flourishing index		
Mean	6.9	6.9
Standard Deviation	1.4	1.4
Min, Max	0.0, 10.0	1.0, 10.0
(Missing)	74 (2.4%)	73 (2.3%)
Flourishing index		
Mean	7.3	7.3
Standard Deviation	1.5	1.4
Min, Max	0.0, 10.0	1.1, 10.0
(Missing)	68 (2.2%)	70 (2.2%)
Happiness & life satisfaction		
Mean	6.8	6.9
Standard Deviation	2.1	1.9
Min, Max	0.0, 10.0	0.0, 10.0
(Missing)	17 (0.6%)	10 (0.3%)
Physical & mental health		
Mean	7.2	7.1
Standard Deviation	1.9	1.9
Min, Max	0.0, 10.0	0.0, 10.0
(Missing)	16 (0.5%)	12 (0.4%)
Meaning & purpose		
Mean	7.3	7.3
Standard Deviation	1.9	1.9
Min, Max	0.0, 10.0	0.0, 10.0
(Missing)	16 (0.5%)	25 (0.8%)
Character & virtue		
Mean	7.8	7.8
Standard Deviation	1.6	1.5
Min, Max	0.0, 10.0	0.5, 10.0
(Missing)	19 (0.6%)	4 (0.1%)
Close social relationships		
Mean	7.4	7.4
Standard Deviation	2.1	2.0
Min, Max	0.0, 10.0	0.0, 10.0
(Missing)	10 (0.3%)	28 (0.9%)
Financial & material security		
Mean	4.7	4.9
Standard Deviation	3.1	3.0
Min, Max	0.0, 10.0	0.0, 10.0
(Missing)	7 (0.2%)	6 (0.2%)
Happiness		
Mean	6.9	6.9
Standard Deviation	2.1	1.9
Min, Max	0.0, 10.0	0.0, 10.0
(Missing)	11 (0.4%)	5 (0.1%)
Life satisfaction		
Mean	6.8	6.8
Standard Deviation	2.3	2.1
Min, Max	0.0, 10.0	0.0, 10.0

Table S26d. Unweighted summary statistics for Wave 1 outcome variables in Spain by retention status.

Outcome	Attritors–Not Observed in Wave 2<br>N = 3,121	Retained–Observed in Wave 2<br>N = 3,203
(Missing)	9 (0.3%)	6 (0.2%)
Current life evaluation		
Mean	6.7	6.7
Standard Deviation	2.1	1.8
Min, Max	0.0, 10.0	0.0, 10.0
(Missing)	7 (0.2%)	1 (<0.1%)
Future life evaluation		
Mean	7.8	7.5
Standard Deviation	1.9	1.8
Min, Max	0.0, 10.0	0.0, 10.0
(Missing)	11 (0.4%)	5 (0.2%)
Optimism		
Mean	8.3	8.2
Standard Deviation	2.0	1.9
Min, Max	0.0, 10.0	0.0, 10.0
(Missing)	8 (0.3%)	9 (0.3%)
Freedom to pursue what's important		
Mean	7.4	7.5
Standard Deviation	2.3	2.2
Min, Max	0.0, 10.0	0.0, 10.0
(Missing)	1 (<0.1%)	2 (<0.1%)
Inner peace, n (%)		
Always	384 (12.3%)	397 (12.4%)
Often	1,321 (42.3%)	1,402 (43.8%)
Rarely	1,209 (38.7%)	1,158 (36.1%)
Never	193 (6.2%)	236 (7.4%)
(Missing)	15 (0.5%)	9 (0.3%)
Life balance, n (%)		
Always	508 (16.3%)	483 (15.1%)
Often	1,860 (59.6%)	1,989 (62.1%)
Rarely	684 (21.9%)	661 (20.6%)
Never	66 (2.1%)	65 (2.0%)
(Missing)	2 (0.1%)	6 (0.2%)
Sense of mastery, n (%)		
Always	823 (26.4%)	717 (22.4%)
Often	1,772 (56.8%)	2,072 (64.7%)
Rarely	470 (15.0%)	352 (11.0%)
Never	43 (1.4%)	40 (1.3%)
(Missing)	14 (0.4%)	22 (0.7%)
Meaningful activities		
Mean	7.3	7.3
Standard Deviation	2.3	2.1
Min, Max	0.0, 10.0	0.0, 10.0
(Missing)	3 (<0.1%)	11 (0.4%)
Understanding purpose		
Mean	7.4	7.3
Standard Deviation	2.3	2.2
Min, Max	0.0, 10.0	0.0, 10.0
(Missing)	13 (0.4%)	13 (0.4%)

Table S26d. Unweighted summary statistics for Wave 1 outcome variables in Spain by retention status.

**Outcome**	**Attritors–Not Observed in Wave 2** N = 3,121	**Retained–Observed in Wave 2** N = 3,203
Self-rated mental health		
Mean	7.4	7.4
Standard Deviation	2.3	2.1
Min, Max	0.0, 10.0	0.0, 10.0
(Missing)	6 (0.2%)	3 (<0.1%)
Traumatic distress, n (%)		
A lot	431 (13.8%)	300 (9.4%)
Some	857 (27.4%)	772 (24.1%)
Not very much	872 (28.0%)	927 (28.9%)
Not at all	954 (30.6%)	1,202 (37.5%)
(Missing)	8 (0.2%)	2 (0.1%)
Depression symptoms composite, n (%)	1,016 (32.7%)	854 (26.8%)
(Missing)	18 (0.6%)	21 (0.7%)
Depression – feel hopeless, n (%)		
Nearly every day	329 (10.5%)	252 (7.9%)
More than half the days	464 (14.9%)	386 (12.1%)
Several days	1,142 (36.6%)	1,083 (33.8%)
Not at all	1,181 (37.8%)	1,468 (45.8%)
(Missing)	6 (0.2%)	14 (0.4%)
Depression – loss of interest, n (%)		
Nearly every day	294 (9.4%)	233 (7.3%)
More than half the days	593 (19.0%)	545 (17.0%)
Several days	1,223 (39.2%)	1,203 (37.6%)
Not at all	995 (31.9%)	1,215 (37.9%)
(Missing)	16 (0.5%)	7 (0.2%)
Anxiety symptoms composite, n (%)	1,154 (37.1%)	904 (28.4%)
(Missing)	12 (0.4%)	20 (0.6%)
Anxiety – feel on edge, n (%)		
Nearly every day	472 (15.1%)	332 (10.4%)
More than half the days	490 (15.7%)	497 (15.5%)
Several days	1,420 (45.5%)	1,380 (43.1%)
Not at all	730 (23.4%)	979 (30.6%)
(Missing)	10 (0.3%)	15 (0.5%)
Anxiety – cannot stop worrying, n (%)		
Nearly every day	398 (12.7%)	303 (9.4%)
More than half the days	496 (15.9%)	383 (12.0%)
Several days	1,041 (33.3%)	1,017 (31.8%)
Not at all	1,181 (37.8%)	1,495 (46.7%)
(Missing)	6 (0.2%)	5 (0.2%)
Suffering, n (%)		
A lot	413 (13.2%)	330 (10.3%)
Some	1,131 (36.2%)	1,182 (36.9%)
Not very much	1,024 (32.8%)	1,132 (35.3%)
Not at all	537 (17.2%)	543 (16.9%)
(Missing)	16 (0.5%)	16 (0.5%)
Relationship contentment		
Mean	7.7	7.7
Standard Deviation	2.1	2.0
Min, Max	0.0, 10.0	0.0, 10.0

Table S26d. Unweighted summary statistics for Wave 1 outcome variables in Spain by retention status.

**Outcome**	**Attritors–Not Observed in Wave 2** N = 3,121	**Retained–Observed in Wave 2** N = 3,203
(Missing)	8 (0.3%)	11 (0.3%)
Relationship satisfaction		
Mean	7.1	7.1
Standard Deviation	2.4	2.3
Min, Max	0.0, 10.0	0.0, 10.0
(Missing)	2 (<0.1%)	21 (0.7%)
Social support		
Mean	8.1	8.1
Standard Deviation	2.3	2.2
Min, Max	0.0, 10.0	0.0, 10.0
(Missing)	25 (0.8%)	10 (0.3%)
Intimate/close friend, n (%)		
Yes	2,720 (87.2%)	2,764 (86.3%)
No	380 (12.2%)	433 (13.5%)
(Missing)	20 (0.6%)	6 (0.2%)
Government approval, n (%)		
Strongly approve	220 (7.1%)	201 (6.3%)
Somewhat approve	799 (25.6%)	1,005 (31.4%)
Neither approve nor disapprove	891 (28.6%)	554 (17.3%)
Somewhat disapprove	724 (23.2%)	762 (23.8%)
Strongly disapprove	468 (15.0%)	668 (20.8%)
(Missing)	19 (0.6%)	13 (0.4%)
Say in government, n (%)		
Agree	438 (14.0%)	463 (14.5%)
Disagree	1,860 (59.6%)	1,965 (61.4%)
Unsure	806 (25.8%)	769 (24.0%)
(Missing)	18 (0.6%)	6 (0.2%)
Belonging in country		
Mean	7.5	7.5
Standard Deviation	2.5	2.4
Min, Max	0.0, 10.0	0.0, 10.0
(Missing)	34 (1.1%)	17 (0.5%)
City/place satisfaction, n (%)		
Satisfied	2,451 (78.5%)	2,623 (81.9%)
Dissatisfied	412 (13.2%)	360 (11.2%)
Unsure	225 (7.2%)	196 (6.1%)
(Missing)	32 (1.0%)	24 (0.8%)
Trust within country, n (%)		
All people	67 (2.1%)	38 (1.2%)
Most people	410 (13.1%)	580 (18.1%)
Some people	1,288 (41.3%)	1,460 (45.6%)
Not very many people	1,208 (38.7%)	1,008 (31.5%)
None	130 (4.2%)	95 (3.0%)
(Missing)	18 (0.6%)	22 (0.7%)
Number of children		
Mean	0.8	0.5
Standard Deviation	1.6	0.9
Min, Max	0.0, 23.0	0.0, 8.0
(Missing)	38 (1.2%)	15 (0.5%)

Table S26d. Unweighted summary statistics for Wave 1 outcome variables in Spain by retention status.

Outcome	Attritors–Not Observed in Wave 2 N = 3,121	Retained–Observed in Wave 2 N = 3,203
Community participation, n (%)		
More than once a week	402 (12.9%)	336 (10.5%)
Once a week	358 (11.5%)	361 (11.3%)
One to three times a month	330 (10.6%)	295 (9.2%)
A few times a year	778 (24.9%)	820 (25.6%)
Never	1,243 (39.8%)	1,387 (43.3%)
(Missing)	11 (0.3%)	4 (0.1%)
Religious attendance, n (%)		
More than once a week	209 (6.7%)	117 (3.7%)
Once a week	343 (11.0%)	334 (10.4%)
One to three times a month	249 (8.0%)	192 (6.0%)
A few times a year	927 (29.7%)	1,049 (32.8%)
Never	1,376 (44.1%)	1,501 (46.8%)
(Missing)	17 (0.5%)	10 (0.3%)
Loneliness		
Mean	3.4	3.3
Standard Deviation	2.7	2.7
Min, Max	0.0, 10.0	0.0, 10.0
(Missing)	8 (0.3%)	0 (0%)
Perceived discrimination, n (%)		
Always	179 (5.7%)	106 (3.3%)
Often	545 (17.5%)	422 (13.2%)
Rarely	1,145 (36.7%)	1,093 (34.1%)
Never	1,249 (40.0%)	1,577 (49.2%)
(Missing)	3 (0.1%)	5 (0.2%)
Orientation to promote good		
Mean	8.1	8.1
Standard Deviation	1.8	1.6
Min, Max	0.0, 10.0	0.0, 10.0
(Missing)	13 (0.4%)	3 (0.1%)
Delayed gratification		
Mean	7.6	7.5
Standard Deviation	2.1	1.9
Min, Max	0.0, 10.0	0.0, 10.0
(Missing)	6 (0.2%)	2 (<0.1%)
Hope		
Mean	8.1	8.0
Standard Deviation	1.9	1.8
Min, Max	0.0, 10.0	0.0, 10.0
(Missing)	14 (0.5%)	9 (0.3%)
Gratitude		
Mean	7.8	7.8
Standard Deviation	2.1	2.1
Min, Max	0.0, 10.0	0.0, 10.0
(Missing)	13 (0.4%)	6 (0.2%)
Showing love/care		
Mean	8.3	8.3
Standard Deviation	2.1	1.9
Min, Max	0.0, 10.0	0.0, 10.0

Table S26d. Unweighted summary statistics for Wave 1 outcome variables in Spain by retention status.

Outcome	Attritors–Not Observed in Wave 2 N = 3,121	Retained–Observed in Wave 2 N = 3,203
(Missing)	9 (0.3%)	6 (0.2%)
Forgivingness, n (%)		
Always	734 (23.5%)	689 (21.5%)
Often	1,655 (53.0%)	1,832 (57.2%)
Rarely	619 (19.8%)	608 (19.0%)
Never	110 (3.5%)	71 (2.2%)
(Missing)	3 (0.1%)	4 (0.1%)
Charitable giving, n (%)		
Yes	991 (31.7%)	1,227 (38.3%)
No	2,118 (67.9%)	1,969 (61.5%)
(Missing)	12 (0.4%)	7 (0.2%)
Helping strangers, n (%)		
Yes	1,847 (59.2%)	1,559 (48.7%)
No	1,265 (40.5%)	1,639 (51.2%)
(Missing)	9 (0.3%)	5 (0.2%)
Volunteering, n (%)		
Yes	586 (18.8%)	510 (15.9%)
No	2,521 (80.8%)	2,685 (83.8%)
(Missing)	14 (0.4%)	8 (0.3%)
Self-rated physical health		
Mean	7.0	6.7
Standard Deviation	2.1	2.0
Min, Max	0.0, 10.0	0.0, 10.0
(Missing)	12 (0.4%)	9 (0.3%)
Health problems, n (%)		
Yes	600 (19.2%)	629 (19.6%)
No	2,470 (79.1%)	2,541 (79.3%)
(Missing)	51 (1.6%)	33 (1.0%)
Pain in past 4 weeks, n (%)		
A lot	442 (14.2%)	379 (11.8%)
Some	1,060 (34.0%)	1,088 (34.0%)
Not very much	1,004 (32.2%)	1,102 (34.4%)
None at all	611 (19.6%)	633 (19.8%)
(Missing)	3 (0.1%)	1 (0.0%)
Number of cigarettes per day		
Mean	3.5	3.5
Standard Deviation	6.7	6.7
Min, Max	0.0, 75.0	0.0, 60.0
(Missing)	48 (1.5%)	29 (0.9%)
Number of drinks per week		
Mean	3.4	3.5
Standard Deviation	8.1	6.7
Min, Max	0.0, 85.0	0.0, 95.0
(Missing)	40 (1.3%)	29 (0.9%)
Days exercise per week		
Mean	2.6	2.7
Standard Deviation	2.3	2.4
Min, Max	0.0, 7.0	0.0, 7.0
(Missing)	2 (<0.1%)	4 (0.1%)

Table S26d. Unweighted summary statistics for Wave 1 outcome variables in Spain by retention status.

**Outcome**	**Attritors–Not Observed in Wave 2** N = 3,121	**Retained–Observed in Wave 2** N = 3,203
Financial security		
Mean	4.6	4.8
Standard Deviation	3.3	3.2
Min, Max	0.0, 10.0	0.0, 10.0
(Missing)	6 (0.2%)	2 (<0.1%)
Material security		
Mean	4.9	5.1
Standard Deviation	3.4	3.2
Min, Max	0.0, 10.0	0.0, 10.0
(Missing)	2 (<0.1%)	4 (0.1%)
Educational attainment (16+ years), n (%)		
Up to 8	565 (18.1%)	362 (11.3%)
9-15	1,984 (63.6%)	2,121 (66.2%)
16+	571 (18.3%)	717 (22.4%)
(Missing)	0 (0%)	3 (0.1%)
Currently employed, n (%)		
Employed for an employer	1,352 (43.3%)	1,505 (47.0%)
Self-employed	320 (10.3%)	262 (8.2%)
Retired	486 (15.6%)	784 (24.5%)
Student	329 (10.5%)	143 (4.5%)
Homemaker	163 (5.2%)	200 (6.2%)
Unemployed and looking for a job	387 (12.4%)	263 (8.2%)
None of these/Other	76 (2.4%)	45 (1.4%)
(Missing)	8 (0.2%)	1 (0.0%)
Financially comfortable/getting by, n (%)		
Living comfortably on present income	568 (18.2%)	615 (19.2%)
Getting by on present income	1,250 (40.1%)	1,464 (45.7%)
Finding it difficult on present income	906 (29.0%)	835 (26.1%)
Finding it very difficult on present income	298 (9.5%)	253 (7.9%)
(Missing)	99 (3.2%)	35 (1.1%)
Own home, n (%)		
Someone in this household owns this home	1,545 (49.5%)	2,020 (63.1%)
Someone in this household rents this home	665 (21.3%)	526 (16.4%)
Both	109 (3.5%)	71 (2.2%)
Neither	706 (22.6%)	550 (17.2%)
Rent	0 (0%)	0 (0%)
Own	0 (0%)	0 (0%)
Something else	0 (0%)	0 (0%)
(Missing)	96 (3.1%)	36 (1.1%)
Religious/spiritual connection, n (%)		
Always	641 (20.5%)	458 (14.3%)
Often	773 (24.8%)	864 (27.0%)
Rarely	943 (30.2%)	1,030 (32.2%)
Never	762 (24.4%)	844 (26.4%)
(Missing)	2 (0.1%)	7 (0.2%)
Belief in life after death, n (%)		
Yes	1,351 (43.3%)	1,183 (36.9%)
No	787 (25.2%)	932 (29.1%)
Unsure	952 (30.5%)	1,070 (33.4%)

Table S26d. Unweighted summary statistics for Wave 1 outcome variables in Spain by retention status.

Outcome	Attritors–Not Observed in Wave 2<br>N = 3,121	Retained–Observed in Wave 2<br>N = 3,203
(Missing)	31 (1.0%)	18 (0.6%)
Transformative religious experience, n (%)		
Yes	795 (25.5%)	551 (17.2%)
No	2,303 (73.8%)	2,643 (82.5%)
(Missing)	23 (0.7%)	10 (0.3%)
Religious reading or listening, n (%)		
More than once a day	171 (5.5%)	106 (3.3%)
About once a day	302 (9.7%)	180 (5.6%)
Sometimes	1,083 (34.7%)	1,158 (36.1%)
Never	1,540 (49.4%)	1,740 (54.3%)
(Missing)	25 (0.8%)	20 (0.6%)
Prayer or meditation, n (%)		
More than once a day	351 (11.3%)	255 (8.0%)
About once a day	553 (17.7%)	514 (16.0%)
Sometimes	1,134 (36.3%)	1,292 (40.3%)
Never	1,067 (34.2%)	1,139 (35.6%)
(Missing)	16 (0.5%)	4 (0.1%)
Belief in God/gods/spiritual forces, n (%)		
One God	1,462 (46.8%)	1,363 (42.6%)
More than one god	77 (2.5%)	60 (1.9%)
An impersonal spiritual force	515 (16.5%)	563 (17.6%)
None of these	668 (21.4%)	756 (23.6%)
Unsure	382 (12.3%)	452 (14.1%)
(Missing)	17 (0.5%)	9 (0.3%)
Religious centrality, n (%)		
Agree	805 (25.8%)	872 (27.2%)
Disagree	646 (20.7%)	647 (20.2%)
Not relevant	1,213 (38.9%)	1,257 (39.2%)
Unsure	403 (12.9%)	398 (12.4%)
(Missing)	53 (1.7%)	30 (0.9%)
Religious/spiritual comfort, n (%)		
Agree	1,173 (37.6%)	1,146 (35.8%)
Disagree	593 (19.0%)	588 (18.4%)
Not relevant	1,007 (32.3%)	1,105 (34.5%)
Unsure	318 (10.2%)	354 (11.0%)
(Missing)	29 (0.9%)	10 (0.3%)
Feel loved by God, n (%)		
Agree	1,198 (38.4%)	1,117 (34.9%)
Disagree	552 (17.7%)	631 (19.7%)
Not relevant	989 (31.7%)	1,039 (32.4%)
Unsure	354 (11.4%)	405 (12.6%)
(Missing)	29 (0.9%)	11 (0.4%)
Feel punished by God, n (%)		
Agree	329 (10.5%)	277 (8.7%)
Disagree	1,455 (46.6%)	1,585 (49.5%)
Not relevant	954 (30.6%)	990 (30.9%)
Unsure	351 (11.3%)	342 (10.7%)
(Missing)	32 (1.0%)	9 (0.3%)
Experienced religious criticism, n (%)		

Table S26d. Unweighted summary statistics for Wave 1 outcome variables in Spain by retention status.

Outcome	Attritors–Not Observed in Wave 2 N = 3,121	Retained–Observed in Wave 2 N = 3,203
Agree	253 (8.1%)	156 (4.9%)
Disagree	1,269 (40.7%)	1,435 (44.8%)
Not relevant	1,151 (36.9%)	1,258 (39.3%)
Unsure	407 (13.0%)	346 (10.8%)
(Missing)	42 (1.3%)	9 (0.3%)
Faith-sharing, n (%)		
Agree	876 (28.1%)	792 (24.7%)
Disagree	943 (30.2%)	1,150 (35.9%)
Not relevant	1,083 (34.7%)	1,092 (34.1%)
Unsure	187 (6.0%)	164 (5.1%)
(Missing)	32 (1.0%)	4 (0.1%)

Note. N (%); this table is based on non-imputed data. Cumulative percentages for variables may not add up to 100% due to rounding.

Table S26e. Summary of fitted attrition model in Spain

Characteristic	Odds Ratio	95% CI	p-value
**ANNUAL_WEIGHT_R2**	1.12	1.04, 1.20	0.003
**Recruitment Survey Mode**			
CAWI	—	—	
CATI	1.16	0.98, 1.36	0.077
**Happiness & life satisfaction**	1.08	0.96, 1.21	0.211
**Physical & mental health**	0.88	0.79, 0.98	0.016
**Meaning & purpose**	0.97	0.87, 1.09	0.644
**Character & virtue**	0.97	0.90, 1.06	0.503
**Close social relationships**	0.98	0.89, 1.06	0.582
**Financial & material security**	1.01	0.93, 1.09	0.813
**Extraversion**	0.93	0.86, 1.00	0.056
**Openness to experience**	0.97	0.90, 1.05	0.440
**Agreeableness**	0.94	0.87, 1.01	0.097
**Conscientiousness**	1.05	0.97, 1.14	0.245
**Neuroticism**	1.16	1.06, 1.26	9.50e-04
**Depression symptoms composite**	1.00	0.91, 1.10	0.963
**Anxiety symptoms composite**	0.92	0.84, 1.01	0.086
**Loneliness**	1.06	0.97, 1.15	0.185
**Days exercise per week**	1.06	0.99, 1.15	0.114
**Year of birth (age group)**			
1973-1983 (current age: 40-49 years)	—	—	
1983-1993 (current age: 30-39 years)	0.74	0.61, 0.89	0.002
1963-1973 (current age: 50-59 years)	1.35	1.11, 1.64	0.002
1998-2005 (current age: 18-24 years)	0.39	0.27, 0.56	2.43e-07
1953-1963 (current age: 60-69 years)	1.90	1.40, 2.56	3.11e-05
1993-1998 (current age: 25-29 years)	0.53	0.40, 0.70	7.33e-06
1943-1953 (current age: 70-79 years)	1.21	0.76, 1.91	0.425
1943 or earlier (current age: 80+ years)	1.96	0.80, 4.80	0.141
**Gender of respondent**			
Female	—	—	
Male	0.96	0.83, 1.12	0.623
Other	0.10	0.01, 0.85	0.034
**Marital status**			
Married	—	—	
Single/Never been married	0.80	0.67, 0.96	0.019
Domestic partner	0.86	0.67, 1.12	0.264
Divorced	0.87	0.66, 1.13	0.295
Separated	0.80	0.55, 1.15	0.222
Widowed	0.95	0.57, 1.60	0.854
**Employment status**			
Employed for an employer	—	—	
Unemployed and looking for a job	0.98	0.77, 1.24	0.871
Retired	0.83	0.61, 1.12	0.223
Self-employed	0.74	0.59, 0.92	0.007
Student	1.03	0.70, 1.51	0.899
Homemaker	1.00	0.69, 1.45	0.981
None of these/Other	0.73	0.47, 1.15	0.177
**Religious attendance**			
Never	—	—	
A few times a year	0.98	0.83, 1.15	0.797
Once a week	0.96	0.73, 1.25	0.754
One to three times a month	0.88	0.66, 1.17	0.369

Table S26e. Summary of fitted attrition model in Spain

**Characteristic**	**Odds Ratio**	**95% CI**	**p-value**
More than once a week	0.57	0.41, 0.81	0.002
**Educational attainment (16+ years)**			
9-15	—	—	
16+	1.19	1.01, 1.41	0.039
Up to 8	0.46	0.35, 0.61	1.18e-07
**Born in This country**			
Born in this country	—	—	
Born in another country	0.73	0.59, 0.90	0.003
**Urbanicity**			
A small town or village	—	—	
A large city	1.12	0.95, 1.33	0.166
A rural area or on a farm	0.87	0.69, 1.09	0.229
A suburb of a large city	0.81	0.63, 1.05	0.107
**Monthly household income**			
Spain: 251 - 1,000 euros	—	—	
Spain: 1,001 - 1,250 euros	1.21	0.92, 1.59	0.173
Spain: 1,251 – 1,500 euros	1.05	0.79, 1.40	0.721
(None/No household income)	0.56	0.37, 0.83	0.004
Spain: 1,501 - 1,750 euros	1.27	0.91, 1.76	0.156
Spain: 1,751 – 2,000 euros	1.36	0.97, 1.92	0.075
Spain: 2,001 - 2,250 euros	1.07	0.77, 1.49	0.696
Spain: 250 euros or less	1.00	0.69, 1.44	0.981
Spain: 2,251 – 2,500 euros	1.41	1.00, 1.99	0.051
Spain: 2,751 – 3,000 euros	1.16	0.80, 1.70	0.435
Spain: 2,501 - 2,750 euros	1.39	0.94, 2.07	0.098
Spain: 3,001 - 3,500 euros	1.50	1.01, 2.22	0.043
Spain: 3,501 - 4,000 euros	1.16	0.76, 1.78	0.492
Spain: 4,001 - 5,000 euros	1.20	0.73, 1.97	0.479
Spain: 5,001 – 6,000 euros	0.79	0.47, 1.32	0.363
Spain: More than 6,000 euros	0.80	0.47, 1.36	0.413

Abbreviations: CI = Confidence Interval, OR = Odds Ratio

Notes. N=6290; attrition weights were estimated using the ’survey::svyglm(family=quasibinomial(’logit’))’ function. All continuous predictors were standardized and all categorical predictors used the most common category as the reference group. Reported p-values are based on the fitted regression model and no adjustments for multiple testing were done within this table.

Table S26f. Summary of principal components in Spain

PC	Percent Explained by each PC	Cumulative Percent Explained
1	31.83	31.83
2	6.26	38.09
3	5.44	43.53
4	2.58	46.11
5	2.01	48.12
6	1.77	49.89
**7**	**1.56**	**51.45**
8	1.53	52.99
9	1.42	54.40
10	1.41	55.81
11	1.36	57.17
12	1.32	58.49
13	1.27	59.75
14	1.18	60.94
15	1.17	62.10
16	1.14	63.24
17	1.11	64.36
18	1.05	65.41
19	1.03	66.44
20	1.01	67.45

Notes. N=6290; PCA was conducted using 'survey::svyprcomp(.)' function using all available contemporaneous exposures at wave 1. All PCs were standardized prior to being used as predictors. The bolded row represented the number of retained components for analysis was 7.

Table S26g. Associations of forgivingness with adult well-being and other outcomes at Wave 2 in Spain

Outcome	Model 1: Demographic and Childhood Variables as Covariates					Model 2: Demographic, Childhood, and Other Wave 1 Confounding Variables (Via Principal Components) as Covariates				
	RR	ES	95% CI	SE	p-value	RR	ES	95% CI	SE	p-value
Human Flourishing										
Secure flourishing index		0.06	(0.02,0.10)	0.02	1.25e-03**		0.03	(-0.00,0.06)	0.01	0.067
Flourishing index		0.07	(0.04,0.11)	0.02	6.29e-05***		0.03	(0.00,0.06)	0.01	0.041*
Happiness & life satisfaction		0.02	(-0.02,0.06)	0.02	0.261		-0.00	(-0.04,0.03)	0.02	0.780
Physical & mental health		0.04	(-0.01,0.08)	0.02	0.106		0.01	(-0.02,0.05)	0.02	0.448
Meaning & purpose		0.07	(0.03,0.11)	0.02	1.8e-03**		0.03	(-0.01,0.06)	0.02	0.175
Character & virtue		0.11	(0.06,0.15)	0.02	1.08e-06***		0.07	(0.03,0.11)	0.02	1.42e-03**
Close social relationships		0.05	(0.01,0.09)	0.02	0.008*		0.01	(-0.02,0.05)	0.02	0.430
Financial & material security		-0.01	(-0.05,0.03)	0.02	0.649		0.01	(-0.03,0.04)	0.02	0.754
Psychological Well-Being										
Happiness (a)		0.03	(-0.01,0.07)	0.02	0.111		0.01	(-0.03,0.05)	0.02	0.662
Life satisfaction (a)		0.01	(-0.03,0.05)	0.02	0.742		-0.02	(-0.05,0.02)	0.02	0.425
Current life evaluation		0.01	(-0.03,0.06)	0.02	0.590		-0.01	(-0.05,0.04)	0.02	0.788
Future life evaluation		0.05	(0.01,0.09)	0.02	0.014*		0.03	(-0.01,0.06)	0.02	0.150
Optimism		0.07	(0.03,0.11)	0.02	4.06e-04***		0.03	(-0.01,0.07)	0.02	0.099
Freedom to pursue what's important		0.00	(-0.04,0.04)	0.02	0.920		-0.03	(-0.07,0.01)	0.02	0.141
Inner peace	1.00		(0.98,1.02)	0.01	0.814	1.00		(0.98,1.02)	0.01	0.973
Life balance	1.02		(1.00,1.03)	0.01	0.051	1.01		(0.99,1.03)	0.01	0.294
Sense of mastery	1.00		(0.99,1.02)	0.01	0.548	1.00		(0.98,1.01)	0.01	0.611
Meaningful activities (c)		0.03	(-0.01,0.07)	0.02	0.179		-0.00	(-0.04,0.04)	0.02	0.884
Understanding purpose (c)		0.08	(0.04,0.12)	0.02	2.05e-04***		0.05	(0.01,0.09)	0.02	0.030*
Self-rated mental health (b)		0.03	(-0.01,0.07)	0.02	0.184		0.01	(-0.03,0.04)	0.02	0.767
Psychological Distress										
Traumatic distress	1.01		(0.99,1.03)	0.01	0.506	1.00		(0.98,1.02)	0.01	0.816
Depression symptoms composite	1.00		(0.98,1.01)	0.01	0.601	1.00		(0.98,1.02)	0.01	0.905
Depression – feel hopeless	1.00		(0.99,1.02)	0.01	0.839	1.00		(0.99,1.02)	0.01	0.581
Depression – loss of interest	1.00		(0.98,1.02)	0.01	0.657	1.00		(0.98,1.02)	0.01	0.947
Anxiety symptoms composite	1.00		(0.98,1.02)	0.01	0.706	1.00		(0.98,1.02)	0.01	0.763
Anxiety – feel on edge	1.01		(0.99,1.03)	0.01	0.460	1.01		(0.99,1.03)	0.01	0.370
Anxiety – cannot stop worrying	1.00		(0.98,1.02)	0.01	0.737	1.00		(0.98,1.02)	0.01	0.836
Suffering	1.01		(0.99,1.03)	0.01	0.249	1.01		(0.99,1.03)	0.01	0.467
Social Well-Being										
Relationship contentment (e)		0.04	(-0.01,0.09)	0.02	0.085		0.01	(-0.04,0.06)	0.02	0.704
Relationship satisfaction (e)		0.05	(0.01,0.09)	0.02	0.021*		0.02	(-0.02,0.06)	0.02	0.423
Social support		0.08	(0.03,0.12)	0.02	4.81e-04***		0.05	(0.01,0.09)	0.02	0.010*
Intimate/close friend	1.02		(1.00,1.04)	0.01	0.011*	1.01		(1.00,1.03)	0.01	0.073
Government approval	1.03		(1.01,1.05)	0.01	0.007*	1.02		(1.00,1.04)	0.01	0.037*
Say in government	1.01		(0.99,1.03)	0.01	0.298	1.00		(0.99,1.02)	0.01	0.693
Belonging in country		0.07	(0.03,0.11)	0.02	1.41e-03**		0.04	(0.00,0.08)	0.02	0.049*
City/place satisfaction	1.00		(0.99,1.02)	0.01	0.767	1.00		(0.98,1.01)	0.01	0.717
Trust within country	1.03		(1.01,1.05)	0.01	0.005*	1.02		(1.00,1.04)	0.01	0.016*
Social Participation										
Ever been married	1.00		(0.99,1.02)	0.01	0.601	1.00		(0.99,1.02)	0.01	0.589
Currently divorced	1.00		(0.99,1.01)	0.00	0.978	1.00		(0.99,1.01)	0.01	0.845
Number of children		0.00	(-0.03,0.03)	0.02	0.990		0.01	(-0.03,0.04)	0.02	0.747
Weekly+ community participation	1.01		(0.99,1.03)	0.01	0.267	1.01		(0.99,1.02)	0.01	0.529
Weekly+ religious attendance	1.00		(0.99,1.01)	0.01	0.837	0.99		(0.98,1.01)	0.01	0.377
Social Distress										
Loneliness		-0.03	(-0.07,0.01)	0.02	0.129		-0.01	(-0.05,0.02)	0.02	0.470
Perceived discrimination	1.00		(0.99,1.02)	0.01	0.702	1.00		(0.99,1.02)	0.01	0.780
Character & Prosocial Behavior										
Orientation to promote good (d)		0.09	(0.04,0.14)	0.02	2.01e-04***		0.05	(0.01,0.10)	0.02	0.016*
Delayed gratification (d)		0.08	(0.03,0.13)	0.02	1.05e-03**		0.05	(0.01,0.10)	0.02	0.032*
Hope		0.08	(0.04,0.12)	0.02	3.09e-04***		0.04	(-0.01,0.08)	0.02	0.095
Gratitude		0.10	(0.05,0.15)	0.02	5.88e-05***		0.06	(0.01,0.10)	0.02	0.013*
Showing love/care		0.09	(0.04,0.13)	0.02	1.15e-04***		0.05	(0.01,0.09)	0.02	0.023*
Forgivingness	1.17		(1.15,1.20)	0.01	2.22e-16***	1.17		(1.14,1.19)	0.01	2.22e-16***
Charitable giving	1.02		(1.00,1.04)	0.01	0.095	1.00		(0.98,1.02)	0.01	0.729
Helping strangers	1.05		(1.03,1.07)	0.01	5.11e-06***	1.03		(1.01,1.05)	0.01	4.75e-03**
Volunteering	1.02		(1.00,1.03)	0.01	0.038*	1.00		(0.99,1.02)	0.01	0.591
Physical Health & Health Behavior										
Self-rated physical health (b)		0.03	(-0.01,0.07)	0.02	0.165		0.02	(-0.02,0.06)	0.02	0.350
Health problems	1.00		(0.99,1.02)	0.01	0.680	1.00		(0.98,1.01)	0.01	0.678
Pain in past 4 weeks	1.00		(0.98,1.02)	0.01	0.740	0.99		(0.97,1.01)	0.01	0.328
Daily smoker	1.00		(0.98,1.02)	0.01	0.711	0.99		(0.97,1.02)	0.01	0.616
Number of drinks per week		-0.00	(-0.05,0.05)	0.02	0.997		0.00	(-0.05,0.05)	0.02	0.918
Days exercise per week		-0.00	(-0.05,0.04)	0.02	0.843		-0.02	(-0.07,0.02)	0.02	0.334
Socioeconomic Outcomes										
Financial security (f)		-0.01	(-0.05,0.04)	0.02	0.717		0.00	(-0.04,0.05)	0.02	0.858
Material security (f)		-0.01	(-0.05,0.03)	0.02	0.738		0.01	(-0.03,0.05)	0.02	0.763
Educational attainment (16+ years)	0.99		(0.98,1.00)	0.00	0.124	0.99		(0.98,1.00)	0.00	0.115
Currently employed	0.99		(0.98,1.01)	0.01	0.453	0.99		(0.98,1.01)	0.01	0.490
Financially comfortable/getting by	0.99		(0.97,1.01)	0.01	0.267	0.99		(0.97,1.01)	0.01	0.519
Own home	1.00		(0.98,1.02)	0.01	0.662	1.00		(0.98,1.02)	0.01	0.804
Income – top quintile	1.00		(0.98,1.02)	0.01	0.820	1.00		(0.98,1.02)	0.01	0.858
Religion & Spirituality										
Religious/spiritual connection	1.03		(1.01,1.05)	0.01	0.011*	1.01		(0.99,1.03)	0.01	0.525
Belief in life after death	1.02		(1.00,1.04)	0.01	0.039*	1.01		(0.99,1.03)	0.01	0.493
Transformative religious experience	1.01		(1.00,1.03)	0.01	0.123	1.00		(0.99,1.02)	0.01	0.915
Religious reading or listening	1.01		(1.00,1.02)	0.01	0.106	1.00		(0.99,1.01)	0.00	0.572
Prayer or meditation	1.02		(1.00,1.03)	0.01	0.022*	1.00		(0.99,1.02)	0.01	0.656
Belief in God/gods/spiritual forces	1.02		(1.00,1.04)	0.01	0.069	1.00		(0.99,1.02)	0.01	0.747
Religious centrality	1.00		(0.99,1.02)	0.01	0.594	0.99		(0.97,1.01)	0.01	0.184
Religious/spiritual comfort	1.01		(0.99,1.02)	0.01	0.551	0.99		(0.97,1.00)	0.01	0.083
Feel loved by God	1.02		(1.00,1.04)	0.01	0.027*	1.00		(0.98,1.01)	0.01	0.826
Feel punished by God	1.00		(0.98,1.01)	0.01	0.618	0.99		(0.98,1.01)	0.01	0.272
Experienced religious criticism	1.00		(0.99,1.01)	0.01	0.429	1.00		(0.99,1.01)	0.01	0.736
Faith-sharing	1.01		(0.99,1.03)	0.01	0.302	1.00		(0.98,1.01)	0.01	0.567

Notes. N=6290; Reference for focal predictor: never/rarely. RR, risk-ratio, null effect is 1.00; ES, effect size measure for standardized regression coefficient, null effect is 0.00; SE, standard error, the SE reported for binary/Likert-type outcomes where risk-ratios are on the log(RR) scale; CI, confidence interval; p-value, a Wald-type test of the null hypothesis that the effect of the focal predictor is zero; (a) item part of the Happiness & Life Satisfaction domain of the Secure Flourishing Index; (b) item part of the Physical & Mental Health domain of the Secure Flourishing Index; (c) item part of the Meaning & Purpose domain of the Secure Flourishing Index; (d) item part of the Character & Virtue domain of the Secure Flourishing Index; (e) item part of the Subjective Social Connectedness domain of the Secure Flourishing Index; (f) item part of the Financial & Material Security domain of the Secure Flourishing Index.

Multiple imputation was performed to impute missing data on the covariates, exposure, and outcomes. All models controlled for sociodemographic and childhood factors assessed at Wave 1. For Model 2 with PC (principal components), the first seven principal components of the entire set of contemporaneous confounders assessed at Wave 1 were included as additional covariates of the outcomes at Wave 2.

An outcome-wide analytic approach was used, and a separate model was run for each outcome. A different type of model was run depending on the nature of the outcome: (1) for each binary outcome, a weighted generalized linear model (with a log link and Poisson distribution) was used to estimate an RR; and (2) for each continuous outcome, a weighted linear regression model was used to estimate a ES. All effect sizes were standardized. For continuous outcomes, the ES represents the change in SD on the outcome between the lower and upper categories of the binary focal predictor. For binary outcomes, the RR represents the change in risk of being in the upper category compared to the lower category between the lower and upper categories of the binary focal predictor.

P-value significance thresholds: $p < 0.05$*, $p < 0.005$**, (Bonferroni) $p < 6.41e-04$***, correction for multiple testing using Bonferroni adjusted significant threshold.

Table S26h. Associations of forgivingness with adult well-being and other outcomes at Wave 2 in Spain using complete-case analyses with attrition weights.

	Model 1: Demographic and Childhood Variables as Covariates					Model 2: Demographic, Childhood, and Other Wave 1 Confounding Variables (Via Principal Components) as Covariates				
Outcome	RR	ES	95% CI	SE	p-value	RR	ES	95% CI	SE	p-value
Human Flourishing										
Secure flourishing index		0.06	(0.02,0.11)	0.02	0.009*		0.02	(-0.01,0.06)	0.02	0.220
Flourishing index		0.08	(0.03,0.13)	0.02	1.58e-03**		0.03	(-0.01,0.06)	0.02	0.190
Happiness & life satisfaction		0.02	(-0.02,0.07)	0.02	0.296		-0.01	(-0.05,0.03)	0.02	0.558
Physical & mental health		0.03	(-0.02,0.08)	0.02	0.199		0.00	(-0.03,0.04)	0.02	0.836
Meaning & purpose		0.06	(0.02,0.11)	0.02	0.008*		0.01	(-0.03,0.06)	0.02	0.478
Character & virtue		0.12	(0.07,0.18)	0.03	1.15e-05***		0.08	(0.03,0.13)	0.03	2.31e-03**
Close social relationships		0.07	(0.02,0.11)	0.02	0.007*		0.02	(-0.02,0.07)	0.02	0.312
Financial & material security		-0.00	(-0.05,0.04)	0.02	0.836		0.01	(-0.03,0.05)	0.02	0.719
Psychological Well-Being										
Happiness (a)		0.03	(-0.01,0.08)	0.02	0.155		-0.00	(-0.04,0.04)	0.02	0.884
Life satisfaction (a)		0.01	(-0.03,0.06)	0.02	0.558		-0.02	(-0.06,0.02)	0.02	0.370
Current life evaluation		0.01	(-0.03,0.06)	0.02	0.582		-0.02	(-0.06,0.03)	0.02	0.481
Future life evaluation		0.05	(0.00,0.10)	0.02	0.036*		0.02	(-0.02,0.06)	0.02	0.286
Optimism		0.08	(0.03,0.13)	0.02	1.12e-03**		0.03	(-0.01,0.07)	0.02	0.171
Freedom to pursue what's important		-0.00	(-0.05,0.05)	0.02	0.961		-0.04	(-0.08,0.01)	0.02	0.099
Inner peace	1.00		(0.98,1.03)	0.01	0.821	1.00		(0.97,1.02)	0.01	0.851
Life balance	1.02		(1.00,1.04)	0.01	0.078	1.01		(0.99,1.03)	0.01	0.483
Sense of mastery	1.00		(0.99,1.02)	0.01	0.563	0.99		(0.98,1.01)	0.01	0.475
Meaningful activities (c)		0.03	(-0.01,0.08)	0.02	0.180		-0.01	(-0.05,0.03)	0.02	0.590
Understanding purpose (c)		0.08	(0.03,0.13)	0.02	9.7e-04**		0.04	(-0.01,0.08)	0.02	0.099
Self-rated mental health (b)		0.03	(-0.01,0.08)	0.02	0.149		0.00	(-0.03,0.04)	0.02	0.816
Psychological Distress										
Traumatic distress	1.01		(0.99,1.03)	0.01	0.419	1.00		(0.98,1.03)	0.01	0.690
Depression symptoms composite	0.99		(0.97,1.02)	0.01	0.614	1.00		(0.98,1.02)	0.01	0.918
Depression – feel hopeless	1.00		(0.98,1.03)	0.01	0.658	1.01		(0.99,1.03)	0.01	0.403
Depression – loss of interest	0.99		(0.97,1.02)	0.01	0.636	1.00		(0.98,1.02)	0.01	0.935
Anxiety symptoms composite	0.99		(0.97,1.01)	0.01	0.375	0.99		(0.97,1.01)	0.01	0.441
Anxiety – feel on edge	1.01		(0.98,1.03)	0.01	0.572	1.01		(0.99,1.03)	0.01	0.429
Anxiety – cannot stop worrying	1.00		(0.97,1.02)	0.01	0.661	1.00		(0.98,1.02)	0.01	0.888
Suffering	1.00		(0.98,1.03)	0.01	0.717	1.00		(0.98,1.02)	0.01	0.913
Social Well-Being										
Relationship contentment (e)		0.06	(0.01,0.11)	0.02	0.009*		0.02	(-0.02,0.06)	0.02	0.340
Relationship satisfaction (e)		0.06	(0.01,0.11)	0.03	0.015*		0.02	(-0.03,0.07)	0.02	0.371
Social support		0.08	(0.03,0.13)	0.03	1.55e-03**		0.05	(0.00,0.10)	0.02	0.043*
Intimate/close friend	1.02		(1.00,1.04)	0.01	0.027*	1.01		(1.00,1.03)	0.01	0.116
Government approval	1.03		(1.01,1.05)	0.01	3.6e-03**	1.02		(1.00,1.04)	0.01	0.046*
Say in government	1.01		(1.00,1.03)	0.01	0.125	1.01		(0.99,1.02)	0.01	0.470
Belonging in country		0.08	(0.03,0.12)	0.02	9.99e-04**		0.04	(-0.00,0.09)	0.02	0.051
City/place satisfaction	1.00		(0.98,1.02)	0.01	0.991	0.99		(0.98,1.01)	0.01	0.407
Trust within country	1.03		(1.01,1.05)	0.01	0.012*	1.03		(1.00,1.05)	0.01	0.032*
Social Participation										
Ever been married	1.01		(0.99,1.03)	0.01	0.323	1.01		(0.99,1.03)	0.01	0.320
Currently divorced	1.00		(0.99,1.01)	0.01	0.752	1.00		(0.99,1.01)	0.01	0.719
Number of children		0.02	(-0.02,0.06)	0.02	0.241		0.01	(-0.03,0.05)	0.02	0.554
Weekly+ community participation	1.01		(0.99,1.03)	0.01	0.288	1.00		(0.98,1.02)	0.01	0.720
Weekly+ religious attendance	1.00		(0.99,1.01)	0.01	0.935	1.00		(0.98,1.01)	0.01	0.457
Social Distress										
Loneliness		-0.03	(-0.07,0.02)	0.02	0.226		-0.01	(-0.05,0.03)	0.02	0.797
Perceived discrimination	1.00		(0.99,1.02)	0.01	0.595	1.00		(0.98,1.02)	0.01	0.851
Character & Prosocial Behavior										
Orientation to promote good (d)		0.10	(0.05,0.15)	0.03	2.01e-04***		0.06	(0.01,0.10)	0.02	0.022*
Delayed gratification (d)		0.10	(0.05,0.16)	0.03	1.36e-04***		0.07	(0.02,0.13)	0.03	4.91e-03**
Hope		0.08	(0.03,0.12)	0.02	9.4e-04**		0.03	(-0.02,0.07)	0.02	0.232
Gratitude		0.09	(0.05,0.14)	0.02	4.03e-05***		0.04	(0.00,0.08)	0.02	0.038*
Showing love/care		0.08	(0.04,0.13)	0.02	6.82e-04**		0.04	(-0.00,0.08)	0.02	0.081
Forgivingness	1.18		(1.16,1.21)	0.01	2.22e-16***	1.17		(1.15,1.20)	0.01	2.22e-16***
Charitable giving	1.02		(1.00,1.04)	0.01	0.050	1.01		(0.99,1.03)	0.01	0.255
Helping strangers	1.06		(1.03,1.08)	0.01	1.32e-06***	1.04		(1.02,1.07)	0.01	9.31e-05***
Volunteering	1.01		(0.99,1.03)	0.01	0.190	1.00		(0.99,1.02)	0.01	0.766
Physical Health & Health Behavior										
Self-rated physical health (b)		0.02	(-0.03,0.07)	0.02	0.395		0.00	(-0.04,0.04)	0.02	0.901
Health problems	1.01		(0.99,1.03)	0.01	0.387	1.00		(0.98,1.02)	0.01	0.984
Pain in past 4 weeks	0.99		(0.96,1.01)	0.01	0.209	0.98		(0.96,1.00)	0.01	0.055
Daily smoker	1.01		(0.99,1.03)	0.01	0.265	1.01		(0.98,1.03)	0.01	0.578
Number of drinks per week		0.04	(-0.01,0.08)	0.02	0.094		0.04	(-0.01,0.08)	0.02	0.088
Days exercise per week		-0.02	(-0.07,0.03)	0.03	0.394		-0.04	(-0.09,0.01)	0.03	0.131
Socioeconomic Outcomes										
Financial security (f)		-0.00	(-0.05,0.04)	0.02	0.873		0.01	(-0.04,0.05)	0.02	0.768
Material security (f)		-0.00	(-0.05,0.04)	0.02	0.827		0.01	(-0.03,0.05)	0.02	0.723
Educational attainment (16+ years)	1.00		(0.99,1.00)	0.00	0.284	1.00		(0.99,1.00)	0.00	0.280
Currently employed	0.99		(0.97,1.00)	0.01	0.078	0.99		(0.97,1.00)	0.01	0.137
Financially comfortable/getting by	0.98		(0.96,1.00)	0.01	0.054	0.99		(0.97,1.01)	0.01	0.194
Own home	1.01		(0.99,1.03)	0.01	0.457	1.01		(0.99,1.04)	0.01	0.310
Income – top quintile	1.00		(0.98,1.02)	0.01	0.795	1.00		(0.98,1.02)	0.01	0.893
Religion & Spirituality										
Religious/spiritual connection	1.03		(1.01,1.05)	0.01	0.009*	1.01		(0.99,1.03)	0.01	0.363
Belief in life after death	1.02		(1.00,1.04)	0.01	0.036*	1.01		(0.99,1.03)	0.01	0.441
Transformative religious experience	1.01		(1.00,1.03)	0.01	0.170	1.00		(0.99,1.02)	0.01	0.929
Religious reading or listening	1.01		(1.00,1.02)	0.00	0.043*	1.01		(1.00,1.01)	0.00	0.269
Prayer or meditation	1.02		(1.00,1.04)	0.01	0.022*	1.01		(0.99,1.02)	0.01	0.473
Belief in God/gods/spiritual forces	1.03		(1.00,1.05)	0.01	0.016*	1.01		(0.99,1.03)	0.01	0.248
Religious centrality	1.00		(0.98,1.02)	0.01	0.858	0.99		(0.97,1.00)	0.01	0.108
Religious/spiritual comfort	1.00		(0.98,1.02)	0.01	0.896	0.98		(0.96,1.00)	0.01	0.039*
Feel loved by God	1.02		(1.00,1.04)	0.01	0.082	1.00		(0.98,1.02)	0.01	0.832
Feel punished by God	1.00		(0.99,1.01)	0.01	0.942	1.00		(0.98,1.01)	0.01	0.491
Experienced religious criticism	1.01		(1.00,1.02)	0.01	0.101	1.00		(0.99,1.01)	0.01	0.435
Faith-sharing	1.02		(1.00,1.03)	0.01	0.111	1.00		(0.99,1.02)	0.01	0.762

Table S26h. Associations of forgivingness with adult well-being and other outcomes at Wave 2 in Spain using complete-case analyses with attrition weights.

	Model 1: Demographic and Childhood Variables as Covariates					Model 2: Demographic, Childhood, and Other Wave 1 Confounding Variables (Via Principal Components) as Covariates				
Outcome	RR	ES	95% CI	SE	p-value	RR	ES	95% CI	SE	p-value

Notes. N=2924; Reference for focal predictor: never/rarely. RR, risk-ratio, null effect is 1.00; ES, effect size measure for standardized regression coefficient, null effect is 0.00; SE, standard error, the SE reported for binary/Likert-type outcomes where risk-ratios are on the log(RR) scale; CI, confidence interval; p-value, a Wald-type test of the null hypothesis that the effect of the focal predictor is zero; (a) item part of the Happiness & Life Satisfaction domain of the Secure Flourishing Index; (b) item part of the Physical & Mental Health domain of the Secure Flourishing Index; (c) item part of the Meaning & Purpose domain of the Secure Flourishing Index; (d) item part of the Character & Virtue domain of the Secure Flourishing Index; (e) item part of the Subjective Social Connectedness domain of the Secure Flourishing Index; (f) item part of the Financial & Material Security domain of the Secure Flourishing Index.

Attrition weights were computed to adjust the complete case data (those who responded at Wave 2 to at least 50% of the questions) and multiple imputation was used to impute missing data on all remaining within wave on the covariates, exposure, and outcomes. All models controlled for sociodemographic and childhood factors assessed at Wave 1. For Model 2 with PC (principal components), the first seven principal components of the entire set of contemporaneous confounders assessed at Wave 1 were included as additional covariates of the outcomes at Wave 2.

An outcome-wide analytic approach was used, and a separate model was run for each outcome. A different type of model was run depending on the nature of the outcome: (1) for each binary outcome, a weighted generalized linear model (with a log link and Poisson distribution) was used to estimate an RR; and (2) for each continuous outcome, a weighted linear regression model was used to estimate a ES. All effect sizes were standardized. For continuous outcomes, the ES represents the change in SD on the outcome between the lower and upper categories of the binary focal predictor. For binary outcomes, the RR represents the change in risk of being in the upper category compared to the lower category between the lower and upper categories of the binary focal predictor.

P-value significance thresholds: $p < 0.05$*, $p < 0.005$**, (Bonferroni) $p < 6.41e-04$***, correction for multiple testing using Bonferroni adjusted significant threshold.

Table S26i. Sensitivity analysis of forgivingness outcome-wide results to unmeasured confounding using E-values in Spain

	Multiple Imputation				Complete Case w/ Attrition Weights			
	Model 1: Demographics and Childhood Variables as Covariates		Model 2: Demographics, Childhood, and Other Wave 1 Confounders (Via Principal Components) as Covariates		Model 1: Demographics and Childhood Variables as Covariates		Model 2: Demographics, Childhood, and Other Wave 1 Confounders (Via Principal Components) as Covariates	
Outcome	EE	ECI	EE	ECI	EE	ECI	EE	ECI
Human Flourishing								
Secure flourishing index	1.54	1.29	1.31	1.00	1.57	1.23	1.30	1.00
Flourishing index	1.63	1.40	1.33	1.06	1.66	1.34	1.31	1.00
Happiness & life satisfaction	1.28	1.00	1.11	1.00	1.30	1.00	1.19	1.00
Physical & mental health	1.37	1.00	1.21	1.00	1.35	1.00	1.11	1.00
Meaning & purpose	1.59	1.31	1.31	1.00	1.58	1.24	1.22	1.00
Character & virtue	1.84	1.57	1.59	1.31	1.96	1.60	1.68	1.33
Close social relationships	1.50	1.21	1.22	1.00	1.59	1.25	1.28	1.00
Financial & material security	1.16	1.00	1.13	1.00	1.11	1.00	1.15	1.00
Psychological Well-Being								
Happiness	1.36	1.00	1.15	1.00	1.37	1.00	1.09	1.00
Life satisfaction	1.14	1.00	1.23	1.00	1.21	1.00	1.25	1.00
Current life evaluation	1.20	1.00	1.13	1.00	1.20	1.00	1.22	1.00
Future life evaluation	1.48	1.18	1.32	1.00	1.48	1.10	1.28	1.00
Optimism	1.63	1.36	1.36	1.00	1.67	1.35	1.34	1.00
Freedom to pursue what's important	1.07	1.00	1.33	1.00	1.05	1.00	1.39	1.00
Inner peace	1.08	1.00	1.03	1.00	1.09	1.00	1.08	1.00
Life balance	1.25	1.00	1.17	1.00	1.26	1.00	1.15	1.00
Sense of mastery	1.11	1.00	1.10	1.00	1.12	1.00	1.13	1.00
Meaningful activities	1.34	1.00	1.09	1.00	1.36	1.00	1.19	1.00
Understanding purpose	1.68	1.41	1.44	1.12	1.68	1.36	1.38	1.00
Self-rated mental health	1.32	1.00	1.13	1.00	1.36	1.00	1.11	1.00
Psychological Distress								
Traumatic distress	1.15	1.00	1.08	1.00	1.18	1.00	1.12	1.00
Depression symptoms composite	1.12	1.00	1.05	1.00	1.13	1.00	1.05	1.00
Depression – feel hopeless	1.07	1.00	1.12	1.00	1.12	1.00	1.16	1.00
Depression – loss of interest	1.11	1.00	1.04	1.00	1.12	1.00	1.05	1.00
Anxiety symptoms composite	1.11	1.00	1.09	1.00	1.18	1.00	1.16	1.00
Anxiety – feel on edge	1.15	1.00	1.16	1.00	1.14	1.00	1.16	1.00
Anxiety – cannot stop worrying	1.10	1.00	1.07	1.00	1.12	1.00	1.06	1.00
Suffering	1.20	1.00	1.14	1.00	1.11	1.00	1.06	1.00
Social Well-Being								
Relationship contentment	1.43	1.00	1.16	1.00	1.55	1.22	1.26	1.00
Relationship satisfaction	1.48	1.15	1.23	1.00	1.57	1.20	1.28	1.00
Social support	1.65	1.37	1.50	1.20	1.69	1.35	1.48	1.06
Intimate/close friend	1.28	1.12	1.23	1.00	1.28	1.08	1.23	1.00
Government approval	1.34	1.16	1.29	1.06	1.38	1.19	1.29	1.03
Say in government	1.17	1.00	1.09	1.00	1.21	1.00	1.13	1.00
Belonging in country	1.61	1.32	1.42	1.04	1.66	1.35	1.44	1.00
City/place satisfaction	1.09	1.00	1.09	1.00	1.02	1.00	1.15	1.00
Trust within country	1.33	1.16	1.30	1.11	1.36	1.14	1.32	1.08
Social Participation								
Ever been married	1.10	1.00	1.11	1.00	1.17	1.00	1.17	1.00
Currently divorced	1.02	1.00	1.05	1.00	1.07	1.00	1.08	1.00
Number of children	1.02	1.00	1.12	1.00	1.29	1.00	1.20	1.00
Weekly+ community participation	1.17	1.00	1.12	1.00	1.19	1.00	1.10	1.00
Weekly+ religious attendance	1.06	1.00	1.13	1.00	1.04	1.00	1.12	1.00
Social Distress								
Loneliness	1.34	1.00	1.21	1.00	1.33	1.00	1.12	1.00
Perceived discrimination	1.09	1.00	1.08	1.00	1.12	1.00	1.07	1.00
Character & Prosocial Behavior								
Orientation to promote good	1.73	1.43	1.50	1.18	1.79	1.45	1.52	1.15
Delayed gratification	1.67	1.37	1.49	1.12	1.83	1.48	1.64	1.29
Hope	1.66	1.38	1.37	1.00	1.66	1.35	1.31	1.00
Gratitude	1.80	1.51	1.53	1.21	1.78	1.48	1.44	1.08
Showing love/care	1.72	1.44	1.47	1.15	1.70	1.38	1.41	1.00
Forgivingness	2.29	2.14	2.25	2.10	2.37	2.20	2.32	2.15
Charitable giving	1.25	1.00	1.10	1.00	1.29	1.00	1.20	1.00
Helping strangers	1.49	1.34	1.35	1.17	1.55	1.38	1.47	1.29
Volunteering	1.23	1.05	1.10	1.00	1.20	1.00	1.08	1.00
Physical Health & Health Behavior								
Self-rated physical health	1.34	1.00	1.25	1.00	1.26	1.00	1.08	1.00
Health problems	1.10	1.00	1.10	1.00	1.17	1.00	1.02	1.00
Pain in past 4 weeks	1.10	1.00	1.19	1.00	1.23	1.00	1.30	1.00
Daily smoker	1.11	1.00	1.13	1.00	1.21	1.00	1.14	1.00
Number of drinks per week	1.01	1.00	1.08	1.00	1.38	1.00	1.39	1.00
Days exercise per week	1.11	1.00	1.28	1.00	1.28	1.00	1.40	1.00
Socioeconomic Outcomes								
Financial security	1.15	1.00	1.10	1.00	1.10	1.00	1.14	1.00
Material security	1.14	1.00	1.13	1.00	1.12	1.00	1.15	1.00
Educational attainment (16+ years)	1.15	1.00	1.15	1.00	1.12	1.00	1.12	1.00
Currently employed	1.13	1.00	1.13	1.00	1.21	1.00	1.20	1.00
Financially comfortable/getting by	1.20	1.00	1.15	1.00	1.28	1.00	1.22	1.00
Own home	1.12	1.00	1.09	1.00	1.17	1.00	1.20	1.00
Income – top quintile	1.08	1.00	1.07	1.00	1.09	1.00	1.07	1.00
Religion & Spirituality								
Religious/spiritual connection	1.32	1.14	1.14	1.00	1.34	1.15	1.17	1.00
Belief in life after death	1.30	1.06	1.15	1.00	1.30	1.06	1.16	1.00
Transformative religious experience	1.20	1.00	1.05	1.00	1.20	1.00	1.04	1.00
Religious reading or listening	1.17	1.00	1.09	1.00	1.18	1.03	1.13	1.00
Prayer or meditation	1.25	1.08	1.09	1.00	1.27	1.09	1.13	1.00
Belief in God/gods/spiritual forces	1.25	1.00	1.09	1.00	1.32	1.12	1.20	1.00
Religious centrality	1.12	1.00	1.19	1.00	1.07	1.00	1.22	1.00
Religious/spiritual comfort	1.13	1.00	1.23	1.00	1.06	1.00	1.27	1.05
Feel loved by God	1.26	1.08	1.07	1.00	1.25	1.00	1.07	1.00
Feel punished by God	1.09	1.00	1.14	1.00	1.03	1.00	1.11	1.00
Experienced religious criticism	1.11	1.00	1.07	1.00	1.17	1.00	1.11	1.00
Faith-sharing	1.17	1.00	1.12	1.00	1.23	1.00	1.09	1.00

Notes. EE, E-value for estimate; ECI, E-value for the limit of the confidence interval. The formula for calculating E-values can be found in VanderWeele and Ding (2017). E-values for estimate are the minimum strength of association on the risk ratio scale that an unmeasured confounder would need to have with both the exposure and the outcome to fully explain away the observed association between the exposure and outcome, conditional on the measured covariates. E-values for the 95% CI closest to the null denote the minimum strength of association on the risk ratio scale that an unmeasured confounder would need to have with both the exposure and the outcome to shift the CI to include the null value, conditional on the measured covariates.

Table S27a. Weighted summary statistics for demographic and childhood variables in Sweden

**Characteristic**	**Wave 1** N = 15,068	**Wave 2** N = 11,611
Forgivingness, n (%)		
Always	2,001 (13.3%)	1,475 (12.7%)
Often	9,424 (62.5%)	7,282 (62.7%)
Rarely	3,260 (21.6%)	2,520 (21.7%)
Never	281 (1.9%)	238 (2.0%)
(Missing)	101 (0.7%)	97 (0.8%)
Year of birth, n (%)		
1943 or earlier (current age: 80+ years)	589 (3.9%)	542 (4.7%)
1943-1953 (current age: 70-79 years)	2,153 (14.3%)	1,682 (14.5%)
1953-1963 (current age: 60-69 years)	2,064 (13.7%)	1,590 (13.7%)
1963-1973 (current age: 50-59 years)	2,467 (16.4%)	1,906 (16.4%)
1973-1983 (current age: 40-49 years)	2,276 (15.1%)	1,791 (15.4%)
1983-1993 (current age: 30-39 years)	2,455 (16.3%)	1,893 (16.3%)
1993-1998 (current age: 25-29 years)	1,432 (9.5%)	1,050 (9.0%)
1998-2005 (current age: 18-24 years)	1,632 (10.8%)	1,157 (10.0%)
(Missing)	0 (0%)	0 (0%)
Age of participant		
Mean	48.6	49.3
Standard Deviation	18.7	18.7
Min, Max	18.0, 98.0	18.0, 98.0
Gender, n (%)		
Male	7,494 (49.7%)	5,721 (49.3%)
Female	7,533 (50.0%)	5,858 (50.4%)
Other	28 (0.2%)	22 (0.2%)
(Missing)	12 (0.1%)	11 (0.1%)
Respondent marital status, n (%)		
Single/Never been married	3,962 (26.3%)	2,909 (25.1%)
Married	6,247 (41.5%)	4,847 (41.7%)
Separated	429 (2.8%)	369 (3.2%)
Divorced	794 (5.3%)	594 (5.1%)
Widowed	421 (2.8%)	365 (3.1%)
Domestic partner	3,140 (20.8%)	2,477 (21.3%)
(Missing)	75 (0.5%)	51 (0.4%)
Education (years), n (%)		
Up to 8	265 (1.8%)	148 (1.3%)
9-15	11,048 (73.3%)	8,302 (71.5%)
16+	3,755 (24.9%)	3,161 (27.2%)
(Missing)	0 (0%)	0 (0%)
Employment status, n (%)		
Employed for an employer	7,972 (52.9%)	6,138 (52.9%)
Self-employed	1,193 (7.9%)	827 (7.1%)
Retired	3,696 (24.5%)	2,980 (25.7%)
Student	1,419 (9.4%)	952 (8.2%)
Homemaker	77 (0.5%)	47 (0.4%)
Unemployed and looking for a job	343 (2.3%)	309 (2.7%)
None of these/Other	351 (2.3%)	324 (2.8%)
(Missing)	18 (0.1%)	34 (0.3%)
Current religious service attendance, n (%)		
More than once a week	234 (1.6%)	204 (1.8%)
Once a week	429 (2.8%)	311 (2.7%)

Table S27a. Weighted summary statistics for demographic and childhood variables in Sweden

**Characteristic**	**Wave 1**<br>N = 15,068	**Wave 2**<br>N = 11,611
One to three times a month	481 (3.2%)	384 (3.3%)
A few times a year	3,868 (25.7%)	2,726 (23.5%)
Never	10,010 (66.4%)	7,948 (68.5%)
(Missing)	45 (0.3%)	38 (0.3%)
Immigration status, n (%)		
Born in this country	13,926 (92.4%)	10,730 (92.4%)
Born in another country	1,049 (7.0%)	814 (7.0%)
(Missing)	92 (0.6%)	67 (0.6%)
Parental marital status around age 12, n (%)		
Parents were married	10,781 (71.6%)	8,345 (71.9%)
Parents were divorced	1,968 (13.1%)	1,466 (12.6%)
Parents were never married	1,814 (12.0%)	1,429 (12.3%)
One or both of them had died	358 (2.4%)	264 (2.3%)
Unsure	92 (0.6%)	63 (0.5%)
(Missing)	55 (0.4%)	44 (0.4%)
Religious service attendance around age 12, n (%)		
At least once a week	945 (6.3%)	731 (6.3%)
One to three times a month	1,340 (8.9%)	1,059 (9.1%)
Less than once a month	6,150 (40.8%)	4,701 (40.5%)
Never	6,581 (43.7%)	5,080 (43.8%)
(Missing)	52 (0.3%)	41 (0.4%)
Relationship with mother when growing up, n (%)		
Very good	8,719 (57.9%)	6,701 (57.7%)
Somewhat good	4,520 (30.0%)	3,514 (30.3%)
Somewhat bad	1,204 (8.0%)	930 (8.0%)
Very bad	379 (2.5%)	288 (2.5%)
(Does not apply)	217 (1.4%)	161 (1.4%)
(Missing)	29 (0.2%)	17 (0.1%)
Relationship with father when growing up, n (%)		
Very good	7,095 (47.1%)	5,395 (46.5%)
Somewhat good	4,888 (32.4%)	3,836 (33.0%)
Somewhat bad	1,609 (10.7%)	1,274 (11.0%)
Very bad	741 (4.9%)	559 (4.8%)
(Does not apply)	720 (4.8%)	536 (4.6%)
(Missing)	15 (0.1%)	11 (0.1%)
Felt like an outsider in family when growing up, n (%)		
Yes	1,899 (12.6%)	1,478 (12.7%)
No	13,000 (86.3%)	9,998 (86.1%)
(Missing)	169 (1.1%)	135 (1.2%)
Experienced abuse when growing up, n (%)		
Yes	2,308 (15.3%)	1,797 (15.5%)
No	12,716 (84.4%)	9,780 (84.2%)
(Missing)	44 (0.3%)	34 (0.3%)
Self-rated health when growing up, n (%)		
Excellent	5,652 (37.5%)	4,319 (37.2%)
Very good	5,132 (34.1%)	3,942 (34.0%)
Good	2,706 (18.0%)	2,109 (18.2%)
Fair	1,131 (7.5%)	890 (7.7%)
Poor	411 (2.7%)	329 (2.8%)
(Missing)	37 (0.2%)	23 (0.2%)

Table S27a. Weighted summary statistics for demographic and childhood variables in Sweden

**Characteristic**	**Wave 1**<br>N = 15,068	**Wave 2**<br>N = 11,611
Subjective financial status of family growing up, n (%)		
Lived comfortably	5,946 (39.5%)	4,590 (39.5%)
Got by	7,693 (51.1%)	5,942 (51.2%)
Found it difficult	1,262 (8.4%)	957 (8.2%)
Found it very difficult	147 (1.0%)	106 (0.9%)
(Missing)	21 (0.1%)	17 (0.1%)
Religious affiliation growing up, n (%)		
Christianity	10,495 (69.7%)	8,140 (70.1%)
Taoism	0 (0%)	0 (0%)
Confucianism	4 (0.0%)	1 (0.0%)
Primal, Animist, or Folk religion	31 (0.2%)	26 (0.2%)
Spiritism	0 (0%)	0 (0%)
Umbanda, Candomblé, and other African-derived religions	0 (0%)	0 (0%)
Chinese folk/traditional religion	0 (0%)	0 (0%)
Islam	459 (3.0%)	337 (2.9%)
Hinduism	16 (0.1%)	9 (0.1%)
Buddhism	41 (0.3%)	31 (0.3%)
Judaism	48 (0.3%)	40 (0.3%)
Sikhism	9 (0.1%)	4 (0.0%)
Baha'i	3 (0.0%)	3 (0.0%)
Jainism	0 (0%)	0 (0%)
Shinto	1 (<0.0%)	0 (0%)
Some other religion	68 (0.5%)	58 (0.5%)
No religion/Atheist/Agnostic	3,867 (25.7%)	2,943 (25.3%)
(Missing)	26 (0.2%)	20 (0.2%)

Note. N (%); this table is based on non-imputed data. Cumulative percentages for variables may not add up to 100% due to rounding. Wave 1 characteristics weighted using the Gallup provided sampling weight, ANNUAL_WEIGHT_R2; Wave 2 characteristics weighted accounting for attrition by using the adjusted Wave 1 weight, ANNUAL_WEIGHT_R2, multiplied by the created attrition weight to account for dropout, to maintain nationally representative estimates for Wave 2 characteristics.

Table S27b. Weighted summary statistics for outcome variables in Sweden

**Outcome**	**Wave 1**<br>N = 15,068	**Wave 2**<br>N = 11,611
Secure flourishing index		
Mean	7.1	7.1
Standard Deviation	1.5	1.5
Min, Max	0.0, 10.0	0.0, 10.0
(Missing)	508 (3.4%)	417 (3.6%)
Flourishing index		
Mean	7.0	7.1
Standard Deviation	1.6	1.5
Min, Max	0.0, 10.0	0.0, 10.0
(Missing)	457 (3.0%)	380 (3.3%)
Happiness & life satisfaction		
Mean	7.0	7.0
Standard Deviation	2.0	1.9
Min, Max	0.0, 10.0	0.0, 10.0
(Missing)	62 (0.4%)	47 (0.4%)
Physical & mental health		
Mean	6.8	6.7
Standard Deviation	1.9	1.8
Min, Max	0.0, 10.0	0.0, 10.0
(Missing)	48 (0.3%)	30 (0.3%)
Meaning & purpose		
Mean	6.8	6.9
Standard Deviation	2.2	2.1
Min, Max	0.0, 10.0	0.0, 10.0
(Missing)	162 (1.1%)	118 (1.0%)
Character & virtue		
Mean	7.3	7.4
Standard Deviation	1.6	1.5
Min, Max	0.0, 10.0	0.0, 10.0
(Missing)	170 (1.1%)	168 (1.4%)
Close social relationships		
Mean	7.2	7.3
Standard Deviation	2.2	2.2
Min, Max	0.0, 10.0	0.0, 10.0
(Missing)	97 (0.6%)	83 (0.7%)
Financial & material security		
Mean	7.3	7.4
Standard Deviation	2.5	2.5
Min, Max	0.0, 10.0	0.0, 10.0
(Missing)	65 (0.4%)	49 (0.4%)
Happiness		
Mean	7.0	7.0
Standard Deviation	2.0	1.9
Min, Max	0.0, 10.0	0.0, 10.0
(Missing)	38 (0.3%)	18 (0.2%)
Life satisfaction		
Mean	7.1	7.0
Standard Deviation	2.1	2.0
Min, Max	0.0, 10.0	0.0, 10.0
(Missing)	34 (0.2%)	31 (0.3%)
Current life evaluation		

Table S27b. Weighted summary statistics for outcome variables in Sweden

Outcome	Wave 1<br>N = 15,068	Wave 2<br>N = 11,611
Mean	7.2	6.7
Standard Deviation	1.8	1.7
Min, Max	0.0, 10.0	0.0, 10.0
(Missing)	46 (0.3%)	13 (0.1%)
Future life evaluation		
Mean	7.7	7.5
Standard Deviation	1.8	1.9
Min, Max	0.0, 10.0	0.0, 10.0
(Missing)	43 (0.3%)	49 (0.4%)
Optimism		
Mean	7.3	7.3
Standard Deviation	2.3	2.3
Min, Max	0.0, 10.0	0.0, 10.0
(Missing)	52 (0.3%)	46 (0.4%)
Freedom to pursue what's important		
Mean	7.5	7.4
Standard Deviation	2.3	2.3
Min, Max	0.0, 10.0	0.0, 10.0
(Missing)	39 (0.3%)	32 (0.3%)
Inner peace, n (%)		
Always	1,685 (11.2%)	1,163 (10.0%)
Often	10,514 (69.8%)	8,164 (70.3%)
Rarely	2,670 (17.7%)	2,122 (18.3%)
Never	141 (0.9%)	117 (1.0%)
(Missing)	59 (0.4%)	45 (0.4%)
Life balance, n (%)		
Always	1,256 (8.3%)	868 (7.5%)
Often	10,584 (70.2%)	8,163 (70.3%)
Rarely	2,964 (19.7%)	2,351 (20.2%)
Never	156 (1.0%)	157 (1.4%)
(Missing)	108 (0.7%)	72 (0.6%)
Sense of mastery, n (%)		
Always	2,310 (15.3%)	1,653 (14.2%)
Often	10,492 (69.6%)	8,014 (69.0%)
Rarely	2,046 (13.6%)	1,750 (15.1%)
Never	133 (0.9%)	108 (0.9%)
(Missing)	87 (0.6%)	86 (0.7%)
Meaningful activities		
Mean	7.0	7.0
Standard Deviation	2.2	2.2
Min, Max	0.0, 10.0	0.0, 10.0
(Missing)	44 (0.3%)	23 (0.2%)
Understanding purpose		
Mean	6.6	6.8
Standard Deviation	2.6	2.5
Min, Max	0.0, 10.0	0.0, 10.0
(Missing)	121 (0.8%)	99 (0.9%)
Self-rated mental health		
Mean	7.1	7.1
Standard Deviation	2.3	2.2
Min, Max	0.0, 10.0	0.0, 10.0

Table S27b. Weighted summary statistics for outcome variables in Sweden

Outcome	Wave 1<br>N = 15,068	Wave 2<br>N = 11,611
(Missing)	19 (0.1%)	17 (0.1%)
Traumatic distress, n (%)		
A lot	709 (4.7%)	572 (4.9%)
Some	3,072 (20.4%)	2,373 (20.4%)
Not very much	5,742 (38.1%)	4,402 (37.9%)
Not at all	5,379 (35.7%)	4,073 (35.1%)
(Missing)	166 (1.1%)	191 (1.6%)
Depression symptoms composite, n (%)	2,802 (18.8%)	2,611 (22.7%)
(Missing)	152 (1.0%)	93 (0.8%)
Depression – feel hopeless, n (%)		
Nearly every day	923 (6.1%)	710 (6.1%)
More than half the days	1,206 (8.0%)	979 (8.4%)
Several days	4,415 (29.3%)	3,592 (30.9%)
Not at all	8,460 (56.1%)	6,302 (54.3%)
(Missing)	64 (0.4%)	27 (0.2%)
Depression – loss of interest, n (%)		
Nearly every day	860 (5.7%)	809 (7.0%)
More than half the days	1,652 (11.0%)	1,718 (14.8%)
Several days	3,960 (26.3%)	3,198 (27.5%)
Not at all	8,493 (56.4%)	5,811 (50.0%)
(Missing)	103 (0.7%)	75 (0.6%)
Anxiety symptoms composite, n (%)	2,439 (16.3%)	1,950 (16.9%)
(Missing)	96 (0.6%)	84 (0.7%)
Anxiety – feel on edge, n (%)		
Nearly every day	978 (6.5%)	749 (6.5%)
More than half the days	1,313 (8.7%)	1,076 (9.3%)
Several days	5,416 (35.9%)	4,319 (37.2%)
Not at all	7,309 (48.5%)	5,418 (46.7%)
(Missing)	53 (0.3%)	49 (0.4%)
Anxiety – cannot stop worrying, n (%)		
Nearly every day	752 (5.0%)	576 (5.0%)
More than half the days	1,113 (7.4%)	956 (8.2%)
Several days	3,434 (22.8%)	2,808 (24.2%)
Not at all	9,711 (64.4%)	7,230 (62.3%)
(Missing)	58 (0.4%)	42 (0.4%)
Suffering, n (%)		
A lot	798 (5.3%)	644 (5.5%)
Some	4,423 (29.4%)	3,711 (32.0%)
Not very much	6,341 (42.1%)	4,926 (42.4%)
Not at all	3,437 (22.8%)	2,283 (19.7%)
(Missing)	68 (0.5%)	47 (0.4%)
Relationship contentment		
Mean	7.4	7.5
Standard Deviation	2.2	2.1
Min, Max	0.0, 10.0	0.0, 10.0
(Missing)	44 (0.3%)	47 (0.4%)
Relationship satisfaction		
Mean	7.0	7.1
Standard Deviation	2.4	2.4
Min, Max	0.0, 10.0	0.0, 10.0
(Missing)	65 (0.4%)	49 (0.4%)

Table S27b. Weighted summary statistics for outcome variables in Sweden

**Outcome**	**Wave 1** N = 15,068	**Wave 2** N = 11,611
Social support		
Mean	8.1	8.3
Standard Deviation	2.1	2.0
Min, Max	0.0, 10.0	0.0, 10.0
(Missing)	57 (0.4%)	22 (0.2%)
Intimate/close friend, n (%)		
Yes	13,013 (86.4%)	9,986 (86.0%)
No	2,019 (13.4%)	1,596 (13.7%)
(Missing)	37 (0.2%)	29 (0.2%)
Government approval, n (%)		
Strongly approve	745 (4.9%)	1,553 (13.4%)
Somewhat approve	2,808 (18.6%)	1,828 (15.7%)
Neither approve nor disapprove	4,292 (28.5%)	3,617 (31.2%)
Somewhat disapprove	3,999 (26.5%)	2,284 (19.7%)
Strongly disapprove	3,140 (20.8%)	2,234 (19.2%)
(Missing)	85 (0.6%)	95 (0.8%)
Say in government, n (%)		
Agree	3,260 (21.6%)	2,474 (21.3%)
Disagree	7,826 (51.9%)	6,258 (53.9%)
Unsure	3,909 (25.9%)	2,807 (24.2%)
(Missing)	74 (0.5%)	72 (0.6%)
Belonging in country		
Mean	8.6	8.5
Standard Deviation	2.0	2.0
Min, Max	0.0, 10.0	0.0, 10.0
(Missing)	56 (0.4%)	27 (0.2%)
City/place satisfaction, n (%)		
Satisfied	12,268 (81.4%)	9,433 (81.2%)
Dissatisfied	1,428 (9.5%)	1,053 (9.1%)
Unsure	1,311 (8.7%)	1,083 (9.3%)
(Missing)	61 (0.4%)	42 (0.4%)
Trust within country, n (%)		
All people	59 (0.4%)	36 (0.3%)
Most people	5,613 (37.2%)	4,286 (36.9%)
Some people	6,142 (40.8%)	4,806 (41.4%)
Not very many people	3,094 (20.5%)	2,334 (20.1%)
None	76 (0.5%)	75 (0.6%)
(Missing)	85 (0.6%)	74 (0.6%)
Number of children		
Mean	0.6	0.5
Standard Deviation	1.0	1.0
Min, Max	0.0, 18.0	0.0, 14.0
(Missing)	98 (0.7%)	1,190 (10%)
Community participation, n (%)		
More than once a week	1,630 (10.8%)	1,286 (11.1%)
Once a week	1,268 (8.4%)	992 (8.5%)
One to three times a month	1,843 (12.2%)	1,415 (12.2%)
A few times a year	3,322 (22.0%)	2,401 (20.7%)
Never	6,981 (46.3%)	5,495 (47.3%)
(Missing)	24 (0.2%)	22 (0.2%)
Religious attendance, n (%)		

Table S27b. Weighted summary statistics for outcome variables in Sweden

Outcome	Wave 1<br>N = 15,068	Wave 2<br>N = 11,611
More than once a week	234 (1.6%)	204 (1.8%)
Once a week	429 (2.8%)	311 (2.7%)
One to three times a month	481 (3.2%)	384 (3.3%)
A few times a year	3,868 (25.7%)	2,726 (23.5%)
Never	10,010 (66.4%)	7,948 (68.5%)
(Missing)	45 (0.3%)	38 (0.3%)
Loneliness		
Mean	3.1	3.0
Standard Deviation	2.7	2.6
Min, Max	0.0, 10.0	0.0, 10.0
(Missing)	30 (0.2%)	22 (0.2%)
Perceived discrimination, n (%)		
Always	262 (1.7%)	188 (1.6%)
Often	1,271 (8.4%)	1,025 (8.8%)
Rarely	5,294 (35.1%)	4,193 (36.1%)
Never	8,176 (54.3%)	6,140 (52.9%)
(Missing)	65 (0.4%)	66 (0.6%)
Orientation to promote good		
Mean	7.5	7.6
Standard Deviation	1.7	1.6
Min, Max	0.0, 10.0	0.0, 10.0
(Missing)	90 (0.6%)	55 (0.5%)
Delayed gratification		
Mean	7.0	7.2
Standard Deviation	2.0	1.9
Min, Max	0.0, 10.0	0.0, 10.0
(Missing)	112 (0.7%)	142 (1.2%)
Hope		
Mean	7.2	7.2
Standard Deviation	2.2	2.2
Min, Max	0.0, 10.0	0.0, 10.0
(Missing)	61 (0.4%)	38 (0.3%)
Gratitude		
Mean	7.5	7.5
Standard Deviation	2.3	2.2
Min, Max	0.0, 10.0	0.0, 10.0
(Missing)	48 (0.3%)	38 (0.3%)
Showing love/care		
Mean	8.1	8.1
Standard Deviation	1.9	1.8
Min, Max	0.0, 10.0	0.0, 10.0
(Missing)	45 (0.3%)	27 (0.2%)
Forgivingness, n (%)		
Always	2,001 (13.3%)	1,475 (12.7%)
Often	9,424 (62.5%)	7,282 (62.7%)
Rarely	3,260 (21.6%)	2,520 (21.7%)
Never	281 (1.9%)	238 (2.0%)
(Missing)	101 (0.7%)	97 (0.8%)
Charitable giving, n (%)		
Yes	7,657 (50.8%)	5,305 (45.7%)
No	7,363 (48.9%)	6,261 (53.9%)

Table S27b. Weighted summary statistics for outcome variables in Sweden

**Outcome**	**Wave 1**<br>N = 15,068	**Wave 2**<br>N = 11,611
(Missing)	49 (0.3%)	45 (0.4%)
Helping strangers, n (%)		
Yes	6,603 (43.8%)	4,667 (40.2%)
No	8,422 (55.9%)	6,916 (59.6%)
(Missing)	43 (0.3%)	27 (0.2%)
Volunteering, n (%)		
Yes	1,652 (11.0%)	1,310 (11.3%)
No	13,350 (88.6%)	10,259 (88.4%)
(Missing)	66 (0.4%)	42 (0.4%)
Self-rated physical health		
Mean	6.4	6.4
Standard Deviation	2.1	2.0
Min, Max	0.0, 10.0	0.0, 10.0
(Missing)	35 (0.2%)	14 (0.1%)
Health problems, n (%)		
Yes	3,861 (25.6%)	3,399 (29.3%)
No	11,157 (74.0%)	8,161 (70.3%)
(Missing)	49 (0.3%)	52 (0.4%)
Pain in past 4 weeks, n (%)		
A lot	1,353 (9.0%)	1,167 (10.1%)
Some	4,631 (30.7%)	3,594 (31.0%)
Not very much	5,913 (39.2%)	4,655 (40.1%)
None at all	3,130 (20.8%)	2,168 (18.7%)
(Missing)	42 (0.3%)	26 (0.2%)
Number of cigarettes per day		
Mean	0.9	0.8
Standard Deviation	3.4	3.2
Min, Max	0.0, 97.0	0.0, 46.0
(Missing)	446 (3.0%)	290 (2.5%)
Number of drinks per week		
Mean	2.9	2.7
Standard Deviation	4.6	4.7
Min, Max	0.0, 97.0	0.0, 97.0
(Missing)	200 (1.3%)	123 (1.1%)
Days exercise per week		
Mean	2.8	2.6
Standard Deviation	2.3	2.2
Min, Max	0.0, 7.0	0.0, 7.0
(Missing)	22 (0.1%)	19 (0.2%)
Financial security		
Mean	7.2	7.2
Standard Deviation	2.8	2.9
Min, Max	0.0, 10.0	0.0, 10.0
(Missing)	31 (0.2%)	13 (0.1%)
Material security		
Mean	7.5	7.5
Standard Deviation	2.6	2.5
Min, Max	0.0, 10.0	0.0, 10.0
(Missing)	48 (0.3%)	45 (0.4%)
Educational attainment (16+ years), n (%)		
Up to 8	265 (1.8%)	148 (1.3%)

Table S27b. Weighted summary statistics for outcome variables in Sweden

**Outcome**	**Wave 1**<br>N = 15,068	**Wave 2**<br>N = 11,611
9-15	11,048 (73.3%)	8,302 (71.5%)
16+	3,755 (24.9%)	3,161 (27.2%)
(Missing)	0 (0%)	0 (0%)
Currently employed, n (%)		
Employed for an employer	7,972 (52.9%)	6,138 (52.9%)
Self-employed	1,193 (7.9%)	827 (7.1%)
Retired	3,696 (24.5%)	2,980 (25.7%)
Student	1,419 (9.4%)	952 (8.2%)
Homemaker	77 (0.5%)	47 (0.4%)
Unemployed and looking for a job	343 (2.3%)	309 (2.7%)
None of these/Other	351 (2.3%)	324 (2.8%)
(Missing)	18 (0.1%)	34 (0.3%)
Financially comfortable/getting by, n (%)		
Living comfortably on present income	6,672 (44.3%)	5,152 (44.4%)
Getting by on present income	7,453 (49.5%)	5,283 (45.5%)
Finding it difficult on present income	701 (4.7%)	905 (7.8%)
Finding it very difficult on present income	171 (1.1%)	214 (1.8%)
(Missing)	71 (0.5%)	56 (0.5%)
Own home, n (%)		
Someone in this household owns this home	0 (0%)	0 (0%)
Someone in this household rents this home	0 (0%)	0 (0%)
Both	0 (0%)	0 (0%)
Neither	0 (0%)	0 (0%)
Rent	4,303 (28.6%)	3,285 (28.3%)
Own	10,513 (69.8%)	8,054 (69.4%)
Something else	218 (1.4%)	234 (2.0%)
(Missing)	34 (0.2%)	38 (0.3%)
Religious/spiritual connection, n (%)		
Always	1,014 (6.7%)	1,014 (8.7%)
Often	2,766 (18.4%)	1,931 (16.6%)
Rarely	5,915 (39.3%)	4,082 (35.2%)
Never	5,321 (35.3%)	4,536 (39.1%)
(Missing)	52 (0.3%)	48 (0.4%)
Belief in life after death, n (%)		
Yes	4,397 (29.2%)	3,226 (27.8%)
No	5,497 (36.5%)	4,319 (37.2%)
Unsure	5,099 (33.8%)	3,989 (34.4%)
(Missing)	75 (0.5%)	77 (0.7%)
Transformative religious experience, n (%)		
Yes	1,988 (13.2%)	1,432 (12.3%)
No	13,009 (86.3%)	10,131 (87.3%)
(Missing)	71 (0.5%)	49 (0.4%)
Religious reading or listening, n (%)		
More than once a day	194 (1.3%)	182 (1.6%)
About once a day	487 (3.2%)	376 (3.2%)
Sometimes	3,643 (24.2%)	2,689 (23.2%)
Never	10,680 (70.9%)	8,333 (71.8%)
(Missing)	64 (0.4%)	31 (0.3%)
Prayer or meditation, n (%)		
More than once a day	529 (3.5%)	427 (3.7%)
About once a day	1,166 (7.7%)	813 (7.0%)

Table S27b. Weighted summary statistics for outcome variables in Sweden

**Outcome**	**Wave 1**<br>N = 15,068	**Wave 2**<br>N = 11,611
Sometimes	4,440 (29.5%)	3,205 (27.6%)
Never	8,895 (59.0%)	7,143 (61.5%)
(Missing)	39 (0.3%)	24 (0.2%)
Belief in God/gods/spiritual forces, n (%)		
One God	2,954 (19.6%)	2,169 (18.7%)
More than one god	408 (2.7%)	281 (2.4%)
An impersonal spiritual force	3,158 (21.0%)	2,185 (18.8%)
None of these	5,854 (38.9%)	4,815 (41.5%)
Unsure	2,623 (17.4%)	2,108 (18.2%)
(Missing)	71 (0.5%)	55 (0.5%)
Religious centrality, n (%)		
Agree	1,846 (12.3%)	1,293 (11.1%)
Disagree	4,773 (31.7%)	3,315 (28.6%)
Not relevant	6,973 (46.3%)	5,968 (51.4%)
Unsure	1,336 (8.9%)	916 (7.9%)
(Missing)	139 (0.9%)	118 (1.0%)
Religious/spiritual comfort, n (%)		
Agree	3,216 (21.3%)	2,180 (18.8%)
Disagree	3,406 (22.6%)	2,438 (21.0%)
Not relevant	6,881 (45.7%)	5,889 (50.7%)
Unsure	1,427 (9.5%)	989 (8.5%)
(Missing)	137 (0.9%)	115 (1.0%)
Feel loved by God, n (%)		
Agree	2,672 (17.7%)	1,888 (16.3%)
Disagree	3,547 (23.5%)	2,524 (21.7%)
Not relevant	7,020 (46.6%)	5,881 (50.6%)
Unsure	1,668 (11.1%)	1,183 (10.2%)
(Missing)	161 (1.1%)	135 (1.2%)
Feel punished by God, n (%)		
Agree	614 (4.1%)	410 (3.5%)
Disagree	6,658 (44.2%)	4,811 (41.4%)
Not relevant	6,476 (43.0%)	5,471 (47.1%)
Unsure	1,178 (7.8%)	799 (6.9%)
(Missing)	142 (0.9%)	119 (1.0%)
Experienced religious criticism, n (%)		
Agree	353 (2.3%)	225 (1.9%)
Disagree	4,533 (30.1%)	3,205 (27.6%)
Not relevant	9,295 (61.7%)	7,452 (64.2%)
Unsure	701 (4.7%)	568 (4.9%)
(Missing)	186 (1.2%)	162 (1.4%)
Faith-sharing, n (%)		
Agree	2,822 (18.7%)	1,912 (16.5%)
Disagree	3,692 (24.5%)	2,598 (22.4%)
Not relevant	7,706 (51.1%)	6,417 (55.3%)
Unsure	701 (4.7%)	541 (4.7%)
(Missing)	147 (1.0%)	143 (1.2%)

Note. N (%); this table is based on non-imputed data. Cumulative percentages for variables may not add up to 100% due to rounding. Wave 1 characteristics weighted using the Gallup provided sampling weight, ANNUAL_WEIGHT_R2; Wave 2 characteristics weighted accounting for attrition by using the adjusted Wave 1 weight, ANNUAL_WEIGHT_R2, multiplied by the created attrition weight to account for dropout, to maintain nationally representative estimates for Wave 2 characteristics.

Table S27c. Unweighted summary statistics for demographic and childhood variables in Sweden by retention status

**Characteristic**	**Attritors–Not Observed in Wave 2** N = 3,467	**Retained–Observed in Wave 2** N = 11,521
Forgivingness, n (%)		
Always	524 (15.1%)	1,469 (12.8%)
Often	2,076 (59.9%)	7,295 (63.3%)
Rarely	774 (22.3%)	2,471 (21.4%)
Never	81 (2.3%)	199 (1.7%)
(Missing)	12 (0.4%)	88 (0.8%)
Year of birth, n (%)		
1943 or earlier (current age: 80+ years)	63 (1.8%)	520 (4.5%)
1943-1953 (current age: 70-79 years)	196 (5.6%)	1,934 (16.8%)
1953-1963 (current age: 60-69 years)	204 (5.9%)	1,838 (16.0%)
1963-1973 (current age: 50-59 years)	347 (10.0%)	2,098 (18.2%)
1973-1983 (current age: 40-49 years)	437 (12.6%)	1,824 (15.8%)
1983-1993 (current age: 30-39 years)	665 (19.2%)	1,781 (15.5%)
1993-1998 (current age: 25-29 years)	626 (18.0%)	811 (7.0%)
1998-2005 (current age: 18-24 years)	930 (26.8%)	716 (6.2%)
(Missing)	0 (0%)	0 (0%)
Age of participant		
Mean	37.4	51.9
Standard Deviation	17.2	17.8
Min, Max	18.0, 93.0	18.0, 98.0
Gender, n (%)		
Male	1,866 (53.8%)	5,595 (48.6%)
Female	1,586 (45.7%)	5,902 (51.2%)
Other	13 (0.4%)	16 (0.1%)
(Missing)	2 (0.1%)	10 (0.1%)
Respondent marital status, n (%)		
Single/Never been married	1,303 (37.6%)	2,654 (23.0%)
Married	965 (27.8%)	5,230 (45.4%)
Separated	101 (2.9%)	326 (2.8%)
Divorced	121 (3.5%)	666 (5.8%)
Widowed	52 (1.5%)	366 (3.2%)
Domestic partner	897 (25.9%)	2,233 (19.4%)
(Missing)	29 (0.8%)	46 (0.4%)
Education (years), n (%)		
Up to 8	57 (1.6%)	207 (1.8%)
9-15	2,734 (78.9%)	8,263 (71.7%)
16+	677 (19.5%)	3,051 (26.5%)
(Missing)	0 (0%)	0 (0%)
Employment status, n (%)		
Employed for an employer	1,922 (55.4%)	6,011 (52.2%)
Self-employed	294 (8.5%)	893 (7.8%)
Retired	353 (10.2%)	3,303 (28.7%)
Student	687 (19.8%)	738 (6.4%)
Homemaker	17 (0.5%)	60 (0.5%)
Unemployed and looking for a job	118 (3.4%)	225 (2.0%)
None of these/Other	69 (2.0%)	279 (2.4%)
(Missing)	7 (0.2%)	11 (0.1%)
Current religious service attendance, n (%)		
More than once a week	71 (2.1%)	162 (1.4%)

Table S27c. Unweighted summary statistics for demographic and childhood variables in Sweden by retention status

Characteristic	Attritors–Not Observed in Wave 2<br>N = 3,467	Retained–Observed in Wave 2<br>N = 11,521
Once a week	150 (4.3%)	279 (2.4%)
One to three times a month	143 (4.1%)	337 (2.9%)
A few times a year	775 (22.4%)	3,068 (26.6%)
Never	2,315 (66.8%)	7,643 (66.3%)
(Missing)	13 (0.4%)	32 (0.3%)
Immigration status, n (%)		
Born in this country	3,160 (91.1%)	10,692 (92.8%)
Born in another country	287 (8.3%)	759 (6.6%)
(Missing)	21 (0.6%)	71 (0.6%)
Parental marital status around age 12, n (%)		
Parents were married	2,203 (63.5%)	8,510 (73.9%)
Parents were divorced	566 (16.3%)	1,397 (12.1%)
Parents were never married	576 (16.6%)	1,235 (10.7%)
One or both of them had died	71 (2.1%)	284 (2.5%)
Unsure	41 (1.2%)	51 (0.4%)
(Missing)	10 (0.3%)	44 (0.4%)
Religious service attendance around age 12, n (%)		
At least once a week	256 (7.4%)	686 (6.0%)
One to three times a month	318 (9.2%)	1,015 (8.8%)
Less than once a month	1,344 (38.8%)	4,771 (41.4%)
Never	1,539 (44.4%)	5,009 (43.5%)
(Missing)	10 (0.3%)	41 (0.4%)
Relationship with mother when growing up, n (%)		
Very good	2,036 (58.7%)	6,638 (57.6%)
Somewhat good	988 (28.5%)	3,506 (30.4%)
Somewhat bad	288 (8.3%)	910 (7.9%)
Very bad	96 (2.8%)	281 (2.4%)
(Does not apply)	48 (1.4%)	169 (1.5%)
(Missing)	11 (0.3%)	18 (0.2%)
Relationship with father when growing up, n (%)		
Very good	1,675 (48.3%)	5,385 (46.7%)
Somewhat good	1,046 (30.2%)	3,813 (33.1%)
Somewhat bad	374 (10.8%)	1,226 (10.6%)
Very bad	211 (6.1%)	528 (4.6%)
(Does not apply)	158 (4.6%)	558 (4.8%)
(Missing)	4 (0.1%)	11 (0.1%)
Felt like an outsider in family when growing up, n (%)		
Yes	521 (15.0%)	1,372 (11.9%)
No	2,913 (84.0%)	10,015 (86.9%)
(Missing)	33 (1.0%)	134 (1.2%)
Experienced abuse when growing up, n (%)		
Yes	555 (16.0%)	1,742 (15.1%)
No	2,902 (83.7%)	9,746 (84.6%)
(Missing)	10 (0.3%)	33 (0.3%)
Self-rated health when growing up, n (%)		
Excellent	1,195 (34.5%)	4,423 (38.4%)
Very good	1,205 (34.8%)	3,900 (33.9%)

Table S27c. Unweighted summary statistics for demographic and childhood variables in Sweden by retention status

**Characteristic**	**Attritors–Not Observed in Wave 2** N = 3,467	**Retained–Observed in Wave 2** N = 11,521
Good	649 (18.7%)	2,044 (17.7%)
Fair	295 (8.5%)	832 (7.2%)
Poor	111 (3.2%)	299 (2.6%)
(Missing)	13 (0.4%)	23 (0.2%)
Subjective financial status of family growing up, n (%)		
Lived comfortably	1,545 (44.6%)	4,376 (38.0%)
Got by	1,542 (44.5%)	6,101 (53.0%)
Found it difficult	326 (9.4%)	930 (8.1%)
Found it very difficult	51 (1.5%)	96 (0.8%)
(Missing)	3 (0.1%)	18 (0.2%)
Religious affiliation growing up, n (%)		
Christianity	2,025 (58.4%)	8,399 (72.9%)
Taoism	0 (0%)	0 (0%)
Confucianism	2 (0.1%)	1 (0.0%)
Primal, Animist, or Folk religion	9 (0.3%)	22 (0.2%)
Spiritism	0 (0%)	0 (0%)
Umbanda, Candomblé, and other African-derived religions	0 (0%)	0 (0%)
Chinese folk/traditional religion	0 (0%)	0 (0%)
Islam	204 (5.9%)	257 (2.2%)
Hinduism	7 (0.2%)	10 (0.1%)
Buddhism	16 (0.5%)	25 (0.2%)
Judaism	7 (0.2%)	40 (0.3%)
Sikhism	6 (0.2%)	3 (0.0%)
Baha'i	0 (0%)	3 (0.0%)
Jainism	0 (0%)	0 (0%)
Shinto	1 (0.0%)	0 (0%)
Some other religion	18 (0.5%)	50 (0.4%)
No religion/Atheist/Agnostic	1,165 (33.6%)	2,692 (23.4%)
(Missing)	6 (0.2%)	21 (0.2%)

Note. N (%); this table is based on non-imputed data. Cumulative percentages for variables may not add up to 100% due to rounding.

Table S27d. Unweighted summary statistics for Wave 1 outcome variables in Sweden by retention status.

**Outcome**	**Attritors–Not Observed in Wave 2** N = 3,467	**Retained–Observed in Wave 2** N = 11,521
Secure flourishing index		
Mean	6.8	7.1
Standard Deviation	1.6	1.5
Min, Max	0.1, 10.0	0.0, 10.0
(Missing)	114 (3.3%)	391 (3.4%)
Flourishing index		
Mean	6.8	7.1
Standard Deviation	1.6	1.6
Min, Max	0.1, 10.0	0.0, 10.0
(Missing)	97 (2.8%)	357 (3.1%)
Happiness & life satisfaction		
Mean	6.8	7.1
Standard Deviation	2.0	1.9
Min, Max	0.0, 10.0	0.0, 10.0
(Missing)	8 (0.2%)	54 (0.5%)
Physical & mental health		
Mean	6.5	6.8
Standard Deviation	1.9	1.8
Min, Max	0.0, 10.0	0.0, 10.0
(Missing)	10 (0.3%)	37 (0.3%)
Meaning & purpose		
Mean	6.5	6.9
Standard Deviation	2.3	2.1
Min, Max	0.0, 10.0	0.0, 10.0
(Missing)	26 (0.8%)	135 (1.2%)
Character & virtue		
Mean	7.3	7.2
Standard Deviation	1.6	1.6
Min, Max	0.0, 10.0	0.0, 10.0
(Missing)	34 (1.0%)	135 (1.2%)
Close social relationships		
Mean	7.0	7.3
Standard Deviation	2.3	2.2
Min, Max	0.0, 10.0	0.0, 10.0
(Missing)	29 (0.8%)	68 (0.6%)
Financial & material security		
Mean	6.9	7.4
Standard Deviation	2.7	2.5
Min, Max	0.0, 10.0	0.0, 10.0
(Missing)	19 (0.5%)	45 (0.4%)
Happiness		
Mean	6.8	7.1
Standard Deviation	2.1	1.9
Min, Max	0.0, 10.0	0.0, 10.0
(Missing)	7 (0.2%)	31 (0.3%)
Life satisfaction		
Mean	6.8	7.1
Standard Deviation	2.2	2.1
Min, Max	0.0, 10.0	0.0, 10.0

Table S27d. Unweighted summary statistics for Wave 1 outcome variables in Sweden by retention status.

**Outcome**	**Attritors–Not Observed in Wave 2**<br>N = 3,467	**Retained–Observed in Wave 2**<br>N = 11,521
(Missing)	4 (0.1%)	30 (0.3%)
Current life evaluation		
Mean	6.9	7.2
Standard Deviation	1.8	1.7
Min, Max	0.0, 10.0	0.0, 10.0
(Missing)	13 (0.4%)	33 (0.3%)
Future life evaluation		
Mean	8.0	7.6
Standard Deviation	1.7	1.8
Min, Max	0.0, 10.0	0.0, 10.0
(Missing)	7 (0.2%)	35 (0.3%)
Optimism		
Mean	7.2	7.3
Standard Deviation	2.5	2.3
Min, Max	0.0, 10.0	0.0, 10.0
(Missing)	13 (0.4%)	39 (0.3%)
Freedom to pursue what's important		
Mean	7.1	7.6
Standard Deviation	2.3	2.2
Min, Max	0.0, 10.0	0.0, 10.0
(Missing)	11 (0.3%)	28 (0.2%)
Inner peace, n (%)		
Always	353 (10.2%)	1,321 (11.5%)
Often	2,278 (65.7%)	8,174 (70.9%)
Rarely	765 (22.1%)	1,897 (16.5%)
Never	53 (1.5%)	88 (0.8%)
(Missing)	18 (0.5%)	41 (0.4%)
Life balance, n (%)		
Always	282 (8.1%)	967 (8.4%)
Often	2,279 (65.7%)	8,243 (71.5%)
Rarely	819 (23.6%)	2,135 (18.5%)
Never	57 (1.6%)	99 (0.9%)
(Missing)	30 (0.9%)	77 (0.7%)
Sense of mastery, n (%)		
Always	583 (16.8%)	1,717 (14.9%)
Often	2,296 (66.2%)	8,135 (70.6%)
Rarely	537 (15.5%)	1,501 (13.0%)
Never	35 (1.0%)	97 (0.8%)
(Missing)	16 (0.4%)	71 (0.6%)
Meaningful activities		
Mean	6.6	7.1
Standard Deviation	2.3	2.2
Min, Max	0.0, 10.0	0.0, 10.0
(Missing)	8 (0.2%)	36 (0.3%)
Understanding purpose		
Mean	6.3	6.7
Standard Deviation	2.6	2.5
Min, Max	0.0, 10.0	0.0, 10.0
(Missing)	18 (0.5%)	101 (0.9%)

Table S27d. Unweighted summary statistics for Wave 1 outcome variables in Sweden by retention status.

**Outcome**	**Attritors–Not Observed in Wave 2** N = 3,467	**Retained–Observed in Wave 2** N = 11,521
Self-rated mental health		
Mean	6.6	7.3
Standard Deviation	2.4	2.2
Min, Max	0.0, 10.0	0.0, 10.0
(Missing)	4 (0.1%)	15 (0.1%)
Traumatic distress, n (%)		
A lot	216 (6.2%)	491 (4.3%)
Some	743 (21.4%)	2,314 (20.1%)
Not very much	1,343 (38.7%)	4,369 (37.9%)
Not at all	1,142 (32.9%)	4,205 (36.5%)
(Missing)	23 (0.7%)	142 (1.2%)
Depression symptoms composite, n (%)	916 (26.6%)	1,883 (16.5%)
(Missing)	28 (0.8%)	124 (1.1%)
Depression – feel hopeless, n (%)		
Nearly every day	306 (8.8%)	616 (5.3%)
More than half the days	374 (10.8%)	830 (7.2%)
Several days	1,136 (32.8%)	3,261 (28.3%)
Not at all	1,643 (47.4%)	6,761 (58.7%)
(Missing)	9 (0.3%)	54 (0.5%)
Depression – loss of interest, n (%)		
Nearly every day	256 (7.4%)	601 (5.2%)
More than half the days	558 (16.1%)	1,093 (9.5%)
Several days	995 (28.7%)	2,947 (25.6%)
Not at all	1,635 (47.2%)	6,801 (59.0%)
(Missing)	23 (0.7%)	79 (0.7%)
Anxiety symptoms composite, n (%)	823 (23.8%)	1,614 (14.1%)
(Missing)	16 (0.5%)	79 (0.7%)
Anxiety – feel on edge, n (%)		
Nearly every day	335 (9.7%)	642 (5.6%)
More than half the days	421 (12.1%)	890 (7.7%)
Several days	1,430 (41.3%)	3,965 (34.4%)
Not at all	1,274 (36.7%)	5,980 (51.9%)
(Missing)	7 (0.2%)	45 (0.4%)
Anxiety – cannot stop worrying, n (%)		
Nearly every day	299 (8.6%)	454 (3.9%)
More than half the days	372 (10.7%)	740 (6.4%)
Several days	868 (25.0%)	2,551 (22.1%)
Not at all	1,919 (55.3%)	7,729 (67.1%)
(Missing)	10 (0.3%)	47 (0.4%)
Suffering, n (%)		
A lot	242 (7.0%)	554 (4.8%)
Some	1,099 (31.7%)	3,304 (28.7%)
Not very much	1,420 (40.9%)	4,887 (42.4%)
Not at all	691 (19.9%)	2,724 (23.6%)
(Missing)	16 (0.5%)	52 (0.5%)
Relationship contentment		
Mean	7.2	7.5
Standard Deviation	2.3	2.2
Min, Max	0.0, 10.0	0.0, 10.0

Table S27d. Unweighted summary statistics for Wave 1 outcome variables in Sweden by retention status.

**Outcome**	**Attritors–Not Observed in Wave 2** N = 3,467	**Retained–Observed in Wave 2** N = 11,521
(Missing)	11 (0.3%)	33 (0.3%)
Relationship satisfaction		
Mean	6.8	7.0
Standard Deviation	2.5	2.4
Min, Max	0.0, 10.0	0.0, 10.0
(Missing)	20 (0.6%)	46 (0.4%)
Social support		
Mean	8.1	8.2
Standard Deviation	2.2	2.1
Min, Max	0.0, 10.0	0.0, 10.0
(Missing)	8 (0.2%)	49 (0.4%)
Intimate/close friend, n (%)		
Yes	3,050 (88.0%)	9,896 (85.9%)
No	409 (11.8%)	1,597 (13.9%)
(Missing)	9 (0.2%)	28 (0.2%)
Government approval, n (%)		
Strongly approve	166 (4.8%)	575 (5.0%)
Somewhat approve	636 (18.4%)	2,156 (18.7%)
Neither approve nor disapprove	1,034 (29.8%)	3,238 (28.1%)
Somewhat disapprove	926 (26.7%)	3,051 (26.5%)
Strongly disapprove	672 (19.4%)	2,449 (21.3%)
(Missing)	33 (0.9%)	52 (0.5%)
Say in government, n (%)		
Agree	778 (22.4%)	2,466 (21.4%)
Disagree	1,766 (50.9%)	6,017 (52.2%)
Unsure	908 (26.2%)	2,980 (25.9%)
(Missing)	16 (0.5%)	57 (0.5%)
Belonging in country		
Mean	8.3	8.6
Standard Deviation	2.2	2.0
Min, Max	0.0, 10.0	0.0, 10.0
(Missing)	19 (0.5%)	37 (0.3%)
City/place satisfaction, n (%)		
Satisfied	2,597 (74.9%)	9,598 (83.3%)
Dissatisfied	467 (13.5%)	959 (8.3%)
Unsure	392 (11.3%)	915 (7.9%)
(Missing)	12 (0.3%)	49 (0.4%)
Trust within country, n (%)		
All people	34 (1.0%)	25 (0.2%)
Most people	1,036 (29.9%)	4,537 (39.4%)
Some people	1,418 (40.9%)	4,692 (40.7%)
Not very many people	934 (26.9%)	2,153 (18.7%)
None	35 (1.0%)	41 (0.4%)
(Missing)	10 (0.3%)	74 (0.6%)
Number of children		
Mean	0.8	0.5
Standard Deviation	1.3	1.0
Min, Max	0.0, 15.0	0.0, 18.0
(Missing)	30 (0.9%)	68 (0.6%)

Table S27d. Unweighted summary statistics for Wave 1 outcome variables in Sweden by retention status.

**Outcome**	**Attritors–Not Observed in Wave 2**<br>N = 3,467	**Retained–Observed in Wave 2**<br>N = 11,521
Community participation, n (%)		
More than once a week	449 (13.0%)	1,175 (10.2%)
Once a week	278 (8.0%)	983 (8.5%)
One to three times a month	344 (9.9%)	1,486 (12.9%)
A few times a year	723 (20.8%)	2,580 (22.4%)
Never	1,670 (48.1%)	5,277 (45.8%)
(Missing)	4 (0.1%)	21 (0.2%)
Religious attendance, n (%)		
More than once a week	71 (2.1%)	162 (1.4%)
Once a week	150 (4.3%)	279 (2.4%)
One to three times a month	143 (4.1%)	337 (2.9%)
A few times a year	775 (22.4%)	3,068 (26.6%)
Never	2,315 (66.8%)	7,643 (66.3%)
(Missing)	13 (0.4%)	32 (0.3%)
Loneliness		
Mean	3.4	3.0
Standard Deviation	2.7	2.7
Min, Max	0.0, 10.0	0.0, 10.0
(Missing)	7 (0.2%)	22 (0.2%)
Perceived discrimination, n (%)		
Always	85 (2.5%)	176 (1.5%)
Often	372 (10.7%)	896 (7.8%)
Rarely	1,327 (38.3%)	3,943 (34.2%)
Never	1,670 (48.2%)	6,455 (56.0%)
(Missing)	13 (0.4%)	51 (0.4%)
Orientation to promote good		
Mean	7.4	7.6
Standard Deviation	1.8	1.7
Min, Max	0.0, 10.0	0.0, 10.0
(Missing)	17 (0.5%)	73 (0.6%)
Delayed gratification		
Mean	7.2	6.9
Standard Deviation	2.0	2.0
Min, Max	0.0, 10.0	0.0, 10.0
(Missing)	20 (0.6%)	91 (0.8%)
Hope		
Mean	7.3	7.2
Standard Deviation	2.3	2.2
Min, Max	0.0, 10.0	0.0, 10.0
(Missing)	19 (0.5%)	42 (0.4%)
Gratitude		
Mean	7.3	7.5
Standard Deviation	2.3	2.2
Min, Max	0.0, 10.0	0.0, 10.0
(Missing)	9 (0.3%)	39 (0.3%)
Showing love/care		
Mean	7.9	8.1
Standard Deviation	2.0	1.8
Min, Max	0.0, 10.0	0.0, 10.0

Table S27d. Unweighted summary statistics for Wave 1 outcome variables in Sweden by retention status.

**Outcome**	**Attritors–Not Observed in Wave 2** N = 3,467	**Retained–Observed in Wave 2** N = 11,521
(Missing)	9 (0.3%)	35 (0.3%)
Forgivingness, n (%)		
Always	524 (15.1%)	1,469 (12.8%)
Often	2,076 (59.9%)	7,295 (63.3%)
Rarely	774 (22.3%)	2,471 (21.4%)
Never	81 (2.3%)	199 (1.7%)
(Missing)	12 (0.4%)	88 (0.8%)
Charitable giving, n (%)		
Yes	1,509 (43.5%)	6,098 (52.9%)
No	1,950 (56.2%)	5,385 (46.7%)
(Missing)	9 (0.3%)	39 (0.3%)
Helping strangers, n (%)		
Yes	1,970 (56.8%)	4,617 (40.1%)
No	1,489 (42.9%)	6,871 (59.6%)
(Missing)	9 (0.2%)	34 (0.3%)
Volunteering, n (%)		
Yes	401 (11.6%)	1,243 (10.8%)
No	3,058 (88.2%)	10,221 (88.7%)
(Missing)	8 (0.2%)	57 (0.5%)
Self-rated physical health		
Mean	6.4	6.4
Standard Deviation	2.1	2.1
Min, Max	0.0, 10.0	0.0, 10.0
(Missing)	7 (0.2%)	28 (0.2%)
Health problems, n (%)		
Yes	761 (22.0%)	3,075 (26.7%)
No	2,695 (77.7%)	8,408 (73.0%)
(Missing)	11 (0.3%)	38 (0.3%)
Pain in past 4 weeks, n (%)		
A lot	319 (9.2%)	1,027 (8.9%)
Some	1,025 (29.6%)	3,580 (31.1%)
Not very much	1,379 (39.8%)	4,504 (39.1%)
None at all	732 (21.1%)	2,383 (20.7%)
(Missing)	14 (0.4%)	28 (0.2%)
Number of cigarettes per day		
Mean	0.9	0.8
Standard Deviation	3.5	3.3
Min, Max	0.0, 97.0	0.0, 97.0
(Missing)	95 (2.7%)	349 (3.0%)
Number of drinks per week		
Mean	2.9	2.9
Standard Deviation	4.6	4.6
Min, Max	0.0, 97.0	0.0, 97.0
(Missing)	46 (1.3%)	152 (1.3%)
Days exercise per week		
Mean	2.9	2.7
Standard Deviation	2.2	2.3
Min, Max	0.0, 7.0	0.0, 7.0
(Missing)	6 (0.2%)	17 (0.1%)

Table S27d. Unweighted summary statistics for Wave 1 outcome variables in Sweden by retention status.

**Outcome**	**Attritors–Not Observed in Wave 2** N = 3,467	**Retained–Observed in Wave 2** N = 11,521
Financial security		
Mean	6.7	7.3
Standard Deviation	3.0	2.8
Min, Max	0.0, 10.0	0.0, 10.0
(Missing)	10 (0.3%)	21 (0.2%)
Material security		
Mean	7.2	7.5
Standard Deviation	2.8	2.5
Min, Max	0.0, 10.0	0.0, 10.0
(Missing)	16 (0.5%)	32 (0.3%)
Educational attainment (16+ years), n (%)		
Up to 8	57 (1.6%)	207 (1.8%)
9-15	2,734 (78.9%)	8,263 (71.7%)
16+	677 (19.5%)	3,051 (26.5%)
(Missing)	0 (0%)	0 (0%)
Currently employed, n (%)		
Employed for an employer	1,922 (55.4%)	6,011 (52.2%)
Self-employed	294 (8.5%)	893 (7.8%)
Retired	353 (10.2%)	3,303 (28.7%)
Student	687 (19.8%)	738 (6.4%)
Homemaker	17 (0.5%)	60 (0.5%)
Unemployed and looking for a job	118 (3.4%)	225 (2.0%)
None of these/Other	69 (2.0%)	279 (2.4%)
(Missing)	7 (0.2%)	11 (0.1%)
Financially comfortable/getting by, n (%)		
Living comfortably on present income	1,458 (42.1%)	5,176 (44.9%)
Getting by on present income	1,742 (50.2%)	5,673 (49.2%)
Finding it difficult on present income	201 (5.8%)	499 (4.3%)
Finding it very difficult on present income	47 (1.3%)	123 (1.1%)
(Missing)	20 (0.6%)	51 (0.4%)
Own home, n (%)		
Someone in this household owns this home	0 (0%)	0 (0%)
Someone in this household rents this home	0 (0%)	0 (0%)
Both	0 (0%)	0 (0%)
Neither	0 (0%)	0 (0%)
Rent	1,168 (33.7%)	3,120 (27.1%)
Own	2,198 (63.4%)	8,251 (71.6%)
Something else	87 (2.5%)	131 (1.1%)
(Missing)	15 (0.4%)	19 (0.2%)
Religious/spiritual connection, n (%)		
Always	265 (7.7%)	744 (6.5%)
Often	645 (18.6%)	2,107 (18.3%)
Rarely	1,209 (34.9%)	4,669 (40.5%)
Never	1,335 (38.5%)	3,962 (34.4%)
(Missing)	12 (0.4%)	39 (0.3%)
Belief in life after death, n (%)		
Yes	1,190 (34.3%)	3,191 (27.7%)
No	1,073 (30.9%)	4,387 (38.1%)
Unsure	1,191 (34.3%)	3,882 (33.7%)

Table S27d. Unweighted summary statistics for Wave 1 outcome variables in Sweden by retention status.

Outcome	Attritors–Not Observed in Wave 2<br>N = 3,467	Retained–Observed in Wave 2<br>N = 11,521
(Missing)	13 (0.4%)	61 (0.5%)
Transformative religious experience, n (%)		
Yes	549 (15.8%)	1,432 (12.4%)
No	2,900 (83.6%)	10,037 (87.1%)
(Missing)	18 (0.5%)	52 (0.5%)
Religious reading or listening, n (%)		
More than once a day	69 (2.0%)	125 (1.1%)
About once a day	150 (4.3%)	337 (2.9%)
Sometimes	806 (23.2%)	2,816 (24.4%)
Never	2,439 (70.3%)	8,185 (71.0%)
(Missing)	4 (0.1%)	59 (0.5%)
Prayer or meditation, n (%)		
More than once a day	146 (4.2%)	380 (3.3%)
About once a day	270 (7.8%)	890 (7.7%)
Sometimes	1,027 (29.6%)	3,390 (29.4%)
Never	2,021 (58.3%)	6,826 (59.2%)
(Missing)	3 (0.1%)	36 (0.3%)
Belief in God/gods/spiritual forces, n (%)		
One God	733 (21.1%)	2,207 (19.2%)
More than one god	104 (3.0%)	302 (2.6%)
An impersonal spiritual force	698 (20.1%)	2,442 (21.2%)
None of these	1,290 (37.2%)	4,531 (39.3%)
Unsure	618 (17.8%)	1,992 (17.3%)
(Missing)	25 (0.7%)	46 (0.4%)
Religious centrality, n (%)		
Agree	427 (12.3%)	1,410 (12.2%)
Disagree	1,112 (32.1%)	3,636 (31.6%)
Not relevant	1,604 (46.3%)	5,333 (46.3%)
Unsure	307 (8.9%)	1,021 (8.9%)
(Missing)	17 (0.5%)	121 (1.0%)
Religious/spiritual comfort, n (%)		
Agree	762 (22.0%)	2,438 (21.2%)
Disagree	778 (22.4%)	2,610 (22.7%)
Not relevant	1,591 (45.9%)	5,254 (45.6%)
Unsure	320 (9.2%)	1,100 (9.5%)
(Missing)	17 (0.5%)	119 (1.0%)
Feel loved by God, n (%)		
Agree	680 (19.6%)	1,980 (17.2%)
Disagree	749 (21.6%)	2,777 (24.1%)
Not relevant	1,651 (47.6%)	5,334 (46.3%)
Unsure	368 (10.6%)	1,291 (11.2%)
(Missing)	20 (0.6%)	139 (1.2%)
Feel punished by God, n (%)		
Agree	223 (6.4%)	391 (3.4%)
Disagree	1,383 (39.9%)	5,234 (45.4%)
Not relevant	1,548 (44.7%)	4,896 (42.5%)
Unsure	295 (8.5%)	877 (7.6%)
(Missing)	18 (0.5%)	122 (1.1%)
Experienced religious criticism, n (%)		

Table S27d. Unweighted summary statistics for Wave 1 outcome variables in Sweden by retention status.

Outcome	Attritors–Not Observed in Wave 2 N = 3,467	Retained–Observed in Wave 2 N = 11,521
Agree	133 (3.8%)	220 (1.9%)
Disagree	1,046 (30.2%)	3,463 (30.1%)
Not relevant	2,044 (58.9%)	7,199 (62.5%)
Unsure	217 (6.3%)	483 (4.2%)
(Missing)	28 (0.8%)	156 (1.4%)
Faith-sharing, n (%)		
Agree	707 (20.4%)	2,103 (18.3%)
Disagree	806 (23.2%)	2,865 (24.9%)
Not relevant	1,755 (50.6%)	5,909 (51.3%)
Unsure	180 (5.2%)	518 (4.5%)
(Missing)	19 (0.5%)	126 (1.1%)

Note. N (%); this table is based on non-imputed data. Cumulative percentages for variables may not add up to 100% due to rounding.

Table S27e. Summary of fitted attrition model in Sweden

Characteristic	Odds Ratio	95% CI	p-value
**ANNUAL_WEIGHT_R2**	1.03	0.95, 1.12	0.472
**Happiness & life satisfaction**	0.95	0.86, 1.04	0.251
**Physical & mental health**	1.09	1.01, 1.19	0.031
**Meaning & purpose**	1.04	0.96, 1.13	0.333
**Character & virtue**	0.88	0.83, 0.93	1.48e-05
**Close social relationships**	1.01	0.94, 1.09	0.768
**Financial & material security**	1.05	1.0, 1.12	0.076
**Extraversion**	0.96	0.90, 1.01	0.114
**Openness to experience**	0.92	0.87, 0.97	0.003
**Agreeableness**	1.06	1.01, 1.12	0.031
**Conscientiousness**	1.16	1.10, 1.22	9.99e-08
**Neuroticism**	0.98	0.92, 1.04	0.545
**Depression symptoms composite**	0.93	0.88, 0.99	0.026
**Anxiety symptoms composite**	0.99	0.93, 1.05	0.733
**Loneliness**	1.09	1.01, 1.17	0.018
**Days exercise per week**	0.96	0.91, 1.01	0.128
**Year of birth (age group)**			
1983-1993 (current age: 30-39 years)	—	—	
1963-1973 (current age: 50-59 years)	2.44	2.02, 2.96	6.76e-20
1953-1963 (current age: 60-69 years)	3.47	2.73, 4.41	4.50e-24
1943-1953 (current age: 70-79 years)	3.49	2.49, 4.87	2.75e-13
1973-1983 (current age: 40-49 years)	1.61	1.35, 1.92	1.71e-07
1998-2005 (current age: 18-24 years)	0.32	0.26, 0.39	6.28e-29
1993-1998 (current age: 25-29 years)	0.48	0.41, 0.57	6.97e-17
1943 or earlier (current age: 80+ years)	3.08	2.03, 4.67	1.20e-07
**Gender of respondent**			
Male	—	—	
Female	1.12	1.00, 1.25	0.055
Other	1.00	0.40, 2.53	0.993
**Marital status**			
Married	—	—	
Domestic partner	0.96	0.83, 1.11	0.576
Single/Never been married	1.05	0.88, 1.25	0.584
Divorced	0.83	0.64, 1.07	0.154
Separated	0.75	0.56, 1.00	0.053
Widowed	0.73	0.50, 1.07	0.111
**Employment status**			
Employed for an employer	—	—	
Retired	1.11	0.86, 1.45	0.417
Student	1.04	0.86, 1.26	0.666
Self-employed	0.74	0.60, 0.92	0.008
Unemployed and looking for a job	0.80	0.60, 1.07	0.136
None of these/Other	1.34	0.96, 1.88	0.083
Homemaker	1.28	0.66, 2.47	0.467
**Religious attendance**			
Never	—	—	
A few times a year	0.99	0.87, 1.13	0.922
One to three times a month	0.85	0.63, 1.15	0.288
Once a week	0.79	0.59, 1.06	0.113
More than once a week	1.01	0.65, 1.57	0.948
**Educational attainment (16+ years)**			
9-15	—	—	

Table S27e. Summary of fitted attrition model in Sweden

**Characteristic**	**Odds Ratio**	**95% CI**	**p-value**
16+	1.53	1.35, 1.74	1.04e-10
Up to 8	0.67	0.46, 0.97	0.033
**Born in This country**			
Born in this country	—	—	
Born in another country	0.93	0.76, 1.14	0.486
**Urbanicity**			
A large city	—	—	
A small town or village	1.04	0.91, 1.18	0.579
A suburb of a large city	1.05	0.91, 1.22	0.464
A rural area or on a farm	0.98	0.82, 1.17	0.835
**Monthly household income**			
Sweden: 40,001 - 50,000 SEK	—	—	
Sweden: 15,001 - 25,000 SEK	1.09	0.87, 1.38	0.450
Sweden: 50,001 - 60,000 SEK	1.15	0.93, 1.42	0.208
Sweden: 35,001 - 40,000 SEK	1.05	0.84, 1.31	0.643
Sweden: 60,001 - 70,000 SEK	1.16	0.93, 1.44	0.185
Sweden: 25,001 - 30,000 SEK	1.17	0.93, 1.47	0.180
Sweden: 30,001 - 35,000 SEK	1.06	0.85, 1.33	0.611
Sweden: 70,001 - 80,000 SEK	1.05	0.83, 1.33	0.670
Sweden: 80,001 - 100,000 SEK	1.05	0.83, 1.33	0.689
Sweden: 10,001 - 15,000 SEK	1.49	1.12, 1.98	0.006
Sweden: More than 100,000 SEK	0.76	0.59, 0.97	0.028
(None/No household income)	1.19	0.85, 1.65	0.305
Sweden: 10,000 SEK or less	2.00	1.33, 3.02	9.37e-04

Abbreviations: CI = Confidence Interval, OR = Odds Ratio

Notes. N=15068; attrition weights were estimated using the ’survey::svyglm(family=quasibinomial(‘logit’))’ function. All continuous predictors were standardized and all categorical predictors used the most common category as the reference group. Reported p-values are based on the fitted regression model and no adjustments for multiple testing were done within this table.

Table S27f. Summary of principal components in Sweden

PC	Percent Explained by each PC	Cumulative Percent Explained
1	32.68	32.68
2	7.94	40.62
3	5.55	46.17
4	2.38	48.55
5	2.15	50.70
6	1.72	52.42
**7**	**1.62**	**54.04**
8	1.58	55.62
9	1.47	57.10
10	1.42	58.52
11	1.37	59.88
12	1.33	61.21
13	1.28	62.49
14	1.22	63.71
15	1.15	64.85
16	1.09	65.95
17	1.08	67.03
18	1.01	68.04
19	0.97	69.00
20	0.94	69.95

Notes. N=15068; PCA was conducted using 'survey::svyprcomp(.)' function using all available contemporaneous exposures at wave 1. All PCs were standardized prior to being used as predictors. The bolded row represented the number of retained components for analysis was 7.

Table S27g. Associations of forgivingness with adult well-being and other outcomes at Wave 2 in Sweden

	Model 1: Demographic and Childhood Variables as Covariates					Model 2: Demographic, Childhood, and Other Wave 1 Confounding Variables (Via Principal Components) as Covariates				
Outcome	RR	ES	95% CI	SE	p-value	RR	ES	95% CI	SE	p-value
Human Flourishing										
Secure flourishing index		0.08	(0.06,0.10)	0.01	2.22e-16***		0.01	(-0.00,0.02)	0.01	0.163
Flourishing index		0.09	(0.07,0.11)	0.01	2.22e-16***		0.01	(-0.00,0.02)	0.01	0.185
Happiness & life satisfaction		0.06	(0.04,0.08)	0.01	6.95e-09***		-0.01	(-0.02,0.01)	0.01	0.277
Physical & mental health		0.05	(0.03,0.07)	0.01	7.42e-07***		-0.00	(-0.02,0.01)	0.01	0.570
Meaning & purpose		0.08	(0.06,0.10)	0.01	1.54e-13***		0.00	(-0.01,0.02)	0.01	0.632
Character & virtue		0.10	(0.08,0.13)	0.01	2.22e-16***		0.05	(0.03,0.07)	0.01	4.76e-06***
Close social relationships		0.07	(0.05,0.09)	0.01	2.6e-11***		0.01	(-0.01,0.02)	0.01	0.409
Financial & material security		0.03	(0.01,0.05)	0.01	2.22e-03**		0.01	(-0.01,0.02)	0.01	0.533
Psychological Well-Being										
Happiness (a)		0.06	(0.04,0.08)	0.01	1.3e-08***		-0.01	(-0.02,0.01)	0.01	0.312
Life satisfaction (a)		0.05	(0.03,0.07)	0.01	1.55e-06***		-0.01	(-0.03,0.01)	0.01	0.390
Current life evaluation		0.04	(0.02,0.06)	0.01	1.99e-04***		-0.01	(-0.03,0.01)	0.01	0.177
Future life evaluation		0.06	(0.04,0.08)	0.01	2.36e-09***		0.01	(-0.01,0.03)	0.01	0.263
Optimism		0.09	(0.07,0.11)	0.01	6.66e-16***		0.02	(0.00,0.04)	0.01	0.030*
Freedom to pursue what's important		0.04	(0.02,0.06)	0.01	5.17e-04***		-0.01	(-0.03,0.01)	0.01	0.270
Inner peace	1.02		(1.01,1.03)	0.00	2.82e-06***	1.00		(0.99,1.01)	0.00	0.778
Life balance	1.02		(1.01,1.03)	0.00	1.19e-05***	1.00		(0.99,1.01)	0.00	0.904
Sense of mastery	1.02		(1.01,1.03)	0.00	1.96e-06***	1.00		(0.99,1.01)	0.00	0.618
Meaningful activities (c)		0.06	(0.04,0.08)	0.01	5.4e-09***		-0.00	(-0.02,0.02)	0.01	0.760
Understanding purpose (c)		0.07	(0.05,0.09)	0.01	7.69e-11***		0.01	(-0.01,0.03)	0.01	0.329
Self-rated mental health (b)		0.04	(0.02,0.06)	0.01	4.81e-04***		-0.02	(-0.04,-0.00)	0.01	0.012*
Psychological Distress										
Traumatic distress	0.99		(0.98,1.00)	0.01	0.056	0.99		(0.98,1.00)	0.01	0.132
Depression symptoms composite	0.99		(0.98,1.00)	0.00	0.036*	1.00		(1.00,1.01)	0.00	0.263
Depression – feel hopeless	0.99		(0.98,1.00)	0.00	0.026*	1.01		(1.00,1.01)	0.00	0.114
Depression – loss of interest	0.99		(0.98,1.00)	0.00	0.133	1.00		(0.99,1.01)	0.00	0.621
Anxiety symptoms composite	0.99		(0.98,0.99)	0.00	1.41e-03**	1.00		(0.99,1.01)	0.00	0.868
Anxiety – feel on edge	0.99		(0.98,1.00)	0.00	0.024*	1.00		(1.00,1.01)	0.00	0.296
Anxiety – cannot stop worrying	0.99		(0.98,1.00)	0.00	0.013*	1.00		(0.99,1.01)	0.00	0.618
Suffering	0.99		(0.98,1.00)	0.01	0.024*	1.00		(0.99,1.01)	0.00	0.539
Social Well-Being										
Relationship contentment (e)		0.07	(0.05,0.09)	0.01	2.54e-11***		0.01	(-0.01,0.03)	0.01	0.202
Relationship satisfaction (e)		0.06	(0.04,0.08)	0.01	6.88e-08***		0.00	(-0.02,0.02)	0.01	0.815
Social support		0.10	(0.07,0.12)	0.01	2.22e-16***		0.05	(0.03,0.07)	0.01	2.32e-06***
Intimate/close friend	1.02		(1.01,1.03)	0.00	1.21e-05***	1.01		(1.00,1.01)	0.00	0.179
Government approval	1.00		(0.99,1.01)	0.01	0.359	0.99		(0.98,1.00)	0.01	0.108
Say in government	1.01		(1.00,1.01)	0.00	0.165	1.00		(0.99,1.01)	0.00	0.998
Belonging in country		0.06	(0.04,0.08)	0.01	5.06e-09***		0.04	(0.02,0.06)	0.01	5.99e-04***
City/place satisfaction	1.03		(1.02,1.04)	0.00	1.11e-11***	1.02		(1.01,1.03)	0.00	4.67e-07***
Trust within country	1.03		(1.03,1.04)	0.00	1.8e-12***	1.03		(1.02,1.04)	0.00	9.91e-10***
Social Participation										
Ever been married	1.00		(1.00,1.01)	0.00	0.698	1.00		(1.00,1.01)	0.00	0.795
Currently divorced	1.00		(1.00,1.00)	0.00	0.877	1.00		(1.00,1.00)	0.00	0.908
Number of children		0.01	(-0.01,0.02)	0.01	0.467		0.00	(-0.01,0.02)	0.01	0.628
Weekly+ community participation	1.01		(1.00,1.01)	0.00	0.123	1.00		(0.99,1.01)	0.00	0.922
Weekly+ religious attendance	1.00		(1.00,1.00)	0.00	0.482	1.00		(1.00,1.00)	0.00	0.676
Social Distress										
Loneliness		-0.04	(-0.06,-0.02)	0.01	1.7e-04***		0.01	(-0.01,0.03)	0.01	0.267
Perceived discrimination	0.98		(0.97,0.99)	0.00	9e-09***	0.98		(0.98,0.99)	0.00	6.78e-07***
Character & Prosocial Behavior										
Orientation to promote good (d)		0.12	(0.10,0.14)	0.01	2.22e-16***		0.06	(0.03,0.08)	0.01	1.58e-07***
Delayed gratification (d)		0.06	(0.03,0.08)	0.01	2.03e-06***		0.03	(0.00,0.05)	0.01	0.031*
Hope		0.10	(0.08,0.12)	0.01	2.22e-16***		0.03	(0.01,0.05)	0.01	0.005*
Gratitude		0.09	(0.07,0.11)	0.01	1.58e-14***		0.02	(-0.00,0.04)	0.01	0.086
Showing love/care		0.09	(0.06,0.11)	0.01	1.62e-14***		0.03	(0.01,0.05)	0.01	2.67e-03**
Forgivingness	1.23		(1.22,1.24)	0.00	2.22e-16***	1.23		(1.21,1.24)	0.00	2.22e-16***
Charitable giving	1.02		(1.01,1.03)	0.01	1.17e-05***	1.02		(1.01,1.03)	0.01	1.68e-03**
Helping strangers	1.02		(1.01,1.03)	0.01	5.02e-04***	1.01		(1.00,1.01)	0.00	0.298
Volunteering	1.00		(0.99,1.01)	0.00	0.860	1.00		(0.99,1.00)	0.00	0.186
Physical Health & Health Behavior										
Self-rated physical health (b)		0.05	(0.03,0.07)	0.01	2.83e-06***		0.02	(-0.00,0.03)	0.01	0.117
Health problems	0.99		(0.98,1.00)	0.00	0.019*	0.99		(0.99,1.00)	0.00	0.190
Pain in past 4 weeks	0.99		(0.98,1.00)	0.01	0.068	0.99		(0.99,1.00)	0.00	0.256
Daily smoker	1.00		(0.99,1.00)	0.00	0.451	1.00		(0.99,1.00)	0.00	0.580
Number of drinks per week		-0.00	(-0.02,0.02)	0.01	0.712		-0.00	(-0.02,0.02)	0.01	0.987
Days exercise per week		0.01	(-0.01,0.03)	0.01	0.434		-0.00	(-0.03,0.02)	0.01	0.709
Socioeconomic Outcomes										
Financial security (f)		0.01	(-0.00,0.03)	0.01	0.148		-0.00	(-0.02,0.02)	0.01	0.828
Material security (f)		0.04	(0.02,0.06)	0.01	1.32e-04***		0.01	(-0.01,0.03)	0.01	0.196
Educational attainment (16+ years)	1.00		(1.00,1.00)	0.00	0.986	1.00		(1.00,1.00)	0.00	0.796
Currently employed	1.00		(0.99,1.01)	0.00	0.775	1.00		(0.99,1.01)	0.00	0.819
Financially comfortable/getting by	1.00		(1.00,1.01)	0.00	0.650	1.00		(0.99,1.00)	0.00	0.684
Own home	0.98		(0.98,0.99)	0.00	6.44e-04**	0.99		(0.98,1.00)	0.00	2.29e-03**
Income – top quintile	0.99		(0.98,1.00)	0.00	0.127	0.99		(0.98,1.00)	0.00	0.082
Religion & Spirituality										
Religious/spiritual connection	1.01		(1.01,1.02)	0.00	4.5e-04***	1.00		(0.99,1.01)	0.00	0.957
Belief in life after death	1.01		(1.00,1.02)	0.00	0.038*	0.99		(0.99,1.00)	0.00	0.144
Transformative religious experience	1.01		(1.00,1.01)	0.00	0.058	1.00		(0.99,1.01)	0.00	0.946
Religious reading or listening	1.00		(1.00,1.01)	0.00	0.623	1.00		(1.00,1.01)	0.00	0.612
Prayer or meditation	1.01		(1.00,1.01)	0.00	0.018*	1.00		(1.00,1.01)	0.00	0.200
Belief in God/gods/spiritual forces	1.01		(1.01,1.02)	0.00	2.07e-03**	1.00		(0.99,1.01)	0.00	0.837
Religious centrality	1.01		(1.00,1.01)	0.00	0.008*	1.00		(1.00,1.01)	0.00	0.400
Religious/spiritual comfort	1.02		(1.01,1.02)	0.00	3.82e-06***	1.00		(1.00,1.01)	0.00	0.125
Feel loved by God	1.01		(1.01,1.02)	0.00	1.21e-04***	1.00		(1.00,1.01)	0.00	0.366
Feel punished by God	0.99		(0.99,1.00)	0.00	0.007*	0.99		(0.99,1.00)	0.00	0.011*
Experienced religious criticism	1.00		(0.99,1.00)	0.00	0.069	1.00		(0.99,1.00)	0.00	0.109
Faith-sharing	1.01		(1.00,1.02)	0.00	2.68e-03**	1.00		(1.00,1.01)	0.00	0.342

Notes. N=15068; Reference for focal predictor: never/rarely. RR, risk-ratio, null effect is 1.00; ES, effect size measure for standardized regression coefficient, null effect is 0.00; SE, standard error, the SE reported for binary/Likert-type outcomes where risk-ratios are on the log(RR) scale; CI, confidence interval; p-value, a Wald-type test of the null hypothesis that the effect of the focal predictor is zero; (a) item part of the Happiness & Life Satisfaction domain of the Secure Flourishing Index; (b) item part of the Physical & Mental Health domain of the Secure Flourishing Index; (c) item part of the Meaning & Purpose domain of the Secure Flourishing Index; (d) item part of the Character & Virtue domain of the Secure Flourishing Index; (e) item part of the Subjective Social Connectedness domain of the Secure Flourishing Index; (f) item part of the Financial & Material Security domain of the Secure Flourishing Index.

Multiple imputation was performed to impute missing data on the covariates, exposure, and outcomes. All models controlled for sociodemographic and childhood factors assessed at Wave 1. For Model 2 with PC (principal components), the first seven principal components of the entire set of contemporaneous confounders assessed at Wave 1 were included as additional covariates of the outcomes at Wave 2.

An outcome-wide analytic approach was used, and a separate model was run for each outcome. A different type of model was run depending on the nature of the outcome: (1) for each binary outcome, a weighted generalized linear model (with a log link and Poisson distribution) was used to estimate an RR; and (2) for each continuous outcome, a weighted linear regression model was used to estimate a ES. All effect sizes were standardized. For continuous outcomes, the ES represents the change in SD on the outcome between the lower and upper categories of the binary focal predictor. For binary outcomes, the RR represents the change in risk of being in the upper category compared to the lower category between the lower and upper categories of the binary focal predictor.

P-value significance thresholds: $p < 0.05$*, $p < 0.005$**, (Bonferroni) $p < 6.41e-04$***, correction for multiple testing using Bonferroni adjusted significant threshold.

Table S27h. Associations of forgivingness with adult well-being and other outcomes at Wave 2 in Sweden using complete-case analyses with attrition weights.

	Model 1: Demographic and Childhood Variables as Covariates					Model 2: Demographic, Childhood, and Other Wave 1 Confounding Variables (Via Principal Components) as Covariates				
Outcome	RR	ES	95% CI	SE	p-value	RR	ES	95% CI	SE	p-value
Human Flourishing										
Secure flourishing index		0.08	(0.06,0.10)	0.01	6.56e-13***		0.01	(-0.01,0.02)	0.01	0.441
Flourishing index		0.09	(0.06,0.11)	0.01	4.66e-14***		0.01	(-0.01,0.02)	0.01	0.442
Happiness & life satisfaction		0.05	(0.03,0.07)	0.01	4.75e-06***		-0.01	(-0.03,0.01)	0.01	0.163
Physical & mental health		0.05	(0.03,0.07)	0.01	1.46e-05***		-0.01	(-0.03,0.01)	0.01	0.196
Meaning & purpose		0.07	(0.05,0.10)	0.01	5.41e-11***		0.00	(-0.02,0.02)	0.01	0.761
Character & virtue		0.10	(0.08,0.12)	0.01	2.22e-16***		0.04	(0.02,0.06)	0.01	2.1e-04***
Close social relationships		0.08	(0.05,0.10)	0.01	1.37e-10***		0.01	(-0.01,0.04)	0.01	0.164
Financial & material security		0.02	(0.00,0.05)	0.01	0.027*		0.00	(-0.02,0.02)	0.01	0.764
Psychological Well-Being										
Happiness (a)		0.05	(0.03,0.07)	0.01	6.41e-06***		-0.01	(-0.03,0.01)	0.01	0.152
Life satisfaction (a)		0.05	(0.03,0.07)	0.01	2.11e-05***		-0.01	(-0.03,0.01)	0.01	0.227
Current life evaluation		0.04	(0.02,0.06)	0.01	5.09e-04***		-0.02	(-0.04,0.00)	0.01	0.134
Future life evaluation		0.07	(0.04,0.09)	0.01	6.23e-09***		0.01	(-0.01,0.03)	0.01	0.353
Optimism		0.09	(0.07,0.12)	0.01	1.67e-14***		0.02	(0.00,0.05)	0.01	0.022*
Freedom to pursue what's important		0.05	(0.02,0.07)	0.01	7.72e-05***		-0.00	(-0.02,0.02)	0.01	0.893
Inner peace	1.02		(1.01,1.03)	0.00	6.31e-05***	1.00		(0.99,1.01)	0.00	0.776
Life balance	1.02		(1.01,1.03)	0.00	3.38e-05***	1.00		(0.99,1.01)	0.00	0.910
Sense of mastery	1.02		(1.01,1.03)	0.00	9.47e-06***	1.00		(0.99,1.01)	0.00	0.650
Meaningful activities (c)		0.06	(0.04,0.09)	0.01	3.37e-08***		-0.00	(-0.02,0.02)	0.01	0.756
Understanding purpose (c)		0.07	(0.05,0.09)	0.01	1.12e-09***		0.01	(-0.01,0.03)	0.01	0.453
Self-rated mental health (b)		0.03	(0.01,0.05)	0.01	0.008*		-0.03	(-0.05,-0.01)	0.01	2.43e-03**
Psychological Distress										
Traumatic distress	0.99		(0.98,1.00)	0.01	0.098	0.99		(0.98,1.00)	0.01	0.190
Depression symptoms composite	0.99		(0.98,1.00)	0.00	0.106	1.00		(1.00,1.01)	0.00	0.325
Depression – feel hopeless	0.99		(0.98,1.00)	0.00	0.060	1.01		(1.00,1.01)	0.00	0.212
Depression – loss of interest	0.99		(0.98,1.00)	0.00	0.100	1.00		(0.99,1.01)	0.00	0.949
Anxiety symptoms composite	0.99		(0.98,0.99)	0.00	2.05e-03**	1.00		(0.99,1.01)	0.00	0.621
Anxiety – feel on edge	0.99		(0.98,1.00)	0.00	0.042*	1.00		(0.99,1.01)	0.00	0.582
Anxiety – cannot stop worrying	0.99		(0.98,1.00)	0.00	0.019*	1.00		(0.99,1.01)	0.00	0.967
Suffering	0.99		(0.98,1.00)	0.01	0.047*	1.01		(1.00,1.02)	0.00	0.211
Social Well-Being										
Relationship contentment (e)		0.08	(0.06,0.10)	0.01	2.7e-11***		0.02	(-0.00,0.04)	0.01	0.083
Relationship satisfaction (e)		0.07	(0.04,0.09)	0.01	1.66e-08***		0.01	(-0.01,0.03)	0.01	0.354
Social support		0.10	(0.08,0.13)	0.01	2.22e-16***		0.06	(0.03,0.08)	0.01	1.01e-06***
Intimate/close friend	1.02		(1.01,1.03)	0.00	2.82e-05***	1.00		(1.00,1.01)	0.00	0.284
Government approval	0.99		(0.98,1.00)	0.01	0.213	0.99		(0.98,1.00)	0.01	0.028*
Say in government	1.01		(1.00,1.01)	0.00	0.242	1.00		(0.99,1.01)	0.00	0.734
Belonging in country		0.07	(0.04,0.09)	0.01	8.45e-08***		0.04	(0.02,0.07)	0.01	8.01e-04**
City/place satisfaction	1.03		(1.02,1.04)	0.00	1.79e-10***	1.02		(1.02,1.03)	0.00	5.42e-07***
Trust within country	1.04		(1.03,1.05)	0.01	1.7e-12***	1.03		(1.02,1.04)	0.01	5.14e-10***
Social Participation										
Ever been married	1.00		(1.00,1.01)	0.00	0.729	1.00		(1.00,1.01)	0.00	0.810
Currently divorced	1.00		(1.00,1.00)	0.00	0.868	1.00		(1.00,1.00)	0.00	0.923
Number of children		0.00	(-0.02,0.02)	0.01	0.900		-0.00	(-0.02,0.02)	0.01	0.975
Weekly+ community participation	1.01		(1.00,1.02)	0.00	0.106	1.00		(0.99,1.01)	0.00	0.753
Weekly+ religious attendance	1.00		(1.00,1.01)	0.00	0.568	1.00		(1.00,1.00)	0.00	0.710
Social Distress										
Loneliness		-0.04	(-0.06,-0.02)	0.01	8.74e-04**		0.01	(-0.01,0.03)	0.01	0.263
Perceived discrimination	0.98		(0.97,0.98)	0.00	1.78e-08***	0.98		(0.97,0.99)	0.00	2.11e-07***
Character & Prosocial Behavior										
Orientation to promote good (d)		0.12	(0.09,0.14)	0.01	2.22e-16***		0.05	(0.03,0.08)	0.01	5.45e-06***
Delayed gratification (d)		0.05	(0.03,0.08)	0.01	6.02e-06***		0.02	(-0.00,0.04)	0.01	0.082
Hope		0.10	(0.07,0.12)	0.01	1.11e-15***		0.02	(0.00,0.05)	0.01	0.023*
Gratitude		0.09	(0.06,0.11)	0.01	1.85e-13***		0.02	(-0.00,0.04)	0.01	0.120
Showing love/care		0.09	(0.06,0.11)	0.01	4.6e-12***		0.03	(0.01,0.05)	0.01	0.010*
Forgivingness	1.23		(1.21,1.24)	0.01	2.22e-16***	1.22		(1.21,1.24)	0.01	2.22e-16***
Charitable giving	1.02		(1.01,1.03)	0.01	1.79e-05***	1.02		(1.01,1.03)	0.01	6.51e-04**
Helping strangers	1.02		(1.01,1.03)	0.01	3.35e-03**	1.00		(0.99,1.01)	0.01	0.571
Volunteering	1.00		(0.99,1.01)	0.00	0.877	1.00		(0.99,1.00)	0.00	0.265
Physical Health & Health Behavior										
Self-rated physical health (b)		0.05	(0.03,0.08)	0.01	2.44e-06***		0.01	(-0.01,0.03)	0.01	0.328
Health problems	0.99		(0.98,1.00)	0.01	0.005*	1.00		(0.99,1.00)	0.00	0.288
Pain in past 4 weeks	0.99		(0.98,1.00)	0.01	0.081	1.00		(0.99,1.01)	0.01	0.575
Daily smoker	0.99		(0.99,1.00)	0.00	0.142	0.99		(0.99,1.00)	0.00	0.091
Number of drinks per week		0.00	(-0.02,0.03)	0.01	0.854		0.00	(-0.02,0.03)	0.01	0.697
Days exercise per week		0.01	(-0.02,0.03)	0.01	0.494		-0.01	(-0.03,0.01)	0.01	0.420
Socioeconomic Outcomes										
Financial security (f)		0.01	(-0.01,0.04)	0.01	0.219		-0.00	(-0.02,0.02)	0.01	0.986
Material security (f)		0.03	(0.01,0.06)	0.01	4.94e-03**		0.01	(-0.02,0.03)	0.01	0.559
Educational attainment (16+ years)	1.00		(1.00,1.00)	0.00	0.721	1.00		(1.00,1.00)	0.00	0.991
Currently employed	1.00		(1.00,1.01)	0.00	0.621	1.00		(0.99,1.01)	0.00	0.947
Financially comfortable/getting by	1.00		(0.99,1.01)	0.00	0.909	1.00		(0.99,1.00)	0.00	0.571
Own home	0.98		(0.97,0.99)	0.01	6.75e-04**	0.99		(0.98,1.00)	0.01	0.007*
Income – top quintile	0.99		(0.98,1.00)	0.01	0.197	0.99		(0.98,1.00)	0.01	0.141
Religion & Spirituality										
Religious/spiritual connection	1.01		(1.01,1.02)	0.00	1.33e-03**	1.00		(0.99,1.01)	0.00	0.815
Belief in life after death	1.01		(1.00,1.02)	0.01	0.008*	1.00		(0.99,1.01)	0.00	0.554
Transformative religious experience	1.01		(1.00,1.02)	0.00	0.025*	1.00		(0.99,1.01)	0.00	0.835
Religious reading or listening	1.00		(0.99,1.00)	0.00	0.847	1.00		(0.99,1.00)	0.00	0.855
Prayer or meditation	1.01		(1.00,1.01)	0.00	0.035*	1.00		(1.00,1.01)	0.00	0.370
Belief in God/gods/spiritual forces	1.02		(1.01,1.03)	0.01	8.52e-04**	1.00		(0.99,1.01)	0.00	0.686
Religious centrality	1.01		(1.00,1.01)	0.00	0.017*	1.00		(1.00,1.01)	0.00	0.630
Religious/spiritual comfort	1.02		(1.01,1.03)	0.00	1.25e-05***	1.00		(1.00,1.01)	0.00	0.188
Feel loved by God	1.01		(1.00,1.02)	0.00	1.86e-03**	1.00		(0.99,1.01)	0.00	0.950
Feel punished by God	0.99		(0.99,1.00)	0.00	3.43e-03**	0.99		(0.99,1.00)	0.00	3.02e-03**
Experienced religious criticism	1.00		(0.99,1.00)	0.00	0.044*	1.00		(0.99,1.00)	0.00	0.056
Faith-sharing	1.01		(1.00,1.02)	0.00	0.012*	1.00		(0.99,1.01)	0.00	0.666

Table S27h. Associations of forgivingness with adult well-being and other outcomes at Wave 2 in Sweden using complete-case analyses with attrition weights.

	Model 1: Demographic and Childhood Variables as Covariates					Model 2: Demographic, Childhood, and Other Wave 1 Confounding Variables (Via Principal Components) as Covariates				
Outcome	RR	ES	95% CI	SE	p-value	RR	ES	95% CI	SE	p-value

Notes. N=11609; Reference for focal predictor: never/rarely. RR, risk-ratio, null effect is 1.00; ES, effect size measure for standardized regression coefficient, null effect is 0.00; SE, standard error, the SE reported for binary/Likert-type outcomes where risk-ratios are on the log(RR) scale; CI, confidence interval; p-value, a Wald-type test of the null hypothesis that the effect of the focal predictor is zero; (a) item part of the Happiness & Life Satisfaction domain of the Secure Flourishing Index; (b) item part of the Physical & Mental Health domain of the Secure Flourishing Index; (c) item part of the Meaning & Purpose domain of the Secure Flourishing Index; (d) item part of the Character & Virtue domain of the Secure Flourishing Index; (e) item part of the Subjective Social Connectedness domain of the Secure Flourishing Index; (f) item part of the Financial & Material Security domain of the Secure Flourishing Index.

Attrition weights were computed to adjust the complete case data (those who responded at Wave 2 to at least 50% of the questions) and multiple imputation was used to impute missing data on all remaining within wave on the covariates, exposure, and outcomes. All models controlled for sociodemographic and childhood factors assessed at Wave 1. For Model 2 with PC (principal components), the first seven principal components of the entire set of contemporaneous confounders assessed at Wave 1 were included as additional covariates of the outcomes at Wave 2.

An outcome-wide analytic approach was used, and a separate model was run for each outcome. A different type of model was run depending on the nature of the outcome: (1) for each binary outcome, a weighted generalized linear model (with a log link and Poisson distribution) was used to estimate an RR; and (2) for each continuous outcome, a weighted linear regression model was used to estimate a ES. All effect sizes were standardized. For continuous outcomes, the ES represents the change in SD on the outcome between the lower and upper categories of the binary focal predictor. For binary outcomes, the RR represents the change in risk of being in the upper category compared to the lower category between the lower and upper categories of the binary focal predictor.

P-value significance thresholds: $p < 0.05$*, $p < 0.005$**, (Bonferroni) $p < 6.41e-04$***, correction for multiple testing using Bonferroni adjusted significant threshold.

Table S27i. Sensitivity analysis of forgivingness outcome-wide results to unmeasured confounding using E-values in Sweden

	Multiple Imputation				Complete Case w/ Attrition Weights			
	Model 1: Demographics and Childhood Variables as Covariates		Model 2: Demographics, Childhood, and Other Wave 1 Confounders (Via Principal Components) as Covariates		Model 1: Demographics and Childhood Variables as Covariates		Model 2: Demographics, Childhood, and Other Wave 1 Confounders (Via Principal Components) as Covariates	
Outcome	EE	ECI	EE	ECI	EE	ECI	EE	ECI
Human Flourishing								
Secure flourishing index	1.67	1.56	1.16	1.00	1.65	1.52	1.14	1.00
Flourishing index	1.71	1.59	1.16	1.00	1.69	1.55	1.14	1.00
Happiness & life satisfaction	1.51	1.39	1.15	1.00	1.48	1.33	1.20	1.00
Physical & mental health	1.48	1.34	1.11	1.00	1.45	1.31	1.19	1.00
Meaning & purpose	1.63	1.50	1.10	1.00	1.63	1.48	1.09	1.00
Character & virtue	1.81	1.68	1.45	1.31	1.78	1.63	1.42	1.26
Close social relationships	1.60	1.47	1.14	1.00	1.64	1.49	1.21	1.00
Financial & material security	1.32	1.17	1.12	1.00	1.29	1.08	1.09	1.00
Psychological Well-Being								
Happiness	1.51	1.38	1.15	1.00	1.48	1.33	1.21	1.00
Life satisfaction	1.48	1.34	1.15	1.00	1.46	1.31	1.19	1.00
Current life evaluation	1.40	1.25	1.20	1.00	1.40	1.24	1.22	1.00
Future life evaluation	1.54	1.41	1.18	1.00	1.57	1.43	1.17	1.00
Optimism	1.71	1.58	1.26	1.07	1.74	1.59	1.29	1.10
Freedom to pursue what’s important	1.39	1.23	1.18	1.00	1.45	1.29	1.06	1.00
Inner peace	1.27	1.20	1.05	1.00	1.26	1.18	1.06	1.00
Life balance	1.28	1.20	1.04	1.00	1.28	1.19	1.04	1.00
Sense of mastery	1.27	1.20	1.07	1.00	1.27	1.19	1.07	1.00
Meaningful activities	1.55	1.42	1.08	1.00	1.56	1.41	1.09	1.00
Understanding purpose	1.60	1.47	1.16	1.00	1.60	1.46	1.15	1.00
Self-rated mental health	1.37	1.22	1.27	1.11	1.33	1.15	1.32	1.17
Psychological Distress								
Traumatic distress	1.18	1.00	1.15	1.00	1.17	1.00	1.14	1.00
Depression symptoms composite	1.17	1.04	1.12	1.00	1.16	1.00	1.12	1.00
Depression – feel hopeless	1.16	1.05	1.13	1.00	1.16	1.00	1.12	1.00
Depression – loss of interest	1.14	1.00	1.08	1.00	1.16	1.00	1.03	1.00
Anxiety symptoms composite	1.21	1.12	1.04	1.00	1.22	1.12	1.08	1.00
Anxiety – feel on edge	1.17	1.06	1.10	1.00	1.17	1.03	1.08	1.00
Anxiety – cannot stop worrying	1.17	1.07	1.07	1.00	1.18	1.07	1.02	1.00
Suffering	1.20	1.07	1.09	1.00	1.19	1.02	1.14	1.00
Social Well-Being								
Relationship contentment	1.61	1.48	1.19	1.00	1.66	1.51	1.25	1.00
Relationship satisfaction	1.53	1.39	1.08	1.00	1.58	1.43	1.17	1.00
Social support	1.76	1.63	1.47	1.33	1.81	1.65	1.52	1.37
Intimate/close friend	1.26	1.18	1.13	1.00	1.25	1.17	1.11	1.00
Government approval	1.12	1.00	1.16	1.00	1.14	1.00	1.20	1.06
Say in government	1.14	1.00	1.00	1.00	1.13	1.00	1.06	1.00
Belonging in country	1.56	1.42	1.37	1.22	1.58	1.42	1.41	1.23
City/place satisfaction	1.35	1.28	1.29	1.21	1.37	1.29	1.31	1.23
Trust within country	1.38	1.31	1.34	1.27	1.40	1.32	1.37	1.29
Social Participation								
Ever been married	1.05	1.00	1.04	1.00	1.05	1.00	1.04	1.00
Currently divorced	1.03	1.00	1.02	1.00	1.03	1.00	1.02	1.00
Number of children	1.13	1.00	1.11	1.00	1.06	1.00	1.03	1.00
Weekly+ community participation	1.14	1.00	1.03	1.00	1.15	1.00	1.06	1.00
Weekly+ religious attendance	1.05	1.00	1.04	1.00	1.05	1.00	1.04	1.00
Social Distress								
Loneliness	1.40	1.25	1.18	1.00	1.39	1.22	1.18	1.00
Perceived discrimination	1.28	1.21	1.25	1.19	1.30	1.23	1.28	1.21
Character & Prosocial Behavior								
Orientation to promote good	1.90	1.76	1.50	1.37	1.90	1.74	1.48	1.33
Delayed gratification	1.52	1.36	1.30	1.08	1.50	1.34	1.26	1.00
Hope	1.78	1.64	1.32	1.16	1.76	1.62	1.29	1.09
Gratitude	1.70	1.57	1.23	1.00	1.71	1.56	1.23	1.00
Showing love/care	1.69	1.56	1.34	1.18	1.70	1.55	1.34	1.14
Forgivingness	2.64	2.56	2.61	2.53	2.62	2.53	2.59	2.51
Charitable giving	1.29	1.20	1.24	1.13	1.30	1.21	1.26	1.16
Helping strangers	1.25	1.15	1.12	1.00	1.24	1.13	1.09	1.00
Volunteering	1.04	1.00	1.12	1.00	1.04	1.00	1.11	1.00
Physical Health & Health Behavior								
Self-rated physical health	1.47	1.33	1.22	1.00	1.49	1.34	1.17	1.00
Health problems	1.19	1.07	1.13	1.00	1.22	1.11	1.12	1.00
Pain in past 4 weeks	1.17	1.00	1.13	1.00	1.18	1.00	1.09	1.00
Daily smoker	1.08	1.00	1.07	1.00	1.13	1.00	1.14	1.00
Number of drinks per week	1.10	1.00	1.02	1.00	1.07	1.00	1.11	1.00
Days exercise per week	1.16	1.00	1.10	1.00	1.15	1.00	1.17	1.00
Socioeconomic Outcomes								
Financial security	1.21	1.00	1.07	1.00	1.20	1.00	1.02	1.00
Material security	1.40	1.26	1.20	1.00	1.35	1.17	1.13	1.00
Educational attainment (16+ years)	1.01	1.00	1.04	1.00	1.04	1.00	1.01	1.00
Currently employed	1.05	1.00	1.04	1.00	1.07	1.00	1.02	1.00
Financially comfortable/getting by	1.06	1.00	1.06	1.00	1.03	1.00	1.07	1.00
Own home	1.23	1.14	1.21	1.12	1.25	1.15	1.22	1.10
Income – top quintile	1.14	1.00	1.16	1.00	1.14	1.00	1.15	1.00
Religion & Spirituality								
Religious/spiritual connection	1.22	1.14	1.02	1.00	1.23	1.13	1.05	1.00
Belief in life after death	1.17	1.04	1.13	1.00	1.21	1.10	1.09	1.00
Transformative religious experience	1.14	1.00	1.02	1.00	1.16	1.05	1.04	1.00
Religious reading or listening	1.05	1.00	1.05	1.00	1.03	1.00	1.03	1.00
Prayer or meditation	1.14	1.05	1.10	1.00	1.14	1.04	1.09	1.00
Belief in God/gods/spiritual forces	1.23	1.13	1.05	1.00	1.26	1.15	1.07	1.00
Religious centrality	1.16	1.07	1.08	1.00	1.16	1.06	1.06	1.00
Religious/spiritual comfort	1.24	1.18	1.11	1.00	1.25	1.18	1.11	1.00
Feel loved by God	1.22	1.14	1.09	1.00	1.21	1.12	1.02	1.00
Feel punished by God	1.14	1.07	1.13	1.06	1.15	1.08	1.16	1.09
Experienced religious criticism	1.09	1.00	1.09	1.00	1.12	1.02	1.11	1.00
Faith-sharing	1.20	1.11	1.10	1.00	1.19	1.08	1.07	1.00

Notes. EE, E-value for estimate; ECI, E-value for the limit of the confidence interval. The formula for calculating E-values can be found in VanderWeele and Ding (2017). E-values for estimate are the minimum strength of association on the risk ratio scale that an unmeasured confounder would need to have with both the exposure and the outcome to fully explain away the observed association between the exposure and outcome, conditional on the measured covariates. E-values for the 95% CI closest to the null denote the minimum strength of association on the risk ratio scale that an unmeasured confounder would need to have with both the exposure and the outcome to shift the CI to include the null value, conditional on the measured covariates.

Table S28a. Weighted summary statistics for demographic and childhood variables in Tanzania

**Characteristic**	**Wave 1** N = 9,075	**Wave 2** N = 5,579
Forgivingness, n (%)		
Always	4,626 (51.0%)	2,381 (42.7%)
Often	2,961 (32.6%)	2,168 (38.9%)
Rarely	1,304 (14.4%)	916 (16.4%)
Never	169 (1.9%)	114 (2.0%)
(Missing)	16 (0.2%)	0 (<0.0%)
Year of birth, n (%)		
1943 or earlier (current age: 80+ years)	85 (0.9%)	59 (1.1%)
1943-1953 (current age: 70-79 years)	275 (3.0%)	183 (3.3%)
1953-1963 (current age: 60-69 years)	527 (5.8%)	366 (6.6%)
1963-1973 (current age: 50-59 years)	839 (9.2%)	544 (9.8%)
1973-1983 (current age: 40-49 years)	1,425 (15.7%)	934 (16.7%)
1983-1993 (current age: 30-39 years)	1,985 (21.9%)	1,318 (23.6%)
1993-1998 (current age: 25-29 years)	1,259 (13.9%)	841 (15.1%)
1998-2005 (current age: 18-24 years)	2,678 (29.5%)	1,334 (23.9%)
(Missing)	2 (0.0%)	0 (0%)
Age of participant		
Mean	36.0	37.6
Standard Deviation	15.4	15.2
Min, Max	18.0, 99.0	19.0, 99.0
(Missing)	2 (<0.1%)	0 (0%)
Gender, n (%)		
Male	4,263 (47.0%)	2,626 (47.1%)
Female	4,812 (53.0%)	2,953 (52.9%)
Other	0 (0%)	0 (0%)
(Missing)	0 (0%)	0 (0%)
Respondent marital status, n (%)		
Single/Never been married	2,425 (26.7%)	1,323 (23.7%)
Married	5,461 (60.2%)	3,205 (57.5%)
Separated	388 (4.3%)	345 (6.2%)
Divorced	98 (1.1%)	75 (1.3%)
Widowed	422 (4.6%)	312 (5.6%)
Domestic partner	274 (3.0%)	311 (5.6%)
(Missing)	7 (0.1%)	8 (0.1%)
Education (years), n (%)		
Up to 8	6,848 (75.5%)	4,176 (74.9%)
9-15	2,123 (23.4%)	1,317 (23.6%)
16+	103 (1.1%)	86 (1.5%)
(Missing)	2 (0.0%)	0 (0%)
Employment status, n (%)		
Employed for an employer	493 (5.4%)	417 (7.5%)
Self-employed	4,553 (50.2%)	3,098 (55.5%)
Retired	117 (1.3%)	69 (1.2%)
Student	365 (4.0%)	146 (2.6%)
Homemaker	1,793 (19.8%)	1,106 (19.8%)
Unemployed and looking for a job	1,557 (17.2%)	660 (11.8%)
None of these/Other	190 (2.1%)	72 (1.3%)
(Missing)	6 (0.1%)	11 (0.2%)
Current religious service attendance, n (%)		
More than once a week	2,566 (28.3%)	1,905 (34.2%)

Table S28a. Weighted summary statistics for demographic and childhood variables in Tanzania

**Characteristic**	**Wave 1** N = 9,075	**Wave 2** N = 5,579
Once a week	4,286 (47.2%)	2,277 (40.8%)
One to three times a month	1,101 (12.1%)	539 (9.7%)
A few times a year	831 (9.2%)	637 (11.4%)
Never	289 (3.2%)	216 (3.9%)
(Missing)	1 (0.0%)	4 (0.1%)
Immigration status, n (%)		
Born in this country	9,046 (99.7%)	5,563 (99.7%)
Born in another country	28 (0.3%)	13 (0.2%)
(Missing)	2 (0.0%)	2 (0.0%)
Parental marital status around age 12, n (%)		
Parents were married	6,901 (76.0%)	4,250 (76.2%)
Parents were divorced	695 (7.7%)	460 (8.2%)
Parents were never married	773 (8.5%)	466 (8.4%)
One or both of them had died	311 (3.4%)	193 (3.5%)
Unsure	46 (0.5%)	26 (0.5%)
(Missing)	349 (3.9%)	184 (3.3%)
Religious service attendance around age 12, n (%)		
At least once a week	5,559 (61.3%)	3,469 (62.2%)
One to three times a month	2,395 (26.4%)	1,452 (26.0%)
Less than once a month	337 (3.7%)	207 (3.7%)
Never	598 (6.6%)	338 (6.1%)
(Missing)	186 (2.0%)	113 (2.0%)
Relationship with mother when growing up, n (%)		
Very good	7,726 (85.1%)	4,708 (84.4%)
Somewhat good	809 (8.9%)	541 (9.7%)
Somewhat bad	85 (0.9%)	56 (1.0%)
Very bad	88 (1.0%)	47 (0.8%)
(Does not apply)	298 (3.3%)	177 (3.2%)
(Missing)	68 (0.8%)	50 (0.9%)
Relationship with father when growing up, n (%)		
Very good	6,801 (74.9%)	4,149 (74.4%)
Somewhat good	1,109 (12.2%)	716 (12.8%)
Somewhat bad	211 (2.3%)	132 (2.4%)
Very bad	257 (2.8%)	179 (3.2%)
(Does not apply)	552 (6.1%)	319 (5.7%)
(Missing)	145 (1.6%)	85 (1.5%)
Felt like an outsider in family when growing up, n (%)		
Yes	738 (8.1%)	444 (8.0%)
No	8,316 (91.6%)	5,124 (91.8%)
(Missing)	21 (0.2%)	11 (0.2%)
Experienced abuse when growing up, n (%)		
Yes	723 (8.0%)	498 (8.9%)
No	8,322 (91.7%)	5,063 (90.8%)
(Missing)	30 (0.3%)	17 (0.3%)
Self-rated health when growing up, n (%)		
Excellent	2,410 (26.6%)	1,519 (27.2%)
Very good	2,019 (22.2%)	1,264 (22.6%)
Good	2,943 (32.4%)	1,795 (32.2%)
Fair	1,182 (13.0%)	719 (12.9%)
Poor	466 (5.1%)	256 (4.6%)

Table S28a. Weighted summary statistics for demographic and childhood variables in Tanzania

**Characteristic**	**Wave 1** N = 9,075	**Wave 2** N = 5,579
(Missing)	55 (0.6%)	27 (0.5%)
Subjective financial status of family growing up, n (%)		
Lived comfortably	2,623 (28.9%)	1,626 (29.2%)
Got by	2,894 (31.9%)	1,754 (31.4%)
Found it difficult	2,686 (29.6%)	1,660 (29.8%)
Found it very difficult	811 (8.9%)	508 (9.1%)
(Missing)	61 (0.7%)	30 (0.5%)
Religious affiliation growing up, n (%)		
Christianity	5,650 (62.3%)	3,409 (61.1%)
Taoism	0 (0%)	0 (0%)
Confucianism	0 (0%)	0 (0%)
Primal, Animist, or Folk religion	11 (0.1%)	9 (0.2%)
Spiritism	0 (0%)	0 (0%)
Umbanda, Candomblé, and other African-derived religions	0 (0%)	0 (0%)
Chinese folk/traditional religion	0 (0%)	0 (0%)
Islam	3,062 (33.7%)	1,985 (35.6%)
Hinduism	0 (0%)	0 (0%)
Buddhism	0 (0%)	0 (0%)
Judaism	0 (0%)	0 (0%)
Sikhism	0 (0%)	0 (0%)
Baha’i	1 (<0.0%)	0 (0%)
Jainism	0 (0%)	0 (0%)
Shinto	0 (0%)	0 (0%)
Some other religion	0 (0%)	0 (0%)
No religion/Atheist/Agnostic	344 (3.8%)	171 (3.1%)
(Missing)	7 (0.1%)	4 (0.1%)

Note. N (%); this table is based on non-imputed data. Cumulative percentages for variables may not add up to 100% due to rounding. Wave 1 characteristics weighted using the Gallup provided sampling weight, ANNUAL_WEIGHT_R2; Wave 2 characteristics weighted accounting for attrition by using the adjusted Wave 1 weight, ANNUAL_WEIGHT_R2, multiplied by the created attrition weight to account for dropout, to maintain nationally representative estimates for Wave 2 characteristics.

Table S28b. Weighted summary statistics for outcome variables in Tanzania

Outcome	Wave 1<br>N = 9,075	Wave 2<br>N = 5,579
Secure flourishing index		
Mean	7.2	7.1
Standard Deviation	1.8	1.9
Min, Max	0.0, 10.0	0.0, 10.0
(Missing)	237 (2.6%)	42 (0.7%)
Flourishing index		
Mean	7.5	7.3
Standard Deviation	1.8	1.9
Min, Max	0.0, 10.0	0.0, 10.0
(Missing)	222 (2.5%)	37 (0.7%)
Happiness & life satisfaction		
Mean	6.0	5.7
Standard Deviation	3.0	3.1
Min, Max	0.0, 10.0	0.0, 10.0
(Missing)	22 (0.2%)	4 (<0.1%)
Physical & mental health		
Mean	8.4	8.1
Standard Deviation	2.2	2.4
Min, Max	0.0, 10.0	0.0, 10.0
(Missing)	41 (0.4%)	13 (0.2%)
Meaning & purpose		
Mean	7.7	7.4
Standard Deviation	2.5	2.7
Min, Max	0.0, 10.0	0.0, 10.0
(Missing)	113 (1.3%)	8 (0.1%)
Character & virtue		
Mean	7.8	7.7
Standard Deviation	2.4	2.6
Min, Max	0.0, 10.0	0.0, 10.0
(Missing)	45 (0.5%)	16 (0.3%)
Close social relationships		
Mean	7.6	7.6
Standard Deviation	2.8	2.9
Min, Max	0.0, 10.0	0.0, 10.0
(Missing)	36 (0.4%)	5 (<0.1%)
Financial & material security		
Mean	5.8	6.0
Standard Deviation	3.3	3.4
Min, Max	0.0, 10.0	0.0, 10.0
(Missing)	21 (0.2%)	4 (<0.1%)
Happiness		
Mean	6.6	6.3
Standard Deviation	3.3	3.4
Min, Max	0.0, 10.0	0.0, 10.0
(Missing)	14 (0.2%)	2 (<0.1%)
Life satisfaction		
Mean	5.4	5.1
Standard Deviation	3.8	3.8
Min, Max	0.0, 10.0	0.0, 10.0
(Missing)	9 (0.1%)	1 (<0.1%)
Current life evaluation		

Table S28b. Weighted summary statistics for outcome variables in Tanzania

Outcome	Wave 1<br>N = 9,075	Wave 2<br>N = 5,579
Mean	4.4	4.9
Standard Deviation	3.3	3.3
Min, Max	0.0, 10.0	0.0, 10.0
(Missing)	18 (0.2%)	0 (<0.1%)
Future life evaluation		
Mean	6.7	6.8
Standard Deviation	3.0	3.1
Min, Max	0.0, 10.0	0.0, 10.0
(Missing)	598 (6.6%)	85 (1.5%)
Optimism		
Mean	8.7	8.4
Standard Deviation	2.4	2.8
Min, Max	0.0, 10.0	0.0, 10.0
(Missing)	14 (0.2%)	7 (0.1%)
Freedom to pursue what's important		
Mean	8.4	8.2
Standard Deviation	2.6	2.9
Min, Max	0.0, 10.0	0.0, 10.0
(Missing)	10 (0.1%)	2 (<0.1%)
Inner peace, n (%)		
Always	2,866 (31.6%)	1,468 (26.3%)
Often	2,703 (29.8%)	1,537 (27.6%)
Rarely	3,292 (36.3%)	2,402 (43.0%)
Never	205 (2.3%)	168 (3.0%)
(Missing)	9 (0.1%)	4 (0.1%)
Life balance, n (%)		
Always	1,771 (19.5%)	832 (14.9%)
Often	2,223 (24.5%)	1,182 (21.2%)
Rarely	4,655 (51.3%)	3,272 (58.7%)
Never	395 (4.3%)	287 (5.1%)
(Missing)	31 (0.3%)	6 (0.1%)
Sense of mastery, n (%)		
Always	3,285 (36.2%)	1,795 (32.2%)
Often	2,678 (29.5%)	1,464 (26.3%)
Rarely	2,922 (32.2%)	2,147 (38.5%)
Never	170 (1.9%)	171 (3.1%)
(Missing)	21 (0.2%)	1 (0.0%)
Meaningful activities		
Mean	7.3	7.0
Standard Deviation	3.2	3.3
Min, Max	0.0, 10.0	0.0, 10.0
(Missing)	25 (0.3%)	3 (<0.1%)
Understanding purpose		
Mean	8.0	7.7
Standard Deviation	3.0	3.2
Min, Max	0.0, 10.0	0.0, 10.0
(Missing)	93 (1.0%)	7 (0.1%)
Self-rated mental health		
Mean	9.0	8.7
Standard Deviation	2.1	2.4
Min, Max	0.0, 10.0	0.0, 10.0

Table S28b. Weighted summary statistics for outcome variables in Tanzania

Outcome	Wave 1<br>N = 9,075	Wave 2<br>N = 5,579
(Missing)	29 (0.3%)	9 (0.2%)
Traumatic distress, n (%)		
A lot	932 (10.3%)	651 (11.7%)
Some	2,283 (25.2%)	1,942 (34.8%)
Not very much	1,248 (13.7%)	664 (11.9%)
Not at all	4,604 (50.7%)	2,317 (41.5%)
(Missing)	8 (0.1%)	4 (0.1%)
Depression symptoms composite, n (%)	4,083 (45.2%)	2,588 (46.5%)
(Missing)	46 (0.5%)	20 (0.4%)
Depression – feel hopeless, n (%)		
Nearly every day	1,367 (15.1%)	836 (15.0%)
More than half the days	2,336 (25.7%)	1,609 (28.8%)
Several days	1,523 (16.8%)	947 (17.0%)
Not at all	3,833 (42.2%)	2,174 (39.0%)
(Missing)	16 (0.2%)	12 (0.2%)
Depression – loss of interest, n (%)		
Nearly every day	1,559 (17.2%)	1,020 (18.3%)
More than half the days	2,295 (25.3%)	1,269 (22.8%)
Several days	1,373 (15.1%)	906 (16.2%)
Not at all	3,814 (42.0%)	2,371 (42.5%)
(Missing)	34 (0.4%)	12 (0.2%)
Anxiety symptoms composite, n (%)	2,877 (31.8%)	1,777 (32.0%)
(Missing)	40 (0.4%)	25 (0.4%)
Anxiety – feel on edge, n (%)		
Nearly every day	905 (10.0%)	536 (9.6%)
More than half the days	1,913 (21.1%)	1,264 (22.7%)
Several days	1,216 (13.4%)	736 (13.2%)
Not at all	5,028 (55.4%)	3,031 (54.3%)
(Missing)	14 (0.1%)	12 (0.2%)
Anxiety – cannot stop worrying, n (%)		
Nearly every day	1,062 (11.7%)	530 (9.5%)
More than half the days	1,788 (19.7%)	1,176 (21.1%)
Several days	1,207 (13.3%)	744 (13.3%)
Not at all	4,988 (55.0%)	3,115 (55.8%)
(Missing)	30 (0.3%)	14 (0.3%)
Suffering, n (%)		
A lot	1,091 (12.0%)	707 (12.7%)
Some	3,812 (42.0%)	2,862 (51.3%)
Not very much	1,605 (17.7%)	685 (12.3%)
Not at all	2,552 (28.1%)	1,317 (23.6%)
(Missing)	14 (0.2%)	8 (0.1%)
Relationship contentment		
Mean	7.8	7.7
Standard Deviation	3.0	3.2
Min, Max	0.0, 10.0	0.0, 10.0
(Missing)	17 (0.2%)	2 (<0.1%)
Relationship satisfaction		
Mean	7.4	7.4
Standard Deviation	3.3	3.3
Min, Max	0.0, 10.0	0.0, 10.0
(Missing)	22 (0.2%)	4 (<0.1%)

Table S28b. Weighted summary statistics for outcome variables in Tanzania

Outcome	Wave 1<br>N = 9,075	Wave 2<br>N = 5,579
Social support		
Mean	7.4	7.1
Standard Deviation	3.3	3.5
Min, Max	0.0, 10.0	0.0, 10.0
(Missing)	6 (<0.1%)	3 (<0.1%)
Intimate/close friend, n (%)		
Yes	6,776 (74.7%)	4,410 (79.0%)
No	2,290 (25.2%)	1,163 (20.8%)
(Missing)	9 (0.1%)	6 (0.1%)
Government approval, n (%)		
Strongly approve	4,578 (50.4%)	3,075 (55.1%)
Somewhat approve	3,420 (37.7%)	2,026 (36.3%)
Neither approve nor disapprove	395 (4.4%)	74 (1.3%)
Somewhat disapprove	269 (3.0%)	161 (2.9%)
Strongly disapprove	365 (4.0%)	228 (4.1%)
(Missing)	48 (0.5%)	15 (0.3%)
Say in government, n (%)		
Agree	3,806 (41.9%)	2,413 (43.2%)
Disagree	1,674 (18.4%)	777 (13.9%)
Unsure	3,572 (39.4%)	2,379 (42.6%)
(Missing)	23 (0.2%)	11 (0.2%)
Belonging in country		
Mean	8.2	8.3
Standard Deviation	2.8	2.9
Min, Max	0.0, 10.0	0.0, 10.0
(Missing)	38 (0.4%)	6 (0.1%)
City/place satisfaction, n (%)		
Satisfied	7,397 (81.5%)	4,167 (74.7%)
Dissatisfied	1,200 (13.2%)	979 (17.6%)
Unsure	477 (5.3%)	427 (7.7%)
(Missing)	2 (0.0%)	6 (0.1%)
Trust within country, n (%)		
All people	595 (6.6%)	316 (5.7%)
Most people	643 (7.1%)	458 (8.2%)
Some people	5,402 (59.5%)	3,758 (67.4%)
Not very many people	1,934 (21.3%)	787 (14.1%)
None	384 (4.2%)	225 (4.0%)
(Missing)	119 (1.3%)	35 (0.6%)
Number of children		
Mean	2.6	2.8
Standard Deviation	2.0	2.1
Min, Max	0.0, 19.0	0.0, 25.0
(Missing)	1 (<0.1%)	21 (0.4%)
Community participation, n (%)		
More than once a week	484 (5.3%)	358 (6.4%)
Once a week	1,375 (15.2%)	743 (13.3%)
One to three times a month	1,292 (14.2%)	851 (15.2%)
A few times a year	2,253 (24.8%)	1,354 (24.3%)
Never	3,647 (40.2%)	2,259 (40.5%)
(Missing)	24 (0.3%)	16 (0.3%)
Religious attendance, n (%)		

Table S28b. Weighted summary statistics for outcome variables in Tanzania

Outcome	Wave 1<br>N = 9,075	Wave 2<br>N = 5,579
More than once a week	2,566 (28.3%)	1,905 (34.2%)
Once a week	4,286 (47.2%)	2,277 (40.8%)
One to three times a month	1,101 (12.1%)	539 (9.7%)
A few times a year	831 (9.2%)	637 (11.4%)
Never	289 (3.2%)	216 (3.9%)
(Missing)	1 (0.0%)	4 (0.1%)
Loneliness		
Mean	3.4	3.5
Standard Deviation	3.5	3.6
Min, Max	0.0, 10.0	0.0, 10.0
(Missing)	17 (0.2%)	1 (<0.1%)
Perceived discrimination, n (%)		
Always	442 (4.9%)	240 (4.3%)
Often	791 (8.7%)	513 (9.2%)
Rarely	3,235 (35.6%)	2,383 (42.7%)
Never	4,584 (50.5%)	2,435 (43.6%)
(Missing)	23 (0.3%)	8 (0.1%)
Orientation to promote good		
Mean	8.2	8.0
Standard Deviation	2.7	2.9
Min, Max	0.0, 10.0	0.0, 10.0
(Missing)	27 (0.3%)	6 (0.1%)
Delayed gratification		
Mean	7.5	7.4
Standard Deviation	3.2	3.3
Min, Max	0.0, 10.0	0.0, 10.0
(Missing)	28 (0.3%)	10 (0.2%)
Hope		
Mean	8.3	8.1
Standard Deviation	2.7	2.9
Min, Max	0.0, 10.0	0.0, 10.0
(Missing)	31 (0.3%)	2 (<0.1%)
Gratitude		
Mean	7.8	7.9
Standard Deviation	2.9	3.0
Min, Max	0.0, 10.0	0.0, 10.0
(Missing)	52 (0.6%)	6 (<0.1%)
Showing love/care		
Mean	8.7	8.7
Standard Deviation	2.3	2.5
Min, Max	0.0, 10.0	0.0, 10.0
(Missing)	20 (0.2%)	2 (<0.1%)
Forgivingness, n (%)		
Always	4,626 (51.0%)	2,381 (42.7%)
Often	2,961 (32.6%)	2,168 (38.9%)
Rarely	1,304 (14.4%)	916 (16.4%)
Never	169 (1.9%)	114 (2.0%)
(Missing)	16 (0.2%)	0 (<0.0%)
Charitable giving, n (%)		
Yes	2,443 (26.9%)	2,707 (48.5%)
No	6,625 (73.0%)	2,861 (51.3%)

Table S28b. Weighted summary statistics for outcome variables in Tanzania

Outcome	Wave 1<br>N = 9,075	Wave 2<br>N = 5,579
(Missing)	8 (0.1%)	11 (0.2%)
Helping strangers, n (%)		
Yes	3,021 (33.3%)	3,100 (55.6%)
No	6,021 (66.3%)	2,458 (44.1%)
(Missing)	33 (0.4%)	21 (0.4%)
Volunteering, n (%)		
Yes	989 (10.9%)	1,247 (22.3%)
No	8,082 (89.1%)	4,321 (77.5%)
(Missing)	4 (0.0%)	11 (0.2%)
Self-rated physical health		
Mean	7.8	7.4
Standard Deviation	3.0	3.2
Min, Max	0.0, 10.0	0.0, 10.0
(Missing)	14 (0.2%)	4 (<0.1%)
Health problems, n (%)		
Yes	1,380 (15.2%)	1,137 (20.4%)
No	7,685 (84.7%)	4,430 (79.4%)
(Missing)	10 (0.1%)	12 (0.2%)
Pain in past 4 weeks, n (%)		
A lot	1,117 (12.3%)	926 (16.6%)
Some	3,570 (39.3%)	2,557 (45.8%)
Not very much	1,731 (19.1%)	770 (13.8%)
None at all	2,652 (29.2%)	1,325 (23.8%)
(Missing)	5 (0.1%)	1 (0.0%)
Number of cigarettes per day		
Mean	0.2	0.3
Standard Deviation	1.9	2.0
Min, Max	0.0, 97.0	0.0, 97.0
(Missing)	192 (2.1%)	29 (0.5%)
Number of drinks per week		
Mean	0.5	0.4
Standard Deviation	4.1	2.9
Min, Max	0.0, 97.0	0.0, 97.0
(Missing)	196 (2.2%)	32 (0.6%)
Days exercise per week		
Mean	3.2	3.3
Standard Deviation	2.7	2.8
Min, Max	0.0, 7.0	0.0, 7.0
(Missing)	99 (1.1%)	77 (1.4%)
Financial security		
Mean	5.7	5.8
Standard Deviation	3.7	3.7
Min, Max	0.0, 10.0	0.0, 10.0
(Missing)	16 (0.2%)	2 (<0.1%)
Material security		
Mean	5.9	6.2
Standard Deviation	3.7	3.8
Min, Max	0.0, 10.0	0.0, 10.0
(Missing)	11 (0.1%)	4 (<0.1%)
Educational attainment (16+ years), n (%)		
Up to 8	6,848 (75.5%)	4,176 (74.9%)

Table S28b. Weighted summary statistics for outcome variables in Tanzania

**Outcome**	**Wave 1**<br>N = 9,075	**Wave 2**<br>N = 5,579
9-15	2,123 (23.4%)	1,317 (23.6%)
16+	103 (1.1%)	86 (1.5%)
(Missing)	2 (0.0%)	0 (0%)
Currently employed, n (%)		
Employed for an employer	493 (5.4%)	417 (7.5%)
Self-employed	4,553 (50.2%)	3,098 (55.5%)
Retired	117 (1.3%)	69 (1.2%)
Student	365 (4.0%)	146 (2.6%)
Homemaker	1,793 (19.8%)	1,106 (19.8%)
Unemployed and looking for a job	1,557 (17.2%)	660 (11.8%)
None of these/Other	190 (2.1%)	72 (1.3%)
(Missing)	6 (0.1%)	11 (0.2%)
Financially comfortable/getting by, n (%)		
Living comfortably on present income	622 (6.9%)	663 (11.9%)
Getting by on present income	3,878 (42.7%)	2,584 (46.3%)
Finding it difficult on present income	2,241 (24.7%)	1,313 (23.5%)
Finding it very difficult on present income	2,260 (24.9%)	998 (17.9%)
(Missing)	74 (0.8%)	21 (0.4%)
Own home, n (%)		
Someone in this household owns this home	6,416 (70.7%)	3,534 (63.3%)
Someone in this household rents this home	2,171 (23.9%)	1,627 (29.2%)
Both	68 (0.8%)	50 (0.9%)
Neither	412 (4.5%)	356 (6.4%)
Rent	0 (0%)	0 (0%)
Own	0 (0%)	0 (0%)
Something else	0 (0%)	0 (0%)
(Missing)	7 (0.1%)	12 (0.2%)
Religious/spiritual connection, n (%)		
Always	4,794 (52.8%)	2,831 (50.7%)
Often	2,558 (28.2%)	1,627 (29.2%)
Rarely	1,555 (17.1%)	960 (17.2%)
Never	161 (1.8%)	160 (2.9%)
(Missing)	7 (0.1%)	2 (0.0%)
Belief in life after death, n (%)		
Yes	5,474 (60.3%)	3,433 (61.5%)
No	1,986 (21.9%)	1,132 (20.3%)
Unsure	1,600 (17.6%)	1,002 (18.0%)
(Missing)	15 (0.2%)	12 (0.2%)
Transformative religious experience, n (%)		
Yes	6,231 (68.7%)	3,870 (69.4%)
No	2,813 (31.0%)	1,701 (30.5%)
(Missing)	31 (0.3%)	8 (0.1%)
Religious reading or listening, n (%)		
More than once a day	2,278 (25.1%)	1,693 (30.3%)
About once a day	4,018 (44.3%)	2,185 (39.2%)
Sometimes	2,282 (25.1%)	1,303 (23.4%)
Never	482 (5.3%)	392 (7.0%)
(Missing)	15 (0.2%)	5 (0.1%)
Prayer or meditation, n (%)		
More than once a day	3,061 (33.7%)	2,141 (38.4%)
About once a day	3,785 (41.7%)	2,158 (38.7%)

Table S28b. Weighted summary statistics for outcome variables in Tanzania

Outcome	**Wave 1**<br>N = 9,075	**Wave 2**<br>N = 5,579
Sometimes	1,883 (20.7%)	981 (17.6%)
Never	338 (3.7%)	288 (5.2%)
(Missing)	8 (0.1%)	10 (0.2%)
Belief in God/gods/spiritual forces, n (%)		
One God	8,621 (95.0%)	5,217 (93.5%)
More than one god	180 (2.0%)	75 (1.4%)
An impersonal spiritual force	101 (1.1%)	74 (1.3%)
None of these	33 (0.4%)	38 (0.7%)
Unsure	135 (1.5%)	168 (3.0%)
(Missing)	4 (0.0%)	6 (0.1%)
Religious centrality, n (%)		
Agree	8,221 (90.6%)	5,043 (90.4%)
Disagree	416 (4.6%)	211 (3.8%)
Not relevant	59 (0.6%)	21 (0.4%)
Unsure	377 (4.2%)	302 (5.4%)
(Missing)	2 (0.0%)	1 (0.0%)
Religious/spiritual comfort, n (%)		
Agree	8,624 (95.0%)	5,270 (94.5%)
Disagree	202 (2.2%)	107 (1.9%)
Not relevant	52 (0.6%)	20 (0.4%)
Unsure	197 (2.2%)	181 (3.2%)
(Missing)	0 (<0.0%)	1 (0.0%)
Feel loved by God, n (%)		
Agree	8,820 (97.2%)	5,466 (98.0%)
Disagree	121 (1.3%)	35 (0.6%)
Not relevant	12 (0.1%)	7 (0.1%)
Unsure	117 (1.3%)	70 (1.3%)
(Missing)	5 (0.1%)	1 (0.0%)
Feel punished by God, n (%)		
Agree	3,151 (34.7%)	2,281 (40.9%)
Disagree	4,183 (46.1%)	2,285 (41.0%)
Not relevant	265 (2.9%)	40 (0.7%)
Unsure	1,444 (15.9%)	965 (17.3%)
(Missing)	31 (0.3%)	8 (0.1%)
Experienced religious criticism, n (%)		
Agree	4,184 (46.1%)	2,843 (51.0%)
Disagree	3,428 (37.8%)	1,634 (29.3%)
Not relevant	238 (2.6%)	74 (1.3%)
Unsure	1,213 (13.4%)	1,023 (18.3%)
(Missing)	11 (0.1%)	4 (0.1%)
Faith-sharing, n (%)		
Agree	7,549 (83.2%)	4,775 (85.6%)
Disagree	962 (10.6%)	392 (7.0%)
Not relevant	220 (2.4%)	51 (0.9%)
Unsure	341 (3.8%)	357 (6.4%)
(Missing)	4 (0.0%)	4 (0.1%)

Note. N (%); this table is based on non-imputed data. Cumulative percentages for variables may not add up to 100% due to rounding. Wave 1 characteristics weighted using the Gallup provided sampling weight, ANNUAL_WEIGHT_R2; Wave 2 characteristics weighted accounting for attrition by using the adjusted Wave 1 weight, ANNUAL_WEIGHT_R2, multiplied by the created attrition weight to account for dropout, to maintain nationally representative estimates for Wave 2 characteristics.

Table S28c. Unweighted summary statistics for demographic and childhood variables in Tanzania by retention status

Characteristic	Attritors–Not Observed in Wave 2<br>N = 3,242	Retained–Observed in Wave 2<br>N = 5,567
Forgivingness, n (%)		
Always	1,666 (51.4%)	2,822 (50.7%)
Often	1,049 (32.4%)	1,827 (32.8%)
Rarely	465 (14.4%)	801 (14.4%)
Never	58 (1.8%)	107 (1.9%)
(Missing)	4 (0.1%)	11 (0.2%)
Year of birth, n (%)		
1943 or earlier (current age: 80+ years)	46 (1.4%)	33 (0.6%)
1943-1953 (current age: 70-79 years)	127 (3.9%)	133 (2.4%)
1953-1963 (current age: 60-69 years)	177 (5.4%)	337 (6.1%)
1963-1973 (current age: 50-59 years)	252 (7.8%)	574 (10.3%)
1973-1983 (current age: 40-49 years)	428 (13.2%)	973 (17.5%)
1983-1993 (current age: 30-39 years)	684 (21.1%)	1,248 (22.4%)
1993-1998 (current age: 25-29 years)	460 (14.2%)	760 (13.6%)
1998-2005 (current age: 18-24 years)	1,069 (33.0%)	1,507 (27.1%)
(Missing)	0 (0%)	2 (0.0%)
Age of participant		
Mean	35.4	36.5
Standard Deviation	16.2	14.8
Min, Max	18.0, 99.0	18.0, 99.0
(Missing)	0 (0%)	2 (<0.1%)
Gender, n (%)		
Male	1,353 (41.7%)	2,824 (50.7%)
Female	1,890 (58.3%)	2,743 (49.3%)
Other	0 (0%)	0 (0%)
(Missing)	0 (0%)	0 (0%)
Respondent marital status, n (%)		
Single/Never been married	904 (27.9%)	1,441 (25.9%)
Married	1,881 (58.0%)	3,436 (61.7%)
Separated	144 (4.4%)	232 (4.2%)
Divorced	27 (0.8%)	70 (1.3%)
Widowed	186 (5.7%)	215 (3.9%)
Domestic partner	99 (3.0%)	167 (3.0%)
(Missing)	1 (0.0%)	5 (0.1%)
Education (years), n (%)		
Up to 8	2,523 (77.8%)	4,108 (73.8%)
9-15	691 (21.3%)	1,385 (24.9%)
16+	28 (0.9%)	74 (1.3%)
(Missing)	1 (0.0%)	1 (<0.0%)
Employment status, n (%)		
Employed for an employer	165 (5.1%)	316 (5.7%)
Self-employed	1,557 (48.0%)	2,879 (51.7%)
Retired	42 (1.3%)	72 (1.3%)
Student	119 (3.7%)	239 (4.3%)
Homemaker	714 (22.0%)	1,009 (18.1%)
Unemployed and looking for a job	573 (17.7%)	935 (16.8%)
None of these/Other	73 (2.2%)	111 (2.0%)
(Missing)	0 (0%)	7 (0.1%)
Current religious service attendance, n (%)		

Table S28c. Unweighted summary statistics for demographic and childhood variables in Tanzania by retention status

Characteristic	Attritors–Not Observed in Wave 2<br>N = 3,242	Retained–Observed in Wave 2<br>N = 5,567
More than once a week	898 (27.7%)	1,598 (28.7%)
Once a week	1,569 (48.4%)	2,584 (46.4%)
One to three times a month	410 (12.6%)	655 (11.8%)
A few times a year	274 (8.4%)	538 (9.7%)
Never	92 (2.8%)	191 (3.4%)
(Missing)	0 (0%)	1 (0.0%)
Immigration status, n (%)		
Born in this country	3,226 (99.5%)	5,557 (99.8%)
Born in another country	16 (0.5%)	9 (0.2%)
(Missing)	0 (0%)	2 (0.0%)
Parental marital status around age 12, n (%)		
Parents were married	2,440 (75.3%)	4,265 (76.6%)
Parents were divorced	236 (7.3%)	441 (7.9%)
Parents were never married	291 (9.0%)	456 (8.2%)
One or both of them had died	108 (3.3%)	195 (3.5%)
Unsure	18 (0.6%)	27 (0.5%)
(Missing)	150 (4.6%)	184 (3.3%)
Religious service attendance around age 12, n (%)		
At least once a week	1,923 (59.3%)	3,488 (62.7%)
One to three times a month	888 (27.4%)	1,431 (25.7%)
Less than once a month	120 (3.7%)	206 (3.7%)
Never	241 (7.4%)	334 (6.0%)
(Missing)	71 (2.2%)	108 (1.9%)
Relationship with mother when growing up, n (%)		
Very good	2,776 (85.6%)	4,720 (84.8%)
Somewhat good	264 (8.2%)	527 (9.5%)
Somewhat bad	31 (0.9%)	52 (0.9%)
Very bad	39 (1.2%)	45 (0.8%)
(Does not apply)	114 (3.5%)	173 (3.1%)
(Missing)	18 (0.6%)	50 (0.9%)
Relationship with father when growing up, n (%)		
Very good	2,434 (75.1%)	4,168 (74.9%)
Somewhat good	376 (11.6%)	706 (12.7%)
Somewhat bad	82 (2.5%)	122 (2.2%)
Very bad	79 (2.4%)	173 (3.1%)
(Does not apply)	217 (6.7%)	314 (5.6%)
(Missing)	55 (1.7%)	85 (1.5%)
Felt like an outsider in family when growing up, n (%)		
Yes	277 (8.5%)	437 (7.8%)
No	2,957 (91.2%)	5,120 (92.0%)
(Missing)	9 (0.3%)	11 (0.2%)
Experienced abuse when growing up, n (%)		
Yes	222 (6.8%)	488 (8.8%)
No	3,010 (92.8%)	5,061 (90.9%)
(Missing)	11 (0.3%)	18 (0.3%)
Self-rated health when growing up, n (%)		
Excellent	828 (25.5%)	1,519 (27.3%)

Table S28c. Unweighted summary statistics for demographic and childhood variables in Tanzania by retention status

**Characteristic**	**Attritors–Not Observed in Wave 2** N = 3,242	**Retained–Observed in Wave 2** N = 5,567
Very good	702 (21.7%)	1,261 (22.7%)
Good	1,073 (33.1%)	1,779 (32.0%)
Fair	419 (12.9%)	730 (13.1%)
Poor	194 (6.0%)	252 (4.5%)
(Missing)	26 (0.8%)	26 (0.5%)
Subjective financial status of family growing up, n (%)		
Lived comfortably	940 (29.0%)	1,605 (28.8%)
Got by	1,031 (31.8%)	1,778 (31.9%)
Found it difficult	957 (29.5%)	1,652 (29.7%)
Found it very difficult	284 (8.7%)	505 (9.1%)
(Missing)	30 (0.9%)	27 (0.5%)
Religious affiliation growing up, n (%)		
Christianity	2,067 (63.8%)	3,407 (61.2%)
Taoism	0 (0%)	0 (0%)
Confucianism	0 (0%)	0 (0%)
Primal, Animist, or Folk religion	1 (0.0%)	11 (0.2%)
Spiritism	0 (0%)	0 (0%)
Umbanda, Candomblé, and other African-derived religions	0 (0%)	0 (0%)
Chinese folk/traditional religion	0 (0%)	0 (0%)
Islam	1,015 (31.3%)	1,975 (35.5%)
Hinduism	0 (0%)	0 (0%)
Buddhism	0 (0%)	0 (0%)
Judaism	0 (0%)	0 (0%)
Sikhism	0 (0%)	0 (0%)
Baha'i	1 (0.0%)	0 (0%)
Jainism	0 (0%)	0 (0%)
Shinto	0 (0%)	0 (0%)
Some other religion	0 (0%)	0 (0%)
No religion/Atheist/Agnostic	156 (4.8%)	170 (3.1%)
(Missing)	3 (0.1%)	4 (0.1%)

Note. N (%); this table is based on non-imputed data. Cumulative percentages for variables may not add up to 100% due to rounding.

Table S28d. Unweighted summary statistics for Wave 1 outcome variables in Tanzania by retention status.

**Outcome**	**Attritors–Not Observed in Wave 2** N = 3,242	**Retained–Observed in Wave 2** N = 5,567
Secure flourishing index		
Mean	7.2	7.3
Standard Deviation	1.8	1.8
Min, Max	0.0, 10.0	0.2, 10.0
(Missing)	78 (2.4%)	154 (2.8%)
Flourishing index		
Mean	7.5	7.5
Standard Deviation	1.8	1.8
Min, Max	0.0, 10.0	0.2, 10.0
(Missing)	72 (2.2%)	146 (2.6%)
Happiness & life satisfaction		
Mean	6.1	5.9
Standard Deviation	3.0	3.0
Min, Max	0.0, 10.0	0.0, 10.0
(Missing)	9 (0.3%)	12 (0.2%)
Physical & mental health		
Mean	8.3	8.5
Standard Deviation	2.2	2.1
Min, Max	0.0, 10.0	0.0, 10.0
(Missing)	14 (0.4%)	26 (0.5%)
Meaning & purpose		
Mean	7.6	7.7
Standard Deviation	2.5	2.5
Min, Max	0.0, 10.0	0.0, 10.0
(Missing)	37 (1.1%)	74 (1.3%)
Character & virtue		
Mean	7.7	7.9
Standard Deviation	2.5	2.4
Min, Max	0.0, 10.0	0.0, 10.0
(Missing)	12 (0.4%)	32 (0.6%)
Close social relationships		
Mean	7.5	7.6
Standard Deviation	2.7	2.8
Min, Max	0.0, 10.0	0.0, 10.0
(Missing)	13 (0.4%)	21 (0.4%)
Financial & material security		
Mean	5.7	5.9
Standard Deviation	3.3	3.3
Min, Max	0.0, 10.0	0.0, 10.0
(Missing)	8 (0.2%)	13 (0.2%)
Happiness		
Mean	6.6	6.6
Standard Deviation	3.3	3.3
Min, Max	0.0, 10.0	0.0, 10.0
(Missing)	5 (0.2%)	9 (0.2%)
Life satisfaction		
Mean	5.6	5.2
Standard Deviation	3.7	3.8
Min, Max	0.0, 10.0	0.0, 10.0

Table S28d. Unweighted summary statistics for Wave 1 outcome variables in Tanzania by retention status.

**Outcome**	**Attritors–Not Observed in Wave 2** N = 3,242	**Retained–Observed in Wave 2** N = 5,567
(Missing)	5 (0.2%)	4 (<0.1%)
Current life evaluation		
Mean	4.4	4.4
Standard Deviation	3.3	3.3
Min, Max	0.0, 10.0	0.0, 10.0
(Missing)	8 (0.3%)	9 (0.2%)
Future life evaluation		
Mean	6.7	6.7
Standard Deviation	3.0	3.1
Min, Max	0.0, 10.0	0.0, 10.0
(Missing)	265 (8.2%)	304 (5.5%)
Optimism		
Mean	8.6	8.8
Standard Deviation	2.4	2.3
Min, Max	0.0, 10.0	0.0, 10.0
(Missing)	4 (0.1%)	10 (0.2%)
Freedom to pursue what's important		
Mean	8.3	8.5
Standard Deviation	2.6	2.6
Min, Max	0.0, 10.0	0.0, 10.0
(Missing)	6 (0.2%)	4 (<0.1%)
Inner peace, n (%)		
Always	1,038 (32.0%)	1,741 (31.3%)
Often	1,016 (31.3%)	1,596 (28.7%)
Rarely	1,102 (34.0%)	2,110 (37.9%)
Never	84 (2.6%)	114 (2.0%)
(Missing)	3 (0.1%)	7 (0.1%)
Life balance, n (%)		
Always	668 (20.6%)	1,043 (18.7%)
Often	837 (25.8%)	1,312 (23.6%)
Rarely	1,573 (48.5%)	2,967 (53.3%)
Never	152 (4.7%)	229 (4.1%)
(Missing)	13 (0.4%)	17 (0.3%)
Sense of mastery, n (%)		
Always	1,156 (35.7%)	2,036 (36.6%)
Often	986 (30.4%)	1,608 (28.9%)
Rarely	1,029 (31.7%)	1,811 (32.5%)
Never	65 (2.0%)	98 (1.8%)
(Missing)	6 (0.2%)	14 (0.3%)
Meaningful activities		
Mean	7.3	7.4
Standard Deviation	3.2	3.2
Min, Max	0.0, 10.0	0.0, 10.0
(Missing)	8 (0.3%)	16 (0.3%)
Understanding purpose		
Mean	8.0	8.1
Standard Deviation	2.9	3.0
Min, Max	0.0, 10.0	0.0, 10.0
(Missing)	31 (1.0%)	60 (1.1%)

Table S28d. Unweighted summary statistics for Wave 1 outcome variables in Tanzania by retention status.

**Outcome**	**Attritors–Not Observed in Wave 2** N = 3,242	**Retained–Observed in Wave 2** N = 5,567
Self-rated mental health		
Mean	8.9	9.0
Standard Deviation	2.1	2.1
Min, Max	0.0, 10.0	0.0, 10.0
(Missing)	10 (0.3%)	19 (0.3%)
Traumatic distress, n (%)		
A lot	319 (9.8%)	589 (10.6%)
Some	802 (24.7%)	1,418 (25.5%)
Not very much	483 (14.9%)	720 (12.9%)
Not at all	1,636 (50.5%)	2,836 (50.9%)
(Missing)	3 (0.1%)	5 (0.1%)
Depression symptoms composite, n (%)	1,485 (46.0%)	2,473 (44.7%)
(Missing)	12 (0.4%)	34 (0.6%)
Depression – feel hopeless, n (%)		
Nearly every day	510 (15.7%)	812 (14.6%)
More than half the days	803 (24.8%)	1,472 (26.4%)
Several days	565 (17.4%)	908 (16.3%)
Not at all	1,361 (42.0%)	2,362 (42.4%)
(Missing)	3 (0.1%)	14 (0.2%)
Depression – loss of interest, n (%)		
Nearly every day	561 (17.3%)	952 (17.1%)
More than half the days	831 (25.6%)	1,394 (25.0%)
Several days	478 (14.8%)	857 (15.4%)
Not at all	1,363 (42.0%)	2,340 (42.0%)
(Missing)	10 (0.3%)	24 (0.4%)
Anxiety symptoms composite, n (%)	1,055 (32.6%)	1,733 (31.3%)
(Missing)	11 (0.3%)	29 (0.5%)
Anxiety – feel on edge, n (%)		
Nearly every day	352 (10.8%)	521 (9.4%)
More than half the days	681 (21.0%)	1,177 (21.1%)
Several days	449 (13.8%)	728 (13.1%)
Not at all	1,757 (54.2%)	3,133 (56.3%)
(Missing)	4 (0.1%)	9 (0.2%)
Anxiety – cannot stop worrying, n (%)		
Nearly every day	403 (12.4%)	623 (11.2%)
More than half the days	615 (19.0%)	1,126 (20.2%)
Several days	425 (13.1%)	748 (13.4%)
Not at all	1,792 (55.3%)	3,049 (54.8%)
(Missing)	8 (0.3%)	22 (0.4%)
Suffering, n (%)		
A lot	420 (13.0%)	633 (11.4%)
Some	1,272 (39.2%)	2,449 (44.0%)
Not very much	614 (18.9%)	935 (16.8%)
Not at all	933 (28.8%)	1,541 (27.7%)
(Missing)	4 (0.1%)	10 (0.2%)
Relationship contentment		
Mean	7.7	7.8
Standard Deviation	3.0	3.1
Min, Max	0.0, 10.0	0.0, 10.0

Table S28d. Unweighted summary statistics for Wave 1 outcome variables in Tanzania by retention status.

**Outcome**	**Attritors–Not Observed in Wave 2** N = 3,242	**Retained–Observed in Wave 2** N = 5,567
(Missing)	6 (0.2%)	10 (0.2%)
Relationship satisfaction		
Mean	7.3	7.4
Standard Deviation	3.3	3.3
Min, Max	0.0, 10.0	0.0, 10.0
(Missing)	8 (0.2%)	14 (0.2%)
Social support		
Mean	7.5	7.3
Standard Deviation	3.1	3.4
Min, Max	0.0, 10.0	0.0, 10.0
(Missing)	1 (<0.1%)	5 (<0.1%)
Intimate/close friend, n (%)		
Yes	2,284 (70.4%)	4,324 (77.7%)
No	952 (29.4%)	1,241 (22.3%)
(Missing)	6 (0.2%)	2 (0.0%)
Government approval, n (%)		
Strongly approve	1,602 (49.4%)	2,850 (51.2%)
Somewhat approve	1,218 (37.6%)	2,103 (37.8%)
Neither approve nor disapprove	162 (5.0%)	217 (3.9%)
Somewhat disapprove	108 (3.3%)	150 (2.7%)
Strongly disapprove	127 (3.9%)	228 (4.1%)
(Missing)	25 (0.8%)	20 (0.4%)
Say in government, n (%)		
Agree	1,348 (41.6%)	2,350 (42.2%)
Disagree	649 (20.0%)	964 (17.3%)
Unsure	1,237 (38.1%)	2,240 (40.2%)
(Missing)	9 (0.3%)	13 (0.2%)
Belonging in country		
Mean	8.2	8.3
Standard Deviation	2.7	2.8
Min, Max	0.0, 10.0	0.0, 10.0
(Missing)	14 (0.4%)	23 (0.4%)
City/place satisfaction, n (%)		
Satisfied	2,625 (80.9%)	4,560 (81.9%)
Dissatisfied	452 (14.0%)	707 (12.7%)
Unsure	164 (5.1%)	301 (5.4%)
(Missing)	1 (0.0%)	0 (0%)
Trust within country, n (%)		
All people	201 (6.2%)	379 (6.8%)
Most people	225 (6.9%)	400 (7.2%)
Some people	1,916 (59.1%)	3,331 (59.8%)
Not very many people	715 (22.0%)	1,157 (20.8%)
None	144 (4.4%)	227 (4.1%)
(Missing)	42 (1.3%)	74 (1.3%)
Number of children		
Mean	2.5	2.7
Standard Deviation	2.1	2.0
Min, Max	0.0, 19.0	0.0, 18.0
Community participation, n (%)		

Table S28d. Unweighted summary statistics for Wave 1 outcome variables in Tanzania by retention status.

**Outcome**	**Attritors–Not Observed in Wave 2** N = 3,242	**Retained–Observed in Wave 2** N = 5,567
More than once a week	154 (4.7%)	320 (5.8%)
Once a week	483 (14.9%)	854 (15.3%)
One to three times a month	440 (13.6%)	818 (14.7%)
A few times a year	816 (25.2%)	1,369 (24.6%)
Never	1,339 (41.3%)	2,193 (39.4%)
(Missing)	10 (0.3%)	13 (0.2%)
Religious attendance, n (%)		
More than once a week	898 (27.7%)	1,598 (28.7%)
Once a week	1,569 (48.4%)	2,584 (46.4%)
One to three times a month	410 (12.6%)	655 (11.8%)
A few times a year	274 (8.4%)	538 (9.7%)
Never	92 (2.8%)	191 (3.4%)
(Missing)	0 (0%)	1 (0.0%)
Loneliness		
Mean	3.4	3.3
Standard Deviation	3.5	3.6
Min, Max	0.0, 10.0	0.0, 10.0
(Missing)	6 (0.2%)	11 (0.2%)
Perceived discrimination, n (%)		
Always	161 (5.0%)	268 (4.8%)
Often	320 (9.9%)	439 (7.9%)
Rarely	1,101 (34.0%)	2,051 (36.8%)
Never	1,649 (50.9%)	2,798 (50.3%)
(Missing)	11 (0.3%)	11 (0.2%)
Orientation to promote good		
Mean	8.1	8.3
Standard Deviation	2.7	2.7
Min, Max	0.0, 10.0	0.0, 10.0
(Missing)	6 (0.2%)	20 (0.4%)
Delayed gratification		
Mean	7.4	7.6
Standard Deviation	3.2	3.2
Min, Max	0.0, 10.0	0.0, 10.0
(Missing)	8 (0.2%)	20 (0.4%)
Hope		
Mean	8.2	8.4
Standard Deviation	2.7	2.6
Min, Max	0.0, 10.0	0.0, 10.0
(Missing)	14 (0.4%)	15 (0.3%)
Gratitude		
Mean	7.7	7.9
Standard Deviation	2.9	2.9
Min, Max	0.0, 10.0	0.0, 10.0
(Missing)	16 (0.5%)	35 (0.6%)
Showing love/care		
Mean	8.6	8.8
Standard Deviation	2.3	2.3
Min, Max	0.0, 10.0	0.0, 10.0
(Missing)	7 (0.2%)	12 (0.2%)

Table S28d. Unweighted summary statistics for Wave 1 outcome variables in Tanzania by retention status.

**Outcome**	**Attritors–Not Observed in Wave 2**<br>N = 3,242	**Retained–Observed in Wave 2**<br>N = 5,567
Forgivingness, n (%)		
Always	1,666 (51.4%)	2,822 (50.7%)
Often	1,049 (32.4%)	1,827 (32.8%)
Rarely	465 (14.4%)	801 (14.4%)
Never	58 (1.8%)	107 (1.9%)
(Missing)	4 (0.1%)	11 (0.2%)
Charitable giving, n (%)		
Yes	804 (24.8%)	1,583 (28.4%)
No	2,437 (75.2%)	3,978 (71.5%)
(Missing)	1 (0.0%)	6 (0.1%)
Helping strangers, n (%)		
Yes	988 (30.5%)	1,965 (35.3%)
No	2,243 (69.2%)	3,582 (64.3%)
(Missing)	12 (0.4%)	21 (0.4%)
Volunteering, n (%)		
Yes	309 (9.5%)	662 (11.9%)
No	2,933 (90.5%)	4,903 (88.1%)
(Missing)	1 (0.0%)	3 (0.0%)
Self-rated physical health		
Mean	7.7	7.9
Standard Deviation	3.0	3.0
Min, Max	0.0, 10.0	0.0, 10.0
(Missing)	5 (0.2%)	8 (0.1%)
Health problems, n (%)		
Yes	508 (15.7%)	828 (14.9%)
No	2,730 (84.2%)	4,734 (85.0%)
(Missing)	4 (0.1%)	5 (0.1%)
Pain in past 4 weeks, n (%)		
A lot	420 (13.0%)	660 (11.8%)
Some	1,234 (38.1%)	2,241 (40.2%)
Not very much	661 (20.4%)	1,010 (18.1%)
None at all	926 (28.6%)	1,653 (29.7%)
(Missing)	0 (0.0%)	4 (0.1%)
Number of cigarettes per day		
Mean	0.2	0.2
Standard Deviation	1.3	2.2
Min, Max	0.0, 30.0	0.0, 97.0
(Missing)	82 (2.5%)	102 (1.8%)
Number of drinks per week		
Mean	0.5	0.5
Standard Deviation	4.4	3.9
Min, Max	0.0, 97.0	0.0, 97.0
(Missing)	86 (2.7%)	100 (1.8%)
Days exercise per week		
Mean	3.1	3.4
Standard Deviation	2.7	2.7
Min, Max	0.0, 7.0	0.0, 7.0
(Missing)	41 (1.3%)	55 (1.0%)
Financial security		

Table S28d. Unweighted summary statistics for Wave 1 outcome variables in Tanzania by retention status.

**Outcome**	**Attritors–Not Observed in Wave 2**<br>N = 3,242	**Retained–Observed in Wave 2**<br>N = 5,567
Mean	5.6	5.8
Standard Deviation	3.6	3.8
Min, Max	0.0, 10.0	0.0, 10.0
(Missing)	6 (0.2%)	9 (0.2%)
Material security		
Mean	5.8	5.9
Standard Deviation	3.7	3.8
Min, Max	0.0, 10.0	0.0, 10.0
(Missing)	3 (0.1%)	8 (0.1%)
Educational attainment (16+ years), n (%)		
Up to 8	2,523 (77.8%)	4,108 (73.8%)
9-15	691 (21.3%)	1,385 (24.9%)
16+	28 (0.9%)	74 (1.3%)
(Missing)	1 (0.0%)	1 (<0.0%)
Currently employed, n (%)		
Employed for an employer	165 (5.1%)	316 (5.7%)
Self-employed	1,557 (48.0%)	2,879 (51.7%)
Retired	42 (1.3%)	72 (1.3%)
Student	119 (3.7%)	239 (4.3%)
Homemaker	714 (22.0%)	1,009 (18.1%)
Unemployed and looking for a job	573 (17.7%)	935 (16.8%)
None of these/Other	73 (2.2%)	111 (2.0%)
(Missing)	0 (0%)	7 (0.1%)
Financially comfortable/getting by, n (%)		
Living comfortably on present income	219 (6.8%)	385 (6.9%)
Getting by on present income	1,332 (41.1%)	2,445 (43.9%)
Finding it difficult on present income	833 (25.7%)	1,335 (24.0%)
Finding it very difficult on present income	836 (25.8%)	1,352 (24.3%)
(Missing)	22 (0.7%)	51 (0.9%)
Own home, n (%)		
Someone in this household owns this home	2,184 (67.4%)	4,069 (73.1%)
Someone in this household rents this home	861 (26.6%)	1,227 (22.0%)
Both	31 (1.0%)	33 (0.6%)
Neither	164 (5.1%)	232 (4.2%)
Rent	0 (0%)	0 (0%)
Own	0 (0%)	0 (0%)
Something else	0 (0%)	0 (0%)
(Missing)	2 (0.1%)	6 (0.1%)
Religious/spiritual connection, n (%)		
Always	1,676 (51.7%)	2,986 (53.6%)
Often	973 (30.0%)	1,498 (26.9%)
Rarely	547 (16.9%)	964 (17.3%)
Never	43 (1.3%)	116 (2.1%)
(Missing)	3 (0.1%)	4 (0.1%)
Belief in life after death, n (%)		
Yes	1,913 (59.0%)	3,410 (61.3%)
No	710 (21.9%)	1,217 (21.9%)
Unsure	615 (19.0%)	928 (16.7%)
(Missing)	4 (0.1%)	11 (0.2%)

Table S28d. Unweighted summary statistics for Wave 1 outcome variables in Tanzania by retention status.

**Outcome**	**Attritors–Not Observed in Wave 2** N = 3,242	**Retained–Observed in Wave 2** N = 5,567
Transformative religious experience, n (%)		
Yes	2,216 (68.3%)	3,836 (68.9%)
No	1,017 (31.4%)	1,711 (30.7%)
(Missing)	10 (0.3%)	21 (0.4%)
Religious reading or listening, n (%)		
More than once a day	757 (23.3%)	1,467 (26.3%)
About once a day	1,443 (44.5%)	2,456 (44.1%)
Sometimes	857 (26.4%)	1,350 (24.2%)
Never	181 (5.6%)	285 (5.1%)
(Missing)	5 (0.2%)	9 (0.2%)
Prayer or meditation, n (%)		
More than once a day	1,013 (31.3%)	1,976 (35.5%)
About once a day	1,440 (44.4%)	2,215 (39.8%)
Sometimes	687 (21.2%)	1,137 (20.4%)
Never	102 (3.2%)	230 (4.1%)
(Missing)	0 (0%)	9 (0.2%)
Belief in God/gods/spiritual forces, n (%)		
One God	3,086 (95.2%)	5,281 (94.9%)
More than one god	65 (2.0%)	110 (2.0%)
An impersonal spiritual force	38 (1.2%)	60 (1.1%)
None of these	10 (0.3%)	23 (0.4%)
Unsure	41 (1.3%)	92 (1.7%)
(Missing)	2 (0.1%)	2 (0.0%)
Religious centrality, n (%)		
Agree	2,922 (90.1%)	5,063 (90.9%)
Disagree	175 (5.4%)	223 (4.0%)
Not relevant	23 (0.7%)	33 (0.6%)
Unsure	122 (3.8%)	246 (4.4%)
(Missing)	0 (0.0%)	2 (0.0%)
Religious/spiritual comfort, n (%)		
Agree	3,097 (95.5%)	5,271 (94.7%)
Disagree	74 (2.3%)	121 (2.2%)
Not relevant	15 (0.5%)	36 (0.6%)
Unsure	56 (1.7%)	139 (2.5%)
(Missing)	0 (0.0%)	0 (0%)
Feel loved by God, n (%)		
Agree	3,152 (97.2%)	5,410 (97.2%)
Disagree	52 (1.6%)	64 (1.2%)
Not relevant	4 (0.1%)	8 (0.1%)
Unsure	33 (1.0%)	82 (1.5%)
(Missing)	1 (0.0%)	4 (0.1%)
Feel punished by God, n (%)		
Agree	1,119 (34.5%)	1,942 (34.9%)
Disagree	1,488 (45.9%)	2,574 (46.2%)
Not relevant	114 (3.5%)	140 (2.5%)
Unsure	511 (15.8%)	892 (16.0%)
(Missing)	10 (0.3%)	20 (0.4%)
Experienced religious criticism, n (%)		
Agree	1,446 (44.6%)	2,628 (47.2%)

Table S28d. Unweighted summary statistics for Wave 1 outcome variables in Tanzania by retention status.

**Outcome**	**Attritors–Not Observed in Wave 2**<br>N = 3,242	**Retained–Observed in Wave 2**<br>N = 5,567
Disagree	1,262 (38.9%)	2,058 (37.0%)
Not relevant	93 (2.9%)	136 (2.4%)
Unsure	435 (13.4%)	743 (13.3%)
(Missing)	7 (0.2%)	3 (0.1%)
Faith-sharing, n (%)		
Agree	2,652 (81.8%)	4,686 (84.2%)
Disagree	376 (11.6%)	550 (9.9%)
Not relevant	75 (2.3%)	140 (2.5%)
Unsure	139 (4.3%)	188 (3.4%)
(Missing)	0 (0.0%)	3 (0.1%)

Note. N (%); this table is based on non-imputed data. Cumulative percentages for variables may not add up to 100% due to rounding.

Table S28e. Summary of fitted attrition model in Tanzania

Characteristic	Odds Ratio	95% CI	p-value
**ANNUAL_WEIGHT_R2**	0.88	0.81, 0.95	0.001
**Happiness & life satisfaction**	0.89	0.83, 0.96	0.002
**Physical & mental health**	1.03	0.96, 1.10	0.436
**Meaning & purpose**	1.01	0.94, 1.08	0.805
**Character & virtue**	1.06	0.99, 1.13	0.102
**Close social relationships**	1.02	0.95, 1.09	0.629
**Financial & material security**	1.08	1.01, 1.16	0.032
**Extraversion**	1.04	0.98, 1.10	0.185
**Openness to experience**	1.07	1.01, 1.13	0.022
**Agreeableness**	0.95	0.89, 1.00	0.068
**Conscientiousness**	1.03	0.97, 1.09	0.315
**Neuroticism**	1.04	0.98, 1.11	0.182
**Depression symptoms composite**	0.98	0.92, 1.05	0.533
**Anxiety symptoms composite**	1.00	0.94, 1.07	0.905
**Loneliness**	1.03	0.96, 1.10	0.365
**Days exercise per week**	1.04	0.98, 1.10	0.241
**Year of birth (age group)**			
1983-1993 (current age: 30-39 years)	—	—	
1998-2005 (current age: 18-24 years)	0.84	0.69, 1.01	0.069
1973-1983 (current age: 40-49 years)	1.26	1.06, 1.49	0.008
1993-1998 (current age: 25-29 years)	0.90	0.75, 1.07	0.219
1963-1973 (current age: 50-59 years)	1.31	1.07, 1.59	0.008
1953-1963 (current age: 60-69 years)	1.27	0.96, 1.66	0.090
1943-1953 (current age: 70-79 years)	0.82	0.54, 1.23	0.327
1943 or earlier (current age: 80+ years)	0.55	0.30, 1.01	0.054
**Gender of respondent**			
Female	—	—	
Male	1.33	1.17, 1.52	1.50e-05
**Marital status**			
Married	—	—	
Single/Never been married	0.95	0.81, 1.11	0.486
Separated	0.86	0.67, 1.11	0.242
Widowed	0.80	0.59, 1.08	0.138
Domestic partner	0.98	0.72, 1.33	0.895
Divorced	1.48	0.89, 2.46	0.132
**Employment status**			
Self-employed	—	—	
Homemaker	1.01	0.87, 1.17	0.925
Unemployed and looking for a job	1.02	0.86, 1.21	0.826
Employed for an employer	0.97	0.75, 1.24	0.796
Student	1.27	0.92, 1.76	0.147
Retired	0.98	0.61, 1.56	0.924
None of these/Other	0.97	0.62, 1.51	0.881
**Religious attendance**			
Once a week	—	—	
More than once a week	1.02	0.91, 1.15	0.713
One to three times a month	0.99	0.83, 1.18	0.906
A few times a year	1.18	0.98, 1.43	0.079
Never	1.31	0.92, 1.85	0.128
**Educational attainment (16+ years)**			
Up to 8	—	—	
9-15	1.18	1.01, 1.38	0.032

Table S28e. Summary of fitted attrition model in Tanzania

Characteristic	Odds Ratio	95% CI	p-value
16+	1.35	0.89, 2.07	0.160
**Born in This country**			
Born in this country	—	—	
Born in another country	0.35	0.11, 1.07	0.066
**Race plurality (prominent race/ethnic group [0] or not [1])**	1.04	0.98, 1.10	0.174
**Urbanicity**			
A small town or village	—	—	
A rural area or on a farm	0.87	0.74, 1.03	0.105
A large city	0.87	0.67, 1.13	0.292
A suburb of a large city	0.90	0.70, 1.15	0.403
**Monthly household income**			
Tanzania: 20,001 – 50,000 shillings/month	—	—	
Tanzania: 50,001 – 100,000 shillings/month	1.03	0.87, 1.21	0.759
Tanzania: 20,000 shillings/month or less	1.02	0.84, 1.24	0.836
Tanzania: 100,001 – 150,000 shillings/month	1.12	0.93, 1.33	0.227
Tanzania: 150,001 – 200,000 shillings/month	0.99	0.77, 1.28	0.964
Tanzania: 200,001 – 250,000 shillings/month	0.62	0.48, 0.80	2.58e-04
Tanzania: 250,001 – 300,000 shillings/month	1.23	0.90, 1.67	0.188
Tanzania: 300,001 – 400,000 shillings/month	0.90	0.67, 1.20	0.472
(None/No household income)	0.81	0.57, 1.16	0.249
Tanzania: 400,001 – 500,000 shillings/month	1.36	0.87, 2.14	0.177
Tanzania: 500,001 – 700,000 shillings/month	1.27	0.80, 2.02	0.309
Tanzania: More than 700,000 shillings/month	0.77	0.49, 1.19	0.235

Abbreviations: CI = Confidence Interval, OR = Odds Ratio

Notes. N=9075; attrition weights were estimated using the 'survey::svyglm(family=quasibinomial('logit'))' function. All continuous predictors were standardized and all categorical predictors used the most common category as the reference group. Reported p-values are based on the fitted regression model and no adjustments for multiple testing were done within this table.

Table S28f. Summary of principal components in Tanzania

PC	Percent Explained by each PC	Cumulative Percent Explained
1	29.09	29.09
2	5.00	34.09
3	2.50	36.59
4	2.14	38.73
5	2.03	40.75
6	1.80	42.55
**7**	**1.76**	**44.31**
8	1.53	45.84
9	1.45	47.29
10	1.40	48.69
11	1.38	50.07
12	1.28	51.35
13	1.25	52.60
14	1.22	53.82
15	1.19	55.01
16	1.18	56.19
17	1.14	57.33
18	1.13	58.46
19	1.10	59.56
20	1.08	60.64

Notes. N=9075; PCA was conducted using 'survey::svyprcomp(.)' function using all available contemporaneous exposures at wave 1. All PCs were standardized prior to being used as predictors. The bolded row represented the number of retained components for analysis was 7.

Table S28g. Associations of forgivingness with adult well-being and other outcomes at Wave 2 in Tanzania

Outcome	Model 1: Demographic and Childhood Variables as Covariates					Model 2: Demographic, Childhood, and Other Wave 1 Confounding Variables (Via Principal Components) as Covariates				
	RR	ES	95% CI	SE	p-value	RR	ES	95% CI	SE	p-value
Human Flourishing										
Secure flourishing index		0.03	(0.00,0.05)	0.01	0.046*		0.00	(-0.02,0.03)	0.01	0.734
Flourishing index		0.04	(0.01,0.07)	0.01	4.71e-03**		0.02	(-0.01,0.05)	0.01	0.177
Happiness & life satisfaction		-0.01	(-0.04,0.03)	0.02	0.712		-0.01	(-0.04,0.02)	0.02	0.421
Physical & mental health		0.02	(-0.01,0.05)	0.02	0.210		0.01	(-0.02,0.04)	0.02	0.657
Meaning & purpose		0.03	(-0.00,0.06)	0.01	0.066		0.01	(-0.02,0.04)	0.01	0.519
Character & virtue		0.05	(0.01,0.08)	0.02	0.006*		0.03	(0.00,0.07)	0.02	0.042*
Close social relationships		0.05	(0.02,0.08)	0.01	1.39e-03**		0.03	(-0.00,0.06)	0.02	0.076
Financial & material security		-0.02	(-0.05,0.01)	0.01	0.194		-0.04	(-0.06,-0.01)	0.01	0.011*
Psychological Well-Being										
Happiness (a)		0.00	(-0.03,0.03)	0.02	0.949		-0.01	(-0.04,0.03)	0.02	0.705
Life satisfaction (a)		-0.01	(-0.04,0.02)	0.02	0.535		-0.01	(-0.05,0.02)	0.02	0.380
Current life evaluation		0.01	(-0.03,0.04)	0.02	0.691		0.00	(-0.03,0.04)	0.02	0.782
Future life evaluation		0.00	(-0.03,0.04)	0.02	0.973		-0.00	(-0.04,0.03)	0.02	0.961
Optimism		0.05	(0.01,0.08)	0.02	0.006*		0.03	(-0.00,0.07)	0.02	0.055
Freedom to pursue what's important		0.03	(-0.01,0.06)	0.02	0.100		0.01	(-0.02,0.05)	0.02	0.483
Inner peace	1.05		(1.03,1.06)	0.01	1.17e-07***	1.03		(1.02,1.05)	0.01	3.36e-05***
Life balance	1.02		(1.00,1.04)	0.01	0.018*	1.01		(1.00,1.03)	0.01	0.084
Sense of mastery	1.04		(1.02,1.06)	0.01	5.53e-04***	1.02		(1.00,1.04)	0.01	0.025*
Meaningful activities (c)		0.01	(-0.02,0.04)	0.02	0.530		-0.00	(-0.03,0.03)	0.02	0.940
Understanding purpose (c)		0.03	(0.00,0.06)	0.02	0.038*		0.02	(-0.01,0.05)	0.02	0.299
Self-rated mental health (b)		0.03	(-0.00,0.06)	0.02	0.076		0.02	(-0.01,0.05)	0.02	0.268
Psychological Distress										
Traumatic distress	1.00		(0.99,1.02)	0.01	0.771	1.01		(0.99,1.02)	0.01	0.440
Depression symptoms composite	1.00		(0.99,1.02)	0.01	0.672	1.00		(0.99,1.02)	0.01	0.673
Depression – feel hopeless	1.00		(0.98,1.01)	0.01	0.626	1.00		(0.98,1.02)	0.01	0.940
Depression – loss of interest	1.01		(1.00,1.03)	0.01	0.149	1.01		(0.99,1.03)	0.01	0.236
Anxiety symptoms composite	1.00		(0.99,1.02)	0.01	0.827	1.01		(0.99,1.02)	0.01	0.422
Anxiety – feel on edge	1.00		(0.99,1.01)	0.01	0.970	1.01		(0.99,1.02)	0.01	0.494
Anxiety – cannot stop worrying	1.00		(0.99,1.01)	0.01	0.861	1.00		(0.99,1.02)	0.01	0.728
Suffering	1.00		(0.99,1.02)	0.01	0.530	1.01		(1.00,1.02)	0.01	0.185
Social Well-Being										
Relationship contentment (e)		0.03	(-0.00,0.07)	0.02	0.053		0.02	(-0.02,0.05)	0.02	0.394
Relationship satisfaction (e)		0.04	(0.01,0.08)	0.02	0.012*		0.03	(-0.01,0.06)	0.02	0.104
Social support		0.01	(-0.03,0.04)	0.02	0.752		-0.01	(-0.04,0.03)	0.02	0.680
Intimate/close friend	1.00		(0.99,1.01)	0.01	0.828	0.99		(0.98,1.01)	0.01	0.262
Government approval	1.00		(0.99,1.01)	0.00	0.773	1.00		(0.99,1.01)	0.00	0.866
Say in government	1.00		(0.98,1.02)	0.01	0.905	1.00		(0.98,1.01)	0.01	0.810
Belonging in country		0.05	(0.02,0.09)	0.02	0.005*		0.04	(0.00,0.07)	0.02	0.053
City/place satisfaction	1.00		(0.98,1.01)	0.01	0.665	1.00		(0.98,1.01)	0.01	0.645
Trust within country	1.00		(0.99,1.01)	0.01	0.785	1.00		(0.99,1.01)	0.01	0.694
Social Participation										
Ever been married	1.00		(0.99,1.01)	0.01	0.805	1.00		(0.99,1.01)	0.01	0.520
Currently divorced	1.00		(1.00,1.00)	0.00	0.888	1.00		(1.00,1.00)	0.00	0.715
Number of children		0.01	(-0.01,0.04)	0.01	0.319		0.02	(-0.02,0.05)	0.02	0.334
Weekly+ community participation	1.00		(0.99,1.02)	0.01	0.581	1.00		(0.99,1.01)	0.01	0.816
Weekly+ religious attendance	1.00		(0.99,1.02)	0.01	0.601	1.00		(0.98,1.01)	0.01	0.630
Social Distress										
Loneliness		-0.01	(-0.04,0.03)	0.02	0.699		0.00	(-0.03,0.04)	0.02	0.840
Perceived discrimination	1.00		(0.99,1.02)	0.01	0.461	1.01		(1.00,1.02)	0.01	0.260
Character & Prosocial Behavior										
Orientation to promote good (d)		0.05	(0.01,0.09)	0.02	0.008*		0.04	(0.00,0.07)	0.02	0.040*
Delayed gratification (d)		0.02	(-0.01,0.06)	0.02	0.187		0.02	(-0.02,0.05)	0.02	0.346
Hope		0.03	(-0.00,0.07)	0.02	0.079		0.02	(-0.02,0.05)	0.02	0.386
Gratitude		0.03	(-0.01,0.07)	0.02	0.100		0.02	(-0.02,0.05)	0.02	0.363
Showing love/care		0.05	(0.01,0.08)	0.02	0.022*		0.03	(-0.01,0.06)	0.02	0.187
Forgivingness	1.07		(1.05,1.09)	0.01	2.22e-16***	1.06		(1.05,1.08)	0.01	6.46e-14***
Charitable giving	1.02		(1.00,1.03)	0.01	0.064	1.01		(0.99,1.03)	0.01	0.216
Helping strangers	1.01		(0.99,1.03)	0.01	0.203	1.01		(0.99,1.02)	0.01	0.454
Volunteering	1.00		(0.99,1.02)	0.01	0.736	1.00		(0.99,1.01)	0.01	0.965
Physical Health & Health Behavior										
Self-rated physical health (b)		0.01	(-0.03,0.04)	0.02	0.639		-0.00	(-0.04,0.03)	0.02	0.858
Health problems	1.00		(0.99,1.01)	0.01	0.735	1.00		(0.99,1.01)	0.01	0.960
Pain in past 4 weeks	1.01		(0.99,1.02)	0.01	0.382	1.01		(1.00,1.03)	0.01	0.159
Daily smoker	1.00		(0.99,1.00)	0.00	0.269	1.00		(0.99,1.01)	0.00	0.546
Number of drinks per week		0.00	(-0.03,0.03)	0.02	0.894		0.00	(-0.03,0.04)	0.02	0.845
Days exercise per week		0.04	(0.00,0.08)	0.02	0.043*		0.03	(-0.01,0.07)	0.02	0.131
Socioeconomic Outcomes										
Financial security (f)		-0.02	(-0.05,0.01)	0.02	0.244		-0.03	(-0.06,-0.00)	0.02	0.047*
Material security (f)		-0.01	(-0.05,0.02)	0.02	0.401		-0.03	(-0.06,0.00)	0.02	0.073
Educational attainment (16+ years)	1.00		(1.00,1.00)	0.00	0.514	1.00		(1.00,1.00)	0.00	0.517
Currently employed	0.99		(0.98,1.01)	0.01	0.348	0.99		(0.98,1.01)	0.01	0.282
Financially comfortable/getting by	1.00		(0.98,1.02)	0.01	0.989	1.00		(0.98,1.01)	0.01	0.638
Own home	0.99		(0.97,1.00)	0.01	0.065	0.99		(0.97,1.00)	0.01	0.071
Income – top quintile	1.00		(0.99,1.01)	0.01	0.860	1.00		(0.98,1.01)	0.01	0.518
Religion & Spirituality										
Religious/spiritual connection	1.03		(1.01,1.04)	0.01	1.31e-04***	1.01		(1.00,1.03)	0.01	0.058
Belief in life after death	1.02		(1.00,1.03)	0.01	0.076	1.01		(0.99,1.03)	0.01	0.337
Transformative religious experience	1.02		(1.01,1.04)	0.01	6.09e-04***	1.01		(1.00,1.02)	0.01	0.133
Religious reading or listening	1.01		(1.00,1.03)	0.01	0.168	1.00		(0.99,1.02)	0.01	0.696
Prayer or meditation	1.01		(0.99,1.02)	0.01	0.228	1.00		(0.99,1.01)	0.01	0.996
Belief in God/gods/spiritual forces	1.00		(1.00,1.01)	0.00	0.455	1.00		(0.99,1.01)	0.00	0.876
Religious centrality	1.01		(1.00,1.02)	0.01	0.186	1.00		(0.99,1.01)	0.01	0.550
Religious/spiritual comfort	1.00		(0.99,1.01)	0.00	0.766	1.00		(0.99,1.01)	0.00	0.697
Feel loved by God	1.00		(1.00,1.01)	0.00	0.491	1.00		(1.00,1.01)	0.00	0.770
Feel punished by God	0.98		(0.97,1.00)	0.01	0.049*	0.99		(0.97,1.01)	0.01	0.197
Experienced religious criticism	0.98		(0.96,1.00)	0.01	0.025*	0.99		(0.97,1.00)	0.01	0.149
Faith-sharing	1.00		(0.99,1.02)	0.01	0.557	1.00		(0.99,1.01)	0.01	0.822

Notes. N=9075; Reference for focal predictor: never/rarely. RR, risk-ratio, null effect is 1.00; ES, effect size measure for standardized regression coefficient, null effect is 0.00; SE, standard error, the SE reported for binary/Likert-type outcomes where risk-ratios are on the log(RR) scale; CI, confidence interval; p-value, a Wald-type test of the null hypothesis that the effect of the focal predictor is zero; (a) item part of the Happiness & Life Satisfaction domain of the Secure Flourishing Index; (b) item part of the Physical & Mental Health domain of the Secure Flourishing Index; (c) item part of the Meaning & Purpose domain of the Secure Flourishing Index; (d) item part of the Character & Virtue domain of the Secure Flourishing Index; (e) item part of the Subjective Social Connectedness domain of the Secure Flourishing Index; (f) item part of the Financial & Material Security domain of the Secure Flourishing Index.

Multiple imputation was performed to impute missing data on the covariates, exposure, and outcomes. All models controlled for sociodemographic and childhood factors assessed at Wave 1. For Model 2 with PC (principal components), the first seven principal components of the entire set of contemporaneous confounders assessed at Wave 1 were included as additional covariates of the outcomes at Wave 2.

An outcome-wide analytic approach was used, and a separate model was run for each outcome. A different type of model was run depending on the nature of the outcome: (1) for each binary outcome, a weighted generalized linear model (with a log link and Poisson distribution) was used to estimate an RR; and (2) for each continuous outcome, a weighted linear regression model was used to estimate a ES. All effect sizes were standardized. For continuous outcomes, the ES represents the change in SD on the outcome between the lower and upper categories of the binary focal predictor. For binary outcomes, the RR represents the change in risk of being in the upper category compared to the lower category between the lower and upper categories of the binary focal predictor.

P-value significance thresholds: $p < 0.05$*, $p < 0.005$**, (Bonferroni) $p < 6.41e-04$***, correction for multiple testing using Bonferroni adjusted significant threshold.

Table S28h. Associations of forgivingness with adult well-being and other outcomes at Wave 2 in Tanzania using complete-case analyses with attrition weights.

	Model 1: Demographic and Childhood Variables as Covariates					Model 2: Demographic, Childhood, and Other Wave 1 Confounding Variables (Via Principal Components) as Covariates				
Outcome	RR	ES	95% CI	SE	p-value	RR	ES	95% CI	SE	p-value
Human Flourishing										
Secure flourishing index		0.02	(-0.02,0.05)	0.02	0.295		-0.00	(-0.03,0.03)	0.02	0.947
Flourishing index		0.03	(-0.00,0.06)	0.02	0.089		0.01	(-0.02,0.04)	0.02	0.458
Happiness & life satisfaction		-0.02	(-0.06,0.01)	0.02	0.227		-0.03	(-0.06,0.01)	0.02	0.145
Physical & mental health		0.02	(-0.01,0.05)	0.02	0.157		0.01	(-0.02,0.04)	0.02	0.428
Meaning & purpose		0.02	(-0.01,0.05)	0.02	0.190		0.01	(-0.03,0.04)	0.02	0.722
Character & virtue		0.03	(-0.00,0.07)	0.02	0.056		0.02	(-0.01,0.06)	0.02	0.174
Close social relationships		0.05	(0.01,0.08)	0.02	0.006*		0.03	(-0.00,0.06)	0.02	0.089
Financial & material security		-0.02	(-0.06,0.02)	0.02	0.272		-0.04	(-0.07,-0.00)	0.02	0.031*
Psychological Well-Being										
Happiness (a)		-0.01	(-0.05,0.02)	0.02	0.515		-0.02	(-0.05,0.02)	0.02	0.327
Life satisfaction (a)		-0.02	(-0.06,0.01)	0.02	0.212		-0.03	(-0.06,0.01)	0.02	0.183
Current life evaluation		0.00	(-0.04,0.04)	0.02	0.903		0.00	(-0.04,0.04)	0.02	0.841
Future life evaluation		0.00	(-0.04,0.04)	0.02	0.881		0.01	(-0.03,0.04)	0.02	0.780
Optimism		0.04	(0.00,0.07)	0.02	0.033*		0.02	(-0.01,0.06)	0.02	0.178
Freedom to pursue what's important		0.02	(-0.01,0.06)	0.02	0.157		0.01	(-0.02,0.05)	0.02	0.510
Inner peace	1.05		(1.03,1.07)	0.01	3.53e-07***	1.04		(1.02,1.05)	0.01	1.22e-04***
Life balance	1.02		(1.00,1.04)	0.01	0.037*	1.01		(0.99,1.03)	0.01	0.169
Sense of mastery	1.03		(1.02,1.05)	0.01	7.45e-05***	1.02		(1.00,1.04)	0.01	0.028*
Meaningful activities (c)		0.00	(-0.03,0.04)	0.02	0.815		-0.01	(-0.04,0.03)	0.02	0.775
Understanding purpose (c)		0.03	(-0.00,0.07)	0.02	0.073		0.02	(-0.02,0.05)	0.02	0.362
Self-rated mental health (b)		0.03	(-0.00,0.06)	0.02	0.069		0.02	(-0.01,0.05)	0.02	0.196
Psychological Distress										
Traumatic distress	1.00		(0.99,1.02)	0.01	0.808	1.01		(0.99,1.02)	0.01	0.521
Depression symptoms composite	1.00		(0.98,1.01)	0.01	0.802	1.00		(0.98,1.01)	0.01	0.617
Depression – feel hopeless	0.99		(0.97,1.01)	0.01	0.288	0.99		(0.98,1.01)	0.01	0.467
Depression – loss of interest	1.01		(0.99,1.03)	0.01	0.246	1.01		(0.99,1.02)	0.01	0.378
Anxiety symptoms composite	1.00		(0.99,1.02)	0.01	0.867	1.01		(0.99,1.02)	0.01	0.537
Anxiety – feel on edge	1.00		(0.98,1.02)	0.01	0.853	1.00		(0.99,1.02)	0.01	0.581
Anxiety – cannot stop worrying	1.00		(0.99,1.02)	0.01	0.908	1.01		(0.99,1.02)	0.01	0.518
Suffering	1.00		(0.99,1.02)	0.01	0.668	1.01		(0.99,1.03)	0.01	0.326
Social Well-Being										
Relationship contentment (e)		0.03	(0.00,0.07)	0.02	0.041*		0.02	(-0.02,0.05)	0.02	0.343
Relationship satisfaction (e)		0.05	(0.01,0.09)	0.02	0.007*		0.04	(-0.00,0.07)	0.02	0.055
Social support		0.00	(-0.03,0.04)	0.02	0.851		-0.01	(-0.04,0.03)	0.02	0.596
Intimate/close friend	1.00		(0.98,1.01)	0.01	0.479	0.99		(0.98,1.00)	0.01	0.172
Government approval	1.00		(0.99,1.01)	0.00	0.458	1.00		(0.99,1.01)	0.00	0.501
Say in government	1.00		(0.98,1.02)	0.01	0.771	0.99		(0.98,1.01)	0.01	0.536
Belonging in country		0.06	(0.02,0.09)	0.02	3.53e-03**		0.04	(0.01,0.08)	0.02	0.024*
City/place satisfaction	0.99		(0.98,1.01)	0.01	0.329	0.99		(0.98,1.01)	0.01	0.338
Trust within country	1.00		(0.99,1.02)	0.01	0.718	1.00		(0.99,1.02)	0.01	0.530
Social Participation										
Ever been married	1.00		(0.99,1.01)	0.01	0.848	1.00		(0.99,1.02)	0.01	0.512
Currently divorced	1.00		(0.99,1.00)	0.00	0.754	1.00		(1.00,1.00)	0.00	0.987
Number of children		0.02	(-0.01,0.06)	0.02	0.201		0.02	(-0.02,0.05)	0.02	0.317
Weekly+ community participation	1.01		(0.99,1.02)	0.01	0.455	1.00		(0.99,1.02)	0.01	0.702
Weekly+ religious attendance	1.00		(0.99,1.02)	0.01	0.947	0.99		(0.98,1.01)	0.01	0.383
Social Distress										
Loneliness		-0.02	(-0.06,0.02)	0.02	0.406		-0.01	(-0.05,0.03)	0.02	0.588
Perceived discrimination	1.01		(1.00,1.02)	0.01	0.088	1.01		(1.00,1.02)	0.01	0.043*
Character & Prosocial Behavior										
Orientation to promote good (d)		0.05	(0.01,0.08)	0.02	0.013*		0.03	(-0.00,0.07)	0.02	0.058
Delayed gratification (d)		0.01	(-0.02,0.04)	0.02	0.504		0.01	(-0.03,0.04)	0.02	0.674
Hope		0.02	(-0.01,0.05)	0.02	0.171		0.01	(-0.03,0.04)	0.02	0.637
Gratitude		0.03	(-0.00,0.07)	0.02	0.061		0.02	(-0.01,0.05)	0.02	0.241
Showing love/care		0.04	(0.00,0.08)	0.02	0.031*		0.02	(-0.02,0.06)	0.02	0.297
Forgivingness	1.07		(1.05,1.09)	0.01	1.13e-12***	1.06		(1.04,1.08)	0.01	2.57e-10***
Charitable giving	1.02		(1.00,1.03)	0.01	0.052	1.01		(1.00,1.03)	0.01	0.131
Helping strangers	1.00		(0.98,1.02)	0.01	0.787	1.00		(0.98,1.02)	0.01	0.763
Volunteering	1.00		(0.99,1.01)	0.01	0.888	1.00		(0.99,1.01)	0.01	0.834
Physical Health & Health Behavior										
Self-rated physical health (b)		0.01	(-0.02,0.05)	0.02	0.507		0.00	(-0.03,0.04)	0.02	0.857
Health problems	0.99		(0.98,1.01)	0.01	0.387	0.99		(0.98,1.01)	0.01	0.403
Pain in past 4 weeks	1.01		(0.99,1.03)	0.01	0.296	1.02		(1.00,1.03)	0.01	0.083
Daily smoker	1.00		(0.99,1.00)	0.00	0.261	1.00		(0.99,1.01)	0.00	0.553
Number of drinks per week		-0.00	(-0.04,0.03)	0.02	0.787		-0.00	(-0.04,0.03)	0.02	0.900
Days exercise per week		0.04	(0.00,0.08)	0.02	0.028*		0.03	(-0.01,0.07)	0.02	0.126
Socioeconomic Outcomes										
Financial security (f)		-0.02	(-0.06,0.01)	0.02	0.187		-0.03	(-0.07,-0.00)	0.02	0.040*
Material security (f)		-0.01	(-0.05,0.02)	0.02	0.478		-0.03	(-0.07,0.00)	0.02	0.061
Educational attainment (16+ years)	1.00		(1.00,1.00)	0.00	0.285	1.00		(1.00,1.00)	0.00	0.411
Currently employed	0.99		(0.98,1.01)	0.01	0.347	0.99		(0.98,1.01)	0.01	0.377
Financially comfortable/getting by	1.00		(0.98,1.02)	0.01	0.887	1.00		(0.98,1.01)	0.01	0.674
Own home	0.99		(0.97,1.00)	0.01	0.125	0.99		(0.97,1.00)	0.01	0.129
Income – top quintile	1.00		(0.98,1.01)	0.01	0.713	0.99		(0.98,1.01)	0.01	0.394
Religion & Spirituality										
Religious/spiritual connection	1.03		(1.01,1.05)	0.01	2.69e-04***	1.02		(1.00,1.03)	0.01	0.043*
Belief in life after death	1.02		(1.00,1.03)	0.01	0.083	1.01		(0.99,1.03)	0.01	0.360
Transformative religious experience	1.02		(1.01,1.04)	0.01	0.008*	1.01		(0.99,1.03)	0.01	0.181
Religious reading or listening	1.01		(1.00,1.03)	0.01	0.116	1.00		(0.99,1.02)	0.01	0.647
Prayer or meditation	1.01		(0.99,1.02)	0.01	0.432	1.00		(0.98,1.01)	0.01	0.591
Belief in God/gods/spiritual forces	1.00		(0.99,1.01)	0.00	0.775	1.00		(0.99,1.01)	0.00	0.664
Religious centrality	1.01		(1.00,1.02)	0.01	0.090	1.01		(1.00,1.02)	0.01	0.238
Religious/spiritual comfort	1.00		(0.99,1.01)	0.00	0.555	1.00		(0.99,1.01)	0.00	0.783
Feel loved by God	1.00		(1.00,1.01)	0.00	0.439	1.00		(0.99,1.01)	0.00	0.638
Feel punished by God	0.99		(0.97,1.00)	0.01	0.120	0.99		(0.98,1.01)	0.01	0.337
Experienced religious criticism	0.97		(0.95,0.99)	0.01	3.32e-03**	0.98		(0.96,1.00)	0.01	0.046*
Faith-sharing	1.00		(0.99,1.01)	0.01	0.897	1.00		(0.98,1.01)	0.01	0.572

Table S28h. Associations of forgivingness with adult well-being and other outcomes at Wave 2 in Tanzania using complete-case analyses with attrition weights.

	Model 1: Demographic and Childhood Variables as Covariates					Model 2: Demographic, Childhood, and Other Wave 1 Confounding Variables (Via Principal Components) as Covariates				
Outcome	RR	ES	95% CI	SE	p-value	RR	ES	95% CI	SE	p-value

Notes. N=5583; Reference for focal predictor: never/rarely. RR, risk-ratio, null effect is 1.00; ES, effect size measure for standardized regression coefficient, null effect is 0.00; SE, standard error, the SE reported for binary/Likert-type outcomes where risk-ratios are on the log(RR) scale; CI, confidence interval; p-value, a Wald-type test of the null hypothesis that the effect of the focal predictor is zero; (a) item part of the Happiness & Life Satisfaction domain of the Secure Flourishing Index; (b) item part of the Physical & Mental Health domain of the Secure Flourishing Index; (c) item part of the Meaning & Purpose domain of the Secure Flourishing Index; (d) item part of the Character & Virtue domain of the Secure Flourishing Index; (e) item part of the Subjective Social Connectedness domain of the Secure Flourishing Index; (f) item part of the Financial & Material Security domain of the Secure Flourishing Index.

Attrition weights were computed to adjust the complete case data (those who responded at Wave 2 to at least 50% of the questions) and multiple imputation was used to impute missing data on all remaining within wave on the covariates, exposure, and outcomes. All models controlled for sociodemographic and childhood factors assessed at Wave 1. For Model 2 with PC (principal components), the first seven principal components of the entire set of contemporaneous confounders assessed at Wave 1 were included as additional covariates of the outcomes at Wave 2.

An outcome-wide analytic approach was used, and a separate model was run for each outcome. A different type of model was run depending on the nature of the outcome: (1) for each binary outcome, a weighted generalized linear model (with a log link and Poisson distribution) was used to estimate an RR; and (2) for each continuous outcome, a weighted linear regression model was used to estimate a ES. All effect sizes were standardized. For continuous outcomes, the ES represents the change in SD on the outcome between the lower and upper categories of the binary focal predictor. For binary outcomes, the RR represents the change in risk of being in the upper category compared to the lower category between the lower and upper categories of the binary focal predictor.

P-value significance thresholds: p < 0.05*, p < 0.005**, (Bonferroni) p < 6.41e-04***, correction for multiple testing using Bonferroni adjusted significant threshold.

Table S28i. Sensitivity analysis of forgivingness outcome-wide results to unmeasured confounding using E-values in Tanzania

Outcome	Multiple Imputation				Complete Case w/ Attrition Weights			
	Model 1: Demographics and Childhood Variables as Covariates		Model 2: Demographics, Childhood, and Other Wave 1 Confounders (Via Principal Components) as Covariates		Model 1: Demographics and Childhood Variables as Covariates		Model 2: Demographics, Childhood, and Other Wave 1 Confounders (Via Principal Components) as Covariates	
	EE	ECI	EE	ECI	EE	ECI	EE	ECI
Human Flourishing								
Secure flourishing index	1.34	1.04	1.11	1.00	1.26	1.00	1.05	1.00
Flourishing index	1.44	1.21	1.27	1.00	1.35	1.00	1.20	1.00
Happiness & life satisfaction	1.14	1.00	1.22	1.00	1.29	1.00	1.33	1.00
Physical & mental health	1.28	1.00	1.15	1.00	1.30	1.00	1.21	1.00
Meaning & purpose	1.34	1.00	1.18	1.00	1.29	1.00	1.13	1.00
Character & virtue	1.49	1.22	1.40	1.07	1.39	1.00	1.31	1.00
Close social relationships	1.50	1.27	1.34	1.00	1.50	1.22	1.36	1.00
Financial & material security	1.28	1.00	1.41	1.17	1.28	1.00	1.42	1.10
Psychological Well-Being								
Happiness	1.06	1.00	1.14	1.00	1.20	1.00	1.26	1.00
Life satisfaction	1.19	1.00	1.23	1.00	1.32	1.00	1.33	1.00
Current life evaluation	1.15	1.00	1.12	1.00	1.08	1.00	1.11	1.00
Future life evaluation	1.04	1.00	1.05	1.00	1.09	1.00	1.13	1.00
Optimism	1.50	1.23	1.40	1.00	1.42	1.10	1.31	1.00
Freedom to pursue what's important	1.34	1.00	1.21	1.00	1.32	1.00	1.20	1.00
Inner peace	1.51	1.38	1.42	1.28	1.53	1.38	1.43	1.27
Life balance	1.29	1.10	1.24	1.00	1.29	1.06	1.22	1.00
Sense of mastery	1.43	1.26	1.32	1.10	1.42	1.27	1.28	1.08
Meaningful activities	1.19	1.00	1.06	1.00	1.11	1.00	1.13	1.00
Understanding purpose	1.39	1.08	1.25	1.00	1.38	1.00	1.24	1.00
Self-rated mental health	1.34	1.00	1.26	1.00	1.36	1.00	1.29	1.00
Psychological Distress								
Traumatic distress	1.08	1.00	1.15	1.00	1.08	1.00	1.13	1.00
Depression symptoms composite	1.10	1.00	1.10	1.00	1.08	1.00	1.12	1.00
Depression – feel hopeless	1.12	1.00	1.04	1.00	1.19	1.00	1.15	1.00
Depression – loss of interest	1.22	1.00	1.19	1.00	1.19	1.00	1.17	1.00
Anxiety symptoms composite	1.07	1.00	1.14	1.00	1.06	1.00	1.13	1.00
Anxiety – feel on edge	1.03	1.00	1.13	1.00	1.07	1.00	1.12	1.00
Anxiety – cannot stop worrying	1.06	1.00	1.09	1.00	1.05	1.00	1.13	1.00
Suffering	1.12	1.00	1.19	1.00	1.11	1.00	1.18	1.00
Social Well-Being								
Relationship contentment	1.40	1.00	1.24	1.00	1.39	1.06	1.24	1.00
Relationship satisfaction	1.47	1.19	1.35	1.00	1.52	1.22	1.41	1.00
Social support	1.13	1.00	1.15	1.00	1.10	1.00	1.18	1.00
Intimate/close friend	1.06	1.00	1.16	1.00	1.13	1.00	1.19	1.00
Government approval	1.06	1.00	1.05	1.00	1.10	1.00	1.10	1.00
Say in government	1.06	1.00	1.08	1.00	1.09	1.00	1.14	1.00
Belonging in country	1.53	1.24	1.41	1.02	1.55	1.26	1.45	1.13
City/place satisfaction	1.10	1.00	1.11	1.00	1.16	1.00	1.16	1.00
Trust within country	1.07	1.00	1.09	1.00	1.09	1.00	1.12	1.00
Social Participation								
Ever been married	1.06	1.00	1.11	1.00	1.06	1.00	1.12	1.00
Currently divorced	1.03	1.00	1.05	1.00	1.05	1.00	1.01	1.00
Number of children	1.23	1.00	1.24	1.00	1.30	1.00	1.27	1.00
Weekly+ community participation	1.11	1.00	1.07	1.00	1.14	1.00	1.09	1.00
Weekly+ religious attendance	1.11	1.00	1.11	1.00	1.04	1.00	1.16	1.00
Social Distress								
Loneliness	1.15	1.00	1.10	1.00	1.25	1.00	1.19	1.00
Perceived discrimination	1.12	1.00	1.16	1.00	1.19	1.00	1.22	1.03
Character & Prosocial Behavior								
Orientation to promote good	1.52	1.22	1.43	1.08	1.49	1.18	1.39	1.00
Delayed gratification	1.31	1.00	1.25	1.00	1.20	1.00	1.16	1.00
Hope	1.38	1.00	1.25	1.00	1.30	1.00	1.16	1.00
Gratitude	1.37	1.00	1.26	1.00	1.38	1.00	1.28	1.00
Showing love/care	1.48	1.16	1.33	1.00	1.44	1.11	1.27	1.00
Forgivingness	1.69	1.57	1.64	1.52	1.66	1.52	1.60	1.46
Charitable giving	1.26	1.00	1.20	1.00	1.26	1.00	1.22	1.00
Helping strangers	1.21	1.00	1.15	1.00	1.09	1.00	1.10	1.00
Volunteering	1.09	1.00	1.03	1.00	1.05	1.00	1.07	1.00
Physical Health & Health Behavior								
Self-rated physical health	1.16	1.00	1.09	1.00	1.20	1.00	1.09	1.00
Health problems	1.08	1.00	1.03	1.00	1.15	1.00	1.14	1.00
Pain in past 4 weeks	1.16	1.00	1.21	1.00	1.19	1.00	1.25	1.00
Daily smoker	1.12	1.00	1.09	1.00	1.13	1.00	1.09	1.00
Number of drinks per week	1.08	1.00	1.10	1.00	1.12	1.00	1.08	1.00
Days exercise per week	1.43	1.07	1.36	1.00	1.44	1.11	1.35	1.00
Socioeconomic Outcomes								
Financial security	1.27	1.00	1.37	1.04	1.31	1.00	1.40	1.07
Material security	1.23	1.00	1.37	1.00	1.21	1.00	1.38	1.00
Educational attainment (16+ years)	1.04	1.00	1.04	1.00	1.04	1.00	1.04	1.00
Currently employed	1.16	1.00	1.18	1.00	1.16	1.00	1.16	1.00
Financially comfortable/getting by	1.02	1.00	1.11	1.00	1.06	1.00	1.11	1.00
Own home	1.24	1.00	1.23	1.00	1.24	1.00	1.24	1.00
Income – top quintile	1.06	1.00	1.12	1.00	1.10	1.00	1.16	1.00
Religion & Spirituality								
Religious/spiritual connection	1.36	1.23	1.23	1.00	1.39	1.24	1.26	1.04
Belief in life after death	1.26	1.00	1.17	1.00	1.25	1.00	1.17	1.00
Transformative religious experience	1.33	1.20	1.20	1.00	1.33	1.15	1.22	1.00
Religious reading or listening	1.21	1.00	1.10	1.00	1.23	1.00	1.12	1.00
Prayer or meditation	1.18	1.00	1.01	1.00	1.14	1.00	1.11	1.00
Belief in God/gods/spiritual forces	1.09	1.00	1.04	1.00	1.06	1.00	1.07	1.00
Religious centrality	1.16	1.00	1.10	1.00	1.20	1.00	1.16	1.00
Religious/spiritual comfort	1.06	1.00	1.07	1.00	1.09	1.00	1.06	1.00
Feel loved by God	1.08	1.00	1.05	1.00	1.09	1.00	1.07	1.00
Feel punished by God	1.26	1.02	1.20	1.00	1.23	1.00	1.17	1.00
Experienced religious criticism	1.30	1.09	1.23	1.00	1.37	1.19	1.29	1.03
Faith-sharing	1.11	1.00	1.07	1.00	1.05	1.00	1.11	1.00

Notes. EE, E-value for estimate; ECI, E-value for the limit of the confidence interval. The formula for calculating E-values can be found in VanderWeele and Ding (2017). E-values for estimate are the minimum strength of association on the risk ratio scale that an unmeasured confounder would need to have with both the exposure and the outcome to fully explain away the observed association between the exposure and outcome, conditional on the measured covariates. E-values for the 95% CI closest to the null denote the minimum strength of association on the risk ratio scale that an unmeasured confounder would need to have with both the exposure and the outcome to shift the CI to include the null value, conditional on the measured covariates.

Table S29a. Weighted summary statistics for demographic and childhood variables in Turkey

**Characteristic**	**Wave 1** N = 1,473	**Wave 2** N = 497
Forgivingness, n (%)		
Always	318 (21.6%)	112 (22.5%)
Often	283 (19.2%)	86 (17.2%)
Rarely	509 (34.6%)	183 (36.8%)
Never	350 (23.8%)	117 (23.5%)
(Missing)	12 (0.8%)	0 (0%)
Year of birth, n (%)		
1943 or earlier (current age: 80+ years)	20 (1.4%)	5 (1.1%)
1943-1953 (current age: 70-79 years)	72 (4.9%)	20 (4.1%)
1953-1963 (current age: 60-69 years)	171 (11.6%)	53 (10.7%)
1963-1973 (current age: 50-59 years)	211 (14.3%)	89 (18.0%)
1973-1983 (current age: 40-49 years)	295 (20.0%)	85 (17.1%)
1983-1993 (current age: 30-39 years)	309 (21.0%)	108 (21.8%)
1993-1998 (current age: 25-29 years)	155 (10.5%)	69 (13.9%)
1998-2005 (current age: 18-24 years)	240 (16.3%)	67 (13.4%)
(Missing)	0 (0%)	0 (0%)
Age of participant		
Mean	42.2	42.2
Standard Deviation	16.4	15.6
Min, Max	18.0, 93.0	19.0, 83.0
Gender, n (%)		
Male	736 (50.0%)	255 (51.2%)
Female	737 (50.0%)	242 (48.8%)
Other	0 (0%)	0 (0%)
(Missing)	0 (0%)	0 (0%)
Respondent marital status, n (%)		
Single/Never been married	375 (25.4%)	118 (23.8%)
Married	941 (63.9%)	325 (65.4%)
Separated	12 (0.8%)	2 (0.5%)
Divorced	60 (4.0%)	19 (3.7%)
Widowed	69 (4.7%)	31 (6.3%)
Domestic partner	0 (0%)	0 (0%)
(Missing)	17 (1.1%)	2 (0.3%)
Education (years), n (%)		
Up to 8	452 (30.7%)	134 (27.0%)
9-15	730 (49.6%)	229 (46.2%)
16+	290 (19.7%)	133 (26.8%)
(Missing)	0 (0%)	0 (0%)
Employment status, n (%)		
Employed for an employer	403 (27.3%)	146 (29.3%)
Self-employed	248 (16.8%)	76 (15.3%)
Retired	206 (14.0%)	69 (13.9%)
Student	112 (7.6%)	23 (4.5%)
Homemaker	361 (24.5%)	108 (21.7%)
Unemployed and looking for a job	86 (5.8%)	43 (8.7%)
None of these/Other	58 (3.9%)	26 (5.3%)
(Missing)	0 (0%)	6 (1.3%)
Current religious service attendance, n (%)		
More than once a week	499 (33.9%)	212 (42.7%)
Once a week	274 (18.6%)	67 (13.6%)

Table S29a. Weighted summary statistics for demographic and childhood variables in Turkey

**Characteristic**	**Wave 1**<br>N = 1,473	**Wave 2**<br>N = 497
One to three times a month	175 (11.9%)	52 (10.4%)
A few times a year	252 (17.1%)	78 (15.6%)
Never	268 (18.2%)	81 (16.2%)
(Missing)	5 (0.4%)	7 (1.5%)
Immigration status, n (%)		
Born in this country	1,413 (95.9%)	476 (95.9%)
Born in another country	60 (4.1%)	21 (4.1%)
(Missing)	0 (0%)	0 (0%)
Parental marital status around age 12, n (%)		
Parents were married	1,325 (89.9%)	455 (91.6%)
Parents were divorced	58 (4.0%)	14 (2.8%)
Parents were never married	7 (0.5%)	1 (0.2%)
One or both of them had died	60 (4.1%)	23 (4.6%)
Unsure	4 (0.3%)	3 (0.6%)
(Missing)	18 (1.2%)	1 (0.2%)
Religious service attendance around age 12, n (%)		
At least once a week	609 (41.3%)	230 (46.3%)
One to three times a month	236 (16.0%)	69 (13.9%)
Less than once a month	220 (15.0%)	64 (12.9%)
Never	390 (26.5%)	124 (24.9%)
(Missing)	18 (1.2%)	10 (2.1%)
Relationship with mother when growing up, n (%)		
Very good	971 (65.9%)	315 (63.3%)
Somewhat good	398 (27.0%)	147 (29.7%)
Somewhat bad	47 (3.2%)	18 (3.5%)
Very bad	27 (1.9%)	11 (2.2%)
(Does not apply)	21 (1.4%)	5 (0.9%)
(Missing)	8 (0.6%)	1 (0.3%)
Relationship with father when growing up, n (%)		
Very good	801 (54.4%)	259 (52.2%)
Somewhat good	421 (28.6%)	139 (28.0%)
Somewhat bad	73 (5.0%)	40 (8.1%)
Very bad	95 (6.5%)	34 (6.8%)
(Does not apply)	58 (4.0%)	16 (3.2%)
(Missing)	24 (1.6%)	9 (1.8%)
Felt like an outsider in family when growing up, n (%)		
Yes	157 (10.7%)	48 (9.7%)
No	1,307 (88.7%)	438 (88.3%)
(Missing)	9 (0.6%)	10 (2.0%)
Experienced abuse when growing up, n (%)		
Yes	159 (10.8%)	53 (10.6%)
No	1,290 (87.6%)	441 (88.7%)
(Missing)	25 (1.7%)	3 (0.6%)
Self-rated health when growing up, n (%)		
Excellent	369 (25.0%)	124 (24.9%)
Very good	412 (28.0%)	137 (27.6%)
Good	424 (28.8%)	140 (28.2%)
Fair	220 (14.9%)	75 (15.1%)
Poor	48 (3.3%)	21 (4.2%)
(Missing)	1 (0.0%)	0 (0%)

Table S29a. Weighted summary statistics for demographic and childhood variables in Turkey

Characteristic	Wave 1<br>N = 1,473	Wave 2<br>N = 497
Subjective financial status of family growing up, n (%)		
Lived comfortably	496 (33.7%)	156 (31.5%)
Got by	648 (44.0%)	226 (45.5%)
Found it difficult	219 (14.8%)	72 (14.5%)
Found it very difficult	109 (7.4%)	43 (8.6%)
(Missing)	2 (0.1%)	0 (0%)
Religious affiliation growing up, n (%)		
Christianity	1 (0.1%)	1 (0.1%)
Taoism	0 (0%)	0 (0%)
Confucianism	0 (0%)	0 (0%)
Primal, Animist, or Folk religion	0 (0%)	0 (0%)
Spiritism	0 (0%)	0 (0%)
Umbanda, Candomblé, and other African-derived religions	0 (0%)	0 (0%)
Chinese folk/traditional religion	0 (0%)	0 (0%)
Islam	1,441 (97.8%)	485 (97.6%)
Hinduism	0 (0%)	0 (0%)
Buddhism	0 (0%)	0 (0%)
Judaism	1 (0.1%)	0 (0%)
Sikhism	0 (0%)	0 (0%)
Baha'i	0 (0%)	0 (0%)
Jainism	0 (0%)	0 (0%)
Shinto	0 (0%)	0 (0%)
Some other religion	0 (0%)	0 (0%)
No religion/Atheist/Agnostic	13 (0.9%)	5 (1.1%)
(Missing)	18 (1.2%)	6 (1.2%)

Note. N (%); this table is based on non-imputed data. Cumulative percentages for variables may not add up to 100% due to rounding. Wave 1 characteristics weighted using the Gallup provided sampling weight, ANNUAL_WEIGHT_R2; Wave 2 characteristics weighted accounting for attrition by using the adjusted Wave 1 weight, ANNUAL_WEIGHT_R2, multiplied by the created attrition weight to account for dropout, to maintain nationally representative estimates for Wave 2 characteristics.

Table S29b. Weighted summary statistics for outcome variables in Turkey

Outcome	Wave 1<br>N = 1,473	Wave 2<br>N = 497
Secure flourishing index		
Mean	6.3	6.1
Standard Deviation	2.0	2.0
Min, Max	0.0, 10.0	0.0, 10.0
(Missing)	31 (2.1%)	25 (5.0%)
Flourishing index		
Mean	6.6	6.4
Standard Deviation	2.0	2.0
Min, Max	0.0, 10.0	0.0, 10.0
(Missing)	28 (1.9%)	24 (4.8%)
Happiness & life satisfaction		
Mean	5.3	5.2
Standard Deviation	2.9	2.7
Min, Max	0.0, 10.0	0.0, 10.0
(Missing)	6 (0.4%)	0 (<0.1%)
Physical & mental health		
Mean	6.5	6.2
Standard Deviation	2.5	2.5
Min, Max	0.0, 10.0	0.0, 10.0
(Missing)	2 (0.1%)	4 (0.9%)
Meaning & purpose		
Mean	6.7	6.2
Standard Deviation	2.6	2.7
Min, Max	0.0, 10.0	0.0, 10.0
(Missing)	13 (0.9%)	11 (2.2%)
Character & virtue		
Mean	7.4	7.2
Standard Deviation	2.2	2.2
Min, Max	0.0, 10.0	0.0, 10.0
(Missing)	0 (<0.1%)	8 (1.7%)
Close social relationships		
Mean	7.0	7.0
Standard Deviation	2.7	2.6
Min, Max	0.0, 10.0	0.0, 10.0
(Missing)	6 (0.4%)	2 (0.4%)
Financial & material security		
Mean	4.9	5.0
Standard Deviation	3.3	3.0
Min, Max	0.0, 10.0	0.0, 10.0
(Missing)	3 (0.2%)	1 (0.1%)
Happiness		
Mean	5.5	5.3
Standard Deviation	3.0	2.8
Min, Max	0.0, 10.0	0.0, 10.0
Life satisfaction		
Mean	5.2	5.2
Standard Deviation	3.3	3.0
Min, Max	0.0, 10.0	0.0, 10.0
(Missing)	6 (0.4%)	0 (<0.1%)
Current life evaluation		
Mean	5.2	5.0

Table S29b. Weighted summary statistics for outcome variables in Turkey

Outcome	Wave 1<br>N = 1,473	Wave 2<br>N = 497
Standard Deviation	2.6	2.4
Min, Max	0.0, 10.0	0.0, 10.0
(Missing)	3 (0.2%)	0 (<0.1%)
Future life evaluation		
Mean	6.2	5.9
Standard Deviation	3.0	3.1
Min, Max	0.0, 10.0	0.0, 10.0
(Missing)	106 (7.2%)	28 (5.7%)
Optimism		
Mean	7.7	7.5
Standard Deviation	2.5	2.5
Min, Max	0.0, 10.0	0.0, 10.0
(Missing)	5 (0.4%)	7 (1.4%)
Freedom to pursue what's important		
Mean	7.3	6.9
Standard Deviation	3.0	3.0
Min, Max	0.0, 10.0	0.0, 10.0
(Missing)	1 (<0.1%)	0 (0%)
Inner peace, n (%)		
Always	353 (24.0%)	97 (19.6%)
Often	360 (24.5%)	140 (28.1%)
Rarely	625 (42.4%)	203 (40.9%)
Never	126 (8.5%)	53 (10.7%)
(Missing)	8 (0.6%)	3 (0.7%)
Life balance, n (%)		
Always	294 (20.0%)	84 (16.8%)
Often	395 (26.8%)	157 (31.6%)
Rarely	652 (44.2%)	208 (41.9%)
Never	119 (8.1%)	42 (8.5%)
(Missing)	14 (0.9%)	6 (1.1%)
Sense of mastery, n (%)		
Always	634 (43.0%)	198 (39.9%)
Often	444 (30.2%)	167 (33.6%)
Rarely	326 (22.1%)	113 (22.7%)
Never	63 (4.3%)	18 (3.7%)
(Missing)	5 (0.4%)	0 (0.0%)
Meaningful activities		
Mean	6.1	5.8
Standard Deviation	3.1	2.9
Min, Max	0.0, 10.0	0.0, 10.0
(Missing)	3 (0.2%)	10 (2.0%)
Understanding purpose		
Mean	7.2	6.6
Standard Deviation	3.0	3.1
Min, Max	0.0, 10.0	0.0, 10.0
(Missing)	10 (0.7%)	1 (0.2%)
Self-rated mental health		
Mean	6.3	5.9
Standard Deviation	3.0	3.0
Min, Max	0.0, 10.0	0.0, 10.0
(Missing)	0 (0%)	2 (0.3%)

Table S29b. Weighted summary statistics for outcome variables in Turkey

Outcome	Wave 1<br>N = 1,473	Wave 2<br>N = 497
Traumatic distress, n (%)		
A lot	397 (27.0%)	151 (30.3%)
Some	374 (25.4%)	109 (22.0%)
Not very much	245 (16.7%)	95 (19.2%)
Not at all	448 (30.4%)	138 (27.8%)
(Missing)	8 (0.6%)	3 (0.7%)
Depression symptoms composite, n (%)	630 (43.1%)	246 (50.0%)
(Missing)	12 (0.8%)	5 (0.9%)
Depression – feel hopeless, n (%)		
Nearly every day	261 (17.7%)	101 (20.4%)
More than half the days	258 (17.5%)	108 (21.7%)
Several days	400 (27.2%)	113 (22.8%)
Not at all	548 (37.2%)	173 (34.8%)
(Missing)	6 (0.4%)	1 (0.3%)
Depression – loss of interest, n (%)		
Nearly every day	292 (19.8%)	114 (22.9%)
More than half the days	243 (16.5%)	94 (19.0%)
Several days	412 (28.0%)	102 (20.5%)
Not at all	519 (35.2%)	183 (36.9%)
(Missing)	6 (0.4%)	3 (0.7%)
Anxiety symptoms composite, n (%)	617 (42.3%)	204 (41.1%)
(Missing)	13 (0.9%)	0 (<0.1%)
Anxiety – feel on edge, n (%)		
Nearly every day	345 (23.4%)	130 (26.2%)
More than half the days	248 (16.9%)	98 (19.7%)
Several days	438 (29.7%)	123 (24.7%)
Not at all	437 (29.6%)	146 (29.3%)
(Missing)	5 (0.4%)	0 (0.1%)
Anxiety – cannot stop worrying, n (%)		
Nearly every day	248 (16.8%)	74 (15.0%)
More than half the days	209 (14.2%)	66 (13.3%)
Several days	333 (22.6%)	105 (21.2%)
Not at all	676 (45.9%)	251 (50.4%)
(Missing)	8 (0.5%)	0 (0.1%)
Suffering, n (%)		
A lot	367 (24.9%)	105 (21.1%)
Some	524 (35.5%)	206 (41.4%)
Not very much	329 (22.3%)	103 (20.7%)
Not at all	246 (16.7%)	82 (16.5%)
(Missing)	8 (0.5%)	1 (0.3%)
Relationship contentment		
Mean	7.3	7.3
Standard Deviation	2.9	2.8
Min, Max	0.0, 10.0	0.0, 10.0
Relationship satisfaction		
Mean	6.7	6.7
Standard Deviation	2.9	2.8
Min, Max	0.0, 10.0	0.0, 10.0
(Missing)	6 (0.4%)	2 (0.3%)
Social support		
Mean	5.5	5.4

Table S29b. Weighted summary statistics for outcome variables in Turkey

Outcome	Wave 1<br>N = 1,473	Wave 2<br>N = 497
Standard Deviation	3.5	3.4
Min, Max	0.0, 10.0	0.0, 10.0
(Missing)	1 (<0.1%)	1 (0.2%)
Intimate/close friend, n (%)		
Yes	1,144 (77.6%)	367 (73.9%)
No	321 (21.8%)	126 (25.4%)
(Missing)	9 (0.6%)	3 (0.7%)
Government approval, n (%)		
Strongly approve	282 (19.2%)	67 (13.6%)
Somewhat approve	320 (21.7%)	114 (23.0%)
Neither approve nor disapprove	191 (13.0%)	68 (13.6%)
Somewhat disapprove	158 (10.7%)	71 (14.2%)
Strongly disapprove	487 (33.1%)	172 (34.6%)
(Missing)	35 (2.4%)	5 (1.0%)
Say in government, n (%)		
Agree	427 (29.0%)	107 (21.6%)
Disagree	672 (45.6%)	303 (61.1%)
Unsure	365 (24.8%)	80 (16.1%)
(Missing)	10 (0.7%)	6 (1.2%)
Belonging in country		
Mean	6.9	7.4
Standard Deviation	3.4	3.2
Min, Max	0.0, 10.0	0.0, 10.0
(Missing)	14 (0.9%)	8 (1.7%)
City/place satisfaction, n (%)		
Satisfied	943 (64.0%)	286 (57.5%)
Dissatisfied	321 (21.8%)	125 (25.2%)
Unsure	203 (13.8%)	83 (16.7%)
(Missing)	5 (0.4%)	3 (0.6%)
Trust within country, n (%)		
All people	66 (4.5%)	15 (3.0%)
Most people	150 (10.2%)	24 (4.9%)
Some people	316 (21.5%)	106 (21.4%)
Not very many people	509 (34.6%)	177 (35.7%)
None	412 (28.0%)	169 (34.0%)
(Missing)	20 (1.3%)	5 (1.0%)
Number of children		
Mean	1.0	1.2
Standard Deviation	1.3	1.6
Min, Max	0.0, 8.0	0.0, 10.0
(Missing)	2 (0.1%)	43 (8.6%)
Community participation, n (%)		
More than once a week	167 (11.3%)	63 (12.7%)
Once a week	138 (9.4%)	35 (7.0%)
One to three times a month	209 (14.2%)	70 (14.1%)
A few times a year	321 (21.8%)	128 (25.7%)
Never	629 (42.7%)	200 (40.3%)
(Missing)	9 (0.6%)	2 (0.3%)
Religious attendance, n (%)		
More than once a week	499 (33.9%)	212 (42.7%)
Once a week	274 (18.6%)	67 (13.6%)

Table S29b. Weighted summary statistics for outcome variables in Turkey

Outcome	Wave 1<br>N = 1,473	Wave 2<br>N = 497
One to three times a month	175 (11.9%)	52 (10.4%)
A few times a year	252 (17.1%)	78 (15.6%)
Never	268 (18.2%)	81 (16.2%)
(Missing)	5 (0.4%)	7 (1.5%)
Loneliness		
Mean	4.6	4.6
Standard Deviation	3.5	3.2
Min, Max	0.0, 10.0	0.0, 10.0
(Missing)	8 (0.5%)	3 (0.5%)
Perceived discrimination, n (%)		
Always	202 (13.7%)	75 (15.2%)
Often	219 (14.8%)	70 (14.2%)
Rarely	636 (43.2%)	181 (36.4%)
Never	407 (27.6%)	167 (33.7%)
(Missing)	10 (0.6%)	3 (0.6%)
Orientation to promote good		
Mean	7.7	7.5
Standard Deviation	2.4	2.5
Min, Max	0.0, 10.0	0.0, 10.0
(Missing)	0 (0%)	2 (0.3%)
Delayed gratification		
Mean	7.2	6.9
Standard Deviation	2.7	2.7
Min, Max	0.0, 10.0	0.0, 10.0
(Missing)	0 (<0.1%)	7 (1.3%)
Hope		
Mean	7.8	7.4
Standard Deviation	2.7	2.8
Min, Max	0.0, 10.0	0.0, 10.0
(Missing)	10 (0.7%)	7 (1.3%)
Gratitude		
Mean	7.0	6.8
Standard Deviation	2.7	2.4
Min, Max	0.0, 10.0	0.0, 10.0
(Missing)	66 (4.5%)	6 (1.2%)
Showing love/care		
Mean	7.9	7.7
Standard Deviation	2.6	2.6
Min, Max	0.0, 10.0	0.0, 10.0
(Missing)	0 (0%)	2 (0.4%)
Forgivingness, n (%)		
Always	318 (21.6%)	112 (22.5%)
Often	283 (19.2%)	86 (17.2%)
Rarely	509 (34.6%)	183 (36.8%)
Never	350 (23.8%)	117 (23.5%)
(Missing)	12 (0.8%)	0 (0%)
Charitable giving, n (%)		
Yes	450 (30.6%)	225 (45.3%)
No	1,018 (69.1%)	267 (53.7%)
(Missing)	4 (0.3%)	5 (1.0%)
Helping strangers, n (%)		

Table S29b. Weighted summary statistics for outcome variables in Turkey

Outcome	Wave 1<br>N = 1,473	Wave 2<br>N = 497
Yes	876 (59.5%)	367 (73.9%)
No	577 (39.2%)	127 (25.5%)
(Missing)	19 (1.3%)	3 (0.6%)
Volunteering, n (%)		
Yes	212 (14.4%)	95 (19.2%)
No	1,261 (85.6%)	399 (80.2%)
(Missing)	0 (0%)	3 (0.6%)
Self-rated physical health		
Mean	6.7	6.4
Standard Deviation	2.8	2.6
Min, Max	0.0, 10.0	0.0, 10.0
(Missing)	2 (0.1%)	3 (0.5%)
Health problems, n (%)		
Yes	204 (13.9%)	94 (18.9%)
No	1,251 (84.9%)	401 (80.8%)
(Missing)	18 (1.2%)	2 (0.3%)
Pain in past 4 weeks, n (%)		
A lot	361 (24.5%)	104 (21.0%)
Some	422 (28.7%)	137 (27.6%)
Not very much	317 (21.5%)	113 (22.8%)
None at all	368 (25.0%)	140 (28.2%)
(Missing)	5 (0.4%)	2 (0.4%)
Number of cigarettes per day		
Mean	9.6	9.6
Standard Deviation	13.2	13.5
Min, Max	0.0, 97.0	0.0, 80.0
(Missing)	9 (0.6%)	17 (3.4%)
Number of drinks per week		
Mean	1.1	1.0
Standard Deviation	5.2	5.1
Min, Max	0.0, 80.0	0.0, 63.0
(Missing)	21 (1.4%)	26 (5.2%)
Days exercise per week		
Mean	2.8	2.5
Standard Deviation	2.8	2.8
Min, Max	0.0, 7.0	0.0, 7.0
(Missing)	18 (1.2%)	1 (0.2%)
Financial security		
Mean	4.6	4.7
Standard Deviation	3.5	3.2
Min, Max	0.0, 10.0	0.0, 10.0
(Missing)	2 (0.1%)	1 (0.1%)
Material security		
Mean	5.3	5.2
Standard Deviation	3.6	3.3
Min, Max	0.0, 10.0	0.0, 10.0
(Missing)	1 (0.1%)	0 (0%)
Educational attainment (16+ years), n (%)		
Up to 8	452 (30.7%)	134 (27.0%)
9-15	730 (49.6%)	229 (46.2%)
16+	290 (19.7%)	133 (26.8%)

Table S29b. Weighted summary statistics for outcome variables in Turkey

Outcome	Wave 1<br>N = 1,473	Wave 2<br>N = 497
(Missing)	0 (0%)	0 (0%)
Currently employed, n (%)		
Employed for an employer	403 (27.3%)	146 (29.3%)
Self-employed	248 (16.8%)	76 (15.3%)
Retired	206 (14.0%)	69 (13.9%)
Student	112 (7.6%)	23 (4.5%)
Homemaker	361 (24.5%)	108 (21.7%)
Unemployed and looking for a job	86 (5.8%)	43 (8.7%)
None of these/Other	58 (3.9%)	26 (5.3%)
(Missing)	0 (0%)	6 (1.3%)
Financially comfortable/getting by, n (%)		
Living comfortably on present income	189 (12.9%)	50 (10.1%)
Getting by on present income	561 (38.1%)	204 (41.1%)
Finding it difficult on present income	371 (25.2%)	140 (28.3%)
Finding it very difficult on present income	333 (22.6%)	97 (19.5%)
(Missing)	19 (1.3%)	5 (0.9%)
Own home, n (%)		
Someone in this household owns this home	764 (51.8%)	254 (51.1%)
Someone in this household rents this home	442 (30.0%)	149 (30.0%)
Both	60 (4.1%)	32 (6.4%)
Neither	183 (12.4%)	56 (11.3%)
Rent	0 (0%)	0 (0%)
Own	0 (0%)	0 (0%)
Something else	0 (0%)	0 (0%)
(Missing)	24 (1.6%)	6 (1.1%)
Religious/spiritual connection, n (%)		
Always	719 (48.8%)	254 (51.1%)
Often	222 (15.0%)	77 (15.6%)
Rarely	317 (21.5%)	101 (20.3%)
Never	184 (12.5%)	59 (11.9%)
(Missing)	31 (2.1%)	6 (1.1%)
Belief in life after death, n (%)		
Yes	985 (66.9%)	315 (63.4%)
No	223 (15.2%)	85 (17.2%)
Unsure	261 (17.7%)	95 (19.2%)
(Missing)	4 (0.2%)	1 (0.3%)
Transformative religious experience, n (%)		
Yes	424 (28.8%)	129 (25.9%)
No	1,027 (69.7%)	361 (72.7%)
(Missing)	22 (1.5%)	7 (1.4%)
Religious reading or listening, n (%)		
More than once a day	284 (19.3%)	110 (22.1%)
About once a day	289 (19.6%)	83 (16.6%)
Sometimes	685 (46.5%)	227 (45.7%)
Never	209 (14.2%)	76 (15.4%)
(Missing)	6 (0.4%)	1 (0.2%)
Prayer or meditation, n (%)		
More than once a day	772 (52.4%)	272 (54.7%)
About once a day	252 (17.1%)	86 (17.4%)
Sometimes	311 (21.1%)	100 (20.1%)
Never	134 (9.1%)	38 (7.6%)

Table S29b. Weighted summary statistics for outcome variables in Turkey

**Outcome**	**Wave 1**<br>N = 1,473	**Wave 2**<br>N = 497
(Missing)	4 (0.3%)	1 (0.3%)
Belief in God/gods/spiritual forces, n (%)		
One God	1,217 (82.6%)	418 (84.2%)
More than one god	14 (1.0%)	6 (1.2%)
An impersonal spiritual force	22 (1.5%)	15 (3.0%)
None of these	138 (9.4%)	37 (7.5%)
Unsure	74 (5.0%)	19 (3.8%)
(Missing)	8 (0.6%)	2 (0.4%)
Religious centrality, n (%)		
Agree	1,057 (71.7%)	346 (69.6%)
Disagree	202 (13.7%)	90 (18.2%)
Not relevant	93 (6.3%)	29 (5.9%)
Unsure	115 (7.8%)	27 (5.4%)
(Missing)	6 (0.4%)	5 (0.9%)
Religious/spiritual comfort, n (%)		
Agree	1,135 (77.1%)	398 (80.1%)
Disagree	148 (10.1%)	48 (9.7%)
Not relevant	87 (5.9%)	24 (4.8%)
Unsure	97 (6.6%)	26 (5.3%)
(Missing)	5 (0.4%)	1 (0.1%)
Feel loved by God, n (%)		
Agree	1,120 (76.1%)	396 (79.8%)
Disagree	116 (7.9%)	45 (9.0%)
Not relevant	82 (5.6%)	22 (4.4%)
Unsure	144 (9.8%)	30 (6.1%)
(Missing)	10 (0.7%)	3 (0.7%)
Feel punished by God, n (%)		
Agree	446 (30.3%)	144 (29.0%)
Disagree	692 (47.0%)	264 (53.2%)
Not relevant	124 (8.4%)	43 (8.7%)
Unsure	197 (13.4%)	41 (8.2%)
(Missing)	14 (1.0%)	5 (0.9%)
Experienced religious criticism, n (%)		
Agree	544 (37.0%)	202 (40.6%)
Disagree	629 (42.7%)	208 (41.8%)
Not relevant	116 (7.9%)	49 (9.9%)
Unsure	178 (12.1%)	35 (7.0%)
(Missing)	6 (0.4%)	3 (0.7%)
Faith-sharing, n (%)		
Agree	881 (59.8%)	304 (61.2%)
Disagree	348 (23.6%)	120 (24.2%)
Not relevant	107 (7.2%)	33 (6.7%)
Unsure	127 (8.6%)	37 (7.4%)
(Missing)	10 (0.7%)	3 (0.6%)

Note. N (%); this table is based on non-imputed data. Cumulative percentages for variables may not add up to 100% due to rounding. Wave 1 characteristics weighted using the Gallup provided sampling weight, ANNUAL_WEIGHT_R2; Wave 2 characteristics weighted accounting for attrition by using the adjusted Wave 1 weight, ANNUAL_WEIGHT_R2, multiplied by the created attrition weight to account for dropout, to maintain nationally representative estimates for Wave 2 characteristics.

Table S29c. Unweighted summary statistics for demographic and childhood variables in Turkey by retention status

**Characteristic**	**Attritors–Not Observed in Wave 2** N = 836	**Retained–Observed in Wave 2** N = 525
Forgivingness, n (%)		
Always	189 (22.6%)	103 (19.7%)
Often	148 (17.7%)	117 (22.3%)
Rarely	296 (35.4%)	173 (32.9%)
Never	195 (23.4%)	129 (24.6%)
(Missing)	8 (1.0%)	3 (0.6%)
Year of birth, n (%)		
1943 or earlier (current age: 80+ years)	11 (1.3%)	9 (1.6%)
1943-1953 (current age: 70-79 years)	45 (5.4%)	21 (4.0%)
1953-1963 (current age: 60-69 years)	86 (10.3%)	74 (14.1%)
1963-1973 (current age: 50-59 years)	100 (11.9%)	100 (19.0%)
1973-1983 (current age: 40-49 years)	164 (19.6%)	109 (20.7%)
1983-1993 (current age: 30-39 years)	179 (21.4%)	106 (20.2%)
1993-1998 (current age: 25-29 years)	87 (10.4%)	57 (10.8%)
1998-2005 (current age: 18-24 years)	165 (19.8%)	50 (9.5%)
(Missing)	0 (0%)	0 (0%)
Age of participant		
Mean	40.8	44.8
Standard Deviation	16.6	15.7
Min, Max	18.0, 93.0	18.0, 82.0
Gender, n (%)		
Male	403 (48.3%)	280 (53.4%)
Female	433 (51.7%)	245 (46.6%)
Other	0 (0%)	0 (0%)
(Missing)	0 (0%)	0 (0%)
Respondent marital status, n (%)		
Single/Never been married	238 (28.4%)	103 (19.5%)
Married	503 (60.2%)	373 (71.1%)
Separated	7 (0.9%)	4 (0.7%)
Divorced	32 (3.8%)	24 (4.6%)
Widowed	43 (5.2%)	20 (3.8%)
Domestic partner	0 (0%)	0 (0%)
(Missing)	13 (1.5%)	2 (0.4%)
Education (years), n (%)		
Up to 8	252 (30.2%)	167 (31.8%)
9-15	425 (50.8%)	248 (47.2%)
16+	159 (19.0%)	111 (21.1%)
(Missing)	0 (0%)	0 (0%)
Employment status, n (%)		
Employed for an employer	215 (25.8%)	160 (30.5%)
Self-employed	146 (17.5%)	81 (15.5%)
Retired	110 (13.1%)	82 (15.6%)
Student	76 (9.1%)	25 (4.8%)
Homemaker	215 (25.7%)	117 (22.3%)
Unemployed and looking for a job	49 (5.8%)	31 (5.9%)
None of these/Other	26 (3.1%)	29 (5.6%)
(Missing)	0 (0%)	0 (0%)
Current religious service attendance, n (%)		
More than once a week	287 (34.3%)	174 (33.1%)

Table S29c. Unweighted summary statistics for demographic and childhood variables in Turkey by retention status

Characteristic	Attritors–Not Observed in Wave 2<br>N = 836	Retained–Observed in Wave 2<br>N = 525
Once a week	159 (19.1%)	93 (17.7%)
One to three times a month	106 (12.7%)	54 (10.2%)
A few times a year	145 (17.4%)	87 (16.6%)
Never	136 (16.2%)	116 (22.0%)
(Missing)	3 (0.3%)	2 (0.4%)
Immigration status, n (%)		
Born in this country	799 (95.6%)	507 (96.6%)
Born in another country	37 (4.4%)	18 (3.4%)
(Missing)	0 (0%)	0 (0%)
Parental marital status around age 12, n (%)		
Parents were married	747 (89.4%)	477 (90.9%)
Parents were divorced	37 (4.4%)	16 (3.1%)
Parents were never married	6 (0.7%)	0 (0.1%)
One or both of them had died	30 (3.6%)	27 (5.1%)
Unsure	1 (0.2%)	3 (0.5%)
(Missing)	14 (1.7%)	2 (0.3%)
Religious service attendance around age 12, n (%)		
At least once a week	341 (40.8%)	223 (42.4%)
One to three times a month	140 (16.7%)	77 (14.8%)
Less than once a month	130 (15.6%)	73 (13.8%)
Never	216 (25.9%)	145 (27.6%)
(Missing)	9 (1.1%)	7 (1.4%)
Relationship with mother when growing up, n (%)		
Very good	555 (66.4%)	341 (65.0%)
Somewhat good	221 (26.5%)	147 (28.1%)
Somewhat bad	28 (3.4%)	15 (2.9%)
Very bad	14 (1.7%)	11 (2.2%)
(Does not apply)	12 (1.4%)	8 (1.5%)
(Missing)	5 (0.6%)	2 (0.5%)
Relationship with father when growing up, n (%)		
Very good	466 (55.8%)	272 (51.7%)
Somewhat good	236 (28.2%)	154 (29.3%)
Somewhat bad	42 (5.0%)	26 (5.0%)
Very bad	52 (6.2%)	37 (7.1%)
(Does not apply)	32 (3.9%)	22 (4.1%)
(Missing)	8 (1.0%)	15 (2.8%)
Felt like an outsider in family when growing up, n (%)		
Yes	94 (11.3%)	50 (9.5%)
No	737 (88.2%)	471 (89.7%)
(Missing)	5 (0.5%)	4 (0.8%)
Experienced abuse when growing up, n (%)		
Yes	94 (11.2%)	52 (9.9%)
No	723 (86.5%)	470 (89.5%)
(Missing)	19 (2.2%)	3 (0.6%)
Self-rated health when growing up, n (%)		
Excellent	214 (25.6%)	126 (24.0%)
Very good	235 (28.2%)	145 (27.6%)

Table S29c. Unweighted summary statistics for demographic and childhood variables in Turkey by retention status

**Characteristic**	**Attritors–Not Observed in Wave 2** N = 836	**Retained–Observed in Wave 2** N = 525
Good	237 (28.4%)	155 (29.5%)
Fair	122 (14.6%)	82 (15.6%)
Poor	27 (3.3%)	17 (3.3%)
(Missing)	0 (0.1%)	0 (0%)
Subjective financial status of family growing up, n (%)		
Lived comfortably	286 (34.2%)	172 (32.7%)
Got by	354 (42.4%)	247 (47.1%)
Found it difficult	134 (16.0%)	66 (12.6%)
Found it very difficult	61 (7.3%)	40 (7.6%)
(Missing)	1 (0.2%)	0 (0%)
Religious affiliation growing up, n (%)		
Christianity	0 (0%)	1 (0.2%)
Taoism	0 (0%)	0 (0%)
Confucianism	0 (0%)	0 (0%)
Primal, Animist, or Folk religion	0 (0%)	0 (0%)
Spiritism	0 (0%)	0 (0%)
Umbanda, Candomblé, and other African-derived religions	0 (0%)	0 (0%)
Chinese folk/traditional religion	0 (0%)	0 (0%)
Islam	818 (97.9%)	513 (97.7%)
Hinduism	0 (0%)	0 (0%)
Buddhism	0 (0%)	0 (0%)
Judaism	1 (0.1%)	0 (0%)
Sikhism	0 (0%)	0 (0%)
Baha'i	0 (0%)	0 (0%)
Jainism	0 (0%)	0 (0%)
Shinto	0 (0%)	0 (0%)
Some other religion	0 (0%)	0 (0%)
No religion/Atheist/Agnostic	7 (0.9%)	5 (0.9%)
(Missing)	10 (1.2%)	7 (1.3%)

Note. N (%); this table is based on non-imputed data. Cumulative percentages for variables may not add up to 100% due to rounding.

Table S29d. Unweighted summary statistics for Wave 1 outcome variables in Turkey by retention status.

**Outcome**	**Attritors–Not Observed in Wave 2**<br>N = 836	**Retained–Observed in Wave 2**<br>N = 525
Secure flourishing index		
Mean	6.3	6.4
Standard Deviation	1.9	2.1
Min, Max	0.0, 10.0	0.3, 10.0
(Missing)	15 (1.8%)	14 (2.6%)
Flourishing index		
Mean	6.5	6.7
Standard Deviation	1.9	2.0
Min, Max	0.0, 10.0	0.4, 10.0
(Missing)	13 (1.6%)	13 (2.4%)
Happiness & life satisfaction		
Mean	5.3	5.4
Standard Deviation	2.9	3.0
Min, Max	0.0, 10.0	0.0, 10.0
(Missing)	2 (0.3%)	4 (0.7%)
Physical & mental health		
Mean	6.5	6.5
Standard Deviation	2.5	2.5
Min, Max	0.0, 10.0	0.0, 10.0
(Missing)	1 (0.2%)	0 (0%)
Meaning & purpose		
Mean	6.7	6.7
Standard Deviation	2.6	2.7
Min, Max	0.0, 10.0	0.0, 10.0
(Missing)	5 (0.6%)	8 (1.5%)
Character & virtue		
Mean	7.4	7.4
Standard Deviation	2.1	2.2
Min, Max	0.0, 10.0	0.0, 10.0
Close social relationships		
Mean	6.9	7.2
Standard Deviation	2.7	2.6
Min, Max	0.0, 10.0	0.0, 10.0
(Missing)	4 (0.5%)	1 (0.3%)
Financial & material security		
Mean	4.9	5.0
Standard Deviation	3.3	3.4
Min, Max	0.0, 10.0	0.0, 10.0
(Missing)	2 (0.2%)	1 (0.2%)
Happiness		
Mean	5.4	5.7
Standard Deviation	3.0	2.9
Min, Max	0.0, 10.0	0.0, 10.0
Life satisfaction		
Mean	5.2	5.2
Standard Deviation	3.3	3.3
Min, Max	0.0, 10.0	0.0, 10.0
(Missing)	2 (0.3%)	3 (0.6%)
Current life evaluation		

Table S29d. Unweighted summary statistics for Wave 1 outcome variables in Turkey by retention status.

Outcome	Attritors–Not Observed in Wave 2 N = 836	Retained–Observed in Wave 2 N = 525
Mean	5.2	5.2
Standard Deviation	2.7	2.5
Min, Max	0.0, 10.0	0.0, 10.0
(Missing)	0 (<0.1%)	3 (0.5%)
Future life evaluation		
Mean	6.4	5.9
Standard Deviation	2.9	3.1
Min, Max	0.0, 10.0	0.0, 10.0
(Missing)	64 (7.6%)	33 (6.4%)
Optimism		
Mean	7.6	7.7
Standard Deviation	2.4	2.6
Min, Max	0.0, 10.0	0.0, 10.0
(Missing)	2 (0.2%)	4 (0.7%)
Freedom to pursue what's important		
Mean	7.2	7.4
Standard Deviation	3.0	3.0
Min, Max	0.0, 10.0	0.0, 10.0
(Missing)	0 (0%)	1 (0.3%)
Inner peace, n (%)		
Always	204 (24.4%)	122 (23.2%)
Often	200 (23.9%)	134 (25.6%)
Rarely	361 (43.2%)	215 (41.0%)
Never	66 (7.9%)	51 (9.7%)
(Missing)	5 (0.6%)	3 (0.6%)
Life balance, n (%)		
Always	161 (19.3%)	112 (21.3%)
Often	225 (26.9%)	140 (26.6%)
Rarely	371 (44.4%)	230 (43.8%)
Never	69 (8.2%)	41 (7.7%)
(Missing)	10 (1.1%)	3 (0.5%)
Sense of mastery, n (%)		
Always	356 (42.6%)	230 (43.9%)
Often	251 (30.0%)	160 (30.5%)
Rarely	187 (22.4%)	113 (21.6%)
Never	38 (4.6%)	20 (3.7%)
(Missing)	3 (0.4%)	2 (0.3%)
Meaningful activities		
Mean	6.1	6.2
Standard Deviation	3.1	3.1
Min, Max	0.0, 10.0	0.0, 10.0
(Missing)	2 (0.3%)	1 (0.1%)
Understanding purpose		
Mean	7.2	7.1
Standard Deviation	2.9	3.1
Min, Max	0.0, 10.0	0.0, 10.0
(Missing)	2 (0.3%)	7 (1.3%)
Self-rated mental health		
Mean	6.2	6.3

Table S29d. Unweighted summary statistics for Wave 1 outcome variables in Turkey by retention status.

Outcome	Attritors–Not Observed in Wave 2<br>N = 836	Retained–Observed in Wave 2<br>N = 525
Standard Deviation	3.0	3.0
Min, Max	0.0, 10.0	0.0, 10.0
Traumatic distress, n (%)		
A lot	227 (27.1%)	140 (26.6%)
Some	226 (27.1%)	116 (22.1%)
Not very much	134 (16.1%)	93 (17.8%)
Not at all	246 (29.4%)	171 (32.5%)
(Missing)	3 (0.3%)	5 (1.0%)
Depression symptoms composite, n (%)	376 (45.4%)	202 (38.6%)
(Missing)	8 (0.9%)	3 (0.6%)
Depression – feel hopeless, n (%)		
Nearly every day	157 (18.7%)	83 (15.8%)
More than half the days	153 (18.3%)	84 (16.0%)
Several days	232 (27.8%)	137 (26.1%)
Not at all	290 (34.7%)	220 (42.0%)
(Missing)	4 (0.5%)	1 (0.2%)
Depression – loss of interest, n (%)		
Nearly every day	177 (21.2%)	90 (17.2%)
More than half the days	144 (17.3%)	79 (15.1%)
Several days	228 (27.3%)	153 (29.2%)
Not at all	282 (33.8%)	200 (38.1%)
(Missing)	4 (0.4%)	2 (0.4%)
Anxiety symptoms composite, n (%)	351 (42.5%)	219 (41.9%)
(Missing)	9 (1.1%)	2 (0.4%)
Anxiety – feel on edge, n (%)		
Nearly every day	198 (23.7%)	120 (22.8%)
More than half the days	148 (17.7%)	80 (15.2%)
Several days	251 (30.1%)	153 (29.1%)
Not at all	234 (28.0%)	173 (32.9%)
(Missing)	5 (0.5%)	0 (0%)
Anxiety – cannot stop worrying, n (%)		
Nearly every day	138 (16.5%)	92 (17.5%)
More than half the days	118 (14.2%)	75 (14.3%)
Several days	199 (23.8%)	106 (20.1%)
Not at all	376 (44.9%)	251 (47.7%)
(Missing)	5 (0.6%)	2 (0.4%)
Suffering, n (%)		
A lot	211 (25.2%)	128 (24.4%)
Some	301 (36.0%)	182 (34.7%)
Not very much	189 (22.6%)	114 (21.7%)
Not at all	130 (15.5%)	100 (19.0%)
(Missing)	5 (0.7%)	2 (0.3%)
Relationship contentment		
Mean	7.1	7.5
Standard Deviation	3.0	2.8
Min, Max	0.0, 10.0	0.0, 10.0
Relationship satisfaction		
Mean	6.6	7.0
Standard Deviation	3.0	2.8

Table S29d. Unweighted summary statistics for Wave 1 outcome variables in Turkey by retention status.

**Outcome**	**Attritors–Not Observed in Wave 2** N = 836	**Retained–Observed in Wave 2** N = 525
Min, Max	0.0, 10.0	0.0, 10.0
(Missing)	4 (0.5%)	1 (0.1%)
Social support		
Mean	5.5	5.4
Standard Deviation	3.5	3.5
Min, Max	0.0, 10.0	0.0, 10.0
Intimate/close friend, n (%)		
Yes	644 (77.0%)	414 (78.9%)
No	185 (22.1%)	111 (21.1%)
(Missing)	7 (0.9%)	0 (0%)
Government approval, n (%)		
Strongly approve	165 (19.8%)	95 (18.1%)
Somewhat approve	185 (22.2%)	110 (20.9%)
Neither approve nor disapprove	129 (15.5%)	43 (8.1%)
Somewhat disapprove	92 (11.0%)	53 (10.1%)
Strongly disapprove	247 (29.5%)	210 (39.9%)
(Missing)	17 (2.1%)	15 (2.9%)
Say in government, n (%)		
Agree	262 (31.3%)	128 (24.4%)
Disagree	354 (42.3%)	273 (52.0%)
Unsure	213 (25.5%)	122 (23.3%)
(Missing)	7 (0.9%)	1 (0.2%)
Belonging in country		
Mean	6.8	7.0
Standard Deviation	3.4	3.3
Min, Max	0.0, 10.0	0.0, 10.0
(Missing)	5 (0.7%)	8 (1.5%)
City/place satisfaction, n (%)		
Satisfied	520 (62.2%)	354 (67.5%)
Dissatisfied	198 (23.6%)	96 (18.3%)
Unsure	114 (13.7%)	74 (14.1%)
(Missing)	4 (0.4%)	1 (0.2%)
Trust within country, n (%)		
All people	43 (5.1%)	17 (3.2%)
Most people	81 (9.7%)	58 (11.1%)
Some people	190 (22.8%)	99 (18.9%)
Not very many people	264 (31.6%)	212 (40.4%)
None	246 (29.4%)	132 (25.2%)
(Missing)	12 (1.4%)	6 (1.2%)
Number of children		
Mean	1.1	1.0
Standard Deviation	1.4	1.3
Min, Max	0.0, 8.0	0.0, 8.0
(Missing)	0 (<0.1%)	1 (0.2%)
Community participation, n (%)		
More than once a week	93 (11.1%)	61 (11.7%)
Once a week	87 (10.4%)	38 (7.3%)
One to three times a month	116 (13.9%)	77 (14.7%)
A few times a year	172 (20.6%)	127 (24.2%)

Table S29d. Unweighted summary statistics for Wave 1 outcome variables in Turkey by retention status.

Outcome	Attritors–Not Observed in Wave 2<br>N = 836	Retained–Observed in Wave 2<br>N = 525
Never	361 (43.2%)	219 (41.8%)
(Missing)	6 (0.7%)	2 (0.3%)
Religious attendance, n (%)		
More than once a week	287 (34.3%)	174 (33.1%)
Once a week	159 (19.1%)	93 (17.7%)
One to three times a month	106 (12.7%)	54 (10.2%)
A few times a year	145 (17.4%)	87 (16.6%)
Never	136 (16.2%)	116 (22.0%)
(Missing)	3 (0.3%)	2 (0.4%)
Loneliness		
Mean	4.7	4.4
Standard Deviation	3.5	3.5
Min, Max	0.0, 10.0	0.0, 10.0
(Missing)	1 (0.1%)	7 (1.3%)
Perceived discrimination, n (%)		
Always	109 (13.0%)	79 (15.0%)
Often	121 (14.5%)	82 (15.6%)
Rarely	355 (42.5%)	234 (44.5%)
Never	245 (29.4%)	127 (24.3%)
(Missing)	6 (0.7%)	3 (0.6%)
Orientation to promote good		
Mean	7.6	7.7
Standard Deviation	2.4	2.3
Min, Max	0.0, 10.0	0.0, 10.0
Delayed gratification		
Mean	7.2	7.1
Standard Deviation	2.5	2.9
Min, Max	0.0, 10.0	0.0, 10.0
Hope		
Mean	7.8	7.7
Standard Deviation	2.6	2.8
Min, Max	0.0, 10.0	0.0, 10.0
(Missing)	5 (0.6%)	5 (0.9%)
Gratitude		
Mean	7.0	6.9
Standard Deviation	2.7	2.7
Min, Max	0.0, 10.0	0.0, 10.0
(Missing)	34 (4.1%)	27 (5.2%)
Showing love/care		
Mean	7.9	7.9
Standard Deviation	2.6	2.6
Min, Max	0.0, 10.0	0.0, 10.0
Forgivingness, n (%)		
Always	189 (22.6%)	103 (19.7%)
Often	148 (17.7%)	117 (22.3%)
Rarely	296 (35.4%)	173 (32.9%)
Never	195 (23.4%)	129 (24.6%)
(Missing)	8 (1.0%)	3 (0.6%)
Charitable giving, n (%)		

Table S29d. Unweighted summary statistics for Wave 1 outcome variables in Turkey by retention status.

**Outcome**	**Attritors–Not Observed in Wave 2** N = 836	**Retained–Observed in Wave 2** N = 525
Yes	258 (30.9%)	157 (29.9%)
No	575 (68.8%)	366 (69.8%)
(Missing)	2 (0.3%)	2 (0.3%)
Helping strangers, n (%)		
Yes	492 (58.9%)	318 (60.7%)
No	331 (39.7%)	201 (38.3%)
(Missing)	12 (1.5%)	5 (1.0%)
Volunteering, n (%)		
Yes	108 (12.9%)	91 (17.3%)
No	728 (87.1%)	434 (82.7%)
(Missing)	0 (0%)	0 (0%)
Self-rated physical health		
Mean	6.7	6.7
Standard Deviation	2.7	2.8
Min, Max	0.0, 10.0	0.0, 10.0
(Missing)	1 (0.2%)	0 (0%)
Health problems, n (%)		
Yes	111 (13.2%)	79 (15.1%)
No	712 (85.2%)	443 (84.4%)
(Missing)	13 (1.5%)	3 (0.5%)
Pain in past 4 weeks, n (%)		
A lot	216 (25.8%)	115 (22.0%)
Some	243 (29.1%)	146 (27.9%)
Not very much	174 (20.8%)	120 (22.9%)
None at all	199 (23.9%)	143 (27.2%)
(Missing)	4 (0.5%)	1 (0.1%)
Number of cigarettes per day		
Mean	9.9	9.1
Standard Deviation	13.2	13.2
Min, Max	0.0, 97.0	0.0, 96.0
(Missing)	6 (0.7%)	2 (0.4%)
Number of drinks per week		
Mean	1.3	0.9
Standard Deviation	5.6	4.3
Min, Max	0.0, 80.0	0.0, 50.0
(Missing)	7 (0.8%)	14 (2.7%)
Days exercise per week		
Mean	2.7	3.0
Standard Deviation	2.7	2.9
Min, Max	0.0, 7.0	0.0, 7.0
(Missing)	9 (1.1%)	8 (1.5%)
Financial security		
Mean	4.6	4.7
Standard Deviation	3.5	3.6
Min, Max	0.0, 10.0	0.0, 10.0
(Missing)	1 (0.2%)	0 (0%)
Material security		
Mean	5.2	5.3
Standard Deviation	3.6	3.6

Table S29d. Unweighted summary statistics for Wave 1 outcome variables in Turkey by retention status.

**Outcome**	**Attritors–Not Observed in Wave 2** N = 836	**Retained–Observed in Wave 2** N = 525
Min, Max	0.0, 10.0	0.0, 10.0
Educational attainment (16+ years), n (%)		
Up to 8	252 (30.2%)	167 (31.8%)
9-15	425 (50.8%)	248 (47.2%)
16+	159 (19.0%)	111 (21.1%)
(Missing)	0 (0%)	0 (0%)
Currently employed, n (%)		
Employed for an employer	215 (25.8%)	160 (30.5%)
Self-employed	146 (17.5%)	81 (15.5%)
Retired	110 (13.1%)	82 (15.6%)
Student	76 (9.1%)	25 (4.8%)
Homemaker	215 (25.7%)	117 (22.3%)
Unemployed and looking for a job	49 (5.8%)	31 (5.9%)
None of these/Other	26 (3.1%)	29 (5.6%)
(Missing)	0 (0%)	0 (0%)
Financially comfortable/getting by, n (%)		
Living comfortably on present income	108 (12.9%)	67 (12.8%)
Getting by on present income	310 (37.1%)	210 (39.9%)
Finding it difficult on present income	223 (26.7%)	116 (22.1%)
Finding it very difficult on present income	180 (21.6%)	129 (24.7%)
(Missing)	14 (1.7%)	2 (0.5%)
Own home, n (%)		
Someone in this household owns this home	407 (48.7%)	305 (58.0%)
Someone in this household rents this home	267 (31.9%)	138 (26.3%)
Both	37 (4.4%)	19 (3.6%)
Neither	106 (12.7%)	63 (11.9%)
Rent	0 (0%)	0 (0%)
Own	0 (0%)	0 (0%)
Something else	0 (0%)	0 (0%)
(Missing)	20 (2.4%)	1 (0.2%)
Religious/spiritual connection, n (%)		
Always	409 (48.9%)	255 (48.6%)
Often	139 (16.7%)	63 (11.9%)
Rarely	188 (22.4%)	103 (19.7%)
Never	80 (9.6%)	95 (18.1%)
(Missing)	20 (2.4%)	9 (1.7%)
Belief in life after death, n (%)		
Yes	570 (68.2%)	337 (64.2%)
No	109 (13.1%)	101 (19.3%)
Unsure	155 (18.5%)	85 (16.2%)
(Missing)	2 (0.2%)	2 (0.4%)
Transformative religious experience, n (%)		
Yes	250 (30.0%)	139 (26.5%)
No	571 (68.3%)	380 (72.3%)
(Missing)	14 (1.7%)	6 (1.2%)
Religious reading or listening, n (%)		
More than once a day	165 (19.7%)	97 (18.4%)
About once a day	165 (19.8%)	101 (19.3%)
Sometimes	397 (47.5%)	234 (44.6%)

Table S29d. Unweighted summary statistics for Wave 1 outcome variables in Turkey by retention status.

Outcome	Attritors–Not Observed in Wave 2<br>N = 836	Retained–Observed in Wave 2<br>N = 525
Never	105 (12.6%)	92 (17.5%)
(Missing)	4 (0.5%)	1 (0.3%)
Prayer or meditation, n (%)		
More than once a day	440 (52.7%)	272 (51.8%)
About once a day	156 (18.7%)	74 (14.0%)
Sometimes	169 (20.3%)	119 (22.7%)
Never	67 (8.1%)	59 (11.2%)
(Missing)	2 (0.3%)	1 (0.3%)
Belief in God/gods/spiritual forces, n (%)		
One God	699 (83.6%)	424 (80.7%)
More than one god	9 (1.0%)	5 (0.9%)
An impersonal spiritual force	9 (1.1%)	12 (2.3%)
None of these	67 (8.0%)	63 (12.0%)
Unsure	46 (5.5%)	21 (4.0%)
(Missing)	7 (0.8%)	0 (0.1%)
Religious centrality, n (%)		
Agree	612 (73.3%)	361 (68.7%)
Disagree	103 (12.3%)	86 (16.4%)
Not relevant	52 (6.2%)	35 (6.6%)
Unsure	65 (7.7%)	42 (8.0%)
(Missing)	4 (0.5%)	2 (0.3%)
Religious/spiritual comfort, n (%)		
Agree	660 (78.9%)	386 (73.5%)
Disagree	63 (7.5%)	79 (15.0%)
Not relevant	49 (5.9%)	31 (6.0%)
Unsure	61 (7.3%)	28 (5.3%)
(Missing)	4 (0.4%)	1 (0.2%)
Feel loved by God, n (%)		
Agree	659 (78.8%)	371 (70.7%)
Disagree	49 (5.8%)	63 (11.9%)
Not relevant	44 (5.2%)	33 (6.3%)
Unsure	77 (9.3%)	57 (10.8%)
(Missing)	8 (0.9%)	1 (0.2%)
Feel punished by God, n (%)		
Agree	255 (30.5%)	156 (29.8%)
Disagree	374 (44.7%)	270 (51.4%)
Not relevant	76 (9.1%)	37 (7.1%)
Unsure	121 (14.4%)	60 (11.4%)
(Missing)	11 (1.3%)	2 (0.4%)
Experienced religious criticism, n (%)		
Agree	305 (36.5%)	199 (37.9%)
Disagree	356 (42.6%)	225 (42.9%)
Not relevant	67 (8.0%)	40 (7.6%)
Unsure	104 (12.4%)	60 (11.5%)
(Missing)	4 (0.5%)	1 (0.2%)
Faith-sharing, n (%)		
Agree	498 (59.6%)	316 (60.2%)
Disagree	189 (22.7%)	134 (25.5%)
Not relevant	63 (7.5%)	35 (6.7%)

Table S29d. Unweighted summary statistics for Wave 1 outcome variables in Turkey by retention status.

Outcome	Attritors–Not Observed in Wave 2 N = 836	Retained–Observed in Wave 2 N = 525
Unsure	78 (9.3%)	39 (7.3%)
(Missing)	8 (0.9%)	1 (0.2%)

Note. N (%); this table is based on non-imputed data. Cumulative percentages for variables may not add up to 100% due to rounding.

Table S29e. Summary of fitted attrition model in Turkey

Characteristic	Odds Ratio	95% CI	p-value
**ANNUAL_WEIGHT_R2**	1.05	0.80, 1.38	0.716
**Recruitment Survey Mode**			
CATI	—	—	
CAPI	0.19	0.12, 0.31	6.88e-12
**Happiness & life satisfaction**	1.23	0.98, 1.53	0.071
**Physical & mental health**	0.88	0.71, 1.09	0.230
**Meaning & purpose**	0.91	0.73, 1.14	0.411
**Character & virtue**	0.93	0.78, 1.12	0.451
**Close social relationships**	1.23	1.02, 1.49	0.031
**Financial & material security**	0.96	0.81, 1.15	0.673
**Extraversion**	1.02	0.87, 1.19	0.811
**Openness to experience**	1.07	0.92, 1.25	0.382
**Agreeableness**	0.90	0.76, 1.06	0.192
**Conscientiousness**	0.93	0.79, 1.10	0.396
**Neuroticism**	1.15	0.97, 1.36	0.109
**Depression symptoms composite**	0.86	0.71, 1.05	0.131
**Anxiety symptoms composite**	1.15	0.94, 1.39	0.173
**Loneliness**	1.00	0.83, 1.21	0.966
**Days exercise per week**	1.15	0.98, 1.34	0.083
**Year of birth (age group)**			
1983-1993 (current age: 30-39 years)	—	—	
1973-1983 (current age: 40-49 years)	1.03	0.68, 1.55	0.893
1998-2005 (current age: 18-24 years)	0.51	0.27, 0.97	0.041
1963-1973 (current age: 50-59 years)	1.85	1.12, 3.06	0.017
1993-1998 (current age: 25-29 years)	1.17	0.68, 2.01	0.559
1953-1963 (current age: 60-69 years)	1.60	0.77, 3.31	0.204
1943-1953 (current age: 70-79 years)	0.70	0.22, 2.26	0.549
1943 or earlier (current age: 80+ years)	1.95	0.31, 12.2	0.474
**Gender of respondent**			
Male	—	—	
Female	0.80	0.55, 1.16	0.237
**Marital status**			
Married	—	—	
Single/Never been married	0.80	0.54, 1.20	0.286
Divorced	0.98	0.52, 1.87	0.962
Widowed	0.64	0.25, 1.66	0.360
Separated	0.61	0.15, 2.45	0.485
**Employment status**			
Employed for an employer	—	—	
Self-employed	0.61	0.40, 0.93	0.020
Homemaker	0.79	0.46, 1.36	0.405
Retired	0.72	0.39, 1.32	0.287
Student	0.96	0.47, 1.98	0.920
Unemployed and looking for a job	1.09	0.61, 1.95	0.774
None of these/Other	1.67	0.80, 3.50	0.176
**Religious attendance**			
More than once a week	—	—	
Never	1.50	0.99, 2.29	0.057
A few times a year	1.27	0.80, 2.04	0.312
Once a week	1.00	0.64, 1.56	0.996
One to three times a month	1.15	0.68, 1.95	0.594
**Educational attainment (16+ years)**			

Table S29e. Summary of fitted attrition model in Turkey

**Characteristic**	**Odds Ratio**	**95% CI**	**p-value**
9-15	—	—	
16+	0.99	0.69, 1.42	0.951
Up to 8	0.93	0.58, 1.48	0.762
**Born in This country**			
Born in this country	—	—	
Born in another country	0.81	0.33, 1.96	0.633
**Urbanicity**			
A large city	—	—	
A small town or village	1.23	0.86, 1.78	0.260
A suburb of a large city	0.83	0.53, 1.30	0.419
A rural area or on a farm	0.80	0.42, 1.52	0.500
**Monthly household income**			
Turkey: More than 12,000 TL	—	—	
Turkey: 10,001 - 12,000 TL	1.21	0.79, 1.87	0.383
Turkey: 8,001 - 10,000 TL	0.86	0.45, 1.65	0.644
Turkey: 6,001 - 8,000 TL	1.26	0.66, 2.38	0.484
(None/No household income)	0.40	0.15, 1.04	0.059
Turkey: 5,001 – 6,000 TL	0.30	0.10, 0.91	0.033
Turkey: 4,501 – 5,000 TL	0.61	0.17, 2.17	0.441
Turkey: 4,001 - 4,500 TL	0.42	0.08, 2.07	0.283
Turkey: 1,001 - 1,500 TL	2.52	0.83, 7.61	0.102
Turkey: 3,001 - 3,500 TL	1.21	0.27, 5.53	0.803
Turkey: 3,501 – 4,000 TL	1.15	0.22, 6.08	0.873
Turkey: 101 - 500 TL	0.46	0.05, 4.38	0.501
Turkey: 2,001 - 2,500 TL	0.08	0.01, 0.93	0.043
Turkey: 100 TL or less	0.94	0.09, 9.62	0.957
Turkey: 1,501 - 2,000 TL	0.24	0.02, 2.43	0.229
Turkey: 2,501 – 3,000 TL	0.61	0.04, 8.90	0.717
Turkey: 501 - 1,000 TL	1.84	0.26, 12.8	0.537

Abbreviations: CI = Confidence Interval, OR = Odds Ratio

Notes. N=1473; attrition weights were estimated using the ’survey::svyglm(family=quasibinomial(‘logit’))’ function. All continuous predictors were standardized and all categorical predictors used the most common category as the reference group. Reported p-values are based on the fitted regression model and no adjustments for multiple testing were done within this table.

Table S29f. Summary of principal components in Turkey

PC	Percent Explained by each PC	Cumulative Percent Explained
1	28.14	28.14
2	7.19	35.33
3	3.30	38.63
4	2.51	41.14
5	2.27	43.41
6	2.10	45.51
**7**	**1.85**	**47.36**
8	1.66	49.02
9	1.51	50.54
10	1.48	52.02
11	1.46	53.47
12	1.38	54.86
13	1.30	56.16
14	1.28	57.44
15	1.27	58.70
16	1.24	59.95
17	1.19	61.14
18	1.14	62.28
19	1.12	63.41
20	1.09	64.50

Notes. N=1473; PCA was conducted using 'survey::svyprcomp(.)' function using all available contemporaneous exposures at wave 1. All PCs were standardized prior to being used as predictors. The bolded row represented the number of retained components for analysis was 7.

Table S29g. Associations of forgivingness with adult well-being and other outcomes at Wave 2 in Turkey

	Model 1: Demographic and Childhood Variables as Covariates					Model 2: Demographic, Childhood, and Other Wave 1 Confounding Variables (Via Principal Components) as Covariates				
Outcome	RR	ES	95% CI	SE	p-value	RR	ES	95% CI	SE	p-value
Human Flourishing										
Secure flourishing index		0.12	(0.05,0.19)	0.04	1e-03**		0.11	(0.05,0.17)	0.03	4.16e-04***
Flourishing index		0.11	(0.04,0.18)	0.04	3.75e-03**		0.10	(0.04,0.16)	0.03	1.33e-03**
Happiness & life satisfaction		0.12	(0.03,0.20)	0.04	0.009*		0.11	(0.03,0.19)	0.04	0.011*
Physical & mental health		0.07	(-0.02,0.15)	0.05	0.157		0.07	(-0.02,0.15)	0.04	0.121
Meaning & purpose		0.05	(-0.03,0.14)	0.04	0.223		0.04	(-0.04,0.12)	0.04	0.344
Character & virtue		0.08	(0.00,0.16)	0.04	0.053		0.08	(0.00,0.16)	0.04	0.053
Close social relationships		0.06	(-0.02,0.14)	0.04	0.127		0.07	(-0.00,0.14)	0.04	0.070
Financial & material security		0.10	(0.02,0.18)	0.04	0.013*		0.08	(0.01,0.15)	0.04	0.037*
Psychological Well-Being										
Happiness (a)		0.11	(0.02,0.20)	0.05	0.024*		0.11	(0.02,0.20)	0.05	0.023*
Life satisfaction (a)		0.09	(-0.01,0.18)	0.05	0.080		0.08	(-0.01,0.18)	0.05	0.086
Current life evaluation		0.04	(-0.06,0.14)	0.05	0.473		0.02	(-0.07,0.11)	0.05	0.646
Future life evaluation		0.03	(-0.07,0.13)	0.05	0.570		0.02	(-0.07,0.12)	0.05	0.663
Optimism		0.08	(-0.01,0.17)	0.05	0.081		0.08	(-0.01,0.17)	0.05	0.097
Freedom to pursue what's important		-0.02	(-0.10,0.07)	0.05	0.729		-0.01	(-0.10,0.08)	0.05	0.768
Inner peace	1.03		(0.98,1.08)	0.02	0.241	1.03		(0.98,1.07)	0.02	0.273
Life balance	1.03		(0.99,1.07)	0.02	0.181	1.03		(0.99,1.07)	0.02	0.164
Sense of mastery	0.99		(0.96,1.03)	0.02	0.680	0.99		(0.96,1.03)	0.02	0.647
Meaningful activities (c)		0.05	(-0.04,0.13)	0.04	0.276		0.04	(-0.05,0.13)	0.04	0.367
Understanding purpose (c)		0.04	(-0.04,0.12)	0.04	0.354		0.03	(-0.05,0.10)	0.04	0.526
Self-rated mental health (b)		0.10	(-0.00,0.20)	0.05	0.068		0.10	(0.00,0.20)	0.05	0.052
Psychological Distress										
Traumatic distress	1.00		(0.96,1.04)	0.02	0.980	0.99		(0.96,1.03)	0.02	0.794
Depression symptoms composite	1.03		(0.98,1.07)	0.02	0.248	1.02		(0.98,1.07)	0.02	0.300
Depression – feel hopeless	1.02		(0.97,1.06)	0.02	0.435	1.02		(0.98,1.06)	0.02	0.412
Depression – loss of interest	1.04		(0.99,1.09)	0.03	0.153	1.03		(0.98,1.09)	0.03	0.213
Anxiety symptoms composite	1.02		(0.99,1.06)	0.02	0.198	1.02		(0.98,1.05)	0.02	0.338
Anxiety – feel on edge	1.01		(0.97,1.05)	0.02	0.564	1.01		(0.97,1.04)	0.02	0.781
Anxiety – cannot stop worrying	1.01		(0.97,1.06)	0.02	0.540	1.01		(0.97,1.05)	0.02	0.702
Suffering	0.97		(0.93,1.02)	0.02	0.284	0.97		(0.93,1.02)	0.02	0.214
Social Well-Being										
Relationship contentment (e)		0.04	(-0.05,0.13)	0.04	0.362		0.05	(-0.03,0.13)	0.04	0.221
Relationship satisfaction (e)		0.06	(-0.02,0.15)	0.04	0.160		0.06	(-0.02,0.14)	0.04	0.147
Social support		0.04	(-0.06,0.13)	0.05	0.436		0.03	(-0.06,0.12)	0.05	0.495
Intimate/close friend	1.02		(0.99,1.06)	0.02	0.222	1.02		(0.99,1.06)	0.02	0.197
Government approval	1.04		(0.99,1.09)	0.02	0.090	1.03		(0.99,1.08)	0.02	0.167
Say in government	1.02		(0.98,1.07)	0.02	0.352	1.02		(0.97,1.07)	0.03	0.450
Belonging in country		0.09	(0.02,0.17)	0.04	0.018*		0.08	(0.00,0.15)	0.04	0.038*
City/place satisfaction	1.01		(0.97,1.06)	0.02	0.549	1.01		(0.97,1.06)	0.02	0.517
Trust within country	1.02		(0.98,1.07)	0.02	0.236	1.02		(0.98,1.05)	0.02	0.429
Social Participation										
Ever been married	1.00		(0.98,1.01)	0.01	0.627	1.00		(0.98,1.01)	0.01	0.545
Currently divorced	1.00		(0.99,1.02)	0.01	0.928	1.00		(0.99,1.02)	0.01	0.976
Number of children		0.03	(-0.04,0.09)	0.03	0.426		0.04	(-0.03,0.10)	0.03	0.240
Weekly+ community participation	1.00		(0.97,1.03)	0.02	0.963	1.00		(0.97,1.03)	0.02	0.842
Weekly+ religious attendance	1.00		(0.96,1.04)	0.02	0.967	1.00		(0.96,1.04)	0.02	0.951
Social Distress										
Loneliness		-0.02	(-0.11,0.07)	0.05	0.657		-0.02	(-0.11,0.07)	0.05	0.663
Perceived discrimination	1.01		(0.96,1.06)	0.02	0.734	1.01		(0.96,1.05)	0.02	0.822
Character & Prosocial Behavior										
Orientation to promote good (d)		0.09	(0.00,0.18)	0.05	0.052		0.09	(-0.00,0.18)	0.05	0.065
Delayed gratification (d)		0.04	(-0.04,0.13)	0.04	0.309		0.04	(-0.04,0.13)	0.04	0.308
Hope		0.07	(-0.02,0.16)	0.05	0.140		0.07	(-0.03,0.16)	0.05	0.163
Gratitude		0.06	(-0.06,0.17)	0.06	0.344		0.06	(-0.06,0.17)	0.06	0.345
Showing love/care		0.13	(0.04,0.22)	0.04	0.005*		0.12	(0.03,0.21)	0.04	0.008*
Forgivingness	1.18		(1.12,1.23)	0.02	3.47e-08***	1.17		(1.12,1.23)	0.02	1.46e-07***
Charitable giving	0.98		(0.94,1.03)	0.02	0.442	0.98		(0.93,1.02)	0.02	0.321
Helping strangers	0.97		(0.93,1.01)	0.02	0.154	0.96		(0.92,1.01)	0.02	0.120
Volunteering	0.98		(0.94,1.03)	0.02	0.425	0.98		(0.93,1.02)	0.02	0.300
Physical Health & Health Behavior										
Self-rated physical health (b)		0.00	(-0.08,0.09)	0.04	0.926		0.00	(-0.08,0.08)	0.04	0.927
Health problems	0.99		(0.96,1.02)	0.02	0.505	0.98		(0.95,1.01)	0.02	0.307
Pain in past 4 weeks	0.99		(0.94,1.04)	0.03	0.696	0.99		(0.94,1.04)	0.03	0.566
Daily smoker	0.98		(0.94,1.02)	0.02	0.307	0.98		(0.94,1.02)	0.02	0.347
Number of drinks per week		-0.02	(-0.10,0.05)	0.04	0.536		-0.03	(-0.11,0.05)	0.04	0.455
Days exercise per week		-0.00	(-0.09,0.09)	0.05	0.959		-0.01	(-0.10,0.07)	0.04	0.780
Socioeconomic Outcomes										
Financial security (f)		0.11	(0.01,0.20)	0.05	0.030*		0.09	(0.00,0.18)	0.05	0.053
Material security (f)		0.06	(-0.02,0.14)	0.04	0.119		0.05	(-0.03,0.12)	0.04	0.239
Educational attainment (16+ years)	1.01		(0.99,1.04)	0.01	0.299	1.01		(0.99,1.04)	0.01	0.324
Currently employed	1.00		(0.97,1.04)	0.02	0.924	1.00		(0.96,1.04)	0.02	0.985
Financially comfortable/getting by	1.01		(0.97,1.06)	0.02	0.553	1.01		(0.96,1.06)	0.02	0.682
Own home	1.00		(0.96,1.05)	0.02	0.940	1.00		(0.96,1.04)	0.02	0.939
Income – top quintile	1.01		(0.98,1.04)	0.02	0.607	1.01		(0.98,1.04)	0.02	0.621
Religion & Spirituality										
Religious/spiritual connection	1.01		(0.96,1.05)	0.02	0.769	1.00		(0.96,1.05)	0.02	0.876
Belief in life after death	1.01		(0.97,1.05)	0.02	0.569	1.01		(0.97,1.05)	0.02	0.803
Transformative religious experience	1.05		(1.01,1.10)	0.02	0.031*	1.04		(1.00,1.09)	0.02	0.058
Religious reading or listening	1.03		(0.98,1.08)	0.02	0.249	1.02		(0.97,1.07)	0.02	0.414
Prayer or meditation	1.00		(0.97,1.04)	0.02	0.861	1.00		(0.96,1.03)	0.02	0.936
Belief in God/gods/spiritual forces	1.01		(0.98,1.03)	0.01	0.667	1.00		(0.98,1.03)	0.01	0.722
Religious centrality	1.00		(0.97,1.04)	0.02	0.815	1.00		(0.97,1.03)	0.01	0.872
Religious/spiritual comfort	1.01		(0.97,1.05)	0.02	0.612	1.01		(0.98,1.04)	0.02	0.578
Feel loved by God	1.00		(0.97,1.04)	0.02	0.887	1.00		(0.97,1.04)	0.02	0.985
Feel punished by God	1.02		(0.98,1.06)	0.02	0.382	1.02		(0.98,1.06)	0.02	0.390
Experienced religious criticism	1.01		(0.98,1.05)	0.02	0.500	1.01		(0.98,1.05)	0.02	0.545
Faith-sharing	0.98		(0.94,1.01)	0.02	0.217	0.97		(0.94,1.01)	0.02	0.152

Notes. N=1473; Reference for focal predictor: never/rarely. RR, risk-ratio, null effect is 1.00; ES, effect size measure for standardized regression coefficient, null effect is 0.00; SE, standard error, the SE reported for binary/Likert-type outcomes where risk-ratios are on the log(RR) scale; CI, confidence interval; p-value, a Wald-type test of the null hypothesis that the effect of the focal predictor is zero; (a) item part of the Happiness & Life Satisfaction domain of the Secure Flourishing Index; (b) item part of the Physical & Mental Health domain of the Secure Flourishing Index; (c) item part of the Meaning & Purpose domain of the Secure Flourishing Index; (d) item part of the Character & Virtue domain of the Secure Flourishing Index; (e) item part of the Subjective Social Connectedness domain of the Secure Flourishing Index; (f) item part of the Financial & Material Security domain of the Secure Flourishing Index.

Multiple imputation was performed to impute missing data on the covariates, exposure, and outcomes. All models controlled for sociodemographic and childhood factors assessed at Wave 1. For Model 2 with PC (principal components), the first seven principal components of the entire set of contemporaneous confounders assessed at Wave 1 were included as additional covariates of the outcomes at Wave 2.

An outcome-wide analytic approach was used, and a separate model was run for each outcome. A different type of model was run depending on the nature of the outcome: (1) for each binary outcome, a weighted generalized linear model (with a log link and Poisson distribution) was used to estimate an RR; and (2) for each continuous outcome, a weighted linear regression model was used to estimate a ES. All effect sizes were standardized. For continuous outcomes, the ES represents the change in SD on the outcome between the lower and upper categories of the binary focal predictor. For binary outcomes, the RR represents the change in risk of being in the upper category compared to the lower category between the lower and upper categories of the binary focal predictor.

P-value significance thresholds: $p < 0.05$*, $p < 0.005$**, (Bonferroni) $p < 6.41e-04$***, correction for multiple testing using Bonferroni adjusted significant threshold.

Table S29h. Associations of forgivingness with adult well-being and other outcomes at Wave 2 in Turkey using complete-case analyses with attrition weights.

Outcome	Model 1: Demographic and Childhood Variables as Covariates					Model 2: Demographic, Childhood, and Other Wave 1 Confounding Variables (Via Principal Components) as Covariates				
	RR	ES	95% CI	SE	p-value	RR	ES	95% CI	SE	p-value
Human Flourishing										
Secure flourishing index		0.12	(0.03,0.22)	0.05	0.009*		0.12	(0.04,0.20)	0.04	2.41e-03**
Flourishing index		0.12	(0.03,0.21)	0.05	0.012*		0.12	(0.04,0.19)	0.04	3.73e-03**
Happiness & life satisfaction		0.11	(0.01,0.20)	0.05	0.025*		0.11	(0.03,0.20)	0.04	0.010*
Physical & mental health		0.06	(-0.04,0.17)	0.05	0.239		0.06	(-0.04,0.16)	0.05	0.247
Meaning & purpose		0.08	(-0.02,0.18)	0.05	0.115		0.07	(-0.01,0.15)	0.04	0.076
Character & virtue		0.11	(0.01,0.22)	0.05	0.038*		0.11	(0.02,0.21)	0.05	0.023*
Close social relationships		0.11	(0.02,0.20)	0.05	0.023*		0.11	(0.02,0.19)	0.04	0.011*
Financial & material security		0.10	(-0.01,0.21)	0.06	0.076		0.10	(-0.00,0.20)	0.05	0.059
Psychological Well-Being										
Happiness (a)		0.10	(-0.00,0.20)	0.05	0.063		0.10	(0.00,0.19)	0.05	0.040*
Life satisfaction (a)		0.10	(0.01,0.20)	0.05	0.031*		0.11	(0.03,0.20)	0.04	0.011*
Current life evaluation		0.00	(-0.10,0.10)	0.05	0.942		0.01	(-0.09,0.10)	0.05	0.852
Future life evaluation		0.05	(-0.05,0.15)	0.05	0.310		0.04	(-0.06,0.14)	0.05	0.455
Optimism		0.17	(0.06,0.28)	0.05	1.88e-03**		0.17	(0.06,0.27)	0.05	1.69e-03**
Freedom to pursue what's important		-0.04	(-0.15,0.07)	0.06	0.435		-0.04	(-0.14,0.06)	0.05	0.399
Inner peace	1.00		(0.95,1.05)	0.03	0.964	1.00		(0.96,1.05)	0.02	0.902
Life balance	1.02		(0.97,1.07)	0.03	0.417	1.03		(0.98,1.08)	0.03	0.266
Sense of mastery	1.04		(0.99,1.09)	0.02	0.119	1.03		(0.99,1.08)	0.02	0.110
Meaningful activities (c)		0.10	(0.00,0.19)	0.05	0.047*		0.10	(0.01,0.19)	0.05	0.037*
Understanding purpose (c)		0.04	(-0.06,0.15)	0.06	0.419		0.04	(-0.05,0.12)	0.04	0.419
Self-rated mental health (b)		0.13	(0.03,0.24)	0.05	0.015*		0.13	(0.03,0.23)	0.05	0.014*
Psychological Distress										
Traumatic distress	0.97		(0.92,1.02)	0.03	0.274	0.96		(0.91,1.02)	0.03	0.169
Depression symptoms composite	1.04		(0.99,1.10)	0.03	0.098	1.04		(0.99,1.09)	0.03	0.142
Depression – feel hopeless	1.03		(0.98,1.09)	0.03	0.213	1.03		(0.98,1.09)	0.03	0.178
Depression – loss of interest	1.06		(1.00,1.12)	0.03	0.042*	1.05		(1.00,1.11)	0.03	0.076
Anxiety symptoms composite	1.02		(0.96,1.07)	0.03	0.566	1.01		(0.96,1.06)	0.02	0.779
Anxiety – feel on edge	0.98		(0.93,1.03)	0.03	0.387	0.97		(0.92,1.02)	0.03	0.236
Anxiety – cannot stop worrying	0.99		(0.94,1.04)	0.03	0.656	0.98		(0.94,1.03)	0.02	0.398
Suffering	0.97		(0.92,1.02)	0.03	0.208	0.96		(0.92,1.01)	0.02	0.116
Social Well-Being										
Relationship contentment (e)		0.07	(-0.03,0.17)	0.05	0.171		0.07	(-0.02,0.15)	0.04	0.119
Relationship satisfaction (e)		0.13	(0.04,0.21)	0.04	4.92e-03**		0.13	(0.04,0.21)	0.04	3.85e-03**
Social support		0.05	(-0.06,0.17)	0.06	0.339		0.07	(-0.04,0.18)	0.05	0.194
Intimate/close friend	1.01		(0.96,1.07)	0.03	0.615	1.01		(0.96,1.06)	0.03	0.699
Government approval	1.06		(1.00,1.12)	0.03	0.034*	1.05		(1.00,1.10)	0.02	0.057
Say in government	1.03		(0.98,1.09)	0.03	0.211	1.03		(0.98,1.08)	0.02	0.218
Belonging in country		0.02	(-0.10,0.14)	0.06	0.718		0.03	(-0.07,0.13)	0.05	0.572
City/place satisfaction	0.99		(0.94,1.04)	0.03	0.711	0.99		(0.95,1.04)	0.02	0.830
Trust within country	1.01		(0.96,1.06)	0.02	0.644	1.00		(0.96,1.05)	0.02	0.982
Social Participation										
Ever been married	0.99		(0.98,1.01)	0.01	0.388	0.99		(0.98,1.01)	0.01	0.406
Currently divorced	1.01		(1.00,1.02)	0.01	0.209	1.01		(1.00,1.02)	0.01	0.139
Number of children		0.06	(-0.05,0.18)	0.06	0.290		0.02	(-0.07,0.12)	0.05	0.611
Weekly+ community participation	0.97		(0.93,1.02)	0.02	0.268	0.98		(0.93,1.02)	0.02	0.293
Weekly+ religious attendance	1.02		(0.98,1.08)	0.02	0.327	1.02		(0.97,1.06)	0.02	0.515
Social Distress										
Loneliness		0.06	(-0.06,0.17)	0.06	0.332		0.03	(-0.07,0.12)	0.05	0.606
Perceived discrimination	1.02		(0.97,1.07)	0.03	0.512	1.01		(0.97,1.06)	0.02	0.575
Character & Prosocial Behavior										
Orientation to promote good (d)		0.15	(0.04,0.26)	0.06	0.008*		0.16	(0.06,0.26)	0.05	2.55e-03**
Delayed gratification (d)		0.05	(-0.06,0.15)	0.05	0.384		0.04	(-0.06,0.14)	0.05	0.434
Hope		0.03	(-0.08,0.15)	0.06	0.571		0.02	(-0.08,0.11)	0.05	0.684
Gratitude		0.13	(0.02,0.24)	0.06	0.022*		0.12	(0.02,0.22)	0.05	0.021*
Showing love/care		0.13	(0.02,0.24)	0.05	0.017*		0.14	(0.03,0.24)	0.05	0.013*
Forgivingness	1.24		(1.18,1.29)	0.02	2.22e-16***	1.22		(1.16,1.28)	0.02	4e-15***
Charitable giving	0.99		(0.94,1.04)	0.03	0.586	0.99		(0.94,1.04)	0.03	0.630
Helping strangers	0.98		(0.93,1.03)	0.02	0.371	0.98		(0.94,1.02)	0.02	0.341
Volunteering	0.98		(0.94,1.02)	0.02	0.273	0.98		(0.94,1.02)	0.02	0.262
Physical Health & Health Behavior										
Self-rated physical health (b)		-0.03	(-0.14,0.08)	0.06	0.575		-0.03	(-0.13,0.07)	0.05	0.570
Health problems	0.99		(0.95,1.03)	0.02	0.687	0.99		(0.96,1.03)	0.02	0.733
Pain in past 4 weeks	1.00		(0.95,1.05)	0.03	0.972	0.99		(0.94,1.04)	0.02	0.623
Daily smoker	1.00		(0.94,1.06)	0.03	0.950	1.00		(0.95,1.06)	0.03	0.885
Number of drinks per week		-0.05	(-0.19,0.09)	0.07	0.476		-0.03	(-0.16,0.10)	0.07	0.683
Days exercise per week		-0.05	(-0.16,0.06)	0.06	0.371		-0.02	(-0.12,0.08)	0.05	0.730
Socioeconomic Outcomes										
Financial security (f)		0.13	(0.02,0.23)	0.06	0.026*		0.13	(0.03,0.23)	0.05	0.012*
Material security (f)		0.06	(-0.05,0.17)	0.06	0.285		0.06	(-0.05,0.17)	0.06	0.298
Educational attainment (16+ years)	1.01		(0.99,1.03)	0.01	0.363	1.01		(0.99,1.03)	0.01	0.340
Currently employed	0.99		(0.95,1.04)	0.02	0.759	0.99		(0.94,1.04)	0.02	0.645
Financially comfortable/getting by	1.02		(0.96,1.07)	0.03	0.587	1.02		(0.97,1.08)	0.03	0.435
Own home	0.98		(0.93,1.03)	0.03	0.396	0.98		(0.92,1.03)	0.03	0.359
Income – top quintile	1.01		(0.97,1.05)	0.02	0.757	1.01		(0.97,1.05)	0.02	0.641
Religion & Spirituality										
Religious/spiritual connection	1.01		(0.96,1.06)	0.03	0.598	0.99		(0.94,1.03)	0.02	0.492
Belief in life after death	1.03		(0.98,1.08)	0.02	0.207	1.02		(0.97,1.07)	0.02	0.415
Transformative religious experience	1.04		(1.00,1.09)	0.02	0.053	1.04		(1.00,1.09)	0.02	0.068
Religious reading or listening	1.04		(0.98,1.09)	0.03	0.178	1.03		(0.98,1.08)	0.02	0.251
Prayer or meditation	1.02		(0.98,1.06)	0.02	0.448	1.00		(0.96,1.04)	0.02	0.977
Belief in God/gods/spiritual forces	1.00		(0.97,1.02)	0.01	0.809	0.98		(0.96,1.01)	0.01	0.220
Religious centrality	1.01		(0.97,1.05)	0.02	0.784	0.99		(0.96,1.03)	0.02	0.761
Religious/spiritual comfort	1.01		(0.97,1.04)	0.02	0.753	0.99		(0.96,1.02)	0.01	0.383
Feel loved by God	1.01		(0.98,1.05)	0.02	0.456	1.00		(0.97,1.03)	0.02	0.774
Feel punished by God	1.05		(1.00,1.11)	0.03	0.050	1.04		(0.99,1.09)	0.03	0.158
Experienced religious criticism	0.99		(0.94,1.05)	0.03	0.842	0.99		(0.94,1.05)	0.03	0.765
Faith-sharing	1.00		(0.95,1.05)	0.03	0.901	0.99		(0.94,1.03)	0.02	0.558

Table S29h. Associations of forgivingness with adult well-being and other outcomes at Wave 2 in Turkey using complete-case analyses with attrition weights.

	Model 1: Demographic and Childhood Variables as Covariates					Model 2: Demographic, Childhood, and Other Wave 1 Confounding Variables (Via Principal Components) as Covariates				
Outcome	RR	ES	95% CI	SE	p-value	RR	ES	95% CI	SE	p-value

Notes. N=500; Reference for focal predictor: never/rarely. RR, risk-ratio, null effect is 1.00; ES, effect size measure for standardized regression coefficient, null effect is 0.00; SE, standard error, the SE reported for binary/Likert-type outcomes where risk-ratios are on the log(RR) scale; CI, confidence interval; p-value, a Wald-type test of the null hypothesis that the effect of the focal predictor is zero; (a) item part of the Happiness & Life Satisfaction domain of the Secure Flourishing Index; (b) item part of the Physical & Mental Health domain of the Secure Flourishing Index; (c) item part of the Meaning & Purpose domain of the Secure Flourishing Index; (d) item part of the Character & Virtue domain of the Secure Flourishing Index; (e) item part of the Subjective Social Connectedness domain of the Secure Flourishing Index; (f) item part of the Financial & Material Security domain of the Secure Flourishing Index.

Attrition weights were computed to adjust the complete case data (those who responded at Wave 2 to at least 50% of the questions) and multiple imputation was used to impute missing data on all remaining within wave on the covariates, exposure, and outcomes. All models controlled for sociodemographic and childhood factors assessed at Wave 1. For Model 2 with PC (principal components), the first seven principal components of the entire set of contemporaneous confounders assessed at Wave 1 were included as additional covariates of the outcomes at Wave 2.

An outcome-wide analytic approach was used, and a separate model was run for each outcome. A different type of model was run depending on the nature of the outcome: (1) for each binary outcome, a weighted generalized linear model (with a log link and Poisson distribution) was used to estimate an RR; and (2) for each continuous outcome, a weighted linear regression model was used to estimate a ES. All effect sizes were standardized. For continuous outcomes, the ES represents the change in SD on the outcome between the lower and upper categories of the binary focal predictor. For binary outcomes, the RR represents the change in risk of being in the upper category compared to the lower category between the lower and upper categories of the binary focal predictor.

P-value significance thresholds: $p < 0.05$*, $p < 0.005$**, (Bonferroni) $p < 6.41e-04$***, correction for multiple testing using Bonferroni adjusted significant threshold.

Table S29i. Sensitivity analysis of forgivingness outcome-wide results to unmeasured confounding using E-values in Turkey

	Multiple Imputation				Complete Case w/ Attrition Weights			
	Model 1: Demographics and Childhood Variables as Covariates		Model 2: Demographics, Childhood, and Other Wave 1 Confounders (Via Principal Components) as Covariates		Model 1: Demographics and Childhood Variables as Covariates		Model 2: Demographics, Childhood, and Other Wave 1 Confounders (Via Principal Components) as Covariates	
Outcome	EE	ECI	EE	ECI	EE	ECI	EE	ECI
Human Flourishing								
Secure flourishing index	1.79	1.42	1.75	1.42	1.82	1.31	1.82	1.39
Flourishing index	1.73	1.34	1.71	1.38	1.79	1.28	1.78	1.35
Happiness & life satisfaction	1.78	1.31	1.76	1.30	1.73	1.19	1.76	1.29
Physical & mental health	1.51	1.00	1.52	1.00	1.50	1.00	1.48	1.00
Meaning & purpose	1.44	1.00	1.36	1.00	1.58	1.00	1.55	1.00
Character & virtue	1.60	1.03	1.59	1.02	1.76	1.12	1.76	1.21
Close social relationships	1.49	1.00	1.53	1.00	1.73	1.20	1.72	1.27
Financial & material security	1.70	1.26	1.59	1.12	1.69	1.00	1.70	1.00
Psychological Well-Being								
Happiness	1.76	1.22	1.74	1.22	1.67	1.00	1.68	1.11
Life satisfaction	1.63	1.00	1.62	1.00	1.71	1.15	1.76	1.28
Current life evaluation	1.34	1.00	1.25	1.00	1.09	1.00	1.15	1.00
Future life evaluation	1.30	1.00	1.25	1.00	1.43	1.00	1.35	1.00
Optimism	1.60	1.00	1.58	1.00	2.08	1.50	2.06	1.50
Freedom to pursue what’s important	1.20	1.00	1.19	1.00	1.38	1.00	1.37	1.00
Inner peace	1.30	1.00	1.29	1.00	1.05	1.00	1.08	1.00
Life balance	1.30	1.00	1.30	1.00	1.26	1.00	1.32	1.00
Sense of mastery	1.14	1.00	1.15	1.00	1.36	1.00	1.34	1.00
Meaningful activities	1.41	1.00	1.36	1.00	1.68	1.06	1.67	1.12
Understanding purpose	1.35	1.00	1.27	1.00	1.39	1.00	1.33	1.00
Self-rated mental health	1.69	1.00	1.71	1.07	1.87	1.28	1.83	1.27
Psychological Distress								
Traumatic distress	1.03	1.00	1.11	1.00	1.32	1.00	1.37	1.00
Depression symptoms composite	1.30	1.00	1.27	1.00	1.41	1.00	1.37	1.00
Depression – feel hopeless	1.23	1.00	1.23	1.00	1.34	1.00	1.35	1.00
Depression – loss of interest	1.37	1.00	1.34	1.00	1.49	1.07	1.44	1.00
Anxiety symptoms composite	1.28	1.00	1.22	1.00	1.22	1.00	1.13	1.00
Anxiety – feel on edge	1.17	1.00	1.11	1.00	1.27	1.00	1.32	1.00
Anxiety – cannot stop worrying	1.20	1.00	1.15	1.00	1.18	1.00	1.25	1.00
Suffering	1.29	1.00	1.32	1.00	1.34	1.00	1.38	1.00
Social Well-Being								
Relationship contentment	1.36	1.00	1.43	1.00	1.53	1.00	1.51	1.00
Relationship satisfaction	1.49	1.00	1.49	1.00	1.84	1.36	1.84	1.37
Social support	1.35	1.00	1.31	1.00	1.45	1.00	1.53	1.00
Intimate/close friend	1.27	1.00	1.28	1.00	1.19	1.00	1.16	1.00
Government approval	1.39	1.00	1.35	1.00	1.49	1.10	1.43	1.00
Say in government	1.27	1.00	1.24	1.00	1.34	1.00	1.32	1.00
Belonging in country	1.66	1.21	1.58	1.11	1.25	1.00	1.30	1.00
City/place satisfaction	1.20	1.00	1.21	1.00	1.16	1.00	1.11	1.00
Trust within country	1.28	1.00	1.21	1.00	1.18	1.00	1.03	1.00
Social Participation								
Ever been married	1.10	1.00	1.11	1.00	1.13	1.00	1.13	1.00
Currently divorced	1.04	1.00	1.02	1.00	1.14	1.00	1.16	1.00
Number of children	1.27	1.00	1.35	1.00	1.49	1.00	1.26	1.00
Weekly+ community participation	1.04	1.00	1.09	1.00	1.30	1.00	1.28	1.00
Weekly+ religious attendance	1.04	1.00	1.05	1.00	1.28	1.00	1.21	1.00
Social Distress								
Loneliness	1.24	1.00	1.24	1.00	1.45	1.00	1.27	1.00
Perceived discrimination	1.15	1.00	1.11	1.00	1.22	1.00	1.20	1.00
Character & Prosocial Behavior								
Orientation to promote good	1.64	1.04	1.62	1.00	1.95	1.36	2.00	1.45
Delayed gratification	1.39	1.00	1.39	1.00	1.41	1.00	1.36	1.00
Hope	1.54	1.00	1.51	1.00	1.33	1.00	1.23	1.00
Gratitude	1.46	1.00	1.46	1.00	1.85	1.23	1.80	1.22
Showing love/care	1.86	1.38	1.81	1.33	1.87	1.27	1.89	1.29
Forgivingness	2.13	1.83	2.10	1.79	2.43	2.14	2.36	2.06
Charitable giving	1.24	1.00	1.28	1.00	1.20	1.00	1.18	1.00
Helping strangers	1.34	1.00	1.36	1.00	1.26	1.00	1.26	1.00
Volunteering	1.25	1.00	1.29	1.00	1.27	1.00	1.27	1.00
Physical Health & Health Behavior								
Self-rated physical health	1.09	1.00	1.09	1.00	1.31	1.00	1.30	1.00
Health problems	1.18	1.00	1.22	1.00	1.15	1.00	1.13	1.00
Pain in past 4 weeks	1.17	1.00	1.21	1.00	1.05	1.00	1.18	1.00
Daily smoker	1.27	1.00	1.26	1.00	1.07	1.00	1.10	1.00
Number of drinks per week	1.26	1.00	1.30	1.00	1.43	1.00	1.28	1.00
Days exercise per week	1.07	1.00	1.18	1.00	1.43	1.00	1.22	1.00
Socioeconomic Outcomes								
Financial security	1.74	1.19	1.65	1.05	1.83	1.20	1.85	1.30
Material security	1.50	1.00	1.40	1.00	1.48	1.00	1.47	1.00
Educational attainment (16+ years)	1.20	1.00	1.19	1.00	1.17	1.00	1.17	1.00
Currently employed	1.06	1.00	1.03	1.00	1.14	1.00	1.17	1.00
Financially comfortable/getting by	1.21	1.00	1.17	1.00	1.21	1.00	1.25	1.00
Own home	1.06	1.00	1.06	1.00	1.27	1.00	1.29	1.00
Income – top quintile	1.15	1.00	1.14	1.00	1.13	1.00	1.16	1.00
Religion & Spirituality								
Religious/spiritual connection	1.13	1.00	1.09	1.00	1.19	1.00	1.21	1.00
Belief in life after death	1.18	1.00	1.11	1.00	1.33	1.00	1.24	1.00
Transformative religious experience	1.45	1.11	1.41	1.00	1.40	1.00	1.40	1.00
Religious reading or listening	1.31	1.00	1.25	1.00	1.36	1.00	1.31	1.00
Prayer or meditation	1.09	1.00	1.06	1.00	1.22	1.00	1.03	1.00
Belief in God/gods/spiritual forces	1.11	1.00	1.10	1.00	1.09	1.00	1.22	1.00
Religious centrality	1.10	1.00	1.07	1.00	1.12	1.00	1.11	1.00
Religious/spiritual comfort	1.16	1.00	1.16	1.00	1.11	1.00	1.19	1.00
Feel loved by God	1.08	1.00	1.03	1.00	1.19	1.00	1.11	1.00
Feel punished by God	1.23	1.00	1.22	1.00	1.45	1.00	1.36	1.00
Experienced religious criticism	1.19	1.00	1.18	1.00	1.11	1.00	1.14	1.00
Faith-sharing	1.28	1.00	1.31	1.00	1.09	1.00	1.20	1.00

Notes. EE, E-value for estimate; ECI, E-value for the limit of the confidence interval. The formula for calculating E-values can be found in VanderWeele and Ding (2017). E-values for estimate are the minimum strength of association on the risk ratio scale that an unmeasured confounder would need to have with both the exposure and the outcome to fully explain away the observed association between the exposure and outcome, conditional on the measured covariates. E-values for the 95% CI closest to the null denote the minimum strength of association on the risk ratio scale that an unmeasured confounder would need to have with both the exposure and the outcome to shift the CI to include the null value, conditional on the measured covariates.

Table S30a. Weighted summary statistics for demographic and childhood variables in United Kingdom

**Characteristic**	**Wave 1**<br>N = 5,368	**Wave 2**<br>N = 3,615
Forgivingness, n (%)		
Always	910 (17.0%)	593 (16.4%)
Often	2,900 (54.0%)	1,900 (52.6%)
Rarely	1,325 (24.7%)	938 (25.9%)
Never	222 (4.1%)	180 (5.0%)
(Missing)	10 (0.2%)	4 (0.1%)
Year of birth, n (%)		
1943 or earlier (current age: 80+ years)	151 (2.8%)	130 (3.6%)
1943-1953 (current age: 70-79 years)	676 (12.6%)	485 (13.4%)
1953-1963 (current age: 60-69 years)	831 (15.5%)	583 (16.1%)
1963-1973 (current age: 50-59 years)	949 (17.7%)	632 (17.5%)
1973-1983 (current age: 40-49 years)	823 (15.3%)	572 (15.8%)
1983-1993 (current age: 30-39 years)	967 (18.0%)	654 (18.1%)
1993-1998 (current age: 25-29 years)	432 (8.0%)	290 (8.0%)
1998-2005 (current age: 18-24 years)	538 (10.0%)	268 (7.4%)
(Missing)	1 (0.0%)	0 (0%)
Age of participant		
Mean	48.5	49.9
Standard Deviation	17.9	17.7
Min, Max	18.0, 95.0	19.0, 93.0
(Missing)	1 (<0.1%)	0 (0%)
Gender, n (%)		
Male	2,585 (48.2%)	1,712 (47.4%)
Female	2,760 (51.4%)	1,886 (52.2%)
Other	15 (0.3%)	11 (0.3%)
(Missing)	8 (0.1%)	5 (0.1%)
Respondent marital status, n (%)		
Single/Never been married	1,516 (28.2%)	965 (26.7%)
Married	2,473 (46.1%)	1,708 (47.2%)
Separated	113 (2.1%)	85 (2.3%)
Divorced	417 (7.8%)	288 (8.0%)
Widowed	277 (5.2%)	195 (5.4%)
Domestic partner	523 (9.7%)	361 (10.0%)
(Missing)	49 (0.9%)	14 (0.4%)
Education (years), n (%)		
Up to 8	1,279 (23.8%)	579 (16.0%)
9-15	2,125 (39.6%)	1,474 (40.8%)
16+	1,956 (36.4%)	1,562 (43.2%)
(Missing)	8 (0.2%)	0 (0%)
Employment status, n (%)		
Employed for an employer	2,840 (52.9%)	1,844 (51.0%)
Self-employed	460 (8.6%)	355 (9.8%)
Retired	1,211 (22.6%)	862 (23.9%)
Student	257 (4.8%)	108 (3.0%)
Homemaker	181 (3.4%)	130 (3.6%)
Unemployed and looking for a job	207 (3.9%)	131 (3.6%)
None of these/Other	203 (3.8%)	154 (4.3%)
(Missing)	9 (0.2%)	32 (0.9%)
Current religious service attendance, n (%)		
More than once a week	287 (5.3%)	186 (5.1%)

Table S30a. Weighted summary statistics for demographic and childhood variables in United Kingdom

Characteristic	Wave 1<br>N = 5,368	Wave 2<br>N = 3,615
Once a week	493 (9.2%)	316 (8.7%)
One to three times a month	295 (5.5%)	204 (5.6%)
A few times a year	1,147 (21.4%)	788 (21.8%)
Never	3,135 (58.4%)	2,111 (58.4%)
(Missing)	10 (0.2%)	11 (0.3%)
Immigration status, n (%)		
Born in this country	4,658 (86.8%)	3,149 (87.1%)
Born in another country	684 (12.8%)	461 (12.8%)
(Missing)	25 (0.5%)	4 (0.1%)
Parental marital status around age 12, n (%)		
Parents were married	4,311 (80.3%)	2,912 (80.6%)
Parents were divorced	495 (9.2%)	346 (9.6%)
Parents were never married	326 (6.1%)	191 (5.3%)
One or both of them had died	161 (3.0%)	119 (3.3%)
Unsure	41 (0.8%)	38 (1.1%)
(Missing)	34 (0.6%)	9 (0.2%)
Religious service attendance around age 12, n (%)		
At least once a week	1,695 (31.6%)	1,126 (31.2%)
One to three times a month	736 (13.7%)	482 (13.3%)
Less than once a month	906 (16.9%)	612 (16.9%)
Never	2,002 (37.3%)	1,375 (38.0%)
(Missing)	28 (0.5%)	19 (0.5%)
Relationship with mother when growing up, n (%)		
Very good	3,391 (63.2%)	2,242 (62.0%)
Somewhat good	1,364 (25.4%)	961 (26.6%)
Somewhat bad	333 (6.2%)	238 (6.6%)
Very bad	150 (2.8%)	101 (2.8%)
(Does not apply)	105 (1.9%)	63 (1.7%)
(Missing)	26 (0.5%)	10 (0.3%)
Relationship with father when growing up, n (%)		
Very good	2,828 (52.7%)	1,907 (52.8%)
Somewhat good	1,411 (26.3%)	965 (26.7%)
Somewhat bad	426 (7.9%)	311 (8.6%)
Very bad	332 (6.2%)	204 (5.6%)
(Does not apply)	342 (6.4%)	213 (5.9%)
(Missing)	29 (0.5%)	15 (0.4%)
Felt like an outsider in family when growing up, n (%)		
Yes	1,057 (19.7%)	637 (17.6%)
No	4,266 (79.5%)	2,944 (81.4%)
(Missing)	46 (0.8%)	35 (1.0%)
Experienced abuse when growing up, n (%)		
Yes	878 (16.4%)	600 (16.6%)
No	4,444 (82.8%)	2,998 (82.9%)
(Missing)	46 (0.9%)	18 (0.5%)
Self-rated health when growing up, n (%)		
Excellent	2,122 (39.5%)	1,448 (40.1%)
Very good	1,753 (32.7%)	1,093 (30.2%)
Good	1,002 (18.7%)	710 (19.7%)
Fair	337 (6.3%)	253 (7.0%)
Poor	134 (2.5%)	92 (2.6%)

Table S30a. Weighted summary statistics for demographic and childhood variables in United Kingdom

**Characteristic**	**Wave 1** N = 5,368	**Wave 2** N = 3,615
(Missing)	19 (0.4%)	18 (0.5%)
Subjective financial status of family growing up, n (%)		
Lived comfortably	2,504 (46.6%)	1,592 (44.0%)
Got by	1,954 (36.4%)	1,383 (38.2%)
Found it difficult	656 (12.2%)	456 (12.6%)
Found it very difficult	230 (4.3%)	163 (4.5%)
(Missing)	24 (0.5%)	21 (0.6%)
Religious affiliation growing up, n (%)		
Christianity	3,421 (63.7%)	2,311 (63.9%)
Taoism	1 (0.0%)	0 (0%)
Confucianism	3 (0.1%)	1 (0.0%)
Primal, Animist, or Folk religion	20 (0.4%)	8 (0.2%)
Spiritism	0 (0%)	0 (0%)
Umbanda, Candomblé, and other African-derived religions	0 (0%)	0 (0%)
Chinese folk/traditional religion	0 (0%)	0 (0%)
Islam	240 (4.5%)	183 (5.1%)
Hinduism	87 (1.6%)	49 (1.4%)
Buddhism	16 (0.3%)	18 (0.5%)
Judaism	54 (1.0%)	35 (1.0%)
Sikhism	28 (0.5%)	10 (0.3%)
Baha'i	4 (0.1%)	4 (0.1%)
Jainism	0 (<0.0%)	0 (<0.0%)
Shinto	0 (0%)	0 (0%)
Some other religion	25 (0.5%)	19 (0.5%)
No religion/Atheist/Agnostic	1,447 (26.9%)	956 (26.4%)
(Missing)	21 (0.4%)	21 (0.6%)

Note. N (%); this table is based on non-imputed data. Cumulative percentages for variables may not add up to 100% due to rounding. Wave 1 characteristics weighted using the Gallup provided sampling weight, ANNUAL_WEIGHT_R2; Wave 2 characteristics weighted accounting for attrition by using the adjusted Wave 1 weight, ANNUAL_WEIGHT_R2, multiplied by the created attrition weight to account for dropout, to maintain nationally representative estimates for Wave 2 characteristics.

Table S30b. Weighted summary statistics for outcome variables in United Kingdom

**Outcome**	**Wave 1**<br>N = 5,368	**Wave 2**<br>N = 3,615
Secure flourishing index		
Mean	6.8	6.7
Standard Deviation	1.7	1.7
Min, Max	0.0, 10.0	0.2, 10.0
(Missing)	177 (3.3%)	163 (4.5%)
Flourishing index		
Mean	6.8	6.8
Standard Deviation	1.7	1.7
Min, Max	0.0, 10.0	0.1, 10.0
(Missing)	150 (2.8%)	147 (4.1%)
Happiness & life satisfaction		
Mean	6.6	6.5
Standard Deviation	2.1	2.1
Min, Max	0.0, 10.0	0.0, 10.0
(Missing)	49 (0.9%)	28 (0.8%)
Physical & mental health		
Mean	6.6	6.5
Standard Deviation	2.1	2.1
Min, Max	0.0, 10.0	0.0, 10.0
(Missing)	11 (0.2%)	62 (1.7%)
Meaning & purpose		
Mean	6.7	6.8
Standard Deviation	2.3	2.3
Min, Max	0.0, 10.0	0.0, 10.0
(Missing)	22 (0.4%)	25 (0.7%)
Character & virtue		
Mean	7.3	7.2
Standard Deviation	1.7	1.7
Min, Max	0.0, 10.0	0.0, 10.0
(Missing)	38 (0.7%)	36 (1.0%)
Close social relationships		
Mean	7.1	7.0
Standard Deviation	2.3	2.4
Min, Max	0.0, 10.0	0.0, 10.0
(Missing)	56 (1.0%)	25 (0.7%)
Financial & material security		
Mean	6.3	6.4
Standard Deviation	2.7	2.8
Min, Max	0.0, 10.0	0.0, 10.0
(Missing)	37 (0.7%)	22 (0.6%)
Happiness		
Mean	6.7	6.6
Standard Deviation	2.1	2.1
Min, Max	0.0, 10.0	0.0, 10.0
(Missing)	13 (0.2%)	16 (0.5%)
Life satisfaction		
Mean	6.5	6.4
Standard Deviation	2.3	2.2
Min, Max	0.0, 10.0	0.0, 10.0
(Missing)	38 (0.7%)	16 (0.5%)
Current life evaluation		

Table S30b. Weighted summary statistics for outcome variables in United Kingdom

Outcome	Wave 1<br>N = 5,368	Wave 2<br>N = 3,615
Mean	6.5	6.3
Standard Deviation	2.0	2.0
Min, Max	0.0, 10.0	0.0, 10.0
(Missing)	15 (0.3%)	8 (0.2%)
Future life evaluation		
Mean	7.1	7.0
Standard Deviation	2.1	2.2
Min, Max	0.0, 10.0	0.0, 10.0
(Missing)	13 (0.2%)	14 (0.4%)
Optimism		
Mean	6.9	6.8
Standard Deviation	2.5	2.5
Min, Max	0.0, 10.0	0.0, 10.0
(Missing)	35 (0.6%)	22 (0.6%)
Freedom to pursue what's important		
Mean	7.0	7.0
Standard Deviation	2.5	2.5
Min, Max	0.0, 10.0	0.0, 10.0
(Missing)	15 (0.3%)	12 (0.3%)
Inner peace, n (%)		
Always	735 (13.7%)	480 (13.3%)
Often	3,081 (57.4%)	2,058 (56.9%)
Rarely	1,353 (25.2%)	959 (26.5%)
Never	180 (3.4%)	89 (2.5%)
(Missing)	18 (0.3%)	30 (0.8%)
Life balance, n (%)		
Always	523 (9.7%)	395 (10.9%)
Often	3,173 (59.1%)	2,085 (57.7%)
Rarely	1,493 (27.8%)	1,029 (28.5%)
Never	157 (2.9%)	95 (2.6%)
(Missing)	23 (0.4%)	12 (0.3%)
Sense of mastery, n (%)		
Always	775 (14.4%)	523 (14.5%)
Often	3,503 (65.3%)	2,304 (63.7%)
Rarely	988 (18.4%)	698 (19.3%)
Never	80 (1.5%)	72 (2.0%)
(Missing)	22 (0.4%)	17 (0.5%)
Meaningful activities		
Mean	6.9	6.9
Standard Deviation	2.3	2.3
Min, Max	0.0, 10.0	0.0, 10.0
(Missing)	11 (0.2%)	16 (0.4%)
Understanding purpose		
Mean	6.6	6.6
Standard Deviation	2.7	2.7
Min, Max	0.0, 10.0	0.0, 10.0
(Missing)	11 (0.2%)	11 (0.3%)
Self-rated mental health		
Mean	6.7	6.8
Standard Deviation	2.5	2.5
Min, Max	0.0, 10.0	0.0, 10.0

Table S30b. Weighted summary statistics for outcome variables in United Kingdom

Outcome	Wave 1<br>N = 5,368	Wave 2<br>N = 3,615
(Missing)	10 (0.2%)	10 (0.3%)
Traumatic distress, n (%)		
A lot	480 (8.9%)	271 (7.5%)
Some	1,302 (24.3%)	834 (23.1%)
Not very much	1,635 (30.5%)	1,119 (31.0%)
Not at all	1,926 (35.9%)	1,386 (38.3%)
(Missing)	25 (0.5%)	4 (0.1%)
Depression symptoms composite, n (%)	1,597 (29.9%)	1,041 (29.0%)
(Missing)	23 (0.4%)	23 (0.6%)
Depression – feel hopeless, n (%)		
Nearly every day	501 (9.3%)	351 (9.7%)
More than half the days	774 (14.4%)	498 (13.8%)
Several days	1,663 (31.0%)	1,080 (29.9%)
Not at all	2,411 (44.9%)	1,667 (46.1%)
(Missing)	18 (0.3%)	18 (0.5%)
Depression – loss of interest, n (%)		
Nearly every day	522 (9.7%)	361 (10.0%)
More than half the days	966 (18.0%)	619 (17.1%)
Several days	1,595 (29.7%)	1,060 (29.3%)
Not at all	2,279 (42.5%)	1,571 (43.5%)
(Missing)	6 (0.1%)	5 (0.1%)
Anxiety symptoms composite, n (%)	1,567 (29.4%)	1,058 (29.4%)
(Missing)	33 (0.6%)	21 (0.6%)
Anxiety – feel on edge, n (%)		
Nearly every day	600 (11.2%)	393 (10.9%)
More than half the days	769 (14.3%)	544 (15.0%)
Several days	1,828 (34.1%)	1,200 (33.2%)
Not at all	2,147 (40.0%)	1,464 (40.5%)
(Missing)	24 (0.5%)	14 (0.4%)
Anxiety – cannot stop worrying, n (%)		
Nearly every day	628 (11.7%)	395 (10.9%)
More than half the days	747 (13.9%)	491 (13.6%)
Several days	1,541 (28.7%)	1,066 (29.5%)
Not at all	2,444 (45.5%)	1,657 (45.8%)
(Missing)	9 (0.2%)	7 (0.2%)
Suffering, n (%)		
A lot	780 (14.5%)	487 (13.5%)
Some	1,945 (36.2%)	1,358 (37.6%)
Not very much	1,662 (31.0%)	1,104 (30.5%)
Not at all	964 (18.0%)	655 (18.1%)
(Missing)	17 (0.3%)	11 (0.3%)
Relationship contentment		
Mean	7.3	7.2
Standard Deviation	2.3	2.3
Min, Max	0.0, 10.0	0.0, 10.0
(Missing)	31 (0.6%)	22 (0.6%)
Relationship satisfaction		
Mean	7.0	6.9
Standard Deviation	2.5	2.6
Min, Max	0.0, 10.0	0.0, 10.0
(Missing)	29 (0.5%)	9 (0.2%)

Table S30b. Weighted summary statistics for outcome variables in United Kingdom

Outcome	Wave 1<br>N = 5,368	Wave 2<br>N = 3,615
Social support		
Mean	7.7	7.7
Standard Deviation	2.5	2.4
Min, Max	0.0, 10.0	0.0, 10.0
(Missing)	17 (0.3%)	36 (1.0%)
Intimate/close friend, n (%)		
Yes	4,405 (82.1%)	2,915 (80.6%)
No	940 (17.5%)	678 (18.8%)
(Missing)	23 (0.4%)	22 (0.6%)
Government approval, n (%)		
Strongly approve	230 (4.3%)	127 (3.5%)
Somewhat approve	843 (15.7%)	499 (13.8%)
Neither approve nor disapprove	1,173 (21.8%)	756 (20.9%)
Somewhat disapprove	1,290 (24.0%)	967 (26.7%)
Strongly disapprove	1,821 (33.9%)	1,250 (34.6%)
(Missing)	12 (0.2%)	16 (0.4%)
Say in government, n (%)		
Agree	1,241 (23.1%)	952 (26.3%)
Disagree	3,127 (58.2%)	1,943 (53.7%)
Unsure	991 (18.5%)	717 (19.8%)
(Missing)	9 (0.2%)	3 (0.1%)
Belonging in country		
Mean	7.1	6.9
Standard Deviation	2.5	2.6
Min, Max	0.0, 10.0	0.0, 10.0
(Missing)	21 (0.4%)	27 (0.8%)
City/place satisfaction, n (%)		
Satisfied	3,751 (69.9%)	2,497 (69.1%)
Dissatisfied	1,014 (18.9%)	723 (20.0%)
Unsure	566 (10.5%)	371 (10.3%)
(Missing)	38 (0.7%)	23 (0.6%)
Trust within country, n (%)		
All people	108 (2.0%)	58 (1.6%)
Most people	972 (18.1%)	711 (19.7%)
Some people	2,476 (46.1%)	1,538 (42.5%)
Not very many people	1,687 (31.4%)	1,253 (34.7%)
None	104 (1.9%)	42 (1.2%)
(Missing)	21 (0.4%)	13 (0.4%)
Number of children		
Mean	0.6	0.4
Standard Deviation	1.0	1.1
Min, Max	0.0, 10.0	0.0, 54.0
(Missing)	17 (0.3%)	449 (12%)
Community participation, n (%)		
More than once a week	551 (10.3%)	349 (9.7%)
Once a week	616 (11.5%)	460 (12.7%)
One to three times a month	654 (12.2%)	394 (10.9%)
A few times a year	991 (18.5%)	653 (18.1%)
Never	2,550 (47.5%)	1,744 (48.3%)
(Missing)	7 (0.1%)	13 (0.4%)
Religious attendance, n (%)		

Table S30b. Weighted summary statistics for outcome variables in United Kingdom

**Outcome**	**Wave 1** N = 5,368	**Wave 2** N = 3,615
More than once a week	287 (5.3%)	186 (5.1%)
Once a week	493 (9.2%)	316 (8.7%)
One to three times a month	295 (5.5%)	204 (5.6%)
A few times a year	1,147 (21.4%)	788 (21.8%)
Never	3,135 (58.4%)	2,111 (58.4%)
(Missing)	10 (0.2%)	11 (0.3%)
Loneliness		
Mean	3.6	3.7
Standard Deviation	2.9	2.9
Min, Max	0.0, 10.0	0.0, 10.0
(Missing)	7 (0.1%)	4 (0.1%)
Perceived discrimination, n (%)		
Always	278 (5.2%)	155 (4.3%)
Often	942 (17.5%)	622 (17.2%)
Rarely	2,174 (40.5%)	1,451 (40.2%)
Never	1,963 (36.6%)	1,376 (38.1%)
(Missing)	12 (0.2%)	10 (0.3%)
Orientation to promote good		
Mean	7.6	7.5
Standard Deviation	1.8	1.8
Min, Max	0.0, 10.0	0.0, 10.0
(Missing)	19 (0.3%)	24 (0.7%)
Delayed gratification		
Mean	6.9	6.9
Standard Deviation	2.0	2.0
Min, Max	0.0, 10.0	0.0, 10.0
(Missing)	28 (0.5%)	15 (0.4%)
Hope		
Mean	7.4	7.3
Standard Deviation	2.3	2.2
Min, Max	0.0, 10.0	0.0, 10.0
(Missing)	111 (2.1%)	37 (1.0%)
Gratitude		
Mean	7.4	7.3
Standard Deviation	2.4	2.5
Min, Max	0.0, 10.0	0.0, 10.0
(Missing)	115 (2.1%)	40 (1.1%)
Showing love/care		
Mean	8.1	8.0
Standard Deviation	2.0	2.0
Min, Max	0.0, 10.0	0.0, 10.0
(Missing)	11 (0.2%)	18 (0.5%)
Forgivingness, n (%)		
Always	910 (17.0%)	593 (16.4%)
Often	2,900 (54.0%)	1,900 (52.6%)
Rarely	1,325 (24.7%)	938 (25.9%)
Never	222 (4.1%)	180 (5.0%)
(Missing)	10 (0.2%)	4 (0.1%)
Charitable giving, n (%)		
Yes	3,204 (59.7%)	2,035 (56.3%)
No	2,159 (40.2%)	1,547 (42.8%)

Table S30b. Weighted summary statistics for outcome variables in United Kingdom

Outcome	Wave 1<br>N = 5,368	Wave 2<br>N = 3,615
(Missing)	6 (0.1%)	33 (0.9%)
Helping strangers, n (%)		
Yes	2,969 (55.3%)	1,726 (47.7%)
No	2,386 (44.4%)	1,874 (51.8%)
(Missing)	13 (0.2%)	15 (0.4%)
Volunteering, n (%)		
Yes	1,561 (29.1%)	935 (25.9%)
No	3,796 (70.7%)	2,667 (73.8%)
(Missing)	10 (0.2%)	12 (0.3%)
Self-rated physical health		
Mean	6.4	6.2
Standard Deviation	2.3	2.3
Min, Max	0.0, 10.0	0.0, 10.0
(Missing)	4 (<0.1%)	57 (1.6%)
Health problems, n (%)		
Yes	1,623 (30.2%)	1,165 (32.2%)
No	3,702 (69.0%)	2,430 (67.2%)
(Missing)	43 (0.8%)	21 (0.6%)
Pain in past 4 weeks, n (%)		
A lot	959 (17.9%)	629 (17.4%)
Some	1,860 (34.7%)	1,287 (35.6%)
Not very much	1,584 (29.5%)	1,090 (30.2%)
None at all	948 (17.7%)	597 (16.5%)
(Missing)	18 (0.3%)	12 (0.3%)
Number of cigarettes per day		
Mean	1.8	1.7
Standard Deviation	5.2	5.2
Min, Max	0.0, 70.0	0.0, 80.0
(Missing)	54 (1.0%)	98 (2.7%)
Number of drinks per week		
Mean	4.2	4.1
Standard Deviation	8.6	8.8
Min, Max	0.0, 97.0	0.0, 97.0
(Missing)	37 (0.7%)	73 (2.0%)
Days exercise per week		
Mean	2.5	2.5
Standard Deviation	2.4	2.3
Min, Max	0.0, 7.0	0.0, 7.0
(Missing)	8 (0.2%)	3 (<0.1%)
Financial security		
Mean	6.0	6.1
Standard Deviation	3.0	3.1
Min, Max	0.0, 10.0	0.0, 10.0
(Missing)	8 (0.2%)	5 (0.1%)
Material security		
Mean	6.6	6.7
Standard Deviation	2.8	2.9
Min, Max	0.0, 10.0	0.0, 10.0
(Missing)	28 (0.5%)	17 (0.5%)
Educational attainment (16+ years), n (%)		
Up to 8	1,279 (23.8%)	579 (16.0%)

Table S30b. Weighted summary statistics for outcome variables in United Kingdom

Outcome	Wave 1<br>N = 5,368	Wave 2<br>N = 3,615
9-15	2,125 (39.6%)	1,474 (40.8%)
16+	1,956 (36.4%)	1,562 (43.2%)
(Missing)	8 (0.2%)	0 (0%)
Currently employed, n (%)		
Employed for an employer	2,840 (52.9%)	1,844 (51.0%)
Self-employed	460 (8.6%)	355 (9.8%)
Retired	1,211 (22.6%)	862 (23.9%)
Student	257 (4.8%)	108 (3.0%)
Homemaker	181 (3.4%)	130 (3.6%)
Unemployed and looking for a job	207 (3.9%)	131 (3.6%)
None of these/Other	203 (3.8%)	154 (4.3%)
(Missing)	9 (0.2%)	32 (0.9%)
Financially comfortable/getting by, n (%)		
Living comfortably on present income	1,760 (32.8%)	1,286 (35.6%)
Getting by on present income	2,335 (43.5%)	1,559 (43.1%)
Finding it difficult on present income	892 (16.6%)	530 (14.7%)
Finding it very difficult on present income	327 (6.1%)	220 (6.1%)
(Missing)	53 (1.0%)	20 (0.5%)
Own home, n (%)		
Someone in this household owns this home	3,089 (57.5%)	2,117 (58.6%)
Someone in this household rents this home	1,545 (28.8%)	1,003 (27.7%)
Both	274 (5.1%)	167 (4.6%)
Neither	403 (7.5%)	296 (8.2%)
Rent	0 (0%)	0 (0%)
Own	0 (0%)	0 (0%)
Something else	0 (0%)	0 (0%)
(Missing)	56 (1.0%)	32 (0.9%)
Religious/spiritual connection, n (%)		
Always	903 (16.8%)	639 (17.7%)
Often	1,252 (23.3%)	754 (20.9%)
Rarely	1,499 (27.9%)	936 (25.9%)
Never	1,696 (31.6%)	1,281 (35.4%)
(Missing)	18 (0.3%)	5 (0.1%)
Belief in life after death, n (%)		
Yes	2,062 (38.4%)	1,283 (35.5%)
No	1,510 (28.1%)	1,103 (30.5%)
Unsure	1,748 (32.6%)	1,203 (33.3%)
(Missing)	49 (0.9%)	26 (0.7%)
Transformative religious experience, n (%)		
Yes	1,148 (21.4%)	686 (19.0%)
No	4,194 (78.1%)	2,906 (80.4%)
(Missing)	26 (0.5%)	23 (0.6%)
Religious reading or listening, n (%)		
More than once a day	218 (4.1%)	158 (4.4%)
About once a day	481 (9.0%)	271 (7.5%)
Sometimes	1,391 (25.9%)	783 (21.7%)
Never	3,250 (60.6%)	2,371 (65.6%)
(Missing)	27 (0.5%)	32 (0.9%)
Prayer or meditation, n (%)		
More than once a day	560 (10.4%)	330 (9.1%)
About once a day	701 (13.1%)	404 (11.2%)

Table S30b. Weighted summary statistics for outcome variables in United Kingdom

Outcome	Wave 1<br>N = 5,368	Wave 2<br>N = 3,615
Sometimes	1,591 (29.6%)	988 (27.3%)
Never	2,496 (46.5%)	1,867 (51.7%)
(Missing)	20 (0.4%)	25 (0.7%)
Belief in God/gods/spiritual forces, n (%)		
One God	1,990 (37.1%)	1,297 (35.9%)
More than one god	146 (2.7%)	124 (3.4%)
An impersonal spiritual force	707 (13.2%)	452 (12.5%)
None of these	1,586 (29.5%)	1,146 (31.7%)
Unsure	905 (16.9%)	583 (16.1%)
(Missing)	34 (0.6%)	14 (0.4%)
Religious centrality, n (%)		
Agree	1,471 (27.4%)	897 (24.8%)
Disagree	904 (16.8%)	582 (16.1%)
Not relevant	2,352 (43.8%)	1,712 (47.3%)
Unsure	618 (11.5%)	402 (11.1%)
(Missing)	24 (0.4%)	22 (0.6%)
Religious/spiritual comfort, n (%)		
Agree	1,769 (33.0%)	1,176 (32.5%)
Disagree	699 (13.0%)	405 (11.2%)
Not relevant	2,292 (42.7%)	1,680 (46.5%)
Unsure	582 (10.8%)	312 (8.6%)
(Missing)	26 (0.5%)	43 (1.2%)
Feel loved by God, n (%)		
Agree	1,675 (31.2%)	1,050 (29.0%)
Disagree	770 (14.3%)	446 (12.3%)
Not relevant	2,204 (41.1%)	1,644 (45.5%)
Unsure	683 (12.7%)	421 (11.7%)
(Missing)	36 (0.7%)	53 (1.5%)
Feel punished by God, n (%)		
Agree	537 (10.0%)	301 (8.3%)
Disagree	2,069 (38.5%)	1,307 (36.2%)
Not relevant	2,175 (40.5%)	1,611 (44.6%)
Unsure	543 (10.1%)	350 (9.7%)
(Missing)	44 (0.8%)	46 (1.3%)
Experienced religious criticism, n (%)		
Agree	441 (8.2%)	269 (7.5%)
Disagree	1,453 (27.1%)	892 (24.7%)
Not relevant	3,014 (56.1%)	2,120 (58.6%)
Unsure	428 (8.0%)	294 (8.1%)
(Missing)	32 (0.6%)	40 (1.1%)
Faith-sharing, n (%)		
Agree	1,218 (22.7%)	741 (20.5%)
Disagree	1,257 (23.4%)	749 (20.7%)
Not relevant	2,539 (47.3%)	1,846 (51.1%)
Unsure	329 (6.1%)	261 (7.2%)
(Missing)	26 (0.5%)	18 (0.5%)

Note. N (%); this table is based on non-imputed data. Cumulative percentages for variables may not add up to 100% due to rounding. Wave 1 characteristics weighted using the Gallup provided sampling weight, ANNUAL_WEIGHT_R2; Wave 2 characteristics weighted accounting for attrition by using the adjusted Wave 1 weight, ANNUAL_WEIGHT_R2, multiplied by the created attrition weight to account for dropout, to maintain nationally representative estimates for Wave 2 characteristics.

Table S30c. Unweighted summary statistics for demographic and childhood variables in United Kingdom by retention status

Characteristic	Attritors–Not Observed in Wave 2<br>N = 1,715	Retained–Observed in Wave 2<br>N = 3,541
Forgivingness, n (%)		
Always	344 (20.1%)	535 (15.1%)
Often	921 (53.7%)	1,920 (54.2%)
Rarely	384 (22.4%)	923 (26.1%)
Never	62 (3.6%)	158 (4.5%)
(Missing)	5 (0.3%)	5 (0.1%)
Year of birth, n (%)		
1943 or earlier (current age: 80+ years)	42 (2.5%)	107 (3.0%)
1943-1953 (current age: 70-79 years)	178 (10.4%)	492 (13.9%)
1953-1963 (current age: 60-69 years)	206 (12.0%)	622 (17.6%)
1963-1973 (current age: 50-59 years)	195 (11.4%)	759 (21.4%)
1973-1983 (current age: 40-49 years)	223 (13.0%)	592 (16.7%)
1983-1993 (current age: 30-39 years)	384 (22.4%)	546 (15.4%)
1993-1998 (current age: 25-29 years)	192 (11.2%)	219 (6.2%)
1998-2005 (current age: 18-24 years)	295 (17.2%)	205 (5.8%)
(Missing)	0 (0.0%)	1 (0.0%)
Age of participant		
Mean	43.6	51.3
Standard Deviation	18.8	16.7
Min, Max	18.0, 95.0	18.0, 92.0
Gender, n (%)		
Male	849 (49.5%)	1,677 (47.4%)
Female	857 (49.9%)	1,852 (52.3%)
Other	8 (0.4%)	6 (0.2%)
(Missing)	2 (0.1%)	6 (0.2%)
Respondent marital status, n (%)		
Single/Never been married	575 (33.5%)	889 (25.1%)
Married	745 (43.4%)	1,687 (47.6%)
Separated	43 (2.5%)	66 (1.9%)
Divorced	96 (5.6%)	320 (9.0%)
Widowed	71 (4.2%)	204 (5.8%)
Domestic partner	165 (9.6%)	348 (9.8%)
(Missing)	20 (1.2%)	27 (0.8%)
Education (years), n (%)		
Up to 8	409 (23.9%)	842 (23.8%)
9-15	675 (39.3%)	1,408 (39.7%)
16+	631 (36.8%)	1,283 (36.2%)
(Missing)	0 (0%)	9 (0.2%)
Employment status, n (%)		
Employed for an employer	929 (54.2%)	1,847 (52.2%)
Self-employed	139 (8.1%)	312 (8.8%)
Retired	325 (18.9%)	874 (24.7%)
Student	135 (7.8%)	105 (3.0%)
Homemaker	43 (2.5%)	138 (3.9%)
Unemployed and looking for a job	68 (4.0%)	134 (3.8%)
None of these/Other	72 (4.2%)	126 (3.6%)
(Missing)	5 (0.3%)	4 (0.1%)
Current religious service attendance, n (%)		
More than once a week	128 (7.5%)	144 (4.1%)

Table S30c. Unweighted summary statistics for demographic and childhood variables in United Kingdom by retention status

Characteristic	Attritors–Not Observed in Wave 2<br>N = 1,715	Retained–Observed in Wave 2<br>N = 3,541
Once a week	193 (11.3%)	282 (8.0%)
One to three times a month	84 (4.9%)	207 (5.8%)
A few times a year	393 (22.9%)	725 (20.5%)
Never	915 (53.3%)	2,175 (61.4%)
(Missing)	2 (0.1%)	8 (0.2%)
Immigration status, n (%)		
Born in this country	1,456 (84.9%)	3,113 (87.9%)
Born in another country	243 (14.1%)	422 (11.9%)
(Missing)	17 (1.0%)	6 (0.2%)
Parental marital status around age 12, n (%)		
Parents were married	1,326 (77.3%)	2,906 (82.1%)
Parents were divorced	152 (8.8%)	334 (9.4%)
Parents were never married	157 (9.2%)	150 (4.2%)
One or both of them had died	45 (2.6%)	114 (3.2%)
Unsure	12 (0.7%)	28 (0.8%)
(Missing)	23 (1.3%)	8 (0.2%)
Religious service attendance around age 12, n (%)		
At least once a week	560 (32.7%)	1,096 (31.0%)
One to three times a month	270 (15.8%)	443 (12.5%)
Less than once a month	293 (17.1%)	593 (16.7%)
Never	582 (34.0%)	1,391 (39.3%)
(Missing)	9 (0.5%)	19 (0.5%)
Relationship with mother when growing up, n (%)		
Very good	1,127 (65.7%)	2,184 (61.7%)
Somewhat good	388 (22.6%)	958 (27.0%)
Somewhat bad	99 (5.8%)	229 (6.5%)
Very bad	52 (3.0%)	94 (2.6%)
(Does not apply)	35 (2.1%)	67 (1.9%)
(Missing)	14 (0.8%)	10 (0.3%)
Relationship with father when growing up, n (%)		
Very good	891 (52.0%)	1,881 (53.1%)
Somewhat good	438 (25.6%)	946 (26.7%)
Somewhat bad	127 (7.4%)	292 (8.3%)
Very bad	127 (7.4%)	194 (5.5%)
(Does not apply)	120 (7.0%)	212 (6.0%)
(Missing)	12 (0.7%)	16 (0.4%)
Felt like an outsider in family when growing up, n (%)		
Yes	406 (23.7%)	613 (17.3%)
No	1,295 (75.5%)	2,897 (81.8%)
(Missing)	14 (0.8%)	30 (0.9%)
Experienced abuse when growing up, n (%)		
Yes	292 (17.0%)	565 (16.0%)
No	1,397 (81.5%)	2,959 (83.6%)
(Missing)	26 (1.5%)	17 (0.5%)
Self-rated health when growing up, n (%)		
Excellent	654 (38.1%)	1,429 (40.3%)
Very good	612 (35.7%)	1,093 (30.9%)

Table S30c. Unweighted summary statistics for demographic and childhood variables in United Kingdom by retention status

**Characteristic**	**Attritors–Not Observed in Wave 2** N = 1,715	**Retained–Observed in Wave 2** N = 3,541
Good	309 (18.0%)	675 (19.1%)
Fair	93 (5.4%)	241 (6.8%)
Poor	43 (2.5%)	88 (2.5%)
(Missing)	4 (0.2%)	16 (0.4%)
Subjective financial status of family growing up, n (%)		
Lived comfortably	883 (51.5%)	1,550 (43.8%)
Got by	563 (32.8%)	1,364 (38.5%)
Found it difficult	204 (11.9%)	441 (12.4%)
Found it very difficult	59 (3.4%)	170 (4.8%)
(Missing)	7 (0.4%)	17 (0.5%)
Religious affiliation growing up, n (%)		
Christianity	1,040 (60.6%)	2,322 (65.6%)
Taoism	1 (0.1%)	0 (0%)
Confucianism	2 (0.1%)	1 (0.0%)
Primal, Animist, or Folk religion	9 (0.6%)	9 (0.3%)
Spiritism	0 (0%)	0 (0%)
Umbanda, Candomblé, and other African-derived religions	0 (0%)	0 (0%)
Chinese folk/traditional religion	0 (0%)	0 (0%)
Islam	96 (5.6%)	134 (3.8%)
Hinduism	41 (2.4%)	41 (1.2%)
Buddhism	4 (0.2%)	12 (0.3%)
Judaism	13 (0.8%)	41 (1.1%)
Sikhism	15 (0.9%)	11 (0.3%)
Baha'i	0 (0.0%)	4 (0.1%)
Jainism	0 (0%)	0 (<0.0%)
Shinto	0 (0%)	0 (0%)
Some other religion	5 (0.3%)	20 (0.6%)
No religion/Atheist/Agnostic	483 (28.2%)	929 (26.2%)
(Missing)	2 (0.1%)	19 (0.5%)

Note. N (%); this table is based on non-imputed data. Cumulative percentages for variables may not add up to 100% due to rounding.

Table S30d. Unweighted summary statistics for Wave 1 outcome variables in United Kingdom by retention status.

**Outcome**	**Attritors–Not Observed in Wave 2** N = 1,715	**Retained–Observed in Wave 2** N = 3,541
Secure flourishing index		
Mean	6.7	6.8
Standard Deviation	1.6	1.7
Min, Max	0.0, 10.0	0.4, 10.0
(Missing)	67 (3.9%)	104 (2.9%)
Flourishing index		
Mean	6.8	6.9
Standard Deviation	1.7	1.7
Min, Max	0.0, 10.0	0.4, 10.0
(Missing)	49 (2.9%)	98 (2.8%)
Happiness & life satisfaction		
Mean	6.5	6.6
Standard Deviation	2.1	2.1
Min, Max	0.0, 10.0	0.0, 10.0
(Missing)	14 (0.8%)	35 (1.0%)
Physical & mental health		
Mean	6.4	6.6
Standard Deviation	2.2	2.1
Min, Max	0.0, 10.0	0.0, 10.0
(Missing)	5 (0.3%)	6 (0.2%)
Meaning & purpose		
Mean	6.7	6.8
Standard Deviation	2.2	2.3
Min, Max	0.0, 10.0	0.0, 10.0
(Missing)	10 (0.6%)	10 (0.3%)
Character & virtue		
Mean	7.3	7.2
Standard Deviation	1.7	1.7
Min, Max	0.0, 10.0	0.0, 10.0
(Missing)	13 (0.8%)	24 (0.7%)
Close social relationships		
Mean	7.1	7.1
Standard Deviation	2.3	2.3
Min, Max	0.0, 10.0	0.0, 10.0
(Missing)	21 (1.3%)	32 (0.9%)
Financial & material security		
Mean	6.2	6.4
Standard Deviation	2.7	2.8
Min, Max	0.0, 10.0	0.0, 10.0
(Missing)	21 (1.2%)	12 (0.3%)
Happiness		
Mean	6.6	6.7
Standard Deviation	2.1	2.1
Min, Max	0.0, 10.0	0.0, 10.0
(Missing)	5 (0.3%)	8 (0.2%)
Life satisfaction		
Mean	6.4	6.5
Standard Deviation	2.4	2.3
Min, Max	0.0, 10.0	0.0, 10.0

Table S30d. Unweighted summary statistics for Wave 1 outcome variables in United Kingdom by retention status.

**Outcome**	**Attritors–Not Observed in Wave 2** N = 1,715	**Retained–Observed in Wave 2** N = 3,541
(Missing)	10 (0.6%)	27 (0.8%)
Current life evaluation		
Mean	6.5	6.5
Standard Deviation	2.0	2.0
Min, Max	0.0, 10.0	0.0, 10.0
(Missing)	8 (0.5%)	5 (0.1%)
Future life evaluation		
Mean	7.3	7.0
Standard Deviation	2.2	2.1
Min, Max	0.0, 10.0	0.0, 10.0
(Missing)	7 (0.4%)	5 (0.1%)
Optimism		
Mean	7.0	6.9
Standard Deviation	2.5	2.5
Min, Max	0.0, 10.0	0.0, 10.0
(Missing)	16 (0.9%)	17 (0.5%)
Freedom to pursue what's important		
Mean	7.0	7.0
Standard Deviation	2.5	2.4
Min, Max	0.0, 10.0	0.0, 10.0
(Missing)	9 (0.5%)	6 (0.2%)
Inner peace, n (%)		
Always	236 (13.8%)	483 (13.6%)
Often	936 (54.6%)	2,092 (59.1%)
Rarely	470 (27.4%)	846 (23.9%)
Never	68 (3.9%)	107 (3.0%)
(Missing)	5 (0.3%)	14 (0.4%)
Life balance, n (%)		
Always	187 (10.9%)	320 (9.0%)
Often	949 (55.4%)	2,172 (61.3%)
Rarely	513 (29.9%)	941 (26.6%)
Never	54 (3.2%)	98 (2.8%)
(Missing)	11 (0.7%)	10 (0.3%)
Sense of mastery, n (%)		
Always	270 (15.8%)	484 (13.7%)
Often	1,081 (63.0%)	2,357 (66.6%)
Rarely	322 (18.8%)	645 (18.2%)
Never	31 (1.8%)	46 (1.3%)
(Missing)	11 (0.6%)	9 (0.3%)
Meaningful activities		
Mean	6.8	6.9
Standard Deviation	2.3	2.3
Min, Max	0.0, 10.0	0.0, 10.0
(Missing)	6 (0.3%)	4 (0.1%)
Understanding purpose		
Mean	6.6	6.6
Standard Deviation	2.7	2.7
Min, Max	0.0, 10.0	0.0, 10.0
(Missing)	5 (0.3%)	6 (0.2%)

Table S30d. Unweighted summary statistics for Wave 1 outcome variables in United Kingdom by retention status.

**Outcome**	**Attritors–Not Observed in Wave 2** N = 1,715	**Retained–Observed in Wave 2** N = 3,541
Self-rated mental health		
Mean	6.5	6.9
Standard Deviation	2.5	2.5
Min, Max	0.0, 10.0	0.0, 10.0
(Missing)	5 (0.3%)	4 (0.1%)
Traumatic distress, n (%)		
A lot	209 (12.2%)	249 (7.0%)
Some	482 (28.1%)	779 (22.0%)
Not very much	534 (31.1%)	1,064 (30.1%)
Not at all	479 (27.9%)	1,437 (40.6%)
(Missing)	12 (0.7%)	12 (0.3%)
Depression symptoms composite, n (%)	640 (37.5%)	894 (25.4%)
(Missing)	6 (0.4%)	17 (0.5%)
Depression – feel hopeless, n (%)		
Nearly every day	195 (11.4%)	288 (8.1%)
More than half the days	309 (18.0%)	436 (12.3%)
Several days	549 (32.0%)	1,076 (30.4%)
Not at all	656 (38.3%)	1,730 (48.9%)
(Missing)	6 (0.4%)	12 (0.3%)
Depression – loss of interest, n (%)		
Nearly every day	196 (11.4%)	309 (8.7%)
More than half the days	424 (24.7%)	496 (14.0%)
Several days	524 (30.6%)	1,035 (29.2%)
Not at all	571 (33.3%)	1,695 (47.9%)
(Missing)	0 (0%)	6 (0.2%)
Anxiety symptoms composite, n (%)	627 (36.8%)	880 (25.0%)
(Missing)	13 (0.7%)	19 (0.5%)
Anxiety – feel on edge, n (%)		
Nearly every day	234 (13.6%)	344 (9.7%)
More than half the days	310 (18.1%)	429 (12.1%)
Several days	592 (34.5%)	1,196 (33.8%)
Not at all	571 (33.3%)	1,558 (44.0%)
(Missing)	9 (0.5%)	15 (0.4%)
Anxiety – cannot stop worrying, n (%)		
Nearly every day	255 (14.9%)	348 (9.8%)
More than half the days	288 (16.8%)	432 (12.2%)
Several days	524 (30.6%)	977 (27.6%)
Not at all	645 (37.6%)	1,779 (50.2%)
(Missing)	4 (0.2%)	5 (0.1%)
Suffering, n (%)		
A lot	315 (18.3%)	434 (12.3%)
Some	627 (36.6%)	1,276 (36.0%)
Not very much	526 (30.7%)	1,102 (31.1%)
Not at all	239 (14.0%)	720 (20.3%)
(Missing)	8 (0.5%)	8 (0.2%)
Relationship contentment		
Mean	7.3	7.3
Standard Deviation	2.4	2.3
Min, Max	0.0, 10.0	0.0, 10.0

Table S30d. Unweighted summary statistics for Wave 1 outcome variables in United Kingdom by retention status.

**Outcome**	**Attritors–Not Observed in Wave 2** N = 1,715	**Retained–Observed in Wave 2** N = 3,541
(Missing)	11 (0.6%)	20 (0.6%)
Relationship satisfaction		
Mean	6.9	7.0
Standard Deviation	2.5	2.5
Min, Max	0.0, 10.0	0.0, 10.0
(Missing)	13 (0.7%)	15 (0.4%)
Social support		
Mean	7.6	7.7
Standard Deviation	2.5	2.5
Min, Max	0.0, 10.0	0.0, 10.0
(Missing)	9 (0.5%)	7 (0.2%)
Intimate/close friend, n (%)		
Yes	1,404 (81.8%)	2,910 (82.2%)
No	297 (17.3%)	625 (17.6%)
(Missing)	15 (0.9%)	6 (0.2%)
Government approval, n (%)		
Strongly approve	115 (6.7%)	101 (2.9%)
Somewhat approve	255 (14.8%)	574 (16.2%)
Neither approve nor disapprove	419 (24.4%)	720 (20.3%)
Somewhat disapprove	353 (20.6%)	923 (26.1%)
Strongly disapprove	573 (33.4%)	1,212 (34.2%)
(Missing)	1 (0.1%)	11 (0.3%)
Say in government, n (%)		
Agree	408 (23.8%)	805 (22.7%)
Disagree	994 (58.0%)	2,068 (58.4%)
Unsure	310 (18.1%)	663 (18.7%)
(Missing)	3 (0.2%)	5 (0.2%)
Belonging in country		
Mean	7.0	7.1
Standard Deviation	2.5	2.5
Min, Max	0.0, 10.0	0.0, 10.0
(Missing)	7 (0.4%)	13 (0.4%)
City/place satisfaction, n (%)		
Satisfied	1,199 (69.9%)	2,473 (69.8%)
Dissatisfied	315 (18.4%)	679 (19.2%)
Unsure	186 (10.8%)	367 (10.4%)
(Missing)	15 (0.9%)	22 (0.6%)
Trust within country, n (%)		
All people	59 (3.5%)	41 (1.2%)
Most people	283 (16.5%)	675 (19.1%)
Some people	738 (43.0%)	1,698 (48.0%)
Not very many people	589 (34.3%)	1,052 (29.7%)
None	40 (2.3%)	61 (1.7%)
(Missing)	6 (0.4%)	14 (0.4%)
Number of children		
Mean	0.7	0.5
Standard Deviation	1.1	1.0
Min, Max	0.0, 9.0	0.0, 10.0
(Missing)	10 (0.6%)	6 (0.2%)

Table S30d. Unweighted summary statistics for Wave 1 outcome variables in United Kingdom by retention status.

Outcome	Attritors–Not Observed in Wave 2 N = 1,715	Retained–Observed in Wave 2 N = 3,541
Community participation, n (%)		
More than once a week	205 (11.9%)	328 (9.3%)
Once a week	207 (12.1%)	394 (11.1%)
One to three times a month	226 (13.2%)	410 (11.6%)
A few times a year	334 (19.5%)	632 (17.9%)
Never	740 (43.2%)	1,773 (50.1%)
(Missing)	3 (0.2%)	4 (0.1%)
Religious attendance, n (%)		
More than once a week	128 (7.5%)	144 (4.1%)
Once a week	193 (11.3%)	282 (8.0%)
One to three times a month	84 (4.9%)	207 (5.8%)
A few times a year	393 (22.9%)	725 (20.5%)
Never	915 (53.3%)	2,175 (61.4%)
(Missing)	2 (0.1%)	8 (0.2%)
Loneliness		
Mean	3.7	3.5
Standard Deviation	2.9	2.9
Min, Max	0.0, 10.0	0.0, 10.0
(Missing)	3 (0.2%)	4 (0.1%)
Perceived discrimination, n (%)		
Always	128 (7.4%)	136 (3.8%)
Often	344 (20.0%)	569 (16.1%)
Rarely	680 (39.7%)	1,451 (41.0%)
Never	559 (32.6%)	1,379 (38.9%)
(Missing)	5 (0.3%)	7 (0.2%)
Orientation to promote good		
Mean	7.6	7.6
Standard Deviation	1.9	1.8
Min, Max	0.0, 10.0	0.0, 10.0
(Missing)	6 (0.4%)	12 (0.3%)
Delayed gratification		
Mean	7.0	6.9
Standard Deviation	2.1	2.0
Min, Max	0.0, 10.0	0.0, 10.0
(Missing)	11 (0.6%)	17 (0.5%)
Hope		
Mean	7.5	7.3
Standard Deviation	2.2	2.3
Min, Max	0.0, 10.0	0.0, 10.0
(Missing)	43 (2.5%)	64 (1.8%)
Gratitude		
Mean	7.5	7.3
Standard Deviation	2.3	2.4
Min, Max	0.0, 10.0	0.0, 10.0
(Missing)	53 (3.1%)	56 (1.6%)
Showing love/care		
Mean	8.1	8.1
Standard Deviation	2.0	2.0
Min, Max	0.0, 10.0	0.0, 10.0

Table S30d. Unweighted summary statistics for Wave 1 outcome variables in United Kingdom by retention status.

**Outcome**	**Attritors–Not Observed in Wave 2** N = 1,715	**Retained–Observed in Wave 2** N = 3,541
(Missing)	8 (0.5%)	1 (<0.1%)
Forgivingness, n (%)		
Always	344 (20.1%)	535 (15.1%)
Often	921 (53.7%)	1,920 (54.2%)
Rarely	384 (22.4%)	923 (26.1%)
Never	62 (3.6%)	158 (4.5%)
(Missing)	5 (0.3%)	5 (0.1%)
Charitable giving, n (%)		
Yes	1,035 (60.3%)	2,100 (59.3%)
No	678 (39.6%)	1,438 (40.6%)
(Missing)	2 (0.1%)	3 (0.1%)
Helping strangers, n (%)		
Yes	1,028 (60.0%)	1,861 (52.5%)
No	683 (39.8%)	1,671 (47.2%)
(Missing)	4 (0.2%)	10 (0.3%)
Volunteering, n (%)		
Yes	533 (31.1%)	988 (27.9%)
No	1,179 (68.8%)	2,545 (71.9%)
(Missing)	3 (0.1%)	8 (0.2%)
Self-rated physical health		
Mean	6.3	6.4
Standard Deviation	2.4	2.2
Min, Max	0.0, 10.0	0.0, 10.0
(Missing)	2 (0.1%)	2 (<0.1%)
Health problems, n (%)		
Yes	515 (30.0%)	1,075 (30.4%)
No	1,178 (68.7%)	2,448 (69.1%)
(Missing)	22 (1.3%)	18 (0.5%)
Pain in past 4 weeks, n (%)		
A lot	336 (19.6%)	596 (16.8%)
Some	624 (36.4%)	1,190 (33.6%)
Not very much	476 (27.7%)	1,082 (30.6%)
None at all	269 (15.7%)	667 (18.8%)
(Missing)	10 (0.6%)	6 (0.2%)
Number of cigarettes per day		
Mean	1.7	1.9
Standard Deviation	4.9	5.3
Min, Max	0.0, 70.0	0.0, 46.0
(Missing)	25 (1.5%)	26 (0.7%)
Number of drinks per week		
Mean	4.2	4.1
Standard Deviation	9.4	8.1
Min, Max	0.0, 97.0	0.0, 97.0
(Missing)	20 (1.2%)	14 (0.4%)
Days exercise per week		
Mean	2.5	2.6
Standard Deviation	2.3	2.4
Min, Max	0.0, 7.0	0.0, 7.0
(Missing)	5 (0.3%)	2 (<0.1%)

Table S30d. Unweighted summary statistics for Wave 1 outcome variables in United Kingdom by retention status.

**Outcome**	**Attritors–Not Observed in Wave 2** N = 1,715	**Retained–Observed in Wave 2** N = 3,541
Financial security		
Mean	6.0	6.1
Standard Deviation	2.9	3.1
Min, Max	0.0, 10.0	0.0, 10.0
(Missing)	5 (0.3%)	3 (<0.1%)
Material security		
Mean	6.4	6.7
Standard Deviation	2.9	2.8
Min, Max	0.0, 10.0	0.0, 10.0
(Missing)	17 (1.0%)	9 (0.3%)
Educational attainment (16+ years), n (%)		
Up to 8	409 (23.9%)	842 (23.8%)
9-15	675 (39.3%)	1,408 (39.7%)
16+	631 (36.8%)	1,283 (36.2%)
(Missing)	0 (0%)	9 (0.2%)
Currently employed, n (%)		
Employed for an employer	929 (54.2%)	1,847 (52.2%)
Self-employed	139 (8.1%)	312 (8.8%)
Retired	325 (18.9%)	874 (24.7%)
Student	135 (7.8%)	105 (3.0%)
Homemaker	43 (2.5%)	138 (3.9%)
Unemployed and looking for a job	68 (4.0%)	134 (3.8%)
None of these/Other	72 (4.2%)	126 (3.6%)
(Missing)	5 (0.3%)	4 (0.1%)
Financially comfortable/getting by, n (%)		
Living comfortably on present income	566 (33.0%)	1,157 (32.7%)
Getting by on present income	738 (43.0%)	1,551 (43.8%)
Finding it difficult on present income	285 (16.6%)	589 (16.6%)
Finding it very difficult on present income	97 (5.6%)	226 (6.4%)
(Missing)	30 (1.8%)	19 (0.5%)
Own home, n (%)		
Someone in this household owns this home	921 (53.7%)	2,119 (59.8%)
Someone in this household rents this home	527 (30.7%)	978 (27.6%)
Both	109 (6.3%)	155 (4.4%)
Neither	126 (7.3%)	270 (7.6%)
Rent	0 (0%)	0 (0%)
Own	0 (0%)	0 (0%)
Something else	0 (0%)	0 (0%)
(Missing)	32 (1.9%)	19 (0.5%)
Religious/spiritual connection, n (%)		
Always	350 (20.4%)	521 (14.7%)
Often	402 (23.4%)	824 (23.3%)
Rarely	458 (26.7%)	1,015 (28.6%)
Never	498 (29.0%)	1,173 (33.1%)
(Missing)	8 (0.5%)	9 (0.2%)
Belief in life after death, n (%)		
Yes	724 (42.2%)	1,280 (36.1%)
No	418 (24.4%)	1,074 (30.3%)
Unsure	547 (31.9%)	1,167 (33.0%)

Table S30d. Unweighted summary statistics for Wave 1 outcome variables in United Kingdom by retention status.

Outcome	Attritors–Not Observed in Wave 2<br>N = 1,715	Retained–Observed in Wave 2<br>N = 3,541
(Missing)	26 (1.5%)	19 (0.6%)
Transformative religious experience, n (%)		
Yes	460 (26.8%)	643 (18.2%)
No	1,241 (72.3%)	2,889 (81.6%)
(Missing)	15 (0.9%)	9 (0.3%)
Religious reading or listening, n (%)		
More than once a day	102 (6.0%)	104 (2.9%)
About once a day	181 (10.5%)	285 (8.0%)
Sometimes	471 (27.5%)	885 (25.0%)
Never	951 (55.4%)	2,252 (63.6%)
(Missing)	10 (0.6%)	16 (0.5%)
Prayer or meditation, n (%)		
More than once a day	226 (13.2%)	312 (8.8%)
About once a day	235 (13.7%)	449 (12.7%)
Sometimes	523 (30.5%)	1,032 (29.2%)
Never	721 (42.0%)	1,741 (49.2%)
(Missing)	11 (0.6%)	7 (0.2%)
Belief in God/gods/spiritual forces, n (%)		
One God	676 (39.4%)	1,264 (35.7%)
More than one god	50 (2.9%)	91 (2.6%)
An impersonal spiritual force	225 (13.1%)	468 (13.2%)
None of these	471 (27.5%)	1,090 (30.8%)
Unsure	278 (16.2%)	611 (17.2%)
(Missing)	15 (0.9%)	17 (0.5%)
Religious centrality, n (%)		
Agree	521 (30.3%)	908 (25.7%)
Disagree	309 (18.0%)	572 (16.1%)
Not relevant	692 (40.4%)	1,623 (45.8%)
Unsure	181 (10.6%)	428 (12.1%)
(Missing)	12 (0.7%)	10 (0.3%)
Religious/spiritual comfort, n (%)		
Agree	615 (35.9%)	1,106 (31.2%)
Disagree	231 (13.5%)	451 (12.7%)
Not relevant	677 (39.4%)	1,581 (44.6%)
Unsure	177 (10.3%)	394 (11.1%)
(Missing)	15 (0.9%)	9 (0.2%)
Feel loved by God, n (%)		
Agree	584 (34.1%)	1,045 (29.5%)
Disagree	259 (15.1%)	493 (13.9%)
Not relevant	659 (38.4%)	1,509 (42.6%)
Unsure	198 (11.5%)	475 (13.4%)
(Missing)	15 (0.9%)	19 (0.5%)
Feel punished by God, n (%)		
Agree	221 (12.9%)	293 (8.3%)
Disagree	661 (38.6%)	1,365 (38.5%)
Not relevant	637 (37.1%)	1,506 (42.5%)
Unsure	175 (10.2%)	355 (10.0%)
(Missing)	20 (1.2%)	21 (0.6%)
Experienced religious criticism, n (%)		

Table S30d. Unweighted summary statistics for Wave 1 outcome variables in United Kingdom by retention status.

Outcome	Attritors–Not Observed in Wave 2<br>N = 1,715	Retained–Observed in Wave 2<br>N = 3,541
Agree	197 (11.5%)	223 (6.3%)
Disagree	470 (27.4%)	952 (26.9%)
Not relevant	878 (51.2%)	2,093 (59.1%)
Unsure	155 (9.0%)	260 (7.3%)
(Missing)	16 (0.9%)	14 (0.4%)
Faith-sharing, n (%)		
Agree	462 (27.0%)	714 (20.2%)
Disagree	397 (23.2%)	835 (23.6%)
Not relevant	744 (43.4%)	1,756 (49.6%)
Unsure	101 (5.9%)	222 (6.3%)
(Missing)	10 (0.6%)	14 (0.4%)

Note. N (%); this table is based on non-imputed data. Cumulative percentages for variables may not add up to 100% due to rounding.

Table S30e. Summary of fitted attrition model in United Kingdom

Characteristic	Odds Ratio	95% CI	p-value
**ANNUAL_WEIGHT_R2**	0.92	0.82, 1.03	0.136
**Recruitment Survey Mode**			
CAWI	—	—	
CATI	0.88	0.73, 1.05	0.149
**Happiness & life satisfaction**	0.93	0.79, 1.09	0.361
**Physical & mental health**	1.01	0.89, 1.16	0.841
**Meaning & purpose**	1.05	0.90, 1.23	0.509
**Character & virtue**	0.96	0.86, 1.07	0.492
**Close social relationships**	0.91	0.81, 1.03	0.145
**Financial & material security**	1.04	0.94, 1.15	0.428
**Extraversion**	0.94	0.86, 1.03	0.209
**Openness to experience**	0.86	0.79, 0.95	0.002
**Agreeableness**	1.16	1.05, 1.27	0.002
**Conscientiousness**	1.06	0.97, 1.17	0.196
**Neuroticism**	1.02	0.91, 1.15	0.681
**Depression symptoms composite**	0.84	0.76, 0.94	0.002
**Anxiety symptoms composite**	0.92	0.83, 1.03	0.166
**Loneliness**	1.01	0.91, 1.13	0.837
**Days exercise per week**	1.06	0.97, 1.16	0.198
**Year of birth (age group)**			
1953-1963 (current age: 60-69 years)	—	—	
1943-1953 (current age: 70-79 years)	1.04	0.73, 1.49	0.815
1963-1973 (current age: 50-59 years)	1.22	0.88, 1.69	0.241
1983-1993 (current age: 30-39 years)	0.51	0.36, 0.71	8.47e-05
1973-1983 (current age: 40-49 years)	0.89	0.63, 1.25	0.507
1993-1998 (current age: 25-29 years)	0.40	0.26, 0.61	1.93e-05
1998-2005 (current age: 18-24 years)	0.25	0.15, 0.43	4.31e-07
1943 or earlier (current age: 80+ years)	0.92	0.54, 1.57	0.755
**Gender of respondent**			
Female	—	—	
Male	0.96	0.81, 1.15	0.674
Other	1.01	0.13, 8.07	0.993
**Marital status**			
Married	—	—	
Single/Never been married	1.16	0.91, 1.48	0.229
Domestic partner	1.00	0.74, 1.36	0.979
Divorced	1.12	0.79, 1.58	0.526
Widowed	1.19	0.79, 1.80	0.404
Separated	0.75	0.45, 1.26	0.279
**Employment status**			
Employed for an employer	—	—	
Retired	0.90	0.64, 1.26	0.529
Self-employed	1.01	0.75, 1.37	0.946
None of these/Other	0.87	0.55, 1.36	0.532
Homemaker	1.74	0.97, 3.13	0.064
Unemployed and looking for a job	1.52	0.92, 2.49	0.100
Student	1.15	0.68, 1.93	0.609
**Religious attendance**			
Never	—	—	
A few times a year	0.79	0.64, 0.98	0.032
Once a week	0.72	0.53, 0.97	0.033
More than once a week	0.54	0.37, 0.78	9.37e-04

Table S30e. Summary of fitted attrition model in United Kingdom

Characteristic	Odds Ratio	95% CI	p-value
One to three times a month	1.14	0.79, 1.64	0.484
**Educational attainment (16+ years)**			
9-15	—	—	
16+	1.12	0.93, 1.34	0.242
Up to 8	0.91	0.63, 1.31	0.596
**Born in This country**			
Born in this country	—	—	
Born in another country	1.10	0.85, 1.41	0.467
**Race plurality (prominent race/ethnic group [0] or not [1])**	0.96	0.88, 1.05	0.403
**Urbanicity**			
A small town or village	—	—	
A suburb of a large city	1.01	0.81, 1.25	0.956
A large city	0.88	0.71, 1.09	0.233
A rural area or on a farm	0.94	0.71, 1.24	0.643
**Monthly household income**			
United Kingdom: 1,501 - 2,000 pounds	—	—	
United Kingdom: 1,001 - 1,500 pounds	1.12	0.81, 1.56	0.486
United Kingdom: 3,001 - 4,000 pounds	1.37	0.98, 1.90	0.065
United Kingdom: 2,001 - 2,500 pounds	0.89	0.64, 1.25	0.506
United Kingdom: 2,501 - 3,000 pounds	1.16	0.83, 1.62	0.387
United Kingdom: 501 - 1,000 pounds	0.82	0.57, 1.17	0.271
United Kingdom: 4,001 - 5,000 pounds	1.21	0.83, 1.78	0.325
United Kingdom: More than 8,000 pounds	1.16	0.77, 1.74	0.487
United Kingdom: 5,001 - 6,000 pounds	0.81	0.53, 1.25	0.343
United Kingdom: 6,001 - 8,000 pounds	0.73	0.48, 1.10	0.136
United Kingdom: 500 pounds or less	0.96	0.56, 1.63	0.871
(None/No household income)	0.70	0.41, 1.21	0.205

Abbreviations: CI = Confidence Interval, OR = Odds Ratio

Notes. N=5368; attrition weights were estimated using the 'survey::svyglm(family=quasibinomial('logit'))' function. All continuous predictors were standardized and all categorical predictors used the most common category as the reference group. Reported p-values are based on the fitted regression model and no adjustments for multiple testing were done within this table.

Table S30f. Summary of principal components in United Kingdom

PC	Percent Explained by each PC	Cumulative Percent Explained
1	35.89	35.89
2	7.56	43.45
3	6.94	50.39
4	2.46	52.84
5	2.20	55.04
6	1.73	56.77
**7**	**1.45**	**58.23**
8	1.44	59.66
9	1.32	60.98
10	1.25	62.24
11	1.23	63.46
12	1.20	64.66
13	1.14	65.80
14	1.07	66.86
15	1.03	67.89
16	1.01	68.90
17	0.99	69.89
18	0.96	70.84
19	0.95	71.79
20	0.90	72.69

Notes. N=5368; PCA was conducted using 'survey::svyprcomp(.)' function using all available contemporaneous exposures at wave 1. All PCs were standardized prior to being used as predictors. The bolded row represented the number of retained components for analysis was 7.

Table S30g. Associations of forgivingness with adult well-being and other outcomes at Wave 2 in United Kingdom

Outcome	Model 1: Demographic and Childhood Variables as Covariates					Model 2: Demographic, Childhood, and Other Wave 1 Confounding Variables (Via Principal Components) as Covariates				
	RR	ES	95% CI	SE	p-value	RR	ES	95% CI	SE	p-value
Human Flourishing										
Secure flourishing index		0.11	(0.07,0.14)	0.02	1.2e-08***		0.02	(-0.01,0.05)	0.01	0.120
Flourishing index		0.12	(0.08,0.15)	0.02	3.97e-10***		0.02	(-0.00,0.05)	0.01	0.065
Happiness & life satisfaction		0.09	(0.05,0.13)	0.02	5.96e-06***		0.02	(-0.01,0.05)	0.02	0.276
Physical & mental health		0.07	(0.04,0.11)	0.02	1.46e-04***		0.02	(-0.01,0.05)	0.02	0.136
Meaning & purpose		0.08	(0.04,0.12)	0.02	5.74e-05***		-0.01	(-0.04,0.03)	0.02	0.753
Character & virtue		0.14	(0.09,0.18)	0.02	4.34e-10***		0.06	(0.02,0.10)	0.02	2.98e-03**
Close social relationships		0.09	(0.05,0.13)	0.02	4.69e-06***		0.01	(-0.02,0.05)	0.02	0.419
Financial & material security		0.03	(-0.01,0.07)	0.02	0.174		-0.00	(-0.03,0.03)	0.02	0.981
Psychological Well-Being										
Happiness (a)		0.09	(0.05,0.13)	0.02	2.91e-05***		0.02	(-0.02,0.05)	0.02	0.284
Life satisfaction (a)		0.08	(0.04,0.12)	0.02	1.14e-04***		0.01	(-0.02,0.05)	0.02	0.434
Current life evaluation		0.05	(0.01,0.09)	0.02	0.013*		-0.00	(-0.04,0.03)	0.02	0.897
Future life evaluation		0.05	(0.01,0.09)	0.02	0.010*		-0.01	(-0.04,0.03)	0.02	0.712
Optimism		0.10	(0.06,0.15)	0.02	3.83e-06***		0.03	(-0.01,0.07)	0.02	0.123
Freedom to pursue what's important		0.04	(0.00,0.09)	0.02	0.043*		-0.02	(-0.06,0.02)	0.02	0.384
Inner peace	1.03		(1.01,1.05)	0.01	9.82e-04**	1.01		(0.99,1.03)	0.01	0.395
Life balance	1.04		(1.02,1.06)	0.01	2.73e-04***	1.01		(0.99,1.03)	0.01	0.159
Sense of mastery	1.02		(1.00,1.04)	0.01	0.014*	1.00		(0.99,1.02)	0.01	0.719
Meaningful activities (c)		0.08	(0.04,0.13)	0.02	2.33e-04***		0.01	(-0.03,0.05)	0.02	0.748
Understanding purpose (c)		0.06	(0.02,0.11)	0.02	1.74e-03**		-0.01	(-0.05,0.02)	0.02	0.433
Self-rated mental health (b)		0.07	(0.03,0.11)	0.02	4.27e-04***		0.01	(-0.02,0.05)	0.02	0.419
Psychological Distress										
Traumatic distress	0.99		(0.97,1.01)	0.01	0.321	0.99		(0.97,1.01)	0.01	0.253
Depression symptoms composite	0.99		(0.97,1.00)	0.01	0.144	1.00		(0.98,1.02)	0.01	0.859
Depression – feel hopeless	0.98		(0.97,1.00)	0.01	0.085	1.00		(0.98,1.02)	0.01	0.858
Depression – loss of interest	0.99		(0.97,1.01)	0.01	0.152	1.00		(0.98,1.02)	0.01	0.873
Anxiety symptoms composite	0.99		(0.97,1.01)	0.01	0.412	1.00		(0.99,1.02)	0.01	0.648
Anxiety – feel on edge	0.99		(0.97,1.01)	0.01	0.391	1.00		(0.98,1.02)	0.01	0.717
Anxiety – cannot stop worrying	0.99		(0.97,1.01)	0.01	0.253	1.00		(0.98,1.02)	0.01	0.995
Suffering	0.99		(0.97,1.01)	0.01	0.543	1.00		(0.98,1.02)	0.01	0.911
Social Well-Being										
Relationship contentment (e)		0.09	(0.05,0.13)	0.02	2.97e-05***		0.02	(-0.02,0.05)	0.02	0.403
Relationship satisfaction (e)		0.08	(0.04,0.13)	0.02	3.84e-04***		0.01	(-0.03,0.05)	0.02	0.615
Social support		0.10	(0.06,0.14)	0.02	2.46e-06***		0.05	(0.01,0.09)	0.02	0.013*
Intimate/close friend	1.03		(1.01,1.05)	0.01	2.41e-03**	1.01		(0.99,1.03)	0.01	0.189
Government approval	1.01		(0.99,1.02)	0.01	0.348	1.00		(0.99,1.02)	0.01	0.918
Say in government	1.02		(1.00,1.04)	0.01	0.101	1.01		(0.99,1.02)	0.01	0.536
Belonging in country		0.04	(-0.00,0.08)	0.02	0.063		-0.01	(-0.05,0.03)	0.02	0.790
City/place satisfaction	1.03		(1.01,1.05)	0.01	0.006*	1.02		(1.00,1.04)	0.01	0.076
Trust within country	1.03		(1.01,1.06)	0.01	0.012*	1.03		(1.00,1.05)	0.01	0.034*
Social Participation										
Ever been married	1.00		(0.99,1.02)	0.01	0.579	1.00		(0.99,1.02)	0.01	0.561
Currently divorced	1.00		(0.99,1.01)	0.00	0.787	1.00		(0.99,1.01)	0.00	0.742
Number of children		0.01	(-0.03,0.04)	0.02	0.647		0.00	(-0.03,0.03)	0.02	0.953
Weekly+ community participation	1.01		(1.00,1.03)	0.01	0.099	1.01		(0.99,1.03)	0.01	0.312
Weekly+ religious attendance	1.00		(0.99,1.01)	0.00	0.743	1.00		(0.99,1.01)	0.00	0.876
Social Distress										
Loneliness		-0.05	(-0.10,-0.01)	0.02	0.018*		-0.00	(-0.04,0.04)	0.02	0.922
Perceived discrimination	0.99		(0.98,1.01)	0.01	0.551	1.00		(0.98,1.01)	0.01	0.588
Character & Prosocial Behavior										
Orientation to promote good (d)		0.14	(0.10,0.19)	0.02	6.27e-09***		0.07	(0.02,0.11)	0.02	2.51e-03**
Delayed gratification (d)		0.09	(0.05,0.14)	0.02	3.8e-05***		0.03	(-0.01,0.08)	0.02	0.115
Hope		0.09	(0.05,0.14)	0.02	3.15e-05***		0.01	(-0.03,0.05)	0.02	0.577
Gratitude		0.12	(0.07,0.16)	0.02	3.62e-08***		0.04	(0.00,0.08)	0.02	0.030*
Showing love/care		0.14	(0.09,0.18)	0.02	2.42e-08***		0.07	(0.03,0.12)	0.02	1.28e-03**
Forgivingness	1.24		(1.21,1.26)	0.01	2.22e-16***	1.23		(1.20,1.25)	0.01	2.22e-16***
Charitable giving	1.02		(1.00,1.04)	0.01	0.071	1.00		(0.98,1.03)	0.01	0.643
Helping strangers	1.03		(1.01,1.05)	0.01	3.89e-03**	1.01		(0.99,1.03)	0.01	0.162
Volunteering	1.01		(0.99,1.03)	0.01	0.166	1.01		(0.99,1.02)	0.01	0.515
Physical Health & Health Behavior										
Self-rated physical health (b)		0.05	(0.01,0.09)	0.02	0.007*		0.03	(-0.01,0.06)	0.02	0.127
Health problems	1.00		(0.98,1.02)	0.01	0.997	1.00		(0.98,1.01)	0.01	0.547
Pain in past 4 weeks	0.99		(0.97,1.01)	0.01	0.333	0.99		(0.97,1.01)	0.01	0.224
Daily smoker	1.01		(1.00,1.03)	0.01	0.061	1.01		(1.00,1.02)	0.01	0.105
Number of drinks per week		-0.01	(-0.05,0.03)	0.02	0.694		0.00	(-0.04,0.04)	0.02	0.910
Days exercise per week		0.02	(-0.02,0.07)	0.02	0.288		0.00	(-0.04,0.05)	0.02	0.867
Socioeconomic Outcomes										
Financial security (f)		0.01	(-0.03,0.05)	0.02	0.732		-0.02	(-0.05,0.02)	0.02	0.365
Material security (f)		0.04	(0.00,0.08)	0.02	0.042*		0.02	(-0.02,0.06)	0.02	0.386
Educational attainment (16+ years)	0.99		(0.98,1.00)	0.01	0.305	0.99		(0.98,1.00)	0.01	0.288
Currently employed	1.00		(0.99,1.01)	0.01	0.769	1.00		(0.99,1.01)	0.01	0.820
Financially comfortable/getting by	1.00		(0.98,1.01)	0.01	0.630	0.99		(0.97,1.01)	0.01	0.209
Own home	0.99		(0.97,1.01)	0.01	0.247	0.99		(0.97,1.01)	0.01	0.383
Income – top quintile	1.00		(0.98,1.02)	0.01	0.756	1.00		(0.98,1.02)	0.01	0.864
Religion & Spirituality										
Religious/spiritual connection	1.03		(1.01,1.05)	0.01	2.73e-03**	1.01		(0.99,1.02)	0.01	0.500
Belief in life after death	1.01		(0.99,1.03)	0.01	0.369	0.99		(0.97,1.01)	0.01	0.217
Transformative religious experience	1.02		(1.00,1.03)	0.01	0.025*	1.00		(0.99,1.02)	0.01	0.589
Religious reading or listening	1.01		(1.00,1.02)	0.01	0.086	1.00		(0.99,1.01)	0.00	0.567
Prayer or meditation	1.02		(1.01,1.04)	0.01	2.92e-04***	1.01		(1.00,1.02)	0.01	0.110
Belief in God/gods/spiritual forces	1.02		(1.00,1.03)	0.01	0.114	1.00		(0.98,1.01)	0.01	0.672
Religious centrality	1.01		(1.00,1.03)	0.01	0.090	1.00		(0.98,1.01)	0.01	0.532
Religious/spiritual comfort	1.02		(1.00,1.03)	0.01	0.057	0.99		(0.98,1.01)	0.01	0.273
Feel loved by God	1.02		(1.00,1.03)	0.01	0.030*	1.00		(0.98,1.01)	0.01	0.533
Feel punished by God	1.00		(0.99,1.01)	0.01	0.679	1.00		(0.98,1.01)	0.01	0.502
Experienced religious criticism	1.00		(0.99,1.01)	0.00	0.560	1.00		(0.99,1.01)	0.00	0.946
Faith-sharing	1.01		(0.99,1.02)	0.01	0.351	1.00		(0.98,1.01)	0.01	0.539

Notes. N=5368; Reference for focal predictor: never/rarely. RR, risk-ratio, null effect is 1.00; ES, effect size measure for standardized regression coefficient, null effect is 0.00; SE, standard error, the SE reported for binary/Likert-type outcomes where risk-ratios are on the log(RR) scale; CI, confidence interval; p-value, a Wald-type test of the null hypothesis that the effect of the focal predictor is zero; (a) item part of the Happiness & Life Satisfaction domain of the Secure Flourishing Index; (b) item part of the Physical & Mental Health domain of the Secure Flourishing Index; (c) item part of the Meaning & Purpose domain of the Secure Flourishing Index; (d) item part of the Character & Virtue domain of the Secure Flourishing Index; (e) item part of the Subjective Social Connectedness domain of the Secure Flourishing Index; (f) item part of the Financial & Material Security domain of the Secure Flourishing Index.

Multiple imputation was performed to impute missing data on the covariates, exposure, and outcomes. All models controlled for sociodemographic and childhood factors assessed at Wave 1. For Model 2 with PC (principal components), the first seven principal components of the entire set of contemporaneous confounders assessed at Wave 1 were included as additional covariates of the outcomes at Wave 2.

An outcome-wide analytic approach was used, and a separate model was run for each outcome. A different type of model was run depending on the nature of the outcome: (1) for each binary outcome, a weighted generalized linear model (with a log link and Poisson distribution) was used to estimate an RR; and (2) for each continuous outcome, a weighted linear regression model was used to estimate a ES. All effect sizes were standardized. For continuous outcomes, the ES represents the change in SD on the outcome between the lower and upper categories of the binary focal predictor. For binary outcomes, the RR represents the change in risk of being in the upper category compared to the lower category between the lower and upper categories of the binary focal predictor.

P-value significance thresholds: $p < 0.05$*, $p < 0.005$**, (Bonferroni) $p < 6.41e-04$***, correction for multiple testing using Bonferroni adjusted significant threshold.

Table S30h. Associations of forgivingness with adult well-being and other outcomes at Wave 2 in United Kingdom using complete-case analyses with attrition weights.

	Model 1: Demographic and Childhood Variables as Covariates					Model 2: Demographic, Childhood, and Other Wave 1 Confounding Variables (Via Principal Components) as Covariates				
Outcome	RR	ES	95% CI	SE	p-value	RR	ES	95% CI	SE	p-value
Human Flourishing										
Secure flourishing index		0.12	(0.07,0.17)	0.02	3.77e-07***		0.03	(-0.01,0.06)	0.02	0.116
Flourishing index		0.13	(0.08,0.17)	0.02	1.03e-07***		0.03	(-0.01,0.06)	0.02	0.134
Happiness & life satisfaction		0.10	(0.05,0.15)	0.02	4.19e-05***		0.02	(-0.02,0.06)	0.02	0.380
Physical & mental health		0.09	(0.04,0.14)	0.02	2.51e-04***		0.03	(-0.01,0.07)	0.02	0.168
Meaning & purpose		0.08	(0.03,0.13)	0.02	9.35e-04**		-0.01	(-0.05,0.03)	0.02	0.554
Character & virtue		0.15	(0.10,0.20)	0.03	1e-08***		0.07	(0.03,0.12)	0.02	1.87e-03**
Close social relationships		0.11	(0.06,0.15)	0.02	5.14e-06***		0.02	(-0.02,0.06)	0.02	0.350
Financial & material security		0.05	(-0.00,0.09)	0.03	0.071		0.02	(-0.03,0.07)	0.02	0.397
Psychological Well-Being										
Happiness (a)		0.10	(0.05,0.16)	0.03	4.9e-05***		0.02	(-0.02,0.07)	0.02	0.282
Life satisfaction (a)		0.09	(0.04,0.14)	0.03	3.36e-04***		0.01	(-0.03,0.05)	0.02	0.610
Current life evaluation		0.05	(-0.00,0.09)	0.03	0.074		-0.01	(-0.06,0.03)	0.02	0.560
Future life evaluation		0.04	(-0.01,0.08)	0.02	0.147		-0.03	(-0.08,0.02)	0.02	0.191
Optimism		0.10	(0.05,0.15)	0.02	6.15e-05***		0.01	(-0.03,0.06)	0.02	0.491
Freedom to pursue what's important		0.06	(0.01,0.11)	0.03	0.012*		-0.00	(-0.05,0.04)	0.02	0.909
Inner peace	1.03		(1.01,1.06)	0.01	2.63e-03**	1.00		(0.98,1.02)	0.01	0.762
Life balance	1.04		(1.02,1.07)	0.01	6.14e-04***	1.01		(0.99,1.03)	0.01	0.330
Sense of mastery	1.03		(1.01,1.05)	0.01	4.96e-03**	1.01		(0.99,1.03)	0.01	0.455
Meaningful activities (c)		0.08	(0.03,0.13)	0.03	1.22e-03**		-0.01	(-0.05,0.04)	0.02	0.813
Understanding purpose (c)		0.07	(0.02,0.12)	0.02	3.94e-03**		-0.02	(-0.06,0.03)	0.02	0.434
Self-rated mental health (b)		0.09	(0.04,0.13)	0.02	1.83e-04***		0.01	(-0.02,0.05)	0.02	0.476
Psychological Distress										
Traumatic distress	0.98		(0.95,1.00)	0.01	0.051	0.98		(0.96,1.00)	0.01	0.069
Depression symptoms composite	0.98		(0.96,1.00)	0.01	0.046*	1.00		(0.98,1.02)	0.01	0.981
Depression – feel hopeless	0.97		(0.95,1.00)	0.01	0.016*	1.00		(0.98,1.01)	0.01	0.641
Depression – loss of interest	0.98		(0.96,1.00)	0.01	0.075	1.00		(0.98,1.02)	0.01	0.964
Anxiety symptoms composite	0.99		(0.96,1.01)	0.01	0.193	1.00		(0.98,1.02)	0.01	0.662
Anxiety – feel on edge	0.99		(0.97,1.01)	0.01	0.277	1.01		(0.99,1.03)	0.01	0.424
Anxiety – cannot stop worrying	0.98		(0.96,1.00)	0.01	0.059	1.00		(0.98,1.02)	0.01	0.745
Suffering	0.99		(0.97,1.01)	0.01	0.306	1.00		(0.98,1.02)	0.01	0.857
Social Well-Being										
Relationship contentment (e)		0.10	(0.05,0.15)	0.02	1.54e-05***		0.02	(-0.03,0.06)	0.02	0.454
Relationship satisfaction (e)		0.10	(0.06,0.15)	0.02	1.53e-05***		0.02	(-0.02,0.07)	0.02	0.323
Social support		0.12	(0.07,0.16)	0.02	2.21e-06***		0.06	(0.01,0.11)	0.02	0.013*
Intimate/close friend	1.03		(1.01,1.05)	0.01	8.3e-04**	1.02		(1.00,1.04)	0.01	0.098
Government approval	1.00		(0.99,1.02)	0.01	0.617	1.00		(0.98,1.01)	0.01	0.703
Say in government	1.02		(1.00,1.04)	0.01	0.064	1.01		(0.99,1.03)	0.01	0.404
Belonging in country		0.05	(0.00,0.10)	0.03	0.040*		-0.00	(-0.05,0.04)	0.02	0.876
City/place satisfaction	1.03		(1.01,1.06)	0.01	0.006*	1.02		(1.00,1.04)	0.01	0.094
Trust within country	1.05		(1.03,1.08)	0.01	3.1e-05***	1.04		(1.02,1.07)	0.01	1.48e-04***
Social Participation										
Ever been married	1.00		(0.99,1.01)	0.01	0.751	1.00		(0.99,1.02)	0.01	0.744
Currently divorced	1.00		(0.99,1.01)	0.00	0.985	1.00		(0.99,1.01)	0.00	0.977
Number of children		0.00	(-0.04,0.04)	0.02	0.961		-0.00	(-0.04,0.04)	0.02	0.949
Weekly+ community participation	1.01		(0.99,1.03)	0.01	0.225	1.01		(0.99,1.03)	0.01	0.268
Weekly+ religious attendance	1.00		(0.99,1.01)	0.01	0.946	1.00		(0.99,1.01)	0.01	0.748
Social Distress										
Loneliness		-0.08	(-0.12,-0.03)	0.02	2.44e-03**		-0.02	(-0.06,0.03)	0.02	0.473
Perceived discrimination	0.99		(0.97,1.01)	0.01	0.255	0.99		(0.97,1.01)	0.01	0.304
Character & Prosocial Behavior										
Orientation to promote good (d)		0.15	(0.10,0.20)	0.03	6.74e-09***		0.07	(0.03,0.12)	0.02	1.82e-03**
Delayed gratification (d)		0.12	(0.07,0.17)	0.03	9.35e-06***		0.06	(0.01,0.10)	0.02	0.024*
Hope		0.10	(0.05,0.15)	0.02	2.52e-05***		0.01	(-0.03,0.05)	0.02	0.664
Gratitude		0.10	(0.05,0.15)	0.02	5.24e-05***		0.02	(-0.03,0.06)	0.02	0.464
Showing love/care		0.14	(0.09,0.19)	0.03	5.39e-08***		0.07	(0.03,0.12)	0.02	2.48e-03**
Forgivingness	1.24		(1.22,1.27)	0.01	2.22e-16***	1.23		(1.20,1.26)	0.01	2.22e-16***
Charitable giving	1.02		(1.00,1.05)	0.01	0.050*	1.01		(0.99,1.03)	0.01	0.294
Helping strangers	1.03		(1.01,1.06)	0.01	0.007*	1.02		(1.00,1.04)	0.01	0.116
Volunteering	1.00		(0.98,1.02)	0.01	0.837	1.00		(0.98,1.02)	0.01	0.818
Physical Health & Health Behavior										
Self-rated physical health (b)		0.07	(0.02,0.11)	0.02	0.008*		0.04	(-0.01,0.08)	0.02	0.111
Health problems	0.99		(0.97,1.02)	0.01	0.601	0.99		(0.97,1.01)	0.01	0.368
Pain in past 4 weeks	0.98		(0.96,1.00)	0.01	0.120	0.99		(0.96,1.01)	0.01	0.195
Daily smoker	1.01		(1.00,1.03)	0.01	0.071	1.01		(0.99,1.02)	0.01	0.462
Number of drinks per week		-0.00	(-0.05,0.04)	0.02	0.908		0.01	(-0.03,0.05)	0.02	0.687
Days exercise per week		0.02	(-0.03,0.07)	0.03	0.535		0.01	(-0.04,0.06)	0.02	0.740
Socioeconomic Outcomes										
Financial security (f)		0.02	(-0.03,0.07)	0.02	0.501		-0.00	(-0.05,0.04)	0.02	0.828
Material security (f)		0.07	(0.02,0.12)	0.03	0.008*		0.04	(-0.01,0.09)	0.03	0.093
Educational attainment (16+ years)	0.99		(0.98,1.00)	0.01	0.046*	0.99		(0.98,1.00)	0.01	0.062
Currently employed	0.99		(0.98,1.01)	0.01	0.356	0.99		(0.98,1.01)	0.01	0.400
Financially comfortable/getting by	0.99		(0.97,1.01)	0.01	0.344	0.99		(0.97,1.00)	0.01	0.113
Own home	0.99		(0.96,1.01)	0.01	0.233	0.99		(0.97,1.02)	0.01	0.573
Income – top quintile	1.00		(0.98,1.03)	0.01	0.808	1.00		(0.98,1.03)	0.01	0.917
Religion & Spirituality										
Religious/spiritual connection	1.02		(1.00,1.04)	0.01	0.089	1.00		(0.98,1.02)	0.01	0.970
Belief in life after death	1.00		(0.98,1.02)	0.01	0.938	0.98		(0.96,1.00)	0.01	0.095
Transformative religious experience	1.02		(1.00,1.04)	0.01	0.054	1.01		(0.99,1.02)	0.01	0.312
Religious reading or listening	1.01		(0.99,1.02)	0.01	0.334	1.00		(0.99,1.02)	0.01	0.587
Prayer or meditation	1.02		(1.00,1.03)	0.01	0.014*	1.01		(1.00,1.02)	0.01	0.221
Belief in God/gods/spiritual forces	1.01		(0.99,1.03)	0.01	0.522	0.99		(0.97,1.01)	0.01	0.201
Religious centrality	1.00		(0.98,1.02)	0.01	0.965	0.99		(0.97,1.00)	0.01	0.055
Religious/spiritual comfort	1.00		(0.99,1.03)	0.01	0.623	0.99		(0.97,1.00)	0.01	0.075
Feel loved by God	1.01		(0.99,1.03)	0.01	0.219	0.99		(0.98,1.01)	0.01	0.364
Feel punished by God	0.99		(0.97,1.01)	0.01	0.219	0.99		(0.98,1.01)	0.01	0.202
Experienced religious criticism	1.00		(0.98,1.01)	0.01	0.707	1.00		(0.98,1.01)	0.01	0.533
Faith-sharing	1.00		(0.98,1.02)	0.01	0.867	0.99		(0.97,1.01)	0.01	0.229

Table S30h. Associations of forgivingness with adult well-being and other outcomes at Wave 2 in United Kingdom using complete-case analyses with attrition weights.

Outcome	Model 1: Demographic and Childhood Variables as Covariates					Model 2: Demographic, Childhood, and Other Wave 1 Confounding Variables (Via Principal Components) as Covariates				
	RR	ES	95% CI	SE	p-value	RR	ES	95% CI	SE	p-value

Notes. N=3619; Reference for focal predictor: never/rarely. RR, risk-ratio, null effect is 1.00; ES, effect size measure for standardized regression coefficient, null effect is 0.00; SE, standard error, the SE reported for binary/Likert-type outcomes where risk-ratios are on the log(RR) scale; CI, confidence interval; p-value, a Wald-type test of the null hypothesis that the effect of the focal predictor is zero; (a) item part of the Happiness & Life Satisfaction domain of the Secure Flourishing Index; (b) item part of the Physical & Mental Health domain of the Secure Flourishing Index; (c) item part of the Meaning & Purpose domain of the Secure Flourishing Index; (d) item part of the Character & Virtue domain of the Secure Flourishing Index; (e) item part of the Subjective Social Connectedness domain of the Secure Flourishing Index; (f) item part of the Financial & Material Security domain of the Secure Flourishing Index.

Attrition weights were computed to adjust the complete case data (those who responded at Wave 2 to at least 50% of the questions) and multiple imputation was used to impute missing data on all remaining within wave on the covariates, exposure, and outcomes. All models controlled for sociodemographic and childhood factors assessed at Wave 1. For Model 2 with PC (principal components), the first seven principal components of the entire set of contemporaneous confounders assessed at Wave 1 were included as additional covariates of the outcomes at Wave 2.

An outcome-wide analytic approach was used, and a separate model was run for each outcome. A different type of model was run depending on the nature of the outcome: (1) for each binary outcome, a weighted generalized linear model (with a log link and Poisson distribution) was used to estimate an RR; and (2) for each continuous outcome, a weighted linear regression model was used to estimate a ES. All effect sizes were standardized. For continuous outcomes, the ES represents the change in SD on the outcome between the lower and upper categories of the binary focal predictor. For binary outcomes, the RR represents the change in risk of being in the upper category compared to the lower category between the lower and upper categories of the binary focal predictor.

P-value significance thresholds: $p < 0.05$*, $p < 0.005$**, (Bonferroni) $p < 6.41e-04$***, correction for multiple testing using Bonferroni adjusted significant threshold.

Table S30i. Sensitivity analysis of forgivingness outcome-wide results to unmeasured confounding using E-values in United Kingdom

	Multiple Imputation				Complete Case w/ Attrition Weights			
	Model 1: Demographics and Childhood Variables as Covariates		Model 2: Demographics, Childhood, and Other Wave 1 Confounders (Via Principal Components) as Covariates		Model 1: Demographics and Childhood Variables as Covariates		Model 2: Demographics, Childhood, and Other Wave 1 Confounders (Via Principal Components) as Covariates	
Outcome	EE	ECI	EE	ECI	EE	ECI	EE	ECI
Human Flourishing								
Secure flourishing index	1.78	1.56	1.25	1.00	1.85	1.58	1.31	1.00
Flourishing index	1.84	1.63	1.28	1.00	1.88	1.62	1.30	1.00
Happiness & life satisfaction	1.70	1.47	1.23	1.00	1.74	1.46	1.23	1.00
Physical & mental health	1.58	1.35	1.27	1.00	1.67	1.39	1.30	1.00
Meaning & purpose	1.64	1.40	1.12	1.00	1.63	1.34	1.18	1.00
Character & virtue	1.96	1.71	1.50	1.25	2.04	1.74	1.58	1.30
Close social relationships	1.71	1.47	1.20	1.00	1.77	1.51	1.24	1.00
Financial & material security	1.30	1.00	1.03	1.00	1.41	1.00	1.24	1.00
Psychological Well-Being								
Happiness	1.69	1.44	1.24	1.00	1.76	1.47	1.28	1.00
Life satisfaction	1.64	1.39	1.20	1.00	1.68	1.39	1.17	1.00
Current life evaluation	1.46	1.18	1.08	1.00	1.41	1.00	1.19	1.00
Future life evaluation	1.46	1.19	1.13	1.00	1.35	1.00	1.32	1.00
Optimism	1.76	1.51	1.33	1.00	1.73	1.45	1.21	1.00
Freedom to pursue what's important	1.41	1.06	1.23	1.00	1.52	1.20	1.08	1.00
Inner peace	1.36	1.21	1.15	1.00	1.36	1.19	1.09	1.00
Life balance	1.40	1.25	1.21	1.00	1.41	1.24	1.17	1.00
Sense of mastery	1.29	1.11	1.09	1.00	1.33	1.16	1.14	1.00
Meaningful activities	1.65	1.39	1.13	1.00	1.64	1.33	1.11	1.00
Understanding purpose	1.54	1.28	1.20	1.00	1.55	1.26	1.22	1.00
Self-rated mental health	1.57	1.33	1.20	1.00	1.67	1.39	1.20	1.00
Psychological Distress								
Traumatic distress	1.18	1.00	1.19	1.00	1.29	1.00	1.27	1.00
Depression symptoms composite	1.22	1.00	1.06	1.00	1.28	1.03	1.02	1.00
Depression – feel hopeless	1.23	1.00	1.06	1.00	1.31	1.11	1.11	1.00
Depression – loss of interest	1.22	1.00	1.06	1.00	1.26	1.00	1.03	1.00
Anxiety symptoms composite	1.16	1.00	1.11	1.00	1.22	1.00	1.11	1.00
Anxiety – feel on edge	1.16	1.00	1.10	1.00	1.19	1.00	1.15	1.00
Anxiety – cannot stop worrying	1.18	1.00	1.01	1.00	1.27	1.00	1.09	1.00
Suffering	1.13	1.00	1.05	1.00	1.19	1.00	1.07	1.00
Social Well-Being								
Relationship contentment	1.69	1.44	1.21	1.00	1.74	1.47	1.21	1.00
Relationship satisfaction	1.65	1.37	1.17	1.00	1.75	1.48	1.26	1.00
Social support	1.76	1.51	1.45	1.17	1.83	1.55	1.50	1.19
Intimate/close friend	1.33	1.18	1.20	1.00	1.36	1.21	1.23	1.00
Government approval	1.15	1.00	1.04	1.00	1.11	1.00	1.09	1.00
Say in government	1.23	1.00	1.13	1.00	1.26	1.00	1.16	1.00
Belonging in country	1.38	1.00	1.12	1.00	1.46	1.08	1.09	1.00
City/place satisfaction	1.35	1.17	1.26	1.00	1.36	1.17	1.25	1.00
Trust within country	1.35	1.14	1.31	1.07	1.47	1.31	1.43	1.27
Social Participation								
Ever been married	1.10	1.00	1.10	1.00	1.07	1.00	1.07	1.00
Currently divorced	1.06	1.00	1.06	1.00	1.01	1.00	1.01	1.00
Number of children	1.14	1.00	1.05	1.00	1.05	1.00	1.05	1.00
Weekly+ community participation	1.22	1.00	1.16	1.00	1.19	1.00	1.18	1.00
Weekly+ religious attendance	1.06	1.00	1.04	1.00	1.03	1.00	1.07	1.00
Social Distress								
Loneliness	1.47	1.16	1.07	1.00	1.59	1.29	1.22	1.00
Perceived discrimination	1.12	1.00	1.11	1.00	1.19	1.00	1.18	1.00
Character & Prosocial Behavior								
Orientation to promote good	2.00	1.72	1.56	1.28	2.03	1.74	1.59	1.30
Delayed gratification	1.71	1.45	1.34	1.00	1.84	1.54	1.48	1.14
Hope	1.71	1.45	1.18	1.00	1.75	1.47	1.16	1.00
Gratitude	1.83	1.60	1.38	1.10	1.73	1.45	1.22	1.00
Showing love/care	1.96	1.69	1.58	1.31	1.99	1.69	1.58	1.29
Forgivingness	2.58	2.44	2.52	2.38	2.59	2.43	2.52	2.36
Charitable giving	1.26	1.00	1.12	1.00	1.29	1.01	1.19	1.00
Helping strangers	1.33	1.17	1.21	1.00	1.35	1.16	1.25	1.00
Volunteering	1.20	1.00	1.13	1.00	1.07	1.00	1.08	1.00
Physical Health & Health Behavior								
Self-rated physical health	1.46	1.20	1.29	1.00	1.54	1.23	1.35	1.00
Health problems	1.01	1.00	1.12	1.00	1.13	1.00	1.16	1.00
Pain in past 4 weeks	1.17	1.00	1.20	1.00	1.25	1.00	1.22	1.00
Daily smoker	1.20	1.00	1.17	1.00	1.21	1.00	1.12	1.00
Number of drinks per week	1.14	1.00	1.07	1.00	1.08	1.00	1.15	1.00
Days exercise per week	1.28	1.00	1.09	1.00	1.21	1.00	1.14	1.00
Socioeconomic Outcomes								
Financial security	1.13	1.00	1.22	1.00	1.22	1.00	1.11	1.00
Material security	1.40	1.06	1.23	1.00	1.56	1.23	1.40	1.00
Educational attainment (16+ years)	1.12	1.00	1.13	1.00	1.20	1.02	1.19	1.00
Currently employed	1.07	1.00	1.06	1.00	1.13	1.00	1.13	1.00
Financially comfortable/getting by	1.10	1.00	1.18	1.00	1.16	1.00	1.22	1.00
Own home	1.18	1.00	1.15	1.00	1.21	1.00	1.14	1.00
Income – top quintile	1.09	1.00	1.07	1.00	1.09	1.00	1.06	1.00
Religion & Spirituality								
Religious/spiritual connection	1.33	1.17	1.13	1.00	1.25	1.00	1.03	1.00
Belief in life after death	1.16	1.00	1.18	1.00	1.05	1.00	1.23	1.00
Transformative religious experience	1.24	1.07	1.10	1.00	1.24	1.00	1.15	1.00
Religious reading or listening	1.16	1.00	1.08	1.00	1.14	1.00	1.09	1.00
Prayer or meditation	1.28	1.18	1.16	1.00	1.25	1.10	1.15	1.00
Belief in God/gods/spiritual forces	1.22	1.00	1.10	1.00	1.14	1.00	1.19	1.00
Religious centrality	1.20	1.00	1.11	1.00	1.03	1.00	1.21	1.00
Religious/spiritual comfort	1.23	1.00	1.15	1.00	1.12	1.00	1.22	1.00
Feel loved by God	1.24	1.07	1.11	1.00	1.19	1.00	1.14	1.00
Feel punished by God	1.08	1.00	1.10	1.00	1.17	1.00	1.17	1.00
Experienced religious criticism	1.08	1.00	1.03	1.00	1.08	1.00	1.10	1.00
Faith-sharing	1.14	1.00	1.11	1.00	1.06	1.00	1.17	1.00

Notes. EE, E-value for estimate; ECI, E-value for the limit of the confidence interval. The formula for calculating E-values can be found in VanderWeele and Ding (2017). E-values for estimate are the minimum strength of association on the risk ratio scale that an unmeasured confounder would need to have with both the exposure and the outcome to fully explain away the observed association between the exposure and outcome, conditional on the measured covariates. E-values for the 95% CI closest to the null denote the minimum strength of association on the risk ratio scale that an unmeasured confounder would need to have with both the exposure and the outcome to shift the CI to include the null value, conditional on the measured covariates.

Table S31a. Weighted summary statistics for demographic and childhood variables in United States

**Characteristic**	**Wave 1** N = 38,312	**Wave 2** N = 32,257
Forgivingness, n (%)		
Always	7,120 (18.6%)	5,630 (17.5%)
Often	23,156 (60.4%)	19,664 (61.0%)
Rarely	7,206 (18.8%)	6,254 (19.4%)
Never	738 (1.9%)	620 (1.9%)
(Missing)	92 (0.2%)	89 (0.3%)
Year of birth, n (%)		
1943 or earlier (current age: 80+ years)	1,553 (4.1%)	1,505 (4.7%)
1943-1953 (current age: 70-79 years)	4,433 (11.6%)	4,054 (12.6%)
1953-1963 (current age: 60-69 years)	7,676 (20.0%)	6,633 (20.6%)
1963-1973 (current age: 50-59 years)	7,391 (19.3%)	5,947 (18.4%)
1973-1983 (current age: 40-49 years)	5,970 (15.6%)	5,170 (16.0%)
1983-1993 (current age: 30-39 years)	6,965 (18.2%)	5,868 (18.2%)
1993-1998 (current age: 25-29 years)	2,844 (7.4%)	2,211 (6.9%)
1998-2005 (current age: 18-24 years)	1,480 (3.9%)	868 (2.7%)
(Missing)	0 (0%)	0 (0%)
Age of participant		
Mean	51.4	52.4
Standard Deviation	16.8	16.7
Min, Max	18.0, 99.0	19.0, 99.0
Gender, n (%)		
Male	17,982 (46.9%)	15,153 (47.0%)
Female	19,905 (52.0%)	16,779 (52.0%)
Other	305 (0.8%)	255 (0.8%)
(Missing)	121 (0.3%)	69 (0.2%)
Respondent marital status, n (%)		
Single/Never been married	7,995 (20.9%)	6,425 (19.9%)
Married	21,638 (56.5%)	18,436 (57.2%)
Separated	652 (1.7%)	482 (1.5%)
Divorced	3,930 (10.3%)	3,367 (10.4%)
Widowed	2,080 (5.4%)	1,857 (5.8%)
Domestic partner	1,827 (4.8%)	1,530 (4.7%)
(Missing)	189 (0.5%)	159 (0.5%)
Education (years), n (%)		
Up to 8	104 (0.3%)	75 (0.2%)
9-15	22,794 (59.5%)	18,852 (58.4%)
16+	15,328 (40.0%)	13,322 (41.3%)
(Missing)	86 (0.2%)	8 (0.0%)
Employment status, n (%)		
Employed for an employer	19,874 (51.9%)	16,800 (52.1%)
Self-employed	3,274 (8.5%)	2,698 (8.4%)
Retired	9,778 (25.5%)	8,576 (26.6%)
Student	795 (2.1%)	569 (1.8%)
Homemaker	2,063 (5.4%)	1,543 (4.8%)
Unemployed and looking for a job	1,263 (3.3%)	1,003 (3.1%)
None of these/Other	1,181 (3.1%)	934 (2.9%)
(Missing)	84 (0.2%)	133 (0.4%)
Current religious service attendance, n (%)		
More than once a week	2,685 (7.0%)	2,453 (7.6%)
Once a week	6,254 (16.3%)	5,244 (16.3%)

Table S31a. Weighted summary statistics for demographic and childhood variables in United States

**Characteristic**	**Wave 1** N = 38,312	**Wave 2** N = 32,257
One to three times a month	2,868 (7.5%)	2,394 (7.4%)
A few times a year	9,067 (23.7%)	6,900 (21.4%)
Never	17,319 (45.2%)	15,044 (46.6%)
(Missing)	120 (0.3%)	222 (0.7%)
Immigration status, n (%)		
Born in this country	35,040 (91.5%)	29,502 (91.5%)
Born in another country	2,903 (7.6%)	2,456 (7.6%)
(Missing)	369 (1.0%)	299 (0.9%)
Parental marital status around age 12, n (%)		
Parents were married	28,371 (74.1%)	23,991 (74.4%)
Parents were divorced	6,143 (16.0%)	5,115 (15.9%)
Parents were never married	2,371 (6.2%)	1,936 (6.0%)
One or both of them had died	1,030 (2.7%)	914 (2.8%)
Unsure	343 (0.9%)	257 (0.8%)
(Missing)	54 (0.1%)	44 (0.1%)
Religious service attendance around age 12, n (%)		
At least once a week	19,303 (50.4%)	16,248 (50.4%)
One to three times a month	6,734 (17.6%)	5,668 (17.6%)
Less than once a month	5,609 (14.6%)	4,864 (15.1%)
Never	6,518 (17.0%)	5,353 (16.6%)
(Missing)	148 (0.4%)	124 (0.4%)
Relationship with mother when growing up, n (%)		
Very good	21,001 (54.8%)	17,760 (55.1%)
Somewhat good	11,506 (30.0%)	9,696 (30.1%)
Somewhat bad	3,446 (9.0%)	2,937 (9.1%)
Very bad	1,602 (4.2%)	1,279 (4.0%)
(Does not apply)	636 (1.7%)	487 (1.5%)
(Missing)	121 (0.3%)	98 (0.3%)
Relationship with father when growing up, n (%)		
Very good	15,777 (41.2%)	13,235 (41.0%)
Somewhat good	12,712 (33.2%)	10,719 (33.2%)
Somewhat bad	4,692 (12.2%)	4,041 (12.5%)
Very bad	2,430 (6.3%)	2,005 (6.2%)
(Does not apply)	2,656 (6.9%)	2,219 (6.9%)
(Missing)	46 (0.1%)	38 (0.1%)
Felt like an outsider in family when growing up, n (%)		
Yes	9,435 (24.6%)	7,820 (24.2%)
No	28,516 (74.4%)	24,155 (74.9%)
(Missing)	361 (0.9%)	282 (0.9%)
Experienced abuse when growing up, n (%)		
Yes	9,627 (25.1%)	8,035 (24.9%)
No	28,442 (74.2%)	24,013 (74.4%)
(Missing)	243 (0.6%)	209 (0.6%)
Self-rated health when growing up, n (%)		
Excellent	17,674 (46.1%)	15,031 (46.6%)
Very good	12,259 (32.0%)	10,232 (31.7%)
Good	5,944 (15.5%)	5,003 (15.5%)
Fair	1,908 (5.0%)	1,556 (4.8%)
Poor	446 (1.2%)	361 (1.1%)
(Missing)	82 (0.2%)	74 (0.2%)

Table S31a. Weighted summary statistics for demographic and childhood variables in United States

**Characteristic**	**Wave 1**<br>N = 38,312	**Wave 2**<br>N = 32,257
Subjective financial status of family growing up, n (%)		
Lived comfortably	15,474 (40.4%)	12,963 (40.2%)
Got by	15,647 (40.8%)	13,268 (41.1%)
Found it difficult	4,954 (12.9%)	4,164 (12.9%)
Found it very difficult	2,216 (5.8%)	1,844 (5.7%)
(Missing)	21 (0.1%)	18 (0.1%)
Religious affiliation growing up, n (%)		
Christianity	31,211 (81.5%)	26,316 (81.6%)
Taoism	17 (0.0%)	14 (0.0%)
Confucianism	8 (0.0%)	7 (0.0%)
Primal, Animist, or Folk religion	51 (0.1%)	37 (0.1%)
Spiritism	0 (0%)	0 (0%)
Umbanda, Candomblé, and other African-derived religions	0 (0%)	0 (0%)
Chinese folk/traditional religion	0 (0%)	0 (0%)
Islam	195 (0.5%)	157 (0.5%)
Hinduism	184 (0.5%)	129 (0.4%)
Buddhism	158 (0.4%)	128 (0.4%)
Judaism	843 (2.2%)	737 (2.3%)
Sikhism	41 (0.1%)	19 (0.1%)
Baha’i	5 (0.0%)	5 (0.0%)
Jainism	11 (0.0%)	11 (0.0%)
Shinto	6 (0.0%)	5 (0.0%)
Some other religion	315 (0.8%)	262 (0.8%)
No religion/Atheist/Agnostic	5,183 (13.5%)	4,350 (13.5%)
(Missing)	84 (0.2%)	79 (0.2%)

Note. N (%); this table is based on non-imputed data. Cumulative percentages for variables may not add up to 100% due to rounding. Wave 1 characteristics weighted using the Gallup provided sampling weight, ANNUAL_WEIGHT_R2; Wave 2 characteristics weighted accounting for attrition by using the adjusted Wave 1 weight, ANNUAL_WEIGHT_R2, multiplied by the created attrition weight to account for dropout, to maintain nationally representative estimates for Wave 2 characteristics.

Table S31b. Weighted summary statistics for outcome variables in United States

Outcome	Wave 1<br>N = 38,312	Wave 2<br>N = 32,257
Secure flourishing index		
Mean	7.2	7.1
Standard Deviation	1.6	1.6
Min, Max	0.0, 10.0	0.0, 10.0
(Missing)	503 (1.3%)	1,280 (4.0%)
Flourishing index		
Mean	7.3	7.2
Standard Deviation	1.6	1.6
Min, Max	0.0, 10.0	0.0, 10.0
(Missing)	448 (1.2%)	1,225 (3.8%)
Happiness & life satisfaction		
Mean	7.1	6.8
Standard Deviation	1.9	1.9
Min, Max	0.0, 10.0	0.0, 10.0
(Missing)	70 (0.2%)	90 (0.3%)
Physical & mental health		
Mean	7.1	7.0
Standard Deviation	1.8	1.8
Min, Max	0.0, 10.0	0.0, 10.0
(Missing)	42 (0.1%)	820 (2.5%)
Meaning & purpose		
Mean	7.3	7.2
Standard Deviation	2.1	2.1
Min, Max	0.0, 10.0	0.0, 10.0
(Missing)	81 (0.2%)	159 (0.5%)
Character & virtue		
Mean	7.7	7.6
Standard Deviation	1.5	1.5
Min, Max	0.0, 10.0	0.0, 10.0
(Missing)	174 (0.5%)	129 (0.4%)
Close social relationships		
Mean	7.3	7.2
Standard Deviation	2.2	2.2
Min, Max	0.0, 10.0	0.0, 10.0
(Missing)	119 (0.3%)	80 (0.2%)
Financial & material security		
Mean	7.0	6.8
Standard Deviation	2.8	2.8
Min, Max	0.0, 10.0	0.0, 10.0
(Missing)	61 (0.2%)	62 (0.2%)
Happiness		
Mean	7.1	6.9
Standard Deviation	1.9	1.9
Min, Max	0.0, 10.0	0.0, 10.0
(Missing)	23 (<0.1%)	44 (0.1%)
Life satisfaction		
Mean	7.0	6.8
Standard Deviation	2.1	2.0
Min, Max	0.0, 10.0	0.0, 10.0
(Missing)	48 (0.1%)	46 (0.1%)
Current life evaluation		

Table S31b. Weighted summary statistics for outcome variables in United States

Outcome	Wave 1<br>N = 38,312	Wave 2<br>N = 32,257
Mean	7.1	6.7
Standard Deviation	1.8	1.8
Min, Max	0.0, 10.0	0.0, 10.0
(Missing)	25 (<0.1%)	21 (<0.1%)
Future life evaluation		
Mean	7.8	7.5
Standard Deviation	1.9	2.0
Min, Max	0.0, 10.0	0.0, 10.0
(Missing)	73 (0.2%)	65 (0.2%)
Optimism		
Mean	7.8	7.6
Standard Deviation	2.2	2.2
Min, Max	0.0, 10.0	0.0, 10.0
(Missing)	194 (0.5%)	165 (0.5%)
Freedom to pursue what's important		
Mean	7.3	7.1
Standard Deviation	2.5	2.5
Min, Max	0.0, 10.0	0.0, 10.0
(Missing)	44 (0.1%)	22 (<0.1%)
Inner peace, n (%)		
Always	6,330 (16.5%)	4,970 (15.4%)
Often	24,591 (64.2%)	20,528 (63.6%)
Rarely	6,758 (17.6%)	5,668 (17.6%)
Never	534 (1.4%)	475 (1.5%)
(Missing)	100 (0.3%)	615 (1.9%)
Life balance, n (%)		
Always	3,510 (9.2%)	2,658 (8.2%)
Often	25,641 (66.9%)	21,583 (66.9%)
Rarely	8,302 (21.7%)	7,214 (22.4%)
Never	768 (2.0%)	605 (1.9%)
(Missing)	90 (0.2%)	196 (0.6%)
Sense of mastery, n (%)		
Always	6,870 (17.9%)	4,902 (15.2%)
Often	26,782 (69.9%)	22,991 (71.3%)
Rarely	4,168 (10.9%)	3,796 (11.8%)
Never	288 (0.8%)	331 (1.0%)
(Missing)	203 (0.5%)	236 (0.7%)
Meaningful activities		
Mean	7.4	7.2
Standard Deviation	2.1	2.1
Min, Max	0.0, 10.0	0.0, 10.0
(Missing)	22 (<0.1%)	53 (0.2%)
Understanding purpose		
Mean	7.1	7.1
Standard Deviation	2.5	2.5
Min, Max	0.0, 10.0	0.0, 10.0
(Missing)	60 (0.2%)	109 (0.3%)
Self-rated mental health		
Mean	7.4	7.3
Standard Deviation	2.2	2.1
Min, Max	0.0, 10.0	0.0, 10.0

Table S31b. Weighted summary statistics for outcome variables in United States

Outcome	Wave 1<br>N = 38,312	Wave 2<br>N = 32,257
(Missing)	23 (<0.1%)	166 (0.5%)
Traumatic distress, n (%)		
A lot	2,498 (6.5%)	1,969 (6.1%)
Some	7,564 (19.7%)	6,504 (20.2%)
Not very much	12,751 (33.3%)	10,998 (34.1%)
Not at all	15,435 (40.3%)	12,695 (39.4%)
(Missing)	64 (0.2%)	90 (0.3%)
Depression symptoms composite, n (%)	6,937 (18.2%)	5,699 (17.8%)
(Missing)	170 (0.4%)	174 (0.5%)
Depression – feel hopeless, n (%)		
Nearly every day	1,979 (5.2%)	1,591 (4.9%)
More than half the days	3,440 (9.0%)	2,865 (8.9%)
Several days	11,412 (29.8%)	9,694 (30.1%)
Not at all	21,396 (55.8%)	18,048 (56.0%)
(Missing)	84 (0.2%)	58 (0.2%)
Depression – loss of interest, n (%)		
Nearly every day	2,127 (5.6%)	1,584 (4.9%)
More than half the days	4,167 (10.9%)	3,504 (10.9%)
Several days	11,629 (30.4%)	10,088 (31.3%)
Not at all	20,294 (53.0%)	16,956 (52.6%)
(Missing)	95 (0.2%)	125 (0.4%)
Anxiety symptoms composite, n (%)	8,426 (22.1%)	6,913 (21.5%)
(Missing)	135 (0.4%)	98 (0.3%)
Anxiety – feel on edge, n (%)		
Nearly every day	2,978 (7.8%)	2,479 (7.7%)
More than half the days	4,642 (12.1%)	3,754 (11.6%)
Several days	14,058 (36.7%)	12,013 (37.2%)
Not at all	16,554 (43.2%)	13,943 (43.2%)
(Missing)	80 (0.2%)	67 (0.2%)
Anxiety – cannot stop worrying, n (%)		
Nearly every day	2,929 (7.6%)	2,331 (7.2%)
More than half the days	3,795 (9.9%)	3,113 (9.6%)
Several days	10,989 (28.7%)	9,714 (30.1%)
Not at all	20,529 (53.6%)	17,055 (52.9%)
(Missing)	71 (0.2%)	44 (0.1%)
Suffering, n (%)		
A lot	3,159 (8.2%)	2,494 (7.7%)
Some	12,806 (33.4%)	11,082 (34.4%)
Not very much	14,668 (38.3%)	12,430 (38.5%)
Not at all	7,564 (19.7%)	6,146 (19.1%)
(Missing)	115 (0.3%)	104 (0.3%)
Relationship contentment		
Mean	7.5	7.4
Standard Deviation	2.2	2.2
Min, Max	0.0, 10.0	0.0, 10.0
(Missing)	60 (0.2%)	40 (0.1%)
Relationship satisfaction		
Mean	7.1	7.0
Standard Deviation	2.4	2.4
Min, Max	0.0, 10.0	0.0, 10.0
(Missing)	67 (0.2%)	45 (0.1%)

Table S31b. Weighted summary statistics for outcome variables in United States

**Outcome**	**Wave 1** N = 38,312	**Wave 2** N = 32,257
Social support		
Mean	8.2	8.1
Standard Deviation	2.3	2.3
Min, Max	0.0, 10.0	0.0, 10.0
(Missing)	52 (0.1%)	1,615 (5.0%)
Intimate/close friend, n (%)		
Yes	33,445 (87.3%)	28,104 (87.1%)
No	4,730 (12.3%)	3,990 (12.4%)
(Missing)	138 (0.4%)	162 (0.5%)
Government approval, n (%)		
Strongly approve	1,194 (3.1%)	911 (2.8%)
Somewhat approve	8,210 (21.4%)	6,281 (19.5%)
Neither approve nor disapprove	4,893 (12.8%)	3,883 (12.0%)
Somewhat disapprove	9,005 (23.5%)	8,119 (25.2%)
Strongly disapprove	14,933 (39.0%)	12,973 (40.2%)
(Missing)	77 (0.2%)	90 (0.3%)
Say in government, n (%)		
Agree	10,057 (26.3%)	8,201 (25.4%)
Disagree	20,635 (53.9%)	17,879 (55.4%)
Unsure	7,568 (19.8%)	6,114 (19.0%)
(Missing)	52 (0.1%)	63 (0.2%)
Belonging in country		
Mean	7.2	7.1
Standard Deviation	2.6	2.6
Min, Max	0.0, 10.0	0.0, 10.0
(Missing)	184 (0.5%)	269 (0.8%)
City/place satisfaction, n (%)		
Satisfied	27,049 (70.6%)	22,819 (70.7%)
Dissatisfied	7,895 (20.6%)	6,209 (19.2%)
Unsure	3,163 (8.3%)	2,995 (9.3%)
(Missing)	205 (0.5%)	234 (0.7%)
Trust within country, n (%)		
All people	59 (0.2%)	42 (0.1%)
Most people	3,668 (9.6%)	3,049 (9.5%)
Some people	17,093 (44.6%)	13,979 (43.3%)
Not very many people	16,664 (43.5%)	14,413 (44.7%)
None	707 (1.8%)	653 (2.0%)
(Missing)	121 (0.3%)	121 (0.4%)
Number of children		
Mean	0.5	0.5
Standard Deviation	1.2	1.2
Min, Max	0.0, 90.0	0.0, 66.0
(Missing)	247 (0.6%)	7,068 (22%)
Community participation, n (%)		
More than once a week	2,562 (6.7%)	2,379 (7.4%)
Once a week	2,781 (7.3%)	2,557 (7.9%)
One to three times a month	5,128 (13.4%)	4,531 (14.0%)
A few times a year	11,795 (30.8%)	9,572 (29.7%)
Never	15,990 (41.7%)	13,148 (40.8%)
(Missing)	55 (0.1%)	71 (0.2%)
Religious attendance, n (%)		

Table S31b. Weighted summary statistics for outcome variables in United States

Outcome	Wave 1<br>N = 38,312	Wave 2<br>N = 32,257
More than once a week	2,685 (7.0%)	2,453 (7.6%)
Once a week	6,254 (16.3%)	5,244 (16.3%)
One to three times a month	2,868 (7.5%)	2,394 (7.4%)
A few times a year	9,067 (23.7%)	6,900 (21.4%)
Never	17,319 (45.2%)	15,044 (46.6%)
(Missing)	120 (0.3%)	222 (0.7%)
Loneliness		
Mean	3.1	3.1
Standard Deviation	2.8	2.7
Min, Max	0.0, 10.0	0.0, 10.0
(Missing)	27 (<0.1%)	58 (0.2%)
Perceived discrimination, n (%)		
Always	1,609 (4.2%)	1,128 (3.5%)
Often	8,049 (21.0%)	6,387 (19.8%)
Rarely	19,597 (51.2%)	16,562 (51.3%)
Never	8,983 (23.4%)	8,131 (25.2%)
(Missing)	75 (0.2%)	49 (0.2%)
Orientation to promote good		
Mean	8.0	7.8
Standard Deviation	1.6	1.6
Min, Max	0.0, 10.0	0.0, 10.0
(Missing)	54 (0.1%)	56 (0.2%)
Delayed gratification		
Mean	7.5	7.4
Standard Deviation	1.9	1.9
Min, Max	0.0, 10.0	0.0, 10.0
(Missing)	132 (0.3%)	83 (0.3%)
Hope		
Mean	7.8	7.6
Standard Deviation	2.1	2.1
Min, Max	0.0, 10.0	0.0, 10.0
(Missing)	1,883 (4.9%)	572 (1.8%)
Gratitude		
Mean	8.2	8.1
Standard Deviation	2.1	2.2
Min, Max	0.0, 10.0	0.0, 10.0
(Missing)	1,866 (4.9%)	544 (1.7%)
Showing love/care		
Mean	8.6	8.5
Standard Deviation	1.6	1.7
Min, Max	0.0, 10.0	0.0, 10.0
(Missing)	28 (<0.1%)	335 (1.0%)
Forgivingness, n (%)		
Always	7,120 (18.6%)	5,630 (17.5%)
Often	23,156 (60.4%)	19,664 (61.0%)
Rarely	7,206 (18.8%)	6,254 (19.4%)
Never	738 (1.9%)	620 (1.9%)
(Missing)	92 (0.2%)	89 (0.3%)
Charitable giving, n (%)		
Yes	19,814 (51.7%)	18,014 (55.8%)
No	18,455 (48.2%)	14,090 (43.7%)

Table S31b. Weighted summary statistics for outcome variables in United States

Outcome	Wave 1<br>N = 38,312	Wave 2<br>N = 32,257
(Missing)	43 (0.1%)	152 (0.5%)
Helping strangers, n (%)		
Yes	22,379 (58.4%)	17,484 (54.2%)
No	15,891 (41.5%)	14,690 (45.5%)
(Missing)	42 (0.1%)	83 (0.3%)
Volunteering, n (%)		
Yes	10,874 (28.4%)	10,058 (31.2%)
No	27,369 (71.4%)	22,108 (68.5%)
(Missing)	69 (0.2%)	90 (0.3%)
Self-rated physical health		
Mean	6.9	6.7
Standard Deviation	1.9	1.9
Min, Max	0.0, 10.0	0.0, 10.0
(Missing)	29 (<0.1%)	807 (2.5%)
Health problems, n (%)		
Yes	9,163 (23.9%)	8,390 (26.0%)
No	29,048 (75.8%)	23,742 (73.6%)
(Missing)	100 (0.3%)	125 (0.4%)
Pain in past 4 weeks, n (%)		
A lot	5,210 (13.6%)	4,421 (13.7%)
Some	14,266 (37.2%)	11,826 (36.7%)
Not very much	13,471 (35.2%)	11,580 (35.9%)
None at all	5,338 (13.9%)	4,408 (13.7%)
(Missing)	26 (0.1%)	23 (0.1%)
Number of cigarettes per day		
Mean	1.1	1.0
Standard Deviation	4.4	4.3
Min, Max	0.0, 97.0	0.0, 97.0
(Missing)	239 (0.6%)	1,365 (4.2%)
Number of drinks per week		
Mean	2.8	2.4
Standard Deviation	6.0	5.4
Min, Max	0.0, 97.0	0.0, 97.0
(Missing)	176 (0.5%)	1,207 (3.7%)
Days exercise per week		
Mean	2.6	2.5
Standard Deviation	2.2	2.2
Min, Max	0.0, 7.0	0.0, 7.0
(Missing)	54 (0.1%)	72 (0.2%)
Financial security		
Mean	6.7	6.6
Standard Deviation	3.0	3.0
Min, Max	0.0, 10.0	0.0, 10.0
(Missing)	24 (<0.1%)	30 (<0.1%)
Material security		
Mean	7.2	7.1
Standard Deviation	2.8	2.9
Min, Max	0.0, 10.0	0.0, 10.0
(Missing)	40 (0.1%)	35 (0.1%)
Educational attainment (16+ years), n (%)		
Up to 8	104 (0.3%)	75 (0.2%)

Table S31b. Weighted summary statistics for outcome variables in United States

Outcome	Wave 1<br>N = 38,312	Wave 2<br>N = 32,257
9-15	22,794 (59.5%)	18,852 (58.4%)
16+	15,328 (40.0%)	13,322 (41.3%)
(Missing)	86 (0.2%)	8 (0.0%)
Currently employed, n (%)		
Employed for an employer	19,874 (51.9%)	16,800 (52.1%)
Self-employed	3,274 (8.5%)	2,698 (8.4%)
Retired	9,778 (25.5%)	8,576 (26.6%)
Student	795 (2.1%)	569 (1.8%)
Homemaker	2,063 (5.4%)	1,543 (4.8%)
Unemployed and looking for a job	1,263 (3.3%)	1,003 (3.1%)
None of these/Other	1,181 (3.1%)	934 (2.9%)
(Missing)	84 (0.2%)	133 (0.4%)
Financially comfortable/getting by, n (%)		
Living comfortably on present income	16,829 (43.9%)	14,392 (44.6%)
Getting by on present income	14,784 (38.6%)	12,243 (38.0%)
Finding it difficult on present income	4,841 (12.6%)	3,829 (11.9%)
Finding it very difficult on present income	1,766 (4.6%)	1,582 (4.9%)
(Missing)	91 (0.2%)	210 (0.7%)
Own home, n (%)		
Someone in this household owns this home	26,881 (70.2%)	22,869 (70.9%)
Someone in this household rents this home	7,992 (20.9%)	6,600 (20.5%)
Both	1,378 (3.6%)	1,150 (3.6%)
Neither	1,989 (5.2%)	1,419 (4.4%)
Rent	0 (0%)	0 (0%)
Own	0 (0%)	0 (0%)
Something else	0 (0%)	0 (0%)
(Missing)	72 (0.2%)	218 (0.7%)
Religious/spiritual connection, n (%)		
Always	10,070 (26.3%)	9,560 (29.6%)
Often	12,615 (32.9%)	9,102 (28.2%)
Rarely	8,802 (23.0%)	6,980 (21.6%)
Never	6,748 (17.6%)	6,551 (20.3%)
(Missing)	77 (0.2%)	64 (0.2%)
Belief in life after death, n (%)		
Yes	21,473 (56.0%)	17,548 (54.4%)
No	6,657 (17.4%)	5,798 (18.0%)
Unsure	10,033 (26.2%)	8,721 (27.0%)
(Missing)	148 (0.4%)	189 (0.6%)
Transformative religious experience, n (%)		
Yes	13,810 (36.0%)	11,532 (35.8%)
No	24,368 (63.6%)	20,596 (63.8%)
(Missing)	134 (0.3%)	129 (0.4%)
Religious reading or listening, n (%)		
More than once a day	2,548 (6.7%)	2,458 (7.6%)
About once a day	5,507 (14.4%)	4,733 (14.7%)
Sometimes	13,884 (36.2%)	11,120 (34.5%)
Never	16,183 (42.2%)	13,772 (42.7%)
(Missing)	190 (0.5%)	173 (0.5%)
Prayer or meditation, n (%)		
More than once a day	8,048 (21.0%)	6,780 (21.0%)
About once a day	8,605 (22.5%)	7,088 (22.0%)

Table S31b. Weighted summary statistics for outcome variables in United States

Outcome	Wave 1<br>N = 38,312	Wave 2<br>N = 32,257
Sometimes	13,073 (34.1%)	10,577 (32.8%)
Never	8,493 (22.2%)	7,731 (24.0%)
(Missing)	93 (0.2%)	80 (0.2%)
Belief in God/gods/spiritual forces, n (%)		
One God	22,513 (58.8%)	18,969 (58.8%)
More than one god	917 (2.4%)	741 (2.3%)
An impersonal spiritual force	4,700 (12.3%)	3,821 (11.8%)
None of these	5,537 (14.5%)	4,909 (15.2%)
Unsure	4,534 (11.8%)	3,718 (11.5%)
(Missing)	111 (0.3%)	98 (0.3%)
Religious centrality, n (%)		
Agree	17,370 (45.3%)	14,633 (45.4%)
Disagree	6,621 (17.3%)	4,787 (14.8%)
Not relevant	10,454 (27.3%)	9,599 (29.8%)
Unsure	3,703 (9.7%)	3,107 (9.6%)
(Missing)	164 (0.4%)	131 (0.4%)
Religious/spiritual comfort, n (%)		
Agree	22,111 (57.7%)	18,039 (55.9%)
Disagree	3,864 (10.1%)	2,870 (8.9%)
Not relevant	9,530 (24.9%)	8,999 (27.9%)
Unsure	2,724 (7.1%)	2,277 (7.1%)
(Missing)	83 (0.2%)	73 (0.2%)
Feel loved by God, n (%)		
Agree	21,624 (56.4%)	17,856 (55.4%)
Disagree	3,578 (9.3%)	2,707 (8.4%)
Not relevant	9,228 (24.1%)	8,334 (25.8%)
Unsure	3,758 (9.8%)	3,244 (10.1%)
(Missing)	123 (0.3%)	116 (0.4%)
Feel punished by God, n (%)		
Agree	3,084 (8.0%)	2,481 (7.7%)
Disagree	23,247 (60.7%)	18,752 (58.1%)
Not relevant	8,828 (23.0%)	8,428 (26.1%)
Unsure	3,051 (8.0%)	2,499 (7.7%)
(Missing)	102 (0.3%)	97 (0.3%)
Experienced religious criticism, n (%)		
Agree	2,765 (7.2%)	2,197 (6.8%)
Disagree	16,009 (41.8%)	12,832 (39.8%)
Not relevant	15,841 (41.3%)	14,258 (44.2%)
Unsure	3,572 (9.3%)	2,859 (8.9%)
(Missing)	125 (0.3%)	111 (0.3%)
Faith-sharing, n (%)		
Agree	14,001 (36.5%)	11,302 (35.0%)
Disagree	11,332 (29.6%)	9,123 (28.3%)
Not relevant	10,899 (28.4%)	10,140 (31.4%)
Unsure	1,983 (5.2%)	1,594 (4.9%)
(Missing)	97 (0.3%)	98 (0.3%)

Note. N (%); this table is based on non-imputed data. Cumulative percentages for variables may not add up to 100% due to rounding. Wave 1 characteristics weighted using the Gallup provided sampling weight, ANNUAL_WEIGHT_R2; Wave 2 characteristics weighted accounting for attrition by using the adjusted Wave 1 weight, ANNUAL_WEIGHT_R2, multiplied by the created attrition weight to account for dropout, to maintain nationally representative estimates for Wave 2 characteristics.

Table S31c. Unweighted summary statistics for demographic and childhood variables in United States by retention status

**Characteristic**	**Attritors–Not Observed in Wave 2** N = 6,487	**Retained–Observed in Wave 2** N = 32,313
Forgivingness, n (%)		
Always	1,293 (19.9%)	5,898 (18.3%)
Often	3,781 (58.3%)	19,701 (61.0%)
Rarely	1,248 (19.2%)	6,043 (18.7%)
Never	154 (2.4%)	587 (1.8%)
(Missing)	11 (0.2%)	83 (0.3%)
Year of birth, n (%)		
1943 or earlier (current age: 80+ years)	243 (3.8%)	1,334 (4.1%)
1943-1953 (current age: 70-79 years)	472 (7.3%)	4,080 (12.6%)
1953-1963 (current age: 60-69 years)	958 (14.8%)	6,892 (21.3%)
1963-1973 (current age: 50-59 years)	1,066 (16.4%)	6,461 (20.0%)
1973-1983 (current age: 40-49 years)	1,114 (17.2%)	4,910 (15.2%)
1983-1993 (current age: 30-39 years)	1,479 (22.8%)	5,507 (17.0%)
1993-1998 (current age: 25-29 years)	780 (12.0%)	2,033 (6.3%)
1998-2005 (current age: 18-24 years)	374 (5.8%)	1,097 (3.4%)
(Missing)	0 (0%)	0 (0%)
Age of participant		
Mean	46.9	52.6
Standard Deviation	17.0	16.5
Min, Max	18.0, 99.0	18.0, 99.0
Gender, n (%)		
Male	2,852 (44.0%)	15,402 (47.7%)
Female	3,516 (54.2%)	16,610 (51.4%)
Other	72 (1.1%)	232 (0.7%)
(Missing)	48 (0.7%)	68 (0.2%)
Respondent marital status, n (%)		
Single/Never been married	1,642 (25.3%)	6,391 (19.8%)
Married	3,220 (49.6%)	18,793 (58.2%)
Separated	172 (2.7%)	474 (1.5%)
Divorced	678 (10.4%)	3,300 (10.2%)
Widowed	344 (5.3%)	1,764 (5.5%)
Domestic partner	390 (6.0%)	1,441 (4.5%)
(Missing)	40 (0.6%)	150 (0.5%)
Education (years), n (%)		
Up to 8	28 (0.4%)	75 (0.2%)
9-15	4,127 (63.6%)	18,898 (58.5%)
16+	2,320 (35.8%)	13,265 (41.1%)
(Missing)	12 (0.2%)	76 (0.2%)
Employment status, n (%)		
Employed for an employer	3,678 (56.7%)	16,380 (50.7%)
Self-employed	541 (8.3%)	2,778 (8.6%)
Retired	1,194 (18.4%)	8,812 (27.3%)
Student	194 (3.0%)	598 (1.8%)
Homemaker	336 (5.2%)	1,756 (5.4%)
Unemployed and looking for a job	286 (4.4%)	976 (3.0%)
None of these/Other	246 (3.8%)	940 (2.9%)
(Missing)	11 (0.2%)	74 (0.2%)
Current religious service attendance, n (%)		
More than once a week	408 (6.3%)	2,321 (7.2%)

Table S31c. Unweighted summary statistics for demographic and childhood variables in United States by retention status

Characteristic	Attritors–Not Observed in Wave 2<br>N = 6,487	Retained–Observed in Wave 2<br>N = 32,313
Once a week	925 (14.3%)	5,438 (16.8%)
One to three times a month	515 (7.9%)	2,384 (7.4%)
A few times a year	1,670 (25.7%)	7,483 (23.2%)
Never	2,946 (45.4%)	14,591 (45.2%)
(Missing)	24 (0.4%)	96 (0.3%)
Immigration status, n (%)		
Born in this country	5,886 (90.7%)	29,612 (91.6%)
Born in another country	526 (8.1%)	2,406 (7.4%)
(Missing)	75 (1.2%)	296 (0.9%)
Parental marital status around age 12, n (%)		
Parents were married	4,493 (69.3%)	24,310 (75.2%)
Parents were divorced	1,169 (18.0%)	5,023 (15.5%)
Parents were never married	563 (8.7%)	1,801 (5.6%)
One or both of them had died	144 (2.2%)	907 (2.8%)
Unsure	107 (1.6%)	230 (0.7%)
(Missing)	11 (0.2%)	43 (0.1%)
Religious service attendance around age 12, n (%)		
At least once a week	3,139 (48.4%)	16,439 (50.9%)
One to three times a month	1,162 (17.9%)	5,654 (17.5%)
Less than once a month	904 (13.9%)	4,787 (14.8%)
Never	1,256 (19.4%)	5,311 (16.4%)
(Missing)	27 (0.4%)	123 (0.4%)
Relationship with mother when growing up, n (%)		
Very good	3,362 (51.8%)	17,950 (55.6%)
Somewhat good	1,964 (30.3%)	9,685 (30.0%)
Somewhat bad	609 (9.4%)	2,875 (8.9%)
Very bad	379 (5.8%)	1,219 (3.8%)
(Does not apply)	151 (2.3%)	484 (1.5%)
(Missing)	22 (0.3%)	101 (0.3%)
Relationship with father when growing up, n (%)		
Very good	2,549 (39.3%)	13,457 (41.6%)
Somewhat good	2,165 (33.4%)	10,707 (33.1%)
Somewhat bad	772 (11.9%)	3,985 (12.3%)
Very bad	501 (7.7%)	1,940 (6.0%)
(Does not apply)	493 (7.6%)	2,188 (6.8%)
(Missing)	8 (0.1%)	38 (0.1%)
Felt like an outsider in family when growing up, n (%)		
Yes	1,899 (29.3%)	7,590 (23.5%)
No	4,503 (69.4%)	24,449 (75.7%)
(Missing)	86 (1.3%)	274 (0.8%)
Experienced abuse when growing up, n (%)		
Yes	1,815 (28.0%)	7,894 (24.4%)
No	4,634 (71.4%)	24,211 (74.9%)
(Missing)	39 (0.6%)	208 (0.6%)
Self-rated health when growing up, n (%)		
Excellent	2,657 (41.0%)	15,317 (47.4%)
Very good	2,163 (33.3%)	10,233 (31.7%)

Table S31c. Unweighted summary statistics for demographic and childhood variables in United States by retention status

Characteristic	Attritors–Not Observed in Wave 2<br>N = 6,487	Retained–Observed in Wave 2<br>N = 32,313
Good	1,136 (17.5%)	4,854 (15.0%)
Fair	425 (6.5%)	1,485 (4.6%)
Poor	99 (1.5%)	348 (1.1%)
(Missing)	8 (0.1%)	76 (0.2%)
Subjective financial status of family growing up, n (%)		
Lived comfortably	2,602 (40.1%)	13,074 (40.5%)
Got by	2,565 (39.5%)	13,300 (41.2%)
Found it difficult	893 (13.8%)	4,113 (12.7%)
Found it very difficult	424 (6.5%)	1,809 (5.6%)
(Missing)	3 (0.0%)	18 (0.1%)
Religious affiliation growing up, n (%)		
[truncated: 316,682 more chars]
